# Millimeter-Wave Spectroscopy of Methylfuran Isomers: Local vs. Global Treatments of the Internal Rotation

**DOI:** 10.3390/molecules27113591

**Published:** 2022-06-02

**Authors:** Jonas Bruckhuisen, Sathapana Chawananon, Isabelle Kleiner, Anthony Roucou, Guillaume Dhont, Colwyn Bracquart, Pierre Asselin, Arnaud Cuisset

**Affiliations:** 1 LPCA, Laboratoire de Physico-Chimie de l’Atmosphère, Université du Littoral Côte d’Opale, UR4493, F-59140 Dunkerque, France; jonas.bruckhuisen@univ-littoral.fr (J.B.); anthony.roucou@univ-littoral.fr (A.R.); guillaume.dhont@univ-littoral.fr (G.D.); colwyn.bracquart@etu.univ-lyon1.fr (C.B.); 2 CNRS, MONARIS, UMR8233, Sorbonne gé, 4 Pl Jussieu, F-75005 Paris, France; sathapana.chawananon@sorbonne-universite.fr (S.C.); pierre.asselin@upmc.fr (P.A.); 3 CNRS UMR 7583, Laboratoire Interuniversitaire des Systèmes Atmosphériques, Université Paris Cité and Université Paris Est Créteil, F-75013 Paris, France; isabelle.kleiner@lisa.ipsl.fr

**Keywords:** methylfuran, mm-wave spectroscopy, internal rotation, excited torsional states, local vs. global approaches

## Abstract

Methylfurans are methylated aromatic heterocyclic volatile organic compounds and primary or secondary pollutants in the atmosphere due to their capability to form secondary organic aerosols in presence of atmospheric oxidants. There is therefore a significant interest to monitor these molecules in the gas phase. High resolution spectroscopic studies of methylated furan compounds are generally limited to pure rotational spectroscopy in the vibrational ground state. This lack of results might be explained by the difficulties arisen from the internal rotation of the methyl group inducing non-trivial patterns in the rotational spectra. In this study, we discuss the benefits to assign the mm-wave rotational-torsional spectra of methylfuran with the global approach of the *BELGI*-C${}_{s}$ code compared to local approaches such as XIAM and ERHAM. The global approach reproduces the observed rotational lines of 2-methylfuran and 3-methylfuran in the mm-wave region at the experimental accuracy for the ground v${}_{t}=0$ and the first torsional v${}_{t}=1$ states with a unique set of molecular parameters. In addition, the $V_{3}$ and $V_{6}$ parameters describing the internal rotation potential barrier may be determined with a high degree of accuracy with the global approach. Finally, a discussion with other heterocyclic compounds enables the study of the influence of the electronic environment on the hindered rotation of the methyl group.

## 1. Introduction

**Figure 1:**
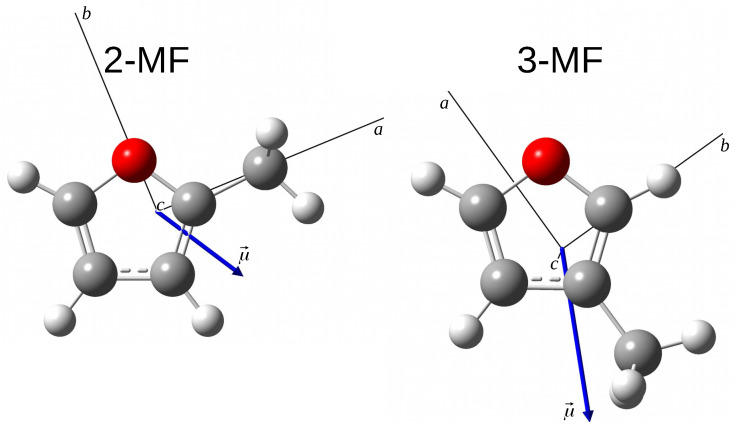
Optimized geometries of the 2-MF and 3-MF isomers determined at the B3LYP/aug-cc-pVTZ level of theory. The axes are the principal axes of inertia. The blue arrow is the permanent electric dipole moment.

Furan and its derivatives (furans) are heterocyclic organic compounds belonging to the family of oxygenated five-membered aromatic molecules. They are primary and secondary pollutants in the atmosphere emitted from multiple sources such as oil refining, coal mining and gasification, biomass and fossil fuel or waste combustions [1,2]. Furans and other heterocyclic compounds such as pyrroles (with N as the heteroatom in the ring) are produced by the pyrolysis of cellulose and are major components of the emission of wildfire burnings [3]. Such compounds as the volatile furan (C${}_{4}$H${}_{4}$O) and methylfurans (C${}_{5}$H${}_{6}$O) (see Figure 1) or the less volatile furaldehyde (C${}_{5}$H${}_{4}$O${}_{2}$) have emission levels 70 to 120 times higher compared to CO [4]. Once furans are emitted, they will undergo gas phase chemistry and, to an extent, will be photolyzed at actinic wavelengths to produce tropospheric ozone or they will react with the main atmospheric oxidants (OH, Cl atoms, ozone) leading to the formation of secondary organic aerosols (SOA) [5]. A similar process occurs during the night by the oxidation of furans with NO${}_{3}$ radicals [1,2,3]. These SOA affect the climate both directly and indirectly and large uncertainties still remain on their radiative forcing [6]. For all these reasons, there is a fundamental interest to monitor furans directly in the atmosphere or in atmospheric simulation chambers to characterize their reactivity and their ability to produce SOA.

Up today, the monitoring of volatile organic compounds (VOC) involved in biomass burning emissions such as furans is mostly performed off-line with chemical methods based on gas chromatography (GC) and mass spectrometry (MS). A GC-MS instrument provides extensive chemical details of discrete air samples collected in wildfires or during a controlled combustion process in the laboratory. These measurements can be complemented by real-time middle or low resolution measurements with an open-path Fourier transform infrared spectroscopy instrument [4,7]. To the best of our knowledge, high-resolution spectroscopic methods were never used to measure on-line individual signatures of furans at trace level in realistic environment. Trace gas detection in complex chemical mixtures with IR and THz techniques are generally focused on a limited panel of 55 light atmospheric molecules listed in the high-resolution HITRAN database [8]. No high resolution data of furans are present in this database, only vibrational cross-sections of furan, methylfurans (MF) and furaldehyde may be used for quantitative spectroscopy in the IR at middle/low resolution. This might be explained by the difficulty to resolve highly congested room temperature rovibrational spectra of these molecules. To overcome this challenge, one first need to measure the spectra in laboratory and assign pure rotational and low-frequency rovibrational spectra of furans at room temperature. Several groups have performed high-resolution pure rotational spectroscopic studies of furans in millimeter-wave at room temperature. We can cite the furan ground state analysis of Wlodarczack et al. [9]; the ground and first torsional states of trans-furfural and ground and excited state of cis-furfural analyses of Motiyenko et al. [10] assigning in total seventeen states. In 1971 Ogata and Kozima recorded the microwave spectrum of the 3-MF between 8.2 and 30 GHz and assigned 20 A and 16 E species transitions [11] and more recently, the ground and first torsional states of the 2-MF isomer were analyzed by Finneran et al. requiring a specific treatment of the internal rotation of the methyl group [12]. Finally, we can mention the study of furan performed by Tokaryk et al. using FT-Far-IR spectroscopy based on a synchrotron source which constitutes, to the best of our knowledge, the only high-resolution rovibrational study of a furan compound [13].

In this study, new high-resolution millimeter-wave measurements have been performed on 3-MF with a versatile solid-state spectrometer already used for the investigation of other VOC such as methoxyphenol [14] or catechol [15] and gas phase monitoring in an atmospheric simulation chamber [16,17]. As in Ref. [12] with 2-MF, the spectroscopic assignment was complicated by the large amplitude motion associated with the internal rotation of the methyl group. Unlike the work of Finneran et al. where the internal rotation was treated with two local approaches, we have succeeded in this study, using a global treatment, to reproduce the spectra in the ground and first excited torsional states at almost the experimental accuracy with a common set of molecular parameters. Amongst them, the $V_{3}$ and $V_{6}$ constants of 2-MF and 3-MF are obtained with a high degree of accuracy allowing to characterise finely the internal rotation potential for both MF isomers.

## 2. Results

The optimized geometries of 2-MF and 3-MF isomers at the B3LYP/aug-cc-pVTZ level of theory are presented in Figure 1. Both isomers are prolate asymmetric tops with Ray’s asymmetry parameter $\kappa_{2}-\mathrm{MF}=-0.69$ and $\kappa_{3}-\mathrm{MF}=-0.73$. Their permanent dipole moment is oriented in the (a,b) plane with the $|\mu_{a}\left|/\right|\mu_{b}|$ ratio of around 0.6 [18] and 1.9 [11], respectively. Both *a*-type and *b*-type transitions are observed in the MF rotational spectra: the most intense transitions are expected to be *b*-type for 2-MF and *a*-type for 3-MF. One difficulty in the assignment process, already mentioned by Finneran et al. in Ref. [12], lies in the possible mixing of energy levels arising from comparable asymmetry and internal rotor splitting. Therefore, we were careful to keep only consistent labels for the energy levels. This difficulty which is generally observed in the rotational-torsional spectra of other small organic molecules containing a methyl internal rotor will be discussed more in detail in Section 3.1.

### 2.1. Global Fit of the Vibrational Ground State and First Torsional State of 2-MF

**Figure 2:**
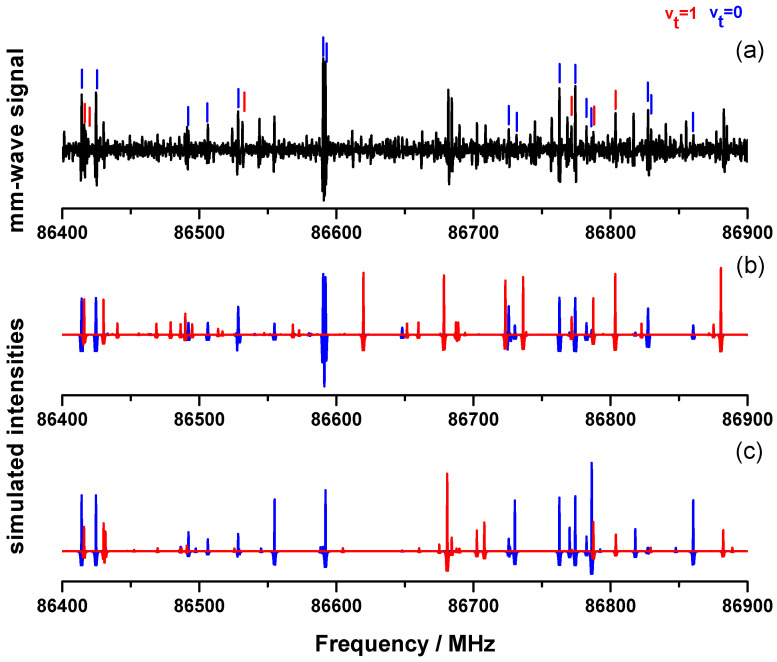
(**a**) Experimental mm-wave spectrum of 2-MF measured in this work (black) in the 86.4–86.9 GHz frequency range. Blue and red sticks correspond respectively to assigned v${}_{t}=0$ and v${}_{t}=1$ lines in Ref. [12]. (**b**) Simulated spectrum with the v${}_{t}=0$ (blue) and v${}_{t}=1$ (red) parameters fitted in Ref. [12] using *ERHAM* (local approach). (**c**) Simulated spectrum of the present work with the v${}_{t}=0$ (blue) and v${}_{t}=1$ (red) using *BELGI*-C${}_{s}$ (global approach) and the fitted parameters from Table S1.

Finneran et al. [12] recorded in 2012 a broad spectrum of 2-MF from 8.7 GHz to 960 GHz. In the 8.7–18.3 GHz range they used a room-temperature chirped-pulse Fourier transform microwave spectrometer. Above 75 GHz, the mm-wave and submm-wave rotational spectra of 2-MF have also been measured by those authors [12] using a direct absorption flow cell spectrometer [19]. The spectra have been obtained in the following spectral ranges: 75–125 GHz, 130–450 GHz, 460–650 GHz, 700–732 GHz, and 760–969 GHz. In the present work, we used a similar spectrometer with an updated technology, a double-pass absorption cell and a room temperature detection based on zero-biased detector (ZBD) [20]. As a test, we decided therefore to measure again the 2-MF spectrum with the first stage of multiplication (×9) covering the 70–110 GHz range. In Figure 2, a part of this spectrum is presented from 86.4 GHz to 86.9 GHz, and we tagged the rotational lines previously assigned in Ref. [12].

Only a few lines remain unassigned. As already mentioned by Finneran et al. [12], apart from the methyl torsion band at $158\;{\mathrm{cm}}^{-1}$, two other low frequency modes occur below $400\;{\mathrm{cm}}^{-1}$ associated with an out-of-plane bending ($237\;{\mathrm{cm}}^{-1}$) and an in-plane bending ($339\;{\mathrm{cm}}^{-1}$) of the methyl group. Those states are most likely populated at room temperature and thus can be responsible for those unassigned lines. In Ref. [12], Finneran et al. have used the eXtended Internal Axis Method (*XIAM*) [21] and the Effective Rotational-torsional Hamiltonian (*ERHAM*) [22] to fit separately the ground state (v${}_{t}=0$) and the first excited torsional state (v${}_{t}=1$) parameters within two “local” approaches. Unlike the *XIAM* code which failed to fit v${}_{t}=1$, the *ERHAM* code succeeded in fitting both v${}_{t}=0$ and v${}_{t}=1$ states separately within their experimental accuracy (see Table 1). We plotted in Figure 2b the simulated spectrum with the *ERHAM* parameters listed in Table 1 of [12] using the prediction published in their supplementary data. Most of the observed rotational lines are predicted taking into account assignments from v${}_{t}=0$ and v${}_{t}=1$ states. The remaining unassigned lines belonging to other excited states involving either v${}_{t}>1$ states or higher frequency vibrational modes are significantly less intense. In this work, we decided to perform a new fit using a global approach with a unique set of parameters for both the v${}_{t}=0$ and v${}_{t}=1$ states by means of the *BELGI*-C${}_{s}$ code [23].

For *XIAM*, as shown in Ref. [12], due to the lack of sufficient higher order distortion constants, as well as high order torsion-rotation terms, their fit leads to several difficulties at higher $K_{a}$ values. For this reason, we decided to start the global fit with *BELGI*-C${}_{s}$ using only the assigned transitions from *ERHAM* for v${}_{t}=0$ and v${}_{t}=1$ states. However, the conversion of the quantum numbers used to label energy levels and molecular transitions in *ERHAM* to the labelling scheme used in the *BELGI*-C${}_{s}$ is not straightforward since the values of $K_{a}$ and $K_{c}$ are approximately determined by the (assumed) order of energy levels for rigid asymmetric rotors. These labels may be incorrect when serious rotational-torsional interactions occur. Thus, for the global fit with *BELGI*-C${}_{s}$ many of the $K_{c}$ values were reassigned. In the range up to J=70 (limit of the *BELGI*-C${}_{s}$ code), only 14 transitions (which correspond to 7 distinct frequencies) with $K_{a}$ = 53 and 54 used in *ERHAM* were excluded in our *BELGI*-C${}_{s}$ fit, as we could not find correct labelling for those levels. As for the *ERHAM* fit, the *BELGI-C${}_{s}$* fit succeeded to reproduce the experimental 2-MF linelist of Ref. [12] at almost their experimental accuracy.

The complete set of the Rho Axis Method (RAM) parameters including centrifugal distortion and higher order terms used in the *BELGI*-C${}_{s}$ is provided in the Supplementary Materials (Table S1). In Table 1, we present the fitted rotational constants for both the (v${}_{t}=0$) and (v${}_{t}=1$) states, after transforming them into the principal axis method (PAM). In addition, the global approach allows us to fit the height of the internal rotation barrier $V_{3}$ and its higher-order correction $V_{6}$ with a high degree of accuracy. This is also the case for some structural parameters like the angles between the internal rotation axis and the principal inertia axes. We notice the good agreement of these experimentally derived parameters with those obtained from the B3LYP/aug-cc-pVTZ calculations presented in Section 4.1.1 and discussed in Section 3.2.

Figure 2c shows 500 MHz of a 2-MF spectrum simulated with the unique set of molecular constants obtained from the *BELGI*-C${}_{s}$ fit (The complete line list is provided in Table S3 of the Supplementary Material). As for Figure 2b, a simulated spectrum based on the *ERHAM* fit, the rotational lines belonging to the v${}_{t}=0$ and v${}_{t}=1$ states are plotted respectively in blue and in red. A direct comparison between the local *ERHAM* and global *BELGI*-C${}_{s}$ fits may be performed. Both predictions are able to reproduce reasonably the measured line positions of the 2-MF spectrum. Nevertheless, some differences are observed between these two simulated spectra especially when considering the relative intensities. Indeed the Figure 2b,c exhibit a few line intensities which are not in agreement with the experimental ones (e.g.,: the v${}_{t}=1$ line centered around 86,620 MHz for the *ERHAM* prediction or the v${}_{t}=0$ line centered around 86,786 MHz for the *BELGI*-C${}_{s}$ prediction). The differences between local (*XIAM* and *ERHAM*) and global (*BELGI*-C${}_{s}$) are discussed in more details in Section 4.1.2.

### 2.2. Global Fit of the Vibrational Ground State and First Torsional State of 3-MF

Concerning 3-MF, a microwave study was performed in 1971 by Ogata et al. [11] in the 8.2–30 GHz range, providing a first estimation of the rotational constants for the vibrational ground state. This study allowed them to determine the permanent dipole moment from Stark measurements (1.03 D) and constants associated to the CH${}_{3}$ internal rotation like the $V_{3}$ barrier height ($381\;{\mathrm{cm}}^{-1}$) from the observed A-E splittings of 16 transitions. In our present work the mm-wave spectral analysis of 3-MF allowed us to identify 2463 new transitions in the v${}_{t}=0$ state and 2017 in the first excited v${}_{t}=1$ state up to J=50.

**Figure 3:**
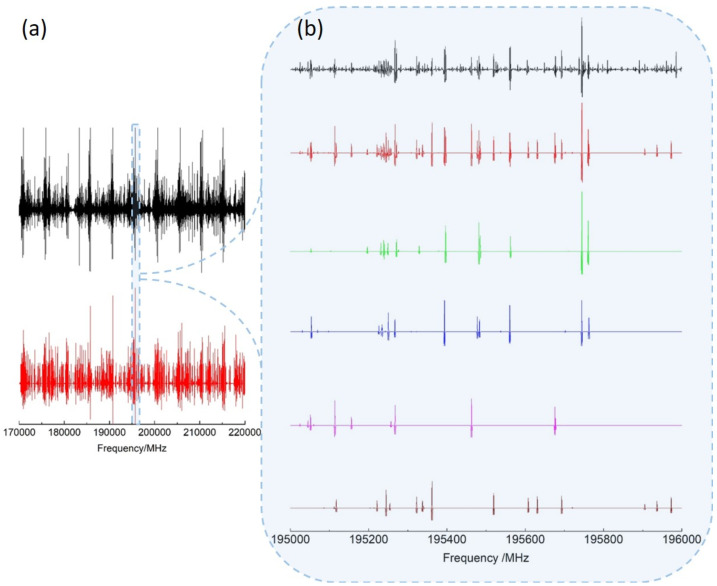
(**a**) Experimental (black) and calculated (red) rotational spectra of 3-MF in the 170–220 GHz frequency range. (**b**) Zoom on a 1GHz range highlighting the different species contributing to the calculated spectrum: v${}_{t}=0$ A (green) and E (blue) species; v${}_{t}=1$ A (magenta) and E (brown) species.

As shown in Figure 3, a simulated spectrum (red) consisting of the v${}_{t}=0$ A- (green) and E-species (blue) as well as v${}_{t}=1$ A- (magenta) and E-species (brown) rotational lines predicted with our *BELGI*-C${}_{s}$ fit reproduces the experimental spectrum (black) well. For the frequency range of 70–220 GHz, $J_{max}=50$ and $K_{a,max}=31$ for v${}_{t}=0$ and v${}_{t}=1$ was sufficient to assign the most intense lines. Remaining unassigned weaker lines are probably associated to the out-of-plane and in-plane methyl bending modes predicted respectively at 267 cm${}^{-1}$ and 321 cm${}^{-1}$ with anharmonic B3LYP/aug-cc-pVTZ frequency calculation.

To compare the efficiency of local and global approaches (see Section 3.1), the same data set (excluding one line) was fitted using the *XIAM* code for the v${}_{t}=0$ and v${}_{t}=1$ states separately. The results of these fits are given in Table 2 along with the theoretical B3LYP/aug-cc-pVTZ calculation. Again, as for the 2-MF, our PAM values fitted by the *BELGI*-C${}_{s}$ code agree reasonably well with the theoretical calculations. The complete set of constants (19 parameters) obtained from the *BELGI*-C${}_{s}$ global fit in the RAM axis system is presented in Table S2 and the line list deduced from our global *BELGI*-C${}_{s}$ fit is given in Table S4 of the Supplementary Materials.

## 3. Discussion

### 3.1. “Local” versus “Global” Approach

A “local” method treats separately each torsional v${}_{t}$ state, whereas a “global” method explicitly takes into account all the interactions between the different v${}_{t}$ states. Both *XIAM* and *ERHAM* programs adjust the parameters of each torsional state separately: they belong to “local” approaches. *BELGI*-C${}_{s}$ on the other hand allows, for a given vibrational state, the fit of several torsional states together within the same rotation-torsion Hamiltonian matrix, see Section 4.1.2. A comparison of the results obtained with a “local” approach with those issued from a “global” approach has already been introduced in several papers [23,25,26]. Local approaches have shown their efficiency and high speed, especially in the first steps of assignments, but they sometimes fail when the potential barrier is low or when reaching higher excited torsional states. Finally one of the differences between *XIAM* and *ERHAM* is that the potential barrier does not appear explicitly in the *ERHAM* method. The information about the barrier is thus hidden in the tunneling coefficients of the internal rotation energy.

For 2-MF, as mentioned by Finneran et al. [12], *ERHAM* computes very quickly and accurately the line positions and intensities up to J=120 and provides much better RMS deviations (108 kHz for v${}_{t}=0$ and 113 kHz for v${}_{t}=1$) than *XIAM* (263 kHz and 16 MHz respectively). *ERHAM* used five and ten tunneling parameters, (the $Bkpr_{q}$ parameters listed in Tables 1 and 2 of Ref. [12] and defined in Refs. [22,27]) to fit v${}_{t}=0$ and 1 respectively. The *XIAM* fit stays of rather poor quality, even after they added four sextic centrifugal distortion, and three rotation-torsion parameters, (see third column of Table 1 [12]). On the other hand, *XIAM* provides a direct value for the potential barrier [21].

Our global fit for 2-MF with *BELGI*-C${}_{s}$ simultaneously includes both the v${}_{t}=0$ and 1 rotational transitions and achieves a similar quality as *ERHAM*. The reserved memory by the *BELGI*-C${}_{s}$ program allows the construction of the Hamiltonian matrix for *J* up to 70 with an Hamiltonian matrix dimension is $\left(2J+1\right)\times 9$. Even though we only assigned transitions of v${}_{t}=0$ and 1, we take into account interactions within the lowest nine torsional states, a truncation usually allowing for the best lowest states energy calculation. The RMS deviations obtained by *BELGI*-C${}_{s}$ are 107 and 114 kHz for v${}_{t}=0$ and 1 respectively. As shown in our Supplementary Table S1, we use in total 21 parameters (plus *F* which is fixed): the three rotational constants *A*, *B* and *C*, the off diagonal inertia moment, $D_{ab}$, the five fourth order centrifugal distortion terms, and only one sextic centrifugal constant ($H_{K}$). We also fit $V_{3}$ and $V_{6}$, ρ the coupling term between internal and global rotation, seven fourth order and one six order rotation-torsion terms.

Compared to the *XIAM* values, our values, after transforming them into the PAM, are in reasonable agreement, considering the difference between the two methods (see Table 1). One of the differences between *XIAM* and *BELGI*-C${}_{s}$ is that *XIAM* fits the three angles ∠(i,x), ∠(i,y) and ∠(i,z) between the internal axis of the methyl group and the principal axes, whereas *BELGI*-C${}_{s}$ fits the $D_{ab}$ term. We have to diagonalize the inertia tensor, using a procedure described in [25], to get the eigenvalues which are the *A*, *B* and *C* values in the PAM. The eigenvectors generated by this diagonalization of the tensor of inertia are related to the angles ∠(i,x), ∠(i,y) and ∠(i,z) reported in Table 1. Compared to *XIAM* our *A*, *B*, *C* parameters in the PAM only differ by 0.0002, 0.05, 0.01 % to *XIAM*. The ∠(i,x), ∠(i,y) and ∠(i,z) angles have also close values, showing that *XIAM* does well with determining the structure, even with a poor quality of fit. Compared to the theoretical B3LYP calculation, the agreement of our PAM values *A*, *B*, *C* from the *BELGI*-C${}_{s}$ code is 0.9, 0.2, 0.4 %. The $V_{3}$ from *BELGI*-C${}_{s}$ only differs by 1.8 % with its value from *XIAM*, and by 11.8 % with the B3LYP calculation, which is also satisfying. We note that a good order of magnitude is obtained as well for $V_{6}$ by the theoretical method compared to the *BELGI*-C${}_{s}$ value. The angle between the PAM axis and the RAM *z* axis is listed in Table 1 as “β” to be coherent with *ERHAM* notation. We called this angle $\theta_{RAM}$ in our previous paper [28] (see Equation (6) of the theoretical section). Its order of magnitude is conserved between *BELGI*-C${}_{s}$ and *ERHAM*, but *ERHAM* has two distinct values for v${}_{t}=0$ and 1. Finally we note that in Table 1, we do not indicate centrifugal distortion terms for *BELGI*-C${}_{s}$. Indeed, their values are fitted in the RAM system, but there is no way to easily convert them into PAM values.

For 3-MF, our global *BELGI*-C${}_{s}$ fit includes 19 parameters (see Table S2 of the Supplementary Materials): like for the 2-MF fit, we performed a fit on *A*, *B*, *C*, $D_{ab}$, the five quartic and one sextic ($H_{K}$) centrifugal distortion constants, the parameter ρ, and the two first Fourier expansion coefficients $V_{3}$ and $V_{6}$ of the potential barrier. For 3-MF, we were able to also fit the *F* parameter and four higher order interaction terms between internal and global rotation. Our RMS deviations (181 kHz and 174 kHz for v${}_{t}=0$ and 1 respectively) are slightly above the averaged measurement accuracy estimated to 150 kHz. Near 4500 rotational transitions involving energy levels up to J=50 have been assigned in the 70–220 GHz scanned range. The 30 lines with low *J* values (18 A species lines and 12 E species lines) from Ogata and Kozima [11] with a weight of 100 kHz were also included to the fit. For those lines, the RMS deviation was 116 kHz. Sixteen lines from [11] showed large observed-calculated values of more than three times the measurement accuracy and we decided to discard them. As indicated in Table 2, for 3-MF the *XIAM* fit was of rather poor quality, with RMSs of 200.6 kHz and 1500 kHz for v${}_{t}=0$ and v${}_{t}=1$ respectively. However our *BELGI*-C${}_{s}$ values for *A*, *B*, *C* transformed in the PAM only differ by 0.005, 0.04, 0.02% with those of *XIAM* in v${}_{t}=0$. The ∠(i,a) angle differs by 0.2%. The $V_{3}$ values differs by 6% and 8% with *XIAM* v${}_{t}=0$ and v${}_{t}=1$, respectively. Again, as for the 2-MF, our PAM values fitted with the *BELGI*-C${}_{s}$ code agree reasonably well with the B3LYP theoretical calculations.

For both the 2- and 3-MF, the threefold torsional potential barrier is rather high ($V_{3}\approx 420\;{\mathrm{cm}}^{-1}$ and $358\;{\mathrm{cm}}^{-1}$ respectively), and the value of the reduced height $s=4V_{3}/\left(9F\right)$ (with the internal rotation constant F≈5.64 cm${}^{-1}$ for 2-MF and F≈5.32 cm${}^{-1}$ for 3-MF) is 33 and 30, respectively. Torsional splittings reach values of ≃15MHz in v${}_{t}=0$ and ≃380MHz in v${}_{t}=1$ states. This classifies them as rather good candidates to apply “local” approaches at least in the v${}_{t}=0$ state. Using the global approach, small internal rotation splittings may result into correlation between the parameters. As it was already observed in other small organic molecules (such as for methyl formate ${\mathrm{HCOOCH}}_{3}$ [29]), the assignment was complicated by two main factors: (i) 2-MF and 3-MF have two small-amplitude vibrations [12]: the out-of-plane bendings of the methyl group at 237 and 267 cm${}^{-1}$, respectively and the in-plane bending mode at 339 and 321 cm${}^{-1}$, respectively. These modes are lying below the barrier and therefore are expected to cause some perturbations on the rotation-torsional energy levels. (ii) The value of the ρ parameter which represents the coupling term between internal rotation and global rotation in the kinetic energy operator is only 0.056. This low value does not allow us to get much information about the torsion-rotation coupling. In this complicated and rich room-temperature spectra of internal rotors, it is thus very useful to have various tools to first analyze the spectra (using the “local” codes which are very efficient and easy to use), and then fit globally the torsional manifold (with the “global” codes which are very powerful in predicting higher torsional states that can be populated). It is clear that so far no automatic procedure can really handle the complicated torsional-rotational manifold, and even more the vibrational-rotational-torsion manifold. A new code is currently built by Ilyushin and collaborators towards that goal [30].

### 3.2. Influence of the Isomerism on the Internal Rotation of the Methyl Group, Comparison with Other Heterocyclic Compounds

**Figure 4:**
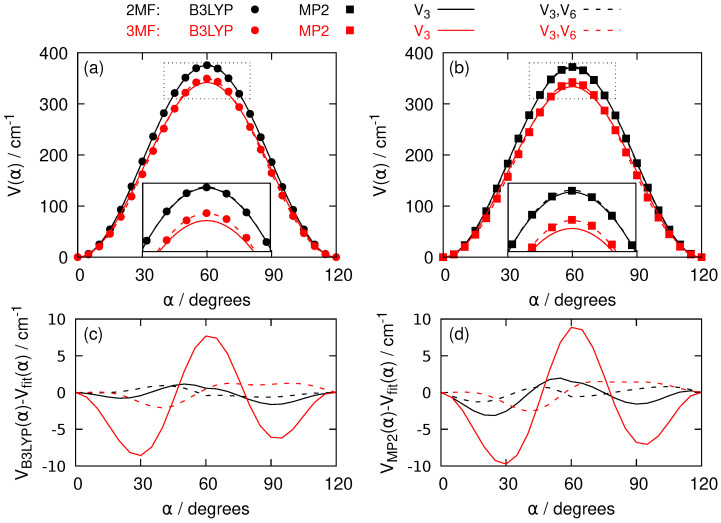
(**a**) Torsional potential *V* as a function of the torsional angle α for 2-MF (black dots and curves) and 3-MF (red dots and curves). The electronic energies from B3LYP calculations with the aug-cc-pVTZ basis set are represented by dots. The solid curves come from the B3LYP data after a fit on $V_{3}$ and fixing $V_{6}$ to zero in Equation (5). The dashed curves are obtained after a fit on both $V_{3}$ and $V_{6}$ in Equation (5). A blow-up around the maximum of the curves is pictured in the inset. (**b**) Same as (**a**) for MP2 data. The MP2 electronic energies are shown as squares. (**c**) Energy differences between the B3LYP results and the adjusted curves with the same color and style code as (**a**). (**d**) Same as (**c**) for MP2 data.

The hindered internal rotation of the methyl group in 2-MF and 3-MF is due to the one-dimensional torsional potential. The first two terms of its threefold Fourier series, see Equation (5), introduce a $V_{3}$ and a $V_{6}$ parameter. Quantum chemistry calculations based on Density Functional Theory (DFT) B3LYP method and the single reference Møller-Plesset perturbation theory at second order (MP2) method give the dependence of the energy of the electronic ground state on the torsional angle α. Figure 4 indicates that B3LYP and MP2 methods provide nearly identical barriers with slightly higher value with B3LYP. Furthermore, the barrier is lower in 3-MF than in 2-MF. This lowering of the barrier might be interpreted by the larger distance between the oxygen atom and the methyl group which slightly flattens the torsional potential. While the fits with just the $V_{3}$ parameter already qualitatively reproduce the data, the fits are improved by adding the $V_{6}$ term in the series expansion, in particular near the top of the barrier as it can be seen in the insets of Figure 4a,b and confirmed by the differences between the computed electronic energies and the fitted ones in Figure 4c,d.

The values of $V_{3}$ and $V_{6}$ obtained from quantum chemical calculations can be compared with the spectroscopic parameters of Table 1 for 2-MF and Table 2 for 3-MF obtained by fitting the experimental line positions. We observe that the $V_{3}$ parameter determined from quantum computations underestimates the XIAM or BELGI-Cs values by 10% for 2-MF (3% for 3-MF) while the $V_{6}$ parameter (in absolute value) underestimates the fitted values by a factor 5 or more for 2-MF (overestimates by a factor 4 for 3-MF). Such a disagreement is not surprising, because the $V_{3}$ and $V_{6}$ parameters obtained from the *XIAM* and *BELGI*-C${}_{s}$ codes consider the averaging over the harmonic motion of the nuclei belonging to the furan frame and of the zero-point energy in the torsional potential.

Because the previous measurements on 2-MF and 3-MF were focused on the ground state [11] or treat the v${}_{t}=0$ and v${}_{t}=1$ states independently in a local approach [12], those prior experimental studies dedicated to large amplitude internal rotation were only able to fit the $V_{3}$ barrier height without the higher-order $V_{6}$ correction. That was also the case for other heterocyclic analogs of methylfuran (MF) as methylthiophene (MT) and methylpyrrole (MP). In Table 3, we notice that the $V_{3}$ parameters were experimentally determined for the 2- and 3- isomers of each heterocyclic compounds but the higher-order $V_{6}$ correction could not be determined before our study of 2-MF and 3-MF mm-wave spectra since it requires the access to the v${}_{t}=1$ excited torsional state and a global fit including both v${}_{t}=0$ and v${}_{t}=1$ allowed by the *BELGI*-C${}_{s}$ code. Therefore no $V_{6}$ experimental values exist for the other heterocyclic compounds and the comparison should be performed with $V_{3}$ and $V_{6}$ values fitted on the theoretical data (hereafter B3LYP/aug-cc-pVTZ calculations).

The internal rotation potential and particularly the $V_{3}$ parameter is sensitive to both steric and electronic effects. In heterocyclic compounds, electronic effects are usually dominant [32,34]. When we compare the $V_{3}$ values of the three heterocyclic compounds, we can observe that: first, the variation of $V_{3}$ is stronger when the methyl group is substituted to the second position (2-MF, 2-MT and 2-MP) compared to its third position (3-MF, 3-MT and 3-MP); second, $V_{3}$ is larger for MF compared to MT and MP (${\left(V_{3}\right)}_{MF}>{\left(V_{3}\right)}_{MP}>{\left(V_{3}\right)}_{MT}$ for the *2-* position and ${\left(V_{3}\right)}_{MF}>{\left(V_{3}\right)}_{MP}≃{\left(V_{3}\right)}_{MT}$ for the *3-* isomers) since a larger electro-negativity of the oxygen which creates an electronic environment hindering the internal methyl rotation.

Concerning the $V_{6}$ term listed in Table 3, we observe larger values for the 3- isomers compared to the 2- ones as if the electronic effects tend to reduce the anharmonicity of the potential. Moreover we can observe negative $V_{6}$ values for the three 3- isomers where $|V_{6}/V_{3}|>3\%$. These negative $V_{6}$ are generally observed for internal rotation potential with significant anharmonicity, we can cite as example molecules such as acetaldehyde [35], methyl ketene [36] or meta-cresol [37].

## 4. Materials and Methods

### 4.1. Theoretical Methods

#### 4.1.1. Quantum Chemistry Calculations

The quantum chemical investigations on 2-MF and 3-MF were performed with the Gaussian 16 package [38]. The B3LYP [39] functional was used in the DFT calculations with the default ultrafine grid. We also performed molecular orbital based calculations with MP2 [40]. All our results were obtained with the correlation consistent basis set aug–cc–pVTZ [41]. The convergence criteria of the geometry optimisations of both 2-MF and 3-MF isomers were set to the *tight* option. The one dimensional energy curves describing the electronic energy as a function of the internal rotation of the methyl group are presented on Figure 4 and all results are listed in Table 3. Each energy value is the result of a geometry optimization on all the coordinates except the frozen angle describing the internal rotation. A step size of $5^{\circ }$ was chosen between two successive angles.

#### 4.1.2. Internal Rotation Hamiltonian

There exist several approaches and codes to treat rotational spectra of molecules containing one or two internal rotor(s). Codes are available at the “Programs for ROtational SPEctroscopy” (PROSPE) [42], managed by Z. Kisiel. Among them, the three codes we are using in the present work, i.e., the program *XIAM* written by Hartwig and Dreizler [21] which can handle up to three internal rotors, the program called the “ERHAM” (*ERHAM*) developed by P. Groner for one or two-tops [22,27] and the *BELGI* series of codes developed by Hougen and Kleiner for asymmetric tops containing one or two internal rotors, with $C_{s}$ [43] (*BELGI*-C${}_{s}$) and $C_{1}$ (*BELGI*-C${}_{1}$) [29] symmetries. Finally, Ilyushin has developed the *RAM36* code for one-top in the same RAM approach [44].

The Hamiltonian used in the *BELGI*-C${}_{s}$ code is the RAM internal-rotation Hamiltonian based on the work of Kirtman [45], Lees and Baker [46], and Herbst et al. [47]. *XIAM* and *ERHAM* can be qualified as “combined axis methods” (CAM) because they set up the Hamiltonian (or parts of it) in the Rho-Axis-System (RAS) but then apply a transformation back to the PAS. *ERHAM* and *XIAM* allow to fit each torsional state separately (neglecting thus matrix elements off-diagonal in the torsional quantum number v${}_{t}$). We thus classify these two methods as “*local*” ones because they fit each torsional state by itself, even though they treat the A and E states together. In the *BELGI* code, all the torsional states, up to a given truncation level are taken into account and fit together, so we call it a “global” method. This global approach is particularly successful for low barriers or high excited torsional states.

Since rather complete descriptions of these methods already exist in the literature [48], we will not repeat here such a general description. We only emphasize here the various characteristics that the three methods and programs had to face for the present study.

The RAM is a choice of an axis system which eliminates in the rotation-torsion Hamiltonian the Coriolis type terms (the *x*, *y*, *z* axis are related to *b*, *c*, *a* for prolate molecules. For an asymmetric top with a plan of symmetry as the case here, the Coriolis cross term $-2F\rho_{y}p_{\alpha }J_{y}$ does not exist because of symmetry restrictions.) $-2F\rho_{x}p_{a}J_{x}$ and $-2F\rho_{y}p_{\alpha }J_{y}$, where $J_{x}$ and $J_{y}$ designate the components of the rotational angular momentum in the molecular axis system, $p_{\alpha }$ is the internal rotational angular momentum, *F* is the internal rotation constant and ρ is the coupling constant. The RAM Hamiltonian may be written as [47]:

(1) $$H_{RAM}=H_{T}+H_{R}+H_{cd}+H_{int}$$

where $H_{T}$ is the torsional Hamiltonian, $H_{R}$ the rotational Hamiltonian, $H_{cd}$ the usual centrifugal distortion Hamiltonian, and $H_{int}$ contain higher order torsional-rotational interaction terms:

(2) $$H_{T}=F{\left(\rho_{a}-\rho J_{z}\right)}^{2}+V\left(\alpha \right)$$

(3) $$H_{R}=A{\left(\cos\theta_{RAM}J_{z}-\sin\theta_{RAM}J_{x}\right)}^{2}+B{\left(\sin\theta_{RAM}J_{z}+\cos\theta_{RAM}J_{x}\right)}^{2}+CJ_{y}^{2}$$

For this prolate asymmetric top, the reduction *A* and the representation $I^{r}$ were chosen. By grouping the terms and if *x*, *y*, *z* → *b*, *c*, *a* we get:

(4) $$H_{R}=A_{RAM}J_{a}^{2}+B_{RAM}J_{b}^{2}+C_{RAM}J_{c}^{2}+D_{ab}\left(J_{a}J_{b}+J_{b}J_{a}\right)$$

The potential function which hinders the internal rotation of the methyl group is described by a threefold Fourier series depending on the torsional angle α:

(5) $$V\left(\alpha \right)=\frac{V_{3}}{2}\left(1-\cos3\alpha \right)+\frac{V_{6}}{2}\left(1-\cos6\alpha \right)+\ldots$$

The relation between the rotational constants *A*, *B*, *C* in the principal axis system and the constants in the Rho Axis system is given by the angle $\theta_{RAM}$ which turns the PAS into the RAS:

(6) $$\tan2\theta_{RAM}=\frac{2D_{ab}}{A_{RAM}-B_{RAM}}$$

From a fit of the spectrum, the following parameters are derived from the *BELGI*-$C_{s}$ code: $V_{3}$, *F*, $A_{RAM}$, $B_{RAM}$, $C_{RAM}$, $D_{ab}$ and ρ (plus higher-order parameters).

In the *ERHAM* code [22,27], the overall rotation-internal rotation Hamiltonian is set up in a product basis of symmetric rotor eigenfunctions with eigenfunctions of the internal rotation Hamiltonian set up in a Rho Axis system. It is assumed that the internal rotation Hamiltonian has already been solved. In the matrix elements of the effective Hamiltonian, the integrals over the internal rotation variables are expressed as Fourier series. Their coefficients are called “tunneling coefficients”, and they are the fitting parameters of the effective rotational Hamiltonian in *ERHAM*.

In the *XIAM* code [21], the Hamiltonian is set up in the principal axes system of the entire molecule but then the internal rotation operator of each top is set up in its own Rho Axis system (RAS) and after diagonalization, the resulting eigenvalues are transformed (rotated) back into the principal axis system. The fitted parameters are the rotational constants *A*, *B*, *C*, the centrifugal distortion constants up to the 6th order, and for each methyl top the direction of the top axis in the PAM system ∠(i,x), ∠(i,y), ∠(i,z) the internal rotation constant *F* and the potential barrier height $V_{3}$. Only some higher (4th order) coupling terms between internal rotation and overall rotation are sometimes implemented, and each torsional state is fit by itself.

### 4.2. Room Temperature Millimeter-Wave Spectroscopy

The Doppler limited room temperature mm-wave absorption spectra of 2- and 3-MF in the range of 70–110 GHz and 170–220 GHz were recorded at the LPCA laboratory using the solid state THz spectrometer described in detail in Ref. [20]. The measurements were performed in flux conditions with a low pressure of about P=5Pa allowing to limit the consumption of MF and to reduce the collisional broadening up to the Doppler broadening limit ($\Delta \nu_{\mathrm{Doppler}}∼$ 150 kHz (FWHM) at ν≃ 100 GHz and T≃300 K). Due to the high volatility of MFs ($P_{vap}≃200$ hPa for 2-MF and $P_{vap}≃100$ hPa for 3-MF at 300 K), the samples of 2-MF and 3-MF were cooled with a mixture of ice and liquid nitrogen and the flows were regulated with a micro-valve. The spectra were recorded using a combination of a frequency multiplier chain (Virginia Diodes, Inc., Charlottesville, VA, USA) exciting the molecule and a ZBD, which was an unbiased Schottky diode mounted in a wave-guide operating in detection mode.

After up-converting the synthesized microwave frequency by the multiplication chain the guided radiation is launched into free space using a horn antenna, collimated by an off-axis parabolic mirror, passing a polarization grid and propagated through a 125cm long and 5.6cm diameter stainless steel absorption cell closed by two Teflon windows. The interaction path-length is doubled [49] with a roof-top reflector positioned at the output of the gas cell. The reflected beam is returned to the output parabolic mirror which ensures the focusing onto the detector. The second harmonic of the frequency modulated signal were recorded in 100 kHz steps with a time constant of 100 ms using a modulation depth of 50 kHz and a modulation frequency of 43 kHz delivered by the frequency synthesizer.

A post treatment with a FFT band-pass filter cuts off lower frequencies and the noisy higher frequency parts of the spectra reducing the noise level and removing the low frequency baseline variations caused by stationary waves between the source, the polarization grid, the cell, and the detector [28].

## 5. Conclusions

2-MF and 3-MF isomers are oxygenated VOCs emitted from multiple sources in the atmosphere especially natural or industrial biomass combustion processes. Their atmospheric reactivity leads to the formation of SOAs which directly or indirectly affect the climate. Mm-wave spectroscopy is an interesting approach to monitor them but it requires a prior determination of reliable rotational-torsional line lists. In this study, we propose a global approach using the *BELGI*-C${}_{s}$ code providing a unique set of fitted parameters able to reproduce, at the experimental accuracy, the 2-MF and 3-MF observed mm-wave lines belonging to the ground v${}_{t}=0$ and the first torsional v${}_{t}=1$ states up to high *J* and *K* values. Unlike 2-MF for which our assignment was based on previous measurements performed by Finneran et al. in Ref. [12], the mm-wave spectrum of 3-MF has been measured in this study in the 70–220 GHz frequency range with the mm-wave spectrometer developed at the LPCA [20]. For both isomers we compared our global approach using *BELGI*-C${}_{s}$ with local approaches based on the *XIAM* and *ERHAM* codes. Two advantages of the *BELGI*-C${}_{s}$ global approach are highlighted: (i) the ability to fit at the experimental accuracy the A and E components involving both v${}_{t}=0$ and v${}_{t}=1$ states (*XIAM* failed to fit correctly rotational lines of 2-MF and 3-MF in v${}_{t}=1$); (ii) the possibility to determine with a high degree of accuracy the $V_{3}$ and $V_{6}$ parameters of the internal rotation potential (in the *ERHAM* method, the potential barrier does not appear explicitly and $V_{6}$ could not be determined). Based on DFT calculations, these ($V_{3}$, $V_{6}$) parameters are discussed considering other sulfur and nitrogen heterocyclic analog in order to better understand the influence of the electronic environment on the internal rotation barrier and its higher-order correction term. To confirm experimentally the identified trends, we propose to investigate in the mm-wave domain the rotational-torsional spectroscopy of MP and MT already studied in the microwave domain [31,32,33,34]. Finally, in order to assign the remaining mm-wave lines of 2-MF and 3-MF, a rovibrational analysis of the lowest energy vibrational modes will be undertaken by means of synchrotron-based FT-Far-IR high-resolution spectroscopy as it was done recently by our group on the catechol molecule [15].

## Figures and Tables

**Table 1:** Torsion-rotation constants of 2-MF for the v${}_{t}=0$ and v${}_{t}=1$ states obtained from DFT calculations (this work), from the fits of microwave and mm-wave data with local *XIAM* and *ERHAM* approaches (Ref. [12]) and global *BELGI*-C${}_{s}$ approach (this work). The complete set of parameters obtained for *XIAM* and *ERHAM* can be found in Tables 1 and 2 of Ref. [12]. The complete set of parameters obtained for *BELGI*-C${}_{s}$ is given in the Supplementary Table S1. The numbers in parentheses represent 1σ standard deviation in units of the last significant digit. All parameters are in the Principal Axis System (PAS).

	Unit	*B3LYP* ${}^{a}$	*XIAM* v${}_{t}=0$	*ERHAM* v${}_{t}=0$ ${}^{b}$	*ERHAM* v${}_{t}=1$ ${}^{b}$	*BELGI-C*${}_{s}$ v${}_{t}=0;1$ ${}^{c}$
		Present Work	Ref. [12]	Ref. [12]	Ref. [12]	Present Work
A	MHz	8871.678	8792.22489 (33)	8791.54486 (12)	8790.0922 (18)	8792.209 (28)
B	MHz	3548.740	3542.64071 (20)	3543.321804 (46)	3540.94203 (22)	3544.381 (32)
C	MHz	2575.200	2565.560151 (38)	2565.603243 (36)	2564.946450 (65)	2565.8202 (91)
$\Delta_{J}$	kHz	0.26076	1.539273 (68)	0.2645388 (58)	0.268855 (50)	
$\Delta_{JK}$	kHz	1.3921	−2.41323 (31)	1.408544 (66)	1.8300 (31)	
$\Delta_{K}$	kHz	0.991	0.99269 (31)	1.000997 (83)	−1.947 (21)	
$\delta_{J}$	kHz	0.071346	0.564919 (32)	0.0724207 (24)	0.074930 (24)	
$\delta_{K}$	kHz	0.7186	−0.46139 (17)	0.687123 (86)	0.6824 (14)	
$\Phi_{JK}$	mHz		−0.669 (45)	−0.6653 (83)	−2.852 (63)	
$\Phi_{KJ}$	mHz		0.568 (50)	[0.0]	[0.0]	
$\Phi_{K}$	mHz		[0.0]	1.882 (20)	−7.01 (64)	
$\varphi_{JK}$	mHz		−0.502 (29)	[0.0]	[0.0]	
ρ	unitless	0.0549	0.0549511 (44)	0.0550406 (14)	0.0554463 (90)	0.0551544 (10)
*F*	GHz	170.257	169.30759 ${}^{d}$			169.0829 ${}^{d}$
$V_{3}$	${\mathrm{cm}}^{-1}$	376.12(22 ^*e*^)	412.873 (74)			420.3157 (35)
$V_{6}$	${\mathrm{cm}}^{-1}$	−1.49(22 ^*e*^)				−9.010 (11)
$\epsilon_{1}$	MHz			−119.459 (11)	3781.55 (72)	
∠(i,a)	degree	4.48	3.30 (13)			4.6345 (53)
∠(i,b)	degree	85.52	86.70 (13)			85.3655 (83)
∠(i,c)	degree	90.00	90.009 (18)			90.00
β	degree			1.908 (13)	2.1785 (31)	1.871
$s^{\;f}$	unitless	29.435	32.492			33.1218
$J_{max}$			95	120	117	70
$K_{a,max}$			53	54	15	53
$N(v_{t}=0)$ (A; E)			8006 ${}^{h}$ (2495; 5511)	11793 ${}^{i}$		14152 ${}^{g}$ (7766; 6386)
$N(v_{t}=1)$ (A; E)					2580 ${}^{i}$	3742 ${}^{g}$ (1836; 1906)
WRMS ${}^{j}$	unitless		1.754	0.718	0.753	1.0733; 1.1431
RMS ${}^{k}$	kHz		263	108	113	107; 114

^*a*^ Calculation with the aug-cc-pVTZ basis set. ^*b*^ The complete set of higher order tunneling terms used in *ERHAM* are listed in Tables 1 and 2 of Ref. [12]. ^*c*^ Parameters in the PAS after transforming them from the RAM. The complete set of RAM parameters including centrifugal distortion and higher order terms used in our fit are listed in Table S1 of the supplementary data. ^*d*^ In *XIAM F* is a derived parameter from *F*_0_, see supplementary data in Ref. [12]. In *BELGI*-C_*s*_ *F* was fixed to the best value obtained by fitting it. ^*e*^ Standard error of the analytical fit based on Equation (5) as given by Gnuplot [24]. ^*f*^ Reduced barrier height: *s* = 4*V*_3_/(9*F*). ^*g*^ *N* corresponds to the number of assigned transitions for v_*t*_ = 0 and v_*t*_ = 1. In the *BELGI*-C_*s*_ fit 14,152 and 3742 correspond to the number of transitions fitted. ^*h*^ In *XIAM* only 8006 unblended lines were included in the fit. ^*i*^ In the *ERHAM* fit 11,793 and 2580 correspond to the number of distinct frequencies used for the fits of Ref. [12]. ^*j*^ WRMS is the weighted unitless root mean square deviation of the fit: $\sqrt{\frac{1}{N}\sum_{i}{\left(\frac{f_{i}^{obs.}-f_{i}^{calc.}}{\Delta_{i}}\right)}^{2}}$ with *N* the total number of observed ($f_{i}^{obs.}$) and calculated ($f_{i}^{calc.}$) frequencies and $\Delta_{i}$ the estimated error on the frequency measurement. ^*k*^ RMS is the Root mean square deviation of the fit in kHz.

**Table 2:** Torsion-rotation constants of the 3-MF obtained from the mm-wave (70–220 GHz) analysis using the *XIAM* code for v${}_{t}$ = 0 and v${}_{t}$ = 1 separately, and *BELGI*-C${}_{s}$ with a global fit analysis. These values are compared with quantum chemistry calculations (B3LYP method) and with the microwave analysis performed by Ogata et al. [11]. The numbers in parentheses represent 1σ standard deviation in units of the last significant digit. All parameters are in the principal axes system.

	Unit	*B3LYP* ${}^{a}$	Ref. [11]	*XIAM* v${}_{t}$ = 0	*XIAM* v${}_{t}$ = 1	*BELGI*-C${}_{s}$ v${}_{t}=0,\;1$ ${}^{b}$
*A*	MHz	8956.659	8890.83 (15) ${}^{k}$	8890.9101 (10)	8891.799 (11)	8890.483 (14)
*B*	MHz	3379.791	3366.91 (5) ${}^{k}$	3367.05272 (32)	3364.7173 (25)	3368.235 (13)
*C*	MHz	2491.517	2479.32 (5) ${}^{k}$	2479.20065 (31)	2478.2235 (23)	2479.69788 (28)
$\Delta_{J}$	kHz	0.24058		0.24309 (12)	0.24004 (90)	
$\Delta_{JK}$	kHz	1.5663		1.59104 (88)	1.6429 (25)	
$\Delta_{K}$	kHz	0.97		1.4636 (30)	1.717 (56)	
$\delta_{J}$	kHz	0.06369		0.064188 (34)	0.06267 (30)	
$\delta_{K}$	kHz	0.7979		0.7466 (26)	0.4967 (45)	
$\Phi_{KJ}$	mHz			−18.1 (13)	−18.1 ${}^{c}$	
$\varphi_{jk}$	mHz			−12.1 (12)	−12.1 ${}^{c}$	
ρ	unitless	0.0554		0.055676201 ${}^{d}$	0.055620821 ${}^{d}$	0.05564643 (33)
*F*	GHz	171.195	167.8	169.1046 ^*e*^	169.2800 ^*e*^	159.541(78)
∠(i,a)	degree	179.43	179.9438	179.9425	179.9425	179.495 (39)
∠(i,b)	degree	89.43	89.9438	89.9425	89.9425	89.483 (38)
∠(i,c)	degree	90.00	89.999866	90.0000	90.0000	90.000
$V_{3}$	${\mathrm{cm}}^{-1}$	348.92 (50 ${}^{f}$)	381 (1)	382.032 (44)	385.4706 (44)	357.77(22)
$V_{6}$	${\mathrm{cm}}^{-1}$	−10.81(50 ${}^{f}$)				−2.481 (13)
s ${}^{g}$	unitless	27.156	30.22 (8)	30.101	30.3406	29.879 (17)
$J_{max}$				50	50	50
$K_{a,max}$				31	31	31
*N*(v${}_{t}$=0) (A; E) ${}^{h}$			36 (20; 16)	2463 (1296; 1167)		2463 (1296; 1167)
*N*(v${}_{t}$=1) (A; E) ${}^{h}$			0		2016 (1003; 1013)	2017 (1003;1014)
WRMS (v${}_{t}$ = 0; v${}_{t}$ = 1) ${}^{i}$	unitless			1.3	10.0	1.21; 1.16
RMS (v${}_{t}$ = 0; v${}_{t}$ = 1) ${}^{j}$	kHz			200.6	1500	181; 174

^*a*^ Calculation with the aug-cc-pVTZ basis set. ^*b*^ The complete set of RAM parameters including centrifugal distortion and higher order terms used in our fit are listed in Table S2 of the supplementary data. Parameters were transformed from RAM to PAM system. ^*c*^ Taken from the v_*t*_ = 0 *XIAM* fit. ^*d*^ Derived from *F*. ^*e*^ In *XIAM F* is derived from *F*_0_, fitted to 159.6895(10) GHz for v_*t*_ = 0 and 159.8645(13) GHz for v_*t*_ = 1. ^*f*^ Standard error of the analytical fit based on Equation (5) as given by Gnuplot [24]. ^*g*^ Reduced barrier height: *s* = 4*V*_3_/(9 × *F*). ^*h*^ *N* corresponds to the number of assigned transitions for v_*t*_ = 0 and v_*t*_ = 1. ^*i*^ WRMS is the weighted unitless root mean square deviation of the fit: $\sqrt{\frac{1}{N_{b}}\sum_{i}{\left(\frac{f_{i}^{obs.}-f_{i}^{calc.}}{\Delta_{i}}\right)}^{2}}$ where *N_b_* is the number of distinct observed ($f_{i}^{obs.}$) and calculated ($f_{i}^{calc.}$) frequencies, for v_*t*_ = 0 *N_b_* = 1845, for v_*t*_ = 1 *N_b_* = 1538 and $\Delta_{i}$ is the measurement uncertainty. ^*j*^ RMS is the root mean square deviation of the fit in kHz. ^*k*^ Previous fit of Ogata et al. [11] using only A species transitions and refined from A–E splitting analysis with *A_A_* − *A* = 2.33 MHz and *B_A_* − *B* = 0.00 MHz.

**Table 3:** $V_{3}$ and $V_{6}$ barriers for 2- and 3- methylfuran (MF), methylthiophene (MT) and methylpyrrole (MP) calculated with B3LYP/aug-cc-pVTZ as the function of the methyl groups torsional angle α. Uncertainties are the standard error given by Gnuplot [24] for the fit of the DFT potential based on Equation (5). The experimental values determined in this work for MF or in the literature for MT and MP are mentioned in square brackets.

Molecule	$V_{3}$ /cm${}^{-1}$	$V_{6}$ /cm${}^{-1}$	100 $V_{6}/V_{3}$
2-MF	376.12 (22) [420.3157 (35)] ${}^{a}$	−1.49 (22) [−9.010 (11)] ${}^{a}$	−0.40 [−2.143]
2-MT	198.08 (33) [194.1] ${}^{b}$	1.71 (33)	0.86
2-MP	263.62 (67) [279.7183 (26)] ${}^{c}$	1.96 (67)	0.74
3-MF	348.92 (50) [357.77 (22)] ${}^{a}$	−10.81 (50) [−2.481 (13)] ${}^{a}$	−3.10 [−0.693]
3-MT	254.47 (20) [259] ${}^{d}$	−8.27 (20)	−3.25
3-MP	223.55 (34) [245.14101 (89)] ^*e*^	−7.71 (34)	−3.45

^*a*^ Experimental value obtained from *BELGI*-C_*s*_. ^*b*^ Experimental value taken from [31]. ^*c*^ Experimental value taken from [32]. ^*d*^ Experimental value taken from [33]. ^*e*^ Experimental value taken from [34].

## Data Availability

The data presented in this study is available in the Supplementary Materials.

## Supplementary Materials

The following supporting information can be downloaded at https://www.mdpi.com/article/10.3390/molecules27113591/s1, a directory *“supp-mat”* of 4 Tables: S1 and S2 contain the full set of RAM parameters fitted *BELGI*-C${}_{s}$, respectively for 2-MF and 3-MF. S3 and S4 are the produced rotational-torsional mm-wave line lists, respectively for 2-MF and 3-MF.

## The Following Abbreviations Are Used in This Manuscript

B3LYP	Becke, 3-parameter, Lee-Yang-Parr
CAM	Combined Axis Method
DFT	Density Functional Theory
ERHAM	Effective Rotational-torsional HAMiltonian
FFT	Fast Fourier Transform
GC-MS	Gas Chromatography—Mass Spectrometry
GS	Ground State
IR	InfraRed
MF	Methyl Furan
MP	Methyl Pyrrole
MT	Methyl Tiophene
MP2	Møller-Plesset perturbation theory at 2nd order
PAM	Principal Axis Method
PAS	Principal Axis System
RAM	Rho Axis Method
RAS	Rho Axis System
RMS	Root Mean Square
WRMS	Weighted Root Mean Square
RAS	Rho Axis System
SOA	Secondary Organic Aerosol
VOC	Volatile Organic Compound
XIAM	eXtended Internal Axis Method
ZBD	Zero Bias Detector
