# Supplementary material for: Millimeter-Wave Spectroscopy of Methylfuran Isomers: Local vs. Global Treatments of the Internal Rotation
Source: Molecules. 2022 Jun 2;27(11):3591. doi: 10.3390/molecules27113591 (PMC9182110; doi:10.3390/molecules27113591)
Supplement: Supplementary file 1 [file molecules-27-03591-s001.zip › S1 table 2MF.pdf]

Supporting Informations - table S1

Table S1: Molecular constants in the RAM system of 2-methylfuran obtained by a global fit using program BELGI-C_s.

Operator[a]	Constant[b]	Unit	Present work[c]
P_a^2	A	cm^{-1}	0.29308980(67)
P_b^2	B	cm^{-1}	0.11841455(81)
P_c^2	C	cm^{-1}	0.08558655(30)
$\{P_a, P_b\}$	D_{ab}	cm^{-1}	-0.005714(10)
$-P^4$	Δ_J	cm^{-1}	0. 877042(31)*10^{-8}
$-P^2P_a^2$	Δ_{JK}	cm^{-1}	0.4673(24)*10^{-7}
$-P_a^4$	Δ_K	cm^{-1}	0.35765(32)*10^{-7}
$-2P^2(P_b^2 - P_c^2)$	δ_J	cm^{-1}	0.238514(17)*10^{-7}
$-\{P_a^2, (P_b^2 - P_c^2)\}$	δ_K	cm^{-1}	0.35765(32)*10^{-7}
P_α^2	F	cm^{-1}	5.64^{fixed}
$(1/2)(1 - cos(3\alpha))$	V_3	cm^{-1}	420.3157(35)
$(1/2)(1 - cos(6\alpha))$	V_6	cm^{-1}	-9.010(11)
P_aP_γ	ρ	unitless	0.0551544(10)
$(1 - cos3\alpha)P_a^2$	k_5	cm^{-1}	0.1363(14)*10^{-3}
$(1 - cos3\alpha)P^2$	F_v	cm^{-1}	-0.168(25)*10^{-3}
$(1 - cos3\alpha)(P_b^2 - P_c^2)$	c_2	cm^{-1}	-0.48350(37)*10^{-4}
$P_\alpha^2\{P_a, P_b\}$	Δ_{ab}	cm^{-1}	-0.5554(40)*10^{-5}
$P_aP_\alpha P^2$	L_v	cm^{-1}	0.1226(71)*10^{-6}
$P_\alpha^2P^2$	G_v	cm^{-1}	-0.1087(56)*10^{-5}
P_α^4	k_4	cm^{-1}	-0.2702(27)*10^{-3}
$(1 - cos6\alpha)P^2$	N_v	cm^{-1}	0.296(20)*10^{-4}
P_a^6	H_K	cm^{-1}	0.35(10)*10^{-13}
$N_{\nu t=0}/N_{\nu t=1}/N_{tot}$			14152/ 3742/ 17894
$\sigma_{\nu t=0}/\sigma_{\nu t=1}/\sigma_{tot}$		kHz	107.3/ 114.3/ 108.8

[a] All constants refer to a rho-axis system, therefore the inertia tensor is not diagonal and the constants cannot be directly compared to those of a principal axis system. P_a, P_b, P_c are the components of the overall rotation angular momentum, P_γ is the angular momentum of the internal rotor rotating around the internal rotor axis by an angle γ. {u,v} is the anti commutator uv + vu.

[b] The product of the parameter and operator from a given row yields the term actually used in the vibration-rotation-torsion Hamiltonian, except for F, ρ and A, which occur in the Hamiltonian in the form $F(P_\gamma - \rho P_a)^2 + AP_a^2$.

$N_{\nu t=0}/N_{\nu t=1}/N_{tot}$ are the numbers of $\nu_t = 0$ transitions, $\nu t = 1$ transitions and the total number of transitions.

$\sigma_{\nu t=0}/\sigma_{\nu t=1}/\sigma_{tot}$ are the respective standard deviations.

[c] Values of the parameters from the present fit. Statistical uncertainties are shown as one standard uncertainty in the last digit.
