# Supplementary material for: Millimeter-Wave Spectroscopy of Methylfuran Isomers: Local vs. Global Treatments of the Internal Rotation
Source: Molecules. 2022 Jun 2;27(11):3591. doi: 10.3390/molecules27113591 (PMC9182110; doi:10.3390/molecules27113591)
Supplement: Supplementary file 1 [file molecules-27-03591-s001.zip › S2 table 3MF.pdf]

Supporting Informations - table S2

Table S2: Molecular constants in the RAM system of 3-methylfuran obtained by a global fit using program BELGI-C_s.

Operator[a]	Constant[b]	Unit	Present work[c]
P_a^2	A	cm^{-1}	0.29655253(32)
P_b^2	B	cm^{-1}	0.11235429(31)
P_c^2	C	cm^{-1}	0.0827138179(94)
$\{P_a, P_b\}$	D_{ab}	cm^{-1}	-0.000615(48)
$-P^4$	Δ_J	cm^{-1}	0.8094(27)*10^{-8}
$-P^2P_a^2$	Δ_{JK}	cm^{-1}	0.7023(16)*10^{-7}
$-P_a^4$	Δ_K	cm^{-1}	0.4421(39)*10^{-7}
$-2P^2(P_b^2 - P_c^2)$	δ_J	cm^{-1}	0.214090(83) *10^{-8}
$-\{P_a^2, (P_b^2 - P_c^2)\}$	δ_K	cm^{-1}	0.39601(17)*10^{-7}
P_α^2	F	cm^{-1}	5.3217(26)
$(1/2)(1 - cos(3\alpha))$	V_3	cm^{-1}	357.77(22)
$(1/2)(1 - cos(6\alpha))$	V_6	cm^{-1}	-2.481(13)
P_aP_γ	ρ	unitless	0.05564643(33)
$(1 - cos3\alpha)P_a^2$	k_5	cm^{-1}	0.226600(82)*10^{-3}
$(1 - cos3\alpha)P^2$	F_v	cm^{-1}	-0.12522(16)*10^{-3}
$(1 - cos3\alpha)(P_b^2 - P_c^2)$	c_2	cm^{-1}	-0.527100(72)*10^{-4}
$P_\alpha^2\{P_a, P_b\}$	Δ_{ab}	cm^{-1}	——
$P_aP_\alpha P^2$	L_v	cm^{-1}	0.3885(29)*10^{-6}
$P_\alpha^2P^2$	G_v	cm^{-1}	-0.8908(60)*10^{-6}
P_α^4	k_4	cm^{-1}	——
$(1 - cos6\alpha)P^2$	N_v	cm^{-1}	——
P_a^6	H_K	cm^{-1}	-0.138(75)*10^{-10}
$(1 - cos3\gamma)P_a^2P^2$	k_{5J}	cm^{-1}	0.544 (40)*10^{-9}
$N_{\nu t=0}/N_{\nu t=1}/N_{tot}$[d]			2463/ 2017/ 4480
$\sigma_{\nu t=0}/\sigma_{\nu t=1}/\sigma_{tot}$[d]		kHz	181/ 174/ 178

[a] All constants refer to a rho-axis system, therefore the inertia tensor is not diagonal and the constants cannot be directly compared to those of a principal axis system. P_a, P_b, P_c are the components of the overall rotation angular momentum, P_γ is the angular momentum of the internal rotor rotating around the internal rotor axis by an angle γ. {u,v} is the anti commutator uv + vu.

[b] The product of the parameter and operator from a given row yields the term actually used in the vibration-rotation-torsion Hamiltonian, except for F, ρ and A, which occur in the Hamiltonian in the form $F(P_\gamma - \rho P_a)^2 + AP_a^2$.

[c] Values of the parameters from the present fit. Statistical uncertainties are shown as one standard uncertainty in the last digit. [d] $N_{\nu t=0}/N_{\nu t=1}/N_{tot}$ are the numbers of $\nu t = 0$ transitions, $\nu t = 1$ transitions and the total number of transitions. In the fit 1845 and 1538 distinct frequencies were taken into account for $\nu_t = 0$ state and 1 respectively. 30 transitions from Ogata et al.[?] were weighted 100 kHz and fit with an rms of 116 kHz. Present data was weighted 150 kHz and fit with an rms of 178 kHz.
$\sigma_{\nu t=0}/\sigma_{\nu t=1}/\sigma_{tot}$ are the respective standard deviations.
