# Supplementary material for: Millimeter-Wave Spectroscopy of Methylfuran Isomers: Local vs. Global Treatments of the Internal Rotation
Source: Molecules. 2022 Jun 2;27(11):3591. doi: 10.3390/molecules27113591 (PMC9182110; doi:10.3390/molecules27113591)
Supplement: Supplementary file 1 [file molecules-27-03591-s001.zip › S3 linelist 2MF.pdf]

Supporting Informations - Line list of 2-methylfuran Millimeter-wave spectroscopy of methylfuran isomers: local vs global treatments of the internal rotation

Jonas Bruckhuisen, Sathapana Chawananon, Isabelle Kleiner, Anthony Roucou, Guillaume Dhont, Colwyn Bracquart, Pierre Asselin and Arnaud Cuisset

Table S3: Line list of the rotational transitions frequencies ($\nu_{Obs.}$) of 2-methylfuran measured by Finneran *et al.* (I.A. Finneran, S.T. Shipman, S.L. Widicus Weaver, J. Mol. Spectrosc. 280 (2012), 27-33.) fitted globally for the ground and excited torsional states with an accuracy of 100 kHz using the BELGI-C_s code.

J'	K'_a	K'_c	J''	K''_a	K''_c	Sym.	$v_{t_{CH_3}}$	$\nu_{Obs.}$	$\nu_{Obs.} - \nu_{Calc.}$	E_{low}
upper level			lower level					MHz	MHz	cm^{-1}
30	9	21	30	9	22	A	0	8737.845	-0.060	179.6836
15	7	9	14	8	6	A	0	8746.202	-0.029	101.0577
30	9	21	30	9	22	E	0	8761.228	0.042	179.6904
15	7	8	14	8	6	E	0	8780.521	0.211	101.0704
15	7	8	14	8	7	A	0	8781.616	-0.010	101.0577
15	10	6	16	9	8	E	0	8803.152	-0.012	110.6801
15	7	9	14	8	7	E	0	8812.984	0.014	101.0595
15	10	5	16	9	8	A	0	8852.627	-0.043	110.6798
26	13	14	25	14	11	A	0	8922.112	-0.022	171.4832
26	13	13	25	14	11	E	0	8963.929	0.059	171.4897
26	13	14	25	14	12	E	0	8967.258	-0.023	171.4761
37	19	19	36	20	16	A	0	9166.467	0.004	280.5063
37	19	18	36	20	17	A	0	9166.467	0.004	280.5063
37	19	18	36	20	16	E	0	9215.813	-0.050	280.5037
46	27	20	47	26	21	A	0	9218.190	-0.033	428.0984
46	27	19	47	26	22	A	0	9218.190	-0.033	428.0984
48	25	24	47	26	22	E	0	9433.880	-0.086	428.0859
48	25	24	47	26	21	A	0	9445.063	-0.033	428.0984
48	25	23	47	26	22	A	0	9445.063	-0.033	428.0984
35	21	14	36	20	16	E	0	9455.353	-0.036	280.5037
48	25	23	47	26	21	E	0	9480.695	0.061	428.0882
35	21	15	36	20	17	E	0	9488.381	0.017	280.4935
35	21	15	36	20	16	A	0	9503.377	0.049	280.5063
35	21	14	36	20	17	A	0	9503.377	0.049	280.5063
59	31	28	58	32	26	E	0	9705.930	-0.160	614.2165
24	15	9	25	14	11	E	0	9715.696	-0.055	171.4897
24	15	10	25	14	12	E	0	9718.993	0.061	171.4761
59	31	29	58	32	26	A	0	9743.654	0.053	614.2232
59	31	28	58	32	27	A	0	9743.654	0.053	614.2232
59	31	29	58	32	27	E	0	9750.214	-0.276	614.2101
24	15	10	25	14	11	A	0	9760.001	0.004	171.4832
24	15	9	25	14	12	A	0	9760.001	0.004	171.4832
13	4	9	13	4	10	A	0	9837.935	0.013	89.1446
13	4	9	13	4	10	E	0	9846.783	0.014	89.1553
17	8	10	16	9	7	A	0	9895.986	-0.057	110.6798
17	8	9	16	9	8	A	0	9907.765	-0.039	110.6798
13	9	5	14	8	7	E	0	9919.189	0.019	101.0595
17	8	9	16	9	7	E	0	9922.670	0.011	110.6919
13	9	4	14	8	6	E	0	9947.425	-0.007	101.0704
17	8	10	16	9	8	E	0	9952.734	0.027	110.6801
28	14	15	27	15	12	A	0	10073.075	-0.022	187.9188
28	14	15	27	15	13	E	0	10115.414	-0.045	187.9104
28	14	14	27	15	12	E	0	10117.649	0.031	187.9239
39	20	19	38	21	18	A	0	10320.531	-0.035	303.7510
39	20	20	38	21	17	A	0	10320.531	-0.035	303.7510
44	26	18	45	25	20	E	0	10326.562	-0.060	399.2435
39	20	20	38	21	18	E	0	10336.620	-0.035	303.7379
44	26	18	45	25	21	A	0	10361.330	-0.001	399.2527
44	26	19	45	25	20	A	0	10361.330	-0.001	399.2527
39	20	19	38	21	17	E	0	10369.183	0.065	303.7470
50	26	25	49	27	23	E	0	10584.868	0.014	458.1346
33	20	13	34	19	15	E	0	10591.757	-0.050	258.4640
50	26	24	49	27	23	A	0	10601.115	-0.000	458.1465
50	26	25	49	27	22	A	0	10601.115	-0.000	458.1465
50	26	24	49	27	22	E	0	10632.736	0.082	458.1354
34	10	24	34	10	25	E	0	10634.867	0.038	210.5414
33	20	13	34	19	16	A	0	10640.610	-0.040	258.4650
33	20	14	34	19	15	A	0	10640.610	-0.040	258.4650
10	3	7	10	3	8	A	0	10807.700	0.022	80.2755
22	14	9	23	13	11	E	0	10843.973	0.010	156.2462
22	14	8	23	13	10	E	0	10846.247	-0.022	156.2598
61	32	29	60	33	27	E	0	10860.323	0.155	651.0621
22	14	8	23	13	11	A	0	10887.815	0.020	156.2520
22	14	9	23	13	10	A	0	10887.815	0.021	156.2520
64	37	28	65	36	29	A	0	10898.691	-0.011	755.1714
64	37	27	65	36	30	A	0	10898.691	-0.011	755.1714
61	32	29	60	33	28	A	0	10900.533	-0.029	651.0674
61	32	30	60	33	27	A	0	10900.533	-0.029	651.0674
11	8	4	12	7	6	E	0	11027.206	-0.057	92.6439
19	9	11	18	10	8	A	0	11042.112	-0.012	121.5068
19	9	10	18	10	9	A	0	11045.941	-0.017	121.5068
20	6	14	20	6	15	A	0	11052.363	0.152	117.8265
11	8	3	12	7	5	E	0	11059.747	-0.026	92.6537
19	9	10	18	10	8	E	0	11069.593	-0.107	121.5180
11	8	4	12	7	5	A	0	11074.886	0.029	92.6405
11	8	3	12	7	6	A	0	11076.254	0.040	92.6405
19	9	11	18	10	9	E	0	11095.079	-0.024	121.5055
27	8	19	27	8	20	A	0	11166.415	0.019	158.2935
27	8	19	27	8	20	E	0	11189.580	0.060	158.3012
30	15	16	29	16	13	A	0	11225.172	-0.025	205.5587
30	15	15	29	16	14	A	0	11225.172	-0.026	205.5587
53	31	22	54	30	24	E	0	11242.194	0.053	544.1289
30	15	16	29	16	14	E	0	11264.228	-0.030	205.5491
30	15	15	29	16	13	E	0	11272.103	0.160	205.5623
10	4	7	9	5	4	E	0	11415.721	-0.048	81.2964
42	25	17	43	24	19	E	0	11464.289	-0.056	371.6014
41	21	21	40	22	18	A	0	11475.574	-0.001	328.1991
41	21	20	40	22	19	A	0	11475.574	-0.001	328.1991
41	21	21	40	22	19	E	0	11486.299	-0.072	328.1857
10	4	7	9	5	4	A	0	11493.139	0.026	81.2833
42	25	18	43	24	19	A	0	11502.572	-0.107	371.6095
42	25	17	43	24	20	A	0	11502.572	-0.107	371.6095
42	25	18	43	24	20	E	0	11509.577	0.084	371.5962
41	21	20	40	22	18	E	0	11522.732	0.053	328.1937
10	4	7	9	5	5	E	0	11641.392	0.013	81.2889
31	19	12	32	18	14	E	0	11726.175	-0.028	237.6279
52	27	26	51	28	23	E	0	11736.618	-0.051	489.3856
31	19	13	32	18	15	E	0	11750.484	0.076	237.6158
52	27	26	51	28	23	A	0	11757.832	0.003	489.3967
52	27	25	51	28	24	A	0	11757.832	0.003	489.3967
31	19	13	32	18	14	A	0	11775.380	0.044	237.6273
31	19	12	32	18	15	A	0	11775.380	0.044	237.6273
52	27	25	51	28	24	E	0	11785.064	0.064	489.3849
20	13	8	21	12	10	E	0	11964.921	-0.192	142.2208
20	13	7	21	12	9	E	0	11972.767	-0.106	142.2343
63	33	31	62	34	28	A	0	12057.987	-0.022	689.1123
63	33	30	62	34	29	A	0	12057.987	-0.022	689.1123
62	36	27	63	35	29	E	0	12060.713	0.063	715.1264
2	0	2	1	0	1	A	0	12093.572	-0.012	67.4541

J'	K_a'	K_c'	J''	K_a''	K_c''	Sym.	$v_{t_{CH_3}}$	$\nu_{Obs.}$	$\nu_{Obs.} - \nu_{Calc.}$	E_{low}
21	10	12	20	11	9	A	0	12188.484	0.027	133.5386
21	10	11	20	11	10	A	0	12189.641	-0.047	133.5386
21	10	11	20	11	9	E	0	12219.448	0.046	133.5488
21	10	12	20	11	10	E	0	12239.750	0.042	133.5357
51	30	22	52	29	24	E	0	12334.971	-0.084	510.8851
32	16	16	31	17	15	A	0	12378.311	-0.017	224.4026
32	16	17	31	17	14	A	0	12378.311	-0.017	224.4026
51	30	21	52	29	23	E	0	12382.682	0.267	510.8873
32	16	17	31	17	15	E	0	12413.567	-0.048	224.3920
32	16	16	31	17	14	E	0	12426.754	0.045	224.4047
40	24	16	41	23	18	E	0	12600.424	-0.159	345.1623
43	22	21	42	23	20	A	0	12631.315	-0.097	353.8503
43	22	22	42	23	19	A	0	12631.315	-0.097	353.8503
43	22	22	42	23	20	E	0	12636.786	-0.021	353.8369
40	24	16	41	23	19	A	0	12642.066	-0.009	345.1691
40	24	17	41	23	18	A	0	12642.066	-0.009	345.1691
40	24	17	41	23	19	E	0	12643.596	0.089	345.1557
43	22	21	42	23	19	E	0	12676.529	0.041	353.8435
29	18	11	30	17	13	E	0	12858.262	0.017	217.9956
29	18	12	30	17	14	E	0	12877.734	0.165	217.9829
54	28	26	53	29	24	E	0	12889.337	-0.064	521.8387
29	18	11	30	17	14	A	0	12907.056	0.006	217.9935
29	18	12	30	17	13	A	0	12907.056	0.006	217.9935
12	5	8	11	6	5	E	0	12949.825	-0.005	87.7177
18	12	7	19	11	9	E	0	13081.588	0.026	129.4001
18	12	6	19	11	8	E	0	13094.577	-0.096	129.4132
17	5	12	17	5	13	E	0	13143.288	0.004	103.8221
60	35	26	61	34	27	A	0	13191.200	-0.049	676.3062
60	35	25	61	34	28	A	0	13191.200	-0.049	676.3062
65	34	32	64	35	30	E	0	13206.910	-0.196	728.3455
12	5	8	11	6	6	E	0	13210.372	0.027	87.7090
10	4	6	9	5	5	A	0	13229.549	-0.010	81.2820
23	11	12	22	12	11	A	0	13336.317	-0.185	146.7750
10	4	6	9	5	5	E	0	13350.338	0.068	81.2889
23	11	12	22	12	10	E	0	13370.980	0.058	146.7841
23	11	13	22	12	11	E	0	13385.846	-0.044	146.7707
49	29	21	50	28	23	E	0	13472.646	-0.036	478.8469
49	29	21	50	28	22	A	0	13493.961	0.027	478.8588
49	29	20	50	28	23	A	0	13493.961	0.027	478.8588
34	17	18	33	18	15	A	0	13532.363	-0.038	244.4504
34	17	17	33	18	16	A	0	13532.363	-0.038	244.4504
34	17	18	33	18	16	E	0	13563.424	-0.066	244.4390
34	17	17	33	18	15	E	0	13581.849	0.037	244.4510
12	5	7	11	6	5	E	0	13657.411	0.208	87.7177
38	23	15	39	22	17	E	0	13735.140	-0.023	319.9262
12	5	7	11	6	6	A	0	13750.867	-0.056	87.7039
38	23	16	39	22	18	E	0	13775.374	0.057	319.9183
38	23	16	39	22	17	A	0	13779.288	-0.014	319.9317
38	23	15	39	22	18	A	0	13779.288	-0.014	319.9317
45	23	22	44	24	20	E	0	13830.521	0.018	380.6964
24	7	17	24	7	18	A	0	13855.000	0.041	139.1435
24	7	17	24	7	18	E	0	13877.165	0.006	139.1521
12	5	7	11	6	6	E	0	13917.654	-0.064	87.7090
27	17	10	28	16	12	E	0	13987.496	-0.035	199.5673
27	17	11	28	16	13	E	0	14001.764	0.041	199.5541
27	17	11	28	16	12	A	0	14035.389	-0.001	199.5637
27	17	10	28	16	13	A	0	14035.389	-0.001	199.5637
56	29	27	55	30	25	E	0	14043.064	0.022	555.4936
16	11	6	17	10	8	E	0	14192.532	0.313	117.7842
16	11	5	17	10	7	E	0	14210.467	-0.037	117.7967
16	11	5	17	10	8	A	0	14240.899	0.006	117.7855
14	6	9	13	7	6	A	0	14363.216	-0.085	95.3307
58	34	24	59	33	26	E	0	14379.434	0.062	638.6690
25	12	14	24	13	11	A	0	14485.262	0.029	161.2160
25	12	13	24	13	11	E	0	14523.799	0.048	161.2239
25	12	14	24	13	12	E	0	14533.172	-0.048	161.2103
14	6	8	13	7	6	E	0	14548.690	-0.135	95.3438
47	28	20	48	27	21	E	0	14608.963	0.028	448.0109
14	6	8	13	7	7	A	0	14614.810	-0.037	95.3305
47	28	19	48	27	22	A	0	14634.891	-0.023	448.0221
47	28	20	48	27	21	A	0	14634.891	-0.023	448.0221
47	28	19	48	27	22	E	0	14657.302	0.063	448.0101
36	18	18	35	19	17	A	0	14687.344	0.001	265.7021
36	18	19	35	19	16	A	0	14687.344	0.001	265.7021
36	18	19	35	19	17	E	0	14713.815	-0.049	265.6899
36	18	18	35	19	16	E	0	14737.175	0.012	265.7011
42	12	30	42	12	31	E	0	14850.597	-0.036	283.5014
36	22	14	37	21	16	E	0	14867.798	-0.081	295.8934
14	4	10	14	4	11	A	0	14889.608	-0.052	92.0409
36	22	15	37	21	17	E	0	14904.942	0.193	295.8843
36	22	14	37	21	17	A	0	14914.105	-0.011	295.8975
36	22	15	37	21	16	A	0	14914.105	-0.011	295.8975
47	24	24	46	25	22	E	0	14939.806	-0.025	408.7483
47	24	23	46	25	22	A	0	14945.259	-0.028	408.7613
47	24	24	46	25	21	A	0	14945.259	-0.028	408.7613
47	24	23	46	25	21	E	0	14984.703	0.009	408.7521
25	16	9	26	15	11	E	0	15113.475	-0.091	182.3430
25	16	10	26	15	12	E	0	15122.586	0.143	182.3295
14	10	5	15	9	7	E	0	15295.598	-0.004	107.3730
14	10	4	15	9	6	E	0	15319.043	0.229	107.3848
14	10	4	15	9	7	A	0	15344.679	0.010	107.3727
69	36	33	68	37	32	A	0	15532.826	0.066	810.4487
69	36	34	68	37	31	A	0	15532.826	0.067	810.4487
16	7	10	15	8	7	A	0	15551.559	0.082	104.1621
16	7	9	15	8	7	E	0	15616.963	0.009	104.1748
27	13	15	26	14	12	A	0	15635.716	-0.005	176.8615
27	13	14	26	14	12	E	0	15677.598	0.053	176.8680
27	13	15	26	14	13	E	0	15681.392	-0.032	176.8544
45	27	18	46	26	20	E	0	15743.638	-0.069	418.3772
45	27	19	46	26	20	A	0	15774.101	-0.007	418.3875
45	27	18	46	26	21	A	0	15774.101	-0.007	418.3875
45	27	19	46	26	21	E	0	15791.638	0.062	418.3749
38	19	20	37	20	17	A	0	15843.092	0.005	288.1573
38	19	19	37	20	18	A	0	15843.092	0.005	288.1573
38	19	20	37	20	18	E	0	15864.662	-0.069	288.1446
38	19	19	37	20	17	E	0	15892.727	0.032	288.1548
34	21	13	35	20	15	E	0	15998.456	-0.022	273.0640
34	21	14	35	20	16	E	0	16031.665	0.072	273.0538
34	21	14	35	20	15	A	0	16046.198	-0.038	273.0666
34	21	13	35	20	16	A	0	16046.198	-0.038	273.0666
49	25	25	48	26	23	E	0	16092.374	-0.054	438.0081
49	25	25	48	26	22	A	0	16103.219	0.024	438.0206
49	25	24	48	26	23	A	0	16103.219	0.024	438.0206
49	25	24	48	26	22	E	0	16138.961	-0.078	438.0104
35	10	25	35	10	26	E	0	16153.972	0.028	217.9781
23	15	8	24	14	10	E	0	16235.710	-0.032	166.3229

J'	K_a'	K_c'	J''	K_a''	K_c''	Sym.	$v_{t_{CH_3}}$	$\nu_{Obs.}$	$\nu_{Obs.} - \nu_{Calc.}$	E_{low}
23	15	9	24	14	11	E	0	16239.254	0.063	166.3093
23	15	9	24	14	10	A	0	16279.910	0.001	166.3165
23	15	8	24	14	11	A	0	16279.910	0.001	166.3165
60	31	29	59	32	27	E	0	16353.040	0.026	626.4076
60	31	30	59	32	28	E	0	16397.484	-0.039	626.4013
12	9	3	13	8	5	E	0	16417.464	-0.020	98.1776
12	9	3	13	8	6	A	0	16438.477	-0.035	98.1648
21	6	15	21	6	16	A	0	16603.624	-0.031	122.2331
54	32	23	55	31	25	E	0	16615.858	0.076	567.0000
54	32	22	55	31	25	A	0	16621.315	-0.050	567.0131
54	32	23	55	31	24	A	0	16621.315	-0.050	567.0131
21	6	15	21	6	16	E	0	16623.735	0.052	122.2425
54	32	22	55	31	24	E	0	16659.835	0.079	567.0052
18	8	11	17	9	8	A	0	16701.689	-0.018	114.1986
18	8	10	17	9	9	A	0	16731.501	-0.029	114.1986
18	8	10	17	9	8	E	0	16737.801	0.022	114.2106
18	8	11	17	9	9	E	0	16767.657	0.004	114.1989
29	14	16	28	15	14	E	0	16830.324	0.002	193.7029
29	14	15	28	15	13	E	0	16832.080	0.023	193.7164
28	8	20	28	8	21	A	0	16843.189	0.038	164.2143
28	8	20	28	8	21	E	0	16871.954	0.040	164.2221
43	26	17	44	25	19	E	0	16876.773	-0.094	389.9461
43	26	17	44	25	20	A	0	16911.418	0.091	389.9554
43	26	18	44	25	19	A	0	16911.418	0.091	389.9554
43	26	18	44	25	20	E	0	16923.788	0.089	389.9423
40	20	20	39	21	19	A	0	16999.575	0.001	311.8160
40	20	21	39	21	18	A	0	16999.575	0.001	311.8160
40	20	21	39	21	19	E	0	17016.053	-0.044	311.8029
40	20	20	39	21	18	E	0	17048.413	0.059	311.8120
32	20	12	33	19	14	E	0	17126.614	-0.047	251.4382
32	20	13	33	19	15	E	0	17155.673	0.079	251.4270
32	20	12	33	19	15	A	0	17175.550	0.210	251.4392
32	20	13	33	19	14	A	0	17175.550	0.210	251.4392
51	26	25	50	27	24	A	0	17261.588	-0.082	468.4823
51	26	26	50	27	23	A	0	17261.588	-0.082	468.4823
51	26	25	50	27	23	E	0	17293.605	0.076	468.4712
21	14	8	22	13	10	E	0	17351.294	0.017	151.4935
21	14	7	22	13	9	E	0	17353.214	-0.083	151.5072
21	14	7	22	13	10	A	0	17394.770	0.006	151.4993
21	14	8	22	13	9	A	0	17394.770	0.006	151.4993
63	37	27	64	36	28	A	0	17460.554	-0.052	741.7504
63	37	26	64	36	29	A	0	17460.554	-0.052	741.7504
62	32	30	61	33	28	E	0	17509.174	-0.150	663.6663
62	32	30	61	33	29	A	0	17549.469	-0.014	663.6716
62	32	31	61	33	28	A	0	17549.469	-0.014	663.6716
62	32	31	61	33	29	E	0	17551.643	0.168	663.6587
52	31	22	53	30	23	A	0	17762.027	0.069	532.9844
52	31	21	53	30	24	A	0	17762.027	0.069	532.9844
11	4	8	10	5	5	E	0	17792.367	0.003	83.3709
52	31	21	53	30	23	E	0	17797.543	0.508	532.9753
20	9	11	19	10	10	A	0	17851.596	-0.071	125.4399
20	9	11	19	10	9	E	0	17872.385	0.018	125.4511
20	9	12	19	10	10	E	0	17898.528	0.038	125.4387
31	15	17	30	16	14	A	0	17940.222	-0.002	211.7653
31	15	17	30	16	15	E	0	17979.737	-0.066	211.7557
31	15	16	30	16	14	E	0	17987.145	0.044	211.7689
41	25	16	42	24	18	E	0	18008.424	0.164	362.7177
41	25	17	42	24	18	A	0	18046.336	-0.025	362.7258
41	25	16	42	24	19	A	0	18046.336	-0.025	362.7258
41	25	17	42	24	19	E	0	18053.317	-0.144	362.7125
42	21	22	41	22	19	A	0	18156.745	-0.004	336.6780
42	21	21	41	22	20	A	0	18156.745	-0.004	336.6780
42	21	22	41	22	20	E	0	18167.913	-0.057	336.6646
42	21	21	41	22	19	E	0	18204.230	0.130	336.6726
30	19	11	31	18	13	E	0	18252.029	-0.036	231.0160
30	19	12	31	18	14	E	0	18276.465	0.019	231.0040
41	16	26	40	17	24	E	0	75036.095	0.004	291.6644
12	5	7	11	5	6	E	1	75038.251	-0.085	214.1224
41	16	25	40	17	23	E	0	75044.426	0.029	291.6768
24	7	18	24	6	19	E	0	75138.393	-0.002	136.6458
24	7	18	24	6	19	A	0	75157.338	0.029	136.6366
18	3	16	18	2	17	A	1	75187.642	0.028	229.4750
42	11	31	42	10	32	A	0	75238.403	-0.026	278.6898
42	11	31	42	10	32	E	0	75240.264	0.002	278.6970
18	2	16	18	1	17	E	0	75263.149	0.026	100.7820
18	2	16	18	1	17	A	0	75273.990	0.050	100.7708
18	2	16	18	1	17	E	1	75301.894	-0.008	229.1145
12	5	7	11	5	6	A	0	75314.342	-0.003	85.6503
54	23	32	53	24	29	A	0	75318.349	-0.023	472.1217
54	23	31	53	24	30	A	0	75318.349	-0.023	472.1217
28	9	19	28	8	20	E	0	75413.889	-0.037	164.7849
12	2	10	11	2	9	E	1	75421.412	-0.045	210.5768
20	3	17	20	2	18	A	1	75423.272	0.078	239.4784
12	2	10	11	2	9	E	0	75432.986	0.011	82.2421
12	2	10	11	2	9	A	0	75435.765	0.006	82.2305
28	9	19	28	8	20	A	0	75458.047	0.014	164.7762
18	3	16	18	2	17	E	0	75516.513	0.014	100.7824
18	3	16	18	2	17	A	0	75528.013	0.022	100.7712
22	8	14	22	7	15	E	0	75536.962	-0.026	129.4252
25	7	19	24	8	16	E	0	75542.562	-0.004	141.8981
22	8	14	22	7	15	A	0	75546.573	0.035	129.4161
18	3	16	18	2	17	E	1	75559.993	-0.022	229.1149
20	3	17	20	2	18	E	0	75683.362	0.041	110.8023
20	3	17	20	2	18	A	0	75690.837	0.055	110.7916
43	17	27	42	18	25	E	0	76040.560	-0.010	315.4362
43	17	26	42	18	24	E	0	76054.664	0.022	315.4480
10	3	8	9	2	7	E	0	76237.950	-0.010	77.7437
10	3	8	9	2	7	A	0	76251.134	0.012	77.7320
20	4	17	20	3	18	A	1	76426.775	0.078	239.4809
20	4	17	20	3	18	E	0	76733.238	0.032	110.8049
20	4	17	20	3	18	A	0	76743.393	0.029	110.7942
20	4	17	20	3	18	E	1	76746.197	-0.023	239.1380
33	10	23	33	9	24	E	0	76967.194	-0.043	200.9766
33	10	23	33	9	24	A	0	77020.561	-0.002	200.9683
45	18	28	44	19	26	E	0	77064.736	0.001	340.4116
45	18	27	44	19	25	E	0	77084.321	0.050	340.4227
12	4	8	11	4	7	A	0	77413.967	0.001	84.0657
12	4	8	11	4	7	E	0	77414.958	0.014	84.0766
25	5	20	25	4	21	A	0	77503.316	0.022	139.0087
25	5	20	25	4	21	E	0	77507.395	0.043	139.0182
21	8	14	21	7	14	E	0	77615.515	-0.096	124.7122
22	5	18	22	4	19	E	1	77654.080	-0.052	250.2199
22	5	18	22	4	19	E	0	77678.694	0.024	121.8869
22	5	18	22	4	19	A	0	77688.182	0.036	121.8768
25	7	19	25	6	20	E	0	77745.338	0.006	141.8247
25	7	19	25	6	20	A	0	77761.539	0.025	141.8155

J'	K'_a	K'_c	J''	K''_a	K''_c	Sym.	$v_{t_{\mathrm{CH_3}}}$	$\nu_{\mathrm{Obs.}}$	$\nu_{\mathrm{Obs.}} - \nu_{\mathrm{Calc.}}$	E_{low}
44	12	32	44	11	33	E	0	77966.279	-0.045	300.4739
38	11	27	38	10	28	E	0	77989.184	-0.025	242.9660
44	12	32	44	11	33	A	0	77996.678	-0.014	300.4667
38	11	27	38	10	28	A	0	78043.489	0.006	242.9581
21	8	13	21	7	14	A	0	78052.622	0.022	124.7032
13	3	11	12	3	10	E	0	78359.606	0.001	84.9558
13	3	11	12	3	10	A	0	78360.824	0.011	84.9446
32	11	21	31	12	19	E	0	78462.060	0.212	197.3702
45	12	33	45	11	34	E	0	78561.003	-0.001	310.3969
45	12	33	45	11	34	A	0	78573.528	-0.025	310.3899
11	3	9	10	2	8	A	0	78760.633	0.004	79.8884
24	6	19	24	5	20	E	0	78850.426	0.022	134.0156
24	6	19	24	5	20	A	0	78860.831	0.023	134.0060
14	1	13	13	2	12	A	1	79023.819	0.015	214.5549
14	1	13	13	2	12	E	1	79058.625	-0.032	214.1874
14	1	13	13	2	12	E	0	79070.439	-0.003	85.8529
14	1	13	13	2	12	A	0	79071.337	-0.007	85.8415
14	1	13	13	1	12	E	1	79362.912	0.009	214.1773
15	0	15	14	1	14	A	1	79403.668	0.027	215.0754
15	1	15	14	1	14	A	1	79405.500	0.022	215.0754
15	0	15	14	0	14	A	1	79407.485	0.012	215.0753
15	1	15	14	0	14	A	1	79409.332	0.022	215.0753
15	0	15	14	1	14	E	1	79417.420	-0.022	214.6974
15	1	15	14	1	14	E	1	79419.371	-0.044	214.6974
15	0	15	14	0	14	E	1	79421.521	-0.011	214.6973
15	1	15	14	0	14	E	1	79423.501	-0.002	214.6973
15	0	15	14	1	14	E	0	79434.701	-0.021	86.3650
15	0	15	14	1	14	A	0	79435.154	0.010	86.3533
15	1	15	14	1	14	E	0	79436.641	-0.014	86.3650
15	1	15	14	1	14	A	0	79437.123	0.040	86.3533
15	0	15	14	0	14	E	0	79438.719	-0.023	86.3649
15	0	15	14	0	14	A	0	79439.166	-0.010	86.3532
15	1	15	14	0	14	E	0	79440.651	-0.025	86.3649
15	1	15	14	0	14	A	0	79441.121	0.006	86.3532
14	2	13	13	1	12	A	1	79469.008	0.053	214.5453
14	2	13	13	1	12	E	0	79533.406	0.019	85.8428
14	2	13	13	1	12	A	0	79535.313	0.005	85.8314
28	6	22	28	5	23	A	0	79568.217	0.019	158.3724
28	6	22	28	5	23	E	0	79578.904	0.038	158.3814
43	12	31	43	11	32	E	0	79799.119	-0.051	290.7362
24	8	17	24	7	18	E	0	79912.100	0.325	139.1521
23	8	16	23	7	17	A	0	79992.950	0.004	134.0836
40	10	30	40	9	31	A	0	80115.725	-0.057	257.7720
40	10	30	40	9	31	E	0	80139.224	0.014	257.7791
22	8	15	22	7	16	E	0	80271.812	-0.105	129.2426
25	8	18	25	7	19	E	0	80276.337	0.000	144.4180
25	8	18	25	7	19	A	0	80307.695	0.021	144.4093
19	3	17	19	2	18	E	0	80508.791	0.017	104.2820
19	3	17	19	2	18	A	0	80521.171	0.058	104.2708
19	3	17	19	2	18	E	1	80558.087	-0.036	232.6139
21	3	18	21	2	19	A	1	80818.891	0.097	243.4852
26	7	20	26	6	21	E	0	80937.686	-0.007	147.1849
13	6	7	12	6	6	E	0	81031.258	0.033	90.2061
21	3	18	21	2	19	E	0	81115.406	0.017	114.8120
21	3	18	21	2	19	E	1	81121.176	-0.059	243.1446
21	3	18	21	2	19	A	0	81124.482	0.015	114.8014
26	8	19	26	7	20	E	0	81138.196	-0.032	149.8846
13	5	9	12	5	8	A	0	81337.324	-0.003	88.1401
19	8	11	19	7	12	A	0	81345.234	0.022	116.0067
13	5	9	12	5	8	E	0	81346.905	0.001	88.1496
12	3	10	11	2	9	E	0	81355.285	-0.039	82.2421
14	2	12	13	3	11	A	0	81357.985	-0.004	87.5585
14	2	12	13	3	11	E	0	81361.006	-0.014	87.5696
12	3	10	11	2	9	A	0	81366.490	-0.009	82.2305
21	4	18	21	3	19	A	1	81401.265	0.074	243.4865
20	8	13	20	7	14	A	0	81501.159	0.026	120.1788
21	4	18	21	3	19	E	0	81726.923	0.021	114.8134
46	12	34	46	11	35	A	0	81735.681	-0.121	320.4769
21	4	18	21	3	19	A	0	81737.723	0.043	114.8028
21	4	18	21	3	19	E	1	81745.872	-0.063	243.1460
15	3	12	14	4	11	A	0	82021.352	-0.034	92.0409
15	3	12	14	4	11	E	0	82035.935	0.024	92.0516
13	5	8	12	5	7	E	0	82085.889	0.223	88.1732
13	5	8	12	5	7	A	0	82093.162	-0.009	88.1625
32	10	22	32	9	23	E	0	82100.572	-0.038	193.7155
32	10	22	32	9	23	A	0	82152.460	0.007	193.7074
19	8	12	19	7	13	A	0	82162.565	0.016	115.9771
42	16	27	41	17	25	E	0	82368.244	-0.025	300.2157
42	16	26	41	17	24	E	0	82375.796	0.044	300.2281
18	8	10	18	7	11	A	0	82395.402	0.028	112.0083
19	5	14	18	6	13	A	0	82459.816	-0.013	109.6336
18	8	10	18	7	11	E	0	82465.921	-0.003	112.0182
23	5	19	23	4	20	E	1	82522.972	-0.069	254.7385
23	5	19	23	4	20	E	0	82539.263	0.024	126.4059
23	5	19	23	4	20	A	0	82548.828	0.031	126.3959
27	8	20	27	7	21	E	0	82572.330	-0.013	155.5469
27	8	20	27	7	21	A	0	82595.593	0.017	155.5384
18	8	11	18	7	12	E	0	82663.007	-0.045	112.0009
18	8	11	18	7	12	A	0	82799.618	0.013	111.9938
37	11	26	37	10	27	E	0	83013.433	-0.065	234.5613
37	11	26	37	10	27	A	0	83072.869	0.023	234.5535
17	8	9	17	7	10	A	0	83192.080	0.039	108.2353
17	8	9	17	7	10	E	0	83239.924	-0.020	108.2462
17	8	10	17	7	11	E	0	83267.192	-0.008	108.2346
18	1	17	18	0	18	A	1	83282.270	-0.055	226.6967
25	6	20	25	5	21	E	0	83315.578	0.017	139.0455
25	6	20	25	5	21	A	0	83325.148	0.033	139.0361
44	17	28	43	18	26	E	0	83325.835	-0.002	324.4005
44	17	27	43	18	25	E	0	83339.242	0.053	324.4123
17	8	10	17	7	11	A	0	83380.380	0.012	108.2286
26	9	17	26	8	18	E	0	83566.768	-0.036	152.8260
26	9	17	26	8	18	A	0	83588.862	0.017	152.8177
42	12	30	42	11	31	E	0	83642.782	-0.033	281.2068
18	1	17	18	0	18	E	0	83680.658	0.016	97.9907
18	2	17	18	1	18	E	0	83691.945	0.035	97.9908
18	1	17	18	0	18	A	0	83695.308	0.053	97.9791
42	12	30	42	11	31	A	0	83700.824	0.014	281.1995
48	13	35	48	12	36	E	0	83705.463	-0.016	344.2577
18	2	17	18	1	18	A	0	83706.620	0.062	97.9791
14	3	12	13	3	11	A	1	83721.009	0.008	216.2636
48	13	35	48	12	36	A	0	83734.961	-0.025	344.2511
18	1	17	18	0	18	E	1	83760.308	0.017	226.3205
18	2	17	18	1	18	E	1	83771.829	0.001	226.3205
14	3	12	13	3	11	E	0	83786.192	-0.000	87.5696
14	3	12	13	3	11	A	0	83787.692	0.011	87.5585
14	3	12	13	3	11	E	1	83792.052	-0.021	215.9041

J'	K_a'	K_c'	J''	K_a''	K_c''	Sym.	$v_{t_{CH_3}}$	$\nu_{Obs.}$	$\nu_{Obs.} - \nu_{Calc.}$	E_{low}
16	8	9	16	7	10	E	0	83804.571	-0.013	104.6857
16	8	8	16	7	9	A	0	83806.766	0.053	104.6838
16	8	8	16	7	9	E	0	83826.409	-0.023	104.6958
16	8	9	16	7	10	A	0	83889.001	0.006	104.6809
26	5	21	26	4	22	E	1	84119.392	-0.191	272.5624
15	1	14	14	2	13	A	1	84245.730	0.014	217.1961
26	5	21	26	4	22	A	0	84248.935	0.030	144.2223
26	5	21	26	4	22	E	0	84249.796	0.037	144.2317
15	8	8	15	7	9	E	0	84256.454	0.005	101.3535
13	3	11	12	2	10	E	0	84281.934	-0.018	84.7582
15	1	14	14	2	13	E	1	84284.531	-0.023	216.8300
15	8	7	15	7	8	A	0	84287.633	0.020	101.3506
15	8	7	15	7	8	E	0	84288.024	0.002	101.3633
13	3	11	12	2	10	A	0	84291.546	-0.006	84.7468
15	1	14	14	2	13	E	0	84297.121	-0.005	88.4958
15	1	14	14	2	13	A	0	84298.222	-0.005	88.4844
46	18	29	45	19	27	E	0	84309.876	0.002	349.7890
15	8	8	15	7	9	A	0	84321.106	0.002	101.3494
15	2	14	14	2	13	A	1	84327.159	0.005	217.1961
46	18	28	45	19	26	E	0	84328.816	0.034	349.8000
15	2	14	14	2	13	E	1	84371.005	-0.023	216.8300
15	2	14	14	2	13	E	0	84382.276	-0.005	88.4958
15	2	14	14	2	13	A	0	84383.592	-0.004	88.4844
15	1	14	14	1	13	A	1	84400.803	0.005	217.1909
17	4	13	16	5	12	A	0	84409.836	-0.001	100.2619
17	4	13	16	5	12	E	0	84437.048	0.039	100.2720
15	1	14	14	1	13	E	1	84448.210	-0.012	216.8245
15	1	14	14	1	13	E	0	84458.750	-0.004	88.4904
15	1	14	14	1	13	A	0	84460.226	-0.005	88.4790
15	2	14	14	1	13	A	1	84482.245	0.011	217.1909
16	0	16	15	1	15	A	1	84534.101	0.021	217.7241
15	2	14	14	1	13	E	1	84534.666	-0.030	216.8245
16	0	16	15	0	15	A	1	84535.928	0.011	217.7240
16	1	16	15	0	15	A	1	84536.811	0.018	217.7240
15	2	14	14	1	13	E	0	84543.909	0.000	88.4904
15	2	14	14	1	13	A	0	84545.581	-0.020	88.4790
16	0	16	15	1	15	E	1	84547.827	-0.019	217.3465
16	1	16	15	1	15	E	1	84548.770	-0.020	217.3465
16	0	16	15	0	15	E	1	84549.802	-0.015	217.3465
16	1	16	15	0	15	E	1	84550.737	-0.025	217.3465
16	0	16	15	1	15	E	0	84566.439	-0.031	89.0148
16	0	16	15	1	15	A	0	84566.905	0.013	89.0030
16	1	16	15	1	15	E	0	84567.378	-0.015	89.0148
16	1	16	15	1	15	A	0	84567.831	0.012	89.0030
16	0	16	15	0	15	E	0	84568.374	-0.029	89.0147
16	0	16	15	0	15	A	0	84568.827	-0.004	89.0030
16	1	16	15	0	15	E	0	84569.306	-0.021	89.0147
16	1	16	15	0	15	A	0	84569.767	0.009	89.0030
28	8	21	28	7	22	E	0	84622.399	0.006	161.3994
14	8	7	14	7	8	E	0	84624.877	-0.020	98.2368
49	13	36	49	12	37	A	0	84650.999	-0.034	355.0585
14	8	6	14	7	7	E	0	84657.555	-0.023	98.2465
27	7	21	27	6	22	E	0	84662.368	0.013	152.7229
27	7	21	27	6	22	A	0	84674.107	0.019	152.7139
14	8	7	14	7	8	A	0	84679.464	-0.012	98.2331
13	4	9	12	4	8	A	0	84687.223	0.007	86.6479
13	4	9	12	4	8	E	0	84688.740	-0.009	86.6589
31	10	22	30	11	19	A	0	84826.471	-0.023	186.7043
13	8	6	13	7	7	E	0	84920.567	-0.004	95.3340
13	8	5	13	7	6	E	0	84952.925	-0.038	95.3438
20	2	18	20	1	19	A	1	85062.410	0.009	236.6411
13	3	10	12	3	9	E	1	85097.432	-0.022	214.0294
13	3	10	12	3	9	A	1	85101.501	0.031	214.3907
20	3	18	20	2	19	A	1	85132.926	-0.002	236.6412
14	2	12	13	2	11	A	1	85146.507	0.020	216.1379
12	8	5	12	7	6	E	0	85154.723	0.002	92.6439
13	3	10	12	3	9	E	0	85170.490	-0.022	85.6920
13	3	10	12	3	9	A	0	85171.686	0.013	85.6806
12	8	4	12	7	5	E	0	85186.689	-0.026	92.6537
14	2	12	13	2	11	E	1	85244.329	-0.018	215.7743
14	2	12	13	2	11	E	0	85245.092	-0.020	87.4400
14	2	12	13	2	11	A	0	85248.458	0.003	87.4287
48	19	30	47	20	28	E	0	85315.340	0.005	376.3810
11	8	4	11	7	5	E	0	85337.390	0.018	90.1652
48	19	29	47	20	27	E	0	85339.483	0.048	376.3911
47	13	34	47	12	35	E	0	85346.326	-0.023	333.6314
11	8	3	11	7	4	E	0	85369.027	-0.012	90.1751
47	13	34	47	12	35	A	0	85393.480	-0.010	333.6246
20	2	18	20	1	19	E	0	85434.231	0.025	107.9525
20	2	18	20	1	19	A	0	85447.241	0.046	107.9414
10	8	3	10	7	4	E	0	85477.198	-0.045	87.8967
20	3	18	20	2	19	A	0	85521.902	0.054	107.9415
10	8	3	10	7	4	A	0	85524.545	0.004	87.8934
24	7	17	23	8	16	A	0	85553.905	-0.020	136.7519
20	3	18	20	2	19	E	1	85563.728	-0.018	236.2839
24	7	17	23	8	16	E	0	85623.177	0.059	136.7590
61	26	36	60	27	34	E	0	85664.550	0.022	583.5202
61	26	35	60	27	34	A	0	85675.545	0.005	583.5317
61	26	36	60	27	33	A	0	85675.545	0.005	583.5317
33	11	23	32	12	20	A	0	85787.523	-0.013	204.0709
22	3	19	22	2	20	A	1	86085.115	0.089	247.6623
24	4	20	24	3	21	E	1	86210.566	-0.077	259.4242
24	4	20	24	3	21	E	0	86251.245	0.033	131.0922
24	4	20	24	3	21	A	0	86257.520	0.050	131.0823
50	20	30	49	21	29	A	0	86315.411	-0.016	404.1886
14	10	4	13	10	3	A	0	86388.940	0.034	105.0030
14	10	5	13	10	4	A	0	86388.940	0.034	105.0030
14	10	4	13	10	3	E	0	86388.940	-0.112	105.0141
14	10	5	13	10	4	E	0	86389.446	0.042	105.0016
22	3	19	22	2	20	E	0	86414.136	0.025	118.9921
22	4	19	22	3	20	A	1	86416.018	0.098	247.6630
22	3	19	22	2	20	A	0	86424.623	0.039	118.9816
22	3	19	22	2	20	E	1	86430.214	-0.039	247.3242
28	8	21	27	9	18	A	0	86492.049	-0.028	161.3293
28	8	21	27	9	18	E	0	86506.246	0.025	161.3366
14	9	5	13	9	4	A	0	86528.393	0.025	101.3902
14	9	5	13	9	4	E	0	86528.393	-0.087	101.4022
14	9	6	13	9	5	E	0	86529.023	-0.006	101.3904
35	12	24	34	13	21	A	0	86554.784	-0.080	222.6450
25	9	16	25	8	17	A	0	86590.414	0.011	147.2281
25	9	16	25	8	17	E	0	86592.033	-0.038	147.2363
14	8	6	13	8	5	A	0	86725.791	-0.138	98.1648
14	8	6	13	8	5	E	0	86725.791	-0.027	98.1776
14	8	7	13	8	6	E	0	86726.676	-0.007	98.1666
35	12	24	34	13	22	E	0	86730.230	-0.049	222.6398
22	4	19	22	3	20	E	0	86762.792	0.028	118.9928

J'	K_a'	K_c'	J''	K_a''	K_c''	Sym.	$v_{t_{CH_3}}$	$\nu_{Obs.}$	$\nu_{Obs.} - \nu_{Calc.}$	E_{low}
14	4	11	13	4	10	A	1	86771.588	0.011	217.8399
22	4	19	22	3	20	A	0	86774.319	0.042	118.9823
33	11	22	32	12	21	A	0	86782.526	-0.021	204.0694
63	27	37	62	28	34	A	0	86786.314	-0.016	619.3290
63	27	36	62	28	35	A	0	86786.314	-0.016	619.3290
22	4	19	22	3	20	E	1	86787.486	-0.049	247.3250
29	6	23	29	5	24	E	1	86803.657	-0.245	292.6170
14	4	11	13	4	10	E	0	86827.185	-0.031	89.1553
14	4	11	13	4	10	A	0	86827.615	0.030	89.1446
35	12	23	34	13	21	E	0	86860.553	0.140	222.6529
35	12	23	34	13	22	A	0	86937.154	-0.006	222.6444
31	10	21	31	9	22	E	0	86998.891	-0.044	186.7160
14	7	8	13	7	7	A	0	87016.898	0.040	95.3305
29	6	23	29	5	24	A	0	87017.771	0.021	164.2807
14	7	7	13	7	6	E	0	87021.203	0.000	95.3438
14	7	8	13	7	7	E	0	87022.361	0.003	95.3340
29	6	23	29	5	24	E	0	87024.989	0.028	164.2895
31	10	21	31	9	22	A	0	87044.275	0.025	186.7082
24	5	20	24	4	21	A	1	87210.841	0.195	259.7470
37	13	25	36	14	22	A	0	87292.956	-0.040	242.4250
31	10	21	30	11	20	A	0	87295.960	-0.026	186.6998
29	8	22	29	7	23	E	0	87296.498	0.005	167.4373
29	8	22	29	7	23	A	0	87313.157	0.022	167.4289
47	12	35	47	11	36	A	0	87365.822	-0.059	330.7104
52	21	32	51	22	30	E	0	87376.190	-0.008	433.1745
47	12	35	47	11	36	E	0	87387.262	-0.012	330.7165
37	13	25	36	14	23	E	0	87408.159	-0.004	242.4186
37	13	24	36	14	22	E	0	87418.641	0.035	242.4318
14	6	9	13	6	8	A	0	87431.155	0.009	92.8934
37	13	24	36	14	23	A	0	87434.675	0.009	242.4248
14	6	9	13	6	8	E	0	87465.184	0.007	92.8998
14	3	12	13	2	11	A	1	87492.277	0.021	216.1379
24	5	20	24	4	21	E	1	87519.144	-0.077	259.4281
24	5	20	24	4	21	E	0	87529.286	0.022	131.0960
24	5	20	24	4	21	A	0	87539.201	0.038	131.0860
14	6	8	13	6	7	E	0	87543.494	-0.034	92.9090
44	11	33	44	10	34	A	0	87573.157	-0.053	297.5455
14	6	8	13	6	7	A	0	87575.834	-0.002	92.8968
44	11	33	44	10	34	E	0	87601.673	-0.003	297.5518
15	2	13	14	3	12	A	1	87646.470	0.003	219.0563
15	2	13	14	3	12	E	1	87649.152	-0.055	218.6991
15	2	13	14	3	12	A	0	87663.075	-0.005	90.3533
15	2	13	14	3	12	E	0	87664.376	-0.001	90.3644
14	3	12	13	2	11	E	0	87670.265	-0.019	87.4400
14	3	12	13	2	11	A	0	87678.153	0.007	87.4287
14	3	12	13	2	11	E	1	87684.087	0.021	215.7743
14	5	10	13	5	9	A	0	87720.425	0.003	90.8532
14	5	10	13	5	9	E	0	87724.858	0.001	90.8631
26	6	21	26	5	22	E	1	88010.008	-0.071	272.5787
26	6	21	26	5	22	E	0	88059.111	0.012	144.2475
26	6	21	26	5	22	A	0	88068.240	0.028	144.2382
39	14	25	38	15	24	A	0	88114.913	0.028	263.4102
39	14	25	38	15	23	E	0	88137.136	0.036	263.4157
39	14	26	38	15	24	E	0	88140.333	0.011	263.4027
19	1	18	19	0	19	A	1	88218.710	-0.062	230.0297
50	13	37	50	12	38	A	0	88262.364	-0.061	366.0249
50	13	37	50	12	38	E	0	88271.707	-0.007	366.0307
41	10	31	41	9	32	A	0	88329.348	-0.050	266.5365
41	10	31	41	9	32	E	0	88359.754	0.004	266.5431
32	7	25	32	6	26	E	1	88379.287	-0.286	314.8840
54	22	33	53	23	30	A	0	88414.535	-0.009	463.3882
54	22	33	53	23	31	E	0	88426.427	0.034	463.3755
36	11	25	36	10	26	E	0	88535.373	-0.055	226.4073
36	11	25	36	10	26	A	0	88594.532	0.007	226.3998
19	1	18	19	0	19	E	0	88642.048	0.014	101.3250
19	2	18	19	1	19	E	0	88647.714	0.019	101.3250
19	1	18	19	0	19	A	0	88657.634	0.044	101.3134
19	2	18	19	1	19	A	0	88663.319	0.048	101.3134
32	7	25	32	6	26	A	0	88690.572	0.012	186.5531
32	7	25	32	6	26	E	0	88705.158	0.031	186.5612
19	1	18	19	0	19	E	1	88727.600	0.016	229.6540
19	2	18	19	1	19	E	1	88733.420	0.023	229.6540
28	7	22	28	6	23	E	0	88843.632	0.001	158.4359
28	7	22	28	6	23	A	0	88853.777	-0.004	158.4271
41	12	29	41	11	30	E	0	88869.091	-0.030	271.9083
41	15	26	40	16	25	A	0	88901.453	0.038	285.6002
35	8	27	35	7	28	E	1	88904.073	-0.369	339.3514
24	9	15	24	8	16	A	0	88910.250	0.006	141.8900
41	12	29	41	11	30	A	0	88933.855	0.013	271.9012
41	15	27	40	16	25	E	0	88940.769	0.000	285.5915
41	15	26	40	16	24	E	0	88941.450	0.045	285.6043
15	3	13	14	3	12	A	1	89048.170	0.000	219.0563
29	9	20	28	10	19	A	0	89048.810	-0.042	170.5353
38	9	29	38	8	30	A	0	89072.453	-0.015	237.6934
14	5	9	13	5	8	E	0	89081.601	0.017	90.9113
14	5	9	13	5	8	A	0	89083.421	0.007	90.9009
38	9	29	38	8	30	E	0	89100.202	0.019	237.7004
28	9	20	28	8	21	E	0	89106.332	-0.024	164.2221
15	3	13	14	3	12	E	1	89117.520	-0.026	218.6991
15	3	13	14	3	12	E	0	89118.865	-0.002	90.3644
15	3	13	14	3	12	A	0	89120.590	-0.003	90.3533
28	9	20	28	8	21	A	0	89144.558	0.017	164.2143
46	13	33	46	12	34	E	0	89170.649	-0.023	323.2101
27	9	19	27	8	20	E	0	89187.233	-0.018	158.3012
46	13	33	46	12	34	A	0	89231.605	0.010	323.2033
27	9	19	27	8	20	A	0	89233.219	0.009	158.2935
35	8	27	35	7	28	A	0	89311.423	-0.009	211.0280
35	8	27	35	7	28	E	0	89333.360	0.026	211.0356
16	1	15	15	2	14	A	1	89422.787	0.022	220.0089
52	14	38	52	13	39	E	0	89423.797	-0.003	391.8026
52	14	38	52	13	39	A	0	89451.762	-0.027	391.7966
29	9	21	29	8	22	E	0	89456.963	-0.021	170.3492
16	1	15	15	2	14	E	1	89463.955	-0.029	219.6443
16	2	15	15	2	14	A	1	89464.876	-0.014	220.0089
16	1	15	15	2	14	E	0	89477.482	0.009	91.3105
16	1	15	15	2	14	A	0	89478.685	-0.003	91.2991
29	9	21	29	8	22	A	0	89489.709	0.014	170.3414
16	1	15	15	1	14	A	1	89504.205	0.004	220.0062
16	2	15	15	2	14	E	1	89508.962	-0.027	219.6443
16	2	15	15	2	14	E	0	89521.666	-0.001	91.3105
16	2	15	15	2	14	A	0	89522.990	-0.013	91.2991
16	2	15	15	1	14	A	1	89546.339	0.012	220.0062
16	1	15	15	1	14	E	1	89550.438	-0.020	219.6414
16	1	15	15	1	14	E	0	89562.616	-0.012	91.3076
16	1	15	15	1	14	A	0	89564.054	-0.003	91.2963
26	9	18	26	8	19	E	0	89590.784	-0.025	152.5911

J'	K'_a	K'_c	J''	K''_a	K''_c	Sym.	$v_{t_{CH_3}}$	$\nu_{Obs.}$	$\nu_{Obs.} - \nu_{Calc.}$	E_{low}
16	2	15	15	1	14	E	1	89595.468	0.005	219.6414
16	2	15	15	1	14	E	0	89606.823	0.001	91.3076
16	2	15	15	1	14	A	0	89608.370	-0.003	91.2963
26	9	18	26	8	19	A	0	89649.501	-0.001	152.5834
17	0	17	16	1	16	A	1	89663.850	0.022	220.5438
17	1	17	16	1	16	A	1	89664.277	0.035	220.5438
17	0	17	16	0	16	A	1	89664.702	-0.001	220.5438
17	1	17	16	0	16	A	1	89665.167	0.051	220.5438
17	0	17	16	1	16	E	1	89677.485	-0.033	220.1668
17	1	17	16	1	16	E	1	89677.965	-0.003	220.1668
17	0	17	16	0	16	E	1	89678.423	-0.041	220.1667
17	1	17	16	0	16	E	1	89678.896	-0.016	220.1667
17	0	17	16	1	16	E	0	89697.456	-0.038	91.8356
17	0	17	16	1	16	A	0	89697.918	0.002	91.8239
17	1	17	16	1	16	E	0	89697.918	-0.015	91.8356
17	1	17	16	1	16	A	0	89698.377	0.021	91.8239
17	0	17	16	0	16	E	0	89698.377	-0.041	91.8356
17	0	17	16	0	16	A	0	89698.838	-0.004	91.8239
17	1	17	16	0	16	E	0	89698.838	-0.018	91.8356
17	1	17	16	0	16	A	0	89699.296	0.014	91.8239
43	16	27	42	17	26	A	0	89756.848	0.275	308.9945
15	2	13	14	2	12	A	1	89992.286	0.051	218.9780
21	2	19	21	1	20	A	1	90072.057	0.005	240.4807
15	2	13	14	2	12	E	1	90088.935	0.010	218.6177
15	2	13	14	2	12	E	0	90089.537	-0.012	90.2835
15	2	13	14	2	12	A	0	90092.774	0.004	90.2723
21	3	19	21	2	20	A	1	90109.336	-0.006	240.4808
25	9	17	25	8	18	E	0	90206.510	-0.019	147.0957
25	9	17	25	8	18	A	0	90286.859	0.016	147.0881
30	9	22	30	8	23	E	0	90334.319	-0.009	176.6771
30	9	22	30	8	23	A	0	90362.551	0.011	176.6694
23	9	15	23	8	15	E	0	90384.245	-0.032	136.8034
30	9	22	29	10	19	E	0	90400.102	0.005	176.6749
21	2	19	21	1	20	E	0	90469.534	0.031	111.7943
21	2	19	21	1	20	A	0	90483.544	0.057	111.7832
27	5	22	27	4	23	E	1	90484.165	-0.167	277.9422
21	3	19	21	2	20	E	0	90509.013	0.028	111.7943
21	3	19	21	2	20	A	0	90523.148	0.050	111.7833
21	2	19	21	1	20	E	1	90529.237	0.003	240.1249
30	8	23	30	7	24	E	0	90567.518	0.012	173.6561
21	3	19	21	2	20	E	1	90569.797	-0.009	240.1249
30	8	23	30	7	24	A	0	90581.350	0.019	173.6479
27	5	22	27	4	23	E	0	90588.303	0.036	149.6117
27	5	22	27	4	23	A	0	90590.419	0.030	149.6025
58	24	34	57	25	32	E	0	90600.304	0.028	527.3889
23	9	14	23	8	15	A	0	90679.291	0.022	136.7951
45	17	29	44	18	27	E	0	90698.694	-0.019	333.5822
45	17	28	44	18	26	E	0	90711.314	0.033	333.5940
53	14	39	53	13	40	E	0	90735.135	-0.013	403.4932
23	9	14	23	8	15	E	0	90743.094	-0.006	136.8034
22	6	16	21	7	15	E	0	90792.023	0.070	124.6066
51	14	37	51	13	38	E	0	90830.544	-0.019	380.2883
51	14	37	51	13	38	A	0	90877.940	-0.013	380.2821
24	9	16	24	8	17	E	0	90937.722	-0.053	141.8177
16	3	13	15	4	12	A	0	90938.435	-0.010	95.1317
14	3	11	13	3	10	A	1	90943.347	0.012	217.2294
16	3	13	15	4	12	E	0	90949.616	0.018	95.1424
14	3	11	13	3	10	E	1	90986.893	-0.026	216.8679
14	3	11	13	3	10	E	0	91032.953	-0.006	88.5330
14	3	11	13	3	10	A	0	91035.175	0.012	88.5216
24	9	16	24	8	17	A	0	91048.821	0.019	141.8103
23	3	20	23	2	21	A	1	91263.947	0.108	252.0100
30	10	20	30	9	21	E	0	91278.218	-0.030	179.9826
30	10	20	30	9	21	A	0	91311.363	0.012	179.9750
15	3	13	14	2	12	A	1	91393.957	0.019	218.9780
3	1	2	3	0	3	A	1	9141.224	-0.019	197.1841
15	3	13	14	2	12	E	0	91544.030	-0.008	90.2835
15	3	13	14	2	12	A	0	91550.286	0.002	90.2723
15	3	13	14	2	12	E	1	91557.283	0.018	218.6177
23	3	20	23	2	21	E	0	91622.478	0.025	123.3428
23	3	20	23	2	21	A	0	91634.196	0.043	123.3324
23	3	20	23	2	21	E	1	91647.417	-0.045	251.6744
31	9	23	31	8	24	E	0	91809.335	-0.006	183.2005
25	4	21	25	3	22	E	1	91814.289	-0.083	264.2864
23	4	20	23	3	21	E	0	91817.724	0.034	123.3432
23	4	20	23	3	21	A	0	91830.054	0.043	123.3328
31	9	23	31	8	24	A	0	91833.476	0.006	183.1928
25	4	21	25	3	22	E	0	91839.675	0.034	135.9548
14	4	10	13	4	9	A	1	91853.953	0.028	218.1721
23	9	15	23	8	16	A	0	91856.379	0.018	136.7519
14	4	10	13	4	9	A	0	91879.330	0.006	89.4728
14	4	10	13	4	9	E	0	91880.699	0.025	89.4838
3	1	2	3	0	3	E	0	9194.982	-0.000	68.4646
3	1	2	3	0	3	A	0	9197.058	-0.010	68.4526
22	9	13	22	8	14	A	0	92033.904	0.005	131.9361
22	9	13	22	8	14	E	0	92108.859	-0.021	131.9448
25	5	21	25	4	22	A	1	92261.177	0.180	264.6040
15	12	3	14	12	2	A	0	92429.247	-0.058	116.2589
15	12	4	14	12	3	A	0	92429.247	-0.058	116.2589
22	9	14	22	8	15	E	0	92502.421	-0.052	131.9201
15	11	5	14	11	4	A	0	92525.118	-0.011	111.8793
15	11	4	14	11	3	A	0	92525.118	-0.011	111.8793
15	11	4	14	11	3	E	0	92525.118	-0.190	111.8894
15	11	5	14	11	4	E	0	92525.642	0.044	111.8763
49	19	31	48	20	28	A	0	92569.404	0.082	386.4002
25	5	21	25	4	22	E	1	92592.727	-0.093	264.2885
25	5	21	25	4	22	E	0	92597.385	0.012	135.9568
49	19	31	48	20	29	E	0	92598.800	0.005	386.3883
25	5	21	25	4	22	A	0	92607.841	0.029	135.9470
22	9	14	22	8	15	A	0	92651.079	-0.028	131.9137
15	10	5	14	10	4	A	0	92651.545	0.034	107.8846
15	10	6	14	10	5	A	0	92651.545	0.035	107.8846
15	10	5	14	10	4	E	0	92651.545	-0.129	107.8958
15	10	6	14	10	5	E	0	92652.096	-0.030	107.8832
15	4	12	14	4	11	E	0	92661.319	-0.016	92.0516
15	4	12	14	4	11	A	0	92662.169	-0.027	92.0409
27	8	19	26	9	18	A	0	92698.947	-0.042	155.5738
27	8	19	26	9	18	E	0	92782.859	0.027	155.5796
15	9	6	14	9	5	A	0	92823.685	-0.007	104.2765
15	9	6	14	9	5	E	0	92823.685	-0.116	104.2885
15	9	7	14	9	6	E	0	92824.478	-0.020	104.2767
22	9	13	22	8	15	E	0	92848.168	-0.208	131.9201
27	6	22	27	5	23	E	1	92958.054	-0.097	277.9514
8	3	6	7	4	3	E	0	9300.088	0.057	76.0805
27	6	22	27	5	23	E	0	93000.491	0.007	149.6207
27	6	22	27	5	23	A	0	93009.539	0.014	149.6115

J'	K'_a	K'_c	J''	K''_a	K''_c	Sym.	$v_{t_{\mathrm{CH_3}}}$	$\nu_{\mathrm{Obs.}}$	$\nu_{\mathrm{Obs.}} - \nu_{\mathrm{Calc.}}$	E_{low}
15	8	8	14	8	7	A	0	93067.782	-0.002	101.0577
15	8	7	14	8	6	E	0	93068.297	-0.035	101.0704
15	8	7	14	8	6	A	0	93068.752	-0.038	101.0577
15	8	8	14	8	7	E	0	93069.422	0.004	101.0595
21	9	12	21	8	13	A	0	93086.476	0.017	127.3068
3	1	2	3	0	3	E	1	9312.467	-0.020	196.8007
21	9	12	21	8	13	E	0	93149.122	-0.037	127.3163
21	9	13	21	8	14	E	0	93259.343	-0.031	127.3012
29	7	23	29	6	24	E	1	93308.926	-0.084	292.6506
8	3	6	7	4	3	A	0	9337.443	-0.041	76.0681
29	7	23	29	6	24	E	0	93393.820	-0.001	164.3220
21	9	13	21	8	14	A	0	93394.687	0.003	127.2957
29	7	23	29	6	24	A	0	93402.867	-0.001	164.3134
15	7	9	14	7	8	A	0	93426.139	-0.017	98.2331
15	7	9	14	7	8	E	0	93437.851	-0.015	98.2368
15	7	8	14	7	7	A	0	93448.042	-0.027	98.2335
16	2	14	15	3	13	A	1	93492.280	-0.004	222.0266
16	2	14	15	3	13	E	1	93517.858	-0.067	221.6717
16	2	14	15	3	13	A	0	93533.976	-0.017	93.3261
16	2	14	15	3	13	E	0	93533.976	-0.036	93.3371
20	1	19	20	0	20	E	0	93598.749	0.000	104.8304
20	1	19	20	1	20	E	0	93598.749	0.046	104.8304
20	2	19	20	0	20	E	0	93601.561	-0.053	104.8304
20	2	19	20	0	20	E	0	93601.561	-0.053	104.8304
20	2	19	20	0	20	E	0	93601.561	-0.053	104.8304
20	2	19	20	1	20	E	0	93601.561	-0.008	104.8304
51	20	31	50	21	29	E	0	93614.263	0.040	414.6055
20	1	19	20	0	20	A	0	93615.292	0.051	104.8188
20	2	19	20	1	20	A	0	93618.125	0.054	104.8188
34	11	24	33	12	21	A	0	93621.532	-0.042	211.0033
20	2	19	20	1	20	E	1	93692.989	-0.011	233.1586
30	6	24	30	5	25	E	1	93831.252	-0.234	298.6882
15	6	10	14	6	9	A	0	93898.750	-0.022	95.8098
35	11	24	35	10	25	E	0	93912.759	-0.057	218.5169
20	9	11	20	8	12	A	0	93919.337	0.022	122.9026
32	9	24	32	8	25	E	0	93920.809	-0.015	189.9138
15	6	10	14	6	9	E	0	93926.035	-0.016	95.8173
32	9	24	32	8	25	A	0	93941.101	-0.018	189.9062
20	9	11	20	8	12	E	0	93954.207	-0.051	122.9133
20	9	12	20	8	13	E	0	93958.917	-0.011	122.9016
35	11	24	35	10	25	A	0	93966.493	-0.004	218.5097
15	5	11	14	5	10	A	1	94005.156	0.003	222.4591
30	6	24	30	5	25	A	0	94015.222	-0.006	170.3521
30	6	24	30	5	25	E	0	94018.736	0.005	170.3607
15	5	11	14	5	10	A	0	94049.971	-0.020	93.7793
15	5	11	14	5	10	E	0	94052.098	-0.009	93.7892
20	9	12	20	8	13	A	0	94065.651	-0.006	122.8974
15	6	9	14	6	8	E	0	94177.553	-0.043	95.8291
15	6	9	14	6	8	A	0	94202.694	-0.014	95.8180
16	3	14	15	3	13	A	1	94303.454	0.017	222.0266
16	3	14	15	3	13	E	1	94373.359	-0.060	221.6717
31	8	24	31	7	25	E	0	94378.029	-0.006	180.0524
16	3	14	15	3	13	E	0	94378.681	-0.022	93.3371
16	3	14	15	3	13	A	0	94380.596	-0.018	93.3261
31	8	24	31	7	25	A	0	94389.481	0.004	180.0443
36	12	25	35	13	22	A	0	94397.094	-0.039	229.9870
51	13	38	51	12	39	A	0	94399.066	-0.085	377.1333
51	13	38	51	12	39	E	0	94425.211	-0.047	377.1386
7	5	3	6	4	3	E	0	94440.482	-0.035	74.6266
7	5	3	6	4	2	A	0	94464.570	-0.024	74.6195
7	5	2	6	4	2	E	0	94479.736	-0.031	74.6328
7	5	2	6	4	3	A	0	94496.502	0.022	74.6185
45	13	32	45	12	33	E	0	94554.824	-0.064	313.0174
19	9	10	19	8	11	A	0	94589.425	0.040	118.7201
50	14	36	50	13	37	E	0	94590.351	-0.020	368.9751
53	21	33	52	22	31	E	0	94591.982	0.012	444.0075
17	2	16	16	2	15	A	1	94596.909	0.019	222.9931
19	9	10	19	8	11	E	0	94598.334	-0.052	118.7318
36	12	25	35	13	23	E	0	94612.664	-0.034	229.9816
17	1	16	16	1	15	A	1	94617.455	-0.048	222.9917
17	1	16	16	2	15	E	1	94617.884	-0.033	222.6300
45	13	32	45	12	33	A	0	94624.232	-0.009	313.0108
17	1	16	16	2	15	E	0	94632.453	-0.028	94.2966
17	1	16	16	2	15	A	0	94633.727	-0.033	94.2853
17	2	16	16	1	15	A	1	94639.021	0.005	222.9917
17	2	16	16	2	15	E	1	94641.003	-0.038	222.6300
50	14	36	50	13	37	A	0	94653.380	-0.005	368.9690
17	2	16	16	2	15	A	0	94656.437	-0.033	94.2853
17	1	16	16	1	15	E	1	94662.908	-0.014	222.6285
17	1	16	16	1	15	E	0	94676.658	-0.018	94.2951
17	1	16	16	1	15	A	0	94678.046	-0.028	94.2838
17	2	16	16	1	15	E	1	94685.991	-0.055	222.6285
17	2	16	16	1	15	E	0	94699.287	-0.034	94.2951
17	2	16	16	1	15	A	0	94700.760	-0.024	94.2838
40	12	28	40	11	29	E	0	94742.991	-0.048	262.8603
18	0	18	17	1	17	A	1	94793.298	0.052	223.5347
18	1	18	17	0	17	A	1	94793.850	-0.005	223.5347
29	10	19	29	9	20	A	0	94806.026	-0.003	173.5056
18	0	18	17	1	17	E	1	94806.823	-0.018	223.1581
18	1	18	17	0	17	E	1	94807.465	-0.038	223.1581
40	12	28	40	11	29	A	0	94808.817	0.001	262.8535
54	14	40	54	13	41	A	0	94826.818	-0.081	415.3326
18	0	18	17	1	17	E	0	94828.137	-0.037	94.8276
18	1	18	17	1	17	E	0	94828.137	-0.244	94.8276
18	0	18	17	1	17	A	0	94828.674	0.081	94.8159
18	1	18	17	1	17	A	0	94828.674	-0.127	94.8159
18	0	18	17	0	17	E	0	94828.674	0.061	94.8276
18	1	18	17	0	17	E	0	94828.674	-0.146	94.8276
18	0	18	17	0	17	A	0	94829.225	0.192	94.8159
18	1	18	17	0	17	A	0	94829.225	-0.015	94.8159
54	14	40	54	13	41	E	0	94840.289	-0.032	415.3378
20	5	15	19	6	14	A	0	94845.299	0.010	113.6253
20	5	15	19	6	14	E	0	94883.597	0.027	113.6347
16	2	14	15	2	13	A	1	94894.018	0.031	221.9799
16	2	14	15	2	13	E	1	94986.247	-0.018	221.6227
16	2	14	15	2	13	E	0	94988.475	-0.026	93.2886
16	2	14	15	2	13	A	0	94991.494	-0.012	93.2774
22	2	20	22	1	21	A	1	95063.075	-0.006	244.4913
48	12	36	48	11	37	A	0	95068.901	-0.091	341.0799
38	13	26	37	14	23	A	0	95069.566	-0.057	250.1776
22	3	20	22	2	21	A	1	95082.546	-0.018	244.4914
18	9	10	18	8	11	E	0	95099.495	-0.021	114.7582
48	12	36	48	11	37	E	0	95102.485	-0.032	341.0854
18	9	9	18	8	10	E	0	95128.047	-0.049	114.7689
56	15	41	56	14	42	E	0	95133.676	-0.033	443.1077
18	9	9	18	8	10	A	0	95135.169	0.050	114.7567

J'	K'_a	K'_c	J''	K''_a	K''_c	Sym.	$v_{t_{\mathrm{CH_3}}}$	$\nu_{\mathrm{Obs.}}$	$\nu_{\mathrm{Obs.}} - \nu_{\mathrm{Calc.}}$	E_{low}
56	15	41	56	14	42	A	0	95159.543	-0.044	443.1023
18	9	10	18	8	11	A	0	95163.057	-0.033	114.7557
38	13	25	37	14	24	A	0	95342.103	-0.023	250.1772
34	11	23	33	12	22	A	0	95423.699	-0.037	211.0001
18	4	14	17	5	13	A	0	95436.432	-0.018	103.8120
18	4	14	17	5	13	E	0	95460.331	0.016	103.8221
22	2	20	22	1	21	E	0	95485.535	0.001	115.8071
22	2	20	22	1	21	A	0	95500.520	0.044	115.7961
22	3	20	22	2	21	E	0	95506.249	0.015	115.8071
22	3	20	22	2	21	A	0	95521.289	0.042	115.7961
17	9	9	17	8	10	E	0	95538.616	-0.025	111.0121
22	2	20	22	1	21	E	1	95551.316	-0.030	244.1370
17	9	8	17	8	9	E	0	95567.571	-0.050	111.0228
17	9	8	17	8	9	A	0	95582.707	0.065	111.0103
17	9	9	17	8	10	A	0	95593.751	-0.036	111.0099
55	22	34	54	23	31	A	0	95601.392	-0.030	474.6341
34	11	23	33	12	22	E	0	95641.186	-0.107	210.9982
55	22	33	54	23	31	E	0	95650.925	0.143	474.6282
16	3	14	15	2	13	A	1	95705.147	0.007	221.9799
40	14	27	39	15	24	A	0	95754.241	-0.048	271.5742
16	3	14	15	2	13	E	0	95833.170	-0.022	93.2886
16	3	14	15	2	13	A	0	95838.111	-0.016	93.2774
16	3	14	15	2	13	E	1	95841.738	-0.020	221.6227
40	14	27	39	15	25	E	0	95852.635	-0.023	271.5668
40	14	26	39	15	25	A	0	95854.719	-0.035	271.5740
40	14	26	39	15	24	E	0	95857.001	0.058	271.5797
32	8	25	31	9	22	E	0	95865.319	-0.041	186.7160
16	9	8	16	8	9	E	0	95903.113	-0.015	107.4811
16	9	7	16	8	8	E	0	95931.638	-0.053	107.4919
16	9	7	16	8	8	A	0	95950.402	0.151	107.4793
16	9	8	16	8	9	A	0	95954.323	-0.062	107.4791
33	7	26	33	6	27	E	1	96142.948	-0.289	321.6522
15	9	7	15	8	8	E	0	96203.224	-0.056	104.1640
15	9	6	15	8	7	E	0	96231.331	-0.050	104.1748
45	11	34	45	10	35	A	0	96261.867	-0.079	307.1790
55	15	40	55	14	41	E	0	96266.225	-0.027	430.7060
9	3	6	8	2	7	E	0	96280.612	-0.021	75.2529
9	3	6	8	2	7	A	0	96286.602	0.016	75.2413
45	11	34	45	10	35	E	0	96297.410	-0.021	307.1847
15	5	10	14	5	9	A	0	96299.462	-0.015	93.8724
15	5	10	14	5	9	E	0	96300.400	-0.011	93.8827
55	15	40	55	14	41	A	0	96313.101	-0.031	430.7003
10	4	7	9	3	6	E	0	96315.662	-0.034	78.4645
10	4	7	9	3	6	A	0	96339.399	-0.014	78.4531
15	3	12	14	3	11	A	1	96376.302	0.001	220.2629
24	3	21	24	2	22	A	1	96383.932	0.090	256.5284
33	7	26	33	6	27	A	0	96423.231	-0.017	193.3213
33	7	26	33	6	27	E	0	96433.824	0.013	193.3292
14	9	6	14	8	7	E	0	96448.194	-0.006	101.0595
15	3	12	14	3	11	E	1	96462.108	-0.028	219.9029
31	8	24	30	9	21	E	0	96469.175	-0.033	179.9826
14	9	5	14	8	6	E	0	96475.854	-0.058	101.0704
24	4	21	24	3	22	A	1	96485.395	0.085	256.5286
15	3	12	14	3	11	E	0	96486.507	-0.019	91.5695
15	3	12	14	3	11	A	0	96489.810	-0.021	91.5582
28	5	23	28	4	24	E	1	96506.306	-0.152	283.4896
42	15	27	41	16	26	A	0	96525.480	0.016	294.1758
42	15	28	41	16	26	E	0	96556.621	0.001	294.1673
42	15	27	41	16	25	E	0	96557.292	0.028	294.1801
28	5	23	28	4	24	E	0	96587.364	0.018	155.1596
28	5	23	28	4	24	A	0	96592.010	-0.003	155.1505
57	23	35	56	24	32	A	0	96642.850	-0.030	506.4504
13	9	5	13	8	6	E	0	96645.849	-0.004	98.1666
57	23	35	56	24	33	E	0	96649.870	0.013	506.4378
33	9	25	33	8	26	E	0	96671.591	-0.017	196.8119
13	9	4	13	8	5	E	0	96673.194	-0.056	98.1776
33	9	25	33	8	26	A	0	96688.325	-0.014	196.8045
13	9	5	13	8	6	A	0	96694.114	-0.055	98.1648
32	10	22	31	11	21	A	0	96762.807	-0.050	193.2200
24	3	21	24	2	22	E	0	96769.904	0.012	127.8643
24	3	21	24	2	22	A	0	96782.728	0.029	127.8539
24	3	21	24	2	22	E	1	96802.602	-0.053	256.1952
12	9	4	12	8	5	E	0	96803.222	-0.021	95.4843
12	9	3	12	8	4	E	0	96830.321	-0.069	95.4953
12	9	4	12	8	5	A	0	96851.309	-0.003	95.4826
24	4	21	24	3	22	E	0	96877.551	0.010	127.8645
24	4	21	24	3	22	A	0	96890.745	0.033	127.8541
24	4	21	24	3	22	E	1	96913.575	-0.056	256.1954
11	9	3	11	8	4	E	0	96926.548	-0.019	93.0117
26	4	22	26	3	23	A	1	96934.709	0.189	269.6306
11	9	2	11	8	3	E	0	96953.451	-0.068	93.0227
9	4	5	8	3	6	E	0	96973.826	-0.006	76.3907
11	9	3	11	8	4	A	0	96974.472	0.003	93.0099
9	4	5	8	3	6	A	0	96977.244	0.002	76.3795
31	9	23	30	10	20	A	0	96988.320	-0.032	183.0208
31	9	23	30	10	20	E	0	97000.333	0.032	183.0273
10	9	1	10	8	2	E	0	97048.133	-0.010	90.7589
10	9	2	10	8	3	A	0	97069.125	0.007	90.7462
26	4	22	26	3	23	E	1	97253.883	-0.108	269.3183
26	4	22	26	3	23	E	0	97266.637	0.019	140.9873
26	4	22	26	3	23	A	0	97276.318	0.021	140.9775
44	16	29	43	17	27	E	0	97332.520	-0.027	317.9726
44	16	28	43	17	26	E	0	97338.337	0.040	317.9849
42	10	32	42	9	33	A	0	97407.025	-0.066	275.4406
42	10	32	42	9	33	E	0	97439.750	-0.003	275.4468
28	10	18	28	9	19	A	0	97561.431	0.006	167.2932
28	10	18	28	9	19	E	0	97578.133	-0.064	167.3004
26	5	22	26	4	23	E	0	97706.762	0.010	140.9884
26	5	22	26	4	23	E	1	97707.529	-0.079	269.3195
26	5	22	26	4	23	A	0	97717.900	0.029	140.9786
59	24	35	58	25	33	E	0	97740.195	0.019	539.4606
36	8	28	36	7	29	A	0	97747.226	-0.026	218.4998
36	8	28	36	7	29	E	0	97765.832	0.014	218.5071
39	9	30	39	8	31	A	0	98016.539	-0.055	245.8768
28	6	23	28	5	24	E	1	98034.522	-0.102	283.4949
39	9	30	39	8	31	E	0	98043.043	-0.001	245.8835
28	6	23	28	5	24	E	0	98071.925	0.009	155.1646
21	2	20	21	1	21	A	1	98080.459	-0.098	237.2091
28	6	23	28	5	24	A	0	98081.199	0.011	155.1555
32	10	23	32	9	24	E	0	98104.010	-0.030	193.0466
32	10	23	32	9	24	A	0	98143.117	-0.004	193.0397
30	7	24	30	6	25	E	1	98147.325	-0.097	298.7080
46	17	30	45	18	28	E	0	98166.060	-0.018	342.9822
46	17	29	45	18	27	E	0	98177.807	0.006	342.9940
25	7	18	24	8	17	A	0	98178.864	-0.053	141.8103
31	10	22	31	9	23	E	0	98218.146	-0.039	186.2629

J'	K'_a	K'_c	J''	K''_a	K''_c	Sym.	$v_{t_{CH_3}}$	$\nu_{Obs.}$	$\nu_{Obs.} - \nu_{Calc.}$	E_{low}
30	7	24	30	6	25	E	0	98223.828	-0.001	170.3797
30	7	24	30	6	25	A	0	98232.242	-0.001	170.3712
25	7	18	24	8	17	E	0	98243.048	0.056	141.8177
31	10	22	31	9	23	A	0	98264.215	0.003	186.2560
16	4	13	15	4	12	E	0	98355.356	-0.019	95.1424
16	4	13	15	4	12	A	0	98356.672	-0.023	95.1317
33	10	24	33	9	25	E	0	98452.625	-0.033	200.0366
33	10	24	33	9	25	A	0	98486.394	-0.006	200.0297
21	1	20	21	0	21	E	0	98552.034	-0.000	108.5069
21	2	20	21	1	21	E	0	98553.439	0.011	108.5069
21	1	20	21	0	21	A	0	98569.508	0.053	108.4953
21	2	20	21	1	21	A	0	98570.910	0.055	108.4953
32	8	25	32	7	26	E	0	98649.064	-0.002	186.6232
21	2	20	21	1	21	E	1	98650.520	-0.010	236.8343
32	8	25	32	7	26	A	0	98658.625	-0.008	186.6153
16	12	4	15	12	3	A	0	98663.388	0.003	119.3420
16	12	5	15	12	4	A	0	98663.388	0.003	119.3420
16	12	5	15	12	4	E	0	98663.829	-0.000	119.3375
30	10	21	30	9	22	E	0	98687.098	-0.035	179.6904
34	11	23	34	10	24	E	0	98701.555	-0.066	210.8962
30	10	21	30	9	22	A	0	98743.706	-0.006	179.6836
34	11	23	34	10	24	A	0	98744.689	-0.004	210.8893
16	11	6	15	11	5	E	0	98780.403	0.056	114.9626
15	4	11	14	4	10	A	0	98839.214	-0.012	92.5375
15	4	11	14	4	10	E	0	98839.871	-0.005	92.5486
16	10	6	15	10	5	A	0	98933.561	0.019	110.9751
16	10	6	15	10	5	E	0	98933.561	-0.161	110.9863
16	10	7	15	10	6	E	0	98934.272	-0.019	110.9737
17	3	14	16	4	13	A	0	99000.821	-0.019	98.4126
17	3	14	16	4	13	E	0	99008.544	-0.005	98.4232
48	18	31	47	19	29	E	0	99045.003	-0.006	369.1958
17	2	15	16	3	14	A	1	99049.516	-0.009	225.1722
48	18	30	47	19	28	E	0	99062.497	0.006	369.2068
17	2	15	16	3	14	E	1	99092.491	-0.052	224.8197
17	2	15	16	3	14	E	0	99107.858	-0.020	96.4852
17	2	15	16	3	14	A	0	99108.708	-0.028	96.4743
16	9	7	15	9	6	A	0	99143.584	-0.030	107.3727
16	9	7	15	9	6	E	0	99143.584	-0.112	107.3848
16	9	8	15	9	7	E	0	99144.547	-0.017	107.3730
34	10	25	34	9	26	E	0	99356.549	-0.023	207.2272
34	10	25	34	9	26	A	0	99385.606	-0.017	207.2204
29	10	20	29	9	21	E	0	99398.917	-0.022	173.3331
11	4	8	10	3	7	E	0	99441.451	-0.045	80.6474
16	8	9	15	8	8	A	0	99441.959	0.032	104.1621
16	8	8	15	8	7	E	0	99443.373	-0.013	104.1748
16	8	8	15	8	7	A	0	99444.693	-0.060	104.1621
16	8	9	15	8	8	E	0	99444.693	-0.022	104.1640
11	4	8	10	3	7	A	0	99463.453	-0.004	80.6360
29	10	20	29	9	21	A	0	99473.084	-0.003	173.3265
17	3	15	16	3	14	A	1	99506.967	-0.022	225.1722
17	3	15	16	3	14	E	1	99578.123	-0.047	224.8197
17	3	15	16	3	14	E	0	99585.881	-0.022	96.4852
17	3	15	16	3	14	A	0	99587.916	-0.033	96.4743
27	10	17	27	9	18	A	0	99701.267	-0.002	161.3293
18	1	17	17	2	16	A	1	99715.100	-0.009	226.1485
18	2	17	17	2	16	A	1	99725.966	-0.008	226.1485
18	1	17	17	1	16	A	1	99736.621	-0.001	226.1478
18	2	17	17	1	16	A	1	99747.478	-0.010	226.1478
6	6	1	5	5	1	E	0	99752.486	-0.028	75.1025
18	1	17	17	2	16	E	1	99758.273	-0.036	225.7869
18	2	17	17	2	16	E	1	99769.995	-0.065	225.7869
18	1	17	17	2	16	E	0	99774.052	-0.025	97.4539
18	1	17	17	2	16	A	0	99775.361	-0.027	97.4427
18	1	17	17	1	16	E	1	99781.407	-0.026	225.7861
18	2	17	17	2	16	E	0	99785.534	-0.018	97.4539
18	2	17	17	2	16	A	0	99786.875	-0.023	97.4427
6	6	0	5	5	0	E	0	99790.808	-0.045	75.1100
18	2	17	17	1	16	E	1	99793.141	-0.043	225.7861
6	6	0	5	5	1	A	0	99796.087	-0.006	75.0962
18	1	17	17	1	16	E	0	99796.701	-0.021	97.4532
18	1	17	17	1	16	A	0	99798.072	-0.026	97.4419
18	2	17	17	1	16	E	0	99808.170	-0.027	97.4532
18	2	17	17	1	16	A	0	99809.581	-0.027	97.4419
17	2	15	16	2	14	A	1	99860.674	-0.004	225.1452
16	7	10	15	7	9	A	0	99874.003	-0.033	101.3494
16	7	10	15	7	9	E	0	99896.567	-0.013	101.3535
16	7	9	15	7	8	E	0	99904.942	-0.035	101.3633
54	15	39	54	14	40	E	0	99917.678	-0.026	418.5013
16	7	9	15	7	8	A	0	99925.623	-0.030	101.3506
17	2	15	16	2	14	E	1	99947.982	-0.054	224.7911
17	2	15	16	2	14	E	0	99952.550	-0.019	96.4570
17	2	15	16	2	14	A	0	99955.335	-0.022	96.4460
19	1	19	18	1	18	E	0	99958.637	-0.144	97.9908
19	0	19	18	1	18	E	0	99958.638	-0.046	97.9908
19	0	19	18	1	18	A	0	99959.016	-0.084	97.9791
19	1	19	18	0	18	E	0	99959.016	0.028	97.9907
19	1	19	18	0	18	A	0	99959.390	-0.015	97.9791
54	15	39	54	14	40	A	0	99981.942	-0.012	418.4957
34	9	26	34	8	27	E	0	100029.478	-0.020	203.8906
23	2	21	23	1	22	A	1	100041.040	-0.011	248.6730
34	9	26	34	8	27	A	0	100043.027	-0.012	203.8833
23	3	21	23	2	22	A	1	100051.113	-0.011	248.6730
49	14	35	49	13	36	E	0	100088.991	-0.043	357.8881
49	14	35	49	13	36	A	0	100162.198	-0.002	357.8821
28	10	19	28	9	20	E	0	100250.406	-0.040	167.1944
16	5	12	15	5	11	A	0	100295.117	-0.027	96.9164
16	5	12	15	5	11	E	0	100296.050	0.003	96.9265
17	3	15	16	2	14	A	1	100318.144	0.002	225.1452
28	10	19	28	9	20	A	0	100351.936	-0.014	167.1879
16	6	11	15	6	10	A	0	100380.028	-0.022	98.9419
16	6	11	15	6	10	E	0	100394.949	-0.019	98.9503
31	6	25	31	5	26	E	1	100411.334	-0.204	304.9246
17	3	15	16	2	14	E	0	100430.563	-0.029	96.4570
17	3	15	16	2	14	E	1	100433.642	-0.021	224.7911
17	3	15	16	2	14	A	0	100434.545	-0.025	96.4460
23	2	21	23	1	22	E	0	100488.071	0.009	119.9909
23	3	21	23	2	22	E	0	100498.809	0.008	119.9909
23	2	21	23	1	22	A	0	100503.979	0.042	119.9800
23	3	21	23	2	22	A	0	100514.751	0.036	119.9800
8	5	4	7	4	3	A	0	100516.586	-0.022	76.0681
8	5	4	7	4	4	E	0	100547.757	0.041	76.0735
23	2	21	23	1	22	E	1	100559.630	-0.042	248.3201
8	5	3	7	4	3	E	0	100561.334	-0.031	76.0805
39	12	27	39	11	28	E	0	100574.727	-0.057	254.0775
8	5	3	7	4	4	A	0	100633.313	0.029	76.0645
39	12	27	39	11	28	A	0	100636.220	0.002	254.0710

J'	K'_a	K'_c	J''	K''_a	K''_c	Sym.	$v_{t_{CH_3}}$	$\nu_{Obs.}$	$\nu_{Obs.} - \nu_{Calc.}$	E_{low}
44	13	31	44	12	32	E	0	100744.216	-0.051	303.0746
44	13	31	44	12	32	A	0	100816.047	-0.005	303.0684
60	16	44	60	15	45	E	0	100846.306	-0.025	498.1719
60	16	44	60	15	45	A	0	100869.522	-0.064	498.1670
35	10	26	35	9	27	E	0	100882.605	-0.018	214.6130
52	20	33	51	21	31	E	0	100904.930	-0.011	425.2338
35	10	26	35	9	27	A	0	100907.224	-0.033	214.6062
65	27	39	64	28	36	A	0	100910.480	-0.046	645.7337
65	27	38	64	28	37	A	0	100910.480	-0.046	645.7337
16	6	10	15	6	9	E	0	100961.151	-0.014	98.9705
16	6	10	15	6	9	A	0	100973.353	-0.011	98.9603
27	10	18	27	9	19	E	0	101163.089	-0.014	161.2762
27	10	18	27	9	19	A	0	101295.857	-0.008	161.2700
26	10	16	26	9	17	A	0	101367.463	0.006	155.6059
26	10	16	26	9	17	E	0	101439.134	-0.073	155.6135
16	3	13	15	3	12	A	1	101447.957	0.011	223.4777
58	15	43	58	14	44	E	0	101454.121	-0.037	468.4034
25	3	22	25	2	23	A	1	101463.844	0.088	261.2176
55	14	41	55	13	42	A	0	101481.249	-0.113	427.3153
55	14	41	55	13	42	E	0	101512.120	-0.049	427.3199
25	4	22	25	3	23	A	1	101518.868	0.104	261.2177
16	3	13	15	3	12	E	1	101567.089	-0.020	223.1206
16	3	13	15	3	12	E	0	101574.998	-0.023	94.7880
16	3	13	15	3	12	A	0	101579.230	-0.025	94.7768
59	16	43	59	15	44	E	0	101666.476	-0.015	484.8834
59	16	43	59	15	44	A	0	101712.099	-0.049	484.8783
54	21	34	53	22	31	A	0	101854.678	-0.004	455.0700
25	3	22	25	2	23	E	0	101875.790	0.007	132.5566
12	4	9	11	3	8	E	0	101886.491	-0.043	83.0597
25	3	22	25	2	23	A	0	101889.648	0.033	132.5463
12	4	9	11	3	8	A	0	101907.848	-0.004	83.0482
25	3	22	25	2	23	E	1	101915.397	-0.066	260.8868
25	4	22	25	3	23	E	0	101934.352	0.010	132.5567
25	4	22	25	3	23	A	0	101948.408	0.024	132.5464
25	4	22	25	3	23	E	1	101975.943	-0.065	260.8869
26	10	17	26	9	18	E	0	102087.668	-0.034	155.5796
37	12	26	36	13	23	E	0	102204.667	0.036	237.5590
27	4	23	27	3	24	A	1	102218.557	0.205	274.8294
26	10	17	26	9	18	A	0	102240.301	-0.006	155.5738
29	5	24	29	4	25	E	1	102266.151	-0.137	289.2058
29	5	24	29	4	25	E	0	102328.199	0.005	160.8762
29	5	24	29	4	25	A	0	102334.971	0.006	160.8672
27	4	23	27	3	24	E	1	102579.361	-0.102	274.5205
27	4	23	27	3	24	E	0	102581.639	0.010	146.1900
27	4	23	27	3	24	A	0	102592.671	0.025	146.1804
52	13	39	52	12	40	A	0	102607.996	-0.107	388.3740
52	13	39	52	12	40	E	0	102646.539	-0.041	388.3787
25	10	15	25	9	16	A	0	102682.791	0.000	150.1164
33	11	22	33	10	23	E	0	102704.140	-0.070	203.5440
33	11	22	33	10	23	A	0	102730.193	-0.003	203.5374
25	10	15	25	9	16	E	0	102753.917	-0.056	150.1247
27	5	23	27	4	24	E	0	102832.871	0.003	146.1906
27	5	23	27	4	24	E	1	102839.103	-0.097	274.5211
27	5	23	27	4	24	A	0	102844.788	0.030	146.1809
39	13	27	38	14	24	A	0	102976.229	-0.032	258.1520
25	10	16	25	9	17	E	0	102990.202	-0.021	150.1047
22	1	21	22	0	22	A	1	103005.140	-0.107	241.0555
22	2	21	22	1	22	A	1	103005.748	-0.138	241.0555
36	10	27	36	9	28	E	0	103064.520	-0.028	222.1882
36	10	27	36	9	28	A	0	103084.944	-0.022	222.1815
25	10	16	25	9	17	A	0	103140.883	0.009	150.0998
39	13	27	38	14	25	E	0	103171.338	-0.045	258.1456
29	6	24	29	5	25	E	1	103188.080	-0.105	289.2087
29	6	24	29	5	25	E	0	103220.703	0.003	160.8789
29	6	24	29	5	25	A	0	103230.443	0.008	160.8700
31	7	25	31	6	26	E	0	103251.893	0.003	176.6082
31	7	25	31	6	26	A	0	103260.090	0.005	176.5999
33	8	26	33	7	27	E	0	103290.532	-0.009	193.3665
33	8	26	33	7	27	A	0	103298.763	-0.011	193.3588
25	10	15	25	9	17	E	0	103353.707	-0.047	150.1047
39	13	26	38	14	24	E	0	103395.344	0.042	258.1586
34	7	27	34	6	28	E	1	103412.036	-0.250	328.5819
7	2	6	6	3	3	A	0	10347.064	-0.016	73.3142
39	13	26	38	14	25	A	0	103486.777	-0.030	258.1512
22	1	21	22	0	22	E	0	103502.674	-0.002	112.3546
22	1	21	22	1	22	E	0	103502.674	0.008	112.3546
22	2	21	22	0	22	E	0	103503.366	-0.004	112.3546
22	2	21	22	0	22	E	0	103503.366	-0.004	112.3546
22	2	21	22	0	22	E	0	103503.366	-0.004	112.3546
22	2	21	22	1	22	E	0	103503.366	0.005	112.3546
7	2	6	6	3	3	E	0	10351.164	-0.004	73.3256
22	1	21	22	0	22	A	0	103521.083	0.060	112.3430
22	2	21	22	1	22	A	0	103521.758	0.048	112.3430
41	14	28	40	15	25	A	0	103576.399	-0.035	279.9589
22	1	21	22	0	22	E	1	103605.362	-0.004	240.6811
22	2	21	22	1	22	E	1	103606.071	-0.003	240.6811
37	12	25	36	13	24	A	0	103642.344	-0.029	237.5495
34	7	27	34	6	28	A	0	103654.366	-0.016	200.2513
34	7	27	34	6	28	E	0	103660.745	0.006	200.2589
16	5	11	15	5	10	A	0	103689.616	-0.041	97.0846
16	5	11	15	5	10	E	0	103691.883	-0.023	97.0950
41	14	28	40	15	26	E	0	103707.488	-0.029	279.9516
24	10	14	24	9	15	A	0	103740.377	0.012	144.8557
41	14	27	40	15	25	E	0	103740.934	-0.116	279.9645
24	10	14	24	9	15	E	0	103789.793	-0.055	144.8650
13	4	10	12	3	9	E	0	103826.766	-0.104	85.6920
17	4	14	16	4	13	A	1	103833.389	0.005	227.1013
24	10	15	24	9	16	E	0	103842.923	-0.031	144.8511
23	6	17	22	7	16	A	0	103844.665	-0.033	129.2340
13	4	10	12	3	9	A	0	103847.682	-0.014	85.6806
58	23	36	57	24	33	A	0	103862.172	-0.038	518.3256
23	6	17	22	7	16	E	0	103894.805	0.036	129.2426
17	4	14	16	4	13	E	0	103915.460	-0.014	98.4232
17	4	14	16	4	13	A	0	103917.208	-0.010	98.4126
35	9	27	35	8	28	E	0	103932.737	-0.029	211.1462
17	4	14	16	4	13	E	1	103935.430	-0.039	226.7561
35	9	27	35	8	28	A	0	103943.584	-0.017	211.1390
24	10	15	24	9	16	A	0	103970.794	-0.002	144.8474
24	10	14	24	9	16	E	0	104206.980	-0.361	144.8511
49	12	37	49	11	38	A	0	104223.397	-0.091	351.5819
49	12	37	49	11	38	E	0	104263.930	-0.022	351.5869
43	15	28	42	16	27	A	0	104289.290	0.014	302.9715
43	15	28	42	16	26	E	0	104306.300	0.045	302.9758
18	2	16	17	3	15	A	1	104423.210	-0.007	228.4914
18	2	16	17	3	15	E	1	104478.155	-0.038	228.1413
18	2	16	17	3	15	E	0	104492.747	0.015	99.8070

J'	K'_a	K'_c	J''	K''_a	K''_c	Sym.	$v_{t_{CH_3}}$	$\nu_{Obs.}$	$\nu_{Obs.} - \nu_{Calc.}$	E_{low}
18	2	16	17	3	15	A	0	104494.171	0.014	99.7962
23	10	13	23	9	14	A	0	104606.109	0.056	139.8199
23	10	14	23	9	15	E	0	104619.046	0.016	139.8183
23	10	13	23	9	14	E	0	104626.515	-0.056	139.8303
35	11	24	34	12	23	A	0	104632.908	-0.117	218.1539
18	3	16	17	3	15	A	1	104675.838	0.020	228.4914
23	10	14	23	9	15	A	0	104717.010	0.001	139.8159
18	3	16	17	3	15	E	1	104748.042	-0.015	228.1413
10	4	6	9	3	7	E	0	104755.319	-0.006	78.2400
18	3	16	17	3	15	E	0	104757.580	-0.003	99.8070
18	3	16	17	3	15	A	0	104759.705	-0.013	99.7962
10	4	6	9	3	7	A	0	104763.519	0.011	78.2287
19	1	18	18	2	17	A	1	104848.280	0.002	229.4750
19	2	18	18	2	17	A	1	104853.728	0.016	229.4750
19	1	18	18	1	17	A	1	104859.151	0.008	229.4747
19	2	18	18	1	17	A	1	104864.609	0.032	229.4747
18	2	16	17	2	15	A	1	104880.672	-0.009	228.4762
60	24	37	59	25	34	A	0	104889.281	-0.001	551.7567
19	1	18	18	2	17	E	1	104891.708	-0.038	229.1149
19	2	18	18	2	17	E	1	104897.680	0.020	229.1149
19	1	18	18	1	17	E	1	104903.442	-0.056	229.1145
19	1	18	18	2	17	E	0	104908.793	-0.014	100.7824
19	2	18	18	1	17	E	1	104909.392	-0.019	229.1145
19	1	18	18	2	17	A	0	104910.107	-0.025	100.7712
19	2	18	18	2	17	E	0	104914.545	-0.021	100.7824
19	2	18	18	2	17	A	0	104915.899	-0.010	100.7712
19	1	18	18	1	17	E	0	104920.259	-0.023	100.7820
19	1	18	18	1	17	A	0	104921.626	-0.016	100.7708
19	2	18	18	1	17	E	0	104926.024	-0.017	100.7820
19	2	18	18	1	17	A	0	104927.406	-0.014	100.7708
18	2	16	17	2	15	E	0	104970.732	-0.024	99.7911
18	2	16	17	2	15	A	0	104973.363	-0.007	99.7802
24	3	22	24	2	23	A	1	105014.671	0.051	253.0257
20	0	20	19	0	19	A	1	105051.737	-0.022	230.0297
20	0	20	19	1	19	A	1	105051.737	0.069	230.0298
20	1	20	19	0	19	A	1	105051.737	-0.065	230.0297
20	1	20	19	1	19	A	1	105051.737	0.026	230.0298
20	0	20	19	0	19	E	1	105065.099	-0.050	229.6540
20	0	20	19	1	19	E	1	105065.099	0.050	229.6540
20	1	20	19	0	19	E	1	105065.099	-0.097	229.6540
20	1	20	19	1	19	E	1	105065.099	0.003	229.6540
20	0	20	19	0	19	E	0	105089.148	-0.052	101.3250
20	0	20	19	1	19	E	0	105089.148	0.045	101.3250
20	1	20	19	0	19	E	0	105089.148	-0.098	101.3250
20	1	20	19	1	19	E	0	105089.148	-0.000	101.3250
20	0	20	19	0	19	A	0	105089.571	-0.042	101.3134
20	0	20	19	1	19	A	0	105089.571	0.055	101.3134
20	1	20	19	0	19	A	0	105089.571	-0.088	101.3134
20	1	20	19	1	19	A	0	105089.571	0.010	101.3134
28	8	20	27	9	19	A	0	105113.353	-0.078	161.2700
18	3	16	17	2	15	A	1	105133.294	0.012	228.4762
58	16	42	58	15	43	E	0	105167.020	-0.013	471.7876
28	8	20	27	9	19	E	0	105189.125	0.048	161.2762
58	16	42	58	15	43	A	0	105231.649	-0.025	471.7824
18	3	16	17	2	15	E	0	105235.601	-0.006	99.7911
17	10	7	16	10	6	A	0	105236.552	0.028	114.2752
17	10	7	16	10	6	E	0	105236.552	-0.169	114.2864
17	10	8	16	10	7	E	0	105237.416	-0.008	114.2738
18	3	16	17	2	15	A	0	105238.909	-0.022	99.7802
22	10	13	22	9	14	E	0	105300.925	-0.022	135.0057
22	10	12	22	9	13	E	0	105323.330	-0.030	135.0172
22	10	12	22	9	13	A	0	105324.982	-0.020	135.0060
22	10	13	22	9	14	A	0	105376.019	-0.012	135.0042
16	4	12	15	4	11	E	0	105461.271	-0.025	95.8455
16	4	12	15	4	11	A	0	105461.706	0.003	95.8345
24	2	22	24	1	23	E	0	105480.727	0.018	124.3458
14	4	11	13	3	10	E	0	105483.535	-0.039	88.5330
24	3	22	24	2	23	E	0	105486.259	0.031	124.3458
53	15	38	53	14	39	E	0	105487.867	-0.005	406.5197
17	9	9	16	9	8	E	0	105491.339	-0.008	110.6801
24	2	22	24	1	23	A	0	105497.540	0.041	124.3349
24	3	22	24	2	23	A	0	105503.077	0.038	124.3349
14	4	11	13	3	10	A	0	105503.615	0.007	88.5216
24	2	22	24	1	23	E	1	105557.854	-0.039	252.6742
24	3	22	24	2	23	E	1	105563.566	-0.047	252.6742
53	15	38	53	14	39	A	0	105563.980	-0.001	406.5143
47	17	31	46	18	29	E	0	105735.645	-0.008	352.6012
37	8	29	37	7	30	A	0	105742.498	-0.017	226.1285
47	17	30	46	18	28	E	0	105746.530	0.026	352.6129
46	11	35	46	10	36	A	0	105749.175	-0.079	316.9495
37	8	29	37	7	30	E	0	105756.540	0.012	226.1355
46	11	35	46	10	36	E	0	105786.691	-0.021	316.9548
19	4	15	18	5	14	A	0	105795.375	-0.009	107.5622
19	4	15	18	5	14	E	0	105815.169	0.022	107.5722
17	8	10	16	8	9	A	0	105850.574	-0.001	107.4791
17	8	9	16	8	8	E	0	105854.346	-0.004	107.4919
17	8	10	16	8	9	E	0	105855.822	-0.013	107.4811
17	8	9	16	8	8	A	0	105857.868	-0.040	107.4793
38	12	26	38	11	27	E	0	105860.899	-0.050	245.5674
21	10	12	21	9	13	E	0	105888.652	-0.002	130.4119
7	6	2	6	5	2	E	0	105898.466	-0.024	76.3356
37	10	28	37	9	29	E	0	105899.770	-0.015	229.9478
37	10	28	37	9	29	A	0	105916.243	-0.020	229.9412
21	10	11	21	9	12	A	0	105928.061	0.033	130.4118
7	6	1	6	5	1	E	0	105936.821	-0.053	76.3431
7	6	1	6	5	2	A	0	105942.085	-0.205	76.3293
21	10	12	21	9	13	A	0	105950.380	0.005	130.4110
18	3	15	17	4	14	A	0	106240.909	-0.003	101.8789
18	3	15	17	4	14	E	0	106245.532	0.013	101.8894
17	3	14	16	3	13	A	1	106277.733	0.031	226.8616
17	7	11	16	7	10	A	0	106359.202	-0.001	104.6809
20	10	11	20	9	12	E	0	106390.924	-0.024	126.0357
17	7	11	16	7	10	E	0	106393.222	0.002	104.6857
17	3	14	16	3	13	E	0	106414.302	-0.023	98.1762
20	10	10	20	9	11	E	0	106415.782	-0.052	126.0473
17	3	14	16	3	13	A	0	106419.088	-0.002	98.1651
17	5	13	16	5	12	A	0	106428.230	0.032	100.2619
17	5	13	16	5	12	E	0	106428.230	-0.068	100.2720
20	10	10	20	9	11	A	0	106436.651	0.094	126.0354
17	7	10	16	7	9	E	0	106440.832	-0.005	104.6958
9	5	5	8	4	4	A	0	106443.525	0.006	77.7314
20	10	11	20	9	12	A	0	106445.806	-0.032	126.0351
17	7	10	16	7	9	A	0	106472.575	-0.006	104.6838
26	3	23	26	2	24	A	1	106515.717	0.108	266.0776
26	4	23	26	3	24	A	1	106545.206	0.129	266.0777
48	14	34	48	13	35	E	0	106557.125	-0.031	347.0498

J'	K_a'	K_c'	J''	K_a''	K_c''	Sym.	$v_{t_{CH_3}}$	$\nu_{Obs.}$	$\nu_{Obs.} - \nu_{Calc.}$	E_{low}
49	18	31	48	19	29	E	0	106563.849	-0.022	379.2377
64	17	47	64	16	48	E	0	106571.687	0.002	556.9939
64	17	47	64	16	48	A	0	106591.835	-0.064	556.9895
32	6	26	32	5	27	E	1	106613.022	-0.155	311.3277
48	14	34	48	13	35	A	0	106634.205	0.013	347.0442
43	10	33	43	9	34	A	0	106702.361	-0.059	284.4885
15	4	12	14	3	11	A	1	106728.242	0.028	220.2629
43	10	33	43	9	34	E	0	106733.318	-0.006	284.4943
32	6	26	32	5	27	E	0	106739.177	0.010	183.0008
32	6	26	32	5	27	A	0	106741.940	0.005	182.9926
40	9	31	40	8	32	A	0	106753.312	-0.045	254.2111
40	9	31	40	8	32	E	0	106775.927	0.014	254.2174
9	5	4	8	4	5	A	0	106790.179	0.017	77.7211
19	10	10	19	9	11	E	0	106818.071	-0.012	121.8756
19	10	9	19	9	10	E	0	106842.421	-0.032	121.8872
17	6	12	16	6	11	A	0	106854.053	-0.007	102.2902
17	6	12	16	6	11	E	0	106861.487	-0.010	102.2991
19	10	10	19	9	11	A	0	106869.605	-0.077	121.8751
36	11	26	36	10	27	E	0	106932.853	-0.021	225.6261
26	3	23	26	2	24	E	0	106952.617	0.022	137.4197
26	3	23	26	2	24	A	0	106967.458	0.062	137.4095
36	11	26	36	10	27	A	0	106972.595	0.001	225.6200
26	4	23	26	3	24	E	0	106984.077	0.006	137.4198
26	3	23	26	2	24	E	1	106998.472	-0.091	265.7493
26	4	23	26	3	24	A	0	106999.058	0.067	137.4095
43	13	30	43	12	31	E	0	107005.094	-0.036	293.3980
26	4	23	26	3	24	E	1	107031.163	-0.038	265.7493
63	17	46	63	16	47	E	0	107043.209	-0.006	542.8194
35	11	25	35	10	26	E	0	107068.664	-0.034	217.9781
43	13	30	43	12	31	A	0	107073.653	-0.001	293.3922
63	17	46	63	16	47	A	0	107086.946	-0.057	542.8147
35	11	25	35	10	26	A	0	107114.800	-0.002	217.9721
33	10	23	32	11	22	A	0	107128.848	-0.023	199.9640
15	4	12	14	3	11	A	0	107130.647	0.006	91.5582
21	5	16	20	6	15	E	0	107147.309	0.037	117.8359
18	10	9	18	9	10	E	0	107179.509	0.002	117.9304
18	10	8	18	9	9	E	0	107203.317	-0.050	117.9421
37	11	27	37	10	28	E	0	107290.326	-0.033	233.4802
37	11	27	37	10	28	A	0	107324.770	-0.004	233.4742
17	10	8	17	9	9	E	0	107483.531	-0.002	114.1989
17	10	7	17	9	8	E	0	107506.934	-0.021	114.2106
17	10	8	17	9	9	A	0	107532.543	-0.203	114.1986
34	11	24	34	10	25	E	0	107590.949	-0.005	210.5414
34	11	24	34	10	25	A	0	107646.025	0.008	210.5355
16	10	7	16	9	8	E	0	107737.447	-0.008	110.6801
16	10	6	16	9	7	E	0	107760.484	-0.030	110.6919
16	10	7	16	9	8	A	0	107786.166	-0.042	110.6798
28	4	24	28	3	25	E	0	107819.302	0.013	151.5631
28	4	24	28	3	25	E	1	107825.823	-0.086	279.8930
28	4	24	28	3	25	A	0	107831.517	0.036	151.5536
30	5	25	30	4	26	E	1	107835.908	-0.125	295.0912
30	5	25	30	4	26	E	0	107882.691	0.011	166.7621
30	5	25	30	4	26	A	0	107891.205	0.021	166.7532
17	6	11	16	6	10	E	0	107928.715	0.003	102.3383
17	6	11	16	6	10	A	0	107932.777	-0.003	102.3284
15	10	6	15	9	7	E	0	107947.726	-0.002	107.3730
28	5	24	28	4	25	E	0	107960.570	0.019	151.5634
15	10	5	15	9	6	E	0	107970.440	-0.047	107.3848
28	5	24	28	4	25	E	1	107972.300	-0.092	279.8933
28	5	24	28	4	25	A	0	107973.326	0.062	151.5539
15	10	6	15	9	7	A	0	107996.183	0.004	107.3727
34	8	27	34	7	28	E	1	108105.396	-0.091	328.6050
14	10	4	14	9	5	E	0	108142.569	-0.046	104.2885
14	10	5	14	9	6	A	0	108168.343	0.013	104.2765
34	8	27	34	7	28	E	0	108211.794	-0.007	200.2811
34	8	27	34	7	28	A	0	108219.210	-0.009	200.2735
38	11	28	38	10	29	E	0	108230.810	-0.029	241.5348
13	10	4	13	9	5	E	0	108259.699	-0.027	101.3904
38	11	28	38	10	29	A	0	108260.339	-0.009	241.5288
13	10	3	13	9	4	E	0	108281.995	-0.048	101.4022
36	9	28	36	8	29	E	0	108299.061	-0.004	218.5757
32	7	26	32	6	27	E	1	108340.910	-0.126	311.3343
30	6	25	30	5	26	E	1	108381.172	-0.099	295.0928
33	11	23	33	10	24	E	0	108386.778	-0.017	203.3206
12	10	2	12	9	3	E	0	108393.367	-0.038	98.7252
30	6	25	30	5	26	E	0	108408.824	0.014	166.7636
32	7	26	32	6	27	E	0	108409.172	0.008	183.0070
32	7	26	32	6	27	A	0	108417.495	0.009	182.9989
30	6	25	30	5	26	A	0	108419.193	0.019	166.7548
12	10	2	12	9	3	A	0	108419.193	0.026	98.7132
12	10	3	12	9	4	A	0	108419.193	0.026	98.7132
31	11	20	31	10	21	A	0	108441.751	0.003	189.6117
23	1	22	23	0	23	E	0	108451.215	0.019	116.3734
23	2	22	23	1	23	E	0	108451.557	0.027	116.3734
33	11	23	33	10	24	A	0	108456.139	0.009	203.3148
23	1	22	23	0	23	A	0	108470.526	0.061	116.3618
23	2	22	23	1	23	A	0	108470.881	0.082	116.3618
31	11	20	31	10	21	E	0	108475.139	-0.062	189.6180
23	1	22	23	0	23	E	1	108559.422	-0.030	244.6989
23	2	22	23	1	23	E	1	108559.806	0.008	244.6989
59	15	44	59	14	45	A	0	108617.025	-0.111	481.2552
16	4	13	15	3	12	A	1	108633.875	0.023	223.4777
59	15	44	59	14	45	E	0	108652.482	-0.025	481.2591
16	4	13	15	3	12	E	0	108980.765	-0.033	94.7880
16	4	13	15	3	12	A	0	108997.493	-0.013	94.7768
18	4	15	17	4	14	A	1	109265.989	0.001	230.5648
32	11	22	32	10	23	E	0	109348.847	0.003	196.3190
38	10	29	38	9	30	E	0	109351.033	-0.004	237.8873
18	4	15	17	4	14	E	0	109355.951	0.014	101.8894
18	4	15	17	4	14	A	0	109358.055	-0.001	101.8789
38	10	29	38	9	30	A	0	109363.977	-0.008	237.8808
32	11	22	32	10	23	A	0	109441.217	-0.002	196.3134
19	2	17	18	3	16	A	1	109685.819	0.002	231.9830
19	2	17	18	3	16	E	0	109762.673	-0.006	103.3014
19	2	17	18	3	16	A	0	109764.444	-0.010	103.2906
39	11	29	39	10	30	E	0	109816.837	-0.028	249.7840
19	3	17	18	3	16	A	1	109822.862	0.013	231.9830
39	11	29	39	10	30	A	0	109841.627	-0.006	249.7781
19	3	17	18	3	16	E	1	109895.756	-0.011	231.6353
19	3	17	18	3	16	E	0	109906.838	-0.002	103.3014
19	3	17	18	3	16	A	0	109909.024	-0.007	103.2906
19	2	17	18	2	16	A	1	109938.425	0.008	231.9746
25	2	23	25	1	24	A	1	109970.559	-0.003	257.5494
25	3	23	25	2	24	A	1	109973.184	0.001	257.5494
20	1	19	19	2	18	A	1	109978.238	0.006	232.9726
20	2	19	19	2	18	A	1	109980.941	0.015	232.9726

J'	K_a'	K_c'	J''	K_a''	K_c''	Sym.	$v_{t_{CH_3}}$	$\nu_{Obs.}$	$\nu_{Obs.} - \nu_{Calc.}$	E_{low}
20	1	19	19	1	18	A	1	109983.667	0.001	232.9724
20	2	19	19	1	18	A	1	109986.349	-0.012	232.9724
20	2	19	19	2	18	E	1	110024.669	-0.029	232.6139
19	2	17	18	2	16	A	0	110029.999	-0.017	103.2817
20	2	19	19	1	18	E	1	110030.598	-0.014	232.6137
20	1	19	19	2	18	E	0	110040.155	-0.002	104.2820
20	1	19	19	2	18	A	0	110041.481	-0.006	104.2708
20	2	19	19	2	18	E	0	110043.008	-0.014	104.2820
20	2	19	19	2	18	A	0	110044.349	-0.013	104.2708
20	1	19	19	1	18	E	0	110045.903	-0.012	104.2818
20	1	19	19	1	18	A	0	110047.245	-0.019	104.2707
20	2	19	19	1	18	E	0	110048.760	-0.021	104.2818
20	2	19	19	1	18	A	0	110050.121	-0.019	104.2707
19	3	17	18	2	16	A	1	110075.405	-0.045	231.9746
19	3	17	18	2	16	E	1	110165.620	-0.012	231.6263
19	3	17	18	2	16	E	0	110171.698	0.007	103.2925
19	3	17	18	2	16	A	0	110174.578	-0.014	103.2817
21	0	21	20	0	20	A	1	110180.832	0.012	233.5339
21	0	21	20	1	20	A	1	110180.832	0.055	233.5339
21	1	21	20	0	20	A	1	110180.832	-0.007	233.5339
21	1	21	20	1	20	A	1	110180.832	0.035	233.5339
21	0	21	20	0	20	E	1	110194.030	-0.065	233.1586
21	0	21	20	1	20	E	1	110194.030	-0.018	233.1586
21	1	21	20	0	20	E	1	110194.030	-0.087	233.1586
21	1	21	20	1	20	E	1	110194.030	-0.040	233.1586
21	0	21	20	0	20	E	0	110219.474	-0.034	104.8304
21	1	21	20	1	20	E	0	110219.474	-0.010	104.8304
21	0	21	20	0	20	A	0	110219.908	-0.010	104.8188
21	0	21	20	1	20	A	0	110219.908	0.035	104.8188
21	1	21	20	0	20	A	0	110219.908	-0.032	104.8188
21	1	21	20	1	20	A	0	110219.908	0.014	104.8188
37	12	25	37	11	26	E	0	110349.559	-0.049	237.3303
62	17	45	62	16	46	E	0	110351.561	-0.003	528.8330
37	12	25	37	11	26	A	0	110386.951	0.005	237.3245
31	11	21	31	10	22	E	0	110388.658	-0.021	189.5391
35	7	28	35	6	29	A	0	110399.981	-0.014	207.3455
35	7	28	35	6	29	E	0	110402.549	0.003	207.3529
62	17	45	62	16	46	A	0	110415.748	-0.035	528.8283
30	11	19	30	10	20	A	0	110426.089	0.019	183.0208
25	2	23	25	1	24	E	0	110465.814	0.009	128.8718
25	3	23	25	2	24	E	0	110468.633	0.015	128.8718
25	2	23	25	1	24	A	0	110483.552	0.056	128.8609
25	3	23	25	2	24	A	0	110486.384	0.064	128.8609
31	11	21	31	10	22	A	0	110511.856	-0.000	189.5338
25	2	23	25	1	24	E	1	110548.354	-0.029	257.1993
25	3	23	25	2	24	E	1	110551.295	-0.011	257.1993
57	16	41	57	15	42	E	0	110766.240	-0.001	458.9116
57	16	41	57	15	42	A	0	110844.395	0.004	458.9067
18	3	15	17	3	14	A	1	111014.089	0.062	230.4067
17	4	14	16	3	13	A	1	111019.308	0.018	226.8616
17	5	12	16	5	11	A	0	111121.995	-0.002	100.5433
17	5	12	16	5	11	E	0	111124.571	0.015	100.5538
18	3	15	17	3	14	E	0	111152.420	-0.024	101.7258
18	3	15	17	3	14	A	0	111157.282	-0.008	101.7149
18	3	15	17	3	14	E	1	111158.385	-0.009	230.0581
18	12	6	17	12	5	A	0	111174.868	0.003	126.1325
18	12	7	17	12	6	A	0	111174.868	0.003	126.1325
18	12	7	17	12	6	E	0	111175.524	0.014	126.1281
17	4	14	16	3	13	E	0	111321.203	-0.048	98.1762
17	4	14	16	3	13	A	0	111335.455	-0.013	98.1651
18	11	8	17	11	7	A	0	111341.298	0.027	121.7647
18	11	7	17	11	6	A	0	111341.298	0.026	121.7647
18	11	8	17	11	7	E	0	111342.116	0.015	121.7617
30	11	20	30	10	21	E	0	111447.903	0.008	182.9822
27	3	24	27	2	25	A	1	111547.252	0.130	271.1086
30	11	20	30	10	21	A	0	111597.760	0.003	182.9773
17	4	13	16	4	12	E	0	111678.236	-0.012	99.3633
17	4	13	16	4	12	A	0	111679.970	-0.002	99.3523
26	7	19	25	8	18	E	0	111732.854	0.038	147.0957
31	9	22	30	10	21	A	0	111849.464	-0.030	182.9773
18	9	10	17	9	9	A	0	111865.284	0.017	114.1986
18	9	10	17	9	9	E	0	111867.175	0.005	114.1989
42	14	28	41	15	27	A	0	111894.695	-0.019	288.5650
40	13	27	39	14	26	A	0	111933.837	-0.015	266.3477
31	9	22	30	10	21	E	0	111937.153	0.050	182.9822
27	3	24	27	2	25	E	0	112008.365	0.025	142.4538
29	11	18	29	10	19	A	0	112010.117	0.013	176.6680
27	3	24	27	2	25	A	0	112024.107	0.037	142.4436
27	4	24	27	3	25	E	0	112025.109	0.029	142.4538
8	6	3	7	5	3	E	0	112034.467	-0.024	77.7768
27	4	24	27	3	25	A	0	112040.916	0.041	142.4437
27	3	24	27	2	25	E	1	112060.070	-0.051	270.7826
8	6	2	7	5	2	E	0	112072.932	-0.024	77.7843
27	4	24	27	3	25	E	1	112077.513	-0.015	270.7826
8	6	2	7	5	3	A	0	112079.577	0.089	77.7705
29	11	18	29	10	19	E	0	112082.913	-0.066	176.6749
40	11	30	40	10	31	A	0	112097.144	-0.033	258.2163
10	5	6	9	4	5	A	0	112138.288	0.011	79.6143
61	24	38	60	25	35	A	0	112139.167	-0.025	564.2613
44	15	30	43	16	28	E	0	112188.297	-0.012	311.9801
52	15	37	52	14	38	E	0	112197.693	-0.014	394.7855
44	15	29	43	16	27	E	0	112206.657	0.032	311.9927
44	15	29	43	16	28	A	0	112213.880	-0.006	311.9883
53	13	40	53	12	41	A	0	112217.442	-0.110	399.7443
53	13	40	53	12	41	E	0	112262.693	-0.028	399.7485
52	15	37	52	14	38	A	0	112279.172	0.011	394.7804
18	8	10	17	8	9	E	0	112305.402	0.002	111.0228
18	8	11	17	8	10	E	0	112306.294	0.000	111.0121
18	8	10	17	8	9	A	0	112313.681	-0.021	111.0103
67	18	49	67	17	50	E	0	112407.372	-0.017	604.5127
18	5	14	17	5	13	E	0	112427.525	-0.019	103.8221
18	5	14	17	5	13	A	0	112428.102	0.000	103.8120
29	11	19	29	10	20	E	0	112490.091	-0.011	176.6487
33	6	27	33	5	28	E	1	112523.434	-0.125	317.8988
33	6	27	33	5	28	E	0	112626.677	0.013	189.5723
33	6	27	33	5	28	A	0	112631.786	0.013	189.5643
38	12	26	37	13	25	A	0	112645.582	-0.020	245.3368
29	11	19	29	10	20	A	0	112648.760	0.003	176.6445
46	16	31	45	17	28	A	0	112685.487	-0.031	336.6169
46	16	31	45	17	29	E	0	112757.409	-0.002	336.6076
46	16	30	45	17	28	E	0	112763.709	0.013	336.6198
19	3	16	18	4	15	E	1	112765.292	-0.076	233.8709
42	13	29	42	12	30	E	0	112775.743	-0.048	283.9968
19	3	16	18	4	15	A	0	112793.865	-0.022	105.5267
19	3	16	18	4	15	E	0	112795.990	-0.004	105.5372
42	13	29	42	12	30	A	0	112836.098	-0.003	283.9914

J'	K'_a	K'_c	J''	K''_a	K''_c	Sym.	$v_{t_{CH_3}}$	$\nu_{Obs.}$	$\nu_{Obs.} - \nu_{Calc.}$	E_{low}
18	7	12	17	7	11	A	0	112876.723	-0.002	108.2286
18	7	12	17	7	11	E	0	112910.446	0.004	108.2346
29	4	25	29	3	26	E	0	113002.852	0.025	157.1068
29	4	25	29	3	26	A	0	113016.110	0.027	157.0974
29	4	25	29	3	26	E	1	113016.928	-0.058	285.4359
10	5	5	9	4	6	A	0	113021.122	0.018	79.5886
1	1	1	0	0	0	A	1	11303.654	0.109	195.9825
37	9	29	37	8	30	E	0	113036.034	-0.009	226.1773
37	9	29	37	8	30	A	0	113043.172	-0.019	226.1705
18	7	11	17	7	10	E	0	113079.472	0.052	108.2462
29	5	25	29	4	26	E	0	113081.221	0.015	157.1069
29	5	25	29	4	26	A	0	113094.795	0.029	157.0975
29	5	25	29	4	26	E	1	113098.433	-0.070	285.4361
18	7	11	17	7	10	A	0	113110.384	0.015	108.2353
38	8	30	38	7	31	A	0	113190.194	-0.040	233.9177
38	8	30	38	7	31	E	0	113199.547	-0.015	233.9244
47	14	33	47	13	34	E	0	113221.071	-0.060	336.4783
35	8	28	35	7	29	E	1	113225.830	-0.095	335.6893
31	5	26	31	4	27	E	1	113271.229	-0.106	301.1462
18	6	13	17	6	12	A	0	113293.888	-0.012	105.8545
31	5	26	31	4	27	E	0	113305.759	0.031	172.8178
31	5	26	31	4	27	A	0	113315.688	0.004	172.8091
35	8	28	35	7	29	E	0	113330.005	-0.017	207.3659
35	8	28	35	7	29	A	0	113337.063	-0.026	207.3585
39	10	30	39	9	31	E	0	113352.221	-0.032	246.0030
28	11	17	28	10	18	E	0	113357.966	-0.068	170.5553
39	10	30	39	9	31	A	0	113362.162	-0.042	245.9967
24	1	23	24	0	24	E	0	113398.042	0.086	120.5633
24	2	23	24	1	24	E	0	113398.042	-0.076	120.5633
24	1	23	24	0	24	A	0	113418.271	0.129	120.5517
24	2	23	24	1	24	A	0	113418.271	-0.033	120.5517
28	11	18	28	10	19	E	0	113487.967	-0.023	170.5384
1	1	1	0	0	0	E	0	11355.741	-0.037	67.2623
33	7	27	33	6	28	E	1	113577.674	-0.122	317.9025
1	1	1	0	0	0	A	0	11358.701	-0.024	67.2503
31	6	26	31	5	27	E	1	113588.292	-0.097	301.1471
35	10	26	34	11	23	E	0	113608.308	-0.007	214.1885
31	6	26	31	5	27	E	0	113610.705	-0.004	172.8186
11	4	7	10	3	8	E	0	113617.352	-0.014	80.2867
31	6	26	31	5	27	A	0	113621.808	0.004	172.8099
11	4	7	10	3	8	A	0	113626.023	-0.011	80.2755
28	11	18	28	10	19	A	0	113632.884	-0.015	170.5353
33	7	27	33	6	28	E	0	113642.007	-0.009	189.5759
33	7	27	33	6	28	A	0	113650.716	-0.013	189.5678
18	4	15	17	3	14	A	1	114007.589	0.013	230.4067
36	12	24	36	11	25	E	0	114009.445	-0.077	229.3605
36	12	24	36	11	25	A	0	114024.573	-0.014	229.3550
50	12	38	50	11	39	E	0	114145.509	-0.042	362.2232
50	18	33	49	19	31	E	0	114148.376	-0.056	389.4770
65	26	39	64	27	38	A	0	114178.075	-0.118	635.5534
65	26	40	64	27	37	A	0	114178.075	-0.118	635.5534
18	4	15	17	3	14	E	0	114262.815	-0.047	101.7258
18	4	15	17	3	14	A	0	114274.398	-0.036	101.7149
18	4	15	17	3	14	E	1	114304.743	0.050	230.0581
27	11	16	27	10	17	A	0	114363.211	-0.003	164.6549
27	11	16	27	10	17	E	0	114396.283	-0.077	164.6639
27	11	17	27	10	18	E	0	114416.525	-0.039	164.6506
27	11	17	27	10	18	A	0	114532.880	-0.019	164.6488
36	11	25	35	12	24	A	0	114606.554	-0.053	225.5321
19	4	16	18	4	15	E	1	114694.898	-0.040	233.8709
19	4	16	18	4	15	E	0	114696.520	-0.037	105.5372
19	4	16	18	4	15	A	0	114699.032	0.042	105.5267
20	2	18	19	3	17	A	1	114883.871	-0.010	235.6463
26	2	24	26	1	25	A	1	114925.835	-0.024	262.2441
26	3	24	26	2	25	A	1	114927.169	-0.010	262.2441
52	19	34	51	20	32	E	0	114939.342	0.095	417.7181
20	3	18	19	3	17	A	1	114957.093	-0.011	235.6463
20	2	18	19	3	17	E	0	114965.561	-0.027	106.9675
20	2	18	19	3	17	A	0	114967.547	-0.022	106.9567
41	9	32	41	8	33	A	0	114991.886	-0.056	262.7008
41	11	31	41	10	32	E	0	115003.153	-0.008	266.8439
41	9	32	41	8	33	E	0	115009.418	0.008	262.7068
41	11	31	41	10	32	A	0	115019.053	-0.025	266.8383
20	2	18	19	2	17	A	1	115020.932	0.018	235.6418
20	3	18	19	3	17	E	1	115030.277	-0.045	235.3010
20	3	18	19	3	17	E	0	115042.870	-0.009	106.9675
20	3	18	19	3	17	A	0	115045.090	-0.007	106.9567
20	3	18	19	2	17	A	1	115094.150	0.014	235.6418
21	1	20	20	2	19	A	1	115106.707	0.011	236.6412
21	2	20	20	2	19	A	1	115108.013	-0.009	236.6412
21	1	20	20	1	19	A	1	115109.336	-0.054	236.6411
20	2	18	19	2	17	E	0	115109.764	0.015	106.9627
21	2	20	20	1	19	A	1	115110.728	0.012	236.6411
20	2	18	19	2	17	A	0	115112.136	-0.010	106.9519
21	1	20	20	2	19	E	1	115150.114	-0.024	236.2839
21	2	20	20	2	19	E	1	115151.577	-0.023	236.2839
21	1	20	20	2	19	E	0	115169.932	0.005	107.9526
21	1	20	20	2	19	A	0	115171.273	0.016	107.9415
21	2	20	20	2	19	E	0	115171.273	-0.069	107.9526
21	2	20	20	2	19	A	0	115172.727	0.049	107.9415
21	1	20	20	1	19	E	0	115172.727	-0.067	107.9525
21	1	20	20	1	19	A	0	115174.179	0.046	107.9414
21	2	20	20	1	19	E	0	115174.179	-0.029	107.9525
21	2	20	20	1	19	A	0	115175.547	-0.006	107.9414
20	3	18	19	2	17	E	0	115187.029	-0.011	106.9627
20	3	18	19	2	17	A	0	115189.670	-0.004	106.9519
26	11	16	26	10	17	E	0	115253.488	0.000	158.9848
20	4	16	19	5	15	A	0	115256.416	-0.005	111.5077
26	11	15	26	10	16	A	0	115259.549	0.031	158.9871
26	11	15	26	10	16	E	0	115265.875	-0.048	158.9971
20	4	16	19	5	15	E	0	115271.579	0.030	111.5177
22	0	22	21	0	21	A	1	115309.882	0.024	237.2091
22	0	22	21	1	21	A	1	115309.882	0.044	237.2091
22	1	22	21	0	21	A	1	115309.882	0.015	237.2091
22	1	22	21	1	21	A	1	115309.882	0.035	237.2091
22	0	22	21	0	21	E	1	115322.983	-0.038	236.8343
22	0	22	21	1	21	E	1	115322.983	-0.016	236.8343
22	1	22	21	0	21	E	1	115322.983	-0.048	236.8343
22	1	22	21	1	21	E	1	115322.983	-0.026	236.8343
26	11	16	26	10	17	A	0	115342.117	-0.000	158.9842
22	0	22	21	0	21	E	0	115349.753	-0.043	108.5069
22	0	22	21	1	21	E	0	115349.753	-0.022	108.5069
22	1	22	21	0	21	E	0	115349.753	-0.053	108.5069
22	1	22	21	1	21	E	0	115349.753	-0.032	108.5069
22	0	22	21	0	21	A	0	115350.207	0.004	108.4953
22	0	22	21	1	21	A	0	115350.207	0.025	108.4953

J'	K'_a	K'_c	J''	K''_a	K''_c	Sym.	$v_{t_{\mathrm{CH_3}}}$	$\nu_{\mathrm{Obs.}}$	$\nu_{\mathrm{Obs.}} - \nu_{\mathrm{Calc.}}$	E_{low}
22	1	22	21	0	21	A	0	115350.207	-0.006	108.4953
22	1	22	21	1	21	A	0	115350.207	0.016	108.4953
26	2	24	26	1	25	E	0	115444.929	0.023	133.5689
26	3	24	26	2	25	E	0	115446.364	0.035	133.5689
26	2	24	26	1	25	A	0	115463.553	0.067	133.5581
26	3	24	26	2	25	A	0	115464.977	0.061	133.5581
26	2	24	26	1	25	E	1	115532.700	-0.022	261.8955
26	3	24	26	2	25	E	1	115534.199	-0.005	261.8955
40	12	29	40	11	30	E	0	115614.902	-0.024	261.9606
40	12	29	40	11	30	A	0	115654.976	-0.012	261.9555
44	10	34	44	9	35	A	0	115719.698	-0.061	293.6856
44	10	34	44	9	35	E	0	115746.198	-0.006	293.6910
39	12	28	39	11	29	E	0	115761.703	-0.023	253.4471
19	3	16	18	3	15	A	1	115771.274	0.012	234.1097
19	3	16	18	3	15	E	0	115906.408	-0.003	105.4334
19	3	16	18	3	15	A	0	115911.026	-0.006	105.4227
19	3	16	18	3	15	E	1	115911.661	-0.006	233.7660
61	17	44	61	16	45	E	0	115937.813	0.001	515.0628
25	11	14	25	10	15	E	0	116010.775	-0.044	153.5522
61	17	44	61	16	45	A	0	116017.080	-0.018	515.0583
25	11	14	25	10	15	A	0	116022.335	0.045	153.5415
41	12	30	41	11	31	A	0	116025.978	-0.021	270.6749
25	11	15	25	10	16	A	0	116060.909	-0.004	153.5402
69	28	41	68	29	40	A	0	116264.395	0.003	711.6502
69	28	42	68	29	39	A	0	116264.395	0.003	711.6502
38	12	27	38	11	28	E	0	116326.078	-0.016	245.1450
38	12	27	38	11	28	A	0	116379.948	-0.001	245.1400
28	3	25	28	2	26	A	1	116563.440	0.131	276.3104
36	7	29	36	6	30	E	1	116565.295	-0.139	342.9354
28	4	25	28	3	26	A	1	116571.630	0.122	276.3104
24	11	14	24	10	15	E	0	116634.314	0.004	148.3149
56	21	36	55	22	34	E	0	116653.213	0.021	477.8108
24	11	13	24	10	14	E	0	116655.101	-0.041	148.3271
24	11	13	24	10	14	A	0	116676.501	0.059	148.3161
56	21	35	55	22	33	E	0	116684.433	0.020	477.8188
24	11	14	24	10	15	A	0	116693.732	-0.019	148.3155
37	11	27	36	12	24	E	0	116787.147	0.043	233.1634
35	12	23	35	11	24	A	0	116924.892	0.013	221.6441
35	12	23	35	11	24	E	0	116940.661	-0.058	221.6495
42	12	31	42	11	32	E	0	116977.367	-0.018	279.5995
42	12	31	42	11	32	A	0	117006.965	-0.021	279.5944
28	3	25	28	2	26	E	0	117048.256	0.030	147.6588
28	4	25	28	3	26	E	0	117057.074	0.031	147.6588
28	3	25	28	2	26	A	0	117064.911	0.059	147.6487
28	4	25	28	3	26	A	0	117073.759	0.052	147.6487
28	3	25	28	2	26	E	1	117105.408	-0.039	275.9867
28	4	25	28	3	26	E	1	117114.603	-0.040	275.9868
24	6	18	23	7	17	A	0	117135.436	-0.014	134.0836
23	11	13	23	10	14	E	0	117193.150	0.002	143.3080
37	12	26	37	11	27	E	0	117194.589	-0.011	237.0590
23	11	12	23	10	13	E	0	117213.542	-0.049	143.3203
23	11	12	23	10	13	A	0	117239.804	0.058	143.3092
23	11	13	23	10	14	A	0	117247.128	-0.031	143.3089
37	12	26	37	11	27	A	0	117260.139	-0.003	237.0542
19	13	7	18	13	6	A	0	117302.298	-0.007	134.5911
19	13	6	18	13	5	A	0	117302.298	-0.007	134.5911
19	13	7	18	13	6	E	0	117302.908	-0.000	134.5852
18	4	14	17	4	13	A	1	117329.521	0.026	231.7690
7	7	1	6	6	1	E	0	117335.077	-0.001	78.4299
7	7	0	6	6	0	E	0	117369.891	-0.023	78.4387
11	5	7	10	4	6	E	0	117377.605	-0.036	81.7342
7	7	0	6	6	1	A	0	117380.563	0.024	78.4251
18	4	14	17	4	13	E	1	117400.486	-0.003	231.4202
4	1	3	3	2	2	A	0	11744.432	-0.056	69.2386
11	5	7	10	4	6	A	0	117440.448	0.017	81.7233
18	4	14	17	4	13	E	0	117451.618	0.013	103.0885
19	12	7	18	12	6	A	0	117454.224	-0.010	129.8409
19	12	8	18	12	7	A	0	117454.224	-0.010	129.8409
18	4	14	17	4	13	A	0	117454.837	0.027	103.0775
4	1	3	3	2	2	E	0	11754.971	-0.018	69.2501
58	22	37	57	23	34	A	0	117545.522	0.012	509.6741
19	4	16	18	3	15	A	1	117593.453	0.037	234.1097
19	11	9	18	11	8	E	0	117651.657	0.047	125.4757
22	11	12	22	10	13	E	0	117677.154	0.007	138.5181
56	16	40	56	15	41	E	0	117680.369	-0.007	446.2810
22	11	11	22	10	12	E	0	117696.964	-0.042	138.5304
22	11	11	22	10	12	A	0	117725.503	0.099	138.5193
22	11	12	22	10	13	A	0	117728.388	-0.039	138.5192
41	13	28	41	12	29	E	0	117748.136	-0.049	274.8726
25	1	24	25	0	25	A	1	117771.998	-0.118	253.6209
25	2	24	25	1	25	A	1	117771.998	-0.190	253.6209
41	13	28	41	12	29	A	0	117795.511	-0.001	274.8677
19	4	16	18	3	15	E	0	117806.959	-0.014	105.4334
19	4	16	18	3	15	A	0	117816.129	-0.005	105.4227
40	10	31	40	9	32	E	0	117817.810	-0.004	254.2921
19	4	16	18	3	15	E	1	117841.276	0.039	233.7660
60	15	45	60	14	46	E	0	117877.286	-0.018	494.2399
38	9	30	38	8	31	E	0	118051.584	0.003	233.9495
38	9	30	38	8	31	A	0	118057.743	-0.010	233.9429
21	11	11	21	10	12	E	0	118094.985	0.008	133.9440
21	11	10	21	10	11	E	0	118114.252	-0.041	133.9564
30	4	26	30	3	27	E	0	118147.584	0.026	162.8211
9	6	4	8	5	4	E	0	118152.193	-0.006	79.4274
30	4	26	30	3	27	A	0	118161.840	0.043	162.8118
30	4	26	30	3	27	E	1	118168.257	-0.068	291.1495
9	6	4	8	5	3	A	0	118189.682	-0.027	79.4213
30	5	26	30	4	27	E	0	118190.561	0.026	162.8212
9	6	3	8	5	4	A	0	118201.877	0.072	79.4209
30	5	26	30	4	27	A	0	118204.990	0.042	162.8119
30	5	26	30	4	27	E	1	118213.086	-0.068	291.1496
34	6	28	34	5	29	E	1	118220.583	-0.112	324.6385
36	12	25	36	11	26	E	0	118256.922	-0.006	229.1930
19	9	11	18	9	10	A	0	118271.725	0.027	117.9300
19	9	10	18	9	9	E	0	118273.074	0.007	117.9421
19	9	10	18	9	9	A	0	118274.070	-0.032	117.9301
19	9	11	18	9	10	E	0	118274.604	-0.007	117.9304
19	5	15	18	5	14	E	0	118281.586	0.000	107.5722
19	5	15	18	5	14	A	0	118282.750	-0.000	107.5622
34	6	28	34	5	29	E	0	118305.778	0.016	196.3127
34	6	28	34	5	29	A	0	118312.791	0.020	196.3048
36	12	25	36	11	26	A	0	118341.312	0.003	229.1883
25	1	24	25	0	25	A	0	118364.438	0.119	124.9127
25	2	24	25	1	25	A	0	118364.438	0.041	124.9127
18	5	13	17	5	12	A	0	118422.218	-0.002	104.2499
18	5	13	17	5	12	E	0	118424.277	0.006	104.2605
20	11	10	20	10	11	E	0	118454.266	0.006	129.5845

J'	K'_a	K'_c	J''	K''_a	K''_c	Sym.	$v_{t_{CH_3}}$	$\nu_{Obs.}$	$\nu_{Obs.} - \nu_{Calc.}$	E_{low}
25	1	24	25	0	25	E	1	118462.477	0.058	253.2478
25	2	24	25	1	25	E	1	118462.477	-0.024	253.2478
20	11	9	20	10	10	E	0	118473.044	-0.059	129.5969
60	23	38	59	24	35	A	0	118485.846	-0.004	542.7276
60	23	38	59	24	36	E	0	118495.590	0.069	542.7153
20	11	10	20	10	11	A	0	118503.161	-0.186	129.5857
42	11	32	42	10	33	E	0	118552.995	-0.014	275.6450
42	11	32	42	10	33	A	0	118565.061	-0.031	275.6395
36	8	29	36	7	30	E	0	118575.595	0.008	214.6205
36	8	29	36	7	30	A	0	118582.668	-0.016	214.6133
32	5	27	32	4	28	E	1	118611.761	-0.093	307.3713
43	12	32	43	11	33	E	0	118631.343	-0.016	288.7130
32	5	27	32	4	28	E	0	118636.301	0.019	179.0435
29	8	21	28	9	20	A	0	118647.504	-0.466	167.1879
32	5	27	32	4	28	A	0	118647.504	0.019	179.0349
43	12	32	43	11	33	A	0	118655.885	-0.029	288.7080
29	8	21	28	9	20	E	0	118720.868	0.066	167.1944
34	10	24	33	11	23	E	0	118723.309	0.029	206.9360
19	11	9	19	10	10	E	0	118761.722	0.015	125.4387
19	8	12	18	8	11	A	0	118779.532	-0.011	114.7557
19	11	8	19	10	9	E	0	118780.043	-0.107	125.4511
32	6	27	32	5	28	E	1	118793.437	-0.082	307.3717
19	8	12	18	8	11	E	0	118799.007	-0.004	114.7582
19	8	11	18	8	10	E	0	118802.772	-0.005	114.7689
19	8	11	18	8	10	A	0	118819.825	-0.010	114.7567
32	6	27	32	5	28	A	0	118822.417	0.033	179.0354
20	3	17	19	4	16	A	0	118831.911	0.010	109.3526
20	3	17	19	4	16	E	0	118832.146	-0.014	109.3630
34	7	28	34	6	29	E	1	118851.712	-0.090	324.6406
22	5	17	21	6	16	A	0	118894.965	-0.008	122.2331
34	7	28	34	6	29	E	0	118911.589	0.014	196.3146
34	7	28	34	6	29	A	0	118920.877	0.014	196.3067
22	5	17	21	6	16	E	0	118925.668	0.039	122.2425
41	13	29	40	14	26	E	0	119004.190	-0.024	274.7772
18	11	8	18	10	9	E	0	119023.307	0.023	121.5055
18	11	7	18	10	8	E	0	119041.339	-0.049	121.5180
18	11	8	18	10	9	A	0	119071.531	-0.001	121.5068
41	13	29	40	14	26	A	0	119120.584	-0.015	274.7714
34	12	22	34	11	23	A	0	119233.942	0.006	214.1831
51	15	36	51	14	37	E	0	119237.838	-0.020	383.3181
17	11	7	17	10	8	E	0	119244.336	0.008	117.7842
17	11	6	17	10	7	E	0	119262.114	-0.033	117.7967
34	12	22	34	11	23	E	0	119282.227	-0.077	214.1885
17	11	7	17	10	8	A	0	119292.322	0.015	117.7855
51	15	36	51	14	37	A	0	119318.333	0.017	383.3135
19	7	13	18	7	12	A	0	119416.595	-0.004	111.9938
35	12	24	35	11	25	E	0	119417.253	0.004	221.5495
11	5	6	10	4	7	A	0	119427.891	0.022	81.6666
16	11	6	16	10	7	E	0	119429.655	0.011	114.2738
19	7	13	18	7	12	E	0	119438.509	0.011	112.0009
16	11	5	16	10	6	E	0	119447.192	-0.034	114.2864
46	14	32	46	13	33	E	0	119462.434	-0.035	326.1845
16	11	6	16	10	7	A	0	119477.428	0.020	114.2752
35	12	24	35	11	25	A	0	119529.402	0.011	221.5450
4	1	3	4	0	4	A	1	11953.580	-0.007	197.9607
46	14	32	46	13	33	A	0	119530.149	0.009	326.1798
15	11	5	15	10	6	E	0	119583.600	0.012	110.9737
15	11	4	15	10	5	E	0	119600.932	-0.041	110.9863
43	14	30	42	15	27	A	0	119619.223	-0.004	297.3956
15	11	5	15	10	6	A	0	119631.200	0.026	110.9751
15	11	4	15	10	5	A	0	119631.200	0.027	110.9751
19	6	14	18	6	13	A	0	119669.079	-0.006	109.6336
19	6	14	18	6	13	E	0	119671.083	0.004	109.6429
14	11	4	14	10	5	E	0	119710.134	0.019	107.8832
14	11	3	14	10	4	E	0	119727.293	-0.045	107.8958
14	11	4	14	10	5	A	0	119757.583	0.029	107.8846
14	11	3	14	10	4	A	0	119757.583	0.029	107.8846
13	11	3	13	10	4	E	0	119812.855	0.023	105.0016
13	11	2	13	10	3	E	0	119829.882	-0.041	105.0141
43	14	30	42	15	28	E	0	119830.902	-0.053	297.3881
19	7	12	18	7	11	E	0	119851.464	-0.002	112.0182
19	7	12	18	7	11	A	0	119869.996	-0.000	112.0083
27	2	25	27	1	26	A	1	119876.395	0.000	267.1099
27	3	25	27	2	26	A	1	119877.036	-0.020	267.1099
12	11	2	12	10	3	E	0	119895.040	0.013	102.3282
12	11	1	12	10	2	E	0	119911.959	-0.054	102.3408
12	11	2	12	10	3	A	0	119942.278	0.029	102.3296
12	11	1	12	10	2	A	0	119942.278	0.029	102.3296
20	4	17	19	4	16	E	1	119953.708	-0.026	237.6968
20	4	17	19	4	16	E	0	119959.327	-0.008	109.3630
20	4	17	19	4	16	A	0	119962.004	-0.006	109.3526
11	11	0	11	10	1	E	0	119976.549	-0.053	99.8752
21	2	19	20	3	18	A	1	120045.827	0.007	239.4809
4	1	3	4	0	4	E	0	12008.040	-0.018	69.2417
21	3	19	20	3	18	A	1	120084.447	0.011	239.4809
4	1	3	4	0	4	A	0	12009.952	0.024	69.2298
21	2	19	20	3	18	E	1	120115.597	-0.028	239.1380
39	8	31	39	7	32	A	0	120117.700	-0.021	241.8701
21	2	19	20	2	18	A	1	120119.059	0.017	239.4784
39	8	31	39	7	32	E	0	120122.874	0.011	241.8766
21	2	19	20	3	18	E	0	120130.795	-0.006	110.8049
21	2	19	20	3	18	A	0	120132.890	-0.006	110.7942
21	3	19	20	2	18	A	1	120157.662	0.004	239.4784
21	3	19	20	3	18	E	0	120171.685	-0.013	110.8049
21	3	19	20	3	18	A	0	120173.921	-0.007	110.7942
21	2	19	20	2	18	E	0	120208.081	-0.009	110.8023
21	2	19	20	2	18	A	0	120210.411	-0.013	110.7916
45	15	31	44	16	29	E	0	120219.472	-0.012	321.2193
22	1	21	21	2	20	A	1	120234.543	0.015	240.4808
22	2	21	21	2	20	A	1	120235.177	0.001	240.4808
22	1	21	21	1	20	A	1	120235.880	0.026	240.4807
22	2	21	21	1	20	A	1	120236.481	-0.021	240.4807
21	3	19	20	2	18	E	0	120248.978	-0.010	110.8023
21	3	19	20	2	18	A	0	120251.444	-0.012	110.7916
22	1	21	21	2	20	E	1	120277.826	-0.010	240.1249
22	2	21	21	2	20	E	1	120278.514	-0.040	240.1249
22	1	21	21	1	20	E	1	120279.282	-0.015	240.1249
22	2	21	21	1	20	E	1	120279.991	-0.024	240.1249
57	14	43	57	13	44	E	0	120295.895	-0.032	451.6683
22	1	21	21	2	20	E	0	120299.013	-0.011	111.7943
22	2	21	21	2	20	E	0	120299.711	-0.007	111.7943
22	1	21	21	2	20	A	0	120300.359	0.008	111.7833
22	1	21	21	1	20	E	0	120300.359	-0.080	111.7943
22	2	21	21	2	20	A	0	120301.095	0.048	111.7833
22	1	21	21	1	20	A	0	120301.747	-0.024	111.7832
22	2	21	21	1	20	A	0	120302.450	-0.017	111.7832

J'	K'_a	K'_c	J''	K''_a	K''_c	Sym.	$v_{t_{CH_3}}$	$\nu_{Obs.}$	$\nu_{Obs.} - \nu_{Calc.}$	E_{low}
27	2	25	27	1	26	E	0	120419.125	0.036	138.4370
27	3	25	27	2	26	E	0	120419.843	0.038	138.4370
64	25	40	63	26	38	E	0	120428.751	-0.111	612.4290
64	25	40	63	26	37	A	0	120430.695	-0.007	612.4407
23	0	23	22	0	22	A	1	120438.881	0.020	241.0555
23	0	23	22	1	22	A	1	120438.881	0.029	241.0555
23	1	23	22	0	22	A	1	120438.881	0.016	241.0555
23	1	23	22	1	22	A	1	120438.881	0.025	241.0555
23	0	23	22	0	22	E	1	120451.881	-0.032	240.6811
23	1	23	22	1	22	E	1	120451.881	-0.027	240.6811
23	0	23	22	0	22	E	0	120480.006	-0.044	112.3546
23	0	23	22	1	22	E	0	120480.006	-0.034	112.3546
23	1	23	22	0	22	E	0	120480.006	-0.049	112.3546
23	1	23	22	1	22	E	0	120480.006	-0.039	112.3546
23	0	23	22	0	22	A	0	120480.467	0.013	112.3430
23	0	23	22	1	22	A	0	120480.467	0.023	112.3430
23	1	23	22	0	22	A	0	120480.467	0.008	112.3430
23	1	23	22	1	22	A	0	120480.467	0.018	112.3430
27	2	25	27	1	26	E	1	120512.005	-0.003	266.7627
27	3	25	27	2	26	E	1	120512.732	-0.023	266.7627
70	19	51	70	18	52	E	0	120574.353	-0.018	654.1966
47	16	32	46	17	29	A	0	120575.998	-0.046	346.2659
20	3	17	19	3	16	A	1	120602.600	0.019	237.9714
34	12	23	34	11	24	E	0	120606.684	0.049	214.1303
70	19	51	70	18	52	A	0	120635.468	-0.066	654.1926
47	16	32	46	17	30	E	0	120668.444	0.003	346.2567
20	3	17	19	3	16	E	0	120732.704	-0.019	109.2996
20	3	17	19	3	16	A	0	120736.995	-0.008	109.2891
34	12	23	34	11	24	A	0	120748.661	0.007	214.1262
41	13	28	40	14	27	A	0	120777.478	0.013	274.7682
4	1	3	4	0	4	E	1	12083.049	-0.039	197.5775
44	12	33	44	11	34	E	0	120976.405	-0.020	298.0148
65	18	47	65	17	48	E	0	121015.308	-0.006	574.9724
33	12	21	33	11	22	A	0	121092.435	0.012	206.9641
65	18	47	65	17	48	A	0	121094.814	-0.037	574.9683
49	17	33	48	18	30	A	0	121155.510	-0.029	372.5097
33	12	21	33	11	22	E	0	121160.637	-0.069	206.9698
49	17	32	48	18	31	A	0	121190.284	0.005	372.5096
49	17	33	48	18	31	E	0	121217.225	0.005	372.4996
49	17	32	48	18	30	E	0	121227.401	0.059	372.5112
9	7	3	10	6	5	E	0	12124.255	-0.324	85.4332
29	3	26	29	2	27	A	1	121567.636	0.125	281.6832
29	4	26	29	3	27	A	1	121571.909	0.133	281.6832
9	7	2	10	6	4	E	0	12160.407	-0.461	85.4419
70	18	52	70	17	53	E	0	121622.361	0.087	650.1397
20	4	17	19	3	16	A	1	121679.328	0.023	237.9714
9	7	3	10	6	4	A	0	12169.303	-0.025	85.4284
9	7	2	10	6	5	A	0	12173.359	0.067	85.4282
33	12	22	33	11	23	E	0	121783.647	0.039	206.9360
40	13	27	40	12	28	E	0	121851.496	-0.034	266.0206
20	4	17	19	3	16	E	0	121859.889	-0.007	109.2996
20	4	17	19	3	16	A	0	121867.107	-0.004	109.2891
40	13	27	40	12	28	A	0	121879.796	0.004	266.0160
20	4	17	19	3	16	E	1	121883.325	0.022	237.6324
33	12	22	33	11	23	A	0	121943.377	0.003	206.9325
29	3	26	29	2	27	E	0	122075.748	0.035	153.0348
29	4	26	29	3	27	E	0	122080.342	0.024	153.0348
29	3	26	29	2	27	A	0	122093.276	0.061	153.0248
29	4	26	29	3	27	A	0	122097.892	0.053	153.0248
12	5	8	11	4	7	E	0	122106.820	-0.042	84.0766
29	3	26	29	2	27	E	1	122138.040	-0.037	281.3618
29	4	26	29	3	27	E	1	122142.851	-0.041	281.3619
12	5	8	11	4	7	A	0	122148.702	0.020	84.0657
39	12	27	38	13	26	A	0	122287.215	-0.026	253.3488
39	12	27	38	13	26	E	0	122454.779	-0.216	253.3476
54	13	41	54	12	42	A	0	122472.975	-0.111	411.2473
54	13	41	54	12	42	E	0	122519.219	-0.039	411.2510
19	6	13	18	6	12	A	0	122529.570	-0.000	109.7686
19	6	13	18	6	12	E	0	122532.121	0.019	109.7783
32	12	20	32	11	21	A	0	122614.226	0.008	199.9810
37	7	30	37	6	31	E	1	122619.808	-0.125	350.3622
19	4	15	18	4	14	A	1	122634.047	0.019	235.6827
42	9	33	42	8	34	A	0	122640.284	-0.039	271.3498
43	11	33	43	10	34	E	0	122656.189	-0.016	284.6216
41	10	32	41	9	33	A	0	122658.982	-0.021	262.7468
43	11	33	43	10	34	A	0	122665.000	-0.035	284.6163
32	12	20	32	11	21	E	0	122680.672	-0.053	199.9874
19	4	15	18	4	14	E	1	122759.914	-0.015	235.3363
37	7	30	37	6	31	E	0	122767.884	0.012	222.0404
37	7	30	37	6	31	A	0	122771.066	-0.004	222.0333
19	4	15	18	4	14	E	0	122782.364	-0.010	107.0063
19	4	15	18	4	14	A	0	122787.022	-0.014	106.9954
32	12	21	32	11	22	E	0	122920.301	0.002	199.9665
60	17	43	60	16	44	E	0	123018.434	-0.009	501.5358
32	12	21	32	11	22	A	0	123076.525	0.005	199.9640
39	9	31	39	8	32	E	1	123123.181	-0.057	370.2087
20	16	4	19	16	3	A	0	123226.564	-0.056	155.0564
20	16	5	19	16	4	A	0	123226.564	-0.056	155.0564
20	16	5	19	16	4	E	0	123226.976	0.008	155.0466
39	9	31	39	8	32	E	0	123261.983	0.004	241.8914
31	4	27	31	3	28	E	0	123263.576	0.024	168.7062
39	9	31	39	8	32	A	0	123267.668	-0.011	241.8850
31	4	27	31	3	28	A	0	123278.758	0.043	168.6969
31	5	27	31	4	28	E	0	123286.866	-0.001	168.7062
31	4	27	31	3	28	E	1	123290.168	-0.059	297.0337
31	5	27	31	4	28	A	0	123302.171	0.041	168.6970
26	1	25	26	0	26	A	0	123309.288	0.093	129.4449
26	2	25	26	1	26	A	0	123309.288	0.055	129.4449
31	5	27	31	4	28	E	1	123314.553	-0.065	297.0338
20	15	6	19	15	5	A	0	123319.393	-0.041	149.1539
20	15	5	19	15	4	A	0	123319.393	-0.041	149.1539
20	15	6	19	15	5	E	0	123319.857	-0.022	149.1453
26	1	25	26	0	26	E	1	123411.795	0.026	257.7789
26	2	25	26	1	26	E	1	123411.795	-0.013	257.7789
8	7	2	7	6	2	E	0	123482.312	-0.000	79.8679
8	7	1	7	6	1	E	0	123517.166	-0.017	79.8767
8	7	1	7	6	2	A	0	123527.792	-0.002	79.8631
20	13	8	19	13	7	A	0	123573.070	-0.020	138.5039
20	13	7	19	13	6	A	0	123573.070	-0.020	138.5039
20	13	8	19	13	7	E	0	123573.822	0.020	138.4980
21	4	17	20	5	16	A	0	123726.697	-0.010	115.6435
21	4	17	20	5	16	E	0	123737.209	0.015	115.6535
20	12	8	19	12	7	A	0	123750.712	-0.022	133.7588
20	12	9	19	12	8	A	0	123750.712	-0.021	133.7588
20	12	8	19	12	7	E	0	123751.135	0.063	133.7678
20	12	9	19	12	8	E	0	123751.643	0.007	133.7544

J'	K'_a	K'_c	J''	K''_a	K''_c	Sym.	$v_{t_{CH_3}}$	$\nu_{Obs.}$	$\nu_{Obs.} - \nu_{Calc.}$	E_{low}
35	6	29	35	5	30	E	1	123764.964	-0.092	331.5474
37	8	30	37	7	31	E	1	123795.060	-0.086	350.3667
35	6	29	35	5	30	E	0	123835.969	0.012	203.2222
35	6	29	35	5	30	A	0	123844.544	0.016	203.2145
21	4	17	20	5	16	A	1	123852.845	-0.016	244.3096
12	4	8	11	3	9	E	0	123876.936	0.010	82.5268
31	12	19	31	11	20	A	0	123883.644	0.010	193.2289
33	5	28	33	4	29	E	1	123884.653	-0.087	313.7664
37	8	30	37	7	31	E	0	123894.222	0.012	222.0446
33	5	28	33	4	29	E	0	123900.921	0.019	185.4395
37	8	30	37	7	31	A	0	123901.633	0.005	222.0376
33	5	28	33	4	29	A	0	123913.221	0.015	185.4310
31	12	19	31	11	20	E	0	123928.353	-0.073	193.2363
20	11	10	19	11	9	A	0	123980.810	-0.019	129.4030
20	11	10	19	11	9	E	0	123981.980	-0.009	129.4001
33	6	28	33	5	29	E	1	123987.402	-0.080	313.7667
20	5	16	19	5	15	E	0	123988.260	-0.002	111.5177
20	5	16	19	5	15	A	0	123989.997	-0.005	111.5077
31	12	20	31	11	21	E	0	123993.586	0.048	193.2213
33	6	28	33	5	29	A	0	124011.875	0.031	185.4312
45	12	34	45	11	35	A	0	124014.091	-0.052	307.4948
51	12	39	51	11	40	A	0	124042.011	-0.097	372.9957
51	12	39	51	11	40	E	0	124081.194	-0.031	372.9997
31	12	20	31	11	21	A	0	124126.591	0.002	193.2200
35	7	29	35	6	30	E	1	124136.652	-0.081	331.5485
57	21	37	56	22	35	E	0	124159.566	0.038	489.5152
44	13	32	44	12	33	E	0	124167.952	-0.023	302.0501
45	10	35	45	9	36	A	0	124183.578	-0.061	303.0366
35	7	29	35	6	30	E	0	124191.606	0.004	203.2233
35	7	29	35	6	30	A	0	124201.602	0.018	203.2156
45	10	35	45	9	36	E	0	124204.473	-0.011	303.0417
44	13	32	44	12	33	A	0	124208.049	-0.021	302.0459
10	6	5	9	5	5	E	0	124242.020	-0.014	81.2889
10	6	5	9	5	4	A	0	124263.192	-0.004	81.2833
10	6	4	9	5	4	E	0	124277.732	-0.006	81.2964
20	10	11	19	10	10	E	0	124289.453	0.017	125.4387
10	6	4	9	5	5	A	0	124305.368	0.028	81.2820
43	13	31	43	12	32	E	0	124315.769	-0.021	292.6701
43	13	31	43	12	32	A	0	124361.630	-0.017	292.6659
21	3	18	20	4	17	A	1	124437.924	0.007	242.0302
21	3	18	20	4	17	E	1	124490.606	-0.035	241.6980
21	3	18	20	4	17	E	0	124512.980	-0.004	113.3644
21	3	18	20	4	17	A	0	124513.997	-0.004	113.3541
45	13	33	45	12	34	E	0	124572.399	-0.017	311.6357
45	13	33	45	12	34	A	0	124607.087	-0.024	311.6315
48	11	37	48	10	38	A	0	124654.358	-0.078	336.9219
48	11	37	48	10	38	E	0	124684.559	-0.015	336.9264
20	9	12	19	9	11	A	0	124711.635	0.006	121.8751
20	9	11	19	9	10	E	0	124714.822	0.002	121.8872
20	9	12	19	9	11	E	0	124716.578	0.006	121.8756
20	9	11	19	9	10	A	0	124717.565	0.001	121.8753
28	2	26	28	1	27	A	1	124822.869	-0.060	272.1468
28	3	26	28	2	27	A	1	124823.270	0.012	272.1468
42	13	30	42	12	31	E	0	124911.810	-0.018	283.5014
45	14	31	45	13	32	E	0	124915.457	-0.040	316.1714
30	12	18	30	11	19	A	0	124960.286	0.018	186.7043
42	13	30	42	12	31	A	0	124964.589	-0.014	283.4974
45	14	31	45	13	32	A	0	124971.606	0.004	316.1672
30	12	18	30	11	19	E	0	124976.500	-0.049	186.7128
30	12	19	30	11	20	E	0	124980.406	-0.001	186.6997
8	3	5	7	4	4	A	0	13215.224	-0.005	76.0645
6	2	4	5	3	3	A	0	13257.415	-0.051	72.0506
8	2	7	7	3	4	A	0	13418.087	0.002	74.7937
3	0	3	2	1	2	A	0	13446.143	0.001	68.0041
3	0	3	2	1	2	E	0	13448.462	0.000	68.0160
3	0	3	2	1	2	A	1	13484.113	0.005	196.7344
26	0	26	25	0	25	A	1	135825.665	0.093	253.6209
26	0	26	25	1	25	A	1	135825.665	0.094	253.6209
26	1	26	25	0	25	A	1	135825.665	0.093	253.6209
26	1	26	25	1	25	A	1	135825.665	0.094	253.6209
26	0	26	25	0	25	E	1	135838.273	-0.028	253.2478
26	1	26	25	1	25	E	1	135838.273	-0.028	253.2478
3	0	3	2	1	2	E	1	13596.449	0.002	196.3471
26	1	25	25	1	24	A	1	140745.011	-0.025	257.5494
26	1	25	25	2	24	A	1	140745.011	0.048	257.5494
26	2	25	25	1	24	A	1	140745.011	-0.060	257.5494
26	2	25	25	2	24	A	1	140745.011	0.013	257.5494
26	1	25	25	1	24	E	1	140787.598	-0.053	257.1993
26	1	25	25	2	24	E	1	140787.598	0.030	257.1993
26	2	25	25	1	24	E	1	140787.598	-0.092	257.1993
26	2	25	25	2	24	E	1	140787.598	-0.010	257.1993
27	0	27	26	0	26	A	1	140954.348	-0.006	258.1516
27	0	27	26	1	26	A	1	140954.348	-0.006	258.1516
27	1	27	26	0	26	A	1	140954.348	-0.007	258.1516
27	1	27	26	1	26	A	1	140954.348	-0.006	258.1516
27	0	27	26	0	26	E	1	140966.918	-0.060	257.7789
27	0	27	26	1	26	E	1	140966.918	-0.060	257.7789
27	1	27	26	0	26	E	1	140966.918	-0.060	257.7789
27	1	27	26	1	26	E	1	140966.918	-0.060	257.7789
5	2	3	5	1	4	A	1	14185.700	-0.029	199.4372
4	2	2	4	1	3	A	1	14200.428	-0.023	198.3594
5	2	3	5	1	4	E	0	14306.415	-0.007	70.7208
5	2	3	5	1	4	A	0	14311.508	-0.007	70.7090
4	2	2	4	1	3	E	0	14334.766	0.015	69.6422
4	2	2	4	1	3	A	0	14339.955	0.011	69.6304
41	7	34	41	6	35	E	1	145060.006	-0.132	381.7546
39	6	33	39	5	34	E	1	145106.724	-0.108	360.8802
47	15	33	47	14	34	E	0	145498.020	0.011	340.0750
25	3	22	24	4	21	A	1	145551.740	0.041	259.7470
47	15	33	47	14	34	A	0	145580.670	-0.019	340.0732
24	5	20	23	5	19	E	1	145587.347	-0.015	257.4912
54	14	41	54	13	42	E	0	145589.927	0.016	411.4748
24	5	20	23	5	19	E	0	145594.166	0.018	129.1591
25	4	22	24	4	21	A	1	145609.471	0.070	259.7470
25	3	22	24	4	21	E	1	145646.639	-0.058	259.4281
25	3	22	24	4	21	E	0	145663.310	-0.028	131.0960
25	3	22	24	4	21	A	0	145666.187	-0.004	131.0860
23	8	15	22	8	14	A	0	145671.000	0.019	131.9361
26	2	24	25	3	23	A	1	145697.591	-0.048	261.2177
26	3	24	25	3	23	A	1	145699.089	0.094	261.2177
26	2	24	25	2	23	A	1	145700.448	0.115	261.2176
26	3	24	25	2	23	A	1	145701.678	-0.010	261.2176
25	4	22	24	3	21	A	1	145716.203	0.023	259.7434
25	4	22	24	4	21	E	0	145724.870	0.082	131.0960
25	4	22	24	4	21	A	0	145727.880	0.017	131.0860
26	2	24	25	3	23	E	1	145768.955	-0.029	260.8869

J'	K'_a	K'_c	J''	K''_a	K''_c	Sym.	$v_{t_{CH_3}}$	$\nu_{Obs.}$	$\nu_{Obs.} - \nu_{Calc.}$	E_{low}
26	3	24	25	3	23	E	1	145770.414	-0.093	260.8869
26	2	24	25	2	23	E	1	145771.953	-0.037	260.8868
26	3	24	25	2	23	E	1	145773.474	-0.039	260.8868
25	3	22	24	3	21	E	0	145776.718	0.049	131.0922
25	3	22	24	3	21	A	0	145779.906	-0.003	131.0823
26	2	24	25	3	23	E	0	145790.664	-0.029	132.5567
26	2	24	25	3	23	A	0	145792.888	0.009	132.5464
26	3	24	25	2	23	E	0	145795.031	-0.015	132.5566
26	3	24	25	2	23	A	0	145797.242	-0.007	132.5463
25	4	22	24	3	21	E	1	145827.085	-0.028	259.4242
25	4	22	24	3	21	E	0	145838.114	-0.006	131.0922
25	4	22	24	3	21	A	0	145841.538	-0.043	131.0823
27	1	26	26	1	25	A	1	145872.739	0.044	262.2441
27	1	26	26	2	25	A	1	145872.739	0.079	262.2441
27	2	26	26	1	25	A	1	145872.739	0.027	262.2441
27	2	26	26	2	25	A	1	145872.739	0.062	262.2441
27	1	26	26	1	25	E	1	145915.105	-0.024	261.8955
27	1	26	26	2	25	E	1	145915.105	0.015	261.8955
27	2	26	26	1	25	E	1	145915.105	-0.043	261.8955
27	2	26	26	2	25	E	1	145915.105	-0.004	261.8955
27	1	26	26	1	25	E	0	145943.397	0.018	133.5689
27	2	26	26	2	25	E	0	145943.397	0.038	133.5689
27	1	26	26	1	25	A	0	145944.631	-0.050	133.5581
27	1	26	26	2	25	A	0	145944.631	-0.012	133.5581
27	2	26	26	1	25	A	0	145944.631	-0.068	133.5581
27	2	26	26	2	25	A	0	145944.631	-0.030	133.5581
48	10	38	48	9	39	A	0	146050.996	-0.059	332.0501
48	10	38	48	9	39	E	0	146057.606	0.028	332.0544
28	0	28	27	0	27	A	1	146083.095	0.028	262.8533
28	0	28	27	1	27	A	1	146083.095	0.028	262.8533
28	1	28	27	0	27	A	1	146083.095	0.028	262.8533
28	1	28	27	1	27	A	1	146083.095	0.028	262.8533
28	0	28	27	0	27	E	1	146095.542	-0.045	262.4811
28	1	28	27	1	27	E	1	146095.542	-0.045	262.4811
28	0	28	27	0	27	A	0	146130.714	-0.202	134.1482
28	0	28	27	1	27	A	0	146130.714	-0.201	134.1482
28	1	28	27	0	27	A	0	146130.714	-0.202	134.1482
28	1	28	27	1	27	A	0	146130.714	-0.201	134.1482
28	0	28	27	0	27	E	0	146130.714	0.187	134.1597
28	1	28	27	1	27	E	0	146130.714	0.187	134.1597
56	19	37	55	20	35	E	0	146134.997	-0.195	462.5798
24	4	20	23	4	19	E	0	146512.437	0.118	129.0821
24	4	20	23	4	19	A	0	146517.908	0.034	129.0722
24	4	20	23	4	19	E	1	146519.817	0.069	257.4125
48	15	33	47	16	32	E	0	146531.302	-0.235	350.2818
35	14	22	35	13	23	E	0	147039.005	0.008	229.9816
35	14	21	35	13	22	E	0	147045.703	-0.067	229.9946
46	15	32	46	14	33	E	0	147048.625	-0.018	330.0594
45	15	30	45	14	31	A	0	147049.710	0.047	320.3358
35	14	21	35	13	22	A	0	147071.845	-0.018	229.9870
35	14	22	35	13	23	A	0	147103.448	-0.045	229.9858
56	15	42	56	14	43	E	0	147130.527	-0.003	439.9373
14	6	9	13	5	8	A	0	147165.350	0.026	90.9009
34	3	31	34	2	32	E	1	147175.863	0.051	310.8015
34	4	31	34	3	32	E	1	147175.863	-0.119	310.8015
10	8	3	9	7	3	E	0	147208.450	-0.012	85.8376
10	8	2	9	7	3	A	0	147255.285	0.034	85.8343
31	1	30	31	0	31	A	1	147273.857	-0.298	283.3710
31	2	30	31	1	31	A	1	147273.857	-0.299	283.3710
24	5	20	23	4	19	A	1	147669.862	0.081	257.7303
34	14	21	34	13	22	E	0	147902.950	-0.101	222.6398
24	5	20	23	4	19	E	0	147903.781	0.077	129.0821
34	14	20	34	13	21	E	0	147909.977	-0.046	222.6529
24	5	20	23	4	19	A	0	147913.306	0.022	129.0722
34	14	20	34	13	21	A	0	147943.766	0.066	222.6450
12	7	6	11	6	6	E	0	147945.076	0.084	87.7090
34	14	21	34	13	22	A	0	147958.858	-0.121	222.6444
12	7	5	11	6	5	E	0	147980.112	-0.026	87.7177
12	7	6	11	6	5	A	0	147983.322	0.001	87.7043
31	1	30	31	0	31	E	0	147992.060	0.016	154.6840
31	2	30	31	1	31	E	0	147992.060	0.015	154.6840
12	7	5	11	6	6	A	0	147997.924	0.039	87.7039
5	2	3	5	1	4	E	1	14801.149	0.085	199.0581
48	11	38	48	10	39	E	1	148064.584	0.133	460.3632
3	2	1	3	1	2	A	1	14825.255	-0.034	197.4891
48	11	38	48	10	39	E	0	148280.565	-0.041	332.0651
48	11	38	48	10	39	A	0	148282.791	-0.051	332.0609
51	16	35	51	15	36	E	0	148578.894	0.030	387.2954
36	4	32	36	3	33	E	0	148586.170	-0.001	200.6935
36	5	32	36	4	33	E	0	148587.228	0.095	200.6935
36	4	32	36	3	33	A	0	148605.503	0.029	200.6848
36	5	32	36	4	33	A	0	148606.545	0.103	200.6848
45	15	31	45	14	32	E	0	148608.310	0.063	320.2723
25	5	20	24	6	19	A	0	148617.273	0.001	136.6366
51	16	35	51	15	36	A	0	148619.359	-0.012	387.2935
33	14	20	33	13	21	E	0	148679.314	0.029	215.5192
33	14	19	33	13	20	E	0	148685.579	-0.099	215.5323
23	5	18	22	5	17	E	0	149087.688	-0.040	126.2094
9	3	7	8	4	4	A	0	14909.549	-0.021	77.7314
23	5	18	22	5	17	A	0	149093.938	0.081	126.1990
24	12	12	23	12	11	A	0	149130.869	0.017	151.5368
24	12	13	23	12	12	E	0	149132.458	0.006	151.5325
56	16	41	56	15	42	E	0	149187.781	-0.017	444.8451
56	16	41	56	15	42	A	0	149226.109	-0.100	444.8433
55	16	40	55	15	41	E	0	149284.842	-0.027	432.8612
44	15	29	44	14	30	A	0	149303.670	0.162	310.7511
44	15	29	44	14	30	E	0	149355.112	-0.067	310.7536
32	14	19	32	13	20	E	0	149376.351	0.012	208.6185
32	14	18	32	13	19	E	0	149382.030	-0.013	208.6317
32	14	18	32	13	19	A	0	149421.627	0.073	208.6237
32	14	19	32	13	20	A	0	149424.738	-0.066	208.6236
46	10	37	46	9	38	E	0	149502.866	-0.003	307.5939
46	10	37	46	9	38	A	0	149507.481	-0.018	307.5890
33	2	31	33	1	32	A	1	149511.792	-0.118	299.8965
33	3	31	33	2	32	A	1	149511.792	-0.127	299.8965
38	5	33	38	4	34	E	0	149690.278	0.024	219.9763
24	6	19	23	6	18	E	0	149693.530	0.047	131.6526
38	5	33	38	4	34	E	1	149701.433	-0.123	348.2985
3	2	1	3	1	2	E	0	14971.188	0.006	68.7713
38	6	33	38	5	34	A	0	149711.830	0.049	219.9685
57	16	42	57	15	43	E	0	149725.815	-0.042	457.0331
24	6	19	23	6	18	E	1	149731.313	-0.921	259.9803
3	2	1	3	1	2	A	0	14974.537	-0.000	68.7594
57	16	42	57	15	43	A	0	149758.527	-0.066	457.0312
25	4	21	24	5	20	A	1	149883.737	0.021	262.6560
54	16	39	54	15	40	E	0	149919.284	0.056	421.0877

J'	K'_a	K'_c	J''	K''_a	K''_c	Sym.	$v_{t_{CH_3}}$	$\nu_{Obs.}$	$\nu_{Obs.} - \nu_{Calc.}$	E_{low}
25	4	21	24	5	20	E	1	149941.829	-0.018	262.3474
54	16	39	54	15	40	A	0	149968.548	-0.038	421.0861
25	4	21	24	5	20	E	0	149973.701	-0.012	134.0156
25	4	21	24	5	20	A	0	149974.808	0.023	134.0060
31	14	18	31	13	19	E	0	150001.911	0.010	201.9367
31	14	17	31	13	18	E	0	150006.911	-0.044	201.9500
55	14	42	55	13	43	E	0	150015.364	-0.026	423.0373
55	14	42	55	13	43	A	0	150019.785	-0.107	423.0347
44	8	36	44	7	37	E	0	150036.843	0.008	284.1370
44	8	36	44	7	37	A	0	150043.196	-0.134	284.1315
31	14	17	31	13	18	A	0	150047.483	0.119	201.9420
31	14	18	31	13	19	A	0	150048.680	-0.112	201.9419
24	10	14	23	10	13	E	0	150099.940	0.031	143.3203
24	10	15	23	10	14	E	0	150101.923	0.037	143.3080
24	10	14	23	10	13	A	0	150104.652	0.057	143.3092
44	15	30	44	14	31	E	0	150136.088	0.015	310.7143
44	9	36	44	8	37	E	1	150176.083	0.077	412.4505
33	2	31	33	1	32	E	0	150194.133	-0.003	171.2387
33	3	31	33	2	32	E	0	150194.133	-0.013	171.2387
33	2	31	33	1	32	A	0	150218.863	0.122	171.2281
33	3	31	33	2	32	A	0	150218.863	0.112	171.2281
9	2	8	8	3	5	A	0	15025.827	0.310	76.5054
44	15	30	44	14	31	A	0	150289.382	0.036	310.7139
44	9	36	44	8	37	E	0	150300.482	0.033	284.1379
44	9	36	44	8	37	A	0	150308.145	0.008	284.1324
9	2	8	8	3	5	E	0	15031.380	-0.031	76.5167
33	2	31	33	1	32	E	1	150315.341	0.005	299.5577
33	3	31	33	2	32	E	1	150315.341	-0.006	299.5577
40	6	34	40	5	35	E	1	150324.815	-0.049	368.6385
40	7	34	40	6	35	E	1	150346.625	-0.081	368.6386
40	6	34	40	5	35	E	0	150356.511	0.091	240.3181
40	6	34	40	5	35	A	0	150370.261	0.034	240.3111
40	7	34	40	6	35	E	0	150377.012	0.003	240.3182
40	7	34	40	6	35	A	0	150390.887	-0.031	240.3112
42	7	35	42	6	36	E	1	150432.332	-0.058	390.0260
57	15	43	57	14	44	E	0	150468.337	-0.090	452.0140
57	15	43	57	14	44	A	0	150479.727	-0.075	452.0118
6	2	4	6	1	5	A	1	15051.737	-0.021	200.7143
42	7	35	42	6	36	E	0	150512.947	0.008	261.7087
42	8	35	42	7	36	E	1	150514.896	-0.053	390.0262
42	7	35	42	6	36	A	0	150523.407	-0.028	261.7025
30	14	17	30	13	18	E	0	150562.798	0.047	195.4726
30	14	16	30	13	17	E	0	150567.212	-0.007	195.4860
42	8	35	42	7	36	E	0	150590.678	-0.156	261.7089
54	12	42	54	11	43	A	0	150593.515	-0.129	406.2240
42	8	35	42	7	36	A	0	150601.671	-0.029	261.7027
54	12	42	54	11	43	E	0	150614.540	0.022	406.2270
53	13	41	53	12	42	E	1	150628.810	0.383	523.3273
25	5	21	24	5	20	A	1	150659.730	-0.023	262.6560
26	3	23	25	4	22	A	1	150694.358	-0.125	264.6040
26	4	23	25	4	22	A	1	150725.345	0.038	264.6040
26	3	23	25	3	22	A	1	150752.209	0.024	264.6021
26	4	23	25	3	22	A	1	150782.984	-0.025	264.6021
26	3	23	25	4	22	E	1	150791.500	-0.040	264.2885
25	5	21	24	5	20	E	0	150792.939	0.043	134.0156
25	5	21	24	5	20	A	0	150796.487	-0.025	134.0060
26	3	23	25	4	22	E	0	150808.919	-0.027	135.9568
26	3	23	25	4	22	A	0	150811.903	0.012	135.9470
27	2	25	26	3	24	A	1	150821.890	0.015	266.0777
27	3	25	26	2	24	A	1	150823.997	0.088	266.0776
26	4	23	25	4	22	E	1	150825.619	-0.082	264.2885
24	9	16	23	9	15	A	0	150840.324	0.040	139.8159
26	4	23	25	4	22	E	0	150841.826	-0.057	135.9568
26	4	23	25	4	22	A	0	150844.910	-0.043	135.9470
26	3	23	25	3	22	E	1	150855.068	-0.023	264.2864
26	3	23	25	3	22	E	0	150870.391	-0.006	135.9548
26	3	23	25	3	22	A	0	150873.533	-0.030	135.9449
24	9	16	23	9	15	E	0	150878.027	0.066	139.8183
26	4	23	25	3	22	E	1	150889.234	-0.017	264.2864
27	2	25	26	3	24	E	1	150892.829	-0.064	265.7493
27	3	25	26	2	24	E	1	150895.048	-0.135	265.7493
26	4	23	25	3	22	E	0	150903.329	-0.005	135.9548
26	4	23	25	3	22	A	0	150906.630	0.004	135.9449
27	2	25	26	3	24	E	0	150916.057	-0.044	137.4198
27	3	25	26	2	24	A	0	150920.475	-0.007	137.4095
53	16	38	53	15	39	E	0	150977.720	-0.023	409.5304
28	1	27	27	1	26	A	1	151000.394	-0.011	267.1099
28	1	27	27	2	26	A	1	151000.394	0.005	267.1099
28	2	27	27	1	26	A	1	151000.394	-0.019	267.1099
28	2	27	27	2	26	A	1	151000.394	-0.002	267.1099
28	1	27	27	1	26	E	1	151042.633	-0.033	266.7627
28	1	27	27	2	26	E	1	151042.633	-0.014	266.7627
28	2	27	27	1	26	E	1	151042.633	-0.042	266.7627
28	2	27	27	2	26	E	1	151042.633	-0.023	266.7627
56	17	39	56	16	40	E	0	151062.675	-0.024	450.2064
29	14	16	29	13	17	E	0	151064.887	0.001	189.2253
29	14	15	29	13	16	E	0	151068.787	-0.047	189.2387
28	1	27	27	1	26	E	0	151072.338	0.008	138.4370
28	2	27	27	2	26	E	0	151072.338	0.018	138.4370
28	1	27	27	1	26	A	0	151073.592	-0.035	138.4262
28	1	27	27	2	26	A	0	151073.592	-0.017	138.4262
28	2	27	27	1	26	A	0	151073.592	-0.044	138.4262
28	2	27	27	2	26	A	0	151073.592	-0.026	138.4262
29	14	15	29	13	16	A	0	151109.948	0.129	189.2307
29	14	16	29	13	17	A	0	151109.948	-0.118	189.2307
25	4	21	24	4	20	A	1	151206.629	0.081	262.6119
29	0	29	28	0	28	A	1	151211.761	0.055	267.7261
29	0	29	28	1	28	A	1	151211.761	0.055	267.7261
29	1	29	28	0	28	A	1	151211.761	0.054	267.7261
29	1	29	28	1	28	A	1	151211.761	0.055	267.7261
29	0	29	28	0	28	E	1	151224.096	-0.026	267.3543
29	0	29	28	1	28	E	1	151224.096	-0.026	267.3543
29	1	29	28	0	28	E	1	151224.096	-0.026	267.3543
29	1	29	28	1	28	E	1	151224.096	-0.026	267.3543
29	0	29	28	0	28	A	0	151260.601	-0.208	139.0226
29	0	29	28	1	28	A	0	151260.601	-0.208	139.0226
29	1	29	28	0	28	A	0	151260.601	-0.208	139.0226
29	1	29	28	1	28	A	0	151260.601	-0.208	139.0226
29	0	29	28	0	28	E	0	151260.601	0.178	139.0341
29	1	29	28	1	28	E	0	151260.601	0.178	139.0341
35	3	32	35	2	33	A	1	151434.564	0.163	317.5098
35	4	32	35	3	33	A	1	151434.564	0.090	317.5098
28	14	15	28	13	16	E	0	151513.677	0.021	183.1939
28	14	14	28	13	15	E	0	151517.107	-0.037	183.2073
57	13	44	57	12	45	E	0	151522.874	-0.121	446.6141
28	14	15	28	13	16	A	0	151558.254	-0.039	183.1994

J'	K'_a	K'_c	J''	K''_a	K''_c	Sym.	$v_{t_{\mathrm{CH_3}}}$	$\nu_{\mathrm{Obs.}}$	$\nu_{\mathrm{Obs.}} - \nu_{\mathrm{Calc.}}$	E_{low}
6	2	4	6	1	5	E	0	15156.518	-0.009	71.9989
6	2	4	6	1	5	A	0	15160.899	0.047	71.9871
24	8	17	23	8	16	E	0	151657.735	0.000	136.7590
24	7	18	23	7	17	A	0	151691.823	-0.087	134.0836
24	7	18	23	7	17	E	0	151692.867	0.073	134.0922
4	2	2	4	1	3	E	1	15176.867	0.190	197.9806
43	15	29	43	14	30	A	0	151764.834	-0.030	301.3856
27	14	14	27	13	15	E	0	151913.773	-0.092	177.3774
27	14	13	27	13	14	E	0	151916.973	0.030	177.3909
15	6	10	14	5	9	E	0	151922.304	-0.052	93.8827
27	14	13	27	13	14	A	0	151958.031	0.011	177.3830
15	6	10	14	5	9	A	0	151980.682	0.002	93.8724
25	5	21	24	4	20	A	1	151982.664	0.079	262.6119
35	3	32	35	2	33	E	0	152076.924	0.106	188.8816
35	4	32	35	3	33	E	0	152076.924	0.026	188.8816
23	6	17	22	6	16	A	0	152082.621	0.030	127.6250
23	6	17	22	6	16	E	0	152083.752	-0.018	127.6351
35	3	32	35	2	33	A	0	152099.358	0.094	188.8720
35	4	32	35	3	33	A	0	152099.358	0.012	188.8720
35	3	32	35	2	33	E	1	152165.601	-0.075	317.2024
35	4	32	35	3	33	E	1	152165.601	-0.161	317.2024
25	5	21	24	4	20	E	0	152184.274	-0.007	133.9692
32	1	31	32	0	32	A	1	152188.002	-0.308	288.9281
32	2	31	32	1	32	A	1	152188.002	-0.308	288.9281
25	5	21	24	4	20	A	0	152191.943	0.020	133.9595
25	5	21	24	4	20	E	1	152209.248	-0.042	262.2999
26	14	13	26	13	14	E	0	152269.885	0.035	171.7752
26	14	12	26	13	13	E	0	152272.517	-0.052	171.7887
50	16	34	50	15	35	E	0	152280.969	-0.089	376.3311
50	16	34	50	15	35	A	0	152298.523	0.006	376.3295
26	14	13	26	13	14	A	0	152313.676	0.014	171.7808
52	16	37	52	15	38	E	0	152343.138	0.004	398.1942
52	16	37	52	15	38	A	0	152406.571	-0.045	398.1930
49	10	39	49	9	40	A	0	152462.026	-0.043	342.0490
49	10	39	49	9	40	E	0	152465.465	-0.014	342.0531
9	9	0	8	8	0	E	0	152526.214	-0.003	86.8551
9	9	1	8	8	0	A	0	152547.157	0.002	86.8424
9	9	0	8	8	1	A	0	152547.157	0.002	86.8424
25	14	12	25	13	13	E	0	152585.580	0.019	166.3864
25	14	11	25	13	12	E	0	152587.922	-0.040	166.3999
25	14	11	25	13	12	A	0	152629.034	-0.004	166.3921
47	14	34	46	15	31	E	0	152635.842	0.044	334.9836
24	8	16	23	8	15	E	0	152735.144	0.033	136.8034
24	8	16	23	8	15	A	0	152739.180	-0.128	136.7951
42	15	27	42	14	28	A	0	152837.815	0.003	292.2974
24	14	11	24	13	12	E	0	152864.639	0.036	161.2103
24	14	10	24	13	11	E	0	152866.691	-0.034	161.2239
42	15	27	42	14	28	E	0	152881.112	-0.065	292.3013
24	14	10	24	13	11	A	0	152907.804	0.012	161.2160
24	14	11	24	13	12	A	0	152907.804	0.011	161.2160
32	1	31	32	0	32	E	0	152930.712	0.071	160.2428
32	2	31	32	1	32	E	0	152930.712	0.071	160.2428
32	1	31	32	0	32	A	0	152958.135	0.048	160.2313
32	2	31	32	1	32	A	0	152958.135	0.048	160.2313
59	16	44	59	15	45	E	0	152962.372	-0.027	481.9951
59	16	44	59	15	45	A	0	152983.265	-0.076	481.9934
42	15	28	42	14	29	E	0	153007.207	0.029	292.2843
32	1	31	32	0	32	E	1	153086.420	0.005	288.5579
32	2	31	32	1	32	E	1	153086.420	0.004	288.5579
23	14	10	23	13	11	E	0	153110.315	0.030	156.2462
23	14	9	23	13	10	E	0	153112.110	-0.053	156.2598
42	15	28	42	14	29	A	0	153147.595	-0.055	292.2859
23	14	9	23	13	10	A	0	153153.233	0.012	156.2520
23	14	10	23	13	11	A	0	153153.233	0.011	156.2520
49	15	35	48	16	32	A	0	153271.790	-0.035	360.4326
22	14	9	22	13	10	E	0	153325.686	0.025	151.4935
22	14	8	22	13	9	E	0	153327.297	-0.027	151.5072
11	8	4	10	7	4	E	0	153343.101	-0.012	87.8967
22	14	8	22	13	9	A	0	153368.381	0.006	151.4993
22	14	9	22	13	10	A	0	153368.381	0.006	151.4993
11	8	3	10	7	3	E	0	153374.091	-0.055	87.9066
11	8	3	10	7	4	A	0	153389.952	-0.020	87.8934
21	14	8	21	13	9	E	0	153513.664	0.112	146.9516
21	14	7	21	13	8	E	0	153514.951	-0.078	146.9653
21	14	7	21	13	8	A	0	153556.081	0.012	146.9575
21	14	8	21	13	9	A	0	153556.081	0.012	146.9575
29	7	22	28	8	21	A	0	153650.852	-0.026	164.2143
20	14	7	20	13	8	E	0	153676.613	0.040	142.6200
20	14	6	20	13	7	E	0	153677.835	-0.056	142.6336
24	5	19	23	5	18	A	1	153700.390	0.053	259.8319
29	7	22	28	8	21	E	0	153702.362	0.044	164.2221
20	14	6	20	13	7	A	0	153718.954	0.034	142.6258
20	14	7	20	13	8	A	0	153718.954	0.034	142.6258
19	14	6	19	13	7	E	0	153817.143	-0.015	138.4980
19	14	5	19	13	6	E	0	153818.283	-0.054	138.5117
49	11	39	49	10	40	E	0	153857.984	0.011	342.0594
19	14	5	19	13	6	A	0	153859.309	-0.047	138.5039
19	14	6	19	13	7	A	0	153859.309	-0.047	138.5039
49	11	39	49	10	40	A	0	153860.372	0.022	342.0553
51	16	36	51	15	37	E	0	153903.992	0.028	387.0830
24	5	19	23	5	18	E	0	153910.574	-0.003	131.1825
24	5	19	23	5	18	A	0	153918.063	0.045	131.1723
18	14	5	18	13	6	E	0	153937.524	-0.045	134.5852
18	14	4	18	13	5	E	0	153938.574	-0.056	134.5989
51	16	36	51	15	37	A	0	153979.527	0.120	387.0820
13	7	7	12	6	7	E	0	153992.965	0.017	90.1973
13	7	6	12	6	7	A	0	154059.165	0.041	90.1918
17	14	3	17	13	4	A	0	154081.865	-0.007	130.8870
17	14	4	17	13	5	A	0	154081.865	-0.007	130.8870
57	19	39	56	20	36	A	0	154146.636	0.075	474.3488
16	14	2	16	13	3	A	0	154168.121	0.099	127.3912
16	14	3	16	13	4	A	0	154168.121	0.099	127.3912
27	6	21	26	7	20	A	0	154188.410	0.016	149.8761
27	6	21	26	7	20	E	0	154219.239	-0.016	149.8846
41	15	26	41	14	27	A	0	154261.233	-0.005	283.4200
41	15	26	41	14	27	E	0	154279.096	-0.077	283.4249
41	15	27	41	14	28	E	0	154315.246	0.057	283.4109
47	9	38	47	8	39	E	1	154335.998	0.031	445.3709
6	2	4	6	1	5	E	1	15438.553	0.015	200.3354
41	15	27	41	14	28	A	0	154428.408	-0.063	283.4139
34	2	32	34	1	33	A	1	154443.095	-0.023	305.9596
34	3	32	34	2	33	A	1	154443.095	-0.027	305.9596
58	15	44	58	14	45	E	0	154448.842	0.030	464.2667
58	15	44	58	14	45	A	0	154455.521	-0.055	464.2647
47	9	38	47	8	39	E	0	154534.418	-0.001	317.0661
47	9	38	47	8	39	A	0	154537.389	-0.072	317.0615

J'	K_a'	K_c'	J''	K_a''	K_c''	Sym.	$v_{t_{CH_3}}$	$\nu_{Obs.}$	$\nu_{Obs.} - \nu_{Calc.}$	E_{low}
39	5	34	39	4	35	E	0	154785.453	0.039	227.3956
39	6	34	39	5	35	E	0	154788.034	0.062	227.3956
39	5	34	39	4	35	E	1	154800.533	-0.075	355.7166
39	5	34	39	4	35	A	0	154803.022	0.142	227.3878
39	6	34	39	5	35	A	0	154805.479	0.029	227.3878
25	14	11	24	14	10	A	0	154895.616	-0.062	166.3165
25	14	12	24	14	11	A	0	154895.616	-0.062	166.3165
25	14	12	24	14	11	E	0	154896.973	0.121	166.3093
47	10	38	47	9	39	E	0	155020.152	-0.036	317.0679
47	10	38	47	9	39	A	0	155025.351	-0.045	317.0633
25	6	20	24	6	19	A	1	155130.890	0.060	265.2759
34	2	32	34	1	33	E	0	155148.324	0.029	177.3044
34	3	32	34	2	33	E	0	155148.324	0.024	177.3044
25	6	20	24	6	19	E	0	155258.082	0.028	136.6458
25	6	20	24	6	19	A	0	155260.817	-0.001	136.6366
34	2	32	34	1	33	E	1	155273.827	-0.037	305.6222
34	3	32	34	2	33	E	1	155273.827	-0.042	305.6222
49	16	33	49	15	34	A	0	155317.277	-0.033	365.6238
15	6	9	14	5	10	A	0	155323.342	0.055	93.7793
49	16	33	49	15	34	E	0	155328.528	-0.077	365.6252
15	6	9	14	5	10	E	0	155331.642	0.017	93.7892
26	4	22	25	5	21	A	1	155368.036	0.030	267.6815
45	8	37	45	7	38	E	1	155418.684	-0.084	421.4055
26	4	22	25	5	21	E	1	155452.722	0.011	267.3770
26	4	22	25	5	21	E	0	155478.201	0.010	139.0455
26	4	22	25	5	21	A	0	155480.366	-0.010	139.0361
40	15	25	40	14	26	E	0	155511.147	-0.006	274.7772
41	6	35	41	5	36	E	1	155514.550	-0.057	376.5672
41	7	35	41	6	36	E	1	155526.630	-0.006	376.5672
25	12	14	24	12	13	A	0	155530.770	0.035	156.5112
25	12	14	24	12	13	E	0	155532.619	0.002	156.5070
41	6	35	41	5	36	E	0	155541.563	0.015	248.2479
45	8	37	45	7	38	E	0	155547.849	-0.022	293.0942
41	7	35	41	6	36	E	0	155552.823	-0.032	248.2480
45	8	37	45	7	38	A	0	155555.499	-0.068	293.0889
41	6	35	41	5	36	A	0	155556.226	0.074	248.2411
41	7	35	41	6	36	A	0	155567.477	-0.040	248.2411
45	9	37	45	8	38	E	1	155582.096	-0.047	421.4061
40	15	26	40	14	27	A	0	155605.402	-0.031	274.7682
52	11	41	52	10	42	A	0	155641.668	-0.093	378.7271
52	11	41	52	10	42	E	0	155650.599	-0.108	378.7304
50	16	35	50	15	36	A	0	155656.551	-0.027	376.1988
45	9	37	45	8	38	A	0	155710.120	-0.070	293.0894
60	16	45	60	15	46	A	0	155715.780	-0.074	494.7551
43	7	36	43	6	37	E	1	155753.006	-0.078	398.4671
43	8	36	43	7	37	E	1	155799.957	0.014	398.4672
26	5	22	25	5	21	A	1	155814.636	0.033	267.6815
27	3	24	26	4	23	A	1	155823.949	0.028	269.6317
43	7	36	43	6	37	E	0	155827.167	0.007	270.1510
43	7	36	43	6	37	A	0	155838.581	0.011	270.1449
27	4	24	26	4	23	A	1	155840.240	0.025	269.6317
27	3	24	26	3	23	A	1	155854.740	-0.003	269.6306
27	4	24	26	3	23	A	1	155871.146	0.107	269.6306
43	8	36	43	7	37	A	0	155882.854	-0.016	270.1451
27	3	24	26	4	23	E	1	155921.812	-0.001	269.3195
27	3	24	26	4	23	E	0	155940.349	-0.022	140.9884
27	3	24	26	4	23	A	0	155943.353	-0.003	140.9786
28	2	26	27	3	25	A	1	155946.263	0.002	271.1086
28	3	26	27	2	25	A	1	155947.289	0.013	271.1086
26	5	22	25	5	21	E	0	155951.236	-0.026	139.0455
26	5	22	25	5	21	A	0	155954.957	-0.056	139.0361
27	3	24	26	3	23	E	1	155956.047	0.073	269.3183
27	4	24	26	4	23	E	0	155957.844	0.001	140.9884
27	4	24	26	4	23	A	0	155960.878	-0.020	140.9786
27	3	24	26	3	23	E	0	155973.199	-0.109	140.9873
27	4	24	26	3	23	E	1	155974.231	0.084	269.3183
27	3	24	26	3	23	A	0	155976.407	-0.011	140.9775
27	4	24	26	3	23	E	0	155990.828	0.047	140.9873
27	4	24	26	3	23	A	0	155993.996	0.035	140.9775
25	11	15	24	11	14	A	0	155999.156	0.055	152.2080
25	11	14	24	11	13	A	0	156000.461	-0.164	152.2080
25	11	14	24	11	13	E	0	156000.461	0.064	152.2183
25	11	15	24	11	14	E	0	156002.204	-0.025	152.2054
28	2	26	27	3	25	E	0	156041.663	0.018	142.4538
28	3	26	27	3	25	E	0	156041.663	-0.348	142.4538
28	2	26	27	2	25	E	0	156042.705	0.327	142.4538
28	3	26	27	2	25	E	0	156042.705	-0.038	142.4538
28	2	26	27	3	25	A	0	156043.837	0.025	142.4437
28	3	26	27	2	25	A	0	156044.890	-0.025	142.4436
16	6	11	15	5	10	A	0	156061.256	0.002	97.0846
29	1	28	28	1	27	A	1	156128.148	0.009	272.1468
29	1	28	28	2	27	A	1	156128.148	0.017	272.1468
29	2	28	28	1	27	A	1	156128.148	0.005	272.1468
29	2	28	28	2	27	A	1	156128.148	0.013	272.1468
26	4	22	25	4	21	A	1	156144.119	0.075	267.6556
29	1	28	28	1	27	E	1	156170.177	-0.056	271.8009
29	1	28	28	2	27	E	1	156170.177	-0.047	271.8009
29	2	28	28	1	27	E	1	156170.177	-0.060	271.8009
29	2	28	28	2	27	E	1	156170.177	-0.051	271.8009
29	1	28	28	1	27	E	0	156201.319	0.009	143.4763
29	2	28	28	2	27	E	0	156201.319	0.014	143.4763
29	1	28	28	1	27	A	0	156202.554	-0.048	143.4655
29	1	28	28	2	27	A	0	156202.554	-0.039	143.4655
29	2	28	28	1	27	A	0	156202.554	-0.052	143.4655
29	2	28	28	2	27	A	0	156202.554	-0.043	143.4655
26	4	22	25	4	21	E	1	156294.703	-0.006	267.3490
30	0	30	29	0	29	A	1	156340.297	0.030	272.7700
30	0	30	29	1	29	A	1	156340.297	0.030	272.7700
30	1	30	29	0	29	A	1	156340.297	0.030	272.7700
30	1	30	29	1	29	A	1	156340.297	0.030	272.7700
30	0	30	29	0	29	E	1	156352.546	-0.035	272.3986
30	1	30	29	1	29	E	1	156352.546	-0.035	272.3986
30	0	30	29	0	29	A	0	156390.427	-0.197	144.0681
30	0	30	29	1	29	A	0	156390.427	-0.196	144.0681
30	1	30	29	0	29	A	0	156390.427	-0.197	144.0681
30	1	30	29	1	29	A	0	156390.427	-0.197	144.0681
30	0	30	29	1	29	E	0	156390.427	0.186	144.0796
30	1	30	29	0	29	E	0	156390.427	0.186	144.0796
36	3	33	36	2	34	A	1	156394.468	0.110	324.0794
36	4	33	36	3	34	A	1	156394.468	0.074	324.0794
26	5	21	25	6	20	A	0	156404.124	-0.041	141.8155
26	5	21	25	6	20	E	0	156412.404	0.015	141.8247
26	5	21	25	6	20	A	1	156470.833	-0.043	270.4505
24	7	17	23	7	16	A	0	156596.347	-0.057	134.3822
24	7	17	23	7	16	E	0	156601.421	-0.055	134.3914
39	15	25	39	14	26	E	0	156611.882	-0.066	266.3427

J'	K_a'	K_c'	J''	K_a''	K_c''	Sym.	$v_{t_{\mathrm{CH_3}}}$	$\nu_{\mathrm{Obs.}}$	$\nu_{\mathrm{Obs.}} - \nu_{\mathrm{Calc.}}$	E_{low}
39	15	24	39	14	25	E	0	156612.930	0.009	266.3557
25	10	16	24	10	15	A	0	156631.956	0.018	148.3155
39	15	24	39	14	25	A	0	156636.123	0.017	266.3494
25	10	15	24	10	14	E	0	156644.625	-0.093	148.3271
25	10	16	24	10	15	E	0	156645.629	0.055	148.3149
25	10	15	24	10	14	A	0	156654.717	-0.059	148.3161
39	15	25	39	14	26	A	0	156681.246	-0.012	266.3477
26	5	22	25	4	21	E	0	156770.455	0.009	139.0182
26	5	22	25	4	21	A	0	156776.753	0.014	139.0087
26	5	22	25	4	21	E	1	156782.485	-0.003	267.3490
36	3	33	36	2	34	E	0	157058.533	0.058	195.4546
36	4	33	36	3	34	E	0	157058.533	0.018	195.4546
36	3	33	36	2	34	A	0	157081.781	0.072	195.4451
36	4	33	36	3	34	A	0	157081.781	0.032	195.4451
33	1	32	33	0	33	A	1	157101.435	-0.378	294.6562
33	2	32	33	1	33	A	1	157101.435	-0.378	294.6562
36	3	33	36	2	34	E	1	157151.116	-0.060	323.7742
36	4	33	36	3	34	E	1	157151.116	-0.103	323.7742
2	2	0	2	1	1	A	1	15718.657	-0.017	196.8320
60	17	44	60	16	45	E	0	157349.535	-0.007	499.9503
49	16	34	49	15	35	A	0	157361.718	-0.016	365.5452
59	17	43	59	16	44	E	0	157413.362	-0.051	487.0974
59	17	43	59	16	44	A	0	157456.150	-0.026	487.0964
25	9	17	24	9	16	A	0	157461.810	-0.049	144.8474
25	9	17	24	9	16	E	0	157498.286	-0.019	144.8511
38	15	24	38	14	25	E	0	157603.198	0.030	258.1456
38	15	23	38	14	24	E	0	157605.431	-0.029	258.1586
38	15	23	38	14	24	A	0	157638.748	0.055	258.1520
38	15	24	38	14	25	A	0	157661.234	-0.026	258.1512
65	19	46	65	18	47	E	0	157679.094	-0.071	579.0090
25	9	16	24	9	15	E	0	157680.589	-0.005	144.8650
25	9	16	24	9	15	A	0	157712.410	0.058	144.8557
65	19	46	65	18	47	A	0	157763.474	-0.069	579.0076
48	16	32	48	15	33	A	0	157825.802	-0.023	355.1681
48	16	32	48	15	33	E	0	157863.907	-0.130	355.1695
25	7	19	24	7	18	A	0	157865.031	0.007	139.1435
25	7	19	24	7	18	E	0	157865.031	0.041	139.1521
33	1	32	33	0	33	E	0	157868.648	0.059	165.9728
33	2	32	33	1	33	E	0	157868.648	0.058	165.9728
5	1	4	5	0	5	A	1	15787.548	-0.015	198.9106
60	18	42	60	17	43	E	0	157875.258	0.040	505.6392
33	1	32	33	0	33	A	0	157897.029	0.095	165.9613
33	2	32	33	1	33	A	0	157897.029	0.095	165.9613
52	12	41	52	11	42	A	0	157971.158	-0.265	378.7388
52	12	41	52	11	42	E	0	157971.158	0.115	378.7421
33	1	32	33	0	33	E	1	158029.374	-0.003	294.2864
33	2	32	33	1	33	E	1	158029.374	-0.003	294.2864
58	17	42	58	16	43	E	0	158043.244	-0.011	474.4544
58	17	42	58	16	43	A	0	158091.221	-0.039	474.4535
55	12	43	55	11	44	A	0	158132.164	-0.109	417.6140
55	12	43	55	11	44	E	0	158147.811	-0.029	417.6167
25	8	18	24	8	17	A	0	158223.779	-0.040	141.8103
25	8	18	24	8	17	E	0	158229.563	0.012	141.8177
25	5	20	24	5	19	A	1	158286.920	0.088	264.9588
5	1	4	5	0	5	E	0	15844.731	0.001	70.1923
5	1	4	5	0	5	A	0	15846.377	0.005	70.1804
37	15	23	37	14	24	E	0	158500.372	0.006	250.1713
37	15	22	37	14	23	E	0	158502.283	-0.042	250.1844
25	5	20	24	5	19	E	0	158506.661	0.003	136.3164
25	5	20	24	5	19	A	0	158514.618	-0.065	136.3064
25	5	20	24	5	19	E	1	158527.354	0.008	264.6412
37	15	22	37	14	23	A	0	158541.054	0.055	250.1776
37	15	23	37	14	24	A	0	158551.904	-0.077	250.1772
50	10	40	50	9	41	A	0	158598.585	-0.098	352.2141
50	10	40	50	9	41	E	0	158599.720	0.057	352.2180
38	4	34	38	3	35	E	0	158640.409	0.148	214.6846
38	5	34	38	4	35	E	0	158640.409	-0.108	214.6846
10	9	2	9	8	2	E	0	158647.846	-0.020	88.6923
38	4	34	38	3	35	A	0	158661.250	0.170	214.6761
38	5	34	38	4	35	A	0	158661.250	-0.088	214.6761
10	9	1	9	8	1	E	0	158674.357	-0.036	88.7033
24	6	18	23	6	17	E	0	158677.139	-0.120	132.7081
24	6	18	23	6	17	A	0	158678.191	0.071	132.6979
10	9	2	9	8	1	A	0	158695.325	0.017	88.6906
10	9	1	9	8	2	A	0	158695.325	0.017	88.6906
38	4	34	38	3	35	E	1	158697.464	0.060	343.0049
38	5	34	38	4	35	E	1	158697.464	-0.213	343.0049
2	2	0	2	1	1	A	0	15873.604	-0.009	68.1020
67	16	51	67	15	52	E	0	158738.651	0.070	595.1114
2	2	0	2	1	1	E	0	15882.238	0.036	68.1138
48	16	33	48	15	34	E	0	158895.881	0.058	355.1218
5	1	4	5	0	5	E	1	15894.624	-0.013	198.5279
59	15	45	59	14	46	E	0	158985.301	-0.018	476.6919
59	15	45	59	14	46	A	0	158988.091	-0.120	476.6901
48	16	33	48	15	34	A	0	159038.122	-0.005	355.1220
57	17	41	57	16	42	E	0	159126.696	-0.005	462.0274
61	16	46	61	15	47	E	0	159150.406	-0.014	507.6979
61	16	46	61	15	47	A	0	159160.212	-0.100	507.6965
57	17	41	57	16	42	A	0	159180.049	-0.127	462.0267
17	6	12	16	5	11	E	0	159186.479	-0.024	100.5538
17	6	12	16	5	11	A	0	159225.675	0.017	100.5433
50	11	40	50	10	41	E	1	159243.467	0.149	480.5169
36	15	22	36	14	23	E	0	159312.425	0.021	242.4186
36	15	21	36	14	22	E	0	159313.620	-0.059	242.4318
36	15	21	36	14	22	A	0	159355.223	0.096	242.4250
36	15	22	36	14	23	A	0	159360.273	-0.052	242.4248
35	2	33	35	1	34	A	1	159372.516	-0.107	312.1937
35	3	33	35	2	34	A	1	159372.516	-0.109	312.1937
50	11	40	50	10	41	E	0	159457.915	-0.027	352.2216
50	11	40	50	10	41	A	0	159460.637	-0.066	352.2178
12	8	5	11	7	5	E	0	159464.826	-0.003	90.1652
12	8	4	11	7	4	E	0	159495.977	-0.004	90.1751
12	8	4	11	7	5	A	0	159511.738	-0.160	90.1618
58	13	45	58	12	46	A	0	159805.901	-0.141	458.7033
58	13	45	58	12	46	E	0	159829.384	-0.012	458.7054
40	5	35	40	4	36	E	0	159867.031	0.005	234.9855
40	6	35	40	5	36	E	0	159868.397	0.017	234.9855
40	5	35	40	4	36	A	0	159885.397	0.170	234.9779
40	6	35	40	5	36	A	0	159886.713	0.125	234.9779
47	16	31	47	15	32	A	0	159945.891	-0.043	344.9557
14	7	8	13	6	7	A	0	159977.855	0.007	92.8968
47	16	31	47	15	32	E	0	159997.826	-0.106	344.9574
14	7	8	13	6	8	E	0	159999.002	0.020	92.8998
14	7	7	13	6	7	E	0	160015.874	-0.033	92.9090
25	8	17	24	8	16	A	0	160032.131	-0.061	141.8900
25	8	17	24	8	16	E	0	160033.199	0.056	141.8981

J'	K'_a	K'_c	J''	K''_a	K''_c	Sym.	$v_{t_{CH_3}}$	$\nu_{Obs.}$	$\nu_{Obs.} - \nu_{Calc.}$	E_{low}
54	17	37	54	16	38	E	0	160053.154	-0.061	426.2441
54	17	37	54	16	38	A	0	160083.359	-0.032	426.2433
35	15	20	35	14	21	A	0	160090.776	0.103	234.8927
35	2	33	35	1	34	E	0	160100.717	0.033	183.5412
35	3	33	35	2	34	E	0	160100.717	0.030	183.5412
35	2	33	35	1	34	A	0	160127.050	0.090	183.5308
35	3	33	35	2	34	A	0	160127.050	0.087	183.5308
57	14	44	57	13	45	A	0	160141.495	-0.198	446.6700
57	14	44	57	13	45	E	0	160142.435	0.043	446.6722
64	15	49	64	14	50	E	0	160214.399	-0.011	547.4612
48	9	39	48	8	40	E	0	160230.457	-0.008	326.7097
48	9	39	48	8	40	A	0	160234.948	-0.043	326.7053
48	10	39	48	9	40	E	1	160354.834	0.105	455.0143
47	16	32	47	15	33	E	0	160493.891	0.036	344.9283
48	10	39	48	9	40	E	0	160519.835	-0.028	326.7107
48	10	39	48	9	40	A	0	160525.801	0.065	326.7063
56	17	40	56	16	41	E	0	160545.425	0.006	449.8214
26	6	21	25	6	20	A	1	160557.036	-0.019	270.4505
56	17	40	56	16	41	A	0	160605.460	-0.032	449.8209
26	17	10	25	17	9	A	0	160609.493	-0.115	189.1076
26	17	9	25	17	8	A	0	160609.493	-0.115	189.1076
26	17	10	25	17	9	E	0	160610.323	0.010	189.0969
47	16	32	47	15	33	A	0	160647.135	-0.026	344.9292
42	6	36	42	5	37	E	1	160680.752	-0.098	384.6662
27	4	23	26	5	22	A	1	160691.996	0.018	272.8789
26	6	21	25	6	20	E	0	160694.736	-0.065	141.8247
26	6	21	25	6	20	A	0	160698.098	-0.011	141.8155
42	6	36	42	5	37	E	0	160703.825	0.056	256.3482
34	15	19	34	14	20	E	0	160712.113	0.012	227.5866
42	6	36	42	5	37	A	0	160719.091	-0.036	256.3415
42	7	36	42	6	37	A	0	160725.337	0.017	256.3415
34	15	19	34	14	20	A	0	160755.467	-0.045	227.5798
34	15	20	34	14	21	A	0	160756.406	-0.173	227.5798
26	16	10	25	16	9	A	0	160778.929	-0.095	182.8436
26	16	11	25	16	10	A	0	160778.929	-0.095	182.8436
26	16	11	25	16	10	E	0	160779.837	-0.062	182.8339
27	4	23	26	5	22	E	1	160793.602	-0.065	272.5787
27	4	23	26	5	22	E	0	160815.245	-0.003	144.2475
27	4	23	26	5	22	A	0	160818.097	-0.034	144.2382
46	8	38	46	7	39	E	1	160865.557	0.040	430.5307
28	3	25	27	4	24	A	1	160946.827	-0.004	274.8299
46	9	38	46	8	39	E	1	160960.158	0.011	430.5310
28	4	25	27	3	24	A	1	160971.680	0.018	274.8294
26	15	12	25	15	11	A	0	160983.902	-0.058	176.9681
26	15	11	25	15	10	A	0	160983.902	-0.058	176.9681
46	8	38	46	7	39	E	0	160986.209	0.011	302.2207
46	8	38	46	7	39	A	0	160994.917	-0.007	302.2156
44	7	37	44	6	38	E	1	161032.907	-0.052	407.0781
28	3	25	27	4	24	E	1	161044.833	-0.014	274.5211
28	4	25	27	4	24	E	1	161054.439	0.013	274.5211
44	8	37	44	7	38	E	1	161059.269	-0.040	407.0781
28	3	25	27	3	24	E	1	161063.063	0.043	274.5205
28	3	25	27	4	24	E	0	161064.772	-0.018	146.1906
28	3	25	27	4	24	A	0	161067.793	0.004	146.1809
28	4	25	27	3	24	E	1	161072.590	-0.009	274.5205
28	4	25	27	4	24	E	0	161073.958	-0.015	146.1906
46	9	38	46	8	39	E	0	161075.074	0.001	302.2210
28	4	25	27	4	24	A	0	161076.992	-0.018	146.1809
28	3	25	27	3	24	E	0	161082.352	0.089	146.1900
46	9	38	46	8	39	A	0	161084.092	-0.144	302.2158
28	3	25	27	3	24	A	0	161085.302	-0.029	146.1804
27	5	23	26	5	22	A	0	161087.795	0.009	144.2382
28	4	25	27	3	24	E	0	161091.417	-0.029	146.1900
28	4	25	27	3	24	A	0	161094.535	-0.017	146.1804
44	7	37	44	6	38	E	0	161101.680	0.001	278.7632
44	7	37	44	6	38	A	0	161113.910	-0.010	278.7574
44	8	37	44	7	38	E	0	161126.440	0.046	278.7633
29	2	27	28	2	26	E	0	161167.687	-0.124	147.6588
29	3	27	28	3	26	E	0	161167.687	0.061	147.6588
29	2	27	28	2	26	A	0	161169.912	-0.057	147.6487
29	2	27	28	3	26	A	0	161169.912	0.310	147.6487
29	3	27	28	2	26	A	0	161169.912	-0.239	147.6487
29	3	27	28	3	26	A	0	161169.912	0.128	147.6487
26	14	12	25	14	11	A	0	161235.930	0.134	171.4832
26	14	13	25	14	12	A	0	161235.930	0.134	171.4832
26	14	13	25	14	12	E	0	161237.206	0.075	171.4761
30	1	29	29	1	28	A	1	161255.877	0.000	277.3546
30	1	29	29	2	28	A	1	161255.877	0.004	277.3546
30	2	29	29	1	28	A	1	161255.877	-0.001	277.3546
30	2	29	29	2	28	A	1	161255.877	0.002	277.3546
27	4	23	26	4	22	E	1	161281.476	0.031	272.5624
27	4	23	26	4	22	E	0	161288.342	0.023	144.2317
27	4	23	26	4	22	A	0	161292.698	-0.070	144.2223
30	1	29	29	1	28	E	1	161297.798	-0.010	277.0102
30	1	29	29	2	28	E	1	161297.798	-0.005	277.0102
30	2	29	29	1	28	E	1	161297.798	-0.012	277.0102
30	2	29	29	2	28	E	1	161297.798	-0.007	277.0102
33	15	18	33	14	19	E	0	161312.488	-0.138	220.4919
33	15	19	33	14	20	E	0	161313.417	0.129	220.4786
30	1	29	29	1	28	E	0	161330.311	0.014	148.6866
30	2	29	29	2	28	E	0	161330.311	0.016	148.6866
30	1	29	29	1	28	A	0	161331.558	-0.026	148.6759
30	1	29	29	2	28	A	0	161331.558	-0.022	148.6759
30	2	29	29	1	28	A	0	161331.558	-0.028	148.6759
30	2	29	29	2	28	A	0	161331.558	-0.024	148.6759
37	3	34	37	2	35	A	1	161350.816	0.135	330.8199
37	4	34	37	3	35	A	1	161350.816	0.117	330.8199
33	15	18	33	14	19	A	0	161356.513	0.209	220.4852
27	5	23	26	4	22	A	1	161391.414	0.032	272.8640
31	0	31	30	0	30	A	1	161468.789	0.043	277.9850
31	0	31	30	1	30	A	1	161468.789	0.043	277.9850
31	1	31	30	0	30	A	1	161468.789	0.043	277.9850
31	1	31	30	1	30	A	1	161468.789	0.043	277.9850
31	0	31	30	0	30	E	1	161480.928	-0.029	277.6140
31	1	31	30	1	30	E	1	161480.928	-0.029	277.6140
31	0	31	30	0	30	A	0	161520.156	-0.201	149.2847
31	0	31	30	1	30	A	0	161520.156	-0.201	149.2847
31	1	31	30	0	30	A	0	161520.156	-0.201	149.2847
31	1	31	30	1	30	A	0	161520.156	-0.201	149.2847
31	0	31	30	0	30	E	0	161520.156	0.178	149.2963
31	1	31	30	1	30	E	0	161520.156	0.178	149.2963
26	13	14	25	13	13	A	0	161551.120	-0.056	166.3921
27	5	23	26	4	22	E	0	161557.017	-0.014	144.2317
27	5	23	26	4	22	A	0	161562.412	-0.011	144.2223
3	2	1	3	1	2	E	1	16165.707	0.204	197.1113
46	16	30	46	15	31	A	0	161769.330	-0.031	334.9812

J'	K'_a	K'_c	J''	K''_a	K''_c	Sym.	$v_{t_{CH_3}}$	$\nu_{Obs.}$	$\nu_{Obs.} - \nu_{Calc.}$	E_{low}
46	16	30	46	15	31	E	0	161815.999	-0.085	334.9836
32	15	17	32	14	18	E	0	161854.993	-0.034	213.6146
32	15	18	32	14	19	E	0	161856.218	0.005	213.6012
55	13	43	55	12	44	A	0	161881.219	-0.040	417.6349
55	13	43	55	12	44	E	0	161882.424	-0.028	417.6375
32	15	18	32	14	19	A	0	161898.913	-0.085	213.6079
26	12	15	25	12	14	A	0	161955.560	0.062	161.6992
26	12	15	25	12	14	E	0	161957.689	-0.004	161.6950
46	16	31	46	15	32	E	0	162019.704	-0.003	334.9644
37	3	34	37	2	35	E	0	162036.406	0.058	202.1987
37	4	34	37	3	35	E	0	162036.406	0.038	202.1987
37	3	34	37	2	35	A	0	162060.404	0.046	202.1892
37	4	34	37	3	35	A	0	162060.404	0.026	202.1892
37	3	34	37	2	35	E	1	162132.707	-0.041	330.5169
37	4	34	37	3	35	E	1	162132.707	-0.062	330.5169
46	16	31	46	15	32	A	0	162165.361	-0.014	334.9664
53	11	42	53	10	43	A	0	162166.825	-0.089	389.5841
53	11	42	53	10	43	E	0	162172.629	0.008	389.5872
55	17	39	55	16	40	E	0	162185.399	-0.001	437.8408
16	5	11	15	4	12	E	0	162228.352	0.023	95.1424
16	6	10	15	5	11	E	0	162240.699	0.016	96.9265
16	6	10	15	5	11	A	0	162246.682	0.024	96.9164
31	15	16	31	14	17	E	0	162344.237	-0.052	206.9537
31	15	17	31	14	18	E	0	162345.981	0.038	206.9402
31	15	17	31	14	18	A	0	162388.144	-0.027	206.9470
26	11	16	25	11	15	A	0	162488.942	0.057	157.4115
26	11	15	25	11	14	E	0	162491.343	0.090	157.4219
26	11	15	25	11	14	A	0	162492.376	-0.041	157.4116
26	11	16	25	11	15	E	0	162493.390	0.055	157.4091
19	6	14	18	5	13	E	0	162606.398	-0.090	108.2107
19	6	14	18	5	13	A	0	162644.440	0.016	108.2001
30	15	15	30	14	16	E	0	162784.851	-0.048	200.5083
30	15	16	30	14	17	E	0	162786.991	0.019	200.4948
34	1	33	34	0	34	E	0	162805.928	-0.004	171.8738
34	2	33	34	1	34	E	0	162805.928	-0.004	171.8738
30	15	15	30	14	16	A	0	162828.706	0.008	200.5017
34	1	33	34	0	34	A	0	162835.272	0.098	171.8623
34	2	33	34	1	34	A	0	162835.272	0.098	171.8623
54	17	37	53	18	36	A	0	162841.456	-0.142	426.1512
32	8	24	31	9	23	A	0	162883.924	-0.006	186.2560
34	1	33	34	0	34	E	1	162971.808	0.126	300.1860
34	2	33	34	1	34	E	1	162971.808	0.126	300.1860
39	4	35	39	3	36	A	1	163016.935	0.407	350.5263
39	5	35	39	4	36	A	1	163016.935	0.288	350.5263
26	5	21	25	5	20	E	0	163039.780	-0.002	141.6036
26	5	21	25	5	20	A	0	163047.722	0.009	141.5939
26	5	21	25	5	20	E	1	163063.119	-0.009	269.9291
22	6	17	21	5	16	E	0	163081.584	-0.074	121.4099
22	6	17	21	5	16	A	0	163117.385	0.004	121.3994
20	6	15	19	5	14	E	0	163120.238	-0.053	112.3948
20	6	15	19	5	14	A	0	163158.292	0.001	112.3841
21	6	16	20	5	15	E	0	163173.200	-0.054	116.7996
29	15	14	29	14	15	E	0	163180.840	-0.086	194.2778
29	15	15	29	14	16	E	0	163183.406	0.033	194.2643
21	6	16	20	5	15	A	0	163210.679	0.040	116.7890
23	6	18	22	5	17	A	0	163213.612	-0.054	126.1990
29	15	15	29	14	16	A	0	163224.719	0.001	194.2712
59	18	41	59	17	42	E	0	163226.062	-0.038	492.6351
26	10	17	25	10	16	E	0	163230.816	0.005	153.5400
26	10	16	25	10	15	E	0	163236.332	0.182	153.5522
62	16	47	62	15	48	A	0	163249.778	-0.077	520.8131
26	10	16	25	10	15	A	0	163255.172	-0.017	153.5415
2	1	2	1	0	1	E	1	16326.308	-0.007	195.8025
59	18	41	59	17	42	A	0	163283.010	-0.010	492.6345
27	5	22	26	6	21	A	1	163330.974	-0.010	275.8061
27	5	22	26	6	21	A	0	163340.250	-0.058	147.1758
27	5	22	26	6	21	E	0	163344.431	0.016	147.1849
45	16	29	45	15	30	A	0	163362.386	-0.014	325.2408
45	16	29	45	15	30	E	0	163388.278	-0.001	325.2441
53	17	36	53	16	37	E	0	163404.700	-0.065	414.6619
53	17	36	53	16	37	A	0	163409.611	-0.017	414.6613
45	16	30	45	15	31	E	0	163458.030	-0.033	325.2294
28	15	13	28	14	14	E	0	163536.016	-0.064	188.2614
28	15	14	28	14	15	E	0	163538.874	0.014	188.2478
28	15	13	28	14	14	A	0	163579.820	-0.018	188.2548
45	16	30	45	15	31	A	0	163580.905	0.045	325.2327
53	12	42	53	11	43	A	0	163633.748	-0.274	389.5910
53	12	42	53	11	43	E	0	163633.748	0.199	389.5940
39	4	35	39	3	36	E	0	163656.502	0.089	221.9366
39	5	35	39	4	36	E	0	163656.502	-0.043	221.9366
39	4	35	39	3	36	A	0	163678.090	0.120	221.9281
39	5	35	39	4	36	A	0	163678.090	-0.012	221.9281
39	4	35	39	3	36	E	1	163716.886	-0.001	350.2556
39	5	35	39	4	36	E	1	163716.886	-0.141	350.2556
24	6	19	23	5	18	E	0	163786.524	-0.050	131.1825
24	6	19	23	5	18	A	0	163815.447	0.017	131.1723
27	15	12	27	14	13	E	0	163853.681	-0.076	182.4583
27	15	13	27	14	14	E	0	163856.836	0.003	182.4447
26	7	20	25	7	19	E	0	163887.089	-0.073	144.4180
26	7	20	25	7	19	A	0	163888.118	0.063	144.4093
27	15	13	27	14	14	A	0	163897.506	0.014	182.4518
27	15	12	27	14	13	A	0	163897.506	0.015	182.4518
54	17	38	54	16	39	A	0	164028.776	-0.056	426.0885
26	9	18	25	9	17	A	0	164108.235	-0.014	150.0998
26	9	18	25	9	17	E	0	164133.314	-0.018	150.1047
26	15	11	26	14	12	E	0	164137.025	-0.057	176.8680
26	15	12	26	14	13	E	0	164140.442	0.021	176.8544
26	15	12	26	14	13	A	0	164180.801	0.008	176.8615
64	17	48	64	16	49	E	0	164209.689	0.008	553.3302
25	7	18	24	7	17	A	0	164272.920	0.084	139.6057
25	7	18	24	7	17	E	0	164277.586	-0.023	139.6150
36	2	34	36	1	35	A	1	164300.433	-0.144	318.5989
36	3	34	36	2	35	A	1	164300.433	-0.145	318.5989
2	1	2	1	0	1	A	1	16433.505	0.008	196.1862
25	15	10	25	14	11	E	0	164388.886	-0.054	171.4897
25	15	11	25	14	12	E	0	164392.567	0.056	171.4761
25	15	11	25	14	12	A	0	164432.644	0.016	171.4832
25	15	10	25	14	11	A	0	164432.644	0.016	171.4832
64	19	45	64	18	46	E	0	164506.405	-0.036	564.8258
28	6	22	27	7	21	A	0	164531.001	-0.109	155.5384
51	10	41	51	9	42	A	0	164532.744	0.007	362.5462
26	9	17	25	9	16	E	0	164550.931	0.016	150.1247
28	6	22	27	7	21	E	0	164554.619	0.035	155.5469
26	9	17	25	9	16	A	0	164570.516	-0.006	150.1164
24	15	9	24	14	10	E	0	164611.922	-0.086	166.3229
24	15	10	24	14	11	E	0	164615.846	0.062	166.3093

J'	K'_a	K'_c	J''	K''_a	K''_c	Sym.	$v_{t_{CH_3}}$	$\nu_{Obs.}$	$\nu_{Obs.} - \nu_{Calc.}$	E_{low}
24	15	10	24	14	11	A	0	164655.687	0.012	166.3165
24	15	9	24	14	10	A	0	164655.687	0.012	166.3165
26	8	19	25	8	18	A	0	164745.563	-0.027	147.0881
26	8	19	25	8	18	E	0	164749.033	-0.019	147.0957
25	6	19	24	6	18	E	0	164763.726	-0.010	138.0010
25	6	19	24	6	18	A	0	164766.896	-0.020	137.9908
44	16	28	44	15	29	A	0	164772.037	-0.078	315.7313
44	16	28	44	15	29	E	0	164773.074	0.030	315.7355
11	9	3	10	8	3	E	0	164792.441	0.004	90.7479
44	16	29	44	15	30	E	0	164793.715	-0.050	315.7223
23	15	8	23	14	9	E	0	164808.757	-0.015	161.3671
23	15	9	23	14	10	E	0	164812.750	0.022	161.3534
11	9	2	10	8	2	E	0	164818.995	-0.011	90.7589
11	9	3	10	8	2	A	0	164839.922	0.030	90.7462
11	9	2	10	8	3	A	0	164839.922	0.029	90.7462
51	11	41	51	10	42	E	1	164842.276	0.191	490.8457
23	15	9	23	14	10	A	0	164852.429	0.012	161.3606
23	15	8	23	14	9	A	0	164852.429	0.012	161.3606
2	1	2	1	0	1	E	0	16487.376	0.014	67.4660
44	16	29	44	15	30	A	0	164889.832	-0.019	315.7270
2	1	2	1	0	1	A	0	16489.919	-0.004	67.4541
41	5	36	41	4	37	E	1	164958.610	-0.165	371.0648
41	6	36	41	5	37	E	1	164959.564	0.026	371.0648
22	15	7	22	14	8	E	0	164981.509	-0.038	156.6216
22	15	8	22	14	9	E	0	164985.676	0.015	156.6079
22	15	8	22	14	9	A	0	165025.234	0.062	156.6151
22	15	7	22	14	8	A	0	165025.234	0.062	156.6151
36	2	34	36	1	35	E	0	165051.524	0.068	189.9491
51	11	41	51	10	42	E	0	165053.373	-0.025	362.5519
51	11	41	51	10	42	A	0	165056.624	-0.077	362.5483
36	2	34	36	1	35	A	0	165078.658	0.098	189.9387
21	15	6	21	14	7	E	0	165132.456	-0.042	152.0860
25	6	20	24	5	19	E	0	165133.996	-0.054	136.3164
21	15	7	21	14	8	E	0	165136.841	0.093	152.0723
25	6	20	24	5	19	A	0	165158.240	0.010	136.3064
56	12	44	56	11	45	A	0	165160.019	-0.108	429.1658
56	12	44	56	11	45	E	0	165171.375	-0.047	429.1682
21	15	7	21	14	8	A	0	165176.110	0.006	152.0796
21	15	6	21	14	7	A	0	165176.110	0.006	152.0796
36	2	34	36	1	35	E	1	165185.492	0.007	318.2642
36	3	34	36	2	35	E	1	165185.492	0.006	318.2642
20	15	5	20	14	6	E	0	165263.568	-0.077	147.7598
20	15	6	20	14	7	E	0	165268.052	0.039	147.7461
20	15	6	20	14	7	A	0	165307.239	0.006	147.7534
20	15	5	20	14	6	A	0	165307.239	0.006	147.7534
19	15	4	19	14	5	E	0	165376.825	-0.057	143.6425
19	15	5	19	14	6	E	0	165381.298	-0.055	143.6288
19	15	5	19	14	6	A	0	165420.451	-0.002	143.6361
19	15	4	19	14	5	A	0	165420.451	-0.002	143.6361
64	18	47	64	17	48	E	0	165436.040	-0.021	558.8076
63	18	46	63	17	47	E	0	165459.275	-0.011	545.0853
18	15	3	18	14	4	E	0	165473.891	-0.090	139.7337
18	15	4	18	14	5	E	0	165478.554	0.016	139.7200
63	18	46	63	17	47	A	0	165500.675	-0.060	545.0849
18	15	4	18	14	5	A	0	165517.553	0.017	139.7273
18	15	3	18	14	4	A	0	165517.553	0.017	139.7273
17	15	2	17	14	3	E	0	165556.396	-0.208	136.0330
17	15	3	17	14	4	E	0	165561.284	0.049	136.0193
13	8	6	12	7	6	E	0	165568.297	-0.004	92.6439
13	8	5	12	7	5	E	0	165599.570	-0.051	92.6537
17	15	3	17	14	4	A	0	165600.149	0.007	136.0266
17	15	2	17	14	3	A	0	165600.149	0.007	136.0266
13	8	6	12	7	5	A	0	165614.601	0.073	92.6405
13	8	5	12	7	6	A	0	165615.942	-0.063	92.6405
58	14	45	58	13	46	A	0	165642.327	-0.097	458.7394
58	14	45	58	13	46	E	0	165644.570	-0.032	458.7414
49	9	40	49	8	41	E	1	165650.161	0.092	464.8239
16	15	2	16	14	3	A	0	165670.037	0.209	132.5336
16	15	1	16	14	2	A	0	165670.037	0.209	132.5336
53	17	37	53	16	38	E	0	165741.728	0.036	414.5665
49	9	40	49	8	41	E	0	165821.804	-0.026	336.5219
43	7	37	43	6	38	E	1	165830.665	-0.037	392.9357
53	17	37	53	16	38	A	0	165846.429	0.051	414.5669
43	6	37	43	5	38	E	0	165846.429	-0.101	264.6189
43	6	37	43	5	38	A	0	165862.675	0.069	264.6124
43	7	37	43	6	38	A	0	165866.014	0.059	264.6124
27	6	22	26	6	21	A	1	165878.844	0.022	275.8061
15	7	8	14	6	8	E	0	165910.216	-0.050	95.8291
28	4	24	27	5	23	A	1	165919.641	0.012	278.2475
15	7	9	14	6	9	E	0	165971.630	-0.043	95.8173
49	10	40	49	9	41	E	0	165992.210	-0.033	336.5224
49	10	40	49	9	41	A	0	165998.732	-0.086	336.5182
43	16	27	43	15	28	E	0	166012.965	-0.045	306.4551
43	16	28	43	15	29	E	0	166019.129	0.003	306.4423
27	6	22	26	6	21	E	0	166025.275	-0.070	147.1849
27	6	22	26	6	21	A	0	166029.148	0.050	147.1758
28	4	24	27	5	23	E	0	166051.210	-0.001	149.6207
28	4	24	27	5	23	A	0	166054.480	-0.032	149.6115
28	5	24	27	5	23	A	1	166060.699	0.008	278.2475
29	3	26	28	4	25	A	1	166066.928	0.017	280.1988
29	4	26	28	4	25	A	1	166071.370	0.027	280.1988
29	3	26	28	3	25	A	1	166075.438	-0.008	280.1985
29	4	26	28	3	25	A	1	166079.893	0.015	280.1985
43	16	28	43	15	29	A	0	166093.837	-0.031	306.4480
65	18	48	65	17	49	E	0	166099.384	0.045	572.7326
15	7	8	14	6	9	A	0	166110.232	0.006	95.8098
29	3	26	28	4	25	E	1	166164.586	-0.066	279.8933
29	4	26	28	4	25	E	1	166169.601	-0.057	279.8933
28	4	24	27	4	23	A	1	166172.505	0.070	278.2390
29	3	26	28	3	25	E	1	166174.199	-0.031	279.8930
29	4	26	28	3	25	E	1	166179.001	-0.235	279.8930
29	3	26	28	4	25	E	0	166186.108	-0.007	151.5634
29	3	26	28	4	25	A	0	166189.090	-0.020	151.5539
29	4	26	28	4	25	E	0	166190.960	0.059	151.5634
29	4	26	28	4	25	A	0	166193.929	0.012	151.5539
30	2	28	29	2	27	A	1	166195.950	-0.049	281.6832
30	2	28	29	3	27	A	1	166195.950	0.118	281.6832
30	3	28	29	2	27	A	1	166195.950	-0.130	281.6832
30	3	28	29	3	27	A	1	166195.950	0.036	281.6832
29	3	26	28	3	25	A	0	166198.237	-0.094	151.5536
29	4	26	28	3	25	E	0	166200.137	0.053	151.5631
29	4	26	28	3	25	A	0	166203.125	-0.013	151.5536
28	5	24	27	5	23	A	0	166205.527	0.011	149.6115
47	8	39	47	7	40	E	1	166254.231	0.118	439.8253
30	2	28	29	2	27	E	1	166265.902	-0.078	281.3618
30	2	28	29	3	27	E	1	166265.902	0.113	281.3619

J'	K'_a	K'_c	J''	K''_a	K''_c	Sym.	$v_{t_{CH_3}}$	$\nu_{Obs.}$	$\nu_{Obs.} - \nu_{Calc.}$	E_{low}
30	3	28	29	2	27	E	1	166265.902	-0.172	281.3618
30	3	28	29	3	27	E	1	166265.902	0.018	281.3619
45	7	38	45	6	39	E	1	166279.575	-0.019	415.8591
30	2	28	29	2	27	E	0	166293.642	-0.069	153.0348
30	3	28	29	3	27	E	0	166293.642	0.023	153.0348
30	2	28	29	2	27	A	0	166295.798	-0.059	153.0248
30	2	28	29	3	27	A	0	166295.798	0.123	153.0248
30	3	28	29	2	27	A	0	166295.798	-0.149	153.0248
30	3	28	29	3	27	A	0	166295.798	0.033	153.0248
38	3	35	38	2	36	A	1	166303.785	0.056	337.7314
38	4	35	38	3	36	A	1	166303.785	0.047	337.7314
47	9	39	47	8	40	E	1	166308.362	-0.019	439.8254
28	4	24	27	4	23	E	1	166309.468	0.002	277.9422
28	5	24	27	4	23	A	1	166313.526	0.029	278.2390
28	4	24	27	4	23	E	0	166319.858	-0.064	149.6117
28	4	24	27	4	23	A	0	166324.121	-0.046	149.6025
45	7	38	45	6	39	E	0	166343.830	-0.000	287.5456
47	8	39	47	7	40	E	0	166368.078	-0.014	311.5166
45	8	38	45	7	39	A	0	166370.639	-0.014	287.5399
47	8	39	47	7	40	A	0	166377.695	-0.029	311.5117
31	1	30	30	1	29	A	1	166383.609	0.009	282.7335
31	1	30	30	2	29	A	1	166383.609	0.011	282.7335
31	2	30	30	1	29	A	1	166383.609	0.009	282.7335
31	2	30	30	2	29	A	1	166383.609	0.010	282.7335
47	9	39	47	8	40	E	0	166418.846	-0.064	311.5168
31	1	30	30	1	29	E	1	166425.346	-0.026	282.3905
31	1	30	30	2	29	E	1	166425.346	-0.024	282.3905
31	2	30	30	1	29	E	1	166425.346	-0.027	282.3905
31	2	30	30	2	29	E	1	166425.346	-0.025	282.3905
47	9	39	47	8	40	A	0	166428.756	-0.046	311.5118
31	1	30	30	2	29	E	0	166459.290	0.018	154.0680
31	2	30	30	1	29	E	0	166459.290	0.015	154.0680
31	1	30	30	1	29	A	0	166460.517	-0.040	154.0573
31	1	30	30	2	29	A	0	166460.517	-0.038	154.0573
31	2	30	30	1	29	A	0	166460.517	-0.040	154.0573
31	2	30	30	2	29	A	0	166460.517	-0.038	154.0573
28	5	24	27	4	23	E	1	166465.494	-0.035	277.9422
28	5	24	27	4	23	E	0	166470.370	0.002	149.6117
28	5	24	27	4	23	A	0	166475.170	-0.000	149.6025
32	0	32	31	0	31	A	1	166597.164	0.026	283.3710
32	0	32	31	1	31	A	1	166597.164	0.026	283.3710
32	1	32	31	0	31	A	1	166597.164	0.026	283.3710
32	1	32	31	1	31	A	1	166597.164	0.026	283.3710
32	0	32	31	0	31	E	1	166609.221	-0.028	283.0004
32	1	32	31	1	31	E	1	166609.221	-0.028	283.0004
32	0	32	31	0	31	A	0	166649.800	-0.206	154.6725
32	0	32	31	1	31	A	0	166649.800	-0.206	154.6725
32	1	32	31	0	31	A	0	166649.800	-0.206	154.6725
32	1	32	31	1	31	A	0	166649.800	-0.206	154.6725
32	0	32	31	0	31	E	0	166649.800	0.171	154.6840
32	1	32	31	1	31	E	0	166649.800	0.171	154.6840
27	18	9	26	18	8	A	0	166731.057	-0.156	201.1112
27	18	10	26	18	9	A	0	166731.057	-0.156	201.1112
27	18	9	26	18	8	E	0	166731.804	0.022	201.1116
27	17	11	26	17	10	A	0	166890.151	-0.132	194.4650
27	17	10	26	17	9	A	0	166890.151	-0.132	194.4650
27	17	11	26	17	10	E	0	166891.092	0.004	194.4542
26	6	21	25	5	20	A	1	166908.053	0.122	270.2387
35	1	34	35	0	35	A	1	166926.570	-0.452	306.6257
35	2	34	35	1	35	A	1	166926.570	-0.452	306.6257
38	3	35	38	2	36	E	0	167010.863	0.061	209.1138
38	4	35	38	3	36	E	0	167010.863	0.051	209.1138
38	3	35	38	2	36	A	0	167035.660	0.079	209.1044
38	4	35	38	3	36	A	0	167035.660	0.069	209.1044
38	3	35	38	2	36	E	1	167110.727	-0.038	337.4307
38	4	35	38	3	36	E	1	167110.727	-0.048	337.4307
42	16	26	42	15	27	E	0	167134.059	-0.047	297.4009
42	16	27	42	15	28	E	0	167136.824	-0.017	297.3881
42	16	26	42	15	27	A	0	167165.685	0.023	297.3956
61	18	44	61	17	45	E	0	167176.816	0.029	518.2751
42	16	27	42	15	28	A	0	167197.490	-0.044	297.3944
27	15	13	26	15	12	A	0	167310.409	-0.068	182.3379
27	15	12	26	15	11	A	0	167310.409	-0.068	182.3379
26	6	21	25	5	20	E	0	167322.178	-0.015	141.6036
26	6	21	25	5	20	A	0	167341.665	0.008	141.5939
27	5	22	26	5	21	A	1	167417.288	0.126	275.6698
59	13	46	59	12	47	A	0	167472.108	-0.106	470.9535
59	13	46	59	12	47	E	0	167490.082	-0.024	470.9553
52	17	36	52	16	37	E	0	167517.417	0.029	403.2758
26	8	18	25	8	17	A	0	167572.078	-0.002	147.2281
56	13	44	56	12	45	A	0	167573.482	-0.117	429.1784
56	13	44	56	12	45	E	0	167575.071	-0.039	429.1807
26	8	18	25	8	17	E	0	167576.212	0.028	147.2363
27	14	13	26	14	12	A	0	167593.690	-0.087	176.8615
27	14	14	26	14	13	A	0	167593.690	-0.086	176.8615
27	14	13	26	14	12	E	0	167594.526	0.038	176.8680
27	5	22	26	5	21	E	0	167626.871	0.044	147.0420
27	5	22	26	5	21	A	0	167634.237	-0.014	147.0326
52	17	36	52	16	37	A	0	167645.934	0.059	403.2767
58	18	40	58	17	41	E	0	167652.547	-0.038	479.9082
58	18	40	58	17	41	A	0	167693.099	-0.012	479.9081
35	1	34	35	0	35	E	0	167742.759	0.053	177.9459
35	2	34	35	1	35	E	0	167742.759	0.053	177.9459
63	16	48	63	15	49	E	0	167893.273	-0.023	534.1025
35	1	34	35	0	35	E	1	167913.414	0.049	306.2567
35	2	34	35	1	35	E	1	167913.414	0.049	306.2567
27	13	15	26	13	14	A	0	167949.350	-0.033	171.7808
27	13	14	26	13	13	E	0	167950.267	0.153	171.7887
27	13	15	26	13	14	E	0	167951.252	-0.022	171.7752
40	4	36	40	3	37	A	1	168006.597	0.401	357.9448
40	5	36	40	4	37	A	1	168006.597	0.341	357.9448
41	16	25	41	15	26	E	0	168152.227	-0.052	288.5711
41	16	26	41	15	27	E	0	168154.811	-0.002	288.5583
41	16	25	41	15	26	A	0	168191.103	0.037	288.5656
41	16	26	41	15	27	A	0	168206.997	-0.065	288.5650
51	17	34	51	16	35	A	0	168521.817	-0.025	392.2509
51	17	34	51	16	35	E	0	168564.114	-0.103	392.2515
60	18	43	60	17	44	E	0	168640.654	0.028	505.1989
40	4	36	40	3	37	E	0	168666.400	0.066	229.3594
40	5	36	40	4	37	E	0	168666.400	-0.001	229.3594
40	4	36	40	3	37	A	0	168688.711	0.098	229.3511
40	5	36	40	4	37	A	0	168688.711	0.030	229.3511
60	18	43	60	17	44	A	0	168697.621	-0.049	505.1990
40	4	36	40	3	37	E	1	168729.899	-0.040	357.6771
40	5	36	40	4	37	E	1	168729.899	-0.112	357.6771
62	14	48	62	13	49	E	0	169000.731	-0.009	514.9421

J'	K'_a	K'_c	J''	K''_a	K''_c	Sym.	$v_{t_{CH_3}}$	$\nu_{Obs.}$	$\nu_{Obs.} - \nu_{Calc.}$	E_{low}
27	11	16	26	11	15	E	0	169016.867	0.058	162.8420
27	11	17	26	11	16	E	0	169018.962	-0.078	162.8293
27	11	16	26	11	15	A	0	169020.042	0.049	162.8318
68	16	52	68	15	53	E	0	169055.052	0.016	609.4866
40	16	24	40	15	25	E	0	169078.786	-0.031	279.9645
40	16	25	40	15	26	E	0	169081.951	0.014	279.9516
40	16	25	40	15	26	A	0	169129.174	-0.095	279.9586
37	2	35	37	1	36	A	1	169226.935	-0.174	325.1751
37	3	35	37	2	36	A	1	169226.935	-0.174	325.1751
54	12	43	54	11	44	E	0	169314.467	-0.141	400.6135
54	12	43	54	11	44	A	0	169315.426	0.002	400.6107
61	15	47	61	14	48	A	0	169320.130	-0.046	502.0485
61	15	47	61	14	48	E	0	169322.565	-0.034	502.0499
51	17	35	51	16	36	A	0	169384.358	-0.019	392.2182
28	5	23	27	6	22	A	1	169571.645	-0.012	281.3392
28	5	23	27	6	22	E	1	169579.778	-0.083	281.0522
28	5	23	27	6	22	A	0	169636.979	-0.022	152.7139
28	5	23	27	6	22	E	0	169638.091	0.019	152.7229
67	18	50	67	17	51	E	0	169704.213	-0.002	601.1635
67	18	50	67	17	51	A	0	169722.184	-0.109	601.1630
27	7	21	26	7	20	E	0	169750.003	-0.003	149.8846
27	7	21	26	7	20	A	0	169751.736	0.009	149.8761
27	10	18	26	10	17	A	0	169821.364	0.003	158.9842
27	10	18	26	10	17	E	0	169855.951	-0.013	158.9848
17	6	11	16	5	12	E	0	169873.347	-0.000	100.2720
17	6	11	16	5	12	A	0	169884.341	0.048	100.2619
27	10	17	26	10	16	E	0	169886.438	0.066	158.9971
27	10	17	26	10	16	A	0	169916.439	0.141	158.9871
39	16	23	39	15	24	E	0	169922.674	-0.024	271.5797
39	16	24	39	15	25	E	0	169926.547	0.004	271.5668
7	2	5	7	1	6	A	1	16994.087	-0.007	202.1801
27	6	22	26	5	21	A	1	169965.130	0.129	275.6698
39	16	23	39	15	24	A	0	169967.322	0.083	271.5742
39	16	24	39	15	25	A	0	169970.878	-0.100	271.5740
42	5	37	42	4	38	E	0	169996.344	0.203	250.6777
42	6	37	42	5	38	E	0	169996.344	-0.169	250.6777
37	2	35	37	1	36	E	0	170000.790	0.045	196.5281
37	3	35	37	2	36	E	0	170000.790	0.044	196.5281
42	5	37	42	4	38	A	0	170015.962	0.212	250.6704
42	6	37	42	5	38	A	0	170015.962	-0.163	250.6704
10	10	1	9	9	1	E	0	170081.684	-0.007	91.9310
10	10	0	9	9	0	E	0	170103.480	0.013	91.9428
10	10	0	9	9	1	A	0	170129.199	0.020	91.9308
10	10	1	9	9	0	A	0	170129.199	0.020	91.9308
37	2	35	37	1	36	E	1	170138.836	-0.034	324.8417
37	3	35	37	2	36	E	1	170138.836	-0.034	324.8417
63	19	44	63	18	45	E	0	170283.851	-0.022	550.9338
27	6	22	26	5	21	E	0	170307.737	-0.018	147.0420
52	10	42	52	9	43	E	0	170314.293	-0.023	373.0494
52	10	42	52	9	43	A	0	170316.712	-0.050	373.0460
27	6	22	26	5	21	A	0	170322.989	-0.052	147.0326
26	6	20	25	6	19	E	0	170323.797	0.061	143.4969
26	6	20	25	6	19	A	0	170329.301	0.003	143.4869
63	19	44	63	18	45	A	0	170346.537	-0.030	550.9337
59	18	42	59	17	43	E	0	170351.798	0.002	492.3481
27	6	22	26	5	21	E	1	170398.037	0.111	275.3683
52	11	42	52	10	43	E	1	170420.378	0.229	501.3427
50	17	33	50	16	34	A	0	170539.045	-0.045	381.4096
35	9	26	34	10	25	A	0	170570.144	-0.040	210.5355
50	17	33	50	16	34	E	0	170584.717	-0.086	381.4106
52	11	42	52	10	43	E	0	170627.284	-0.049	373.0506
52	11	42	52	10	43	A	0	170631.196	-0.070	373.0472
35	9	26	34	10	25	E	0	170649.460	0.048	210.5414
38	16	22	38	15	23	E	0	170691.471	-0.043	263.4157
55	17	39	54	18	36	A	0	170693.855	0.056	437.5591
38	16	23	38	15	24	E	0	170696.056	-0.002	263.4027
38	16	22	38	15	23	A	0	170737.061	0.094	263.4102
38	16	23	38	15	24	A	0	170738.579	-0.126	263.4102
27	9	19	26	9	18	A	0	170765.835	0.033	155.5738
27	9	19	26	9	18	E	0	170780.562	-0.001	155.5796
7	2	5	7	1	6	E	0	17081.438	-0.012	73.4661
7	2	5	7	1	6	A	0	17084.659	-0.002	73.4543
50	17	34	50	16	35	E	0	170889.113	0.061	381.3885
59	19	40	58	20	39	A	0	170926.727	-0.074	498.5576
12	9	4	11	8	4	E	0	170930.714	0.014	93.0117
12	9	3	11	8	3	E	0	170957.297	-0.035	93.0227
12	9	3	11	8	4	A	0	170978.190	0.006	93.0099
44	6	38	44	5	39	A	0	170989.238	0.009	273.0538
44	7	38	44	6	39	A	0	170991.122	0.094	273.0538
50	17	34	50	16	35	A	0	171034.762	-0.033	381.3910
29	4	25	28	5	24	A	1	171091.594	0.024	283.7866
28	6	23	27	6	22	A	1	171119.686	0.039	281.3392
29	5	25	28	5	24	A	1	171169.253	-0.031	283.7866
50	9	41	50	8	42	E	1	171170.625	0.040	474.8034
27	8	20	26	8	19	A	0	171182.098	0.004	152.5834
27	8	20	26	8	19	E	0	171184.106	-0.015	152.5911
30	3	27	29	4	26	A	1	171186.094	0.017	285.7384
30	4	27	29	4	26	A	1	171188.451	0.090	285.7384
30	3	27	29	3	26	A	1	171190.568	0.059	285.7382
30	4	27	29	3	26	A	1	171192.769	-0.025	285.7382
29	4	25	28	5	24	E	1	171209.254	0.009	283.4949
29	4	25	28	5	24	E	0	171228.374	-0.018	155.1646
29	4	25	28	5	24	A	0	171231.879	-0.051	155.1555
39	3	36	39	2	37	A	1	171253.860	0.048	344.8139
39	4	36	39	3	37	A	1	171253.860	0.044	344.8139
28	6	23	27	6	22	E	1	171264.062	-0.029	281.0522
28	6	23	27	6	22	E	0	171273.131	0.043	152.7229
28	6	23	27	6	22	A	0	171277.050	-0.129	152.7139
30	3	27	29	4	26	E	1	171283.294	-0.040	285.4361
30	4	27	29	4	26	E	1	171285.871	-0.058	285.4361
30	3	27	29	3	26	E	1	171288.289	-0.050	285.4359
30	4	27	29	3	26	E	1	171290.837	-0.098	285.4359
30	3	27	29	4	26	E	0	171306.386	-0.006	157.1069
30	3	27	29	4	26	A	0	171309.298	-0.076	157.0975
30	4	27	29	3	26	E	0	171313.670	0.018	157.1068
30	4	27	29	3	26	A	0	171316.646	-0.021	157.0974
59	14	46	59	13	47	A	0	171317.941	-0.122	470.9755
31	2	29	30	2	28	A	1	171321.088	-0.011	287.2269
31	2	29	30	3	28	A	1	171321.088	0.071	287.2269
31	3	29	30	2	28	A	1	171321.088	-0.051	287.2269
31	3	29	30	3	28	A	1	171321.088	0.031	287.2269
50	9	41	50	8	42	E	0	171333.387	-0.053	346.5029
57	18	39	57	17	40	A	0	171334.447	0.050	467.4483
50	9	41	50	8	42	A	0	171340.167	-0.056	346.4988
29	4	25	28	4	24	E	1	171365.314	0.007	283.4896
29	4	25	28	4	24	E	0	171378.824	-0.014	155.1596

J'	K_a'	K_c'	J''	K_a''	K_c''	Sym.	$v_{t_{CH_3}}$	$\nu_{Obs.}$	$\nu_{Obs.} - \nu_{Calc.}$	E_{low}
29	4	25	28	4	24	A	0	171382.939	0.005	155.1505
31	2	29	30	2	28	E	1	171390.668	-0.056	286.9079
31	2	29	30	3	28	E	1	171390.668	0.039	286.9079
31	3	29	30	2	28	E	1	171390.668	-0.102	286.9079
31	3	29	30	3	28	E	1	171390.668	-0.008	286.9079
37	16	21	37	15	22	E	0	171391.832	-0.034	255.4715
37	16	22	37	15	23	E	0	171397.077	0.026	255.4584
31	2	29	30	2	28	E	0	171419.923	-0.045	158.5817
31	3	29	30	3	28	E	0	171419.923	0.000	158.5817
31	2	29	30	2	28	A	0	171422.058	-0.047	158.5718
31	2	29	30	3	28	A	0	171422.058	0.043	158.5718
31	3	29	30	2	28	A	0	171422.058	-0.091	158.5718
31	3	29	30	3	28	A	0	171422.058	-0.001	158.5718
50	10	41	50	9	42	E	0	171432.667	-0.065	346.5032
50	10	41	50	9	42	A	0	171439.899	-0.116	346.4992
29	5	25	28	4	24	E	1	171451.829	-0.000	283.4896
29	5	25	28	4	24	E	0	171462.014	0.012	155.1596
29	5	25	28	4	24	A	0	171466.436	0.014	155.1505
46	7	39	46	6	40	E	1	171498.449	-0.018	424.8101
16	7	10	15	6	9	A	0	171499.570	0.075	98.9603
46	8	39	46	7	40	E	1	171506.554	-0.038	424.8102
32	1	31	31	1	30	A	1	171511.220	-0.073	288.2835
32	1	31	31	2	30	A	1	171511.220	-0.072	288.2835
32	2	31	31	1	30	A	1	171511.220	-0.073	288.2835
32	2	31	31	2	30	A	1	171511.220	-0.073	288.2835
32	1	31	31	1	30	E	1	171552.862	-0.049	287.9419
32	1	31	31	2	30	E	1	171552.862	-0.048	287.9419
32	2	31	31	1	30	E	1	171552.862	-0.050	287.9419
32	2	31	31	2	30	E	1	171552.862	-0.049	287.9419
46	7	39	46	6	40	E	0	171558.901	-0.029	296.4981
27	9	18	26	9	17	A	0	171582.529	0.043	155.6059
32	1	31	31	1	30	E	0	171588.249	0.023	159.6205
32	2	31	31	2	30	E	0	171588.249	0.024	159.6205
32	1	31	31	1	30	A	0	171589.486	-0.018	159.6098
32	1	31	31	2	30	A	0	171589.486	-0.017	159.6098
32	2	31	31	1	30	A	0	171589.486	-0.019	159.6098
32	2	31	31	2	30	A	0	171589.486	-0.018	159.6098
48	8	40	48	7	41	E	1	171596.447	0.103	449.2894
48	9	40	48	8	41	E	1	171627.187	0.005	449.2895
16	7	9	15	6	9	E	0	171637.617	-0.029	98.9705
14	8	7	13	7	7	E	0	171647.274	0.020	95.3340
14	8	6	13	7	6	E	0	171678.710	-0.071	95.3438
14	8	7	13	7	6	A	0	171691.839	-0.061	95.3307
48	8	40	48	7	41	E	0	171704.898	-0.031	320.9822
26	7	19	25	7	18	E	0	171719.454	0.079	145.0948
33	0	33	32	0	32	A	1	171725.515	0.074	288.9281
33	0	33	32	1	32	A	1	171725.515	0.074	288.9281
33	1	33	32	0	32	A	1	171725.515	0.074	288.9281
33	1	33	32	1	32	A	1	171725.515	0.074	288.9281
33	0	33	32	0	32	E	1	171737.441	-0.011	288.5579
33	1	33	32	1	32	E	1	171737.441	-0.011	288.5579
33	0	33	32	0	32	A	0	171779.360	-0.206	160.2313
33	0	33	32	1	32	A	0	171779.360	-0.206	160.2313
33	1	33	32	0	32	A	0	171779.360	-0.206	160.2313
33	1	33	32	1	32	A	0	171779.360	-0.206	160.2313
33	0	33	32	0	32	E	0	171779.360	0.168	160.2428
33	1	33	32	1	32	E	0	171779.360	0.168	160.2428
57	12	45	57	11	46	A	0	171793.378	-0.121	440.8812
57	12	45	57	11	46	E	0	171801.428	-0.031	440.8834
36	1	35	36	0	36	A	1	171838.304	-0.488	312.8670
36	2	35	36	1	36	A	1	171838.304	-0.488	312.8670
39	3	36	39	2	37	E	0	171982.154	0.000	216.1999
39	4	36	39	3	37	E	0	171982.154	-0.004	216.1999
39	3	36	39	2	37	A	0	172007.796	0.103	216.1906
39	4	36	39	3	37	A	0	172007.796	0.098	216.1906
36	16	20	36	15	21	E	0	172029.508	-0.072	247.7459
36	16	21	36	15	22	E	0	172035.326	-0.018	247.7327
36	16	21	36	15	22	A	0	172075.777	-0.155	247.7405
39	3	36	39	2	37	E	1	172085.500	-0.046	344.5154
39	4	36	39	3	37	E	1	172085.500	-0.051	344.5154
28	5	23	27	5	22	A	1	172119.549	0.053	281.2543
16	7	9	15	6	10	A	0	172137.135	0.028	98.9419
58	18	41	58	17	42	E	0	172205.205	0.069	479.7262
58	18	41	58	17	42	A	0	172280.352	-0.027	479.7269
49	17	32	49	16	33	A	0	172303.757	-0.019	370.8046
28	5	23	27	5	22	E	0	172319.002	0.001	152.6334
28	5	23	27	5	22	A	0	172325.766	-0.025	152.6243
28	5	23	27	5	22	E	1	172331.599	0.006	280.9604
49	17	32	49	16	33	E	0	172335.364	-0.096	370.8064
7	2	5	7	1	6	E	1	17245.898	-0.034	201.8020
49	17	33	49	16	34	E	0	172453.016	-0.009	370.7906
49	17	33	49	16	34	A	0	172582.408	-0.031	370.7942
35	16	19	35	15	20	E	0	172609.778	-0.067	240.2382
35	16	20	35	15	21	E	0	172616.097	-0.036	240.2249
35	16	19	35	15	20	A	0	172655.949	0.061	240.2328
36	1	35	36	0	36	A	0	172710.092	0.115	184.1777
36	2	35	36	1	36	A	0	172710.092	0.115	184.1777
41	4	37	41	3	38	A	1	172990.809	0.333	365.5342
41	5	37	41	4	38	A	1	172990.809	0.302	365.5342
64	16	49	64	15	50	E	0	172993.684	-0.017	547.5597
28	18	10	27	18	9	A	0	173005.237	-0.114	206.6727
28	18	11	27	18	10	A	0	173005.237	-0.114	206.6727
28	18	11	27	18	10	E	0	173006.093	0.003	206.6611
34	16	18	34	15	19	E	0	173137.261	-0.061	232.9474
34	16	19	34	15	20	E	0	173144.085	0.001	232.9341
34	16	19	34	15	20	A	0	173183.400	-0.013	232.9421
57	13	45	57	12	46	A	0	173320.214	-0.083	440.8887
57	13	45	57	12	46	E	0	173321.853	0.094	440.8908
28	16	12	27	16	11	A	0	173395.302	-0.043	193.7798
28	16	13	27	16	12	A	0	173395.302	-0.043	193.7798
28	16	13	27	16	12	E	0	173396.351	-0.117	193.7702
67	19	49	67	18	50	E	0	173429.643	0.049	606.8242
68	19	50	68	18	51	E	0	173454.062	-0.035	621.4161
33	16	17	33	15	18	E	0	173616.159	-0.065	225.8728
33	16	18	33	15	19	E	0	173623.421	0.010	225.8594
29	6	23	28	7	22	A	0	173649.685	0.009	161.3910
28	15	14	27	15	13	A	0	173652.919	-0.020	187.9188
28	15	13	27	15	12	A	0	173652.919	-0.020	187.9188
28	15	13	27	15	12	E	0	173653.738	0.040	187.9239
28	15	14	27	15	13	E	0	173654.350	0.031	187.9104
33	16	17	33	15	18	A	0	173662.229	0.009	225.8675
29	6	23	28	7	22	E	0	173666.030	0.031	161.3994
28	6	23	27	5	22	A	1	173667.581	0.096	281.2543
41	4	37	41	3	38	E	0	173670.678	0.035	236.9532
41	5	37	41	4	38	E	0	173670.680	0.002	236.9532
41	4	37	41	3	38	A	0	173693.725	0.091	236.9450

J'	K'_a	K'_c	J''	K''_a	K''_c	Sym.	$v_{t_{CH_3}}$	$\nu_{Obs.}$	$\nu_{Obs.} - \nu_{Calc.}$	E_{low}
41	5	37	41	4	38	A	0	173693.725	0.057	236.9450
41	4	37	41	3	38	E	1	173737.292	0.100	365.2695
41	5	37	41	4	38	E	1	173737.292	0.063	365.2695
48	17	31	48	16	32	A	0	173868.246	-0.027	360.4326
48	17	31	48	16	32	E	0	173876.548	-0.100	360.4353
48	17	32	48	16	33	E	0	173917.453	0.003	360.4220
28	6	23	27	5	22	E	0	173953.997	-0.020	152.6334
28	6	23	27	5	22	A	0	173965.970	0.001	152.6243
28	14	14	27	14	13	A	0	173970.549	-0.044	182.4518
28	14	15	27	14	14	A	0	173970.549	-0.041	182.4518
28	14	14	27	14	13	E	0	173971.455	0.080	182.4583
28	14	15	27	14	14	E	0	173972.369	0.077	182.4447
28	6	23	27	5	22	E	1	174015.890	0.069	280.9604
48	17	32	48	16	33	A	0	174021.528	-0.023	360.4269
32	16	16	32	15	17	E	0	174050.340	-0.039	219.0135
32	16	17	32	15	18	E	0	174057.970	0.023	219.0001
32	16	16	32	15	17	A	0	174096.326	-0.005	219.0082
57	18	40	57	17	41	E	0	174112.730	0.032	467.3353
38	2	36	38	1	37	A	1	174152.122	-0.210	331.9223
38	3	36	38	2	37	A	1	174152.122	-0.210	331.9223
57	18	40	57	17	41	A	0	174204.503	-0.036	467.3363
28	13	16	27	13	15	E	0	174372.496	-0.004	177.3774
43	5	38	43	4	39	A	1	174415.963	-0.784	387.3281
55	11	44	55	10	45	A	0	174432.136	-0.108	411.7956
55	11	44	55	10	45	E	0	174433.426	-0.055	411.7982
31	16	15	31	15	16	E	0	174443.229	-0.052	212.3689
31	16	16	31	15	17	E	0	174451.233	0.038	212.3555
31	16	15	31	15	16	A	0	174489.173	-0.017	212.3637
60	13	47	60	12	48	A	0	174613.473	-0.142	483.3647
60	13	47	60	12	48	E	0	174627.097	-0.036	483.3662
30	16	14	30	15	15	E	0	174798.103	-0.039	205.9383
30	16	15	30	15	16	E	0	174806.402	0.038	205.9248
30	16	14	30	15	15	A	0	174843.997	-0.012	205.9331
30	16	15	30	15	16	A	0	174843.997	-0.013	205.9331
28	12	17	27	12	16	A	0	174886.434	0.045	172.7189
28	12	16	27	12	15	A	0	174887.626	0.073	172.7189
28	12	16	27	12	15	E	0	174887.626	-0.117	172.7281
28	12	17	27	12	16	E	0	174889.641	-0.007	172.7149
62	15	48	62	14	49	A	0	174917.874	-0.165	514.9785
62	15	48	62	14	49	E	0	174921.913	-0.067	514.9796
38	2	36	38	1	37	E	0	174948.713	0.042	203.2781
38	3	36	38	2	37	E	0	174948.713	0.042	203.2781
38	2	36	38	1	37	A	0	174977.493	0.076	203.2678
38	3	36	38	2	37	A	0	174977.493	0.076	203.2678
55	12	44	55	11	45	E	0	174987.204	-0.008	411.8005
55	12	44	55	11	45	A	0	174988.427	-0.107	411.7979
17	5	12	16	4	13	E	0	174997.557	0.047	98.4232
17	5	12	16	4	13	A	0	174999.417	0.036	98.4126
43	5	38	43	4	39	E	0	175046.271	0.107	258.7800
43	6	38	43	5	39	E	0	175046.271	-0.087	258.7800
43	5	38	43	4	39	A	0	175066.572	0.123	258.7728
43	6	38	43	5	39	A	0	175066.572	-0.072	258.7728
43	5	38	43	4	39	E	1	175073.776	0.017	387.0958
43	6	38	43	5	39	E	1	175073.776	-0.191	387.0958
62	19	43	62	18	44	E	0	175085.263	-0.049	537.3233
38	2	36	38	1	37	E	1	175090.766	-0.034	331.5903
38	3	36	38	2	37	E	1	175090.766	-0.035	331.5903
29	16	13	29	15	14	E	0	175117.809	-0.112	199.7210
29	16	14	29	15	15	E	0	175126.447	0.026	199.7075
29	16	13	29	15	14	A	0	175163.756	0.008	199.7158
29	16	14	29	15	15	A	0	175163.756	0.008	199.7158
27	6	21	26	6	20	A	1	175165.891	0.083	277.8098
65	19	47	65	18	48	A	0	175185.261	-0.030	578.2735
47	17	30	47	16	31	E	0	175256.479	0.009	350.2943
47	17	30	47	16	31	A	0	175269.822	-0.016	350.2909
47	17	31	47	16	32	E	0	175271.745	-0.000	350.2818
27	8	19	26	8	18	A	0	175326.288	-0.028	152.8177
27	8	19	26	8	18	E	0	175332.283	0.013	152.8260
47	17	31	47	16	32	A	0	175352.350	0.009	350.2878
29	5	24	28	6	23	A	1	175381.314	0.003	287.0472
27	6	21	26	6	20	E	1	175384.356	-0.002	277.4972
27	6	21	26	6	20	E	0	175393.960	-0.087	149.1783
27	6	21	26	6	20	A	0	175401.727	0.009	149.1685
28	16	12	28	15	13	E	0	175405.266	-0.089	193.7164
28	16	13	28	15	14	E	0	175414.109	0.007	193.7029
29	5	24	28	6	23	E	1	175440.834	-0.075	286.7649
28	16	12	28	15	13	A	0	175451.067	-0.076	193.7113
28	16	13	28	15	14	A	0	175451.067	-0.076	193.7113
28	7	22	27	7	21	E	0	175454.326	-0.037	155.5469
28	7	22	27	7	21	A	0	175456.822	-0.049	155.5384
29	5	24	28	6	23	E	0	175484.634	-0.036	158.4359
29	5	24	28	6	23	A	0	175485.745	0.038	158.4271
28	11	18	27	11	17	A	0	175570.941	0.036	168.4692
28	11	17	27	11	16	E	0	175580.484	-0.008	168.4798
28	11	18	27	11	17	E	0	175582.310	0.022	168.4671
28	11	17	27	11	16	A	0	175587.664	-0.019	168.4697
27	16	11	27	15	12	E	0	175662.859	-0.127	187.9239
27	16	12	27	15	13	E	0	175671.980	0.027	187.9104
27	16	11	27	15	12	A	0	175708.732	-0.005	187.9188
27	16	12	27	15	13	A	0	175708.732	-0.005	187.9188
26	16	10	26	15	11	E	0	175893.138	-0.041	182.3430
26	16	11	26	15	12	E	0	175902.412	0.069	182.3295
26	16	10	26	15	11	A	0	175938.899	0.001	182.3379
26	16	11	26	15	12	A	0	175938.899	0.001	182.3379
53	10	43	53	9	44	E	0	175984.458	0.050	383.7170
53	10	43	53	9	44	A	0	175988.060	-0.032	383.7138
45	6	39	45	5	40	E	1	176069.983	-0.088	409.9860
45	7	39	45	6	40	E	1	176071.089	-0.009	409.9860
25	16	9	25	15	10	E	0	176098.090	-0.057	176.9732
45	6	39	45	5	40	A	0	176101.019	-0.056	281.6658
45	7	39	45	6	40	A	0	176102.096	0.061	281.6658
25	16	10	25	15	11	E	0	176107.511	0.027	176.9596
56	18	39	56	17	40	A	0	176122.247	-0.030	455.1782
25	16	9	25	15	10	A	0	176143.840	0.007	176.9681
25	16	10	25	15	11	A	0	176143.840	0.007	176.9681
53	11	43	53	10	44	E	0	176170.099	-0.041	383.7176
53	11	43	53	10	44	A	0	176174.713	-0.028	383.7145
40	3	37	40	2	38	A	1	176201.271	0.077	352.0673
40	4	37	40	3	38	A	1	176201.271	0.075	352.0673
11	10	2	10	9	2	E	0	176229.970	0.030	93.9842
30	4	26	29	5	25	A	1	176232.604	0.014	289.4962
11	10	1	10	9	1	E	0	176251.692	-0.050	93.9961
11	10	1	10	9	2	A	0	176277.444	0.012	93.9841
11	10	2	10	9	1	A	0	176277.444	0.012	93.9841
24	16	8	24	15	9	E	0	176279.912	-0.042	171.8138
24	16	9	24	15	10	E	0	176289.522	0.078	171.8003

J'	K'_a	K'_c	J''	K''_a	K''_c	Sym.	$v_{t_{CH_3}}$	$\nu_{Obs.}$	$\nu_{Obs.} - \nu_{Calc.}$	E_{low}
29	6	24	28	6	23	A	1	176301.778	0.051	287.0472
31	3	28	30	3	27	A	1	176307.461	-0.096	291.4485
31	4	28	30	3	27	A	1	176308.572	-0.154	291.4485
30	4	26	29	4	25	A	1	176310.291	-0.013	289.4936
24	16	8	24	15	9	A	0	176325.586	-0.025	171.8088
24	16	9	24	15	10	A	0	176325.586	-0.025	171.8088
30	4	26	29	5	25	E	0	176372.732	-0.011	160.8789
30	4	26	29	5	25	A	0	176376.408	0.003	160.8700
30	5	26	29	5	25	E	1	176400.564	-0.016	289.2087
31	3	28	30	4	27	E	1	176401.928	-0.015	291.1496
31	4	28	30	4	27	E	1	176403.278	-0.002	291.1496
31	3	28	30	3	27	E	1	176404.566	0.027	291.1495
31	4	28	30	3	27	E	1	176405.840	-0.036	291.1495
30	5	26	29	5	25	E	0	176418.216	0.021	160.8789
30	5	26	29	5	25	A	0	176422.088	0.045	160.8700
31	3	28	30	4	27	E	0	176426.628	-0.005	162.8212
31	4	28	30	4	27	E	0	176427.869	-0.035	162.8212
31	3	28	30	4	27	A	0	176429.533	-0.066	162.8119
31	4	28	30	3	27	E	0	176430.527	0.149	162.8211
31	3	28	30	3	27	A	0	176432.114	0.028	162.8118
31	4	28	30	3	27	A	0	176433.347	-0.016	162.8118
30	4	26	29	4	25	E	1	176439.546	-0.133	289.2058
23	16	7	23	15	8	E	0	176440.542	0.002	166.8645
32	2	30	31	2	29	A	1	176446.436	-0.027	292.9415
32	2	30	31	3	29	A	1	176446.436	0.013	292.9415
32	3	30	31	2	29	A	1	176446.436	-0.046	292.9415
32	3	30	31	3	29	A	1	176446.436	-0.006	292.9415
23	16	8	23	15	9	E	0	176450.241	0.076	166.8509
30	4	26	29	4	25	E	0	176455.901	-0.007	160.8762
29	6	24	28	6	23	A	0	176464.701	0.034	158.4271
28	10	19	27	10	18	A	0	176470.845	-0.059	164.6488
23	16	7	23	15	8	A	0	176486.112	-0.057	166.8595
23	16	8	23	15	9	A	0	176486.112	-0.057	166.8595
30	5	26	29	4	25	E	0	176501.358	-0.001	160.8762
30	5	26	29	4	25	A	0	176505.477	-0.055	160.8672
46	17	29	46	16	30	E	0	176506.585	0.027	340.3812
28	10	19	27	10	18	E	0	176510.815	-0.046	164.6506
32	2	30	31	2	29	E	1	176515.688	-0.059	292.6249
32	2	30	31	3	29	E	1	176515.688	-0.013	292.6249
32	3	30	31	2	29	E	1	176515.688	-0.082	292.6249
32	3	30	31	3	29	E	1	176515.688	-0.036	292.6249
46	17	29	46	16	30	A	0	176535.221	0.019	340.3773
32	2	30	31	2	29	E	0	176546.482	-0.021	164.2997
32	2	30	31	3	29	E	0	176546.482	0.023	164.2997
32	3	30	31	2	29	E	0	176546.482	-0.043	164.2997
32	3	30	31	3	29	E	0	176546.482	0.001	164.2997
32	2	30	31	2	29	A	0	176548.601	-0.028	164.2898
32	2	30	31	3	29	A	0	176548.601	0.016	164.2898
32	3	30	31	2	29	A	0	176548.601	-0.050	164.2898
32	3	30	31	3	29	A	0	176548.601	-0.006	164.2898
46	17	30	46	16	31	A	0	176578.606	-0.047	340.3757
22	16	6	22	15	7	E	0	176581.672	-0.053	162.1248
22	16	7	22	15	8	E	0	176591.476	0.010	162.1112
28	10	18	27	10	17	E	0	176618.864	0.047	164.6639
22	16	6	22	15	7	A	0	176627.247	-0.079	162.1198
22	16	7	22	15	8	A	0	176627.247	-0.079	162.1198
33	1	32	32	1	31	A	1	176638.959	0.015	294.0045
33	1	32	32	2	31	A	1	176638.959	0.015	294.0045
33	2	32	32	1	31	A	1	176638.959	0.015	294.0045
33	2	32	32	2	31	A	1	176638.959	0.015	294.0045
28	10	18	27	10	17	A	0	176653.415	-0.002	164.6549
33	1	32	32	1	31	E	1	176680.382	-0.031	293.6643
33	1	32	32	2	31	E	1	176680.382	-0.031	293.6643
33	2	32	32	1	31	E	1	176680.382	-0.032	293.6643
33	2	32	32	2	31	E	1	176680.382	-0.031	293.6643
51	10	42	51	9	43	E	1	176688.390	0.186	484.9521
47	7	40	47	6	41	E	1	176693.636	-0.019	433.9314
47	8	40	47	7	41	E	1	176698.100	-0.016	433.9314
21	16	5	21	15	6	E	0	176705.152	-0.063	157.5942
21	16	6	21	15	7	E	0	176715.099	0.039	157.5806
33	1	32	32	1	31	E	0	176717.151	0.012	165.3440
33	2	32	32	2	31	E	0	176717.151	0.012	165.3440
33	1	32	32	1	31	A	0	176718.376	-0.036	165.3335
33	1	32	32	2	31	A	0	176718.376	-0.036	165.3335
33	2	32	32	2	31	A	0	176718.376	-0.036	165.3335
47	7	40	47	6	41	A	0	176765.326	-0.014	305.6154
47	8	40	47	7	41	A	0	176769.505	-0.006	305.6154
63	14	49	63	13	50	E	0	176780.181	-0.008	528.0523
51	9	42	51	8	43	E	0	176782.662	-0.023	356.6530
51	9	42	51	8	43	A	0	176790.338	-0.024	356.6492
20	16	4	20	15	5	E	0	176812.518	-0.105	153.2724
51	10	42	51	9	43	A	0	176847.914	-0.043	356.6493
34	0	34	33	0	33	A	1	176853.698	0.047	294.6562
34	0	34	33	1	33	A	1	176853.698	0.047	294.6562
34	1	34	33	0	33	A	1	176853.698	0.047	294.6562
34	1	34	33	1	33	A	1	176853.698	0.047	294.6562
20	16	4	20	15	5	A	0	176858.175	-0.001	153.2674
20	16	5	20	15	6	A	0	176858.175	-0.001	153.2674
34	0	34	33	0	33	E	1	176865.536	-0.026	294.2864
34	0	34	33	1	33	E	1	176865.536	-0.026	294.2864
34	1	34	33	0	33	E	1	176865.536	-0.026	294.2864
34	1	34	33	1	33	E	1	176865.536	-0.026	294.2864
17	7	11	16	6	10	A	0	176885.328	-0.005	102.3284
49	8	41	49	7	42	E	1	176900.678	0.082	458.9232
19	16	3	19	15	4	E	0	176905.446	-0.012	149.1589
34	0	34	33	0	33	A	0	176908.856	-0.177	165.9613
34	0	34	33	1	33	A	0	176908.856	-0.177	165.9613
34	1	34	33	0	33	A	0	176908.856	-0.177	165.9613
34	1	34	33	1	33	A	0	176908.856	-0.177	165.9613
34	0	34	33	0	33	E	0	176908.856	0.194	165.9728
34	1	34	33	1	33	E	0	176908.856	0.194	165.9728
19	16	4	19	15	5	E	0	176915.504	0.037	149.1453
49	9	41	49	8	42	E	1	176918.056	0.083	458.9232
29	5	24	28	5	23	A	1	176929.331	0.029	286.9955
40	3	37	40	2	38	A	0	176977.094	0.124	223.4477
40	4	37	40	3	38	A	0	176977.094	0.121	223.4477
18	16	2	18	15	3	E	0	176985.076	-0.068	145.2533
49	8	41	49	7	42	E	0	177004.781	-0.031	330.6176
49	8	41	49	7	42	A	0	177015.982	-0.013	330.6130
49	9	41	49	8	42	E	0	177020.961	-0.029	330.6177
40	3	37	40	2	38	E	1	177057.293	-0.076	351.7711
40	4	37	40	3	38	E	1	177057.293	-0.078	351.7711
13	9	5	12	8	5	E	0	177059.439	0.006	95.4843
60	14	47	60	13	48	A	0	177097.809	-0.142	483.3780
60	14	47	60	13	48	E	0	177101.287	-0.056	483.3795
13	9	4	12	8	5	A	0	177106.971	-0.002	95.4826

J'	K'_a	K'_c	J''	K''_a	K''_c	Sym.	$v_{t_{CH_3}}$	$\nu_{Obs.}$	$\nu_{Obs.} - \nu_{Calc.}$	E_{low}
29	5	24	28	5	23	E	0	177119.657	-0.028	158.3814
29	5	24	28	5	23	E	1	177124.903	-0.234	286.7088
29	5	24	28	5	23	A	0	177125.938	0.053	158.3724
33	8	25	32	9	24	A	0	177393.555	-0.024	193.0397
28	9	20	27	9	19	A	0	177414.814	-0.006	161.2700
28	9	20	27	9	19	E	0	177423.427	-0.092	161.2762
33	8	25	32	9	24	E	0	177453.313	0.099	193.0466
28	8	21	27	8	20	A	0	177503.455	-0.034	158.2935
28	8	21	27	8	20	E	0	177504.417	0.003	158.3012
45	17	28	45	16	29	E	0	177645.980	-0.075	330.6942
45	17	29	45	16	30	E	0	177653.170	-0.005	330.6817
45	17	28	45	16	29	A	0	177683.960	0.037	330.6900
15	8	8	14	7	8	E	0	177694.301	-0.014	98.2368
45	17	29	45	16	30	A	0	177706.236	-0.071	330.6892
15	8	7	14	7	7	E	0	177725.922	0.012	98.2465
15	8	8	14	7	7	A	0	177734.190	-0.036	98.2335
15	8	7	14	7	8	A	0	177748.793	0.078	98.2331
29	6	24	28	5	23	A	1	177849.817	0.099	286.9955
55	18	38	55	17	39	A	0	177986.233	-0.029	443.2528
29	6	24	28	5	23	E	0	178095.334	-0.022	158.3814
29	6	24	28	5	23	A	0	178104.861	0.016	158.3724
17	7	10	16	6	11	A	0	178229.663	0.025	102.2902
63	19	45	63	18	46	E	0	178411.449	-0.067	550.6044
65	16	50	65	15	51	A	0	178436.808	-0.097	561.1834
65	16	50	65	15	51	E	0	178440.970	-0.028	561.1841
63	19	45	63	18	46	A	0	178471.488	-0.051	550.6054
69	16	53	69	15	54	E	0	178543.291	0.004	624.0094
18	6	12	17	5	13	E	0	178563.638	0.008	103.8221
18	6	12	17	5	13	A	0	178574.475	0.010	103.8120
42	4	38	42	3	39	E	0	178669.888	0.014	244.7180
42	5	38	42	4	39	E	0	178669.888	-0.003	244.7180
44	17	27	44	16	28	E	0	178687.637	-0.106	321.2318
42	4	38	42	3	39	A	0	178693.624	0.060	244.7098
42	5	38	42	4	39	A	0	178693.624	0.043	244.7098
44	17	28	44	16	29	E	0	178695.179	-0.023	321.2193
31	7	24	30	8	23	A	0	178729.075	-0.037	176.6694
44	17	27	44	16	28	A	0	178730.728	0.021	321.2275
42	4	38	42	3	39	E	1	178739.117	-0.067	373.0328
42	5	38	42	4	39	E	1	178739.117	-0.085	373.0328
44	17	28	44	16	29	A	0	178741.921	-0.062	321.2271
31	7	24	30	8	23	E	0	178765.481	-0.039	176.6771
27	7	20	26	7	19	A	0	178766.630	0.081	150.8131
27	7	20	26	7	19	E	0	178767.950	0.047	150.8227
28	9	19	27	9	18	E	0	178792.070	0.009	161.3366
28	9	19	27	9	18	A	0	178793.236	-0.025	161.3293
61	19	42	61	18	43	E	0	179067.210	-0.069	523.9838
39	2	37	39	1	38	A	1	179076.099	-0.245	338.8405
39	3	37	39	2	38	A	1	179076.099	-0.245	338.8405
61	19	42	61	18	43	A	0	179098.144	-0.031	523.9846
29	19	11	28	19	10	A	0	179123.238	-0.182	219.4667
29	19	10	28	19	9	A	0	179123.238	-0.182	219.4667
29	19	11	28	19	10	E	0	179124.172	0.078	219.4543
54	18	36	54	17	37	A	0	179155.824	-0.013	431.5831
54	18	36	54	17	37	E	0	179196.097	-0.080	431.5829
29	18	11	28	18	10	A	0	179290.383	-0.134	212.4436
29	18	12	28	18	11	A	0	179290.383	-0.134	212.4436
29	18	12	28	18	11	E	0	179291.352	-0.001	212.4319
29	17	13	28	17	12	A	0	179488.054	-0.083	205.8086
29	17	12	28	17	11	A	0	179488.054	-0.083	205.8086
29	17	13	28	17	12	E	0	179489.064	-0.103	205.7979
54	18	37	54	17	38	E	0	179625.012	0.028	431.5571
43	17	26	43	16	27	E	0	179641.299	-0.073	311.9927
43	17	27	43	16	28	E	0	179649.595	0.067	311.9801
43	17	26	43	16	27	A	0	179686.979	-0.000	311.9885
43	17	27	43	16	28	A	0	179692.454	-0.077	311.9883
29	16	13	28	16	12	A	0	179724.711	-0.040	199.5637
29	16	14	28	16	13	A	0	179724.711	-0.040	199.5637
54	18	37	54	17	38	A	0	179765.668	-0.038	431.5600
28	6	22	27	6	21	A	1	179833.399	0.086	283.6527
39	2	37	39	1	38	E	0	179895.375	0.043	210.1992
39	3	37	39	2	38	E	0	179895.375	0.043	210.1992
39	2	37	39	1	38	A	0	179925.010	0.117	210.1889
39	3	37	39	2	38	A	0	179925.010	0.117	210.1889
29	15	15	28	15	14	A	0	180012.086	-0.061	193.7113
29	15	14	28	15	13	A	0	180012.086	-0.061	193.7113
29	15	14	28	15	13	E	0	180013.110	0.127	193.7164
29	15	15	28	15	14	E	0	180013.799	0.103	193.7029
39	2	37	39	1	38	E	1	180041.341	-0.038	338.5099
39	3	37	39	2	38	E	1	180041.341	-0.038	338.5099
28	6	22	27	6	21	E	0	180085.366	0.031	155.0288
44	5	39	44	4	40	E	0	180087.892	0.059	267.0531
44	6	39	44	5	40	E	0	180087.892	-0.041	267.0531
28	6	22	27	6	21	A	0	180094.422	-0.022	155.0192
44	5	39	44	4	40	A	0	180108.847	0.071	267.0460
44	6	39	44	5	40	A	0	180108.847	-0.030	267.0460
44	5	39	44	4	40	E	1	180117.847	-0.013	395.3675
44	6	39	44	5	40	E	1	180117.847	-0.121	395.3675
62	19	44	62	18	45	E	0	180351.323	-0.006	537.1121
29	14	16	28	14	15	A	0	180367.259	-0.011	188.2548
29	14	15	28	14	14	E	0	180368.227	0.090	188.2614
29	14	16	28	14	15	E	0	180369.197	0.014	188.2478
62	19	44	62	18	45	A	0	180419.500	-0.022	537.1134
42	17	25	42	16	26	E	0	180514.974	-0.039	302.9758
42	17	26	42	16	27	E	0	180523.894	-0.006	302.9632
42	17	25	42	16	26	A	0	180561.998	0.083	302.9716
42	17	26	42	16	27	A	0	180564.432	-0.152	302.9715
56	12	45	56	11	46	E	0	180634.426	-0.026	423.1554
56	12	45	56	11	46	A	0	180636.267	-0.103	423.1530
63	15	49	63	14	50	A	0	180685.254	-0.062	528.0744
63	15	49	63	14	50	E	0	180690.114	-0.030	528.0754
29	13	17	28	13	16	A	0	180815.510	0.013	183.1994
29	13	16	28	13	15	E	0	180816.567	0.121	183.2073
29	13	17	28	13	16	E	0	180817.899	-0.054	183.1939
29	7	23	28	7	22	A	1	180861.996	0.040	290.0001
30	5	25	29	6	24	A	1	180906.665	-0.011	292.9280
30	5	25	29	6	24	E	1	181000.960	-0.044	292.6506
29	7	23	28	7	22	E	0	181010.454	-0.077	161.3994
29	7	23	28	7	22	A	0	181013.752	-0.002	161.3910
29	7	23	28	7	22	E	1	181017.552	-0.055	289.7250
30	5	25	29	6	24	E	0	181034.729	0.005	164.3220
30	5	25	29	6	24	A	0	181037.148	-0.005	164.3134
53	18	35	53	17	36	A	0	181094.406	-0.043	420.1121
53	18	35	53	17	36	E	0	181128.617	-0.062	420.1125
41	3	38	41	2	39	A	1	181146.183	0.075	359.4918
41	4	38	41	3	39	A	1	181146.183	0.074	359.4918
41	17	24	41	16	25	E	0	181315.595	-0.023	294.1801

J'	K'_a	K'_c	J''	K''_a	K''_c	Sym.	$v_{t_{CH_3}}$	$\nu_{Obs.}$	$\nu_{Obs.} - \nu_{Calc.}$	E_{low}
41	17	25	41	16	26	E	0	181325.176	-0.016	294.1673
61	13	48	61	12	49	A	0	181349.502	-0.108	495.9387
31	4	27	30	5	26	A	1	181357.317	0.007	295.3761
54	10	44	54	9	45	E	1	181362.045	0.201	522.8415
41	17	24	41	16	25	A	0	181363.177	0.070	294.1759
41	17	25	41	16	26	A	0	181364.319	-0.040	294.1758
29	12	18	28	12	17	A	0	181396.299	0.024	178.5525
31	4	27	30	4	26	A	1	181399.654	0.016	295.3747
31	5	27	30	4	26	A	1	181422.471	0.013	295.3747
32	3	29	31	4	28	A	1	181424.919	0.002	297.3295
32	4	29	31	3	28	A	1	181426.692	0.012	297.3295
53	18	36	53	17	37	A	0	181442.989	-0.025	420.0990
30	6	25	29	6	24	A	1	181444.116	0.107	292.9280
31	4	27	30	5	26	E	1	181479.044	0.027	295.0928
31	4	27	30	5	26	E	0	181499.651	0.001	166.7636
31	4	27	30	5	26	A	0	181503.391	0.025	166.7548
31	5	27	30	5	26	E	1	181504.881	0.137	295.0928
32	3	29	31	4	28	E	1	181520.898	-0.062	297.0338
32	4	29	31	3	28	E	1	181523.058	0.078	297.0337
31	5	27	30	5	26	E	0	181524.260	0.024	166.7636
31	4	27	30	4	26	E	1	181526.437	-0.004	295.0912
31	4	27	30	4	26	E	0	181545.083	-0.018	166.7621
32	3	29	31	4	28	E	0	181547.304	0.006	168.7062
31	4	27	30	4	26	A	0	181549.125	0.121	166.7532
32	4	29	31	3	28	E	0	181549.125	-0.092	168.7062
32	3	29	31	4	28	A	0	181550.259	0.013	168.6970
54	10	44	54	9	45	E	0	181568.184	-0.047	394.5530
31	5	27	30	4	26	E	0	181569.670	-0.017	166.7621
33	2	31	32	2	30	A	1	181572.002	-0.028	298.8271
33	2	31	32	3	30	A	1	181572.002	-0.008	298.8271
33	3	31	32	2	30	A	1	181572.002	-0.037	298.8271
33	3	31	32	3	30	A	1	181572.002	-0.018	298.8271
30	6	25	29	6	24	E	0	181606.319	0.015	164.3220
30	6	25	29	6	24	A	0	181610.767	-0.013	164.3134
33	2	31	32	2	30	E	1	181640.959	-0.028	298.5128
33	2	31	32	3	30	E	1	181640.959	-0.006	298.5128
33	3	31	32	2	30	E	1	181640.959	-0.039	298.5128
33	3	31	32	3	30	E	1	181640.959	-0.017	298.5128
30	6	24	29	7	23	A	0	181649.516	0.003	167.4289
30	6	24	29	7	23	E	0	181659.602	-0.033	167.4373
33	2	31	32	2	30	E	0	181673.240	-0.012	170.1887
33	2	31	32	3	30	E	0	181673.240	0.009	170.1887
33	3	31	32	2	30	E	0	181673.240	-0.022	170.1887
33	3	31	32	3	30	E	0	181673.240	-0.001	170.1887
33	2	31	32	2	30	A	0	181675.354	-0.014	170.1789
33	2	31	32	3	30	A	0	181675.354	0.008	170.1789
33	3	31	32	2	30	A	0	181675.354	-0.024	170.1789
33	3	31	32	3	30	A	0	181675.354	-0.003	170.1789
18	7	12	17	6	11	E	0	181749.582	0.003	105.9384
34	1	33	33	1	32	A	1	181766.549	0.006	299.8965
34	1	33	33	2	32	A	1	181766.549	0.006	299.8965
34	2	33	33	1	32	A	1	181766.549	0.006	299.8965
34	2	33	33	2	32	A	1	181766.549	0.006	299.8965
34	1	33	33	1	32	E	1	181807.840	-0.025	299.5577
34	1	33	33	2	32	E	1	181807.840	-0.025	299.5577
34	2	33	33	1	32	E	1	181807.840	-0.026	299.5577
34	2	33	33	2	32	E	1	181807.840	-0.025	299.5577
18	7	12	17	6	11	A	0	181829.272	-0.008	105.9286
34	1	33	33	1	32	E	0	181846.022	0.018	171.2387
34	2	33	33	2	32	E	0	181846.022	0.018	171.2387
34	1	33	33	1	32	A	0	181847.242	-0.031	171.2281
34	1	33	33	2	32	A	0	181847.242	-0.030	171.2281
34	2	33	33	1	32	A	0	181847.242	-0.031	171.2281
34	2	33	33	2	32	A	0	181847.242	-0.030	171.2281
48	7	41	48	6	42	E	1	181868.278	-0.011	443.2229
48	8	41	48	7	42	E	1	181870.713	-0.008	443.2229
41	3	38	41	2	39	E	0	181916.659	0.049	230.8851
41	4	38	41	3	39	E	0	181916.659	0.048	230.8851
41	3	38	41	2	39	A	0	181943.659	0.010	230.8760
41	4	38	41	3	39	A	0	181943.659	0.008	230.8760
35	0	35	34	0	34	A	1	181981.800	0.036	300.5554
35	0	35	34	1	34	A	1	181981.800	0.036	300.5554
35	1	35	34	0	34	A	1	181981.800	0.036	300.5554
35	1	35	34	1	34	A	1	181981.800	0.036	300.5554
35	0	35	34	0	34	E	1	181993.547	-0.029	300.1860
35	0	35	34	1	34	E	1	181993.547	-0.029	300.1860
35	1	35	34	0	34	E	1	181993.547	-0.029	300.1860
35	1	35	34	1	34	E	1	181993.547	-0.029	300.1860
30	5	25	29	5	24	E	1	182009.331	-0.092	292.6170
30	5	25	29	5	24	E	0	182010.401	0.007	164.2895
30	5	25	29	5	24	A	0	182016.124	0.011	164.2807
41	3	38	41	2	39	E	1	182026.366	-0.106	359.1978
41	4	38	41	3	39	E	1	182026.366	-0.107	359.1978
52	9	43	52	8	44	E	1	182031.481	0.141	495.2695
35	0	35	34	0	34	A	0	182038.197	-0.207	171.8623
35	0	35	34	1	34	A	0	182038.197	-0.207	171.8623
35	1	35	34	0	34	A	0	182038.197	-0.207	171.8623
35	1	35	34	1	34	A	0	182038.197	-0.207	171.8623
35	0	35	34	0	34	E	0	182038.197	0.161	171.8738
35	1	35	34	1	34	E	0	182038.197	0.161	171.8738
40	17	23	40	16	24	E	0	182049.218	-0.071	285.6043
40	17	24	40	16	25	E	0	182059.509	0.018	285.5915
52	10	43	52	9	44	E	1	182066.728	0.150	495.2696
29	11	19	28	11	18	A	0	182166.783	-0.062	174.3256
50	8	42	50	7	43	E	1	182173.061	0.082	468.7268
52	9	43	52	8	44	E	0	182181.837	0.017	366.9724
29	11	19	28	11	18	E	0	182185.928	0.063	174.3239
29	11	18	28	11	17	E	0	182187.099	0.001	174.3365
52	9	43	52	8	44	A	0	182190.233	-0.052	366.9688
29	11	18	28	11	17	A	0	182201.358	-0.028	174.3267
50	9	42	50	8	43	A	0	182294.537	-0.047	340.4185
66	20	46	66	19	47	E	0	182356.423	-0.054	598.4889
12	10	3	11	9	3	E	0	182375.344	-0.034	96.2448
12	10	2	11	9	2	E	0	182397.181	-0.037	96.2567
12	10	2	11	9	3	A	0	182422.898	0.017	96.2447
12	10	3	11	9	2	A	0	182422.898	0.017	96.2447
61	19	43	61	18	44	A	0	182444.656	0.024	523.8532
30	6	25	29	5	24	E	0	182581.927	-0.049	164.2895
30	6	25	29	5	24	A	0	182589.722	-0.019	164.2807
30	6	25	29	5	24	E	1	182602.102	0.017	292.6170
39	17	22	39	16	23	E	0	182721.369	-0.072	277.2477
39	17	23	39	16	24	E	0	182732.206	-0.009	277.2349
39	17	22	39	16	23	A	0	182769.328	0.108	277.2437
52	18	34	52	17	35	A	0	182815.147	-0.003	408.8761
52	18	34	52	17	35	E	0	182830.167	-0.037	408.8773
52	18	35	52	17	36	E	0	182899.707	-0.003	408.8636

J'	K'_a	K'_c	J''	K''_a	K''_c	Sym.	$v_{t_{CH_3}}$	$\nu_{Obs.}$	$\nu_{Obs.} - \nu_{Calc.}$	E_{low}
61	14	48	61	13	49	A	0	182927.475	-0.132	495.9467
61	14	48	61	13	49	E	0	182930.925	-0.059	495.9480
43	4	39	43	3	40	A	1	182945.256	0.376	381.2257
43	5	39	43	4	40	A	1	182945.256	0.368	381.2257
29	10	20	28	10	19	A	0	183151.069	0.081	170.5353
14	9	6	13	8	6	E	0	183174.881	-0.001	98.1666
28	8	20	27	8	19	A	0	183180.205	-0.039	158.6659
29	10	20	28	10	19	E	0	183183.729	-0.024	170.5384
28	8	20	27	8	19	E	0	183186.808	0.000	158.6745
14	9	5	13	8	5	E	0	183201.658	-0.071	98.1776
14	9	5	13	8	6	A	0	183222.510	-0.030	98.1648
38	17	21	38	16	22	E	0	183336.889	-0.035	269.1094
38	17	22	38	16	23	E	0	183348.214	-0.005	269.0965
38	17	21	38	16	22	A	0	183384.713	0.029	269.1054
29	10	19	28	10	18	E	0	183462.149	-0.004	170.5553
29	10	19	28	10	18	A	0	183488.749	-0.011	170.5475
43	4	39	43	3	40	E	0	183664.485	-0.003	252.6536
43	5	39	43	4	40	E	0	183664.485	-0.011	252.6536
29	8	22	28	8	21	A	0	183684.653	-0.043	164.2143
29	8	22	28	8	21	E	0	183684.653	0.023	164.2221
43	4	39	43	3	40	A	0	183688.930	0.066	252.6456
43	5	39	43	4	40	A	0	183688.930	0.057	252.6456
16	8	9	15	7	9	E	0	183701.141	-0.023	101.3535
16	8	9	15	7	8	A	0	183728.061	-0.024	101.3506
16	8	8	15	7	8	E	0	183731.388	-0.020	101.3633
43	4	39	43	3	40	E	1	183736.312	-0.071	380.9671
43	5	39	43	4	40	E	1	183736.312	-0.080	380.9671
16	8	8	15	7	9	A	0	183767.372	0.060	101.3494
37	17	20	37	16	21	E	0	183900.063	-0.045	261.1885
37	17	21	37	16	22	E	0	183911.960	0.087	261.1755
37	17	20	37	16	21	A	0	183947.817	0.003	261.1845
40	2	38	40	1	39	A	1	183999.013	-0.217	345.9298
40	3	38	40	2	39	A	1	183999.013	-0.217	345.9298
29	9	21	28	9	20	A	0	184029.813	-0.036	167.1879
29	9	21	28	9	20	E	0	184035.258	-0.001	167.1944
66	16	51	66	15	52	E	0	184136.855	-0.025	574.9745
59	12	47	59	11	48	A	0	184245.552	-0.103	464.8077
59	12	47	59	11	48	E	0	184248.995	-0.055	464.8094
29	6	23	28	6	22	A	1	184305.415	0.125	289.6513
51	18	33	51	17	34	E	0	184352.229	-0.017	397.8742
51	18	33	51	17	34	A	0	184359.317	-0.015	397.8722
60	19	42	60	18	43	E	0	184377.161	0.006	510.8241
51	18	34	51	17	35	E	0	184379.931	0.000	397.8619
36	17	19	36	16	20	E	0	184414.897	-0.053	253.4842
36	17	20	36	16	21	E	0	184427.117	-0.027	253.4712
36	17	20	36	16	21	A	0	184462.601	-0.009	253.4803
51	18	34	51	17	35	A	0	184466.395	-0.023	397.8683
60	19	42	60	18	43	A	0	184475.809	-0.025	510.8262
18	7	11	17	6	12	A	0	184485.934	-0.013	105.8545
18	7	11	17	6	12	E	0	184507.812	-0.034	105.8637
68	20	49	68	19	50	E	0	184541.160	-0.022	627.2019
29	6	23	28	6	22	E	0	184565.774	-0.006	161.0358
29	6	23	28	6	22	A	0	184575.451	0.014	161.0265
68	20	49	68	19	50	A	0	184592.862	-0.022	627.2031
29	6	23	28	6	22	E	1	184597.565	0.069	289.3550
40	2	38	40	1	39	E	0	184840.843	0.025	217.2914
40	3	38	40	2	39	E	0	184840.843	0.025	217.2914
35	17	18	35	16	19	E	0	184885.046	-0.014	245.9958
35	17	19	35	16	20	E	0	184897.639	-0.002	245.9828
35	17	18	35	16	19	A	0	184932.628	-0.005	245.9920
40	2	38	40	1	39	E	1	184990.662	-0.033	345.6005
40	3	38	40	2	39	E	1	184990.662	-0.033	345.6005
45	5	40	45	4	41	E	0	185122.002	0.044	275.4971
45	6	40	45	5	41	E	0	185122.002	-0.007	275.4971
45	5	40	45	4	41	A	0	185143.610	0.065	275.4900
45	6	40	45	5	41	A	0	185143.610	0.013	275.4900
45	5	40	45	4	41	E	1	185154.157	-0.026	403.8099
45	6	40	45	5	41	E	1	185154.157	-0.082	403.8099
30	20	10	29	20	9	A	0	185243.773	-0.085	232.8469
30	20	11	29	20	10	A	0	185243.773	-0.085	232.8469
30	20	10	29	20	9	E	0	185244.571	0.058	232.8441
34	17	17	34	16	18	E	0	185313.745	0.003	238.7226
28	7	21	27	7	20	E	0	185319.481	-0.131	156.7857
28	7	21	27	7	20	A	0	185320.818	-0.020	156.7761
34	17	18	34	16	19	E	0	185326.672	-0.003	238.7096
34	17	17	34	16	18	A	0	185361.237	-0.011	238.7188
30	19	12	29	19	11	A	0	185401.895	-0.100	225.4416
30	19	11	29	19	10	A	0	185401.895	-0.100	225.4416
30	19	12	29	19	11	E	0	185402.820	0.062	225.4293
30	18	12	29	18	11	A	0	185587.064	-0.109	218.4241
30	18	13	29	18	12	A	0	185587.064	-0.109	218.4241
30	18	13	29	18	12	E	0	185588.064	-0.052	218.4125
33	17	16	33	16	17	E	0	185703.973	-0.062	231.6640
33	17	17	33	16	18	E	0	185717.298	0.013	231.6509
50	18	32	50	17	33	E	0	185732.047	-0.075	387.1007
50	18	33	50	17	34	E	0	185747.023	-0.028	387.0888
33	17	17	33	16	18	A	0	185751.505	0.026	231.6602
33	17	16	33	16	17	A	0	185751.505	0.027	231.6602
50	18	32	50	17	33	A	0	185756.709	0.009	387.0982
57	11	46	57	10	47	E	1	185786.944	0.358	562.9534
30	17	14	29	17	13	A	0	185806.305	-0.093	211.7957
30	17	13	29	17	12	A	0	185806.305	-0.093	211.7957
30	17	14	29	17	13	E	0	185807.383	-0.172	211.7850
50	18	33	50	17	34	A	0	185814.246	-0.029	387.0961
67	15	52	67	14	53	E	0	186022.706	0.039	588.9063
57	11	46	57	10	47	E	0	186046.990	-0.016	434.6775
57	11	46	57	10	47	A	0	186048.462	-0.097	434.6753
32	17	15	32	16	16	E	0	186058.699	-0.047	224.8192
32	17	16	32	16	17	E	0	186072.338	0.054	224.8061
42	3	39	42	2	40	A	1	186088.842	0.086	367.0872
42	4	39	42	3	40	A	1	186088.842	0.086	367.0872
32	17	16	32	16	17	A	0	186106.086	-0.044	224.8155
32	17	15	32	16	16	A	0	186106.086	-0.043	224.8155
19	7	13	18	6	12	A	0	186127.484	-0.025	109.7686
29	9	20	28	9	19	A	0	186244.119	-0.037	167.2932
29	9	20	28	9	19	E	0	186247.478	0.095	167.3004
31	5	26	30	6	25	A	1	186252.559	0.001	298.9803
47	7	41	47	6	42	E	1	186259.211	-0.105	427.7185
47	6	41	47	5	42	E	0	186268.288	0.102	299.4076
47	7	41	47	6	42	E	0	186268.288	-0.165	299.4076
47	6	41	47	5	42	A	0	186286.975	0.111	299.4016
47	7	41	47	6	42	A	0	186286.975	-0.157	299.4016
36	9	27	35	10	26	E	0	186333.431	0.065	217.9781
31	5	26	30	6	25	E	1	186369.011	-0.069	298.7080
31	17	14	31	16	15	E	0	186380.410	-0.065	218.1877
30	15	16	29	15	15	A	0	186388.885	-0.059	199.7158

J'	K_a'	K_c'	J''	K_a''	K_c''	Sym.	$v_{t_{CH_3}}$	$\nu_{Obs.}$	$\nu_{Obs.} - \nu_{Calc.}$	E_{low}
30	15	15	29	15	14	A	0	186388.885	-0.060	199.7158
30	15	15	29	15	14	E	0	186389.914	0.050	199.7210
30	15	16	29	15	15	E	0	186390.737	0.059	199.7075
31	17	15	31	16	16	E	0	186394.277	0.005	218.1746
31	5	26	30	6	25	E	0	186396.558	-0.009	170.3797
31	5	26	30	6	25	A	0	186399.853	-0.023	170.3712
31	17	15	31	16	16	A	0	186427.829	0.026	218.1840
31	17	14	31	16	15	A	0	186427.829	0.026	218.1840
67	20	48	67	19	49	A	0	186427.829	-0.447	612.6106
30	7	24	29	7	23	E	0	186436.235	-0.079	167.4373
30	7	24	29	7	23	A	0	186440.136	-0.018	167.4289
59	19	41	59	18	42	A	0	186461.339	-0.046	498.0330
32	4	28	31	5	27	A	1	186474.067	-0.004	301.4263
32	5	28	31	5	27	A	1	186486.314	0.056	301.4263
32	5	28	31	4	27	A	1	186508.905	-0.172	301.4256
33	3	30	32	4	29	A	1	186545.154	-0.005	303.3812
33	4	30	32	3	29	A	1	186546.084	0.031	303.3812
64	15	50	64	14	51	A	0	186551.596	-0.065	541.3361
64	15	50	64	14	51	E	0	186556.834	-0.044	541.3368
31	6	26	30	6	25	A	1	186561.347	0.027	298.9803
32	4	28	31	5	27	E	1	186595.847	-0.043	301.1471
32	5	28	31	5	27	E	1	186609.619	-0.098	301.1471
32	4	28	31	5	27	E	0	186617.935	-0.001	172.8186
32	4	28	31	5	27	A	0	186621.650	-0.017	172.8099
32	5	28	31	5	27	E	0	186631.108	-0.004	172.8186
32	5	28	31	4	27	E	1	186635.438	-0.007	301.1462
33	3	30	32	4	29	E	1	186640.504	-0.062	303.0887
33	4	30	32	3	29	E	1	186641.503	-0.094	303.0887
32	4	28	31	4	27	E	0	186642.562	0.040	172.8178
32	4	28	31	4	27	A	0	186646.370	0.012	172.8091
32	5	28	31	4	27	E	0	186655.687	-0.010	172.8178
32	5	28	31	4	27	A	0	186659.588	-0.005	172.8091
65	20	45	65	19	46	E	0	186666.294	-0.109	584.2686
33	3	30	32	4	29	E	0	186668.535	-0.022	174.7620
33	4	30	32	3	29	E	0	186669.532	-0.003	174.7620
33	3	30	32	4	29	A	0	186671.496	0.009	174.7529
33	4	30	32	3	29	A	0	186672.493	0.024	174.7528
30	17	14	30	16	15	E	0	186685.713	0.042	211.7557
34	2	32	33	2	31	A	1	186697.758	0.007	304.8837
34	2	32	33	3	31	A	1	186697.758	0.016	304.8837
34	3	32	33	2	31	A	1	186697.758	0.002	304.8837
34	3	32	33	3	31	A	1	186697.758	0.011	304.8837
31	6	26	30	6	25	E	1	186711.835	-0.026	298.7080
30	17	14	30	16	15	A	0	186718.894	-0.021	211.7653
30	17	13	30	16	14	A	0	186718.894	-0.021	211.7653
31	6	26	30	6	25	E	0	186726.117	-0.017	170.3797
31	6	26	30	6	25	A	0	186730.707	0.018	170.3712
34	2	32	33	2	31	E	1	186766.381	-0.012	304.5717
34	2	32	33	3	31	E	1	186766.381	-0.001	304.5717
34	3	32	33	2	31	E	1	186766.381	-0.017	304.5717
34	3	32	33	3	31	E	1	186766.381	-0.006	304.5717
30	14	17	29	14	16	A	0	186784.880	-0.056	194.2712
30	14	16	29	14	15	E	0	186785.968	0.076	194.2778
30	14	17	29	14	16	E	0	186787.011	-0.069	194.2643
31	5	26	30	5	25	A	1	186789.927	0.037	298.9624
34	2	32	33	2	31	E	0	186800.144	-0.020	176.2486
34	2	32	33	3	31	E	0	186800.144	-0.009	176.2486
34	3	32	33	2	31	E	0	186800.144	-0.025	176.2486
34	3	32	33	3	31	E	0	186800.144	-0.014	176.2486
34	2	32	33	2	31	A	0	186802.253	-0.017	176.2389
34	2	32	33	3	31	A	0	186802.253	-0.007	176.2389
34	3	32	33	2	31	A	0	186802.253	-0.022	176.2389
34	3	32	33	3	31	A	0	186802.253	-0.012	176.2389
42	3	39	42	2	40	E	0	186880.184	0.022	238.4843
42	4	39	42	3	40	E	0	186880.184	0.022	238.4843
55	10	45	55	9	46	E	1	186885.178	0.246	533.8443
35	1	34	34	1	33	A	1	186894.091	0.011	305.9596
35	1	34	34	2	33	A	1	186894.091	0.011	305.9596
35	2	34	34	1	33	A	1	186894.091	0.011	305.9596
35	2	34	34	2	33	A	1	186894.091	0.011	305.9596
42	3	39	42	2	40	A	0	186908.032	0.093	238.4752
42	4	39	42	3	40	A	0	186908.032	0.093	238.4752
35	1	34	34	1	33	E	1	186935.247	-0.012	305.6222
35	1	34	34	2	33	E	1	186935.247	-0.012	305.6222
35	2	34	34	1	33	E	1	186935.247	-0.012	305.6222
35	2	34	34	2	33	E	1	186935.247	-0.012	305.6222
29	17	13	29	16	14	E	0	186948.744	0.006	205.5491
55	11	45	55	10	46	E	1	186953.526	0.305	533.8445
31	5	26	30	5	25	E	1	186961.766	0.024	298.6882
31	5	26	30	5	25	E	0	186968.037	-0.112	170.3607
31	5	26	30	5	25	A	0	186973.488	-0.015	170.3521
35	1	34	34	1	33	E	0	186974.825	0.016	177.3044
35	2	34	34	2	33	E	0	186974.825	0.016	177.3044
35	1	34	34	1	33	A	0	186976.049	-0.025	177.2939
35	1	34	34	2	33	A	0	186976.049	-0.025	177.2939
35	2	34	34	1	33	A	0	186976.049	-0.025	177.2939
35	2	34	34	2	33	A	0	186976.049	-0.025	177.2939
29	17	13	29	16	14	A	0	186981.702	-0.023	205.5587
29	17	12	29	16	13	A	0	186981.702	-0.023	205.5587
49	18	31	49	17	32	E	0	186993.003	-0.025	376.5549
49	18	32	49	17	33	E	0	187004.958	0.031	376.5430
49	7	42	49	6	43	E	1	187024.898	0.060	452.6847
49	8	42	49	7	43	E	1	187026.138	-0.017	452.6847
49	18	31	49	17	32	A	0	187028.924	0.005	376.5521
49	18	32	49	17	33	A	0	187059.192	-0.059	376.5510
31	6	26	30	5	25	A	1	187098.696	0.043	298.9624
36	0	36	35	0	35	A	1	187109.829	0.052	306.6257
36	0	36	35	1	35	A	1	187109.829	0.052	306.6257
36	1	36	35	0	35	A	1	187109.829	0.052	306.6257
36	1	36	35	1	35	A	1	187109.829	0.052	306.6257
36	0	36	35	0	35	E	1	187121.481	-0.010	306.2567
36	1	36	35	1	35	E	1	187121.481	-0.010	306.2567
36	0	36	35	1	35	A	0	187167.471	-0.204	177.9345
36	1	36	35	0	35	A	0	187167.471	-0.204	177.9345
36	1	36	35	1	35	A	0	187167.471	-0.204	177.9345
36	0	36	35	0	35	E	0	187167.471	0.161	177.9459
36	1	36	35	1	35	E	0	187167.471	0.161	177.9459
28	17	11	28	16	12	E	0	187171.127	-0.033	199.5673
28	17	12	28	16	13	A	0	187218.311	-0.028	199.5637
28	17	11	28	16	12	A	0	187218.311	-0.028	199.5637
70	16	54	70	15	55	E	0	187227.888	0.010	638.6845
30	13	17	29	13	16	E	0	187287.548	0.041	189.2387
30	13	18	29	13	17	E	0	187289.218	0.003	189.2253
31	6	26	30	5	25	E	0	187297.692	-0.024	170.3607
31	6	26	30	5	25	A	0	187304.379	0.063	170.3521
27	17	10	27	16	11	E	0	187383.559	-0.040	193.7834

J'	K'_a	K'_c	J''	K''_a	K''_c	Sym.	$v_{t_{CH_3}}$	$\nu_{Obs.}$	$\nu_{Obs.} - \nu_{Calc.}$	E_{low}
27	17	11	27	16	12	E	0	187398.278	0.087	193.7702
51	8	43	51	7	44	E	1	187418.223	0.156	478.7003
27	17	11	27	16	12	A	0	187430.720	-0.014	193.7798
27	17	10	27	16	11	A	0	187430.720	-0.014	193.7798
51	8	43	51	7	44	A	0	187528.373	-0.004	350.3939
51	9	43	51	8	44	A	0	187533.369	-0.028	350.3939
53	9	44	53	8	45	E	0	187539.589	-0.044	377.4613
53	9	44	53	8	45	A	0	187548.740	-0.066	377.4578
53	10	44	53	9	45	E	0	187558.185	-0.033	377.4614
53	10	44	53	9	45	A	0	187567.421	-0.074	377.4579
26	17	9	26	16	10	E	0	187573.630	-0.043	188.2102
26	17	10	26	16	11	E	0	187588.402	-0.011	188.1970
26	17	10	26	16	11	A	0	187620.805	0.037	188.2066
26	17	9	26	16	10	A	0	187620.805	0.037	188.2066
58	19	39	58	18	40	A	0	187627.153	-0.059	485.5017
11	11	1	10	10	1	E	0	187663.356	0.020	97.6043
11	11	0	10	10	0	E	0	187680.062	-0.040	97.6169
11	11	1	10	10	0	A	0	187710.257	0.014	97.6057
11	11	0	10	10	1	A	0	187710.257	0.014	97.6057
25	17	8	25	16	9	E	0	187743.097	-0.031	182.8472
25	17	9	25	16	10	E	0	187758.033	0.034	182.8339
62	13	49	62	12	50	A	0	187779.734	-0.116	508.6771
62	13	49	62	12	50	E	0	187787.309	-0.051	508.6782
25	17	9	25	16	10	A	0	187790.177	-0.007	182.8436
25	17	8	25	16	9	A	0	187790.177	-0.007	182.8436
24	17	7	24	16	8	E	0	187893.550	-0.059	177.6939
24	17	8	24	16	9	E	0	187908.605	0.009	177.6806
30	12	19	29	12	18	A	0	187938.545	0.064	184.6032
24	17	8	24	16	9	A	0	187940.646	0.015	177.6904
24	17	7	24	16	8	A	0	187940.646	0.015	177.6904
30	12	18	29	12	17	E	0	187942.391	0.043	184.6125
23	17	6	23	16	7	E	0	188026.710	0.041	172.7499
23	17	7	23	16	8	E	0	188041.807	0.050	172.7367
23	17	7	23	16	8	A	0	188073.658	0.001	172.7464
23	17	6	23	16	7	A	0	188073.658	0.001	172.7464
22	17	5	22	16	6	E	0	188143.679	-0.092	168.0149
48	18	30	48	17	31	E	0	188149.696	-0.055	366.2352
22	17	6	22	16	7	E	0	188158.978	0.030	168.0017
48	18	31	48	17	32	E	0	188161.394	-0.094	366.2232
22	17	6	22	16	7	A	0	188190.745	0.016	168.0114
22	17	5	22	16	6	A	0	188190.745	0.016	168.0114
48	18	30	48	17	31	A	0	188192.244	0.058	366.2322
48	18	31	48	17	32	A	0	188207.782	-0.056	366.2317
21	17	4	21	16	5	E	0	188246.241	-0.058	163.4885
21	17	5	21	16	6	E	0	188261.705	0.152	163.4752
21	17	5	21	16	6	A	0	188293.224	-0.004	163.4850
21	17	4	21	16	5	A	0	188293.224	-0.004	163.4850
20	17	3	20	16	4	E	0	188335.507	-0.050	159.1702
58	19	40	58	18	41	A	0	188366.691	-0.024	485.4735
20	17	4	20	16	5	A	0	188382.447	-0.012	159.1668
20	17	3	20	16	4	A	0	188382.447	-0.012	159.1668
66	20	47	66	19	48	E	0	188391.247	-0.003	598.2459
19	17	3	19	16	4	A	0	188459.666	0.008	155.0564
19	17	2	19	16	3	A	0	188459.666	0.008	155.0564
18	17	1	18	16	2	E	0	188479.081	-0.048	151.1569
18	17	2	18	16	3	E	0	188494.565	0.012	151.1437
13	10	4	12	9	4	E	0	188515.787	-0.131	98.7133
18	17	2	18	16	3	A	0	188525.990	0.007	151.1535
18	17	1	18	16	2	A	0	188525.990	0.007	151.1535
13	10	3	12	9	3	E	0	188537.771	-0.038	98.7252
13	10	3	12	9	4	A	0	188563.450	0.008	98.7132
31	6	25	30	7	24	E	1	188632.978	-0.218	301.9818
44	4	40	44	3	41	E	0	188654.897	0.014	260.7603
44	5	40	44	4	41	E	0	188654.897	0.010	260.7603
19	6	13	18	5	14	E	0	188668.219	0.030	107.5722
19	6	13	18	5	14	A	0	188676.008	0.075	107.5622
44	4	40	44	3	41	A	0	188680.006	0.070	260.7523
44	5	40	44	4	41	A	0	188680.006	0.065	260.7523
31	6	25	30	7	24	A	0	188733.361	-0.007	173.6479
31	6	25	30	7	24	E	0	188738.593	0.017	173.6561
62	14	49	62	13	50	A	0	188767.520	-0.116	508.6819
62	14	49	62	13	50	E	0	188770.689	-0.051	508.6829
30	11	20	29	11	19	A	0	188800.825	0.004	180.4021
30	11	20	29	11	19	E	0	188830.668	0.010	180.4010
30	11	19	29	11	18	E	0	188844.963	0.021	180.4136
30	11	19	29	11	18	A	0	188869.415	-0.022	180.4043
30	6	24	29	6	23	E	0	189004.143	-0.022	167.1923
30	6	24	29	6	23	A	0	189013.613	0.021	167.1833
30	6	24	29	6	23	E	1	189037.067	0.061	295.5125
47	18	29	47	17	30	E	0	189213.002	-0.019	356.1402
47	18	30	47	17	31	E	0	189225.267	-0.080	356.1282
47	18	29	47	17	30	A	0	189259.022	0.049	356.1373
47	18	30	47	17	31	A	0	189266.782	-0.100	356.1370
15	9	7	14	8	7	E	0	189272.668	-0.030	101.0595
15	9	6	14	8	6	E	0	189299.682	-0.031	101.0704
15	9	7	14	8	6	A	0	189320.394	0.257	101.0577
15	9	6	14	8	7	A	0	189320.394	-0.235	101.0577
32	7	25	31	8	24	A	0	189430.283	0.003	183.1928
32	7	25	31	8	24	E	0	189457.872	-0.006	183.2005
20	7	14	19	6	13	E	0	189511.766	-0.050	113.8655
20	7	14	19	6	13	A	0	189560.630	0.059	113.8557
59	18	41	58	19	40	A	0	189597.434	-0.079	491.7568
17	8	10	16	7	9	A	0	189653.009	0.002	104.6838
17	8	10	16	7	10	E	0	189660.387	-0.033	104.6857
17	8	9	16	7	9	E	0	189680.749	-0.033	104.6958
30	8	23	29	8	22	E	0	189707.301	-0.026	170.3492
30	8	23	29	8	22	A	0	189708.390	0.039	170.3414
57	19	38	57	18	39	A	0	189742.264	-0.032	473.1634
17	8	9	16	7	10	A	0	189751.224	0.040	104.6809
57	19	38	57	18	39	E	0	189775.006	-0.164	473.1626
41	2	39	41	1	40	E	0	189785.263	0.055	224.5546
41	3	39	41	2	40	E	0	189785.263	0.055	224.5546
41	2	39	41	1	40	A	0	189816.487	0.101	224.5444
41	3	39	41	2	40	A	0	189816.487	0.101	224.5444
30	10	21	29	10	20	A	0	189851.800	-0.020	176.6445
30	10	21	29	10	20	E	0	189872.853	-0.011	176.6487
60	12	48	60	11	49	E	1	189878.832	0.641	605.2828
41	2	39	41	1	40	E	1	189938.739	-0.087	352.8621
41	3	39	41	2	40	E	1	189938.739	-0.087	352.8621
67	16	52	67	15	53	A	0	189989.069	-0.119	588.9301
57	19	39	57	18	40	E	0	190041.175	0.019	473.1430
46	5	41	46	4	42	E	0	190149.249	0.009	284.1118
46	6	41	46	5	42	E	0	190149.249	-0.017	284.1118
46	5	41	46	4	42	A	0	190171.414	-0.040	284.1049
46	6	41	46	5	42	A	0	190171.414	-0.066	284.1049
46	5	41	46	4	42	E	1	190183.416	-0.021	412.4231

J'	K'_a	K'_c	J''	K''_a	K''_c	Sym.	$v_{t_{CH_3}}$	$\nu_{Obs.}$	$\nu_{Obs.} - \nu_{Calc.}$	E_{low}
46	6	41	46	5	42	E	1	190183.416	-0.049	412.4231
46	18	28	46	17	29	E	0	190191.433	-0.042	346.2688
46	18	29	46	17	30	E	0	190204.526	-0.009	346.2567
46	18	28	46	17	29	A	0	190239.267	0.065	346.2659
46	18	29	46	17	30	A	0	190243.025	-0.090	346.2657
64	20	44	64	19	45	E	0	190275.455	-0.063	570.3132
64	20	44	64	19	45	A	0	190296.886	-0.067	570.3149
30	10	20	29	10	19	E	0	190439.540	0.009	176.6749
30	10	20	29	10	19	A	0	190453.500	0.028	176.6680
65	20	46	65	19	47	E	0	190502.580	-0.013	584.1149
65	20	46	65	19	47	A	0	190574.090	-0.026	584.1171
30	9	22	29	9	21	A	0	190581.219	0.024	173.3265
30	9	22	29	9	21	E	0	190584.682	0.011	173.3331
65	14	51	65	13	52	A	0	190841.593	-0.100	554.7551
29	8	21	28	8	20	A	0	190949.372	0.013	164.7762
29	8	21	28	8	20	E	0	190955.226	-0.019	164.7849
27	7	21	26	6	20	A	0	190965.125	0.073	149.1685
28	7	22	27	6	21	E	0	190985.072	-0.043	155.0288
34	8	26	33	9	25	A	0	191036.519	-0.018	200.0297
34	8	26	33	9	25	E	0	191088.036	0.027	200.0366
45	18	27	45	17	28	E	0	191092.400	-0.053	336.6198
45	18	28	45	17	29	E	0	191106.256	0.024	336.6076
45	18	28	45	17	29	A	0	191142.763	-0.143	336.6168
29	7	22	28	7	21	E	0	191319.649	0.075	162.9673
29	7	22	28	7	21	A	0	191323.602	-0.032	162.9577
48	6	42	48	5	43	E	1	191336.735	0.047	436.8406
48	7	42	48	6	43	E	1	191336.735	-0.105	436.8406
48	6	42	48	5	43	E	0	191344.160	0.066	308.5314
48	7	42	48	6	43	E	0	191344.160	-0.074	308.5314
48	6	42	48	5	43	A	0	191363.433	0.066	308.5255
48	7	42	48	6	43	A	0	191363.433	-0.075	308.5255
31	21	11	30	21	10	A	0	191366.119	-0.044	246.8131
31	21	10	30	21	9	A	0	191366.119	-0.044	246.8131
31	21	10	30	21	9	E	0	191366.778	-0.044	246.8088
32	5	27	31	6	26	A	1	191489.425	-0.017	305.2033
26	7	20	25	6	19	E	0	191498.448	-0.080	143.4969
31	20	11	30	20	10	A	0	191516.482	-0.107	239.0260
31	20	12	30	20	11	A	0	191516.482	-0.107	239.0260
31	20	11	30	20	10	E	0	191517.399	0.085	239.0232
26	7	20	25	6	19	A	0	191542.634	0.012	143.4869
31	7	25	30	7	24	A	1	191584.544	0.069	302.2465
33	4	29	32	5	28	A	1	191587.500	0.028	307.6468
33	5	29	32	5	28	A	1	191593.934	0.009	307.6468
33	4	29	32	4	28	A	1	191599.624	-0.034	307.6464
33	5	29	32	4	28	A	1	191606.209	0.097	307.6464
32	5	27	31	6	26	E	1	191619.333	-0.022	304.9360
56	19	37	56	18	38	A	0	191620.766	-0.062	461.0621
56	19	37	56	18	38	E	0	191640.619	-0.025	461.0619
32	5	27	31	6	26	E	0	191643.504	-0.005	176.6082
32	5	27	31	6	26	A	0	191647.334	-0.013	176.5999
32	6	27	31	6	26	A	1	191664.384	-0.026	305.2033
34	3	31	33	3	30	A	1	191666.234	-0.082	309.6037
34	3	31	33	4	30	A	1	191666.234	0.218	309.6037
34	4	31	33	3	30	A	1	191666.234	-0.233	309.6037
34	4	31	33	4	30	A	1	191666.234	0.067	309.6037
31	19	13	30	19	12	A	0	191691.083	-0.093	231.6260
31	19	12	30	19	11	A	0	191691.083	-0.093	231.6260
31	19	13	30	19	12	E	0	191692.075	0.040	231.6136
58	11	47	58	10	48	E	0	191701.791	0.043	446.3690
58	11	47	58	10	48	A	0	191704.211	-0.116	446.3670
33	4	29	32	5	28	E	1	191708.799	-0.022	307.3717
33	5	29	32	5	28	E	1	191716.169	-0.021	307.3717
33	4	29	32	4	28	E	1	191722.738	0.090	307.3713
33	5	29	32	4	28	E	1	191729.986	-0.030	307.3713
33	4	29	32	5	28	E	0	191732.513	0.020	179.0439
33	4	29	32	5	28	A	0	191736.220	0.004	179.0354
33	5	29	32	5	28	E	0	191739.478	-0.015	179.0439
33	5	29	32	5	28	A	0	191743.218	-0.032	179.0354
33	4	29	32	4	28	E	0	191745.651	-0.017	179.0435
33	4	29	32	4	28	A	0	191749.344	-0.107	179.0349
56	19	38	56	18	39	E	0	191750.456	0.163	461.0477
33	5	29	32	4	28	E	0	191752.663	-0.006	179.0435
31	7	25	30	7	24	E	0	191754.172	-0.022	173.6561
33	5	29	32	4	28	A	0	191756.474	-0.011	179.0349
31	7	25	30	7	24	A	0	191758.523	-0.008	173.6479
34	3	31	33	3	30	E	1	191761.018	-0.124	309.3143
34	3	31	33	4	30	E	1	191761.018	0.224	309.3143
34	4	31	33	3	30	E	1	191761.018	-0.300	309.3143
34	4	31	33	4	30	E	1	191761.018	0.048	309.3143
34	3	31	33	3	30	E	0	191790.677	-0.091	180.9886
34	3	31	33	4	30	E	0	191790.677	0.237	180.9886
34	4	31	33	3	30	E	0	191790.677	-0.257	180.9886
34	4	31	33	4	30	E	0	191790.677	0.072	180.9886
34	3	31	33	4	30	A	0	191793.587	0.237	180.9796
34	4	31	33	4	30	A	0	191793.587	0.070	180.9796
34	3	31	33	3	30	A	0	191793.588	-0.093	180.9796
34	4	31	33	3	30	A	0	191793.588	-0.260	180.9796
32	5	27	31	5	26	A	1	191798.272	0.068	305.1930
32	6	27	31	6	26	E	1	191814.849	0.001	304.9360
35	2	33	34	2	32	A	1	191823.575	-0.010	311.1113
35	2	33	34	3	32	A	1	191823.575	-0.006	311.1113
35	3	33	34	2	32	A	1	191823.575	-0.013	311.1113
35	3	33	34	3	32	A	1	191823.575	-0.008	311.1113
32	6	27	31	6	26	A	0	191835.527	0.045	176.5999
43	3	40	43	2	41	E	0	191841.525	0.011	246.2545
43	4	40	43	3	41	E	0	191841.525	0.010	246.2545
56	19	38	56	18	39	A	0	191864.791	-0.010	461.0530
43	3	40	43	2	41	A	0	191870.114	0.091	246.2455
43	4	40	43	3	41	A	0	191870.114	0.091	246.2455
35	2	33	34	2	32	E	1	191891.901	-0.022	310.8015
35	2	33	34	3	32	E	1	191891.901	-0.016	310.8015
35	3	33	34	2	32	E	1	191891.901	-0.024	310.8015
35	3	33	34	3	32	E	1	191891.901	-0.019	310.8015
31	18	13	30	18	12	A	0	191895.785	-0.014	224.6146
31	18	14	30	18	13	A	0	191895.785	-0.014	224.6146
31	18	14	30	18	13	E	0	191896.812	-0.045	224.6030
21	7	15	20	6	14	E	0	191913.474	-0.061	118.2051
44	18	26	44	17	27	E	0	191922.338	-0.020	327.1922
35	2	33	34	2	32	E	0	191927.181	-0.016	182.4796
35	3	33	34	3	32	E	0	191927.181	-0.014	182.4796
35	2	33	34	2	32	A	0	191929.287	-0.008	182.4700
35	2	33	34	3	32	A	0	191929.287	-0.003	182.4700
35	3	33	34	2	32	A	0	191929.287	-0.011	182.4700
35	3	33	34	3	32	A	0	191929.287	-0.006	182.4700
44	18	27	44	17	28	E	0	191936.804	-0.002	327.1799
29	7	23	28	6	22	A	0	191939.512	-0.003	161.0265

J'	K'_a	K'_c	J''	K''_a	K''_c	Sym.	$v_{t_{CH_3}}$	$\nu_{Obs.}$	$\nu_{Obs.} - \nu_{Calc.}$	E_{low}
43	3	40	43	2	41	E	1	191957.247	-0.092	374.5640
43	4	40	43	3	41	E	1	191957.247	-0.092	374.5640
21	7	15	20	6	14	A	0	191959.556	-0.019	118.1952
32	5	27	31	5	26	E	1	191962.058	-0.079	304.9246
44	18	26	44	17	27	A	0	191971.345	0.086	327.1893
44	18	27	44	17	28	A	0	191972.021	-0.134	327.1893
32	6	27	31	5	26	A	1	191973.141	-0.030	305.1930
32	5	27	31	5	26	A	0	191978.121	-0.038	176.5889
36	1	35	35	1	34	A	1	192021.546	-0.001	312.1937
36	1	35	35	2	34	A	1	192021.546	-0.001	312.1937
36	2	35	35	1	34	A	1	192021.546	-0.001	312.1937
36	2	35	35	2	34	A	1	192021.546	-0.001	312.1937
36	1	35	35	1	34	E	1	192062.558	-0.027	311.8577
36	1	35	35	2	34	E	1	192062.558	-0.027	311.8577
36	2	35	35	1	34	E	1	192062.558	-0.027	311.8577
36	2	35	35	2	34	E	1	192062.558	-0.027	311.8577
36	1	35	35	1	34	E	0	192103.557	0.010	183.5412
36	2	35	35	2	34	E	0	192103.557	0.010	183.5412
36	1	35	35	1	34	A	0	192104.783	-0.025	183.5308
36	1	35	35	2	34	A	0	192104.783	-0.024	183.5308
36	2	35	35	1	34	A	0	192104.783	-0.025	183.5308
36	2	35	35	2	34	A	0	192104.783	-0.024	183.5308
31	17	15	30	17	14	A	0	192138.227	-0.078	217.9935
31	17	14	30	17	13	A	0	192138.227	-0.078	217.9935
32	6	27	31	5	26	E	1	192157.619	-0.011	304.9246
32	6	27	31	5	26	E	0	192160.449	-0.016	176.5973
32	6	27	31	5	26	A	0	192166.259	-0.035	176.5889
37	0	37	36	0	36	A	1	192237.746	0.059	312.8670
37	0	37	36	1	36	A	1	192237.746	0.059	312.8670
37	1	37	36	0	36	A	1	192237.746	0.059	312.8670
37	1	37	36	1	36	A	1	192237.746	0.059	312.8670
37	0	37	36	0	36	E	1	192249.284	-0.020	312.4984
37	1	37	36	1	36	E	1	192249.284	-0.020	312.4984
37	0	37	36	0	36	A	0	192296.650	-0.195	184.1777
37	0	37	36	1	36	A	0	192296.650	-0.195	184.1777
37	1	37	36	0	36	A	0	192296.650	-0.195	184.1777
37	1	37	36	1	36	A	0	192296.650	-0.195	184.1777
37	0	37	36	0	36	E	0	192296.650	0.168	184.1892
37	1	37	36	1	36	E	0	192296.650	0.168	184.1892
25	7	19	24	6	18	E	0	192375.057	-0.046	138.0010
25	7	19	24	6	18	A	0	192421.474	-0.010	137.9908
31	16	15	30	16	14	A	0	192429.370	-0.048	211.7653
31	16	16	30	16	15	A	0	192429.370	-0.048	211.7653
31	16	15	30	16	14	E	0	192430.453	0.071	211.7689
31	16	16	30	16	15	E	0	192431.055	0.057	211.7557
65	15	51	65	14	52	A	0	192462.911	-0.115	554.7635
65	15	51	65	14	52	E	0	192468.207	-0.050	554.7640
64	20	45	64	19	46	E	0	192628.768	0.040	570.2179
52	8	44	52	7	45	E	1	192639.489	0.100	488.8438
52	9	44	52	8	45	E	1	192642.527	0.167	488.8438
43	18	25	43	17	26	E	0	192686.876	0.026	317.9849
43	18	26	43	17	27	E	0	192701.910	-0.002	317.9726
64	20	45	64	19	46	A	0	192714.033	-0.037	570.2204
54	9	45	54	8	46	E	1	192719.930	0.225	516.4130
54	10	45	54	9	46	E	1	192731.189	0.171	516.4131
52	8	44	52	7	45	A	0	192747.974	-0.047	360.5394
52	9	44	52	8	45	A	0	192750.742	-0.038	360.5394
31	15	17	30	15	16	A	0	192784.183	-0.052	205.9331
31	15	16	30	15	15	A	0	192784.183	-0.054	205.9331
31	15	16	30	15	15	E	0	192785.250	0.007	205.9383
31	15	17	30	15	16	E	0	192786.208	0.040	205.9248
54	9	45	54	8	46	E	0	192862.557	-0.022	388.1198
24	7	18	23	6	17	E	0	193187.322	-0.050	132.7081
31	14	18	30	14	17	A	0	193224.790	-0.002	200.5017
31	14	17	30	14	16	E	0	193225.895	0.041	200.5083
31	14	18	30	14	17	E	0	193227.116	-0.081	200.4948
22	7	16	21	6	15	E	0	193232.419	-0.085	122.7970
24	7	18	23	6	17	A	0	193234.573	-0.007	132.6979
31	6	25	30	6	24	A	1	193270.383	-0.046	302.0950
22	7	16	21	6	15	A	0	193278.643	0.008	122.7869
55	19	36	55	18	37	E	0	193307.764	-0.142	449.1954
55	19	36	55	18	37	A	0	193308.625	0.040	449.1948
55	19	37	55	18	38	E	0	193352.888	-0.033	449.1833
42	18	24	42	17	25	E	0	193390.926	-0.055	308.9972
42	18	25	42	17	26	E	0	193406.604	0.000	308.9848
42	18	24	42	17	25	A	0	193440.047	0.087	308.9945
55	19	37	55	18	38	A	0	193444.697	-0.025	449.1898
31	6	25	30	6	24	E	0	193515.260	0.003	173.4968
31	6	25	30	6	24	A	0	193523.991	-0.018	173.4881
31	6	25	30	6	24	E	1	193541.796	0.001	301.8181
23	7	17	22	6	16	E	0	193578.331	-0.017	127.6351
23	7	17	22	6	16	A	0	193625.360	0.099	127.6250
45	4	41	45	3	42	E	0	193641.429	0.016	269.0379
45	5	41	45	4	42	E	0	193641.429	0.014	269.0379
45	4	41	45	3	42	A	0	193667.187	0.055	269.0300
45	5	41	45	4	42	A	0	193667.187	0.053	269.0300
45	4	41	45	3	42	E	1	193717.887	-0.085	397.3482
45	5	41	45	4	42	E	1	193717.887	-0.087	397.3482
30	7	24	29	6	23	E	0	193780.813	-0.031	167.1923
31	13	19	30	13	18	A	0	193784.555	-0.083	195.4779
30	7	24	29	6	23	A	0	193804.221	-0.012	167.1833
12	11	2	11	10	2	E	0	193811.577	0.016	99.8626
12	11	1	11	10	1	E	0	193828.308	-0.042	99.8752
12	11	2	11	10	1	A	0	193858.490	0.018	99.8641
12	11	1	11	10	2	A	0	193858.490	0.018	99.8641
30	9	21	29	9	20	A	0	193948.133	-0.017	173.5056
30	9	21	29	9	20	E	0	193954.254	0.004	173.5130
41	18	23	41	17	24	E	0	194039.247	-0.043	300.2281
41	18	24	41	17	25	E	0	194055.426	0.001	300.2157
41	18	23	41	17	24	A	0	194088.223	0.024	300.2255
69	21	48	69	20	49	E	0	194117.110	-0.058	648.3021
69	21	48	69	20	49	A	0	194164.052	-0.069	648.3038
31	12	20	30	12	19	A	0	194515.066	0.005	190.8721
31	12	19	30	12	18	E	0	194522.426	0.001	190.8816
31	12	20	30	12	19	E	0	194524.854	0.151	190.8686
31	12	19	30	12	18	A	0	194527.224	-0.020	190.8725
63	14	50	63	13	51	A	0	194591.315	-0.107	521.5836
63	14	50	63	13	51	E	0	194594.031	-0.057	521.5844
40	18	22	40	17	23	E	0	194635.850	-0.031	291.6768
14	10	5	13	9	5	E	0	194649.105	-0.026	101.3904
40	18	23	40	17	24	E	0	194652.482	-0.001	291.6644
14	10	4	13	9	4	E	0	194671.161	0.066	101.4022
40	18	22	40	17	23	A	0	194684.693	-0.005	291.6743
14	10	4	13	9	5	A	0	194696.710	0.020	101.3902
42	2	40	42	1	41	E	0	194728.609	0.038	231.9889
42	3	40	42	2	41	E	0	194728.609	0.038	231.9889

J'	K_a'	K_c'	J''	K_a''	K_c''	Sym.	$v_{t_{CH_3}}$	$\nu_{Obs.}$	$\nu_{Obs.} - \nu_{Calc.}$	E_{low}
42	2	40	42	1	41	A	0	194760.633	0.082	231.9787
42	3	40	42	2	41	A	0	194760.633	0.082	231.9787
63	20	44	63	19	45	A	0	194817.286	-0.033	556.5586
54	19	35	54	18	36	E	0	194818.876	-0.026	437.5602
54	19	35	54	18	36	A	0	194838.445	-0.015	437.5591
54	19	36	54	18	37	E	0	194842.010	-0.034	437.5488
42	2	40	42	1	41	E	1	194885.805	-0.037	360.2947
42	3	40	42	2	41	E	1	194885.805	-0.037	360.2947
54	19	36	54	18	37	A	0	194912.951	-0.041	437.5563
32	6	26	31	7	25	E	1	195052.350	-0.092	308.3777
32	6	26	31	7	25	A	1	195058.837	0.025	308.6370
32	6	26	31	7	25	A	0	195129.187	-0.009	180.0443
32	6	26	31	7	25	E	0	195130.788	0.001	180.0524
47	5	42	47	4	43	E	0	195170.308	0.025	292.8974
47	6	42	47	5	43	E	0	195170.308	0.011	292.8974
39	18	21	39	17	22	E	0	195184.434	-0.051	283.3427
47	5	42	47	4	43	A	0	195193.149	0.037	292.8906
47	6	42	47	5	43	A	0	195193.149	0.024	292.8906
39	18	22	39	17	23	E	0	195201.498	-0.015	283.3302
47	5	42	47	4	43	E	1	195206.177	-0.060	421.2071
47	6	42	47	5	43	E	1	195206.177	-0.074	421.2071
39	18	21	39	17	22	A	0	195233.199	-0.006	283.3402
16	9	8	15	8	8	E	0	195347.844	0.001	104.1640
16	9	7	15	8	7	E	0	195375.038	-0.039	104.1748
31	11	21	30	11	20	A	0	195472.202	-0.005	186.6998
18	8	11	17	7	10	A	0	195476.466	0.076	108.2353
31	11	21	30	11	20	E	0	195511.585	0.013	186.6997
18	8	10	17	7	10	E	0	195545.318	-0.026	108.2462
31	8	24	30	8	23	E	0	195564.717	-0.006	176.6771
31	8	24	30	8	23	A	0	195566.670	-0.007	176.6694
31	11	20	30	11	19	E	0	195570.516	-0.033	186.7128
18	8	11	17	7	11	E	0	195573.500	0.006	108.2346
31	11	20	30	11	19	A	0	195603.851	-0.026	186.7043
38	18	20	38	17	21	E	0	195688.467	-0.042	275.2249
38	18	20	38	17	21	A	0	195737.120	-0.015	275.2225
62	20	42	62	19	43	A	0	195959.745	-0.042	543.1656
44	3	41	44	2	42	A	1	195967.970	0.023	382.7910
44	4	41	44	3	42	A	1	195967.970	0.023	382.7910
37	18	19	37	17	20	E	0	196151.007	-0.075	267.3227
37	18	20	37	17	21	E	0	196168.794	-0.062	267.3102
37	18	19	37	17	20	A	0	196199.606	-0.011	267.3204
53	19	34	53	18	35	E	0	196201.487	-0.026	426.1543
53	19	35	53	18	36	E	0	196218.525	-0.050	426.1429
53	19	34	53	18	35	A	0	196234.376	-0.015	426.1527
53	19	35	53	18	36	A	0	196274.428	0.004	426.1512
49	6	43	49	5	44	E	1	196404.602	0.053	446.1333
49	7	43	49	6	44	E	1	196404.602	-0.026	446.1333
49	6	43	49	5	44	E	0	196410.508	0.025	317.8258
49	7	43	49	6	44	E	0	196410.508	-0.048	317.8258
31	7	25	30	6	24	E	0	196530.875	0.001	173.4968
31	7	25	30	6	24	A	0	196549.171	-0.001	173.4881
31	10	22	30	10	21	A	0	196558.100	-0.008	182.9773
31	10	22	30	10	21	E	0	196570.804	0.017	182.9822
36	18	18	36	17	19	E	0	196575.055	-0.032	259.6356
36	18	19	36	17	20	E	0	196593.217	0.033	259.6230
36	18	18	36	17	19	A	0	196623.503	-0.032	259.6333
36	18	19	36	17	20	A	0	196623.503	-0.033	259.6333
31	7	25	30	6	24	E	1	196653.237	0.108	301.8181
33	5	28	32	6	27	A	1	196662.619	0.010	311.5965
34	4	30	33	5	29	A	1	196699.949	0.009	314.0377
34	5	30	33	5	29	A	1	196703.375	0.045	314.0377
34	4	30	33	4	29	A	1	196706.221	-0.173	314.0375
34	5	30	33	4	29	A	1	196709.810	0.026	314.0375
30	7	23	29	7	22	E	0	196759.718	-0.067	169.3491
30	7	23	29	7	22	A	0	196766.654	-0.003	169.3396
35	3	32	34	3	31	A	1	196787.541	-0.056	315.9970
35	3	32	34	4	31	A	1	196787.541	0.095	315.9970
35	4	32	34	3	31	A	1	196787.541	-0.131	315.9970
35	4	32	34	4	31	A	1	196787.541	0.020	315.9970
33	5	28	32	6	27	E	1	196799.900	-0.142	311.3343
32	7	26	31	7	25	A	1	196811.105	0.064	308.6370
34	4	30	33	5	29	E	1	196820.477	-0.034	313.7667
33	5	28	32	6	27	E	0	196822.900	0.000	183.0070
33	5	28	32	6	27	A	0	196827.015	-0.023	182.9989
34	5	30	33	4	29	E	1	196831.762	-0.012	313.7664
33	5	28	32	5	27	A	1	196837.605	0.028	311.5907
34	4	30	33	5	29	E	0	196845.912	-0.011	185.4397
34	4	30	33	5	29	A	0	196849.615	-0.012	185.4312
34	5	30	33	5	29	E	0	196849.615	0.000	185.4397
34	4	30	33	4	29	A	0	196856.614	-0.046	185.4310
34	5	30	33	4	29	E	0	196856.614	-0.002	185.4395
34	5	30	33	4	29	A	0	196860.329	-0.041	185.4310
35	3	32	34	3	31	E	1	196881.718	-0.069	315.7108
35	3	32	34	4	31	E	1	196881.718	0.107	315.7108
35	4	32	34	3	31	E	1	196881.718	-0.157	315.7108
35	4	32	34	4	31	E	1	196881.718	0.018	315.7108
33	6	28	32	6	27	E	1	196910.065	-0.086	311.3343
35	3	32	34	3	31	E	0	196913.018	-0.054	187.3860
35	3	32	34	4	31	E	0	196913.018	0.112	187.3860
35	4	32	34	3	31	E	0	196913.018	-0.137	187.3860
35	4	32	34	4	31	E	0	196913.018	0.029	187.3860
35	3	32	34	3	31	A	0	196915.908	-0.057	187.3771
35	3	32	34	4	31	A	0	196915.908	0.109	187.3771
35	4	32	34	3	31	A	0	196915.908	-0.141	187.3771
35	4	32	34	4	31	A	0	196915.908	0.026	187.3771
32	28	4	31	28	3	E	0	196915.908	0.114	318.4469
44	3	41	44	2	42	E	1	196919.344	-0.108	382.5036
44	4	41	44	3	42	E	1	196919.344	-0.108	382.5036
33	6	28	32	6	27	E	0	196928.060	-0.072	183.0070
33	6	28	32	6	27	A	0	196932.713	0.004	182.9989
33	6	28	32	5	27	A	1	196935.549	0.045	311.5907
36	2	34	35	2	33	A	1	196949.530	0.029	317.5098
36	2	34	35	3	33	A	1	196949.530	0.031	317.5098
36	3	34	35	2	33	A	1	196949.530	0.028	317.5098
36	3	34	35	3	33	A	1	196949.530	0.030	317.5098
35	18	17	35	17	18	E	0	196963.190	-0.000	252.1629
32	7	26	31	7	25	E	1	196975.805	0.012	308.3777
35	18	18	35	17	19	E	0	196981.548	-0.033	252.1503
32	7	26	31	7	25	A	0	196992.892	0.009	180.0443
33	5	28	32	5	27	E	1	196995.411	-0.124	311.3277
33	5	28	32	5	27	E	0	197010.353	0.064	183.0008
35	18	17	35	17	18	A	0	197011.544	-0.013	252.1607
35	18	18	35	17	19	A	0	197011.544	-0.013	252.1607
33	5	28	32	5	27	A	0	197015.149	-0.023	182.9926
36	2	34	35	2	33	E	1	197017.518	-0.024	317.2024
36	2	34	35	3	33	E	1	197017.518	-0.021	317.2024

J'	K'_a	K'_c	J''	K''_a	K''_c	Sym.	$v_{t_{CH_3}}$	$\nu_{Obs.}$	$\nu_{Obs.} - \nu_{Calc.}$	E_{low}
36	3	34	35	2	33	E	1	197017.518	-0.025	317.2024
36	3	34	35	3	33	E	1	197017.518	-0.023	317.2024
31	9	23	30	9	22	A	0	197037.562	-0.046	179.6836
31	9	23	30	9	22	E	0	197039.780	0.044	179.6904
36	2	34	35	2	33	E	0	197054.307	-0.013	188.8816
36	3	34	35	3	33	E	0	197054.307	-0.012	188.8816
36	2	34	35	2	33	A	0	197056.356	-0.052	188.8720
36	2	34	35	3	33	A	0	197056.356	-0.050	188.8720
36	3	34	35	2	33	A	0	197056.356	-0.054	188.8720
36	3	34	35	3	33	A	0	197056.356	-0.051	188.8720
33	6	28	32	5	27	E	1	197105.602	-0.042	311.3277
33	6	28	32	5	27	E	0	197115.526	0.005	183.0008
33	6	28	32	5	27	A	0	197120.820	-0.024	182.9926
37	1	36	36	1	35	A	1	197148.936	0.002	318.5989
37	1	36	36	2	35	A	1	197148.936	0.002	318.5989
37	2	36	36	1	35	A	1	197148.936	0.002	318.5989
37	2	36	36	2	35	A	1	197148.936	0.002	318.5989
37	1	36	36	1	35	E	1	197189.811	-0.025	318.2642
37	1	36	36	2	35	E	1	197189.811	-0.025	318.2642
37	2	36	36	1	35	E	1	197189.811	-0.025	318.2642
37	2	36	36	2	35	E	1	197189.811	-0.025	318.2642
37	1	36	36	1	35	E	0	197232.231	0.021	189.9491
37	2	36	36	2	35	E	0	197232.231	0.021	189.9491
37	1	36	36	1	35	A	0	197233.443	-0.023	189.9387
37	1	36	36	2	35	A	0	197233.443	-0.023	189.9387
37	2	36	36	1	35	A	0	197233.443	-0.023	189.9387
37	2	36	36	2	35	A	0	197233.443	-0.023	189.9387
34	18	16	34	17	17	E	0	197317.817	-0.049	244.9040
34	18	17	34	17	18	E	0	197336.539	0.016	244.8914
38	0	38	37	0	37	A	1	197365.563	0.072	319.2793
38	0	38	37	1	37	A	1	197365.563	0.072	319.2793
38	1	38	37	0	37	A	1	197365.563	0.072	319.2793
38	1	38	37	1	37	A	1	197365.563	0.072	319.2793
38	0	38	37	0	37	E	1	197377.004	-0.006	318.9111
38	0	38	37	1	37	E	1	197377.004	-0.006	318.9111
38	1	38	37	0	37	E	1	197377.004	-0.006	318.9111
38	1	38	37	1	37	E	1	197377.004	-0.006	318.9111
41	1	40	41	0	41	A	0	197388.556	0.142	217.9602
41	2	40	41	1	41	A	0	197388.556	0.142	217.9602
38	0	38	37	0	37	A	0	197425.716	-0.191	190.5920
38	0	38	37	1	37	A	0	197425.716	-0.191	190.5920
38	1	38	37	0	37	A	0	197425.716	-0.191	190.5920
38	1	38	37	1	37	A	0	197425.716	-0.191	190.5920
38	0	38	37	0	37	E	0	197425.716	0.169	190.6035
38	1	38	37	1	37	E	0	197425.716	0.169	190.6035
52	19	34	52	18	35	A	0	197535.192	-0.084	414.9734
31	10	21	30	10	20	A	0	197588.175	-0.025	183.0208
33	18	15	33	17	16	E	0	197641.396	-0.023	237.8584
33	18	16	33	17	17	E	0	197660.363	0.046	237.8457
33	18	15	33	17	16	A	0	197689.623	-0.014	237.8562
33	18	16	33	17	17	A	0	197689.623	-0.014	237.8562
32	20	12	31	20	11	A	0	197799.038	-0.120	245.4143
32	20	13	31	20	12	A	0	197799.038	-0.120	245.4143
32	20	12	31	20	11	E	0	197800.015	0.056	245.4116
53	8	45	53	7	46	E	1	197839.876	0.133	499.1573
53	9	45	53	8	46	E	1	197841.498	0.128	499.1573
32	18	14	32	17	15	E	0	197936.002	0.001	231.0254
32	18	15	32	17	16	E	0	197955.158	0.040	231.0128
57	10	47	57	9	48	E	0	197960.742	-0.038	428.0743
32	18	14	32	17	15	A	0	197984.146	-0.007	231.0233
32	18	15	32	17	16	A	0	197984.146	-0.007	231.0233
32	19	14	31	19	13	A	0	197991.309	-0.075	238.0201
32	19	13	31	19	12	A	0	197991.309	-0.075	238.0201
32	19	14	31	19	13	E	0	197992.354	0.007	238.0078
32	6	26	31	6	25	E	0	198146.394	-0.010	179.9518
32	6	26	31	6	25	A	0	198154.322	-0.038	179.9434
32	6	26	31	6	25	E	1	198163.718	-0.058	308.2739
20	7	13	19	6	14	E	0	198175.050	-0.019	113.6347
20	7	13	19	6	14	A	0	198185.219	-0.003	113.6253
31	18	13	31	17	14	E	0	198203.569	-0.059	224.4047
32	18	14	31	18	13	A	0	198216.864	-0.028	231.0155
32	18	15	31	18	14	A	0	198216.864	-0.028	231.0155
32	18	15	31	18	14	E	0	198217.973	-0.100	231.0040
31	18	14	31	17	15	E	0	198222.981	0.038	224.3920
31	18	13	31	17	14	A	0	198251.683	-0.034	224.4026
31	18	14	31	17	15	A	0	198251.683	-0.034	224.4026
61	20	41	61	19	42	A	0	198254.146	-0.021	529.9587
61	20	41	61	19	42	E	0	198281.710	-0.071	529.9568
66	15	52	66	14	53	A	0	198380.314	-0.115	568.3568
66	15	52	66	14	53	E	0	198385.372	-0.044	568.3571
30	8	22	29	8	21	A	0	198434.374	-0.003	171.1455
30	8	22	29	8	21	E	0	198438.442	-0.009	171.1545
30	18	12	30	17	13	E	0	198446.159	-0.033	217.9956
30	18	13	30	17	14	E	0	198465.708	0.023	217.9829
32	17	16	31	17	15	A	0	198484.425	-0.032	224.4026
32	17	15	31	17	14	A	0	198484.425	-0.032	224.4026
30	18	12	30	17	13	A	0	198494.215	-0.008	217.9935
30	18	13	30	17	14	A	0	198494.215	-0.008	217.9935
46	4	42	46	3	43	E	0	198624.404	0.019	277.4864
46	5	42	46	4	43	E	0	198624.404	0.018	277.4864
51	19	32	51	18	33	E	0	198645.894	-0.015	404.0235
51	19	33	51	18	34	E	0	198662.219	-0.020	404.0121
29	18	11	29	17	12	E	0	198665.423	-0.049	211.7977
29	18	12	29	17	13	E	0	198685.163	0.039	211.7850
51	19	32	51	18	33	A	0	198691.470	0.022	404.0218
51	19	33	51	18	34	A	0	198702.214	-0.138	404.0214
29	18	11	29	17	12	A	0	198713.421	-0.027	211.7957
29	18	12	29	17	13	A	0	198713.421	-0.027	211.7957
43	2	41	43	1	42	A	1	198761.483	-0.354	368.2236
43	3	41	43	2	42	A	1	198761.483	-0.354	368.2236
61	20	42	61	19	43	A	0	198774.624	-0.063	529.9389
33	7	26	32	8	25	A	0	198806.017	0.077	189.9062
32	16	16	31	16	15	A	0	198806.017	-0.113	218.1840
32	16	17	31	16	16	A	0	198806.017	-0.112	218.1840
32	16	16	31	16	15	E	0	198807.112	-0.073	218.1877
32	16	17	31	16	16	E	0	198807.877	-0.009	218.1746
33	7	26	32	8	25	E	0	198825.141	-0.003	189.9138
28	18	10	28	17	11	E	0	198863.087	-0.057	205.8106
28	18	11	28	17	12	E	0	198882.966	0.028	205.7979
28	18	10	28	17	11	A	0	198911.065	-0.003	205.8086
28	18	11	28	17	12	A	0	198911.065	-0.003	205.8086
27	18	9	27	17	10	E	0	199040.698	-0.092	200.0339
27	18	10	27	17	11	E	0	199060.761	0.050	200.0211
27	18	9	27	17	10	A	0	199088.656	-0.010	200.0319
27	18	10	27	17	11	A	0	199088.656	-0.010	200.0319
32	15	18	31	15	17	A	0	199198.932	-0.051	212.3637

J'	K'_a	K'_c	J''	K''_a	K''_c	Sym.	$v_{t_{CH_3}}$	$\nu_{Obs.}$	$\nu_{Obs.} - \nu_{Calc.}$	E_{low}
32	15	18	31	15	17	E	0	199201.137	0.004	212.3555
26	18	9	26	17	10	E	0	199219.993	0.055	194.4542
26	18	8	26	17	9	A	0	199247.708	-0.028	194.4650
26	18	9	26	17	10	A	0	199247.708	-0.028	194.4650
25	18	7	25	17	8	E	0	199341.840	-0.061	189.1096
25	18	8	25	17	9	E	0	199362.063	0.029	189.0969
25	18	7	25	17	8	A	0	199389.673	-0.019	189.1076
25	18	8	25	17	9	A	0	199389.673	-0.019	189.1076
24	18	6	24	17	7	E	0	199468.078	-0.042	183.9613
24	18	7	24	17	8	E	0	199488.397	0.056	183.9486
24	18	6	24	17	7	A	0	199515.842	-0.029	183.9594
24	18	7	24	17	8	A	0	199515.842	-0.029	183.9594
23	18	5	23	17	6	E	0	199579.752	-0.077	179.0218
23	18	6	23	17	7	E	0	199600.185	0.057	179.0091
23	18	5	23	17	6	A	0	199627.539	-0.006	179.0199
23	18	6	23	17	7	A	0	199627.539	-0.006	179.0199
43	2	41	43	1	42	E	0	199671.004	0.037	239.5942
43	3	41	43	2	42	E	0	199671.004	0.037	239.5942
22	18	4	22	17	5	E	0	199678.162	-0.068	174.2907
32	14	18	31	14	17	A	0	199688.215	-0.062	206.9470
32	14	19	31	14	18	A	0	199688.215	0.059	206.9470
32	14	18	31	14	17	E	0	199689.365	0.016	206.9537
32	14	19	31	14	18	E	0	199690.904	0.042	206.9402
22	18	5	22	17	6	E	0	199698.653	0.059	174.2780
43	2	41	43	1	42	A	0	199703.856	0.110	239.5841
43	3	41	43	2	42	A	0	199703.856	0.110	239.5841
22	18	4	22	17	5	A	0	199725.883	-0.028	174.2888
22	18	5	22	17	6	A	0	199725.883	-0.028	174.2888
50	19	31	50	18	32	E	0	199728.981	-0.046	393.2961
50	19	32	50	18	33	E	0	199746.041	-0.009	393.2846
21	18	3	21	17	4	E	0	199764.401	-0.059	169.7677
50	19	31	50	18	32	A	0	199776.929	-0.004	393.2944
50	19	32	50	18	33	A	0	199782.337	-0.122	393.2942
21	18	3	21	17	4	A	0	199812.097	-0.014	169.7658
21	18	4	21	17	5	A	0	199812.097	-0.014	169.7658
43	2	41	43	1	42	E	1	199831.760	-0.044	367.8984
43	3	41	43	2	42	E	1	199831.760	-0.044	367.8984
13	11	3	12	10	3	E	0	199957.519	0.016	102.3282
13	11	2	12	10	2	E	0	199974.270	-0.058	102.3408
32	7	26	31	6	25	E	0	200003.678	-0.111	179.9518
13	11	3	12	10	2	A	0	200004.466	0.040	102.3296
13	11	2	12	10	3	A	0	200004.466	0.040	102.3296
32	7	26	31	6	25	A	0	200018.027	-0.018	179.9434
48	5	43	48	4	44	E	0	200185.626	0.005	301.8539
48	6	43	48	5	44	E	0	200185.626	-0.002	301.8539
48	5	43	48	4	44	A	0	200209.110	0.062	301.8472
48	6	43	48	5	44	A	0	200209.110	0.055	301.8472
48	5	43	48	4	44	E	1	200222.995	-0.126	430.1619
48	6	43	48	5	44	E	1	200222.995	-0.133	430.1619
69	15	54	69	14	55	A	0	200275.144	0.007	617.3286
60	20	40	60	19	41	A	0	200292.348	-0.030	516.9910
32	13	20	31	13	19	A	0	200312.155	0.012	201.9419
32	13	19	31	13	18	A	0	200314.090	0.002	201.9420
32	13	19	31	13	18	E	0	200314.090	-0.171	201.9500
32	13	20	31	13	19	E	0	200316.469	0.044	201.9367
60	20	41	60	19	42	E	0	200477.741	-0.081	516.9743
20	6	14	19	5	15	E	0	200483.434	-0.001	111.5177
20	6	14	19	5	15	A	0	200486.420	0.027	111.5077
60	20	41	60	19	42	A	0	200592.604	-0.040	516.9796
49	19	31	49	18	32	E	0	200748.045	-0.026	382.7808
15	10	6	14	9	6	E	0	200772.234	0.007	104.2767
49	19	30	49	18	31	A	0	200779.442	0.076	382.7907
49	19	31	49	18	32	A	0	200781.998	-0.112	382.7906
15	10	5	14	9	5	E	0	200794.239	-0.050	104.2885
15	10	5	14	9	6	A	0	200819.847	0.006	104.2765
68	21	48	68	20	49	A	0	200843.656	-0.035	633.3605
45	3	42	45	2	43	A	1	200904.847	0.061	390.8994
45	4	42	45	3	43	A	1	200904.847	0.061	390.8994
33	6	27	32	7	26	A	1	200925.106	0.025	315.2020
33	6	27	32	7	26	E	1	200982.502	-0.064	314.9481
33	6	27	32	7	26	E	0	201040.377	-0.023	186.6232
33	6	27	32	7	26	A	0	201041.343	0.019	186.6153
32	12	21	31	12	20	A	0	201127.891	-0.000	197.3605
32	12	20	31	12	19	E	0	201142.547	-0.026	197.3702
32	12	21	31	12	20	E	0	201143.476	0.012	197.3573
19	8	12	18	7	11	A	0	201145.578	0.014	112.0083
32	12	20	31	12	19	A	0	201152.745	-0.018	197.3612
32	8	25	31	8	24	E	0	201259.186	-0.015	183.2005
32	8	25	31	8	24	A	0	201262.024	-0.014	183.1928
19	8	11	18	7	11	E	0	201268.750	0.049	112.0182
67	21	46	67	20	47	E	0	201316.193	-0.012	618.8941
67	21	46	67	20	47	A	0	201329.226	-0.052	618.8966
17	9	9	16	8	9	E	0	201394.471	-0.005	107.4811
17	9	8	16	8	8	E	0	201421.923	-0.048	107.4919
31	7	24	30	7	23	A	1	201427.059	0.116	304.5161
17	9	9	16	8	8	A	0	201440.020	-0.061	107.4793
17	9	8	16	8	9	A	0	201444.941	0.110	107.4791
19	8	12	18	7	12	E	0	201462.047	-0.015	112.0009
50	6	44	50	5	45	E	1	201463.478	-0.007	455.5967
50	7	44	50	6	45	E	1	201463.478	-0.049	455.5967
50	6	44	50	5	45	E	0	201468.227	0.012	327.2910
50	7	44	50	6	45	E	0	201468.227	-0.026	327.2910
50	6	44	50	5	45	A	0	201488.626	0.008	327.2853
50	7	44	50	6	45	A	0	201488.626	-0.030	327.2853
19	8	11	18	7	12	A	0	201648.850	0.056	111.9938
48	19	30	48	18	31	E	0	201674.941	-0.013	372.4996
31	7	24	30	7	23	E	0	201695.419	0.045	175.9123
31	7	24	30	7	23	A	0	201704.628	0.006	175.9030
48	19	29	48	18	30	A	0	201706.068	0.027	372.5097
48	19	30	48	18	31	A	0	201707.238	-0.139	372.5096
45	3	42	45	2	43	E	0	201758.276	0.020	262.3080
45	4	42	45	3	43	E	0	201758.276	0.020	262.3080
34	5	29	33	6	28	A	1	201800.133	0.058	318.1598
35	4	31	34	5	30	A	1	201812.665	-0.019	320.5990
35	5	31	34	5	30	A	1	201814.465	0.013	320.5990
35	4	31	34	4	30	A	1	201816.014	-0.061	320.5989
35	5	31	34	4	30	A	1	201817.863	0.019	320.5989
34	6	29	33	6	28	A	1	201854.240	-0.034	318.1598
31	9	22	30	9	21	A	0	201855.331	0.029	179.9750
31	9	22	30	9	21	E	0	201863.066	0.017	179.9826
45	3	42	45	2	43	E	1	201879.473	-0.076	390.6142
45	4	42	45	3	43	E	1	201879.473	-0.076	390.6142
34	5	29	33	5	28	A	1	201898.016	0.015	318.1565
36	3	33	35	3	32	A	1	201909.431	-0.026	322.5611
36	3	33	35	4	32	A	1	201909.431	0.049	322.5612
36	4	33	35	3	32	A	1	201909.431	-0.063	322.5611

J'	K'_a	K'_c	J''	K''_a	K''_c	Sym.	$v_{t_{CH_3}}$	$\nu_{Obs.}$	$\nu_{Obs.} - \nu_{Calc.}$	E_{low}
36	4	33	35	4	32	A	1	201909.431	0.012	322.5612
35	5	31	34	5	30	E	1	201934.329	-0.039	320.3320
35	4	31	34	4	30	E	1	201936.189	-0.030	320.3319
35	5	31	34	4	30	E	1	201938.240	-0.024	320.3319
34	5	29	33	6	28	E	1	201941.235	-0.035	317.9025
34	6	29	33	5	28	A	1	201952.259	0.059	318.1565
35	4	31	34	5	30	E	0	201959.536	-0.002	192.0059
35	5	31	34	5	30	E	0	201961.483	0.012	192.0059
35	4	31	34	5	30	A	0	201963.173	-0.043	191.9976
35	4	31	34	4	30	E	0	201963.173	-0.057	192.0058
34	5	29	33	6	28	E	0	201964.187	0.002	189.5759
35	5	31	34	5	30	A	0	201965.188	0.031	191.9976
35	5	31	34	4	30	E	0	201965.188	0.026	192.0058
35	4	31	34	4	30	A	0	201966.918	-0.006	191.9974
33	7	27	32	7	26	A	1	201978.451	0.022	315.2020
36	3	33	35	3	32	E	1	202002.968	-0.073	322.2781
36	3	33	35	4	32	E	1	202002.968	0.015	322.2781
36	4	33	35	3	32	E	1	202002.968	-0.118	322.2781
36	4	33	35	4	32	E	1	202002.968	-0.029	322.2781
34	6	29	33	6	28	E	0	202022.621	-0.003	189.5759
34	6	29	33	6	28	A	0	202027.184	0.010	189.5678
36	3	33	35	3	32	E	0	202035.950	-0.027	193.9543
36	3	33	35	4	32	E	0	202035.950	0.056	193.9543
36	4	33	35	3	32	E	0	202035.950	-0.069	193.9543
36	4	33	35	4	32	E	0	202035.950	0.014	193.9543
36	3	33	35	3	32	A	0	202038.829	-0.023	193.9455
36	3	33	35	4	32	A	0	202038.829	0.061	193.9455
36	4	33	35	3	32	A	0	202038.829	-0.064	193.9455
36	4	33	35	4	32	A	0	202038.829	0.019	193.9455
34	5	29	33	5	28	E	1	202051.301	-0.078	317.8988
37	2	35	36	2	34	A	1	202075.449	-0.018	324.0794
37	2	35	36	3	34	A	1	202075.449	-0.017	324.0794
37	3	35	36	2	34	A	1	202075.449	-0.018	324.0794
37	3	35	36	3	34	A	1	202075.449	-0.017	324.0794
34	6	29	33	5	28	E	1	202112.695	-0.010	317.8988
59	20	39	59	19	40	A	0	202124.858	0.018	504.2591
59	20	39	59	19	40	E	0	202129.758	-0.035	504.2583
34	6	29	33	5	28	A	0	202132.841	-0.004	189.5643
37	2	35	36	2	34	E	1	202143.220	-0.001	323.7742
37	2	35	36	3	34	E	1	202143.220	0.000	323.7742
37	3	35	36	2	34	E	1	202143.220	-0.002	323.7742
37	3	35	36	3	34	E	1	202143.220	-0.001	323.7742
33	7	27	32	7	26	E	1	202146.892	-0.020	314.9481
33	7	27	32	7	26	E	0	202160.935	-0.050	186.6232
33	7	27	32	7	26	A	0	202166.013	0.060	186.6153
32	11	22	31	11	21	A	0	202177.968	0.008	193.2200
37	2	35	36	2	34	E	0	202181.483	-0.018	195.4546
37	3	35	36	3	34	E	0	202181.483	-0.017	195.4546
37	2	35	36	2	34	A	0	202183.560	-0.020	195.4451
37	2	35	36	3	34	A	0	202183.560	-0.019	195.4451
37	3	35	36	2	34	A	0	202183.560	-0.021	195.4451
37	3	35	36	3	34	A	0	202183.560	-0.020	195.4451
59	20	40	59	19	41	E	0	202199.183	-0.037	504.2462
32	11	22	31	11	21	E	0	202216.696	-0.008	193.2213
38	1	37	37	1	36	A	1	202276.275	0.038	325.1751
38	1	37	37	2	36	A	1	202276.275	0.038	325.1751
38	2	37	37	1	36	A	1	202276.275	0.038	325.1751
38	2	37	37	2	36	A	1	202276.275	0.038	325.1751
59	20	40	59	19	41	A	0	202294.865	-0.052	504.2527
38	1	37	37	1	36	E	1	202316.971	-0.033	324.8417
38	1	37	37	2	36	E	1	202316.971	-0.033	324.8417
38	2	37	37	1	36	E	1	202316.971	-0.033	324.8417
38	2	37	37	2	36	E	1	202316.971	-0.033	324.8417
38	1	37	37	1	36	E	0	202360.815	0.024	196.5281
38	2	37	37	2	36	E	0	202360.815	0.024	196.5281
38	1	37	37	1	36	A	0	202362.026	-0.017	196.5177
38	1	37	37	2	36	A	0	202362.026	-0.017	196.5177
38	2	37	37	1	36	A	0	202362.026	-0.017	196.5177
38	2	37	37	2	36	A	0	202362.026	-0.017	196.5177
32	11	21	31	11	20	E	0	202390.188	-0.086	193.2363
52	7	45	52	6	46	E	1	202404.494	0.125	482.0923
52	8	45	52	7	46	E	1	202404.494	-0.077	482.0923
32	11	21	31	11	20	A	0	202422.269	0.089	193.2289
52	7	45	52	6	46	E	0	202452.466	0.068	353.7904
52	8	45	52	7	46	E	0	202452.466	-0.117	353.7904
52	7	45	52	6	46	A	0	202469.763	0.051	353.7857
52	8	45	52	7	46	A	0	202469.763	-0.136	353.7857
39	0	39	38	0	38	A	1	202493.261	0.076	325.8627
39	0	39	38	1	38	A	1	202493.261	0.076	325.8627
39	1	39	38	0	38	A	1	202493.261	0.076	325.8627
39	1	39	38	1	38	A	1	202493.261	0.076	325.8627
39	0	39	38	0	38	E	1	202504.593	-0.015	325.4949
39	0	39	38	1	38	E	1	202504.593	-0.015	325.4949
39	1	39	38	0	38	E	1	202504.593	-0.015	325.4949
39	1	39	38	1	38	E	1	202504.593	-0.015	325.4949
47	19	28	47	18	29	E	0	202513.448	-0.054	362.4517
47	19	29	47	18	30	E	0	202532.599	-0.006	362.4401
39	0	39	38	0	38	A	0	202554.650	-0.212	197.1775
39	0	39	38	1	38	A	0	202554.650	-0.212	197.1775
39	1	39	38	0	38	A	0	202554.650	-0.212	197.1775
39	1	39	38	1	38	A	0	202554.650	-0.212	197.1775
39	0	39	38	0	38	E	0	202554.650	0.146	197.1889
39	1	39	38	1	38	E	0	202554.650	0.146	197.1889
33	6	27	32	6	26	A	1	202677.356	0.047	315.1435
33	6	27	32	6	26	E	0	202897.866	0.081	186.5612
33	6	27	32	6	26	A	0	202904.962	-0.048	186.5531
32	10	23	31	10	22	A	0	203248.566	-0.030	189.5338
32	10	23	31	10	22	E	0	203256.365	-0.175	189.5391
56	9	47	56	8	48	E	1	203284.088	0.231	538.2350
56	10	47	56	9	48	E	1	203287.649	0.264	538.2350
46	19	27	46	18	28	E	0	203306.556	-0.037	352.6129
46	19	28	46	18	29	E	0	203326.264	-0.027	352.6012
46	19	27	46	18	28	A	0	203356.391	0.109	352.6116
32	9	24	31	9	23	A	0	203369.649	-0.037	186.2560
32	9	24	31	9	23	E	0	203370.730	0.045	186.2629
56	9	47	56	8	48	A	0	203433.159	-0.066	409.9430
56	10	47	56	9	48	A	0	203436.376	-0.112	409.9430
35	8	27	34	9	26	A	0	203462.208	-0.060	207.2204
47	4	43	47	3	44	E	0	203604.076	0.003	286.1059
47	5	43	47	4	44	E	0	203604.076	0.003	286.1059
47	4	43	47	3	44	A	0	203631.157	0.063	286.0982
47	5	43	47	4	44	A	0	203631.157	0.062	286.0982
67	14	53	67	13	54	A	0	203644.870	-0.173	582.1131
67	14	53	67	13	54	E	0	203652.588	0.043	582.1132
47	4	43	47	3	44	E	1	203684.575	-0.072	414.4129
47	5	43	47	4	44	E	1	203684.575	-0.072	414.4129

J'	K'_a	K'_c	J''	K''_a	K''_c	Sym.	$v_{t_{CH_3}}$	$\nu_{Obs.}$	$\nu_{Obs.} - \nu_{Calc.}$	E_{low}
33	7	27	32	6	26	A	1	203730.749	0.091	315.1435
33	22	11	32	22	10	A	0	203752.681	-0.047	267.9528
33	22	12	32	22	11	A	0	203752.681	-0.047	267.9528
58	20	38	58	19	39	E	0	203773.834	-0.038	491.7601
58	20	38	58	19	39	A	0	203787.804	-0.035	491.7603
58	20	39	58	19	40	E	0	203807.877	0.018	491.7492
58	20	39	58	19	40	A	0	203882.409	-0.045	491.7568
33	21	13	32	21	12	A	0	203910.048	-0.293	259.7887
33	21	12	32	21	11	A	0	203910.048	-0.293	259.7887
33	21	12	32	21	11	E	0	203911.182	0.037	259.7845
33	7	27	32	6	26	E	0	204018.351	-0.019	186.5612
33	7	27	32	6	26	A	0	204029.640	0.001	186.5531
45	19	26	45	18	27	E	0	204040.462	-0.031	342.9940
45	19	27	45	18	28	E	0	204060.722	-0.016	342.9822
33	7	27	32	6	26	E	1	204070.261	-0.002	314.8840
45	19	26	45	18	27	A	0	204090.148	0.052	342.9927
33	20	13	32	20	12	A	0	204091.806	-0.132	252.0121
33	20	14	32	20	13	A	0	204091.806	-0.132	252.0121
33	20	13	32	20	12	E	0	204092.874	0.057	252.0095
66	21	45	66	20	46	A	0	204159.141	-0.112	604.5743
33	19	15	32	19	14	A	0	204302.994	-0.058	244.6244
33	19	14	32	19	13	A	0	204302.994	-0.058	244.6244
33	19	15	32	19	14	E	0	204304.084	-0.041	244.6121
33	18	15	32	18	14	A	0	204550.926	-0.040	237.6273
33	18	16	32	18	15	A	0	204550.926	-0.040	237.6273
33	18	15	32	18	14	E	0	204552.114	0.123	237.6279
44	2	42	44	1	43	E	0	204612.511	0.060	247.3706
44	3	42	44	2	43	E	0	204612.511	0.060	247.3706
44	2	42	44	1	43	A	0	204646.152	0.128	247.3605
44	3	42	44	2	43	A	0	204646.152	0.128	247.3605
44	19	25	44	18	26	E	0	204719.400	-0.063	333.5940
44	19	26	44	18	27	E	0	204740.211	0.001	333.5822
44	19	25	44	18	26	A	0	204768.944	-0.003	333.5928
44	2	42	44	1	43	E	1	204776.746	-0.019	375.6730
44	3	42	44	2	43	E	1	204776.746	-0.019	375.6730
33	17	17	32	17	16	A	0	204845.451	-0.030	231.0233
33	17	16	32	17	15	A	0	204845.451	-0.030	231.0233
32	10	22	31	10	21	A	0	204939.083	0.004	189.6117
32	10	22	31	10	21	E	0	204940.608	-0.000	189.6180
49	5	44	49	4	45	E	0	205195.701	-0.016	310.9813
49	6	44	49	5	45	E	0	205195.701	-0.020	310.9813
33	16	17	32	16	16	A	0	205200.119	-0.014	224.8155
33	16	18	32	16	17	A	0	205200.119	-0.013	224.8155
33	16	17	32	16	16	E	0	205201.229	-0.054	224.8192
33	16	18	32	16	17	E	0	205202.146	0.068	224.8061
66	21	46	66	20	47	A	0	205208.610	-0.023	604.5339
49	5	44	49	4	45	A	0	205219.752	0.021	310.9747
49	6	44	49	5	45	A	0	205219.752	0.017	310.9747
49	5	44	49	4	45	E	1	205234.467	-0.095	439.2874
49	6	44	49	5	45	E	1	205234.467	-0.099	439.2874
12	12	1	11	11	1	E	0	205244.579	0.009	103.8641
12	12	0	11	11	0	E	0	205256.051	-0.052	103.8772
12	12	0	11	11	1	A	0	205290.289	0.017	103.8671
12	12	1	11	11	0	A	0	205290.289	0.017	103.8671
57	20	38	57	19	39	E	0	205301.817	0.030	479.4821
57	20	37	57	19	38	A	0	205307.728	-0.030	479.4925
43	19	24	43	18	25	E	0	205347.333	-0.031	324.4123
57	20	38	57	19	39	A	0	205359.459	0.003	479.4906
43	19	25	43	18	26	E	0	205368.569	-0.003	324.4005
43	19	24	43	18	25	A	0	205396.694	-0.026	324.4111
31	8	23	30	8	22	A	0	205476.262	0.077	177.7646
31	8	23	30	8	22	E	0	205477.682	0.051	177.7737
33	15	19	32	15	18	A	0	205634.194	-0.034	219.0082
33	15	18	32	15	17	E	0	205635.400	-0.038	219.0135
33	15	19	32	15	18	E	0	205636.629	0.014	219.0001
42	19	23	42	18	24	E	0	205927.661	-0.059	315.4480
42	19	24	42	18	25	E	0	205949.371	0.021	315.4362
42	19	23	42	18	24	A	0	205976.928	-0.019	315.4470
14	11	4	13	10	4	E	0	206099.517	-0.002	105.0016
14	11	3	13	10	3	E	0	206116.357	-0.032	105.0141
14	11	4	13	10	3	A	0	206146.504	0.044	105.0030
14	11	3	13	10	4	A	0	206146.504	0.044	105.0030
21	7	14	20	6	15	A	0	206158.168	0.019	117.8265
33	14	20	32	14	19	A	0	206176.594	0.132	213.6079
33	14	19	32	14	18	E	0	206177.827	-0.012	213.6146
33	14	20	32	14	19	E	0	206179.533	-0.007	213.6012
32	7	25	31	7	24	E	0	206257.058	-0.023	182.6401
32	7	25	31	7	24	A	0	206267.823	-0.022	182.6311
41	19	22	41	18	23	E	0	206463.698	-0.055	306.7005
34	6	28	33	7	27	A	1	206483.649	-0.000	321.9392
41	19	23	41	18	24	E	0	206485.777	0.006	306.6887
41	19	22	41	18	23	A	0	206512.844	-0.012	306.6996
51	6	45	51	5	46	E	1	206514.186	-0.073	465.2308
51	7	45	51	6	46	E	1	206514.186	-0.095	465.2308
51	6	45	51	5	46	E	0	206518.032	-0.006	336.9269
51	7	45	51	6	46	E	0	206518.032	-0.025	336.9269
51	6	45	51	5	46	A	0	206538.968	-0.012	336.9214
51	7	45	51	6	46	A	0	206538.968	-0.032	336.9214
20	8	13	19	7	12	A	0	206576.798	0.015	116.0067
34	6	28	33	7	27	E	1	206584.099	-0.070	321.6910
34	6	28	33	7	27	E	0	206627.924	-0.007	193.3665
34	6	28	33	7	27	A	0	206630.504	-0.017	193.3588
65	21	44	65	20	45	A	0	206635.984	-0.055	590.4978
65	21	44	65	20	45	E	0	206655.029	-0.068	590.4951
56	20	36	56	19	37	E	0	206665.440	-0.026	467.4544
56	20	37	56	19	38	E	0	206685.718	-0.034	467.4438
56	20	36	56	19	37	A	0	206704.164	0.026	467.4539
46	3	43	46	2	44	E	0	206713.946	0.032	270.5912
46	4	43	46	3	44	E	0	206713.946	0.032	270.5912
56	20	37	56	19	38	A	0	206731.864	-0.020	467.4529
46	3	43	46	2	44	A	0	206744.551	-0.023	270.5824
46	4	43	46	3	44	A	0	206744.551	-0.023	270.5824
20	8	12	19	7	12	E	0	206776.146	-0.036	116.0160
33	8	26	32	8	25	E	0	206802.438	-0.023	189.9138
33	8	26	32	8	25	A	0	206806.100	0.008	189.9062
34	7	27	33	8	26	E	1	206816.832	-0.218	325.1327
46	3	43	46	2	44	E	1	206837.706	-0.053	398.8957
46	4	43	46	3	44	E	1	206837.706	-0.053	398.8957
33	13	21	32	13	20	A	0	206870.859	0.024	208.6236
33	13	20	32	13	19	E	0	206874.095	-0.108	208.6317
33	13	20	32	13	19	A	0	206875.043	0.015	208.6237
33	13	21	32	13	20	E	0	206876.640	0.046	208.6185
16	10	7	15	9	7	E	0	206882.050	0.031	107.3730
16	10	6	15	9	6	E	0	206904.178	-0.031	107.3848
35	5	30	34	6	29	A	1	206918.615	0.017	324.8929
36	4	32	35	5	31	A	1	206926.218	-0.029	327.3308

J'	K'_a	K'_c	J''	K''_a	K''_c	Sym.	$v_{t_{CH_3}}$	$\nu_{Obs.}$	$\nu_{Obs.} - \nu_{Calc.}$	E_{low}
36	5	32	35	5	31	A	1	206927.232	0.070	327.3308
36	4	32	35	4	31	A	1	206927.961	-0.054	327.3308
16	10	6	15	9	7	A	0	206929.755	0.034	107.3727
35	6	30	34	6	29	A	1	206948.302	0.009	324.8929
40	19	21	40	18	22	E	0	206958.365	-0.066	298.1692
35	5	30	34	5	29	A	1	206972.822	0.024	324.8911
40	19	22	40	18	23	E	0	206980.823	0.019	298.1573
34	7	27	33	8	26	A	0	206986.105	-0.025	196.8045
34	7	27	33	8	26	E	0	206998.132	0.004	196.8119
35	6	30	34	5	29	A	1	207002.580	0.087	324.8911
40	19	22	40	18	23	A	0	207007.367	-0.051	298.1683
40	19	21	40	18	22	A	0	207007.367	-0.049	298.1683
37	3	34	36	3	33	A	1	207031.770	-0.020	329.2961
37	3	34	36	4	33	A	1	207031.770	0.018	329.2961
37	4	34	36	3	33	A	1	207031.770	-0.038	329.2961
37	4	34	36	4	33	A	1	207031.770	-0.001	329.2961
36	4	32	35	5	31	E	1	207044.859	-0.034	327.0679
36	5	32	35	5	31	E	1	207045.933	-0.027	327.0679
36	4	32	35	4	31	E	1	207046.922	-0.016	327.0678
36	5	32	35	4	31	E	1	207047.966	-0.038	327.0678
35	5	30	34	6	29	E	1	207061.213	-0.025	324.6406
36	4	32	35	5	31	E	0	207073.906	-0.030	198.7426
36	5	32	35	5	31	E	0	207074.975	0.035	198.7426
36	4	32	35	4	31	E	0	207075.815	-0.052	198.7425
36	5	32	35	4	31	E	0	207076.806	-0.066	198.7425
36	4	32	35	5	31	A	0	207077.606	0.022	198.7344
36	5	32	35	5	31	A	0	207078.613	0.020	198.7344
36	4	32	35	4	31	A	0	207079.468	-0.057	198.7343
36	5	32	35	4	31	A	0	207080.525	-0.010	198.7343
35	5	30	34	6	29	E	0	207085.064	-0.009	196.3146
35	5	30	34	6	29	A	0	207089.435	0.000	196.3067
35	6	30	34	6	29	E	1	207095.017	-0.033	324.6406
34	7	28	33	7	27	A	1	207105.936	0.054	321.9392
35	6	30	34	6	29	E	0	207117.220	0.021	196.3146
35	6	30	34	6	29	A	0	207121.632	-0.075	196.3067
37	3	34	36	3	33	E	1	207124.752	-0.041	329.0162
37	3	34	36	4	33	E	1	207124.752	0.003	329.0162
37	4	34	36	3	33	E	1	207124.752	-0.063	329.0162
37	4	34	36	4	33	E	1	207124.752	-0.019	329.0162
35	5	30	34	5	29	E	0	207143.527	0.015	196.3127
35	5	30	34	5	29	A	0	207148.155	0.026	196.3048
35	6	30	34	5	29	E	1	207156.361	-0.016	324.6385
37	3	34	36	3	33	E	0	207159.359	-0.014	200.6935
37	3	34	36	4	33	E	0	207159.359	0.028	200.6935
37	4	34	36	3	33	E	0	207159.359	-0.034	200.6935
37	4	34	36	4	33	E	0	207159.359	0.007	200.6935
37	3	34	36	3	33	A	0	207162.203	-0.027	200.6848
37	3	34	36	4	33	A	0	207162.203	0.015	200.6848
37	4	34	36	3	33	A	0	207162.203	-0.047	200.6848
37	4	34	36	4	33	A	0	207162.203	-0.006	200.6848
35	6	30	34	5	29	E	0	207175.634	-0.003	196.3127
35	6	30	34	5	29	A	0	207180.404	0.003	196.3048
38	2	36	37	2	35	A	1	207201.436	-0.023	330.8199
38	2	36	37	3	35	A	1	207201.436	-0.023	330.8199
38	3	36	37	2	35	A	1	207201.436	-0.024	330.8199
38	3	36	37	3	35	A	1	207201.436	-0.023	330.8199
65	21	45	65	20	46	A	0	207260.170	-0.050	590.4740
38	2	36	37	2	35	E	1	207268.902	-0.033	330.5169
38	2	36	37	3	35	E	1	207268.902	-0.032	330.5169
38	3	36	37	2	35	E	1	207268.902	-0.033	330.5169
38	3	36	37	3	35	E	1	207268.902	-0.032	330.5169
34	7	28	33	7	27	E	1	207276.620	0.017	321.6910
34	7	28	33	7	27	E	0	207292.180	-0.003	193.3665
34	7	28	33	7	27	A	0	207297.235	-0.072	193.3588
38	2	36	37	2	35	E	0	207308.718	0.002	202.1987
38	3	36	37	3	35	E	0	207308.718	0.002	202.1987
38	2	36	37	2	35	A	0	207310.779	-0.008	202.1892
38	2	36	37	3	35	A	0	207310.779	-0.008	202.1892
38	3	36	37	2	35	A	0	207310.779	-0.008	202.1892
38	3	36	37	3	35	A	0	207310.779	-0.008	202.1892
39	1	38	38	1	37	A	1	207403.452	0.004	331.9223
39	1	38	38	2	37	A	1	207403.452	0.004	331.9223
39	2	38	38	1	37	A	1	207403.452	0.004	331.9223
39	2	38	38	2	37	A	1	207403.452	0.004	331.9223
18	9	10	17	8	10	E	0	207405.806	-0.004	111.0121
39	19	20	39	18	21	E	0	207414.451	-0.039	289.8533
18	9	9	17	8	9	E	0	207433.458	-0.038	111.0228
39	19	21	39	18	22	E	0	207437.232	0.043	289.8414
39	1	38	38	1	37	E	1	207444.062	-0.022	331.5903
39	1	38	38	2	37	E	1	207444.062	-0.022	331.5903
39	2	38	38	1	37	E	1	207444.062	-0.022	331.5903
39	2	38	38	2	37	E	1	207444.062	-0.022	331.5903
18	9	10	17	8	9	A	0	207447.431	-0.007	111.0103
18	9	9	17	8	10	A	0	207460.516	0.081	111.0099
39	19	21	39	18	22	A	0	207463.313	-0.053	289.8525
39	19	20	39	18	21	A	0	207463.313	-0.053	289.8525
39	1	38	38	1	37	E	0	207489.297	0.014	203.2781
39	2	38	38	2	37	E	0	207489.297	0.014	203.2781
39	1	38	38	1	37	A	0	207490.514	-0.018	203.2678
39	1	38	38	2	37	A	0	207490.514	-0.018	203.2678
39	2	38	38	1	37	A	0	207490.514	-0.018	203.2678
39	2	38	38	2	37	A	0	207490.514	-0.018	203.2678
34	6	28	33	6	27	A	1	207537.070	0.072	321.9041
53	7	46	53	6	47	E	0	207552.857	0.041	363.9358
53	8	46	53	7	47	E	0	207552.857	-0.057	363.9358
53	7	46	53	6	47	A	0	207570.675	0.037	363.9312
53	8	46	53	7	47	A	0	207570.675	-0.062	363.9312
40	0	40	39	0	39	A	1	207620.845	0.079	332.6172
40	0	40	39	1	39	A	1	207620.845	0.079	332.6172
40	1	40	39	0	39	A	1	207620.845	0.079	332.6172
40	1	40	39	1	39	A	1	207620.845	0.079	332.6172
40	0	40	39	0	39	E	1	207632.079	-0.015	332.2497
40	1	40	39	1	39	E	1	207632.079	-0.015	332.2497
40	0	40	39	0	39	A	0	207683.523	-0.181	203.9340
40	0	40	39	1	39	A	0	207683.523	-0.181	203.9340
40	1	40	39	0	39	A	0	207683.523	-0.181	203.9340
40	1	40	39	1	39	A	0	207683.523	-0.181	203.9340
40	0	40	39	0	39	E	0	207683.523	0.174	203.9454
40	1	40	39	1	39	E	0	207683.523	0.174	203.9454
34	6	28	33	6	27	A	0	207755.149	0.000	193.3213
48	4	44	48	3	45	A	1	207765.940	0.252	423.4455
48	5	44	48	4	45	A	1	207765.940	0.252	423.4455
33	12	22	32	12	21	A	0	207778.346	-0.010	204.0694
33	12	22	32	12	21	E	0	207803.130	0.006	204.0667
33	12	21	32	12	20	E	0	207808.652	-0.115	204.0796
33	12	21	32	12	20	A	0	207827.503	-0.023	204.0709

J'	K_a'	K_c'	J''	K_a''	K_c''	Sym.	$v_{t_{CH_3}}$	$\nu_{Obs.}$	$\nu_{Obs.} - \nu_{Calc.}$	E_{low}
38	19	20	38	18	21	E	0	207857.496	0.029	281.7404
38	19	20	38	18	21	A	0	207883.244	0.003	281.7515
38	19	19	38	18	20	A	0	207883.244	0.003	281.7515
55	20	35	55	19	36	E	0	207947.442	-0.037	455.6435
55	20	36	55	19	37	E	0	207967.641	-0.006	455.6328
55	20	35	55	19	36	A	0	207991.867	0.033	455.6429
55	20	36	55	19	37	A	0	208006.432	-0.027	455.6424
34	7	28	33	6	27	A	1	208159.322	0.092	321.9041
37	19	18	37	18	19	E	0	208220.707	-0.021	273.8656
37	19	19	37	18	20	E	0	208244.052	0.056	273.8537
37	19	19	37	18	20	A	0	208269.388	-0.012	273.8649
37	19	18	37	18	19	A	0	208269.388	-0.012	273.8649
34	7	28	33	6	27	E	0	208412.757	-0.011	193.3292
34	7	28	33	6	27	A	0	208421.941	0.006	193.3213
34	7	28	33	6	27	E	1	208440.984	0.035	321.6522
57	9	48	57	8	49	E	1	208528.987	0.255	549.4007
57	10	48	57	9	49	E	1	208530.931	0.248	549.4007
36	19	17	36	18	18	E	0	208575.430	-0.034	266.1927
48	4	44	48	3	45	E	0	208580.727	0.006	294.8964
48	5	44	48	4	45	E	0	208580.727	0.006	294.8964
36	19	18	36	18	19	E	0	208598.856	-0.123	266.1807
48	4	44	48	3	45	A	0	208608.497	0.119	294.8888
48	5	44	48	4	45	A	0	208608.497	0.119	294.8888
36	19	18	36	18	19	A	0	208624.024	-0.019	266.1920
36	19	17	36	18	18	A	0	208624.024	-0.019	266.1920
48	4	44	48	3	45	E	1	208662.962	-0.102	423.2016
48	5	44	48	4	45	E	1	208662.962	-0.102	423.2016
64	21	43	64	20	44	A	0	208835.970	-0.039	576.6625
64	21	43	64	20	44	E	0	208855.885	-0.091	576.6601
35	19	16	35	18	17	E	0	208900.691	-0.042	258.7329
33	11	23	32	11	22	A	0	208911.485	-0.016	199.9640
35	19	17	35	18	18	E	0	208924.501	0.027	258.7209
33	11	23	32	11	22	E	0	208939.853	0.039	199.9665
35	19	17	35	18	18	A	0	208949.168	-0.058	258.7323
35	19	16	35	18	17	A	0	208949.168	-0.058	258.7323
54	20	34	54	19	35	E	0	209135.045	-0.008	444.0587
54	20	35	54	19	36	E	0	209155.758	-0.040	444.0480
54	20	34	54	19	35	A	0	209182.492	0.016	444.0582
54	20	35	54	19	36	A	0	209189.880	-0.164	444.0579
34	19	15	34	18	16	E	0	209198.417	-0.047	251.4858
64	21	44	64	20	45	A	0	209200.647	-0.020	576.6487
34	19	16	34	18	17	E	0	209222.467	0.057	251.4738
34	19	16	34	18	17	A	0	209246.849	-0.028	251.4852
34	19	15	34	18	16	A	0	209246.849	-0.028	251.4852
33	11	22	32	11	21	E	0	209328.811	0.025	199.9874
33	11	22	32	11	21	A	0	209349.292	-0.028	199.9810
33	19	14	33	18	15	E	0	209470.398	-0.073	244.4510
33	19	15	33	18	16	E	0	209494.594	-0.007	244.4390
33	19	15	33	18	16	A	0	209518.807	0.001	244.4504
33	19	14	33	18	15	A	0	209518.807	0.001	244.4504
45	2	43	45	1	44	E	0	209553.206	0.136	255.3180
45	3	43	45	2	44	E	0	209553.206	0.136	255.3180
33	9	25	32	9	24	A	0	209553.207	-0.106	193.0397
33	9	25	32	9	24	E	0	209553.207	-0.037	193.0466
45	2	43	45	1	44	A	0	209587.589	0.155	255.3080
45	3	43	45	2	44	A	0	209587.589	0.155	255.3080
32	19	13	32	18	14	E	0	209718.417	-0.038	237.6279
45	2	43	45	1	44	E	1	209720.685	-0.090	383.6187
45	3	43	45	2	44	E	1	209720.685	-0.090	383.6187
32	19	14	32	18	15	E	0	209742.802	0.047	237.6158
32	9	23	31	9	22	A	0	209830.871	-0.004	186.7082
32	9	23	31	9	22	E	0	209838.927	-0.006	186.7160
33	10	24	32	10	23	A	0	209896.555	-0.037	196.3134
33	10	24	32	10	23	E	0	209901.878	0.015	196.3190
31	19	12	31	18	13	E	0	209943.994	-0.037	231.0160
31	19	13	31	18	14	E	0	209968.517	0.035	231.0040
31	19	13	31	18	14	A	0	209992.227	-0.001	231.0155
31	19	12	31	18	13	A	0	209992.227	-0.001	231.0155
34	22	12	33	22	11	A	0	210023.998	-0.102	274.7493
34	22	13	33	22	12	A	0	210023.998	-0.102	274.7493
34	22	12	33	22	11	E	0	210024.893	-0.011	274.7436
30	19	11	30	18	12	E	0	210148.652	-0.065	224.6151
30	19	12	30	18	13	E	0	210173.320	0.017	224.6030
50	5	45	50	4	46	E	0	210200.975	-0.011	320.2795
50	6	45	50	5	46	E	0	210200.975	-0.013	320.2795
50	5	45	50	4	46	A	0	210225.586	0.013	320.2730
50	6	45	50	5	46	A	0	210225.586	0.011	320.2730
53	20	33	53	19	34	E	0	210236.404	-0.074	432.6988
50	5	45	50	4	46	E	1	210240.911	-0.067	448.5838
50	6	45	50	5	46	E	1	210240.911	-0.069	448.5838
53	20	34	53	19	35	E	0	210257.925	-0.021	432.6881
53	20	33	53	19	34	A	0	210285.506	0.070	432.6984
53	20	34	53	19	35	A	0	210289.185	-0.095	432.6983
29	19	10	29	18	11	E	0	210333.921	-0.032	218.4245
29	19	11	29	18	12	E	0	210358.672	0.011	218.4125
29	19	11	29	18	12	A	0	210382.003	-0.027	218.4241
29	19	10	29	18	11	A	0	210382.003	-0.027	218.4241
34	20	14	33	20	13	A	0	210395.254	-0.057	258.8199
34	20	15	33	20	14	A	0	210395.254	-0.057	258.8199
34	20	14	33	20	13	E	0	210396.297	0.022	258.8173
28	19	9	28	18	10	E	0	210501.059	-0.045	212.4440
28	19	10	28	18	11	E	0	210525.952	0.031	212.4319
28	19	10	28	18	11	A	0	210549.104	-0.023	212.4436
28	19	9	28	18	10	A	0	210549.104	-0.023	212.4436
34	19	16	33	19	15	A	0	210626.542	-0.086	251.4392
34	19	15	33	19	14	A	0	210626.542	-0.086	251.4392
34	19	16	33	19	15	E	0	210627.738	-0.083	251.4270
33	7	26	32	7	25	A	0	210637.668	-0.030	189.5115
27	19	8	27	18	9	E	0	210651.422	-0.042	206.6731
33	7	26	32	7	25	E	1	210669.640	0.059	317.8320
27	19	9	27	18	10	E	0	210676.426	0.049	206.6611
27	19	9	27	18	10	A	0	210699.344	-0.091	206.6727
27	19	8	27	18	9	A	0	210699.344	-0.091	206.6727
47	3	44	47	2	45	A	1	210773.535	-0.033	407.6290
47	4	44	47	3	45	A	1	210773.535	-0.033	407.6290
26	19	7	26	18	8	E	0	210786.201	-0.058	201.1116
26	19	8	26	18	9	E	0	210811.441	0.183	201.0995
63	21	42	63	20	43	A	0	210814.449	-0.034	563.0649
63	21	42	63	20	43	E	0	210823.216	-0.050	563.0629
26	19	8	26	18	9	A	0	210834.159	-0.025	201.1112
26	19	7	26	18	8	A	0	210834.159	-0.025	201.1112
34	18	16	33	18	15	A	0	210898.535	-0.022	244.4504
34	18	17	33	18	16	A	0	210898.535	-0.022	244.4504
34	18	16	33	18	15	E	0	210899.806	0.129	244.4510
25	19	6	25	18	7	E	0	210906.603	-0.057	195.7589
63	21	43	63	20	44	E	0	210926.578	-0.050	563.0506

J'	K'_a	K'_c	J''	K''_a	K''_c	Sym.	$v_{t_{\mathrm{CH_3}}}$	$\nu_{\mathrm{Obs.}}$	$\nu_{\mathrm{Obs.}} - \nu_{\mathrm{Calc.}}$	E_{low}
25	19	7	25	18	8	E	0	210931.888	0.154	195.7469
25	19	7	25	18	8	A	0	210954.470	-0.070	195.7586
25	19	6	25	18	7	A	0	210954.470	-0.070	195.7586
24	19	5	24	18	6	E	0	211013.762	-0.010	190.6149
63	21	43	63	20	44	A	0	211023.814	-0.017	563.0570
24	19	6	24	18	7	E	0	211038.958	0.045	190.6028
24	19	6	24	18	7	A	0	211061.614	0.004	190.6145
24	19	5	24	18	6	A	0	211061.614	0.004	190.6145
23	19	5	23	18	6	A	0	211156.397	-0.054	185.6788
23	19	4	23	18	5	A	0	211156.397	-0.054	185.6788
22	19	3	22	18	4	E	0	211192.245	-0.052	180.9513
34	17	18	33	17	17	A	0	211222.007	-0.032	237.8562
34	17	17	33	17	16	A	0	211222.007	-0.032	237.8562
34	17	17	33	17	16	E	0	211223.200	-0.030	237.8584
34	17	18	33	17	17	E	0	211223.835	0.029	237.8457
22	19	4	22	18	5	A	0	211240.108	0.046	180.9510
22	19	3	22	18	4	A	0	211240.108	0.046	180.9510
52	20	32	52	19	33	E	0	211258.819	-0.024	421.5628
52	20	33	52	19	34	E	0	211280.967	-0.060	421.5520
13	12	2	12	11	2	E	0	211392.704	0.025	106.3275
13	12	1	12	11	1	E	0	211404.171	-0.066	106.3406
13	12	1	12	11	2	A	0	211438.421	0.032	106.3305
13	12	2	12	11	1	A	0	211438.421	0.032	106.3305
21	8	14	20	7	13	E	0	211536.094	-0.061	120.2451
52	6	46	52	5	47	E	1	211557.528	-0.010	475.0355
52	7	46	52	6	47	E	1	211557.528	-0.021	475.0355
52	6	46	52	5	47	E	0	211560.613	0.001	346.7335
52	7	46	52	6	47	E	0	211560.613	-0.009	346.7335
52	6	46	52	5	47	A	0	211582.093	0.015	346.7281
52	7	46	52	6	47	A	0	211582.093	0.005	346.7281
34	16	18	33	16	17	A	0	211612.230	-0.038	231.6602
34	16	19	33	16	18	A	0	211612.230	-0.036	231.6602
34	16	18	33	16	17	E	0	211613.419	-0.104	231.6640
34	16	19	33	16	18	E	0	211614.385	-0.033	231.6509
21	8	14	20	7	13	A	0	211642.801	0.024	120.2360
47	3	44	47	2	45	E	0	211667.946	0.025	279.0455
47	4	44	47	3	45	E	0	211667.946	0.025	279.0455
47	3	44	47	2	45	E	1	211794.091	-0.107	407.3482
47	4	44	47	3	45	E	1	211794.091	-0.107	407.3482
35	6	29	34	7	28	A	1	211846.529	0.051	328.8475
32	8	24	31	8	23	E	0	211972.264	-0.107	184.6277
35	6	29	34	7	28	E	0	212009.365	-0.090	200.2811
35	6	29	34	7	28	A	0	212013.073	-0.026	200.2735
36	5	31	35	6	30	A	1	212027.919	0.032	331.7960
37	4	33	36	5	32	A	1	212040.863	0.046	334.2332
37	5	33	36	4	32	A	1	212042.191	-0.013	334.2332
36	6	31	35	6	30	A	1	212043.984	-0.022	331.7960
36	5	31	35	5	30	A	1	212057.665	0.083	331.7950
36	6	31	35	5	30	A	1	212073.708	0.007	331.7950
34	15	20	33	15	19	A	0	212091.097	0.000	225.8675
34	15	19	33	15	18	E	0	212092.403	-0.021	225.8728
34	15	20	33	15	19	E	0	212093.708	-0.037	225.8594
38	3	35	37	3	34	A	1	212154.489	-0.020	336.2020
38	3	35	37	4	34	A	1	212154.489	-0.001	336.2020
38	4	35	37	3	34	A	1	212154.489	-0.029	336.2020
38	4	35	37	4	34	A	1	212154.489	-0.011	336.2020
37	5	33	36	4	32	E	1	212160.148	0.071	333.9741
36	5	31	35	6	30	E	1	212170.573	-0.022	331.5485
37	4	33	36	5	32	E	0	212189.283	-0.053	205.6499
37	5	33	36	4	32	E	0	212190.784	-0.076	205.6498
37	4	33	36	5	32	A	0	212192.932	-0.022	205.6418
37	5	33	36	4	32	A	0	212194.470	-0.016	205.6417
36	5	31	35	6	30	E	0	212195.777	-0.096	203.2233
36	5	31	35	6	30	A	0	212200.227	-0.023	203.2156
36	5	31	35	5	30	E	1	212204.359	-0.047	331.5474
51	20	31	51	19	32	E	0	212208.355	0.005	410.6496
36	6	31	35	6	30	E	0	212213.384	0.013	203.2233
34	8	27	33	8	26	E	0	212213.384	-0.058	196.8119
36	6	31	35	6	30	A	0	212217.789	-0.043	203.2156
34	8	27	33	8	26	A	0	212217.789	0.036	196.8045
36	6	31	35	5	30	E	1	212222.852	-0.024	331.5474
36	5	31	35	5	30	E	0	212227.922	-0.077	203.2222
70	22	48	70	21	49	A	0	212231.243	0.690	669.7276
51	20	32	51	19	33	E	0	212231.243	0.037	410.6388
36	5	31	35	5	30	A	0	212232.492	-0.031	203.2145
15	11	5	14	10	5	E	0	212235.745	0.031	107.8832
36	6	31	35	5	30	E	0	212245.516	0.019	203.2222
38	3	35	37	3	34	E	1	212246.910	-0.041	335.9251
38	3	35	37	4	34	E	1	212246.910	-0.019	335.9251
38	4	35	37	3	34	E	1	212246.910	-0.052	335.9251
38	4	35	37	4	34	E	1	212246.910	-0.030	335.9251
36	6	31	35	5	30	A	0	212250.093	-0.011	203.2145
15	11	4	14	10	4	E	0	212252.596	-0.050	107.8958
51	20	31	51	19	32	A	0	212258.240	0.008	410.6494
51	20	32	51	19	33	A	0	212259.090	-0.079	410.6494
38	3	35	37	3	34	E	0	212283.104	-0.066	207.6036
38	4	35	37	4	34	E	0	212283.104	-0.056	207.6036
38	3	35	37	3	34	A	0	212286.006	-0.005	207.5950
38	3	35	37	4	34	A	0	212286.006	0.016	207.5950
38	4	35	37	3	34	A	0	212286.006	-0.015	207.5950
38	4	35	37	4	34	A	0	212286.006	0.006	207.5950
39	2	37	38	2	36	A	1	212327.445	-0.014	337.7314
39	2	37	38	3	36	A	1	212327.445	-0.014	337.7314
39	3	37	38	2	36	A	1	212327.445	-0.014	337.7314
39	3	37	38	3	36	A	1	212327.445	-0.014	337.7314
39	2	37	38	2	36	E	1	212394.628	-0.034	337.4307
39	2	37	38	3	36	E	1	212394.628	-0.034	337.4307
39	3	37	38	2	36	E	1	212394.628	-0.034	337.4307
39	3	37	38	3	36	E	1	212394.628	-0.034	337.4307
35	7	29	34	7	28	E	0	212397.218	-0.007	200.2811
35	7	29	34	7	28	A	0	212402.411	-0.017	200.2735
39	2	37	38	2	36	E	0	212435.935	-0.009	209.1138
39	3	37	38	3	36	E	0	212435.935	-0.009	209.1138
39	2	37	38	2	36	A	0	212437.993	-0.014	209.1044
39	2	37	38	3	36	A	0	212437.993	-0.014	209.1044
39	3	37	38	2	36	A	0	212437.993	-0.014	209.1044
39	3	37	38	3	36	A	0	212437.993	-0.014	209.1044
35	6	29	34	6	28	A	1	212468.761	0.050	328.8268
40	1	39	39	1	38	A	1	212530.571	0.011	338.8405
40	1	39	39	2	38	A	1	212530.571	0.011	338.8405
40	2	39	39	1	38	A	1	212530.571	0.011	338.8405
40	2	39	39	2	38	A	1	212530.571	0.011	338.8405
33	10	23	32	10	22	A	0	212543.945	-0.028	196.4477
33	10	23	32	10	22	E	0	212549.438	-0.007	196.4541
40	1	39	39	1	38	E	1	212571.064	-0.004	338.5099
40	1	39	39	2	38	E	1	212571.064	-0.004	338.5099

J'	K_a'	K_c'	J''	K_a''	K_c''	Sym.	$v_{t_{CH_3}}$	$\nu_{Obs.}$	$\nu_{Obs.} - \nu_{Calc.}$	E_{low}
40	2	39	39	1	38	E	1	212571.064	-0.004	338.5099
40	2	39	39	2	38	E	1	212571.064	-0.004	338.5099
54	7	47	54	6	48	E	1	212596.031	0.064	502.5495
54	8	47	54	7	48	E	1	212596.031	0.008	502.5495
62	21	41	62	20	42	E	0	212602.936	-0.019	549.7007
62	21	41	62	20	42	A	0	212611.405	0.044	549.7021
40	1	39	39	1	38	E	0	212617.689	0.009	210.1992
40	2	39	39	2	38	E	0	212617.689	0.009	210.1992
40	1	39	39	1	38	A	0	212618.903	-0.022	210.1889
40	1	39	39	2	38	A	0	212618.903	-0.022	210.1889
40	2	39	39	1	38	A	0	212618.903	-0.022	210.1889
40	2	39	39	2	38	A	0	212618.903	-0.022	210.1889
54	7	47	54	6	48	E	0	212642.755	0.005	374.2517
54	8	47	54	7	48	E	0	212642.755	-0.046	374.2517
54	7	47	54	6	48	A	0	212661.046	-0.012	374.2472
54	8	47	54	7	48	A	0	212661.046	-0.064	374.2472
35	6	29	34	6	28	E	0	212673.667	-0.039	200.2589
35	6	29	34	6	28	A	0	212679.879	-0.007	200.2513
34	14	21	33	14	20	A	0	212691.247	-0.036	220.4852
34	14	20	33	14	19	A	0	212691.749	-0.177	220.4852
34	14	20	33	14	19	E	0	212692.945	-0.005	220.4919
34	14	21	33	14	20	E	0	212694.843	-0.012	220.4786
62	21	42	62	20	43	A	0	212729.488	-0.012	549.6976
41	0	41	40	0	40	A	1	212748.299	0.067	339.5427
41	0	41	40	1	40	A	1	212748.299	0.067	339.5427
41	1	41	40	0	40	A	1	212748.299	0.067	339.5427
41	1	41	40	1	40	A	1	212748.299	0.067	339.5427
41	0	41	40	0	40	E	1	212759.449	-0.016	339.1756
41	1	41	40	1	40	E	1	212759.449	-0.016	339.1756
41	0	41	40	0	40	A	0	212812.245	-0.186	210.8615
41	0	41	40	1	40	A	0	212812.245	-0.186	210.8615
41	1	41	40	0	40	A	0	212812.245	-0.186	210.8615
41	1	41	40	1	40	A	0	212812.245	-0.186	210.8615
41	0	41	40	0	40	E	0	212812.245	0.166	210.8729
41	1	41	40	1	40	E	0	212812.245	0.166	210.8729
35	7	29	34	6	28	A	1	212830.799	0.076	328.8268
17	10	8	16	9	8	E	0	212974.854	-0.026	110.6801
17	10	7	16	9	7	E	0	212997.208	-0.027	110.6919
17	10	7	16	9	8	A	0	213022.680	-0.056	110.6798
35	7	29	34	6	28	E	0	213061.455	-0.022	200.2589
35	7	29	34	6	28	A	0	213069.168	-0.047	200.2513
35	7	29	34	6	28	E	1	213072.427	0.012	328.5819
50	20	30	50	19	31	E	0	213090.358	-0.138	399.9583
50	20	31	50	19	32	E	0	213113.921	-0.054	399.9474
50	20	30	50	19	31	A	0	213140.553	0.162	399.9582
56	8	48	56	7	49	E	1	213336.190	0.270	531.1189
56	9	48	56	8	49	E	1	213336.190	0.012	531.1189
19	9	10	18	8	10	E	0	213401.122	-0.041	114.7689
19	9	11	18	8	10	A	0	213405.417	-0.018	114.7567
56	8	48	56	7	49	E	0	213427.162	0.094	402.8268
56	9	48	56	8	49	E	0	213427.162	-0.141	402.8268
19	9	10	18	8	11	A	0	213438.620	0.046	114.7557
56	8	48	56	7	49	A	0	213442.236	0.044	402.8234
56	9	48	56	8	49	A	0	213442.236	-0.193	402.8234
34	13	21	33	13	20	E	0	213468.590	-0.016	215.5323
46	2	44	46	1	45	A	1	213516.054	-0.468	392.0566
46	3	44	46	2	45	A	1	213516.054	-0.468	392.0566
49	4	45	49	3	46	E	0	213554.574	0.031	303.8578
49	5	45	49	4	46	E	0	213554.574	0.031	303.8578
49	4	45	49	3	46	A	0	213582.886	0.059	303.8503
49	5	45	49	4	46	A	0	213582.886	0.059	303.8503
49	4	45	49	3	46	E	1	213638.395	-0.106	432.1612
49	5	45	49	4	46	E	1	213638.395	-0.106	432.1612
21	8	13	20	7	14	A	0	213691.170	-0.006	120.1788
58	9	49	58	8	50	E	1	213752.575	0.202	560.7362
58	10	49	58	9	50	E	1	213753.711	0.266	560.7362
60	10	50	60	9	51	E	1	213814.228	0.401	591.3938
60	11	50	60	10	51	E	1	213818.329	0.410	591.3938
49	20	29	49	19	30	E	0	213910.130	-0.069	389.4880
49	20	30	49	19	31	E	0	213934.229	-0.025	389.4770
49	20	29	49	19	30	A	0	213960.066	0.069	389.4880
35	7	28	34	8	27	E	1	214062.859	-0.151	332.2110
35	7	28	34	8	27	A	0	214193.903	0.028	203.8833
61	21	40	61	20	41	A	0	214255.598	-0.039	536.5717
61	21	41	61	20	42	E	0	214261.494	-0.050	536.5608
61	21	41	61	20	42	A	0	214321.101	-0.079	536.5693
34	12	23	33	12	22	A	0	214466.831	-0.012	211.0001
36	8	28	35	9	27	A	0	214475.812	-0.041	214.6062
46	2	44	46	1	45	E	0	214492.852	-0.015	263.4365
46	3	44	46	2	45	E	0	214492.852	-0.015	263.4365
34	12	23	33	12	22	E	0	214502.349	-0.084	210.9982
36	8	28	35	9	27	E	0	214507.409	0.001	214.6130
46	2	44	46	1	45	A	0	214528.097	0.079	263.4265
46	3	44	46	2	45	A	0	214528.097	0.079	263.4265
34	12	22	33	12	21	E	0	214532.194	0.011	211.0113
34	12	22	33	12	21	A	0	214560.962	-0.096	211.0033
46	2	44	46	1	45	E	1	214663.812	-0.062	391.7353
46	3	44	46	2	45	E	1	214663.812	-0.062	391.7353
48	20	28	48	19	29	E	0	214671.835	-0.049	379.2377
48	20	28	48	19	29	A	0	214721.550	0.014	379.2378
34	7	27	33	7	26	E	0	214975.418	-0.026	196.5459
34	7	27	33	7	26	A	0	214986.268	-0.015	196.5376
36	6	30	35	7	29	A	1	217089.376	-0.058	335.9261
39	3	36	38	3	35	A	1	217277.548	0.006	343.2787
39	3	36	38	4	35	A	1	217277.548	0.015	343.2787
39	4	36	38	3	35	A	1	217277.548	0.002	343.2787
39	4	36	38	4	35	A	1	217277.548	0.011	343.2787
37	6	32	36	5	31	E	1	217303.743	-0.090	338.6257
39	3	36	38	3	35	E	1	217369.391	-0.052	343.0049
39	3	36	38	4	35	E	1	217369.391	-0.041	343.0049
39	4	36	38	3	35	E	1	217369.391	-0.057	343.0049
39	4	36	38	4	35	E	1	217369.391	-0.046	343.0049
39	3	36	38	3	35	E	0	217407.216	-0.080	214.6846
39	4	36	38	4	35	E	0	217407.216	-0.075	214.6846
39	4	36	38	3	35	A	0	217410.102	-0.023	214.6761
40	2	38	39	2	37	A	1	217453.444	-0.002	344.8139
40	2	38	39	3	37	A	1	217453.444	-0.002	344.8139
40	3	38	39	2	37	A	1	217453.444	-0.002	344.8139
40	3	38	39	3	37	A	1	217453.444	-0.002	344.8139
40	2	38	39	2	37	E	1	217520.342	-0.042	344.5154
40	2	38	39	3	37	E	1	217520.342	-0.041	344.5154
40	3	38	39	2	37	E	1	217520.342	-0.042	344.5154
40	3	38	39	3	37	E	1	217520.342	-0.041	344.5154
40	2	38	39	2	37	E	0	217563.144	-0.023	216.1999
40	3	38	39	3	37	E	0	217563.144	-0.023	216.1999
40	2	38	39	2	37	A	0	217565.220	-0.002	216.1906

J'	K'_a	K'_c	J''	K''_a	K''_c	Sym.	$v_{t_{CH_3}}$	$\nu_{Obs.}$	$\nu_{Obs.} - \nu_{Calc.}$	E_{low}
40	2	38	39	3	37	A	0	217565.220	-0.002	216.1906
40	3	38	39	2	37	A	0	217565.220	-0.002	216.1906
40	3	38	39	3	37	A	0	217565.220	-0.002	216.1906
41	1	40	40	1	39	A	1	217657.588	0.018	345.9298
41	1	40	40	2	39	A	1	217657.588	0.018	345.9298
41	2	40	40	1	39	A	1	217657.588	0.018	345.9298
41	2	40	40	2	39	A	1	217657.588	0.018	345.9298
41	1	40	40	1	39	E	1	217698.075	0.123	345.6005
41	1	40	40	2	39	E	1	217698.075	0.123	345.6005
41	2	40	40	1	39	E	1	217698.075	0.123	345.6005
41	2	40	40	2	39	E	1	217698.075	0.123	345.6005
41	1	40	40	1	39	E	0	217745.944	-0.034	217.2914
41	2	40	40	2	39	E	0	217745.944	-0.034	217.2914
41	1	40	40	1	39	A	0	217747.231	0.012	217.2811
41	1	40	40	2	39	A	0	217747.231	0.012	217.2811
41	2	40	40	1	39	A	0	217747.231	0.012	217.2811
41	2	40	40	2	39	A	0	217747.231	0.012	217.2811
42	0	42	41	0	41	A	1	217875.568	-0.010	346.6392
42	0	42	41	1	41	A	1	217875.568	-0.010	346.6392
42	1	42	41	0	41	A	1	217875.568	-0.010	346.6392
42	1	42	41	1	41	A	1	217875.568	-0.010	346.6392
42	0	42	41	0	41	E	1	217886.701	-0.015	346.2725
42	1	42	41	1	41	E	1	217886.701	-0.015	346.2725
42	0	42	41	0	41	A	0	217940.847	-0.192	217.9602
42	0	42	41	1	41	A	0	217940.847	-0.192	217.9602
42	1	42	41	0	41	A	0	217940.847	-0.192	217.9602
42	1	42	41	1	41	A	0	217940.847	-0.192	217.9602
42	0	42	41	0	41	E	0	217940.847	0.157	217.9716
42	1	42	41	1	41	E	0	217940.847	0.157	217.9716
42	20	23	42	19	24	E	0	218252.016	-0.007	322.3059
42	20	22	42	19	23	A	0	218273.711	-0.037	322.3176
42	20	23	42	19	24	A	0	218273.711	-0.038	322.3176
16	11	6	15	10	6	E	0	218363.950	0.015	110.9737
16	11	5	15	10	5	E	0	218380.910	-0.039	110.9863
16	11	6	15	10	5	A	0	218410.974	0.028	110.9751
16	11	5	15	10	6	A	0	218410.974	0.027	110.9751
58	21	37	58	20	38	E	0	218416.204	-0.029	498.5573
58	21	37	58	20	38	A	0	218462.548	0.045	498.5579
31	8	24	30	7	23	E	0	218494.480	-0.098	175.9123
31	8	24	30	7	23	A	0	218542.172	-0.016	175.9030
50	4	46	50	3	47	A	0	218554.788	0.153	312.9828
50	5	46	50	4	47	A	0	218554.788	0.153	312.9828
57	8	49	57	7	50	E	0	218562.809	0.037	413.8234
57	9	49	57	8	50	E	0	218562.809	-0.089	413.8234
35	15	21	34	15	20	A	0	218570.821	0.005	232.9421
57	8	49	57	7	50	A	0	218578.322	-0.016	413.8201
57	9	49	57	8	50	A	0	218578.322	-0.142	413.8201
33	8	26	32	7	25	E	0	218603.764	-0.021	189.5201
33	8	26	32	7	25	A	0	218637.856	0.004	189.5115
41	20	21	41	19	22	E	0	218675.397	-0.019	313.5874
41	20	22	41	19	23	E	0	218703.034	0.321	313.5763
41	20	21	41	19	22	A	0	218723.980	-0.030	313.5881
41	20	22	41	19	23	A	0	218723.980	-0.030	313.5881
18	10	9	17	9	9	E	0	219046.684	0.006	114.1989
18	10	8	17	9	8	E	0	219069.204	-0.038	114.2106
18	10	9	17	9	8	A	0	219094.548	0.225	114.1986
40	20	20	40	19	21	A	0	219140.349	-0.036	305.0733
40	20	21	40	19	22	A	0	219140.349	-0.036	305.0733
20	9	12	19	8	12	E	0	219291.649	-0.040	118.7209
20	9	11	19	8	11	E	0	219313.146	-0.060	118.7318
67	22	45	67	21	46	A	0	219383.153	-0.014	625.6123
67	22	45	67	21	46	E	0	219392.954	-0.116	625.6093
35	7	28	34	7	27	E	0	219415.446	-0.068	203.7167
38	20	18	38	19	19	E	0	219831.483	-0.009	288.6849
38	20	19	38	19	20	E	0	219859.609	0.067	288.6738
23	8	16	22	7	15	E	0	219861.704	-0.053	129.4252
38	20	18	38	19	19	A	0	219879.731	-0.011	288.6858
38	20	19	38	19	20	A	0	219879.731	-0.011	288.6858
23	8	16	22	7	15	A	0	219921.681	0.009	129.4161
22	8	14	21	7	15	A	0	219982.534	-0.041	124.5982
22	8	14	21	7	15	E	0	219992.362	-0.003	124.6066
35	13	23	34	13	22	A	0	220089.825	0.008	222.6444
35	13	22	34	13	21	E	0	220100.716	-0.041	222.6529
35	13	23	34	13	22	E	0	220102.669	-0.016	222.6398
35	13	22	34	13	21	A	0	220107.478	-0.107	222.6450
37	20	17	37	19	18	E	0	220158.415	0.026	280.8111
37	20	18	37	19	19	E	0	220186.695	0.047	280.7999
34	8	27	33	7	26	E	0	220190.716	-0.042	196.5459
37	20	17	37	19	18	A	0	220206.693	0.157	280.8120
37	20	18	37	19	19	A	0	220206.693	0.157	280.8120
34	8	27	33	7	26	A	0	220217.905	-0.001	196.5376
52	5	47	52	4	48	A	0	220224.189	0.029	339.3822
52	6	47	52	5	48	A	0	220224.189	0.029	339.3822
34	10	24	33	10	23	A	0	220404.965	-0.082	203.5374
34	10	24	33	10	23	E	0	220413.178	0.004	203.5440
36	20	16	36	19	17	E	0	220458.976	-0.067	273.1500
36	20	17	36	19	18	E	0	220487.538	0.044	273.1388
36	20	16	36	19	17	A	0	220507.080	-0.015	273.1509
36	20	17	36	19	18	A	0	220507.080	-0.015	273.1509
36	7	29	35	8	28	A	0	220672.096	-0.105	211.1390
36	7	29	35	8	28	E	0	220674.436	0.080	211.1462
35	20	15	35	19	16	E	0	220734.930	-0.161	265.7011
56	21	35	56	20	36	E	0	220736.082	0.142	474.3480
56	21	36	56	20	37	E	0	220761.495	-0.039	474.3381
35	20	16	35	19	17	E	0	220763.781	0.065	265.6899
35	20	15	35	19	16	A	0	220783.018	-0.036	265.7021
35	20	16	35	19	17	A	0	220783.018	-0.036	265.7021
56	21	35	56	20	36	A	0	220785.107	-0.041	474.3488
56	21	36	56	20	37	A	0	220787.725	-0.090	474.3487
34	20	14	34	19	15	E	0	220988.036	-0.046	258.4640
34	20	15	34	19	16	E	0	221016.914	0.049	258.4528
34	20	14	34	19	15	A	0	221035.932	-0.029	258.4650
34	20	15	34	19	16	A	0	221035.932	-0.029	258.4650
35	12	24	34	12	23	A	0	221191.990	-0.027	218.1539
33	20	13	33	19	14	E	0	221219.402	-0.076	251.4382
35	12	24	34	12	23	E	0	221233.131	0.010	218.1533
33	20	14	33	19	15	E	0	221248.424	0.020	251.4270
33	20	13	33	19	14	A	0	221267.250	-0.028	251.4392
33	20	14	33	19	15	A	0	221267.250	-0.028	251.4392
29	8	22	28	7	21	E	0	221301.808	-0.078	162.9673
35	12	23	34	12	22	E	0	221333.499	0.008	218.1673
29	8	22	28	7	21	A	0	221357.442	-0.010	162.9577
35	12	23	34	12	22	A	0	221367.050	-0.026	218.1603
35	9	27	34	9	26	E	0	221418.686	-0.028	207.2272
35	9	27	34	9	26	A	0	221420.838	-0.023	207.2204
32	20	12	32	19	13	E	0	221430.594	-0.072	244.6233

J'	K'_a	K'_c	J''	K''_a	K''_c	Sym.	$v_{t_{\mathrm{CH_3}}}$	$\nu_{\mathrm{Obs.}}$	$\nu_{\mathrm{Obs.}} - \nu_{\mathrm{Calc.}}$	E_{low}
32	20	12	32	19	13	A	0	221478.378	-0.013	244.6244
32	20	13	32	19	14	A	0	221478.378	-0.013	244.6244
49	3	46	49	2	47	A	0	221604.203	0.074	296.4584
49	4	46	49	3	47	A	0	221604.203	0.074	296.4584
54	6	48	54	5	49	E	1	221623.994	0.089	495.1570
54	7	48	54	6	49	E	1	221623.994	0.086	495.1570
54	6	48	54	5	49	E	0	221626.280	0.010	366.8590
54	7	48	54	6	49	E	0	221626.280	0.008	366.8590
54	6	48	54	5	49	A	0	221648.746	0.012	366.8538
54	7	48	54	6	49	A	0	221648.746	0.009	366.8538
31	20	12	31	19	13	E	0	221652.210	0.079	238.0078
31	20	11	31	19	12	A	0	221670.578	-0.039	238.0201
31	20	12	31	19	13	A	0	221670.578	-0.039	238.0201
49	3	46	49	2	47	E	1	221702.019	-0.137	424.7660
49	4	46	49	3	47	E	1	221702.019	-0.137	424.7660
55	21	34	55	20	35	E	0	221777.774	-0.054	462.5798
30	20	10	30	19	11	E	0	221797.571	-0.042	231.6249
55	21	35	55	20	36	E	0	221804.064	-0.047	462.5699
30	20	10	30	19	11	A	0	221845.098	-0.106	231.6260
30	20	11	30	19	12	A	0	221845.098	-0.106	231.6260
29	20	9	29	19	10	E	0	221955.777	-0.034	225.4405
29	20	10	29	19	11	E	0	221985.247	0.070	225.4293
29	20	9	29	19	10	A	0	222003.310	-0.032	225.4416
29	20	10	29	19	11	A	0	222003.310	-0.032	225.4416
28	20	8	28	19	9	E	0	222098.629	-0.055	219.4656
28	20	9	28	19	10	E	0	222128.116	-0.017	219.4543
28	20	8	28	19	9	A	0	222146.128	-0.032	219.4667
28	20	9	28	19	10	A	0	222146.128	-0.032	219.4667
38	5	33	37	6	32	A	1	222237.952	0.003	346.1121
38	6	33	37	6	32	A	1	222242.611	0.030	346.1121
38	5	33	37	5	32	A	1	222246.619	-0.005	346.1118
38	6	33	37	5	32	A	1	222251.274	0.017	346.1118
37	6	31	36	7	30	A	1	222261.758	0.037	343.1743
39	4	35	38	4	34	A	1	222273.130	-0.075	348.5497
39	4	35	38	5	34	A	1	222273.130	0.166	348.5497
39	5	35	38	4	34	A	1	222273.130	-0.198	348.5497
39	5	35	38	5	34	A	1	222273.130	0.044	348.5497
27	20	7	27	19	8	A	0	222274.665	-0.069	213.7009
27	20	8	27	19	9	A	0	222274.665	-0.069	213.7009
38	5	33	37	6	32	E	1	222378.716	-0.088	345.8742
38	6	33	37	6	32	E	1	222384.226	0.047	345.8742
39	4	35	38	4	34	E	1	222388.780	-0.147	348.2985
39	4	35	38	5	34	E	1	222388.780	0.138	348.2985
39	5	35	38	4	34	E	1	222388.780	-0.293	348.2985
39	5	35	38	5	34	E	1	222388.780	-0.008	348.2985
26	20	6	26	19	7	A	0	222390.137	0.045	208.1439
26	20	7	26	19	8	A	0	222390.137	0.045	208.1439
38	6	33	37	5	32	E	1	222394.146	-0.037	345.8739
40	3	37	39	3	36	A	1	222400.818	-0.011	350.5263
40	3	37	39	4	36	A	1	222400.818	-0.006	350.5263
40	4	37	39	3	36	A	1	222400.818	-0.013	350.5263
40	4	37	39	4	36	A	1	222400.818	-0.009	350.5263
38	5	33	37	6	32	E	0	222407.738	-0.006	217.5507
37	6	31	36	7	30	E	1	222415.748	0.095	342.9432
38	6	33	37	6	32	A	0	222417.216	0.049	217.5433
38	5	33	37	5	32	E	0	222417.216	0.022	217.5504
38	6	33	37	5	32	E	0	222422.162	-0.097	217.5504
39	4	35	38	4	34	E	0	222423.424	-0.026	219.9763
39	5	35	38	5	34	E	0	222423.424	0.104	219.9763
39	4	35	38	4	34	A	0	222426.863	-0.147	219.9685
39	4	35	38	5	34	A	0	222426.863	0.121	219.9685
39	5	35	38	4	34	A	0	222426.863	-0.284	219.9685
39	5	35	38	5	34	A	0	222426.863	-0.016	219.9685
37	6	31	36	7	30	E	0	222443.512	-0.049	214.6205
37	6	31	36	7	30	A	0	222448.197	-0.009	214.6133
37	6	31	36	6	30	A	1	222469.646	0.125	343.1674
40	3	37	39	3	36	E	1	222492.143	-0.063	350.2556
40	3	37	39	4	36	E	1	222492.143	-0.057	350.2556
40	4	37	39	3	36	E	1	222492.143	-0.065	350.2556
40	4	37	39	4	36	E	1	222492.143	-0.060	350.2556
40	3	37	39	3	36	E	0	222531.678	-0.013	221.9366
40	4	37	39	4	36	E	0	222531.678	-0.010	221.9366
40	3	37	39	3	36	A	0	222534.483	-0.015	221.9281
40	3	37	39	4	36	A	0	222534.483	-0.010	221.9281
40	4	37	39	3	36	A	0	222534.483	-0.018	221.9281
40	4	37	39	4	36	A	0	222534.483	-0.013	221.9281
37	7	31	36	7	30	E	1	222549.290	-0.055	342.9432
41	2	39	40	2	38	A	1	222579.392	-0.014	352.0673
41	2	39	40	3	38	A	1	222579.392	-0.014	352.0673
41	3	39	40	2	38	A	1	222579.392	-0.014	352.0673
41	3	39	40	3	38	A	1	222579.392	-0.014	352.0673
37	7	31	36	6	30	A	1	222587.412	0.040	343.1674
24	8	17	23	7	16	E	0	222636.032	-0.061	134.3914
41	2	39	40	2	38	E	1	222646.064	-0.019	351.7711
41	2	39	40	3	38	E	1	222646.064	-0.019	351.7711
41	3	39	40	2	38	E	1	222646.064	-0.019	351.7711
41	3	39	40	3	38	E	1	222646.064	-0.019	351.7711
41	2	39	40	2	38	E	0	222690.352	-0.016	223.4570
41	3	39	40	3	38	E	0	222690.352	-0.016	223.4570
41	2	39	40	2	38	A	0	222692.402	-0.013	223.4477
41	2	39	40	3	38	A	0	222692.402	-0.013	223.4477
41	3	39	40	2	38	A	0	222692.402	-0.013	223.4477
41	3	39	40	3	38	A	0	222692.402	-0.013	223.4477
42	1	41	41	1	40	A	1	222784.509	0.037	353.1900
42	1	41	41	2	40	A	1	222784.509	0.037	353.1900
42	2	41	41	1	40	A	1	222784.509	0.037	353.1900
42	2	41	41	2	40	A	1	222784.509	0.037	353.1900
56	7	49	56	6	50	A	0	222813.849	0.013	395.3911
56	8	49	56	7	50	A	0	222813.849	-0.001	395.3911
42	1	41	41	1	40	E	1	222824.728	-0.003	352.8621
42	1	41	41	2	40	E	1	222824.728	-0.003	352.8621
42	2	41	41	1	40	E	1	222824.728	-0.003	352.8621
42	2	41	41	2	40	E	1	222824.728	-0.003	352.8621
13	13	0	12	12	0	E	0	222831.380	-0.069	110.7238
13	13	1	12	12	0	A	0	222869.202	0.010	110.7148
13	13	0	12	12	1	A	0	222869.202	0.010	110.7148
42	1	41	41	1	40	E	0	222874.131	-0.039	224.5546
42	2	41	41	2	40	E	0	222874.131	-0.039	224.5546
42	1	41	41	1	40	A	0	222875.412	0.005	224.5444
42	1	41	41	2	40	A	0	222875.412	0.005	224.5444
42	2	41	41	1	40	A	0	222875.412	0.005	224.5444
42	2	41	41	2	40	A	0	222875.412	0.005	224.5444
28	8	21	27	7	20	A	0	222993.559	-0.035	156.7761
43	0	43	42	0	42	A	1	223002.876	0.073	353.9067
43	0	43	42	1	42	A	1	223002.876	0.073	353.9067
43	1	43	42	0	42	A	1	223002.876	0.073	353.9067

J'	K'_a	K'_c	J''	K''_a	K''_c	Sym.	$v_{t_{CH_3}}$	$\nu_{Obs.}$	$\nu_{Obs.} - \nu_{Calc.}$	E_{low}
43	1	43	42	1	42	A	1	223002.876	0.073	353.9067
43	0	43	42	0	42	E	1	223013.827	-0.021	353.5404
43	1	43	42	1	42	E	1	223013.827	-0.021	353.5404
36	20	16	35	20	15	E	0	223036.532	-0.045	273.0640
43	0	43	42	0	42	A	0	223069.340	-0.186	225.2299
43	0	43	42	1	42	A	0	223069.340	-0.186	225.2299
43	1	43	42	0	42	A	0	223069.340	-0.186	225.2299
43	1	43	42	1	42	A	0	223069.340	-0.186	225.2299
43	0	43	42	0	42	E	0	223069.340	0.161	225.2413
43	1	43	42	1	42	E	0	223069.340	0.161	225.2413
51	4	47	51	3	48	A	0	223524.069	0.094	322.2862
51	4	47	51	4	48	A	0	223524.069	0.094	322.2862
51	5	47	51	3	48	A	0	223524.069	0.094	322.2862
51	5	47	51	4	48	A	0	223524.069	0.094	322.2862
36	18	18	35	18	17	A	0	223636.560	-0.009	258.7323
36	18	19	35	18	18	A	0	223636.560	-0.009	258.7323
53	21	32	53	20	33	E	0	223654.568	0.037	439.7116
58	8	50	58	7	51	E	0	223686.146	-0.031	424.9904
58	9	50	58	8	51	E	0	223686.146	-0.098	424.9904
53	21	32	53	20	33	A	0	223704.175	0.133	439.7128
15	12	3	14	11	4	A	0	223727.600	-0.006	111.8793
15	12	4	14	11	3	A	0	223727.600	-0.006	111.8793
36	17	20	35	17	19	A	0	224024.415	-0.175	252.1607
36	17	19	35	17	18	A	0	224024.415	-0.175	252.1607
36	17	20	35	17	19	E	0	224026.887	0.152	252.1503
17	11	7	16	10	7	E	0	224481.686	-0.066	114.2738
36	16	21	35	16	20	A	0	224494.530	-0.091	245.9920
17	11	6	16	10	7	A	0	224528.845	0.023	114.2752
52	21	32	52	20	33	A	0	224548.526	-0.030	428.6110
36	15	21	35	15	20	A	0	225074.876	-0.059	240.2328
36	15	21	35	15	20	E	0	225076.364	-0.010	240.2382
36	15	22	35	15	21	E	0	225077.977	-0.044	240.2249
19	10	10	18	9	10	E	0	225092.720	0.025	117.9304
21	9	13	20	8	12	A	0	225097.020	0.144	122.9026
19	10	9	18	9	9	E	0	225115.505	-0.014	117.9421
19	10	10	18	9	9	A	0	225140.016	0.017	117.9301
19	10	9	18	9	10	A	0	225141.493	-0.037	117.9300
21	9	12	20	8	12	E	0	225148.773	-0.195	122.9133
34	9	25	33	9	24	A	0	225185.860	-0.007	200.9683
53	5	48	53	4	49	A	0	225217.567	0.044	349.1931
53	6	48	53	5	49	A	0	225217.567	0.044	349.1931
53	5	48	53	4	49	E	1	225233.494	-0.090	477.4979
53	6	48	53	5	49	E	1	225233.494	-0.091	477.4979
21	9	12	20	8	13	A	0	225276.473	-0.030	122.8974
50	3	47	50	2	48	A	1	225565.805	-0.177	434.0057
50	4	47	50	3	48	A	1	225565.805	-0.177	434.0057
36	14	23	35	14	22	A	0	225807.353	-0.110	234.8927
36	14	22	35	14	21	A	0	225810.475	-0.006	234.8927
36	14	22	35	14	21	E	0	225810.475	-0.100	234.8996
36	14	23	35	14	22	E	0	225812.878	-0.047	234.8863
50	21	29	50	20	30	E	0	226021.300	-0.037	407.0663
50	21	30	50	20	31	E	0	226050.366	-0.032	407.0562
36	8	29	35	7	28	A	0	226064.715	0.015	211.0280
50	21	29	50	20	30	A	0	226070.287	0.014	407.0678
50	3	47	50	2	48	E	0	226520.972	-0.025	305.4343
50	4	47	50	3	48	E	0	226520.972	-0.025	305.4343
50	3	47	50	2	48	A	0	226554.546	0.112	305.4257
50	4	47	50	3	48	A	0	226554.546	0.112	305.4257
55	6	49	55	5	50	E	1	226647.955	-0.025	505.4738
55	7	49	55	6	50	E	1	226647.955	-0.026	505.4738
23	8	15	22	7	16	E	0	226669.402	-0.052	129.2426
55	6	49	55	5	50	A	0	226673.230	-0.039	377.1729
55	7	49	55	6	50	A	0	226673.230	-0.040	377.1729
49	21	28	49	20	29	E	0	226706.291	0.004	396.6233
36	13	24	35	13	23	A	0	226753.637	-0.152	229.9858
49	21	28	49	20	29	A	0	226754.935	-0.085	396.6249
36	13	24	35	13	23	E	0	226773.847	-0.162	229.9816
36	13	23	35	13	22	E	0	226775.125	-0.107	229.9946
39	5	34	38	6	33	A	1	227343.171	0.077	353.5253
39	6	34	38	6	33	A	1	227345.766	0.216	353.5253
39	5	34	38	5	33	A	1	227347.765	0.038	353.5251
39	6	34	38	5	33	A	1	227350.042	-0.140	353.5251
40	4	36	39	4	35	A	1	227390.470	-0.027	355.9639
40	4	36	39	5	35	A	1	227390.470	0.096	355.9639
40	5	36	39	4	35	A	1	227390.470	-0.089	355.9639
40	5	36	39	5	35	A	1	227390.470	0.034	355.9639
38	6	32	37	7	31	A	1	227394.340	0.232	350.5921
38	7	32	37	7	31	A	1	227460.226	0.006	350.5921
39	5	34	38	6	33	E	1	227482.582	-0.021	353.2922
39	6	34	38	6	33	E	1	227485.365	-0.106	353.2922
39	5	34	38	5	33	E	1	227487.904	-0.075	353.2920
39	6	34	38	5	33	E	1	227490.765	-0.082	353.2920
40	4	36	39	4	35	E	1	227505.210	-0.047	355.7166
40	4	36	39	5	35	E	1	227505.210	0.098	355.7166
40	5	36	39	4	35	E	1	227505.210	-0.122	355.7166
40	5	36	39	5	35	E	1	227505.210	0.024	355.7166
38	6	32	37	6	31	A	1	227512.097	0.138	350.5882
39	5	34	38	6	33	E	0	227513.530	-0.016	224.9696
39	6	34	38	6	33	E	0	227516.257	0.017	224.9696
39	5	34	38	6	33	A	0	227517.805	-0.036	224.9623
39	6	34	38	5	33	E	0	227521.337	0.033	224.9694
39	5	34	38	5	33	A	0	227522.897	-0.034	224.9621
41	3	38	40	3	37	A	1	227524.284	-0.035	357.9448
41	3	38	40	4	37	A	1	227524.284	-0.033	357.9448
41	4	38	40	3	37	A	1	227524.284	-0.036	357.9448
41	4	38	40	4	37	A	1	227524.284	-0.034	357.9448
39	6	34	38	5	33	A	0	227525.624	-0.016	224.9621
40	4	36	39	4	35	E	0	227541.571	-0.041	227.3956
40	5	36	39	5	35	E	0	227541.571	0.026	227.3956
40	4	36	39	4	35	A	0	227545.107	-0.035	227.3878
40	4	36	39	5	35	A	0	227545.107	0.102	227.3878
40	5	36	39	4	35	A	0	227545.107	-0.105	227.3878
40	5	36	39	5	35	A	0	227545.107	0.032	227.3878
38	6	32	37	7	31	E	1	227552.695	0.019	350.3667
52	4	48	52	3	49	A	1	227572.818	0.501	460.2978
52	5	48	52	4	49	A	1	227572.818	0.501	460.2978
38	7	32	37	6	31	A	1	227578.319	0.248	350.5882
38	6	32	37	7	31	E	0	227580.204	-0.014	222.0446
38	6	32	37	7	31	A	0	227585.039	-0.010	222.0376
41	3	38	40	3	37	E	1	227615.168	-0.019	357.6771
41	3	38	40	4	37	E	1	227615.168	-0.016	357.6771
41	4	38	40	3	37	E	1	227615.168	-0.020	357.6771
41	4	38	40	4	37	E	1	227615.168	-0.017	357.6771
38	7	32	37	7	31	E	1	227628.222	0.075	350.3667
38	7	32	37	7	31	E	0	227651.795	0.049	222.0446
41	3	38	40	3	37	E	0	227656.295	-0.007	229.3594

J'	K'_a	K'_c	J''	K''_a	K''_c	Sym.	$v_{t_{CH_3}}$	$\nu_{Obs.}$	$\nu_{Obs.} - \nu_{Calc.}$	E_{low}
41	4	38	40	4	37	E	0	227656.295	-0.005	229.3594
41	3	38	40	3	37	A	0	227659.093	-0.000	229.3511
41	3	38	40	4	37	A	0	227659.093	0.002	229.3511
41	4	38	40	3	37	A	0	227659.093	-0.001	229.3511
41	4	38	40	4	37	A	0	227659.093	0.001	229.3511
42	2	40	41	2	39	A	1	227705.324	0.001	359.4918
42	2	40	41	3	39	A	1	227705.324	0.001	359.4918
42	3	40	41	2	39	A	1	227705.324	0.001	359.4918
42	3	40	41	3	39	A	1	227705.324	0.001	359.4918
38	7	32	37	6	31	E	1	227761.934	0.094	350.3622
42	2	40	41	2	39	E	1	227771.729	-0.017	359.1978
42	2	40	41	3	39	E	1	227771.729	-0.017	359.1978
42	3	40	41	2	39	E	1	227771.729	-0.017	359.1978
42	3	40	41	3	39	E	1	227771.729	-0.017	359.1978
38	7	32	37	6	31	E	0	227778.841	0.013	222.0404
38	7	32	37	6	31	A	0	227784.580	0.030	222.0333
57	7	50	57	6	51	E	1	227810.997	0.044	534.5143
57	8	50	57	7	51	E	1	227810.997	0.036	534.5143
42	2	40	41	2	39	E	0	227817.529	-0.004	230.8851
42	3	40	41	3	39	E	0	227817.529	-0.004	230.8851
42	2	40	41	2	39	A	0	227819.581	0.009	230.8760
42	2	40	41	3	39	A	0	227819.581	0.009	230.8760
42	3	40	41	2	39	A	0	227819.581	0.009	230.8760
42	3	40	41	3	39	A	0	227819.581	0.009	230.8760
43	1	42	42	1	41	A	1	227911.284	0.023	360.6213
43	1	42	42	2	41	A	1	227911.284	0.023	360.6213
43	2	42	42	1	41	A	1	227911.284	0.023	360.6213
43	2	42	42	2	41	A	1	227911.284	0.023	360.6213
43	1	42	42	1	41	E	1	227951.377	-0.021	360.2947
43	1	42	42	2	41	E	1	227951.377	-0.021	360.2947
43	2	42	42	1	41	E	1	227951.377	-0.021	360.2947
43	2	42	42	2	41	E	1	227951.377	-0.021	360.2947
36	12	25	35	12	24	E	0	227985.043	-0.061	225.5328
43	1	42	42	1	41	E	0	228002.214	-0.038	231.9889
43	2	42	42	2	41	E	0	228002.214	-0.038	231.9889
43	1	42	42	1	41	A	0	228003.490	0.005	231.9787
43	1	42	42	2	41	A	0	228003.490	0.005	231.9787
43	2	42	42	1	41	A	0	228003.490	0.005	231.9787
43	2	42	42	2	41	A	0	228003.490	0.005	231.9787
44	0	44	43	0	43	A	1	228129.996	0.094	361.3453
44	0	44	43	1	43	A	1	228129.996	0.094	361.3453
44	1	44	43	0	43	A	1	228129.996	0.094	361.3453
44	1	44	43	1	43	A	1	228129.996	0.094	361.3453
44	0	44	43	0	43	E	1	228140.829	-0.026	360.9793
44	1	44	43	1	43	E	1	228140.829	-0.026	360.9793
44	0	44	43	0	43	A	0	228197.697	-0.191	232.6707
44	0	44	43	1	43	A	0	228197.697	-0.191	232.6707
44	1	44	43	0	43	A	0	228197.697	-0.191	232.6707
44	1	44	43	1	43	A	0	228197.697	-0.191	232.6707
44	0	44	43	0	43	E	0	228197.697	0.153	232.6821
44	1	44	43	1	43	E	0	228197.697	0.153	232.6821
39	9	30	38	10	29	A	0	228366.605	-0.066	241.5288
52	4	48	52	3	49	E	0	228460.929	0.039	331.7678
52	5	48	52	4	49	E	0	228460.929	0.039	331.7678
35	10	25	34	10	24	E	0	228463.886	0.005	210.8962
46	21	25	46	20	26	E	0	228493.514	-0.059	366.6007
46	21	26	46	20	27	E	0	228524.251	0.053	366.5905
46	21	26	46	20	27	A	0	228541.726	-0.024	366.6026
46	21	25	46	20	26	A	0	228541.726	-0.023	366.6026
37	7	30	36	7	29	E	0	228694.472	-0.040	218.5071
37	23	15	36	23	14	A	0	228698.103	-0.200	304.9244
37	23	14	36	23	13	A	0	228698.103	-0.200	304.9244
37	23	14	36	23	13	E	0	228699.263	0.001	304.9173
37	7	30	36	7	29	A	0	228702.664	-0.001	218.4998
37	7	30	36	7	29	E	1	228704.332	-0.070	346.8236
59	8	51	59	7	52	E	1	228706.699	0.189	564.6129
59	9	51	59	8	52	E	1	228706.699	0.150	564.6129
59	8	51	59	7	52	A	0	228814.650	-0.032	436.3248
59	9	51	59	8	52	A	0	228814.650	-0.068	436.3248
61	22	39	61	21	40	E	0	228879.594	-0.035	543.7168
37	22	16	36	22	15	E	0	228893.563	0.032	296.3814
61	22	40	61	21	41	E	0	228907.520	-0.076	543.7078
61	22	39	61	21	40	A	0	228926.591	-0.047	543.7185
14	13	2	13	12	2	E	0	228973.249	0.020	113.3788
14	13	1	13	12	1	E	0	228979.344	-0.058	113.3923
45	21	24	45	20	25	E	0	229009.058	-0.043	357.0266
14	13	2	13	12	1	A	0	229017.131	0.002	113.3833
14	13	1	13	12	2	A	0	229017.131	0.002	113.3833
45	21	25	45	20	26	E	0	229040.003	-0.044	357.0164
45	21	25	45	20	26	A	0	229057.049	-0.062	357.0286
45	21	24	45	20	25	A	0	229057.049	-0.061	357.0286
37	21	17	36	21	16	A	0	229115.342	-0.120	288.2550
37	21	16	36	21	15	A	0	229115.342	-0.120	288.2550
37	21	16	36	21	15	E	0	229116.634	0.018	288.2509
36	11	26	35	11	25	A	0	229137.910	-0.026	221.5450
36	11	26	35	11	25	E	0	229145.426	0.001	221.5495
36	10	27	35	10	26	A	0	229280.066	-0.079	217.9721
36	10	27	35	10	26	E	0	229281.298	0.050	217.9781
49	2	47	49	1	48	E	0	229307.524	-0.164	288.8181
49	3	47	49	2	48	E	0	229307.524	-0.164	288.8181
61	9	52	61	8	53	E	1	229315.780	0.335	595.7627
61	10	52	61	9	53	E	1	229315.780	0.163	595.7627
49	2	47	49	1	48	A	0	229345.320	0.141	288.8083
49	3	47	49	2	48	A	0	229345.320	0.141	288.8083
37	20	17	36	20	16	A	0	229372.960	-0.040	280.5063
37	20	18	36	20	17	A	0	229372.960	-0.040	280.5063
37	20	18	36	20	17	E	0	229374.306	-0.009	280.4935
61	9	52	61	8	53	A	0	229465.213	0.097	467.4837
61	10	52	61	9	53	A	0	229465.213	-0.060	467.4837
44	21	24	44	20	25	E	0	229519.760	0.013	347.6580
44	21	24	44	20	25	A	0	229536.305	-0.054	347.6703
44	21	23	44	20	24	A	0	229536.305	-0.054	347.6703
37	19	19	36	19	18	A	0	229673.532	-0.026	273.1509
37	19	18	36	19	17	A	0	229673.532	-0.026	273.1509
37	19	18	36	19	17	E	0	229674.979	0.075	273.1500
16	12	5	15	11	5	E	0	229820.108	0.010	114.9626
16	12	4	15	11	4	E	0	229831.816	0.032	114.9757
16	12	4	15	11	5	A	0	229865.879	0.017	114.9656
16	12	5	15	11	4	A	0	229865.879	0.017	114.9656
43	21	22	43	20	23	E	0	229933.874	-0.065	338.5249
37	8	30	36	7	29	E	0	229947.867	-0.065	218.5071
37	8	30	36	7	29	A	0	229960.876	0.009	218.4998
43	21	23	43	20	24	E	0	229965.465	0.012	338.5147
43	21	23	43	20	24	A	0	229981.618	-0.026	338.5270
43	21	22	43	20	23	A	0	229981.618	-0.026	338.5270
37	18	19	36	18	18	A	0	230028.194	-0.008	266.1920

J'	K_a'	K_c'	J''	K_a''	K_c''	Sym.	$v_{t_{CH_3}}$	$\nu_{Obs.}$	$\nu_{Obs.} - \nu_{Calc.}$	E_{low}
37	18	20	36	18	19	A	0	230028.194	-0.008	266.1920
37	18	19	36	18	18	E	0	230029.816	0.175	266.1927
70	23	48	70	22	49	A	0	230080.268	0.030	676.8002
60	22	38	60	21	39	E	0	230093.758	-0.011	530.8674
60	22	39	60	21	40	E	0	230122.359	-0.068	530.8584
60	22	38	60	21	39	A	0	230142.076	0.059	530.8693
60	22	39	60	21	40	A	0	230145.510	-0.121	530.8691
54	5	49	54	4	50	E	0	230180.518	-0.015	359.1810
54	6	49	54	5	50	E	0	230180.518	-0.015	359.1810
54	5	49	54	4	50	E	1	230223.216	-0.010	487.4775
54	6	49	54	5	50	E	1	230223.216	-0.010	487.4775
42	21	21	42	20	22	E	0	230347.316	-0.095	329.5962
42	21	22	42	20	23	E	0	230379.161	-0.013	329.5860
42	21	22	42	20	23	A	0	230394.940	-0.034	329.5985
42	21	21	42	20	22	A	0	230394.940	-0.034	329.5985
37	17	21	36	17	20	A	0	230452.112	-0.007	259.6333
37	17	20	36	17	19	E	0	230453.500	-0.145	259.6356
37	17	21	36	17	20	E	0	230454.580	0.109	259.6230
51	3	48	51	2	49	A	1	230494.166	-0.076	443.1399
51	4	48	51	3	49	A	1	230494.166	-0.076	443.1399
18	11	8	17	10	8	E	0	230586.447	0.018	117.7842
18	11	7	17	10	7	E	0	230603.597	-0.078	117.7967
18	11	7	17	10	8	A	0	230633.588	0.009	117.7855
41	21	20	41	20	21	E	0	230730.646	-0.159	320.8817
22	9	14	21	8	13	A	0	230763.107	-0.211	127.3068
41	21	21	41	20	22	E	0	230763.107	0.309	320.8714
41	21	21	41	20	22	A	0	230778.201	-0.036	320.8839
41	21	20	41	20	21	A	0	230778.201	-0.036	320.8839
22	9	13	21	8	13	E	0	230866.770	-0.040	127.3163
37	16	22	36	16	21	A	0	230966.861	-0.007	253.4803
37	16	21	36	16	20	E	0	230968.347	-0.140	253.4842
37	16	22	36	16	21	E	0	230969.666	-0.075	253.4712
22	9	14	21	8	14	E	0	230976.118	-0.059	127.3012
40	21	19	40	20	20	E	0	231085.854	-0.038	312.3807
20	10	11	19	9	11	E	0	231107.521	0.001	121.8756
40	21	20	40	20	21	E	0	231118.163	0.066	312.3705
20	10	10	19	9	10	E	0	231130.630	-0.023	121.8872
40	21	20	40	20	21	A	0	231133.177	-0.023	312.3830
40	21	19	40	20	20	A	0	231133.177	-0.023	312.3830
22	9	13	21	8	14	A	0	231149.300	0.003	127.2957
20	10	11	19	9	10	A	0	231153.676	-0.005	121.8753
20	10	10	19	9	11	A	0	231158.030	0.122	121.8751
36	11	25	35	11	24	A	0	231165.643	0.045	221.6441
36	11	25	35	11	24	E	0	231169.699	-0.000	221.6495
59	22	37	59	21	38	E	0	231227.841	-0.013	518.2431
59	22	38	59	21	39	E	0	231257.134	-0.071	518.2341
59	22	37	59	21	38	A	0	231276.678	0.075	518.2451
59	22	38	59	21	39	A	0	231278.284	-0.163	518.2451
39	21	18	39	20	19	E	0	231414.317	-0.024	304.0929
39	21	19	39	20	20	E	0	231446.765	0.026	304.0826
39	21	19	39	20	20	A	0	231461.515	-0.017	304.0953
39	21	18	39	20	19	A	0	231461.515	-0.017	304.0953
51	3	48	51	2	49	E	0	231469.366	0.052	314.5726
51	4	48	51	3	49	E	0	231469.366	0.052	314.5726
51	3	48	51	2	49	A	0	231503.598	0.166	314.5641
51	4	48	51	3	49	A	0	231503.598	0.166	314.5641
37	15	23	36	15	22	A	0	231604.354	0.061	247.7405
37	15	22	36	15	21	E	0	231606.236	0.035	247.7459
37	15	23	36	15	22	E	0	231608.024	-0.011	247.7327
56	6	50	56	5	51	E	1	231666.510	-0.043	515.9613
56	7	50	56	6	51	E	1	231666.510	-0.044	515.9613
56	6	50	56	5	51	E	0	231669.083	-0.018	387.6676
56	7	50	56	6	51	E	0	231669.083	-0.018	387.6676
56	6	50	56	5	51	A	0	231692.488	-0.018	387.6627
56	7	50	56	6	51	A	0	231692.488	-0.019	387.6627
38	21	17	38	20	18	E	0	231717.689	-0.035	296.0177
38	21	18	38	20	19	E	0	231750.323	0.023	296.0075
38	21	18	38	20	19	A	0	231764.786	-0.019	296.0202
38	21	17	38	20	18	A	0	231764.786	-0.019	296.0202
69	23	46	69	22	47	E	0	231783.503	-0.057	661.9455
69	23	46	69	22	47	A	0	231797.925	-0.015	661.9485
69	23	47	69	22	48	A	0	231898.574	-0.074	661.9447
37	21	16	37	20	17	E	0	231997.464	-0.065	288.1548
37	21	17	37	20	18	E	0	232030.303	0.037	288.1446
37	21	17	37	20	18	A	0	232044.479	-0.027	288.1573
37	21	16	37	20	17	A	0	232044.479	-0.027	288.1573
38	7	31	37	8	30	A	1	232094.748	0.034	354.7138
38	8	30	37	9	29	E	1	232188.281	-0.153	358.2619
38	7	31	37	8	30	E	1	232198.064	-0.020	354.4960
35	9	26	34	9	25	A	0	232203.123	0.066	208.4797
35	9	26	34	9	25	E	0	232204.590	0.088	208.4882
38	7	31	37	8	30	E	0	232253.640	0.023	226.1773
36	21	15	36	20	16	E	0	232255.043	-0.121	280.5037
38	7	31	37	8	30	A	0	232256.301	0.017	226.1705
36	21	16	36	20	17	E	0	232287.991	-0.058	280.4935
36	21	16	36	20	17	A	0	232302.007	-0.038	280.5063
36	21	15	36	20	16	A	0	232302.007	-0.038	280.5063
38	8	30	37	9	29	A	0	232403.357	0.029	229.9412
37	14	24	36	14	23	A	0	232412.520	-0.117	242.4248
37	14	23	36	14	22	A	0	232418.798	-0.093	242.4250
37	14	24	36	14	23	E	0	232420.083	0.011	242.4186
36	8	28	35	8	27	E	0	232422.089	0.003	214.0154
36	8	28	35	8	27	A	0	232434.455	0.011	214.0071
40	5	35	39	6	34	A	1	232449.453	-0.015	361.1087
40	6	35	39	6	34	A	1	232450.710	-0.052	361.1087
40	5	35	39	5	34	A	1	232451.905	-0.019	361.1086
40	6	35	39	5	34	A	1	232453.214	-0.003	361.1086
35	21	14	35	20	15	E	0	232491.878	-0.088	273.0640
39	6	33	38	7	32	A	1	232505.263	0.045	358.1793
41	4	37	40	4	36	A	1	232508.551	-0.048	363.5488
41	4	37	40	5	36	A	1	232508.551	0.014	363.5489
41	5	37	40	4	36	A	1	232508.551	-0.079	363.5488
41	5	37	40	5	36	A	1	232508.551	-0.017	363.5489
35	21	15	35	20	16	E	0	232525.012	0.028	273.0538
35	21	15	35	20	16	A	0	232538.804	0.049	273.0666
35	21	14	35	20	15	A	0	232538.804	0.049	273.0666
39	7	33	38	7	32	A	1	232541.939	-0.000	358.1793
39	6	33	38	6	32	A	1	232571.358	0.028	358.1771
40	5	35	39	6	34	E	1	232587.425	-0.120	360.8803
40	5	35	39	5	34	E	1	232590.451	0.039	360.8802
40	6	35	39	5	34	E	1	232591.914	-0.018	360.8802
39	7	33	38	6	32	A	1	232608.080	0.028	358.1771
40	5	35	39	6	34	E	0	232620.509	-0.021	232.5587
40	5	35	39	5	34	E	0	232623.466	0.243	232.5586
40	5	35	39	6	34	A	0	232624.722	-0.060	232.5516
40	6	35	39	5	34	E	0	232624.722	0.076	232.5586

J'	K'_a	K'_c	J''	K''_a	K''_c	Sym.	$v_{t_{CH_3}}$	$\nu_{Obs.}$	$\nu_{Obs.} - \nu_{Calc.}$	E_{low}
40	6	35	39	6	34	A	0	232626.188	-0.025	232.5516
37	9	29	36	9	28	E	0	232626.188	-0.055	222.1882
40	5	35	39	5	34	A	0	232627.547	0.058	232.5515
40	6	35	39	5	34	A	0	232628.915	-0.007	232.5515
37	9	29	36	9	28	A	0	232630.262	0.040	222.1815
42	3	39	41	3	38	A	1	232647.941	-0.030	365.5342
42	3	39	41	4	38	A	1	232647.941	-0.028	365.5342
42	4	39	41	3	38	A	1	232647.941	-0.030	365.5342
42	4	39	41	4	38	A	1	232647.941	-0.029	365.5342
41	4	37	40	4	36	E	0	232660.582	-0.028	234.9855
41	5	37	40	5	36	E	0	232660.582	0.006	234.9855
41	4	37	40	4	36	A	0	232664.081	-0.033	234.9779
41	4	37	40	5	36	A	0	232664.081	0.037	234.9779
41	5	37	40	4	36	A	0	232664.081	-0.068	234.9779
41	5	37	40	5	36	A	0	232664.081	0.001	234.9779
39	6	33	38	7	32	E	1	232665.434	-0.085	357.9595
39	6	33	38	7	32	E	0	232694.000	0.163	229.6383
39	6	33	38	7	32	A	0	232698.747	-0.001	229.6314
39	7	33	38	7	32	E	1	232707.805	0.105	357.9595
34	21	13	34	20	14	E	0	232709.231	0.026	265.8353
39	7	33	38	7	32	E	0	232733.687	-0.012	229.6383
42	3	39	41	3	38	E	1	232738.343	0.001	365.2695
42	3	39	41	4	38	E	1	232738.343	0.003	365.2695
42	4	39	41	3	38	E	1	232738.343	0.001	365.2695
42	4	39	41	4	38	E	1	232738.343	0.002	365.2695
39	6	33	38	6	32	E	1	232740.983	-0.007	357.9570
34	21	14	34	20	15	E	0	232742.430	0.087	265.8251
34	21	14	34	20	15	A	0	232755.856	-0.051	265.8379
34	21	13	34	20	14	A	0	232755.856	-0.051	265.8379
39	6	33	38	6	32	A	0	232770.597	-0.009	229.6290
42	3	39	41	3	38	E	0	232781.067	-0.018	236.9532
42	4	39	41	4	38	E	0	232781.067	-0.017	236.9532
42	3	39	41	3	38	A	0	232783.809	-0.052	236.9450
42	3	39	41	4	38	A	0	232783.809	-0.051	236.9450
42	4	39	41	3	38	A	0	232783.809	-0.053	236.9450
42	4	39	41	4	38	A	0	232783.809	-0.052	236.9450
39	7	33	38	6	32	E	0	232805.221	-0.007	229.6359
39	7	33	38	6	32	A	0	232810.615	-0.044	229.6290
43	2	41	42	2	40	A	1	232831.161	-0.023	367.0872
43	2	41	42	3	40	A	1	232831.161	-0.023	367.0872
43	3	41	42	2	40	A	1	232831.161	-0.023	367.0872
43	3	41	42	3	40	A	1	232831.161	-0.023	367.0872
58	7	51	58	6	52	E	1	232866.284	0.053	545.5103
58	8	51	58	7	52	E	1	232866.284	0.049	545.5103
43	2	41	42	2	40	E	1	232897.340	-0.020	366.7954
43	2	41	42	3	40	E	1	232897.340	-0.020	366.7954
43	3	41	42	2	40	E	1	232897.340	-0.020	366.7954
43	3	41	42	3	40	E	1	232897.340	-0.020	366.7954
58	7	51	58	6	52	E	0	232913.872	-0.048	417.2212
58	8	51	58	7	52	E	0	232913.872	-0.051	417.2212
58	7	51	58	6	52	A	0	232933.916	-0.068	417.2174
58	8	51	58	7	52	A	0	232933.916	-0.072	417.2174
33	21	13	33	20	14	E	0	232941.348	0.015	258.8070
43	2	41	42	2	40	E	0	232944.651	0.003	238.4843
43	3	41	42	3	40	E	0	232944.651	0.003	238.4843
43	2	41	42	2	40	A	0	232946.667	-0.012	238.4752
43	2	41	42	3	40	A	0	232946.667	-0.012	238.4752
43	3	41	42	2	40	A	0	232946.667	-0.012	238.4752
43	3	41	42	3	40	A	0	232946.667	-0.012	238.4752
38	8	31	37	8	30	E	1	232984.245	0.013	354.4960
38	8	31	37	8	30	E	0	233004.025	0.039	226.1773
38	8	31	37	8	30	A	0	233009.879	0.208	226.1705
44	1	43	43	1	42	A	1	233037.950	0.018	368.2236
44	1	43	43	2	42	A	1	233037.950	0.018	368.2236
44	2	43	43	1	42	A	1	233037.950	0.018	368.2236
44	2	43	43	2	42	A	1	233037.950	0.018	368.2236
44	1	43	43	1	42	E	1	233077.917	-0.033	367.8984
44	1	43	43	2	42	E	1	233077.917	-0.033	367.8984
44	2	43	43	1	42	E	1	233077.917	-0.033	367.8984
44	2	43	43	2	42	E	1	233077.917	-0.033	367.8984
32	21	11	32	20	12	E	0	233089.734	-0.024	252.0095
32	21	12	32	20	13	E	0	233123.190	0.086	251.9992
44	1	43	43	1	42	E	0	233130.187	-0.032	239.5942
44	2	43	43	2	42	E	0	233130.187	-0.032	239.5942
44	1	43	43	1	42	A	0	233131.462	0.013	239.5841
44	1	43	43	2	42	A	0	233131.462	0.013	239.5841
44	2	43	43	1	42	A	0	233131.462	0.013	239.5841
44	2	43	43	2	42	A	0	233131.462	0.013	239.5841
32	21	12	32	20	13	A	0	233136.215	-0.090	252.0121
32	21	11	32	20	12	A	0	233136.215	-0.090	252.0121
45	0	45	44	0	44	A	1	233256.941	0.067	368.9549
45	0	45	44	1	44	A	1	233256.941	0.067	368.9549
45	1	45	44	0	44	A	1	233256.941	0.067	368.9549
45	1	45	44	1	44	A	1	233256.941	0.067	368.9549
45	0	45	44	0	44	E	1	233267.719	-0.014	368.5893
45	1	45	44	1	44	E	1	233267.719	-0.014	368.5893
57	22	35	57	21	36	E	0	233279.183	-0.022	493.6658
31	21	11	31	20	12	E	0	233288.796	0.044	245.4013
31	21	11	31	20	12	A	0	233301.755	-0.040	245.4143
31	21	10	31	20	11	A	0	233301.755	-0.040	245.4143
45	0	45	44	0	44	A	0	233325.936	-0.186	240.2826
45	0	45	44	1	44	A	0	233325.936	-0.186	240.2826
45	1	45	44	0	44	A	0	233325.936	-0.186	240.2826
45	1	45	44	1	44	A	0	233325.936	-0.186	240.2826
45	0	45	44	0	44	E	0	233325.936	0.155	240.2939
45	1	45	44	1	44	E	0	233325.936	0.155	240.2939
57	22	35	57	21	36	A	0	233328.111	0.145	493.6681
30	21	9	30	20	10	E	0	233405.773	-0.037	239.0232
53	4	49	53	3	50	E	0	233425.221	0.068	341.4131
53	5	49	53	4	50	E	0	233425.221	0.068	341.4131
30	21	10	30	20	11	E	0	233439.391	0.069	239.0130
30	21	10	30	20	11	A	0	233452.172	-0.049	239.0260
30	21	9	30	20	10	A	0	233452.172	-0.049	239.0260
37	13	25	36	13	24	A	0	233455.832	0.107	237.5495
53	4	49	53	3	50	A	0	233455.832	-0.025	341.4059
53	5	49	53	4	50	A	0	233455.832	-0.025	341.4059
37	13	24	36	13	23	E	0	233499.729	0.012	237.5590
38	7	31	37	7	30	E	0	233507.001	-0.036	226.1355
38	7	31	37	7	30	A	0	233514.275	-0.211	226.1285
37	13	24	36	13	23	A	0	233522.686	0.012	237.5518
29	21	8	29	20	9	E	0	233542.193	-0.038	232.8441
29	21	9	29	20	10	E	0	233575.852	0.040	232.8339
28	21	7	28	20	8	E	0	233665.477	-0.055	226.8740
28	21	8	28	20	9	A	0	233711.785	-0.038	226.8767
28	21	7	28	20	8	A	0	233711.785	-0.038	226.8767
60	8	52	60	7	53	E	1	233807.553	0.145	576.1182

J'	K'_a	K'_c	J''	K''_a	K''_c	Sym.	$v_{t_{CH_3}}$	$\nu_{Obs.}$	$\nu_{Obs.} - \nu_{Calc.}$	E_{low}
60	9	52	60	8	53	E	1	233807.553	0.124	576.1182
27	21	7	27	20	8	E	0	233810.496	0.177	221.1022
27	21	7	27	20	8	A	0	233822.828	-0.032	221.1152
27	21	6	27	20	7	A	0	233822.828	-0.032	221.1152
60	8	52	60	7	53	E	0	233900.011	-0.041	447.8356
60	9	52	60	8	53	E	0	233900.011	-0.060	447.8356
26	21	6	26	20	7	E	0	233910.258	0.144	215.5490
60	8	52	60	7	53	A	0	233916.710	-0.098	447.8327
60	9	52	60	8	53	A	0	233916.710	-0.116	447.8327
26	21	6	26	20	7	A	0	233922.574	0.017	215.5620
26	21	5	26	20	6	A	0	233922.574	0.017	215.5620
38	8	31	37	7	30	A	1	233970.357	0.027	354.6746
24	8	16	23	7	17	A	0	234028.163	0.063	134.0836
24	21	4	24	20	5	A	0	234091.136	-0.077	205.0794
24	21	3	24	20	4	A	0	234091.136	-0.077	205.0794
23	21	3	23	20	4	A	0	234161.708	-0.010	200.1495
23	21	2	23	20	3	A	0	234161.708	-0.010	200.1495
56	22	34	56	21	35	E	0	234206.591	0.001	481.7110
56	22	34	56	21	35	A	0	234255.253	0.095	481.7134
38	8	31	37	7	30	E	0	234257.420	0.014	226.1355
38	8	31	37	7	30	A	0	234267.865	-0.007	226.1285
38	8	31	37	7	30	E	1	234293.172	0.032	354.4524
50	2	48	50	1	49	E	1	234427.855	-0.011	425.9117
50	3	48	50	2	49	E	1	234427.855	-0.011	425.9117
62	9	53	62	8	54	E	1	234472.745	0.387	607.7784
62	10	53	62	9	54	E	1	234472.745	0.295	607.7784
37	12	26	36	12	25	A	0	234732.492	-0.040	233.1357
37	12	26	36	12	25	E	0	234756.520	-0.116	233.1376
38	23	16	37	23	15	A	0	234990.544	-0.112	312.5529
38	23	15	37	23	14	A	0	234990.544	-0.112	312.5529
38	23	15	37	23	14	E	0	234991.673	-0.029	312.5459
64	10	54	64	9	55	A	0	235032.037	0.048	512.2190
64	11	54	64	10	55	A	0	235032.037	-0.301	512.2190
55	22	33	55	21	34	E	0	235074.329	-0.027	469.9776
55	22	34	55	21	35	E	0	235106.045	-0.026	469.9685
15	13	3	14	12	3	E	0	235119.551	0.012	116.2544
55	22	33	55	21	34	A	0	235122.727	0.037	469.9802
15	13	2	14	12	2	E	0	235125.687	-0.056	116.2679
15	13	3	14	12	2	A	0	235163.482	0.030	116.2589
15	13	2	14	12	3	A	0	235163.482	0.030	116.2589
55	5	50	55	4	51	A	0	235193.733	0.029	369.3276
55	6	50	55	5	51	A	0	235193.733	0.029	369.3276
38	22	16	37	22	15	A	0	235201.322	-0.065	304.0300
38	22	17	37	22	16	A	0	235201.322	-0.065	304.0300
38	22	16	37	22	15	E	0	235202.507	-0.030	304.0244
55	5	50	55	4	51	E	1	235209.225	-0.117	497.6280
55	6	50	55	5	51	E	1	235209.225	-0.117	497.6280
37	12	25	36	12	24	E	0	235268.909	-0.031	233.1634
37	12	25	36	12	24	A	0	235283.511	-0.004	233.1584
52	3	49	52	2	50	A	1	235421.188	-0.152	452.4450
52	4	49	52	3	50	A	1	235421.188	-0.152	452.4450
38	21	18	37	21	17	A	0	235443.061	-0.066	295.8975
38	21	17	37	21	16	A	0	235443.061	-0.066	295.8975
38	21	17	37	21	16	E	0	235444.335	-0.047	295.8934
37	10	28	36	10	27	A	0	235461.508	-0.011	225.6200
37	10	28	36	10	27	E	0	235461.508	0.028	225.6261
38	20	18	37	20	17	A	0	235722.787	-0.041	288.1573
38	20	19	37	20	18	A	0	235722.787	-0.041	288.1573
38	20	18	37	20	17	E	0	235724.196	0.009	288.1548
37	11	27	36	11	26	A	0	235813.682	-0.018	229.1883
37	11	27	36	11	26	E	0	235819.001	0.036	229.1930
54	22	32	54	21	33	E	0	235886.395	-0.014	458.4648
54	22	33	54	21	34	E	0	235918.630	0.015	458.4557
54	22	32	54	21	33	A	0	235934.487	-0.008	458.4675
17	12	6	16	11	6	E	0	235951.878	0.006	118.2576
17	12	5	16	11	5	E	0	235963.618	-0.009	118.2707
17	12	5	16	11	6	A	0	235997.675	0.004	118.2605
17	12	6	16	11	5	A	0	235997.675	0.004	118.2605
38	19	20	37	19	19	A	0	236049.616	-0.008	280.8120
38	19	19	37	19	18	A	0	236049.616	-0.008	280.8120
38	19	19	37	19	18	E	0	236051.193	0.110	280.8111
23	9	15	22	8	14	A	0	236231.512	0.017	131.9361
23	9	14	22	8	14	E	0	236401.231	0.088	131.9448
52	3	49	52	2	50	E	0	236416.216	-0.180	323.8818
52	4	49	52	3	50	E	0	236416.216	-0.180	323.8818
38	18	20	37	18	19	A	0	236435.754	-0.029	273.8649
38	18	21	37	18	20	A	0	236435.754	-0.029	273.8649
52	3	49	52	2	50	A	0	236451.276	0.090	323.8734
52	4	49	52	3	50	A	0	236451.276	0.090	323.8734
36	10	26	35	10	25	A	0	236537.511	-0.059	218.5097
36	10	26	35	10	25	E	0	236546.993	-0.095	218.5169
52	3	49	52	2	50	E	1	236552.902	-0.133	452.1749
52	4	49	52	3	50	E	1	236552.902	-0.133	452.1749
66	23	44	66	22	45	E	0	236657.914	-0.100	618.7560
66	23	43	66	22	44	A	0	236669.613	0.013	618.7666
19	11	9	18	10	9	E	0	236674.916	0.022	121.5055
53	22	32	53	21	33	E	0	236678.926	-0.045	447.1628
57	6	51	57	5	52	E	1	236680.017	0.020	526.6195
57	7	51	57	6	52	E	1	236680.017	0.020	526.6195
57	6	51	57	5	52	E	0	236682.916	-0.042	398.3280
57	7	51	57	6	52	E	0	236682.916	-0.042	398.3280
37	8	29	36	8	28	E	0	236685.171	-0.051	221.7682
66	23	44	66	22	45	A	0	236686.498	-0.065	618.7660
19	11	8	18	10	8	E	0	236692.242	-0.061	121.5180
53	22	31	53	21	32	A	0	236694.079	-0.070	447.1747
37	8	29	36	8	28	A	0	236697.915	-0.013	221.7603
57	6	51	57	5	52	A	0	236706.806	-0.006	398.3232
57	7	51	57	6	52	A	0	236706.806	-0.006	398.3232
19	11	8	18	10	9	A	0	236722.281	0.119	121.5068
37	8	29	36	8	28	E	1	236739.593	0.078	350.0711
23	9	15	22	8	15	E	0	236781.765	-0.049	131.9201
38	17	22	37	17	21	A	0	236898.235	-0.027	267.3204
38	17	21	37	17	20	E	0	236899.775	-0.139	267.3227
38	17	22	37	17	21	E	0	236900.988	0.150	267.3102
23	9	14	22	8	15	A	0	237021.635	-0.016	131.9137
21	10	12	20	9	12	E	0	237085.005	0.089	126.0357
21	10	11	20	9	11	E	0	237108.333	-0.006	126.0473
21	10	12	20	9	11	A	0	237127.915	-0.022	126.0354
21	10	11	20	9	12	A	0	237138.926	0.053	126.0351
52	22	30	52	21	31	E	0	237357.269	-0.044	436.0981
52	22	31	52	21	32	E	0	237390.373	-0.024	436.0890
52	22	30	52	21	31	A	0	237404.910	-0.002	436.1011
38	16	23	37	16	22	A	0	237461.333	0.005	261.1845
38	16	22	37	16	21	E	0	237463.133	0.035	261.1885
38	16	23	37	16	22	E	0	237464.464	-0.029	261.1755
39	7	32	38	8	31	A	1	237470.170	0.062	362.4790

J'	K'_a	K'_c	J''	K''_a	K''_c	Sym.	$v_{t_{CH_3}}$	$\nu_{Obs.}$	$\nu_{Obs.} - \nu_{Calc.}$	E_{low}
41	5	36	40	6	35	A	1	237557.318	0.012	368.8624
41	6	36	40	5	35	A	1	237559.267	-0.008	368.8624
40	6	34	39	7	33	A	1	237606.060	0.091	365.9361
40	7	34	39	7	33	A	1	237626.098	-0.081	365.9361
42	4	38	41	4	37	A	1	237627.370	-0.012	371.3045
42	4	38	41	5	37	A	1	237627.370	0.019	371.3045
42	5	38	41	4	37	A	1	237627.370	-0.028	371.3045
42	5	38	41	5	37	A	1	237627.370	0.003	371.3045
40	6	34	39	6	33	A	1	237642.743	0.053	365.9349
39	7	32	38	8	31	E	0	237650.186	-0.020	233.9495
39	7	32	38	8	31	A	0	237654.076	-0.019	233.9429
40	7	34	39	6	33	A	1	237662.924	0.024	365.9349
41	5	36	40	6	35	E	1	237693.908	-0.023	368.6386
41	6	36	40	5	35	E	1	237696.263	0.012	368.6385
41	5	36	40	6	35	E	0	237728.993	0.024	240.3182
41	6	36	40	5	35	E	0	237731.113	-0.027	240.3181
41	5	36	40	6	35	A	0	237733.150	-0.028	240.3112
41	6	36	40	5	35	A	0	237735.365	0.004	240.3111
42	4	38	41	4	37	E	1	237740.303	-0.031	371.0648
42	4	38	41	5	37	E	1	237740.303	0.007	371.0648
42	5	38	41	4	37	E	1	237740.303	-0.050	371.0648
42	5	38	41	5	37	E	1	237740.303	-0.013	371.0648
40	6	34	39	7	33	E	1	237766.233	-0.031	365.7218
43	3	40	42	3	39	A	1	237771.721	-0.023	373.2945
43	3	40	42	4	39	A	1	237771.721	-0.023	373.2945
43	4	40	42	3	39	A	1	237771.721	-0.024	373.2945
43	4	40	42	4	39	A	1	237771.721	-0.023	373.2945
42	4	38	41	4	37	E	0	237780.292	-0.023	242.7462
42	5	38	41	5	37	E	0	237780.292	-0.005	242.7462
42	4	38	41	4	37	A	0	237783.771	-0.020	242.7387
42	4	38	41	5	37	A	0	237783.771	0.015	242.7388
42	5	38	41	4	37	A	0	237783.771	-0.038	242.7387
42	5	38	41	5	37	A	0	237783.771	-0.002	242.7388
40	7	34	39	7	33	E	1	237789.605	-0.021	365.7218
40	6	34	39	7	33	E	0	237796.019	-0.038	237.4014
40	6	34	39	7	33	A	0	237800.963	-0.020	237.3947
40	6	34	39	6	33	E	1	237808.469	0.023	365.7204
40	7	34	39	7	33	A	0	237822.908	-0.197	237.3947
40	7	34	39	6	33	E	1	237831.805	-0.003	365.7204
40	6	34	39	6	33	E	0	237835.918	-0.001	237.4001
40	6	34	39	6	33	A	0	237841.117	0.082	237.3934
40	7	34	39	6	33	E	0	237857.919	-0.013	237.4001
43	3	40	42	3	39	E	1	237861.618	-0.014	373.0328
43	3	40	42	4	39	E	1	237861.618	-0.014	373.0328
43	4	40	42	3	39	E	1	237861.618	-0.015	373.0328
43	4	40	42	4	39	E	1	237861.618	-0.014	373.0328
40	7	34	39	6	33	A	0	237863.092	-0.067	237.3934
39	8	32	38	8	31	A	1	237882.201	0.030	362.4790
43	3	40	42	3	39	E	0	237906.006	0.004	244.7180
43	4	40	42	4	39	E	0	237906.006	0.005	244.7180
43	3	40	42	3	39	A	0	237908.758	-0.005	244.7098
43	3	40	42	4	39	A	0	237908.758	-0.005	244.7098
43	4	40	42	3	39	A	0	237908.758	-0.006	244.7098
43	4	40	42	4	39	A	0	237908.758	-0.005	244.7098
44	2	42	43	2	41	A	1	237956.972	-0.006	374.8536
44	2	42	43	3	41	A	1	237956.972	-0.006	374.8536
44	3	42	43	2	41	A	1	237956.972	-0.006	374.8536
44	3	42	43	3	41	A	1	237956.972	-0.006	374.8536
59	7	52	59	6	53	E	0	237962.833	-0.033	428.3902
59	8	52	59	7	53	E	0	237962.833	-0.035	428.3902
59	7	52	59	6	53	A	0	237983.262	-0.064	428.3865
59	8	52	59	7	53	A	0	237983.262	-0.065	428.3865
38	9	30	37	9	29	E	0	238019.498	-0.026	229.9478
44	2	42	43	2	41	E	1	238022.880	-0.032	374.5640
44	2	42	43	3	41	E	1	238022.880	-0.032	374.5640
44	3	42	43	2	41	E	1	238022.880	-0.032	374.5640
44	3	42	43	3	41	E	1	238022.880	-0.032	374.5640
65	23	42	65	22	43	E	0	238028.075	-0.022	604.8290
51	22	30	51	21	31	E	0	238055.903	0.016	425.2338
51	22	30	51	21	31	A	0	238069.819	0.035	425.2460
44	2	42	43	2	41	E	0	238071.688	-0.014	246.2545
44	3	42	43	3	41	E	0	238071.688	-0.014	246.2545
44	2	42	43	2	41	A	0	238073.710	-0.016	246.2455
44	2	42	43	3	41	A	0	238073.710	-0.016	246.2455
44	3	42	43	2	41	A	0	238073.710	-0.016	246.2455
44	3	42	43	3	41	A	0	238073.710	-0.016	246.2455
65	23	43	65	22	44	A	0	238082.258	-0.103	604.8314
39	8	32	38	8	31	E	0	238092.875	0.024	233.9495
39	8	32	38	8	31	A	0	238098.602	-0.007	233.9429
45	1	44	44	1	43	A	1	238164.503	0.022	375.9969
45	1	44	44	2	43	A	1	238164.503	0.022	375.9969
45	2	44	44	1	43	A	1	238164.503	0.022	375.9969
45	2	44	44	2	43	A	1	238164.503	0.022	375.9969
45	1	44	44	1	43	E	1	238204.367	-0.017	375.6730
45	1	44	44	2	43	E	1	238204.367	-0.017	375.6730
45	2	44	44	1	43	E	1	238204.367	-0.017	375.6730
45	2	44	44	2	43	E	1	238204.367	-0.017	375.6730
45	1	44	44	1	43	E	0	238258.038	-0.029	247.3706
45	2	44	44	2	43	E	0	238258.038	-0.029	247.3706
45	1	44	44	1	43	A	0	238259.311	0.018	247.3605
45	1	44	44	2	43	A	0	238259.311	0.018	247.3605
45	2	44	44	1	43	A	0	238259.311	0.018	247.3605
45	2	44	44	2	43	A	0	238259.311	0.018	247.3605
46	0	46	45	0	45	A	1	238383.797	0.081	376.7355
46	0	46	45	1	45	A	1	238383.797	0.081	376.7355
46	1	46	45	0	45	A	1	238383.797	0.081	376.7355
46	1	46	45	1	45	A	1	238383.797	0.081	376.7355
54	4	50	54	3	51	E	0	238387.385	0.006	351.2293
54	5	50	54	4	51	E	0	238387.385	0.006	351.2293
39	7	32	38	7	31	E	1	238392.261	-0.072	362.2413
46	0	46	45	0	45	E	1	238394.471	-0.012	376.3703
46	1	46	45	1	45	E	1	238394.471	-0.012	376.3703
39	7	32	38	7	31	E	0	238400.560	-0.015	233.9244
39	7	32	38	7	31	A	0	238407.990	0.508	233.9177
54	4	50	54	3	51	A	0	238418.670	0.004	351.2222
54	5	50	54	4	51	A	0	238418.670	0.004	351.2222
46	0	46	45	0	45	A	0	238454.042	-0.185	248.0655
46	0	46	45	1	45	A	0	238454.042	-0.185	248.0655
46	1	46	45	0	45	A	0	238454.042	-0.185	248.0655
46	1	46	45	1	45	A	0	238454.042	-0.185	248.0655
46	0	46	45	0	45	E	0	238454.042	0.153	248.0769
46	1	46	45	1	45	E	0	238454.042	0.153	248.0769
54	4	50	54	3	51	E	1	238477.001	-0.144	479.5228
54	5	50	54	4	51	E	1	238477.001	-0.144	479.5228
39	8	32	38	7	31	A	1	238583.195	0.157	362.4556
36	9	27	35	9	26	E	0	238628.714	-0.004	216.2337

J'	K'_a	K'_c	J''	K''_a	K''_c	Sym.	$v_{t_{CH_3}}$	$\nu_{Obs.}$	$\nu_{Obs.} - \nu_{Calc.}$	E_{low}
36	9	27	35	9	26	A	0	238631.161	0.214	216.2251
50	22	28	50	21	29	E	0	238644.149	-0.210	414.6055
50	22	29	50	21	30	E	0	238678.220	0.021	414.5964
50	22	28	50	21	29	A	0	238691.474	-0.034	414.6087
50	22	29	50	21	30	A	0	238691.474	-0.036	414.6087
39	8	32	38	7	31	E	0	238843.238	0.017	233.9244
39	8	32	38	7	31	A	0	238851.983	-0.012	233.9177
39	8	32	38	7	31	E	1	238857.566	0.024	362.2413
61	8	53	61	7	54	E	1	238898.658	0.223	587.7939
61	9	53	61	8	54	E	1	238898.658	0.212	587.7939
37	11	26	36	11	25	A	0	238921.046	-0.110	229.3550
37	11	26	36	11	25	E	0	238928.768	-0.085	229.3605
61	8	53	61	7	54	E	0	238992.198	-0.028	459.5139
61	9	53	61	8	54	E	0	238992.198	-0.038	459.5139
38	14	25	37	14	24	A	0	239051.926	0.077	250.1772
38	14	24	37	14	23	E	0	239060.320	0.006	250.1844
38	14	25	37	14	24	E	0	239062.778	0.094	250.1713
38	14	24	37	14	23	A	0	239064.418	-0.038	250.1776
51	2	49	51	1	50	E	0	239180.772	0.049	306.5944
51	3	49	51	2	50	E	0	239180.772	0.049	306.5944
51	2	49	51	1	50	A	0	239219.891	0.133	306.5846
51	3	49	51	2	50	A	0	239219.891	0.133	306.5846
49	22	27	49	21	28	E	0	239225.669	-0.040	404.1854
49	22	28	49	21	29	E	0	239259.907	0.021	404.1762
49	22	27	49	21	28	A	0	239272.618	-0.033	404.1886
49	22	28	49	21	29	A	0	239272.618	-0.034	404.1886
64	23	41	64	22	42	E	0	239337.709	-0.071	591.1214
64	23	42	64	22	43	E	0	239368.992	-0.144	591.1134
64	23	41	64	22	42	A	0	239384.662	0.102	591.1242
64	23	42	64	22	43	A	0	239389.412	0.061	591.1240
40	9	31	39	10	30	A	0	239651.350	-0.014	249.7781
40	9	31	39	10	30	E	0	239686.659	0.039	249.7840
39	8	31	38	9	30	A	0	239714.005	-0.058	237.8808
39	8	31	38	9	30	E	0	239721.488	-0.001	237.8873
63	9	54	63	8	55	E	0	239754.553	-0.032	491.6928
63	10	54	63	9	55	E	0	239754.553	-0.076	491.6928
48	22	27	48	21	28	E	0	239803.270	-0.047	393.9727
48	22	26	48	21	27	A	0	239815.537	-0.034	393.9852
48	22	27	48	21	28	A	0	239815.537	-0.035	393.9852
56	5	51	56	4	52	A	0	240177.044	0.076	379.6512
56	6	51	56	5	52	A	0	240177.044	0.076	379.6512
38	13	26	37	13	25	A	0	240195.482	-0.036	245.3368
38	13	26	37	13	25	E	0	240234.975	-0.013	245.3343
47	22	25	47	21	26	E	0	240275.870	-0.046	383.9944
42	10	32	41	11	31	A	0	240279.871	-0.073	270.6749
38	13	25	37	13	24	E	0	240288.912	0.089	245.3478
47	22	26	47	21	27	E	0	240310.674	-0.021	383.9852
38	13	25	37	13	24	A	0	240320.034	-0.063	245.3413
47	22	25	47	21	26	A	0	240322.443	-0.032	383.9978
47	22	26	47	21	27	A	0	240322.443	-0.032	383.9978
14	14	0	13	13	1	A	0	240446.932	-0.002	118.1489
14	14	1	13	13	0	A	0	240446.932	-0.002	118.1489
63	23	41	63	22	42	E	0	240595.774	-0.103	577.6319
46	22	24	46	21	25	E	0	240748.981	-0.054	374.2224
46	22	25	46	21	26	E	0	240784.048	-0.033	374.2133
46	22	24	46	21	25	A	0	240795.380	-0.037	374.2260
46	22	25	46	21	26	A	0	240795.380	-0.037	374.2260
38	8	30	37	8	29	E	0	240950.049	-0.023	229.6631
38	8	30	37	8	29	A	0	240962.203	-0.001	229.6557
38	8	30	37	8	29	E	1	241000.798	0.059	357.9679
45	22	23	45	21	24	E	0	241190.037	-0.071	364.6655
45	22	24	45	21	25	E	0	241225.412	0.009	364.6564
45	22	23	45	21	24	A	0	241236.290	-0.034	364.6691
45	22	24	45	21	25	A	0	241236.290	-0.034	364.6691
16	13	4	15	12	4	E	0	241263.171	0.038	119.3375
16	13	3	15	12	3	E	0	241269.309	-0.066	119.3510
39	23	17	38	23	16	A	0	241292.069	-0.097	320.3914
39	23	16	38	23	15	A	0	241292.069	-0.097	320.3914
39	23	16	38	23	15	E	0	241293.196	-0.109	320.3844
16	13	4	15	12	3	A	0	241307.062	-0.000	119.3420
16	13	3	15	12	4	A	0	241307.062	-0.000	119.3420
53	4	50	53	3	51	A	0	241397.863	0.106	333.3537
24	9	16	23	8	15	A	0	241400.797	-0.001	136.7951
38	10	29	37	10	28	E	0	241470.705	-0.072	233.4802
38	10	29	37	10	28	A	0	241471.965	0.010	233.4742
53	3	50	53	2	51	E	1	241500.536	-0.116	461.6531
53	4	50	53	3	51	E	1	241500.536	-0.116	461.6531
39	22	17	38	22	16	A	0	241520.145	-0.099	311.8755
39	22	18	38	22	17	A	0	241520.145	-0.099	311.8755
39	22	17	38	22	16	E	0	241521.396	-0.097	311.8699
38	12	27	37	12	26	A	0	241527.298	-0.037	240.9655
38	12	27	37	12	26	E	0	241542.606	-0.143	240.9682
44	22	22	44	21	23	E	0	241600.889	-0.054	355.3231
44	22	23	44	21	24	E	0	241636.516	0.050	355.3139
44	22	22	44	21	23	A	0	241646.961	-0.041	355.3268
44	22	23	44	21	24	A	0	241646.961	-0.041	355.3268
58	6	52	58	5	53	E	1	241688.653	0.006	537.4484
58	7	52	58	6	53	E	1	241688.653	0.006	537.4484
58	6	52	58	5	53	A	0	241716.494	-0.024	409.1546
58	7	52	58	6	53	A	0	241716.494	-0.024	409.1546
62	23	40	62	22	41	E	0	241745.163	-0.095	564.3755
39	21	19	38	21	18	A	0	241781.990	-0.108	303.7510
39	21	18	38	21	17	A	0	241781.990	-0.108	303.7510
39	21	18	38	21	17	E	0	241783.501	0.042	303.7470
43	22	21	43	21	22	E	0	241983.212	-0.028	346.1947
43	22	22	43	21	23	E	0	242018.925	-0.048	346.1855
43	22	21	43	21	22	A	0	242029.042	-0.108	346.1984
43	22	22	43	21	23	A	0	242029.042	-0.108	346.1984
18	12	7	17	11	7	E	0	242075.296	0.022	121.7617
39	20	19	38	20	18	A	0	242085.414	0.043	296.0202
39	20	20	38	20	19	A	0	242085.414	0.043	296.0202
18	12	6	17	11	6	E	0	242086.994	-0.122	121.7748
39	20	20	38	20	19	E	0	242086.994	0.039	296.0075
18	12	6	17	11	7	A	0	242121.195	0.073	121.7647
18	12	7	17	11	6	A	0	242121.195	0.074	121.7647
25	8	17	24	7	18	E	0	242355.573	0.006	139.1521
25	8	17	24	7	18	A	0	242368.384	0.001	139.1435
42	22	21	42	21	22	E	0	242374.533	0.007	337.2706
42	22	20	42	21	21	A	0	242384.382	0.011	337.2836
42	22	21	42	21	22	A	0	242384.382	0.011	337.2836
39	19	21	38	19	20	A	0	242440.102	-0.038	288.6858
39	19	20	38	19	19	A	0	242440.102	-0.038	288.6858
38	12	26	37	12	25	A	0	242458.951	-0.003	241.0066
24	9	16	23	8	16	E	0	242595.517	0.006	136.7590
42	5	37	41	6	36	A	1	242666.552	-0.068	376.7865
42	6	37	41	5	36	A	1	242667.561	-0.087	376.7865

J'	K'_a	K'_c	J''	K''_a	K''_c	Sym.	$v_{t_{CH_3}}$	$\nu_{Obs.}$	$\nu_{Obs.} - \nu_{Calc.}$	E_{low}
41	6	35	40	7	34	A	1	242702.568	0.029	373.8625
41	22	20	41	21	21	E	0	242704.646	0.004	328.5689
41	22	19	41	21	20	A	0	242714.112	-0.064	328.5819
41	22	20	41	21	21	A	0	242714.112	-0.064	328.5819
40	7	33	39	8	32	A	1	242716.296	-0.040	370.4139
41	7	35	40	6	34	A	1	242733.805	0.025	373.8618
20	11	10	19	10	10	E	0	242743.697	0.001	125.4387
43	4	39	42	4	38	A	1	242746.705	-0.033	379.2309
43	4	39	42	5	38	A	1	242746.705	-0.017	379.2309
43	5	39	42	4	38	A	1	242746.705	-0.040	379.2309
43	5	39	42	5	38	A	1	242746.705	-0.025	379.2309
20	11	9	19	10	9	E	0	242761.249	-0.055	125.4511
20	11	10	19	10	9	A	0	242791.031	0.049	125.4399
42	5	37	41	6	36	E	1	242801.797	-0.016	376.5672
42	6	37	41	5	36	E	1	242802.992	-0.040	376.5672
61	23	39	61	22	40	E	0	242822.744	-0.080	551.3434
42	5	37	41	6	36	E	0	242838.936	0.035	248.2480
42	6	37	41	5	36	E	0	242839.976	-0.062	248.2479
42	5	37	41	6	36	A	0	242843.041	-0.026	248.2411
42	6	37	41	5	36	A	0	242844.207	-0.004	248.2411
43	4	39	42	4	38	E	1	242858.792	-0.038	378.9949
43	4	39	42	5	38	E	1	242858.792	-0.019	378.9949
43	5	39	42	4	38	E	1	242858.792	-0.048	378.9949
43	5	39	42	5	38	E	1	242858.792	-0.029	378.9949
39	18	21	38	18	20	A	0	242860.086	0.071	281.7515
39	18	22	38	18	21	A	0	242860.086	0.071	281.7515
41	6	35	40	7	34	E	1	242861.818	-0.013	373.6536
40	7	33	39	8	32	E	1	242872.159	-0.008	370.2087
41	7	35	40	7	34	E	1	242874.681	0.019	373.6536
41	6	35	40	6	34	E	1	242885.088	-0.105	373.6528
41	6	35	40	7	34	E	0	242893.520	0.012	245.3342
44	3	41	43	3	40	A	1	242895.556	-0.053	381.2257
44	3	41	43	4	40	A	1	242895.556	-0.053	381.2257
44	4	41	43	3	40	A	1	242895.556	-0.053	381.2257
44	4	41	43	4	40	A	1	242895.556	-0.053	381.2257
41	6	35	40	7	34	A	0	242898.300	-0.112	245.3277
43	4	39	42	4	38	E	0	242900.597	-0.017	250.6777
43	5	39	42	5	38	E	0	242900.597	-0.008	250.6777
43	4	39	42	4	38	A	0	242904.049	-0.015	250.6704
43	4	39	42	5	38	A	0	242904.049	0.003	250.6704
43	5	39	42	4	38	A	0	242904.049	-0.024	250.6704
43	5	39	42	5	38	A	0	242904.049	-0.006	250.6704
41	7	35	40	7	34	E	0	242905.483	-0.079	245.3342
40	7	33	39	8	32	E	0	242909.527	-0.133	241.8914
41	7	35	40	7	34	A	0	242910.724	0.196	245.3277
40	7	33	39	8	32	A	0	242914.265	-0.023	241.8850
41	6	35	40	6	34	E	0	242915.536	0.015	245.3335
41	6	35	40	6	34	A	0	242920.532	-0.002	245.3269
41	7	35	40	6	34	E	0	242927.578	0.004	245.3335
41	7	35	40	6	34	A	0	242932.653	0.001	245.3269
24	9	15	23	8	16	A	0	242945.335	-0.064	136.7519
40	22	18	40	21	19	E	0	242974.439	-0.045	320.0889
44	3	41	43	3	40	E	1	242985.009	-0.016	380.9671
44	3	41	43	4	40	E	1	242985.009	-0.016	380.9671
44	4	41	43	3	40	E	1	242985.009	-0.016	380.9671
44	4	41	43	4	40	E	1	242985.009	-0.016	380.9671
40	22	19	40	21	20	E	0	243010.803	0.053	320.0797
24	9	15	23	8	16	E	0	243012.968	-0.037	136.7590
22	10	13	21	9	13	E	0	243017.660	-0.089	130.4119
40	22	18	40	21	19	A	0	243019.959	-0.037	320.0928
40	22	19	40	21	20	A	0	243019.959	-0.037	320.0928
44	3	41	43	3	40	E	0	243030.999	-0.021	252.6536
44	4	41	43	4	40	E	0	243030.999	-0.021	252.6536
44	3	41	43	3	40	A	0	243033.756	-0.011	252.6456
44	3	41	43	4	40	A	0	243033.756	-0.011	252.6456
44	4	41	43	3	40	A	0	243033.756	-0.011	252.6456
44	4	41	43	4	40	A	0	243033.756	-0.011	252.6456
22	10	12	21	9	12	E	0	243041.047	0.036	130.4235
22	10	13	21	9	12	A	0	243052.878	-0.012	130.4118
22	10	12	21	9	13	A	0	243079.719	0.104	130.4110
45	2	43	44	2	42	A	1	243082.667	-0.030	382.7910
45	2	43	44	3	42	A	1	243082.667	-0.030	382.7910
45	3	43	44	2	42	A	1	243082.667	-0.030	382.7910
45	3	43	44	3	42	A	1	243082.667	-0.030	382.7910
40	7	33	39	7	32	A	1	243128.506	0.107	370.4002
40	8	33	39	8	32	E	1	243143.854	-0.009	370.2087
45	2	43	44	2	42	E	1	243148.364	-0.028	382.5036
45	2	43	44	3	42	E	1	243148.364	-0.028	382.5036
45	3	43	44	2	42	E	1	243148.364	-0.028	382.5036
45	3	43	44	3	42	E	1	243148.364	-0.028	382.5036
40	8	33	39	8	32	E	0	243167.509	0.119	241.8914
45	2	43	44	2	42	E	0	243198.683	-0.003	254.1957
45	3	43	44	3	42	E	0	243198.683	-0.003	254.1957
45	2	43	44	2	42	A	0	243200.694	-0.008	254.1868
45	2	43	44	3	42	A	0	243200.694	-0.008	254.1868
45	3	43	44	2	42	A	0	243200.694	-0.008	254.1868
45	3	43	44	3	42	A	0	243200.694	-0.008	254.1868
39	22	17	39	21	18	E	0	243257.795	0.000	311.8120
39	9	31	38	9	30	E	1	243281.401	-0.018	366.2007
46	1	45	45	1	44	A	1	243290.908	0.003	383.9413
46	1	45	45	2	44	A	1	243290.908	0.003	383.9413
46	2	45	45	1	44	A	1	243290.908	0.003	383.9413
46	2	45	45	2	44	A	1	243290.908	0.003	383.9413
39	22	18	39	21	19	E	0	243294.135	-0.076	311.8029
39	22	17	39	21	18	A	0	243303.235	0.045	311.8160
39	22	18	39	21	19	A	0	243303.235	0.045	311.8160
39	9	31	38	9	30	A	0	243308.566	0.031	237.8808
46	1	45	45	1	44	E	1	243330.680	-0.012	383.6187
46	1	45	45	2	44	E	1	243330.680	-0.012	383.6187
46	2	45	45	1	44	E	1	243330.680	-0.012	383.6187
46	2	45	45	2	44	E	1	243330.680	-0.012	383.6187
40	7	33	39	7	32	E	1	243337.331	-0.046	370.1932
55	4	51	55	3	52	E	0	243347.721	0.039	361.2164
55	5	51	55	4	52	E	0	243347.721	0.039	361.2164
40	7	33	39	7	32	E	0	243352.277	-0.028	241.8766
40	7	33	39	7	32	A	0	243358.761	-0.041	241.8701
39	17	23	38	17	22	A	0	243363.924	-0.011	275.2225
39	17	22	38	17	21	E	0	243365.608	-0.115	275.2249
39	17	23	38	17	22	E	0	243366.692	-0.060	275.2123
40	8	33	39	7	32	A	1	243367.742	0.209	370.4002
55	4	51	55	3	52	A	0	243379.616	0.072	361.2094
55	5	51	55	4	52	A	0	243379.616	0.072	361.2094
46	1	45	45	1	44	E	0	243385.755	-0.035	255.3180
46	2	45	45	2	44	E	0	243385.755	-0.035	255.3180
46	1	45	45	1	44	A	0	243387.031	0.018	255.3080
46	1	45	45	2	44	A	0	243387.031	0.018	255.3080

J'	K'_a	K'_c	J''	K''_a	K''_c	Sym.	$v_{t_{CH_3}}$	$\nu_{Obs.}$	$\nu_{Obs.} - \nu_{Calc.}$	E_{low}
46	2	45	45	1	44	A	0	243387.031	0.018	255.3080
46	2	45	45	2	44	A	0	243387.031	0.018	255.3080
55	4	51	55	3	52	E	1	243437.990	-0.174	489.5078
55	5	51	55	4	52	E	1	243437.990	-0.174	489.5078
47	0	47	46	0	46	A	1	243510.510	0.088	384.6871
47	0	47	46	1	46	A	1	243510.510	0.088	384.6871
47	1	47	46	0	46	A	1	243510.510	0.088	384.6871
47	1	47	46	1	46	A	1	243510.510	0.088	384.6871
47	0	47	46	0	46	E	1	243521.081	-0.017	384.3223
47	1	47	46	1	46	E	1	243521.081	-0.017	384.3223
38	22	17	38	21	18	E	0	243556.362	0.050	303.7379
38	22	16	38	21	17	A	0	243565.010	-0.035	303.7510
38	22	17	38	21	18	A	0	243565.010	-0.035	303.7510
47	0	47	46	0	46	A	0	243582.021	-0.176	256.0195
47	0	47	46	1	46	A	0	243582.021	-0.176	256.0195
47	1	47	46	0	46	A	0	243582.021	-0.176	256.0195
47	1	47	46	1	46	A	0	243582.021	-0.176	256.0195
47	0	47	46	0	46	E	0	243582.021	0.159	256.0308
47	1	47	46	1	46	E	0	243582.021	0.159	256.0308
40	8	33	39	7	32	E	1	243608.917	-0.157	370.1932
40	8	33	39	7	32	E	0	243610.087	0.053	241.8766
40	8	33	39	7	32	A	0	243617.665	-0.005	241.8701
37	22	15	37	21	16	E	0	243761.616	0.012	295.8934
37	22	16	37	21	17	E	0	243798.320	0.040	295.8843
60	23	37	60	22	38	E	0	243799.634	-0.025	538.5425
37	22	15	37	21	16	A	0	243806.744	-0.041	295.8975
37	22	16	37	21	17	A	0	243806.744	-0.041	295.8975
60	23	38	60	22	39	E	0	243833.506	-0.044	538.5345
60	23	37	60	22	38	A	0	243847.155	0.126	538.5460
39	16	24	38	16	23	A	0	243979.271	0.017	269.1054
39	16	24	38	16	23	E	0	243982.699	-0.056	269.0965
36	22	14	36	21	15	E	0	243984.440	-0.055	288.2509
36	22	15	36	21	16	E	0	244021.324	0.042	288.2418
36	22	14	36	21	15	A	0	244029.545	-0.032	288.2550
36	22	15	36	21	16	A	0	244029.545	-0.032	288.2550
62	8	54	62	7	55	E	0	244075.470	-0.066	471.3627
62	9	54	62	8	55	E	0	244075.470	-0.072	471.3627
62	8	54	62	7	55	A	0	244092.832	-0.151	471.3601
62	9	54	62	8	55	A	0	244092.832	-0.156	471.3601
52	2	50	52	1	51	E	0	244116.304	0.043	315.7390
52	3	50	52	2	51	E	0	244116.304	0.043	315.7390
52	2	50	52	1	51	A	0	244156.243	0.181	315.7292
52	3	50	52	2	51	A	0	244156.243	0.181	315.7292
35	22	13	35	21	14	E	0	244189.485	-0.055	280.8191
35	22	14	35	21	15	E	0	244226.444	0.015	280.8100
35	22	13	35	21	14	A	0	244234.504	-0.027	280.8233
35	22	14	35	21	15	A	0	244234.504	-0.027	280.8233
52	2	50	52	1	51	E	1	244305.184	-0.022	444.0258
52	3	50	52	2	51	E	1	244305.184	-0.022	444.0258
34	22	12	34	21	13	E	0	244377.819	0.016	273.5977
34	22	13	34	21	14	E	0	244414.893	0.110	273.5885
34	22	12	34	21	13	A	0	244422.639	-0.068	273.6018
34	22	13	34	21	14	A	0	244422.639	-0.068	273.6018
37	9	28	36	9	27	A	0	244428.590	-0.034	224.1850
37	10	27	36	10	26	A	0	244442.809	-0.027	226.3998
37	10	27	36	10	26	E	0	244450.748	-0.034	226.4073
33	22	11	33	21	12	E	0	244550.274	-0.020	266.5862
33	22	12	33	21	13	E	0	244587.404	0.047	266.5771
33	22	11	33	21	12	A	0	244595.064	-0.054	266.5904
33	22	12	33	21	13	A	0	244595.064	-0.054	266.5904
32	22	10	32	21	11	E	0	244707.994	0.012	259.7845
32	22	11	32	21	12	E	0	244745.157	0.038	259.7754
39	15	25	38	15	24	A	0	244747.450	0.468	263.4102
39	15	24	38	15	23	A	0	244748.969	-0.167	263.4102
39	15	24	38	15	23	E	0	244750.101	0.079	263.4157
59	23	37	59	22	38	E	0	244781.857	-0.056	525.9480
59	23	36	59	22	37	A	0	244794.634	0.051	525.9597
31	22	9	31	21	10	E	0	244851.783	-0.009	253.1921
31	22	10	31	21	11	E	0	244889.025	0.031	253.1830
31	22	9	31	21	10	A	0	244896.392	-0.077	253.1964
31	22	10	31	21	11	A	0	244896.392	-0.077	253.1964
30	22	8	30	21	9	E	0	244982.587	-0.021	246.8088
30	22	8	30	21	9	A	0	245027.228	0.008	246.8131
30	22	9	30	21	10	A	0	245027.228	0.008	246.8131
36	9	28	35	8	27	A	0	245061.032	0.005	214.0071
29	22	7	29	21	8	E	0	245101.259	-0.019	240.6343
29	22	8	29	21	9	E	0	245138.635	0.046	240.6251
29	22	7	29	21	8	A	0	245145.760	-0.068	240.6386
29	22	8	29	21	9	A	0	245145.760	-0.068	240.6386
57	5	52	57	4	53	A	0	245157.292	0.013	390.1457
57	6	52	57	5	53	A	0	245157.292	0.013	390.1457
57	5	52	57	4	53	E	1	245171.744	-0.085	518.4414
57	6	52	57	5	53	E	1	245171.744	-0.085	518.4414
28	22	6	28	21	7	E	0	245208.499	-0.114	234.6682
37	9	29	36	8	28	E	0	245218.099	-0.058	221.7682
28	22	7	28	21	8	E	0	245246.094	0.126	234.6591
28	22	6	28	21	7	A	0	245253.085	-0.020	234.6725
28	22	7	28	21	8	A	0	245253.085	-0.020	234.6725
37	9	29	36	8	28	A	0	245256.860	0.056	221.7603
54	3	51	54	2	52	A	1	245272.093	-0.181	471.5680
54	4	51	54	3	52	A	1	245272.093	-0.181	471.5680
39	8	31	38	8	30	E	0	245323.855	-0.022	237.7004
39	8	31	38	8	30	A	0	245334.975	0.007	237.6934
27	22	6	27	21	7	E	0	245342.898	0.116	228.9013
27	22	5	27	21	6	A	0	245349.781	-0.046	228.9147
27	22	6	27	21	7	A	0	245349.781	-0.046	228.9147
39	8	31	38	8	30	E	1	245362.739	0.152	366.0068
26	22	4	26	21	5	E	0	245392.349	0.003	223.3605
26	22	5	26	21	6	E	0	245429.797	0.024	223.3514
25	22	4	25	21	5	E	0	245507.707	0.053	218.0092
25	22	3	25	21	4	A	0	245514.564	0.023	218.0226
25	22	4	25	21	5	A	0	245514.564	0.023	218.0226
37	9	29	36	8	28	E	1	245552.146	0.327	350.0711
70	24	46	70	23	47	E	0	245568.076	-0.034	684.4722
70	24	47	70	23	48	E	0	245601.302	-0.116	684.4652
70	24	47	70	23	48	A	0	245628.471	-0.048	684.4748
58	23	35	58	22	36	E	0	245636.991	-0.044	513.5912
58	23	36	58	22	37	A	0	245683.882	-0.035	513.5950
39	14	26	38	14	25	A	0	245726.986	0.003	258.1512
39	14	25	38	14	24	E	0	245742.450	-0.111	258.1586
39	14	26	38	14	25	E	0	245743.613	0.123	258.1456
39	14	25	38	14	24	A	0	245751.700	-0.023	258.1520
35	9	27	34	8	26	E	0	245901.944	-0.093	206.4106
35	9	27	34	8	26	A	0	245956.794	0.017	206.4019
25	9	17	24	8	16	E	0	246025.370	-0.062	141.8981
25	9	17	24	8	16	A	0	246123.358	0.008	141.8900

J'	K'_a	K'_c	J''	K''_a	K''_c	Sym.	$v_{t_{\mathrm{CH_3}}}$	$\nu_{\mathrm{Obs.}}$	$\nu_{\mathrm{Obs.}} - \nu_{\mathrm{Calc.}}$	E_{low}
40	8	32	39	9	31	A	0	246260.208	-0.004	245.9967
40	8	32	39	9	31	E	0	246262.972	0.013	246.0030
54	3	51	54	2	52	E	0	246307.087	0.007	343.0133
54	4	51	54	3	52	E	0	246307.087	0.007	343.0133
54	3	51	54	2	52	A	0	246343.321	0.122	343.0050
54	4	51	54	3	52	A	0	246343.321	0.122	343.0050
54	3	51	54	2	52	E	1	246446.828	-0.200	471.3022
54	4	51	54	3	52	E	1	246446.828	-0.200	471.3022
57	23	35	57	22	36	E	0	246507.343	-0.052	501.4391
57	23	34	57	22	35	A	0	246518.487	-0.023	501.4511
38	9	30	37	8	29	A	0	246583.105	-0.005	229.6557
15	14	1	14	13	2	A	0	246594.641	0.005	121.0225
15	14	2	14	13	1	A	0	246594.641	0.005	121.0225
59	6	53	59	5	54	E	1	246692.799	-0.006	548.4481
59	7	53	59	6	54	E	1	246692.799	-0.006	548.4481
59	6	53	59	5	54	A	0	246721.892	-0.032	420.1567
59	7	53	59	6	54	A	0	246721.892	-0.032	420.1567
40	27	14	39	27	13	A	0	246874.347	-0.006	366.3654
40	27	13	39	27	12	A	0	246874.347	-0.006	366.3654
38	11	27	37	11	26	A	0	246932.800	-0.034	237.3245
38	11	27	37	11	26	E	0	246942.986	-0.005	237.3303
39	13	27	38	13	26	A	0	246971.087	-0.007	253.3488
39	13	27	38	13	26	E	0	247010.946	-0.044	253.3476
69	24	45	69	23	46	E	0	247067.957	0.027	669.6769
69	24	46	69	23	47	E	0	247101.010	-0.107	669.6700
69	24	45	69	23	46	A	0	247110.852	0.109	669.6805
69	24	46	69	23	47	A	0	247122.321	-0.065	669.6800
39	13	26	38	13	25	E	0	247165.678	-0.064	253.3629
56	23	33	56	22	34	E	0	247255.694	-0.040	489.5233
56	23	34	56	22	35	E	0	247291.537	-0.029	489.5152
56	23	33	56	22	34	A	0	247301.964	0.005	489.5273
39	10	30	38	10	29	E	0	247304.464	0.000	241.5348
39	10	30	38	10	29	A	0	247307.323	0.569	241.5288
56	4	52	56	3	53	A	1	247346.480	0.304	499.8846
56	5	52	56	4	53	A	1	247346.480	0.304	499.8846
40	24	16	39	24	15	A	0	247386.879	-0.079	337.3389
40	24	17	39	24	16	A	0	247386.879	-0.079	337.3389
17	13	5	16	12	5	E	0	247402.799	0.032	122.6286
17	13	4	16	12	4	E	0	247408.995	-0.064	122.6421
17	13	5	16	12	4	A	0	247446.724	0.002	122.6331
17	13	4	16	12	5	A	0	247446.724	0.002	122.6331
40	23	18	39	23	17	A	0	247603.037	-0.094	328.4400
40	23	17	39	23	16	A	0	247603.037	-0.094	328.4400
40	23	17	39	23	16	E	0	247604.252	-0.117	328.4331
34	9	26	33	8	25	E	0	247671.308	-0.055	198.9658
34	9	26	33	8	25	A	0	247732.175	-0.012	198.9569
43	5	38	42	5	37	A	1	247777.580	-0.096	384.8810
43	5	38	42	6	37	A	1	247777.580	0.256	384.8810
43	6	38	42	5	37	A	1	247777.580	-0.278	384.8810
43	6	38	42	6	37	A	1	247777.580	0.074	384.8810
42	6	36	41	7	35	A	1	247798.322	0.042	381.9585
42	7	36	41	7	35	A	1	247804.276	0.023	381.9585
42	6	36	41	6	35	A	1	247809.392	0.081	381.9582
42	7	36	41	6	35	A	1	247815.304	0.021	381.9582
40	22	18	39	22	17	A	0	247849.563	-0.018	319.9317
40	22	19	39	22	18	A	0	247849.563	-0.018	319.9317
40	22	18	39	22	17	E	0	247850.882	-0.054	319.9262
44	4	40	43	4	39	A	1	247866.530	-0.043	387.3281
44	4	40	43	5	39	A	1	247866.530	-0.035	387.3281
44	5	40	43	4	39	A	1	247866.530	-0.047	387.3281
44	5	40	43	5	39	A	1	247866.530	-0.039	387.3281
41	7	34	40	8	33	A	1	247885.934	0.040	378.5180
43	5	38	42	5	37	E	0	247950.535	-0.103	256.3482
43	6	38	42	6	37	E	0	247950.535	0.085	256.3482
43	5	38	42	5	37	A	0	247954.585	-0.179	256.3415
43	5	38	42	6	37	A	0	247954.585	0.214	256.3415
43	6	38	42	5	37	A	0	247954.585	-0.382	256.3415
43	6	38	42	6	37	A	0	247954.585	0.010	256.3415
42	7	36	41	7	35	E	1	247963.019	0.001	381.7550
42	6	36	41	6	35	E	1	247968.856	-0.001	381.7546
42	7	36	41	6	35	E	1	247975.873	0.025	381.7546
44	4	40	43	4	39	E	1	247977.779	-0.056	387.0958
44	4	40	43	5	39	E	1	247977.779	-0.047	387.0958
44	5	40	43	4	39	E	1	247977.779	-0.061	387.0958
44	5	40	43	5	39	E	1	247977.779	-0.052	387.0958
42	6	36	41	7	35	E	0	247989.796	-0.019	253.4367
61	7	54	61	6	55	E	1	247991.125	0.342	579.5218
61	8	54	61	7	55	E	1	247991.125	0.341	579.5218
42	6	36	41	7	35	A	0	247994.661	-0.015	253.4303
42	7	36	41	7	35	E	0	247996.291	-0.074	253.4367
42	6	36	41	6	35	A	0	248006.791	-0.002	253.4299
42	7	36	41	6	35	E	0	248008.373	-0.046	253.4363
42	7	36	41	6	35	A	0	248013.377	-0.002	253.4299
45	3	42	44	3	41	A	1	248019.494	-0.041	389.3278
45	3	42	44	4	41	A	1	248019.494	-0.041	389.3278
45	4	42	44	3	41	A	1	248019.494	-0.041	389.3278
45	4	42	44	4	41	A	1	248019.494	-0.041	389.3278
44	4	40	43	4	39	E	0	248021.406	-0.009	258.7800
44	5	40	43	5	39	E	0	248021.406	-0.004	258.7800
44	4	40	43	5	39	A	0	248024.851	0.021	258.7728
44	5	40	43	4	39	A	0	248024.851	0.007	258.7728
55	23	32	55	22	33	A	0	248037.259	-0.020	477.8230
41	7	34	40	8	33	E	1	248052.832	0.027	378.3192
61	7	54	61	6	55	A	0	248062.964	-0.052	451.2367
61	8	54	61	7	55	A	0	248062.964	-0.053	451.2367
41	7	34	40	8	33	E	0	248087.191	-0.017	250.0026
41	7	34	40	8	33	A	0	248092.276	0.021	249.9964
45	3	42	44	3	41	E	1	248108.466	-0.024	389.0722
45	3	42	44	4	41	E	1	248108.466	-0.023	389.0722
45	4	42	44	3	41	E	1	248108.466	-0.024	389.0722
45	4	42	44	4	41	E	1	248108.466	-0.024	389.0722
41	7	34	40	7	33	A	1	248125.146	0.118	378.5101
40	21	20	39	21	19	A	0	248132.735	-0.040	311.8160
40	21	19	39	21	18	A	0	248132.735	-0.040	311.8160
40	21	20	39	21	19	E	0	248134.162	-0.033	311.8029
45	3	42	44	3	41	E	0	248156.100	-0.009	260.7603
45	4	42	44	4	41	E	0	248156.100	-0.009	260.7603
45	3	42	44	3	41	A	0	248158.841	-0.003	260.7523
45	3	42	44	4	41	A	0	248158.841	-0.003	260.7523
45	4	42	44	3	41	A	0	248158.841	-0.003	260.7523
45	4	42	44	4	41	A	0	248158.841	-0.003	260.7523
19	12	8	18	11	8	E	0	248188.176	0.003	125.4757
19	12	7	18	11	7	E	0	248200.061	-0.062	125.4888
46	2	44	45	2	43	A	1	248208.290	-0.039	390.8994
46	2	44	45	3	43	A	1	248208.290	-0.039	390.8994
46	3	44	45	2	43	A	1	248208.290	-0.039	390.8994

J'	K'_a	K'_c	J''	K''_a	K''_c	Sym.	$v_{t_{CH_3}}$	$\nu_{Obs.}$	$\nu_{Obs.} - \nu_{Calc.}$	E_{low}
46	3	44	45	3	43	A	1	248208.290	-0.039	390.8994
41	8	34	40	8	33	E	1	248209.621	-0.002	378.3192
19	12	7	18	11	8	A	0	248234.133	0.048	125.4786
19	12	8	18	11	7	A	0	248234.133	0.050	125.4786
41	8	34	40	8	33	E	0	248235.602	0.082	250.0026
41	8	34	40	8	33	A	0	248241.246	-0.006	249.9964
41	8	34	40	7	33	A	1	248262.259	0.069	378.5101
46	2	44	45	2	43	E	1	248273.755	-0.036	390.6142
46	2	44	45	3	43	E	1	248273.755	-0.036	390.6142
46	3	44	45	2	43	E	1	248273.755	-0.036	390.6142
46	3	44	45	3	43	E	1	248273.755	-0.036	390.6142
39	12	28	38	12	27	A	0	248315.799	-0.088	249.0220
46	2	44	45	2	43	E	0	248325.596	0.008	262.3080
46	3	44	45	3	43	E	0	248325.596	0.008	262.3080
46	2	44	45	2	43	A	0	248327.589	-0.009	262.2991
46	2	44	45	3	43	A	0	248327.589	-0.009	262.2991
46	3	44	45	2	43	A	0	248327.589	-0.009	262.2991
46	3	44	45	3	43	A	0	248327.589	-0.009	262.2991
56	4	52	56	3	53	A	0	248338.638	0.044	371.3675
56	5	52	56	4	53	A	0	248338.638	0.044	371.3675
41	7	34	40	7	33	E	0	248344.908	-0.029	249.9940
41	7	34	40	7	33	A	0	248351.120	-0.003	249.9877
56	4	52	56	3	53	E	1	248396.963	-0.253	499.6637
56	5	52	56	4	53	E	1	248396.963	-0.253	499.6637
47	1	46	46	1	45	A	1	248417.203	0.006	392.0566
47	1	46	46	2	45	A	1	248417.203	0.006	392.0566
47	2	46	46	1	45	A	1	248417.203	0.006	392.0566
47	2	46	46	2	45	A	1	248417.203	0.006	392.0566
47	1	46	46	1	45	E	1	248456.858	-0.015	391.7353
47	1	46	46	2	45	E	1	248456.858	-0.015	391.7353
47	2	46	46	1	45	E	1	248456.858	-0.015	391.7353
47	2	46	46	2	45	E	1	248456.858	-0.015	391.7353
40	20	20	39	20	19	A	0	248461.120	0.013	304.0953
40	20	21	39	20	20	A	0	248461.120	0.013	304.0953
40	20	21	39	20	20	E	0	248462.784	-0.052	304.0826
68	24	44	68	23	45	E	0	248472.909	-0.025	655.1101
40	9	32	39	9	31	E	1	248479.182	-0.187	374.3157
41	8	34	40	7	33	E	1	248481.323	0.003	378.3101
41	8	34	40	7	33	E	0	248493.231	-0.019	249.9940
41	8	34	40	7	33	A	0	248500.098	-0.022	249.9877
40	9	32	39	9	31	E	0	248502.518	0.055	246.0030
68	24	45	68	23	46	E	0	248506.589	-0.026	655.1031
40	9	32	39	9	31	A	0	248508.151	-0.021	245.9967
47	1	46	46	1	45	E	0	248513.351	-0.036	263.4365
47	2	46	46	2	45	E	0	248513.351	-0.036	263.4365
47	1	46	46	1	45	A	0	248514.636	0.029	263.4265
47	1	46	46	2	45	A	0	248514.636	0.029	263.4265
47	2	46	46	1	45	A	0	248514.636	0.029	263.4265
47	2	46	46	2	45	A	0	248514.636	0.029	263.4265
48	0	48	47	0	47	A	1	248637.088	0.095	392.8098
48	0	48	47	1	47	A	1	248637.088	0.095	392.8098
48	1	48	47	0	47	A	1	248637.088	0.095	392.8098
48	1	48	47	1	47	A	1	248637.088	0.095	392.8098
48	0	48	47	0	47	E	1	248647.642	0.065	392.4452
48	1	48	47	1	47	E	1	248647.642	0.065	392.4452
54	23	31	54	22	32	E	0	248681.619	-0.011	466.3331
48	0	48	47	0	47	A	0	248709.860	-0.172	264.1445
48	0	48	47	1	47	A	0	248709.860	-0.172	264.1445
48	1	48	47	0	47	A	0	248709.860	-0.172	264.1445
48	1	48	47	1	47	A	0	248709.860	-0.172	264.1445
48	0	48	47	0	47	E	0	248709.860	0.161	264.1558
48	1	48	47	1	47	E	0	248709.860	0.161	264.1558
54	23	32	54	22	33	E	0	248718.174	-0.070	466.3251
54	23	31	54	22	32	A	0	248727.282	-0.030	466.3374
21	11	11	20	10	11	E	0	248788.971	0.025	129.5845
21	11	10	20	10	10	E	0	248806.698	-0.099	129.5969
21	11	11	20	10	10	A	0	248836.479	0.249	129.5857
21	11	10	20	10	11	A	0	248836.479	-0.238	129.5857
40	19	22	39	19	21	A	0	248845.672	-0.022	296.7727
40	19	21	39	19	20	A	0	248845.672	-0.022	296.7727
40	19	21	39	19	20	E	0	248847.578	0.180	296.7719
39	11	29	38	11	28	A	0	248888.068	0.029	245.1400
39	11	29	38	11	28	E	0	248890.498	0.008	245.1450
23	10	14	22	9	14	E	0	248898.411	0.040	135.0057
39	9	31	38	8	30	E	0	248905.633	-0.004	237.7004
23	10	14	22	9	13	A	0	248914.588	-0.017	135.0060
23	10	13	22	9	13	E	0	248918.778	-0.055	135.0172
39	9	31	38	8	30	A	0	248929.424	-0.017	237.6934
23	10	13	22	9	14	A	0	248976.668	0.072	135.0042
25	9	16	24	8	17	A	0	249009.915	0.008	141.8103
25	9	16	24	8	17	E	0	249035.959	0.095	141.8177
63	8	55	63	7	56	E	1	249053.918	0.221	611.6566
63	9	55	63	8	56	E	1	249053.918	0.218	611.6566
41	9	32	40	10	31	E	1	249098.656	-0.203	386.5284
63	8	55	63	7	56	E	0	249150.517	-0.104	483.3820
63	9	55	63	8	56	E	0	249150.517	-0.106	483.3820
63	8	55	63	7	56	A	0	249168.295	-0.089	483.3796
63	9	55	63	8	56	A	0	249168.295	-0.092	483.3796
53	2	51	53	1	52	E	1	249242.623	-0.167	453.3393
53	3	51	53	2	52	E	1	249242.623	-0.167	453.3393
40	18	22	39	18	21	A	0	249301.631	-0.012	289.8525
40	18	23	39	18	22	A	0	249301.631	-0.010	289.8525
40	18	22	39	18	21	E	0	249303.164	-0.294	289.8533
40	18	23	39	18	22	E	0	249304.337	0.161	289.8414
53	23	30	53	22	31	E	0	249329.230	-0.023	455.0656
53	23	31	53	22	32	E	0	249366.205	-0.012	455.0575
53	23	30	53	22	31	A	0	249374.680	-0.001	455.0700
41	9	32	40	10	31	A	0	249433.426	-0.060	258.2163
41	9	32	40	10	31	E	0	249458.047	0.024	258.2221
38	9	29	37	9	28	E	0	249604.294	-0.033	232.3465
38	9	29	37	9	28	A	0	249613.927	-0.008	232.3383
67	24	43	67	23	44	E	0	249790.694	-0.049	640.7705
67	24	44	67	23	45	E	0	249825.048	-0.015	640.7635
39	12	27	38	12	26	A	0	249837.184	0.028	249.0942
39	12	27	38	12	26	E	0	249839.193	0.026	249.0986
40	8	32	39	8	31	E	0	249844.719	0.001	245.8835
40	17	24	39	17	23	A	0	249850.121	-0.008	283.3402
40	17	23	39	17	22	E	0	249851.970	-0.093	283.3427
40	17	24	39	17	23	E	0	249853.256	0.050	283.3302
40	8	32	39	8	31	A	0	249854.664	-0.020	245.8768
65	9	56	65	8	57	E	1	249867.696	0.341	644.8463
65	10	56	65	9	57	E	1	249867.696	0.327	644.8463
40	8	32	39	8	31	E	1	249868.947	0.029	374.1912
52	23	29	52	22	30	E	0	249936.572	-0.059	444.0155
33	9	25	32	8	24	E	0	249974.089	-0.251	191.6983
52	23	30	52	22	31	A	0	249981.806	-0.013	444.0201

J'	K'_a	K'_c	J''	K''_a	K''_c	Sym.	$v_{t_{CH_3}}$	$\nu_{Obs.}$	$\nu_{Obs.} - \nu_{Calc.}$	E_{low}
52	23	29	52	22	30	A	0	249981.806	-0.013	444.0201
65	9	56	65	8	57	E	0	250009.294	-0.078	516.5808
65	10	56	65	9	57	E	0	250009.294	-0.091	516.5808
65	9	56	65	8	57	A	0	250023.676	-0.105	516.5792
65	10	56	65	9	57	A	0	250023.676	-0.118	516.5792
33	9	25	32	8	24	A	0	250039.083	0.013	191.6892
58	5	53	58	4	54	E	0	250106.054	0.000	400.8166
58	6	53	58	5	54	E	0	250106.054	0.000	400.8166
26	9	18	25	8	17	E	0	250125.592	-0.030	147.2363
58	5	53	58	4	54	A	0	250134.823	0.013	400.8110
58	6	53	58	5	54	A	0	250134.823	0.013	400.8110
58	5	53	58	4	54	E	1	250148.544	-0.025	529.1044
58	6	53	58	5	54	E	1	250148.544	-0.025	529.1044
26	9	18	25	8	17	A	0	250199.403	-0.004	147.2281
67	10	57	67	9	58	E	1	250415.603	0.654	679.0847
67	11	57	67	10	58	E	1	250415.603	0.593	679.0847
51	23	28	51	22	29	E	0	250505.985	-0.041	433.1825
40	16	25	39	16	24	A	0	250522.172	0.149	277.2437
40	16	24	39	16	23	E	0	250524.191	-0.023	277.2477
40	16	25	39	16	24	E	0	250525.894	-0.037	277.2349
51	23	29	51	22	30	A	0	250550.974	-0.010	433.1871
51	23	28	51	22	29	A	0	250550.974	-0.010	433.1871
67	10	57	67	9	58	E	0	250601.217	-0.038	550.8303
67	11	57	67	10	58	E	0	250601.217	-0.093	550.8303
67	10	57	67	9	58	A	0	250612.298	-0.106	550.8295
67	11	57	67	10	58	A	0	250612.298	-0.162	550.8295
66	24	42	66	23	43	E	0	251027.935	-0.006	626.6570
50	23	27	50	22	28	E	0	251039.544	-0.000	422.5658
66	24	43	66	23	44	E	0	251062.758	-0.152	626.6501
66	24	42	66	23	43	A	0	251074.080	0.095	626.6611
66	24	43	66	23	44	A	0	251075.645	-0.049	626.6610
50	23	28	50	22	29	E	0	251077.388	-0.029	422.5578
50	23	28	50	22	29	A	0	251084.259	-0.027	422.5706
50	23	27	50	22	28	A	0	251084.259	-0.027	422.5706
55	3	52	55	2	53	A	0	251287.767	0.204	352.8273
55	4	52	55	3	53	A	0	251287.767	0.204	352.8273
40	15	26	39	15	25	A	0	251363.763	0.031	271.5740
40	15	25	39	15	24	A	0	251368.095	-0.071	271.5742
40	15	25	39	15	24	E	0	251368.095	-0.001	271.5797
40	15	26	39	15	25	E	0	251370.509	-0.027	271.5668
55	3	52	55	2	53	E	1	251392.016	-0.195	481.1223
55	4	52	55	3	53	E	1	251392.016	-0.195	481.1223
49	23	26	49	22	27	E	0	251539.118	-0.038	412.1651
49	23	27	49	22	28	E	0	251577.267	-0.022	412.1571
49	23	27	49	22	28	A	0	251583.659	-0.033	412.1699
49	23	26	49	22	27	A	0	251583.659	-0.033	412.1699
60	6	54	60	5	55	E	1	251692.701	-0.044	559.6185
60	7	54	60	6	55	E	1	251692.701	-0.044	559.6185
60	6	54	60	5	55	E	0	251698.191	0.014	431.3341
60	7	54	60	6	55	E	0	251698.191	0.013	431.3341
60	6	54	60	5	55	A	0	251723.262	-0.038	431.3297
60	7	54	60	6	55	A	0	251723.262	-0.038	431.3297
38	10	28	37	10	27	A	0	251962.232	0.034	234.5535
38	10	28	37	10	27	E	0	251967.277	-0.004	234.5613
48	23	25	48	22	26	E	0	252006.622	-0.087	401.9797
48	23	26	48	22	27	E	0	252045.066	-0.016	401.9717
48	23	26	48	22	27	A	0	252050.992	-0.058	401.9846
48	23	25	48	22	26	A	0	252050.992	-0.058	401.9846
26	8	18	25	7	19	E	0	252066.730	-0.031	144.4180
26	8	18	25	7	19	A	0	252075.398	-0.040	144.4093
40	9	32	39	8	31	E	0	252084.217	-0.006	245.8835
40	9	32	39	8	31	A	0	252102.644	-0.000	245.8768
65	24	41	65	23	42	E	0	252190.278	-0.056	612.7688
65	24	42	65	23	43	E	0	252225.866	-0.055	612.7619
57	4	53	57	3	54	A	1	252285.700	0.319	510.2086
57	5	53	57	4	54	A	1	252285.700	0.319	510.2086
32	9	24	31	8	23	E	0	252393.365	-0.102	184.6277
40	14	27	39	14	26	A	0	252439.602	0.046	266.3477
47	23	24	47	22	25	E	0	252443.907	-0.028	392.0091
32	9	24	31	8	23	A	0	252459.775	-0.034	184.6186
40	14	27	39	14	26	E	0	252464.637	0.030	266.3427
40	14	26	39	14	25	E	0	252469.921	0.056	266.3557
47	23	25	47	22	26	E	0	252482.545	0.012	392.0011
40	14	26	39	14	25	A	0	252486.773	-0.078	266.3494
47	23	25	47	22	26	A	0	252488.077	-0.015	392.0141
47	23	24	47	22	25	A	0	252488.077	-0.015	392.0141
41	31	11	40	31	10	A	0	252629.030	-0.031	418.6892
41	31	10	40	31	9	A	0	252629.030	-0.031	418.6892
41	30	11	40	30	10	A	0	252738.950	0.061	407.0895
41	30	12	40	30	11	A	0	252738.950	0.061	407.0895
16	14	2	15	13	3	A	0	252740.937	-0.000	124.1031
16	14	3	15	13	2	A	0	252740.937	-0.000	124.1031
54	2	52	54	1	53	A	1	252829.997	-0.934	463.1345
54	3	52	54	2	53	A	1	252829.997	-0.934	463.1345
46	23	23	46	22	24	E	0	252852.420	-0.049	382.2530
44	5	39	43	5	38	A	1	252889.405	-0.061	393.1460
44	5	39	43	6	38	A	1	252889.405	0.121	393.1460
44	6	39	43	5	38	A	1	252889.405	-0.155	393.1460
44	6	39	43	6	38	A	1	252889.405	0.027	393.1460
46	23	24	46	22	25	E	0	252891.223	-0.050	382.2450
43	6	37	42	7	36	A	1	252894.955	0.053	390.2244
46	23	24	46	22	25	A	0	252896.415	-0.037	382.2581
46	23	23	46	22	24	A	0	252896.415	-0.037	382.2581
43	7	37	42	7	36	A	1	252898.036	-0.077	390.2244
43	6	37	42	6	36	A	1	252900.765	-0.110	390.2242
43	7	37	42	6	36	A	1	252904.086	0.000	390.2242
40	10	31	39	10	30	E	0	252967.998	-0.028	249.7840
40	10	31	39	10	30	A	0	252971.361	0.005	249.7781
45	4	41	44	4	40	A	1	252986.763	-0.052	395.5960
45	4	41	44	5	40	A	1	252986.763	-0.048	395.5960
45	5	41	44	4	40	A	1	252986.763	-0.054	395.5960
45	5	41	44	5	40	A	1	252986.763	-0.050	395.5960
41	28	13	40	28	12	A	0	252992.015	0.008	385.0444
41	28	14	40	28	13	A	0	252992.015	0.008	385.0444
42	7	35	41	8	34	A	1	253011.959	0.062	386.7912
62	7	55	62	6	56	E	1	253020.209	0.165	591.2002
62	8	55	62	7	56	E	1	253020.209	0.165	591.2002
44	5	39	43	5	38	E	1	253021.852	-0.085	392.9357
44	5	39	43	6	38	E	1	253021.852	0.133	392.9357
44	6	39	43	5	38	E	1	253021.852	-0.198	392.9357
44	6	39	43	6	38	E	1	253021.852	0.020	392.9357
43	7	37	42	7	36	E	1	253054.561	-0.054	390.0262
43	6	37	42	6	36	E	1	253057.836	0.013	390.0260
43	7	37	42	6	36	E	1	253061.602	-0.003	390.0260
44	5	39	43	5	38	E	0	253063.020	-0.064	264.6189
44	6	39	43	6	38	E	0	253063.020	0.034	264.6190

J'	K'_a	K'_c	J''	K''_a	K''_c	Sym.	$v_{t_{CH_3}}$	$\nu_{Obs.}$	$\nu_{Obs.} - \nu_{Calc.}$	E_{low}
44	5	39	43	5	38	A	0	253067.121	-0.046	264.6124
44	5	39	43	6	38	A	0	253067.121	0.158	264.6124
44	6	39	43	5	38	A	0	253067.121	-0.151	264.6124
44	6	39	43	6	38	A	0	253067.121	0.052	264.6124
62	7	55	62	6	56	E	0	253072.605	-0.052	462.9211
62	8	55	62	7	56	E	0	253072.605	-0.053	462.9211
43	6	37	42	7	36	E	0	253086.833	-0.015	261.7089
43	7	37	42	7	36	E	0	253090.256	-0.126	261.7089
43	6	37	42	7	36	A	0	253091.662	0.005	261.7027
43	6	37	42	6	36	E	0	253093.202	-0.196	261.7087
43	7	37	42	7	36	A	0	253095.337	0.126	261.7027
43	6	37	42	6	36	A	0	253098.415	0.172	261.7025
43	7	37	42	6	36	A	0	253101.800	0.005	261.7025
41	27	15	40	27	14	A	0	253138.832	-0.038	374.6003
41	27	14	40	27	13	A	0	253138.832	-0.038	374.6003
45	4	41	44	4	40	E	0	253142.579	-0.060	267.0531
45	5	41	44	5	40	E	0	253142.579	-0.058	267.0531
45	4	41	44	4	40	A	0	253146.032	-0.007	267.0460
45	4	41	44	5	40	A	0	253146.032	-0.002	267.0460
45	5	41	44	4	40	A	0	253146.032	-0.009	267.0460
45	5	41	44	5	40	A	0	253146.032	-0.005	267.0460
42	7	35	41	7	34	A	1	253149.163	0.104	386.7866
42	7	35	41	8	34	E	1	253184.398	0.107	386.5985
42	7	35	41	8	34	E	0	253217.850	-0.024	258.2828
42	7	35	41	8	34	A	0	253223.155	0.017	258.2768
42	8	35	41	7	34	A	1	253226.949	0.049	386.7866
46	3	43	45	3	42	E	1	253231.967	-0.035	397.3482
46	3	43	45	4	42	E	1	253231.967	-0.035	397.3482
46	4	43	45	3	42	E	1	253231.967	-0.035	397.3482
46	4	43	45	4	42	E	1	253231.967	-0.035	397.3482
45	23	22	45	22	23	E	0	253233.889	0.035	372.7107
45	23	23	45	22	24	E	0	253272.766	-0.081	372.7028
45	23	23	45	22	24	A	0	253277.624	-0.045	372.7159
45	23	22	45	22	23	A	0	253277.624	-0.045	372.7159
46	3	43	45	3	42	E	0	253281.243	-0.004	269.0379
46	4	43	45	4	42	E	0	253281.243	-0.004	269.0379
46	3	43	45	3	42	A	0	253283.963	-0.004	269.0300
46	3	43	45	4	42	A	0	253283.963	-0.004	269.0300
46	4	43	45	3	42	A	0	253283.963	-0.004	269.0300
46	4	43	45	4	42	A	0	253283.963	-0.004	269.0300
25	7	18	24	6	19	E	0	253293.350	0.187	136.6458
57	4	53	57	3	54	A	0	253296.033	0.120	381.6966
57	5	53	57	4	54	A	0	253296.033	0.120	381.6966
42	8	35	41	8	34	E	0	253302.299	-0.020	258.2828
42	8	35	41	8	34	A	0	253307.955	-0.033	258.2768
64	24	41	64	23	42	E	0	253319.146	-0.118	599.0979
47	2	45	46	2	44	A	1	253333.829	-0.036	399.1787
47	2	45	46	3	44	A	1	253333.829	-0.036	399.1787
47	3	45	46	2	44	A	1	253333.829	-0.036	399.1787
47	3	45	46	3	44	A	1	253333.829	-0.036	399.1787
42	7	35	41	7	34	E	1	253341.173	0.064	386.5933
57	4	53	57	3	54	E	1	253354.228	-0.169	509.9904
57	5	53	57	4	54	E	1	253354.228	-0.169	509.9904
42	7	35	41	7	34	E	0	253366.114	-0.073	258.2779
42	7	35	41	7	34	A	0	253372.185	0.051	258.2718
27	9	19	26	8	18	A	0	253393.160	0.031	152.8177
47	2	45	46	2	44	E	1	253399.096	-0.004	398.8957
47	2	45	46	3	44	E	1	253399.096	-0.004	398.8957
47	3	45	46	2	44	E	1	253399.096	-0.004	398.8957
47	3	45	46	3	44	E	1	253399.096	-0.004	398.8957
41	9	33	40	9	32	A	1	253415.806	0.059	382.7975
42	8	35	41	7	34	E	1	253430.645	-0.015	386.5933
42	8	35	41	7	34	E	0	253450.716	0.085	258.2779
47	2	45	46	2	44	E	0	253452.402	0.001	270.5912
47	3	45	46	3	44	E	0	253452.402	0.001	270.5912
47	2	45	46	2	44	A	0	253454.405	0.001	270.5824
47	2	45	46	3	44	A	0	253454.405	0.001	270.5824
47	3	45	46	2	44	A	0	253454.405	0.001	270.5824
47	3	45	46	3	44	A	0	253454.405	0.001	270.5824
42	8	35	41	7	34	A	0	253456.986	0.002	258.2718
41	25	17	40	25	16	A	0	253485.037	-0.036	354.8724
41	25	16	40	25	15	A	0	253485.037	-0.036	354.8724
18	13	6	17	12	6	E	0	253537.109	0.053	126.1281
48	1	47	47	1	46	A	1	253543.386	0.030	400.3429
48	1	47	47	2	46	A	1	253543.386	0.030	400.3429
48	2	47	47	1	46	A	1	253543.386	0.030	400.3429
48	2	47	47	2	46	A	1	253543.386	0.030	400.3429
18	13	6	17	12	5	A	0	253581.058	0.015	126.1325
18	13	5	17	12	6	A	0	253581.058	0.015	126.1325
48	1	47	47	1	46	E	1	253582.916	-0.006	400.0229
48	1	47	47	2	46	E	1	253582.916	-0.006	400.0229
48	2	47	47	1	46	E	1	253582.916	-0.006	400.0229
48	2	47	47	2	46	E	1	253582.916	-0.006	400.0229
44	23	21	44	22	22	E	0	253589.519	-0.022	363.3820
41	9	33	40	9	32	E	1	253616.628	0.097	382.6041
44	23	22	44	22	23	E	0	253628.739	0.028	363.3740
44	23	22	44	22	23	A	0	253633.159	-0.042	363.3872
44	23	21	44	22	22	A	0	253633.159	-0.042	363.3872
48	1	47	47	1	46	E	0	253640.804	-0.048	271.7260
48	2	47	47	2	46	E	0	253640.804	-0.048	271.7260
48	1	47	47	1	46	A	0	253642.092	0.024	271.7160
48	1	47	47	2	46	A	0	253642.092	0.024	271.7160
48	2	47	47	1	46	A	0	253642.092	0.024	271.7160
48	2	47	47	2	46	A	0	253642.092	0.024	271.7160
41	9	33	40	9	32	A	0	253646.550	-0.003	254.2861
41	24	17	40	24	16	A	0	253690.794	-0.011	345.5908
41	24	18	40	24	17	A	0	253690.794	-0.011	345.5908
49	0	49	48	0	48	A	1	253763.513	0.090	401.1034
49	0	49	48	1	48	A	1	253763.513	0.090	401.1034
49	1	49	48	0	48	A	1	253763.513	0.090	401.1034
49	1	49	48	1	48	A	1	253763.513	0.090	401.1034
49	0	49	48	0	48	E	1	253773.911	-0.006	400.7392
49	1	49	48	1	48	E	1	253773.911	-0.006	400.7392
40	13	28	39	13	27	A	0	253777.437	0.001	261.5869
40	13	28	39	13	27	E	0	253808.211	-0.024	261.5870
49	0	49	48	0	48	A	0	253837.549	-0.177	272.4406
49	0	49	48	1	48	A	0	253837.549	-0.177	272.4406
49	1	49	48	0	48	A	0	253837.549	-0.177	272.4406
49	1	49	48	1	48	A	0	253837.549	-0.177	272.4406
49	0	49	48	0	48	E	0	253837.549	0.153	272.4519
49	1	49	48	1	48	E	0	253837.549	0.153	272.4519
41	23	19	40	23	18	A	0	253923.928	0.074	336.6991
41	23	18	40	23	17	A	0	253923.928	0.074	336.6991
43	23	21	43	22	22	E	0	253960.166	-0.079	354.2584
43	23	21	43	22	22	A	0	253964.343	-0.083	354.2716
43	23	20	43	22	21	A	0	253964.343	-0.083	354.2716

J'	K'_a	K'_c	J''	K''_a	K''_c	Sym.	$v_{t_{CH_3}}$	$\nu_{Obs.}$	$\nu_{Obs.} - \nu_{Calc.}$	E_{low}
54	2	52	54	1	53	E	0	253985.467	-0.005	334.5413
54	3	52	54	2	53	E	0	253985.467	-0.005	334.5413
54	2	52	54	1	53	A	0	254026.959	0.163	334.5316
54	3	52	54	2	53	A	0	254026.959	0.163	334.5316
64	8	56	64	7	57	E	1	254119.360	0.198	623.8436
64	9	56	64	8	57	E	1	254119.360	0.197	623.8436
40	13	27	39	13	26	E	0	254153.389	0.001	261.6075
40	13	27	39	13	26	A	0	254174.006	-0.022	261.6031
54	2	52	54	1	53	E	1	254179.670	-0.002	462.8237
54	3	52	54	2	53	E	1	254179.670	-0.002	462.8237
41	22	19	40	22	18	A	0	254189.702	-0.049	328.1991
41	22	20	40	22	19	A	0	254189.702	-0.049	328.1991
41	22	20	40	22	19	E	0	254191.076	0.063	328.1857
42	23	19	42	22	20	E	0	254229.239	-0.041	345.3633
64	8	56	64	7	57	A	0	254236.195	0.078	495.5695
64	9	56	64	8	57	A	0	254236.195	0.077	495.5695
39	9	30	38	9	29	E	0	254266.670	-0.067	240.6724
42	23	20	42	22	21	E	0	254268.786	0.028	345.3554
42	23	20	42	22	21	A	0	254272.627	-0.025	345.3687
42	23	19	42	22	20	A	0	254272.627	-0.025	345.3687
39	9	30	38	9	29	A	0	254279.080	-0.014	240.6645
20	12	9	19	11	9	E	0	254288.217	0.018	129.4001
20	12	8	19	11	8	E	0	254300.223	-0.057	129.4132
20	12	8	19	11	9	A	0	254334.217	0.025	129.4030
63	24	40	63	23	41	E	0	254347.493	-0.095	585.6573
63	24	39	63	23	40	A	0	254356.395	0.038	585.6688
41	21	21	40	21	20	A	0	254495.557	-0.015	320.0928
41	21	20	40	21	19	A	0	254495.557	-0.015	320.0928
41	21	21	40	21	20	E	0	254497.097	-0.025	320.0797
31	9	23	30	8	22	E	0	254500.343	-0.069	177.7737
41	8	33	40	8	32	E	0	254505.935	0.003	254.2174
41	23	19	41	22	20	E	0	254555.525	0.029	336.6646
41	23	19	41	22	20	A	0	254559.087	-0.035	336.6780
41	23	18	41	22	19	A	0	254559.087	-0.035	336.6780
31	9	23	30	8	22	A	0	254566.295	-0.011	177.7646
24	10	15	23	9	14	A	0	254692.365	0.040	139.8199
24	10	15	23	9	15	E	0	254720.881	-0.034	139.8183
24	10	14	23	9	14	E	0	254726.424	-0.054	139.8303
40	23	17	40	22	18	E	0	254781.841	-0.061	328.1937
22	11	12	21	10	12	E	0	254806.218	-0.023	133.9440
40	23	18	40	22	19	E	0	254821.699	0.058	328.1857
24	10	14	23	9	15	A	0	254829.446	0.043	139.8159
41	20	21	40	20	20	A	0	254850.497	-0.038	312.3830
41	20	22	40	20	21	A	0	254850.497	-0.038	312.3830
41	20	21	40	20	20	E	0	254852.266	0.017	312.3807
66	9	57	66	8	58	E	1	254977.277	0.426	657.5428
66	10	57	66	9	58	E	1	254977.277	0.418	657.5428
39	23	17	39	22	18	E	0	255068.358	0.037	319.9183
39	23	17	39	22	18	A	0	255071.436	-0.032	319.9317
39	23	16	39	22	17	A	0	255071.436	-0.032	319.9317
40	12	29	39	12	28	A	0	255074.072	-0.020	257.3050
59	5	54	59	4	55	A	0	255109.706	-0.012	411.6472
59	6	54	59	5	55	A	0	255109.706	-0.012	411.6472
39	11	28	38	11	27	A	0	255114.010	0.007	245.5613
59	5	54	59	4	55	E	1	255122.340	-0.185	539.9381
59	6	54	59	5	55	E	1	255122.340	-0.185	539.9381
39	11	28	38	11	27	E	0	255125.346	0.015	245.5674
66	9	57	66	8	58	A	0	255135.584	-0.126	529.2789
66	10	57	66	9	58	A	0	255135.584	-0.133	529.2789
40	11	30	39	11	29	A	0	255226.756	-0.144	253.4420
40	11	30	39	11	29	E	0	255228.127	-0.014	253.4471
38	23	15	38	22	16	E	0	255256.622	-0.035	311.8699
41	19	23	40	19	22	A	0	255266.871	-0.038	305.0733
41	19	22	40	19	21	A	0	255266.871	-0.039	305.0733
41	19	22	40	19	21	E	0	255268.921	0.175	305.0726
62	24	38	62	23	39	E	0	255277.826	-0.049	572.4462
38	23	16	38	22	17	E	0	255296.598	-0.013	311.8620
38	23	16	38	22	17	A	0	255299.512	-0.034	311.8755
38	23	15	38	22	16	A	0	255299.512	-0.034	311.8755
62	24	39	62	23	40	E	0	255315.111	0.018	572.4393
62	24	38	62	23	39	A	0	255323.042	0.023	572.4510
28	9	20	27	8	19	E	0	255421.181	-0.068	158.6745
37	23	14	37	22	15	E	0	255467.478	-0.014	304.0244
28	9	20	27	8	19	A	0	255481.629	-0.004	158.6659
37	23	15	37	22	16	E	0	255507.594	0.055	304.0165
37	23	15	37	22	16	A	0	255510.245	-0.032	304.0300
37	23	14	37	22	15	A	0	255510.245	-0.032	304.0300
36	23	13	36	22	14	E	0	255661.936	-0.020	296.3894
36	23	14	36	22	15	E	0	255702.146	0.058	296.3814
36	23	14	36	22	15	A	0	255704.630	-0.014	296.3950
36	23	13	36	22	14	A	0	255704.630	-0.014	296.3950
41	18	23	40	18	22	A	0	255761.435	-0.035	298.1683
41	18	24	40	18	23	A	0	255761.435	-0.032	298.1683
35	23	12	35	22	13	E	0	255840.969	-0.019	288.9644
41	9	33	40	8	32	E	0	255880.036	-0.035	254.2174
35	23	13	35	22	14	E	0	255881.291	0.097	288.9565
35	23	13	35	22	14	A	0	255883.553	-0.030	288.9700
35	23	12	35	22	13	A	0	255883.553	-0.030	288.9700
41	9	33	40	8	32	A	0	255894.495	-0.019	254.2111
30	9	22	29	8	21	E	0	255899.035	-0.093	171.1545
34	23	11	34	22	12	E	0	256005.477	-0.008	281.7492
34	23	12	34	22	13	E	0	256045.782	0.023	281.7413
34	23	12	34	22	13	A	0	256047.953	-0.042	281.7549
34	23	11	34	22	12	A	0	256047.953	-0.042	281.7549
70	11	59	70	10	60	A	0	256142.530	-0.091	599.8458
70	12	59	70	11	60	A	0	256142.530	-0.214	599.8458
33	23	10	33	22	11	E	0	256156.262	-0.048	274.7436
61	24	37	61	23	38	E	0	256187.820	-0.085	559.4500
33	23	11	33	22	12	E	0	256196.691	0.047	274.7357
33	23	11	33	22	12	A	0	256198.688	-0.052	274.7493
33	23	10	33	22	11	A	0	256198.688	-0.052	274.7493
61	24	38	61	23	39	E	0	256225.522	-0.074	559.4431
61	24	37	61	23	38	A	0	256232.715	0.010	559.4549
29	9	21	28	8	20	A	0	256331.178	-0.061	164.7762
32	23	10	32	22	11	A	0	256336.502	-0.139	267.9528
32	23	9	32	22	10	A	0	256336.502	-0.139	267.9528
41	17	25	40	17	24	A	0	256357.905	-0.014	291.6743
41	17	24	40	17	23	E	0	256359.982	-0.033	291.6768
41	17	25	40	17	24	E	0	256361.267	-0.015	291.6644
31	23	8	31	22	9	E	0	256420.158	-0.048	261.3595
31	23	9	31	22	10	E	0	256460.708	0.068	261.3516
31	23	9	31	22	10	A	0	256462.451	-0.039	261.3652
31	23	8	31	22	9	A	0	256462.451	-0.039	261.3652
30	23	7	30	22	8	E	0	256534.794	-0.032	254.9806
30	23	8	30	22	9	A	0	256577.041	-0.004	254.9863
30	23	7	30	22	8	A	0	256577.041	-0.004	254.9863

J'	K'_a	K'_c	J''	K''_a	K''_c	Sym.	$v_{t_{CH_3}}$	$\nu_{Obs.}$	$\nu_{Obs.} - \nu_{Calc.}$	E_{low}
29	23	6	29	22	7	E	0	256638.870	-0.006	248.8100
29	23	7	29	22	8	E	0	256679.488	0.099	248.8021
29	23	7	29	22	8	A	0	256680.974	-0.060	248.8157
29	23	6	29	22	7	A	0	256680.974	-0.060	248.8157
61	6	55	61	5	56	E	1	256688.653	-0.059	570.9596
61	7	55	61	6	56	E	1	256688.653	-0.059	570.9596
61	6	55	61	5	56	A	0	256720.866	-0.025	442.6734
61	7	55	61	6	56	A	0	256720.866	-0.025	442.6734
28	23	5	28	22	6	E	0	256733.070	0.016	242.8475
28	23	6	28	22	7	E	0	256773.684	0.088	242.8396
28	23	6	28	22	7	A	0	256775.092	-0.062	242.8533
28	23	5	28	22	6	A	0	256775.092	-0.062	242.8533
27	23	4	27	22	5	E	0	256817.991	-0.037	237.0929
27	23	5	27	22	6	A	0	256860.048	-0.027	237.0987
27	23	4	27	22	5	A	0	256860.048	-0.027	237.0987
26	23	4	26	22	5	E	0	256935.038	0.008	231.5380
26	23	4	26	22	5	A	0	256936.378	-0.059	231.5517
26	23	3	26	22	4	A	0	256936.378	-0.059	231.5517
60	24	36	60	23	37	E	0	257044.409	-0.036	546.6748
60	24	37	60	23	38	E	0	257082.528	-0.048	546.6679
60	24	36	60	23	37	A	0	257088.889	-0.015	546.6799
41	16	25	40	16	24	A	0	257091.793	-0.077	285.6002
41	16	26	40	16	25	E	0	257095.515	-0.067	285.5915
58	4	54	58	3	55	A	1	257223.439	0.380	520.7035
58	5	54	58	4	55	A	1	257223.439	0.380	520.7035
40	12	28	39	12	27	A	0	257464.193	-0.019	257.4279
40	12	28	39	12	27	E	0	257470.756	-0.019	257.4323
42	8	34	41	9	33	A	1	257736.062	0.060	391.2505
42	8	34	41	9	33	E	1	257838.987	-0.036	391.0638
59	24	35	59	23	36	E	0	257850.669	-0.008	534.1199
42	9	33	41	10	32	A	0	257891.789	-0.141	266.8383
59	24	35	59	23	36	A	0	257894.742	-0.064	534.1251
42	8	34	41	9	33	E	0	257907.767	-0.177	262.7527
42	8	34	41	9	33	A	0	257910.578	-0.031	262.7468
15	15	0	14	14	0	E	0	257980.027	-0.059	126.1757
15	15	1	14	14	1	E	0	257984.904	0.032	126.1620
44	6	38	43	7	37	A	1	257993.194	0.008	398.6602
44	7	38	43	7	37	A	1	257994.928	0.027	398.6602
44	7	38	43	6	37	A	1	257998.177	0.065	398.6600
45	5	40	44	5	39	A	1	258002.430	-0.024	401.5815
45	5	40	44	6	39	A	1	258002.430	0.069	401.5815
45	6	40	44	5	39	A	1	258002.430	-0.072	401.5815
45	6	40	44	6	39	A	1	258002.430	0.021	401.5815
41	15	27	40	15	26	A	0	258013.307	-0.041	279.9586
41	15	26	40	15	25	E	0	258020.189	-0.034	279.9645
15	15	1	14	14	0	A	0	258023.431	0.002	126.1694
15	15	0	14	14	1	A	0	258023.431	0.002	126.1694
63	7	56	63	6	57	E	1	258043.893	0.072	603.0492
63	8	56	63	7	57	E	1	258043.893	0.072	603.0492
63	7	56	63	6	57	E	0	258098.204	-0.039	474.7728
63	8	56	63	7	57	E	0	258098.204	-0.039	474.7728
46	4	42	45	4	41	A	1	258107.343	-0.051	404.0347
46	4	42	45	5	41	A	1	258107.343	-0.049	404.0347
46	5	42	45	4	41	A	1	258107.343	-0.052	404.0347
46	5	42	45	5	41	A	1	258107.343	-0.050	404.0347
43	7	36	42	8	35	A	1	258114.657	0.078	395.2334
63	7	56	63	6	57	A	0	258120.015	-0.115	474.7696
63	8	56	63	7	57	A	0	258120.015	-0.115	474.7696
45	5	40	44	5	39	E	1	258133.534	-0.057	401.3756
45	5	40	44	6	39	E	1	258133.534	0.056	401.3756
45	6	40	44	5	39	E	1	258133.534	-0.115	401.3756
45	6	40	44	6	39	E	1	258133.534	-0.002	401.3756
44	6	38	43	7	37	E	1	258147.161	-0.027	398.4672
44	7	38	43	7	37	E	1	258149.213	-0.007	398.4672
44	6	38	43	6	37	E	1	258150.942	-0.028	398.4671
44	7	38	43	6	37	E	1	258153.101	0.099	398.4671
43	8	36	42	8	35	A	1	258158.348	0.021	395.2334
45	5	40	44	5	39	E	0	258176.739	-0.025	273.0602
45	6	40	44	6	39	E	0	258176.739	0.026	273.0602
45	5	40	44	5	39	A	0	258180.792	-0.016	273.0538
45	5	40	44	6	39	A	0	258180.792	0.089	273.0538
45	6	40	44	5	39	A	0	258180.792	-0.070	273.0538
45	6	40	44	6	39	A	0	258180.792	0.035	273.0538
44	6	38	43	7	37	E	0	258185.496	0.010	270.1511
44	7	38	43	7	37	E	0	258187.341	-0.037	270.1511
44	6	38	43	6	37	E	0	258188.961	-0.057	270.1510
44	7	38	43	7	37	A	0	258192.250	0.109	270.1451
44	6	38	43	6	37	A	0	258193.776	-0.014	270.1449
44	7	38	43	6	37	A	0	258195.687	-0.007	270.1449
46	4	42	45	4	41	E	1	258217.007	-0.054	403.8099
46	4	42	45	5	41	E	1	258217.007	-0.051	403.8099
46	5	42	45	4	41	E	1	258217.007	-0.055	403.8099
46	5	42	45	5	41	E	1	258217.007	-0.053	403.8099
43	8	36	42	7	35	A	1	258236.276	0.107	395.2308
58	4	54	58	3	55	A	0	258251.661	0.077	392.1966
58	5	54	58	4	55	A	0	258251.661	0.077	392.1966
46	4	42	45	4	41	E	0	258264.217	-0.002	275.4971
46	5	42	45	5	41	E	0	258264.217	-0.001	275.4971
47	3	44	46	3	43	A	1	258267.535	0.057	406.0448
47	3	44	46	4	43	A	1	258267.535	0.057	406.0448
47	4	44	46	3	43	A	1	258267.535	0.057	406.0448
47	4	44	46	4	43	A	1	258267.535	0.057	406.0448
43	7	36	42	8	35	E	1	258288.918	-0.048	395.0468
43	7	36	42	8	35	E	0	258323.423	0.248	266.7321
43	8	36	42	8	35	E	1	258339.631	0.024	395.0468
47	3	44	46	3	43	E	1	258355.514	-0.025	405.7951
47	3	44	46	4	43	E	1	258355.514	-0.025	405.7951
47	4	44	46	3	43	E	1	258355.514	-0.025	405.7951
47	4	44	46	4	43	E	1	258355.514	-0.025	405.7951
43	8	36	42	8	35	E	0	258370.762	-0.027	266.7321
43	8	36	42	8	35	A	0	258376.113	-0.266	266.7262
43	7	36	42	7	35	E	1	258378.531	0.013	395.0439
47	3	44	46	3	43	E	0	258406.398	-0.010	277.4864
47	4	44	46	4	43	E	0	258406.398	-0.010	277.4864
43	7	36	42	7	35	E	0	258407.723	0.104	266.7293
47	3	44	46	3	43	A	0	258409.100	-0.015	277.4786
47	3	44	46	4	43	A	0	258409.100	-0.015	277.4786
47	4	44	46	3	43	A	0	258409.100	-0.015	277.4786
47	4	44	46	4	43	A	0	258409.100	-0.015	277.4786
43	7	36	42	7	35	A	0	258413.377	-0.000	266.7234
43	8	36	42	7	35	E	1	258429.179	0.020	395.0439
43	8	36	42	7	35	E	0	258455.209	-0.025	266.7293
48	2	46	47	2	45	A	1	258459.241	-0.058	407.6290
48	2	46	47	3	45	A	1	258459.241	-0.058	407.6290
48	3	46	47	2	45	A	1	258459.241	-0.058	407.6290
48	3	46	47	3	45	A	1	258459.241	-0.058	407.6290

J'	K'_a	K'_c	J''	K''_a	K''_c	Sym.	$v_{t_{CH_3}}$	$\nu_{Obs.}$	$\nu_{Obs.} - \nu_{Calc.}$	E_{low}
43	8	36	42	7	35	A	0	258461.227	-0.003	266.7234
41	10	32	40	10	31	E	0	258475.888	-0.025	258.2221
41	10	32	40	10	31	A	0	258480.253	0.085	258.2163
42	9	34	41	9	33	A	1	258509.202	0.052	391.2505
48	2	46	47	2	45	E	1	258524.308	-0.003	407.3482
48	3	46	47	3	45	E	1	258524.308	-0.003	407.3482
48	2	46	47	2	45	E	0	258579.110	-0.006	279.0455
48	3	46	47	3	45	E	0	258579.110	-0.006	279.0455
48	2	46	47	2	45	A	0	258581.120	0.009	279.0367
48	2	46	47	3	45	A	0	258581.120	0.009	279.0367
48	3	46	47	2	45	A	0	258581.120	0.009	279.0367
48	3	46	47	3	45	A	0	258581.120	0.009	279.0367
58	24	35	58	23	36	E	0	258648.432	-0.026	521.7779
58	24	34	58	23	35	A	0	258653.325	-0.019	521.7901
49	1	48	48	1	47	A	1	258669.386	0.010	408.8002
49	1	48	48	2	47	A	1	258669.386	0.010	408.8002
49	2	48	48	1	47	A	1	258669.386	0.010	408.8002
49	2	48	48	2	47	A	1	258669.386	0.010	408.8002
49	1	48	48	1	47	E	1	258708.836	0.001	408.4815
49	2	48	48	2	47	E	1	258708.836	0.001	408.4815
42	9	34	41	9	33	E	1	258712.308	0.042	391.0638
42	9	34	41	9	33	E	0	258737.553	0.003	262.7527
42	9	34	41	9	33	A	0	258743.668	-0.026	262.7468
49	1	48	48	1	47	E	0	258768.156	-0.027	280.1866
49	2	48	48	2	47	E	0	258768.156	-0.027	280.1866
49	1	48	48	1	47	A	0	258769.410	0.015	280.1766
49	1	48	48	2	47	A	0	258769.410	0.015	280.1766
49	2	48	48	1	47	A	0	258769.410	0.015	280.1766
49	2	48	48	2	47	A	0	258769.410	0.015	280.1766
17	14	3	16	13	4	A	0	258884.913	-0.004	127.3912
17	14	4	16	13	3	A	0	258884.913	-0.004	127.3912
50	0	50	49	0	49	A	1	258889.820	0.110	409.5680
50	0	50	49	1	49	A	1	258889.820	0.110	409.5680
50	1	50	49	0	49	A	1	258889.820	0.110	409.5680
50	1	50	49	1	49	A	1	258889.820	0.110	409.5680
50	0	50	49	0	49	E	1	258900.119	0.004	409.2042
50	0	50	49	1	49	E	1	258900.119	0.004	409.2042
50	1	50	49	0	49	E	1	258900.119	0.004	409.2042
50	1	50	49	1	49	E	1	258900.119	0.004	409.2042
39	10	29	38	10	28	A	0	258940.237	-0.052	242.9581
39	10	29	38	10	28	E	0	258941.610	-0.025	242.9660
50	0	50	49	0	49	A	0	258965.107	-0.172	280.9077
50	0	50	49	1	49	A	0	258965.107	-0.172	280.9077
50	1	50	49	0	49	A	0	258965.107	-0.172	280.9077
50	1	50	49	1	49	A	0	258965.107	-0.172	280.9077
50	0	50	49	0	49	E	0	258965.107	0.156	280.9190
50	1	50	49	1	49	E	0	258965.107	0.156	280.9190
60	5	55	60	4	56	A	1	259134.835	-0.429	551.1151
60	6	55	60	5	56	A	1	259134.835	-0.429	551.1151
65	8	57	65	7	58	E	1	259177.410	0.176	636.2011
65	9	57	65	8	58	E	1	259177.410	0.175	636.2011
41	14	28	40	14	27	A	0	259190.324	0.013	274.7682
41	14	28	40	14	27	E	0	259225.315	-0.079	274.7641
42	8	34	41	8	33	E	1	259280.724	0.000	391.0157
42	8	34	41	8	33	E	0	259282.085	0.002	262.7068
42	8	34	41	8	33	A	0	259290.243	0.013	262.7008
65	8	57	65	7	58	A	0	259296.570	-0.126	507.9300
65	9	57	65	8	58	A	0	259296.570	-0.126	507.9300
57	24	33	57	23	34	E	0	259323.689	-0.019	509.6686
57	24	34	57	23	35	E	0	259362.918	-0.073	509.6617
57	24	33	57	23	34	A	0	259367.173	-0.043	509.6741
57	24	34	57	23	35	A	0	259367.173	-0.045	509.6741
19	13	7	18	12	7	E	0	259664.478	0.024	129.8365
19	13	6	18	12	6	E	0	259670.825	-0.058	129.8500
19	13	7	18	12	6	A	0	259708.483	-0.001	129.8409
19	13	6	18	12	7	A	0	259708.483	-0.001	129.8409
42	25	18	41	25	17	A	0	259782.403	-0.057	363.3278
42	25	17	41	25	16	A	0	259782.403	-0.057	363.3278
56	24	32	56	23	33	E	0	259995.644	-0.062	497.7708
42	24	18	41	24	17	A	0	260003.694	-0.079	354.0530
42	24	19	41	24	18	A	0	260003.694	-0.079	354.0530
56	24	33	56	23	34	E	0	260035.184	-0.138	497.7640
56	24	33	56	23	34	A	0	260038.929	0.003	497.7764
60	5	55	60	4	56	E	0	260052.470	0.012	422.6597
60	6	55	60	5	56	E	0	260052.470	0.012	422.6597
67	9	58	67	8	59	E	1	260076.979	0.417	670.4095
67	10	58	67	9	59	E	1	260076.979	0.413	670.4095
60	5	55	60	4	56	A	0	260082.168	0.019	422.6543
60	6	55	60	5	56	A	0	260082.168	0.019	422.6543
42	9	34	41	8	33	E	0	260111.673	-0.016	262.7068
42	9	34	41	8	33	A	0	260123.339	0.023	262.7008
42	9	34	41	8	33	E	1	260154.017	0.050	391.0157
42	23	20	41	23	19	A	0	260254.596	-0.052	345.1691
42	23	19	41	23	18	A	0	260254.596	-0.052	345.1691
70	25	46	70	24	47	E	0	260275.209	-0.147	692.6575
25	10	16	24	9	15	A	0	260353.964	-0.017	144.8557
21	12	10	20	11	10	E	0	260372.723	0.006	133.5357
21	12	9	20	11	9	E	0	260384.853	-0.105	133.5488
21	12	10	20	11	9	A	0	260418.804	0.003	133.5386
25	10	15	24	9	15	E	0	260434.540	-0.026	144.8650
25	10	16	24	9	16	E	0	260488.528	-0.001	144.8511
42	22	20	41	22	19	A	0	260541.084	-0.035	336.6780
42	22	21	41	22	20	A	0	260541.084	-0.035	336.6780
42	22	21	41	22	20	E	0	260542.552	0.054	336.6646
41	13	29	40	13	28	A	0	260605.429	-0.045	270.0520
41	13	29	40	13	28	E	0	260626.034	0.008	270.0532
55	24	31	55	23	32	E	0	260627.801	-0.045	486.0909
25	10	15	24	9	16	A	0	260643.909	0.013	144.8474
55	24	32	55	23	33	E	0	260667.710	-0.063	486.0841
55	24	31	55	23	32	A	0	260670.757	-0.033	486.0966
55	24	32	55	23	33	A	0	260670.757	-0.033	486.0966
23	11	13	22	10	13	E	0	260790.579	0.008	138.5181
23	11	12	22	10	12	E	0	260809.109	0.045	138.5304
23	11	13	22	10	12	A	0	260836.679	-0.084	138.5193
42	21	22	41	21	21	A	0	260870.934	0.011	328.5819
42	21	21	41	21	20	A	0	260870.934	0.011	328.5819
42	21	22	41	21	21	E	0	260872.546	-0.066	328.5689
69	10	59	69	9	60	A	0	260929.679	-0.185	577.4201
69	11	59	69	10	60	A	0	260929.679	-0.201	577.4201
57	3	54	57	2	55	A	0	261173.337	0.108	372.9848
57	4	54	57	3	55	A	0	261173.337	0.108	372.9848
54	24	30	54	23	31	E	0	261222.235	-0.054	474.6282
42	20	22	41	20	21	A	0	261254.045	-0.141	320.8839
42	20	23	41	20	22	A	0	261254.045	-0.141	320.8839
42	20	22	41	20	21	E	0	261256.090	0.059	320.8817
54	24	31	54	23	32	E	0	261262.458	-0.048	474.6214

J'	K'_a	K'_c	J''	K''_a	K''_c	Sym.	$v_{t_{\mathrm{CH_3}}}$	$\nu_{\mathrm{Obs.}}$	$\nu_{\mathrm{Obs.}} - \nu_{\mathrm{Calc.}}$	E_{low}
54	24	30	54	23	31	A	0	261264.935	-0.037	474.6341
54	24	31	54	23	32	A	0	261264.935	-0.037	474.6341
41	13	28	40	13	27	E	0	261274.060	0.060	270.0851
57	3	54	57	2	55	E	1	261279.038	-0.133	501.2751
57	4	54	57	3	55	E	1	261279.038	-0.133	501.2751
41	13	28	40	13	27	A	0	261283.136	-0.034	270.0814
41	11	31	40	11	30	A	0	261402.054	-0.014	261.9555
41	11	31	40	11	30	E	0	261402.054	-0.040	261.9606
54	1	53	54	0	54	A	0	261505.443	0.169	325.8087
54	2	53	54	1	54	A	0	261505.443	0.169	325.8087
62	6	56	62	5	57	E	1	261680.875	-0.059	582.4715
62	7	56	62	6	57	E	1	261680.875	-0.059	582.4715
62	6	56	62	5	57	E	0	261688.981	-0.032	454.1921
62	7	56	62	6	57	E	0	261688.981	-0.032	454.1921
42	19	24	41	19	23	A	0	261704.435	-0.013	313.5881
42	19	23	41	19	22	A	0	261704.435	-0.014	313.5881
42	19	23	41	19	22	E	0	261706.622	0.199	313.5874
62	6	56	62	5	57	A	0	261714.883	-0.037	454.1879
62	7	56	62	6	57	A	0	261714.883	-0.037	454.1879
41	12	30	40	12	29	A	0	261773.042	-0.038	265.8133
41	12	30	40	12	29	E	0	261778.422	-0.016	265.8171
53	24	29	53	23	30	E	0	261780.975	-0.079	463.3823
53	24	30	53	23	31	E	0	261821.452	-0.088	463.3755
53	24	29	53	23	30	A	0	261823.468	-0.020	463.3882
53	24	30	53	23	31	A	0	261823.468	-0.020	463.3882
59	4	55	59	3	56	A	1	262159.576	0.290	531.3692
59	5	55	59	4	56	A	1	262159.576	0.290	531.3692
27	9	18	26	8	19	E	0	262182.286	-0.028	152.5911
27	9	18	26	8	19	A	0	262193.513	0.006	152.5834
42	18	25	41	18	24	A	0	262240.310	-0.041	306.6996
42	18	24	41	18	23	E	0	262242.286	-0.171	306.7005
42	18	25	41	18	24	E	0	262243.399	0.051	306.6887
52	24	28	52	23	29	E	0	262305.960	-0.069	452.3525
52	24	29	52	23	30	E	0	262346.771	0.007	452.3457
52	24	28	52	23	29	A	0	262348.192	-0.034	452.3586
52	24	29	52	23	30	A	0	262348.192	-0.034	452.3586
68	25	43	68	24	44	E	0	262661.311	-0.048	663.3982
68	25	44	68	24	45	E	0	262699.304	-0.063	663.3924
68	25	43	68	24	44	A	0	262705.446	0.242	663.4034
41	9	32	40	9	31	E	0	262739.410	-0.019	257.7791
41	9	32	40	9	31	A	0	262753.455	-0.023	257.7720
41	9	32	40	9	31	E	1	262804.193	0.059	386.0712
51	24	28	51	23	29	E	0	262839.797	-0.156	441.5317
51	24	27	51	23	28	A	0	262840.973	0.017	441.5446
51	24	28	51	23	29	A	0	262840.973	0.017	441.5446
42	17	26	41	17	25	A	0	262888.473	-0.011	300.2255
42	17	25	41	17	24	E	0	262890.719	-0.047	300.2281
42	17	26	41	17	25	E	0	262892.100	-0.069	300.2157
64	7	57	64	6	58	E	1	263062.516	0.071	615.0688
64	8	57	64	7	58	E	1	263062.516	0.071	615.0688
45	6	39	44	7	38	A	1	263093.388	-0.024	407.2659
45	7	39	44	6	38	A	1	263096.074	0.038	407.2659
46	5	41	45	5	40	A	1	263116.433	-0.030	410.1875
46	5	41	45	6	40	A	1	263116.433	0.018	410.1875
46	6	41	45	5	40	A	1	263116.433	-0.054	410.1875
46	6	41	45	6	40	A	1	263116.433	-0.006	410.1875
64	7	57	64	6	58	A	0	263140.998	-0.106	486.7921
64	8	57	64	7	58	A	0	263140.998	-0.106	486.7921
44	7	37	43	8	36	A	1	263205.864	-0.063	403.8446
47	4	43	46	4	42	A	1	263228.190	-0.063	412.6443
47	4	43	46	5	42	A	1	263228.190	-0.062	412.6443
47	5	43	46	4	42	A	1	263228.190	-0.064	412.6443
47	5	43	46	5	42	A	1	263228.190	-0.063	412.6443
44	8	37	43	8	36	A	1	263230.363	0.069	403.8446
46	5	41	45	5	40	E	1	263246.343	0.028	409.9860
46	5	41	45	6	40	E	1	263246.343	0.087	409.9860
46	6	41	45	5	40	E	1	263246.343	-0.001	409.9860
46	6	41	45	6	40	E	1	263246.343	0.057	409.9860
45	6	39	44	6	38	E	1	263247.524	0.032	407.0781
45	7	39	44	6	38	E	1	263248.604	0.028	407.0781
44	7	37	43	7	36	A	1	263249.832	0.158	403.8431
44	8	37	43	7	36	A	1	263274.019	-0.023	403.8431
45	6	39	44	7	38	E	0	263285.996	-0.060	278.7633
45	7	39	44	7	38	E	0	263287.179	0.116	278.7633
45	6	39	44	6	38	E	0	263287.811	-0.138	278.7632
40	11	29	39	11	28	A	0	263291.530	-0.083	254.0710
46	6	41	45	6	40	E	0	263291.530	0.056	281.6721
45	6	39	44	6	38	A	0	263292.725	0.072	278.7574
45	7	39	44	6	38	A	0	263293.735	0.068	278.7574
46	5	41	45	5	40	A	0	263295.481	-0.023	281.6658
46	5	41	45	6	40	A	0	263295.481	0.031	281.6658
46	6	41	45	5	40	A	0	263295.481	-0.050	281.6658
46	6	41	45	6	40	A	0	263295.481	0.004	281.6658
43	8	35	42	9	34	E	0	263315.417	-0.010	271.3832
43	8	35	42	9	34	A	0	263319.468	0.003	271.3776
47	4	43	46	4	42	E	1	263337.128	-0.026	412.4231
47	4	43	46	5	42	E	1	263337.128	-0.025	412.4231
47	5	43	46	4	42	E	1	263337.128	-0.027	412.4231
47	5	43	46	5	42	E	1	263337.128	-0.026	412.4231
47	4	43	46	4	42	E	0	263386.110	0.014	284.1118
47	5	43	46	5	42	E	0	263386.110	0.014	284.1118
47	4	43	46	4	42	A	0	263389.454	0.005	284.1049
47	4	43	46	5	42	A	0	263389.454	0.006	284.1049
47	5	43	46	4	42	A	0	263389.454	0.004	284.1049
47	5	43	46	5	42	A	0	263389.454	0.005	284.1049
48	3	45	47	3	44	A	1	263391.399	-0.051	414.6597
48	3	45	47	4	44	A	1	263391.399	-0.051	414.6597
48	4	45	47	3	44	A	1	263391.399	-0.051	414.6597
48	4	45	47	4	44	A	1	263391.399	-0.051	414.6597
44	8	37	43	8	36	E	1	263408.546	-0.040	403.6641
44	7	37	43	8	36	E	0	263415.904	-0.018	275.3504
44	7	37	43	8	36	A	0	263421.282	-0.006	275.3448
44	7	37	43	7	36	E	1	263430.746	-0.099	403.6624
44	8	37	43	8	36	E	0	263442.552	0.022	275.3504
44	8	37	43	8	36	A	0	263448.039	0.005	275.3448
44	8	37	43	7	36	E	1	263459.231	0.004	403.6624
44	7	37	43	7	36	E	0	263463.472	-0.065	275.3488
44	7	37	43	7	36	A	0	263469.175	0.035	275.3432
48	3	45	47	3	44	E	1	263479.036	-0.043	414.4129
48	3	45	47	4	44	E	1	263479.036	-0.043	414.4129
48	4	45	47	3	44	E	1	263479.036	-0.043	414.4129
48	4	45	47	4	44	E	1	263479.036	-0.043	414.4129
44	8	37	43	7	36	E	0	263490.253	0.108	275.3488
44	8	37	43	7	36	A	0	263495.874	-0.013	275.3432
48	3	45	47	3	44	E	0	263531.572	-0.001	286.1059
48	4	45	47	4	44	E	0	263531.572	-0.001	286.1059

J'	K'_a	K'_c	J''	K''_a	K''_c	Sym.	$v_{t_{CH_3}}$	$\nu_{Obs.}$	$\nu_{Obs.} - \nu_{Calc.}$	E_{low}
48	3	45	47	3	44	A	0	263534.258	-0.008	286.0982
48	3	45	47	4	44	A	0	263534.258	-0.008	286.0982
48	4	45	47	3	44	A	0	263534.258	-0.008	286.0982
48	4	45	47	4	44	A	0	263534.258	-0.008	286.0982
49	2	47	48	2	46	A	1	263584.587	-0.035	416.2503
49	2	47	48	3	46	A	1	263584.587	-0.035	416.2503
49	3	47	48	2	46	A	1	263584.587	-0.035	416.2503
49	3	47	48	3	46	A	1	263584.587	-0.035	416.2503
49	2	47	48	2	46	E	1	263649.418	0.003	415.9716
49	3	47	48	3	46	E	1	263649.418	0.003	415.9716
42	16	27	41	16	26	A	0	263688.271	0.012	294.1758
42	16	26	41	16	25	A	0	263689.691	-0.096	294.1759
42	16	26	41	16	25	E	0	263691.320	-0.050	294.1801
42	16	27	41	16	26	E	0	263693.434	-0.027	294.1673
49	24	25	49	23	26	E	0	263695.352	-0.061	420.5555
49	2	47	48	2	46	E	0	263705.732	0.005	287.6707
49	3	47	48	3	46	E	0	263705.732	0.005	287.6707
49	2	47	48	2	46	A	0	263707.721	0.006	287.6620
49	2	47	48	3	46	A	0	263707.721	0.006	287.6620
49	3	47	48	2	46	A	0	263707.721	0.006	287.6620
49	3	47	48	3	46	A	0	263707.721	0.006	287.6620
49	24	25	49	23	26	A	0	263736.850	-0.118	420.5618
49	24	26	49	23	27	A	0	263736.850	-0.118	420.5618
49	24	26	49	23	27	E	0	263736.850	0.055	420.5488
43	9	35	42	9	34	E	1	263782.196	0.013	399.6935
50	1	49	49	1	48	A	1	263795.269	0.014	417.4285
50	1	49	49	2	48	A	1	263795.269	0.014	417.4285
50	2	49	49	1	48	A	1	263795.269	0.014	417.4285
50	2	49	49	2	48	A	1	263795.269	0.014	417.4285
67	25	43	67	24	44	E	0	263807.380	-0.117	649.0968
43	9	35	42	9	34	E	0	263809.201	-0.020	271.3832
67	25	43	67	24	44	A	0	263812.652	-0.114	649.1080
43	9	35	42	9	34	A	0	263815.399	-0.029	271.3776
42	10	33	41	10	32	E	1	263820.763	-0.021	395.1494
50	1	49	49	1	48	E	1	263834.618	0.009	417.1111
50	2	49	49	2	48	E	1	263834.618	0.009	417.1111
42	10	33	41	10	32	E	0	263849.241	-0.005	266.8439
50	1	49	49	1	48	E	0	263895.341	-0.032	288.8181
50	2	49	49	2	48	E	0	263895.341	-0.032	288.8181
50	1	49	49	1	48	A	0	263896.605	0.023	288.8083
50	1	49	49	2	48	A	0	263896.605	0.023	288.8083
50	2	49	49	1	48	A	0	263896.605	0.023	288.8083
50	2	49	49	2	48	A	0	263896.605	0.023	288.8083
51	0	51	50	0	50	A	1	264015.957	0.105	418.2037
51	0	51	50	1	50	A	1	264015.957	0.105	418.2037
51	1	51	50	0	50	A	1	264015.957	0.105	418.2037
51	1	51	50	1	50	A	1	264015.957	0.105	418.2037
51	0	51	50	0	50	E	1	264026.249	0.081	417.8402
51	1	51	50	1	50	E	1	264026.249	0.081	417.8402
51	0	51	50	0	50	A	0	264092.522	-0.165	289.5459
51	0	51	50	1	50	A	0	264092.522	-0.165	289.5459
51	1	51	50	0	50	A	0	264092.522	-0.165	289.5459
51	1	51	50	1	50	A	0	264092.522	-0.165	289.5459
51	0	51	50	0	50	E	0	264092.522	0.161	289.5571
51	1	51	50	1	50	E	0	264092.522	0.161	289.5571
48	24	24	48	23	25	E	0	264101.908	-0.031	410.3857
16	15	1	15	14	1	E	0	264127.470	-0.051	129.2544
16	15	2	15	14	2	E	0	264132.313	0.029	129.2406
43	8	35	42	8	34	E	1	264133.851	-0.173	399.6644
48	24	24	48	23	25	A	0	264143.338	0.037	410.3921
48	24	25	48	23	26	A	0	264143.338	0.037	410.3921
48	24	25	48	23	26	E	0	264143.338	-0.167	410.3790
43	8	35	42	8	34	E	0	264144.953	-0.079	271.3555
43	8	35	42	8	34	A	0	264152.542	-0.008	271.3498
16	15	2	15	14	1	A	0	264170.853	0.001	129.2480
16	15	1	15	14	2	A	0	264170.853	0.001	129.2480
66	8	58	66	7	59	E	1	264228.626	0.240	648.7290
66	9	58	66	8	59	E	1	264228.626	0.239	648.7290
47	24	23	47	23	24	E	0	264482.530	-0.040	400.4297
47	24	23	47	23	24	A	0	264523.855	0.105	400.4362
47	24	24	47	23	25	A	0	264523.855	0.105	400.4362
47	24	24	47	23	25	E	0	264523.855	-0.452	400.4230
43	9	35	42	8	34	E	0	264638.806	-0.020	271.3555
43	9	35	42	8	34	A	0	264648.507	-0.006	271.3498
43	9	35	42	8	34	E	1	264655.415	-0.011	399.6644
42	15	28	41	15	27	A	0	264697.710	-0.078	288.5650
42	15	27	41	15	26	E	0	264709.513	-0.031	288.5711
42	15	28	41	15	27	E	0	264711.413	-0.020	288.5583
42	15	27	41	15	26	A	0	264715.126	-0.065	288.5656
66	25	41	66	24	42	E	0	264813.237	-0.047	635.0304
46	24	22	46	23	23	E	0	264838.653	0.017	390.6872
66	25	42	66	24	43	E	0	264852.207	-0.131	635.0246
66	25	41	66	24	42	A	0	264856.468	0.047	635.0360
46	24	22	46	23	23	A	0	264879.566	-0.077	390.6938
46	24	23	46	23	24	A	0	264879.566	-0.077	390.6938
46	24	23	46	23	24	E	0	264880.664	0.134	390.6805
44	10	34	43	11	33	A	0	264942.720	-0.062	288.7080
58	3	55	58	2	56	A	1	264961.977	-0.396	511.8653
58	4	55	58	3	56	A	1	264961.977	-0.396	511.8653
44	10	34	43	11	33	E	0	264981.383	-0.028	288.7130
61	5	56	61	4	57	E	0	265022.087	-0.003	433.8375
18	14	4	17	13	5	A	0	265025.530	-0.011	130.8870
18	14	5	17	13	4	A	0	265025.530	-0.011	130.8870
61	5	56	61	4	57	A	0	265052.222	-0.011	433.8322
61	6	56	61	5	57	A	0	265052.222	-0.011	433.8322
68	9	59	68	8	60	E	1	265167.687	0.541	683.4464
68	10	59	68	9	60	E	1	265167.687	0.538	683.4464
45	24	21	45	23	22	E	0	265171.344	-0.056	381.1577
45	24	21	45	23	22	A	0	265212.190	-0.054	381.1643
45	24	22	45	23	23	A	0	265212.190	-0.054	381.1643
45	24	22	45	23	23	E	0	265213.457	0.019	381.1510
43	30	13	42	30	12	A	0	265228.921	-0.056	424.1586
43	30	14	42	30	13	A	0	265228.921	-0.056	424.1586
43	30	13	42	30	12	E	0	265228.921	-0.086	424.1494
40	10	30	39	10	29	E	0	265283.051	-0.016	251.6034
40	10	30	39	10	29	A	0	265285.862	-0.060	251.5954
43	9	34	42	10	33	A	0	265287.121	-0.012	275.6395
43	9	34	42	10	33	E	0	265295.666	0.045	275.6450
68	9	59	68	8	60	A	0	265331.479	-0.201	555.1892
68	10	59	68	9	60	A	0	265331.479	-0.203	555.1892
41	12	29	40	12	28	A	0	265367.340	-0.110	266.0160
43	29	15	42	29	14	A	0	265367.340	-0.021	412.9519
43	29	14	42	29	13	A	0	265367.340	-0.021	412.9519
43	29	14	42	29	13	E	0	265367.340	-0.154	412.9416
41	12	29	40	12	28	E	0	265377.367	0.021	266.0206
44	24	20	44	23	21	E	0	265482.001	-0.061	371.8409

J'	K'_a	K'_c	J''	K''_a	K''_c	Sym.	$v_{t_{\mathrm{CH_3}}}$	$\nu_{\mathrm{Obs.}}$	$\nu_{\mathrm{Obs.}} - \nu_{\mathrm{Calc.}}$	E_{low}
44	24	20	44	23	21	A	0	265522.739	-0.012	371.8475
44	24	21	44	23	22	A	0	265522.739	-0.012	371.8475
44	24	21	44	23	22	E	0	265524.200	-0.033	371.8342
43	27	17	42	27	16	A	0	265689.025	-0.049	391.6971
43	27	16	42	27	15	A	0	265689.025	-0.049	391.6971
43	24	19	43	23	20	E	0	265771.747	-0.017	362.7362
20	13	8	19	12	8	E	0	265783.275	0.020	133.7544
20	13	7	19	12	7	E	0	265789.729	-0.048	133.7678
43	24	19	43	23	20	A	0	265812.268	-0.040	362.7430
43	24	20	43	23	21	A	0	265812.268	-0.040	362.7430
43	24	20	43	23	21	E	0	265814.068	0.009	362.7296
20	13	8	19	12	7	A	0	265827.339	0.000	133.7588
20	13	7	19	12	8	A	0	265827.339	0.000	133.7588
65	25	41	65	24	42	E	0	265837.725	-0.117	621.1752
65	25	40	65	24	41	A	0	265841.079	0.021	621.1867
26	10	17	25	9	16	A	0	265849.221	-0.090	150.1164
70	10	60	70	9	61	E	1	265865.858	0.702	719.2152
70	11	60	70	10	61	E	1	265865.858	0.693	719.2152
43	26	17	42	26	16	A	0	265877.428	-0.002	381.6507
43	26	18	42	26	17	A	0	265877.428	-0.002	381.6507
42	14	29	41	14	28	A	0	265978.590	-0.019	283.4139
26	10	16	25	9	16	E	0	265990.060	-0.062	150.1247
42	14	29	41	14	28	E	0	266019.446	0.002	283.4109
42	24	18	42	23	19	A	0	266081.955	-0.049	353.8503
42	24	19	42	23	20	A	0	266081.955	-0.049	353.8503
42	24	19	42	23	20	E	0	266084.010	0.006	353.8369
43	25	19	42	25	18	A	0	266088.359	-0.043	371.9932
43	25	18	42	25	17	A	0	266088.359	-0.043	371.9932
58	3	55	58	2	56	A	0	266114.737	0.129	383.3200
58	4	55	58	3	56	A	0	266114.737	0.129	383.3200
42	14	28	41	14	27	A	0	266138.518	-0.099	283.4200
26	10	17	25	9	17	E	0	266220.956	-0.077	150.1047
43	24	19	42	24	18	A	0	266326.019	-0.122	362.7258
43	24	20	42	24	19	A	0	266326.019	-0.122	362.7258
41	24	17	41	23	18	A	0	266332.827	-0.053	345.1691
41	24	18	41	23	19	A	0	266332.827	-0.053	345.1691
41	24	18	41	23	19	E	0	266335.119	0.007	345.1557
26	10	16	25	9	17	A	0	266437.278	0.054	150.0998
22	12	11	21	11	11	E	0	266438.787	-0.012	137.8832
22	12	10	21	11	10	E	0	266451.181	-0.051	137.8962
22	12	10	21	11	11	A	0	266485.037	-0.005	137.8860
40	24	16	40	23	17	E	0	266525.776	-0.001	336.6923
40	24	16	40	23	17	A	0	266565.858	-0.071	336.6991
40	24	17	40	23	18	A	0	266565.858	-0.071	336.6991
40	24	17	40	23	18	E	0	266568.403	0.027	336.6856
26	10	16	25	9	17	E	0	266589.850	-0.054	150.1047
43	23	21	42	23	20	A	0	266595.804	-0.035	353.8503
43	23	20	42	23	19	A	0	266595.804	-0.035	353.8503
43	23	21	42	23	20	E	0	266597.157	0.106	353.8369
63	6	57	63	5	58	E	1	266669.441	-0.174	594.1541
63	7	57	63	6	58	E	1	266669.441	-0.174	594.1541
63	6	57	63	5	58	A	0	266705.592	0.003	465.8733
63	7	57	63	6	58	A	0	266705.592	0.003	465.8733
64	25	39	64	24	40	E	0	266727.595	-0.044	607.5534
24	11	14	23	10	14	E	0	266736.218	0.024	143.3080
39	24	15	39	23	16	E	0	266742.016	-0.050	328.4331
24	11	13	23	10	13	E	0	266754.965	-0.085	143.3203
64	25	39	64	24	40	A	0	266769.994	0.007	607.5594
24	11	14	23	10	13	A	0	266779.975	-0.048	143.3092
39	24	15	39	23	16	A	0	266782.059	-0.043	328.4400
39	24	16	39	23	17	A	0	266782.059	-0.043	328.4400
39	24	16	39	23	17	E	0	266784.789	0.041	328.4265
24	11	13	23	10	14	A	0	266788.908	0.072	143.3089
43	22	21	42	22	20	A	0	266904.038	-0.026	345.3687
43	22	22	42	22	21	A	0	266904.038	-0.026	345.3687
43	22	22	42	22	21	E	0	266905.645	0.079	345.3554
42	9	33	41	9	32	E	0	266925.313	-0.020	266.5431
42	9	33	41	9	32	A	0	266938.576	-0.035	266.5365
38	24	14	38	23	15	E	0	266942.337	-0.045	320.3844
38	24	14	38	23	15	A	0	266982.263	-0.046	320.3914
38	24	15	38	23	16	A	0	266982.263	-0.046	320.3914
38	24	15	38	23	16	E	0	266985.215	0.075	320.3778
37	24	13	37	23	14	E	0	267127.590	-0.008	312.5459
37	24	13	37	23	14	A	0	267167.365	-0.057	312.5529
37	24	14	37	23	15	A	0	267167.365	-0.057	312.5529
37	24	14	37	23	15	E	0	267170.436	0.012	312.5393
43	21	23	42	21	22	A	0	267259.309	0.025	337.2836
43	21	22	42	21	21	A	0	267259.309	0.025	337.2836
43	21	23	42	21	22	E	0	267261.090	-0.030	337.2706
36	24	12	36	23	13	E	0	267298.532	-0.019	304.9173
36	24	12	36	23	13	A	0	267338.221	-0.058	304.9244
36	24	13	36	23	14	A	0	267338.221	-0.058	304.9244
36	24	13	36	23	14	E	0	267341.472	0.034	304.9107
42	11	32	41	11	31	E	0	267399.055	-0.040	270.6800
42	11	32	41	11	31	A	0	267400.293	0.006	270.6749
42	13	30	41	13	29	A	0	267441.027	-0.045	278.7449
42	13	30	41	13	29	E	0	267454.733	0.035	278.7467
35	24	11	35	23	12	E	0	267456.038	-0.006	297.4983
35	24	11	35	23	12	A	0	267495.658	-0.024	297.5054
35	24	12	35	23	13	A	0	267495.658	-0.024	297.5054
35	24	12	35	23	13	E	0	267498.997	0.012	297.4918
34	24	10	34	23	11	E	0	267600.837	-0.011	290.2887
63	25	38	63	24	39	E	0	267604.519	-0.023	594.1471
34	24	10	34	23	11	A	0	267640.355	-0.047	290.2957
34	24	11	34	23	12	A	0	267640.355	-0.047	290.2957
34	24	11	34	23	12	E	0	267643.844	0.007	290.2821
63	25	39	63	24	40	E	0	267645.020	0.098	594.1414
63	25	38	63	24	39	A	0	267646.480	-0.024	594.1532
43	20	23	42	20	22	A	0	267672.581	-0.034	329.5985
43	20	24	42	20	23	A	0	267672.581	-0.034	329.5985
43	20	24	42	20	23	E	0	267674.821	-0.020	329.5860
33	24	9	33	23	10	E	0	267733.773	0.070	283.2880
33	24	9	33	23	10	A	0	267773.175	-0.002	283.2951
33	24	10	33	23	11	A	0	267773.175	-0.002	283.2951
33	24	10	33	23	11	E	0	267776.784	0.050	283.2815
32	24	8	32	23	9	E	0	267855.329	0.008	276.4961
32	24	8	32	23	9	A	0	267894.672	-0.049	276.5033
32	24	9	32	23	10	A	0	267894.672	-0.049	276.5033
32	24	9	32	23	10	E	0	267898.446	0.057	276.4896
31	24	7	31	23	8	E	0	267966.422	0.038	269.9128
31	24	7	31	23	8	A	0	268005.626	-0.089	269.9199
31	24	8	31	23	9	A	0	268005.626	-0.089	269.9199
31	24	8	31	23	9	E	0	268009.554	0.070	269.9062
30	24	6	30	23	7	E	0	268067.569	0.020	263.5376
65	7	58	65	6	59	E	1	268076.259	0.042	627.2590
65	8	58	65	7	59	E	1	268076.259	0.042	627.2590

J'	K'_a	K'_c	J''	K''_a	K''_c	Sym.	$v_{t_{CH_3}}$	$\nu_{Obs.}$	$\nu_{Obs.} - \nu_{Calc.}$	E_{low}
30	24	6	30	23	7	A	0	268106.772	-0.043	263.5448
30	24	7	30	23	8	A	0	268106.772	-0.043	263.5448
30	24	7	30	23	8	E	0	268110.684	0.008	263.5311
60	4	56	60	3	57	E	0	268123.652	-0.021	413.7160
60	5	56	60	4	57	E	0	268123.652	-0.021	413.7160
65	7	58	65	6	59	A	0	268157.250	-0.181	498.9853
65	8	58	65	7	59	A	0	268157.250	-0.181	498.9853
43	19	25	42	19	24	A	0	268159.162	0.146	322.3176
43	19	24	42	19	23	A	0	268159.162	0.145	322.3176
29	24	5	29	23	6	E	0	268159.162	-0.286	257.3705
43	19	25	42	19	24	E	0	268161.475	-0.230	322.3059
46	6	40	45	7	39	A	1	268195.630	0.036	416.0418
46	7	40	45	6	39	A	1	268196.946	-0.038	416.0418
29	24	5	29	23	6	A	0	268198.616	-0.037	257.3777
29	24	6	29	23	7	A	0	268198.616	-0.037	257.3777
60	4	56	60	3	57	E	1	268215.377	-0.158	541.9960
60	5	56	60	4	57	E	1	268215.377	-0.158	541.9960
47	5	42	46	5	41	A	1	268231.303	-0.040	418.9641
47	5	42	46	6	41	A	1	268231.303	-0.016	418.9641
47	6	42	46	5	41	A	1	268231.303	-0.052	418.9641
47	6	42	46	6	41	A	1	268231.303	-0.028	418.9641
28	24	4	28	23	5	E	0	268242.631	-0.053	251.4112
28	24	4	28	23	5	A	0	268281.831	-0.004	251.4184
28	24	5	28	23	6	A	0	268281.831	-0.004	251.4184
28	24	5	28	23	6	E	0	268285.969	0.115	251.4047
45	7	38	44	8	37	A	1	268292.790	-0.001	412.6250
45	8	38	44	8	37	A	1	268306.308	0.057	412.6250
45	8	38	44	7	37	A	1	268330.656	0.038	412.6242
46	6	40	45	7	39	E	1	268345.711	0.001	415.8591
46	7	40	45	6	39	E	1	268347.383	0.011	415.8591
48	4	44	47	4	43	A	1	268349.274	-0.071	421.4246
48	4	44	47	5	43	A	1	268349.274	-0.070	421.4246
48	5	44	47	4	43	A	1	268349.274	-0.071	421.4246
48	5	44	47	5	43	A	1	268349.274	-0.070	421.4246
47	5	42	46	5	41	E	1	268359.911	-0.043	418.7669
47	5	42	46	6	41	E	1	268359.911	-0.013	418.7669
47	6	42	46	5	41	E	1	268359.911	-0.058	418.7669
47	6	42	46	6	41	E	1	268359.911	-0.028	418.7669
44	8	36	43	9	35	A	1	268367.188	0.079	408.6655
42	12	31	41	12	30	A	0	268381.209	-0.064	274.5451
42	12	31	41	12	30	E	0	268385.240	0.031	274.5491
46	6	40	45	7	39	A	0	268393.263	0.025	287.5399
46	7	40	45	6	39	A	0	268394.744	-0.045	287.5399
47	5	42	46	5	41	E	0	268407.120	-0.021	290.4545
47	6	42	46	6	41	E	0	268407.120	-0.007	290.4545
47	5	42	46	5	41	A	0	268411.086	-0.020	290.4483
47	5	42	46	6	41	A	0	268411.086	0.008	290.4483
47	6	42	46	5	41	A	0	268411.086	-0.034	290.4483
47	6	42	46	6	41	A	0	268411.086	-0.006	290.4483
48	4	44	47	4	43	E	1	268457.465	-0.031	421.2071
48	4	44	47	5	43	E	1	268457.465	-0.031	421.2071
48	5	44	47	4	43	E	1	268457.465	-0.032	421.2071
48	5	44	47	5	43	E	1	268457.465	-0.031	421.2071
45	7	38	44	8	37	E	1	268465.691	-0.053	412.4505
45	8	38	44	8	37	E	1	268481.554	0.034	412.4505
45	7	38	44	7	37	E	1	268494.169	0.043	412.4495
45	7	38	44	8	37	E	0	268503.485	-0.007	284.1379
48	4	44	47	4	43	E	0	268508.281	0.061	292.8974
48	5	44	47	5	43	E	0	268508.281	0.061	292.8974
48	4	44	47	4	43	A	0	268511.559	0.009	292.8906
48	4	44	47	5	43	A	0	268511.559	0.010	292.8906
48	5	44	47	4	43	A	0	268511.559	0.009	292.8906
48	5	44	47	5	43	A	0	268511.559	0.009	292.8906
49	3	46	48	3	45	A	1	268515.362	-0.039	423.4455
49	3	46	48	4	45	A	1	268515.362	-0.039	423.4455
49	4	46	48	3	45	A	1	268515.362	-0.039	423.4455
49	4	46	48	4	45	A	1	268515.362	-0.039	423.4455
45	8	38	44	8	37	E	0	268518.288	0.049	284.1379
45	8	38	44	8	37	A	0	268523.594	-0.060	284.1324
45	7	38	44	7	37	E	0	268530.318	0.217	284.1370
45	7	38	44	7	37	A	0	268535.555	-0.019	284.1315
45	8	38	44	7	37	E	0	268544.828	-0.019	284.1370
45	8	38	44	7	37	A	0	268550.384	-0.016	284.1315
44	8	36	43	9	35	E	0	268577.010	0.014	280.1829
44	8	36	43	9	35	A	0	268581.838	-0.028	280.1775
49	3	46	48	3	45	E	1	268602.582	-0.023	423.2016
49	3	46	48	4	45	E	1	268602.582	-0.023	423.2016
49	4	46	48	3	45	E	1	268602.582	-0.023	423.2016
49	4	46	48	4	45	E	1	268602.582	-0.023	423.2016
49	3	46	48	3	45	E	0	268656.720	-0.004	294.8964
49	4	46	48	4	45	E	0	268656.720	-0.004	294.8964
49	3	46	48	3	45	A	0	268659.395	-0.010	294.8888
49	3	46	48	4	45	A	0	268659.395	-0.010	294.8888
49	4	46	48	3	45	A	0	268659.395	-0.010	294.8888
49	4	46	48	4	45	A	0	268659.395	-0.010	294.8888
50	2	48	49	2	47	A	1	268709.847	0.018	425.0425
50	2	48	49	3	47	A	1	268709.847	0.018	425.0425
50	3	48	49	2	47	A	1	268709.847	0.018	425.0425
50	3	48	49	3	47	A	1	268709.847	0.018	425.0425
43	18	26	42	18	25	A	0	268739.187	-0.040	315.4470
50	2	48	49	2	47	E	1	268774.421	0.015	424.7660
50	3	48	49	3	47	E	1	268774.421	0.015	424.7660
44	8	36	43	8	35	A	1	268825.957	0.163	408.6502
50	2	48	49	2	47	E	0	268832.240	0.015	296.4670
50	3	48	49	3	47	E	0	268832.240	0.015	296.4670
50	2	48	49	2	47	A	0	268834.223	0.017	296.4584
50	2	48	49	3	47	A	0	268834.223	0.017	296.4584
50	3	48	49	2	47	A	0	268834.223	0.017	296.4584
50	3	48	49	3	47	A	0	268834.223	0.017	296.4584
44	9	36	43	9	35	E	1	268837.923	-0.017	408.4924
44	9	36	43	9	35	E	0	268867.165	-0.054	280.1829
44	9	36	43	9	35	A	0	268873.368	-0.053	280.1775
51	1	50	50	1	49	A	1	268921.028	0.040	426.2277
51	1	50	50	2	49	A	1	268921.028	0.040	426.2277
51	2	50	50	1	49	A	1	268921.028	0.040	426.2277
51	2	50	50	2	49	A	1	268921.028	0.040	426.2277
51	1	50	50	1	49	E	1	268960.246	0.007	425.9117
51	2	50	50	2	49	E	1	268960.246	0.007	425.9117
51	1	50	50	1	49	E	0	269022.395	-0.026	297.6207
51	2	50	50	2	49	E	0	269022.395	-0.026	297.6207
51	1	50	50	1	49	A	0	269023.650	0.024	297.6109
51	1	50	50	2	49	A	0	269023.650	0.024	297.6109
51	2	50	50	1	49	A	0	269023.650	0.024	297.6109
51	2	50	50	2	49	A	0	269023.650	0.024	297.6109
44	8	36	43	8	35	E	1	269052.029	0.071	408.4750
44	8	36	43	8	35	E	0	269070.743	-0.048	280.1665

J'	K_a'	K_c'	J''	K_a''	K_c''	Sym.	$v_{t_{CH_3}}$	$\nu_{Obs.}$	$\nu_{Obs.} - \nu_{Calc.}$	E_{low}
43	10	34	42	10	33	E	1	269082.751	-0.006	403.9496
44	9	36	43	8	35	A	1	269094.684	0.171	408.6502
52	0	52	51	0	51	A	1	269141.891	0.045	427.0103
52	0	52	51	1	51	A	1	269141.891	0.045	427.0103
52	1	52	51	0	51	A	1	269141.891	0.045	427.0103
52	1	52	51	1	51	A	1	269141.891	0.045	427.0103
52	0	52	51	0	51	E	1	269152.103	0.030	426.6472
52	1	52	51	1	51	E	1	269152.103	0.030	426.6472
61	25	36	61	24	37	E	0	269212.856	-0.070	567.9955
52	0	52	51	0	51	A	0	269219.779	-0.167	298.3550
52	0	52	51	1	51	A	0	269219.779	-0.167	298.3550
52	1	52	51	0	51	A	0	269219.779	-0.167	298.3550
52	1	52	51	1	51	A	0	269219.779	-0.167	298.3550
52	0	52	51	0	51	E	0	269219.779	0.156	298.3663
52	1	52	51	1	51	E	0	269219.779	0.156	298.3663
61	25	37	61	24	38	A	0	269254.091	-0.072	568.0019
61	25	36	61	24	37	A	0	269254.091	-0.068	568.0019
61	25	37	61	24	38	E	0	269254.091	0.042	567.9898
67	8	59	67	7	60	E	1	269273.285	0.237	661.4275
67	9	59	67	8	60	E	1	269273.285	0.237	661.4275
44	9	36	43	8	35	E	1	269359.389	0.046	408.4750
44	9	36	43	8	35	E	0	269360.992	-0.021	280.1665
44	9	36	43	8	35	A	0	269369.378	-0.006	280.1610
43	17	27	42	17	26	A	0	269443.212	0.100	308.9945
43	17	26	42	17	25	E	0	269445.613	-0.010	308.9972
43	17	27	42	17	26	E	0	269447.019	-0.154	308.9848
28	9	19	27	8	20	E	0	269790.239	-0.014	158.3012
60	25	35	60	24	36	E	0	269949.681	-0.079	555.2489
62	5	57	62	4	58	A	0	270020.082	-0.009	445.1810
62	6	57	62	5	58	A	0	270020.082	-0.009	445.1810
62	5	57	62	4	58	E	1	270028.894	-0.184	573.4643
62	6	57	62	5	58	E	1	270028.894	-0.184	573.4643
69	9	60	69	8	61	E	1	270249.663	0.462	696.6538
69	10	60	69	9	61	E	1	270249.663	0.461	696.6538
17	15	3	16	14	3	E	0	270278.494	0.038	132.5263
43	16	28	42	16	27	A	0	270315.182	0.017	302.9715
17	15	3	16	14	2	A	0	270317.020	-0.018	132.5336
17	15	2	16	14	3	A	0	270317.020	-0.018	132.5336
43	16	27	42	16	26	A	0	270318.278	-0.012	302.9716
43	16	27	42	16	26	E	0	270319.326	0.061	302.9758
43	16	28	42	16	27	E	0	270321.571	0.026	302.9632
69	9	60	69	8	61	A	0	270416.803	-0.157	568.4000
69	10	60	69	9	61	A	0	270416.803	-0.158	568.4000
59	25	34	59	24	35	E	0	270644.816	-0.114	542.7209
41	10	31	40	10	30	E	0	270959.947	-0.022	260.4522
41	10	31	40	10	30	A	0	270967.101	0.006	260.4444
59	3	56	59	2	57	A	0	271055.179	0.114	393.8262
59	4	56	59	3	57	A	0	271055.179	0.114	393.8262
27	10	18	26	9	17	A	0	271100.146	-0.004	155.6059
44	33	12	43	33	11	A	0	271111.440	-0.139	468.9244
44	33	11	43	33	10	A	0	271111.440	-0.139	468.9244
44	33	11	43	33	10	E	0	271111.440	0.048	468.9192
19	14	6	18	13	6	E	0	271119.976	-0.090	134.5852
19	14	5	18	13	5	E	0	271120.992	0.049	134.5989
19	14	5	18	13	6	A	0	271161.662	-0.000	134.5911
19	14	6	18	13	5	A	0	271161.662	-0.000	134.5911
44	32	12	43	32	11	A	0	271224.656	-0.055	456.5667
44	32	13	43	32	12	A	0	271224.656	-0.055	456.5667
44	32	12	43	32	11	E	0	271224.656	0.070	456.5601
43	9	34	42	9	33	E	0	271237.510	0.086	275.4468
41	11	30	40	11	29	A	0	271242.490	0.065	262.8535
43	9	34	42	9	33	A	0	271249.482	0.008	275.4406
41	11	30	40	11	29	E	0	271251.236	-0.028	262.8603
58	25	33	58	24	34	E	0	271300.623	-0.031	530.4110
27	10	17	26	9	17	E	0	271325.510	-0.069	155.6135
58	25	33	58	24	34	A	0	271340.900	-0.008	530.4178
58	25	34	58	24	35	E	0	271342.623	-0.096	530.4055
44	31	14	43	31	13	A	0	271347.852	-0.088	444.5936
44	31	13	43	31	12	A	0	271347.852	-0.088	444.5936
44	31	13	43	31	12	E	0	271347.852	-0.042	444.5857
43	15	29	42	15	28	A	0	271418.811	-0.020	297.3944
43	15	29	42	15	28	E	0	271439.195	-0.066	297.3881
43	15	28	42	15	27	E	0	271440.411	0.050	297.4009
43	15	28	42	15	27	A	0	271452.055	-0.016	297.3956
44	30	14	43	30	13	E	0	271482.673	-0.087	432.9965
44	29	16	43	29	15	A	0	271630.728	-0.015	421.8036
44	29	15	43	29	14	A	0	271630.728	-0.015	421.8036
44	29	15	43	29	14	E	0	271630.728	-0.173	421.7933
64	6	58	64	5	59	A	0	271693.036	-0.048	477.7294
64	7	58	64	6	59	A	0	271693.036	-0.048	477.7294
41	10	32	40	9	31	E	0	271757.281	-0.038	257.7791
41	10	32	40	9	31	A	0	271800.161	0.002	257.7720
40	10	31	39	9	30	E	0	271858.941	-0.053	249.1539
44	9	35	43	10	34	A	0	271888.030	-0.028	284.6163
44	9	35	43	10	34	E	0	271891.506	0.094	284.6216
21	13	9	20	12	9	E	0	271891.506	-0.070	137.8823
21	13	8	20	12	8	E	0	271898.160	-0.049	137.8957
40	10	31	39	9	30	A	0	271911.463	0.024	249.1463
57	25	32	57	24	33	E	0	271918.926	-0.073	518.3187
21	13	9	20	12	8	A	0	271935.722	-0.006	137.8867
27	10	18	26	9	18	E	0	271943.685	0.018	155.5796
57	25	33	57	24	34	A	0	271958.927	-0.029	518.3256
57	25	32	57	24	33	A	0	271958.927	-0.029	518.3256
57	25	33	57	24	34	E	0	271961.296	-0.040	518.3131
44	27	18	43	27	17	A	0	271975.218	0.062	400.5595
44	27	17	43	27	16	A	0	271975.218	0.062	400.5595
61	4	57	61	3	58	A	1	272027.926	0.276	553.2133
61	5	57	61	4	58	A	1	272027.926	0.276	553.2133
27	10	17	26	9	18	A	0	272245.269	-0.005	155.5738
27	10	17	26	9	18	E	0	272342.946	0.001	155.5796
44	25	20	43	25	19	A	0	272403.067	-0.084	380.8690
44	25	19	43	25	18	A	0	272403.067	-0.084	380.8690
23	12	12	22	11	12	E	0	272483.184	0.005	142.4434
23	12	11	22	11	11	E	0	272495.773	-0.069	142.4564
56	25	31	56	24	32	E	0	272501.849	-0.051	506.4433
23	12	11	22	11	12	A	0	272529.558	-0.069	142.4461
56	25	32	56	24	33	A	0	272541.550	-0.024	506.4504
56	25	31	56	24	32	A	0	272541.550	-0.024	506.4504
56	25	32	56	24	33	E	0	272544.480	-0.011	506.4378
25	11	15	24	10	15	E	0	272636.488	-0.050	148.3149
25	11	14	24	10	14	E	0	272655.512	-0.025	148.3271
44	24	20	43	24	19	A	0	272658.133	-0.068	371.6095
44	24	21	43	24	20	A	0	272658.133	-0.068	371.6095
25	11	15	24	10	14	A	0	272674.502	-0.027	148.3161
25	11	14	24	10	15	A	0	272695.439	0.049	148.3155
43	14	30	42	14	29	A	0	272801.649	0.032	292.2859

J'	K'_a	K'_c	J''	K''_a	K''_c	Sym.	$v_{t_{CH_3}}$	$\nu_{Obs.}$	$\nu_{Obs.} - \nu_{Calc.}$	E_{low}
43	14	30	42	14	29	E	0	272838.100	-0.035	292.2843
42	10	33	41	9	32	E	0	272867.130	-0.006	266.5431
42	10	33	41	9	32	A	0	272900.950	-0.003	266.5365
44	23	22	43	23	21	A	0	272947.683	-0.075	362.7430
44	23	21	43	23	20	A	0	272947.683	-0.075	362.7430
55	25	30	55	24	31	E	0	273051.127	-0.040	494.7845
43	14	29	42	14	28	E	0	273059.102	0.061	292.3013
43	14	29	42	14	28	A	0	273084.844	0.064	292.2974
55	25	31	55	24	32	A	0	273090.532	-0.040	494.7917
55	25	30	55	24	31	A	0	273090.532	-0.040	494.7917
55	25	31	55	24	32	E	0	273093.901	-0.094	494.7790
61	4	57	61	3	58	A	0	273109.503	0.039	424.7223
61	5	57	61	4	58	A	0	273109.503	0.039	424.7223
39	10	30	38	9	29	E	0	273157.686	-0.020	240.6724
66	7	59	66	6	60	A	0	273169.287	-0.095	511.3491
66	8	59	66	7	60	A	0	273169.287	-0.095	511.3491
43	11	33	42	11	32	E	0	273216.015	-0.029	279.5995
44	22	22	43	22	21	A	0	273278.949	-0.034	354.2716
44	22	23	43	22	22	A	0	273278.949	-0.034	354.2716
44	22	22	43	22	21	E	0	273280.721	-0.093	354.2663
47	6	41	46	6	40	A	1	273299.993	-0.112	424.9879
47	6	41	46	7	40	A	1	273299.993	0.367	424.9879
47	7	41	46	6	40	A	1	273299.993	-0.364	424.9879
47	7	41	46	7	40	A	1	273299.993	0.116	424.9879
48	5	43	47	5	42	A	1	273346.929	-0.043	427.9113
48	5	43	47	6	42	A	1	273346.929	-0.031	427.9113
48	6	43	47	5	42	A	1	273346.929	-0.050	427.9113
48	6	43	47	6	42	A	1	273346.929	-0.037	427.9113
46	7	39	45	8	38	A	1	273378.870	-0.060	421.5748
46	8	39	45	8	38	A	1	273386.327	0.020	421.5748
46	7	39	45	7	38	A	1	273392.418	0.028	421.5743
46	8	39	45	7	38	A	1	273399.811	0.043	421.5743
47	6	41	46	7	40	E	1	273447.884	0.030	424.8102
47	7	41	46	6	40	E	1	273448.713	-0.021	424.8101
49	4	45	48	4	44	A	1	273470.561	-0.062	430.3758
49	4	45	48	5	44	A	1	273470.561	-0.061	430.3758
49	5	45	48	4	44	A	1	273470.561	-0.062	430.3758
49	5	45	48	5	44	A	1	273470.561	-0.061	430.3758
48	5	43	47	5	42	E	1	273474.360	-0.020	427.7185
48	5	43	47	6	42	E	1	273474.360	-0.005	427.7185
48	6	43	47	5	42	E	1	273474.360	-0.028	427.7185
48	6	43	47	6	42	E	1	273474.360	-0.013	427.7185
47	6	41	46	6	40	E	0	273493.434	-0.133	296.4981
47	7	41	46	7	40	E	0	273493.434	0.120	296.4981
47	6	41	46	6	40	A	0	273497.958	-0.189	296.4925
47	6	41	46	7	40	A	0	273497.958	0.348	296.4925
47	7	41	46	6	40	A	0	273497.958	-0.472	296.4925
47	7	41	46	7	40	A	0	273497.958	0.065	296.4925
48	5	43	47	5	42	E	0	273523.540	-0.018	299.4076
48	6	43	47	6	42	E	0	273523.540	-0.011	299.4076
48	5	43	47	5	42	A	0	273527.479	-0.008	299.4016
48	5	43	47	6	42	A	0	273527.479	0.006	299.4016
48	6	43	47	5	42	A	0	273527.479	-0.015	299.4016
48	6	43	47	6	42	A	0	273527.479	-0.001	299.4016
45	8	37	44	9	36	A	1	273532.398	0.087	417.6262
42	12	30	41	12	29	E	0	273535.485	-0.155	274.8726
46	7	39	45	8	38	E	1	273549.874	-0.019	421.4061
46	8	39	45	8	38	E	1	273558.586	-0.007	421.4061
54	25	29	54	24	30	E	0	273568.531	0.027	483.3417
46	8	39	45	7	38	E	1	273574.371	0.002	421.4055
49	4	45	48	4	44	E	1	273578.012	-0.030	430.1619
49	4	45	48	5	44	E	1	273578.012	-0.030	430.1619
49	5	45	48	4	44	E	1	273578.012	-0.030	430.1619
49	5	45	48	5	44	E	1	273578.012	-0.030	430.1619
46	7	39	45	8	38	E	0	273589.945	-0.018	293.0947
46	7	39	45	8	38	A	0	273595.241	-0.001	293.0894
46	8	39	45	8	38	E	0	273598.082	0.008	293.0947
46	8	39	45	8	38	A	0	273603.386	-0.012	293.0894
46	7	39	45	7	38	E	0	273604.705	-0.006	293.0942
54	25	30	54	24	31	A	0	273607.629	-0.024	483.3489
54	25	29	54	24	30	A	0	273607.629	-0.024	483.3489
46	7	39	45	7	38	A	0	273610.063	-0.006	293.0889
54	25	30	54	24	31	E	0	273611.466	-0.087	483.3362
46	8	39	45	7	38	E	0	273612.807	-0.014	293.0942
46	8	39	45	7	38	A	0	273618.183	-0.042	293.0889
49	4	45	48	4	44	E	0	273630.537	-0.009	301.8539
49	5	45	48	5	44	E	0	273630.537	-0.009	301.8539
49	4	45	48	4	44	A	0	273633.847	-0.007	301.8472
49	4	45	48	5	44	A	0	273633.847	-0.006	301.8472
49	5	45	48	4	44	A	0	273633.847	-0.007	301.8472
49	5	45	48	5	44	A	0	273633.847	-0.006	301.8472
50	3	47	49	3	46	A	1	273639.245	-0.067	432.4022
50	3	47	49	4	46	A	1	273639.245	-0.067	432.4022
50	4	47	49	3	46	A	1	273639.245	-0.067	432.4022
50	4	47	49	4	46	A	1	273639.245	-0.067	432.4022
44	21	24	43	21	23	A	0	273661.056	-0.075	346.1984
44	21	23	43	21	22	A	0	273661.056	-0.075	346.1984
44	21	24	43	21	23	E	0	273663.097	-0.024	346.1855
45	9	37	44	9	36	A	1	273688.164	0.203	417.6262
45	8	37	44	9	36	E	1	273708.500	-0.006	417.4598
50	3	47	49	3	46	E	1	273726.064	-0.036	432.1612
50	3	47	49	4	46	E	1	273726.064	-0.036	432.1612
50	4	47	49	3	46	E	1	273726.064	-0.036	432.1612
50	4	47	49	4	46	E	1	273726.064	-0.036	432.1612
45	8	37	44	9	36	E	0	273750.950	0.036	289.1514
45	8	37	44	9	36	A	0	273756.257	0.000	289.1462
50	3	47	49	3	46	E	0	273781.844	-0.002	303.8578
50	4	47	49	4	46	E	0	273781.844	-0.002	303.8578
50	3	47	49	3	46	A	0	273784.511	-0.001	303.8503
50	3	47	49	4	46	A	0	273784.511	-0.001	303.8503
50	4	47	49	3	46	A	0	273784.511	-0.001	303.8503
50	4	47	49	4	46	A	0	273784.511	-0.001	303.8503
45	8	37	44	8	36	A	1	273801.178	0.148	417.6172
51	2	49	50	2	48	A	1	273834.865	-0.046	434.0057
51	2	49	50	3	48	A	1	273834.865	-0.046	434.0057
51	3	49	50	2	48	A	1	273834.865	-0.046	434.0057
51	3	49	50	3	48	A	1	273834.865	-0.046	434.0057
45	9	37	44	9	36	E	1	273887.675	0.018	417.4598
51	2	49	50	2	48	E	1	273899.280	0.001	433.7314
51	3	49	50	3	48	E	1	273899.280	0.001	433.7314
45	9	37	44	9	36	A	0	273925.688	-0.018	289.1462
51	2	49	50	2	48	E	0	273958.608	0.004	305.4343
51	3	49	50	3	48	E	0	273958.608	0.004	305.4343
51	2	49	50	2	48	A	0	273960.593	0.014	305.4257
51	2	49	50	3	48	A	0	273960.593	0.014	305.4257
51	3	49	50	2	48	A	0	273960.593	0.014	305.4257

J'	K_a'	K_c'	J''	K_a''	K_c''	Sym.	$v_{t_{\mathrm{CH_3}}}$	$\nu_{\mathrm{Obs.}}$	$\nu_{\mathrm{Obs.}} - \nu_{\mathrm{Calc.}}$	E_{low}
51	3	49	50	3	48	A	0	273960.593	0.014	305.4257
45	8	37	44	8	36	E	1	274015.837	-0.055	417.4496
45	8	37	44	8	36	E	1	274015.837	-0.055	417.4496
52	1	51	51	1	50	A	1	274046.574	0.002	435.1980
52	1	51	51	2	50	A	1	274046.574	0.002	435.1980
52	2	51	51	1	50	A	1	274046.574	0.002	435.1980
52	2	51	51	2	50	A	1	274046.574	0.002	435.1980
53	25	28	53	24	29	E	0	274055.397	-0.117	472.1144
52	1	51	51	1	50	E	1	274085.729	0.006	434.8833
52	2	51	51	2	50	E	1	274085.729	0.006	434.8833
53	25	29	53	24	30	A	0	274094.376	-0.041	472.1217
53	25	28	53	24	29	A	0	274094.376	-0.041	472.1217
53	25	29	53	24	30	E	0	274098.702	-0.064	472.1089
44	20	24	43	20	23	A	0	274106.381	-0.035	338.5270
44	20	25	43	20	24	A	0	274106.381	-0.035	338.5270
44	20	25	43	20	24	E	0	274108.739	-0.087	338.5147
52	1	51	51	1	50	E	0	274149.295	-0.027	306.5944
52	2	51	51	2	50	E	0	274149.295	-0.027	306.5944
52	1	51	51	1	50	A	0	274150.551	0.027	306.5846
52	1	51	51	2	50	A	0	274150.551	0.027	306.5846
52	2	51	51	1	50	A	0	274150.551	0.027	306.5846
52	2	51	51	2	50	A	0	274150.551	0.027	306.5846
45	9	37	44	8	36	E	1	274195.074	0.032	417.4496
45	9	37	44	8	36	E	0	274209.771	-0.009	289.1417
45	9	37	44	8	36	A	0	274217.198	-0.062	289.1364
43	13	31	42	13	30	A	0	274264.358	-0.036	287.6657
53	0	53	52	0	52	A	1	274267.910	0.223	435.9879
53	0	53	52	1	52	A	1	274267.910	0.223	435.9879
53	1	53	52	0	52	A	1	274267.910	0.223	435.9879
53	1	53	52	1	52	A	1	274267.910	0.223	435.9879
53	0	53	52	0	52	E	1	274277.847	0.021	435.6251
53	0	53	52	1	52	E	1	274277.847	0.021	435.6251
53	1	53	52	0	52	E	1	274277.847	0.021	435.6251
53	1	53	52	1	52	E	1	274277.847	0.021	435.6251
44	10	35	43	10	34	E	0	274292.274	0.054	284.6216
44	10	35	43	10	34	A	0	274298.273	-0.011	284.6163
68	8	60	68	7	61	E	1	274311.883	0.273	674.2964
68	9	60	68	8	61	E	1	274311.883	0.273	674.2964
53	0	53	52	0	52	A	0	274346.889	-0.165	307.3353
53	0	53	52	1	52	A	0	274346.889	-0.165	307.3353
53	1	53	52	0	52	A	0	274346.889	-0.165	307.3353
53	1	53	52	1	52	A	0	274346.889	-0.165	307.3353
53	0	53	52	0	52	E	0	274346.889	0.156	307.3465
53	1	53	52	1	52	E	0	274346.889	0.156	307.3465
68	8	60	68	7	61	A	0	274439.839	-0.132	546.0349
68	9	60	68	8	61	A	0	274439.839	-0.132	546.0349
52	25	27	52	24	28	E	0	274513.639	-0.069	461.1021
52	25	28	52	24	29	A	0	274552.345	-0.033	461.1096
52	25	27	52	24	28	A	0	274552.345	-0.033	461.1096
52	25	28	52	24	29	E	0	274557.111	-0.038	461.0967
44	19	26	43	19	25	A	0	274631.337	-0.034	331.2624
44	19	25	43	19	24	A	0	274631.337	-0.036	331.2624
43	12	32	42	12	31	A	0	274867.426	0.076	283.4974
43	12	32	42	12	31	E	0	274869.958	-0.060	283.5014
45	10	35	44	11	34	A	0	274881.617	-0.054	298.0099
45	10	35	44	11	34	E	0	274908.541	-0.009	298.0148
49	12	37	48	13	36	E	0	274921.920	0.020	345.8944
51	25	26	51	24	27	E	0	274944.498	-0.015	450.3045
51	25	27	51	24	28	A	0	274982.952	-0.012	450.3121
51	25	26	51	24	27	A	0	274982.952	-0.012	450.3121
63	5	58	63	4	59	A	0	274985.823	-0.007	456.7007
63	6	58	63	5	59	A	0	274985.823	-0.007	456.7007
51	25	27	51	24	28	E	0	274988.138	0.007	450.2991
63	5	58	63	4	59	E	1	274993.191	-0.035	584.9813
63	6	58	63	5	59	E	1	274993.191	-0.035	584.9813
43	10	34	42	9	33	E	0	275054.638	-0.015	275.4468
44	18	27	43	18	26	A	0	275259.093	-0.016	324.4111
44	18	26	43	18	25	E	0	275261.503	-0.044	324.4123
44	18	27	43	18	26	E	0	275262.694	0.051	324.4005
70	9	61	70	8	62	E	1	275323.744	0.475	710.0314
70	10	61	70	9	62	E	1	275323.744	0.475	710.0314
69	26	44	69	25	45	A	0	275347.775	0.057	686.6469
50	25	25	50	24	26	E	0	275349.208	-0.077	439.7211
50	25	26	50	24	27	A	0	275387.343	-0.182	439.7287
50	25	25	50	24	26	A	0	275387.343	-0.182	439.7287
50	25	26	50	24	27	E	0	275393.052	-0.013	439.7157
38	10	29	37	9	28	E	0	275457.491	-0.077	232.3465
70	9	61	70	8	62	A	0	275494.336	-0.162	581.7811
70	10	61	70	9	62	A	0	275494.336	-0.163	581.7811
16	16	0	15	15	0	E	0	275553.261	-0.063	134.7810
16	16	1	15	15	1	E	0	275563.543	0.028	134.7674
16	16	0	15	15	1	A	0	275598.611	-0.006	134.7761
16	16	1	15	15	0	A	0	275598.611	-0.006	134.7761
44	9	35	43	9	34	E	0	275708.689	0.049	284.4943
44	9	35	43	9	34	A	0	275719.394	-0.010	284.4885
49	25	24	49	24	25	E	0	275729.321	0.015	429.3514
44	9	35	43	9	34	E	1	275734.272	0.064	412.7920
49	25	25	49	24	26	A	0	275767.317	-0.030	429.3592
49	25	24	49	24	25	A	0	275767.317	-0.030	429.3592
49	25	25	49	24	26	E	0	275773.223	-0.013	429.3461
28	10	19	27	9	18	E	0	275862.990	-0.033	161.3366
28	10	19	27	9	18	A	0	275988.501	-0.066	161.3293
42	10	32	41	10	31	E	0	276005.301	-0.034	269.4905
42	10	32	41	10	31	A	0	276016.316	0.013	269.4829
44	17	28	43	17	27	A	0	276023.432	0.214	317.9822
44	17	27	43	17	26	E	0	276026.027	-0.013	317.9849
44	17	28	43	17	27	E	0	276027.733	-0.016	317.9726
43	13	30	42	13	29	A	0	276064.051	-0.050	287.7552
43	13	30	42	13	29	E	0	276068.998	0.074	287.7586
48	25	23	48	24	24	E	0	276085.754	-0.042	419.1952
48	25	24	48	24	25	A	0	276123.600	-0.047	419.2030
48	25	23	48	24	24	A	0	276123.600	-0.047	419.2030
48	25	24	48	24	25	E	0	276129.843	-0.021	419.1899
68	26	42	68	25	43	A	0	276349.182	-0.011	672.1663
68	26	43	68	25	44	E	0	276350.481	-0.109	672.1551
18	15	3	17	14	3	E	0	276417.865	-0.039	136.0330
47	25	22	47	24	23	E	0	276419.860	-0.053	409.2519
18	15	4	17	14	4	E	0	276422.635	0.032	136.0193
47	25	23	47	24	24	A	0	276457.472	-0.114	409.2598
47	25	22	47	24	23	A	0	276457.472	-0.114	409.2598
18	15	4	17	14	3	A	0	276461.195	-0.010	136.0266
18	15	3	17	14	4	A	0	276461.195	-0.010	136.0266
47	25	23	47	24	24	E	0	276464.107	-0.002	409.2466
65	6	59	65	5	60	A	0	276677.500	-0.073	489.7563
65	7	59	65	6	60	A	0	276677.500	-0.073	489.7563
46	25	21	46	24	22	E	0	276732.724	-0.041	399.5213

J'	K'_a	K'_c	J''	K''_a	K''_c	Sym.	$v_{t_{CH_3}}$	$\nu_{Obs.}$	$\nu_{Obs.} - \nu_{Calc.}$	E_{low}
46	25	22	46	24	23	A	0	276770.211	-0.057	399.5292
46	25	21	46	24	22	A	0	276770.211	-0.057	399.5292
46	25	22	46	24	23	E	0	276777.124	0.047	399.5160
44	16	29	43	16	28	A	0	276973.754	-0.011	311.9883
44	16	28	43	16	27	A	0	276979.875	-0.133	311.9885
44	16	28	43	16	27	E	0	276979.875	0.206	311.9927
44	16	29	43	16	28	E	0	276982.046	-0.028	311.9801
45	25	20	45	24	21	E	0	277025.364	-0.042	390.0029
45	36	9	44	36	8	A	0	277037.026	-0.045	517.3351
45	36	10	44	36	9	A	0	277037.026	-0.045	517.3351
45	36	10	44	36	9	E	0	277037.026	-0.230	517.3230
45	25	21	45	24	22	A	0	277062.964	0.218	390.0109
45	25	20	45	24	21	A	0	277062.964	0.218	390.0109
45	25	21	45	24	22	E	0	277069.833	0.008	389.9976
20	14	7	19	13	7	E	0	277250.276	-0.099	138.4980
20	14	6	19	13	6	E	0	277251.405	0.085	138.5117
20	14	6	19	13	7	A	0	277291.995	-0.014	138.5039
20	14	7	19	13	6	A	0	277291.995	-0.014	138.5039
67	26	41	67	25	42	A	0	277296.255	-0.034	657.9078
44	25	20	44	24	21	A	0	277336.017	-0.013	380.7044
44	25	19	44	24	20	A	0	277336.017	-0.013	380.7044
44	25	20	44	24	21	E	0	277343.395	0.036	380.6911
45	33	13	44	33	12	A	0	277346.136	-0.206	477.9677
45	33	12	44	33	11	A	0	277346.136	-0.206	477.9677
45	33	12	44	33	11	E	0	277346.136	-0.016	477.9625
45	32	13	44	32	12	A	0	277467.031	0.011	465.6138
45	32	14	44	32	13	A	0	277467.031	0.011	465.6138
45	32	13	44	32	12	E	0	277467.031	0.132	465.6071
43	25	18	43	24	19	E	0	277553.974	-0.062	371.6014
43	25	19	43	24	20	A	0	277591.134	0.054	371.6095
43	25	18	43	24	19	A	0	277591.134	0.054	371.6095
45	31	15	44	31	14	A	0	277598.605	0.069	453.6448
45	31	14	44	31	13	A	0	277598.605	0.069	453.6448
43	25	19	43	24	20	E	0	277598.605	-0.038	371.5962
45	31	14	44	31	13	E	0	277598.605	0.105	453.6368
42	25	17	42	24	18	E	0	277791.886	-0.025	362.7177
42	25	18	42	24	19	A	0	277828.877	0.056	362.7258
42	25	17	42	24	18	A	0	277828.877	0.056	362.7258
42	25	18	42	24	19	E	0	277836.610	0.011	362.7125
45	9	36	44	10	35	A	0	277928.127	-0.103	293.7660
45	9	36	44	10	35	E	0	277928.127	0.111	293.7710
22	13	10	21	12	10	E	0	277987.367	0.024	142.2208
22	13	9	21	12	9	E	0	277994.061	-0.048	142.2343
41	25	16	41	24	17	E	0	278013.315	-0.037	354.0449
22	13	10	21	12	9	A	0	278031.575	-0.006	142.2252
41	25	17	41	24	18	A	0	278050.100	-0.033	354.0530
41	25	16	41	24	17	A	0	278050.100	-0.033	354.0530
41	25	17	41	24	18	E	0	278058.143	0.030	354.0396
62	4	58	62	3	59	A	0	278059.370	0.104	435.9060
62	5	58	62	4	59	A	0	278059.370	0.104	435.9060
45	28	17	44	28	16	A	0	278075.198	0.029	420.0539
45	28	18	44	28	17	A	0	278075.198	0.029	420.0539
45	28	17	44	28	16	E	0	278075.198	-0.299	420.0427
45	28	18	44	28	17	E	0	278076.363	0.125	420.0418
67	7	60	67	6	61	E	1	278090.327	0.043	652.1514
67	8	60	67	7	61	E	1	278090.327	0.043	652.1514
44	10	35	43	9	34	E	0	278109.484	0.036	284.4943
44	10	35	43	9	34	A	0	278129.643	0.014	284.4885
28	10	18	27	9	19	A	0	278132.890	0.001	161.2700
28	10	18	27	9	19	E	0	278181.194	-0.006	161.2762
66	26	40	66	25	41	A	0	278191.993	-0.025	643.8707
66	26	41	66	25	42	E	0	278194.873	-0.201	643.8592
44	15	30	43	15	29	E	0	278207.415	-0.020	306.4423
44	15	29	43	15	28	A	0	278239.789	0.015	306.4502
40	25	16	40	24	17	A	0	278255.745	-0.120	345.5908
40	25	15	40	24	16	A	0	278255.745	-0.120	345.5908
40	25	16	40	24	17	E	0	278263.988	-0.044	345.5774
45	27	19	44	27	18	A	0	278268.843	0.012	409.6316
45	27	18	44	27	17	A	0	278268.843	0.012	409.6316
45	27	18	44	27	17	E	0	278270.089	0.050	409.6203
48	6	42	47	6	41	A	1	278405.506	-0.087	434.1042
48	6	42	47	7	41	A	1	278405.506	0.164	434.1042
48	7	42	47	6	41	A	1	278405.506	-0.218	434.1042
48	7	42	47	7	41	A	1	278405.506	0.033	434.1042
39	25	14	39	24	15	E	0	278410.298	0.015	337.3306
39	25	15	39	24	16	A	0	278446.802	-0.028	337.3389
39	25	14	39	24	15	A	0	278446.802	-0.028	337.3389
39	25	15	39	24	16	E	0	278455.157	-0.012	337.3254
49	5	44	48	5	43	A	1	278463.194	-0.050	437.0292
49	5	44	48	6	43	A	1	278463.194	-0.044	437.0292
49	6	44	48	5	43	A	1	278463.194	-0.053	437.0292
49	6	44	48	6	43	A	1	278463.194	-0.047	437.0292
47	7	40	46	8	39	A	1	278466.305	0.025	430.6939
47	8	40	46	8	39	A	1	278470.344	0.050	430.6939
47	7	40	46	7	39	A	1	278473.665	0.007	430.6937
47	8	40	46	7	39	A	1	278477.695	0.023	430.6937
26	11	15	25	10	15	E	0	278502.073	0.000	153.5522
24	12	13	23	11	13	E	0	278502.073	-0.134	147.2172
26	11	16	25	10	15	A	0	278508.617	-0.020	153.5415
24	12	12	23	11	12	E	0	278515.126	-0.018	147.2301
29	9	20	28	8	21	E	0	278533.197	-0.026	164.2221
29	9	20	28	8	21	A	0	278545.375	0.033	164.2143
24	12	13	23	11	12	A	0	278548.757	0.204	147.2198
48	6	42	47	6	41	E	1	278551.948	-0.093	433.9314
48	6	42	47	7	41	E	1	278551.948	0.211	433.9314
48	7	42	47	6	41	E	1	278551.948	-0.253	433.9314
48	7	42	47	7	41	E	1	278551.948	0.051	433.9314
26	11	15	25	10	16	A	0	278555.881	0.012	153.5402
38	25	13	38	24	14	E	0	278587.360	-0.008	329.2886
49	5	44	48	5	43	E	1	278589.441	-0.042	436.8406
49	5	44	48	6	43	E	1	278589.441	-0.034	436.8406
49	6	44	48	5	43	E	1	278589.441	-0.045	436.8406
49	6	44	48	6	43	E	1	278589.441	-0.038	436.8406
50	4	46	49	4	45	A	1	278591.978	-0.071	439.4978
50	4	46	49	5	45	A	1	278591.978	-0.071	439.4978
50	5	46	49	4	45	A	1	278591.978	-0.071	439.4978
50	5	46	49	5	45	A	1	278591.978	-0.071	439.4978
48	6	42	47	6	41	E	0	278599.395	-0.071	305.6209
48	7	42	47	7	41	E	0	278599.395	0.062	305.6209
48	6	42	47	6	41	A	0	278603.919	-0.070	305.6154
48	6	42	47	7	41	A	0	278603.919	0.213	305.6154
48	7	42	47	6	41	A	0	278603.919	-0.218	305.6154
48	7	42	47	7	41	A	0	278603.919	0.065	305.6154
47	11	36	46	12	35	A	0	278620.018	-0.199	321.4167
38	25	14	38	24	15	A	0	278623.788	-0.020	329.2969
38	25	13	38	24	14	A	0	278623.788	-0.020	329.2969

J'	K'_a	K'_c	J''	K''_a	K''_c	Sym.	$v_{t_{CH_3}}$	$\nu_{Obs.}$	$\nu_{Obs.} - \nu_{Calc.}$	E_{low}
38	25	14	38	24	15	E	0	278632.319	0.011	329.2835
47	7	40	46	8	39	E	1	278634.928	0.010	430.5310
49	5	44	48	5	43	E	0	278640.639	-0.004	308.5314
49	6	44	48	6	43	E	0	278640.639	-0.001	308.5314
49	5	44	48	5	43	A	0	278644.542	0.005	308.5255
49	5	44	48	6	43	A	0	278644.542	0.013	308.5255
49	6	44	48	5	43	A	0	278644.542	0.002	308.5255
49	6	44	48	6	43	A	0	278644.542	0.009	308.5255
47	8	40	46	7	39	E	1	278648.361	-0.023	430.5307
46	8	38	45	9	37	A	1	278650.854	0.055	426.7555
47	11	36	46	12	35	E	0	278675.906	0.054	321.4209
47	7	40	46	8	39	E	0	278677.442	-0.021	302.2210
47	8	40	46	8	39	E	0	278681.657	-0.234	302.2210
47	7	40	46	8	39	A	0	278682.706	0.032	302.2158
47	7	40	46	7	39	E	0	278685.669	0.096	302.2207
47	8	40	46	8	39	A	0	278687.103	-0.025	302.2158
47	8	40	46	7	39	E	0	278689.965	-0.038	302.2207
47	7	40	46	7	39	A	0	278691.019	0.189	302.2156
47	8	40	46	7	39	A	0	278695.283	-0.001	302.2156
50	4	46	49	4	45	E	1	278698.736	-0.015	439.2874
50	4	46	49	5	45	E	1	278698.736	-0.015	439.2874
50	5	46	49	4	45	E	1	278698.736	-0.015	439.2874
50	5	46	49	5	45	E	1	278698.736	-0.015	439.2874
37	25	12	37	24	13	E	0	278751.173	-0.038	321.4563
50	4	46	49	4	45	E	0	278753.033	-0.002	310.9813
50	5	46	49	5	45	E	0	278753.033	-0.002	310.9813
50	4	46	49	4	45	A	0	278756.336	0.015	310.9747
50	4	46	49	5	45	A	0	278756.336	0.015	310.9747
50	5	46	49	4	45	A	0	278756.336	0.015	310.9747
50	5	46	49	5	45	A	0	278756.336	0.015	310.9747
42	11	31	41	11	30	E	0	278761.843	-0.102	271.9083
51	3	48	50	3	47	A	1	278763.111	-0.060	441.5298
51	3	48	50	4	47	A	1	278763.111	-0.060	441.5298
51	4	48	50	3	47	A	1	278763.111	-0.060	441.5298
51	4	48	50	4	47	A	1	278763.111	-0.060	441.5298
37	25	13	37	24	14	A	0	278787.519	-0.031	321.4647
37	25	12	37	24	13	A	0	278787.519	-0.031	321.4647
37	25	13	37	24	14	E	0	278796.202	0.004	321.4512
46	8	38	45	8	37	A	1	278806.449	-0.001	426.7503
51	3	48	50	3	47	E	1	278849.527	-0.021	441.2918
51	4	48	50	4	47	E	1	278849.527	-0.021	441.2918
44	11	34	43	11	33	E	0	278859.945	-0.021	288.7130
44	11	34	43	11	33	A	0	278863.406	-0.040	288.7080
46	8	38	45	9	37	E	0	278874.374	-0.020	298.2884
46	8	38	45	9	37	A	0	278879.966	-0.012	298.2833
46	9	38	45	8	37	A	1	278895.842	0.165	426.7503
36	25	11	36	24	12	E	0	278902.514	-0.018	313.8334
51	3	48	50	3	47	E	0	278906.919	-0.003	312.9902
51	4	48	50	4	47	E	0	278906.919	-0.003	312.9902
51	3	48	50	3	47	A	0	278909.577	0.000	312.9828
51	3	48	50	4	47	A	0	278909.577	0.000	312.9828
51	4	48	50	3	47	A	0	278909.577	0.000	312.9828
51	4	48	50	4	47	A	0	278909.577	0.000	312.9828
36	25	12	36	24	13	A	0	278938.716	-0.061	313.8418
36	25	11	36	24	12	A	0	278938.716	-0.061	313.8418
36	25	12	36	24	13	E	0	278947.606	0.045	313.8283
52	2	50	51	2	49	A	1	278959.823	-0.042	443.1399
52	2	50	51	3	49	A	1	278959.823	-0.042	443.1399
52	3	50	51	2	49	A	1	278959.823	-0.042	443.1399
52	3	50	51	3	49	A	1	278959.823	-0.042	443.1399
46	9	38	45	9	37	E	0	278971.276	-0.103	298.2884
46	9	38	45	9	37	A	0	278977.318	-0.127	298.2833
46	8	38	45	8	37	E	1	279012.405	-0.013	426.5898
52	2	50	51	2	49	E	1	279024.028	0.004	442.8677
52	3	50	51	3	49	E	1	279024.028	0.004	442.8677
65	26	39	65	25	40	A	0	279039.240	-0.011	630.0542
35	25	10	35	24	11	E	0	279041.854	-0.174	306.4197
46	8	38	45	8	37	E	0	279043.028	-0.010	298.2827
65	26	40	65	25	41	E	0	279043.028	-0.049	630.0426
46	8	38	45	8	37	A	0	279049.365	-0.062	298.2777
35	25	11	35	24	12	A	0	279078.163	-0.022	306.4281
35	25	10	35	24	11	A	0	279078.163	-0.022	306.4281
52	2	50	51	2	49	E	0	279084.878	0.019	314.5726
52	3	50	51	3	49	E	0	279084.878	0.019	314.5726
52	2	50	51	2	49	A	0	279086.847	0.018	314.5641
52	2	50	51	3	49	A	0	279086.847	0.018	314.5641
52	3	50	51	2	49	A	0	279086.847	0.018	314.5641
52	3	50	51	3	49	A	0	279086.847	0.018	314.5641
46	9	38	45	8	37	E	1	279115.792	0.044	426.5898
46	9	38	45	8	37	E	0	279140.050	0.027	298.2827
46	9	38	45	8	37	A	0	279146.893	-0.001	298.2777
34	25	9	34	24	10	E	0	279170.320	-0.045	299.2149
53	1	52	52	1	51	A	1	279172.012	0.008	444.3392
53	1	52	52	2	51	A	1	279172.012	0.008	444.3392
53	2	52	52	1	51	A	1	279172.012	0.008	444.3392
53	2	52	52	2	51	A	1	279172.012	0.008	444.3392
34	25	10	34	24	11	A	0	279206.441	0.002	299.2233
34	25	9	34	24	10	A	0	279206.441	0.002	299.2233
53	2	52	52	2	51	E	1	279211.074	0.019	444.0258
34	25	10	34	24	11	E	0	279215.573	0.112	299.2097
53	1	52	52	1	51	E	0	279276.057	-0.017	315.7390
53	2	52	52	2	51	E	0	279276.057	-0.017	315.7390
53	1	52	52	1	51	A	0	279277.318	0.045	315.7292
53	1	52	52	2	51	A	0	279277.318	0.045	315.7292
53	2	52	52	1	51	A	0	279277.318	0.045	315.7292
53	2	52	52	2	51	A	0	279277.318	0.045	315.7292
33	25	8	33	24	9	E	0	279288.123	-0.062	292.2187
45	23	23	44	23	22	A	0	279310.707	-0.049	371.8475
45	23	22	44	23	21	A	0	279310.707	-0.049	371.8475
45	23	22	44	23	21	E	0	279312.379	-0.185	371.8409
33	25	9	33	24	10	A	0	279324.152	-0.030	292.2271
33	25	8	33	24	9	A	0	279324.152	-0.030	292.2271
33	25	9	33	24	10	E	0	279333.414	0.105	292.2135
54	0	54	53	0	53	A	1	279393.387	0.011	445.1365
54	0	54	53	1	53	A	1	279393.387	0.011	445.1365
54	1	54	53	0	53	A	1	279393.387	0.011	445.1365
54	1	54	53	1	53	A	1	279393.387	0.011	445.1365
32	25	7	32	24	8	E	0	279396.048	-0.060	285.4308
54	0	54	53	0	53	E	1	279403.462	0.036	444.7740
54	1	54	53	1	53	E	1	279403.462	0.036	444.7740
45	10	36	44	10	35	A	0	279417.321	-0.024	293.7660
32	25	8	32	24	9	A	0	279431.964	-0.068	285.4393
32	25	7	32	24	8	A	0	279431.964	-0.068	285.4393
32	25	8	32	24	9	E	0	279441.363	0.108	285.4257
54	0	54	53	0	53	A	0	279473.860	-0.146	316.4865
54	0	54	53	1	53	A	0	279473.860	-0.146	316.4865

J'	K'_a	K'_c	J''	K''_a	K''_c	Sym.	$v_{t_{CH_3}}$	$\nu_{Obs.}$	$\nu_{Obs.} - \nu_{Calc.}$	E_{low}
54	1	54	53	0	53	A	0	279473.860	-0.146	316.4865
54	1	54	53	1	53	A	0	279473.860	-0.146	316.4865
54	0	54	53	0	53	E	0	279473.860	0.171	316.4977
54	1	54	53	1	53	E	0	279473.860	0.171	316.4977
69	8	61	69	7	62	A	0	279476.097	-0.099	559.0776
69	9	61	69	8	62	A	0	279476.097	-0.099	559.0776
31	25	7	31	24	8	A	0	279530.522	-0.065	278.8596
31	25	6	31	24	7	A	0	279530.522	-0.065	278.8596
30	25	6	30	24	7	A	0	279620.373	-0.044	272.4879
30	25	5	30	24	6	A	0	279620.373	-0.044	272.4879
44	14	31	43	14	30	A	0	279653.264	-0.036	301.3856
45	22	23	44	22	22	A	0	279666.294	0.006	363.3872
45	22	24	44	22	23	A	0	279666.294	0.006	363.3872
45	22	24	44	22	23	E	0	279668.122	0.066	363.3740
44	14	31	43	14	30	E	0	279679.724	0.057	301.3852
29	25	5	29	24	6	A	0	279702.006	-0.067	266.3238
29	25	4	29	24	5	A	0	279702.006	-0.067	266.3238
28	25	3	28	24	4	E	0	279740.340	-0.065	260.3588
64	26	38	64	25	39	E	0	279802.338	-0.040	616.4505
64	26	38	64	25	39	A	0	279840.634	-0.001	616.4579
64	26	39	64	25	40	E	0	279845.131	-0.059	616.4461
64	5	59	64	4	60	A	0	279949.576	0.023	468.3913
64	6	59	64	5	60	A	0	279949.576	0.023	468.3913
64	5	59	64	4	60	E	1	279954.973	-0.226	596.6691
64	6	59	64	5	60	E	1	279954.973	-0.226	596.6691
45	21	25	44	21	24	A	0	280076.928	-0.037	355.3268
45	21	24	44	21	23	A	0	280076.928	-0.037	355.3268
45	21	24	44	21	23	E	0	280079.040	-0.048	355.3231
44	14	30	43	14	29	E	0	280126.960	0.008	301.4096
44	14	30	43	14	29	A	0	280141.322	-0.048	301.4066
29	10	20	28	9	19	E	0	280254.740	0.025	167.3004
45	9	36	44	9	35	E	0	280328.825	0.000	293.6910
45	9	36	44	9	35	A	0	280338.420	-0.036	293.6856
29	10	20	28	9	19	A	0	280346.271	-0.022	167.2932
43	10	33	42	10	32	E	0	280530.971	-0.024	278.6970
43	10	33	42	10	32	A	0	280544.793	-0.011	278.6898
45	20	25	44	20	24	A	0	280556.152	-0.062	347.6703
45	20	26	44	20	25	A	0	280556.152	-0.061	347.6703
45	20	25	44	20	24	E	0	280558.589	0.096	347.6682
63	26	37	63	25	38	E	0	280560.674	-0.071	603.0735
63	26	37	63	25	38	A	0	280598.607	-0.013	603.0810
63	26	38	63	25	39	A	0	280598.607	-0.014	603.0810
63	26	38	63	25	39	E	0	280603.755	-0.112	603.0691
61	3	58	61	2	59	A	0	280933.450	0.121	415.3513
61	4	58	61	3	59	A	0	280933.450	0.121	415.3513
44	13	32	43	13	31	A	0	281050.020	-0.023	296.8142
44	13	32	43	13	31	E	0	281057.180	-0.037	296.8169
45	19	27	44	19	26	A	0	281122.300	-0.026	340.4232
44	12	33	43	12	32	A	0	281203.609	-0.011	292.6659
44	12	33	43	12	32	E	0	281205.008	-0.024	292.6701
62	26	36	62	25	37	E	0	281277.912	-0.061	589.9153
62	26	36	62	25	37	A	0	281315.453	-0.031	589.9229
62	26	37	62	25	38	A	0	281315.453	-0.031	589.9229
62	26	37	62	25	38	E	0	281321.281	-0.105	589.9110
36	10	27	35	9	26	E	0	281576.520	-0.080	216.2337
66	6	60	66	5	61	E	1	281616.032	-0.169	630.2262
66	7	60	66	6	61	E	1	281616.032	-0.169	630.2262
66	6	60	66	5	61	E	0	281631.714	-0.166	501.9577
66	7	60	66	6	61	E	0	281631.714	-0.166	501.9577
36	10	27	35	9	26	A	0	281652.457	0.002	216.2251
66	6	60	66	5	61	A	0	281659.160	-0.053	501.9540
66	7	60	66	6	61	A	0	281659.160	-0.053	501.9540
17	16	1	16	15	1	E	0	281700.334	-0.102	138.0647
17	16	2	16	15	2	E	0	281710.642	0.037	138.0511
17	16	1	16	15	2	A	0	281745.713	-0.007	138.0598
17	16	2	16	15	1	A	0	281745.713	-0.007	138.0598
45	18	28	44	18	27	A	0	281801.101	-0.002	333.5928
45	18	27	44	18	26	E	0	281803.694	-0.040	333.5940
45	18	28	44	18	27	E	0	281804.926	-0.019	333.5822
45	10	36	44	9	35	E	0	281811.799	-0.012	293.6910
43	12	31	42	12	30	A	0	281826.410	-0.138	283.9914
45	10	36	44	9	35	A	0	281827.599	0.028	293.6856
43	12	31	42	12	30	E	0	281839.571	-0.014	283.9968
63	4	59	63	3	60	A	1	281891.187	0.238	575.7409
63	5	59	63	4	60	A	1	281891.187	0.238	575.7409
61	26	35	61	25	36	E	0	281956.109	-0.074	576.9755
61	26	35	61	25	36	A	0	281993.308	-0.038	576.9832
61	26	36	61	25	37	A	0	281993.308	-0.039	576.9832
61	26	36	61	25	37	E	0	281999.779	-0.089	576.9712
19	15	4	18	14	4	E	0	282559.090	-0.104	139.7337
19	15	5	18	14	5	E	0	282563.866	0.015	139.7200
60	26	34	60	25	35	E	0	282597.318	-0.040	564.2534
19	15	5	18	14	4	A	0	282602.485	0.010	139.7273
19	15	4	18	14	5	A	0	282602.485	0.010	139.7273
45	17	29	44	17	28	A	0	282630.260	-0.091	327.1893
45	17	28	44	17	27	A	0	282631.411	-0.018	327.1893
45	17	29	44	17	28	E	0	282635.484	-0.035	327.1799
60	26	35	60	25	36	E	0	282641.210	-0.086	564.2492
63	4	59	63	3	60	A	0	283007.809	0.060	447.2606
63	5	59	63	4	60	A	0	283007.809	0.060	447.2606
63	4	59	63	3	60	E	1	283062.273	-0.236	575.5393
63	5	59	63	4	60	E	1	283062.273	-0.236	575.5393
68	7	61	68	6	62	E	1	283091.169	0.110	664.8535
68	8	61	68	7	62	E	1	283091.169	0.110	664.8535
68	7	61	68	6	62	E	0	283157.688	-0.086	536.5916
68	8	61	68	7	62	E	0	283157.688	-0.086	536.5916
68	7	61	68	6	62	A	0	283180.988	-0.140	536.5890
68	8	61	68	7	62	A	0	283180.988	-0.140	536.5890
59	26	33	59	25	34	E	0	283203.299	-0.053	551.7486
59	26	33	59	25	34	A	0	283239.821	-0.042	551.7567
59	26	34	59	25	35	A	0	283239.821	-0.042	551.7567
59	26	34	59	25	35	E	0	283247.439	-0.085	551.7444
21	14	8	20	13	8	E	0	283373.430	-0.078	142.6200
21	14	7	20	13	7	E	0	283374.604	0.070	142.6336
21	14	7	20	13	8	A	0	283415.175	-0.017	142.6258
21	14	8	20	13	7	A	0	283415.175	-0.017	142.6258
46	10	36	45	11	35	A	0	283443.886	-0.041	307.4948
46	10	36	45	11	35	E	0	283461.000	-0.004	307.4996
49	6	43	48	6	42	A	1	283512.652	-0.044	443.3908
49	6	43	48	7	42	A	1	283512.652	0.087	443.3908
49	7	43	48	6	42	A	1	283512.652	-0.112	443.3908
49	7	43	48	7	42	A	1	283512.652	0.019	443.3908
48	7	41	47	8	40	A	1	283555.700	-0.037	439.9827
48	8	41	47	8	40	A	1	283557.921	0.014	439.9827
48	7	41	47	7	40	A	1	283559.761	0.010	439.9826
48	8	41	47	7	40	A	1	283561.916	-0.006	439.9826

J'	K_a'	K_c'	J''	K_a''	K_c''	Sym.	$v_{t_{CH_3}}$	$\nu_{Obs.}$	$\nu_{Obs.} - \nu_{Calc.}$	E_{low}
50	5	45	49	5	44	A	1	283580.028	-0.038	446.3178
50	5	45	49	6	44	A	1	283580.028	-0.035	446.3178
50	6	45	49	5	44	A	1	283580.028	-0.040	446.3178
50	6	45	49	6	44	A	1	283580.028	-0.037	446.3178
46	9	37	45	10	36	E	0	283587.406	-0.029	303.0912
46	9	37	45	10	36	A	0	283589.998	-0.021	303.0863
49	6	43	48	6	42	E	1	283657.287	-0.056	443.2229
49	6	43	48	7	42	E	1	283657.287	0.103	443.2229
49	7	43	48	6	42	E	1	283657.287	-0.140	443.2229
49	7	43	48	7	42	E	1	283657.287	0.020	443.2229
45	16	30	44	16	29	A	0	283665.974	-0.054	321.2271
45	16	29	44	16	28	E	0	283675.267	-0.061	321.2318
50	5	45	49	5	44	E	1	283705.159	-0.009	446.1333
50	5	45	49	6	44	E	1	283705.159	-0.005	446.1333
50	6	45	49	5	44	E	1	283705.159	-0.011	446.1333
50	6	45	49	6	44	E	1	283705.159	-0.007	446.1333
49	6	43	48	6	42	E	0	283706.974	-0.058	314.9139
49	7	43	48	7	42	E	0	283706.974	0.012	314.9139
49	6	43	48	6	42	A	0	283711.461	-0.039	314.9087
49	6	43	48	7	42	A	0	283711.461	0.109	314.9087
49	7	43	48	6	42	A	0	283711.461	-0.117	314.9087
49	7	43	48	7	42	A	0	283711.461	0.031	314.9087
51	4	47	50	4	46	A	1	283713.526	-0.064	448.7906
51	4	47	50	5	46	A	1	283713.526	-0.064	448.7906
51	5	47	50	4	46	A	1	283713.526	-0.064	448.7906
51	5	47	50	5	46	A	1	283713.526	-0.064	448.7906
48	7	41	47	8	40	E	1	283721.932	0.022	439.8254
48	8	41	47	8	40	E	1	283724.476	-0.025	439.8254
48	7	41	47	7	40	E	1	283726.665	-0.009	439.8253
48	8	41	47	7	40	E	1	283729.294	0.028	439.8253
47	8	39	46	9	38	A	1	283744.323	0.122	436.0532
50	5	45	49	5	44	E	0	283758.282	-0.022	317.8258
50	6	45	49	6	44	E	0	283758.282	-0.021	317.8258
50	5	45	49	5	44	A	0	283762.152	-0.010	317.8201
50	5	45	49	6	44	A	0	283762.152	-0.007	317.8201
50	6	45	49	5	44	A	0	283762.152	-0.012	317.8201
50	6	45	49	6	44	A	0	283762.152	-0.009	317.8201
48	7	41	47	8	40	E	0	283766.974	-0.026	311.5168
48	8	41	47	8	40	E	0	283769.383	-0.019	311.5168
48	7	41	47	8	40	A	0	283772.124	-0.013	311.5118
48	8	41	47	7	40	A	0	283779.011	0.004	311.5117
47	9	39	46	9	38	A	1	283794.916	0.049	436.0532
58	26	32	58	25	33	A	0	283812.067	-0.043	539.4688
58	26	33	58	25	34	A	0	283812.067	-0.043	539.4688
51	4	47	50	4	46	E	1	283819.563	-0.027	448.5838
51	4	47	50	5	46	E	1	283819.563	-0.027	448.5838
51	5	47	50	4	46	E	1	283819.563	-0.027	448.5838
51	5	47	50	5	46	E	1	283819.563	-0.027	448.5838
44	13	31	43	13	30	A	0	283828.032	-0.028	296.9637
47	8	39	46	8	38	A	1	283833.491	0.062	436.0502
44	13	31	43	13	30	E	0	283837.093	0.104	296.9673
51	4	47	50	4	46	E	0	283875.660	0.007	320.2795
51	5	47	50	5	46	E	0	283875.660	0.007	320.2795
51	4	47	50	4	46	A	0	283878.903	-0.013	320.2730
51	4	47	50	5	46	A	0	283878.903	-0.013	320.2730
51	5	47	50	4	46	A	0	283878.903	-0.013	320.2730
51	5	47	50	5	46	A	0	283878.903	-0.013	320.2730
30	10	21	29	9	20	E	0	283880.240	0.043	173.5130
47	9	39	46	8	38	A	1	283884.258	0.164	436.0502
52	3	49	51	3	48	A	1	283886.868	-0.095	450.8283
52	3	49	51	4	48	A	1	283886.868	-0.095	450.8283
52	4	49	51	3	48	A	1	283886.868	-0.095	450.8283
52	4	49	51	4	48	A	1	283886.868	-0.095	450.8283
47	8	39	46	9	38	E	1	283928.881	-0.003	435.9001
30	10	21	29	9	20	A	0	283953.944	-0.012	173.5056
47	8	39	46	9	38	E	0	283970.451	-0.031	307.5939
52	3	49	51	3	48	E	1	283972.937	-0.002	450.5932
52	3	49	51	4	48	E	1	283972.937	-0.002	450.5932
52	4	49	51	3	48	E	1	283972.937	-0.002	450.5932
52	4	49	51	4	48	E	1	283972.937	-0.002	450.5932
47	8	39	46	9	38	A	0	283976.159	-0.003	307.5890
47	9	39	46	9	38	E	1	283987.837	-0.080	435.9001
47	9	39	46	9	38	E	0	284025.734	0.005	307.5939
52	3	49	51	3	48	E	0	284031.943	0.003	322.2936
52	4	49	51	4	48	E	0	284031.943	0.003	322.2936
52	3	49	51	3	48	A	0	284034.583	0.001	322.2862
52	3	49	51	4	48	A	0	284034.583	0.001	322.2862
52	4	49	51	3	48	A	0	284034.583	0.001	322.2862
52	4	49	51	4	48	A	0	284034.583	0.001	322.2862
23	13	11	22	12	11	E	0	284068.328	0.054	146.7707
47	8	39	46	8	38	A	0	284073.647	0.018	307.5858
23	13	10	22	12	10	E	0	284075.132	-0.065	146.7841
53	2	51	52	2	50	A	1	284084.636	-0.047	452.4450
53	2	51	52	3	50	A	1	284084.636	-0.047	452.4450
53	3	51	52	2	50	A	1	284084.636	-0.047	452.4450
53	3	51	52	3	50	A	1	284084.636	-0.047	452.4450
47	9	39	46	8	38	E	1	284091.267	0.019	435.8966
23	13	11	22	12	10	A	0	284112.751	0.138	146.7750
23	13	10	22	12	11	A	0	284112.751	0.133	146.7750
47	9	39	46	8	38	E	0	284122.659	-0.055	307.5906
47	9	39	46	8	38	A	0	284129.124	-0.037	307.5858
53	2	51	52	2	50	E	1	284148.636	-0.003	452.1749
53	3	51	52	3	50	E	1	284148.636	-0.003	452.1749
29	10	19	28	9	20	A	0	284206.815	-0.014	167.1879
53	2	51	52	2	50	E	0	284210.991	0.007	323.8818
53	3	51	52	3	50	E	0	284210.991	0.007	323.8818
53	2	51	52	2	50	A	0	284212.947	0.001	323.8734
53	2	51	52	3	50	A	0	284212.947	0.001	323.8734
53	3	51	52	2	50	A	0	284212.947	0.001	323.8734
53	3	51	52	3	50	A	0	284212.947	0.001	323.8734
29	10	19	28	9	20	E	0	284219.777	-0.056	167.1944
27	11	17	26	10	16	A	0	284265.561	-0.029	158.9871
27	11	17	26	10	17	E	0	284272.524	-0.004	158.9848
27	11	16	26	10	16	E	0	284282.680	-0.053	158.9971
54	1	53	53	1	52	A	1	284297.288	0.007	453.6513
54	1	53	53	2	52	A	1	284297.288	0.007	453.6513
54	2	53	53	1	52	A	1	284297.288	0.007	453.6513
54	2	53	53	2	52	A	1	284297.288	0.007	453.6513
57	26	31	57	25	32	E	0	284316.550	-0.100	527.3889
54	2	53	53	2	52	E	1	284336.260	0.025	453.3393
45	11	35	44	11	34	E	0	284346.720	0.066	298.0148
45	11	35	44	11	34	A	0	284351.098	-0.006	298.0099
57	26	31	57	25	32	A	0	284352.550	-0.017	527.3972
57	26	32	57	25	33	A	0	284352.550	-0.017	527.3972
57	26	32	57	25	33	E	0	284361.238	-0.009	527.3848
27	11	16	26	10	17	A	0	284368.178	-0.002	158.9842

J'	K'_a	K'_c	J''	K''_a	K''_c	Sym.	$v_{t_{CH_3}}$	$\nu_{Obs.}$	$\nu_{Obs.} - \nu_{Calc.}$	E_{low}
70	8	62	70	7	63	E	1	284372.043	0.223	700.5458
70	9	62	70	8	63	E	1	284372.043	0.223	700.5458
54	1	53	53	1	52	E	0	284402.647	-0.025	325.0546
54	2	53	53	2	52	E	0	284402.647	-0.025	325.0546
54	1	53	53	1	52	A	0	284403.899	0.031	325.0449
54	1	53	53	2	52	A	0	284403.899	0.031	325.0449
54	2	53	53	1	52	A	0	284403.899	0.031	325.0449
54	2	53	53	2	52	A	0	284403.899	0.031	325.0449
46	10	37	45	10	36	E	1	284457.001	-0.053	431.3927
46	10	37	45	10	36	E	0	284489.296	-0.021	303.0912
25	12	14	24	11	14	E	0	284491.789	0.012	152.2054
46	10	37	45	10	36	A	0	284495.822	0.010	303.0863
35	10	26	34	9	25	E	0	284499.780	-0.073	208.4882
25	12	13	24	11	13	E	0	284504.981	-0.055	152.2183
70	8	62	70	7	63	A	0	284507.388	0.175	572.2910
70	9	62	70	8	63	A	0	284507.388	0.175	572.2910
55	0	55	54	0	54	A	1	284519.042	0.136	454.4560
55	0	55	54	1	54	A	1	284519.042	0.136	454.4560
55	1	55	54	0	54	A	1	284519.042	0.136	454.4560
55	1	55	54	1	54	A	1	284519.042	0.136	454.4560
55	0	55	54	0	54	E	1	284528.887	0.018	454.0939
55	1	55	54	1	54	E	1	284528.887	0.018	454.0939
25	12	14	24	11	13	A	0	284537.940	-0.057	152.2080
25	12	13	24	11	14	A	0	284539.085	-0.047	152.2080
35	10	26	34	9	25	A	0	284575.369	0.002	208.4797
55	0	55	54	0	54	A	0	284600.643	-0.160	325.8087
55	0	55	54	1	54	A	0	284600.643	-0.160	325.8087
55	1	55	54	0	54	A	0	284600.643	-0.160	325.8087
55	1	55	54	1	54	A	0	284600.643	-0.160	325.8087
55	0	55	54	0	54	E	0	284600.643	0.155	325.8200
55	1	55	54	1	54	E	0	284600.643	0.155	325.8200
44	10	34	43	10	33	E	0	284721.496	-0.023	288.0545
44	10	34	43	10	33	A	0	284736.716	-0.027	288.0478
56	26	30	56	25	31	E	0	284827.111	-0.023	515.5330
56	26	30	56	25	31	A	0	284862.710	-0.065	515.5414
56	26	31	56	25	32	A	0	284862.710	-0.065	515.5414
56	26	31	56	25	32	E	0	284871.858	-0.064	515.5289
65	5	60	65	4	61	A	0	284911.556	0.204	480.2527
65	6	60	65	5	61	A	0	284911.556	0.204	480.2527
45	15	31	44	15	30	A	0	284974.981	-0.038	315.7270
45	15	31	44	15	30	E	0	285013.235	-0.014	315.7223
45	15	30	44	15	29	E	0	285060.063	-0.030	315.7355
46	9	37	45	9	36	E	1	285066.303	0.035	431.3406
46	9	37	45	9	36	E	0	285070.319	-0.102	303.0417
46	9	37	45	9	36	A	0	285079.162	0.027	303.0366
45	15	30	44	15	29	A	0	285087.849	-0.079	315.7313
55	26	29	55	25	30	E	0	285308.756	-0.057	503.8925
55	26	29	55	25	30	A	0	285344.162	-0.028	503.9010
55	26	30	55	25	31	A	0	285344.162	-0.028	503.9010
46	24	22	45	24	21	A	0	285352.526	-0.067	390.0109
46	24	23	45	24	22	A	0	285352.526	-0.067	390.0109
43	11	32	42	11	31	A	0	285681.969	-0.112	281.1995
43	11	32	42	11	31	E	0	285683.219	-0.012	281.2068
46	23	24	45	23	23	A	0	285685.165	-0.029	381.1643
46	23	23	45	23	22	A	0	285685.165	-0.029	381.1643
54	26	28	54	25	29	E	0	285763.010	-0.053	492.4669
54	26	28	54	25	29	A	0	285798.191	0.003	492.4755
54	26	29	54	25	30	A	0	285798.191	0.003	492.4755
54	26	29	54	25	30	E	0	285808.111	-0.079	492.4629
62	4	59	62	3	60	E	0	285830.102	0.126	426.3781
46	10	37	45	9	36	E	0	285972.291	-0.012	303.0417
62	3	59	62	2	60	E	1	285978.103	-0.236	554.6481
62	4	59	62	3	60	E	1	285978.103	-0.236	554.6481
46	10	37	45	9	36	A	0	285984.923	-0.004	303.0366
46	22	24	45	22	23	A	0	286066.377	-0.034	372.7159
46	22	25	45	22	24	A	0	286066.377	-0.034	372.7159
46	22	25	45	22	24	E	0	286068.363	0.039	372.7028
53	26	27	53	25	28	E	0	286191.134	-0.057	481.2559
53	26	27	53	25	28	A	0	286226.049	-0.027	481.2645
53	26	28	53	25	29	A	0	286226.049	-0.027	481.2645
53	26	28	53	25	29	E	0	286236.451	-0.018	481.2519
31	10	22	30	9	21	E	0	286496.706	-0.028	179.9826
46	21	26	45	21	25	A	0	286507.289	-0.029	364.6691
46	21	25	45	21	24	A	0	286507.289	-0.029	364.6691
46	21	25	45	21	24	E	0	286509.575	-0.014	364.6655
45	14	32	44	14	31	A	0	286523.296	-0.043	310.7139
45	14	32	44	14	31	E	0	286541.069	-0.005	310.7143
31	10	22	30	9	21	A	0	286563.906	-0.009	179.9750
67	6	61	67	5	62	E	1	286592.260	-0.173	642.5917
67	7	61	67	6	62	E	1	286592.260	-0.173	642.5917
52	26	26	52	25	27	E	0	286594.335	-0.099	470.2589
52	26	26	52	25	27	A	0	286629.082	-0.011	470.2677
52	26	27	52	25	28	A	0	286629.082	-0.011	470.2677
67	6	61	67	5	62	A	0	286638.089	-0.057	514.3225
67	7	61	67	6	62	A	0	286638.089	-0.057	514.3225
52	26	27	52	25	28	E	0	286639.829	-0.023	470.2549
34	10	25	33	9	24	E	0	286745.498	-0.085	200.9766
34	10	25	33	9	24	A	0	286818.774	0.034	200.9683
64	4	60	64	3	61	A	1	286820.995	0.168	587.2610
64	5	60	64	4	61	A	1	286820.995	0.168	587.2610
51	26	25	51	25	26	E	0	286973.911	-0.062	459.4757
51	26	25	51	25	26	A	0	287008.383	-0.032	459.4845
51	26	26	51	25	27	A	0	287008.383	-0.032	459.4845
51	26	26	51	25	27	E	0	287019.526	0.006	459.4717
46	20	26	45	20	25	A	0	287022.671	-0.009	357.0286
46	20	27	45	20	26	A	0	287022.671	-0.009	357.0286
46	20	26	45	20	25	E	0	287025.295	0.177	357.0266
50	26	24	50	25	25	E	0	287330.856	-0.073	448.9057
45	14	31	44	14	30	E	0	287337.412	0.067	310.7536
45	14	31	44	14	30	A	0	287341.758	-0.015	310.7511
50	26	24	50	25	25	A	0	287365.125	-0.040	448.9147
50	26	25	50	25	26	A	0	287365.125	-0.040	448.9147
45	12	34	44	12	33	A	0	287369.192	0.033	302.0459
45	12	34	44	12	33	E	0	287369.192	-0.098	302.0501
50	26	25	50	25	26	E	0	287376.555	-0.041	448.9018
46	19	28	45	19	27	A	0	287632.725	-0.039	349.8004
46	19	27	45	19	26	E	0	287635.216	-0.159	349.8000
49	26	23	49	25	24	E	0	287666.337	-0.033	438.5488
49	26	23	49	25	24	A	0	287700.375	-0.036	438.5578
49	26	24	49	25	25	A	0	287700.375	-0.036	438.5578
49	26	24	49	25	25	E	0	287712.121	-0.025	438.5449
45	13	33	44	13	32	A	0	287768.185	-0.015	306.1890
45	13	33	44	13	32	E	0	287773.695	-0.035	306.1919
70	27	43	70	26	44	E	0	287775.702	-0.117	710.4920
70	27	44	70	26	45	A	0	287812.153	0.016	710.4996
18	16	2	17	15	2	E	0	287846.382	-0.062	141.5554

J'	K_a'	K_c'	J''	K_a''	K_c''	Sym.	$v_{t_{CH_3}}$	$\nu_{Obs.}$	$\nu_{Obs.} - \nu_{Calc.}$	E_{low}
18	16	3	17	15	3	E	0	287856.609	0.024	141.5418
32	10	23	31	9	22	E	0	287890.260	0.035	186.7160
18	16	2	17	15	3	A	0	287891.699	-0.018	141.5505
18	16	3	17	15	2	A	0	287891.699	-0.018	141.5505
33	10	24	32	9	23	E	0	287953.136	-0.017	193.7155
64	4	60	64	3	61	A	0	287955.010	0.042	458.7861
64	5	60	64	4	61	A	0	287955.010	0.042	458.7861
32	10	23	31	9	22	A	0	287957.223	0.013	186.7082
48	26	22	48	25	23	E	0	287981.266	-0.053	428.4044
64	4	60	64	3	61	E	1	288008.362	-0.258	587.0621
64	5	60	64	4	61	E	1	288008.362	-0.258	587.0621
48	26	22	48	25	23	A	0	288015.141	-0.033	428.4135
48	26	23	48	25	24	A	0	288015.141	-0.033	428.4135
33	10	24	32	9	23	A	0	288022.928	0.001	193.7074
48	26	23	48	25	24	E	0	288027.205	0.009	428.4006
69	7	62	69	6	63	E	1	288087.961	0.016	677.7262
69	8	62	69	7	63	E	1	288087.961	0.016	677.7262
69	7	62	69	6	63	A	0	288181.253	-0.105	549.4650
69	8	62	69	7	63	A	0	288181.253	-0.105	549.4650
47	26	21	47	25	22	E	0	288276.716	-0.034	418.4723
47	26	21	47	25	22	A	0	288310.402	-0.028	418.4814
47	26	22	47	25	23	A	0	288310.402	-0.028	418.4814
47	26	22	47	25	23	E	0	288322.704	-0.015	418.4685
55	14	41	54	15	40	E	0	288348.061	-0.006	421.0877
46	18	29	45	18	28	A	0	288366.447	0.030	342.9927
46	18	28	45	18	27	E	0	288369.278	0.002	342.9940
46	18	29	45	18	28	E	0	288370.574	-0.038	342.9822
46	26	20	46	25	21	E	0	288553.567	-0.032	408.7521
46	26	20	46	25	21	A	0	288587.084	-0.029	408.7613
46	26	21	46	25	22	A	0	288587.084	-0.029	408.7613
46	26	21	46	25	22	E	0	288599.649	-0.002	408.7483
50	6	44	49	6	43	A	1	288621.161	-0.030	452.8477
50	6	44	49	7	43	A	1	288621.161	0.038	452.8477
50	7	44	49	6	43	A	1	288621.161	-0.065	452.8477
50	7	44	49	7	43	A	1	288621.161	0.003	452.8477
49	7	42	48	8	41	A	1	288647.705	0.080	449.4412
49	8	42	48	8	41	A	1	288648.833	0.042	449.4412
49	7	42	48	7	41	A	1	288649.794	-0.001	449.4411
49	8	42	48	7	41	A	1	288651.047	0.087	449.4411
69	27	42	69	26	43	E	0	288690.125	-0.097	695.8237
51	5	46	50	5	45	A	1	288697.338	-0.022	455.7770
51	5	46	50	6	45	A	1	288697.338	-0.020	455.7770
51	6	46	50	5	45	A	1	288697.338	-0.023	455.7770
51	6	46	50	6	45	A	1	288697.338	-0.021	455.7770
20	15	6	19	14	6	E	0	288701.268	0.037	143.6288
61	2	59	61	1	60	E	1	288719.167	-0.112	534.0011
61	3	59	61	2	60	E	1	288719.167	-0.112	534.0011
69	27	43	69	26	44	A	0	288726.054	-0.002	695.8315
69	27	43	69	26	44	E	0	288733.314	-0.263	695.8205
20	15	6	19	14	5	A	0	288739.869	-0.018	143.6361
20	15	5	19	14	6	A	0	288739.869	-0.018	143.6361
50	6	44	49	6	43	E	1	288764.072	-0.032	452.6847
50	6	44	49	7	43	E	1	288764.072	0.052	452.6847
50	7	44	49	6	43	E	1	288764.072	-0.075	452.6847
50	7	44	49	7	43	E	1	288764.072	0.008	452.6847
45	10	35	44	10	34	E	0	288787.127	0.021	297.5518
45	10	35	44	10	34	A	0	288802.545	0.210	297.5455
49	7	42	48	8	41	E	1	288811.326	0.025	449.2895
49	7	42	48	7	41	E	1	288813.991	0.099	449.2894
50	6	44	49	6	43	E	0	288816.024	-0.011	324.3774
50	7	44	49	7	43	E	0	288816.024	0.026	324.3774
50	6	44	49	6	43	A	0	288820.357	-0.093	324.3723
50	6	44	49	7	43	A	0	288820.357	-0.016	324.3723
50	7	44	49	6	43	A	0	288820.357	-0.133	324.3723
50	7	44	49	7	43	A	0	288820.357	-0.056	324.3723
51	5	46	50	5	45	E	1	288821.495	0.143	455.5967
51	5	46	50	6	45	E	1	288821.495	0.145	455.5967
51	6	46	50	5	45	E	1	288821.495	0.142	455.5967
51	6	46	50	6	45	E	1	288821.495	0.144	455.5967
48	8	40	47	9	39	A	1	288825.483	0.053	445.5196
52	4	48	51	4	47	A	1	288835.119	-0.097	458.2543
52	4	48	51	5	47	A	1	288835.119	-0.097	458.2543
52	5	48	51	4	47	A	1	288835.119	-0.097	458.2543
52	5	48	51	5	47	A	1	288835.119	-0.097	458.2543
45	26	19	45	25	20	A	0	288846.063	-0.052	399.2527
45	26	20	45	25	21	A	0	288846.063	-0.052	399.2527
48	9	40	47	9	39	A	1	288854.013	0.066	445.5196
45	26	20	45	25	21	E	0	288858.927	0.040	399.2397
49	7	42	48	8	41	A	0	288864.000	-0.020	320.9775
49	8	42	48	8	41	A	0	288865.284	-0.038	320.9775
49	7	42	48	7	41	A	0	288866.459	0.023	320.9775
49	8	42	48	7	41	A	0	288867.734	-0.005	320.9775
51	5	46	50	5	45	E	0	288876.442	-0.017	327.2910
51	6	46	50	6	45	E	0	288876.442	-0.016	327.2910
51	5	46	50	5	45	A	0	288880.280	-0.004	327.2853
51	5	46	50	6	45	A	0	288880.280	-0.002	327.2853
51	6	46	50	5	45	A	0	288880.280	-0.005	327.2853
51	6	46	50	6	45	A	0	288880.280	-0.003	327.2853
48	9	40	47	8	39	A	1	288904.712	0.099	445.5179
52	4	48	51	4	47	E	1	288940.503	-0.023	458.0510
52	4	48	51	5	47	E	1	288940.503	-0.023	458.0510
52	5	48	51	4	47	E	1	288940.503	-0.023	458.0510
52	5	48	51	5	47	E	1	288940.503	-0.023	458.0510
52	4	48	51	4	47	E	0	288998.361	-0.006	329.7485
52	5	48	51	5	47	E	0	288998.361	-0.006	329.7485
52	4	48	51	4	47	A	0	289001.650	0.041	329.7422
52	4	48	51	5	47	A	0	289001.650	0.041	329.7422
52	5	48	51	4	47	A	0	289001.650	0.040	329.7422
52	5	48	51	5	47	A	0	289001.650	0.041	329.7422
47	9	38	46	10	37	E	0	289001.650	-0.381	312.5807
47	9	38	46	10	37	A	0	289006.118	-0.006	312.5761
53	3	50	52	3	49	A	1	289010.607	-0.070	460.2978
53	3	50	52	4	49	A	1	289010.607	-0.070	460.2978
53	4	50	52	3	49	A	1	289010.607	-0.070	460.2978
53	4	50	52	4	49	A	1	289010.607	-0.070	460.2978
48	9	40	47	9	39	E	1	289043.309	0.007	445.3729
48	8	40	47	9	39	E	0	289053.012	-0.007	317.0679
44	26	18	44	25	19	E	0	289055.133	0.045	389.9461
48	8	40	47	9	39	A	0	289058.681	-0.027	317.0633
48	8	40	47	8	39	E	1	289068.878	-0.027	445.3709
48	9	40	47	9	39	E	0	289084.208	-0.007	317.0679
44	26	18	44	25	19	A	0	289088.253	-0.043	389.9554
44	26	19	44	25	20	A	0	289088.253	-0.043	389.9554
48	9	40	47	9	39	A	0	289090.091	0.020	317.0633
53	3	50	52	3	49	E	1	289096.261	0.004	460.0655
53	3	50	52	4	49	E	1	289096.261	0.004	460.0655

J'	K'_a	K'_c	J''	K''_a	K''_c	Sym.	$v_{t_{CH_3}}$	$\nu_{Obs.}$	$\nu_{Obs.} - \nu_{Calc.}$	E_{low}
53	4	50	52	3	49	E	1	289096.261	0.004	460.0655
53	4	50	52	4	49	E	1	289096.261	0.004	460.0655
44	26	19	44	25	20	E	0	289101.215	-0.070	389.9423
48	9	40	47	8	39	E	1	289102.384	0.049	445.3709
48	8	40	47	8	39	E	0	289108.275	0.009	317.0661
48	8	40	47	8	39	A	0	289114.166	-0.074	317.0615
48	9	40	47	8	39	E	0	289139.440	-0.022	317.0661
48	9	40	47	8	39	A	0	289145.572	-0.032	317.0615
53	3	50	52	3	49	E	0	289156.886	-0.001	331.7678
53	4	50	52	4	49	E	0	289156.886	-0.001	331.7678
53	3	50	52	3	49	A	0	289159.521	0.004	331.7606
53	3	50	52	4	49	A	0	289159.521	0.004	331.7606
53	4	50	52	3	49	A	0	289159.521	0.004	331.7606
53	4	50	52	4	49	A	0	289159.521	0.004	331.7606
54	2	52	53	2	51	A	1	289209.306	-0.054	461.9210
54	2	52	53	3	51	A	1	289209.306	-0.054	461.9210
54	3	52	53	2	51	A	1	289209.306	-0.054	461.9210
54	3	52	53	3	51	A	1	289209.306	-0.054	461.9210
46	17	30	45	17	29	A	0	289266.227	0.019	336.6168
46	17	29	45	17	28	A	0	289268.359	-0.041	336.6169
46	17	29	45	17	28	E	0	289270.208	-0.046	336.6198
54	2	52	53	2	51	E	1	289273.140	0.022	461.6531
54	3	52	53	3	51	E	1	289273.140	0.022	461.6531
47	10	38	46	10	37	A	1	289295.390	0.237	441.0296
43	26	17	43	25	18	A	0	289314.433	-0.044	380.8690
43	26	18	43	25	19	A	0	289314.433	-0.044	380.8690
43	26	18	43	25	19	E	0	289327.655	-0.014	380.8559
54	2	52	53	2	51	E	0	289336.981	0.008	333.3621
54	3	52	53	3	51	E	0	289336.981	0.008	333.3621
54	2	52	53	2	51	A	0	289338.931	0.002	333.3537
54	2	52	53	3	51	A	0	289338.931	0.002	333.3537
54	3	52	53	2	51	A	0	289338.931	0.002	333.3537
54	3	52	53	3	51	A	0	289338.931	0.002	333.3537
55	1	54	54	1	53	A	1	289422.422	0.024	463.1345
55	1	54	54	2	53	A	1	289422.422	0.024	463.1345
55	2	54	54	1	53	A	1	289422.422	0.024	463.1345
55	2	54	54	2	53	A	1	289422.422	0.024	463.1345
55	2	54	54	2	53	E	1	289461.261	0.004	462.8237
22	14	9	21	13	9	E	0	289487.881	-0.054	146.9516
22	14	8	21	13	8	E	0	289489.098	0.041	146.9653
42	26	16	42	25	17	E	0	289492.496	-0.018	371.9838
47	10	38	46	10	37	E	1	289508.958	-0.006	440.8812
42	26	16	42	25	17	A	0	289525.429	-0.019	371.9932
42	26	17	42	25	18	A	0	289525.429	-0.019	371.9932
55	1	54	54	1	53	E	0	289529.109	-0.005	334.5413
55	2	54	54	2	53	E	0	289529.109	-0.005	334.5413
55	1	54	54	1	53	A	0	289530.334	0.027	334.5316
55	1	54	54	2	53	A	0	289530.334	0.027	334.5316
55	2	54	54	1	53	A	0	289530.334	0.027	334.5316
55	2	54	54	2	53	A	0	289530.334	0.027	334.5316
42	26	17	42	25	18	E	0	289538.909	0.079	371.9801
47	10	38	46	10	37	E	0	289543.026	-0.021	312.5807
47	10	38	46	10	37	A	0	289549.575	-0.017	312.5761
68	27	42	68	26	43	A	0	289592.304	-0.052	681.3843
68	27	41	68	26	42	A	0	289592.304	-0.048	681.3843
47	9	38	46	9	37	A	1	289632.679	0.140	441.0016
56	0	56	55	0	55	A	1	289644.465	0.190	463.9466
56	0	56	55	1	55	A	1	289644.465	0.190	463.9466
56	1	56	55	0	55	A	1	289644.465	0.190	463.9466
56	1	56	55	1	55	A	1	289644.465	0.190	463.9466
56	0	56	55	0	55	E	1	289654.158	0.006	463.5848
56	1	56	55	1	55	E	1	289654.158	0.006	463.5848
41	26	15	41	25	16	E	0	289689.161	-0.001	363.3184
46	11	36	45	11	35	E	0	289698.223	-0.035	307.4996
46	11	36	45	11	35	A	0	289703.545	0.033	307.4948
41	26	15	41	25	16	A	0	289721.901	-0.070	363.3278
41	26	16	41	25	17	A	0	289721.901	-0.070	363.3278
56	0	56	55	0	55	A	0	289727.280	-0.160	335.3020
56	0	56	55	1	55	A	0	289727.280	-0.160	335.3020
56	1	56	55	0	55	A	0	289727.280	-0.160	335.3020
56	1	56	55	1	55	A	0	289727.280	-0.160	335.3020
41	26	16	41	25	17	E	0	289735.560	0.034	363.3147
47	9	38	46	9	37	E	1	289888.782	0.122	440.8494
47	9	38	46	9	37	E	0	289903.666	-0.247	312.5506
40	26	14	40	25	15	A	0	289904.747	-0.027	354.8724
40	26	15	40	25	16	A	0	289904.747	-0.027	354.8724
47	9	38	46	9	37	A	0	289911.895	-0.021	312.5459
40	26	15	40	25	16	E	0	289918.533	0.041	354.8593
28	11	18	27	10	17	A	0	289920.168	-0.029	164.6549
28	11	17	27	10	17	E	0	289976.770	-0.082	164.6639
28	11	18	27	10	18	E	0	289998.983	0.131	164.6506
39	26	13	39	25	14	E	0	290041.955	-0.025	346.6174
39	26	13	39	25	14	A	0	290074.521	-0.040	346.6269
39	26	14	39	25	15	A	0	290074.521	-0.040	346.6269
44	12	32	43	12	31	A	0	290085.631	-0.029	293.3922
39	26	14	39	25	15	E	0	290088.457	0.028	346.6137
44	12	32	43	12	31	E	0	290097.823	-0.029	293.3980
24	13	12	23	12	12	E	0	290131.877	0.028	151.5325
28	11	17	27	10	18	A	0	290134.528	0.025	164.6488
24	13	11	23	12	11	E	0	290138.926	-0.033	151.5459
24	13	12	23	12	11	A	0	290176.307	-0.002	151.5368
38	26	12	38	25	13	E	0	290199.410	-0.122	338.5813
38	26	12	38	25	13	A	0	290231.943	-0.065	338.5908
38	26	13	38	25	14	A	0	290231.943	-0.065	338.5908
48	11	37	47	12	36	A	0	290337.841	-0.065	331.3953
37	26	11	37	25	12	E	0	290345.331	-0.060	330.7545
37	26	11	37	25	12	A	0	290377.681	-0.088	330.7640
37	26	12	37	25	13	A	0	290377.681	-0.088	330.7640
48	11	37	47	12	36	E	0	290379.550	0.028	331.3994
37	26	12	37	25	13	E	0	290391.930	0.028	330.7508
46	16	31	45	16	30	A	0	290393.831	-0.032	330.6892
46	16	30	45	16	29	E	0	290409.591	-0.160	330.6942
46	16	31	45	16	30	E	0	290410.607	0.021	330.6817
67	27	40	67	26	41	A	0	290413.545	-0.009	667.1574
46	16	30	45	16	29	A	0	290417.106	-0.014	330.6900
67	27	41	67	26	42	E	0	290422.472	-0.133	667.1461
47	10	38	46	9	37	E	0	290444.906	-0.024	312.5506
26	12	15	25	11	15	E	0	290447.217	-0.024	157.4091
47	10	38	46	9	37	A	0	290455.386	0.003	312.5459
26	12	14	25	11	14	E	0	290460.655	-0.219	157.4219
36	26	10	36	25	11	E	0	290480.087	-0.096	323.1366
26	12	15	25	11	14	A	0	290492.742	-0.129	157.4116
26	12	14	25	11	15	A	0	290495.756	0.026	157.4115
36	26	10	36	25	11	A	0	290512.408	-0.061	323.1462
36	26	11	36	25	12	A	0	290512.408	-0.061	323.1462
36	26	11	36	25	12	E	0	290526.760	0.040	323.1330

J'	K'_a	K'_c	J''	K''_a	K''_c	Sym.	$v_{t_{CH_3}}$	$\nu_{Obs.}$	$\nu_{Obs.} - \nu_{Calc.}$	E_{low}
35	26	9	35	25	10	E	0	290604.490	-0.026	315.7276
30	10	20	29	9	21	E	0	290624.079	-0.026	173.3331
30	10	20	29	9	21	A	0	290630.444	-0.008	173.3265
35	26	9	35	25	10	A	0	290636.661	-0.055	315.7371
35	26	10	35	25	11	A	0	290636.661	-0.055	315.7371
35	26	10	35	25	11	E	0	290651.097	0.022	315.7239
34	26	8	34	25	9	E	0	290718.963	-0.010	308.5270
34	26	8	34	25	9	A	0	290751.034	-0.059	308.5366
34	26	9	34	25	10	A	0	290751.034	-0.059	308.5366
33	26	7	33	25	8	E	0	290824.124	0.008	301.5347
33	26	7	33	25	8	A	0	290856.115	-0.046	301.5443
33	26	8	33	25	9	A	0	290856.115	-0.046	301.5443
47	27	21	46	27	20	A	0	290879.834	0.040	428.4059
47	27	20	46	27	19	A	0	290879.834	0.040	428.4059
47	10	37	46	11	36	A	0	290907.041	-0.027	317.1583
63	3	60	63	2	61	E	1	290915.096	-0.177	565.8354
63	4	60	63	3	61	E	1	290915.096	-0.177	565.8354
47	10	37	46	11	36	E	0	290916.631	0.072	317.1629
32	26	6	32	25	7	E	0	290920.493	0.004	294.7505
32	26	6	32	25	7	A	0	290952.401	-0.062	294.7601
32	26	7	32	25	8	A	0	290952.401	-0.062	294.7601
32	26	7	32	25	8	E	0	290967.129	0.038	294.7469
31	26	5	31	25	6	A	0	291040.435	-0.087	288.1838
31	26	6	31	25	7	A	0	291040.435	-0.087	288.1838
31	26	6	31	25	7	E	0	291055.309	0.086	288.1705
30	26	4	30	25	5	A	0	291120.807	-0.033	281.8150
30	26	5	30	25	6	A	0	291120.807	-0.033	281.8150
47	26	21	46	26	20	A	0	291126.110	-0.081	418.3875
47	26	22	46	26	21	A	0	291126.110	-0.081	418.3875
47	26	21	46	26	20	E	0	291127.725	-0.033	418.3772
30	26	5	30	25	6	E	0	291135.653	0.045	281.8018
66	27	39	66	26	40	E	0	291157.538	0.057	653.1418
66	27	40	66	26	41	A	0	291192.002	0.009	653.1501
66	27	39	66	26	40	A	0	291192.002	0.010	653.1501
66	27	40	66	26	41	E	0	291201.611	-0.135	653.1388
47	24	23	46	24	22	A	0	291715.504	-0.051	399.5292
47	24	24	46	24	23	A	0	291715.504	-0.051	399.5292
46	15	32	45	15	31	A	0	291809.279	-0.068	325.2327
46	15	32	45	15	31	E	0	291848.908	-0.034	325.2294
45	13	32	44	13	31	A	0	291876.081	-0.038	306.4312
45	13	32	44	13	31	E	0	291888.126	-0.037	306.4351
65	27	38	65	26	39	E	0	291895.544	-0.190	639.3535
44	11	33	43	11	32	A	0	291934.206	-0.040	290.7288
65	27	39	65	26	40	E	0	291940.213	-0.050	639.3505
46	15	31	45	15	30	E	0	291981.952	0.005	325.2441
46	15	31	45	15	30	A	0	292010.126	-0.034	325.2408
47	23	25	46	23	24	A	0	292071.372	-0.076	390.6938
47	23	24	46	23	23	A	0	292071.372	-0.076	390.6938
47	22	25	46	22	24	A	0	292479.766	-0.042	382.2581
47	22	26	46	22	25	A	0	292479.766	-0.042	382.2581
47	22	26	46	22	25	E	0	292481.914	0.042	382.2450
64	27	37	64	26	38	E	0	292595.408	-0.005	625.7837
64	27	38	64	26	39	A	0	292629.129	-0.003	625.7923
64	27	37	64	26	38	A	0	292629.129	-0.003	625.7923
64	27	38	64	26	39	E	0	292639.981	-0.206	625.7808
46	10	36	45	10	35	E	0	292899.095	-0.012	307.1847
65	4	61	65	3	62	A	0	292901.037	0.072	470.4825
65	5	61	65	4	62	A	0	292901.037	0.072	470.4825
46	10	36	45	10	35	A	0	292913.351	-0.008	307.1790
47	21	27	46	21	26	A	0	292952.839	0.089	374.2260
47	21	26	46	21	25	A	0	292952.839	0.089	374.2260
47	21	26	46	21	25	E	0	292955.207	0.029	374.2224
70	7	63	70	6	64	E	1	293081.178	0.039	690.7696
70	8	63	70	7	64	E	1	293081.178	0.039	690.7696
17	17	0	16	16	0	E	0	293125.738	-0.061	143.9725
17	17	1	16	16	1	E	0	293141.300	0.041	143.9592
17	17	1	16	16	0	A	0	293172.412	-0.031	143.9691
17	17	0	16	16	1	A	0	293172.412	-0.031	143.9691
70	7	63	70	6	64	A	0	293178.084	-0.000	562.5116
70	8	63	70	7	64	A	0	293178.084	-0.000	562.5116
63	27	37	63	26	38	A	0	293291.786	0.022	612.4407
63	27	36	63	26	37	A	0	293291.786	0.022	612.4407
46	12	35	45	12	34	E	0	293350.929	-0.065	311.6357
46	12	35	45	12	34	A	0	293352.165	0.013	311.6315
46	14	33	45	14	32	A	0	293396.164	-0.031	320.2713
46	14	33	45	14	32	E	0	293408.516	-0.029	320.2723
62	2	60	62	1	61	A	0	293487.008	0.205	416.5807
62	3	60	62	2	61	A	0	293487.008	0.205	416.5807
47	20	27	46	20	26	A	0	293506.492	-0.040	366.6026
47	20	28	46	20	27	A	0	293506.492	-0.038	366.6026
47	20	27	46	20	26	E	0	293509.297	0.161	366.6007
51	6	45	50	6	44	A	1	293730.782	-0.107	462.4751
51	6	45	50	7	44	A	1	293730.782	-0.071	462.4751
51	7	45	50	6	44	A	1	293730.782	-0.125	462.4751
51	7	45	50	7	44	A	1	293730.782	-0.090	462.4751
50	7	43	49	8	42	A	1	293741.969	0.006	459.0695
50	8	43	49	7	42	A	1	293743.772	0.020	459.0694
52	5	47	51	5	46	A	1	293814.991	-0.065	465.4069
52	5	47	51	6	46	A	1	293814.991	-0.064	465.4069
52	6	47	51	5	46	A	1	293814.991	-0.065	465.4069
52	6	47	51	6	46	A	1	293814.991	-0.064	465.4069
51	6	45	50	6	44	E	1	293872.098	-0.027	462.3168
51	6	45	50	7	44	E	1	293872.098	0.016	462.3168
51	7	45	50	6	44	E	1	293872.098	-0.050	462.3168
51	7	45	50	7	44	E	1	293872.098	-0.006	462.3168
62	27	35	62	26	36	E	0	293886.534	0.012	599.2977
49	8	41	48	9	40	A	1	293901.968	0.027	455.1547
50	7	43	49	8	42	E	1	293903.197	0.027	458.9232
50	8	43	49	7	42	E	1	293905.348	0.025	458.9232
62	27	36	62	26	37	A	0	293919.496	-0.020	599.3066
62	27	35	62	26	36	A	0	293919.496	-0.020	599.3066
51	6	45	50	6	44	E	0	293926.269	-0.013	334.0113
51	7	45	50	7	44	E	0	293926.269	0.006	334.0113
51	6	45	50	6	44	A	0	293930.628	-0.019	334.0063
51	6	45	50	7	44	A	0	293930.628	0.021	334.0063
51	7	45	50	6	44	A	0	293930.628	-0.039	334.0063
51	7	45	50	7	44	A	0	293930.628	0.001	334.0063
52	5	47	51	5	46	E	1	293937.948	-0.015	465.2308
52	5	47	51	6	46	E	1	293937.948	-0.014	465.2308
52	6	47	51	5	46	E	1	293937.948	-0.016	465.2308
52	6	47	51	6	46	E	1	293937.948	-0.015	465.2308
49	9	41	48	8	40	A	1	293946.464	0.086	455.1538
53	4	49	52	4	48	A	1	293956.803	-0.095	467.8888
53	4	49	52	5	48	A	1	293956.803	-0.095	467.8888
53	5	49	52	4	48	A	1	293956.803	-0.095	467.8888
53	5	49	52	5	48	A	1	293956.803	-0.095	467.8888

J'	K'_a	K'_c	J''	K''_a	K''_c	Sym.	$v_{t_{CH_3}}$	$\nu_{Obs.}$	$\nu_{Obs.} - \nu_{Calc.}$	E_{low}
50	7	43	49	8	42	A	0	293958.287	-0.091	330.6131
50	8	43	49	7	42	A	0	293960.383	0.004	330.6130
19	16	3	18	15	3	E	0	293990.625	-0.047	145.2533
52	5	47	51	5	46	E	0	293995.041	0.004	336.9269
52	6	47	51	6	46	E	0	293995.041	0.004	336.9269
52	5	47	51	5	46	A	0	293998.839	0.010	336.9214
52	5	47	51	6	46	A	0	293998.839	0.011	336.9214
52	6	47	51	5	46	A	0	293998.839	0.010	336.9214
52	6	47	51	6	46	A	0	293998.839	0.011	336.9214
19	16	4	18	15	4	E	0	294000.803	0.025	145.2397
19	16	3	18	15	4	A	0	294035.919	-0.010	145.2484
19	16	4	18	15	3	A	0	294035.919	-0.010	145.2484
48	9	39	47	10	38	A	1	294039.019	0.068	450.6795
53	4	49	52	4	48	E	1	294061.523	-0.009	467.6891
53	4	49	52	5	48	E	1	294061.523	-0.009	467.6891
53	5	49	52	4	48	E	1	294061.523	-0.009	467.6891
53	5	49	52	5	48	E	1	294061.523	-0.009	467.6891
49	8	41	48	9	40	E	1	294084.695	-0.019	455.0143
49	9	41	48	9	40	E	1	294103.334	-0.158	455.0143
53	4	49	52	4	48	E	0	294121.154	0.003	339.3885
53	5	49	52	5	48	E	0	294121.154	0.003	339.3885
53	4	49	52	4	48	A	0	294124.379	0.006	339.3822
53	4	49	52	5	48	A	0	294124.379	0.006	339.3822
53	5	49	52	4	48	A	0	294124.379	0.006	339.3822
53	5	49	52	5	48	A	0	294124.379	0.006	339.3822
49	8	41	48	9	40	E	0	294130.029	-0.018	326.7107
54	3	51	53	3	50	A	1	294134.236	-0.066	469.9381
54	3	51	53	4	50	A	1	294134.236	-0.066	469.9381
54	4	51	53	3	50	A	1	294134.236	-0.066	469.9381
54	4	51	53	4	50	A	1	294134.236	-0.066	469.9381
49	8	41	48	9	40	A	0	294135.649	-0.046	326.7063
49	9	41	48	8	40	E	1	294136.976	0.054	455.0132
49	9	41	48	9	40	E	0	294147.273	-0.246	326.7107
49	9	41	48	9	40	A	0	294153.262	-0.002	326.7063
49	8	41	48	8	40	E	0	294161.236	-0.007	326.7097
47	19	29	46	19	28	A	0	294163.635	-0.005	359.3948
49	8	41	48	8	40	A	0	294167.194	0.136	326.7053
47	19	29	46	19	28	E	0	294167.194	-0.119	359.3835
49	9	41	48	8	40	E	0	294178.697	-0.018	326.7097
49	9	41	48	8	40	A	0	294184.602	-0.025	326.7053
48	9	39	47	10	38	E	1	294206.558	0.190	450.5381
54	3	51	53	3	50	E	1	294219.487	-0.007	469.7087
54	3	51	53	4	50	E	1	294219.487	-0.007	469.7087
54	4	51	53	3	50	E	1	294219.487	-0.007	469.7087
54	4	51	53	4	50	E	1	294219.487	-0.007	469.7087
48	9	39	47	10	38	E	0	294263.286	-0.010	322.2388
48	9	39	47	10	38	A	0	294268.279	-0.022	322.2344
54	3	51	53	3	50	E	0	294281.759	0.005	341.4131
54	4	51	53	4	50	E	0	294281.759	0.005	341.4131
54	3	51	53	3	50	A	0	294284.391	0.019	341.4059
54	3	51	53	4	50	A	0	294284.391	0.019	341.4059
54	4	51	53	3	50	A	0	294284.391	0.019	341.4059
54	4	51	53	4	50	A	0	294284.391	0.019	341.4059
55	2	53	54	2	52	A	1	294333.844	-0.048	471.5680
55	2	53	54	3	52	A	1	294333.844	-0.048	471.5680
55	3	53	54	2	52	A	1	294333.844	-0.048	471.5680
55	3	53	54	3	52	A	1	294333.844	-0.048	471.5680
46	13	34	45	13	33	A	0	294386.825	-0.023	315.7879
46	13	34	45	13	33	E	0	294390.942	-0.096	315.7910
55	2	53	54	2	52	E	1	294397.468	0.014	471.3022
55	3	53	54	3	52	E	1	294397.468	0.014	471.3022
55	2	53	54	2	52	E	0	294462.828	0.007	343.0133
55	3	53	54	3	52	E	0	294462.828	0.007	343.0133
55	2	53	54	2	52	A	0	294464.774	0.003	343.0050
55	2	53	54	3	52	A	0	294464.774	0.003	343.0050
55	3	53	54	2	52	A	0	294464.774	0.003	343.0050
55	3	53	54	3	52	A	0	294464.774	0.003	343.0050
61	27	34	61	26	35	E	0	294481.320	-0.086	586.3805
61	27	35	61	26	36	A	0	294514.052	-0.009	586.3895
61	27	34	61	26	35	A	0	294514.052	-0.009	586.3895
61	27	35	61	26	36	E	0	294526.618	-0.207	586.3777
56	1	55	55	1	54	A	1	294547.368	0.016	472.7886
56	1	55	55	2	54	A	1	294547.368	0.016	472.7886
56	2	55	55	1	54	A	1	294547.368	0.016	472.7886
56	2	55	55	2	54	A	1	294547.368	0.016	472.7886
56	2	55	55	2	54	E	1	294586.158	0.039	472.4791
56	1	55	55	1	54	E	0	294655.351	-0.045	344.1989
56	2	55	55	2	54	E	0	294655.351	-0.045	344.1989
56	1	55	55	1	54	A	0	294656.589	0.003	344.1893
56	1	55	55	2	54	A	0	294656.589	0.003	344.1893
56	2	55	55	1	54	A	0	294656.589	0.003	344.1893
56	2	55	55	2	54	A	0	294656.589	0.003	344.1893
46	14	32	45	14	31	A	0	294728.902	0.095	320.3358
46	14	32	45	14	31	E	0	294731.364	0.034	320.3381
57	0	57	56	0	56	A	1	294769.672	0.190	473.6081
57	0	57	56	1	56	A	1	294769.672	0.190	473.6081
57	1	57	56	0	56	A	1	294769.672	0.190	473.6081
57	1	57	56	1	56	A	1	294769.672	0.190	473.6081
57	0	57	56	0	56	E	1	294779.208	-0.065	473.2466
57	1	57	56	1	56	E	1	294779.208	-0.065	473.2466
67	5	62	67	4	63	E	0	294796.754	-0.093	504.4927
67	6	62	67	5	63	E	0	294796.754	-0.093	504.4927
48	9	39	47	9	38	E	0	294804.285	-0.027	322.2208
48	9	39	47	9	38	A	0	294811.799	0.029	322.2163
21	15	7	20	14	7	E	0	294833.699	0.016	147.7461
48	10	39	47	9	38	A	1	294837.400	0.209	450.6627
57	0	57	56	0	56	A	0	294853.762	-0.152	344.9663
57	0	57	56	1	56	A	0	294853.762	-0.152	344.9663
57	1	57	56	0	56	A	0	294853.762	-0.152	344.9663
57	1	57	56	1	56	A	0	294853.762	-0.152	344.9663
57	0	57	56	0	56	E	0	294853.762	0.158	344.9775
57	1	57	56	1	56	E	0	294853.762	0.158	344.9775
21	15	7	20	14	6	A	0	294872.377	0.002	147.7534
21	15	6	20	14	7	A	0	294872.377	0.002	147.7534
47	11	37	46	11	36	E	1	294900.557	0.061	445.4564
47	11	37	46	11	36	E	0	294940.397	0.092	317.1629
47	11	37	46	11	36	A	0	294946.166	-0.012	317.1583
47	18	29	46	18	28	A	0	294956.556	-0.190	352.6116
47	18	30	46	18	29	A	0	294956.556	0.175	352.6115
47	18	29	46	18	28	E	0	294959.531	0.006	352.6129
47	18	30	46	18	29	E	0	294960.967	-0.032	352.6012
60	27	34	60	26	35	A	0	295077.055	0.082	573.6890
60	27	33	60	26	34	A	0	295077.055	0.082	573.6890
60	27	34	60	26	35	E	0	295090.196	-0.050	573.6771
48	10	39	47	9	38	E	1	295121.136	0.038	450.5190
48	10	39	47	9	38	E	0	295124.888	-0.018	322.2208

J'	K_a'	K_c'	J''	K_a''	K_c''	Sym.	$v_{t_{CH_3}}$	$\nu_{Obs.}$	$\nu_{Obs.} - \nu_{Calc.}$	E_{low}
48	10	39	47	9	38	A	0	295133.826	-0.052	322.2163
29	11	19	28	10	18	A	0	295433.615	-0.009	170.5475
29	11	18	28	10	18	E	0	295545.100	-0.032	170.5553
59	27	32	59	26	33	E	0	295577.661	-0.055	561.1953
23	14	10	22	13	10	E	0	295591.951	-0.032	151.4935
23	14	9	22	13	9	E	0	295593.190	-0.029	151.5072
59	27	33	59	26	34	A	0	295609.715	-0.022	561.2046
59	27	32	59	26	33	A	0	295609.715	-0.022	561.2046
59	27	33	59	26	34	E	0	295623.465	-0.028	561.1926
23	14	9	22	13	10	A	0	295633.787	-0.014	151.4993
23	14	10	22	13	9	A	0	295633.787	-0.014	151.4993
29	11	19	28	10	19	E	0	295673.918	0.063	170.5384
29	11	18	28	10	19	A	0	295864.979	-0.005	170.5353
47	17	31	46	17	30	A	0	295932.602	-0.012	346.2657
47	17	30	46	17	29	A	0	295936.847	-0.130	346.2659
47	17	30	46	17	29	E	0	295938.031	0.051	346.2688
47	17	31	46	17	30	E	0	295940.172	-0.014	346.2567
58	27	31	58	26	32	E	0	296082.027	-0.005	548.9264
58	27	32	58	26	33	A	0	296113.823	0.067	548.9358
58	27	31	58	26	32	A	0	296113.823	0.067	548.9358
58	27	32	58	26	33	E	0	296127.917	-0.052	548.9237
25	13	13	24	12	13	E	0	296175.302	0.011	156.5070
25	13	12	24	12	12	E	0	296182.560	-0.059	156.5204
25	13	13	24	12	12	A	0	296219.879	-0.006	156.5112
27	12	16	26	11	16	E	0	296363.293	-0.011	162.8293
27	12	15	26	11	15	E	0	296377.299	-0.050	162.8420
27	12	16	26	11	15	A	0	296407.162	-0.070	162.8318
27	12	15	26	11	16	A	0	296414.173	0.056	162.8316
57	27	30	57	26	31	E	0	296558.944	0.025	536.8727
69	7	63	69	6	64	E	0	296560.148	0.035	539.5752
69	6	63	69	5	64	A	0	296588.328	-0.073	539.5718
69	7	63	69	6	64	A	0	296588.328	-0.073	539.5718
57	27	31	57	26	32	A	0	296590.338	-0.022	536.8822
57	27	30	57	26	31	A	0	296590.338	-0.022	536.8822
57	27	31	57	26	32	E	0	296605.063	0.059	536.8701
48	29	20	47	29	19	A	0	296750.577	-0.043	459.3018
48	29	19	47	29	18	A	0	296750.577	-0.043	459.3018
48	29	19	47	29	18	E	0	296750.577	-0.322	459.2915
48	28	21	47	28	20	E	0	296963.702	0.051	448.4982
56	27	29	56	26	30	E	0	297009.558	-0.080	525.0338
56	27	30	56	26	31	A	0	297040.805	-0.003	525.0434
56	27	29	56	26	30	A	0	297040.805	-0.003	525.0434
56	27	30	56	26	31	E	0	297055.822	-0.038	525.0312
47	10	37	46	10	36	E	0	297153.780	-0.034	316.9548
47	16	32	46	16	31	A	0	297158.888	-0.038	340.3757
47	16	32	46	16	31	E	0	297183.321	-0.020	340.3688
47	16	31	46	16	30	E	0	297188.072	0.005	340.3812
48	27	22	47	27	21	A	0	297197.543	0.028	438.1086
48	27	21	47	27	20	A	0	297197.543	0.028	438.1086
48	27	21	47	27	20	E	0	297199.033	0.007	438.0974
47	16	31	46	16	30	A	0	297202.294	-0.047	340.3773
55	27	28	55	26	29	E	0	297435.336	-0.050	513.4094
48	26	22	47	26	21	A	0	297460.220	-0.050	428.0984
48	26	23	47	26	22	A	0	297460.220	-0.050	428.0984
55	27	29	55	26	30	E	0	297481.641	-0.093	513.4068
45	11	34	44	11	33	E	0	297482.864	0.004	300.4739
48	10	38	47	11	37	A	0	297551.106	-0.007	326.9966
48	10	38	47	11	37	E	0	297555.055	-0.018	327.0010
31	10	21	30	9	22	E	0	297623.199	0.027	179.6904
31	10	21	30	9	22	A	0	297637.433	-0.023	179.6836
48	25	24	47	25	23	A	0	297755.458	-0.068	418.4814
48	25	23	47	25	22	A	0	297755.458	-0.068	418.4814
66	4	62	66	3	63	E	0	297808.073	-0.035	482.3559
66	5	62	66	4	63	E	0	297808.073	-0.035	482.3559
54	27	27	54	26	28	E	0	297837.228	-0.075	501.9990
66	4	62	66	3	63	A	0	297845.787	0.003	482.3499
66	5	62	66	4	63	A	0	297845.787	0.003	482.3499
54	27	28	54	26	29	A	0	297867.940	-0.031	502.0087
54	27	27	54	26	28	A	0	297867.940	-0.031	502.0087
54	27	28	54	26	29	E	0	297883.716	-0.052	501.9964
66	4	62	66	3	63	E	1	297896.517	-0.290	610.6203
66	5	62	66	4	63	E	1	297896.517	-0.289	610.6203
45	12	33	44	12	32	A	0	298067.880	-0.051	303.0684
45	12	33	44	12	32	E	0	298077.517	-0.023	303.0746
48	24	24	47	24	23	A	0	298089.449	-0.017	409.2598
48	24	25	47	24	24	A	0	298089.449	-0.017	409.2598
45	11	35	44	10	34	E	0	298225.177	-0.031	297.5518
53	27	27	53	26	28	A	0	298246.888	-0.021	490.8120
53	27	26	53	26	27	A	0	298246.888	-0.021	490.8120
53	27	27	53	26	28	E	0	298263.099	0.052	490.7997
45	11	35	44	10	34	A	0	298271.799	0.032	297.5455
48	23	26	47	23	25	A	0	298469.873	-0.041	400.4362
48	23	25	47	23	24	A	0	298469.873	-0.041	400.4362
48	23	26	47	23	25	E	0	298471.834	0.103	400.4230
52	27	25	52	26	26	E	0	298573.878	-0.057	479.8187
63	2	61	63	1	62	E	1	298581.741	-0.302	555.8758
63	3	61	63	2	62	E	1	298581.741	-0.302	555.8758
44	11	34	43	10	33	E	0	298600.003	-0.071	288.0545
52	27	26	52	26	27	A	0	298604.096	-0.051	479.8286
52	27	25	52	26	26	A	0	298604.096	-0.051	479.8286
52	27	26	52	26	27	E	0	298620.498	-0.110	479.8162
44	11	34	43	10	33	A	0	298657.444	0.038	288.0478
47	15	33	46	15	32	A	0	298677.095	-0.044	334.9664
51	7	44	50	8	43	A	1	298838.607	-0.018	468.8677
51	8	44	50	7	43	A	1	298839.577	-0.001	468.8676
52	6	46	51	6	45	A	1	298841.596	-0.035	472.2729
52	6	46	51	7	45	A	1	298841.596	-0.017	472.2729
52	7	46	51	6	45	A	1	298841.596	-0.044	472.2729
52	7	46	51	7	45	A	1	298841.596	-0.026	472.2729
48	22	26	47	22	25	A	0	298906.932	-0.024	392.0141
48	22	27	47	22	26	A	0	298906.932	-0.024	392.0141
48	22	26	47	22	25	E	0	298909.260	-0.100	392.0091
48	22	27	47	22	26	E	0	298909.260	0.079	392.0011
51	27	24	51	26	25	E	0	298910.618	-0.057	469.0481
53	5	48	52	5	47	A	1	298933.008	-0.084	475.2075
53	5	48	52	6	47	A	1	298933.008	-0.084	475.2075
53	6	48	52	5	47	A	1	298933.008	-0.084	475.2075
53	6	48	52	6	47	A	1	298933.008	-0.084	475.2075
51	27	25	51	26	26	A	0	298940.658	-0.018	469.0581
51	27	24	51	26	25	A	0	298940.658	-0.018	469.0581
51	27	25	51	26	26	E	0	298957.436	-0.002	469.0457
50	8	42	49	9	41	A	1	298977.946	0.089	464.9588
52	6	46	51	6	45	E	1	298981.248	0.006	472.1193
52	6	46	51	7	45	E	1	298981.248	0.028	472.1193
52	7	46	51	6	45	E	1	298981.248	-0.006	472.1193
52	7	46	51	7	45	E	1	298981.248	0.017	472.1193

J'	K'_a	K'_c	J''	K''_a	K''_c	Sym.	$v_{t_{\mathrm{CH_3}}}$	$\nu_{\mathrm{Obs.}}$	$\nu_{\mathrm{Obs.}} - \nu_{\mathrm{Calc.}}$	E_{low}
50	9	42	49	9	41	A	1	298986.787	0.109	464.9588
50	8	42	49	8	41	A	1	298993.818	0.040	464.9582
51	7	44	50	8	43	E	1	298997.398	-0.018	468.7268
51	8	44	50	7	43	E	1	298998.564	-0.007	468.7268
50	9	42	49	8	41	A	1	299002.638	0.039	464.9582
47	15	32	46	15	31	E	0	299006.209	-0.010	334.9836
47	15	32	46	15	31	A	0	299025.679	-0.090	334.9812
52	6	46	51	6	45	E	0	299037.609	-0.003	343.8156
52	7	46	51	7	45	E	0	299037.609	0.007	343.8156
52	6	46	51	6	45	A	0	299041.931	0.006	343.8107
52	6	46	51	7	45	A	0	299041.931	0.026	343.8108
52	7	46	51	6	45	A	0	299041.931	-0.005	343.8107
52	7	46	51	7	45	A	0	299041.931	0.016	343.8108
51	8	44	50	7	43	A	0	299056.209	0.037	340.4185
54	4	50	53	4	49	A	1	299078.515	-0.099	477.6941
54	4	50	53	5	49	A	1	299078.515	-0.099	477.6941
54	5	50	53	4	49	A	1	299078.515	-0.099	477.6941
54	5	50	53	5	49	A	1	299078.515	-0.099	477.6941
53	5	48	52	5	47	E	0	299113.988	0.011	346.7335
53	6	48	52	6	47	E	0	299113.988	0.011	346.7335
53	5	48	52	5	47	A	0	299117.746	0.010	346.7281
53	5	48	52	6	47	A	0	299117.746	0.010	346.7281
53	6	48	52	5	47	A	0	299117.746	0.010	346.7281
53	6	48	52	6	47	A	0	299117.746	0.010	346.7281
46	11	36	45	10	35	E	0	299136.316	-0.046	307.1847
47	12	36	46	12	35	E	0	299148.597	-0.037	321.4209
47	12	36	46	12	35	A	0	299150.994	-0.046	321.4167
50	8	42	49	9	41	E	1	299158.123	-0.053	464.8246
46	11	36	45	10	35	A	0	299172.943	-0.002	307.1790
50	8	42	49	8	41	E	1	299176.984	0.031	464.8239
54	4	50	53	4	49	E	1	299182.543	-0.040	477.4979
54	4	50	53	5	49	E	1	299182.543	-0.040	477.4979
54	5	50	53	4	49	E	1	299182.543	-0.040	477.4979
54	5	50	53	5	49	E	1	299182.543	-0.040	477.4979
50	9	42	49	8	41	E	1	299187.430	0.011	464.8239
49	9	40	48	10	39	A	1	299198.413	0.054	460.4974
50	8	42	49	9	41	E	0	299206.035	-0.017	336.5224
50	8	42	49	9	41	A	0	299211.387	-0.244	336.5182
50	9	42	49	9	41	E	0	299215.661	-0.104	336.5224
50	9	42	49	9	41	A	0	299221.387	-0.012	336.5182
50	8	42	49	8	41	E	0	299223.521	-0.004	336.5219
50	27	23	50	26	24	E	0	299227.577	-0.063	458.4901
50	8	42	49	8	41	A	0	299229.168	-0.033	336.5176
50	9	42	49	8	41	E	0	299233.231	-0.006	336.5219
50	9	42	49	8	41	A	0	299238.970	0.001	336.5176
54	4	50	53	4	49	E	0	299243.989	0.009	349.1993
54	5	50	53	5	49	E	0	299243.989	0.009	349.1993
54	4	50	53	4	49	A	0	299247.194	0.013	349.1931
54	4	50	53	5	49	A	0	299247.194	0.013	349.1931
54	5	50	53	4	49	A	0	299247.194	0.013	349.1931
54	5	50	53	5	49	A	0	299247.194	0.013	349.1931
55	3	52	54	3	51	A	1	299257.659	-0.167	479.7494
55	3	52	54	4	51	A	1	299257.659	-0.167	479.7494
55	4	52	54	3	51	A	1	299257.659	-0.167	479.7494
55	4	52	54	4	51	A	1	299257.659	-0.167	479.7494
18	17	1	17	16	1	E	0	299272.469	-0.086	147.4613
50	27	24	50	26	25	E	0	299274.517	0.030	458.4877
18	17	2	17	16	2	E	0	299288.011	0.020	147.4480
18	17	2	17	16	1	A	0	299319.157	-0.032	147.4578
18	17	1	17	16	2	A	0	299319.157	-0.032	147.4578
55	3	52	54	3	51	E	1	299342.632	-0.005	479.5228
55	3	52	54	4	51	E	1	299342.632	-0.005	479.5228
55	4	52	54	3	51	E	1	299342.632	-0.005	479.5228
55	4	52	54	4	51	E	1	299342.632	-0.005	479.5228
49	10	40	48	10	39	A	1	299371.690	0.147	460.4974
49	9	40	48	10	39	E	1	299380.096	0.042	460.3632
55	3	52	54	3	51	E	0	299406.528	-0.000	351.2293
55	4	52	54	4	51	E	0	299406.528	-0.000	351.2293
55	3	52	54	3	51	A	0	299409.161	0.027	351.2222
55	3	52	54	4	51	A	0	299409.161	0.027	351.2222
55	4	52	54	3	51	A	0	299409.161	0.027	351.2222
55	4	52	54	4	51	A	0	299409.161	0.027	351.2222
48	21	28	47	21	27	A	0	299414.004	0.145	383.9978
48	21	27	47	21	26	A	0	299414.004	0.144	383.9978
48	21	27	47	21	26	E	0	299416.489	0.039	383.9944
49	9	40	48	10	39	E	0	299431.998	-0.015	332.0651
49	9	40	48	10	39	A	0	299437.529	-0.009	332.0609
56	2	54	55	2	53	A	1	299458.226	-0.047	481.3859
56	2	54	55	3	53	A	1	299458.226	-0.047	481.3859
56	3	54	55	2	53	A	1	299458.226	-0.047	481.3859
56	3	54	55	3	53	A	1	299458.226	-0.047	481.3859
56	2	54	55	2	53	E	1	299521.663	0.021	481.1223
56	3	54	55	3	53	E	1	299521.663	0.021	481.1223
49	27	22	49	26	23	E	0	299525.687	-0.049	448.1443
49	27	23	49	26	24	A	0	299555.305	-0.042	448.1544
49	27	22	49	26	23	A	0	299555.305	-0.042	448.1544
49	10	40	48	10	39	E	1	299580.488	-0.008	460.3632
56	2	54	55	2	53	E	0	299588.537	0.014	352.8356
56	3	54	55	3	53	E	0	299588.537	0.014	352.8356
56	2	54	55	2	53	A	0	299590.473	0.006	352.8273
56	2	54	55	3	53	A	0	299590.473	0.006	352.8273
56	3	54	55	2	53	A	0	299590.473	0.006	352.8273
56	3	54	55	3	53	A	0	299590.473	0.006	352.8273
49	10	40	48	10	39	E	0	299619.990	0.090	332.0651
49	10	40	48	10	39	A	0	299626.368	0.021	332.0609
49	10	40	48	9	39	A	1	299668.139	0.167	460.4876
57	1	56	56	1	55	A	1	299672.157	0.017	482.6136
57	1	56	56	2	55	A	1	299672.157	0.017	482.6136
57	2	56	56	1	55	A	1	299672.157	0.017	482.6136
57	2	56	56	2	55	A	1	299672.157	0.017	482.6136
57	1	56	56	1	55	E	1	299710.864	0.047	482.3054
57	2	56	56	2	55	E	1	299710.864	0.047	482.3054
49	9	40	48	9	39	E	1	299721.045	-0.016	460.3518
49	9	40	48	9	39	E	0	299752.739	0.131	332.0544
49	9	40	48	9	39	A	0	299759.620	-0.027	332.0501
57	1	56	56	1	55	E	0	299781.491	-0.023	354.0276
57	2	56	56	2	55	E	0	299781.491	-0.023	354.0276
57	1	56	56	1	55	A	0	299782.743	0.042	354.0180
57	1	56	56	2	55	A	0	299782.743	0.042	354.0180
57	2	56	56	1	55	A	0	299782.743	0.042	354.0180
57	2	56	56	2	55	A	0	299782.743	0.042	354.0180
48	27	21	48	26	22	E	0	299805.693	-0.138	438.0104
48	27	22	48	26	23	A	0	299835.229	-0.032	438.0206
48	27	21	48	26	22	A	0	299835.229	-0.032	438.0206
48	27	22	48	26	23	E	0	299852.797	-0.024	438.0081
58	0	58	57	0	57	A	1	299894.635	0.112	483.4405

J'	K'_a	K'_c	J''	K''_a	K''_c	Sym.	$v_{t_{CH_3}}$	$\nu_{Obs.}$	$\nu_{Obs.} - \nu_{Calc.}$	E_{low}
58	0	58	57	1	57	A	1	299894.635	0.112	483.4405
58	1	58	57	0	57	A	1	299894.635	0.112	483.4405
58	1	58	57	1	57	A	1	299894.635	0.112	483.4405
58	0	58	57	0	57	E	1	299904.269	0.042	483.0794
58	1	58	57	1	57	E	1	299904.269	0.042	483.0794
49	10	40	48	9	39	E	1	299921.526	0.022	460.3518
49	10	40	48	9	39	E	0	299940.413	-0.081	332.0544
49	10	40	48	9	39	A	0	299948.424	-0.031	332.0501
58	0	58	57	0	57	A	0	299980.076	-0.146	354.8015
58	0	58	57	1	57	A	0	299980.076	-0.146	354.8015
58	1	58	57	0	57	A	0	299980.076	-0.146	354.8015
58	1	58	57	1	57	A	0	299980.076	-0.146	354.8015
58	0	58	57	0	57	E	0	299980.076	0.162	354.8127
58	1	58	57	1	57	E	0	299980.076	0.162	354.8127
48	20	28	47	20	27	A	0	300008.496	-0.047	376.3930
48	20	29	47	20	28	A	0	300008.496	-0.043	376.3930
48	20	29	47	20	28	E	0	300011.643	-0.161	376.3810
47	27	20	47	26	21	E	0	300068.650	-0.108	428.0882
48	11	38	47	11	37	A	0	300105.037	0.029	326.9966
47	27	21	47	26	22	E	0	300115.790	-0.020	428.0859
20	16	4	19	15	4	E	0	300132.289	-0.079	149.1589
20	16	5	19	15	5	E	0	300142.488	0.054	149.1453
46	13	33	45	13	32	A	0	300170.165	-0.104	316.1672
20	16	4	19	15	5	A	0	300177.597	-0.013	149.1539
20	16	5	19	15	4	A	0	300177.597	-0.013	149.1539
46	13	33	45	13	32	E	0	300184.354	-0.005	316.1714
47	14	34	46	14	33	A	0	300250.536	-0.093	330.0579
47	14	34	46	14	33	E	0	300259.833	0.007	330.0594
43	11	33	42	10	32	E	0	300271.244	0.140	278.6970
46	27	19	46	26	20	E	0	300315.305	-0.012	418.3772
43	11	33	42	10	32	A	0	300338.759	-0.005	278.6898
46	27	20	46	26	21	A	0	300344.365	-0.049	418.3875
46	27	19	46	26	20	A	0	300344.365	-0.049	418.3875
49	11	38	48	12	37	A	0	300406.478	-0.050	341.5614
49	11	38	48	12	37	E	0	300435.483	0.000	341.5654
45	27	18	45	26	19	E	0	300546.229	-0.047	408.8772
45	27	19	45	26	20	A	0	300575.093	-0.126	408.8876
45	27	18	45	26	19	A	0	300575.093	-0.126	408.8876
45	27	19	45	26	20	E	0	300593.449	0.016	408.8750
65	3	62	65	2	63	E	0	300636.960	0.031	460.4605
65	4	62	65	3	63	E	0	300636.960	0.031	460.4605
65	3	62	65	2	63	A	0	300680.543	0.579	460.4529
65	4	62	65	3	63	A	0	300680.543	0.579	460.4529
48	19	30	47	19	29	A	0	300715.986	-0.006	369.2070
48	19	29	47	19	28	E	0	300718.882	-0.107	369.2068
48	19	30	47	19	29	E	0	300720.029	0.066	369.1958
44	27	18	44	26	19	A	0	300791.120	-0.053	399.5983
44	27	17	44	26	18	A	0	300791.120	-0.053	399.5983
44	27	18	44	26	19	E	0	300809.576	-0.000	399.5857
47	13	35	46	13	34	A	0	300874.755	-0.060	325.6076
47	13	35	46	13	34	E	0	300877.614	-0.117	325.6108
30	11	19	29	10	19	E	0	300927.826	-0.095	176.6749
22	15	7	21	14	7	E	0	300955.572	-0.002	152.0860
22	15	8	21	14	8	E	0	300960.058	0.013	152.0723
43	27	16	43	26	17	E	0	300964.194	-0.129	390.5090
70	28	43	70	27	44	E	0	300986.702	-0.210	720.0892
43	27	17	43	26	18	A	0	300992.627	-0.358	390.5195
43	27	16	43	26	17	A	0	300992.627	-0.358	390.5195
22	15	8	21	14	7	A	0	300998.757	-0.027	152.0796
22	15	7	21	14	8	A	0	300998.757	-0.027	152.0796
43	27	17	43	26	18	E	0	301011.519	-0.046	390.5068
42	27	15	42	26	16	E	0	301152.775	-0.033	381.6403
47	11	37	46	10	36	E	0	301177.407	-0.152	316.9548
42	27	16	42	26	17	A	0	301181.304	-0.038	381.6507
42	27	15	42	26	16	A	0	301181.304	-0.038	381.6507
42	27	16	42	26	17	E	0	301200.139	0.054	381.6381
47	11	37	46	10	36	A	0	301205.741	-0.024	316.9495
30	11	20	29	10	20	E	0	301320.741	-0.019	176.6487
41	27	14	41	26	15	E	0	301328.476	-0.015	372.9814
41	27	15	41	26	16	A	0	301356.863	-0.039	372.9919
41	27	14	41	26	15	A	0	301356.863	-0.039	372.9919
41	27	15	41	26	16	E	0	301375.851	0.054	372.9792
40	27	13	40	26	14	E	0	301492.003	-0.002	364.5321
70	6	64	70	5	65	E	1	301504.982	-0.111	680.7125
70	7	64	70	6	65	E	1	301504.982	-0.111	680.7125
40	27	14	40	26	15	A	0	301520.099	-0.204	364.5426
40	27	13	40	26	14	A	0	301520.099	-0.204	364.5426
40	27	14	40	26	15	E	0	301539.336	-0.002	364.5299
70	6	64	70	5	65	A	0	301559.878	-0.078	552.4527
70	7	64	70	6	65	A	0	301559.878	-0.078	552.4527
48	18	30	47	18	29	E	0	301576.025	0.032	362.4517
48	18	31	47	18	30	E	0	301577.546	-0.068	362.4401
48	10	38	47	10	37	E	0	301578.850	0.030	326.8668
30	11	19	29	10	20	A	0	301583.424	-0.011	176.6445
48	10	38	47	10	37	A	0	301590.369	0.146	326.8619
39	27	12	39	26	13	E	0	301643.963	-0.003	356.2922
39	27	13	39	26	14	A	0	301672.081	-0.075	356.3027
39	27	12	39	26	13	A	0	301672.081	-0.075	356.3027
24	14	11	23	13	11	E	0	301683.817	-0.005	156.2462
24	14	10	23	13	10	E	0	301685.134	-0.059	156.2598
39	27	13	39	26	14	E	0	301691.335	0.014	356.2900
30	11	19	29	10	20	E	0	301713.463	-0.093	176.6487
24	14	10	23	13	11	A	0	301725.720	-0.010	156.2520
24	14	11	23	13	10	A	0	301725.720	-0.010	156.2520
69	28	41	69	27	42	A	0	301771.521	-0.002	705.4624
69	28	42	69	27	43	A	0	301771.521	-0.003	705.4624
38	27	12	38	26	13	A	0	301812.977	-0.078	348.2719
38	27	11	38	26	12	A	0	301812.977	-0.078	348.2719
38	27	12	38	26	13	E	0	301832.385	0.046	348.2592
37	27	10	37	26	11	E	0	301915.735	0.158	340.4394
37	27	11	37	26	12	A	0	301943.569	-0.002	340.4500
37	27	10	37	26	11	A	0	301943.569	-0.002	340.4500
37	27	11	37	26	12	E	0	301963.016	0.051	340.4372
36	27	9	36	26	10	E	0	302036.388	0.040	332.8260
36	27	10	36	26	11	A	0	302064.175	-0.079	332.8366
36	27	9	36	26	10	A	0	302064.175	-0.079	332.8366
36	27	10	36	26	11	E	0	302083.806	0.057	332.8239
35	27	8	35	26	9	E	0	302147.845	0.030	325.4211
35	27	9	35	26	10	A	0	302175.586	-0.052	325.4317
35	27	8	35	26	9	A	0	302175.586	-0.052	325.4317
26	13	14	25	12	14	E	0	302195.460	-0.055	161.6950
26	13	13	25	12	13	E	0	302203.047	-0.054	161.7083
28	12	17	27	11	17	E	0	302233.907	-0.005	168.4671
26	13	14	25	12	13	A	0	302240.310	0.061	161.6992
28	12	16	27	11	16	E	0	302248.222	-0.061	168.4798
34	27	7	34	26	8	E	0	302250.516	0.027	318.2243

J'	K'_a	K'_c	J''	K''_a	K''_c	Sym.	$v_{t_{CH_3}}$	$\nu_{Obs.}$	$\nu_{Obs.} - \nu_{Calc.}$	E_{low}
28	12	17	27	11	16	A	0	302273.559	-0.069	168.4697
34	27	8	34	26	9	A	0	302278.181	-0.054	318.2350
34	27	7	34	26	8	A	0	302278.181	-0.054	318.2350
28	12	16	27	11	17	A	0	302289.542	0.014	168.4692
34	27	8	34	26	9	E	0	302297.948	0.042	318.2223
33	27	6	33	26	7	E	0	302344.881	0.012	311.2356
47	14	33	46	14	32	A	0	302351.138	-0.047	330.1669
47	14	33	46	14	32	E	0	302358.588	-0.025	330.1693
33	27	7	33	26	8	A	0	302372.531	-0.011	311.2462
33	27	6	33	26	7	A	0	302372.531	-0.011	311.2462
46	11	35	45	11	34	E	0	302388.263	-0.125	310.3969
33	27	7	33	26	8	E	0	302392.302	0.014	311.2335
46	11	35	45	11	34	A	0	302400.623	-0.043	310.3899
32	27	5	32	26	6	E	0	302431.467	0.037	304.4545
32	27	6	32	26	7	A	0	302458.996	-0.040	304.4652
32	27	5	32	26	6	A	0	302458.996	-0.040	304.4652
68	28	40	68	27	41	E	0	302498.913	-0.077	691.0349
31	27	4	31	26	5	E	0	302510.573	-0.063	297.8811
68	28	40	68	27	41	A	0	302529.382	-0.016	691.0441
68	28	41	68	27	42	A	0	302529.382	-0.017	691.0441
31	27	5	31	26	6	A	0	302538.109	-0.071	297.8918
31	27	4	31	26	5	A	0	302538.109	-0.071	297.8918
68	28	41	68	27	42	E	0	302543.878	-0.228	691.0332
31	27	5	31	26	6	E	0	302558.276	0.218	297.8791
48	17	32	47	17	31	A	0	302631.504	0.007	356.1370
48	17	32	47	17	31	E	0	302641.397	-0.075	356.1282
29	27	3	29	26	4	E	0	302696.245	0.080	285.3541
49	29	21	48	29	20	A	0	303048.083	0.039	469.2003
49	29	20	48	29	19	A	0	303048.083	0.039	469.2003
49	29	20	48	29	19	E	0	303048.083	-0.275	469.1901
49	29	21	48	29	20	E	0	303049.385	0.063	469.1877
42	11	32	41	10	31	E	0	303060.338	-0.058	269.4905
67	28	39	67	27	40	E	0	303219.021	-0.165	676.8353
67	28	39	67	27	40	A	0	303249.122	-0.045	676.8446
67	28	40	67	27	41	A	0	303249.122	-0.045	676.8446
67	28	40	67	27	41	E	0	303264.464	-0.056	676.8336
49	28	21	48	28	20	A	0	303273.359	0.079	458.4158
49	28	22	48	28	21	A	0	303273.359	0.079	458.4158
49	28	22	48	28	21	E	0	303274.750	0.023	458.4038
64	2	62	64	1	63	E	0	303297.769	0.066	438.8119
64	3	62	64	2	63	E	0	303297.769	0.066	438.8119
49	27	23	48	27	22	A	0	303523.664	-0.028	448.0221
49	27	22	48	27	21	A	0	303523.664	-0.028	448.0221
49	27	22	48	27	21	E	0	303525.395	0.081	448.0109
49	10	39	48	11	38	A	0	303616.797	0.031	337.0071
49	10	39	48	11	38	E	0	303616.797	-0.091	337.0112
66	28	38	66	27	39	E	0	303903.004	-0.075	662.8538
66	28	38	66	27	39	A	0	303932.627	-0.025	662.8633
66	28	39	66	27	40	A	0	303932.627	-0.025	662.8633
52	7	45	51	7	44	A	1	303937.647	-0.098	478.8359
52	7	45	51	8	44	A	1	303937.647	0.232	478.8359
52	8	45	51	7	44	A	1	303937.647	-0.273	478.8359
52	8	45	51	8	44	A	1	303937.647	0.058	478.8359
66	28	39	66	27	40	E	0	303948.492	-0.125	662.8522
53	6	47	52	6	46	A	1	303953.236	-0.045	482.2412
53	6	47	52	7	46	A	1	303953.236	-0.035	482.2412
53	7	47	52	6	46	A	1	303953.236	-0.049	482.2412
53	7	47	52	7	46	A	1	303953.236	-0.040	482.2412
48	16	33	47	16	32	A	0	303962.177	-0.109	350.2878
48	16	33	47	16	32	E	0	303995.697	-0.070	350.2818
51	12	39	50	13	38	E	0	303999.584	-0.028	366.9983
48	16	32	47	16	31	E	0	304019.031	-0.053	350.2943
48	16	32	47	16	31	A	0	304041.533	-0.028	350.2909
54	5	49	53	5	48	A	1	304051.320	-0.096	485.1788
54	5	49	53	6	48	A	1	304051.320	-0.096	485.1788
54	6	49	53	5	48	A	1	304051.320	-0.096	485.1788
54	6	49	53	6	48	A	1	304051.320	-0.096	485.1788
51	8	43	50	9	42	A	1	304055.385	0.058	474.9319
51	9	43	50	9	42	A	1	304060.173	-0.005	474.9319
51	8	43	50	8	42	A	1	304064.219	0.072	474.9316
51	9	43	50	8	42	A	1	304069.066	0.067	474.9316
53	6	47	52	6	46	E	1	304091.304	-0.010	482.0923
53	6	47	52	7	46	E	1	304091.304	0.001	482.0923
53	7	47	52	6	46	E	1	304091.304	-0.016	482.0923
53	7	47	52	7	46	E	1	304091.304	-0.004	482.0923
52	7	45	51	7	44	E	1	304094.177	-0.086	478.7003
52	7	45	51	8	44	E	1	304094.177	0.317	478.7003
52	8	45	51	7	44	E	1	304094.177	-0.299	478.7003
52	8	45	51	8	44	E	1	304094.177	0.103	478.7003
49	25	25	48	25	24	A	0	304118.320	-0.050	428.4135
49	25	24	48	25	23	A	0	304118.320	-0.050	428.4135
48	11	38	47	10	37	E	0	304122.188	-0.254	326.8668
48	11	38	47	10	37	A	0	304144.065	-0.053	326.8619
54	5	49	53	5	48	E	1	304172.210	-0.015	485.0109
54	5	49	53	6	48	E	1	304172.210	-0.014	485.0109
54	6	49	53	5	48	E	1	304172.210	-0.015	485.0109
54	6	49	53	6	48	E	1	304172.210	-0.014	485.0109
55	4	51	54	4	50	A	1	304200.230	-0.109	487.6703
55	4	51	54	5	50	A	1	304200.230	-0.109	487.6703
55	5	51	54	4	50	A	1	304200.230	-0.109	487.6703
55	5	51	54	5	50	A	1	304200.230	-0.109	487.6703
54	5	49	53	5	48	E	0	304233.208	-0.016	356.7109
54	6	49	53	6	48	E	0	304233.208	-0.016	356.7109
54	5	49	53	5	48	A	0	304236.967	0.016	356.7056
54	5	49	53	6	48	A	0	304236.967	0.017	356.7056
54	6	49	53	5	48	A	0	304236.967	0.016	356.7056
54	6	49	53	6	48	A	0	304236.967	0.016	356.7056
51	8	43	50	8	42	E	1	304243.265	0.008	474.8034
51	9	43	50	8	42	E	1	304249.046	-0.005	474.8034
51	8	43	50	9	42	A	0	304288.756	-0.139	346.4992
51	9	43	50	9	42	E	0	304288.756	-0.005	346.5032
51	8	43	50	8	42	E	0	304293.093	-0.020	346.5029
51	9	43	50	9	42	A	0	304294.381	0.093	346.4992
51	8	43	50	8	42	A	0	304298.526	-0.138	346.4988
51	9	43	50	8	42	E	0	304298.526	0.052	346.5029
50	9	41	49	10	40	A	1	304308.339	-0.011	470.4834
55	4	51	54	4	50	E	0	304366.840	0.008	359.1810
55	5	51	54	5	50	E	0	304366.840	0.008	359.1810
55	4	51	54	4	50	A	0	304370.026	0.013	359.1750
55	4	51	54	5	50	A	0	304370.026	0.013	359.1750
55	5	51	54	4	50	A	0	304370.026	0.013	359.1750
55	5	51	54	5	50	A	0	304370.026	0.013	359.1750
56	3	53	55	3	52	A	1	304381.163	-0.080	489.7316
56	3	53	55	4	52	A	1	304381.163	-0.080	489.7316
56	4	53	55	3	52	A	1	304381.163	-0.080	489.7316
56	4	53	55	4	52	A	1	304381.163	-0.080	489.7316

J'	K'_a	K'_c	J''	K''_a	K''_c	Sym.	$v_{t_{CH_3}}$	$\nu_{Obs.}$	$\nu_{Obs.} - \nu_{Calc.}$	E_{low}
50	10	41	49	10	40	A	1	304408.713	0.200	470.4834
56	3	53	55	3	52	E	1	304465.694	0.017	489.5078
56	4	53	55	4	52	E	1	304465.694	0.017	489.5078
49	24	25	48	24	24	A	0	304474.638	-0.032	419.2030
49	24	26	48	24	25	A	0	304474.638	-0.032	419.2030
50	9	41	49	9	40	A	1	304481.811	0.278	470.4776
50	9	41	49	10	40	E	1	304497.096	0.070	470.3561
56	3	53	55	3	52	E	0	304531.204	0.002	361.2164
56	4	53	55	4	52	E	0	304531.204	0.002	361.2164
56	3	53	55	3	52	A	0	304533.795	-0.002	361.2094
56	3	53	55	4	52	A	0	304533.795	-0.002	361.2094
56	4	53	55	3	52	A	0	304533.795	-0.002	361.2094
56	4	53	55	4	52	A	0	304533.795	-0.002	361.2094
50	9	41	49	10	40	E	0	304547.240	-0.009	342.0594
57	2	55	56	2	54	A	1	304582.463	-0.036	491.3748
57	2	55	56	3	54	A	1	304582.463	-0.036	491.3748
57	3	55	56	2	54	A	1	304582.463	-0.036	491.3748
57	3	55	56	3	54	A	1	304582.463	-0.036	491.3748
50	10	41	49	10	40	E	1	304613.718	0.063	470.3561
57	2	55	56	2	54	E	1	304645.701	0.022	491.1132
57	3	55	56	3	54	E	1	304645.701	0.022	491.1132
50	10	41	49	10	40	A	0	304662.534	-0.062	342.0553
57	2	55	56	2	54	E	0	304714.089	0.015	362.8288
57	3	55	56	3	54	E	0	304714.089	0.015	362.8288
57	2	55	56	2	54	A	0	304716.018	0.006	362.8206
57	2	55	56	3	54	A	0	304716.018	0.006	362.8206
57	3	55	56	2	54	A	0	304716.018	0.006	362.8206
57	3	55	56	3	54	A	0	304716.018	0.006	362.8206
50	9	41	49	9	40	A	0	304741.939	0.095	342.0490
48	12	37	47	12	36	E	0	304770.782	0.021	331.3994
48	12	37	47	12	36	A	0	304774.230	-0.088	331.3953
58	1	57	57	1	56	A	1	304796.781	0.021	492.6096
58	1	57	57	2	56	A	1	304796.781	0.021	492.6096
58	2	57	57	1	56	A	1	304796.781	0.021	492.6096
58	2	57	57	2	56	A	1	304796.781	0.021	492.6096
50	10	41	49	9	40	E	1	304814.094	-0.004	470.3494
58	1	57	57	1	56	E	1	304835.405	0.056	492.3027
58	2	57	57	2	56	E	1	304835.405	0.056	492.3027
50	10	41	49	9	40	A	0	304851.356	-0.048	342.0490
58	1	57	57	2	56	E	0	304907.442	-0.024	364.0272
58	2	57	57	1	56	E	0	304907.442	-0.024	364.0272
58	1	57	57	1	56	A	0	304908.699	0.048	364.0177
58	1	57	57	2	56	A	0	304908.699	0.048	364.0177
58	2	57	57	1	56	A	0	304908.699	0.048	364.0177
58	2	57	57	2	56	A	0	304908.699	0.048	364.0177
59	0	59	58	0	58	A	1	305019.539	0.144	493.4439
59	0	59	58	1	58	A	1	305019.539	0.144	493.4439
59	1	59	58	0	58	A	1	305019.539	0.144	493.4439
59	1	59	58	1	58	A	1	305019.539	0.144	493.4439
59	0	59	58	0	58	E	1	305029.058	0.044	493.0831
59	1	59	58	1	58	E	1	305029.058	0.044	493.0831
59	0	59	58	0	58	A	0	305106.220	-0.141	364.8078
59	0	59	58	1	58	A	0	305106.220	-0.141	364.8078
59	1	59	58	0	58	A	0	305106.220	-0.141	364.8078
59	1	59	58	1	58	A	0	305106.220	-0.141	364.8078
59	0	59	58	0	58	E	0	305106.220	0.164	364.8190
59	1	59	58	1	58	E	0	305106.220	0.164	364.8190
49	11	39	48	11	38	E	1	305157.754	0.204	465.3021
49	22	27	48	22	26	A	0	305348.286	-0.075	401.9846
49	22	28	48	22	27	A	0	305348.286	-0.075	401.9846
49	22	28	48	22	27	E	0	305350.804	0.050	401.9717
19	17	3	18	16	2	A	0	305464.903	-0.029	151.1535
19	17	2	18	16	3	A	0	305464.903	-0.029	151.1535
46	12	34	45	12	33	A	0	305562.884	-0.031	313.0108
48	15	34	47	15	33	A	0	305571.233	-0.088	344.9292
66	3	63	66	2	64	E	1	305720.073	-0.598	600.4226
66	4	63	66	3	64	E	1	305720.073	-0.598	600.4226
63	28	36	63	27	36	E	0	305753.385	-0.174	622.2140
31	11	21	30	10	20	A	0	305764.391	-0.030	183.0208
63	28	35	63	27	36	A	0	305782.006	0.003	622.2239
63	28	36	63	27	37	A	0	305782.006	0.003	622.2239
63	28	35	63	27	37	E	0	305799.522	-0.104	622.2126
49	21	29	48	21	28	A	0	305891.243	-0.038	393.9852
49	21	28	48	21	27	A	0	305891.243	-0.038	393.9852
49	21	28	48	21	27	E	0	305894.096	0.053	393.9818
48	15	33	47	15	32	A	0	306161.670	0.000	344.9557
49	10	39	48	10	38	A	0	306170.338	-0.322	336.9219
21	16	5	20	15	6	A	0	306315.914	-0.021	153.2674
21	16	6	20	15	5	A	0	306315.914	-0.021	153.2674
62	28	34	62	27	35	A	0	306336.466	-0.015	609.1107
62	28	35	62	27	36	A	0	306336.466	-0.015	609.1107
47	11	36	46	11	35	E	0	306778.163	-0.026	320.4835
47	11	36	46	11	35	A	0	306793.326	-0.006	320.4769
48	14	35	47	14	34	A	0	307059.968	-0.042	340.0732
23	15	9	22	14	8	A	0	307117.831	-0.013	156.6151
23	15	8	22	14	9	A	0	307117.831	-0.013	156.6151
56	14	42	55	15	41	E	0	307183.469	-0.040	432.8612
48	13	36	47	13	35	A	0	307205.158	0.035	335.6437
49	19	31	48	19	30	A	0	307290.986	0.023	379.2378
31	11	20	30	10	21	A	0	307335.486	-0.005	182.9773
60	28	32	60	27	33	A	0	307360.705	-0.020	583.5317
60	28	33	60	27	34	A	0	307360.705	-0.020	583.5317
49	11	39	48	10	38	E	0	307740.988	0.098	336.9264
49	11	39	48	10	38	A	0	307757.684	-0.066	336.9219
25	14	12	24	13	11	A	0	307803.458	-0.011	161.2160
59	28	32	59	27	32	E	0	307805.633	-0.209	571.0547
59	28	31	59	27	32	A	0	307832.972	-0.022	571.0650
59	28	32	59	27	33	A	0	307832.972	-0.022	571.0650
59	28	31	59	27	33	E	0	307852.328	-0.122	571.0535
29	12	18	28	11	17	A	0	308082.158	-0.063	174.3267
29	12	17	28	11	18	A	0	308117.578	0.039	174.3256
49	18	32	48	18	31	A	0	308216.165	-0.064	372.5096
49	18	31	48	18	30	A	0	308217.665	-0.095	372.5097
65	2	63	65	1	64	E	0	308226.081	0.043	450.1792
65	3	63	65	2	64	E	0	308226.081	0.043	450.1792
27	13	15	26	12	14	A	0	308234.104	0.170	167.1014
65	2	63	65	1	64	A	0	308275.698	0.176	450.1700
65	3	63	65	2	64	A	0	308275.698	0.176	450.1700
58	28	30	58	27	31	A	0	308280.249	-0.007	558.8130
58	28	31	58	27	32	A	0	308280.249	-0.007	558.8130
47	13	34	46	13	33	A	0	308585.232	-0.075	326.1798
57	28	29	57	27	30	A	0	308703.643	0.027	546.7754
57	28	30	57	27	31	A	0	308703.643	0.027	546.7754
53	7	46	52	7	45	A	1	309038.270	-0.027	488.9741
53	7	46	52	8	45	A	1	309038.270	0.147	488.9741
53	8	46	52	7	45	A	1	309038.270	-0.119	488.9741

J'	K'_a	K'_c	J''	K''_a	K''_c	Sym.	$v_{t_{CH_3}}$	$\nu_{Obs.}$	$\nu_{Obs.} - \nu_{Calc.}$	E_{low}
53	8	46	52	8	45	A	1	309038.270	0.056	488.9741
50	11	39	49	12	38	A	0	309052.208	-0.021	351.9099
54	6	48	53	6	47	A	1	309065.652	-0.067	492.3800
54	6	48	53	7	47	A	1	309065.652	-0.062	492.3800
54	7	48	53	6	47	A	1	309065.652	-0.069	492.3800
54	7	48	53	7	47	A	1	309065.652	-0.065	492.3800
56	28	28	56	27	29	A	0	309104.110	-0.013	534.9516
56	28	29	56	27	30	A	0	309104.110	-0.013	534.9516
52	8	44	51	9	43	A	1	309135.369	0.017	485.0743
52	9	44	51	8	43	A	1	309142.903	0.048	485.0741
55	5	50	54	5	49	A	1	309169.920	-0.059	495.3209
55	5	50	54	6	49	A	1	309169.920	-0.059	495.3209
55	6	50	54	5	49	A	1	309169.920	-0.059	495.3209
55	6	50	54	6	49	A	1	309169.920	-0.059	495.3209
53	7	46	52	7	45	E	1	309192.444	-0.064	488.8438
53	7	46	52	8	45	E	1	309192.444	0.149	488.8438
53	8	46	52	7	45	E	1	309192.444	-0.177	488.8438
53	8	46	52	8	45	E	1	309192.444	0.036	488.8438
54	6	48	53	6	47	E	1	309202.201	-0.016	492.2357
54	6	48	53	7	47	E	1	309202.201	-0.010	492.2357
54	7	48	53	6	47	E	1	309202.201	-0.019	492.2357
54	7	48	53	7	47	E	1	309202.201	-0.013	492.2357
53	7	46	52	7	45	E	0	309250.244	-0.057	360.5435
53	8	46	52	8	45	E	0	309250.244	0.035	360.5435
53	7	46	52	7	45	A	0	309255.023	-0.051	360.5394
53	7	46	52	8	45	A	0	309255.023	0.146	360.5394
53	8	46	52	7	45	A	0	309255.023	-0.155	360.5394
53	8	46	52	8	45	A	0	309255.023	0.042	360.5394
54	6	48	53	6	47	E	0	309262.950	-0.025	363.9358
54	7	48	53	7	47	E	0	309262.950	-0.023	363.9358
54	6	48	53	6	47	A	0	309267.180	-0.015	363.9312
54	6	48	53	7	47	A	0	309267.180	-0.010	363.9312
54	7	48	53	6	47	A	0	309267.180	-0.018	363.9312
54	7	48	53	7	47	A	0	309267.180	-0.012	363.9312
55	5	50	54	5	49	E	1	309289.822	0.052	495.1570
55	5	50	54	6	49	E	1	309289.822	0.052	495.1570
55	6	50	54	5	49	E	1	309289.822	0.052	495.1570
55	6	50	54	6	49	E	1	309289.822	0.052	495.1570
52	8	44	51	9	43	E	1	309309.795	0.004	484.9521
52	9	44	51	9	43	E	1	309313.035	0.059	484.9521
52	8	44	51	8	43	E	1	309315.602	0.017	484.9519
52	9	44	51	8	43	E	1	309318.742	-0.028	484.9519
56	4	52	55	4	51	A	1	309321.944	-0.112	497.8173
56	4	52	55	5	51	A	1	309321.944	-0.112	497.8173
56	5	52	55	4	51	A	1	309321.944	-0.112	497.8173
56	5	52	55	5	51	A	1	309321.944	-0.112	497.8173
55	5	50	54	5	49	E	0	309352.743	0.015	366.8590
55	6	50	54	6	49	E	0	309352.743	0.015	366.8590
55	5	50	54	5	49	A	0	309356.431	0.007	366.8538
55	5	50	54	6	49	A	0	309356.431	0.007	366.8538
55	6	50	54	5	49	A	0	309356.431	0.007	366.8538
55	6	50	54	6	49	A	0	309356.431	0.007	366.8538
49	17	33	48	17	32	A	0	309364.759	-0.056	366.2317
52	8	44	51	9	43	A	0	309368.604	-0.024	356.6493
52	8	44	51	8	43	E	0	309368.604	0.019	356.6530
52	9	44	51	9	43	A	0	309371.548	-0.037	356.6493
52	8	44	51	8	43	A	0	309374.056	0.035	356.6492
52	9	44	51	8	43	A	0	309377.001	0.024	356.6492
49	17	32	48	17	31	E	0	309377.001	-0.091	366.2352
49	17	32	48	17	31	A	0	309380.996	-0.030	366.2322
51	9	42	50	10	41	A	1	309391.856	0.117	480.6374
56	4	52	55	4	51	E	1	309424.675	-0.054	497.6280
56	4	52	55	5	51	E	1	309424.675	-0.054	497.6280
56	5	52	55	4	51	E	1	309424.675	-0.054	497.6280
56	5	52	55	5	51	E	1	309424.675	-0.054	497.6280
55	28	27	55	27	28	A	0	309482.768	-0.012	523.3415
55	28	28	55	27	29	A	0	309482.768	-0.012	523.3415
56	4	52	55	4	51	E	0	309489.694	0.008	369.3336
56	5	52	55	5	51	E	0	309489.694	0.008	369.3336
56	4	52	55	4	51	A	0	309492.859	0.011	369.3276
56	4	52	55	5	51	A	0	309492.859	0.011	369.3276
56	5	52	55	4	51	A	0	309492.859	0.011	369.3276
56	5	52	55	5	51	A	0	309492.859	0.011	369.3276
57	3	54	56	3	53	A	1	309504.477	-0.065	499.8846
57	3	54	56	4	53	A	1	309504.477	-0.065	499.8846
57	4	54	56	3	53	A	1	309504.477	-0.065	499.8846
57	4	54	56	4	53	A	1	309504.477	-0.065	499.8846
51	10	42	50	9	41	A	1	309549.432	0.134	480.6340
51	9	42	50	10	41	E	1	309582.878	0.069	480.5169
57	3	54	56	3	53	E	1	309588.604	-0.001	499.6637
57	3	54	56	4	53	E	1	309588.604	-0.001	499.6637
57	4	54	56	3	53	E	1	309588.604	-0.001	499.6637
57	4	54	56	4	53	E	1	309588.604	-0.001	499.6637
51	9	42	50	10	41	A	0	309639.248	0.006	352.2178
51	10	42	50	10	41	E	1	309650.096	0.055	480.5169
57	3	54	56	3	53	E	0	309655.770	0.005	371.3745
57	4	54	56	4	53	E	0	309655.770	0.005	371.3745
57	3	54	56	3	53	A	0	309658.356	0.008	371.3675
57	3	54	56	4	53	A	0	309658.356	0.008	371.3675
57	4	54	56	3	53	A	0	309658.356	0.008	371.3675
57	4	54	56	4	53	A	0	309658.356	0.008	371.3675
70	5	65	70	4	66	E	1	309685.742	-0.309	670.3825
70	6	65	70	5	66	E	1	309685.742	-0.309	670.3825
51	9	42	50	9	41	E	1	309699.393	-0.045	480.5130
51	10	42	50	10	41	A	0	309702.184	-0.045	352.2178
58	2	56	57	2	55	A	1	309706.518	-0.046	501.5346
58	2	56	57	3	55	A	1	309706.518	-0.046	501.5346
58	3	56	57	2	55	A	1	309706.518	-0.046	501.5346
58	3	56	57	3	55	A	1	309706.518	-0.046	501.5346
51	9	42	50	9	41	A	0	309748.744	-0.059	352.2141
58	2	56	57	2	55	E	1	309769.598	0.038	501.2751
58	3	56	57	3	55	E	1	309769.598	0.038	501.2751
51	10	42	50	9	41	A	0	309811.781	-0.009	352.2141
58	2	56	57	2	55	E	0	309839.473	0.003	372.9929
58	3	56	57	3	55	E	0	309839.473	0.003	372.9929
58	2	56	57	2	55	A	0	309841.432	0.029	372.9848
58	2	56	57	3	55	A	0	309841.432	0.029	372.9848
58	3	56	57	2	55	A	0	309841.432	0.029	372.9848
58	3	56	57	3	55	A	0	309841.432	0.029	372.9848
59	1	58	58	1	57	A	1	309921.234	0.026	502.7765
59	1	58	58	2	57	A	1	309921.234	0.026	502.7765
59	2	58	58	1	57	A	1	309921.234	0.026	502.7765
59	2	58	58	2	57	A	1	309921.234	0.026	502.7765
59	1	58	58	1	57	E	1	309959.682	-0.027	502.4709
59	2	58	58	2	57	E	1	309959.682	-0.027	502.4709
59	1	58	58	1	57	E	0	310033.229	-0.020	374.1978

J'	K'_a	K'_c	J''	K''_a	K''_c	Sym.	$v_{t_{CH_3}}$	$\nu_{Obs.}$	$\nu_{Obs.} - \nu_{Calc.}$	E_{low}
59	2	58	58	2	57	E	0	310033.229	-0.020	374.1978
59	1	58	58	1	57	A	0	310034.526	0.096	374.1883
59	1	58	58	2	57	A	0	310034.526	0.096	374.1883
59	2	58	58	1	57	A	0	310034.526	0.096	374.1883
59	2	58	58	2	57	A	0	310034.526	0.096	374.1883
60	0	60	59	0	59	A	1	310144.245	0.151	503.6183
60	0	60	59	1	59	A	1	310144.245	0.151	503.6183
60	1	60	59	0	59	A	1	310144.245	0.151	503.6183
60	1	60	59	1	59	A	1	310144.245	0.151	503.6183
60	0	60	59	0	59	E	1	310153.698	0.068	503.2578
60	1	60	59	1	59	E	1	310153.698	0.068	503.2578
53	28	25	53	27	26	A	0	310178.296	-0.041	500.7605
53	28	26	53	27	27	A	0	310178.296	-0.041	500.7605
50	11	40	49	11	39	E	1	310215.517	0.047	475.4810
60	0	60	59	0	59	A	0	310232.191	-0.138	374.9851
60	0	60	59	1	59	A	0	310232.191	-0.138	374.9851
60	1	60	59	0	59	A	0	310232.191	-0.138	374.9851
60	1	60	59	1	59	A	0	310232.191	-0.138	374.9851
60	0	60	59	0	59	E	0	310232.191	0.165	374.9962
60	1	60	59	1	59	E	0	310232.191	0.165	374.9962
48	14	34	47	14	33	A	0	310251.418	-0.089	340.2522
32	11	22	31	10	21	A	0	310354.165	-0.015	189.6117
52	28	24	52	27	25	A	0	310496.968	-0.066	489.7889
52	28	25	52	27	26	A	0	310496.968	-0.066	489.7889
18	18	0	17	17	1	A	0	310744.830	-0.021	153.7483
18	18	1	17	17	0	A	0	310744.830	-0.021	153.7483
51	28	23	51	27	24	A	0	310797.452	-0.025	479.0297
51	28	24	51	27	25	A	0	310797.452	-0.025	479.0297
49	16	34	48	16	33	A	0	310803.815	-0.113	360.4269
48	11	37	47	11	36	E	0	310852.203	-0.102	330.7165
50	10	40	49	10	39	E	1	310860.604	-0.121	475.4253
50	24	26	49	24	25	A	0	310871.488	-0.042	429.3592
50	24	27	49	24	26	A	0	310871.488	-0.042	429.3592
50	10	40	49	10	39	A	0	310878.437	-0.020	347.1346
48	11	37	47	11	36	E	1	310930.141	0.167	458.9782
49	16	33	48	16	32	A	0	310945.501	-0.022	360.4326
50	28	22	50	27	23	A	0	311080.458	-0.018	468.4823
50	28	23	50	27	24	A	0	311080.458	-0.018	468.4823
50	23	28	49	23	27	A	0	311305.099	-0.052	420.5618
50	23	27	49	23	26	A	0	311305.099	-0.052	420.5618
50	23	28	49	23	27	E	0	311307.415	0.158	420.5488
49	28	21	49	27	22	A	0	311346.760	-0.047	458.1465
49	28	22	49	27	23	A	0	311346.760	-0.047	458.1465
69	4	65	69	3	66	A	1	311454.096	-0.028	647.4242
69	5	65	69	4	66	A	1	311454.096	-0.028	647.4242
48	28	20	48	27	21	A	0	311597.136	-0.082	448.0221
48	28	21	48	27	22	A	0	311597.136	-0.082	448.0221
20	17	4	19	16	3	A	0	311609.057	-0.023	155.0564
20	17	3	19	16	4	A	0	311609.057	-0.023	155.0564
50	22	28	49	22	27	A	0	311804.518	-0.039	412.1699
50	22	29	49	22	28	A	0	311804.518	-0.039	412.1699
47	28	19	47	27	20	A	0	311832.386	-0.043	438.1086
47	28	20	47	27	21	A	0	311832.386	-0.043	438.1086
50	11	40	49	10	39	A	0	311850.017	-0.021	347.1346
46	28	18	46	27	19	A	0	312053.090	-0.040	428.4059
46	28	19	46	27	20	A	0	312053.090	-0.040	428.4059
45	28	17	45	27	18	A	0	312259.940	-0.050	418.9137
45	28	18	45	27	19	A	0	312259.940	-0.050	418.9137
50	21	30	49	21	29	A	0	312385.647	-0.051	404.1886
50	21	29	49	21	28	A	0	312385.647	-0.052	404.1886
50	21	29	49	21	28	E	0	312388.678	0.040	404.1854
22	16	6	21	15	6	E	0	312404.753	-0.049	157.5942
22	16	7	21	15	7	E	0	312414.780	0.017	157.5806
44	28	17	44	27	17	E	0	312429.605	-0.024	409.6203
22	16	6	21	15	7	A	0	312449.985	-0.021	157.5892
22	16	7	21	15	6	A	0	312449.985	-0.021	157.5892
44	28	16	44	27	17	A	0	312453.546	-0.105	409.6316
44	28	17	44	27	18	A	0	312453.546	-0.105	409.6316
44	28	16	44	27	18	E	0	312477.253	0.090	409.6196
49	15	35	48	15	34	A	0	312480.306	-0.016	355.1220
49	15	35	48	15	34	E	0	312495.929	0.006	355.1218
43	28	16	43	27	16	E	0	312610.784	-0.060	400.5481
43	28	15	43	27	17	E	0	312658.359	-0.039	400.5475
69	4	65	69	3	66	A	0	312673.565	0.011	518.9771
69	5	65	69	4	66	A	0	312673.565	0.011	518.9771
42	28	15	42	27	15	E	0	312780.064	-0.010	391.6856
42	28	14	42	27	15	A	0	312803.775	-0.064	391.6971
42	28	15	42	27	16	A	0	312803.775	-0.064	391.6971
41	28	14	41	27	14	E	0	312937.854	-0.042	383.0326
41	28	13	41	27	14	A	0	312961.307	-0.238	383.0441
41	28	14	41	27	15	A	0	312961.307	-0.238	383.0441
41	28	13	41	27	15	E	0	312985.499	0.019	383.0320
50	20	31	49	20	30	A	0	313070.387	-0.040	396.6249
40	28	13	40	27	13	E	0	313084.867	-0.002	374.5888
40	28	12	40	27	13	A	0	313108.379	-0.029	374.6003
40	28	13	40	27	14	A	0	313108.379	-0.029	374.6003
24	15	9	23	14	9	E	0	313184.974	-0.064	161.3671
24	15	10	23	14	10	E	0	313189.312	-0.009	161.3534
32	11	21	31	10	22	A	0	313199.605	0.042	189.5338
39	28	12	39	27	12	E	0	313221.542	0.008	366.3539
24	15	10	23	14	9	A	0	313228.176	-0.008	161.3606
24	15	9	23	14	10	A	0	313228.176	-0.008	161.3606
32	11	21	31	10	22	E	0	313230.666	-0.062	189.5391
39	28	11	39	27	12	A	0	313244.941	-0.028	366.3654
39	28	12	39	27	13	A	0	313244.941	-0.028	366.3654
39	28	11	39	27	13	E	0	313269.280	0.144	366.3534
38	28	11	38	27	11	E	0	313348.379	-0.033	358.3278
49	13	37	48	13	36	A	0	313358.152	0.129	345.8910
49	13	37	48	13	36	E	0	313358.152	-0.146	345.8944
38	28	10	38	27	11	A	0	313371.677	-0.072	358.3393
38	28	11	38	27	12	A	0	313371.677	-0.072	358.3393
38	28	10	38	27	12	E	0	313396.031	0.012	358.3272
49	15	34	48	15	33	A	0	313453.868	-0.169	355.1681
49	15	34	48	15	33	E	0	313453.868	0.156	355.1695
37	28	10	37	27	10	E	0	313465.898	-0.109	350.5102
37	28	9	37	27	10	A	0	313489.201	-0.054	350.5217
37	28	10	37	27	11	A	0	313489.201	-0.054	350.5217
37	28	9	37	27	11	E	0	313513.650	0.032	350.5096
36	28	9	36	27	9	E	0	313574.811	0.003	342.9009
36	28	8	36	27	9	A	0	313597.901	-0.070	342.9124
36	28	9	36	27	10	A	0	313597.901	-0.070	342.9124
36	28	8	36	27	10	E	0	313622.418	-0.001	342.9003
35	28	8	35	27	8	E	0	313675.236	-0.049	335.4996
35	28	7	35	27	8	A	0	313698.344	-0.025	335.5112
35	28	8	35	27	9	A	0	313698.344	-0.025	335.5112
35	28	7	35	27	9	E	0	313722.881	-0.014	335.4991

J'	K'_a	K'_c	J''	K''_a	K''_c	Sym.	$v_{t_{CH_3}}$	$\nu_{Obs.}$	$\nu_{Obs.} - \nu_{Calc.}$	E_{low}
34	28	6	34	27	7	A	0	313790.918	0.014	328.3179
34	28	7	34	27	8	A	0	313790.918	0.014	328.3179
49	14	36	48	14	35	A	0	313793.567	-0.095	350.3156
49	14	36	48	14	35	E	0	313799.358	-0.067	350.3176
30	12	19	29	11	19	E	0	313811.069	0.003	180.4010
34	28	6	34	27	8	E	0	313815.550	0.049	328.3058
30	12	19	29	11	18	A	0	313819.294	-0.022	180.4043
30	12	18	29	11	18	E	0	313821.424	-0.068	180.4136
26	14	13	25	13	13	E	0	313822.735	0.043	166.3864
26	14	12	25	13	12	E	0	313824.332	-0.070	166.3999
70	29	42	70	28	42	E	0	313828.221	-0.012	730.1295
33	28	6	33	27	6	E	0	313853.015	-0.060	321.3207
70	29	42	70	28	43	A	0	313854.280	0.039	730.1393
70	29	41	70	28	42	A	0	313854.280	0.039	730.1393
26	14	13	25	13	12	A	0	313864.816	-0.017	166.3921
70	29	41	70	28	43	E	0	313873.486	-0.116	730.1291
33	28	5	33	27	6	A	0	313875.967	-0.049	321.3323
33	28	6	33	27	7	A	0	313875.967	-0.049	321.3323
50	19	32	49	19	31	A	0	313889.875	0.080	389.4880
50	19	31	49	19	30	E	0	313893.210	-0.080	389.4880
30	12	18	29	11	19	A	0	313894.864	-0.082	180.4021
33	28	5	33	27	7	E	0	313900.575	-0.103	321.3202
32	28	5	32	27	5	E	0	313931.365	0.111	314.5426
32	28	4	32	27	5	A	0	313954.118	-0.012	314.5542
32	28	5	32	27	6	A	0	313954.118	-0.012	314.5542
32	28	4	32	27	6	E	0	313978.936	0.087	314.5421
31	28	4	31	27	4	E	0	314002.865	0.025	307.9718
31	28	3	31	27	4	A	0	314025.563	-0.092	307.9834
31	28	4	31	27	5	A	0	314025.563	-0.092	307.9834
31	28	3	31	27	5	E	0	314050.512	0.086	307.9713
54	7	47	53	7	46	A	1	314140.613	-0.018	499.2825
54	7	47	53	8	46	A	1	314140.613	0.073	499.2825
54	8	47	53	7	46	A	1	314140.613	-0.066	499.2825
54	8	47	53	8	46	A	1	314140.613	0.025	499.2825
28	13	16	27	12	16	E	0	314152.194	-0.005	172.7149
28	13	15	27	12	15	E	0	314160.372	-0.056	172.7281
55	6	49	54	6	48	A	1	314178.772	-0.073	502.6893
55	6	49	54	7	48	A	1	314178.772	-0.071	502.6893
55	7	49	54	6	48	A	1	314178.772	-0.074	502.6893
55	7	49	54	7	48	A	1	314178.772	-0.072	502.6893
28	13	16	27	12	15	A	0	314197.451	0.438	172.7189
28	13	15	27	12	16	A	0	314197.451	-0.478	172.7189
53	8	45	52	9	44	A	1	314218.404	0.103	495.3860
53	9	45	52	9	44	A	1	314219.763	0.023	495.3860
53	8	45	52	8	44	A	1	314221.070	0.118	495.3859
53	9	45	52	8	44	A	1	314222.437	0.046	495.3859
33	11	23	32	10	22	E	0	314239.288	-0.050	196.4541
56	5	51	55	5	50	A	1	314288.656	-0.083	505.6337
56	5	51	55	6	50	A	1	314288.656	-0.083	505.6337
56	6	51	55	5	50	A	1	314288.656	-0.084	505.6337
56	6	51	55	6	50	A	1	314288.656	-0.083	505.6337
54	7	47	53	7	46	E	1	314292.589	-0.032	499.1573
54	7	47	53	8	46	E	1	314292.589	0.081	499.1573
54	8	47	53	7	46	E	1	314292.589	-0.092	499.1573
54	8	47	53	8	46	E	1	314292.589	0.021	499.1573
55	6	49	54	6	48	E	1	314313.826	-0.019	502.5495
55	6	49	54	7	48	E	1	314313.826	-0.016	502.5495
55	7	49	54	6	48	E	1	314313.826	-0.021	502.5495
55	7	49	54	7	48	E	1	314313.826	-0.018	502.5495
33	11	23	32	10	22	A	0	314326.577	-0.026	196.4477
54	7	47	53	7	46	E	0	314352.887	-0.023	370.8590
54	8	47	53	8	46	E	0	314352.887	0.026	370.8590
54	7	47	53	7	46	A	0	314357.593	-0.022	370.8550
54	7	47	53	8	46	A	0	314357.593	0.082	370.8550
54	8	47	53	7	46	A	0	314357.593	-0.077	370.8550
54	8	47	53	8	46	A	0	314357.593	0.027	370.8550
55	6	49	54	6	48	E	0	314376.779	-0.007	374.2517
55	7	49	54	7	48	E	0	314376.779	-0.005	374.2517
55	6	49	54	6	48	A	0	314380.948	-0.011	374.2472
55	6	49	54	7	48	A	0	314380.948	-0.008	374.2472
55	7	49	54	6	48	A	0	314380.948	-0.012	374.2472
55	7	49	54	7	48	A	0	314380.948	-0.010	374.2472
53	8	45	52	9	44	E	1	314389.682	0.005	495.2696
53	9	45	52	9	44	E	1	314391.435	0.017	495.2696
53	8	45	52	8	44	E	1	314392.921	0.059	495.2695
53	9	45	52	8	44	E	1	314394.888	0.286	495.2695
56	5	51	55	5	50	E	1	314407.519	-0.013	505.4738
56	5	51	55	6	50	E	1	314407.519	-0.013	505.4738
56	6	51	55	5	50	E	1	314407.519	-0.013	505.4738
56	6	51	55	6	50	E	1	314407.519	-0.013	505.4738
57	4	53	56	4	52	A	1	314443.632	-0.115	508.1352
57	4	53	56	5	52	A	1	314443.632	-0.115	508.1352
57	5	53	56	4	52	A	1	314443.632	-0.115	508.1352
57	5	53	56	5	52	A	1	314443.632	-0.115	508.1352
53	9	45	52	9	44	A	0	314452.838	-0.046	366.9689
53	8	45	52	8	44	A	0	314454.270	0.040	366.9688
53	9	45	52	8	44	A	0	314455.830	-0.011	366.9688
52	9	43	51	10	42	A	1	314462.355	0.103	490.9595
56	5	51	55	5	50	E	0	314472.447	0.000	377.1779
56	6	51	55	6	50	E	0	314472.447	0.000	377.1779
56	5	51	55	5	50	A	0	314476.122	0.010	377.1729
56	5	51	55	6	50	A	0	314476.122	0.010	377.1729
56	6	51	55	5	50	A	0	314476.122	0.010	377.1729
56	6	51	55	6	50	A	0	314476.122	0.010	377.1729
52	9	43	51	9	42	A	1	314519.799	0.152	490.9576
57	4	53	56	4	52	E	1	314545.882	0.096	507.9493
57	4	53	56	5	52	E	1	314545.882	0.096	507.9493
57	5	53	56	4	52	E	1	314545.882	0.096	507.9493
57	5	53	56	5	52	E	1	314545.882	0.096	507.9493
52	10	43	51	9	42	A	1	314552.392	0.139	490.9576
57	4	53	56	4	52	E	0	314612.530	0.006	379.6571
57	5	53	56	5	52	E	0	314612.530	0.006	379.6571
57	4	53	56	4	52	A	0	314615.676	0.010	379.6512
57	4	53	56	5	52	A	0	314615.676	0.010	379.6512
57	5	53	56	4	52	A	0	314615.676	0.010	379.6512
57	5	53	56	5	52	A	0	314615.676	0.010	379.6512
58	3	55	57	3	54	A	1	314627.633	-0.083	510.2086
58	3	55	57	4	54	A	1	314627.633	-0.083	510.2086
58	4	55	57	3	54	A	1	314627.633	-0.083	510.2086
58	4	55	57	4	54	A	1	314627.633	-0.083	510.2086
52	9	43	51	10	42	E	1	314653.025	0.097	490.8457
52	10	43	51	10	42	E	1	314691.334	-0.016	490.8457
33	10	23	32	9	24	E	0	314702.798	-0.005	193.0466
52	9	43	51	10	42	E	0	314705.061	-0.003	362.5519
52	9	43	51	9	42	E	1	314720.135	-0.024	490.8435
52	10	43	51	10	42	E	0	314740.761	-0.009	362.5519

J'	K'_a	K'_c	J''	K''_a	K''_c	Sym.	$v_{t_{CH_3}}$	$\nu_{Obs.}$	$\nu_{Obs.} - \nu_{Calc.}$	E_{low}
52	10	43	51	10	42	A	0	314746.816	-0.043	362.5483
52	10	43	51	9	42	E	1	314758.614	0.033	490.8435
52	9	43	51	9	42	E	0	314767.683	-0.037	362.5498
52	9	43	51	9	42	A	0	314773.973	0.029	362.5462
58	3	55	57	3	54	E	0	314780.216	0.006	381.7035
58	4	55	57	4	54	E	0	314780.216	0.006	381.7035
58	3	55	57	3	54	A	0	314782.796	0.014	381.6966
58	3	55	57	4	54	A	0	314782.796	0.014	381.6966
58	4	55	57	3	54	A	0	314782.796	0.014	381.6966
58	4	55	57	4	54	A	0	314782.796	0.014	381.6966
52	10	43	51	9	42	E	0	314803.368	-0.058	362.5498
52	10	43	51	9	42	A	0	314809.841	-0.004	362.5462
49	11	38	48	11	37	E	0	314826.705	-0.016	341.0854
59	2	57	58	2	56	A	1	314830.419	-0.046	511.8653
59	2	57	58	3	56	A	1	314830.419	-0.046	511.8653
59	3	57	58	2	56	A	1	314830.419	-0.046	511.8653
59	3	57	58	3	56	A	1	314830.419	-0.046	511.8653
49	11	38	48	11	37	A	0	314842.851	-0.090	341.0799
50	18	33	49	18	32	A	0	314889.460	0.014	382.7906
59	2	57	58	2	56	E	1	314893.318	0.039	511.6079
59	2	57	58	3	56	E	1	314893.318	0.039	511.6079
59	3	57	58	2	56	E	1	314893.318	0.039	511.6079
59	3	57	58	3	56	E	1	314893.318	0.039	511.6079
50	18	33	49	18	32	E	0	314896.447	-0.056	382.7808
59	2	57	58	2	56	E	0	314964.730	0.024	383.3281
59	3	57	58	3	56	E	0	314964.730	0.024	383.3281
59	2	57	58	2	56	A	0	314966.648	0.015	383.3200
59	2	57	58	3	56	A	0	314966.648	0.015	383.3200
59	3	57	58	2	56	A	0	314966.648	0.015	383.3200
59	3	57	58	3	56	A	0	314966.648	0.015	383.3200
51	11	41	50	11	40	A	1	315028.710	0.191	485.9465
60	1	59	59	1	58	A	1	315045.493	0.012	513.1144
60	1	59	59	2	58	A	1	315045.493	0.012	513.1144
60	2	59	59	1	58	A	1	315045.493	0.012	513.1144
60	2	59	59	2	58	A	1	315045.493	0.012	513.1144
60	2	59	59	2	58	E	1	315083.878	-0.019	512.8101
60	1	59	59	1	58	E	0	315158.792	-0.066	384.5394
60	2	59	59	2	58	E	0	315158.792	-0.066	384.5394
60	1	59	59	1	58	A	0	315160.068	0.031	384.5300
60	1	59	59	2	58	A	0	315160.068	0.031	384.5300
60	2	59	59	1	58	A	0	315160.068	0.031	384.5300
60	2	59	59	2	58	A	0	315160.068	0.031	384.5300
61	0	61	60	0	60	A	1	315268.781	0.161	513.9636
61	0	61	60	1	60	A	1	315268.781	0.161	513.9636
61	1	61	60	0	60	A	1	315268.781	0.161	513.9636
61	1	61	60	1	60	A	1	315268.781	0.161	513.9636
61	0	61	60	0	60	E	1	315278.128	0.057	513.6034
61	1	61	60	1	60	E	1	315278.128	0.057	513.6034
51	11	41	50	11	40	A	0	315298.218	-0.009	357.5368
61	0	61	60	0	60	A	0	315357.986	-0.135	385.3333
61	0	61	60	1	60	A	0	315357.986	-0.135	385.3333
61	1	61	60	0	60	A	0	315357.986	-0.135	385.3333
61	1	61	60	1	60	A	0	315357.986	-0.135	385.3333
61	0	61	60	0	60	E	0	315357.986	0.165	385.3445
61	1	61	60	1	60	E	0	315357.986	0.165	385.3445
51	30	21	50	30	20	A	0	315435.559	0.036	500.7877
51	30	22	50	30	21	A	0	315435.559	0.036	500.7877
51	30	21	50	30	20	E	0	315435.559	-0.168	500.7786
51	30	22	50	30	21	E	0	315436.987	0.170	500.7747
68	3	65	68	2	66	A	0	315481.950	0.032	496.0738
68	4	65	68	3	66	A	0	315481.950	0.032	496.0738
54	13	41	53	14	40	E	0	315528.050	-0.063	404.8129
50	12	39	49	12	38	E	0	315562.917	-0.010	351.9137
50	12	39	49	12	38	A	0	315568.283	-0.030	351.9099
51	29	23	50	29	22	A	0	315665.197	0.095	489.6278
51	29	22	50	29	21	A	0	315665.197	0.095	489.6278
51	29	22	50	29	21	E	0	315665.197	-0.295	489.6176
51	29	23	50	29	22	E	0	315666.563	-0.014	489.6153
51	10	41	50	10	40	E	0	315674.506	0.001	357.5083
51	10	41	50	10	40	A	0	315682.877	0.020	357.5044
52	12	40	51	13	39	A	0	315825.911	-0.039	377.8392
67	29	39	67	28	39	E	0	315838.584	-0.112	686.9496
67	29	39	67	28	40	A	0	315863.361	-0.050	686.9599
67	29	38	67	28	39	A	0	315863.361	-0.050	686.9599
50	17	34	49	17	33	A	0	316134.376	-0.046	376.5510
50	17	34	49	17	33	E	0	316154.304	-0.076	376.5430
50	17	33	49	17	32	E	0	316155.546	0.063	376.5549
50	17	33	49	17	32	A	0	316164.662	-0.042	376.5521
51	11	41	50	10	40	E	0	316258.737	-0.012	357.5083
51	11	41	50	10	40	A	0	316269.712	-0.096	357.5044
66	29	38	66	28	38	E	0	316444.304	-0.045	672.9909
66	29	38	66	28	39	A	0	316468.678	0.011	673.0014
66	29	37	66	28	38	A	0	316468.678	0.011	673.0014
66	29	37	66	28	39	E	0	316490.233	-0.106	672.9908
51	26	25	50	26	24	A	0	316519.023	-0.088	458.5001
51	26	26	50	26	25	A	0	316519.023	-0.088	458.5001
51	11	40	50	12	39	A	0	316568.443	-0.051	362.4361
51	11	40	50	12	39	E	0	316578.809	-0.104	362.4398
19	18	1	18	17	1	E	0	316843.776	-0.068	157.4439
19	18	2	18	17	2	E	0	316864.379	0.037	157.4312
51	25	27	50	25	26	A	0	316875.887	0.027	448.9147
51	25	26	50	25	25	A	0	316875.887	0.027	448.9147
19	18	1	18	17	2	A	0	316891.190	-0.018	157.4420
19	18	2	18	17	1	A	0	316891.190	-0.018	157.4420
48	13	35	47	13	34	A	0	316913.307	-0.026	336.4731
48	13	35	47	13	34	E	0	316926.654	-0.023	336.4783
65	29	37	65	28	37	E	0	317019.964	-0.156	659.2489
65	29	37	65	28	38	A	0	317044.060	0.002	659.2594
65	29	36	65	28	37	A	0	317044.060	0.002	659.2594
51	24	27	50	24	26	A	0	317280.391	-0.031	439.7287
51	24	28	50	24	27	A	0	317280.391	-0.031	439.7287
38	11	28	37	10	27	E	0	317292.437	-0.090	234.5613
34	11	24	33	10	23	E	0	317369.093	-0.207	203.5440
38	11	28	37	10	27	A	0	317374.862	0.006	234.5535
34	11	24	33	10	23	A	0	317444.210	0.016	203.5374
64	29	36	64	28	37	E	0	317567.250	-0.068	645.7230
70	4	66	70	3	67	E	0	317574.410	-0.042	531.5337
70	5	66	70	4	67	E	0	317574.410	-0.042	531.5337
64	29	36	64	28	37	A	0	317590.885	-0.006	645.7337
64	29	35	64	28	36	A	0	317590.885	-0.006	645.7337
50	16	35	49	16	34	A	0	317682.022	-0.043	370.7942
21	17	4	20	16	4	E	0	317704.338	-0.050	159.1702
50	16	35	49	16	34	E	0	317718.355	0.003	370.7906
21	17	5	20	16	5	E	0	317719.752	0.026	159.1570
21	17	5	20	16	4	A	0	317750.960	-0.027	159.1668
21	17	4	20	16	5	A	0	317750.960	-0.027	159.1668

J'	K'_a	K'_c	J''	K''_a	K''_c	Sym.	$v_{t_{CH_3}}$	$\nu_{Obs.}$	$\nu_{Obs.} - \nu_{Calc.}$	E_{low}
50	16	34	49	16	33	E	0	317906.157	0.017	370.8064
50	16	34	49	16	33	A	0	317929.350	-0.040	370.8046
63	29	35	63	28	36	E	0	318087.101	-0.082	632.4129
63	29	35	63	28	36	A	0	318110.386	-0.020	632.4237
63	29	34	63	28	35	A	0	318110.386	-0.020	632.4237
51	22	29	50	22	28	A	0	318276.065	-0.050	422.5706
51	22	30	50	22	29	A	0	318276.065	-0.049	422.5706
51	22	29	50	22	28	E	0	318278.965	-0.054	422.5658
49	14	35	48	14	34	A	0	318441.451	-0.043	350.6011
49	14	35	48	14	34	E	0	318455.580	-0.025	350.6042
23	16	7	22	15	7	E	0	318533.601	-0.059	162.1248
23	16	8	22	15	8	E	0	318543.562	0.008	162.1112
48	12	36	47	12	35	E	0	318567.553	0.005	333.6314
48	12	36	47	12	35	A	0	318571.776	-0.061	333.6246
23	16	7	22	15	8	A	0	318578.817	-0.024	162.1198
23	16	8	22	15	7	A	0	318578.817	-0.024	162.1198
62	29	34	62	28	35	A	0	318603.784	0.003	619.3290
62	29	33	62	28	34	A	0	318603.784	0.003	619.3290
62	29	33	62	28	34	E	0	318627.210	-0.125	619.3183
50	11	39	49	11	38	E	0	318869.713	0.007	351.5869
51	21	31	50	21	30	A	0	318897.751	-0.091	414.6087
51	21	30	50	21	29	A	0	318897.751	-0.093	414.6087
51	21	31	50	21	30	E	0	318901.081	-0.105	414.5964
61	29	33	61	28	34	E	0	319049.436	-0.133	606.4383
61	29	33	61	28	34	A	0	319072.128	-0.009	606.4493
61	29	32	61	28	33	A	0	319072.128	-0.009	606.4493
61	29	32	61	28	33	E	0	319096.031	-0.071	606.4385
55	7	48	54	7	47	A	1	319244.470	-0.049	509.7611
55	7	48	54	8	47	A	1	319244.470	-0.002	509.7611
55	8	48	54	7	47	A	1	319244.470	-0.074	509.7611
55	8	48	54	8	47	A	1	319244.470	-0.027	509.7611
25	15	10	24	14	10	E	0	319285.143	-0.054	166.3229
25	15	11	24	14	11	E	0	319289.392	0.028	166.3093
56	6	50	55	6	49	A	1	319292.496	-0.072	513.1692
56	6	50	55	7	49	A	1	319292.496	-0.071	513.1692
56	7	50	55	6	49	A	1	319292.496	-0.072	513.1692
56	7	50	55	7	49	A	1	319292.496	-0.071	513.1692
33	11	22	32	10	23	A	0	319300.322	0.034	196.3134
33	11	22	32	10	23	E	0	319302.917	-0.056	196.3190
54	8	46	53	9	45	A	1	319304.194	-0.002	505.8672
54	9	46	53	8	45	A	1	319306.456	0.044	505.8672
50	13	38	49	13	37	E	0	319322.006	-0.093	356.3469
50	13	38	49	13	37	A	0	319323.229	0.058	356.3435
25	15	11	24	14	10	A	0	319328.291	-0.015	166.3165
25	15	10	24	14	11	A	0	319328.291	-0.015	166.3165
37	11	27	36	10	26	E	0	319331.989	-0.065	226.4073
35	11	25	34	10	24	E	0	319378.220	-0.415	210.8962
50	15	36	49	15	35	A	0	319387.184	-0.036	365.5452
55	7	48	54	7	47	E	1	319394.362	-0.002	509.6410
55	7	48	54	8	47	E	1	319394.362	0.057	509.6410
55	8	48	54	7	47	E	1	319394.362	-0.033	509.6410
55	8	48	54	8	47	E	1	319394.362	0.026	509.6410
50	15	36	49	15	35	E	0	319398.675	-0.021	365.5455
57	5	52	56	5	51	A	1	319407.568	-0.091	516.1172
57	5	52	56	6	51	A	1	319407.568	-0.091	516.1172
57	6	52	56	5	51	A	1	319407.568	-0.091	516.1172
57	6	52	56	6	51	A	1	319407.568	-0.091	516.1172
56	6	50	55	6	49	E	1	319426.085	-0.020	513.0339
56	6	50	55	7	49	E	1	319426.085	-0.019	513.0339
56	7	50	55	6	49	E	1	319426.085	-0.021	513.0339
56	7	50	55	7	49	E	1	319426.085	-0.019	513.0339
35	11	25	34	10	24	A	0	319450.451	0.024	210.8893
55	7	48	54	7	47	E	0	319457.103	-0.021	381.3447
55	8	48	54	8	47	E	0	319457.103	0.005	381.3447
55	7	48	54	7	47	A	0	319461.752	-0.012	381.3409
55	7	48	54	8	47	A	0	319461.752	0.043	381.3409
55	8	48	54	7	47	A	0	319461.752	-0.041	381.3409
55	8	48	54	8	47	A	0	319461.752	0.014	381.3409
31	12	20	30	11	19	A	0	319464.908	-0.031	186.7043
54	8	46	53	9	45	E	1	319472.479	-0.063	505.7566
54	9	46	53	8	45	E	1	319475.294	0.066	505.7565
56	6	50	55	6	49	E	0	319491.217	-0.003	384.7381
56	7	50	55	7	49	E	0	319491.217	-0.002	384.7381
56	6	50	55	6	49	A	0	319495.352	0.004	384.7339
56	6	50	55	7	49	A	0	319495.352	0.005	384.7339
56	7	50	55	6	49	A	0	319495.352	0.003	384.7339
56	7	50	55	7	49	A	0	319495.352	0.004	384.7339
31	12	19	30	11	19	E	0	319498.942	-0.033	186.7128
31	12	20	30	11	20	E	0	319505.098	-0.012	186.6997
60	29	32	60	28	33	A	0	319516.514	-0.025	593.7842
60	29	31	60	28	32	A	0	319516.514	-0.025	593.7842
57	5	52	56	5	51	E	1	319525.471	0.001	515.9613
57	5	52	56	6	51	E	1	319525.471	0.001	515.9613
57	6	52	56	5	51	E	1	319525.471	0.001	515.9613
57	6	52	56	6	51	E	1	319525.471	0.001	515.9613
53	9	44	52	10	43	A	1	319527.974	0.099	501.4499
54	8	46	53	9	45	A	0	319536.837	-0.059	377.4579
54	9	46	53	8	45	A	0	319539.445	0.064	377.4578
53	10	44	52	10	43	A	1	319546.365	0.114	501.4499
53	9	44	52	9	43	A	1	319560.577	0.096	501.4488
58	4	54	57	4	53	A	1	319565.288	-0.106	518.6239
58	4	54	57	5	53	A	1	319565.288	-0.106	518.6239
58	5	54	57	4	53	A	1	319565.288	-0.106	518.6239
58	5	54	57	5	53	A	1	319565.288	-0.106	518.6239
57	5	52	56	5	51	E	0	319592.342	0.001	387.6676
57	6	52	56	6	51	E	0	319592.342	0.001	387.6676
57	5	52	56	5	51	A	0	319596.000	0.023	387.6627
57	5	52	56	6	51	A	0	319596.000	0.023	387.6627
57	6	52	56	5	51	A	0	319596.000	0.023	387.6627
57	6	52	56	6	51	A	0	319596.000	0.023	387.6627
31	12	19	30	11	20	A	0	319621.339	-0.029	186.6998
51	20	31	50	20	30	A	0	319632.241	0.009	407.0678
58	4	54	57	4	53	E	1	319666.960	0.150	518.4414
58	4	54	57	5	53	E	1	319666.960	0.150	518.4414
58	5	54	57	4	53	E	1	319666.960	0.150	518.4414
58	5	54	57	5	53	E	1	319666.960	0.150	518.4414
53	9	44	52	10	43	E	1	319716.510	-0.014	501.3427
58	4	54	57	4	53	E	0	319735.339	0.009	390.1514
58	5	54	57	5	53	E	0	319735.339	0.009	390.1514
58	4	54	57	4	53	A	0	319738.459	0.007	390.1457
58	4	54	57	5	53	A	0	319738.459	0.007	390.1457
58	5	54	57	4	53	A	0	319738.459	0.007	390.1457
58	5	54	57	5	53	A	0	319738.459	0.007	390.1457
59	3	56	58	3	55	A	1	319750.659	-0.096	520.7035
59	3	56	58	4	55	A	1	319750.659	-0.096	520.7035
59	4	56	58	3	55	A	1	319750.659	-0.096	520.7035

J'	K'_a	K'_c	J''	K''_a	K''_c	Sym.	$v_{t_{CH_3}}$	$\nu_{Obs.}$	$\nu_{Obs.} - \nu_{Calc.}$	E_{low}
59	4	56	58	4	55	A	1	319750.659	-0.096	520.7035
53	9	44	52	9	43	E	1	319755.001	0.054	501.3414
53	9	44	52	10	43	E	0	319771.002	-0.006	373.0506
53	9	44	52	10	43	A	0	319776.804	-0.045	373.0472
53	10	44	52	10	43	E	0	319791.251	0.057	373.0506
53	10	44	52	10	43	A	0	319797.133	-0.016	373.0472
53	9	44	52	9	43	E	0	319806.692	-0.022	373.0494
53	9	44	52	9	43	A	0	319812.690	-0.061	373.0460
53	10	44	52	9	43	E	0	319826.880	-0.020	373.0494
53	10	44	52	9	43	A	0	319832.912	-0.139	373.0460
59	3	56	58	3	55	E	1	319834.077	-0.017	520.4881
59	3	56	58	4	55	E	1	319834.077	-0.017	520.4881
59	4	56	58	3	55	E	1	319834.077	-0.017	520.4881
59	4	56	58	4	55	E	1	319834.077	-0.017	520.4881
27	14	14	26	13	14	E	0	319865.149	0.010	171.7752
27	14	13	26	13	13	E	0	319867.006	-0.052	171.7887
59	3	56	58	3	55	E	0	319904.558	0.030	392.2034
59	4	56	58	4	55	E	0	319904.558	0.030	392.2034
59	3	56	58	3	55	A	0	319907.197	0.108	392.1966
59	3	56	58	4	55	A	0	319907.197	0.108	392.1966
59	4	56	58	3	55	A	0	319907.197	0.108	392.1966
59	4	56	58	4	55	A	0	319907.197	0.108	392.1966
59	29	31	59	28	32	E	0	319915.908	-0.130	581.3220
59	29	31	59	28	32	A	0	319938.013	0.007	581.3332
59	29	30	59	28	31	A	0	319938.013	0.007	581.3332
60	2	58	59	2	57	A	1	319954.142	-0.056	522.3669
60	2	58	59	3	57	A	1	319954.142	-0.056	522.3669
60	3	58	59	2	57	A	1	319954.142	-0.056	522.3669
60	3	58	59	3	57	A	1	319954.142	-0.056	522.3669
59	29	30	59	28	31	E	0	319962.635	-0.095	581.3224
52	10	42	51	11	41	E	0	319966.010	0.028	368.0575
52	10	42	51	11	41	A	0	319971.003	-0.016	368.0540
60	2	58	59	2	57	E	1	320016.924	0.091	522.1116
60	2	58	59	3	57	E	1	320016.924	0.091	522.1116
60	3	58	59	2	57	E	1	320016.924	0.091	522.1116
60	3	58	59	3	57	E	1	320016.924	0.091	522.1116
52	11	42	51	11	41	A	1	320051.607	0.114	496.4547
36	11	26	35	10	25	E	0	320060.092	-0.086	218.5169
29	13	17	28	12	17	E	0	320080.496	-0.008	178.5486
60	2	58	59	3	57	E	0	320089.786	0.008	393.8341
60	3	58	59	2	57	E	0	320089.786	0.008	393.8341
60	2	58	59	2	57	A	0	320091.709	0.010	393.8262
60	2	58	59	3	57	A	0	320091.709	0.010	393.8262
60	3	58	59	2	57	A	0	320091.709	0.010	393.8262
60	3	58	59	3	57	A	0	320091.709	0.010	393.8262
29	13	17	28	12	16	A	0	320124.562	-0.395	178.5525
29	13	16	28	12	17	A	0	320127.189	-0.006	178.5525
36	11	26	35	10	25	A	0	320134.027	-0.006	218.5097
61	1	60	60	1	59	A	1	320169.583	0.008	523.6232
61	1	60	60	2	59	A	1	320169.583	0.008	523.6232
61	2	60	60	1	59	A	1	320169.583	0.008	523.6232
61	2	60	60	2	59	A	1	320169.583	0.008	523.6232
61	1	60	60	1	59	E	1	320207.941	0.033	523.3201
61	2	60	60	2	59	E	1	320207.941	0.033	523.3201
61	1	60	60	1	59	E	0	320284.266	-0.025	395.0520
61	2	60	60	2	59	E	0	320284.266	-0.025	395.0520
61	1	60	60	1	59	A	0	320285.519	0.051	395.0426
61	1	60	60	2	59	A	0	320285.519	0.051	395.0426
61	2	60	60	1	59	A	0	320285.519	0.051	395.0426
61	2	60	60	2	59	A	0	320285.519	0.051	395.0426
52	11	42	51	11	41	E	0	320314.650	-0.055	368.0575
58	29	30	58	28	31	E	0	320315.949	0.127	569.0848
58	29	30	58	28	31	A	0	320337.501	-0.010	569.0962
58	29	29	58	28	30	A	0	320337.501	-0.010	569.0962
58	29	29	58	28	30	E	0	320362.476	-0.108	569.0853
62	0	62	61	0	61	A	1	320393.138	0.170	524.4798
62	0	62	61	1	61	A	1	320393.138	0.170	524.4798
62	1	62	61	0	61	A	1	320393.138	0.170	524.4798
62	1	62	61	1	61	A	1	320393.138	0.170	524.4798
62	0	62	61	0	61	E	1	320402.393	0.059	524.1200
62	1	62	61	1	61	E	1	320402.393	0.059	524.1200
50	14	37	49	14	36	A	0	320419.164	-0.058	360.7826
50	14	37	49	14	36	E	0	320423.647	-0.052	360.7849
62	0	62	61	0	61	A	0	320483.596	-0.142	395.8525
62	0	62	61	1	61	A	0	320483.596	-0.142	395.8525
62	1	62	61	0	61	A	0	320483.596	-0.142	395.8525
62	1	62	61	1	61	A	0	320483.596	-0.142	395.8525
62	0	62	61	0	61	E	0	320483.596	0.156	395.8637
62	1	62	61	1	61	E	0	320483.596	0.156	395.8637
51	19	33	50	19	32	A	0	320514.088	0.227	399.9582
51	19	32	50	19	31	E	0	320517.708	0.001	399.9583
51	19	33	50	19	32	E	0	320519.100	0.079	399.9474
52	10	42	51	10	41	E	0	320550.188	-0.037	368.0380
52	10	42	51	10	41	A	0	320557.899	-0.071	368.0345
57	29	29	57	28	30	A	0	320715.984	0.002	557.0726
57	29	28	57	28	29	A	0	320715.984	0.002	557.0726
51	12	40	50	12	39	E	0	320782.272	-0.015	362.4398
51	12	40	50	12	39	A	0	320788.291	-0.010	362.4361
52	11	42	51	10	41	E	0	320898.940	-0.008	368.0380
50	15	35	49	15	34	A	0	320948.127	-0.056	365.6238
50	15	35	49	15	34	E	0	320953.677	-0.010	365.6252
56	29	28	56	28	29	E	0	321052.947	-0.198	545.2507
56	29	28	56	28	29	A	0	321074.272	-0.039	545.2622
56	29	27	56	28	28	A	0	321074.272	-0.039	545.2622
56	29	27	56	28	28	E	0	321099.957	-0.067	545.2512
55	29	27	55	28	28	E	0	321392.364	-0.062	533.6531
55	29	27	55	28	28	A	0	321413.326	-0.023	533.6647
55	29	26	55	28	27	A	0	321413.326	-0.023	533.6647
55	29	26	55	28	27	E	0	321439.323	-0.033	533.6537
51	18	34	50	18	33	A	0	321593.943	-0.025	393.2942
51	18	33	50	18	32	A	0	321599.746	-0.129	393.2944
51	18	33	50	18	32	E	0	321600.897	0.071	393.2961
51	18	34	50	18	33	E	0	321602.861	0.029	393.2846
54	29	26	54	28	27	E	0	321713.149	-0.075	522.2680
54	29	26	54	28	27	A	0	321733.805	-0.112	522.2797
54	29	25	54	28	26	A	0	321733.805	-0.112	522.2797
52	30	22	51	30	21	A	0	321741.655	-0.017	511.3095
52	30	23	51	30	22	A	0	321741.655	-0.017	511.3095
52	30	22	51	30	21	E	0	321741.655	-0.249	511.3004
54	29	25	54	28	26	E	0	321760.142	-0.057	522.2686
52	29	24	51	29	23	A	0	321985.147	0.033	500.1573
52	29	23	51	29	22	A	0	321985.147	0.033	500.1573
52	29	23	51	29	22	E	0	321985.147	-0.397	500.1471
53	29	25	53	28	26	E	0	322016.243	-0.082	511.0952
53	29	25	53	28	26	A	0	322036.740	-0.058	511.1069
53	29	24	53	28	25	A	0	322036.740	-0.058	511.1069

J'	K'_a	K'_c	J''	K''_a	K''_c	Sym.	$v_{t_{CH_3}}$	$\nu_{Obs.}$	$\nu_{Obs.} - \nu_{Calc.}$	E_{low}
53	29	24	53	28	25	E	0	322063.306	-0.035	511.0958
52	29	24	52	28	25	E	0	322302.390	-0.095	500.1342
52	29	24	52	28	25	A	0	322322.669	-0.080	500.1460
52	29	23	52	28	24	A	0	322322.669	-0.080	500.1460
52	29	23	52	28	24	E	0	322349.403	-0.134	500.1349
51	29	23	51	28	24	E	0	322572.373	-0.060	489.3849
51	29	23	51	28	24	A	0	322592.439	-0.059	489.3967
51	29	22	51	28	23	A	0	322592.439	-0.059	489.3967
51	29	22	51	28	23	E	0	322619.532	0.018	489.3856
50	29	22	50	28	23	E	0	322826.801	-0.064	478.8469
50	29	22	50	28	23	A	0	322846.688	-0.056	478.8588
50	29	21	50	28	22	A	0	322846.688	-0.056	478.8588
50	29	21	50	28	22	E	0	322873.915	-0.059	478.8477
52	26	26	51	26	25	A	0	322891.702	-0.133	469.0581
52	26	27	51	26	26	A	0	322891.702	-0.133	469.0581
52	26	27	51	26	26	E	0	322892.963	-0.096	469.0457
52	26	26	51	26	25	E	0	322894.087	0.059	469.0481
51	17	35	50	17	34	A	0	322941.747	-0.080	387.0961
51	17	34	50	17	33	E	0	322980.525	-0.177	387.1007
20	18	2	19	17	2	E	0	322989.221	-0.065	161.3446
51	17	34	50	17	33	A	0	322997.159	-0.084	387.0982
20	18	3	19	17	3	E	0	323009.780	0.022	161.3318
20	18	2	19	17	3	A	0	323036.594	-0.049	161.3427
20	18	3	19	17	2	A	0	323036.594	-0.049	161.3427
49	29	21	49	28	22	E	0	323066.372	-0.088	468.5200
51	11	40	50	11	39	E	0	323071.068	-0.062	362.2232
51	11	40	50	11	39	A	0	323084.544	-0.034	362.2188
49	29	21	49	28	22	A	0	323086.093	-0.066	468.5319
49	29	20	49	28	21	A	0	323086.093	-0.066	468.5319
49	29	20	49	28	21	E	0	323113.527	-0.064	468.5208
52	11	41	51	12	40	A	0	323245.175	-0.027	373.1365
52	11	41	51	12	40	E	0	323249.724	-0.036	373.1399
52	25	28	51	25	27	A	0	323271.085	-0.072	459.4845
52	25	27	51	25	26	A	0	323271.085	-0.072	459.4845
48	29	20	48	28	21	E	0	323291.775	-0.091	458.4038
48	29	20	48	28	21	A	0	323311.309	-0.087	458.4158
48	29	19	48	28	20	A	0	323311.309	-0.087	458.4158
47	29	19	47	28	20	E	0	323503.679	-0.032	448.4982
47	29	19	47	28	20	A	0	323523.015	-0.066	448.5102
47	29	18	47	28	19	A	0	323523.015	-0.066	448.5102
47	29	18	47	28	19	E	0	323550.876	-0.000	448.4990
46	29	18	46	28	19	A	0	323721.731	-0.089	438.8149
46	29	17	46	28	18	A	0	323721.731	-0.089	438.8149
46	29	17	46	28	18	E	0	323749.634	-0.144	438.8037
22	17	5	21	16	5	E	0	323843.281	-0.076	163.4885
22	17	6	21	16	6	E	0	323858.674	0.023	163.4752
22	17	6	21	16	5	A	0	323889.904	-0.038	163.4850
22	17	5	21	16	6	A	0	323889.904	-0.038	163.4850
45	29	17	45	28	18	A	0	323908.109	-0.087	429.3295
45	29	16	45	28	17	A	0	323908.109	-0.087	429.3295
45	29	16	45	28	17	E	0	323936.274	-0.033	429.3183
49	12	37	48	12	36	E	0	323988.131	-0.025	344.2577
49	12	37	48	12	36	A	0	323997.396	-0.043	344.2511
44	29	16	44	28	17	E	0	324063.828	-0.006	420.0418
44	29	16	44	28	17	A	0	324082.729	-0.047	420.0539
44	29	15	44	28	16	A	0	324082.729	-0.047	420.0539
44	29	15	44	28	16	E	0	324111.009	-0.020	420.0427
52	23	30	51	23	29	A	0	324194.277	-0.195	441.5446
52	23	29	51	23	28	A	0	324194.277	-0.195	441.5446
43	29	15	43	28	16	E	0	324227.235	-0.055	410.9757
43	29	15	43	28	16	A	0	324246.035	-0.071	410.9879
43	29	14	43	28	15	A	0	324246.035	-0.071	410.9879
56	7	49	55	7	48	A	1	324349.725	-0.043	520.4100
56	7	49	55	8	48	A	1	324349.725	-0.018	520.4100
56	8	49	55	7	48	A	1	324349.725	-0.056	520.4100
56	8	49	55	8	48	A	1	324349.725	-0.031	520.4100
42	29	14	42	28	15	E	0	324380.006	-0.011	402.1189
55	8	47	54	9	46	A	1	324392.915	0.020	516.5181
55	9	47	54	8	46	A	1	324394.072	-0.016	516.5181
42	29	14	42	28	15	A	0	324398.645	-0.068	402.1311
42	29	13	42	28	14	A	0	324398.645	-0.068	402.1311
57	6	51	56	6	50	A	1	324406.707	-0.101	523.8196
57	6	51	56	7	50	A	1	324406.707	-0.101	523.8196
57	7	51	56	6	50	A	1	324406.707	-0.102	523.8196
57	7	51	56	7	50	A	1	324406.707	-0.101	523.8196
58	5	53	57	5	52	A	1	324526.564	-0.139	526.7715
58	5	53	57	6	52	A	1	324526.564	-0.139	526.7715
58	6	53	57	5	52	A	1	324526.564	-0.139	526.7715
58	6	53	57	6	52	A	1	324526.564	-0.139	526.7715
57	6	51	56	6	50	E	1	324538.839	-0.075	523.6888
57	6	51	56	7	50	E	1	324538.839	-0.074	523.6888
57	7	51	56	6	50	E	1	324538.839	-0.075	523.6888
57	7	51	56	7	50	E	1	324538.839	-0.075	523.6888
41	29	13	41	28	14	A	0	324541.023	-0.085	393.4833
41	29	12	41	28	13	A	0	324541.023	-0.085	393.4833
55	8	47	54	9	46	E	1	324558.301	0.024	516.4131
55	9	47	54	8	46	E	1	324559.763	0.030	516.4130
56	7	49	55	7	48	E	0	324562.711	-0.035	392.0006
56	8	49	55	8	48	E	0	324562.711	-0.021	392.0006
56	7	49	55	7	48	A	0	324567.294	-0.028	391.9970
56	7	49	55	8	48	A	0	324567.294	0.001	391.9970
56	8	49	55	7	48	A	0	324567.294	-0.043	391.9970
56	8	49	55	8	48	A	0	324567.294	-0.014	391.9970
41	29	12	41	28	13	E	0	324569.778	0.054	393.4721
54	9	45	53	10	44	A	1	324593.244	0.182	512.1088
54	10	45	53	10	44	A	1	324603.362	0.023	512.1088
57	6	51	56	6	50	E	0	324606.169	-0.029	395.3952
57	7	51	56	7	50	E	0	324606.169	-0.029	395.3952
57	6	51	56	6	50	A	0	324610.268	-0.015	395.3911
57	6	51	56	7	50	A	0	324610.268	-0.014	395.3911
57	7	51	56	6	50	A	0	324610.268	-0.015	395.3911
57	7	51	56	7	50	A	0	324610.268	-0.014	395.3911
55	8	47	54	9	46	A	0	324625.518	0.146	388.1165
49	12	38	48	11	37	E	0	324625.518	-0.198	341.0854
55	9	47	54	8	46	A	0	324626.731	0.015	388.1165
58	5	53	57	5	52	E	1	324643.582	0.032	526.6195
58	5	53	57	6	52	E	1	324643.582	0.032	526.6195
58	6	53	57	5	52	E	1	324643.582	0.032	526.6195
58	6	53	57	6	52	E	1	324643.582	0.032	526.6195
24	16	8	23	15	8	E	0	324656.159	-0.062	166.8645
24	16	9	23	15	9	E	0	324666.114	0.076	166.8509
40	29	12	40	28	13	A	0	324673.739	-0.047	385.0444
40	29	11	40	28	12	A	0	324673.739	-0.047	385.0444
59	4	55	58	4	54	A	1	324686.852	-0.130	529.2835
59	4	55	58	5	54	A	1	324686.852	-0.130	529.2835
59	5	55	58	4	54	A	1	324686.852	-0.130	529.2835

J'	K_a'	K_c'	J''	K_a''	K_c''	Sym.	$v_{t_{CH_3}}$	$\nu_{Obs.}$	$\nu_{Obs.} - \nu_{Calc.}$	E_{low}
59	5	55	58	5	54	A	1	324686.852	-0.130	529.2835
24	16	8	23	15	9	A	0	324701.319	-0.058	166.8595
24	16	9	23	15	8	A	0	324701.319	-0.058	166.8595
58	5	53	57	5	52	E	0	324712.359	-0.018	398.3280
58	6	53	57	6	52	E	0	324712.359	-0.018	398.3280
58	5	53	57	5	52	A	0	324715.975	-0.008	398.3232
58	5	53	57	6	52	A	0	324715.975	-0.008	398.3232
58	6	53	57	5	52	A	0	324715.975	-0.008	398.3232
58	6	53	57	6	52	A	0	324715.975	-0.008	398.3232
52	22	30	51	22	29	A	0	324763.582	-0.057	433.1871
52	22	31	51	22	30	A	0	324763.582	-0.057	433.1871
52	22	31	51	22	30	E	0	324766.592	-0.003	433.1745
59	4	55	58	4	54	E	1	324787.811	0.028	529.1044
59	4	55	58	5	54	E	1	324787.811	0.028	529.1044
59	5	55	58	4	54	E	1	324787.811	0.028	529.1044
59	5	55	58	5	54	E	1	324787.811	0.028	529.1044
39	29	11	39	28	12	A	0	324797.183	-0.042	376.8141
39	29	10	39	28	11	A	0	324797.183	-0.042	376.8141
54	10	45	53	9	44	E	1	324812.690	-0.131	512.0073
39	29	10	39	28	11	E	0	324826.029	-0.010	376.8029
54	9	45	53	10	44	E	0	324835.995	-0.044	383.7176
54	9	45	53	10	44	A	0	324841.759	-0.038	383.7145
54	10	45	53	10	44	E	0	324847.318	-0.048	383.7176
54	10	45	53	10	44	A	0	324853.159	-0.030	383.7145
54	9	45	53	9	44	E	0	324856.229	0.003	383.7170
59	4	55	58	4	54	E	0	324858.073	-0.015	400.8166
59	5	55	58	5	54	E	0	324858.073	-0.015	400.8166
59	4	55	58	4	54	A	0	324861.188	-0.003	400.8110
59	4	55	58	5	54	A	0	324861.188	-0.003	400.8110
59	5	55	58	4	54	A	0	324861.188	-0.003	400.8110
59	5	55	58	5	54	A	0	324861.188	-0.003	400.8110
54	10	45	53	9	44	E	0	324867.514	-0.038	383.7170
60	3	57	59	3	56	A	1	324873.513	-0.142	531.3692
60	3	57	59	4	56	A	1	324873.513	-0.142	531.3692
60	4	57	59	3	56	A	1	324873.513	-0.142	531.3692
60	4	57	59	4	56	A	1	324873.513	-0.142	531.3692
38	29	10	38	28	11	A	0	324911.800	-0.087	368.7923
38	29	9	38	28	10	A	0	324911.800	-0.087	368.7923
49	13	36	48	13	35	A	0	324913.395	-0.091	347.0442
49	13	36	48	13	35	E	0	324923.669	-0.059	347.0498
38	29	9	38	28	10	E	0	324940.789	0.002	368.7810
60	3	57	59	3	56	E	1	324956.643	0.003	531.1566
60	3	57	59	4	56	E	1	324956.643	0.003	531.1566
60	4	57	59	3	56	E	1	324956.643	0.003	531.1566
60	4	57	59	4	56	E	1	324956.643	0.003	531.1566
32	12	21	31	11	20	A	0	324988.921	-0.032	193.2289
37	29	9	37	28	10	E	0	325000.009	-0.010	360.9663
51	16	35	50	16	34	E	0	325001.317	0.028	381.4106
51	16	35	50	16	34	A	0	325014.443	-0.049	381.4096
37	29	9	37	28	10	A	0	325018.129	-0.091	360.9786
37	29	8	37	28	9	A	0	325018.129	-0.091	360.9786
60	3	57	59	3	56	E	0	325028.693	-0.021	402.8743
60	4	57	59	4	56	E	0	325028.693	-0.021	402.8743
60	3	57	59	3	56	A	0	325031.247	-0.017	402.8676
60	3	57	59	4	56	A	0	325031.247	-0.017	402.8676
60	4	57	59	3	56	A	0	325031.247	-0.017	402.8676
60	4	57	59	4	56	A	0	325031.247	-0.017	402.8676
37	29	8	37	28	9	E	0	325047.204	0.004	360.9673
53	10	43	52	11	42	E	1	325065.062	0.040	507.0273
32	12	20	31	11	20	E	0	325070.945	-0.054	193.2363
61	2	59	60	2	58	A	1	325077.679	-0.078	533.0394
61	2	59	60	3	58	A	1	325077.679	-0.078	533.0394
61	3	59	60	2	58	A	1	325077.679	-0.078	533.0394
61	3	59	60	3	58	A	1	325077.679	-0.078	533.0394
51	13	39	50	13	38	E	0	325098.310	0.084	366.9983
51	13	39	50	13	38	A	0	325100.557	-0.039	366.9950
36	29	8	36	28	9	A	0	325116.574	-0.084	353.3729
36	29	7	36	28	8	A	0	325116.574	-0.084	353.3729
53	10	43	52	11	42	E	0	325128.040	-0.044	378.7421
53	10	43	52	11	42	A	0	325133.632	-0.044	378.7388
32	12	21	31	11	21	E	0	325136.769	-0.234	193.2213
61	2	59	60	2	58	E	1	325140.234	0.016	532.7862
61	2	59	60	3	58	E	1	325140.234	0.016	532.7862
61	3	59	60	2	58	E	1	325140.234	0.016	532.7862
61	3	59	60	3	58	E	1	325140.234	0.016	532.7862
35	29	7	35	28	8	A	0	325207.546	-0.074	345.9751
35	29	6	35	28	7	A	0	325207.546	-0.074	345.9751
61	2	59	60	2	58	E	0	325214.684	0.002	404.5112
61	3	59	60	3	58	E	0	325214.684	0.002	404.5112
61	2	59	60	2	58	A	0	325216.583	-0.014	404.5033
61	2	59	60	3	58	A	0	325216.583	-0.014	404.5033
61	3	59	60	2	58	A	0	325216.583	-0.014	404.5033
61	3	59	60	3	58	A	0	325216.583	-0.014	404.5033
35	29	6	35	28	7	E	0	325236.735	-0.001	345.9638
53	11	43	52	11	42	E	1	325285.463	-0.078	507.0273
62	1	61	61	1	60	A	1	325293.496	0.009	534.3029
62	1	61	61	2	60	A	1	325293.496	0.009	534.3029
62	2	61	61	1	60	A	1	325293.496	0.009	534.3029
62	2	61	61	2	60	A	1	325293.496	0.009	534.3029
34	29	5	34	28	6	E	0	325320.708	0.023	338.7736
62	1	61	61	1	60	E	1	325331.777	0.038	534.0011
62	2	61	61	2	60	E	1	325331.777	0.038	534.0011
53	11	43	52	11	42	E	0	325333.972	-0.029	378.7421
53	11	43	52	11	42	A	0	325340.583	-0.042	378.7388
50	12	39	49	11	38	E	0	325361.882	-0.040	351.5869
26	15	11	25	14	11	E	0	325373.434	-0.087	171.4897
26	15	12	25	14	12	E	0	325377.543	-0.009	171.4761
62	1	61	61	1	60	E	0	325409.496	-0.049	405.7355
62	2	61	61	2	60	E	0	325409.496	-0.049	405.7355
62	1	61	61	1	60	A	0	325410.749	0.030	405.7261
62	1	61	61	2	60	A	0	325410.749	0.030	405.7261
62	2	61	61	1	60	A	0	325410.749	0.030	405.7261
62	2	61	61	2	60	A	0	325410.749	0.030	405.7261
26	15	12	25	14	11	A	0	325416.536	-0.052	171.4832
26	15	11	25	14	12	A	0	325416.536	-0.052	171.4832
52	21	32	51	21	31	A	0	325428.496	-0.005	425.2460
52	21	31	51	21	30	E	0	325431.924	0.102	425.2429
53	10	43	52	10	42	E	0	325476.767	-0.039	378.7304
53	10	43	52	10	42	A	0	325484.065	-0.015	378.7271
63	0	63	62	0	62	A	1	325517.287	0.152	535.1669
63	0	63	62	1	62	A	1	325517.287	0.152	535.1669
63	1	63	62	0	62	A	1	325517.287	0.152	535.1669
63	1	63	62	1	62	A	1	325517.287	0.152	535.1669
63	0	63	62	0	62	E	1	325526.458	0.040	534.8074
63	1	63	62	1	62	E	1	325526.458	0.040	534.8074
63	0	63	62	0	62	A	0	325609.013	-0.160	406.5427

J'	K'_a	K'_c	J''	K''_a	K''_c	Sym.	$v_{t_{CH_3}}$	$\nu_{Obs.}$	$\nu_{Obs.} - \nu_{Calc.}$	E_{low}
63	0	63	62	1	62	A	0	325609.013	-0.160	406.5427
63	1	63	62	0	62	A	0	325609.013	-0.160	406.5427
63	1	63	62	1	62	A	0	325609.013	-0.160	406.5427
63	0	63	62	0	62	E	0	325609.013	0.135	406.5539
63	1	63	62	1	62	E	0	325609.013	0.135	406.5539
53	11	43	52	10	42	E	0	325682.659	-0.065	378.7304
53	11	43	52	10	42	A	0	325690.999	-0.030	378.7271
34	11	23	33	10	24	E	0	325811.627	-0.069	203.3206
34	11	23	33	10	24	A	0	325823.192	-0.047	203.3148
52	12	41	51	12	40	E	1	325868.925	0.060	501.4183
28	14	15	27	13	15	E	0	325886.303	0.147	177.3774
28	14	14	27	13	14	E	0	325888.156	-0.163	177.3909
52	12	41	51	12	40	E	0	325918.802	-0.016	373.1399
52	12	41	51	12	40	A	0	325925.236	-0.032	373.1365
28	14	15	27	13	14	A	0	325928.594	-0.013	177.3830
30	13	18	29	12	18	E	0	325969.078	-0.039	184.5995
30	13	17	29	12	17	E	0	325978.091	-0.095	184.6125
53	12	41	52	13	40	A	0	325999.677	-0.058	388.8701
30	13	18	29	12	17	A	0	326012.266	-0.141	184.6033
30	13	17	29	12	18	A	0	326017.655	-0.007	184.6032
53	12	41	52	13	40	E	0	326030.440	-0.029	388.8733
52	20	33	51	20	32	A	0	326215.839	-0.060	417.7296
51	15	37	50	15	36	A	0	326269.410	-0.008	376.1988
51	15	37	50	15	36	E	0	326278.366	-0.063	376.1995
70	30	40	70	29	41	E	0	326537.598	0.144	740.5988
50	14	36	49	14	35	A	0	326867.317	-0.067	361.2232
50	14	36	49	14	35	E	0	326883.207	-0.100	361.2267
51	14	38	50	14	37	E	0	326908.863	-0.084	371.4730
69	30	39	69	29	40	A	0	327137.854	-0.008	726.0209
69	30	40	69	29	41	A	0	327137.854	-0.008	726.0209
69	30	39	69	29	40	E	0	327163.124	-0.111	726.0112
52	19	34	51	19	33	A	0	327164.530	-0.136	410.6494
52	19	33	51	19	32	A	0	327165.671	-0.060	410.6494
52	19	33	51	19	32	E	0	327168.952	-0.054	410.6496
52	19	34	51	19	33	E	0	327170.395	-0.058	410.6388
47	12	36	46	11	35	E	0	327250.912	-0.061	320.4835
47	12	36	46	11	35	A	0	327324.148	-0.006	320.4769
52	11	41	51	11	40	E	0	327453.053	-0.083	372.9997
52	11	41	51	11	40	A	0	327464.973	-0.036	372.9957
53	32	21	52	32	20	A	0	327608.505	-0.089	545.5019
53	32	22	52	32	21	A	0	327608.505	-0.089	545.5019
53	32	21	52	32	20	E	0	327608.505	-0.014	545.4952
68	30	38	68	29	39	A	0	327733.389	-0.019	711.6502
68	30	39	68	29	40	A	0	327733.389	-0.019	711.6502
19	19	0	18	18	0	E	0	328268.245	-0.082	164.1139
67	30	38	67	29	39	E	0	328281.180	-0.149	697.4848
19	19	1	18	18	1	E	0	328293.658	0.025	164.1018
67	30	37	67	29	38	A	0	328300.524	-0.002	697.4959
67	30	38	67	29	39	A	0	328300.524	-0.002	697.4959
53	29	25	52	29	24	A	0	328313.117	0.077	510.8975
53	29	24	52	29	23	A	0	328313.117	0.077	510.8975
53	29	24	52	29	23	E	0	328313.117	-0.397	510.8873
19	19	1	18	18	0	A	0	328315.751	-0.044	164.1136
19	19	0	18	18	1	A	0	328315.751	-0.044	164.1136
52	18	35	51	18	34	A	0	328331.674	-0.067	404.0214
52	18	34	51	18	33	E	0	328341.640	-0.097	404.0235
51	15	36	50	15	35	A	0	328693.576	-0.062	376.3295
51	15	36	50	15	35	E	0	328703.455	-0.027	376.3311
50	12	38	49	12	37	E	0	328751.237	-0.068	355.0648
50	12	38	49	12	37	A	0	328764.783	-0.024	355.0585
66	30	37	66	29	38	E	0	328821.483	-0.117	683.5464
66	30	36	66	29	37	A	0	328840.373	-0.041	683.5576
66	30	37	66	29	38	A	0	328840.373	-0.041	683.5576
21	18	3	20	17	3	E	0	329133.220	-0.071	165.4524
21	18	4	20	17	4	E	0	329153.744	0.013	165.4397
21	18	3	20	17	4	A	0	329180.570	-0.068	165.4505
21	18	4	20	17	3	A	0	329180.570	-0.068	165.4505
53	11	42	52	12	41	A	0	329329.381	0.214	384.0082
53	11	42	52	12	41	E	0	329329.381	-0.282	384.0114
65	30	36	65	29	37	E	0	329335.627	-0.140	669.8235
65	30	35	65	29	36	A	0	329354.193	-0.018	669.8349
65	30	36	65	29	37	A	0	329354.193	-0.018	669.8349
55	13	42	54	14	41	A	0	329372.700	-0.116	416.3286
65	30	35	65	29	36	E	0	329381.435	-0.069	669.8251
55	13	42	54	14	41	E	0	329435.034	-0.027	416.3311
57	7	50	56	7	49	A	1	329456.145	-0.065	531.2291
57	7	50	56	8	49	A	1	329456.145	-0.052	531.2291
57	8	50	56	7	49	A	1	329456.145	-0.071	531.2291
57	8	50	56	8	49	A	1	329456.145	-0.058	531.2291
56	8	48	55	9	47	A	1	329484.453	0.279	527.3387
56	9	48	55	8	47	A	1	329484.453	-0.361	527.3387
58	6	52	57	6	51	A	1	329521.401	-0.093	534.6407
58	6	52	57	7	51	A	1	329521.401	-0.093	534.6407
58	7	52	57	6	51	A	1	329521.401	-0.094	534.6407
58	7	52	57	7	51	A	1	329521.401	-0.093	534.6407
57	7	50	56	7	49	E	1	329601.910	-0.044	531.1189
57	7	50	56	8	49	E	1	329601.910	-0.027	531.1189
57	8	50	56	7	49	E	1	329601.910	-0.052	531.1189
57	8	50	56	8	49	E	1	329601.910	-0.036	531.1189
59	5	54	58	5	53	A	1	329645.684	-0.158	537.5966
59	5	54	58	6	53	A	1	329645.684	-0.158	537.5966
59	6	54	58	5	53	A	1	329645.684	-0.158	537.5966
59	6	54	58	6	53	A	1	329645.684	-0.158	537.5966
58	6	52	57	6	51	E	1	329652.180	-0.020	534.5143
58	6	52	57	7	51	E	1	329652.180	-0.020	534.5143
58	7	52	57	6	51	E	1	329652.180	-0.020	534.5143
58	7	52	57	7	51	E	1	329652.180	-0.020	534.5143
55	9	46	54	10	45	A	1	329660.208	0.059	522.9364
57	7	50	56	7	49	E	0	329669.564	-0.039	402.8268
57	8	50	56	8	49	E	0	329669.564	-0.032	402.8268
57	7	50	56	7	49	A	0	329674.093	-0.025	402.8234
57	7	50	56	8	49	A	0	329674.093	-0.010	402.8234
57	8	50	56	7	49	A	0	329674.093	-0.032	402.8234
57	8	50	56	8	49	A	0	329674.093	-0.018	402.8234
55	10	46	54	9	45	A	1	329676.276	0.142	522.9361
56	8	48	55	8	47	E	0	329711.789	-0.131	398.9480
56	9	48	55	9	47	E	0	329711.789	0.086	398.9480
56	8	48	55	8	47	A	0	329716.816	-0.147	398.9448
56	8	48	55	9	47	A	0	329716.816	0.323	398.9449
56	9	48	55	8	47	A	0	329716.816	-0.399	398.9448
56	9	48	55	9	47	A	0	329716.816	0.071	398.9449
58	6	52	57	6	51	E	0	329721.624	-0.025	406.2229
58	7	52	57	7	51	E	0	329721.624	-0.024	406.2229
58	6	52	57	6	51	A	0	329725.679	-0.010	406.2189
58	6	52	57	7	51	A	0	329725.679	-0.010	406.2189
58	7	52	57	6	51	A	0	329725.679	-0.011	406.2189

J'	K'_a	K'_c	J''	K''_a	K''_c	Sym.	$v_{t_{CH_3}}$	$\nu_{Obs.}$	$\nu_{Obs.} - \nu_{Calc.}$	E_{low}
58	7	52	57	7	51	A	0	329725.679	-0.010	406.2189
59	5	54	58	5	53	E	1	329761.711	-0.028	537.4484
59	5	54	58	6	53	E	1	329761.711	-0.028	537.4484
59	6	54	58	5	53	E	1	329761.711	-0.028	537.4484
59	6	54	58	6	53	E	1	329761.711	-0.028	537.4484
60	4	56	59	4	55	A	1	329808.346	-0.151	540.1139
60	4	56	59	5	55	A	1	329808.346	-0.151	540.1139
60	5	56	59	4	55	A	1	329808.346	-0.151	540.1139
60	5	56	59	5	55	A	1	329808.346	-0.151	540.1139
52	17	36	51	17	35	E	0	329823.633	-0.117	397.8619
64	30	35	64	29	36	E	0	329824.862	-0.049	656.3159
59	5	54	58	5	53	E	0	329832.503	-0.020	409.1593
59	6	54	58	6	53	E	0	329832.503	-0.020	409.1593
59	5	54	58	5	53	A	0	329836.091	-0.009	409.1546
59	5	54	58	6	53	A	0	329836.091	-0.009	409.1546
59	6	54	58	5	53	A	0	329836.091	-0.009	409.1546
59	6	54	58	6	53	A	0	329836.091	-0.009	409.1546
55	10	46	54	10	45	E	1	329849.248	-0.078	522.8419
55	9	46	54	9	45	E	1	329854.741	0.007	522.8415
55	10	46	54	9	45	E	1	329861.606	0.023	522.8415
52	17	35	51	17	34	E	0	329863.697	-0.082	397.8742
64	30	34	64	29	35	E	0	329870.650	-0.066	656.3175
52	17	35	51	17	34	A	0	329886.915	-0.266	397.8722
55	9	46	54	10	45	E	0	329902.704	-0.035	394.5534
55	10	46	54	9	45	A	0	329926.063	-0.074	394.5500
23	17	6	22	16	6	E	0	329978.536	-0.067	168.0149
60	4	56	59	4	55	E	0	329980.779	-0.004	411.6527
60	5	56	59	5	55	E	0	329980.779	-0.004	411.6527
60	4	56	59	4	55	A	0	329983.860	-0.008	411.6472
60	4	56	59	5	55	A	0	329983.860	-0.008	411.6472
60	5	56	59	4	55	A	0	329983.860	-0.008	411.6472
60	5	56	59	5	55	A	0	329983.860	-0.008	411.6472
23	17	7	22	16	7	E	0	329993.848	0.003	168.0017
61	3	58	60	3	57	A	1	329996.303	-0.104	542.2058
61	3	58	60	4	57	A	1	329996.303	-0.104	542.2058
61	4	58	60	3	57	A	1	329996.303	-0.104	542.2058
61	4	58	60	4	57	A	1	329996.303	-0.104	542.2058
23	17	7	22	16	6	A	0	330025.123	-0.049	168.0114
23	17	6	22	16	7	A	0	330025.123	-0.049	168.0114
61	3	58	60	3	57	E	1	330079.051	0.008	541.9960
61	3	58	60	4	57	E	1	330079.051	0.008	541.9960
61	4	58	60	3	57	E	1	330079.051	0.008	541.9960
61	4	58	60	4	57	E	1	330079.051	0.008	541.9960
54	11	44	53	11	43	A	1	330092.978	0.157	517.9737
52	12	41	51	11	40	E	0	330122.166	-0.027	372.9997
53	24	29	52	24	28	A	0	330135.814	-0.089	461.1096
53	24	30	52	24	29	A	0	330135.814	-0.089	461.1096
52	12	41	51	11	40	A	0	330145.045	-0.031	372.9957
61	3	58	60	3	57	E	0	330152.756	-0.003	413.7160
61	4	58	60	4	57	E	0	330152.756	-0.003	413.7160
61	3	58	60	3	57	A	0	330155.295	-0.002	413.7095
61	3	58	60	4	57	A	0	330155.295	-0.002	413.7095
61	4	58	60	3	57	A	0	330155.295	-0.002	413.7095
61	4	58	60	4	57	A	0	330155.295	-0.002	413.7095
54	10	44	53	10	43	A	1	330172.019	0.087	517.9674
54	10	44	53	11	43	E	1	330173.262	0.020	517.8777
62	2	60	61	2	59	A	1	330201.041	-0.099	543.8828
62	2	60	61	3	59	A	1	330201.041	-0.099	543.8828
62	3	60	61	2	59	A	1	330201.041	-0.099	543.8828
62	3	60	61	3	59	A	1	330201.041	-0.099	543.8828
54	10	44	53	11	43	A	0	330239.977	-0.025	389.5910
62	2	60	61	2	59	E	1	330263.438	0.009	543.6317
62	2	60	61	3	59	E	1	330263.438	0.009	543.6317
62	3	60	61	2	59	E	1	330263.438	0.009	543.6317
62	3	60	61	3	59	E	1	330263.438	0.009	543.6317
63	30	34	63	29	35	E	0	330289.925	-0.143	643.0231
54	11	44	53	11	43	E	1	330302.398	-0.155	517.8777
63	30	33	63	29	34	A	0	330307.826	0.006	643.0347
63	30	34	63	29	35	A	0	330307.826	0.006	643.0347
63	30	33	63	29	34	E	0	330335.807	-0.128	643.0248
62	2	60	61	2	59	E	0	330339.407	-0.007	415.3592
62	3	60	61	3	59	E	0	330339.407	-0.007	415.3592
62	2	60	61	2	59	A	0	330341.316	-0.008	415.3513
62	2	60	61	3	59	A	0	330341.316	-0.008	415.3513
62	3	60	61	2	59	A	0	330341.316	-0.008	415.3513
62	3	60	61	3	59	A	0	330341.316	-0.008	415.3513
33	12	22	32	11	21	A	0	330345.081	-0.047	199.9810
54	11	44	53	11	43	E	0	330354.515	-0.017	389.5940
54	11	44	53	11	43	A	0	330361.086	0.057	389.5910
63	1	62	62	2	61	A	1	330417.231	0.017	545.1535
63	2	62	62	1	61	A	1	330417.231	0.017	545.1535
54	10	44	53	10	43	E	0	330440.031	-0.017	389.5872
54	10	44	53	10	43	A	0	330446.953	0.002	389.5841
63	1	62	62	1	61	E	1	330455.413	0.025	544.8530
63	2	62	62	2	61	E	1	330455.413	0.025	544.8530
33	12	21	32	11	21	E	0	330489.393	-0.100	199.9874
54	11	44	53	10	43	E	1	330523.076	0.005	517.8703
63	1	62	62	1	61	E	0	330534.563	-0.054	416.5900
63	2	62	62	2	61	E	0	330534.563	-0.054	416.5900
63	1	62	62	1	61	A	0	330535.822	0.034	416.5807
63	1	62	62	2	61	A	0	330535.822	0.034	416.5807
63	2	62	62	1	61	A	0	330535.822	0.034	416.5807
63	2	62	62	2	61	A	0	330535.822	0.034	416.5807
54	11	44	53	10	43	E	0	330560.407	-0.043	389.5872
54	11	44	53	10	43	A	0	330567.957	-0.020	389.5841
64	0	64	63	0	63	A	1	330641.280	0.161	546.0250
64	0	64	63	1	63	A	1	330641.280	0.161	546.0250
64	1	64	63	0	63	A	1	330641.280	0.161	546.0250
64	1	64	63	1	63	A	1	330641.280	0.161	546.0250
64	0	64	63	0	63	E	1	330650.364	0.045	545.6658
64	1	64	63	1	63	E	1	330650.364	0.045	545.6658
53	23	31	52	23	30	A	0	330660.576	-0.064	452.3586
53	23	30	52	23	29	A	0	330660.576	-0.065	452.3586
53	23	31	52	23	30	E	0	330663.396	0.159	452.3457
52	13	40	51	13	39	E	0	330696.737	-0.003	377.8424
52	13	40	51	13	39	A	0	330700.238	-0.060	377.8392
33	12	22	32	11	22	E	0	330723.419	-0.003	199.9665
64	0	64	63	0	63	A	0	330734.277	-0.148	417.4038
64	0	64	63	1	63	A	0	330734.277	-0.148	417.4038
64	1	64	63	0	63	A	0	330734.277	-0.148	417.4038
64	1	64	63	1	63	A	0	330734.277	-0.148	417.4038
64	0	64	63	0	63	E	0	330734.277	0.145	417.4150
64	1	64	63	1	63	E	0	330734.277	0.145	417.4150
62	30	32	62	29	33	A	0	330749.574	-0.079	629.9565
62	30	33	62	29	34	A	0	330749.574	-0.079	629.9565
25	16	9	24	15	9	E	0	330771.253	-0.083	171.8138

J'	K_a'	K_c'	J''	K_a''	K_c''	Sym.	$v_{t_{CH_3}}$	$\nu_{Obs.}$	$\nu_{Obs.} - \nu_{Calc.}$	E_{low}
25	16	10	24	15	10	E	0	330781.043	-0.020	171.8003
25	16	9	24	15	10	A	0	330816.426	-0.038	171.8088
25	16	10	24	15	9	A	0	330816.426	-0.038	171.8088
33	12	21	32	11	22	A	0	330951.449	-0.042	199.9640
53	12	42	52	12	41	E	0	330996.474	-0.034	384.0114
53	12	42	52	12	41	A	0	331003.202	-0.023	384.0082
61	30	32	61	29	33	E	0	331152.204	-0.121	617.0806
61	30	31	61	29	32	A	0	331169.353	-0.090	617.0924
61	30	32	61	29	33	A	0	331169.353	-0.090	617.0924
53	22	31	52	22	30	A	0	331267.702	-0.076	444.0201
53	22	32	52	22	31	A	0	331267.702	-0.076	444.0201
53	22	32	52	22	31	E	0	331270.891	-0.050	444.0075
27	15	12	26	14	12	E	0	331448.187	-0.058	176.8680
27	15	13	26	14	13	E	0	331452.141	0.021	176.8544
27	15	13	26	14	12	A	0	331491.193	-0.076	176.8615
27	15	12	26	14	13	A	0	331491.193	-0.076	176.8615
52	16	37	51	16	36	A	0	331526.374	0.038	392.2182
52	16	37	51	16	36	E	0	331545.597	-0.065	392.2167
60	30	31	60	29	32	E	0	331551.119	-0.152	604.4302
60	30	30	60	29	31	A	0	331568.082	-0.011	604.4421
60	30	31	60	29	32	A	0	331568.082	-0.011	604.4421
60	30	30	60	29	31	E	0	331597.255	-0.031	604.4321
33	9	24	32	8	25	E	0	331656.414	0.023	189.9138
31	13	19	30	12	19	E	0	331812.429	-0.030	190.8686
31	13	18	30	12	18	E	0	331821.852	-0.104	190.8816
31	13	19	30	12	18	A	0	331852.682	-0.110	190.8725
31	13	18	30	12	19	A	0	331864.655	-0.035	190.8721
29	14	16	28	13	16	E	0	331882.708	-0.131	183.1939
29	14	15	28	13	15	E	0	331885.169	-0.111	183.2073
29	14	16	28	13	15	A	0	331925.474	0.012	183.1994
59	30	30	59	29	31	E	0	331929.823	-0.105	591.9932
59	30	29	59	29	30	A	0	331946.430	-0.036	592.0052
59	30	30	59	29	31	A	0	331946.430	-0.036	592.0052
59	30	29	59	29	30	E	0	331975.921	-0.060	591.9952
53	21	33	52	21	32	A	0	331978.495	-0.034	436.1011
53	21	32	52	21	31	E	0	331982.163	0.107	436.0981
53	11	42	52	11	41	E	0	331998.662	-0.058	383.9224
52	16	36	51	16	35	E	0	332226.545	-0.032	392.2515
58	30	29	58	29	30	E	0	332288.991	-0.133	579.7694
58	30	28	58	29	29	A	0	332305.314	-0.077	579.7815
58	30	29	58	29	30	A	0	332305.314	-0.077	579.7815
58	30	28	58	29	29	E	0	332335.010	-0.201	579.7714
50	13	37	49	13	36	A	0	332376.112	-0.087	357.8821
50	13	37	49	13	36	E	0	332381.906	-0.063	357.8881
57	30	28	57	29	29	E	0	332629.576	-0.080	567.7584
57	30	27	57	29	28	A	0	332645.593	-0.072	567.7705
57	30	28	57	29	29	A	0	332645.593	-0.072	567.7705
57	30	27	57	29	28	E	0	332675.682	-0.090	567.7604
53	20	34	52	20	33	A	0	332822.771	0.045	428.6110
56	30	27	56	29	28	E	0	332952.180	-0.108	555.9599
56	30	26	56	29	27	A	0	332967.990	-0.061	555.9721
56	30	27	56	29	28	A	0	332967.990	-0.061	555.9721
56	30	26	56	29	27	E	0	332998.347	-0.082	555.9620
51	12	39	50	12	38	E	0	333006.771	-0.034	366.0307
35	11	24	34	10	25	E	0	333011.445	-0.081	210.5414
51	12	39	50	12	38	A	0	333023.157	0.026	366.0249
35	11	24	34	10	25	A	0	333027.655	-0.041	210.5355
52	15	38	51	15	37	A	0	333099.072	-0.055	387.0820
52	15	38	51	15	37	E	0	333106.439	-0.053	387.0830
52	14	39	51	14	38	A	0	333227.011	-0.060	382.3751
52	14	39	51	14	38	E	0	333228.864	-0.058	382.3775
55	30	25	55	29	26	A	0	333273.245	-0.039	544.3859
55	30	26	55	29	27	A	0	333273.245	-0.039	544.3859
55	30	25	55	29	26	E	0	333303.857	-0.059	544.3757
54	30	25	54	29	26	E	0	333546.720	-0.044	532.9992
54	30	24	54	29	25	A	0	333562.036	-0.036	533.0116
54	30	25	54	29	26	A	0	333562.036	-0.036	533.0116
54	30	24	54	29	25	E	0	333592.878	-0.065	533.0014
53	12	42	52	11	41	E	0	333665.506	-0.060	383.9224
53	12	42	52	11	41	A	0	333683.236	-0.054	383.9188
53	30	24	53	29	25	E	0	333819.892	-0.107	521.8365
53	30	23	53	29	24	A	0	333835.052	-0.042	521.8489
53	30	24	53	29	25	A	0	333835.052	-0.042	521.8489
53	19	35	52	19	34	A	0	333843.765	-0.085	421.5624
53	19	34	52	19	33	A	0	333845.898	-0.060	421.5625
53	19	34	52	19	33	E	0	333848.954	-0.001	421.5628
53	19	35	52	19	34	E	0	333850.478	-0.058	421.5520
53	30	23	53	29	24	E	0	333866.097	-0.095	521.8387
54	32	22	53	32	21	A	0	333903.646	-0.093	556.4297
54	32	23	53	32	22	A	0	333903.646	-0.093	556.4297
54	32	22	53	32	21	E	0	333903.646	-0.027	556.4231
52	30	23	52	29	24	E	0	334078.020	-0.095	510.8851
52	30	22	52	29	23	A	0	334092.962	-0.048	510.8975
52	30	23	52	29	24	A	0	334092.962	-0.048	510.8975
52	30	22	52	29	23	E	0	334124.281	-0.038	510.8873
54	31	24	53	31	23	A	0	334128.725	-0.028	544.5118
54	31	23	53	31	22	A	0	334128.725	-0.028	544.5118
54	31	23	53	31	22	E	0	334128.725	-0.128	544.5039
54	31	24	53	31	23	E	0	334130.123	-0.018	544.4986
51	30	22	51	29	23	E	0	334321.686	-0.062	500.1448
51	30	21	51	29	22	A	0	334336.403	-0.049	500.1573
51	30	22	51	29	23	A	0	334336.403	-0.049	500.1573
51	30	21	51	29	22	E	0	334367.932	-0.028	500.1471
54	30	24	53	30	23	A	0	334376.097	0.040	532.9844
54	30	25	53	30	24	A	0	334376.097	0.040	532.9844
54	30	24	53	30	23	E	0	334376.097	-0.254	532.9753
20	19	1	19	18	1	E	0	334414.209	-0.059	168.0127
20	19	2	19	18	2	E	0	334439.545	-0.007	168.0006
20	19	2	19	18	1	A	0	334461.661	-0.070	168.0124
20	19	1	19	18	2	A	0	334461.661	-0.070	168.0124
50	30	21	50	29	22	E	0	334551.535	0.027	489.6153
58	7	51	57	7	50	A	1	334563.651	-0.046	542.2186
58	7	51	57	8	50	A	1	334563.651	-0.040	542.2186
58	8	51	57	7	50	A	1	334563.651	-0.050	542.2186
58	8	51	57	8	50	A	1	334563.651	-0.043	542.2186
50	30	20	50	29	21	A	0	334565.972	-0.059	489.6278
50	30	21	50	29	22	A	0	334565.972	-0.059	489.6278
57	8	49	56	8	48	A	1	334577.960	-0.055	538.3291
57	8	49	56	9	48	A	1	334577.960	0.168	538.3291
57	9	49	56	8	48	A	1	334577.960	-0.173	538.3291
57	9	49	56	9	48	A	1	334577.960	0.050	538.3291
50	30	20	50	29	21	E	0	334597.724	-0.001	489.6176
45	12	34	44	11	33	E	0	334622.502	-0.091	300.4739
59	6	53	58	6	52	A	1	334636.458	-0.107	545.6324
59	6	53	58	7	52	A	1	334636.458	-0.107	545.6324
59	7	53	58	6	52	A	1	334636.458	-0.107	545.6324

J'	K'_a	K'_c	J''	K''_a	K''_c	Sym.	$v_{t_{CH_3}}$	$\nu_{Obs.}$	$\nu_{Obs.} - \nu_{Calc.}$	E_{low}
59	7	53	58	7	52	A	1	334636.458	-0.107	545.6324
54	29	26	53	29	25	A	0	334649.040	-0.040	521.8489
54	29	25	53	29	24	A	0	334649.040	-0.040	521.8489
54	29	25	53	29	24	E	0	334649.040	-0.560	521.8387
54	29	26	53	29	25	E	0	334650.889	0.006	521.8365
58	7	51	57	7	50	E	1	334707.444	-0.033	542.1132
58	7	51	57	8	50	E	1	334707.444	-0.025	542.1132
58	8	51	57	7	50	E	1	334707.444	-0.038	542.1132
58	8	51	57	8	50	E	1	334707.444	-0.029	542.1132
54	12	42	53	13	41	A	0	334710.428	-0.076	400.0826
56	9	47	55	10	46	A	1	334730.240	0.000	533.9329
60	5	55	59	5	54	A	1	334764.834	-0.212	548.5924
60	5	55	59	6	54	A	1	334764.834	-0.212	548.5924
60	6	55	59	5	54	A	1	334764.834	-0.212	548.5924
60	6	55	59	6	54	A	1	334764.834	-0.212	548.5924
59	6	53	58	6	52	E	1	334765.996	0.099	545.5103
59	6	53	58	7	52	E	1	334765.996	0.099	545.5103
59	7	53	58	6	52	E	1	334765.996	0.099	545.5103
59	7	53	58	7	52	E	1	334765.996	0.099	545.5103
49	30	20	49	29	21	E	0	334767.958	-0.029	479.2963
58	7	51	57	7	50	E	0	334777.500	-0.049	413.8234
58	8	51	57	8	50	E	0	334777.500	-0.045	413.8234
58	7	51	57	7	50	A	0	334782.021	0.018	413.8201
58	7	51	57	8	50	A	0	334782.021	0.025	413.8201
58	8	51	57	7	50	A	0	334782.021	0.014	413.8201
58	8	51	57	8	50	A	0	334782.021	0.021	413.8201
57	8	49	56	8	48	E	0	334805.214	-0.095	409.9460
57	9	49	56	9	48	E	0	334805.214	0.022	409.9460
57	8	49	56	8	48	A	0	334810.187	-0.077	409.9430
57	8	49	56	9	48	A	0	334810.187	0.175	409.9430
57	9	49	56	8	48	A	0	334810.187	-0.212	409.9430
57	9	49	56	9	48	A	0	334810.187	0.040	409.9430
49	30	19	49	29	20	E	0	334814.179	-0.027	479.2987
59	6	53	58	6	52	E	0	334837.480	-0.026	417.2212
59	7	53	58	7	52	E	0	334837.480	-0.026	417.2212
59	6	53	58	6	52	A	0	334841.494	-0.012	417.2174
59	6	53	58	7	52	A	0	334841.494	-0.012	417.2174
59	7	53	58	6	52	A	0	334841.494	-0.012	417.2174
59	7	53	58	7	52	A	0	334841.494	-0.012	417.2174
60	5	55	59	5	54	E	1	334879.977	-0.033	548.4481
60	5	55	59	6	54	E	1	334879.977	-0.033	548.4481
60	6	55	59	5	54	E	1	334879.977	-0.033	548.4481
60	6	55	59	6	54	E	1	334879.977	-0.033	548.4481
56	9	47	55	10	46	E	1	334908.979	-0.002	533.8445
56	9	47	55	9	46	E	1	334915.805	-0.025	533.8443
56	10	47	55	9	46	E	1	334919.623	-0.009	533.8443
61	4	57	60	4	56	A	1	334929.758	-0.169	551.1151
61	4	57	60	5	56	A	1	334929.758	-0.169	551.1151
61	5	57	60	4	56	A	1	334929.758	-0.169	551.1151
61	5	57	60	5	56	A	1	334929.758	-0.169	551.1151
60	5	55	59	5	54	E	0	334952.735	-0.016	420.1613
60	6	55	59	6	54	E	0	334952.735	-0.016	420.1613
60	5	55	59	5	54	A	0	334956.274	-0.025	420.1567
60	5	55	59	6	54	A	0	334956.274	-0.025	420.1567
60	6	55	59	5	54	A	0	334956.274	-0.025	420.1567
60	6	55	59	6	54	A	0	334956.274	-0.025	420.1567
48	30	18	48	29	19	A	0	334985.893	-0.052	469.2003
48	30	19	48	29	20	A	0	334985.893	-0.052	469.2003
56	10	47	55	9	46	A	0	334987.746	-0.019	405.5550
54	11	43	53	12	42	E	0	335009.613	0.021	395.0523
54	11	43	53	12	42	A	0	335011.788	-0.006	395.0493
48	30	18	48	29	19	E	0	335017.999	0.024	469.1901
55	10	45	54	11	44	A	1	335055.209	0.225	528.9844
61	4	57	60	4	56	E	0	335103.395	-0.009	422.6597
61	5	57	60	5	56	E	0	335103.395	-0.009	422.6597
61	4	57	60	4	56	A	0	335106.477	0.007	422.6543
61	4	57	60	5	56	A	0	335106.477	0.007	422.6543
61	5	57	60	4	56	A	0	335106.477	0.007	422.6543
61	5	57	60	5	56	A	0	335106.477	0.007	422.6543
62	3	59	61	3	58	A	1	335118.913	-0.092	553.2133
62	3	59	61	4	58	A	1	335118.913	-0.092	553.2133
62	4	59	61	3	58	A	1	335118.913	-0.092	553.2133
62	4	59	61	4	58	A	1	335118.913	-0.092	553.2133
47	30	18	47	29	19	E	0	335163.309	-0.057	459.2891
55	10	45	54	10	44	A	1	335165.668	0.102	528.9807
47	30	17	47	29	18	A	0	335177.372	-0.032	459.3018
47	30	18	47	29	19	A	0	335177.372	-0.032	459.3018
62	3	59	61	3	58	E	1	335201.383	0.086	553.0062
62	3	59	61	4	58	E	1	335201.383	0.086	553.0062
62	4	59	61	3	58	E	1	335201.383	0.086	553.0062
62	4	59	61	4	58	E	1	335201.383	0.086	553.0062
47	30	17	47	29	18	E	0	335209.632	0.049	459.2915
55	10	45	54	11	44	E	1	335248.555	0.045	528.8954
22	18	4	21	17	4	E	0	335275.151	-0.137	169.7677
62	3	59	61	4	58	E	0	335276.714	0.057	424.7288
62	4	59	61	3	58	E	0	335276.714	0.057	424.7288
62	3	59	61	3	58	A	0	335279.203	0.018	424.7223
62	3	59	61	4	58	A	0	335279.203	0.018	424.7223
62	4	59	61	3	58	A	0	335279.203	0.018	424.7223
62	4	59	61	4	58	A	0	335279.203	0.018	424.7223
22	18	5	21	17	5	E	0	335295.731	0.038	169.7550
55	10	45	54	11	44	E	0	335309.518	-0.066	400.6135
55	10	45	54	11	44	A	0	335315.554	-0.007	400.6107
22	18	4	21	17	5	A	0	335322.605	-0.020	169.7658
22	18	5	21	17	4	A	0	335322.605	-0.020	169.7658
63	2	61	62	2	60	A	1	335324.171	-0.171	554.8971
63	2	61	62	3	60	A	1	335324.171	-0.171	554.8971
63	3	61	62	2	60	A	1	335324.171	-0.171	554.8971
63	3	61	62	3	60	A	1	335324.171	-0.171	554.8971
46	30	16	46	29	17	A	0	335357.252	0.007	449.6131
46	30	17	46	29	18	A	0	335357.252	0.007	449.6131
55	11	45	54	11	44	E	0	335379.427	0.079	400.6135
51	14	37	50	14	36	A	0	335384.204	-0.222	372.1263
63	2	61	62	2	60	E	1	335386.474	0.012	554.6481
63	2	61	62	3	60	E	1	335386.474	0.012	554.6481
63	3	61	62	2	60	E	1	335386.474	0.012	554.6481
63	3	61	62	3	60	E	1	335386.474	0.012	554.6481
46	30	16	46	29	17	E	0	335389.493	-0.072	449.6028
51	14	37	50	14	36	E	0	335400.529	-0.010	372.1303
55	10	45	54	10	44	E	0	335430.136	0.150	400.6095
55	10	45	54	10	44	A	0	335436.609	0.021	400.6066
34	12	23	33	11	22	A	0	335462.459	-0.193	206.9641
63	2	61	62	2	60	E	0	335464.077	0.108	426.3781
63	3	61	62	3	60	E	0	335464.077	0.108	426.3781
63	2	61	62	2	60	A	0	335465.879	0.005	426.3703
63	2	61	62	3	60	A	0	335465.879	0.005	426.3703

J'	K_a'	K_c'	J''	K_a''	K_c''	Sym.	$v_{t_{CH_3}}$	$\nu_{Obs.}$	$\nu_{Obs.} - \nu_{Calc.}$	E_{low}
63	3	61	62	2	60	A	0	335465.879	0.005	426.3703
63	3	61	62	3	60	A	0	335465.879	0.005	426.3703
55	11	45	54	10	44	E	0	335499.769	0.020	400.6095
55	11	45	54	10	44	A	0	335506.715	-0.011	400.6066
45	30	16	45	29	17	E	0	335512.172	-0.061	440.1212
45	30	15	45	29	16	A	0	335526.014	0.024	440.1339
45	30	16	45	29	17	A	0	335526.014	0.024	440.1339
64	1	63	63	2	62	A	1	335540.805	0.051	556.1750
64	2	63	63	1	62	A	1	335540.805	0.051	556.1750
64	1	63	63	1	62	E	1	335579.036	0.186	555.8758
64	2	63	63	2	62	E	1	335579.036	0.186	555.8758
64	1	63	63	1	62	E	0	335659.373	-0.130	427.6155
64	2	63	63	2	62	E	0	335659.373	-0.130	427.6155
64	1	63	63	1	62	A	0	335660.862	0.190	427.6062
64	1	63	63	2	62	A	0	335660.862	0.190	427.6062
64	2	63	63	1	62	A	0	335660.862	0.190	427.6062
64	2	63	63	2	62	A	0	335660.862	0.190	427.6062
44	30	14	44	29	15	A	0	335684.095	-0.039	430.8642
44	30	15	44	29	16	A	0	335684.095	-0.039	430.8642
34	12	22	33	11	22	E	0	335692.783	-0.106	206.9698
44	30	14	44	29	15	E	0	335716.738	0.033	430.8539
65	0	65	64	0	64	A	1	335765.090	0.173	557.0540
65	0	65	64	1	64	A	1	335765.090	0.173	557.0540
65	1	65	64	0	64	A	1	335765.090	0.173	557.0540
65	1	65	64	1	64	A	1	335765.090	0.173	557.0540
65	0	65	64	0	64	E	1	335774.111	0.077	556.6951
65	1	65	64	1	64	E	1	335774.111	0.077	556.6951
43	30	14	43	29	15	E	0	335818.758	0.100	421.7907
43	30	13	43	29	14	A	0	335832.197	0.034	421.8036
43	30	14	43	29	15	A	0	335832.197	0.034	421.8036
65	0	65	64	0	64	A	0	335859.367	-0.124	428.4360
65	0	65	64	1	64	A	0	335859.367	-0.124	428.4360
65	1	65	64	0	64	A	0	335859.367	-0.124	428.4360
65	1	65	64	1	64	A	0	335859.367	-0.124	428.4360
65	0	65	64	0	64	E	0	335859.367	0.167	428.4471
65	1	65	64	1	64	E	0	335859.367	0.167	428.4471
43	30	13	43	29	14	E	0	335864.859	0.013	421.7933
42	30	13	42	29	14	E	0	335957.110	-0.045	412.9390
42	30	12	42	29	13	A	0	335970.493	-0.055	412.9519
42	30	13	42	29	14	A	0	335970.493	-0.055	412.9519
42	30	12	42	29	13	E	0	336003.321	-0.012	412.9416
54	12	43	53	12	42	E	0	336035.564	-0.026	395.0523
54	12	43	53	12	42	A	0	336042.259	-0.172	395.0493
41	30	12	41	29	13	E	0	336086.484	0.029	404.2960
54	25	30	53	25	29	A	0	336096.574	-0.005	481.2645
54	25	29	53	25	28	A	0	336096.574	-0.005	481.2645
41	30	11	41	29	12	A	0	336099.654	-0.086	404.3089
41	30	12	41	29	13	A	0	336099.654	-0.086	404.3089
24	17	7	23	16	7	E	0	336109.217	-0.072	172.7499
24	17	8	23	16	8	E	0	336124.422	-0.047	172.7367
41	30	11	41	29	12	E	0	336132.669	0.049	404.2986
53	13	41	52	13	40	E	0	336135.983	-0.037	388.8733
53	13	41	52	13	40	A	0	336140.712	0.106	388.8701
24	17	8	23	16	7	A	0	336155.820	-0.020	172.7464
24	17	7	23	16	8	A	0	336155.820	-0.020	172.7464
40	30	11	40	29	12	E	0	336206.982	-0.013	395.8615
40	30	10	40	29	11	A	0	336220.157	-0.024	395.8744
40	30	11	40	29	12	A	0	336220.157	-0.024	395.8744
40	30	10	40	29	11	E	0	336253.188	0.041	395.8641
34	12	23	33	11	23	E	0	336286.038	-0.004	206.9360
39	30	10	39	29	11	E	0	336319.169	-0.035	387.6353
39	30	9	39	29	10	A	0	336332.263	-0.031	387.6482
39	30	10	39	29	11	A	0	336332.263	-0.031	387.6482
38	30	9	38	29	10	E	0	336423.592	0.100	379.6172
38	30	8	38	29	9	A	0	336436.488	-0.008	379.6302
38	30	9	38	29	10	A	0	336436.488	-0.008	379.6302
37	30	8	37	29	9	E	0	336520.323	0.060	371.8071
37	30	7	37	29	8	A	0	336533.113	-0.072	371.8200
37	30	8	37	29	9	A	0	336533.113	-0.072	371.8200
54	24	30	53	24	29	A	0	336583.212	-0.133	472.1217
54	24	31	53	24	30	A	0	336583.212	-0.133	472.1217
34	12	22	33	11	23	A	0	336600.922	-0.124	206.9325
54	11	43	53	11	42	E	0	336676.450	0.014	394.9967
34	12	22	33	11	23	E	0	336707.070	-0.135	206.9360
53	17	37	52	17	36	E	0	336710.109	0.252	408.8636
52	15	37	51	15	36	A	0	336729.677	-0.129	387.2935
52	15	37	51	15	36	E	0	336743.027	-0.125	387.2954
34	30	5	34	29	6	E	0	336769.134	-0.143	349.6224
34	30	4	34	29	5	A	0	336781.933	-0.053	349.6354
34	30	5	34	29	6	A	0	336781.933	-0.053	349.6354
34	30	4	34	29	5	E	0	336815.419	0.088	349.6251
53	17	36	52	17	35	E	0	336822.154	0.009	408.8773
53	17	36	52	17	35	A	0	336846.310	-0.139	408.8761
33	30	3	33	29	4	A	0	336852.323	-0.053	342.6552
33	30	4	33	29	5	A	0	336852.323	-0.053	342.6552
26	16	10	25	15	10	E	0	336877.668	-0.093	176.9732
26	16	11	25	15	11	E	0	336887.405	0.023	176.9596
26	16	10	25	15	11	A	0	336922.843	-0.015	176.9681
26	16	11	25	15	10	A	0	336922.843	-0.015	176.9681
52	12	40	51	12	39	E	0	336968.848	0.104	377.1386
54	23	32	53	23	31	A	0	337141.823	-0.037	463.3882
54	23	31	53	23	30	A	0	337141.823	-0.037	463.3882
28	15	13	27	14	13	E	0	337507.389	-0.066	182.4583
28	15	14	27	14	14	E	0	337511.154	0.002	182.4447
28	15	14	27	14	13	A	0	337550.452	0.022	182.4518
28	15	13	27	14	14	A	0	337550.452	0.020	182.4518
32	13	20	31	12	20	E	0	337604.153	-0.028	197.3573
32	13	19	31	12	19	E	0	337613.753	-0.038	197.3702
32	13	20	31	12	19	A	0	337637.659	-0.033	197.3612
32	13	19	31	12	20	A	0	337663.745	0.029	197.3605
54	12	43	53	11	42	E	0	337702.438	0.002	394.9967
54	12	43	53	11	42	A	0	337716.512	0.024	394.9934
54	22	32	53	22	31	A	0	337789.027	-0.202	455.0700
54	22	33	53	22	32	A	0	337789.027	-0.200	455.0700
54	22	33	53	22	32	E	0	337792.632	0.022	455.0575
30	14	17	29	13	17	E	0	337851.919	-0.047	189.2253
30	14	16	29	13	16	E	0	337854.619	-0.106	189.2387
30	14	17	29	13	16	A	0	337894.865	0.122	189.2307
35	10	25	34	9	26	A	0	338446.754	-0.068	207.2204
35	10	25	34	9	26	E	0	338455.178	-0.104	207.2272
53	16	38	52	16	37	A	0	338471.459	-0.049	403.2767
53	16	38	52	16	37	E	0	338485.614	0.060	403.2758
54	21	34	53	21	33	A	0	338548.836	-0.018	447.1747
54	21	33	53	21	32	E	0	338552.763	0.164	447.1719
70	31	40	70	30	41	A	0	338992.315	0.239	751.4997
70	31	39	70	30	40	A	0	338992.315	0.239	751.4997

J'	K'_a	K'_c	J''	K''_a	K''_c	Sym.	$v_{t_{CH_3}}$	$\nu_{Obs.}$	$\nu_{Obs.} - \nu_{Calc.}$	E_{low}
51	13	38	50	13	37	A	0	339160.139	0.282	368.9690
51	13	38	50	13	37	E	0	339160.139	-0.208	368.9751
44	12	33	43	11	32	E	0	339183.850	-0.151	290.7362
53	14	40	52	14	39	A	0	339364.830	0.182	393.4903
53	14	40	52	14	39	E	0	339364.830	-0.279	393.4929
54	20	35	53	20	34	A	0	339454.103	0.143	439.7128
69	31	39	69	30	40	E	0	339537.325	-0.043	736.9215
53	16	37	52	16	36	A	0	339615.626	0.036	403.3329
53	16	37	52	16	36	E	0	339618.651	-0.151	403.3334
59	7	52	58	7	51	A	1	339672.056	-0.050	553.3784
59	7	52	58	8	51	A	1	339672.056	-0.047	553.3784
59	8	52	58	7	51	A	1	339672.056	-0.052	553.3784
59	8	52	58	8	51	A	1	339672.056	-0.048	553.3784
58	8	50	57	8	49	A	1	339673.634	0.011	549.4894
58	8	50	57	9	49	A	1	339673.634	0.129	549.4894
58	9	50	57	8	49	A	1	339673.634	-0.051	549.4894
58	9	50	57	9	49	A	1	339673.634	0.067	549.4894
60	6	54	59	6	53	A	1	339751.868	-0.097	556.7946
60	6	54	59	7	53	A	1	339751.868	-0.097	556.7946
60	7	54	59	6	53	A	1	339751.868	-0.097	556.7946
60	7	54	59	7	53	A	1	339751.868	-0.097	556.7946
57	9	48	56	10	47	A	1	339803.877	0.134	545.0984
57	10	48	56	10	47	A	1	339805.441	-0.029	545.0984
57	9	48	56	9	47	A	1	339806.987	0.095	545.0983
57	10	48	56	9	47	A	1	339808.702	0.083	545.0983
59	7	52	58	7	51	E	1	339813.959	-0.011	553.2779
59	7	52	58	8	51	E	1	339813.959	-0.006	553.2779
59	8	52	58	7	51	E	1	339813.959	-0.013	553.2779
59	8	52	58	8	51	E	1	339813.959	-0.009	553.2779
58	8	50	57	8	49	E	1	339830.586	-0.074	549.4007
58	8	50	57	9	49	E	1	339830.586	0.072	549.4007
58	9	50	57	8	49	E	1	339830.586	-0.152	549.4007
58	9	50	57	9	49	E	1	339830.586	-0.006	549.4007
53	15	39	52	15	38	A	0	339844.766	-0.133	398.1930
53	15	39	52	15	38	E	0	339850.901	-0.043	398.1942
60	6	54	59	6	53	E	1	339879.959	0.009	556.6769
60	6	54	59	7	53	E	1	339879.959	0.009	556.6769
60	7	54	59	6	53	E	1	339879.959	0.009	556.6769
60	7	54	59	7	53	E	1	339879.959	0.009	556.6769
61	5	56	60	5	55	A	1	339884.194	-0.098	559.7589
61	5	56	60	6	55	A	1	339884.194	-0.098	559.7589
61	6	56	60	5	55	A	1	339884.194	-0.098	559.7589
61	6	56	60	6	55	A	1	339884.194	-0.098	559.7589
59	7	52	58	7	51	E	0	339886.427	-0.026	424.9904
59	8	52	58	8	51	E	0	339886.427	-0.024	424.9904
59	7	52	58	7	51	A	0	339890.837	-0.011	424.9872
59	7	52	58	8	51	A	0	339890.837	-0.007	424.9872
59	8	52	58	7	51	A	0	339890.837	-0.013	424.9872
59	8	52	58	8	51	A	0	339890.837	-0.009	424.9872
58	8	50	57	8	49	E	0	339900.910	-0.043	421.1139
58	9	50	57	9	49	E	0	339900.910	0.020	421.1139
58	8	50	57	8	49	A	0	339905.773	-0.052	421.1111
58	8	50	57	9	49	A	0	339905.773	0.082	421.1111
58	9	50	57	8	49	A	0	339905.773	-0.123	421.1111
58	9	50	57	9	49	A	0	339905.773	0.011	421.1111
60	6	54	59	6	53	E	0	339953.714	-0.003	428.3902
60	7	54	59	7	53	E	0	339953.714	-0.003	428.3902
60	6	54	59	6	53	A	0	339957.666	-0.009	428.3865
60	6	54	59	7	53	A	0	339957.666	-0.009	428.3865
60	7	54	59	6	53	A	0	339957.666	-0.009	428.3865
60	7	54	59	7	53	A	0	339957.666	-0.009	428.3865
57	9	48	56	10	47	E	1	339978.799	-0.053	545.0160
57	10	48	56	9	47	E	1	339984.795	0.043	545.0158
62	4	58	61	4	57	A	1	340051.048	-0.213	562.2872
62	4	58	61	5	57	A	1	340051.048	-0.213	562.2872
62	5	58	61	4	57	A	1	340051.048	-0.213	562.2872
62	5	58	61	5	57	A	1	340051.048	-0.213	562.2872
57	10	48	56	9	47	A	0	340056.239	-0.018	416.7288
61	5	56	60	5	55	E	0	340073.067	0.031	431.3341
61	6	56	60	6	55	E	0	340073.067	0.031	431.3341
61	5	56	60	5	55	A	0	340076.588	0.033	431.3297
61	5	56	60	6	55	A	0	340076.588	0.033	431.3297
61	6	56	60	5	55	A	0	340076.588	0.033	431.3297
61	6	56	60	6	55	A	0	340076.588	0.033	431.3297
68	31	38	68	30	39	A	0	340085.220	-0.000	722.5822
68	31	37	68	30	38	A	0	340085.220	-0.000	722.5822
35	12	24	34	11	23	E	0	340094.271	0.009	214.1885
56	10	46	55	11	45	A	1	340114.334	0.058	540.1628
62	4	58	61	4	57	E	1	340150.447	0.177	562.1181
62	4	58	61	5	57	E	1	340150.447	0.177	562.1181
62	5	58	61	4	57	E	1	340150.447	0.176	562.1181
62	5	58	61	5	57	E	1	340150.447	0.177	562.1181
56	11	46	55	10	45	A	1	340214.900	0.151	540.1606
62	4	58	61	4	57	E	0	340225.856	-0.082	433.8375
62	5	58	61	5	57	E	0	340225.856	-0.082	433.8375
62	4	58	61	4	57	A	0	340229.002	0.016	433.8322
62	4	58	61	5	57	A	0	340229.002	0.016	433.8322
62	5	58	61	4	57	A	0	340229.002	0.016	433.8322
62	5	58	61	5	57	A	0	340229.002	0.016	433.8322
35	12	24	34	11	23	A	0	340235.040	-0.084	214.1831
63	3	60	62	3	59	A	1	340241.319	-0.125	564.3917
63	3	60	62	4	59	A	1	340241.319	-0.125	564.3917
63	4	60	62	3	59	A	1	340241.319	-0.125	564.3917
63	4	60	62	4	59	A	1	340241.319	-0.125	564.3917
56	10	46	55	11	45	E	1	340307.214	-0.017	540.0806
63	3	60	62	3	59	E	1	340323.449	0.052	564.1874
63	3	60	62	4	59	E	1	340323.449	0.052	564.1874
63	4	60	62	3	59	E	1	340323.449	0.052	564.1874
63	4	60	62	4	59	E	1	340323.449	0.052	564.1874
56	11	46	55	11	45	E	1	340350.449	-0.077	540.0806
56	10	46	55	11	45	E	0	340370.030	0.081	411.8005
56	10	46	55	11	45	A	0	340375.876	-0.048	411.7979
56	10	46	55	10	45	E	1	340382.437	0.066	540.0781
63	3	60	62	3	59	E	0	340400.442	0.039	435.9124
63	4	60	62	4	59	E	0	340400.442	0.039	435.9124
63	3	60	62	3	59	A	0	340402.936	0.017	435.9060
63	3	60	62	4	59	A	0	340402.936	0.017	435.9060
63	4	60	62	3	59	A	0	340402.936	0.017	435.9060
63	4	60	62	4	59	A	0	340402.936	0.017	435.9060
56	11	46	55	11	45	E	0	340410.057	0.025	411.8005
56	11	46	55	11	45	A	0	340416.129	-0.101	411.7979
56	11	46	55	10	45	E	1	340425.700	0.035	540.0781
55	11	44	54	12	43	E	0	340428.509	0.050	406.2612
55	11	44	54	12	43	A	0	340432.316	-0.064	406.2584
56	10	46	55	10	45	E	0	340439.782	0.070	411.7982
64	2	62	63	2	61	A	1	340447.340	-0.019	566.0824

J'	K'_a	K'_c	J''	K''_a	K''_c	Sym.	$v_{t_{CH_3}}$	$\nu_{Obs.}$	$\nu_{Obs.} - \nu_{Calc.}$	E_{low}
64	2	62	63	3	61	A	1	340447.340	-0.019	566.0824
64	3	62	63	2	61	A	1	340447.340	-0.019	566.0824
64	3	62	63	3	61	A	1	340447.340	-0.019	566.0824
56	11	46	55	10	45	E	0	340479.786	-0.009	411.7982
56	11	46	55	10	45	A	0	340486.389	0.021	411.7956
64	2	62	63	2	61	E	1	340509.384	0.070	565.8354
64	2	62	63	3	61	E	1	340509.384	0.070	565.8354
64	3	62	63	2	61	E	1	340509.384	0.070	565.8354
64	3	62	63	3	61	E	1	340509.384	0.070	565.8354
54	19	36	53	19	35	A	0	340553.125	-0.071	432.6983
54	19	35	53	19	34	A	0	340557.591	0.304	432.6984
21	19	2	20	18	2	E	0	340559.347	-0.006	172.1183
54	19	36	53	19	35	E	0	340561.032	-0.225	432.6881
21	19	3	20	18	3	E	0	340584.662	0.049	172.1063
64	2	62	63	2	61	E	0	340588.426	0.081	437.5680
64	3	62	63	3	61	E	0	340588.426	0.081	437.5680
64	2	62	63	2	61	A	0	340590.227	-0.016	437.5603
64	2	62	63	3	61	A	0	340590.227	-0.016	437.5603
64	3	62	63	2	61	A	0	340590.227	-0.016	437.5603
64	3	62	63	3	61	A	0	340590.227	-0.016	437.5603
67	31	37	67	30	38	A	0	340593.944	0.129	708.4469
67	31	36	67	30	37	A	0	340593.944	0.129	708.4469
21	19	3	20	18	2	A	0	340606.772	-0.039	172.1180
21	19	2	20	18	3	A	0	340606.772	-0.039	172.1180
35	12	23	34	11	23	E	0	340615.805	0.011	214.1885
65	1	64	64	2	63	A	1	340664.132	0.029	567.3675
65	2	64	64	1	63	A	1	340664.132	0.029	567.3675
65	1	64	64	1	63	E	1	340702.292	0.169	567.0695
65	2	64	64	2	63	E	1	340702.292	0.169	567.0695
55	12	44	54	12	43	A	1	340776.499	0.137	534.6230
65	1	64	64	1	63	E	0	340784.033	-0.168	438.8119
65	2	64	64	2	63	E	0	340784.033	-0.168	438.8119
65	1	64	64	1	63	A	0	340785.515	0.148	438.8026
65	1	64	64	2	63	A	0	340785.515	0.148	438.8026
65	2	64	64	1	63	A	0	340785.515	0.148	438.8026
65	2	64	64	2	63	A	0	340785.515	0.148	438.8026
66	0	66	65	0	65	A	1	340888.728	0.202	568.2539
66	0	66	65	1	65	A	1	340888.728	0.202	568.2539
66	1	66	65	0	65	A	1	340888.728	0.202	568.2539
66	1	66	65	1	65	A	1	340888.728	0.202	568.2539
66	0	66	65	0	65	E	1	340897.674	0.114	567.8953
66	1	66	65	1	65	E	1	340897.674	0.114	567.8953
66	0	66	65	0	65	A	0	340984.236	-0.132	439.6390
66	0	66	65	1	65	A	0	340984.236	-0.132	439.6390
66	1	66	65	0	65	A	0	340984.236	-0.132	439.6390
66	1	66	65	1	65	A	0	340984.236	-0.132	439.6390
66	0	66	65	0	65	E	0	340984.236	0.157	439.6502
66	1	66	65	1	65	E	0	340984.236	0.157	439.6502
55	29	27	54	29	26	A	0	340993.588	0.146	533.0116
55	29	26	54	29	25	A	0	340993.588	0.146	533.0116
55	29	26	54	29	25	E	0	340993.588	-0.422	533.0014
55	29	27	54	29	26	E	0	340995.486	0.122	532.9992
55	12	44	54	12	43	E	1	340999.095	-0.069	534.5345
55	12	44	54	12	43	E	0	341052.081	0.129	406.2612
55	12	44	54	12	43	A	0	341058.817	0.008	406.2584
66	31	36	66	30	37	E	0	341065.219	-0.097	694.5147
66	31	36	66	30	37	A	0	341078.390	-0.053	694.5266
66	31	35	66	30	36	A	0	341078.390	-0.053	694.5266
66	31	35	66	30	36	E	0	341109.769	-0.140	694.5177
36	11	25	35	10	26	E	0	341236.442	-0.018	217.9781
55	28	27	54	28	26	A	0	341314.038	0.028	522.2797
55	28	28	54	28	27	A	0	341314.038	0.028	522.2797
55	28	28	54	28	27	E	0	341316.232	0.070	522.2680
56	13	43	55	14	42	A	0	341397.273	-0.003	428.0388
23	18	5	22	17	5	E	0	341414.629	-0.033	174.2907
23	18	6	22	17	6	E	0	341435.045	0.021	174.2780
54	13	42	53	13	41	E	0	341440.190	0.031	400.0856
56	13	43	55	14	42	E	0	341443.802	0.238	428.0413
54	13	42	53	13	41	A	0	341445.453	-0.126	400.0826
55	11	44	54	11	43	E	0	341454.431	-0.026	406.2270
23	18	5	22	17	6	A	0	341461.906	-0.082	174.2888
23	18	6	22	17	5	A	0	341461.906	-0.082	174.2888
65	31	35	65	30	36	A	0	341540.178	0.116	680.8210
65	31	34	65	30	35	A	0	341540.178	0.116	680.8210
55	27	29	54	27	28	A	0	341671.661	-0.114	511.9445
55	27	28	54	27	27	A	0	341671.661	-0.114	511.9445
55	27	28	54	27	27	E	0	341674.305	0.139	511.9337
54	18	37	53	18	36	A	0	341914.588	-0.042	426.1512
54	18	37	53	18	36	E	0	341937.782	-0.004	426.1429
54	18	36	53	18	35	E	0	341942.326	0.144	426.1543
54	18	36	53	18	35	A	0	341953.067	-0.151	426.1527
64	31	34	64	30	35	E	0	341967.015	-0.142	667.3177
64	31	34	64	30	35	A	0	341979.613	0.025	667.3298
64	31	33	64	30	34	A	0	341979.613	0.025	667.3298
55	26	29	54	26	28	A	0	342073.440	-0.007	502.0087
55	26	30	54	26	29	A	0	342073.440	-0.007	502.0087
55	12	44	54	11	43	E	0	342077.956	0.005	406.2270
25	17	8	24	16	8	E	0	342234.417	-0.092	177.6939
25	17	9	24	16	9	E	0	342249.616	-0.001	177.6806
55	12	43	54	13	42	A	0	342266.577	0.006	411.4720
55	12	43	54	13	42	E	0	342277.854	0.023	411.4748
25	17	9	24	16	8	A	0	342281.019	-0.019	177.6904
25	17	8	24	16	9	A	0	342281.019	-0.019	177.6904
35	12	23	34	11	24	A	0	342306.494	-0.064	214.1262
63	31	33	63	30	34	E	0	342385.436	-0.356	654.0404
63	31	33	63	30	34	A	0	342397.888	-0.007	654.0526
63	31	32	63	30	33	A	0	342397.888	-0.007	654.0526
63	31	32	63	30	33	E	0	342430.437	-0.028	654.0436
55	25	31	54	25	30	A	0	342527.394	-0.052	492.4755
55	25	30	54	25	29	A	0	342527.394	-0.052	492.4755
62	31	32	62	30	33	E	0	342783.910	-0.123	640.9768
62	31	32	62	30	33	A	0	342795.819	-0.005	640.9891
62	31	31	62	30	32	A	0	342795.819	-0.005	640.9891
27	16	11	26	15	11	E	0	342974.080	-0.070	182.3430
27	16	12	26	15	12	E	0	342983.638	-0.015	182.3295
27	16	11	26	15	12	A	0	343019.192	-0.022	182.3379
27	16	12	26	15	11	A	0	343019.192	-0.022	182.3379
55	24	31	54	24	30	A	0	343044.490	-0.036	483.3489
55	24	32	54	24	31	A	0	343044.490	-0.036	483.3489
61	31	31	61	30	32	E	0	343162.695	0.007	628.1267
61	31	31	61	30	32	A	0	343174.167	-0.013	628.1390
61	31	30	61	30	31	A	0	343174.167	-0.013	628.1390
61	31	30	61	30	31	E	0	343207.422	0.030	628.1300
33	13	21	32	12	21	E	0	343337.308	-0.004	204.0667
33	13	20	32	12	20	E	0	343345.401	-0.021	204.0796
33	13	21	32	12	20	A	0	343355.743	-0.020	204.0709

J'	K'_a	K'_c	J''	K''_a	K''_c	Sym.	$v_{t_{CH_3}}$	$\nu_{Obs.}$	$\nu_{Obs.} - \nu_{Calc.}$	E_{low}
33	13	20	32	12	21	A	0	343410.789	-0.065	204.0694
60	31	30	60	30	31	E	0	343522.266	-0.264	615.4896
60	31	30	60	30	31	A	0	343533.643	-0.093	615.5020
60	31	29	60	30	30	A	0	343533.643	-0.093	615.5020
29	15	14	28	14	14	E	0	343549.011	-0.052	188.2614
29	15	15	28	14	15	E	0	343552.579	0.023	188.2478
60	31	29	60	30	30	E	0	343567.194	-0.051	615.4929
29	15	15	28	14	14	A	0	343591.940	-0.044	188.2548
54	17	38	53	17	37	A	0	343591.940	0.003	420.0990
54	17	38	53	17	37	E	0	343623.679	-0.099	420.0951
55	23	33	54	23	32	A	0	343638.679	-0.029	474.6341
55	23	32	54	23	31	A	0	343638.679	-0.029	474.6341
55	23	33	54	23	32	E	0	343641.854	0.180	474.6214
43	12	32	42	11	31	E	0	343662.086	-0.114	281.2068
43	12	32	42	11	31	A	0	343756.260	0.012	281.1995
52	14	38	51	14	37	A	0	343768.891	-0.071	383.3135
52	14	38	51	14	37	E	0	343783.225	-0.079	383.3181
31	14	18	30	13	18	E	0	343789.953	0.006	195.4726
31	14	17	30	13	17	E	0	343793.030	-0.042	195.4860
31	14	18	30	13	17	A	0	343833.129	0.336	195.4780
59	31	29	59	30	30	A	0	343875.082	-0.154	603.0778
59	31	28	59	30	29	A	0	343875.082	-0.154	603.0778
54	17	37	53	17	36	E	0	343875.082	0.398	420.1125
54	17	37	53	17	36	A	0	343891.980	0.150	420.1121
58	31	28	58	30	29	E	0	344188.494	-0.229	590.8534
58	31	28	58	30	29	A	0	344199.379	-0.017	590.8660
58	31	27	58	30	28	A	0	344199.379	-0.017	590.8660
58	31	27	58	30	28	E	0	344233.269	-0.179	590.8569
36	12	25	35	11	24	E	0	344404.241	-0.049	221.6495
57	31	27	57	30	28	E	0	344496.420	-0.058	578.8538
57	31	27	57	30	28	A	0	344506.820	-0.083	578.8664
57	31	26	57	30	27	A	0	344506.820	-0.083	578.8664
36	12	25	35	11	24	A	0	344508.745	-0.101	221.6441
57	31	26	57	30	27	E	0	344541.185	-0.019	578.8573
59	8	51	58	8	50	A	1	344771.136	-0.013	560.8197
59	8	51	58	9	50	A	1	344771.136	0.049	560.8197
59	9	51	58	8	50	A	1	344771.136	-0.046	560.8197
59	9	51	58	9	50	A	1	344771.136	0.016	560.8197
60	7	53	59	7	52	A	1	344781.280	-0.044	564.7087
60	7	53	59	8	52	A	1	344781.280	-0.042	564.7087
60	8	53	59	7	52	A	1	344781.280	-0.045	564.7087
60	8	53	59	8	52	A	1	344781.280	-0.043	564.7087
56	31	26	56	30	27	A	0	344798.341	-0.081	567.0787
56	31	25	56	30	26	A	0	344798.341	-0.081	567.0787
56	31	25	56	30	26	E	0	344832.919	-0.038	567.0696
54	12	42	53	12	41	E	0	344835.744	-0.017	399.7485
61	6	55	60	6	54	A	1	344867.548	-0.096	568.1275
61	6	55	60	7	54	A	1	344867.548	-0.096	568.1275
61	7	55	60	6	54	A	1	344867.548	-0.096	568.1275
61	7	55	60	7	54	A	1	344867.548	-0.096	568.1275
58	9	49	57	10	48	A	1	344880.738	0.049	556.4331
58	10	49	57	9	48	A	1	344883.370	0.013	556.4330
60	7	53	59	7	52	E	1	344921.404	0.086	564.6129
60	7	53	59	8	52	E	1	344921.404	0.089	564.6129
60	8	53	59	7	52	E	1	344921.404	0.085	564.6129
60	8	53	59	8	52	E	1	344921.404	0.087	564.6129
59	8	51	58	8	50	E	1	344925.544	-0.008	560.7362
59	8	51	58	9	50	E	1	344925.544	0.070	560.7362
59	9	51	58	8	50	E	1	344925.544	-0.049	560.7362
59	9	51	58	9	50	E	1	344925.544	0.029	560.7362
61	6	55	60	6	54	E	1	344994.416	0.111	568.0141
61	6	55	60	7	54	E	1	344994.416	0.111	568.0141
61	7	55	60	6	54	E	1	344994.416	0.111	568.0141
61	7	55	60	7	54	E	1	344994.416	0.111	568.0141
60	8	53	59	8	52	E	0	344996.187	-0.012	436.3278
59	8	51	58	8	50	E	0	344998.606	0.028	432.4518
59	9	51	58	9	50	E	0	344998.606	0.061	432.4518
60	7	53	59	7	52	A	0	345000.522	-0.016	436.3248
60	7	53	59	8	52	A	0	345000.522	-0.014	436.3248
60	8	53	59	7	52	A	0	345000.522	-0.017	436.3248
60	8	53	59	8	52	A	0	345000.522	-0.015	436.3248
62	5	57	61	5	56	A	1	345003.376	-0.181	571.0963
62	5	57	61	6	56	A	1	345003.376	-0.181	571.0963
62	6	57	61	5	56	A	1	345003.376	-0.181	571.0963
62	6	57	61	6	56	A	1	345003.376	-0.181	571.0963
58	9	49	57	10	48	E	1	345052.207	0.004	556.3565
58	10	49	57	9	48	E	1	345055.568	0.117	556.3564
61	6	55	60	6	54	A	0	345074.270	0.124	439.7263
61	6	55	60	7	54	A	0	345074.270	0.124	439.7263
61	7	55	60	6	54	A	0	345074.270	0.124	439.7263
61	7	55	60	7	54	A	0	345074.270	0.124	439.7263
62	5	57	61	5	56	E	1	345116.715	0.021	570.9596
62	5	57	61	6	56	E	1	345116.715	0.021	570.9596
62	6	57	61	5	56	E	1	345116.715	0.021	570.9596
62	6	57	61	6	56	E	1	345116.715	0.021	570.9596
57	10	47	56	11	46	A	1	345168.669	0.104	551.5090
63	4	59	62	4	58	A	1	345172.344	-0.142	573.6301
63	4	59	62	5	58	A	1	345172.344	-0.142	573.6301
63	5	59	62	4	58	A	1	345172.344	-0.142	573.6301
63	5	59	62	5	58	A	1	345172.344	-0.142	573.6301
52	13	39	51	13	38	E	0	345190.243	0.177	380.2883
62	5	57	61	5	56	E	0	345193.388	0.036	442.6777
62	6	57	61	6	56	E	0	345193.388	0.036	442.6777
62	5	57	61	5	56	A	0	345196.873	0.030	442.6734
62	5	57	61	6	56	A	0	345196.873	0.030	442.6734
62	6	57	61	5	56	A	0	345196.873	0.030	442.6734
62	6	57	61	6	56	A	0	345196.873	0.030	442.6734
57	11	47	56	10	46	A	1	345226.066	0.114	551.5078
63	4	59	62	4	58	E	1	345270.912	-0.002	573.4643
63	4	59	62	5	58	E	1	345270.912	-0.002	573.4643
63	5	59	62	4	58	E	1	345270.912	-0.002	573.4643
63	5	59	62	5	58	E	1	345270.912	-0.002	573.4643
54	31	24	54	30	25	E	0	345325.963	-0.317	544.1252
63	4	59	62	4	58	E	0	345348.348	-0.025	445.1863
63	5	59	62	5	58	E	0	345348.348	-0.025	445.1863
63	4	59	62	4	58	A	0	345351.409	0.006	445.1810
63	4	59	62	5	58	A	0	345351.409	0.006	445.1810
63	5	59	62	4	58	A	0	345351.409	0.006	445.1810
63	5	59	62	5	58	A	0	345351.409	0.006	445.1810
57	10	47	56	11	46	E	1	345359.272	0.140	551.4334
64	3	61	63	3	60	A	1	345363.517	-0.200	575.7409
64	3	61	63	4	60	A	1	345363.517	-0.200	575.7409
64	4	61	63	3	60	A	1	345363.517	-0.200	575.7409
64	4	61	63	4	60	A	1	345363.517	-0.200	575.7409
54	31	23	54	30	24	E	0	345370.822	-0.171	544.1289
57	10	47	56	10	46	E	1	345402.413	-0.014	551.4320

J'	K'_a	K'_c	J''	K''_a	K''_c	Sym.	$v_{t_{CH_3}}$	$\nu_{Obs.}$	$\nu_{Obs.} - \nu_{Calc.}$	E_{low}
54	16	39	53	16	38	A	0	345409.454	-0.029	414.5669
54	16	39	53	16	38	E	0	345420.375	-0.025	414.5665
57	10	47	56	11	46	E	0	345424.323	-0.040	423.1554
57	11	47	56	10	46	E	1	345427.148	-0.029	551.4320
57	10	47	56	11	46	A	0	345430.352	0.078	423.1530
64	3	61	63	3	60	E	1	345445.382	0.046	575.5393
64	3	61	63	4	60	E	1	345445.382	0.046	575.5393
64	4	61	63	3	60	E	1	345445.382	0.046	575.5393
64	4	61	63	4	60	E	1	345445.382	0.046	575.5393
57	11	47	56	11	46	E	0	345447.039	-0.175	423.1554
57	11	47	56	11	46	A	0	345453.245	-0.011	423.1530
57	10	47	56	10	46	E	0	345464.510	0.063	423.1541
57	11	47	56	10	46	E	0	345487.269	-0.027	423.1541
57	11	47	56	10	46	A	0	345493.611	0.049	423.1517
64	3	61	63	3	60	E	0	345524.009	0.021	447.2669
64	4	61	63	4	60	E	0	345524.009	0.021	447.2669
64	3	61	63	3	60	A	0	345526.514	0.019	447.2606
64	3	61	63	4	60	A	0	345526.514	0.019	447.2606
64	4	61	63	3	60	A	0	345526.514	0.019	447.2606
64	4	61	63	4	60	A	0	345526.514	0.019	447.2606
65	2	63	64	2	62	A	1	345570.050	-0.139	577.4385
65	2	63	64	3	62	A	1	345570.050	-0.139	577.4385
65	3	63	64	2	62	A	1	345570.050	-0.139	577.4385
65	3	63	64	3	62	A	1	345570.050	-0.139	577.4385
53	31	23	53	30	24	A	0	345583.286	-0.050	532.9844
53	31	22	53	30	23	A	0	345583.286	-0.050	532.9844
53	31	22	53	30	23	E	0	345618.534	0.041	532.9753
65	2	63	64	2	62	E	1	345632.060	0.079	577.1936
65	3	63	64	3	62	E	1	345632.060	0.079	577.1936
56	11	45	55	12	44	E	0	345682.727	0.012	417.6375
56	11	45	55	12	44	A	0	345687.668	-0.009	417.6349
65	2	63	64	2	62	E	0	345712.508	-0.028	448.9288
65	3	63	64	3	62	E	0	345712.508	-0.028	448.9288
65	2	63	64	2	62	A	0	345714.375	-0.054	448.9211
65	2	63	64	3	62	A	0	345714.375	-0.054	448.9211
65	3	63	64	2	62	A	0	345714.375	-0.054	448.9211
65	3	63	64	3	62	A	0	345714.375	-0.054	448.9211
66	1	65	65	2	64	A	1	345787.308	0.050	578.7308
66	2	65	65	1	64	A	1	345787.308	0.050	578.7308
52	31	22	52	30	23	A	0	345816.970	-0.112	522.0417
52	31	21	52	30	22	A	0	345816.970	-0.112	522.0417
66	1	65	65	1	64	E	1	345825.275	0.070	578.4341
66	2	65	65	2	64	E	1	345825.275	0.070	578.4341
20	20	0	19	19	0	E	0	345838.271	-0.027	175.0637
20	20	0	19	19	1	A	0	345885.167	-0.065	175.0650
20	20	1	19	19	0	A	0	345885.167	-0.065	175.0650
66	1	65	65	1	64	E	0	345908.554	-0.153	450.1792
66	2	65	65	2	64	E	0	345908.554	-0.153	450.1792
66	1	65	65	1	64	A	0	345910.058	0.187	450.1700
66	1	65	65	2	64	A	0	345910.058	0.187	450.1700
66	2	65	65	1	64	A	0	345910.058	0.187	450.1700
66	2	65	65	2	64	A	0	345910.058	0.187	450.1700
56	12	45	55	12	44	E	1	346001.704	0.072	545.9091
67	0	67	66	0	66	A	1	346012.174	0.232	579.6248
67	0	67	66	1	66	A	1	346012.174	0.232	579.6248
67	1	67	66	0	66	A	1	346012.174	0.232	579.6248
67	1	67	66	1	66	A	1	346012.174	0.232	579.6248
67	0	67	66	0	66	E	1	346021.007	0.111	579.2665
67	1	67	66	1	66	E	1	346021.007	0.111	579.2665
51	31	21	51	30	22	E	0	346028.582	-0.071	511.2965
51	31	21	51	30	22	A	0	346037.774	-0.052	511.3095
51	31	20	51	30	21	A	0	346037.774	-0.052	511.3095
67	0	67	66	0	66	A	0	346108.936	-0.117	451.0130
67	0	67	66	1	66	A	0	346108.936	-0.117	451.0130
67	1	67	66	0	66	A	0	346108.936	-0.117	451.0130
67	1	67	66	1	66	A	0	346108.936	-0.117	451.0130
67	0	67	66	0	66	E	0	346108.936	0.169	451.0242
67	1	67	66	1	66	E	0	346108.936	0.169	451.0242
50	31	20	50	30	21	E	0	346236.944	-0.166	500.7747
50	31	20	50	30	21	A	0	346246.067	-0.043	500.7877
50	31	19	50	30	20	A	0	346246.067	-0.043	500.7877
56	11	45	55	11	44	E	1	346266.518	0.153	545.8868
50	31	19	50	30	20	E	0	346281.807	0.027	500.7786
49	31	19	49	30	20	E	0	346433.587	-0.029	490.4630
49	31	19	49	30	20	A	0	346442.391	-0.062	490.4761
49	31	18	49	30	19	A	0	346442.391	-0.062	490.4761
54	15	40	53	15	39	A	0	346474.162	0.039	409.5290
54	15	40	53	15	39	E	0	346478.597	-0.319	409.5304
48	31	18	48	30	19	E	0	346618.563	-0.116	480.3611
48	31	18	48	30	19	A	0	346627.515	0.155	480.3743
48	31	17	48	30	18	A	0	346627.515	0.155	480.3743
55	13	43	54	13	42	E	0	346635.945	0.007	411.4748
55	13	43	54	13	42	A	0	346641.983	-0.002	411.4720
48	31	17	48	30	18	E	0	346663.261	-0.056	480.3651
56	12	45	55	11	44	E	0	346680.842	0.075	417.6167
56	12	45	55	11	44	A	0	346690.488	-0.007	417.6140
22	19	3	21	18	3	E	0	346703.072	-0.053	176.4311
22	19	4	21	18	4	E	0	346728.403	0.048	176.4191
22	19	4	21	18	3	A	0	346750.524	-0.053	176.4308
22	19	3	21	18	4	A	0	346750.524	-0.053	176.4308
56	31	26	55	31	25	A	0	346765.068	0.131	567.0131
56	31	25	55	31	24	A	0	346765.068	0.131	567.0131
56	31	25	55	31	24	E	0	346765.068	-0.012	567.0052
56	31	26	55	31	25	E	0	346766.529	0.007	567.0000
47	31	17	47	30	18	E	0	346792.845	0.059	470.4690
47	31	17	47	30	18	A	0	346801.271	-0.052	470.4821
47	31	16	47	30	17	A	0	346801.271	-0.052	470.4821
46	31	16	46	30	17	E	0	346956.391	-0.022	460.7862
46	31	16	46	30	17	A	0	346964.790	-0.021	460.7994
46	31	15	46	30	16	A	0	346964.790	-0.021	460.7994
46	31	15	46	30	16	E	0	347001.128	0.113	460.7902
56	30	26	55	30	25	A	0	347041.291	0.185	555.5027
56	30	27	55	30	26	A	0	347041.291	0.185	555.5027
56	30	26	55	30	25	E	0	347041.291	-0.179	555.4936
45	31	15	45	30	16	E	0	347110.027	0.008	451.3127
45	31	15	45	30	16	A	0	347118.244	-0.044	451.3259
45	31	14	45	30	15	A	0	347118.244	-0.044	451.3259
54	16	38	53	16	37	A	0	347217.971	-0.097	414.6613
54	16	38	53	16	37	E	0	347226.071	-0.162	414.6619
44	31	14	44	30	15	A	0	347262.109	-0.088	442.0614
44	31	13	44	30	14	A	0	347262.109	-0.088	442.0614
55	19	37	54	19	36	A	0	347294.487	-0.112	444.0579
44	31	13	44	30	14	E	0	347298.708	0.095	442.0522
55	19	37	54	19	36	E	0	347304.785	-0.058	444.0480
43	31	13	43	30	14	E	0	347388.914	-0.024	432.9925
43	31	13	43	30	14	A	0	347396.958	-0.013	433.0057

J'	K'_a	K'_c	J''	K''_a	K''_c	Sym.	$v_{t_{CH_3}}$	$\nu_{Obs.}$	$\nu_{Obs.} - \nu_{Calc.}$	E_{low}
43	31	12	43	30	13	A	0	347396.958	-0.013	433.0057
42	31	12	42	30	13	E	0	347515.117	0.015	424.1453
42	31	12	42	30	13	A	0	347523.037	0.010	424.1586
42	31	11	42	30	12	A	0	347523.037	0.010	424.1586
24	18	6	23	17	6	E	0	347550.691	-0.050	179.0218
42	12	31	41	11	30	E	0	347554.030	-0.097	271.9083
42	31	11	42	30	12	E	0	347559.760	0.138	424.1494
24	18	7	23	17	7	E	0	347570.907	-0.146	179.0091
24	18	6	23	17	7	A	0	347598.050	-0.003	179.0199
24	18	7	23	17	6	A	0	347598.050	-0.003	179.0199
41	31	11	41	30	12	E	0	347632.958	0.011	415.5066
41	31	11	41	30	12	A	0	347640.758	-0.014	415.5199
41	31	10	41	30	11	A	0	347640.758	-0.014	415.5199
42	12	31	41	11	30	A	0	347645.804	0.391	271.9012
41	31	10	41	30	11	E	0	347677.453	0.007	415.5107
40	31	10	40	30	11	E	0	347742.833	-0.037	407.0762
40	31	10	40	30	11	A	0	347750.535	-0.064	407.0895
40	31	9	40	30	10	A	0	347750.535	-0.064	407.0895
39	31	9	39	30	10	E	0	347845.285	0.035	398.8537
39	31	8	39	30	9	E	0	347889.701	-0.002	398.8579
37	12	26	36	11	25	E	0	347991.265	0.038	229.3605
37	31	7	37	30	8	A	0	348036.294	-0.045	383.0456
37	31	6	37	30	7	A	0	348036.294	-0.045	383.0456
56	27	30	55	27	29	A	0	348063.947	-0.088	523.3415
56	27	29	55	27	28	A	0	348063.947	-0.088	523.3415
56	27	30	55	27	29	E	0	348065.357	0.074	523.3298
56	27	29	55	27	28	E	0	348066.734	0.162	523.3308
37	31	6	37	30	7	E	0	348073.232	-0.032	383.0364
37	12	26	36	11	25	A	0	348075.740	-0.041	229.3550
36	31	5	36	30	6	E	0	348155.122	-0.053	375.4370
36	12	24	35	11	25	A	0	348160.538	-0.046	221.5450
36	12	24	35	11	25	E	0	348177.209	-0.075	221.5495
35	31	5	35	30	6	E	0	348186.462	-0.138	368.0407
35	31	5	35	30	6	A	0	348193.896	-0.048	368.0541
35	31	4	35	30	5	A	0	348193.896	-0.048	368.0541
34	31	4	34	30	5	A	0	348263.894	0.001	360.8693
34	31	3	34	30	4	A	0	348263.894	0.001	360.8693
26	17	9	25	16	9	E	0	348353.227	-0.059	182.8472
26	17	10	25	16	10	E	0	348368.295	-0.017	182.8339
26	17	10	25	16	9	A	0	348399.781	-0.012	182.8436
26	17	9	25	16	10	A	0	348399.781	-0.012	182.8436
56	26	30	55	26	29	A	0	348489.463	-0.062	513.4191
56	26	31	55	26	30	A	0	348489.463	-0.062	513.4191
55	18	38	54	18	37	A	0	348762.854	-0.014	437.5563
55	18	38	54	18	37	E	0	348793.926	-0.041	437.5488
55	18	37	54	18	36	E	0	348814.118	-0.074	437.5602
55	18	37	54	18	36	A	0	348832.229	-0.039	437.5591
56	12	44	55	13	43	A	0	348966.394	-0.152	423.0347
55	12	43	54	12	42	E	0	348987.750	-0.029	411.2510
34	13	22	33	12	21	A	0	348990.943	-0.030	211.0033
34	13	21	33	12	21	E	0	349005.236	-0.025	211.0113
28	16	12	27	15	12	E	0	349058.988	-0.066	187.9239
28	16	13	27	15	13	E	0	349068.396	-0.025	187.9104
34	13	21	33	12	22	A	0	349104.030	0.034	211.0001
28	16	12	27	15	13	A	0	349104.030	-0.052	187.9188
28	16	13	27	15	12	A	0	349104.030	-0.052	187.9188
56	24	32	55	24	31	A	0	349519.800	-0.138	494.7917
56	24	33	55	24	32	A	0	349519.800	-0.138	494.7917
30	15	15	29	14	15	E	0	349570.810	0.020	194.2778
30	15	16	29	14	16	E	0	349574.073	0.022	194.2643
30	15	16	29	14	15	A	0	349613.614	-0.037	194.2712
32	14	19	31	13	19	E	0	349692.765	0.001	201.9367
32	14	18	31	13	18	E	0	349696.268	-0.035	201.9500
32	14	19	31	13	18	A	0	349735.329	-0.110	201.9420
32	14	18	31	13	19	A	0	349737.044	-0.106	201.9419
60	8	52	59	8	51	A	1	349870.360	-0.004	572.3200
60	8	52	59	9	51	A	1	349870.360	0.029	572.3200
60	9	52	59	8	51	A	1	349870.360	-0.021	572.3200
60	9	52	59	9	51	A	1	349870.360	0.012	572.3200
61	7	54	60	7	53	A	1	349891.198	-0.054	576.2094
61	7	54	60	8	53	A	1	349891.198	-0.054	576.2094
61	8	54	60	7	53	A	1	349891.198	-0.055	576.2094
61	8	54	60	8	53	A	1	349891.198	-0.054	576.2094
59	9	50	58	10	49	A	1	349960.985	0.068	567.9371
59	10	50	58	9	49	A	1	349962.301	-0.067	567.9371
62	6	56	61	6	55	A	1	349983.452	-0.105	579.6311
62	6	56	61	7	55	A	1	349983.452	-0.105	579.6311
62	7	56	61	6	55	A	1	349983.452	-0.105	579.6311
62	7	56	61	7	55	A	1	349983.452	-0.105	579.6311
60	8	52	59	8	51	E	1	350022.122	-0.093	572.2417
60	8	52	59	9	51	E	1	350022.122	-0.052	572.2417
60	9	52	59	8	51	E	1	350022.122	-0.115	572.2417
60	9	52	59	9	51	E	1	350022.122	-0.074	572.2417
61	7	54	60	7	53	E	1	350029.384	-0.033	576.1182
61	7	54	60	8	53	E	1	350029.384	-0.032	576.1182
61	8	54	60	7	53	E	1	350029.384	-0.034	576.1182
61	8	54	60	8	53	E	1	350029.384	-0.032	576.1182
60	8	52	59	8	51	E	0	350098.023	0.073	443.9597
60	9	52	59	9	51	E	0	350098.023	0.091	443.9597
60	8	52	59	8	51	A	0	350102.606	-0.057	443.9572
60	8	52	59	9	51	A	0	350102.606	-0.019	443.9572
60	9	52	59	8	51	A	0	350102.606	-0.077	443.9572
60	9	52	59	9	51	A	0	350102.606	-0.039	443.9572
61	7	54	60	7	53	E	0	350106.719	0.027	447.8356
61	8	54	60	8	53	E	0	350106.719	0.028	447.8356
62	6	56	61	6	55	E	1	350108.932	0.015	579.5218
62	6	56	61	7	55	E	1	350108.932	0.015	579.5218
62	7	56	61	6	55	E	1	350108.932	0.015	579.5218
62	7	56	61	7	55	E	1	350108.932	0.015	579.5218
61	7	54	60	7	53	A	0	350110.950	-0.023	447.8327
61	7	54	60	8	53	A	0	350110.950	-0.022	447.8327
61	8	54	60	7	53	A	0	350110.950	-0.024	447.8327
61	8	54	60	8	53	A	0	350110.950	-0.023	447.8327
63	5	58	62	5	57	A	1	350122.563	-0.254	582.6043
63	5	58	62	6	57	A	1	350122.563	-0.254	582.6043
63	6	58	62	5	57	A	1	350122.563	-0.254	582.6043
63	6	58	62	6	57	A	1	350122.563	-0.254	582.6043
59	9	50	58	10	49	E	1	350128.975	0.058	567.8663
59	10	50	58	9	49	E	1	350130.586	-0.109	567.8662
56	23	34	55	23	33	A	0	350151.706	-0.096	486.0966
56	23	33	55	23	32	A	0	350151.706	-0.097	486.0966
62	6	56	61	6	55	E	0	350187.003	0.009	451.2402
62	7	56	61	7	55	E	0	350187.003	0.009	451.2402
62	6	56	61	6	55	A	0	350190.860	-0.012	451.2367
62	6	56	61	7	55	A	0	350190.860	-0.012	451.2367
62	7	56	61	6	55	A	0	350190.860	-0.012	451.2367

J'	K_a'	K_c'	J''	K_a''	K_c''	Sym.	$v_{t_{CH_3}}$	$\nu_{Obs.}$	$\nu_{Obs.} - \nu_{Calc.}$	E_{low}
62	7	56	61	7	55	A	0	350190.860	-0.012	451.2367
59	9	50	58	10	49	A	0	350207.008	0.084	439.5841
59	10	50	58	9	49	A	0	350208.447	-0.110	439.5841
58	10	48	57	11	47	A	1	350222.696	0.100	563.0232
63	5	58	62	5	57	E	1	350235.025	-0.037	582.4715
63	5	58	62	6	57	E	1	350235.025	-0.037	582.4715
63	6	58	62	5	57	E	1	350235.025	-0.037	582.4715
63	6	58	62	6	57	E	1	350235.025	-0.037	582.4715
58	11	48	57	10	47	A	1	350255.309	0.185	563.0226
64	5	60	63	5	59	A	1	350293.463	-0.131	585.1438
64	5	60	63	5	59	A	1	350293.463	-0.131	585.1438
64	5	60	63	5	59	A	1	350293.463	-0.131	585.1438
64	5	60	63	5	59	A	1	350293.463	-0.131	585.1438
63	5	58	62	5	57	E	0	350313.706	0.026	454.1921
63	6	58	62	6	57	E	0	350313.706	0.026	454.1921
63	5	58	62	5	57	A	0	350317.162	0.018	454.1879
63	5	58	62	6	57	A	0	350317.162	0.018	454.1879
63	6	58	62	5	57	A	0	350317.162	0.018	454.1879
63	6	58	62	6	57	A	0	350317.162	0.018	454.1879
64	4	60	63	4	59	E	1	350391.363	-0.085	584.9813
64	4	60	63	5	59	E	1	350391.363	-0.085	584.9813
64	5	60	63	4	59	E	1	350391.363	-0.085	584.9813
64	5	60	63	5	59	E	1	350391.363	-0.085	584.9813
58	10	48	57	11	47	E	1	350409.676	-0.090	562.9542
58	11	48	57	11	47	E	1	350423.761	-0.049	562.9542
58	11	48	57	10	47	E	1	350448.636	0.075	562.9534
64	4	60	63	4	59	E	0	350470.686	-0.014	456.7058
64	5	60	63	5	59	E	0	350470.686	-0.014	456.7058
64	4	60	63	4	59	A	0	350473.676	-0.036	456.7007
64	4	60	63	5	59	A	0	350473.676	-0.036	456.7007
64	5	60	63	4	59	A	0	350473.676	-0.036	456.7007
64	5	60	63	5	59	A	0	350473.676	-0.036	456.7007
58	10	48	57	11	47	E	0	350477.968	-0.038	434.6783
58	10	48	57	11	47	A	0	350483.742	-0.077	434.6761
53	13	40	52	13	39	A	0	350483.742	0.209	391.7966
65	3	62	64	3	61	A	1	350485.619	-0.199	587.2610
65	3	62	64	4	61	A	1	350485.619	-0.199	587.2610
65	4	62	64	3	61	A	1	350485.619	-0.199	587.2610
65	4	62	64	4	61	A	1	350485.619	-0.199	587.2610
58	11	48	57	11	47	E	0	350491.135	0.198	434.6783
58	11	48	57	10	47	E	0	350513.842	0.054	434.6775
58	11	48	57	10	47	A	0	350519.850	0.043	434.6753
65	3	62	64	3	61	E	1	350567.139	0.031	587.0621
65	3	62	64	4	61	E	1	350567.139	0.031	587.0621
65	4	62	64	3	61	E	1	350567.139	0.031	587.0621
65	4	62	64	4	61	E	1	350567.139	0.031	587.0621
65	3	62	64	3	61	E	0	350647.442	0.032	458.7924
65	4	62	64	4	61	E	0	350647.442	0.032	458.7924
65	3	62	64	3	61	A	0	350649.933	0.027	458.7861
65	3	62	64	4	61	A	0	350649.933	0.027	458.7861
65	4	62	64	3	61	A	0	350649.933	0.027	458.7861
65	4	62	64	4	61	A	0	350649.933	0.027	458.7861
38	12	27	37	11	26	A	0	350681.954	-0.005	237.3245
66	2	64	65	2	63	A	1	350692.766	-0.060	588.9654
66	2	64	65	3	63	A	1	350692.766	-0.060	588.9654
66	3	64	65	2	63	A	1	350692.766	-0.060	588.9654
66	3	64	65	3	63	A	1	350692.766	-0.060	588.9654
66	2	64	65	2	63	E	1	350754.515	0.057	588.7226
66	3	64	65	3	63	E	1	350754.515	0.057	588.7226
66	2	64	65	2	63	E	0	350836.676	0.136	460.4605
66	3	64	65	3	63	E	0	350836.676	0.136	460.4605
57	11	46	56	12	45	E	0	350836.676	-0.240	429.1807
66	2	64	65	2	63	A	0	350838.415	-0.013	460.4529
66	2	64	65	3	63	A	0	350838.415	-0.013	460.4529
66	3	64	65	2	63	A	0	350838.415	-0.013	460.4529
66	3	64	65	3	63	A	0	350838.415	-0.013	460.4529
57	11	46	56	12	45	A	0	350842.412	-0.052	429.1784
37	11	26	36	10	27	E	0	350884.145	0.079	225.6261
56	22	35	55	22	34	A	0	350887.101	-0.012	477.8230
67	1	66	66	1	65	A	1	350910.261	0.045	590.2650
67	1	66	66	2	65	A	1	350910.261	0.045	590.2650
67	2	66	66	1	65	A	1	350910.261	0.045	590.2650
67	2	66	66	2	65	A	1	350910.261	0.045	590.2650
67	1	66	66	1	65	E	1	350948.197	0.106	589.9696
67	2	66	66	2	65	E	1	350948.197	0.106	589.9696
53	13	41	52	12	40	E	0	350962.604	-0.026	388.3787
67	1	66	66	1	65	E	0	351032.869	-0.150	461.7175
67	2	66	66	2	65	E	0	351032.869	-0.150	461.7175
67	1	66	66	1	65	A	0	351034.391	0.211	461.7083
67	1	66	66	2	65	A	0	351034.391	0.211	461.7083
67	2	66	66	1	65	A	0	351034.391	0.211	461.7083
67	2	66	66	2	65	A	0	351034.391	0.211	461.7083
55	17	38	54	17	37	E	0	351039.389	-0.028	431.5829
55	17	38	54	17	37	A	0	351046.701	-0.055	431.5831
57	12	46	56	12	45	E	0	351059.624	0.040	429.1807
55	14	42	54	14	41	E	0	351061.372	-0.046	416.3311
57	12	46	56	12	45	A	0	351066.187	-0.074	429.1784
68	0	68	67	0	67	A	1	351135.344	0.180	591.1665
68	0	68	67	1	67	A	1	351135.344	0.180	591.1665
68	1	68	67	0	67	A	1	351135.344	0.180	591.1665
68	1	68	67	1	67	A	1	351135.344	0.180	591.1665
68	0	68	67	0	67	E	1	351144.145	0.109	590.8085
68	1	68	67	1	67	E	1	351144.145	0.109	590.8085
57	11	46	56	11	45	E	0	351211.522	0.050	429.1682
57	11	46	56	11	45	A	0	351219.012	0.160	429.1658
68	0	68	67	0	67	A	0	351233.403	-0.141	462.5580
68	0	68	67	1	67	A	0	351233.403	-0.141	462.5580
68	1	68	67	0	67	A	0	351233.403	-0.141	462.5580
68	1	68	67	1	67	A	0	351233.403	-0.141	462.5580
68	0	68	67	0	67	E	0	351233.403	0.143	462.5691
68	1	68	67	1	67	E	0	351233.403	0.143	462.5691
70	32	38	70	31	39	A	0	351325.263	-0.181	762.8072
70	32	39	70	31	40	A	0	351325.263	-0.181	762.8072
57	12	46	56	11	45	E	0	351434.222	0.082	429.1682
57	12	46	56	11	45	A	0	351442.688	0.039	429.1658
54	13	42	53	12	41	E	0	351545.699	-0.010	399.7485
54	13	42	53	12	41	A	0	351586.447	-0.002	399.7443
57	13	44	56	14	43	A	0	351653.800	-0.026	439.9350
57	13	44	56	14	43	E	0	351686.022	-0.007	439.9373
56	13	44	55	13	43	E	0	351749.924	-0.006	423.0373
56	21	36	55	21	35	A	0	351754.579	0.050	469.9801
56	21	35	55	21	34	E	0	351758.942	0.173	469.9776
53	14	39	52	14	38	A	0	351773.822	-0.020	394.7804
53	14	39	52	14	38	E	0	351784.604	0.036	394.7855
52	13	40	51	12	39	E	0	351795.340	-0.014	377.1386
69	32	37	69	31	38	A	0	351829.294	-0.005	748.2593

J'	K'_a	K'_c	J''	K''_a	K''_c	Sym.	$v_{t_{CH_3}}$	$\nu_{Obs.}$	$\nu_{Obs.} - \nu_{Calc.}$	E_{low}
69	32	38	69	31	39	A	0	351829.294	-0.005	748.2593
40	12	29	39	11	28	E	0	351944.211	-0.053	254.0775
21	20	1	20	19	1	E	0	351983.714	-0.071	179.1675
39	12	28	38	11	27	E	0	351988.142	-0.112	245.5674
21	20	2	20	19	2	E	0	352013.591	0.073	179.1563
40	12	29	39	11	28	A	0	352025.144	0.044	254.0710
21	20	1	20	19	2	A	0	352030.667	-0.050	179.1688
21	20	2	20	19	1	A	0	352030.667	-0.050	179.1688
68	32	36	68	31	37	A	0	352310.041	-0.103	733.9263
68	32	37	68	31	38	A	0	352310.041	-0.103	733.9263
55	16	40	54	16	39	A	0	352316.317	-0.029	426.0885
55	16	40	54	16	39	E	0	352325.306	-0.017	426.0885
68	32	36	68	31	37	E	0	352344.896	-0.013	733.9184
67	32	35	67	31	36	A	0	352768.871	0.003	719.8078
67	32	36	67	31	37	A	0	352768.871	0.003	719.8078
56	20	37	55	20	36	A	0	352795.414	-0.023	462.5807
56	20	36	55	20	35	A	0	352797.046	0.151	462.5808
56	20	36	55	20	35	E	0	352800.425	-0.233	462.5798
56	20	37	55	20	36	E	0	352801.931	0.090	462.5699
23	19	4	22	18	4	E	0	352845.034	-0.049	180.9513
23	19	5	22	18	5	E	0	352870.312	0.033	180.9392
23	19	5	22	18	4	A	0	352892.429	-0.098	180.9510
23	19	4	22	18	5	A	0	352892.429	-0.098	180.9510
55	15	41	54	15	40	A	0	352956.131	-0.055	421.0861
55	15	41	54	15	40	E	0	352959.648	-0.032	421.0877
66	32	34	66	31	35	A	0	353206.367	0.043	705.9037
66	32	35	66	31	36	A	0	353206.367	0.043	705.9037
66	32	34	66	31	35	E	0	353241.829	0.013	705.8959
56	12	44	55	12	43	E	0	353329.765	-0.027	422.8919
56	12	44	55	12	43	A	0	353341.803	-0.157	422.8888
55	13	43	54	12	42	E	0	353345.816	-0.070	411.2510
55	13	43	54	12	42	A	0	353377.096	0.037	411.2473
57	30	27	56	30	26	A	0	353385.734	0.131	567.0787
57	30	28	56	30	27	A	0	353385.734	0.131	567.0787
57	30	27	56	30	26	E	0	353385.734	-0.271	567.0696
57	30	28	56	30	27	E	0	353387.419	-0.110	567.0660
54	15	39	53	15	38	A	0	353609.473	-0.209	410.0355
65	32	33	65	31	34	A	0	353623.397	0.072	692.2135
65	32	34	65	31	35	A	0	353623.397	0.072	692.2135
54	15	39	53	15	38	E	0	353627.352	0.049	410.0384
65	32	33	65	31	34	E	0	353659.066	-0.088	692.2056
25	18	7	24	17	7	E	0	353682.708	-0.093	183.9613
25	18	8	24	17	8	E	0	353702.960	-0.096	183.9486
57	29	29	56	29	28	A	0	353708.259	0.270	555.9721
57	29	28	56	29	27	A	0	353708.259	0.270	555.9721
57	29	28	56	29	27	E	0	353708.259	-0.402	555.9620
57	29	29	56	29	28	E	0	353710.118	-0.042	555.9599
25	18	7	24	17	8	A	0	353730.051	-0.048	183.9594
25	18	8	24	17	7	A	0	353730.051	-0.048	183.9594
64	32	32	64	31	33	A	0	354020.666	0.009	678.7370
64	32	33	64	31	34	A	0	354020.666	0.009	678.7370
64	32	32	64	31	33	E	0	354056.596	-0.209	678.7291
37	12	25	36	11	26	E	0	354300.793	-0.007	229.1930
37	12	25	36	11	26	A	0	354306.072	-0.092	229.1883
63	32	32	63	31	33	E	0	354392.618	-0.103	665.4612
63	32	31	63	31	32	A	0	354399.104	0.033	665.4737
63	32	32	63	31	33	A	0	354399.104	0.033	665.4737
63	32	31	63	31	32	E	0	354435.931	0.411	665.4658
27	17	10	26	16	10	E	0	354464.504	-0.065	188.2102
57	27	31	56	27	30	A	0	354466.592	-0.233	534.9516
57	27	30	56	27	29	A	0	354466.592	-0.233	534.9516
57	27	31	56	27	30	E	0	354468.218	0.054	534.9400
57	27	30	56	27	29	E	0	354469.646	0.133	534.9410
27	17	11	26	16	11	E	0	354479.509	0.008	188.1970
27	17	11	26	16	10	A	0	354511.015	-0.036	188.2066
27	17	10	26	16	11	A	0	354511.015	-0.036	188.2066
35	13	23	34	12	22	A	0	354519.653	-0.080	218.1603
35	13	22	34	12	22	E	0	354573.776	-0.058	218.1673
35	13	23	34	12	23	E	0	354605.518	-0.010	218.1533
35	13	22	34	12	23	A	0	354744.720	-0.018	218.1539
62	32	31	62	31	32	E	0	354753.221	-0.019	652.4109
62	32	30	62	31	31	E	0	354796.097	0.071	652.4156
57	26	31	56	26	30	A	0	354917.262	-0.011	525.0434
57	26	32	56	26	31	A	0	354917.262	-0.011	525.0434
61	8	53	60	8	52	A	1	354971.052	-0.017	583.9905
61	8	53	60	9	52	A	1	354971.052	-0.000	583.9905
61	9	53	60	8	52	A	1	354971.052	-0.026	583.9905
61	9	53	60	9	52	A	1	354971.052	-0.009	583.9905
62	7	55	61	7	54	A	1	355001.770	-0.033	587.8805
62	7	55	61	8	54	A	1	355001.770	-0.032	587.8805
62	8	55	61	7	54	A	1	355001.770	-0.033	587.8805
62	8	55	61	8	54	A	1	355001.770	-0.032	587.8805
60	9	51	59	9	50	A	1	355044.669	-0.022	579.6105
60	9	51	59	10	50	A	1	355044.669	0.488	579.6106
60	10	51	59	9	50	A	1	355044.669	-0.297	579.6105
60	10	51	59	10	50	A	1	355044.669	0.213	579.6106
54	13	41	53	13	40	E	0	355092.324	0.025	403.4932
61	32	30	61	31	31	E	0	355096.061	-0.188	639.5733
63	6	57	62	6	56	A	1	355099.535	-0.128	591.3053
63	6	57	62	7	56	A	1	355099.535	-0.128	591.3053
63	7	57	62	6	56	A	1	355099.535	-0.128	591.3053
63	7	57	62	7	56	A	1	355099.535	-0.128	591.3053
61	32	29	61	31	30	A	0	355102.055	0.043	639.5861
61	32	30	61	31	31	A	0	355102.055	0.043	639.5861
61	8	53	60	8	52	E	1	355120.447	0.003	583.9172
61	8	53	60	9	52	E	1	355120.447	0.025	583.9172
61	9	53	60	8	52	E	1	355120.447	-0.009	583.9172
61	9	53	60	9	52	E	1	355120.447	0.013	583.9172
29	16	13	28	15	13	E	0	355130.877	-0.027	193.7164
62	7	55	61	7	54	E	1	355138.191	0.014	587.7939
62	7	55	61	8	54	E	1	355138.191	0.014	587.7939
62	8	55	61	7	54	E	1	355138.191	0.013	587.7939
62	8	55	61	8	54	E	1	355138.191	0.014	587.7939
29	16	14	28	15	14	E	0	355140.153	0.036	193.7029
29	16	13	28	15	14	A	0	355175.873	-0.021	193.7113
29	16	14	28	15	13	A	0	355175.873	-0.021	193.7113
61	8	53	60	8	52	E	0	355198.792	-0.073	455.6377
61	9	53	60	9	52	E	0	355198.792	-0.064	455.6377
61	8	53	60	8	52	A	0	355203.486	-0.016	455.6354
61	8	53	60	9	52	A	0	355203.486	0.004	455.6354
61	9	53	60	8	52	A	0	355203.486	-0.026	455.6354
61	9	53	60	9	52	A	0	355203.486	-0.006	455.6354
62	7	55	61	7	54	E	0	355217.830	-0.008	459.5139
62	8	55	61	8	54	E	0	355217.830	-0.008	459.5139
62	7	55	61	7	54	A	0	355222.085	0.021	459.5112
62	7	55	61	8	54	A	0	355222.085	0.021	459.5112

J'	K_a'	K_c'	J''	K_a''	K_c''	Sym.	$v_{t_{CH_3}}$	$\nu_{Obs.}$	$\nu_{Obs.} - \nu_{Calc.}$	E_{low}
62	8	55	61	7	54	A	0	355222.085	0.020	459.5112
62	8	55	61	8	54	A	0	355222.085	0.021	459.5112
63	6	57	62	6	56	E	1	355223.691	-0.052	591.2002
63	6	57	62	7	56	E	1	355223.691	-0.052	591.2002
63	7	57	62	6	56	E	1	355223.691	-0.052	591.2002
63	7	57	62	7	56	E	1	355223.691	-0.052	591.2002
64	5	59	63	5	58	A	1	355241.953	-0.105	594.2832
64	5	59	63	6	58	A	1	355241.953	-0.105	594.2832
64	6	59	63	5	58	A	1	355241.953	-0.105	594.2832
64	6	59	63	6	58	A	1	355241.953	-0.105	594.2832
59	10	49	58	11	48	A	1	355279.055	0.173	574.7058
60	9	51	59	9	50	E	0	355285.161	-0.057	451.2678
60	10	51	59	10	50	E	0	355285.161	0.205	451.2679
60	9	51	59	9	50	A	0	355290.242	-0.095	451.2658
60	9	51	59	10	50	A	0	355290.242	0.481	451.2658
60	10	51	59	9	50	A	0	355290.242	-0.406	451.2658
60	10	51	59	10	50	A	0	355290.242	0.169	451.2658
59	11	49	58	10	48	A	1	355297.322	0.136	574.7054
63	6	57	62	6	56	E	0	355303.984	0.009	462.9211
63	7	57	62	7	56	E	0	355303.984	0.009	462.9211
63	6	57	62	6	56	A	0	355307.831	0.018	462.9178
63	6	57	62	7	56	A	0	355307.831	0.018	462.9178
63	7	57	62	6	56	A	0	355307.831	0.018	462.9178
63	7	57	62	7	56	A	0	355307.831	0.018	462.9178
64	5	59	63	5	58	E	1	355353.424	0.004	594.1541
64	5	59	63	6	58	E	1	355353.424	0.004	594.1541
64	6	59	63	5	58	E	1	355353.424	0.004	594.1541
64	6	59	63	6	58	E	1	355353.424	0.004	594.1541
65	4	61	64	4	60	A	1	355414.268	-0.305	596.8283
65	4	61	64	5	60	A	1	355414.268	-0.305	596.8283
65	5	61	64	4	60	A	1	355414.268	-0.305	596.8283
65	5	61	64	5	60	A	1	355414.268	-0.305	596.8283
64	5	59	63	5	58	E	0	355433.966	-0.033	465.8773
64	6	59	63	6	58	E	0	355433.966	-0.033	465.8773
64	5	59	63	5	58	A	0	355437.458	0.022	465.8733
64	5	59	63	6	58	A	0	355437.458	0.022	465.8733
64	6	59	63	5	58	A	0	355437.458	0.022	465.8733
64	6	59	63	6	58	A	0	355437.458	0.022	465.8733
59	10	49	58	11	48	E	1	355462.065	-0.071	574.6431
60	32	28	60	31	29	E	0	355465.173	0.005	626.9531
59	11	49	58	11	48	E	1	355470.101	0.050	574.6431
59	10	49	58	10	48	E	1	355476.073	-0.108	574.6426
59	11	49	58	10	48	E	1	355484.216	0.120	574.6426
65	4	61	64	4	60	E	1	355511.609	-0.250	596.6691
65	4	61	64	5	60	E	1	355511.609	-0.250	596.6691
65	5	61	64	4	60	E	1	355511.609	-0.250	596.6691
65	5	61	64	5	60	E	1	355511.609	-0.250	596.6691
59	10	49	58	11	48	A	0	355539.405	0.059	446.3674
59	11	49	58	11	48	A	0	355546.597	-0.061	446.3674
59	10	49	58	10	48	E	0	355546.597	0.015	446.3690
59	10	49	58	10	48	A	0	355552.307	-0.047	446.3670
33	14	20	32	13	20	E	0	355555.897	0.018	208.6185
59	11	49	58	10	48	A	0	355559.758	0.093	446.3670
33	14	19	32	13	19	E	0	355559.758	-0.124	208.6317
31	15	16	30	14	16	E	0	355570.075	-0.067	200.5083
31	15	17	30	14	17	E	0	355573.080	-0.059	200.4948
65	4	61	64	5	60	E	0	355592.921	0.011	468.3963
65	5	61	64	4	60	E	0	355592.921	0.011	468.3963
65	4	61	64	4	60	A	0	355595.933	0.029	468.3913
65	4	61	64	5	60	A	0	355595.933	0.029	468.3913
65	5	61	64	4	60	A	0	355595.933	0.029	468.3913
65	5	61	64	5	60	A	0	355595.933	0.029	468.3913
33	14	19	32	13	20	A	0	355601.746	-0.007	208.6236
66	3	63	65	3	62	A	1	355607.602	-0.141	598.9519
66	3	63	65	4	62	A	1	355607.602	-0.141	598.9519
66	4	63	65	3	62	A	1	355607.602	-0.141	598.9519
66	4	63	65	4	62	A	1	355607.602	-0.141	598.9519
31	15	17	30	14	16	A	0	355612.926	-0.006	200.5017
56	18	39	55	18	38	A	0	355649.851	-0.082	449.1898
56	18	39	55	18	38	E	0	355686.326	-0.037	449.1833
66	3	63	65	3	62	E	1	355688.741	0.033	598.7558
66	3	63	65	4	62	E	1	355688.741	0.033	598.7558
66	4	63	65	3	62	E	1	355688.741	0.033	598.7558
66	4	63	65	4	62	E	1	355688.741	0.033	598.7558
59	32	27	59	31	28	A	0	355737.465	-0.155	614.5482
59	32	28	59	31	29	A	0	355737.465	-0.155	614.5482
66	3	63	65	3	62	E	0	355770.677	0.014	470.4887
66	4	63	65	4	62	E	0	355770.677	0.014	470.4887
66	3	63	65	3	62	A	0	355773.210	0.062	470.4825
66	4	63	65	4	62	A	0	355773.210	0.062	470.4825
58	12	47	57	12	46	A	1	355790.886	0.143	569.2250
67	2	65	66	2	64	A	1	355815.198	-0.069	600.6633
67	2	65	66	3	64	A	1	355815.198	-0.069	600.6633
67	3	65	66	2	64	A	1	355815.198	-0.069	600.6633
67	3	65	66	3	64	A	1	355815.198	-0.069	600.6633
67	2	65	66	2	64	E	1	355876.833	0.090	600.4226
67	2	65	66	3	64	E	1	355876.833	0.090	600.4226
67	3	65	66	2	64	E	1	355876.833	0.090	600.4226
67	3	65	66	3	64	E	1	355876.833	0.090	600.4226
58	11	47	57	12	46	E	0	355932.983	0.051	440.8908
58	11	47	57	12	46	A	0	355938.724	-0.047	440.8887
67	2	65	66	2	64	E	0	355960.434	0.081	472.1632
67	3	65	66	3	64	E	0	355960.434	0.081	472.1632
67	2	65	66	2	64	A	0	355962.187	-0.049	472.1556
67	2	65	66	3	64	A	0	355962.187	-0.049	472.1556
67	3	65	66	2	64	A	0	355962.187	-0.049	472.1556
67	3	65	66	3	64	A	0	355962.187	-0.049	472.1556
58	12	47	57	12	46	E	1	356001.141	0.045	569.1585
57	24	33	56	24	32	A	0	356010.111	0.012	506.4504
57	24	34	56	24	33	A	0	356010.111	0.012	506.4504
68	1	67	67	1	66	A	1	356033.057	0.083	601.9701
68	1	67	67	2	66	A	1	356033.057	0.083	601.9701
68	2	67	67	1	66	A	1	356033.057	0.083	601.9701
68	2	67	67	2	66	A	1	356033.057	0.083	601.9701
58	12	47	57	12	46	E	0	356064.113	0.074	440.8908
68	1	67	67	1	66	E	1	356070.839	0.060	601.6760
68	2	67	67	2	66	E	1	356070.839	0.060	601.6760
56	13	44	55	12	43	E	0	356107.992	-0.045	422.8919
68	1	67	67	1	66	E	0	356156.988	-0.145	473.4267
68	2	67	67	2	66	E	0	356156.988	-0.145	473.4267
68	1	67	67	1	66	A	0	356158.488	0.196	473.4175
68	1	67	67	2	66	A	0	356158.488	0.196	473.4175
68	2	67	67	1	66	A	0	356158.488	0.196	473.4175
68	2	67	67	2	66	A	0	356158.488	0.196	473.4175
58	11	47	57	11	46	A	0	356162.453	-0.115	440.8812
69	0	69	68	0	68	A	1	356258.416	0.228	602.8791

J'	K_a'	K_c'	J''	K_a''	K_c''	Sym.	$v_{t_{CH_3}}$	$\nu_{Obs.}$	$\nu_{Obs.} - \nu_{Calc.}$	E_{low}
69	0	69	68	1	68	A	1	356258.416	0.228	602.8791
69	1	69	68	0	68	A	1	356258.416	0.228	602.8791
69	1	69	68	1	68	A	1	356258.416	0.228	602.8791
69	0	69	68	0	68	E	1	356267.107	0.128	602.5214
69	1	69	68	1	68	E	1	356267.107	0.128	602.5214
58	12	47	57	11	46	E	0	356286.644	-0.063	440.8834
58	12	47	57	11	46	A	0	356294.291	-0.073	440.8812
57	32	26	57	31	27	E	0	356306.244	0.007	590.3449
57	32	25	57	31	26	A	0	356310.860	-0.119	590.3579
57	32	26	57	31	27	A	0	356310.860	-0.119	590.3579
69	0	69	68	0	68	A	0	356357.721	-0.116	474.2739
69	0	69	68	1	68	A	0	356357.721	-0.116	474.2739
69	1	69	68	0	68	A	0	356357.721	-0.116	474.2739
69	1	69	68	1	68	A	0	356357.721	-0.116	474.2739
69	0	69	68	0	68	E	0	356357.721	0.166	474.2850
69	1	69	68	1	68	E	0	356357.721	0.166	474.2850
56	32	25	56	31	26	E	0	356571.244	-0.048	578.5669
56	32	24	56	31	25	A	0	356575.850	0.042	578.5800
56	32	25	56	31	26	A	0	356575.850	0.042	578.5800
56	32	24	56	31	25	E	0	356613.902	-0.052	578.5720
57	23	35	56	23	34	A	0	356681.775	-0.031	497.7764
57	23	34	56	23	33	A	0	356681.775	-0.032	497.7764
57	13	45	56	13	44	E	0	356806.216	-0.016	434.7704
57	13	45	56	13	44	A	0	356812.975	0.016	434.7680
55	32	23	55	31	24	A	0	356826.764	-0.063	567.0131
55	32	24	55	31	25	A	0	356826.764	-0.063	567.0131
54	32	23	54	31	24	E	0	357060.283	-0.200	555.6440
54	32	22	54	31	23	A	0	357064.496	-0.086	555.6572
54	32	23	54	31	24	A	0	357064.496	-0.086	555.6572
54	32	22	54	31	23	E	0	357103.236	0.143	555.6492
53	32	22	53	31	23	E	0	357285.556	-0.135	544.4986
53	32	21	53	31	22	A	0	357289.561	-0.034	544.5118
53	32	22	53	31	23	A	0	357289.561	-0.034	544.5118
53	32	21	53	31	22	E	0	357328.263	-0.010	544.5039
57	22	36	56	22	35	A	0	357465.236	0.001	489.5273
57	22	36	56	22	35	E	0	357469.283	-0.069	489.5152
52	32	20	52	31	21	A	0	357502.379	0.000	533.5769
52	32	21	52	31	22	A	0	357502.379	0.000	533.5769
56	17	40	55	17	39	A	0	357513.857	-0.063	443.2528
56	17	40	55	17	39	E	0	357530.792	-0.032	443.2507
51	32	20	51	31	21	E	0	357699.775	-0.104	522.8388
51	32	19	51	31	20	A	0	357703.440	0.013	522.8521
51	32	20	51	31	21	A	0	357703.440	0.013	522.8521
50	13	38	49	12	37	E	0	357758.494	-0.003	355.0648
50	32	19	50	31	20	E	0	357889.850	0.016	512.3239
50	32	18	50	31	19	A	0	357893.169	-0.048	512.3373
50	32	19	50	31	20	A	0	357893.169	-0.048	512.3373
50	32	18	50	31	19	E	0	357932.227	-0.104	512.3293
49	32	18	49	31	19	E	0	358068.974	-0.012	502.0188
49	32	17	49	31	18	A	0	358072.116	-0.099	502.0321
49	32	18	49	31	19	A	0	358072.116	-0.099	502.0321
49	32	17	49	31	18	E	0	358111.397	-0.057	502.0242
22	20	2	21	19	2	E	0	358128.428	-0.043	183.4782
22	20	3	21	19	3	E	0	358158.218	0.038	183.4670
22	20	2	21	19	3	A	0	358175.359	-0.040	183.4795
22	20	3	21	19	2	A	0	358175.359	-0.040	183.4795
48	32	16	48	31	17	A	0	358240.836	-0.032	491.9365
48	32	17	48	31	18	A	0	358240.836	-0.032	491.9365
48	32	16	48	31	17	E	0	358279.911	-0.312	491.9285
56	17	39	55	17	38	A	0	358343.441	0.241	443.2927
56	17	39	55	17	38	E	0	358343.441	-0.352	443.2923
57	21	37	56	21	36	A	0	358392.275	0.081	481.7134
47	32	15	47	31	16	A	0	358399.585	-0.029	482.0502
47	32	16	47	31	17	A	0	358399.585	-0.029	482.0502
47	32	15	47	31	16	E	0	358438.995	-0.083	482.0422
46	32	14	46	31	15	A	0	358548.844	-0.032	472.3729
46	32	15	46	31	16	A	0	358548.844	-0.032	472.3729
46	32	14	46	31	15	E	0	358588.518	0.078	472.3649
50	13	38	49	12	37	E	1	358675.523	0.722	483.3066
45	32	13	45	31	14	A	0	358689.031	-0.035	462.9045
45	32	14	45	31	15	A	0	358689.031	-0.035	462.9045
45	32	13	45	31	14	E	0	358728.788	0.066	462.8965
44	32	12	44	31	13	A	0	358820.517	-0.066	453.6448
44	32	13	44	31	14	A	0	358820.517	-0.066	453.6448
43	32	12	43	31	13	E	0	358941.351	0.008	444.5801
43	32	11	43	31	12	A	0	358943.761	-0.051	444.5936
43	32	12	43	31	13	A	0	358943.761	-0.051	444.5936
24	19	5	23	18	5	E	0	358984.664	-0.020	185.6791
24	19	6	23	18	6	E	0	359009.862	0.023	185.6670
24	19	6	23	18	5	A	0	359032.081	-0.039	185.6788
24	19	5	23	18	6	A	0	359032.081	-0.039	185.6788
42	32	10	42	31	11	A	0	359059.091	-0.040	435.7507
42	32	11	42	31	12	A	0	359059.091	-0.040	435.7507
56	16	41	55	16	40	E	0	359170.696	-0.108	437.8408
54	14	40	53	14	39	A	0	359191.706	-0.004	406.5143
54	14	40	53	14	39	E	0	359197.631	0.159	406.5197
41	32	9	41	31	10	E	0	359206.830	-0.026	427.1080
55	13	42	54	13	41	E	0	359215.582	-0.079	415.3378
55	13	42	54	13	41	A	0	359232.983	-0.012	415.3326
40	32	8	40	31	9	E	0	359307.436	-0.059	418.6812
39	32	7	39	31	8	E	0	359401.246	-0.035	410.4622
58	31	28	57	31	27	A	0	359430.818	-0.019	590.3579
58	31	27	57	31	26	A	0	359430.818	-0.019	590.3579
58	31	27	57	31	26	E	0	359430.818	-0.212	590.3500
58	31	28	57	31	27	E	0	359432.674	0.039	590.3449
57	20	38	56	20	37	A	0	359508.928	0.007	474.3487
57	20	37	56	20	36	A	0	359511.707	-0.040	474.3488
57	20	37	56	20	36	E	0	359514.895	-0.206	474.3480
57	20	38	56	20	37	E	0	359516.482	0.095	474.3381
57	13	45	56	12	44	E	0	359584.550	0.073	434.6777
26	18	8	25	17	8	E	0	359810.020	-0.042	189.1096
26	18	9	25	17	9	E	0	359830.237	-0.014	189.0969
26	18	8	25	17	9	A	0	359857.302	-0.042	189.1076
26	18	9	25	17	8	A	0	359857.302	-0.042	189.1076
36	13	24	35	12	23	A	0	359906.416	-0.032	225.5443
36	13	23	35	12	23	E	0	360015.519	-0.058	225.5502
62	8	54	61	8	53	A	1	360073.083	-0.010	595.8310
62	8	54	61	9	53	A	1	360073.083	-0.001	595.8310
62	9	54	61	8	53	A	1	360073.083	-0.015	595.8310
62	9	54	61	9	53	A	1	360073.083	-0.006	595.8310
63	7	56	62	7	55	A	1	360112.830	-0.069	599.7221
63	7	56	62	8	55	A	1	360112.830	-0.068	599.7221
63	8	56	62	7	55	A	1	360112.830	-0.069	599.7221
63	8	56	62	8	55	A	1	360112.830	-0.068	599.7221
61	9	52	60	9	51	A	1	360130.432	-0.050	591.4536
61	9	52	60	10	51	A	1	360130.432	0.225	591.4536

J'	K_a'	K_c'	J''	K_a''	K_c''	Sym.	$v_{t_{CH_3}}$	$\nu_{Obs.}$	$\nu_{Obs.} - \nu_{Calc.}$	E_{low}
61	10	52	60	9	51	A	1	360130.432	-0.197	591.4536
61	10	52	60	10	51	A	1	360130.432	0.078	591.4536
36	13	24	35	12	24	E	0	360146.345	-0.069	225.5328
64	7	58	63	7	57	A	1	360215.787	-0.140	603.1501
64	7	58	63	7	57	A	1	360215.787	-0.140	603.1501
64	7	58	63	7	57	A	1	360215.787	-0.140	603.1501
64	7	58	63	7	57	A	1	360215.787	-0.140	603.1501
62	8	54	61	8	53	E	1	360220.048	-0.010	595.7627
62	8	54	61	9	53	E	1	360220.048	0.001	595.7627
62	9	54	61	8	53	E	1	360220.048	-0.016	595.7627
62	9	54	61	9	53	E	1	360220.048	-0.005	595.7627
63	7	56	62	7	55	E	1	360247.513	-0.006	599.6401
63	7	56	62	8	55	E	1	360247.513	-0.006	599.6401
63	8	56	62	7	55	E	1	360247.513	-0.006	599.6401
63	8	56	62	8	55	E	1	360247.513	-0.006	599.6401
61	9	52	60	9	51	E	1	360291.695	-0.127	591.3938
61	9	52	60	10	51	E	1	360291.695	0.213	591.3938
61	10	52	60	9	51	E	1	360291.695	-0.311	591.3938
61	10	52	60	10	51	E	1	360291.695	0.030	591.3938
62	8	54	61	8	53	E	0	360301.143	-0.005	467.4859
62	9	54	61	9	53	E	0	360301.143	-0.000	467.4859
62	8	54	61	8	53	A	0	360305.718	0.007	467.4837
62	8	54	61	9	53	A	0	360305.718	0.018	467.4837
62	9	54	61	8	53	A	0	360305.718	0.002	467.4837
62	9	54	61	9	53	A	0	360305.718	0.012	467.4837
63	7	56	62	7	55	E	0	360329.569	0.009	471.3627
63	8	56	62	8	55	E	0	360329.569	0.009	471.3627
63	7	56	62	7	55	A	0	360333.633	-0.099	471.3601
63	7	56	62	8	55	A	0	360333.633	-0.099	471.3601
63	8	56	62	7	55	A	0	360333.633	-0.100	471.3601
63	8	56	62	8	55	A	0	360333.633	-0.099	471.3601
64	6	58	63	6	57	E	1	360338.735	-0.011	603.0492
64	6	58	63	7	57	E	1	360338.735	-0.011	603.0492
64	7	58	63	6	57	E	1	360338.735	-0.011	603.0492
64	7	58	63	7	57	E	1	360338.735	-0.011	603.0492
36	13	23	35	12	24	A	0	360341.375	-0.119	225.5321
60	10	50	59	10	49	A	1	360345.395	0.206	586.5566
60	11	50	59	10	49	A	1	360348.997	0.149	586.5566
65	5	60	64	5	59	A	1	360361.096	-0.161	606.1328
65	5	60	64	6	59	A	1	360361.096	-0.161	606.1328
65	6	60	64	5	59	A	1	360361.096	-0.161	606.1328
65	6	60	64	6	59	A	1	360361.096	-0.161	606.1328
61	9	52	60	9	51	E	0	360370.644	-0.088	463.1189
61	10	52	60	10	51	E	0	360370.644	0.055	463.1189
61	9	52	60	9	51	A	0	360375.680	-0.066	463.1170
61	9	52	60	10	51	A	0	360375.680	0.245	463.1170
61	10	52	60	9	51	A	0	360375.680	-0.234	463.1170
61	10	52	60	10	51	A	0	360375.680	0.077	463.1170
58	13	45	57	14	44	A	0	360412.995	-0.046	452.0118
64	6	58	63	6	57	E	0	360421.159	0.027	474.7728
64	7	58	63	7	57	E	0	360421.159	0.027	474.7728
64	6	58	63	6	57	A	0	360424.945	0.014	474.7696
64	6	58	63	7	57	A	0	360424.945	0.014	474.7696
64	7	58	63	6	57	A	0	360424.945	0.014	474.7696
64	7	58	63	7	57	A	0	360424.945	0.014	474.7696
58	13	45	57	14	44	E	0	360433.672	-0.111	452.0140
65	5	60	64	5	59	E	1	360471.794	0.045	606.0074
65	5	60	64	6	59	E	1	360471.794	0.045	606.0074
65	6	60	64	5	59	E	1	360471.794	0.045	606.0074
65	6	60	64	6	59	E	1	360471.794	0.045	606.0074
60	10	50	59	11	49	E	1	360517.485	-0.248	586.5003
60	11	50	59	11	49	E	1	360522.282	0.117	586.5003
60	10	50	59	10	49	E	1	360525.865	0.216	586.5000
60	11	50	59	10	49	E	1	360530.077	-0.004	586.5000
66	4	62	65	4	61	A	1	360535.392	-0.024	608.6837
66	4	62	65	5	61	A	1	360535.392	-0.024	608.6837
66	5	62	65	4	61	A	1	360535.392	-0.024	608.6837
66	5	62	65	5	61	A	1	360535.392	-0.024	608.6837
65	5	60	64	5	59	E	0	360554.296	0.003	477.7333
65	6	60	64	6	59	E	0	360554.296	0.003	477.7333
65	5	60	64	5	59	A	0	360557.749	0.046	477.7294
65	5	60	64	6	59	A	0	360557.749	0.046	477.7294
65	6	60	64	5	59	A	0	360557.749	0.046	477.7294
65	6	60	64	6	59	A	0	360557.749	0.046	477.7294
28	17	11	27	16	11	E	0	360567.175	-0.054	193.7834
60	11	50	59	10	49	A	0	360609.531	-0.082	458.2269
28	17	12	27	16	11	A	0	360613.624	-0.059	193.7798
28	17	11	27	16	12	A	0	360613.624	-0.059	193.7798
66	4	62	65	4	61	E	1	360632.213	0.071	608.5277
66	4	62	65	5	61	E	1	360632.213	0.071	608.5277
66	5	62	65	4	61	E	1	360632.213	0.071	608.5277
66	5	62	65	5	61	E	1	360632.213	0.071	608.5277
66	4	62	65	4	61	E	0	360715.017	0.026	480.2576
66	5	62	65	5	61	E	0	360715.017	0.026	480.2576
66	4	62	65	4	61	A	0	360718.004	0.036	480.2527
66	4	62	65	5	61	A	0	360718.004	0.036	480.2527
66	5	62	65	4	61	A	0	360718.004	0.036	480.2527
66	5	62	65	5	61	A	0	360718.004	0.036	480.2527
67	3	64	66	3	63	A	1	360729.381	-0.106	610.8137
67	3	64	66	4	63	A	1	360729.381	-0.106	610.8137
67	4	64	66	3	63	A	1	360729.381	-0.106	610.8137
67	4	64	66	4	63	A	1	360729.381	-0.106	610.8137
59	11	48	58	12	47	A	1	360732.048	0.195	581.0929
58	12	46	57	13	45	E	0	360746.826	0.049	446.6722
58	12	46	57	13	45	A	0	360748.529	-0.161	446.6700
67	3	64	66	3	63	E	1	360810.144	0.014	610.6203
67	3	64	66	4	63	E	1	360810.144	0.014	610.6203
67	4	64	66	3	63	E	1	360810.144	0.014	610.6203
67	4	64	66	4	63	E	1	360810.144	0.014	610.6203
67	3	64	66	3	63	E	0	360893.758	0.018	482.3559
67	4	64	66	4	63	E	0	360893.758	0.018	482.3559
67	3	64	66	4	63	A	0	360896.230	0.015	482.3499
67	4	64	66	3	63	A	0	360896.230	0.015	482.3499
59	11	48	58	12	47	E	1	360924.165	0.193	581.0335
38	12	26	37	11	27	E	0	360935.960	-0.207	237.0590
68	2	66	67	2	65	A	1	360937.524	0.014	612.5320
68	2	66	67	3	65	A	1	360937.524	0.014	612.5320
68	3	66	67	2	65	A	1	360937.524	0.014	612.5320
68	3	66	67	3	65	A	1	360937.524	0.014	612.5320
38	12	26	37	11	27	A	0	360951.508	0.090	237.0542
59	11	48	58	12	47	E	0	360997.198	0.048	452.7678
68	2	66	67	2	65	E	1	360998.872	0.039	612.2933
68	2	66	67	3	65	E	1	360998.872	0.039	612.2933
68	3	66	67	2	65	E	1	360998.872	0.039	612.2933
68	3	66	67	3	65	E	1	360998.872	0.039	612.2933
59	11	48	58	12	47	A	0	361003.098	0.003	452.7659

J'	K_a'	K_c'	J''	K_a''	K_c''	Sym.	$v_{t_{CH_3}}$	$\nu_{Obs.}$	$\nu_{Obs.} - \nu_{Calc.}$	E_{low}
59	12	48	58	12	47	E	1	361006.738	0.085	581.0335
59	12	48	58	12	47	E	0	361073.458	-0.206	452.7678
68	2	66	67	2	65	E	0	361084.043	0.073	484.0367
68	3	66	67	3	65	E	0	361084.043	0.073	484.0367
68	2	66	67	2	65	A	0	361085.807	-0.041	484.0293
68	2	66	67	3	65	A	0	361085.807	-0.041	484.0293
68	3	66	67	2	65	A	0	361085.807	-0.041	484.0293
68	3	66	67	3	65	A	0	361085.807	-0.041	484.0293
59	11	48	58	11	47	A	0	361134.798	-0.093	452.7615
59	12	48	58	11	47	E	1	361147.941	0.011	581.0287
69	1	68	68	1	67	A	1	361155.479	-0.050	613.8461
69	1	68	68	2	67	A	1	361155.479	-0.050	613.8461
69	2	68	68	1	67	A	1	361155.479	-0.050	613.8461
69	2	68	68	2	67	A	1	361155.479	-0.050	613.8461
30	16	14	29	15	14	E	0	361187.935	-0.071	199.7210
69	1	68	68	1	67	E	1	361193.278	0.012	613.5532
69	1	68	68	2	67	E	1	361193.278	0.012	613.5532
69	2	68	68	1	67	E	1	361193.278	0.012	613.5532
69	2	68	68	2	67	E	1	361193.278	0.012	613.5532
30	16	15	29	15	15	E	0	361197.095	0.052	199.7075
59	12	48	58	11	47	E	0	361204.703	-0.068	452.7634
59	12	48	58	11	47	A	0	361211.845	0.024	452.7615
30	16	14	29	15	15	A	0	361232.928	-0.027	199.7158
30	16	15	29	15	14	A	0	361232.928	-0.026	199.7158
69	1	68	68	1	67	A	0	361282.393	0.190	485.2977
69	1	68	68	2	67	A	0	361282.393	0.190	485.2977
69	2	68	68	1	67	A	0	361282.393	0.190	485.2977
69	2	68	68	2	67	A	0	361282.393	0.190	485.2977
34	14	21	33	13	21	E	0	361374.162	0.022	215.5192
34	14	20	33	13	20	E	0	361378.559	-0.069	215.5323
70	0	70	69	0	69	A	1	361381.267	0.256	614.7626
70	0	70	69	1	69	A	1	361381.267	0.256	614.7626
70	1	70	69	0	69	A	1	361381.267	0.256	614.7626
70	1	70	69	1	69	A	1	361381.267	0.256	614.7626
70	0	70	69	0	69	E	1	361389.842	0.121	614.4052
70	1	70	69	1	69	E	1	361389.842	0.121	614.4052
34	14	21	33	13	20	A	0	361414.007	-0.061	215.5243
34	14	20	33	13	21	A	0	361422.892	0.048	215.5241
70	0	70	69	0	69	A	0	361481.825	-0.103	486.1607
70	0	70	69	1	69	A	0	361481.825	-0.103	486.1607
70	1	70	69	0	69	A	0	361481.825	-0.103	486.1607
70	1	70	69	1	69	A	0	361481.825	-0.103	486.1607
58	13	46	57	13	45	A	1	361540.266	0.326	574.9984
32	15	17	31	14	17	E	0	361544.303	-0.073	206.9537
32	15	18	31	14	18	E	0	361547.091	0.015	206.9402
32	15	18	31	14	17	A	0	361587.088	0.015	206.9470
58	13	46	57	13	45	E	1	361762.912	0.183	574.9299
58	13	46	57	13	45	E	0	361825.136	0.070	446.6722
58	13	46	57	13	45	A	0	361831.880	-0.022	446.6700
58	25	34	57	25	33	A	0	361897.478	-0.033	527.3972
58	25	33	57	25	32	A	0	361897.478	-0.033	527.3972
55	15	40	54	15	39	A	0	362218.547	-0.090	421.8307
55	15	40	54	15	39	E	0	362236.009	-0.049	421.8342
49	13	37	48	12	36	E	0	362424.521	-0.032	344.2577
58	12	46	57	12	45	E	0	362489.750	0.006	446.6141
58	12	46	57	12	45	A	0	362499.385	0.100	446.6116
58	24	34	57	24	33	A	0	362515.483	-0.076	518.3256
58	24	35	57	24	34	A	0	362515.483	-0.076	518.3256
57	18	40	56	18	39	A	0	362574.838	-0.095	461.0530
57	18	40	56	18	39	E	0	362609.390	-0.100	461.0477
57	18	39	56	18	38	A	0	362786.721	0.062	461.0621
56	13	43	55	13	42	E	0	363069.941	0.021	427.3199
56	13	43	55	13	42	A	0	363088.120	-0.025	427.3153
58	23	36	57	23	35	A	0	363229.430	0.001	509.6741
56	16	40	55	16	39	A	0	363258.775	-0.075	438.0877
56	16	40	55	16	39	E	0	363274.200	-0.016	438.0889
21	21	0	20	20	0	E	0	363407.292	-0.053	186.5997
21	21	1	20	20	1	E	0	363441.204	0.053	186.5894
21	21	1	20	20	0	A	0	363453.070	-0.052	186.6025
21	21	0	20	20	1	A	0	363453.070	-0.052	186.6025
70	33	38	70	32	39	A	0	363538.152	0.025	774.5262
70	33	37	70	32	38	A	0	363538.152	0.025	774.5262
58	13	46	57	12	45	E	0	363568.015	-0.018	446.6141
70	33	37	70	32	38	E	0	363575.896	0.194	774.5195
58	13	46	57	12	45	A	0	363582.463	-0.034	446.6116
58	22	37	57	22	36	A	0	364063.969	-0.088	501.4511
23	20	3	22	19	3	E	0	364271.894	-0.052	187.9959
23	20	4	22	19	4	E	0	364301.672	0.045	187.9847
23	20	3	22	19	4	A	0	364318.824	-0.047	187.9972
23	20	4	22	19	3	A	0	364318.824	-0.047	187.9972
68	33	36	68	32	37	A	0	364429.825	-0.010	745.6781
68	33	35	68	32	36	A	0	364429.825	-0.010	745.6781
68	33	35	68	32	36	E	0	364467.960	-0.146	745.6714
57	17	41	56	17	40	A	0	364492.628	-0.044	455.1782
57	17	41	56	17	40	E	0	364505.707	0.078	455.1767
67	33	35	67	32	36	E	0	364843.669	-0.173	731.5625
67	33	35	67	32	36	A	0	364845.612	-0.082	731.5750
67	33	34	67	32	35	A	0	364845.612	-0.082	731.5750
37	13	25	36	12	24	E	0	364872.522	0.066	233.1634
58	21	38	57	21	37	A	0	365055.021	0.210	493.6681
37	13	25	36	12	24	A	0	365096.866	-0.002	233.1584
25	19	6	24	18	6	E	0	365121.277	-0.064	190.6149
25	19	7	24	18	7	E	0	365146.485	0.036	190.6028
25	19	7	24	18	6	A	0	365168.728	-0.038	190.6145
25	19	6	24	18	7	A	0	365168.728	-0.038	190.6145
63	8	55	62	8	54	A	1	365176.228	-0.056	607.8418
63	8	55	62	9	54	A	1	365176.228	-0.051	607.8418
63	9	55	62	8	54	A	1	365176.228	-0.058	607.8418
63	9	55	62	9	54	A	1	365176.228	-0.053	607.8418
62	9	53	61	9	52	A	1	365218.867	-0.000	603.4662
62	9	53	61	10	52	A	1	365218.867	0.147	603.4662
62	10	53	61	9	52	A	1	365218.867	-0.079	603.4662
62	10	53	61	10	52	A	1	365218.867	0.069	603.4662
64	7	57	63	7	56	A	1	365224.398	-0.072	611.7341
64	7	57	63	8	56	A	1	365224.398	-0.072	611.7341
64	8	57	63	7	56	A	1	365224.398	-0.072	611.7341
64	8	57	63	8	56	A	1	365224.398	-0.072	611.7341
66	33	34	66	32	35	E	0	365240.901	-0.131	717.6728
66	33	34	66	32	35	A	0	365242.496	-0.046	717.6854
66	33	33	66	32	34	A	0	365242.496	-0.046	717.6854
37	13	24	36	12	24	E	0	365276.665	-0.127	233.1634
63	8	55	62	8	54	E	1	365320.863	-0.039	607.7784
63	8	55	62	9	54	E	1	365320.863	-0.033	607.7784
63	9	55	62	8	54	E	1	365320.863	-0.042	607.7784
63	9	55	62	9	54	E	1	365320.863	-0.036	607.7784
65	6	59	64	6	58	A	1	365332.099	-0.216	615.1656

J'	K_a'	K_c'	J''	K_a''	K_c''	Sym.	$v_{t_{CH_3}}$	$\nu_{Obs.}$	$\nu_{Obs.} - \nu_{Calc.}$	E_{low}
65	6	59	64	7	58	A	1	365332.099	-0.216	615.1656
65	7	59	64	6	58	A	1	365332.099	-0.216	615.1656
65	7	59	64	7	58	A	1	365332.099	-0.216	615.1656
64	7	57	63	7	56	E	1	365357.391	0.020	611.6566
64	7	57	63	8	56	E	1	365357.391	0.021	611.6566
64	8	57	63	7	56	E	1	365357.391	0.020	611.6566
64	8	57	63	8	56	E	1	365357.391	0.021	611.6566
62	9	53	61	9	52	E	1	365376.959	-0.012	603.4119
62	9	53	61	10	52	E	1	365376.959	0.172	603.4119
62	10	53	61	9	52	E	1	365376.959	-0.111	603.4119
62	10	53	61	10	52	E	1	365376.959	0.073	603.4119
57	15	43	56	15	42	E	0	365385.909	-0.379	444.8451
63	8	55	62	8	54	A	0	365409.158	0.023	479.5022
63	8	55	62	9	54	A	0	365409.158	0.028	479.5022
63	9	55	62	8	54	A	0	365409.158	0.020	479.5022
63	9	55	62	9	54	A	0	365409.158	0.026	479.5022
64	7	57	63	7	56	A	0	365445.911	0.006	483.3796
64	7	57	63	8	56	A	0	365445.911	0.006	483.3796
64	8	57	63	7	56	A	0	365445.911	0.006	483.3796
64	8	57	63	8	56	A	0	365445.911	0.006	483.3796
65	6	59	64	6	58	E	1	365453.753	-0.139	615.0688
65	6	59	64	7	58	E	1	365453.753	-0.139	615.0688
65	7	59	64	6	58	E	1	365453.753	-0.139	615.0688
65	7	59	64	7	58	E	1	365453.753	-0.139	615.0688
62	9	53	61	9	52	A	0	365463.784	-0.053	475.1378
62	9	53	61	10	52	A	0	365463.784	0.115	475.1378
62	10	53	61	9	52	A	0	365463.784	-0.143	475.1378
62	10	53	61	10	52	A	0	365463.784	0.025	475.1378
66	5	61	65	5	60	A	1	365480.265	-0.137	618.1531
66	5	61	65	6	60	A	1	365480.265	-0.137	618.1531
66	6	61	65	5	60	A	1	365480.265	-0.137	618.1531
66	6	61	65	6	60	A	1	365480.265	-0.137	618.1531
65	6	59	64	6	58	A	0	365542.209	0.016	486.7921
65	6	59	64	7	58	A	0	365542.209	0.016	486.7921
65	7	59	64	6	58	A	0	365542.209	0.016	486.7921
65	7	59	64	7	58	A	0	365542.209	0.016	486.7921
61	10	51	60	11	50	E	1	365577.252	0.077	598.5260
61	11	51	60	11	50	E	1	365579.841	0.199	598.5260
61	10	51	60	10	50	E	1	365581.440	-0.168	598.5259
61	11	51	60	10	50	E	1	365584.324	0.250	598.5259
66	5	61	65	5	60	E	1	365590.038	0.004	618.0314
66	5	61	65	6	60	E	1	365590.038	0.004	618.0314
66	6	61	65	5	60	E	1	365590.038	0.004	618.0314
66	6	61	65	6	60	E	1	365590.038	0.004	618.0314
65	33	33	65	32	34	A	0	365621.237	0.153	704.0091
65	33	32	65	32	33	A	0	365621.237	0.153	704.0091
37	13	25	36	12	25	E	0	365647.381	-0.006	233.1376
67	4	63	66	4	62	A	1	365655.825	-0.288	620.7098
67	4	63	66	5	62	A	1	365655.825	-0.288	620.7098
67	5	63	66	4	62	A	1	365655.825	-0.288	620.7098
67	5	63	66	5	62	A	1	365655.825	-0.288	620.7098
66	5	61	65	5	60	A	0	365677.951	0.024	489.7563
66	5	61	65	6	60	A	0	365677.951	0.024	489.7563
66	6	61	65	5	60	A	0	365677.951	0.024	489.7563
66	6	61	65	6	60	A	0	365677.951	0.024	489.7563
67	4	63	66	4	62	E	1	365752.284	-0.002	620.5571
67	4	63	66	5	62	E	1	365752.284	-0.002	620.5571
67	5	63	66	4	62	E	1	365752.284	-0.002	620.5571
67	5	63	66	5	62	E	1	365752.284	-0.002	620.5571
59	31	29	58	31	28	A	0	365775.579	0.203	602.3472
59	31	28	58	31	27	A	0	365775.579	0.203	602.3472
60	11	49	59	12	48	A	1	365779.796	0.310	593.1280
60	12	49	59	12	48	A	1	365820.179	0.246	593.1280
57	17	40	56	17	39	A	0	365822.694	-0.067	455.2458
57	17	40	56	17	39	E	0	365829.123	0.107	455.2454
67	4	63	66	4	62	A	0	365839.935	0.039	492.2849
67	4	63	66	5	62	A	0	365839.935	0.039	492.2849
67	5	63	66	4	62	A	0	365839.935	0.039	492.2849
67	5	63	66	5	62	A	0	365839.935	0.039	492.2849
68	3	65	67	3	64	A	1	365850.910	-0.134	622.8464
68	3	65	67	4	64	A	1	365850.910	-0.134	622.8464
68	4	65	67	3	64	A	1	365850.910	-0.134	622.8464
68	4	65	67	4	64	A	1	365850.910	-0.134	622.8464
55	14	41	54	14	40	A	0	365887.512	0.054	418.4957
37	13	24	36	12	25	A	0	365914.258	-0.056	233.1357
27	18	10	26	17	10	E	0	365951.832	0.033	194.4542
60	11	49	59	12	48	E	1	365970.812	-0.033	593.0753
27	18	9	26	17	10	A	0	365978.914	-0.036	194.4650
27	18	10	26	17	9	A	0	365978.914	-0.036	194.4650
68	3	65	67	3	64	E	0	366016.690	0.053	494.3941
68	4	65	67	4	64	E	0	366016.690	0.053	494.3941
68	3	65	67	3	64	A	0	366019.121	0.019	494.3881
68	3	65	67	4	64	A	0	366019.121	0.019	494.3881
68	4	65	67	3	64	A	0	366019.121	0.019	494.3881
68	4	65	67	4	64	A	0	366019.121	0.019	494.3881
60	11	49	59	12	48	E	0	366045.742	0.016	464.8119
69	2	67	68	2	66	A	1	366059.484	-0.066	624.5716
69	2	67	68	3	66	A	1	366059.484	-0.066	624.5716
69	3	67	68	2	66	A	1	366059.484	-0.066	624.5716
69	3	67	68	3	66	A	1	366059.484	-0.066	624.5716
69	2	67	68	2	66	E	1	366120.777	0.056	624.3350
69	3	67	68	3	66	E	1	366120.777	0.056	624.3350
59	12	47	58	13	46	A	0	366166.721	-0.396	458.7394
60	12	49	59	11	48	E	0	366166.721	0.193	464.8094
60	12	49	59	11	48	A	0	366173.096	-0.033	464.8077
69	2	67	68	2	66	E	0	366207.457	0.067	496.0812
69	3	67	68	3	66	E	0	366207.457	0.067	496.0812
69	2	67	68	2	66	A	0	366209.238	-0.025	496.0738
69	2	67	68	3	66	A	0	366209.238	-0.025	496.0738
69	3	67	68	2	66	A	0	366209.238	-0.025	496.0738
69	3	67	68	3	66	A	0	366209.238	-0.025	496.0738
70	1	69	69	1	68	A	1	366277.977	0.099	625.8930
70	1	69	69	2	68	A	1	366277.977	0.099	625.8930
70	2	69	69	1	68	A	1	366277.977	0.099	625.8930
70	2	69	69	2	68	A	1	366277.977	0.099	625.8930
70	1	69	69	1	68	E	1	366315.647	0.098	625.6013
70	2	69	69	2	68	E	1	366315.647	0.098	625.6013
70	1	69	69	1	68	E	0	366404.617	-0.139	497.3579
70	2	69	69	2	68	E	0	366404.617	-0.139	497.3579
70	1	69	69	1	68	A	0	366406.117	0.207	497.3488
70	1	69	69	2	68	A	0	366406.117	0.207	497.3488
70	2	69	69	1	68	A	0	366406.117	0.207	497.3488
70	2	69	69	2	68	A	0	366406.117	0.207	497.3488
59	13	47	58	13	46	A	1	366536.612	0.221	587.0581
29	17	12	28	16	12	E	0	366659.967	-0.082	199.5673
29	17	13	28	16	13	E	0	366674.785	0.032	199.5541

J'	K'_a	K'_c	J''	K''_a	K''_c	Sym.	$v_{t_{CH_3}}$	$\nu_{Obs.}$	$\nu_{Obs.} - \nu_{Calc.}$	E_{low}
29	17	13	28	16	12	A	0	366706.420	-0.056	199.5637
29	17	12	28	16	13	A	0	366706.420	-0.056	199.5637
59	13	47	58	13	46	E	1	366757.943	0.148	586.9970
59	13	47	58	13	46	E	0	366822.331	0.061	458.7414
59	13	47	58	13	46	A	0	366829.143	0.042	458.7394
61	33	29	61	32	30	A	0	366965.291	-0.103	651.4310
61	33	28	61	32	29	A	0	366965.291	-0.103	651.4310
61	33	29	61	32	30	E	0	366965.291	-0.096	651.4181
61	33	28	61	32	29	E	0	367005.543	-0.049	651.4243
60	14	46	59	15	45	E	0	367091.066	-0.038	481.9951
35	14	22	34	13	22	E	0	367141.668	-0.013	222.6398
35	14	21	34	13	21	E	0	367146.519	-0.008	222.6529
35	14	22	34	13	21	A	0	367176.832	-0.070	222.6450
35	14	21	34	13	22	A	0	367195.825	-0.031	222.6444
31	16	15	30	15	15	E	0	367228.475	-0.049	205.9383
31	16	16	30	15	16	E	0	367237.365	0.003	205.9248
59	12	47	58	12	46	E	0	367241.691	-0.013	458.7054
59	12	47	58	12	46	A	0	367250.224	-0.105	458.7033
60	33	28	60	32	29	A	0	367262.128	0.022	638.8169
60	33	27	60	32	28	A	0	367262.128	0.022	638.8169
60	33	28	60	32	29	E	0	367262.128	-0.232	638.8039
31	16	15	30	15	16	A	0	367273.439	0.010	205.9331
60	33	27	60	32	28	E	0	367302.503	-0.020	638.8102
59	27	33	58	27	32	A	0	367305.150	-0.080	558.8130
59	27	32	58	27	31	A	0	367305.150	-0.080	558.8130
59	27	33	58	27	32	E	0	367306.695	-0.070	558.8015
59	27	32	58	27	31	E	0	367308.214	-0.025	558.8026
33	15	18	32	14	18	E	0	367490.433	-0.032	213.6146
33	15	19	32	14	19	E	0	367492.788	-0.039	213.6012
33	15	19	32	14	18	A	0	367533.114	0.091	213.6079
59	33	27	59	32	28	A	0	367544.395	0.158	626.4143
59	33	26	59	32	27	A	0	367544.395	0.158	626.4143
59	33	27	59	32	28	E	0	367544.395	-0.345	626.4013
59	33	26	59	32	27	E	0	367584.637	-0.223	626.4076
58	19	40	57	19	39	E	0	367756.330	0.009	479.4821
58	19	39	57	19	38	E	0	367765.840	-0.197	479.4928
58	19	39	57	19	38	A	0	367778.453	-0.134	479.4925
58	33	25	58	32	26	E	0	367853.102	-0.053	614.2165
59	13	47	58	12	46	E	0	367900.549	-0.010	458.7054
59	13	47	58	12	46	A	0	367912.267	-0.045	458.7033
59	13	46	58	14	45	A	0	367996.884	-0.025	464.2647
59	13	46	58	14	45	E	0	368008.945	0.026	464.2667
57	33	24	57	32	25	E	0	368108.032	0.095	602.2365
39	12	27	38	11	28	E	0	368364.013	-0.065	245.1450
55	33	23	55	32	24	A	0	368537.462	-0.217	578.9156
55	33	22	55	32	23	A	0	368537.462	-0.217	578.9156
55	33	23	55	32	24	E	0	368539.093	0.029	578.9023
55	33	22	55	32	23	E	0	368579.024	0.013	578.9089
54	33	22	54	32	23	A	0	368754.756	-0.046	567.5676
54	33	21	54	32	22	A	0	368754.756	-0.046	567.5676
54	33	22	54	32	23	E	0	368756.328	-0.052	567.5543
53	33	20	53	32	21	E	0	369001.922	-0.088	556.4231
59	24	35	58	24	34	A	0	369036.878	-0.031	530.4178
59	24	36	58	24	35	A	0	369036.878	-0.031	530.4178
52	33	20	52	32	21	A	0	369154.912	0.019	545.5019
52	33	19	52	32	20	A	0	369154.912	0.019	545.5019
52	33	20	52	32	21	E	0	369156.736	-0.091	545.4885
52	33	19	52	32	20	E	0	369196.586	-0.060	545.4952
51	33	19	51	32	20	A	0	369338.744	-0.013	534.7838
51	33	18	51	32	19	A	0	369338.744	-0.013	534.7838
51	33	19	51	32	20	E	0	369340.736	-0.118	534.7704
51	33	18	51	32	19	E	0	369380.609	-0.021	534.7771
50	33	18	50	32	19	A	0	369512.395	-0.010	524.2753
50	33	17	50	32	18	A	0	369512.395	-0.010	524.2753
50	33	18	50	32	19	E	0	369514.409	-0.249	524.2619
58	18	41	57	18	40	A	0	369534.673	-0.119	473.1472
22	21	1	21	20	1	E	0	369552.311	-0.039	190.9085
22	21	2	21	20	2	E	0	369586.164	0.029	190.8982
22	21	2	21	20	1	A	0	369598.067	-0.060	190.9113
22	21	1	21	20	2	A	0	369598.067	-0.060	190.9113
49	33	17	49	32	18	A	0	369676.253	-0.002	513.9761
49	33	16	49	32	17	A	0	369676.253	-0.002	513.9761
49	33	17	49	32	18	E	0	369678.631	-0.023	513.9627
49	33	16	49	32	17	E	0	369718.256	-0.091	513.9695
59	23	37	58	23	36	A	0	369795.376	-0.063	521.7901
59	23	36	58	23	35	A	0	369795.376	-0.070	521.7901
59	23	36	58	23	35	E	0	369799.519	-0.165	521.7847
48	33	16	48	32	17	E	0	369833.148	-0.098	503.8726
48	33	15	48	32	16	E	0	369872.924	0.026	503.8795
58	18	40	57	18	39	E	0	369882.825	0.022	473.1626
58	18	40	57	18	39	A	0	369893.621	-0.050	473.1634
47	33	15	47	32	16	A	0	369976.124	-0.035	494.0051
47	33	14	47	32	15	A	0	369976.124	-0.035	494.0051
47	33	15	47	32	16	E	0	369978.805	-0.021	493.9915
38	13	26	37	12	25	A	0	370008.914	0.044	241.0066
47	33	14	47	32	15	E	0	370018.555	0.118	493.9984
46	33	14	46	32	15	A	0	370112.825	-0.161	484.3328
46	33	13	46	32	14	A	0	370112.825	-0.161	484.3328
46	33	13	46	32	14	E	0	370155.368	0.022	484.3261
45	33	13	45	32	14	A	0	370241.568	0.010	474.8691
45	33	12	45	32	13	A	0	370241.568	0.010	474.8691
64	8	56	63	8	55	A	1	370280.434	-0.074	620.0227
64	8	56	63	9	55	A	1	370280.434	-0.071	620.0227
64	9	56	63	8	55	A	1	370280.434	-0.075	620.0227
64	9	56	63	9	55	A	1	370280.434	-0.073	620.0227
45	33	12	45	32	13	E	0	370283.978	-0.015	474.8624
38	13	25	37	12	25	E	0	370296.752	0.078	241.0112
63	9	54	62	9	53	A	1	370309.565	0.024	615.6486
63	9	54	62	10	53	A	1	370309.565	0.103	615.6486
63	10	54	62	9	53	A	1	370309.565	-0.018	615.6486
63	10	54	62	10	53	A	1	370309.565	0.061	615.6486
65	7	58	64	7	57	A	1	370336.346	-0.109	623.9167
65	7	58	64	8	57	A	1	370336.346	-0.109	623.9167
65	8	58	64	7	57	A	1	370336.346	-0.109	623.9167
65	8	58	64	8	57	A	1	370336.346	-0.109	623.9167
24	20	4	23	19	4	E	0	370413.735	-0.031	192.7209
64	8	56	63	8	55	E	1	370422.826	-0.009	619.9642
64	8	56	63	9	55	E	1	370422.826	-0.006	619.9642
64	9	56	63	8	55	E	1	370422.826	-0.010	619.9642
64	9	56	63	9	55	E	1	370422.826	-0.007	619.9642
24	20	5	23	19	5	E	0	370443.438	0.024	192.7097
66	6	60	65	6	59	A	1	370448.648	-0.147	627.3518
66	6	60	65	7	59	A	1	370448.648	-0.147	627.3518
66	7	60	65	6	59	A	1	370448.648	-0.147	627.3518
66	7	60	65	7	59	A	1	370448.648	-0.147	627.3518
24	20	4	23	19	5	A	0	370460.656	-0.031	192.7222

J'	K'_a	K'_c	J''	K''_a	K''_c	Sym.	$v_{t_{CH_3}}$	$\nu_{Obs.}$	$\nu_{Obs.} - \nu_{Calc.}$	E_{low}
24	20	5	23	19	4	A	0	370460.656	-0.031	192.7222
63	9	54	62	9	53	E	1	370464.452	-0.068	615.5995
63	9	54	62	10	53	E	1	370464.452	0.030	615.5995
63	10	54	62	9	53	E	1	370464.452	-0.121	615.5995
63	10	54	62	10	53	E	1	370464.452	-0.022	615.5995
65	7	58	64	7	57	E	1	370467.657	-0.008	623.8436
65	7	58	64	8	57	E	1	370467.657	-0.008	623.8436
65	8	58	64	7	57	E	1	370467.657	-0.009	623.8436
65	8	58	64	8	57	E	1	370467.657	-0.008	623.8436
62	10	52	61	11	51	A	1	370469.762	-0.047	610.7652
43	33	11	43	32	12	A	0	370475.411	0.044	456.5667
43	33	10	43	32	11	A	0	370475.411	0.044	456.5667
43	33	11	43	32	12	E	0	370478.622	0.142	456.5531
64	8	56	63	8	55	E	0	370509.214	-0.004	491.6928
64	9	56	63	9	55	E	0	370509.214	-0.002	491.6928
64	8	56	63	8	55	A	0	370513.649	0.012	491.6909
64	8	56	63	9	55	A	0	370513.649	0.014	491.6909
64	9	56	63	8	55	A	0	370513.649	0.010	491.6909
64	9	56	63	9	55	A	0	370513.649	0.013	491.6909
43	33	10	43	32	11	E	0	370517.947	0.013	456.5601
63	9	54	62	9	53	E	0	370549.467	-0.009	487.3300
63	10	54	62	10	53	E	0	370549.467	0.032	487.3300
63	9	54	62	9	53	A	0	370554.367	0.073	487.3284
63	9	54	62	10	53	A	0	370554.367	0.162	487.3284
63	10	54	62	9	53	A	0	370554.367	0.025	487.3284
63	10	54	62	10	53	A	0	370554.367	0.115	487.3284
65	7	58	64	7	57	A	0	370558.533	0.013	495.5695
65	7	58	64	8	57	A	0	370558.533	0.013	495.5695
65	8	58	64	7	57	A	0	370558.533	0.013	495.5695
65	8	58	64	8	57	A	0	370558.533	0.013	495.5695
66	6	60	65	6	59	E	1	370569.137	-0.013	627.2590
66	6	60	65	7	59	E	1	370569.137	-0.013	627.2590
66	7	60	65	6	59	E	1	370569.137	-0.013	627.2590
66	7	60	65	7	59	E	1	370569.137	-0.013	627.2590
42	33	10	42	32	11	A	0	370581.303	0.011	447.7277
42	33	9	42	32	10	A	0	370581.303	0.011	447.7277
67	5	62	66	5	61	A	1	370599.295	-0.180	630.3442
67	5	62	66	6	61	A	1	370599.295	-0.180	630.3442
67	6	62	66	5	61	A	1	370599.295	-0.180	630.3442
67	6	62	66	6	61	A	1	370599.295	-0.180	630.3442
42	33	9	42	32	10	E	0	370623.724	-0.191	447.7210
62	10	52	61	11	51	E	1	370640.602	0.008	610.7204
62	11	52	61	10	51	E	1	370644.538	0.113	610.7204
56	15	41	55	15	40	A	0	370647.060	-0.349	433.9130
66	6	60	65	6	59	E	0	370655.808	-0.036	498.9882
66	7	60	65	7	59	E	0	370655.808	-0.036	498.9882
66	6	60	65	6	59	A	0	370659.600	0.033	498.9853
66	6	60	65	7	59	A	0	370659.600	0.033	498.9853
66	7	60	65	6	59	A	0	370659.600	0.033	498.9853
66	7	60	65	7	59	A	0	370659.600	0.033	498.9853
56	15	41	55	15	40	E	0	370662.554	-0.024	433.9171
41	33	9	41	32	10	A	0	370680.701	0.361	439.0965
41	33	8	41	32	9	A	0	370680.701	0.361	439.0965
59	22	37	58	22	36	A	0	370685.151	0.446	513.5950
59	22	37	58	22	36	E	0	370689.237	0.002	513.5912
67	5	62	66	5	61	E	1	370708.247	-0.012	630.2262
67	5	62	66	6	61	E	1	370708.247	-0.012	630.2262
67	6	62	66	5	61	E	1	370708.247	-0.012	630.2262
67	6	62	66	6	61	E	1	370708.247	-0.012	630.2262
62	10	52	61	11	51	A	0	370727.600	-0.112	482.4528
62	11	52	61	10	51	A	0	370731.310	0.081	482.4527
40	33	8	40	32	9	A	0	370772.965	0.136	430.6731
40	33	7	40	32	8	A	0	370772.965	0.136	430.6731
68	4	64	67	4	63	A	1	370776.457	-0.199	632.9068
68	4	64	67	5	63	A	1	370776.457	-0.199	632.9068
68	5	64	67	4	63	A	1	370776.457	-0.199	632.9068
68	5	64	67	5	63	A	1	370776.457	-0.199	632.9068
67	5	62	66	6	61	E	0	370794.744	0.006	501.9577
67	6	62	66	5	61	E	0	370794.744	0.006	501.9577
67	5	62	66	5	61	A	0	370798.114	0.019	501.9540
67	5	62	66	6	61	A	0	370798.114	0.019	501.9540
67	6	62	66	5	61	A	0	370798.114	0.019	501.9540
67	6	62	66	6	61	A	0	370798.114	0.019	501.9540
61	11	50	60	12	49	A	1	370822.296	0.236	605.3304
61	12	50	60	12	49	A	1	370845.476	0.254	605.3304
68	4	64	67	4	63	E	1	370872.270	-0.013	632.7573
68	4	64	67	5	63	E	1	370872.270	-0.013	632.7573
68	5	64	67	4	63	E	1	370872.270	-0.013	632.7573
68	5	64	67	5	63	E	1	370872.270	-0.013	632.7573
61	12	50	60	11	49	A	1	370885.925	0.256	605.3291
68	4	64	67	4	63	E	0	370958.758	0.020	504.4927
68	5	64	67	5	63	E	0	370958.758	0.020	504.4927
68	4	64	67	4	63	A	0	370961.691	0.011	504.4880
68	4	64	67	5	63	A	0	370961.691	0.011	504.4880
68	5	64	67	4	63	A	0	370961.691	0.011	504.4880
68	5	64	67	5	63	A	0	370961.691	0.011	504.4880
69	3	66	68	3	65	A	1	370972.268	-0.142	635.0499
69	3	66	68	4	65	A	1	370972.268	-0.142	635.0499
69	4	66	68	3	65	A	1	370972.268	-0.142	635.0499
69	4	66	68	4	65	A	1	370972.268	-0.142	635.0499
61	11	50	60	12	49	E	1	371010.760	0.082	605.2844
61	12	50	60	12	49	E	1	371038.184	-0.135	605.2844
69	3	66	68	3	65	E	1	371052.488	0.063	634.8618
69	3	66	68	4	65	E	1	371052.488	0.063	634.8618
69	4	66	68	3	65	E	1	371052.488	0.063	634.8618
69	4	66	68	4	65	E	1	371052.488	0.063	634.8618
61	11	50	60	12	49	E	0	371088.256	0.014	477.0234
61	11	50	60	12	49	A	0	371093.884	-0.219	477.0219
61	12	50	60	12	49	E	0	371113.620	-0.059	477.0234
61	12	50	60	12	49	A	0	371119.677	-0.011	477.0219
61	11	50	60	11	49	E	0	371132.424	-0.105	477.0219
69	3	66	68	3	65	E	0	371139.329	-0.020	506.6031
69	4	66	68	4	65	E	0	371139.329	-0.020	506.6031
69	3	66	68	3	65	A	0	371141.842	0.038	506.5971
69	3	66	68	4	65	A	0	371141.842	0.038	506.5971
69	4	66	68	3	65	A	0	371141.842	0.038	506.5971
69	4	66	68	4	65	A	0	371141.842	0.038	506.5971
61	12	50	60	11	49	A	0	371164.219	-0.004	477.0204
70	2	68	69	2	67	A	1	371181.342	-0.041	636.7820
70	2	68	69	3	67	A	1	371181.342	-0.041	636.7820
70	3	68	69	2	67	A	1	371181.342	-0.041	636.7820
70	3	68	69	3	67	A	1	371181.342	-0.041	636.7820
70	2	68	69	2	67	E	1	371242.499	0.092	636.5474
70	2	68	69	3	67	E	1	371242.499	0.092	636.5474
70	3	68	69	2	67	E	1	371242.499	0.092	636.5474
70	3	68	69	3	67	E	1	371242.499	0.092	636.5474

J'	K_a'	K_c'	J''	K_a''	K_c''	Sym.	$v_{t_{CH_3}}$	$\nu_{Obs.}$	$\nu_{Obs.} - \nu_{Calc.}$	E_{low}
26	19	7	25	18	7	E	0	371254.388	-0.033	195.7589
26	19	8	25	18	8	E	0	371279.492	0.017	195.7469
26	19	8	25	18	7	A	0	371301.788	-0.048	195.7586
26	19	7	25	18	8	A	0	371301.788	-0.048	195.7586
70	2	68	69	2	67	E	0	371330.709	0.102	508.2966
70	3	68	69	3	67	E	0	371330.709	0.102	508.2966
70	2	68	69	2	67	A	0	371332.490	0.014	508.2892
70	2	68	69	3	67	A	0	371332.490	0.014	508.2892
70	3	68	69	2	67	A	0	371332.490	0.014	508.2892
70	3	68	69	3	67	A	0	371332.490	0.014	508.2892
60	12	48	59	13	47	E	0	371411.366	0.009	470.9773
60	12	48	59	13	47	A	0	371416.089	-0.053	470.9755
58	17	42	57	17	41	A	0	371459.033	0.082	467.3363
38	13	25	37	12	26	A	0	371502.077	0.198	240.9655
60	13	48	59	13	47	E	0	371809.418	-0.034	470.9773
60	13	48	59	13	47	A	0	371816.278	0.080	470.9755
28	18	10	27	17	10	E	0	372046.720	-0.053	200.0339
28	18	11	27	17	11	E	0	372066.789	-0.011	200.0211
60	12	48	59	12	47	E	0	372070.185	-0.026	470.9553
60	12	48	59	12	47	A	0	372077.957	-0.168	470.9535
28	18	10	27	17	11	A	0	372093.972	-0.045	200.0319
28	18	11	27	17	10	A	0	372093.972	-0.045	200.0319
60	13	48	59	12	47	E	0	372468.144	-0.163	470.9553
60	30	30	59	30	29	A	0	372469.697	0.309	603.0778
60	30	31	59	30	30	A	0	372469.697	0.309	603.0778
60	30	30	59	30	29	E	0	372469.697	-0.219	603.0687
60	30	31	59	30	30	E	0	372471.375	-0.313	603.0652
60	13	48	59	12	47	A	0	372478.181	-0.000	470.9535
59	14	46	58	14	45	E	0	372499.154	0.389	464.2667
59	14	46	58	14	45	A	0	372504.576	-0.164	464.2647
58	16	43	57	16	42	A	0	372547.827	-0.040	462.0267
30	17	13	29	16	13	E	0	372741.661	-0.066	205.5623
30	17	14	29	16	14	E	0	372756.354	0.061	205.5491
30	17	14	29	16	13	A	0	372788.070	-0.053	205.5587
30	17	13	29	16	14	A	0	372788.070	-0.053	205.5587
36	14	23	35	13	23	E	0	372851.758	-0.164	229.9816
36	14	23	35	13	22	A	0	372876.737	-0.043	229.9870
36	14	22	35	13	23	A	0	372916.503	-0.016	229.9858
59	20	40	58	20	39	A	0	373030.442	-0.016	498.5576
32	16	16	31	15	16	E	0	373250.386	-0.080	212.3689
32	16	17	31	15	17	E	0	373259.068	-0.012	212.3555
32	16	17	31	15	16	A	0	373295.290	-0.030	212.3637
34	15	19	33	14	19	E	0	373405.016	-0.035	220.4919
34	15	20	33	14	20	E	0	373407.011	-0.021	220.4786
34	15	20	33	14	19	A	0	373447.614	0.239	220.4852
58	17	41	57	17	40	A	0	373534.901	-0.056	467.4483
58	17	41	57	17	40	E	0	373545.510	0.010	467.4481
39	13	27	38	12	26	E	0	374395.212	0.051	249.0986
39	13	27	38	12	26	A	0	374521.000	-0.009	249.0942
59	19	41	58	19	40	A	0	374617.746	-0.249	491.7568
60	13	47	59	14	46	A	0	374711.661	-0.033	476.6901
60	13	47	59	14	46	E	0	374717.337	-0.055	476.6919
60	25	36	59	25	35	A	0	374879.953	0.018	551.7567
60	25	35	59	25	34	A	0	374879.953	0.018	551.7567
59	13	46	58	13	45	E	0	374902.479	0.066	464.0367
59	13	46	58	13	45	A	0	374916.485	-0.017	464.0339
65	8	57	64	8	56	A	1	375385.611	-0.039	632.3740
65	8	57	64	9	56	A	1	375385.611	-0.037	632.3740
65	9	57	64	8	56	A	1	375385.611	-0.039	632.3740
65	9	57	64	9	56	A	1	375385.611	-0.038	632.3740
64	9	55	63	9	54	A	1	375402.256	0.016	628.0008
64	9	55	63	10	54	A	1	375402.256	0.058	628.0008
64	10	55	63	9	54	A	1	375402.256	-0.006	628.0008
64	10	55	63	10	54	A	1	375402.256	0.036	628.0008
66	7	59	65	7	58	A	1	375448.696	-0.101	636.2698
66	7	59	65	8	58	A	1	375448.696	-0.101	636.2698
66	8	59	65	7	58	A	1	375448.696	-0.101	636.2698
66	8	59	65	8	58	A	1	375448.696	-0.101	636.2698
65	8	57	64	8	56	E	1	375525.742	0.005	632.3201
65	8	57	64	9	56	E	1	375525.742	0.006	632.3201
65	9	57	64	8	56	E	1	375525.742	0.004	632.3201
65	9	57	64	9	56	E	1	375525.742	0.005	632.3201
63	10	53	62	11	52	A	1	375541.330	0.200	623.1227
63	11	53	62	10	52	A	1	375542.850	-0.001	623.1227
64	9	55	63	9	54	E	1	375554.184	-0.011	627.9569
64	9	55	63	10	54	E	1	375554.184	0.042	627.9569
64	10	55	63	9	54	E	1	375554.184	-0.039	627.9569
64	10	55	63	10	54	E	1	375554.184	0.014	627.9569
67	6	61	66	6	60	A	1	375565.185	-0.158	639.7086
67	6	61	66	7	60	A	1	375565.185	-0.158	639.7086
67	7	61	66	6	60	A	1	375565.185	-0.158	639.7086
67	7	61	66	7	60	A	1	375565.185	-0.158	639.7086
60	24	37	59	24	36	A	0	375574.770	-0.008	542.7276
66	7	59	65	7	58	E	1	375578.344	-0.003	636.2011
66	7	59	65	8	58	E	1	375578.344	-0.003	636.2011
66	8	59	65	7	58	E	1	375578.344	-0.003	636.2011
66	8	59	65	8	58	E	1	375578.344	-0.003	636.2011
65	8	57	64	8	56	E	0	375614.721	-0.027	504.0517
65	9	57	64	9	56	E	0	375614.721	-0.026	504.0517
65	8	57	64	8	56	A	0	375619.091	-0.007	504.0499
65	8	57	64	9	56	A	0	375619.091	-0.006	504.0499
65	9	57	64	8	56	A	0	375619.091	-0.008	504.0499
65	9	57	64	9	56	A	0	375619.091	-0.007	504.0499
64	9	55	63	9	54	E	0	375642.150	0.027	499.6902
64	10	55	63	10	54	E	0	375642.150	0.049	499.6902
64	9	55	63	9	54	A	0	375646.842	-0.007	499.6887
64	9	55	63	10	54	A	0	375646.842	0.041	499.6887
64	10	55	63	9	54	A	0	375646.842	-0.032	499.6887
64	10	55	63	10	54	A	0	375646.842	0.016	499.6887
70	34	36	70	33	37	A	0	375650.089	-0.001	786.6525
70	34	37	70	33	38	A	0	375650.089	-0.001	786.6525
66	7	59	65	7	58	E	0	375667.509	0.007	507.9322
66	8	59	65	8	58	E	0	375667.509	0.007	507.9322
66	7	59	65	7	58	A	0	375671.525	0.007	507.9300
66	7	59	65	8	58	A	0	375671.525	0.007	507.9300
66	8	59	65	7	58	A	0	375671.525	0.007	507.9300
66	8	59	65	8	58	A	0	375671.525	0.007	507.9300
67	6	61	66	6	60	E	1	375684.490	-0.002	639.6199
67	6	61	66	7	60	E	1	375684.490	-0.002	639.6199
67	7	61	66	6	60	E	1	375684.490	-0.002	639.6199
67	7	61	66	7	60	E	1	375684.490	-0.002	639.6199
23	21	2	22	20	2	E	0	375696.573	-0.025	195.4241
63	10	53	62	11	52	E	1	375707.953	0.091	623.0837
68	5	63	67	5	62	A	1	375718.335	-0.128	642.7061
68	5	63	67	6	62	A	1	375718.335	-0.128	642.7061
68	6	63	67	5	62	A	1	375718.335	-0.128	642.7061

J'	K'_a	K'_c	J''	K''_a	K''_c	Sym.	$v_{t_{CH_3}}$	$\nu_{Obs.}$	$\nu_{Obs.} - \nu_{Calc.}$	E_{low}
68	6	63	67	6	62	A	1	375718.335	-0.128	642.7061
23	21	3	22	20	3	E	0	375730.406	0.046	195.4138
23	21	3	22	20	2	A	0	375742.310	-0.063	195.4269
23	21	2	22	20	3	A	0	375742.310	-0.063	195.4269
67	6	61	66	6	60	E	0	375773.302	-0.039	511.3520
67	7	61	66	7	60	E	0	375773.302	-0.039	511.3520
67	6	61	66	6	60	A	0	375777.057	0.030	511.3491
67	6	61	66	7	60	A	0	375777.057	0.030	511.3491
67	7	61	66	6	60	A	0	375777.057	0.030	511.3491
67	7	61	66	7	60	A	0	375777.057	0.030	511.3491
68	5	63	67	5	62	E	1	375826.638	0.229	642.5917
68	5	63	67	6	62	E	1	375826.638	0.229	642.5917
68	6	63	67	5	62	E	1	375826.638	0.229	642.5917
68	6	63	67	6	62	E	1	375826.638	0.229	642.5917
62	11	51	61	12	50	A	1	375864.805	0.226	617.7005
69	4	65	68	4	64	A	1	375896.917	-0.123	645.2746
69	4	65	68	5	64	A	1	375896.917	-0.123	645.2746
69	5	65	68	4	64	A	1	375896.917	-0.123	645.2746
69	5	65	68	5	64	A	1	375896.917	-0.123	645.2746
62	12	51	61	11	50	A	1	375901.138	0.228	617.6997
68	5	63	67	5	62	E	0	375914.859	0.000	514.3261
68	6	63	67	6	62	E	0	375914.859	0.000	514.3261
68	5	63	67	5	62	A	0	375918.227	0.038	514.3225
68	5	63	67	6	62	A	0	375918.227	0.038	514.3225
68	6	63	67	5	62	A	0	375918.227	0.038	514.3225
68	6	63	67	6	62	A	0	375918.227	0.038	514.3225
69	4	65	68	4	64	E	1	375992.155	0.030	645.1282
69	4	65	68	5	64	E	1	375992.155	0.030	645.1282
69	5	65	68	4	64	E	1	375992.155	0.030	645.1282
69	5	65	68	5	64	E	1	375992.155	0.030	645.1282
69	34	35	69	33	36	A	0	376065.086	0.132	772.1366
69	34	36	69	33	37	A	0	376065.086	0.132	772.1366
69	4	65	68	4	64	E	0	376080.414	0.026	516.8666
69	5	65	68	5	64	E	0	376080.414	0.026	516.8666
69	4	65	68	4	64	A	0	376083.346	0.033	516.8620
69	4	65	68	5	64	A	0	376083.346	0.033	516.8620
69	5	65	68	4	64	A	0	376083.346	0.033	516.8620
69	5	65	68	5	64	A	0	376083.346	0.033	516.8620
70	3	67	69	3	66	A	1	376093.362	-0.216	647.4242
70	3	67	69	4	66	A	1	376093.362	-0.216	647.4242
70	3	67	69	4	66	A	1	376093.362	-0.216	647.4242
70	4	67	69	3	66	A	1	376093.362	-0.216	647.4242
62	11	51	61	12	50	E	0	376129.964	-0.193	489.4024
62	11	51	61	12	50	A	0	376135.873	-0.032	489.4011
70	3	67	69	3	66	E	1	376173.403	0.117	647.2388
70	3	67	69	4	66	E	1	376173.403	0.117	647.2388
70	4	67	69	3	66	E	1	376173.403	0.117	647.2388
70	4	67	69	4	66	E	1	376173.403	0.117	647.2388
62	12	51	61	11	50	A	0	376176.067	-0.014	489.4003
70	3	67	69	3	66	E	0	376261.896	0.025	518.9829
70	4	67	69	4	66	E	0	376261.896	0.025	518.9829
70	3	67	69	4	66	A	0	376264.338	0.021	518.9771
70	4	67	69	3	66	A	0	376264.338	0.021	518.9771
60	23	38	59	23	37	A	0	376380.549	-0.119	534.1251
60	23	38	59	23	37	E	0	376384.926	0.186	534.1130
68	34	34	68	33	35	A	0	376461.490	0.067	757.8341
68	34	35	68	33	36	A	0	376461.490	0.067	757.8341
59	18	42	58	18	41	E	0	376543.272	-0.024	485.4703
25	20	5	24	19	5	E	0	376553.401	-0.049	197.6535
61	12	49	60	13	48	E	0	376556.561	0.067	483.3795
61	12	49	60	13	48	A	0	376561.914	0.025	483.3780
25	20	6	24	19	6	E	0	376583.085	0.025	197.6423
25	20	5	24	19	6	A	0	376600.310	-0.054	197.6548
25	20	6	24	19	5	A	0	376600.310	-0.054	197.6548
61	13	49	60	13	48	E	1	376723.507	0.216	611.6307
67	34	33	67	33	34	A	0	376840.163	0.003	743.7449
67	34	34	67	33	35	A	0	376840.163	0.003	743.7449
67	34	34	67	33	35	E	0	376843.997	-0.109	743.7324
67	34	33	67	33	34	E	0	376881.971	0.416	743.7395
39	13	26	38	12	27	A	0	377171.015	-0.059	249.0220
61	13	49	60	12	48	E	0	377192.638	-0.051	483.3662
61	13	49	60	12	48	A	0	377201.437	0.178	483.3647
39	13	26	38	12	27	E	0	377206.932	0.030	249.0252
60	22	39	59	22	38	A	0	377328.079	0.018	525.9597
60	22	38	59	22	37	E	0	377333.026	0.057	525.9560
27	19	8	26	18	8	E	0	377383.193	-0.051	201.1116
27	19	9	26	18	9	E	0	377408.267	0.029	201.0995
27	19	9	26	18	8	A	0	377430.613	-0.035	201.1112
27	19	8	26	18	9	A	0	377430.613	-0.035	201.1112
65	34	31	65	33	32	A	0	377546.903	-0.054	716.2049
65	34	32	65	33	33	A	0	377546.903	-0.054	716.2049
61	33	29	60	33	28	A	0	377865.692	-0.264	651.0674
61	33	28	60	33	27	A	0	377865.692	-0.264	651.0674
61	33	28	60	33	27	E	0	377865.692	-0.069	651.0621
61	33	29	60	33	28	E	0	377867.286	-0.282	651.0544
64	34	30	64	33	31	A	0	377876.225	-0.002	702.7537
64	34	31	64	33	32	A	0	377876.225	-0.002	702.7537
64	34	31	64	33	32	E	0	377880.945	-0.165	702.7409
64	34	30	64	33	31	E	0	377918.510	0.146	702.7483
29	18	11	28	17	11	E	0	378154.298	-0.063	205.8106
29	18	12	28	17	12	E	0	378174.287	-0.004	205.7979
63	34	29	63	33	30	A	0	378189.986	-0.194	689.5145
63	34	30	63	33	31	A	0	378189.986	-0.194	689.5145
63	34	30	63	33	31	E	0	378195.162	-0.187	689.5017
29	18	11	28	17	12	A	0	378201.545	-0.040	205.8086
29	18	12	28	17	11	A	0	378201.545	-0.040	205.8086
63	34	29	63	33	30	E	0	378232.458	-0.080	689.5092
56	14	43	55	13	42	E	0	378261.139	0.011	427.3199
40	13	28	39	12	27	E	0	378364.248	0.017	257.4323
59	17	43	58	17	42	A	0	378387.452	-0.126	479.7269
37	14	24	36	13	24	E	0	378498.296	0.311	237.5460
37	14	24	36	13	23	A	0	378500.599	-0.046	237.5518
62	34	28	62	33	29	E	0	378531.944	0.005	676.4819
37	14	23	36	13	24	A	0	378581.581	-0.039	237.5495
57	15	42	56	15	41	A	0	378644.218	-0.018	446.2765
57	15	42	56	15	41	E	0	378655.265	-0.029	446.2810
61	34	27	61	33	28	A	0	378774.291	-0.050	663.6716
61	34	28	61	33	29	A	0	378774.291	-0.050	663.6716
61	34	28	61	33	29	E	0	378780.039	-0.003	663.6587
31	17	14	30	16	14	E	0	378810.803	-0.054	211.7689
31	17	15	30	16	15	E	0	378825.281	0.011	211.7557
31	17	15	30	16	14	A	0	378857.188	-0.033	211.7653
31	17	14	30	16	15	A	0	378857.188	-0.033	211.7653
59	16	44	58	16	43	A	0	379022.561	-0.102	474.4535
59	16	44	58	16	43	E	0	379026.448	-0.031	474.4544
60	34	26	60	33	27	E	0	379088.500	-0.052	651.0621

J'	K'_a	K'_c	J''	K''_a	K''_c	Sym.	$v_{t_{CH_3}}$	$\nu_{Obs.}$	$\nu_{Obs.} - \nu_{Calc.}$	E_{low}
60	13	47	59	13	46	E	0	379207.199	-0.040	476.5421
60	13	47	59	13	46	A	0	379219.477	-0.048	476.5397
33	16	17	32	15	17	E	0	379251.627	-0.036	219.0135
33	16	18	32	15	18	E	0	379259.999	-0.026	219.0001
35	15	20	34	14	20	E	0	379284.338	-0.047	227.5866
35	15	21	34	14	21	E	0	379285.928	-0.010	227.5733
33	16	18	32	15	17	A	0	379296.441	-0.022	219.0082
59	34	25	59	33	26	A	0	379303.544	-0.118	638.6743
59	34	26	59	33	27	A	0	379303.544	-0.118	638.6743
59	34	26	59	33	27	E	0	379309.686	-0.161	638.6612
35	15	21	34	14	20	A	0	379326.142	-0.123	227.5798
35	15	20	34	14	21	A	0	379327.555	0.005	227.5798
59	34	25	59	33	26	E	0	379346.782	-0.002	638.6690
59	14	46	58	13	45	E	0	379392.258	-0.002	464.0367
59	14	46	58	13	45	A	0	379424.449	0.116	464.0339
58	34	24	58	33	25	A	0	379548.992	-0.013	626.4921
58	34	25	58	33	26	A	0	379548.992	-0.013	626.4921
58	34	24	58	33	25	E	0	379592.322	0.032	626.4868
57	34	23	57	33	24	A	0	379782.038	-0.067	614.5206
57	34	24	57	33	25	A	0	379782.038	-0.067	614.5206
57	34	24	57	33	25	E	0	379788.620	-0.108	614.5074
57	34	23	57	33	24	E	0	379825.633	0.090	614.5153
60	20	41	59	20	40	A	0	379842.390	-0.001	511.0005
60	20	40	59	20	39	E	0	379858.178	-0.057	511.0006
60	20	40	59	20	39	A	0	379860.934	0.017	511.0012
56	34	22	56	33	23	A	0	380003.390	-0.027	602.7595
56	34	23	56	33	24	A	0	380003.390	-0.027	602.7595
56	34	23	56	33	24	E	0	380010.108	-0.135	602.7463
56	34	22	56	33	23	E	0	380046.972	-0.027	602.7542
55	34	21	55	33	22	A	0	380213.290	-0.100	591.2087
55	34	22	55	33	23	A	0	380213.290	-0.100	591.2087
55	34	22	55	33	23	E	0	380220.356	-0.051	591.1955
55	34	21	55	33	22	E	0	380257.010	-0.095	591.2034
58	16	42	57	16	41	A	0	380392.453	-0.047	462.6041
54	34	21	54	33	22	E	0	380419.618	-0.033	579.8547
54	34	20	54	33	21	E	0	380456.323	0.032	579.8626
66	8	58	65	8	57	A	1	380491.564	-0.037	644.8955
66	8	58	65	9	57	A	1	380491.564	-0.037	644.8955
66	9	58	65	8	57	A	1	380491.564	-0.038	644.8955
66	9	58	65	9	57	A	1	380491.564	-0.037	644.8955
65	9	56	64	9	55	A	1	380496.771	0.037	640.5229
65	9	56	64	10	55	A	1	380496.771	0.059	640.5229
65	10	56	64	9	55	A	1	380496.771	0.025	640.5229
65	10	56	64	10	55	A	1	380496.771	0.047	640.5229
67	7	60	66	7	59	A	1	380561.370	-0.076	648.7934
67	7	60	66	8	59	A	1	380561.370	-0.076	648.7934
67	7	60	66	8	59	A	1	380561.370	-0.076	648.7934
67	8	60	66	7	59	A	1	380561.370	-0.076	648.7934
53	34	19	53	33	20	A	0	380600.909	-0.113	568.7369
53	34	20	53	33	21	A	0	380600.909	-0.113	568.7369
53	34	20	53	33	21	E	0	380608.129	-0.265	568.7237
66	8	58	65	8	57	E	1	380629.509	0.010	644.8463
66	8	58	65	9	57	E	1	380629.509	0.011	644.8463
66	9	58	65	8	57	E	1	380629.509	0.010	644.8463
66	9	58	65	9	57	E	1	380629.509	0.010	644.8463
65	9	56	64	9	55	E	1	380645.754	-0.002	640.4840
65	9	56	64	10	55	E	1	380645.754	0.026	640.4840
65	10	56	64	9	55	E	1	380645.754	-0.017	640.4840
65	10	56	64	10	55	E	1	380645.754	0.011	640.4840
68	6	62	67	6	61	A	1	380681.795	-0.136	652.2362
68	6	62	67	7	61	A	1	380681.795	-0.136	652.2362
68	7	62	67	6	61	A	1	380681.795	-0.136	652.2362
68	7	62	67	7	61	A	1	380681.795	-0.136	652.2362
67	7	60	66	7	59	E	1	380689.329	-0.033	648.7290
67	7	60	66	8	59	E	1	380689.329	-0.032	648.7290
67	8	60	66	7	59	E	1	380689.329	-0.033	648.7290
67	8	60	66	8	59	E	1	380689.329	-0.033	648.7290
66	8	58	65	8	57	E	0	380721.102	-0.027	516.5808
66	9	58	65	9	57	E	0	380721.102	-0.027	516.5808
66	8	58	65	8	57	A	0	380725.402	-0.010	516.5792
66	8	58	65	9	57	A	0	380725.402	-0.009	516.5792
66	9	58	65	8	57	A	0	380725.402	-0.010	516.5792
66	9	58	65	9	57	A	0	380725.402	-0.009	516.5792
65	9	56	64	9	55	E	0	380736.602	-0.028	512.2202
65	10	56	64	10	55	E	0	380736.602	-0.016	512.2202
65	9	56	64	9	55	A	0	380741.243	-0.023	512.2190
65	9	56	64	10	55	A	0	380741.243	0.003	512.2190
65	10	56	64	9	55	A	0	380741.243	-0.036	512.2190
65	10	56	64	10	55	A	0	380741.243	-0.011	512.2190
61	26	35	60	26	34	A	0	380752.503	-0.010	573.6890
61	26	36	60	26	35	A	0	380752.503	-0.010	573.6890
61	26	36	60	26	35	E	0	380754.664	-0.111	573.6771
61	26	35	60	26	34	E	0	380756.219	0.028	573.6799
67	7	60	66	7	59	E	0	380780.876	-0.008	520.4631
67	8	60	66	8	59	E	0	380780.876	-0.008	520.4631
67	7	60	66	7	59	A	0	380784.860	0.009	520.4611
67	7	60	66	8	59	A	0	380784.860	0.009	520.4611
67	8	60	66	7	59	A	0	380784.860	0.009	520.4611
67	8	60	66	8	59	A	0	380784.860	0.009	520.4611
68	6	62	67	6	61	E	1	380799.871	-0.018	652.1514
68	6	62	67	7	61	E	1	380799.871	-0.018	652.1514
68	7	62	67	6	61	E	1	380799.871	-0.018	652.1514
68	7	62	67	7	61	E	1	380799.871	-0.018	652.1514
61	13	48	60	14	47	A	0	380813.407	-0.140	489.2853
61	13	48	60	14	47	E	0	380814.971	0.090	489.2869
69	5	64	68	5	63	A	1	380837.211	-0.143	655.2387
69	5	64	68	6	63	A	1	380837.211	-0.143	655.2387
69	6	64	68	5	63	A	1	380837.211	-0.143	655.2387
69	6	64	68	6	63	A	1	380837.211	-0.143	655.2387
55	14	42	54	13	41	E	0	380842.023	0.005	415.3378
68	6	62	67	6	61	E	0	380891.005	0.109	523.8864
68	7	62	67	7	61	E	0	380891.005	0.109	523.8864
68	6	62	67	6	61	A	0	380894.592	0.047	523.8837
68	6	62	67	7	61	A	0	380894.592	0.047	523.8837
68	7	62	67	6	61	A	0	380894.592	0.047	523.8837
68	7	62	67	7	61	A	0	380894.592	0.047	523.8837
63	11	52	62	12	51	A	1	380909.803	0.094	630.2384
63	12	52	62	12	51	A	1	380917.352	0.208	630.2384
69	5	64	68	5	63	E	1	380944.485	0.014	655.1279
69	5	64	68	6	63	E	1	380944.485	0.014	655.1279
69	6	64	68	5	63	E	1	380944.485	0.014	655.1279
69	6	64	68	6	63	E	1	380944.485	0.014	655.1279
22	22	0	21	21	0	E	0	380975.401	-0.018	198.7216
22	22	1	21	21	1	E	0	381013.011	0.187	198.7125
70	4	66	69	4	65	A	1	381017.202	-0.053	657.8132
70	4	66	69	5	65	A	1	381017.202	-0.053	657.8132

J'	K'_a	K'_c	J''	K''_a	K''_c	Sym.	$v_{t_{CH_3}}$	$\nu_{Obs.}$	$\nu_{Obs.} - \nu_{Calc.}$	E_{low}
70	5	66	69	4	65	A	1	381017.202	-0.053	657.8132
70	5	66	69	5	65	A	1	381017.202	-0.053	657.8132
22	22	0	21	21	1	A	0	381019.384	-0.046	198.7260
22	22	1	21	21	0	A	0	381019.384	-0.046	198.7260
69	5	64	68	5	63	E	0	381034.935	0.041	526.8653
69	6	64	68	6	63	E	0	381034.935	0.041	526.8653
69	5	64	68	5	63	A	0	381038.250	0.050	526.8618
69	5	64	68	6	63	A	0	381038.250	0.050	526.8618
69	6	64	68	5	63	A	0	381038.250	0.050	526.8618
69	6	64	68	6	63	A	0	381038.250	0.050	526.8618
63	11	52	62	12	51	E	1	381090.162	0.098	630.2051
70	4	66	69	4	65	E	1	381111.878	0.072	657.6700
70	4	66	69	5	65	E	1	381111.878	0.072	657.6700
70	5	66	69	4	65	E	1	381111.878	0.072	657.6700
70	5	66	69	5	65	E	1	381111.878	0.072	657.6700
63	11	52	62	12	51	A	0	381179.695	-0.337	501.9482
63	11	52	62	11	51	A	0	381194.604	-0.020	501.9477
70	4	66	69	4	65	E	0	381201.869	-0.010	529.4113
70	5	66	69	5	65	E	0	381201.869	-0.010	529.4113
70	4	66	69	4	65	A	0	381204.818	0.030	529.4068
70	4	66	69	5	65	A	0	381204.818	0.030	529.4068
70	5	66	69	4	65	A	0	381204.818	0.030	529.4068
70	5	66	69	5	65	A	0	381204.818	0.030	529.4068
49	34	15	49	33	16	A	0	381258.248	-0.114	526.3072
49	34	16	49	33	17	A	0	381258.248	-0.114	526.3072
49	34	15	49	33	16	E	0	381302.693	-0.013	526.3019
61	25	37	60	25	36	A	0	381393.169	-0.186	564.2613
61	25	36	60	25	35	A	0	381393.169	-0.186	564.2613
48	34	14	48	33	15	E	0	381444.898	0.119	516.2171
41	13	29	40	12	28	E	0	381519.301	-0.179	266.0206
47	34	13	47	33	14	A	0	381534.265	0.128	506.3462
47	34	14	47	33	15	A	0	381534.265	0.128	506.3462
62	12	50	61	13	49	E	0	381641.771	0.048	495.9480
62	13	50	61	13	49	E	0	381782.778	-0.024	495.9480
62	13	50	61	13	49	A	0	381789.363	0.122	495.9467
24	21	3	23	20	3	E	0	381839.669	-0.050	200.1467
24	21	4	23	20	4	E	0	381873.499	0.044	200.1365
24	21	4	23	20	3	A	0	381885.407	-0.087	200.1495
24	21	3	23	20	4	A	0	381885.407	-0.087	200.1495
44	34	10	44	33	11	A	0	381889.539	-0.037	477.9677
44	34	11	44	33	12	A	0	381889.539	-0.037	477.9677
44	34	11	44	33	12	E	0	381898.182	0.064	477.9543
44	34	10	44	33	11	E	0	381934.285	0.035	477.9625
43	34	9	43	33	10	A	0	381993.953	0.114	468.9244
43	34	10	43	33	11	A	0	381993.953	0.114	468.9244
62	13	50	61	12	49	E	0	382020.901	-0.001	495.9401
62	13	50	61	12	49	A	0	382028.535	-0.021	495.9387
60	14	47	59	13	46	E	0	382079.526	-0.018	476.5421
61	24	37	60	24	36	A	0	382129.780	-0.067	555.2554
61	24	38	60	24	37	A	0	382129.780	-0.065	555.2554
60	15	46	59	15	45	E	0	382582.183	0.046	481.9951
60	15	46	59	15	45	A	0	382585.436	-0.029	481.9934
61	14	48	60	14	47	E	0	382624.204	-0.031	489.2869
61	14	48	60	14	47	A	0	382630.927	0.068	489.2853
26	20	6	25	19	6	E	0	382690.465	-0.014	202.7940
26	20	7	25	19	7	E	0	382720.084	0.038	202.7828
26	20	6	25	19	7	A	0	382737.341	-0.047	202.7952
26	20	7	25	19	6	A	0	382737.341	-0.047	202.7952
61	23	39	60	23	38	A	0	382986.001	-0.017	546.6799
61	23	39	60	23	38	E	0	382990.502	0.150	546.6679
40	13	27	39	12	28	A	0	383029.157	-0.057	257.3050
40	13	27	39	12	28	E	0	383034.124	-0.043	257.3085
28	19	9	27	18	9	E	0	383507.004	-0.083	206.6731
60	18	43	59	18	42	A	0	383530.337	0.122	498.0330
28	19	10	27	18	10	E	0	383532.012	0.001	206.6611
60	18	43	59	18	42	E	0	383545.347	0.002	498.0304
28	19	10	27	18	9	A	0	383554.439	-0.038	206.6727
28	19	9	27	18	10	A	0	383554.439	-0.038	206.6727
42	13	30	41	12	29	E	0	383596.770	-0.064	274.8726
44	13	32	43	12	31	A	0	383640.577	-0.003	293.3922
42	13	30	41	12	29	A	0	383676.218	0.044	274.8677
61	13	48	60	13	47	E	0	383687.074	-0.112	489.1911
61	13	48	60	13	47	A	0	383698.067	0.129	489.1891
61	22	39	60	22	38	A	0	383995.855	-0.130	538.5460
61	22	39	60	22	38	E	0	384000.935	0.049	538.5425
38	14	25	37	13	24	A	0	384029.783	-0.036	245.3413
38	14	24	37	13	24	E	0	384059.193	-0.070	245.3478
38	14	25	37	13	25	E	0	384074.541	-0.053	245.3343
38	14	24	37	13	25	A	0	384190.318	-0.031	245.3368
30	18	12	29	17	12	E	0	384253.360	-0.058	211.7977
30	18	13	29	17	13	E	0	384273.265	0.026	211.7850
30	18	12	29	17	13	A	0	384300.591	-0.030	211.7957
30	18	13	29	17	12	A	0	384300.591	-0.030	211.7957
43	13	31	42	12	30	E	0	384335.142	-0.033	283.9968
43	13	31	42	12	30	A	0	384417.038	-0.046	283.9914
60	18	42	59	18	41	A	0	384497.130	-0.058	498.0811
60	18	42	59	18	41	E	0	384501.377	0.016	498.0798
32	17	15	31	16	15	E	0	384865.851	-0.079	218.1877
54	14	41	53	13	40	E	0	384872.895	-0.005	403.4932
32	17	16	31	16	16	E	0	384880.178	0.009	218.1746
32	17	16	31	16	15	A	0	384912.146	-0.113	218.1840
32	17	15	31	16	16	A	0	384912.146	-0.114	218.1840
36	15	21	35	14	21	E	0	385123.914	-0.340	234.8996
36	15	22	35	14	22	E	0	385125.459	0.130	234.8863
36	15	22	35	14	21	A	0	385165.272	0.029	234.8927
36	15	21	35	14	22	A	0	385168.130	-0.023	234.8927
61	21	41	60	21	40	A	0	385207.617	-0.040	530.8691
61	21	40	60	21	39	A	0	385211.312	-0.052	530.8693
34	16	18	33	15	18	E	0	385229.697	-0.051	225.8728
34	16	19	33	15	19	E	0	385237.877	0.049	225.8594
60	17	44	59	17	43	A	0	385248.599	-0.071	492.3485
60	17	44	59	17	43	E	0	385256.592	0.077	492.3481
34	16	19	33	15	18	A	0	385274.467	-0.018	225.8675
59	14	45	58	14	44	A	0	385422.001	0.008	468.3989
61	14	48	60	13	47	E	0	385496.408	-0.132	489.1911
61	14	48	60	13	47	A	0	385515.221	-0.030	489.1891
66	9	57	65	9	56	A	1	385592.847	0.019	653.2149
66	9	57	65	10	56	A	1	385592.847	0.031	653.2149
66	10	57	65	9	56	A	1	385592.847	0.013	653.2149
66	10	57	65	10	56	A	1	385592.847	0.025	653.2149
67	8	59	66	8	58	A	1	385598.242	-0.030	657.5873
67	8	59	66	9	58	A	1	385598.242	-0.029	657.5873
67	9	59	66	8	58	A	1	385598.242	-0.030	657.5873
67	9	59	66	9	58	A	1	385598.242	-0.029	657.5873
68	7	61	67	7	60	A	1	385674.254	-0.100	661.4876
68	7	61	67	8	60	A	1	385674.254	-0.100	661.4876

J'	K'_a	K'_c	J''	K''_a	K''_c	Sym.	$v_{t_{CH_3}}$	$\nu_{Obs.}$	$\nu_{Obs.} - \nu_{Calc.}$	E_{low}
68	8	61	67	7	60	A	1	385674.254	-0.100	661.4876
68	8	61	67	8	60	A	1	385674.254	-0.100	661.4876
65	10	55	64	10	54	A	1	385694.303	0.041	648.3454
65	10	55	64	11	54	A	1	385694.303	0.372	648.3455
65	11	55	64	10	54	A	1	385694.303	-0.138	648.3454
65	11	55	64	11	54	A	1	385694.303	0.193	648.3455
67	8	59	66	8	58	E	1	385734.016	-0.008	657.5428
67	8	59	66	9	58	E	1	385734.016	-0.008	657.5428
67	9	59	66	8	58	E	1	385734.016	-0.008	657.5428
67	9	59	66	9	58	E	1	385734.016	-0.008	657.5428
66	9	57	65	9	56	E	1	385739.009	0.013	653.1810
66	9	57	65	10	56	E	1	385739.009	0.028	653.1810
66	10	57	65	9	56	E	1	385739.009	0.005	653.1810
66	10	57	65	10	56	E	1	385739.009	0.020	653.1810
69	6	63	68	6	62	A	1	385798.388	-0.150	664.9343
69	6	63	68	7	62	A	1	385798.388	-0.150	664.9343
69	7	63	68	6	62	A	1	385798.388	-0.150	664.9343
69	7	63	68	7	62	A	1	385798.388	-0.150	664.9343
68	7	61	67	7	60	E	1	385800.636	-0.027	661.4275
68	7	61	67	8	60	E	1	385800.636	-0.027	661.4275
68	8	61	67	7	60	E	1	385800.636	-0.027	661.4275
68	8	61	67	8	60	E	1	385800.636	-0.027	661.4275
67	8	59	66	8	58	E	0	385828.276	0.013	529.2803
67	9	59	66	9	58	E	0	385828.276	0.013	529.2803
67	8	59	66	8	58	A	0	385832.640	0.160	529.2789
67	8	59	66	9	58	A	0	385832.640	0.160	529.2789
67	9	59	66	8	58	A	0	385832.640	0.159	529.2789
67	9	59	66	9	58	A	0	385832.640	0.160	529.2789
66	9	57	65	9	56	A	0	385837.332	-0.009	524.9191
66	9	57	65	10	56	A	0	385837.332	0.004	524.9191
66	10	57	65	9	56	A	0	385837.332	-0.016	524.9191
66	10	57	65	10	56	A	0	385837.332	-0.003	524.9191
65	10	55	64	10	54	E	1	385853.273	-0.050	648.3174
65	10	55	64	11	54	E	1	385853.273	0.361	648.3174
65	11	55	64	10	54	E	1	385853.273	-0.274	648.3174
65	11	55	64	11	54	E	1	385853.273	0.137	648.3174
68	7	61	67	7	60	E	0	385894.572	0.021	533.1646
68	8	61	67	8	60	E	0	385894.572	0.021	533.1646
68	7	61	67	7	60	A	0	385898.488	0.020	533.1627
68	7	61	67	8	60	A	0	385898.488	0.020	533.1627
68	8	61	67	7	60	A	0	385898.488	0.020	533.1627
68	8	61	67	8	60	A	0	385898.488	0.020	533.1627
69	6	63	68	6	62	E	1	385915.305	-0.015	664.8535
69	6	63	68	7	62	E	1	385915.305	-0.015	664.8535
69	7	63	68	6	62	E	1	385915.305	-0.015	664.8535
69	7	63	68	7	62	E	1	385915.305	-0.015	664.8535
65	10	55	64	10	54	E	0	385945.151	-0.085	520.0597
65	11	55	64	11	54	E	0	385945.151	0.085	520.0597
65	10	55	64	10	54	A	0	385950.104	-0.086	520.0588
65	10	55	64	11	54	A	0	385950.104	0.289	520.0588
65	11	55	64	10	54	A	0	385950.104	-0.289	520.0588
65	11	55	64	11	54	A	0	385950.104	0.086	520.0588
70	5	65	69	5	64	A	1	385955.967	-0.168	667.9421
70	5	65	69	6	64	A	1	385955.967	-0.168	667.9421
70	6	65	69	5	64	A	1	385955.967	-0.168	667.9421
70	6	65	69	6	64	A	1	385955.967	-0.168	667.9421
64	11	53	63	12	52	A	1	385958.699	-0.029	642.9445
64	12	53	63	12	52	A	1	385963.131	0.232	642.9445
64	11	53	63	11	52	A	1	385966.141	-0.023	642.9442
64	12	53	63	11	52	A	1	385970.616	0.282	642.9442
69	6	63	68	7	62	E	0	386008.447	-0.039	536.5916
69	7	63	68	6	62	E	0	386008.447	-0.039	536.5916
69	6	63	68	6	62	A	0	386012.171	0.072	536.5890
69	6	63	68	7	62	A	0	386012.171	0.072	536.5890
69	7	63	68	6	62	A	0	386012.171	0.072	536.5890
69	7	63	68	7	62	A	0	386012.171	0.072	536.5890
70	5	65	69	5	64	E	1	386062.450	0.019	667.8348
70	5	65	69	6	64	E	1	386062.450	0.019	667.8348
70	6	65	69	5	64	E	1	386062.450	0.019	667.8348
70	6	65	69	6	64	E	1	386062.450	0.019	667.8348
64	11	53	63	11	52	E	1	386143.455	0.054	642.9169
70	5	65	69	5	64	E	0	386154.841	0.008	539.5752
70	6	65	69	6	64	E	0	386154.841	0.008	539.5752
70	5	65	69	5	64	A	0	386158.151	0.039	539.5718
70	5	65	69	6	64	A	0	386158.151	0.039	539.5718
70	6	65	69	5	64	A	0	386158.151	0.039	539.5718
70	6	65	69	6	64	A	0	386158.151	0.039	539.5718
62	14	48	61	15	47	E	0	386176.316	0.077	507.6979
64	12	53	63	11	52	A	0	386240.790	-0.069	514.6630
63	12	51	62	13	50	A	1	386420.582	0.218	636.9616
62	13	49	61	14	48	E	0	386498.121	0.022	502.0499
62	13	49	61	14	48	A	0	386499.767	0.106	502.0485
61	20	42	60	20	41	A	0	386690.860	-0.095	523.6707
63	12	51	62	13	50	E	0	386694.450	0.258	508.6829
61	20	42	60	20	41	E	0	386712.466	0.042	523.6615
61	20	41	60	20	40	A	0	386724.393	-0.059	523.6720
63	13	51	62	13	50	E	0	386776.850	-0.213	508.6829
63	13	51	62	13	50	A	0	386783.375	0.062	508.6819
63	12	51	62	12	50	E	0	386835.196	-0.075	508.6782
63	12	51	62	12	50	A	0	386841.827	0.013	508.6771
63	13	51	62	12	50	E	0	386918.025	-0.117	508.6782
63	13	51	62	12	50	A	0	386925.181	0.045	508.6771
23	22	1	22	21	1	E	0	387119.909	-0.004	203.2354
23	22	2	22	21	2	E	0	387157.336	0.037	203.2263
23	22	1	22	21	2	A	0	387163.862	-0.064	203.2398
23	22	2	22	21	1	A	0	387163.862	-0.064	203.2398
62	26	36	61	26	35	A	0	387244.197	-0.259	586.3895
62	26	37	61	26	36	A	0	387244.197	-0.259	586.3895
62	26	37	61	26	36	E	0	387246.764	-0.096	586.3777
62	26	36	61	26	35	E	0	387248.373	0.045	586.3805
62	14	49	61	14	48	A	1	387329.223	0.181	630.3307
70	35	36	70	34	37	A	0	387677.163	-0.081	799.1829
70	35	35	70	34	36	A	0	387677.163	-0.081	799.1829
62	25	38	61	25	37	A	0	387922.236	-0.082	576.9832
62	25	37	61	25	36	A	0	387922.236	-0.082	576.9832
62	25	38	61	25	37	E	0	387925.239	-0.104	576.9712
62	25	37	61	25	36	E	0	387926.684	0.145	576.9755
25	21	4	24	20	4	E	0	387981.279	-0.039	205.0766
25	21	5	24	20	5	E	0	388015.059	0.037	205.0664
25	21	5	24	20	4	A	0	388027.031	-0.060	205.0794
25	21	4	24	20	5	A	0	388027.031	-0.060	205.0794
69	35	35	69	34	36	A	0	388056.337	0.100	784.6808
69	35	34	69	34	35	A	0	388056.337	0.100	784.6808
69	35	34	69	34	35	E	0	388099.104	-0.182	784.6767
62	13	49	61	13	48	E	0	388307.312	-0.141	501.9896
62	13	49	61	13	48	A	0	388316.973	0.000	501.9879

J'	K'_a	K'_c	J''	K''_a	K''_c	Sym.	$v_{t_{CH_3}}$	$\nu_{Obs.}$	$\nu_{Obs.} - \nu_{Calc.}$	E_{low}
68	35	34	68	34	35	A	0	388418.526	-0.106	770.3915
68	35	33	68	34	34	A	0	388418.526	-0.106	770.3915
68	35	34	68	34	35	E	0	388427.765	-0.169	770.3792
68	35	33	68	34	34	E	0	388461.822	-0.105	770.3875
61	19	43	60	19	42	A	0	388508.685	-0.228	516.9796
61	19	43	60	19	42	E	0	388539.046	-0.041	516.9743
62	24	38	61	24	37	A	0	388702.775	-0.068	568.0019
62	24	39	61	24	38	A	0	388702.775	-0.064	568.0019
61	19	42	60	19	41	E	0	388749.109	-0.003	516.9896
61	19	42	60	19	41	A	0	388762.607	-0.056	516.9910
67	35	32	67	34	33	E	0	388808.734	0.197	756.3109
27	20	7	26	19	7	E	0	388824.214	-0.083	208.1426
27	20	8	26	19	8	E	0	388853.856	0.043	208.1314
27	20	7	26	19	8	A	0	388871.140	-0.059	208.1439
27	20	8	26	19	7	A	0	388871.140	-0.059	208.1439
59	16	43	58	16	42	A	0	389083.093	-0.025	475.2926
66	35	32	66	34	33	A	0	389095.847	-0.081	742.4507
66	35	31	66	34	32	A	0	389095.847	-0.081	742.4507
59	16	43	58	16	42	E	0	389101.552	-0.107	475.2956
66	35	32	66	34	33	E	0	389105.706	-0.157	742.4382
66	35	31	66	34	32	E	0	389139.749	0.073	742.4467
60	14	46	59	14	45	E	0	389155.087	-0.049	481.2591
60	14	46	59	14	45	A	0	389173.962	0.033	481.2552
41	13	28	40	12	29	E	0	389226.855	0.029	265.8171
41	13	28	40	12	29	A	0	389238.339	0.046	265.8133
65	35	31	65	34	32	A	0	389411.946	0.016	728.7985
65	35	30	65	34	31	A	0	389411.946	0.016	728.7985
65	35	31	65	34	32	E	0	389422.002	-0.159	728.7860
62	14	49	61	13	48	E	0	389431.881	-0.031	501.9896
39	14	26	38	13	25	A	0	389436.626	-0.080	253.3575
62	14	49	61	13	48	A	0	389446.525	-0.058	501.9879
39	14	25	38	13	25	E	0	389512.899	-0.103	253.3629
39	14	26	38	13	26	E	0	389583.094	-0.001	253.3476
62	23	40	61	23	39	A	0	389612.466	-0.003	559.4549
62	23	39	61	23	38	E	0	389617.287	-0.164	559.4500
29	19	10	28	18	10	E	0	389625.129	-0.041	212.4440
29	19	11	28	18	11	E	0	389650.031	0.017	212.4319
29	19	11	28	18	10	A	0	389672.488	-0.058	212.4436
29	19	10	28	18	11	A	0	389672.488	-0.058	212.4436
64	35	30	64	34	31	A	0	389713.654	0.116	715.3583
64	35	29	64	34	30	A	0	389713.654	0.116	715.3583
39	14	25	38	13	26	A	0	389746.522	-0.033	253.3488
64	35	29	64	34	30	E	0	389757.578	-0.112	715.3543
60	17	43	59	17	42	A	0	389834.958	-0.052	492.6345
60	17	43	59	17	42	E	0	389852.265	0.022	492.6351
63	35	29	63	34	30	A	0	390001.175	-0.084	702.1296
63	35	28	63	34	29	A	0	390001.175	-0.084	702.1296
62	35	28	62	34	29	A	0	390275.789	0.204	689.1123
62	35	27	62	34	28	A	0	390275.789	0.204	689.1123
62	35	28	62	34	29	E	0	390286.598	-0.028	689.0996
62	35	27	62	34	28	E	0	390320.117	0.020	689.1084
31	18	13	30	17	13	E	0	390342.784	-0.060	217.9956
31	18	14	30	17	14	E	0	390362.556	0.014	217.9829
31	18	13	30	17	14	A	0	390389.967	-0.056	217.9935
31	18	14	30	17	13	A	0	390389.967	-0.056	217.9935
61	35	27	61	34	28	A	0	390536.994	0.002	676.3062
61	35	26	61	34	27	A	0	390536.994	0.002	676.3062
61	18	44	60	18	43	A	0	390540.141	0.027	510.8262
61	18	44	60	18	43	E	0	390552.311	-0.042	510.8241
61	35	26	61	34	27	E	0	390581.761	0.094	676.3023
67	9	58	66	9	57	A	1	390690.404	0.061	666.0769
67	9	58	66	10	57	A	1	390690.404	0.067	666.0769
67	10	58	66	9	57	A	1	390690.404	0.058	666.0769
67	10	58	66	10	57	A	1	390690.404	0.064	666.0769
62	22	40	61	22	39	E	0	390694.327	-0.073	551.3514
68	8	60	67	8	59	A	1	390705.510	-0.065	670.4495
68	8	60	67	9	59	A	1	390705.510	-0.065	670.4495
68	9	60	67	8	59	A	1	390705.510	-0.065	670.4495
68	9	60	67	9	59	A	1	390705.510	-0.065	670.4495
66	10	56	65	10	55	A	1	390775.119	0.136	661.2108
66	10	56	65	11	55	A	1	390775.119	0.315	661.2108
66	11	56	65	10	55	A	1	390775.119	0.039	661.2108
66	11	56	65	11	55	A	1	390775.119	0.218	661.2108
60	35	26	60	34	27	A	0	390785.756	-0.186	663.7110
60	35	25	60	34	26	A	0	390785.756	-0.186	663.7110
69	7	62	68	7	61	A	1	390787.403	-0.078	674.3523
69	7	62	68	8	61	A	1	390787.403	-0.078	674.3523
69	8	62	68	7	61	A	1	390787.403	-0.078	674.3523
69	8	62	68	8	61	A	1	390787.403	-0.078	674.3523
60	35	25	60	34	26	E	0	390830.772	0.001	663.7071
67	9	58	66	9	57	E	1	390833.738	0.004	666.0479
67	9	58	66	10	57	E	1	390833.738	0.012	666.0479
67	10	58	66	9	57	E	1	390833.738	-0.000	666.0479
67	10	58	66	10	57	E	1	390833.738	0.008	666.0479
68	8	60	67	8	59	E	1	390839.222	-0.003	670.4095
68	8	60	67	9	59	E	1	390839.222	-0.003	670.4095
68	9	60	67	8	59	E	1	390839.222	-0.003	670.4095
68	9	60	67	9	59	E	1	390839.222	-0.003	670.4095
33	17	16	32	16	16	E	0	390905.250	-0.069	224.8192
69	7	62	68	7	61	E	1	390912.297	0.090	674.2964
69	7	62	68	8	61	E	1	390912.297	0.090	674.2964
69	8	62	68	7	61	E	1	390912.297	0.090	674.2964
69	8	62	68	8	61	E	1	390912.297	0.090	674.2964
70	6	64	69	6	63	A	1	390914.969	-0.171	677.8032
70	6	64	69	7	63	A	1	390914.969	-0.171	677.8032
70	7	64	69	6	63	A	1	390914.969	-0.171	677.8032
70	7	64	69	7	63	A	1	390914.969	-0.171	677.8032
67	9	58	66	9	57	E	0	390930.404	-0.031	537.7902
67	10	58	66	10	57	E	0	390930.404	-0.027	537.7902
67	9	58	66	9	57	A	0	390934.724	-0.172	537.7893
67	9	58	66	10	57	A	0	390934.724	-0.165	537.7893
67	10	58	66	9	57	A	0	390934.724	-0.176	537.7893
67	10	58	66	10	57	A	0	390934.724	-0.169	537.7893
68	8	60	67	8	59	E	0	390936.205	0.137	542.1502
68	9	60	67	9	59	E	0	390936.205	0.137	542.1502
68	8	60	67	8	59	A	0	390940.200	-0.020	542.1489
68	8	60	67	9	59	A	0	390940.200	-0.020	542.1489
68	9	60	67	8	59	A	0	390940.200	-0.020	542.1489
68	9	60	67	9	59	A	0	390940.200	-0.020	542.1489
33	17	17	32	16	16	A	0	390951.569	-0.042	224.8155
33	17	16	32	16	17	A	0	390951.569	-0.042	224.8155
37	15	23	36	14	22	A	0	390958.889	-0.166	242.4250
37	15	22	36	14	23	A	0	390965.465	0.013	242.4248
69	7	62	68	7	61	E	0	391008.475	0.014	546.0367
69	8	62	68	8	61	E	0	391008.475	0.014	546.0367
69	7	62	68	7	61	A	0	391012.332	0.004	546.0349

J'	K'_a	K'_c	J''	K''_a	K''_c	Sym.	$v_{t_{CH_3}}$	$\nu_{Obs.}$	$\nu_{Obs.} - \nu_{Calc.}$	E_{low}
69	7	62	68	8	61	A	0	391012.332	0.004	546.0349
69	8	62	68	7	61	A	0	391012.332	0.004	546.0349
69	8	62	68	8	61	A	0	391012.332	0.004	546.0349
65	12	54	64	11	53	A	1	391018.705	0.091	655.8186
66	10	56	65	10	55	E	0	391025.469	-0.082	532.9335
66	11	56	65	11	55	E	0	391025.469	0.011	532.9335
59	35	25	59	34	26	E	0	391034.571	-0.054	651.3137
59	35	24	59	34	25	E	0	391067.920	0.064	651.3226
70	6	64	69	6	63	E	0	391126.130	0.041	549.4674
70	7	64	69	7	63	E	0	391126.130	0.041	549.4674
70	6	64	69	6	63	A	0	391129.697	0.030	549.4650
70	6	64	69	7	63	A	0	391129.697	0.030	549.4650
70	7	64	69	6	63	A	0	391129.697	0.030	549.4650
70	7	64	69	7	63	A	0	391129.697	0.030	549.4650
35	16	19	34	15	19	E	0	391181.980	-0.148	232.9474
35	16	20	34	15	20	E	0	391189.826	-0.068	232.9341
35	16	19	34	15	20	A	0	391226.836	-0.024	232.9421
58	35	24	58	34	25	A	0	391248.117	-0.133	639.1525
58	35	23	58	34	24	A	0	391248.117	-0.133	639.1525
65	11	54	64	12	53	A	0	391279.956	-0.257	527.5466
57	35	23	57	34	24	A	0	391462.444	-0.020	627.1887
57	35	22	57	34	23	A	0	391462.444	-0.020	627.1887
57	35	23	57	34	24	E	0	391474.551	-0.068	627.1758
64	13	52	63	13	51	A	1	391500.449	0.342	649.8537
57	35	22	57	34	23	E	0	391507.580	-0.120	627.1849
64	12	52	63	13	51	E	1	391642.178	0.167	649.8281
56	35	22	56	34	23	E	0	391678.126	-0.157	615.4221
64	13	52	63	13	51	E	1	391694.384	-0.096	649.8281
56	35	21	56	34	22	E	0	391711.231	-0.061	615.4312
64	12	52	63	13	51	E	0	391730.579	-0.023	521.5844
64	12	52	63	13	51	A	0	391736.310	-0.074	521.5836
55	35	21	55	34	22	A	0	391859.017	-0.054	603.8913
55	35	20	55	34	21	A	0	391859.017	-0.054	603.8913
64	13	52	63	12	51	A	0	391868.142	-0.124	521.5808
63	13	50	62	14	49	E	0	391909.922	-0.036	514.9796
63	13	50	62	14	49	A	0	391913.302	-0.059	514.9785
61	17	45	60	17	44	A	0	392009.596	0.101	505.1990
54	35	20	54	34	21	E	0	392054.775	-0.169	592.5441
54	35	19	54	34	20	E	0	392088.166	0.353	592.5533
53	35	19	53	34	20	A	0	392215.936	0.088	581.4324
53	35	18	53	34	19	A	0	392215.936	0.088	581.4324
53	35	19	53	34	20	E	0	392228.608	-0.096	581.4194
53	35	18	53	34	19	E	0	392261.736	0.229	581.4286
52	35	18	52	34	19	E	0	392393.282	0.037	570.5040
52	35	17	52	34	18	E	0	392426.000	0.017	570.5132
51	35	17	51	34	18	A	0	392535.623	-0.149	559.8107
51	35	16	51	34	17	A	0	392535.623	-0.149	559.8107
51	35	17	51	34	18	E	0	392548.961	0.038	559.7976
63	28	35	62	28	34	A	0	392568.265	-0.069	619.3290
63	28	36	62	28	35	A	0	392568.265	-0.069	619.3290
63	28	35	62	28	34	E	0	392569.821	-0.003	619.3183
51	35	16	51	34	17	E	0	392581.588	-0.010	559.8069
63	14	50	62	14	49	A	0	392607.015	-0.084	514.9785
50	35	16	50	34	17	A	0	392682.822	0.024	549.3133
50	35	15	50	34	16	A	0	392682.822	0.024	549.3133
50	35	16	50	34	17	E	0	392696.111	0.025	549.3002
50	35	15	50	34	16	E	0	392728.668	-0.031	549.3095
64	15	49	63	16	48	E	0	392804.829	-0.001	539.7028
49	35	15	49	34	16	A	0	392821.672	0.016	539.0246
49	35	14	49	34	15	A	0	392821.672	0.016	539.0246
49	35	15	49	34	16	E	0	392835.035	-0.036	539.0115
49	35	14	49	34	15	E	0	392867.599	-0.025	539.0208
48	35	14	48	34	15	A	0	392952.541	-0.132	528.9445
48	35	13	48	34	14	A	0	392952.541	-0.132	528.9445
48	35	14	48	34	15	E	0	392966.156	-0.051	528.9313
48	35	13	48	34	14	E	0	392998.773	0.070	528.9407
63	13	50	62	13	49	E	0	393034.362	-0.055	514.9421
63	13	50	62	13	49	A	0	393042.705	-0.265	514.9408
47	35	13	47	34	14	A	0	393076.123	-0.046	519.0728
47	35	12	47	34	13	A	0	393076.123	-0.046	519.0728
46	35	12	46	34	13	A	0	393192.430	-0.024	509.4092
46	35	11	46	34	12	A	0	393192.430	-0.024	509.4092
46	35	12	46	34	13	E	0	393206.093	-0.112	509.3961
62	15	48	61	15	47	E	0	393221.934	-0.005	507.6979
62	15	48	61	15	47	A	0	393227.105	-0.029	507.6965
46	35	11	46	34	12	E	0	393238.638	0.046	509.4055
24	22	2	23	21	2	E	0	393263.577	-0.109	207.9560
24	22	3	23	21	3	E	0	393301.159	0.109	207.9469
24	22	2	23	21	3	A	0	393307.673	-0.027	207.9603
24	22	3	23	21	2	A	0	393307.673	-0.027	207.9603
44	35	10	44	34	11	E	0	393418.429	-0.114	490.6930
44	35	9	44	34	10	E	0	393450.944	0.117	490.7025
62	20	43	61	20	42	E	0	393605.746	-0.157	536.5608
62	20	42	61	20	41	E	0	393623.938	0.066	536.5708
62	20	42	61	20	41	A	0	393637.442	0.185	536.5717
41	35	7	41	34	8	A	0	393675.973	-0.096	464.2093
41	35	6	41	34	7	A	0	393675.973	-0.096	464.2093
41	35	7	41	34	8	E	0	393690.225	-0.023	464.1961
41	35	6	41	34	7	E	0	393722.359	-0.031	464.2056
63	14	50	62	13	49	E	0	393724.863	-0.007	514.9421
63	14	50	62	13	49	A	0	393736.678	-0.030	514.9408
63	26	37	62	26	36	A	0	393750.496	-0.067	599.3066
63	26	38	62	26	37	A	0	393750.496	-0.067	599.3066
63	26	38	62	26	37	E	0	393753.178	0.060	599.2949
63	14	49	62	15	48	E	0	393768.072	-0.095	520.8144
26	21	5	25	20	5	E	0	394120.918	-0.047	210.2140
26	21	6	25	20	6	E	0	394154.664	0.030	210.2038
26	21	6	25	20	5	A	0	394166.687	-0.049	210.2168
26	21	5	25	20	6	A	0	394166.687	-0.049	210.2168
40	14	27	39	13	26	E	0	394423.757	-0.032	261.6075
63	25	39	62	25	38	A	0	394467.445	0.019	589.9229
63	25	38	62	25	37	A	0	394467.445	0.018	589.9229
63	25	39	62	25	38	E	0	394470.321	-0.316	589.9110
63	25	38	62	25	37	E	0	394471.843	-0.022	589.9153
40	14	27	39	13	26	A	0	394679.704	-0.028	261.6031
40	14	26	39	13	26	E	0	394817.030	-0.094	261.6075
28	20	8	27	19	8	E	0	394954.295	-0.014	213.6997
28	20	9	27	19	9	E	0	394983.788	0.020	213.6885
28	20	8	27	19	9	A	0	395001.153	-0.049	213.7009
28	20	9	27	19	8	A	0	395001.153	-0.049	213.7009
40	14	27	39	13	27	E	0	395036.708	-0.004	261.5870
40	14	26	39	13	27	A	0	395262.277	-0.035	261.5869
63	24	40	62	24	39	A	0	395294.527	-0.020	580.9676
62	19	44	61	19	43	A	0	395506.880	-0.099	529.9389
62	19	44	61	19	43	E	0	395529.965	-0.029	529.9346
30	19	11	29	18	11	E	0	395736.612	-0.052	218.4245

J'	K'_a	K'_c	J''	K''_a	K''_c	Sym.	$v_{t_{CH_3}}$	$\nu_{Obs.}$	$\nu_{Obs.} - \nu_{Calc.}$	E_{low}
30	19	12	29	18	12	E	0	395761.430	0.011	218.4125
30	19	12	29	18	11	A	0	395783.995	-0.030	218.4241
30	19	11	29	18	12	A	0	395783.995	-0.030	218.4241
68	9	59	67	9	58	A	1	395789.124	-0.003	679.1089
68	9	59	67	10	58	A	1	395789.124	0.000	679.1089
68	10	59	67	9	58	A	1	395789.124	-0.005	679.1089
68	10	59	67	10	58	A	1	395789.124	-0.001	679.1089
69	8	61	68	8	60	A	1	395813.384	-0.054	683.4820
69	8	61	68	9	60	A	1	395813.384	-0.054	683.4820
69	9	61	68	8	60	A	1	395813.384	-0.054	683.4820
69	9	61	68	9	60	A	1	395813.384	-0.054	683.4820
67	10	57	66	10	56	A	1	395858.415	0.049	674.2457
67	10	57	66	11	56	A	1	395858.415	0.145	674.2457
67	11	57	66	10	56	A	1	395858.415	-0.003	674.2457
67	11	57	66	11	56	A	1	395858.415	0.094	674.2457
70	7	63	69	7	62	A	1	395900.665	-0.123	687.3876
70	7	63	69	8	62	A	1	395900.665	-0.123	687.3876
70	8	63	69	7	62	A	1	395900.665	-0.123	687.3876
70	8	63	69	8	62	A	1	395900.665	-0.123	687.3876
62	19	43	61	19	42	E	0	395926.577	0.045	529.9568
68	9	59	67	9	58	E	1	395929.915	0.106	679.0847
68	9	59	67	10	58	E	1	395929.915	0.110	679.0847
68	10	59	67	9	58	E	1	395929.915	0.103	679.0847
68	10	59	67	10	58	E	1	395929.915	0.107	679.0847
62	19	43	61	19	42	A	0	395931.489	-0.149	529.9587
69	8	61	68	8	60	E	1	395945.035	0.012	683.4464
69	8	61	68	9	60	E	1	395945.035	0.012	683.4464
69	9	61	68	8	60	E	1	395945.035	0.012	683.4464
69	9	61	68	9	60	E	1	395945.035	0.012	683.4464
42	13	29	41	12	30	E	0	396011.684	0.051	274.5491
70	7	63	69	7	62	E	1	396023.921	-0.035	687.3358
70	7	63	69	8	62	E	1	396023.921	-0.035	687.3358
70	8	63	69	7	62	E	1	396023.921	-0.035	687.3358
70	8	63	69	8	62	E	1	396023.921	-0.035	687.3358
68	9	59	67	9	58	E	0	396029.360	-0.036	550.8303
68	10	59	67	10	58	E	0	396029.360	-0.035	550.8303
68	9	59	67	9	58	A	0	396033.748	-0.026	550.8295
68	9	59	67	10	58	A	0	396033.748	-0.022	550.8295
68	10	59	67	9	58	A	0	396033.748	-0.028	550.8295
68	10	59	67	10	58	A	0	396033.748	-0.024	550.8295
69	8	61	68	8	60	E	0	396044.459	-0.006	555.1904
69	9	61	68	9	60	E	0	396044.459	-0.006	555.1904
69	8	61	68	8	60	A	0	396048.567	0.014	555.1892
69	8	61	68	9	60	A	0	396048.567	0.014	555.1892
69	9	61	68	8	60	A	0	396048.567	0.014	555.1892
69	9	61	68	9	60	A	0	396048.567	0.014	555.1892
66	11	55	65	12	54	A	1	396070.019	0.098	668.8616
66	12	55	65	12	54	A	1	396071.379	0.169	668.8616
66	11	55	65	11	54	A	1	396072.312	0.066	668.8615
66	12	55	65	11	54	A	1	396073.811	0.275	668.8615
67	10	57	66	10	56	E	0	396108.581	-0.033	545.9767
67	11	57	66	11	56	E	0	396108.581	0.018	545.9767
67	10	57	66	10	56	A	0	396113.302	-0.039	545.9761
67	10	57	66	11	56	A	0	396113.302	0.071	545.9761
67	11	57	66	10	56	A	0	396113.302	-0.099	545.9761
67	11	57	66	11	56	A	0	396113.302	0.012	545.9761
70	7	63	69	7	62	E	0	396122.551	-0.023	559.0793
70	8	63	69	8	62	E	0	396122.551	-0.023	559.0793
70	7	63	69	7	62	A	0	396126.484	0.091	559.0776
70	7	63	69	8	62	A	0	396126.484	0.091	559.0776
70	8	63	69	7	62	A	0	396126.484	0.091	559.0776
70	8	63	69	8	62	A	0	396126.484	0.091	559.0776
63	23	40	62	23	39	A	0	396261.196	-0.024	572.4510
63	23	41	62	23	40	E	0	396266.187	0.158	572.4393
66	11	55	65	11	54	E	0	396334.533	0.182	540.5988
66	11	55	65	12	54	A	0	396337.054	0.104	540.5984
66	12	55	65	11	54	A	0	396340.991	-0.010	540.5983
32	18	14	31	17	14	E	0	396421.415	-0.041	224.4047
32	18	15	31	17	15	E	0	396441.162	0.146	224.3920
32	18	14	31	17	15	A	0	396468.464	-0.145	224.4026
32	18	15	31	17	14	A	0	396468.464	-0.145	224.4026
65	12	53	64	13	52	A	1	396486.759	0.270	662.9128
65	13	53	64	13	52	A	1	396512.238	0.292	662.9128
65	12	53	64	12	52	A	1	396531.027	0.390	662.9113
65	13	53	64	12	52	A	1	396556.404	0.309	662.9113
38	15	24	37	14	24	E	0	396665.817	-0.035	250.1713
65	12	53	64	13	52	E	1	396669.553	0.087	662.8936
38	15	24	37	14	23	A	0	396701.258	-0.035	250.1776
38	15	23	37	14	24	A	0	396714.940	-0.024	250.1772
65	12	53	64	13	52	E	0	396760.952	0.044	534.6527
65	12	53	64	13	52	A	0	396766.358	-0.246	534.6521
65	12	53	64	12	52	E	0	396809.175	-0.021	534.6511
65	12	53	64	12	52	A	0	396815.052	-0.112	534.6505
65	13	53	64	12	52	A	0	396843.238	-0.012	534.6505
34	17	17	33	16	17	E	0	396927.203	-0.062	231.6640
34	17	18	33	16	18	E	0	396940.900	-0.192	231.6509
34	17	18	33	16	17	A	0	396973.468	-0.049	231.6602
34	17	17	33	16	18	A	0	396973.468	-0.050	231.6602
36	16	20	35	15	20	E	0	397105.641	-0.312	240.2382
36	16	21	35	15	21	E	0	397113.282	-0.084	240.2249
36	16	21	35	15	20	A	0	397150.465	-0.036	240.2328
64	13	51	63	14	50	A	0	397154.454	-0.002	528.0744
64	14	51	63	14	50	A	1	397276.686	0.541	656.3365
63	22	41	62	22	40	E	0	397415.224	0.137	564.3836
62	18	45	61	18	44	A	0	397532.047	-0.041	523.8532
62	18	45	61	18	44	E	0	397542.436	-0.118	523.8516
60	16	44	59	16	43	A	0	397543.969	0.009	488.2710
60	16	44	59	16	43	E	0	397559.790	0.017	488.2746
64	14	51	63	14	50	E	0	397569.122	-0.194	528.0754
64	14	51	63	14	50	A	0	397575.882	-0.016	528.0744
64	13	51	63	13	50	E	0	397840.398	0.004	528.0523
64	13	51	63	13	50	A	0	397848.140	-0.053	528.0513
64	14	51	63	13	50	E	0	398259.712	-0.057	528.0523
64	14	51	63	13	50	A	0	398269.608	-0.028	528.0513
63	15	49	62	15	48	E	0	398368.564	-0.011	520.8144
63	15	49	62	15	48	A	0	398374.429	0.055	520.8131
60	15	45	59	15	44	E	0	398379.900	-0.033	484.8834
60	15	45	59	15	44	A	0	398386.538	0.017	484.8783
61	17	44	60	17	43	E	0	398450.854	-0.043	505.6392
23	23	0	22	22	0	E	0	398542.451	-0.012	211.4296
23	23	1	22	22	1	E	0	398582.679	-0.290	211.4218
23	23	1	22	22	0	A	0	398584.172	0.048	211.4354
23	23	0	22	22	1	A	0	398584.172	0.048	211.4354
62	17	46	61	17	45	E	0	398642.660	-0.046	518.2751
63	21	43	62	21	42	A	0	398797.869	-0.108	556.7935
64	28	36	63	28	35	A	0	399024.101	-0.112	632.4237

J'	K'_a	K'_c	J''	K''_a	K''_c	Sym.	$v_{t_{CH_3}}$	$\nu_{Obs.}$	$\nu_{Obs.} - \nu_{Calc.}$	E_{low}
64	28	37	63	28	36	A	0	399024.101	-0.112	632.4237
64	28	37	63	28	36	E	0	399027.760	-0.004	632.4129
25	22	3	24	21	3	E	0	399406.339	-0.065	212.8835
25	22	4	24	21	4	E	0	399443.769	0.026	212.8744
25	22	3	24	21	4	A	0	399450.357	-0.062	212.8879
25	22	4	24	21	3	A	0	399450.357	-0.062	212.8879
64	27	38	63	27	37	A	0	399608.710	0.002	622.2239
64	27	37	63	27	36	A	0	399608.710	0.002	622.2239
64	27	38	63	27	37	E	0	399610.747	-0.073	622.2126
64	27	37	63	27	36	E	0	399612.489	-0.130	622.2140
70	36	34	70	35	35	A	0	399632.383	-0.048	812.1144
70	36	35	70	35	36	A	0	399632.383	-0.048	812.1144
70	36	35	70	35	36	E	0	399646.250	-0.335	812.1025
70	36	34	70	35	35	E	0	399676.662	-0.129	812.1118
41	14	28	40	13	27	A	0	399695.971	-0.044	270.0814
62	18	44	61	18	43	A	0	399895.776	-0.060	523.9846
62	18	44	61	18	43	E	0	399908.507	0.008	523.9838
41	14	27	40	13	27	E	0	399915.992	0.053	270.0851
69	36	33	69	35	34	A	0	399979.980	-0.051	797.6249
69	36	34	69	35	35	A	0	399979.980	-0.051	797.6249
69	36	33	69	35	34	E	0	400024.692	0.083	797.6223
27	21	6	26	20	6	E	0	400258.165	-0.035	215.5592
64	26	38	63	26	37	A	0	400271.306	-0.034	612.4407
64	26	39	63	26	38	A	0	400271.306	-0.034	612.4407
64	26	39	63	26	38	E	0	400274.068	0.015	612.4290
64	26	38	63	26	37	E	0	400275.597	-0.025	612.4320
27	21	7	26	20	7	E	0	400291.853	0.026	215.5490
27	21	7	26	20	6	A	0	400303.914	-0.053	215.5620
27	21	6	26	20	7	A	0	400303.914	-0.053	215.5620
68	36	33	68	35	34	E	0	400327.121	-0.267	783.3358
68	36	32	68	35	33	E	0	400357.339	-0.024	783.3452
41	14	28	40	13	28	E	0	400453.749	-0.123	270.0532
64	14	50	63	15	49	A	0	400477.380	-0.110	534.1014
64	14	50	63	15	49	E	0	400483.679	-0.037	534.1025
63	20	44	62	20	43	A	0	400503.622	-0.024	549.6976
63	20	44	62	20	43	E	0	400536.443	0.022	549.6901
63	20	43	62	20	42	E	0	400590.859	0.214	549.7007
63	20	43	62	20	42	A	0	400607.642	0.006	549.7021
67	36	31	67	35	32	A	0	400630.558	-0.031	769.2827
67	36	32	67	35	33	A	0	400630.558	-0.031	769.2827
67	36	32	67	35	33	E	0	400645.416	-0.286	769.2706
67	36	31	67	35	32	E	0	400675.598	0.034	769.2802
41	14	27	40	13	28	A	0	400763.224	-0.075	270.0520
63	14	49	62	14	48	E	0	400813.658	-0.209	520.5794
63	14	49	62	14	48	A	0	400827.931	-0.056	520.5772
41	14	27	40	13	28	E	0	400873.853	-0.161	270.0532
69	9	60	68	9	59	A	1	400889.095	0.054	692.3110
69	9	60	68	10	59	A	1	400889.095	0.056	692.3110
69	10	60	68	9	59	A	1	400889.095	0.053	692.3110
69	10	60	68	10	59	A	1	400889.095	0.055	692.3110
69	17	52	68	18	51	E	0	400898.762	0.095	621.4161
70	8	62	69	8	61	A	1	400921.746	-0.046	696.6849
70	8	62	69	9	61	A	1	400921.746	-0.046	696.6849
70	9	62	69	8	61	A	1	400921.746	-0.046	696.6849
70	9	62	69	9	61	A	1	400921.746	-0.046	696.6849
66	36	30	66	35	31	A	0	400934.523	-0.030	755.4295
66	36	31	66	35	32	A	0	400934.523	-0.030	755.4295
68	10	58	67	10	57	A	1	400944.204	0.090	687.4501
68	10	58	67	11	57	A	1	400944.204	0.142	687.4501
68	11	58	67	10	57	A	1	400944.204	0.062	687.4501
68	11	58	67	11	57	A	1	400944.204	0.114	687.4501
66	36	31	66	35	32	E	0	400949.776	-0.182	755.4174
66	36	30	66	35	31	E	0	400979.731	0.020	755.4270
69	9	60	68	9	59	E	1	401027.152	0.074	692.2915
69	9	60	68	10	59	E	1	401027.152	0.076	692.2915
69	10	60	68	9	59	E	1	401027.152	0.073	692.2915
69	10	60	68	10	59	E	1	401027.152	0.075	692.2915
70	8	62	69	8	61	E	1	401051.330	-0.020	696.6538
70	8	62	69	9	61	E	1	401051.330	-0.019	696.6538
70	9	62	69	8	61	E	1	401051.330	-0.020	696.6538
70	9	62	69	9	61	E	1	401051.330	-0.019	696.6538
29	20	9	28	19	9	E	0	401079.830	-0.047	219.4656
68	10	58	67	10	57	E	1	401092.397	0.033	687.4376
68	10	58	67	11	57	E	1	401092.397	0.098	687.4376
68	11	58	67	10	57	E	1	401092.397	-0.002	687.4376
68	11	58	67	11	57	E	1	401092.397	0.063	687.4376
29	20	10	28	19	10	E	0	401109.311	0.041	219.4543
29	20	9	28	19	10	A	0	401126.706	-0.056	219.4667
29	20	10	28	19	9	A	0	401126.706	-0.056	219.4667
69	9	60	68	9	59	E	0	401129.525	-0.011	564.0404
69	10	60	68	10	59	E	0	401129.525	-0.010	564.0404
69	9	60	68	9	59	A	0	401133.879	0.046	564.0397
69	9	60	68	10	59	A	0	401133.879	0.048	564.0397
69	10	60	68	9	59	A	0	401133.879	0.045	564.0397
69	10	60	68	10	59	A	0	401133.879	0.047	564.0397
70	8	62	69	8	61	E	0	401153.395	0.010	568.4010
70	9	62	69	9	61	E	0	401153.395	0.010	568.4010
70	8	62	69	8	61	A	0	401157.429	0.019	568.4000
70	8	62	69	9	61	A	0	401157.429	0.019	568.4000
70	9	62	69	8	61	A	0	401157.429	0.019	568.4000
70	9	62	69	9	61	A	0	401157.429	0.019	568.4000
68	10	58	67	10	57	E	0	401194.090	-0.031	559.1894
68	11	58	67	11	57	E	0	401194.090	-0.004	559.1894
68	10	58	67	10	57	A	0	401198.713	-0.028	559.1890
68	10	58	67	11	57	A	0	401198.713	0.031	559.1890
68	11	58	67	10	57	A	0	401198.713	-0.060	559.1890
68	11	58	67	11	57	A	0	401198.713	-0.001	559.1890
65	36	29	65	35	30	A	0	401224.884	-0.068	741.7879
65	36	30	65	35	31	A	0	401224.884	-0.068	741.7879
67	12	56	66	11	55	A	0	401399.972	-0.272	553.8187
64	36	28	64	35	29	A	0	401502.217	-0.036	728.3577
64	36	29	64	35	30	A	0	401502.217	-0.036	728.3577
66	13	54	65	13	53	A	1	401532.110	0.348	676.1390
66	12	54	65	12	53	A	1	401543.183	0.538	676.1381
64	36	28	64	35	29	E	0	401547.683	-0.061	728.3552
66	12	54	65	12	53	E	1	401726.410	0.085	676.1251
63	36	27	63	35	28	A	0	401766.831	-0.075	715.1386
63	36	28	63	35	29	A	0	401766.831	-0.075	715.1386
63	36	28	63	35	29	E	0	401782.894	-0.216	715.1264
66	12	54	65	13	53	A	0	401796.329	-0.058	547.8877
31	19	12	30	18	12	E	0	401840.602	-0.081	224.6151
31	19	13	30	18	13	E	0	401865.289	-0.049	224.6030
31	19	13	30	18	12	A	0	401887.984	-0.044	224.6146
31	19	12	30	18	13	A	0	401887.984	-0.044	224.6146
62	36	26	62	35	27	A	0	402019.181	-0.172	702.1305
62	36	27	62	35	28	A	0	402019.181	-0.172	702.1305

J'	K_a'	K_c'	J''	K_a''	K_c''	Sym.	$v_{t_{CH_3}}$	$\nu_{Obs.}$	$\nu_{Obs.} - \nu_{Calc.}$	E_{low}
62	36	27	62	35	28	E	0	402035.821	0.023	702.1182
62	36	26	62	35	27	E	0	402065.573	0.434	702.1281
65	14	52	64	14	51	A	1	402246.091	0.590	669.5882
61	36	25	61	35	26	A	0	402260.002	-0.014	689.3331
61	36	26	61	35	27	A	0	402260.002	-0.014	689.3331
65	13	52	64	14	51	E	0	402284.928	-0.034	541.3368
65	13	52	64	14	51	A	0	402290.352	0.251	541.3361
61	36	25	61	35	26	E	0	402305.963	0.026	689.3307
39	15	25	38	14	24	A	0	402383.732	-0.087	258.1520
39	15	24	38	14	25	A	0	402412.199	-0.052	258.1512
33	18	15	32	17	15	E	0	402487.899	-0.092	231.0254
33	18	16	32	17	16	E	0	402507.408	0.011	231.0128
33	18	15	32	17	16	A	0	402535.081	-0.037	231.0233
33	18	16	32	17	15	A	0	402535.081	-0.037	231.0233
65	14	52	64	14	51	A	0	402543.646	0.049	541.3361
63	19	45	62	19	44	E	0	402549.133	-0.016	543.1280
65	13	52	64	13	51	E	0	402704.386	0.048	541.3228
59	36	23	59	35	24	A	0	402707.650	0.011	664.3696
59	36	24	59	35	25	A	0	402707.650	0.011	664.3696
65	13	52	64	13	51	A	0	402711.518	-0.026	541.3221
59	36	23	59	35	24	E	0	402753.900	0.098	664.3672
58	36	22	58	35	23	A	0	402915.270	-0.121	652.2031
58	36	23	58	35	24	A	0	402915.270	-0.121	652.2031
35	17	18	34	16	18	E	0	402929.736	-0.130	238.7226
64	23	42	63	23	41	A	0	402933.017	-0.064	585.6688
58	36	23	58	35	24	E	0	402933.017	0.326	652.1906
64	23	41	63	23	40	E	0	402938.553	-0.119	585.6642
35	17	19	34	16	19	E	0	402943.464	0.012	238.7096
65	14	52	64	13	51	E	0	402956.550	0.002	541.3228
65	14	52	64	13	51	A	0	402965.091	0.051	541.3221
35	17	19	34	16	18	A	0	402976.020	-0.056	238.7188
35	17	18	34	16	19	A	0	402976.020	-0.059	238.7188
37	16	21	36	15	21	E	0	402998.003	-0.064	247.7459
37	16	22	36	15	22	E	0	403005.066	-0.020	247.7327
63	16	48	62	16	47	E	0	403017.270	0.051	526.2596
63	16	48	62	16	47	A	0	403019.054	-0.041	526.2585
37	16	22	36	15	21	A	0	403042.591	0.157	247.7405
57	36	21	57	35	22	A	0	403112.920	-0.028	640.2465
57	36	22	57	35	23	A	0	403112.920	-0.028	640.2465
65	15	50	64	16	49	A	0	403118.122	-0.127	553.3292
57	36	22	57	35	23	E	0	403130.303	-0.132	640.2340
65	15	50	64	16	49	E	0	403152.360	-0.081	553.3302
57	36	21	57	35	22	E	0	403159.288	-0.034	640.2442
63	19	44	62	19	43	A	0	403228.184	0.026	543.1656
63	19	44	62	19	43	E	0	403230.054	-0.083	543.1635
56	36	20	56	35	21	A	0	403300.606	-0.073	628.4996
56	36	21	56	35	22	A	0	403300.606	-0.073	628.4996
56	36	21	56	35	22	E	0	403318.470	0.127	628.4871
56	36	20	56	35	21	E	0	403346.915	-0.233	628.4973
61	15	46	60	15	45	A	0	403386.086	0.014	498.1670
64	15	50	63	15	49	E	0	403436.110	0.060	534.1025
64	15	50	63	15	49	A	0	403442.228	-0.017	534.1014
61	15	47	60	14	46	E	0	403460.064	-0.045	494.2399
55	36	19	55	35	20	A	0	403478.804	-0.139	616.9623
55	36	20	55	35	21	A	0	403478.804	-0.139	616.9623
55	36	19	55	35	20	E	0	403525.453	-0.046	616.9599
54	36	18	54	35	19	A	0	403648.021	-0.071	605.6342
54	36	19	54	35	20	A	0	403648.021	-0.071	605.6342
54	36	19	54	35	20	E	0	403666.265	0.182	605.6216
43	13	30	42	12	31	E	0	403695.152	-0.196	283.5014
43	13	30	42	12	31	A	0	403712.040	-0.074	283.4974
53	36	17	53	35	18	A	0	403808.425	-0.041	594.5153
53	36	18	53	35	19	A	0	403808.425	-0.041	594.5153
53	36	18	53	35	19	E	0	403826.484	-0.122	594.5027
53	36	17	53	35	18	E	0	403855.205	0.028	594.5130
52	36	16	52	35	17	A	0	403960.438	0.041	583.6054
52	36	17	52	35	18	A	0	403960.438	0.041	583.6054
52	36	17	52	35	18	E	0	403978.690	0.011	583.5928
52	36	16	52	35	17	E	0	404007.200	0.024	583.6031
51	36	15	51	35	16	A	0	404104.095	-0.114	572.9043
51	36	16	51	35	17	A	0	404104.095	-0.114	572.9043
51	36	15	51	35	16	E	0	404150.894	-0.156	572.9020
64	22	43	63	22	42	A	0	404157.511	-0.016	577.6428
64	22	42	63	22	41	A	0	404160.030	-0.044	577.6429
64	22	42	63	22	41	E	0	404164.919	0.186	577.6399
49	36	13	49	35	14	A	0	404368.696	-0.026	552.1277
49	36	14	49	35	15	A	0	404368.696	-0.026	552.1277
49	36	14	49	35	15	E	0	404387.163	-0.216	552.1151
42	14	29	41	13	28	A	0	404391.448	-0.005	278.7969
49	36	13	49	35	14	E	0	404415.629	-0.043	552.1255
63	18	46	62	18	45	A	0	404479.715	-0.027	537.1134
47	36	11	47	35	12	A	0	404604.413	-0.017	532.1844
47	36	12	47	35	13	A	0	404604.413	-0.017	532.1844
47	36	12	47	35	13	E	0	404623.223	-0.077	532.1717
60	15	46	59	14	45	E	0	404646.183	0.013	481.2591
24	23	1	23	22	1	E	0	404686.393	-0.026	216.1483
46	36	10	46	35	11	A	0	404712.134	-0.071	522.5247
46	36	11	46	35	12	A	0	404712.134	-0.071	522.5247
24	23	2	23	22	1	A	0	404728.124	0.041	216.1542
24	23	1	23	22	2	A	0	404728.124	0.041	216.1542
46	36	11	46	35	12	E	0	404731.114	-0.055	522.5120
45	36	9	45	35	10	A	0	404813.691	0.060	513.0729
45	36	10	45	35	11	A	0	404813.691	0.060	513.0729
45	36	10	45	35	11	E	0	404832.731	0.047	513.0602
45	36	9	45	35	10	E	0	404860.802	0.062	513.0707
44	36	8	44	35	9	A	0	404908.943	-0.032	503.8288
44	36	9	44	35	10	A	0	404908.943	-0.032	503.8288
44	36	9	44	35	10	E	0	404928.130	0.017	503.8160
44	36	8	44	35	9	E	0	404956.086	-0.027	503.8266
43	36	7	43	35	8	A	0	404998.527	0.025	494.7922
43	36	8	43	35	9	A	0	404998.527	0.025	494.7922
43	36	8	43	35	9	E	0	405017.653	-0.063	494.7794
43	36	7	43	35	8	E	0	405045.590	-0.074	494.7900
64	14	50	63	14	49	E	0	405084.117	-0.008	533.9491
64	14	50	63	14	49	A	0	405096.435	0.078	533.9474
63	17	47	62	17	46	E	0	405106.273	-0.037	531.5724
42	36	6	42	35	7	E	0	405129.639	-0.009	485.9607
63	15	49	62	14	48	E	0	405414.363	0.088	520.5794
63	15	49	62	14	48	A	0	405446.842	-0.013	520.5772
65	28	37	64	28	36	A	0	405492.212	0.123	645.7337
65	28	38	64	28	37	A	0	405492.212	0.123	645.7337
65	28	37	64	28	36	E	0	405493.905	-0.272	645.7230
65	28	37	64	28	37	E	0	405495.753	-0.069	645.7230
61	16	45	60	16	44	A	0	405520.222	-0.067	501.5316
61	16	45	60	16	44	E	0	405531.482	-0.047	501.5358
26	22	4	25	21	4	E	0	405547.665	-0.045	218.0183

J'	K'_a	K'_c	J''	K''_a	K''_c	Sym.	$v_{t_{CH_3}}$	$\nu_{Obs.}$	$\nu_{Obs.} - \nu_{Calc.}$	E_{low}
26	22	5	25	21	5	E	0	405585.044	0.024	218.0092
26	22	4	25	21	5	A	0	405591.652	-0.074	218.0226
26	22	5	25	21	4	A	0	405591.652	-0.074	218.0226
64	21	44	63	21	43	A	0	405645.496	0.085	570.0960
64	21	44	63	21	43	E	0	405663.252	0.153	570.0863
64	21	43	63	21	42	E	0	405665.064	-0.037	570.0953
64	21	43	63	21	42	A	0	405668.497	-0.076	570.0969
42	14	29	41	13	29	E	0	405847.286	-0.004	278.7467
70	9	61	69	9	60	A	1	405989.950	-0.015	705.6832
70	10	61	69	9	60	A	1	405989.950	-0.015	705.6832
70	10	61	69	10	60	A	1	405989.950	-0.014	705.6832
70	10	61	69	10	60	A	1	405989.950	-0.014	705.6832
69	10	59	68	10	58	A	1	406032.033	0.063	700.8241
69	10	59	68	11	58	A	1	406032.033	0.090	700.8241
69	11	59	68	10	58	A	1	406032.033	0.048	700.8241
69	11	59	68	11	58	A	1	406032.033	0.076	700.8241
70	9	61	69	9	60	E	1	406125.462	0.046	705.6683
70	9	61	69	10	60	E	1	406125.462	0.047	705.6683
70	10	61	69	9	60	E	1	406125.462	0.045	705.6683
70	10	61	69	10	60	E	1	406125.462	0.047	705.6683
69	10	59	68	10	58	E	1	406176.842	-0.002	700.8166
69	10	59	68	11	58	E	1	406176.842	0.033	700.8166
69	11	59	68	10	58	E	1	406176.842	-0.021	700.8166
69	11	59	68	11	58	E	1	406176.842	0.014	700.8166
70	9	61	69	9	60	E	0	406230.734	0.002	577.4206
70	10	61	69	10	60	E	0	406230.734	0.002	577.4206
70	9	61	69	9	60	A	0	406234.956	0.008	577.4201
70	9	61	69	10	60	A	0	406234.956	0.009	577.4201
70	10	61	69	9	60	A	0	406234.956	0.007	577.4201
70	10	61	69	10	60	A	0	406234.956	0.008	577.4201
69	10	59	68	10	58	E	0	406281.792	-0.018	572.5718
69	11	59	68	11	58	E	0	406281.792	-0.003	572.5718
69	10	59	68	10	58	A	0	406286.305	-0.021	572.5715
69	10	59	68	11	58	A	0	406286.305	0.011	572.5715
69	11	59	68	10	58	A	0	406286.305	-0.037	572.5715
69	11	59	68	11	58	A	0	406286.305	-0.006	572.5715
42	14	28	41	13	29	A	0	406296.442	0.002	278.7449
28	21	7	27	20	7	E	0	406392.457	-0.071	221.1124
28	21	8	27	20	8	E	0	406426.144	0.036	221.1022
28	21	8	27	20	7	A	0	406438.229	-0.063	221.1152
28	21	7	27	20	8	A	0	406438.229	-0.063	221.1152
67	12	55	66	13	54	A	1	406551.302	0.446	689.5327
67	12	55	66	12	54	A	1	406565.734	0.302	689.5322
67	13	55	66	12	54	A	1	406573.925	0.204	689.5322
65	14	51	64	15	50	A	0	406580.118	-0.015	547.5588
65	14	51	64	15	50	E	0	406581.807	-0.112	547.5597
67	12	55	66	13	54	A	0	406828.822	-0.022	561.2908
67	13	55	66	13	54	A	0	406837.902	-0.144	561.2908
67	12	55	66	12	54	E	0	406839.446	-0.011	561.2905
67	13	55	66	12	54	A	0	406854.157	-0.019	561.2902
30	20	10	29	19	10	E	0	407200.385	0.061	225.4405
30	20	11	29	19	11	E	0	407229.617	-0.027	225.4293
30	20	10	29	19	11	A	0	407247.164	-0.035	225.4416
30	20	11	29	19	10	A	0	407247.164	-0.035	225.4416
66	13	53	65	14	52	E	0	407358.682	-0.045	554.7640
66	13	53	65	14	52	A	0	407364.171	0.002	554.7635
64	20	45	63	20	44	E	0	407500.056	-0.042	563.0506
65	25	41	64	25	40	A	0	407608.597	-0.108	616.4579
65	25	40	64	25	39	A	0	407608.597	-0.111	616.4579
66	13	53	65	13	52	A	0	407617.643	-0.021	554.7551
64	20	44	63	20	43	E	0	407632.561	0.169	563.0629
32	19	13	31	18	13	E	0	407936.077	-0.206	231.0160
32	19	14	31	18	14	E	0	407960.850	0.022	231.0040
40	15	25	39	14	25	E	0	407980.917	-0.101	266.3557
32	19	14	31	18	13	A	0	407983.650	0.038	231.0155
32	19	13	31	18	14	A	0	407983.650	0.038	231.0155
40	15	26	39	14	25	A	0	407995.871	0.042	266.3494
64	15	50	63	14	49	E	0	408036.395	-0.062	533.9491
40	15	25	39	14	26	A	0	408053.421	-0.012	266.3477
64	15	50	63	14	49	A	0	408061.096	-0.016	533.9474
65	15	51	64	15	50	E	0	408448.493	-0.059	547.5597
65	15	51	64	15	50	A	0	408454.904	-0.058	547.5588
43	14	30	42	13	29	E	0	408516.116	-0.013	287.7586
64	16	49	63	16	48	E	0	408536.181	-0.273	539.7028
65	24	41	64	24	40	A	0	408537.683	0.045	607.5594
34	18	16	33	17	16	E	0	408541.034	-0.061	237.8584
34	18	17	33	17	17	E	0	408560.318	-0.012	237.8457
34	18	16	33	17	17	A	0	408588.139	-0.056	237.8562
34	18	17	33	17	16	A	0	408588.139	-0.056	237.8562
43	14	30	42	13	29	A	0	408629.040	0.042	287.7552
38	16	22	37	15	22	E	0	408854.761	-0.203	255.4715
38	16	23	37	15	23	E	0	408861.480	-0.064	255.4584
38	16	23	37	15	22	A	0	408899.439	0.441	255.4660
36	17	19	35	16	19	E	0	408911.002	-0.056	245.9958
36	17	20	35	16	20	E	0	408924.363	-0.012	245.9828
36	17	20	35	16	19	A	0	408957.187	-0.035	245.9920
65	14	51	64	14	50	E	0	409534.227	-0.024	547.4612
64	19	46	63	19	45	A	0	409571.854	0.029	556.5586
66	33	34	65	33	33	A	0	409625.896	-0.087	716.2049
66	33	33	65	33	32	A	0	409625.896	-0.087	716.2049
66	33	33	65	33	32	E	0	409625.896	0.078	716.1996
65	23	43	64	23	42	A	0	409629.928	0.222	599.1092
65	23	43	64	23	42	E	0	409635.549	0.125	599.0979
25	23	2	24	22	2	E	0	410829.655	-0.027	221.0738
25	23	3	24	22	3	E	0	410869.916	-0.235	221.0660
25	23	3	24	22	2	A	0	410871.407	0.057	221.0797
25	23	2	24	22	3	A	0	410871.407	0.057	221.0797
65	22	44	64	22	43	A	0	410936.729	0.033	591.1240
65	22	43	64	22	42	A	0	410941.538	0.073	591.1242
65	22	44	64	22	43	E	0	410945.635	-0.097	591.1134
70	10	60	69	10	59	A	1	411121.754	0.046	714.3679
70	10	60	69	11	59	A	1	411121.754	0.061	714.3679
70	11	60	69	10	59	A	1	411121.754	0.038	714.3679
70	11	60	69	11	59	A	1	411121.754	0.053	714.3679
70	10	60	69	10	59	E	1	411263.378	0.075	714.3652
70	10	60	69	11	59	E	1	411263.378	0.094	714.3652
70	11	60	69	10	59	E	1	411263.378	0.065	714.3652
70	11	60	69	11	59	E	1	411263.378	0.084	714.3652
69	11	58	68	11	57	A	1	411267.995	0.118	709.0028
69	11	58	68	12	57	A	1	411267.995	0.508	709.0028
69	12	58	68	11	57	A	1	411267.995	-0.094	709.0028
69	12	58	68	12	57	A	1	411267.995	0.295	709.0028
64	18	47	63	18	46	A	0	411352.265	0.013	550.6054
65	15	51	64	14	50	E	0	411400.878	-0.006	547.4612
69	11	58	68	11	57	E	0	411527.428	-0.079	580.7662
69	12	58	68	12	57	E	0	411527.428	0.119	580.7662

J'	K'_a	K'_c	J''	K''_a	K''_c	Sym.	$v_{t_{CH_3}}$	$\nu_{Obs.}$	$\nu_{Obs.} - \nu_{Calc.}$	E_{low}
69	11	58	68	11	57	A	0	411532.175	-0.109	580.7662
69	11	58	68	12	57	A	0	411532.175	0.331	580.7662
69	12	58	68	11	57	A	0	411532.175	-0.351	580.7662
69	12	58	68	12	57	A	0	411532.175	0.090	580.7662
27	22	5	26	21	5	E	0	411687.168	-0.051	223.3605
27	22	6	26	21	6	E	0	411724.605	0.109	223.3514
27	22	5	26	21	6	A	0	411731.163	-0.073	223.3648
27	22	6	26	21	5	A	0	411731.163	-0.073	223.3648
68	12	56	67	13	55	A	0	411865.213	-0.302	574.8614
68	12	56	67	12	55	E	0	411869.002	-0.395	574.8612
68	12	56	67	12	55	A	0	411874.623	-0.094	574.8611
68	13	56	67	12	55	A	0	411879.911	-0.023	574.8611
66	15	51	65	16	50	E	0	411952.903	0.054	567.1362
43	14	29	42	13	30	E	0	411959.801	-0.071	287.6681
66	28	38	65	28	37	A	0	411972.255	-0.078	659.2594
66	28	39	65	28	38	A	0	411972.255	-0.078	659.2594
44	14	31	43	13	30	E	0	412126.875	0.003	296.9673
68	37	32	68	36	33	A	0	412152.287	-0.077	796.7008
68	37	31	68	36	32	A	0	412152.287	-0.077	796.7008
68	37	32	68	36	33	E	0	412172.186	-0.231	796.6892
44	14	31	43	13	30	A	0	412218.156	-0.041	296.9637
66	14	52	65	15	51	E	0	412261.159	-0.186	561.1841
66	14	52	65	15	51	A	0	412262.658	0.154	561.1834
67	13	54	66	14	53	E	1	412297.307	0.251	696.5809
67	13	54	66	14	53	E	0	412399.030	0.037	568.3571
67	13	54	66	14	53	A	0	412404.583	0.046	568.3568
29	21	8	28	20	8	E	0	412523.398	-0.025	226.8740
65	21	45	64	21	44	A	0	412529.963	-0.198	583.6269
29	21	9	28	20	9	E	0	412556.895	-0.054	226.8637
65	21	44	64	21	43	E	0	412562.995	0.000	583.6268
29	21	9	28	20	8	A	0	412569.109	-0.074	226.8767
29	21	8	28	20	9	A	0	412569.109	-0.074	226.8767
65	21	44	64	21	43	A	0	412571.492	0.036	583.6286
66	27	40	65	27	39	A	0	412621.134	-0.110	649.0997
66	27	39	65	27	38	A	0	412621.134	-0.110	649.0997
66	27	40	65	27	39	E	0	412623.662	0.036	649.0886
66	27	39	65	27	38	E	0	412625.597	0.039	649.0901
67	14	54	66	13	53	E	0	412638.142	0.045	568.3521
67	14	54	66	13	53	A	0	412645.087	0.172	568.3517
44	13	31	43	12	32	E	0	412662.273	-0.046	292.6701
44	13	31	43	12	32	A	0	412672.962	0.139	292.6659
66	37	30	66	36	31	A	0	412725.310	-0.259	768.8033
66	37	29	66	36	30	A	0	412725.310	-0.259	768.8033
66	37	30	66	36	31	E	0	412746.023	-0.158	768.7916
66	37	29	66	36	30	E	0	412771.403	-0.047	768.8022
62	16	46	61	16	45	A	0	412812.969	0.124	515.0583
62	16	46	61	16	45	E	0	412818.236	-0.034	515.0628
64	37	28	64	36	29	A	0	413249.118	-0.101	741.7504
64	37	27	64	36	28	A	0	413249.118	-0.101	741.7504
31	20	11	30	19	11	E	0	413314.868	-0.059	231.6249
31	20	12	30	19	12	E	0	413344.173	0.007	231.6136
31	20	11	30	19	12	A	0	413361.757	-0.036	231.6260
31	20	12	30	19	11	A	0	413361.757	-0.036	231.6260
66	15	52	65	15	51	E	0	413426.076	-0.106	561.1841
66	15	52	65	15	51	A	0	413432.618	-0.041	561.1834
63	37	27	63	36	28	A	0	413493.430	-0.066	728.5401
63	37	26	63	36	27	A	0	413493.430	-0.066	728.5401
63	37	27	63	36	28	E	0	413514.815	-0.039	728.5284
41	15	27	40	14	26	A	0	413522.225	-0.100	274.7714
41	15	26	40	14	26	E	0	413531.324	-0.051	274.7772
41	15	27	40	14	27	E	0	413540.535	-0.048	274.7641
41	15	26	40	14	27	A	0	413636.061	-0.057	274.7682
43	12	31	42	11	32	A	0	413645.361	-0.084	279.5944
43	12	31	42	11	32	E	0	413667.524	-0.079	279.5995
62	37	26	62	36	27	A	0	413726.590	-0.012	715.5404
62	37	25	62	36	26	A	0	413726.590	-0.012	715.5404
62	37	25	62	36	26	E	0	413773.156	0.169	715.5395
65	16	50	64	16	49	E	0	413895.821	-0.028	553.3302
65	16	50	64	16	49	A	0	413899.865	-0.116	553.3292
61	37	25	61	36	26	A	0	413948.892	-0.030	702.7511
61	37	24	61	36	25	A	0	413948.892	-0.030	702.7511
61	37	24	61	36	25	E	0	413995.414	-0.000	702.7502
33	19	14	32	18	14	E	0	414022.383	-0.078	237.6279
33	19	15	32	18	15	E	0	414046.874	-0.006	237.6158
33	19	15	32	18	14	A	0	414069.721	-0.051	237.6273
33	19	14	32	18	15	A	0	414069.721	-0.051	237.6273
66	14	52	65	14	51	E	0	414127.956	-0.022	561.1218
66	14	52	65	14	51	A	0	414137.231	-0.101	561.1209
60	37	24	60	36	25	A	0	414160.792	-0.036	690.1718
60	37	23	60	36	24	A	0	414160.792	-0.036	690.1718
66	25	42	65	25	41	A	0	414206.154	-0.165	630.0542
66	25	41	65	25	40	A	0	414206.154	-0.172	630.0542
66	25	42	65	25	41	E	0	414209.993	-0.153	630.0426
66	25	41	65	25	40	E	0	414211.513	0.055	630.0470
59	37	23	59	36	24	A	0	414362.613	-0.070	677.8025
59	37	22	59	36	23	A	0	414362.613	-0.070	677.8025
59	37	23	59	36	24	E	0	414384.793	-0.086	677.7906
59	37	22	59	36	23	E	0	414409.293	-0.073	677.8016
65	20	46	64	20	45	A	0	414470.548	-0.061	576.6487
65	20	46	64	20	45	E	0	414496.554	0.324	576.6433
58	37	22	58	36	23	A	0	414554.766	-0.073	665.6429
58	37	21	58	36	22	A	0	414554.766	-0.073	665.6429
35	18	17	34	17	17	E	0	414579.253	-0.061	244.9040
35	18	18	34	17	18	E	0	414598.348	-0.010	244.8914
58	37	21	58	36	22	E	0	414601.655	0.047	665.6421
35	18	17	34	17	18	A	0	414626.351	-0.034	244.9018
35	18	18	34	17	17	A	0	414626.351	-0.034	244.9018
39	16	23	38	15	23	E	0	414672.646	-0.074	263.4157
39	16	24	38	15	24	E	0	414678.797	-0.016	263.4027
39	16	24	38	15	23	A	0	414716.080	-0.025	263.4102
39	16	23	38	15	24	A	0	414718.060	-0.170	263.4102
57	37	21	57	36	22	A	0	414737.498	-0.143	653.6929
57	37	20	57	36	21	A	0	414737.498	-0.143	653.6929
65	20	45	64	20	44	E	0	414763.931	0.058	576.6601
65	20	45	64	20	44	A	0	414771.497	0.069	576.6625
57	37	20	57	36	21	E	0	414784.553	0.065	653.6921
45	14	32	44	13	31	E	0	414830.949	-0.010	306.4351
37	17	20	36	16	20	E	0	414868.530	-0.066	253.4842
37	17	21	36	16	21	E	0	414881.593	-0.022	253.4712
48	14	35	47	13	34	E	0	414894.038	-0.052	336.4783
56	37	20	56	36	21	A	0	414911.349	-0.074	641.9523
56	37	19	56	36	20	A	0	414911.349	-0.074	641.9523
37	17	21	36	16	20	A	0	414914.696	-0.012	253.4803
56	37	20	56	36	21	E	0	414934.029	-0.110	641.9404
56	37	19	56	36	20	E	0	414958.867	0.524	641.9515
55	37	19	55	36	20	A	0	415076.458	-0.053	630.4209

J'	K_a'	K_c'	J''	K_a''	K_c''	Sym.	$v_{t_{CH_3}}$	$\nu_{Obs.}$	$\nu_{Obs.} - \nu_{Calc.}$	E_{low}
55	37	18	55	36	19	A	0	415076.458	-0.053	630.4209
55	37	19	55	36	20	E	0	415099.319	-0.064	630.4089
55	37	18	55	36	19	E	0	415123.494	-0.005	630.4201
66	24	43	65	24	42	A	0	415190.890	0.011	621.1867
64	15	49	63	15	48	E	0	415223.845	-0.018	538.9550
54	37	18	54	36	19	A	0	415233.167	-0.058	619.0985
54	37	17	54	36	18	A	0	415233.167	-0.058	619.0985
64	15	49	63	15	48	A	0	415242.976	-0.014	538.9517
54	37	18	54	36	19	E	0	415256.140	-0.102	619.0865
54	37	17	54	36	18	E	0	415280.295	0.022	619.0977
66	15	52	65	14	51	E	0	415292.922	0.108	561.1218
66	15	52	65	14	51	A	0	415307.491	0.003	561.1209
53	37	17	53	36	18	A	0	415381.771	-0.100	607.9849
53	37	16	53	36	17	A	0	415381.771	-0.100	607.9849
53	37	17	53	36	18	E	0	415404.855	-0.170	607.9729
53	37	16	53	36	17	E	0	415429.131	0.155	607.9842
52	37	16	52	36	17	A	0	415522.709	-0.045	597.0801
52	37	15	52	36	16	A	0	415522.709	-0.045	597.0801
52	37	16	52	36	17	E	0	415545.979	-0.058	597.0681
52	37	15	52	36	16	E	0	415570.102	0.194	597.0793
67	34	34	66	34	33	E	0	415641.282	-0.277	742.4382
51	37	15	51	36	16	A	0	415656.105	-0.060	586.3837
51	37	14	51	36	15	A	0	415656.105	-0.060	586.3837
51	37	15	51	36	16	E	0	415679.568	-0.003	586.3717
50	37	14	50	36	15	A	0	415782.374	-0.020	575.8958
50	37	13	50	36	14	A	0	415782.374	-0.020	575.8958
50	37	13	50	36	14	E	0	415829.532	-0.104	575.8951
49	37	13	49	36	14	A	0	415901.663	-0.056	565.6160
49	37	12	49	36	13	A	0	415901.663	-0.056	565.6160
49	37	12	49	36	13	E	0	415948.944	-0.053	565.6153
63	17	46	62	17	45	A	0	415973.398	0.011	532.5114
63	17	46	62	17	45	E	0	415992.813	-0.032	532.5139
48	37	12	48	36	13	A	0	416014.413	0.001	555.5443
48	37	11	48	36	12	A	0	416014.413	0.001	555.5443
48	37	12	48	36	13	E	0	416038.077	-0.062	555.5323
48	37	11	48	36	12	E	0	416061.865	0.142	555.5436
24	24	0	23	23	0	E	0	416108.393	-0.023	224.7235
47	37	11	47	36	12	A	0	416120.903	0.163	545.6805
47	37	10	47	36	11	A	0	416120.903	0.163	545.6805
47	37	11	47	36	12	E	0	416144.528	-0.031	545.6684
24	24	0	23	23	1	A	0	416147.104	-0.070	224.7308
24	24	1	23	23	0	A	0	416147.104	-0.070	224.7308
24	24	1	23	23	1	E	0	416151.535	0.051	224.7170
47	37	10	47	36	11	E	0	416167.981	-0.099	545.6799
46	37	10	46	36	11	A	0	416221.011	0.052	536.0245
46	37	9	46	36	10	A	0	416221.011	0.052	536.0245
46	37	10	46	36	11	E	0	416244.900	0.034	536.0124
46	37	9	46	36	10	E	0	416268.124	-0.200	536.0238
45	37	9	45	36	10	A	0	416315.264	-0.061	526.5760
45	37	8	45	36	9	A	0	416315.264	-0.061	526.5760
45	37	9	45	36	10	E	0	416339.081	-0.232	526.5639
70	11	59	69	11	58	A	1	416340.659	0.119	722.7212
70	11	59	69	12	58	A	1	416340.659	0.331	722.7212
70	12	59	69	11	58	A	1	416340.659	0.003	722.7212
70	12	59	69	12	58	A	1	416340.659	0.216	722.7212
66	23	43	65	23	42	E	0	416358.854	-0.029	612.7688
67	32	35	66	32	34	A	0	416398.160	0.050	717.6854
67	32	36	66	32	35	A	0	416398.160	0.050	717.6854
67	32	35	66	32	34	E	0	416398.160	-0.100	717.6787
44	37	8	44	36	9	A	0	416404.167	0.085	517.3351
44	37	7	44	36	8	A	0	416404.167	0.085	517.3351
47	14	34	46	13	33	E	0	416426.724	-0.086	326.1845
46	14	33	45	13	32	A	0	416433.566	0.014	316.1672
44	37	7	44	36	8	E	0	416451.401	-0.086	517.3345
64	18	46	63	18	45	A	0	416455.010	0.003	550.9337
70	11	59	69	11	58	E	0	416599.502	-0.078	594.4933
70	12	59	69	12	58	E	0	416599.502	0.031	594.4933
70	11	59	69	11	58	A	0	416604.152	-0.077	594.4934
70	11	59	69	12	58	A	0	416604.152	0.164	594.4934
70	12	59	69	11	58	A	0	416604.152	-0.209	594.4934
70	12	59	69	12	58	A	0	416604.152	0.033	594.4934
69	12	57	68	13	56	A	1	416632.145	0.466	716.8233
69	13	57	68	13	56	A	1	416634.694	0.383	716.8233
69	12	57	68	12	56	A	1	416636.738	0.374	716.8231
69	13	57	68	12	56	A	1	416639.125	0.130	716.8231
69	13	57	68	12	56	A	0	416915.143	-0.037	588.5998
26	23	3	25	22	3	E	0	416971.923	-0.027	226.2063
26	23	4	25	22	4	E	0	417012.144	-0.251	226.1984
26	23	4	25	22	3	A	0	417013.650	0.028	226.2121
26	23	3	25	22	4	A	0	417013.650	0.028	226.2121
68	13	55	67	14	54	A	1	417142.069	0.224	710.3386
68	13	55	67	14	54	E	0	417422.897	-0.015	582.1162
68	13	55	67	14	54	A	0	417428.391	-0.041	582.1161
65	17	49	64	17	48	E	0	417459.945	-0.206	558.8076
68	14	55	67	14	54	E	0	417475.038	0.065	582.1162
68	14	55	67	14	54	A	0	417480.808	0.022	582.1161
68	14	55	67	13	54	A	0	417569.981	-0.095	582.1131
67	14	53	66	15	52	E	0	417666.189	0.067	574.9745
67	14	53	66	15	52	A	0	417669.164	0.012	574.9740
66	22	45	65	22	44	A	0	417747.952	-0.240	604.8314
66	22	44	65	22	43	A	0	417756.971	-0.016	604.8318
66	22	45	65	22	44	E	0	417759.446	-0.030	604.8211
28	22	6	27	21	6	E	0	417824.452	-0.067	228.9104
28	22	7	27	21	7	E	0	417861.854	0.096	228.9013
28	22	6	27	21	7	A	0	417868.458	-0.079	228.9147
28	22	7	27	21	6	A	0	417868.458	-0.079	228.9147
65	18	48	64	18	47	A	0	418116.356	-0.026	564.3267
65	18	48	64	18	47	E	0	418123.451	0.023	564.3260
65	19	46	64	19	45	E	0	418372.867	-0.121	570.3132
67	15	53	66	15	52	E	0	418384.529	-0.039	574.9745
67	15	53	66	15	52	A	0	418390.975	-0.026	574.9740
67	28	39	66	28	38	A	0	418465.456	0.120	673.0014
67	28	40	66	28	39	A	0	418465.456	0.120	673.0014
67	28	40	66	28	39	E	0	418467.126	-0.125	672.9908
67	28	39	66	28	38	E	0	418469.441	-0.012	672.9909
68	16	52	67	17	51	A	0	418527.630	-0.060	601.1630
68	16	52	67	17	51	E	0	418577.486	-0.020	601.1635
30	21	9	29	20	9	E	0	418650.275	-0.048	232.8441
30	21	10	29	20	10	E	0	418683.813	0.024	232.8339
30	21	10	29	20	9	A	0	418696.016	-0.063	232.8469
30	21	9	29	20	10	A	0	418696.016	-0.063	232.8469
67	14	53	66	14	52	E	0	418831.055	0.097	574.9356
67	14	53	66	14	52	A	0	418839.213	-0.095	574.9350
65	15	50	64	15	49	E	0	418884.184	0.119	552.8054
65	15	50	64	15	49	A	0	418902.149	0.072	552.8027
42	15	28	41	14	27	A	0	418941.618	-0.072	283.4200

J'	K'_a	K'_c	J''	K''_a	K''_c	Sym.	$v_{t_{CH_3}}$	$\nu_{Obs.}$	$\nu_{Obs.} - \nu_{Calc.}$	E_{low}
42	15	27	41	14	27	E	0	418988.677	-0.040	283.4249
42	15	28	41	14	28	E	0	419026.597	-0.025	283.4109
66	16	51	65	16	50	E	0	419121.882	-0.182	567.1362
66	16	51	65	16	50	A	0	419126.933	-0.074	567.1354
67	27	41	66	27	40	A	0	419148.761	-0.061	662.8633
67	27	40	66	27	39	A	0	419148.761	-0.061	662.8633
67	27	41	66	27	40	E	0	419151.431	0.082	662.8522
67	27	40	66	27	39	E	0	419153.422	0.075	662.8538
42	15	27	41	14	28	A	0	419160.978	-0.020	283.4139
32	20	12	31	19	12	E	0	419422.882	-0.037	238.0190
32	20	13	31	19	13	E	0	419452.062	-0.004	238.0078
66	21	46	65	21	45	A	0	419453.689	0.019	597.3874
32	20	12	31	19	13	A	0	419469.702	-0.073	238.0201
32	20	13	31	19	12	A	0	419469.702	-0.073	238.0201
66	21	46	65	21	45	E	0	419482.731	-0.068	597.3791
66	21	45	65	21	44	E	0	419513.052	0.050	597.3884
66	21	45	65	21	44	A	0	419525.945	-0.155	597.3905
67	15	52	66	16	51	A	0	419538.630	-0.069	581.1160
67	15	53	66	14	52	E	0	419549.397	-0.008	574.9356
67	15	52	66	16	51	E	0	419551.907	-0.002	581.1166
67	15	53	66	14	52	A	0	419561.145	-0.012	574.9350
67	26	41	66	26	40	A	0	419927.203	-0.056	653.1501
67	26	42	66	26	41	A	0	419927.203	-0.055	653.1501
67	26	42	66	26	41	E	0	419930.421	-0.069	653.1388
67	26	41	66	26	40	E	0	419932.096	-0.110	653.1418
68	41	28	67	41	27	E	0	420073.366	-0.196	855.3557
68	41	28	67	41	27	A	0	420074.934	0.048	855.3493
68	41	27	67	41	26	A	0	420074.934	0.048	855.3493
68	41	27	67	41	26	E	0	420074.934	-0.136	855.3426
34	19	15	33	18	15	E	0	420098.091	-0.051	244.4510
34	19	16	33	18	16	E	0	420122.416	-0.007	244.4390
34	19	16	33	18	15	A	0	420145.380	-0.054	244.4504
34	19	15	33	18	16	A	0	420145.380	-0.054	244.4504
40	16	24	39	15	24	E	0	420446.819	-0.094	271.5797
40	16	25	39	15	25	E	0	420452.458	-0.015	271.5668
40	16	25	39	15	24	A	0	420488.851	-0.141	271.5742
40	16	24	39	15	25	A	0	420493.604	-0.007	271.5740
36	18	18	35	17	18	E	0	420601.022	-0.064	252.1629
36	18	19	35	17	19	E	0	420619.907	-0.011	252.1503
36	18	18	35	17	19	A	0	420648.067	-0.059	252.1607
36	18	19	35	17	18	A	0	420648.067	-0.059	252.1607
38	17	21	37	16	21	E	0	420799.982	-0.040	261.1885
38	17	22	37	16	22	E	0	420812.673	-0.038	261.1755
67	25	43	66	25	42	A	0	420822.953	-0.022	643.8707
67	25	43	66	25	42	E	0	420826.951	-0.078	643.8592
38	17	22	37	16	21	A	0	420846.049	-0.024	261.1845
66	20	47	65	20	46	A	0	421505.146	-0.111	590.4740
66	20	47	65	20	46	E	0	421524.838	-0.032	590.4694
67	24	44	66	24	43	A	0	421866.774	-0.006	635.0360
70	12	58	69	13	57	A	0	421953.245	-0.230	602.5066
70	13	58	69	13	57	A	0	421954.956	-0.166	602.5066
70	12	58	69	12	57	A	0	421956.468	0.054	602.5065
70	13	58	69	12	57	A	0	421958.046	-0.015	602.5065
66	20	46	65	20	45	A	0	422002.782	-0.104	590.4978
69	13	56	68	14	55	A	1	422160.479	0.435	724.2545
25	24	1	24	23	2	A	0	422290.507	-0.063	229.6545
25	24	2	24	23	1	A	0	422290.507	-0.063	229.6545
25	24	2	24	23	2	E	0	422294.907	0.048	229.6407
69	13	56	68	14	55	E	1	422334.017	0.070	724.2591
69	13	56	68	14	55	E	0	422440.849	0.099	596.0416
69	13	56	68	14	55	A	0	422446.017	-0.157	596.0418
66	15	51	65	15	50	E	0	422696.280	0.022	566.7779
66	15	51	65	15	50	A	0	422712.349	0.002	566.7758
68	14	54	67	15	53	E	0	422896.656	0.001	588.9303
68	14	54	67	15	53	A	0	422900.892	0.078	588.9301
67	23	44	66	23	43	E	0	423109.974	0.074	626.6570
27	23	4	26	22	4	E	0	423112.868	-0.033	231.5459
27	23	5	26	22	5	E	0	423153.073	-0.246	231.5380
27	23	5	26	22	4	A	0	423154.608	0.032	231.5517
27	23	4	26	22	5	A	0	423154.608	0.032	231.5517
68	31	38	67	31	37	E	0	423262.128	-0.287	719.7956
66	17	50	65	17	49	A	0	423331.905	-0.105	572.7320
66	17	50	65	17	49	E	0	423331.905	0.115	572.7326
70	38	32	70	37	34	E	0	423391.062	-0.248	839.1609
70	38	33	70	37	33	E	0	423412.335	0.015	839.1721
68	14	54	67	14	53	E	0	423615.137	0.036	588.9063
68	14	54	67	14	53	A	0	423622.659	-0.004	588.9060
66	19	48	65	19	47	A	0	423625.676	-0.074	584.1171
66	19	48	65	19	47	E	0	423636.365	0.151	584.1149
69	38	31	69	37	32	A	0	423662.440	-0.034	824.7045
69	38	32	69	37	33	A	0	423662.440	-0.034	824.7045
69	38	32	69	37	32	E	0	423708.123	0.104	824.7049
29	22	7	28	21	7	E	0	423959.145	-0.025	234.6682
29	22	8	28	21	8	E	0	423996.379	0.015	234.6591
29	22	7	28	21	8	A	0	424003.116	-0.071	234.6725
29	22	8	28	21	7	A	0	424003.116	-0.071	234.6725
68	15	54	67	14	53	E	0	424053.523	-0.036	588.9063
68	15	54	67	14	53	A	0	424063.310	0.039	588.9060
45	14	31	44	13	32	A	0	424108.782	-0.073	306.1890
67	38	29	67	37	30	A	0	424216.397	0.026	796.4040
67	38	30	67	37	31	A	0	424216.397	0.026	796.4040
43	15	29	42	14	28	A	0	424221.849	-0.056	292.2974
67	16	52	66	16	51	E	0	424243.315	-0.094	581.1166
67	16	52	66	16	51	A	0	424248.770	-0.166	581.1160
43	15	28	42	14	28	E	0	424321.524	-0.013	292.3013
68	29	40	67	29	39	A	0	424334.920	-0.093	697.4959
68	29	39	67	29	38	A	0	424334.920	-0.093	697.4959
68	29	39	67	29	38	E	0	424336.507	0.023	697.4862
68	29	40	67	29	39	E	0	424338.309	-0.606	697.4848
43	15	29	42	14	29	E	0	424446.526	0.086	292.2843
64	17	47	63	17	46	A	0	424454.188	-0.035	546.3867
64	17	47	63	17	46	E	0	424470.459	-0.033	546.3899
66	38	29	66	37	29	E	0	424521.523	0.093	782.5708
67	22	46	66	22	45	A	0	424594.092	0.115	618.7660
43	15	28	42	14	29	A	0	424634.376	-0.085	292.2859
65	38	27	65	37	28	A	0	424723.223	-0.080	768.9473
65	38	28	65	37	29	A	0	424723.223	-0.080	768.9473
31	21	10	30	20	10	E	0	424772.602	-0.030	239.0232
31	21	11	30	20	11	E	0	424806.039	0.008	239.0130
31	21	11	30	20	10	A	0	424818.328	-0.056	239.0260
31	21	10	30	20	11	A	0	424818.328	-0.056	239.0260
64	16	48	63	16	47	E	0	424942.016	-0.007	542.8194
64	16	48	63	16	47	A	0	424949.412	0.084	542.8147
64	38	26	64	37	27	A	0	424960.050	-0.065	755.5349
64	38	27	64	37	28	A	0	424960.050	-0.065	755.5349
68	28	40	67	28	39	A	0	424971.490	-0.024	686.9599

J'	K'_a	K'_c	J''	K''_a	K''_c	Sym.	$v_{t_{CH_3}}$	$\nu_{Obs.}$	$\nu_{Obs.} - \nu_{Calc.}$	E_{low}
68	28	41	67	28	40	A	0	424971.490	-0.024	686.9599
68	28	41	67	28	40	E	0	424973.573	0.026	686.9494
68	28	40	67	28	39	E	0	424975.772	-0.057	686.9496
64	38	26	64	37	28	E	0	424985.877	-0.200	755.5239
64	38	27	64	37	27	E	0	425006.267	0.026	755.5355
65	18	47	64	18	46	E	0	425200.256	-0.008	564.8258
62	38	24	62	37	25	A	0	425402.237	-0.022	729.3409
62	38	25	62	37	26	A	0	425402.237	-0.022	729.3409
62	38	24	62	37	26	E	0	425428.605	-0.045	729.3298
62	38	25	62	37	25	E	0	425448.422	-0.145	729.3415
33	20	13	32	19	13	E	0	425523.512	0.029	244.6233
33	20	14	32	19	14	E	0	425552.367	-0.162	244.6121
33	20	13	32	19	14	A	0	425570.266	-0.063	244.6244
33	20	14	32	19	13	A	0	425570.266	-0.063	244.6244
61	38	23	61	37	24	A	0	425608.039	-0.256	716.5589
61	38	24	61	37	25	A	0	425608.039	-0.256	716.5589
61	38	23	61	37	25	E	0	425634.758	-0.125	716.5478
61	38	24	61	37	24	E	0	425654.689	0.006	716.5596
68	27	42	67	27	41	A	0	425691.347	0.064	676.8446
68	27	41	67	27	40	A	0	425691.347	0.063	676.8446
68	27	42	67	27	41	E	0	425693.458	-0.503	676.8336
68	27	41	67	27	40	E	0	425695.964	-0.061	676.8353
60	38	22	60	37	23	A	0	425804.696	-0.061	703.9867
60	38	23	60	37	24	A	0	425804.696	-0.061	703.9867
60	38	22	60	37	24	E	0	425831.344	-0.190	703.9756
60	38	23	60	37	23	E	0	425851.201	-0.019	703.9874
59	38	21	59	37	22	A	0	425991.724	-0.252	691.6241
59	38	22	59	37	23	A	0	425991.724	-0.252	691.6241
59	38	21	59	37	23	E	0	426018.742	-0.189	691.6130
59	38	22	59	37	22	E	0	426038.570	0.061	691.6248
35	19	16	34	18	16	E	0	426162.126	-0.056	251.4858
58	38	20	58	37	21	A	0	426170.171	-0.101	679.4710
58	38	21	58	37	22	A	0	426170.171	-0.101	679.4710
41	16	25	40	15	25	E	0	426172.467	-0.035	279.9645
41	16	26	40	15	26	E	0	426177.474	-0.045	279.9516
35	19	17	34	18	17	E	0	426186.312	0.003	251.4738
58	38	20	58	37	22	E	0	426197.274	-0.121	679.4598
35	19	17	34	18	16	A	0	426209.391	-0.064	251.4852
35	19	16	34	18	17	A	0	426209.391	-0.064	251.4852
41	16	26	40	15	25	A	0	426211.863	-0.105	279.9589
58	38	21	58	37	21	E	0	426216.792	-0.076	679.4717
41	16	25	40	15	26	A	0	426221.769	0.019	279.9586
68	15	53	67	16	52	E	0	426267.728	-0.022	595.2679
66	19	47	65	19	46	A	0	426297.952	-0.117	584.2700
66	19	47	65	19	46	E	0	426312.663	0.058	584.2686
57	38	19	57	37	20	A	0	426339.923	-0.035	667.5271
57	38	20	57	37	21	A	0	426339.923	-0.035	667.5271
57	38	19	57	37	21	E	0	426367.192	-0.048	667.5159
57	38	20	57	37	20	E	0	426386.602	-0.010	667.5278
67	21	47	66	21	46	E	0	426447.599	0.067	611.3716
56	38	18	56	37	19	A	0	426501.244	-0.092	655.7923
56	38	19	56	37	20	A	0	426501.244	-0.092	655.7923
68	26	42	67	26	41	A	0	426512.333	-0.151	667.1574
68	26	43	67	26	42	A	0	426512.333	-0.149	667.1574
68	26	43	67	26	42	E	0	426515.872	-0.031	667.1461
68	26	42	67	26	41	E	0	426517.625	-0.041	667.1493
67	21	46	66	21	45	A	0	426541.594	-0.010	611.3844
56	38	19	56	37	19	E	0	426547.980	-0.063	655.7930
37	18	19	36	17	19	E	0	426604.674	-0.054	259.6356
37	18	20	36	17	20	E	0	426623.307	-0.021	259.6230
37	18	19	36	17	20	A	0	426651.713	-0.025	259.6333
37	18	20	36	17	19	A	0	426651.713	-0.024	259.6333
39	17	22	38	16	22	E	0	426702.415	-0.232	269.1094
39	17	23	38	16	23	E	0	426714.943	-0.028	269.0965
39	17	23	38	16	22	A	0	426748.626	0.013	269.1054
54	38	16	54	37	17	A	0	426800.290	-0.066	632.9492
54	38	17	54	37	18	A	0	426800.290	-0.066	632.9492
53	38	15	53	37	16	A	0	426938.547	-0.021	621.8406
53	38	16	53	37	17	A	0	426938.547	-0.021	621.8406
53	38	15	53	37	17	E	0	426966.337	-0.065	621.8293
53	38	16	53	37	16	E	0	426985.429	0.023	621.8414
51	38	13	51	37	14	A	0	427193.618	-0.158	600.2485
51	38	14	51	37	15	A	0	427193.618	-0.158	600.2485
51	38	14	51	37	14	E	0	427240.558	-0.121	600.2494
50	38	12	50	37	13	A	0	427311.201	-0.101	589.7648
50	38	13	50	37	14	A	0	427311.201	-0.101	589.7648
50	38	12	50	37	14	E	0	427339.447	-0.020	589.7535
49	38	11	49	37	12	A	0	427422.385	-0.067	579.4890
49	38	12	49	37	13	A	0	427422.385	-0.067	579.4890
49	38	11	49	37	13	E	0	427450.551	-0.163	579.4777
70	14	57	69	13	56	A	0	427511.719	-0.069	610.1330
48	38	10	48	37	11	A	0	427527.467	-0.009	569.4211
48	38	11	48	37	12	A	0	427527.467	-0.009	569.4211
48	38	11	48	37	11	E	0	427574.342	-0.108	569.4220
47	38	9	47	37	10	A	0	427626.579	-0.039	559.5608
47	38	10	47	37	11	A	0	427626.579	-0.039	559.5608
47	38	9	47	37	11	E	0	427655.001	-0.052	559.5495
46	38	8	46	37	9	A	0	427720.112	-0.002	549.9081
46	38	9	46	37	10	A	0	427720.112	-0.002	549.9081
46	38	8	46	37	10	E	0	427748.521	-0.105	549.8968
45	38	7	45	37	8	A	0	427808.385	0.190	540.4628
45	38	8	45	37	9	A	0	427808.385	0.190	540.4628
69	15	55	68	15	54	E	0	428285.358	-0.262	603.0512
69	15	55	68	15	54	A	0	428291.654	-0.098	603.0512
26	24	2	25	23	2	E	0	428394.487	-0.041	234.7776
26	24	2	25	23	3	A	0	428433.224	-0.073	234.7849
26	24	3	25	23	2	A	0	428433.224	-0.073	234.7849
26	24	3	25	23	3	E	0	428437.612	0.050	234.7711
69	14	55	68	14	54	A	0	428465.863	-0.146	603.0365
67	20	48	66	20	47	A	0	428564.354	-0.126	604.5339
67	20	48	66	20	47	E	0	428580.052	0.062	604.5300
69	15	55	68	14	54	E	0	428724.080	0.000	603.0366
69	15	55	68	14	54	A	0	428732.376	0.015	603.0365
69	33	37	68	33	36	A	0	428776.467	-0.072	757.8341
69	33	36	68	33	35	A	0	428776.467	-0.072	757.8341
69	33	36	68	33	35	E	0	428776.467	0.062	757.8287
69	33	37	68	33	36	E	0	428778.907	-0.142	757.8217
69	16	53	68	17	52	A	0	428916.986	0.174	615.6563
69	16	53	68	17	52	E	0	428951.513	-0.018	615.6567
67	17	51	66	17	50	E	0	429004.953	-0.027	586.8534
67	17	51	66	17	50	A	0	429006.607	0.035	586.8529
44	15	30	43	14	29	E	0	429086.724	0.131	301.4096
28	23	5	27	22	5	E	0	429252.156	-0.030	237.0929
68	16	53	67	16	52	E	0	429287.060	-0.071	595.2679
28	23	6	27	22	6	E	0	429292.376	-0.197	237.0850
28	23	6	27	22	5	A	0	429293.879	0.014	237.0987

J'	K_a'	K_c'	J''	K_a''	K_c''	Sym.	$v_{t_{CH_3}}$	$\nu_{Obs.}$	$\nu_{Obs.} - \nu_{Calc.}$	E_{low}
28	23	5	27	22	6	A	0	429293.879	0.014	237.0987
44	15	30	43	14	29	A	0	429314.860	-0.046	301.4066
67	20	47	66	20	46	A	0	429371.573	-0.007	604.5743
67	20	47	66	20	46	E	0	429376.973	-0.022	604.5716
44	15	29	43	14	29	E	0	429481.968	-0.163	301.4096
65	16	50	64	15	49	E	0	429627.421	-0.052	552.8054
65	16	49	64	16	48	E	0	429785.889	-0.090	556.9939
65	16	49	64	16	48	A	0	429798.835	-0.028	556.9895
44	15	30	43	14	30	E	0	429815.751	0.011	301.3852
66	16	51	65	15	50	E	0	429865.446	-0.026	566.7779
68	23	46	67	23	45	A	0	429882.407	0.111	640.7742
68	23	46	67	23	45	E	0	429890.508	0.082	640.7635
66	16	51	65	15	50	A	0	429908.603	-0.136	566.7758
44	15	29	43	14	30	A	0	430072.414	-0.204	301.3856
30	22	8	29	21	8	E	0	430090.711	0.011	240.6343
30	22	9	29	21	9	E	0	430127.856	0.011	240.6251
30	22	8	29	21	9	A	0	430134.651	-0.068	240.6386
30	22	9	29	21	8	A	0	430134.651	-0.068	240.6386
67	19	49	66	19	48	E	0	430599.255	-0.007	598.2459
69	29	41	68	29	40	A	0	430822.130	0.035	711.6502
69	29	40	68	29	39	A	0	430822.130	0.035	711.6502
69	29	40	68	29	39	E	0	430823.687	0.027	711.6405
69	29	41	68	29	40	E	0	430826.180	-0.005	711.6392
32	21	11	31	20	11	E	0	430889.664	-0.052	245.4116
32	21	12	31	20	12	E	0	430923.046	0.007	245.4013
32	21	12	31	20	11	A	0	430935.395	-0.069	245.4143
32	21	11	31	20	12	A	0	430935.395	-0.069	245.4143
68	15	53	67	15	52	E	0	430959.226	-0.024	595.1114
68	15	53	67	15	52	A	0	430971.223	-0.041	595.1103
46	14	32	45	13	33	E	0	431050.500	-0.071	315.7910
46	14	32	45	13	33	A	0	431069.386	-0.075	315.7879
67	18	50	66	18	49	A	0	431192.015	0.032	592.4413
67	18	50	66	18	49	E	0	431196.543	0.065	592.4410
67	16	52	66	15	51	E	0	431412.654	0.030	580.8775
68	22	47	67	22	46	A	0	431476.102	0.116	632.9289
69	28	41	68	28	40	A	0	431491.348	0.046	701.1354
69	28	42	68	28	41	A	0	431491.348	0.046	701.1354
69	28	42	68	28	41	E	0	431493.642	0.182	701.1250
68	22	46	67	22	45	E	0	431500.371	-0.019	632.9275
68	22	46	67	22	45	A	0	431504.551	0.009	632.9301
34	20	14	33	19	14	E	0	431615.725	-0.029	251.4382
34	20	15	33	19	15	E	0	431644.685	-0.001	251.4270
34	20	14	33	19	15	A	0	431662.526	-0.063	251.4392
34	20	15	33	19	14	A	0	431662.526	-0.063	251.4392
42	16	26	41	15	26	E	0	431843.638	-0.012	288.5711
42	16	27	41	15	27	E	0	431848.250	-0.024	288.5583
42	16	27	41	15	26	A	0	431877.876	-0.110	288.5656
42	16	26	41	15	27	A	0	431898.175	-0.014	288.5650
36	19	17	35	18	17	E	0	432213.324	-0.035	258.7329
36	19	18	35	18	18	E	0	432237.330	0.012	258.7209
69	27	43	68	27	42	A	0	432249.123	-0.054	691.0441
69	27	42	68	27	41	A	0	432249.123	-0.055	691.0441
36	19	18	35	18	17	A	0	432260.593	-0.019	258.7323
36	19	17	35	18	18	A	0	432260.593	-0.019	258.7323
69	15	54	68	16	53	A	0	432359.938	0.199	609.5871
69	15	54	68	16	53	E	0	432361.969	-0.022	609.5874
65	17	48	64	17	47	A	0	432397.537	-0.403	560.5450
40	17	23	39	16	23	E	0	432573.447	-0.056	277.2477
40	17	24	39	16	24	E	0	432585.365	-0.056	277.2349
38	18	20	37	17	20	E	0	432588.341	-0.083	267.3227
38	18	21	37	17	21	E	0	432606.786	0.020	267.3102
38	18	20	37	17	21	A	0	432635.363	-0.039	267.3204
38	18	21	37	17	20	A	0	432635.363	-0.037	267.3204
70	14	56	69	15	55	E	1	432963.984	0.267	745.5388
69	26	43	68	26	42	A	0	433115.516	0.034	681.3843
70	15	56	69	15	55	E	0	433241.017	-0.035	617.3373
70	15	56	69	15	55	A	0	433246.830	-0.146	617.3375
70	14	56	69	14	55	E	0	433347.223	-0.088	617.3285
70	14	56	69	14	55	A	0	433353.669	-0.077	617.3286
68	21	48	67	21	47	A	0	433418.548	-0.047	625.6026
68	21	48	67	21	47	E	0	433445.851	-0.001	625.5963
70	15	56	69	14	55	A	0	433513.279	-0.048	617.3286
68	21	47	67	21	46	E	0	433621.515	0.075	625.6093
68	21	47	67	21	46	A	0	433630.383	-0.143	625.6123
25	25	0	24	24	0	E	0	433673.170	-0.043	238.6034
25	25	1	24	24	0	A	0	433708.459	-0.094	238.6120
25	25	0	24	24	1	A	0	433708.459	-0.094	238.6120
25	25	1	24	24	1	E	0	433718.286	0.017	238.5984
45	15	31	44	14	30	E	0	433973.065	0.175	310.7536
68	16	53	67	15	52	E	0	433978.550	-0.081	595.1114
68	16	53	67	15	52	A	0	434003.269	0.000	595.1103
66	18	48	65	18	47	E	0	434084.162	-0.198	579.0090
69	25	45	68	25	44	A	0	434116.989	-0.015	672.1663
45	15	31	44	14	30	A	0	434148.447	-0.108	310.7511
69	16	54	68	16	53	E	0	434277.110	-0.299	609.5874
69	16	54	68	16	53	A	0	434283.370	-0.133	609.5871
45	15	30	44	14	30	E	0	434415.097	-0.175	310.7536
27	24	3	26	23	3	E	0	434536.248	-0.057	240.1150
67	19	48	66	19	47	A	0	434543.988	-0.146	598.4897
67	19	48	66	19	47	E	0	434561.839	0.022	598.4889
27	24	3	26	23	4	A	0	434574.979	-0.101	240.1222
27	24	4	26	23	3	A	0	434574.979	-0.101	240.1222
27	24	4	26	23	4	E	0	434579.359	0.043	240.1084
45	15	31	44	14	31	E	0	435149.329	0.008	310.7143
70	39	32	70	38	33	A	0	435161.850	-0.115	853.2937
70	39	31	70	38	32	A	0	435161.850	-0.115	853.2937
69	15	54	68	15	53	E	0	435381.350	-0.022	609.4866
29	23	6	28	22	6	E	0	435389.403	-0.031	242.8475
69	15	54	68	15	53	A	0	435391.702	-0.042	609.4860
29	23	7	28	22	7	E	0	435429.566	-0.219	242.8396
29	23	7	28	22	6	A	0	435431.105	-0.011	242.8533
29	23	6	28	22	7	A	0	435431.105	-0.011	242.8533
69	39	31	69	38	32	A	0	435435.713	-0.128	838.8364
69	39	30	69	38	31	A	0	435435.713	-0.128	838.8364
69	39	30	69	38	31	E	0	435464.919	-0.154	838.8264
69	39	31	69	38	32	E	0	435480.784	-0.054	838.8383
45	15	30	44	14	31	A	0	435507.138	-0.108	310.7139
68	20	49	67	20	48	A	0	435635.785	0.073	618.8292
70	32	39	69	32	38	E	0	435635.785	-0.271	759.9827
67	39	28	67	38	29	E	0	435978.925	-0.222	810.5443
67	39	29	67	38	30	E	0	435994.655	0.056	810.5564
46	13	33	45	12	34	E	0	436160.450	-0.068	311.6357
66	39	28	66	38	29	A	0	436189.611	-0.219	796.7293
66	39	27	66	38	28	A	0	436189.611	-0.219	796.7293
31	22	9	30	21	9	E	0	436218.541	-0.073	246.8088
66	39	28	66	38	29	E	0	436235.210	0.105	796.7313

J'	K'_a	K'_c	J''	K''_a	K''_c	Sym.	$v_{t_{CH_3}}$	$\nu_{Obs.}$	$\nu_{Obs.} - \nu_{Calc.}$	E_{low}
31	22	10	30	21	10	E	0	436255.684	-0.018	246.7997
31	22	9	30	21	10	A	0	436262.537	-0.095	246.8131
31	22	10	30	21	9	A	0	436262.537	-0.095	246.8131
65	39	27	65	38	28	A	0	436419.773	-0.035	783.1146
65	39	26	65	38	27	A	0	436419.773	-0.035	783.1146
65	39	26	65	38	27	E	0	436449.843	-0.160	783.1045
64	39	26	64	38	27	A	0	436639.476	-0.208	769.7100
64	39	25	64	38	26	A	0	436639.476	-0.208	769.7100
63	39	24	63	38	25	E	0	436880.245	-0.154	756.5053
33	21	12	32	20	12	E	0	437000.807	-0.096	252.0095
33	21	13	32	20	13	E	0	437034.124	-0.018	251.9992
33	21	13	32	20	12	A	0	437046.566	-0.080	252.0121
33	21	12	32	20	13	A	0	437046.566	-0.080	252.0121
61	39	23	61	38	24	A	0	437241.624	-0.346	730.7557
61	39	22	61	38	23	A	0	437241.624	-0.346	730.7557
61	39	23	61	38	24	E	0	437287.563	-0.027	730.7579
69	16	54	68	15	53	E	0	437296.677	-0.112	609.4866
69	16	54	68	15	53	A	0	437315.574	0.066	609.4860
70	29	42	69	29	41	A	0	437321.654	-0.049	726.0209
70	29	41	69	29	40	A	0	437321.654	-0.049	726.0209
70	29	41	69	29	40	E	0	437323.321	-0.048	726.0112
60	39	22	60	38	23	A	0	437424.507	-0.161	718.1900
60	39	21	60	38	22	A	0	437424.507	-0.161	718.1900
43	16	27	42	15	27	E	0	437453.257	-0.114	297.4009
43	16	28	42	15	28	E	0	437458.322	-0.065	297.3881
43	16	28	42	15	27	A	0	437477.865	-0.095	297.3956
59	39	21	59	38	22	A	0	437598.778	-0.062	705.8337
59	39	20	59	38	21	A	0	437598.778	-0.062	705.8337
59	39	20	59	38	21	E	0	437630.027	-0.130	705.8235
35	20	15	34	19	15	E	0	437698.701	-0.108	258.4640
35	20	16	34	19	16	E	0	437727.601	-0.014	258.4528
58	39	20	58	38	21	A	0	437764.632	-0.144	693.6865
58	39	19	58	38	20	A	0	437764.632	-0.144	693.6865
57	39	19	57	38	20	A	0	437922.654	-0.107	681.7483
57	39	18	57	38	19	A	0	437922.654	-0.107	681.7483
57	39	18	57	38	19	E	0	437954.461	0.083	681.7380
70	28	42	69	28	41	A	0	438025.139	-0.026	715.5284
70	28	43	69	28	42	A	0	438025.139	-0.026	715.5284
70	28	43	69	28	42	E	0	438027.379	-0.073	715.5181
56	39	18	56	38	19	A	0	438072.976	-0.098	670.0188
56	39	17	56	38	18	A	0	438072.976	-0.098	670.0188
56	39	18	56	38	19	E	0	438118.919	0.006	670.0211
55	39	17	55	38	18	A	0	438215.886	-0.101	658.4980
55	39	16	55	38	17	A	0	438215.886	-0.101	658.4980
37	19	18	36	18	18	E	0	438250.313	-0.055	266.1927
37	19	19	36	18	19	E	0	438274.132	-0.008	266.1807
37	19	19	36	18	18	A	0	438297.526	-0.075	266.1920
37	19	18	36	18	19	A	0	438297.526	-0.075	266.1920
54	39	16	54	38	17	A	0	438351.655	-0.110	647.1857
54	39	15	54	38	16	A	0	438351.655	-0.110	647.1857
54	39	15	54	38	16	E	0	438383.637	-0.131	647.1754
69	22	48	68	22	47	A	0	438395.911	-0.053	647.3214
41	17	24	40	16	24	E	0	438409.199	-0.104	285.6043
41	17	25	40	16	25	E	0	438420.725	-0.048	285.5915
69	22	48	68	22	47	E	0	438420.725	-0.126	647.3126
69	22	47	68	22	46	E	0	438437.591	0.040	647.3208
69	22	47	68	22	46	A	0	438446.161	-0.075	647.3235
41	17	25	40	16	24	A	0	438455.162	0.272	285.6002
53	39	15	53	38	16	A	0	438480.679	0.015	636.0817
53	39	14	53	38	15	A	0	438480.679	0.015	636.0817
46	15	32	45	14	31	E	0	438484.484	-0.004	320.3381
53	39	14	53	38	15	E	0	438512.873	0.093	636.0714
39	18	21	38	17	21	E	0	438550.115	-0.092	275.2249
39	18	21	38	17	22	A	0	438597.065	-0.090	275.2225
39	18	22	38	17	21	A	0	438597.065	-0.083	275.2225
52	39	14	52	38	15	A	0	438602.878	-0.061	625.1859
52	39	13	52	38	14	A	0	438602.878	-0.061	625.1859
46	15	32	45	14	31	A	0	438616.011	-0.118	320.3358
52	39	13	52	38	14	E	0	438635.143	-0.018	625.1756
52	39	14	52	38	15	E	0	438648.824	-0.055	625.1883
51	39	13	51	38	14	A	0	438718.752	-0.082	614.4982
51	39	12	51	38	13	A	0	438718.752	-0.082	614.4982
51	39	12	51	38	13	E	0	438751.164	0.009	614.4879
70	27	44	69	27	43	A	0	438823.025	-0.066	705.4624
70	27	43	69	27	42	A	0	438823.025	-0.067	705.4624
70	27	44	69	27	43	E	0	438826.092	-0.001	705.4516
50	39	12	50	38	13	A	0	438828.439	-0.152	604.0184
50	39	11	50	38	12	A	0	438828.439	-0.152	604.0184
50	39	12	50	38	13	E	0	438874.787	0.225	604.0208
49	39	11	49	38	12	A	0	438932.372	-0.071	593.7463
49	39	10	49	38	11	A	0	438932.372	-0.071	593.7463
49	39	10	49	38	11	E	0	438964.823	-0.120	593.7360
47	14	33	46	13	34	E	0	439018.033	-0.113	325.6108
48	39	10	48	38	11	A	0	439030.561	-0.060	583.6819
48	39	9	48	38	10	A	0	439030.561	-0.060	583.6819
47	14	33	46	13	34	A	0	439033.690	-0.108	325.6076
46	15	31	45	14	31	E	0	439059.779	-0.095	320.3381
48	39	9	48	38	10	E	0	439063.010	-0.191	583.6715
48	39	10	48	38	11	E	0	439076.596	-0.017	583.6843
47	39	9	47	38	10	A	0	439123.302	-0.046	573.8249
47	39	8	47	38	9	A	0	439123.302	-0.046	573.8249
47	39	8	47	38	9	E	0	439155.967	-0.035	573.8146
70	16	55	69	16	54	E	0	439234.234	-0.001	624.0733
70	16	55	69	16	54	A	0	439240.295	-0.080	624.0732
66	17	49	65	17	48	A	0	439610.973	-0.044	574.9683
66	17	49	65	17	48	E	0	439616.044	-0.089	574.9724
26	25	1	25	24	1	E	0	439815.983	-0.028	243.7320
69	17	53	68	17	52	E	0	439826.712	-0.017	615.6567
69	17	53	68	17	52	A	0	439830.463	-0.084	615.6563
26	25	2	25	24	1	A	0	439851.258	-0.101	243.7406
26	25	1	25	24	2	A	0	439851.258	-0.101	243.7406
26	25	2	25	24	2	E	0	439861.052	-0.002	243.7270
28	24	4	27	23	4	E	0	440676.803	-0.040	245.6594
28	24	4	27	23	5	A	0	440715.512	-0.112	245.6666
28	24	5	27	23	4	A	0	440715.512	-0.112	245.6666
28	24	5	27	23	5	E	0	440719.827	-0.003	245.6529
70	25	46	69	25	45	A	0	440796.417	0.039	686.6469
70	25	46	69	25	45	E	0	440801.154	-0.072	686.6358
70	25	45	69	25	44	E	0	440802.826	0.217	686.6405
69	21	48	68	21	47	E	0	440807.901	-0.219	640.0733
69	21	48	68	21	47	A	0	440810.158	-0.127	640.0766
46	15	31	45	14	32	A	0	440994.074	0.008	320.2713
46	15	31	45	14	32	E	0	441032.387	-0.188	320.2723
70	16	55	69	15	54	E	0	441149.597	-0.055	624.0094
70	16	55	69	15	54	A	0	441164.042	-0.097	624.0091
30	23	7	29	22	7	E	0	441524.184	-0.065	248.8100

J'	K'_a	K'_c	J''	K''_a	K''_c	Sym.	$v_{t_{CH_3}}$	$\nu_{Obs.}$	$\nu_{Obs.} - \nu_{Calc.}$	E_{low}
30	23	8	29	22	8	E	0	441564.375	-0.183	248.8021
30	23	8	29	22	7	A	0	441565.916	-0.019	248.8157
30	23	7	29	22	8	A	0	441565.916	-0.019	248.8157
32	22	10	31	21	10	E	0	442342.309	-0.071	253.1921
32	22	11	31	21	11	E	0	442379.582	0.176	253.1830
32	22	10	31	21	11	A	0	442386.310	-0.089	253.1964
32	22	11	31	21	10	A	0	442386.310	-0.089	253.1964
47	15	33	46	14	32	E	0	442462.292	-0.058	330.1693
47	15	33	46	14	32	A	0	442564.376	-0.086	330.1669
69	20	50	68	20	49	A	0	442701.065	-0.020	633.3605
69	20	50	68	20	49	E	0	442712.728	0.073	633.3576
67	18	49	66	18	48	A	0	442885.199	-0.032	593.4863
67	18	49	66	18	48	E	0	442905.375	-0.016	593.4885
44	16	28	43	15	28	E	0	442992.613	-0.066	306.4551
44	16	29	43	15	28	A	0	442999.536	-0.118	306.4502
44	16	29	43	15	29	E	0	443000.956	-0.244	306.4423
44	16	28	43	15	29	A	0	443079.774	-0.095	306.4480
34	21	13	33	20	13	E	0	443105.411	-0.069	258.8173
68	19	49	67	19	48	E	0	443136.228	-0.025	612.9843
34	21	14	33	20	14	E	0	443138.614	-0.011	258.8070
34	21	14	33	20	13	A	0	443151.203	-0.015	258.8199
34	21	13	33	20	14	A	0	443151.203	-0.015	258.8199
70	23	48	69	23	47	A	0	443536.778	-0.023	669.6800
70	23	47	69	23	46	A	0	443547.712	-0.074	669.6805
36	20	16	35	19	16	E	0	443771.597	-0.072	265.7011
36	20	17	35	19	17	E	0	443800.263	-0.073	265.6899
36	20	16	35	19	17	A	0	443818.392	-0.089	265.7021
36	20	17	35	19	16	A	0	443818.392	-0.089	265.7021
42	17	25	41	16	25	E	0	444206.278	-0.106	294.1801
42	17	26	41	16	26	E	0	444217.286	-0.074	294.1673
69	19	51	68	19	50	A	0	444235.838	-0.089	627.2031
69	19	51	68	19	50	E	0	444243.348	0.028	627.2019
42	17	26	41	16	25	A	0	444251.391	-0.113	294.1759
42	17	25	41	16	26	A	0	444252.958	-0.083	294.1758
38	19	19	37	18	19	E	0	444271.710	-0.102	273.8656
38	19	20	37	18	20	E	0	444295.342	-0.034	273.8537
38	19	20	37	18	19	A	0	444318.952	-0.072	273.8649
38	19	19	37	18	20	A	0	444318.952	-0.072	273.8649
40	18	22	39	17	22	E	0	444487.845	-0.098	283.3427
40	18	23	39	17	23	E	0	444505.633	-0.055	283.3302
40	18	23	39	17	22	A	0	444534.798	-0.048	283.3402
69	20	49	68	20	48	A	0	444688.687	-0.058	633.4706
69	20	49	68	20	48	E	0	444701.795	0.018	633.4684
61	16	46	60	15	45	E	0	444733.201	-0.025	498.1719
69	16	53	68	16	52	E	0	444869.603	-0.020	615.1257
69	16	53	68	16	52	A	0	444887.501	-0.025	615.1236
70	17	54	69	17	53	E	0	445025.736	-0.053	630.3277
70	17	54	69	17	53	A	0	445030.595	0.199	630.3275
70	22	49	69	22	48	A	0	445355.001	-0.209	661.9447
70	22	48	69	22	47	E	0	445431.005	-0.002	661.9455
48	15	34	47	14	33	E	0	445697.480	-0.058	340.2549
27	25	2	26	24	2	E	0	445958.126	-0.034	249.0673
52	15	38	51	14	37	E	0	445974.889	-0.036	383.3181
67	17	50	66	17	49	E	0	445981.373	-0.168	589.6364
67	17	50	66	17	49	A	0	445983.023	-0.035	589.6321
27	25	3	26	24	2	A	0	445993.420	-0.095	249.0758
27	25	2	26	24	3	A	0	445993.420	-0.095	249.0758
27	25	3	26	24	3	E	0	446003.173	-0.012	249.0623
47	15	32	46	14	33	A	0	446623.568	-0.072	330.0579
47	15	32	46	14	33	E	0	446630.150	-0.098	330.0594
29	24	5	28	23	5	E	0	446815.774	-0.052	251.4112
29	24	5	28	23	6	A	0	446854.507	-0.108	251.4184
29	24	6	28	23	5	A	0	446854.507	-0.108	251.4184
29	24	6	28	23	6	E	0	446858.783	-0.001	251.4047
70	40	30	70	39	31	A	0	446917.140	-0.095	867.8092
70	40	31	70	39	32	A	0	446917.140	-0.095	867.8092
70	21	50	69	21	49	A	0	447526.668	-0.059	654.7520
31	23	8	30	22	8	E	0	447656.144	-0.066	254.9806
31	23	9	30	22	9	E	0	447696.310	-0.165	254.9727
31	23	9	30	22	8	A	0	447697.872	-0.030	254.9863
31	23	8	30	22	9	A	0	447697.872	-0.030	254.9863
66	40	26	66	39	27	A	0	447872.931	-0.155	811.2790
66	40	27	66	39	28	A	0	447872.931	-0.155	811.2790
66	40	27	66	39	28	E	0	447917.068	0.009	811.2826
49	15	35	48	14	34	E	0	447930.703	-0.055	350.6042
65	40	25	65	39	26	A	0	448087.039	-0.121	797.6720
65	40	26	65	39	27	A	0	448087.039	-0.121	797.6720
70	21	49	69	21	48	A	0	448104.694	-0.221	654.7805
70	21	49	69	21	48	E	0	448108.689	0.020	654.7771
64	40	24	64	39	25	A	0	448291.741	-0.167	784.2748
64	40	25	64	39	26	A	0	448291.741	-0.167	784.2748
45	16	30	44	15	29	A	0	448425.824	-0.083	315.7313
45	16	29	44	15	29	E	0	448448.202	-0.170	315.7355
33	22	11	32	21	11	E	0	448461.346	-0.093	259.7845
45	16	30	44	15	30	E	0	448471.250	-0.062	315.7223
63	40	23	63	39	24	A	0	448487.493	-0.140	771.0872
63	40	24	63	39	25	A	0	448487.493	-0.140	771.0872
33	22	12	32	21	12	E	0	448498.369	-0.026	259.7754
33	22	11	32	21	12	A	0	448505.381	-0.078	259.7887
33	22	12	32	21	11	A	0	448505.381	-0.078	259.7887
45	16	29	44	15	30	A	0	448580.232	-0.068	315.7270
70	16	54	69	16	53	E	0	448634.476	-0.003	629.9650
62	40	22	62	39	23	A	0	448674.499	-0.128	758.1092
62	40	23	62	39	24	A	0	448674.499	-0.128	758.1092
62	40	22	62	39	23	E	0	448709.260	-0.129	758.1000
62	40	23	62	39	24	E	0	448718.972	0.181	758.1129
61	40	21	61	39	22	A	0	448852.984	-0.196	745.3405
61	40	22	61	39	23	A	0	448852.984	-0.196	745.3405
50	15	36	49	14	35	E	0	448873.803	-0.047	361.2267
60	40	20	60	39	21	A	0	449023.444	-0.125	732.7810
60	40	21	60	39	22	A	0	449023.444	-0.125	732.7810
59	40	19	59	39	20	A	0	449185.958	-0.112	720.4304
59	40	20	59	39	21	A	0	449185.958	-0.112	720.4304
35	21	14	34	20	14	E	0	449202.625	-0.068	265.8353
59	40	19	59	39	20	E	0	449221.193	-0.107	720.4212
59	40	20	59	39	21	E	0	449230.434	0.102	720.4342
35	21	15	34	20	15	E	0	449235.691	-0.044	265.8251
35	21	15	34	20	14	A	0	449248.332	-0.095	265.8379
35	21	14	34	20	15	A	0	449248.332	-0.095	265.8379
58	40	18	58	39	19	A	0	449340.788	-0.161	708.2888
58	40	19	58	39	20	A	0	449340.788	-0.161	708.2888
70	18	53	69	18	52	A	0	449352.248	0.024	636.2099
70	18	53	69	18	52	E	0	449352.248	-0.080	636.2099
58	40	18	58	39	19	E	0	449376.609	0.291	708.2796
56	40	16	56	39	17	A	0	449628.773	-0.107	684.6314
56	40	17	56	39	18	A	0	449628.773	-0.107	684.6314

J'	K_a'	K_c'	J''	K_a''	K_c''	Sym.	$v_{t_{CH_3}}$	$\nu_{Obs.}$	$\nu_{Obs.} - \nu_{Calc.}$	E_{low}
70	20	51	69	20	50	A	0	449737.152	0.041	648.1274
70	20	51	69	20	50	E	0	449747.645	-0.016	648.1249
55	40	15	55	39	16	A	0	449762.408	-0.027	673.1153
55	40	16	55	39	17	A	0	449762.408	-0.027	673.1153
37	20	17	36	19	17	E	0	449833.185	-0.108	273.1500
37	20	18	36	19	18	E	0	449861.800	-0.008	273.1388
37	20	17	36	19	18	A	0	449880.008	-0.087	273.1509
37	20	18	36	19	17	A	0	449880.008	-0.087	273.1509
54	40	14	54	39	15	A	0	449889.281	-0.095	661.8075
54	40	15	54	39	16	A	0	449889.281	-0.095	661.8075
43	17	26	42	16	26	E	0	449960.589	-0.048	302.9758
43	17	27	42	16	27	E	0	449970.995	-0.078	302.9632
43	17	27	42	16	26	A	0	450004.840	0.012	302.9716
43	17	26	42	16	27	A	0	450008.051	-0.087	302.9715
39	19	20	38	18	20	E	0	450276.060	-0.128	281.7523
39	19	21	38	18	21	E	0	450299.506	-0.021	281.7404
39	19	21	38	18	20	A	0	450323.287	-0.093	281.7515
39	19	20	38	18	21	A	0	450323.287	-0.094	281.7515
41	18	23	40	17	23	E	0	450399.189	-0.116	291.6768
41	18	24	40	17	24	E	0	450416.716	0.009	291.6644
41	18	24	40	17	23	A	0	450446.011	-0.152	291.6743
26	26	0	25	25	0	E	0	451236.783	-0.001	253.0692
26	26	0	25	25	1	A	0	451268.139	-0.099	253.0789
26	26	1	25	25	0	A	0	451268.139	-0.099	253.0789
26	26	1	25	25	1	E	0	451283.201	-0.027	253.0657
40	19	21	39	18	21	E	0	456261.885	-0.004	289.8533
42	18	24	41	17	24	E	0	456281.690	-0.057	300.2281
40	19	22	39	18	22	E	0	456284.941	-0.038	289.8414
42	18	25	41	17	25	E	0	456298.721	-0.052	300.2157
40	19	22	39	18	21	A	0	456308.924	-0.136	289.8525
40	19	21	39	18	22	A	0	456308.924	-0.137	289.8525
42	18	25	41	17	24	A	0	456328.422	-0.123	300.2255
42	18	24	41	17	25	A	0	456328.620	-0.023	300.2255
27	26	1	26	25	1	E	0	457378.947	-0.016	258.4027
27	26	1	26	25	2	A	0	457410.308	-0.119	258.4124
27	26	2	26	25	1	A	0	457410.308	-0.119	258.4124
27	26	2	26	25	2	E	0	457425.371	-0.024	258.3991
29	25	4	28	24	4	E	0	458239.414	-0.066	260.3588
29	25	5	28	24	4	A	0	458274.757	-0.097	260.3673
29	25	4	28	24	5	A	0	458274.757	-0.097	260.3673
29	25	5	28	24	5	E	0	458284.441	-0.022	260.3537
31	24	7	30	23	7	E	0	459087.679	-0.089	263.5376
31	24	7	30	23	8	A	0	459126.470	-0.102	263.5448
31	24	8	30	23	7	A	0	459126.470	-0.102	263.5448
31	24	8	30	23	8	E	0	459130.714	0.058	263.5311
47	16	32	46	15	32	E	0	459203.149	0.101	334.9644
33	23	10	32	22	10	E	0	459909.662	-0.105	267.9471
33	23	11	32	22	11	E	0	459949.783	-0.138	267.9392
33	23	11	32	22	10	A	0	459951.375	-0.093	267.9528
33	23	10	32	22	11	A	0	459951.375	-0.093	267.9528
28	27	1	27	26	1	E	0	474940.774	0.188	273.6592
28	27	2	27	26	1	A	0	474967.831	0.081	273.6700
28	27	1	27	26	2	A	0	474967.831	0.081	273.6700
28	27	2	27	26	2	E	0	474987.911	0.119	273.6572
30	26	4	29	25	4	E	0	475800.961	0.211	275.6440
30	26	4	29	25	5	A	0	475832.458	0.211	275.6537
30	26	5	29	25	4	A	0	475832.458	0.211	275.6537
30	26	5	29	25	5	E	0	475847.305	0.175	275.6404
32	25	7	31	24	7	E	0	476649.849	0.134	278.8512
32	25	8	31	24	7	A	0	476685.287	0.168	278.8596
32	25	7	31	24	8	A	0	476685.287	0.168	278.8596
32	25	8	31	24	8	E	0	476694.810	0.203	278.8460
34	24	10	33	23	10	E	0	477475.124	0.196	283.2880
34	24	10	33	23	11	A	0	477513.849	0.092	283.2951
34	24	11	33	23	10	A	0	477513.849	0.092	283.2951
34	24	11	33	23	11	E	0	477517.906	0.234	283.2815
48	17	32	47	16	31	A	0	477892.828	0.086	350.2909
48	17	31	47	16	31	E	0	477895.827	0.094	350.2943
48	17	32	47	16	32	E	0	477913.368	0.150	350.2818
36	23	13	35	22	13	E	0	478256.921	0.236	288.9644
36	23	14	35	22	14	E	0	478296.379	-0.246	288.9565
38	22	16	37	21	16	E	0	478964.174	0.032	295.8934
38	22	17	37	21	17	E	0	479000.940	0.321	295.8843
38	22	16	37	21	17	A	0	479008.228	0.056	295.8975
38	22	17	37	21	16	A	0	479008.228	0.056	295.8975
46	18	28	45	17	28	E	0	479461.748	0.019	336.6198
46	18	29	45	17	29	E	0	479477.129	0.284	336.6076
46	18	29	45	17	28	A	0	479507.378	0.092	336.6169
40	21	20	39	20	20	E	0	479581.121	0.187	304.0826
40	21	20	39	20	19	A	0	479594.435	0.128	304.0953
40	21	19	39	20	20	A	0	479594.435	0.128	304.0953
42	20	22	41	19	22	E	0	479931.363	-0.084	313.5874
42	20	23	41	19	23	E	0	479959.119	0.170	313.5763
42	20	22	41	19	23	A	0	479978.340	0.143	313.5881
42	20	23	41	19	22	A	0	479978.340	0.143	313.5881
44	19	25	43	18	25	E	0	479981.251	0.241	324.4123
44	19	26	43	18	26	E	0	480003.082	0.228	324.4005
44	19	25	43	18	26	A	0	480028.286	0.165	324.4111
29	27	2	28	26	2	E	0	481081.687	0.190	279.4043
29	27	3	28	26	2	A	0	481108.730	0.056	279.4150
29	27	2	28	26	3	A	0	481108.730	0.056	279.4150
29	27	3	28	26	3	E	0	481128.855	0.164	279.4023
31	26	5	30	25	5	E	0	481939.085	0.146	281.8053
31	26	5	30	25	6	A	0	481970.518	0.070	281.8150
31	26	6	30	25	5	A	0	481970.518	0.070	281.8150
31	26	6	30	25	6	E	0	481985.536	0.241	281.8018
33	25	8	32	24	8	E	0	482782.235	0.187	285.4308
33	25	9	32	24	8	A	0	482817.554	0.089	285.4393
33	25	8	32	24	9	A	0	482817.554	0.089	285.4393
33	25	9	32	24	9	E	0	482827.215	0.314	285.4257
49	17	33	48	16	32	A	0	483216.113	0.116	360.4326
49	17	32	48	16	32	E	0	483253.837	0.096	360.4353
49	17	33	48	16	33	E	0	483296.257	0.099	360.4220
49	17	32	48	16	33	A	0	483419.841	0.172	360.4269
35	24	11	34	23	11	E	0	483596.817	0.045	290.2887
35	24	11	34	23	12	A	0	483635.827	0.214	290.2957
35	24	12	34	23	11	A	0	483635.827	0.214	290.2957
35	24	12	34	23	12	E	0	483639.693	0.235	290.2821
37	23	14	36	22	14	E	0	484361.394	0.176	296.3894
37	23	15	36	22	15	E	0	484401.130	0.060	296.3814
37	23	15	36	22	14	A	0	484403.088	0.141	296.3950
37	23	14	36	22	15	A	0	484403.088	0.141	296.3950
39	22	17	38	21	17	E	0	485041.333	0.079	303.7470
39	22	18	38	21	18	E	0	485077.764	0.159	303.7379
39	22	17	38	21	18	A	0	485085.349	0.060	303.7510
39	22	18	38	21	17	A	0	485085.349	0.060	303.7510

J'	K'_a	K'_c	J''	K''_a	K''_c	Sym.	$v_{t_{CH_3}}$	$\nu_{Obs.}$	$\nu_{Obs.} - \nu_{Calc.}$	E_{low}
47	18	29	46	17	29	E	0	485151.239	0.238	346.2688
47	18	30	46	17	30	E	0	485165.606	0.072	346.2567
47	18	30	46	17	29	A	0	485195.281	0.014	346.2659
47	18	29	46	17	30	A	0	485200.451	0.272	346.2657
41	21	20	40	20	20	E	0	485583.277	0.223	312.3807
41	21	21	40	20	21	E	0	485615.560	0.341	312.3705
41	21	21	40	20	20	A	0	485628.823	0.051	312.3830
41	21	20	40	20	21	A	0	485628.823	0.051	312.3830
45	19	27	44	18	27	E	0	485865.772	0.088	333.5822
45	19	27	44	18	26	A	0	485891.165	-0.105	333.5928
45	19	26	44	18	27	A	0	485891.367	0.025	333.5928
43	20	23	42	19	23	E	0	485899.715	0.094	322.3170
43	20	24	42	19	24	E	0	485927.053	0.190	322.3059
28	28	1	27	27	0	E	0	486360.165	0.227	283.7583
28	28	0	27	27	1	A	0	486382.571	0.142	283.7700
28	28	1	27	27	0	A	0	486382.571	0.142	283.7700
28	28	0	27	27	1	E	0	486407.561	0.263	283.7579
52	16	36	51	15	37	E	0	487175.105	0.240	387.0830
52	16	36	51	15	37	A	0	487191.310	0.117	387.0820
30	27	3	29	26	3	E	0	487221.839	0.266	285.3561
30	27	4	29	26	3	A	0	487248.849	0.087	285.3669
30	27	3	29	26	4	A	0	487248.849	0.087	285.3669
30	27	4	29	26	4	E	0	487268.994	0.243	285.3541
32	26	6	31	25	6	E	0	488075.596	0.124	288.1741
32	26	6	31	25	7	A	0	488107.023	0.027	288.1838
32	26	7	31	25	6	A	0	488107.023	0.027	288.1838
32	26	7	31	25	7	E	0	488121.998	0.196	288.1705
50	17	34	49	16	33	A	0	488404.827	-0.070	370.8046
50	17	33	49	16	33	E	0	488491.239	0.295	370.8064
50	17	34	49	16	34	E	0	488607.442	0.038	370.7906
50	17	33	49	16	34	A	0	488780.686	0.242	370.7942
34	25	9	33	24	9	E	0	488911.704	0.116	292.2187
34	25	10	33	24	9	A	0	488947.105	0.087	292.2271
34	25	9	33	24	10	A	0	488947.105	0.087	292.2271
34	25	10	33	24	10	E	0	488956.565	0.167	292.2135
36	24	12	35	23	12	E	0	489714.437	0.188	297.4983
36	24	12	35	23	13	A	0	489753.246	0.145	297.5054
36	24	13	35	23	12	A	0	489753.246	0.145	297.5054
36	24	13	35	23	13	E	0	489757.104	0.236	297.4918
38	23	15	37	22	15	E	0	490459.434	0.240	304.0244
38	23	16	37	22	16	E	0	490498.897	-0.054	304.0165
38	23	16	37	22	15	A	0	490501.143	0.211	304.0300
38	23	15	37	22	16	A	0	490501.143	0.211	304.0300
48	18	31	47	17	31	E	0	490803.094	0.134	356.1282
48	18	31	47	17	30	A	0	490830.779	0.037	356.1373
48	18	30	47	17	31	A	0	490840.736	-0.038	356.1370
40	22	18	39	21	18	E	0	491108.891	0.161	311.8120
40	22	19	39	21	19	E	0	491145.175	0.230	311.8029
40	22	18	39	21	19	A	0	491152.897	0.126	311.8160
40	22	19	39	21	18	A	0	491152.897	0.126	311.8160
42	21	21	41	20	21	E	0	491603.527	0.085	320.8817
42	21	22	41	20	22	E	0	491635.584	0.173	320.8714
42	21	22	41	20	21	A	0	491649.181	0.021	320.8839
42	21	21	41	20	22	A	0	491649.181	0.021	320.8839
46	19	27	45	18	27	E	0	491676.122	0.254	342.9940
46	19	28	45	18	28	E	0	491696.993	0.090	342.9822
46	19	27	45	18	28	A	0	491723.090	0.074	342.9927
44	20	24	43	19	24	E	0	491847.137	0.110	331.2619
44	20	25	43	19	25	E	0	491874.022	0.036	331.2508
44	20	24	43	19	25	A	0	491893.939	0.175	331.2624
44	20	25	43	19	24	A	0	491893.939	0.176	331.2624
29	28	2	28	27	1	E	0	492501.032	0.230	289.5015
29	28	1	28	27	2	A	0	492523.371	0.065	289.5132
29	28	2	28	27	1	A	0	492523.371	0.065	289.5132
29	28	1	28	27	2	E	0	492548.401	0.248	289.5011
31	27	4	30	26	4	E	0	493360.902	0.318	291.5150
31	27	5	30	26	4	A	0	493387.867	0.079	291.5258
31	27	4	30	26	5	A	0	493387.867	0.079	291.5258
31	27	5	30	26	5	E	0	493407.766	0.022	291.5130
51	17	35	50	16	34	A	0	493417.367	0.033	381.4096
33	26	7	32	25	7	E	0	494210.187	0.132	294.7505
33	26	7	32	25	8	A	0	494241.721	0.128	294.7601
33	26	8	32	25	7	A	0	494241.721	0.128	294.7601
33	26	8	32	25	8	E	0	494256.628	0.274	294.7469
35	25	10	34	24	10	E	0	495038.058	0.107	299.2149
35	25	11	34	24	10	A	0	495073.430	0.033	299.2233
35	25	10	34	24	11	A	0	495073.430	0.033	299.2233
35	25	11	34	24	11	E	0	495082.905	0.192	299.2097
37	24	13	36	23	13	E	0	495827.287	0.427	304.9173
37	24	13	36	23	14	A	0	495865.850	0.125	304.9244
37	24	14	36	23	13	A	0	495865.850	0.125	304.9244
37	24	14	36	23	14	E	0	495869.509	0.102	304.9107
49	18	31	48	17	31	E	0	496370.225	0.104	366.2352
49	18	32	48	17	32	E	0	496383.727	0.092	366.2232
49	18	32	48	17	31	A	0	496406.975	-0.001	366.2322
49	18	31	48	17	32	A	0	496427.109	0.073	366.2317
39	23	16	38	22	16	E	0	496549.990	0.028	311.8699
39	23	17	38	22	17	E	0	496589.642	0.028	311.8620
39	23	17	38	22	16	A	0	496591.910	0.198	311.8755
39	23	16	38	22	17	A	0	496591.910	0.198	311.8755
41	22	19	40	21	19	E	0	497165.828	0.129	320.0889
41	22	20	40	21	20	E	0	497201.879	0.116	320.0797
41	22	19	40	21	20	A	0	497209.885	0.138	320.0928
41	22	20	40	21	19	A	0	497209.885	0.138	320.0928
47	19	28	46	18	28	E	0	497473.201	0.174	352.6129
47	19	29	46	18	29	E	0	497493.758	0.154	352.6012
47	19	28	46	18	29	A	0	497520.124	-0.139	352.6115
43	21	22	42	20	22	E	0	497608.599	0.060	329.5962
43	21	23	42	20	23	E	0	497640.505	0.211	329.5860
43	21	23	42	20	22	A	0	497654.345	0.086	329.5985
43	21	22	42	20	23	A	0	497654.345	0.086	329.5985
45	20	25	44	19	25	E	0	497771.968	0.094	340.4227
45	20	26	44	19	26	E	0	497799.142	0.618	340.4116
45	20	25	44	19	26	A	0	497818.715	0.107	340.4232
45	20	26	44	19	25	A	0	497818.715	0.111	340.4232
30	28	3	29	27	2	E	0	498641.158	0.107	295.4514
30	28	2	29	27	3	A	0	498663.631	0.060	295.4631
30	28	3	29	27	2	A	0	498663.631	0.060	295.4631
30	28	2	29	27	3	E	0	498688.698	0.305	295.4510
52	17	35	51	16	36	A	0	499390.689	0.133	392.2182
32	27	5	31	26	5	E	0	499498.417	0.127	297.8811
32	27	6	31	26	5	A	0	499525.601	0.091	297.8918
32	27	5	31	26	6	A	0	499525.601	0.091	297.8918
32	27	6	31	26	6	E	0	499545.624	0.195	297.8791
34	26	8	33	25	8	E	0	500342.520	0.145	301.5347
34	26	8	33	25	9	A	0	500374.062	0.133	301.5443

J'	K'_a	K'_c	J''	K''_a	K''_c	Sym.	$v_{t_{CH_3}}$	$\nu_{Obs.}$	$\nu_{Obs.} - \nu_{Calc.}$	E_{low}
34	26	9	33	25	8	A	0	500374.062	0.133	301.5443
34	26	9	33	25	9	E	0	500389.053	0.414	301.5311
36	25	11	35	24	11	E	0	501160.881	0.144	306.4197
36	25	12	35	24	11	A	0	501196.358	0.161	306.4281
36	25	11	35	24	12	A	0	501196.358	0.161	306.4281
36	25	12	35	24	12	E	0	501205.672	0.228	306.4146
50	18	32	49	17	32	E	0	501887.580	-0.026	376.5549
50	18	33	49	17	33	E	0	501901.393	-0.037	376.5430
50	18	33	49	17	32	A	0	501915.523	0.127	376.5521
38	24	14	37	23	14	E	0	501934.194	0.111	312.5459
38	24	14	37	23	15	A	0	501973.098	0.134	312.5529
38	24	15	37	23	14	A	0	501973.098	0.134	312.5529
38	24	15	37	23	15	E	0	501976.733	0.182	312.5393
40	23	17	39	22	17	E	0	502632.737	-0.101	319.9262
40	23	18	39	22	18	E	0	502672.388	0.014	319.9183
40	23	18	39	22	17	A	0	502674.716	0.117	319.9317
40	23	17	39	22	18	A	0	502674.716	0.117	319.9317
42	22	20	41	21	20	E	0	503211.347	0.110	328.5780
48	19	29	47	18	29	E	0	503232.715	0.224	362.4517
42	22	21	41	21	21	E	0	503247.284	0.145	328.5689
48	19	30	47	18	29	A	0	503279.385	0.238	362.4503
44	21	23	43	20	23	E	0	503597.371	0.321	338.5249
44	21	24	43	20	24	E	0	503628.789	0.215	338.5147
44	21	24	43	20	23	A	0	503642.879	0.104	338.5270
44	21	23	43	20	24	A	0	503642.879	0.104	338.5270
46	20	26	45	19	26	E	0	503672.350	0.123	349.8000
46	20	27	45	19	27	E	0	503698.590	0.048	349.7890
46	20	26	45	19	27	A	0	503719.010	0.048	349.8004
29	29	1	28	28	1	E	0	503919.398	0.040	299.9815
29	29	1	28	28	0	A	0	503936.883	-0.010	299.9939
29	29	0	28	28	1	A	0	503936.883	-0.010	299.9939
29	29	0	28	28	0	E	0	503966.597	0.364	299.9827
53	17	36	52	16	37	A	0	504710.571	-0.098	403.2767
53	17	36	52	16	37	E	0	504748.607	-0.198	403.2758
31	28	4	30	27	3	E	0	504780.625	0.134	301.6081
31	28	3	30	27	4	A	0	504803.162	0.137	301.6198
31	28	4	30	27	3	A	0	504803.162	0.137	301.6198
31	28	3	30	27	4	E	0	504828.077	0.258	301.6077
33	27	6	32	26	6	E	0	505634.620	0.184	304.4545
33	27	7	32	26	6	A	0	505661.753	0.081	304.4652
33	27	6	32	26	7	A	0	505661.753	0.081	304.4652
33	27	7	32	26	7	E	0	505681.720	0.170	304.4525
35	26	9	34	25	9	E	0	506472.278	0.175	308.5270
35	26	9	34	25	10	A	0	506503.747	0.073	308.5366
35	26	10	34	25	9	A	0	506503.747	0.073	308.5366
35	26	10	34	25	10	E	0	506518.495	0.168	308.5234
37	25	12	36	24	12	E	0	507279.520	0.001	313.8334
37	25	13	36	24	12	A	0	507315.129	0.132	313.8418
37	25	12	36	24	13	A	0	507315.129	0.132	313.8418
37	25	13	36	24	13	E	0	507324.391	0.224	313.8283
51	18	33	50	17	33	E	0	507333.209	0.261	387.1007
51	18	34	50	17	33	A	0	507345.009	0.348	387.0982
51	18	34	50	17	34	E	0	507349.840	-0.043	387.0888
39	24	15	38	23	15	E	0	508035.558	0.187	320.3844
39	24	15	38	23	16	A	0	508074.359	0.092	320.3914
39	24	16	38	23	15	A	0	508074.359	0.092	320.3914
39	24	16	38	23	16	E	0	508077.926	0.175	320.3778
41	23	18	40	22	18	E	0	508707.256	0.158	328.1937
41	23	19	40	22	19	E	0	508746.377	-0.133	328.1857
41	23	19	40	22	18	A	0	508749.035	0.163	328.1991
41	23	18	40	22	19	A	0	508749.035	0.163	328.1991
49	19	31	48	18	31	E	0	508970.174	-0.045	372.4996
43	22	21	42	21	21	E	0	509244.501	0.134	337.2798
43	22	22	42	21	22	E	0	509280.183	0.091	337.2706
43	22	21	42	21	22	A	0	509288.582	0.147	337.2836
43	22	22	42	21	21	A	0	509288.582	0.147	337.2836
47	20	27	46	19	27	E	0	509545.820	-0.168	359.3945
45	21	24	44	20	24	E	0	509567.668	0.074	347.6682
47	20	28	46	19	28	E	0	509571.834	-0.106	359.3835
47	20	27	46	19	28	A	0	509592.902	0.173	359.3948
45	21	25	44	20	25	E	0	509599.063	0.197	347.6580
45	21	25	44	20	24	A	0	509613.528	0.204	347.6703
45	21	24	44	20	25	A	0	509613.528	0.203	347.6703
55	17	39	54	16	38	E	0	509846.670	0.267	426.2441
55	17	39	54	16	38	A	0	509933.449	0.422	426.2433
30	29	2	29	28	2	E	0	510059.745	0.220	305.9296
30	29	2	29	28	1	A	0	510077.183	0.108	305.9420
30	29	1	29	28	2	A	0	510077.183	0.108	305.9420
30	29	1	29	28	1	E	0	510106.571	0.177	305.9307
54	17	37	53	16	38	A	0	510131.048	0.058	414.5669
54	17	37	53	16	38	E	0	510138.165	0.229	414.5665
32	28	5	31	27	4	E	0	510919.199	0.291	307.9718
32	28	4	31	27	5	A	0	510941.645	0.185	307.9834
32	28	5	31	27	4	A	0	510941.645	0.185	307.9834
32	28	4	31	27	5	E	0	510966.412	0.191	307.9713
59	17	43	58	16	42	E	0	511222.687	0.181	475.2956
59	17	43	58	16	42	A	0	511324.270	0.164	475.2926
34	27	7	33	26	7	E	0	511768.939	0.190	311.2356
34	27	8	33	26	7	A	0	511795.988	-0.016	311.2462
34	27	7	33	26	8	A	0	511795.988	-0.016	311.2462
34	27	8	33	26	8	E	0	511816.003	0.166	311.2335
56	17	40	55	16	39	E	0	512277.877	0.161	438.0889
56	17	40	55	16	39	A	0	512359.849	0.333	438.0877
36	26	10	35	25	10	E	0	512599.084	0.192	315.7276
36	26	10	35	25	11	A	0	512630.624	0.142	315.7371
36	26	11	35	25	10	A	0	512630.624	0.142	315.7371
36	26	11	35	25	11	E	0	512645.288	0.216	315.7239
52	18	35	51	17	34	A	0	512679.277	0.119	397.8722
52	18	34	51	17	34	E	0	512694.259	0.276	397.8742
52	18	35	51	17	35	E	0	512723.527	0.066	397.8619
52	18	34	51	17	35	A	0	512821.423	0.093	397.8683
58	17	42	57	16	41	E	0	513237.698	0.177	462.6064
38	25	14	37	24	13	A	0	513429.473	0.124	321.4647
38	25	13	37	24	14	A	0	513429.473	0.124	321.4647
38	25	14	37	24	14	E	0	513438.501	0.066	321.4512
40	24	16	39	23	16	E	0	514130.329	0.184	328.4331
40	24	16	39	23	17	A	0	514169.252	0.193	328.4400
40	24	17	39	23	16	A	0	514169.252	0.193	328.4400
40	24	17	39	23	17	E	0	514172.667	0.237	328.4265
50	19	31	49	18	31	E	0	514623.849	0.244	382.7923
50	19	32	49	18	32	E	0	514642.667	0.114	382.7808
50	19	32	49	18	31	A	0	514669.151	0.216	382.7907
50	19	31	49	18	32	A	0	514672.498	0.110	382.7906
42	23	19	41	22	19	E	0	514772.128	0.149	336.6726
42	23	20	41	22	20	E	0	514811.477	0.221	336.6646
42	23	20	41	22	19	A	0	514813.949	0.178	336.6780

J'	K_a'	K_c'	J''	K_a''	K_c''	Sym.	$v_{t_{CH_3}}$	$\nu_{Obs.}$	$\nu_{Obs.} - \nu_{Calc.}$	E_{low}
42	23	19	41	22	20	A	0	514813.949	0.178	336.6780
44	22	22	43	21	22	E	0	515264.106	0.053	346.1947
44	22	23	43	21	23	E	0	515299.750	0.163	346.1855
44	22	22	43	21	23	A	0	515308.250	0.117	346.1984
44	22	23	43	21	22	A	0	515308.250	0.117	346.1984
48	20	28	47	19	28	E	0	515391.029	0.156	369.2068
48	20	29	47	19	29	E	0	515416.503	0.072	369.1958
48	20	28	47	19	29	A	0	515437.777	0.144	369.2070
46	21	25	45	20	25	E	0	515518.928	0.238	357.0266
46	21	26	45	20	26	E	0	515549.819	0.128	357.0164
46	21	26	45	20	25	A	0	515564.483	0.055	357.0286
46	21	25	45	20	26	A	0	515564.483	0.054	357.0286
55	17	38	54	16	39	E	0	515757.171	0.218	426.0885
55	17	38	54	16	39	A	0	515768.594	0.331	426.0885
31	29	3	30	28	3	E	0	516199.295	0.209	312.0843
31	29	3	30	28	2	A	0	516216.781	0.129	312.0967
31	29	2	30	28	3	A	0	516216.781	0.129	312.0967
31	29	2	30	28	2	E	0	516246.135	0.189	312.0854
33	28	6	32	27	5	E	0	517056.301	0.219	314.5426
33	28	5	32	27	6	A	0	517078.763	0.111	314.5542
33	28	6	32	27	5	A	0	517078.763	0.111	314.5542
33	28	5	32	27	6	E	0	517103.575	0.199	314.5421
53	18	36	52	17	35	A	0	517896.778	0.097	408.8761
35	27	8	34	26	8	E	0	517901.100	0.154	318.2243
35	27	9	34	26	8	A	0	517928.371	0.152	318.2350
35	27	8	34	26	9	A	0	517928.371	0.152	318.2350
35	27	9	34	26	9	E	0	517948.064	0.062	318.2223
53	18	35	52	17	35	E	0	517950.820	-0.004	408.8773
53	18	36	52	17	36	E	0	518020.941	0.108	408.8636
53	18	35	52	17	36	A	0	518159.235	-0.007	408.8688
37	26	11	36	25	11	E	0	518722.510	0.133	323.1366
37	26	11	36	25	12	A	0	518754.116	0.128	323.1462
37	26	12	36	25	11	A	0	518754.116	0.128	323.1462
37	26	12	36	25	12	E	0	518768.497	-0.011	323.1330
39	25	14	38	24	14	E	0	519503.538	0.265	329.2886
39	25	15	38	24	14	A	0	519538.872	0.084	329.2969
39	25	14	38	24	15	A	0	519538.872	0.084	329.2969
39	25	15	38	24	15	E	0	519547.805	0.024	329.2835
41	24	17	40	23	17	E	0	520217.947	0.147	336.6923
51	19	32	50	18	32	E	0	520246.844	0.110	393.2961
41	24	17	40	23	18	A	0	520257.030	0.296	336.6991
41	24	18	40	23	17	A	0	520257.030	0.296	336.6991
41	24	18	40	23	18	E	0	520260.096	0.116	336.6856
51	19	33	50	18	32	A	0	520290.283	-0.030	393.2944
51	19	32	50	18	33	A	0	520297.460	0.129	393.2942
43	23	20	42	22	20	E	0	520826.635	-0.047	345.3633
43	23	21	42	22	21	E	0	520865.900	0.089	345.3554
43	23	21	42	22	20	A	0	520868.618	0.128	345.3687
43	23	20	42	22	21	A	0	520868.618	0.128	345.3687
49	20	29	48	19	29	E	0	521204.547	0.162	379.2377
49	20	29	48	19	30	A	0	521251.259	0.074	379.2378
45	22	23	44	21	23	E	0	521269.498	0.303	355.3231
45	22	24	44	21	24	E	0	521304.747	0.226	355.3139
45	22	23	44	21	24	A	0	521313.437	0.147	355.3268
45	22	24	44	21	23	A	0	521313.437	0.147	355.3268
30	30	1	29	29	1	E	0	521477.380	0.156	316.7905
47	21	27	46	20	27	E	0	521479.756	0.299	366.5905
30	30	0	29	29	1	A	0	521489.655	0.083	316.8035
30	30	1	29	29	0	A	0	521489.655	0.083	316.8035
47	21	27	46	20	26	A	0	521494.605	0.106	366.6026
47	21	26	46	20	27	A	0	521494.605	0.105	366.6026
30	30	0	29	29	0	E	0	521523.202	0.210	316.7932
56	17	39	55	16	40	E	0	521775.470	0.047	437.8408
56	17	39	55	16	40	A	0	521795.503	0.386	437.8405
32	29	4	31	28	4	E	0	522338.089	0.234	318.4458
32	29	4	31	28	3	A	0	522355.526	0.086	318.4582
32	29	3	31	28	4	A	0	522355.526	0.086	318.4582
32	29	3	31	28	3	E	0	522384.920	0.214	318.4469
54	18	37	53	17	36	A	0	522965.010	0.150	420.1121
54	18	36	53	17	36	E	0	523070.882	0.021	420.1125
34	28	7	33	27	6	E	0	523192.018	0.242	321.3207
34	28	6	33	27	7	A	0	523214.441	0.075	321.3323
34	28	7	33	27	6	A	0	523214.441	0.075	321.3323
34	28	6	33	27	7	E	0	523239.196	0.146	321.3202
54	18	37	53	17	37	E	0	523248.516	-0.247	420.0951
54	18	36	53	17	37	A	0	523440.565	0.116	420.0990
36	27	9	35	26	9	E	0	524030.983	0.258	325.4211
36	27	10	35	26	9	A	0	524058.131	0.111	325.4317
36	27	9	35	26	10	A	0	524058.131	0.111	325.4317
36	27	10	35	26	10	E	0	524077.998	0.252	325.4190
38	26	12	37	25	12	E	0	524842.419	0.244	330.7545
38	26	12	37	25	13	A	0	524873.901	0.093	330.7640
38	26	13	37	25	12	A	0	524873.901	0.093	330.7640
38	26	13	37	25	13	E	0	524888.443	0.190	330.7508
40	25	15	39	24	15	E	0	525607.472	0.187	337.3306
40	25	16	39	24	15	A	0	525642.936	0.112	337.3389
40	25	15	39	24	16	A	0	525642.936	0.112	337.3389
40	25	16	39	24	16	E	0	525651.856	0.142	337.3254
52	19	33	51	18	33	E	0	525814.923	0.009	404.0235
52	19	34	51	18	34	E	0	525832.736	0.045	404.0121
52	19	34	51	18	33	A	0	525854.830	-0.274	404.0218
52	19	33	51	18	34	A	0	525869.366	0.273	404.0214
42	24	18	41	23	19	A	0	526336.735	0.082	345.1691
42	24	19	41	23	18	A	0	526336.735	0.082	345.1691
42	24	19	41	23	19	E	0	526339.896	0.132	345.1557
44	23	22	43	22	22	E	0	526909.295	-0.030	354.2584
44	23	22	43	22	21	A	0	526912.323	0.139	354.2716
44	23	21	43	22	22	A	0	526912.323	0.139	354.2716
50	20	30	49	19	30	E	0	526983.888	0.102	389.4880
50	20	31	49	19	31	E	0	527008.507	0.050	389.4770
50	20	30	49	19	31	A	0	527030.711	0.043	389.4880
46	22	24	45	21	24	E	0	527258.737	0.113	364.6655
46	22	25	45	21	25	E	0	527293.918	0.192	364.6564
46	22	24	45	21	25	A	0	527302.813	0.077	364.6691
46	22	25	45	21	24	A	0	527302.813	0.077	364.6691
48	21	27	47	20	27	E	0	527356.126	0.061	376.3911
48	21	28	47	20	28	E	0	527386.584	0.130	376.3810
48	21	28	47	20	27	A	0	527401.957	0.131	376.3930
48	21	27	47	20	28	A	0	527401.957	0.128	376.3930
31	30	2	30	29	2	E	0	527616.726	0.057	322.9434
31	30	1	30	29	2	A	0	527629.291	0.258	322.9564
31	30	2	30	29	1	A	0	527629.291	0.258	322.9564
55	18	38	54	17	37	E	0	527646.703	0.217	431.5829
31	30	1	30	29	1	E	0	527662.626	0.194	322.9461
55	18	38	54	17	37	A	0	527835.974	0.076	431.5831
55	18	37	54	17	37	E	0	528010.219	-0.150	431.5829

J'	K'_a	K'_c	J''	K''_a	K''_c	Sym.	$v_{t_{CH_3}}$	$\nu_{Obs.}$	$\nu_{Obs.} - \nu_{Calc.}$	E_{low}
55	18	38	54	17	38	E	0	528418.921	-0.031	431.5571
57	17	40	56	16	41	E	0	528433.439	-0.197	449.8214
57	17	40	56	16	41	A	0	528454.565	-0.089	449.8209
33	29	5	32	28	5	E	0	528475.723	0.082	325.0142
33	29	5	32	28	4	A	0	528493.330	0.086	325.0266
33	29	4	32	28	5	A	0	528493.330	0.086	325.0266
33	29	4	32	28	4	E	0	528522.666	0.188	325.0153
55	18	37	54	17	38	A	0	528680.833	0.052	431.5600
35	28	8	34	27	7	E	0	529325.886	0.144	328.3063
35	28	7	34	27	8	A	0	529348.465	0.112	328.3179
35	28	8	34	27	7	A	0	529348.465	0.112	328.3179
35	28	7	34	27	8	E	0	529373.212	0.221	328.3058
37	27	10	36	26	10	E	0	530157.898	0.128	332.8260
37	27	11	36	26	10	A	0	530185.176	0.088	332.8366
37	27	10	36	26	11	A	0	530185.176	0.088	332.8366
37	27	11	36	26	11	E	0	530205.008	0.256	332.8239
39	26	13	38	25	13	E	0	530957.818	-0.067	338.5813
39	26	13	38	25	14	A	0	530989.700	0.158	338.5908
39	26	14	38	25	13	A	0	530989.700	0.158	338.5908
39	26	14	38	25	14	E	0	531004.177	0.275	338.5776
53	19	34	52	18	34	E	0	531322.340	0.207	414.9758
53	19	35	52	18	35	E	0	531339.788	0.090	414.9645
53	19	35	52	18	34	A	0	531356.072	0.118	414.9741
53	19	34	52	18	35	A	0	531383.546	0.235	414.9734
41	25	16	40	24	16	E	0	531705.479	0.105	345.5826
41	25	17	40	24	16	A	0	531741.068	0.130	345.5908
41	25	16	40	24	17	A	0	531741.068	0.130	345.5908
41	25	17	40	24	17	E	0	531749.834	0.116	345.5774
56	18	39	55	17	38	E	0	532293.714	0.281	443.2923
43	24	19	42	23	19	E	0	532369.337	0.171	353.8435
43	24	19	42	23	20	A	0	532408.061	-0.085	353.8503
43	24	20	42	23	19	A	0	532408.061	-0.085	353.8503
43	24	20	42	23	20	E	0	532411.261	0.151	353.8369
56	18	39	55	17	38	A	0	532439.416	0.339	443.2927
51	20	31	50	19	31	E	0	532726.141	0.085	399.9583
51	20	32	50	19	32	E	0	532750.334	0.106	399.9474
51	20	31	50	19	32	A	0	532773.004	-0.101	399.9582
45	23	22	44	22	22	E	0	532902.211	0.105	363.3820
45	23	23	44	22	23	E	0	532941.222	0.319	363.3740
45	23	23	44	22	22	A	0	532944.073	0.116	363.3872
45	23	22	44	22	23	A	0	532944.073	0.116	363.3872
47	22	25	46	21	25	E	0	533231.171	0.077	374.2224
49	21	28	48	20	28	E	0	533238.924	0.136	386.3984
47	22	26	46	21	26	E	0	533266.028	0.073	374.2133
47	22	25	46	21	26	A	0	533275.382	0.157	374.2260
47	22	26	46	21	25	A	0	533275.382	0.157	374.2260
49	21	28	48	20	29	A	0	533284.637	0.066	386.4002
32	30	3	31	29	3	E	0	533755.670	0.158	329.3028
32	30	2	31	29	3	A	0	533767.963	0.068	329.3158
32	30	3	31	29	2	A	0	533767.963	0.068	329.3158
32	30	2	31	29	2	E	0	533801.444	0.176	329.3055
34	29	6	33	28	6	E	0	534612.474	0.238	331.7897
34	29	6	33	28	5	A	0	534629.956	0.096	331.8021
34	29	5	33	28	6	A	0	534629.956	0.096	331.8021
36	28	9	35	27	8	E	0	535457.935	0.217	335.4996
36	28	8	35	27	9	A	0	535480.474	0.122	335.5112
36	28	9	35	27	8	A	0	535480.474	0.122	335.5112
36	28	8	35	27	9	E	0	535505.106	0.165	335.4991
58	17	41	57	16	42	E	0	536055.028	0.240	462.0274
38	27	11	37	26	11	E	0	536281.889	0.138	340.4394
38	27	12	37	26	11	A	0	536309.192	0.098	340.4500
38	27	11	37	26	12	A	0	536309.192	0.098	340.4500
38	27	12	37	26	12	E	0	536328.929	0.240	340.4372
57	18	40	56	17	39	E	0	536559.010	-0.119	455.2454
57	18	40	56	17	39	A	0	536670.864	0.055	455.2458
54	19	35	53	18	35	E	0	536761.175	0.091	426.1543
54	19	36	53	18	36	E	0	536780.054	0.223	426.1429
54	19	36	53	18	35	A	0	536783.529	0.126	426.1527
54	19	35	53	18	36	A	0	536835.990	0.094	426.1512
40	26	14	39	25	14	E	0	537069.217	0.133	346.6174
40	26	14	39	25	15	A	0	537100.879	0.111	346.6269
40	26	15	39	25	14	A	0	537100.879	0.111	346.6269
40	26	15	39	25	15	E	0	537115.229	0.193	346.6137
64	18	47	63	17	46	E	0	537708.447	0.175	546.3899
42	25	17	41	24	17	E	0	537797.184	0.181	354.0449
42	25	18	41	24	17	A	0	537832.715	0.122	354.0530
42	25	17	41	24	18	A	0	537832.715	0.122	354.0530
42	25	18	41	24	18	E	0	537841.417	0.165	354.0396
52	20	32	51	19	32	E	0	538427.807	-0.042	410.6496
44	24	20	43	23	20	E	0	538431.570	0.067	362.7362
52	20	33	51	19	33	E	0	538451.687	0.207	410.6388
44	24	20	43	23	21	A	0	538470.628	0.119	362.7430
44	24	21	43	23	20	A	0	538470.628	0.119	362.7430
46	23	23	45	22	23	E	0	538921.141	0.155	372.7107
46	23	24	45	22	24	E	0	538959.780	0.183	372.7028
46	23	24	45	22	23	A	0	538963.012	0.150	372.7159
46	23	23	45	22	24	A	0	538963.012	0.150	372.7159
31	31	1	30	30	1	E	0	539033.663	0.218	334.1851
31	31	1	30	30	0	A	0	539040.569	0.124	334.1985
31	31	0	30	30	1	A	0	539040.569	0.124	334.1985
31	31	0	30	30	0	E	0	539077.605	0.108	334.1893
50	21	29	49	20	29	E	0	539095.215	0.291	396.6233
50	21	30	49	20	30	E	0	539124.681	0.079	396.6131
50	21	29	49	20	30	A	0	539140.733	-0.001	396.6249
57	18	39	56	17	40	A	0	539183.333	-0.227	455.1782
48	22	26	47	21	26	E	0	539185.593	0.317	383.9944
57	18	39	56	17	40	E	0	539203.118	-0.379	455.1767
48	22	27	47	21	27	E	0	539219.888	0.012	383.9852
48	22	26	47	21	27	A	0	539229.536	0.105	383.9978
48	22	27	47	21	26	A	0	539229.536	0.105	383.9978
33	30	4	32	29	4	E	0	539893.783	0.200	335.8691
33	30	3	32	29	4	A	0	539906.117	0.132	335.8821
33	30	4	32	29	3	A	0	539906.117	0.132	335.8821
33	30	3	32	29	3	E	0	539939.499	0.168	335.8718
58	18	41	57	17	40	E	0	540292.191	0.090	467.4481
58	18	41	57	17	40	A	0	540382.997	0.157	467.4483
35	29	7	34	28	7	E	0	540747.635	0.213	338.7725
35	29	7	34	28	6	A	0	540765.181	0.112	338.7849
35	29	6	34	28	7	A	0	540765.181	0.112	338.7849
35	29	6	34	28	6	E	0	540794.447	0.220	338.7736
37	28	10	36	27	9	E	0	541587.600	0.171	342.9009
37	28	9	36	27	10	A	0	541610.218	0.129	342.9124
37	28	10	36	27	9	A	0	541610.218	0.129	342.9124
37	28	9	36	27	10	E	0	541634.770	0.149	342.9003
55	19	36	54	18	36	E	0	542122.165	0.067	437.5602
55	19	37	54	18	36	A	0	542124.783	-0.000	437.5591

J'	K'_a	K'_c	J''	K''_a	K''_c	Sym.	$v_{t_{CH_3}}$	$\nu_{Obs.}$	$\nu_{Obs.} - \nu_{Calc.}$	E_{low}
55	19	37	54	18	37	E	0	542147.140	0.252	437.5488
55	19	36	54	18	37	A	0	542223.772	0.112	437.5563
63	18	46	62	17	45	E	0	542340.796	0.075	532.5139
39	27	12	38	26	12	E	0	542402.456	0.137	348.2613
39	27	13	38	26	12	A	0	542429.933	0.243	348.2719
39	27	12	38	26	13	A	0	542429.933	0.243	348.2719
39	27	13	38	26	13	E	0	542449.424	0.215	348.2592
41	26	15	40	25	15	E	0	543175.444	0.112	354.8630
41	26	15	40	25	16	A	0	543207.094	0.050	354.8724
41	26	16	40	25	15	A	0	543207.094	0.050	354.8724
41	26	16	40	25	16	E	0	543221.331	0.118	354.8593
43	25	19	42	24	18	A	0	543917.337	0.114	362.7258
43	25	18	42	24	19	A	0	543917.337	0.114	362.7258
43	25	19	42	24	19	E	0	543925.960	0.211	362.7125
53	20	34	52	19	34	E	0	544108.416	-0.066	421.5520
53	20	34	52	19	33	A	0	544131.143	0.089	421.5625
53	20	33	52	19	34	A	0	544133.634	0.163	421.5624
45	24	21	44	23	21	E	0	544484.112	0.148	371.8409
45	24	21	44	23	22	A	0	544523.065	0.066	371.8475
45	24	22	44	23	21	A	0	544523.065	0.066	371.8475
45	24	22	44	23	22	E	0	544525.780	0.150	371.8342
58	18	40	57	17	41	E	0	544580.802	0.130	467.3353
58	18	40	57	17	41	A	0	544584.727	0.168	467.3363
51	21	30	50	20	30	E	0	544922.307	0.002	407.0663
47	23	24	46	22	24	E	0	544926.253	0.258	382.2530
51	21	31	50	20	31	E	0	544951.710	0.126	407.0562
47	23	25	46	22	25	E	0	544964.505	0.100	382.2450
51	21	31	50	20	30	A	0	544968.123	0.011	407.0678
47	23	25	46	22	24	A	0	544968.123	0.223	382.2581
47	23	24	46	22	25	A	0	544968.123	0.223	382.2581
49	22	27	48	21	27	E	0	545119.482	-0.270	393.9818
49	22	28	48	21	28	E	0	545154.244	0.173	393.9727
49	22	27	48	21	28	A	0	545164.093	0.161	393.9852
49	22	28	48	21	27	A	0	545164.093	0.161	393.9852
32	31	2	31	30	2	E	0	545172.378	0.236	340.5427
32	31	2	31	30	1	A	0	545179.147	-0.014	340.5562
32	31	1	31	30	2	A	0	545179.147	-0.014	340.5562
32	31	1	31	30	1	E	0	545216.467	0.276	340.5470
60	18	43	59	17	42	E	0	545292.357	0.342	492.6351
60	18	43	59	17	42	A	0	545372.352	0.019	492.6345
34	30	5	33	29	5	E	0	546030.781	0.081	342.6422
34	30	4	33	29	5	A	0	546043.236	0.113	342.6552
34	30	5	33	29	4	A	0	546043.236	0.113	342.6552
34	30	4	33	29	4	E	0	546076.813	0.375	342.6449
36	29	8	35	28	8	E	0	546881.114	0.143	345.9627
36	29	8	35	28	7	A	0	546898.812	0.170	345.9751
36	29	7	35	28	8	A	0	546898.812	0.170	345.9751
36	29	7	35	28	7	E	0	546927.983	0.228	345.9638
56	19	38	55	18	37	A	0	547362.475	-0.053	449.1948
56	19	37	55	18	37	E	0	547390.782	0.205	449.1954
56	19	38	55	18	38	E	0	547436.622	-0.034	449.1833
56	19	37	55	18	38	A	0	547545.656	0.271	449.1898
38	28	11	37	27	10	E	0	547714.729	0.143	350.5102
38	28	10	37	27	11	A	0	547737.330	0.057	350.5217
38	28	11	37	27	10	A	0	547737.330	0.057	350.5217
38	28	10	37	27	11	E	0	547761.833	0.090	350.5096
40	27	13	39	26	13	E	0	548519.166	0.057	356.2922
40	27	14	39	26	13	A	0	548546.438	-0.071	356.3027
40	27	13	39	26	14	A	0	548546.438	-0.071	356.3027
40	27	14	39	26	14	E	0	548565.994	0.049	356.2900
42	26	16	41	25	16	E	0	549276.225	0.060	363.3184
42	26	16	41	25	17	A	0	549308.042	0.133	363.3278
42	26	17	41	25	16	A	0	549308.042	0.133	363.3278
42	26	17	41	25	17	E	0	549322.195	0.228	363.3147
44	25	20	43	24	19	A	0	549994.284	0.053	371.6095
44	25	19	43	24	20	A	0	549994.284	0.053	371.6095
44	25	20	43	24	20	E	0	550002.484	-0.129	371.5962
46	24	22	45	23	22	E	0	550526.018	0.249	381.1577
46	24	22	45	23	23	A	0	550564.922	0.085	381.1643
46	24	23	45	23	22	A	0	550564.922	0.085	381.1643
46	24	23	45	23	23	E	0	550567.373	0.093	381.1510
52	21	31	51	20	31	E	0	550718.899	0.332	417.7282
52	21	32	51	20	32	E	0	550747.619	0.203	417.7181
52	21	31	51	20	32	A	0	550764.467	0.001	417.7296
48	23	25	47	22	25	E	0	550915.982	-0.087	392.0091
48	23	26	47	22	26	E	0	550954.305	0.041	392.0011
48	23	26	47	22	25	A	0	550958.161	0.155	392.0141
48	23	25	47	22	26	A	0	550958.161	0.155	392.0141
50	22	28	49	21	28	E	0	551033.199	0.202	404.1854
50	22	29	49	21	29	E	0	551067.050	0.035	404.1762
50	22	28	49	21	29	A	0	551077.234	0.025	404.1886
50	22	29	49	21	28	A	0	551077.234	0.026	404.1886
33	31	3	32	30	3	E	0	551310.412	0.172	347.1070
33	31	3	32	30	2	A	0	551317.395	0.116	347.1204
33	31	2	32	30	3	A	0	551317.395	0.116	347.1204
33	31	2	32	30	2	E	0	551354.545	0.259	347.1112
35	30	6	34	29	6	E	0	552166.832	0.160	349.6224
35	30	5	34	29	6	A	0	552179.314	0.195	349.6354
35	30	6	34	29	5	A	0	552179.314	0.195	349.6354
35	30	5	34	29	5	E	0	552212.494	0.095	349.6251
57	19	38	56	18	39	A	0	552803.605	0.026	461.0530
37	29	9	36	28	9	E	0	553012.848	0.207	353.3606
37	29	9	36	28	8	A	0	553030.370	0.032	353.3729
37	29	8	36	28	9	A	0	553030.370	0.032	353.3729
37	29	8	36	28	8	E	0	553059.640	0.239	353.3617
39	28	12	38	27	11	E	0	553839.079	0.193	358.3278
39	28	11	38	27	12	A	0	553861.666	0.063	358.3393
39	28	12	38	27	11	A	0	553861.666	0.063	358.3393
39	28	11	38	27	12	E	0	553886.178	0.174	358.3272
41	27	14	40	26	14	E	0	554631.902	0.163	364.5321
41	27	15	40	26	14	A	0	554659.273	0.101	364.5426
41	27	14	40	26	15	A	0	554659.273	0.101	364.5426
41	27	15	40	26	15	E	0	554678.671	0.154	364.5299
55	20	35	54	19	35	E	0	555250.598	-0.077	444.0587
55	20	36	54	19	36	E	0	555272.722	0.232	444.0480
55	20	36	54	19	35	A	0	555292.748	-0.034	444.0582
55	20	35	54	19	36	A	0	555302.518	0.014	444.0579
43	26	17	42	25	17	E	0	555371.250	0.149	371.9838
43	26	18	42	25	18	E	0	555416.869	0.050	371.9801
45	25	20	44	24	20	E	0	556027.538	0.230	380.6964
45	25	21	44	24	20	A	0	556063.089	0.094	380.7044
45	25	20	44	24	21	A	0	556063.089	0.094	380.7044
45	25	21	44	24	21	E	0	556071.322	0.100	380.6911
53	21	32	52	20	32	E	0	556481.240	0.117	428.6097
53	21	33	52	20	33	E	0	556509.442	-0.069	428.5996
53	21	32	52	20	33	A	0	556527.212	0.104	428.6110

J'	K_a'	K_c'	J''	K_a''	K_c''	Sym.	$v_{t_{CH_3}}$	$\nu_{Obs.}$	$\nu_{Obs.} - \nu_{Calc.}$	E_{low}
47	24	23	46	23	23	E	0	556556.284	0.190	390.6872
32	32	1	31	31	1	E	0	556587.627	-0.297	352.1653
32	32	0	31	31	1	A	0	556589.782	0.295	352.1789
32	32	1	31	31	0	A	0	556589.782	0.295	352.1789
47	24	23	46	23	24	A	0	556595.291	0.093	390.6938
47	24	24	46	23	23	A	0	556595.291	0.093	390.6938
47	24	24	46	23	24	E	0	556597.702	0.264	390.6805
32	32	0	31	31	0	E	0	556629.828	0.152	352.1710
49	23	26	48	22	26	E	0	556890.064	-0.017	401.9797
51	22	30	50	21	30	E	0	556957.233	0.161	414.5964
51	22	29	50	21	30	A	0	556967.724	0.099	414.6087
51	22	30	50	21	29	A	0	556967.724	0.101	414.6087
58	19	40	57	18	39	A	0	557415.276	0.119	473.1634
34	31	4	33	30	4	E	0	557447.824	0.244	353.8780
34	31	4	33	30	3	A	0	557454.799	0.158	353.8914
34	31	3	33	30	4	A	0	557454.799	0.158	353.8914
34	31	3	33	30	3	E	0	557491.817	0.197	353.8822
58	19	39	57	18	40	A	0	558007.342	0.110	473.1472
36	30	7	35	29	7	E	0	558301.642	0.344	356.8099
36	30	6	35	29	7	A	0	558313.800	0.030	356.8228
36	30	7	35	29	6	A	0	558313.800	0.030	356.8228
36	30	6	35	29	6	E	0	558347.077	0.067	356.8125
38	29	10	37	28	10	E	0	559142.288	0.109	360.9663
38	29	10	37	28	9	A	0	559160.070	0.164	360.9786
38	29	9	37	28	10	A	0	559160.070	0.164	360.9786
38	29	9	37	28	9	E	0	559189.007	0.094	360.9673
40	28	13	39	27	12	E	0	559960.012	0.000	366.3539
40	28	12	39	27	13	A	0	559982.860	0.100	366.3654
40	28	13	39	27	12	A	0	559982.860	0.100	366.3654
40	28	12	39	27	13	E	0	560007.214	0.127	366.3534
42	27	15	41	26	15	E	0	560739.992	0.179	372.9814
56	20	36	55	19	36	E	0	560748.316	0.179	455.6435
42	27	16	41	26	15	A	0	560767.376	0.096	372.9919
42	27	15	41	26	16	A	0	560767.376	0.096	372.9919
56	20	37	55	19	37	E	0	560769.818	0.330	455.6328
42	27	16	41	26	16	E	0	560786.380	-0.146	372.9792
56	20	36	55	19	37	A	0	560804.977	0.177	455.6424
44	26	18	43	25	18	E	0	561459.752	0.121	380.8596
44	26	18	43	25	19	A	0	561491.580	0.134	380.8690
44	26	19	43	25	18	A	0	561491.580	0.134	380.8690
44	26	19	43	25	19	E	0	561505.308	0.052	380.8559
46	25	21	45	24	21	E	0	562087.183	0.048	390.0029
46	25	22	45	24	21	A	0	562122.966	0.105	390.0109
46	25	21	45	24	22	A	0	562122.966	0.105	390.0109
46	25	22	45	24	22	E	0	562130.898	-0.021	389.9976
59	19	41	58	18	40	A	0	562139.828	0.347	485.5017
54	21	33	53	20	33	E	0	562207.334	0.203	439.7116
54	21	34	53	20	34	E	0	562235.142	0.121	439.7015
54	21	33	53	20	34	A	0	562253.253	-0.011	439.7128
48	24	24	47	23	24	E	0	562574.075	0.003	400.4297
48	24	24	47	23	25	A	0	562613.219	0.004	400.4362
48	24	25	47	23	24	A	0	562613.219	0.004	400.4362
48	24	25	47	23	25	E	0	562615.504	0.268	400.4230
33	32	2	32	31	2	E	0	562725.588	-0.260	358.7277
33	32	1	32	31	2	A	0	562727.712	0.281	358.7414
33	32	2	32	31	1	A	0	562727.712	0.281	358.7414
33	32	1	32	31	1	E	0	562767.598	-0.001	358.7334
52	22	30	51	21	30	E	0	562789.136	0.000	425.2429
52	22	31	51	21	31	E	0	562822.655	0.173	425.2338
52	22	30	51	21	31	A	0	562833.569	0.146	425.2460
52	22	31	51	21	30	A	0	562833.569	0.151	425.2460
50	23	27	49	22	27	E	0	562846.912	0.079	412.1651
50	23	28	49	22	28	E	0	562884.549	0.003	412.1571
50	23	28	49	22	27	A	0	562888.921	0.078	412.1699
50	23	27	49	22	28	A	0	562888.921	0.078	412.1699
35	31	5	34	30	5	E	0	563584.110	0.115	360.8559
35	31	5	34	30	4	A	0	563591.184	0.106	360.8693
35	31	4	34	30	5	A	0	563591.184	0.106	360.8693
35	31	4	34	30	4	E	0	563628.159	0.131	360.8601
37	30	8	36	29	8	E	0	564434.525	0.160	364.2047
37	30	7	36	29	8	A	0	564446.913	0.047	364.2177
37	30	8	36	29	7	A	0	564446.913	0.047	364.2177
37	30	7	36	29	7	E	0	564480.164	0.103	364.2074
39	29	11	38	28	11	E	0	565269.363	0.044	368.7800
39	29	11	38	28	10	A	0	565287.234	0.157	368.7923
39	29	10	38	28	11	A	0	565287.234	0.157	368.7923
39	29	10	38	28	10	E	0	565316.176	0.152	368.7810
41	28	14	40	27	13	E	0	566077.815	0.185	374.5888
41	28	13	40	27	14	A	0	566100.550	0.135	374.6003
41	28	14	40	27	13	A	0	566100.550	0.135	374.6003
41	28	13	40	27	14	E	0	566124.692	0.032	374.5882
57	20	37	56	19	37	E	0	566180.602	0.034	467.4544
57	20	38	56	19	38	E	0	566202.283	0.144	467.4438
57	20	38	56	19	37	A	0	566210.269	0.115	467.4539
60	19	42	59	18	41	E	0	566443.160	0.090	498.0798
60	19	42	59	18	41	A	0	566565.108	-0.038	498.0811
43	27	16	42	26	16	E	0	566843.032	0.122	381.6403
43	27	17	42	26	16	A	0	566870.487	0.071	381.6507
43	27	16	42	26	17	A	0	566870.487	0.071	381.6507
43	27	17	42	26	17	E	0	566889.788	0.234	381.6381
45	26	19	44	25	19	E	0	567541.356	0.130	389.9461
45	26	19	44	25	20	A	0	567573.185	0.104	389.9554
45	26	20	44	25	19	A	0	567573.185	0.104	389.9554
45	26	20	44	25	20	E	0	567586.955	0.204	389.9423
55	21	34	54	20	34	E	0	567893.664	0.213	451.0347
55	21	35	54	20	35	E	0	567920.864	0.060	451.0247
55	21	34	54	20	35	A	0	567939.562	-0.287	451.0357
47	25	22	46	24	22	E	0	568137.588	0.216	399.5213
47	25	23	46	24	22	A	0	568173.281	0.140	399.5292
47	25	22	46	24	23	A	0	568173.281	0.140	399.5292
47	25	23	46	24	23	E	0	568181.072	0.055	399.5160
60	19	41	59	18	42	A	0	568346.094	0.071	498.0330
60	19	41	59	18	42	E	0	568380.253	0.000	498.0304
49	24	25	48	23	25	E	0	568578.867	0.081	410.3857
49	24	25	48	23	26	A	0	568617.935	-0.037	410.3921
49	24	26	48	23	25	A	0	568617.935	-0.037	410.3921
49	24	26	48	23	26	E	0	568620.176	0.420	410.3790
53	22	31	52	21	31	E	0	568628.415	0.049	436.0981
53	22	32	52	21	32	E	0	568661.216	-0.123	436.0890
53	22	31	52	21	32	A	0	568672.839	0.138	436.1011
53	22	32	52	21	31	A	0	568672.839	0.150	436.1011
51	23	28	50	22	28	E	0	568785.234	0.188	422.5658
51	23	29	50	22	29	E	0	568822.600	0.108	422.5578
51	23	29	50	22	28	A	0	568827.179	0.080	422.5706
51	23	28	50	22	29	A	0	568827.179	0.080	422.5706
34	32	3	33	31	3	E	0	568862.912	-0.262	365.4967

J'	K'_a	K'_c	J''	K''_a	K''_c	Sym.	$v_{t_{CH_3}}$	$\nu_{Obs.}$	$\nu_{Obs.} - \nu_{Calc.}$	E_{low}
34	32	2	33	31	3	A	0	568865.096	0.318	365.5104
34	32	3	33	31	2	A	0	568865.096	0.318	365.5104
34	32	2	33	31	2	E	0	568905.110	0.185	365.5024
36	31	6	35	30	6	E	0	569719.477	0.173	368.0407
36	31	6	35	30	5	A	0	569726.498	0.086	368.0541
36	31	5	35	30	6	A	0	569726.498	0.086	368.0541
36	31	5	35	30	5	E	0	569763.532	0.205	368.0449
38	30	9	37	29	9	E	0	570565.798	0.147	371.8071
38	30	8	37	29	9	A	0	570578.236	0.054	371.8200
38	30	9	37	29	8	A	0	570578.236	0.054	371.8200
38	30	8	37	29	8	E	0	570611.509	0.181	371.8097
40	29	12	39	28	12	E	0	571393.921	0.136	376.8019
40	29	12	39	28	11	A	0	571411.704	0.127	376.8141
40	29	11	39	28	12	A	0	571411.704	0.127	376.8141
40	29	11	39	28	11	E	0	571440.514	0.058	376.8029
58	20	38	57	19	38	E	0	571540.028	0.120	479.4928
58	20	39	57	19	38	A	0	571555.537	0.223	479.4925
58	20	39	57	19	39	E	0	571564.251	0.069	479.4821
58	20	38	57	19	39	A	0	571624.026	0.171	479.4906
42	28	15	41	27	14	E	0	572191.641	0.245	383.0326
42	28	14	41	27	15	A	0	572214.347	0.129	383.0441
42	28	15	41	27	14	A	0	572214.347	0.129	383.0441
42	28	14	41	27	15	E	0	572238.495	0.121	383.0320
44	27	17	43	26	17	E	0	572940.700	0.105	390.5090
44	27	18	43	26	17	A	0	572968.278	0.136	390.5195
44	27	17	43	26	18	A	0	572968.278	0.136	390.5195
44	27	18	43	26	18	E	0	572987.425	0.261	390.5068
56	21	35	55	20	35	E	0	573536.694	0.096	462.5798
56	21	36	55	20	36	E	0	573563.435	0.060	462.5699
61	19	42	60	18	43	A	0	573578.477	0.006	510.8262
46	26	20	45	25	20	E	0	573615.466	0.138	399.2435
46	26	20	45	25	21	A	0	573647.317	0.090	399.2527
46	26	21	45	25	20	A	0	573647.317	0.090	399.2527
46	26	21	45	25	21	E	0	573660.849	0.104	399.2397
62	19	44	61	18	43	E	0	573929.863	0.366	523.9838
62	19	44	61	18	43	A	0	574011.521	0.093	523.9846
33	33	1	32	32	0	A	0	574136.822	0.150	370.7448
33	33	0	32	32	1	A	0	574136.822	0.150	370.7448
33	33	1	32	32	1	E	0	574140.802	0.237	370.7311
48	25	23	47	24	23	E	0	574177.336	0.037	409.2519
33	33	0	32	32	0	E	0	574179.675	0.213	370.7382
48	25	24	47	24	23	A	0	574213.259	0.146	409.2598
48	25	23	47	24	24	A	0	574213.259	0.146	409.2598
48	25	24	47	24	24	E	0	574220.949	0.156	409.2466
54	22	32	53	21	32	E	0	574439.147	0.139	447.1719
54	22	33	53	21	33	E	0	574471.637	0.057	447.1628
54	22	32	53	21	33	A	0	574483.516	0.114	447.1747
50	24	26	49	23	26	E	0	574569.115	-0.152	420.5555
52	23	29	51	22	29	E	0	574703.403	0.047	433.1825
52	23	30	51	22	30	E	0	574740.805	0.290	433.1745
52	23	30	51	22	29	A	0	574745.533	0.075	433.1871
52	23	29	51	22	30	A	0	574745.533	0.075	433.1871
35	32	4	34	31	4	E	0	574999.513	-0.243	372.4724
35	32	3	34	31	4	A	0	575001.685	0.303	372.4861
35	32	4	34	31	3	A	0	575001.685	0.303	372.4861
35	32	3	34	31	3	E	0	575041.643	0.140	372.4781
37	31	7	36	30	7	E	0	575853.581	0.261	375.4328
37	31	7	36	30	6	A	0	575860.560	0.103	375.4462
37	31	6	36	30	7	A	0	575860.560	0.103	375.4462
37	31	6	36	30	6	E	0	575897.496	0.163	375.4370
63	19	45	62	18	44	E	0	576570.262	0.115	537.3233
39	30	10	38	29	10	E	0	576695.061	0.138	379.6172
39	30	9	38	29	10	A	0	576707.606	0.120	379.6302
39	30	10	38	29	9	A	0	576707.606	0.120	379.6302
39	30	9	38	29	9	E	0	576740.781	0.203	379.6199
59	20	39	58	19	39	E	0	576815.182	0.231	491.7601
59	20	40	58	19	40	E	0	576850.230	0.211	491.7492
59	20	39	58	19	40	A	0	576934.184	0.089	491.7568
41	29	13	40	28	13	E	0	577515.450	0.164	385.0322
41	29	13	40	28	12	A	0	577533.041	-0.074	385.0444
41	29	12	40	28	13	A	0	577533.041	-0.074	385.0444
41	29	12	40	28	12	E	0	577561.995	0.075	385.0332
64	19	46	63	18	45	A	0	578203.774	0.167	550.9337
43	28	16	42	27	15	E	0	578301.129	0.183	391.6856
43	28	15	42	27	16	A	0	578323.938	0.130	391.6971
43	28	16	42	27	15	A	0	578323.938	0.130	391.6971
43	28	15	42	27	16	E	0	578348.030	0.162	391.6850
62	19	43	61	18	44	E	0	578958.057	-0.141	523.8516
62	19	43	61	18	44	A	0	578969.934	-0.062	523.8532
45	27	18	44	26	18	E	0	579032.471	0.058	399.5879
45	27	19	44	26	18	A	0	579060.098	0.094	399.5983
45	27	18	44	26	19	A	0	579060.098	0.094	399.5983
45	27	19	44	26	19	E	0	579078.942	0.043	399.5857
57	21	36	56	20	36	E	0	579132.728	0.050	474.3480
57	21	37	56	20	37	E	0	579158.858	0.018	474.3381
57	21	37	56	20	36	A	0	579177.202	0.098	474.3488
57	21	36	56	20	37	A	0	579180.577	0.075	474.3487
47	26	21	46	25	21	E	0	579681.491	0.134	408.7521
47	26	21	46	25	22	A	0	579713.458	0.155	408.7613
47	26	22	46	25	21	A	0	579713.458	0.155	408.7613
47	26	22	46	25	22	E	0	579726.720	0.062	408.7483
49	25	24	48	24	24	E	0	580206.226	0.073	419.1952
55	22	33	54	21	33	E	0	580219.230	0.408	458.4648
49	25	25	48	24	24	A	0	580242.138	0.121	419.2030
49	25	24	48	24	25	A	0	580242.138	0.121	419.2030
55	22	33	54	21	34	A	0	580263.341	0.053	458.4675
34	33	2	33	32	1	A	0	580273.964	0.146	377.5119
34	33	1	33	32	2	A	0	580273.964	0.146	377.5119
34	33	2	33	32	2	E	0	580277.852	0.163	377.4982
34	33	1	33	32	1	E	0	580316.743	0.154	377.5053
53	23	30	52	22	30	E	0	580600.160	-0.145	444.0155
53	23	31	52	22	31	E	0	580636.911	-0.247	444.0075
53	23	31	52	22	30	A	0	580642.579	0.120	444.0201
53	23	30	52	22	31	A	0	580642.579	0.119	444.0201
36	32	5	35	31	5	E	0	581135.213	-0.220	379.6550
36	32	4	35	31	5	A	0	581137.353	0.268	379.6686
36	32	5	35	31	4	A	0	581137.353	0.268	379.6686
36	32	4	35	31	4	E	0	581177.301	0.125	379.6607
38	31	8	37	30	8	E	0	581985.876	0.028	383.0322
38	31	8	37	30	7	A	0	581993.175	0.160	383.0456
38	31	7	37	30	8	A	0	581993.175	0.160	383.0456
38	31	7	37	30	7	E	0	582029.973	0.124	383.0364
60	20	40	59	19	41	A	0	582177.241	0.225	504.2527
40	30	11	39	29	11	E	0	582822.127	0.191	387.6353
40	30	10	39	29	11	A	0	582834.680	0.147	387.6482
40	30	11	39	29	10	A	0	582834.680	0.147	387.6482

J'	K_a'	K_c'	J''	K_a''	K_c''	Sym.	$v_{t_{CH_3}}$	$\nu_{Obs.}$	$\nu_{Obs.} - \nu_{Calc.}$	E_{low}
40	30	10	39	29	10	E	0	582867.798	0.233	387.6379
42	29	14	41	28	14	E	0	583633.588	0.071	393.4711
42	29	14	41	28	13	A	0	583651.479	0.093	393.4833
42	29	13	41	28	14	A	0	583651.479	0.093	393.4833
42	29	13	41	28	13	E	0	583680.279	0.168	393.4721
44	28	17	43	27	16	E	0	584406.070	0.170	400.5481
44	28	16	43	27	17	A	0	584428.891	0.083	400.5595
44	28	17	43	27	16	A	0	584428.891	0.083	400.5595
44	28	16	43	27	17	E	0	584452.924	0.164	400.5475
63	19	44	62	18	45	E	0	584645.997	0.217	537.1121
58	21	37	57	20	37	E	0	584677.379	0.071	486.3401
58	21	38	57	20	37	A	0	584720.269	0.100	486.3408
58	21	37	57	20	38	A	0	584726.730	-0.173	486.3407
46	27	19	45	26	19	E	0	585117.922	0.036	408.8772
46	27	20	45	26	19	A	0	585145.660	0.135	408.8876
46	27	19	45	26	20	A	0	585145.660	0.135	408.8876
46	27	20	45	26	20	E	0	585164.395	0.111	408.8750
48	26	22	47	25	22	E	0	585738.841	0.137	418.4723
48	26	22	47	25	23	A	0	585770.774	0.073	418.4814
48	26	23	47	25	22	A	0	585770.774	0.073	418.4814
48	26	23	47	25	23	E	0	585784.020	0.141	418.4685
56	22	34	55	21	34	E	0	585965.468	0.107	469.9776
56	22	35	55	21	35	E	0	585997.254	0.212	469.9685
56	22	35	55	21	34	A	0	586009.819	0.024	469.9802
50	25	25	49	24	25	E	0	586223.421	0.283	429.3514
50	25	26	49	24	25	A	0	586259.189	0.133	429.3592
50	25	25	49	24	26	A	0	586259.189	0.133	429.3592
50	25	26	49	24	26	E	0	586266.334	0.036	429.3461
35	33	3	34	32	2	A	0	586410.491	0.121	384.4857
35	33	2	34	32	3	A	0	586410.491	0.121	384.4857
35	33	3	34	32	3	E	0	586414.488	0.270	384.4720
35	33	2	34	32	2	E	0	586453.342	0.223	384.4791
54	23	31	53	22	31	E	0	586474.293	-0.036	455.0656
52	24	28	51	23	28	E	0	586503.437	0.078	441.5385
54	23	32	53	22	32	E	0	586510.942	0.088	455.0575
54	23	32	53	22	31	A	0	586516.664	0.124	455.0700
54	23	31	53	22	32	A	0	586516.664	0.122	455.0700
61	20	42	60	19	41	A	0	586943.733	0.108	516.9910
61	20	41	60	19	41	E	0	587031.143	0.250	516.9896
37	32	6	36	31	6	E	0	587269.804	-0.235	387.0445
37	32	5	36	31	6	A	0	587271.959	0.240	387.0582
37	32	6	36	31	5	A	0	587271.959	0.240	387.0582
37	32	5	36	31	5	E	0	587311.941	0.164	387.0502
61	20	41	60	19	42	A	0	587357.053	0.248	516.9796
39	31	9	38	30	9	E	0	588116.734	0.054	390.8391
39	31	9	38	30	8	A	0	588123.954	0.073	390.8525
39	31	8	38	30	9	A	0	588123.954	0.073	390.8525
39	31	8	38	30	8	E	0	588160.782	0.115	390.8433
41	30	12	40	29	12	E	0	588946.593	0.160	395.8615
41	30	11	40	29	12	A	0	588959.256	0.187	395.8744
41	30	12	40	29	11	A	0	588959.256	0.187	395.8744
41	30	11	40	29	11	E	0	588992.163	0.128	395.8641
43	29	15	42	28	15	E	0	589748.377	0.215	402.1189
43	29	15	42	28	14	A	0	589766.123	0.049	402.1311
43	29	14	42	28	15	A	0	589766.123	0.049	402.1311
43	29	14	42	28	14	E	0	589794.856	0.145	402.1198
59	21	38	58	20	38	E	0	590165.801	0.300	498.5573
59	21	39	58	20	39	E	0	590190.578	0.128	498.5475
59	21	39	58	20	38	A	0	590205.547	0.208	498.5579
59	21	38	58	20	39	A	0	590218.994	0.534	498.5576
45	28	18	44	27	17	E	0	590505.916	0.049	409.6203
45	28	17	44	27	18	A	0	590528.919	0.098	409.6316
45	28	18	44	27	17	A	0	590528.919	0.098	409.6316
45	28	17	44	27	18	E	0	590552.756	0.097	409.6196
47	27	20	46	26	20	E	0	591196.385	-0.132	418.3772
47	27	21	46	26	20	A	0	591224.320	0.112	418.3875
47	27	20	46	26	21	A	0	591224.320	0.112	418.3875
47	27	21	46	26	21	E	0	591243.111	0.293	418.3749
57	22	35	56	21	35	E	0	591675.886	-0.056	481.7110
34	34	0	33	33	1	A	0	591682.129	0.155	389.8959
34	34	1	33	33	0	A	0	591682.129	0.155	389.8959
34	34	1	33	33	1	E	0	591691.652	0.388	389.8823
57	22	35	56	21	36	A	0	591720.647	-0.005	481.7134
34	34	0	33	33	0	E	0	591726.850	0.057	389.8907
49	26	23	48	25	23	E	0	591786.867	0.139	428.4044
49	26	23	48	25	24	A	0	591818.879	0.098	428.4135
49	26	24	48	25	23	A	0	591818.879	0.098	428.4135
49	26	24	48	25	24	E	0	591831.898	0.128	428.4006
51	25	26	50	24	26	E	0	592227.555	0.146	439.7211
51	25	27	50	24	26	A	0	592263.470	0.084	439.7287
51	25	26	50	24	27	A	0	592263.470	0.084	439.7287
51	25	27	50	24	27	E	0	592270.502	0.117	439.7157
55	23	32	54	22	32	E	0	592323.913	0.168	466.3331
55	23	33	54	22	33	E	0	592360.089	0.172	466.3251
55	23	33	54	22	32	A	0	592366.104	0.085	466.3374
55	23	32	54	22	33	A	0	592366.104	0.081	466.3374
53	24	29	52	23	29	E	0	592444.736	0.008	452.3525
62	20	42	61	19	43	A	0	592484.601	-0.548	529.9389
53	24	29	52	23	30	A	0	592484.601	0.473	452.3586
53	24	30	52	23	29	A	0	592484.601	0.473	452.3586
53	24	30	52	23	30	E	0	592484.601	-0.175	452.3457
36	33	4	35	32	3	A	0	592546.298	0.111	391.6661
36	33	3	35	32	4	A	0	592546.298	0.111	391.6661
36	33	4	35	32	4	E	0	592550.171	0.162	391.6524
36	33	3	35	32	3	E	0	592589.036	0.126	391.6595
38	32	7	37	31	7	E	0	593403.179	-0.223	394.6412
38	32	6	37	31	7	A	0	593405.348	0.236	394.6548
38	32	7	37	31	6	A	0	593405.348	0.236	394.6548
38	32	6	37	31	6	E	0	593445.185	0.053	394.6469
40	31	10	39	30	10	E	0	594245.775	0.173	398.8537
40	31	10	39	30	9	A	0	594252.982	0.145	398.8671
40	31	9	39	30	10	A	0	594252.982	0.145	398.8671
40	31	9	39	30	9	E	0	594289.677	0.107	398.8579
42	30	13	41	29	13	E	0	595068.300	0.152	404.2960
42	30	12	41	29	13	A	0	595080.886	0.060	404.3089
42	30	13	41	29	12	A	0	595080.886	0.060	404.3089
42	30	12	41	29	12	E	0	595113.861	0.141	404.2986
60	21	39	59	20	39	E	0	595591.691	0.216	511.0006
60	21	40	59	20	40	E	0	595616.552	0.382	510.9908
60	21	40	59	20	39	A	0	595626.040	0.354	511.0012
60	21	39	59	20	40	A	0	595650.752	-0.074	511.0005
44	29	16	43	28	16	E	0	595859.048	0.157	410.9757
44	29	16	43	28	15	A	0	595876.925	0.076	410.9879
44	29	15	43	28	16	A	0	595876.925	0.076	410.9879
44	29	15	43	28	15	E	0	595905.511	0.120	410.9766
63	20	44	62	19	43	A	0	596330.879	0.186	543.1656

J'	K'_a	K'_c	J''	K''_a	K''_c	Sym.	$v_{t_{CH_3}}$	$\nu_{Obs.}$	$\nu_{Obs.} - \nu_{Calc.}$	E_{low}
46	28	19	45	27	18	E	0	596600.505	0.075	418.9024
46	28	18	45	27	19	A	0	596623.537	0.101	418.9137
46	28	19	45	27	18	A	0	596623.537	0.101	418.9137
46	28	18	45	27	19	E	0	596647.298	0.148	418.9017
48	27	21	47	26	21	E	0	597267.864	0.080	428.0882
48	27	22	47	26	21	A	0	597295.675	0.143	428.0984
48	27	21	47	26	22	A	0	597295.675	0.143	428.0984
48	27	22	47	26	22	E	0	597314.093	0.110	428.0859
58	22	36	57	21	37	A	0	597392.409	-0.149	493.6681
63	20	43	62	19	44	A	0	597585.834	0.027	543.1316
63	20	43	62	19	44	E	0	597634.251	0.170	543.1280
35	34	1	34	33	2	A	0	597818.448	0.152	396.8678
35	34	2	34	33	1	A	0	597818.448	0.152	396.8678
50	26	24	49	25	24	E	0	597824.914	0.153	438.5488
35	34	2	34	33	2	E	0	597827.705	0.140	396.8542
50	26	24	49	25	25	A	0	597856.949	0.076	438.5578
50	26	25	49	25	24	A	0	597856.949	0.076	438.5578
35	34	1	34	33	1	E	0	597862.832	-0.266	396.8626
50	26	25	49	25	25	E	0	597869.877	0.219	438.5449
56	23	34	55	22	34	E	0	598182.581	0.044	477.8108
56	23	34	55	22	33	A	0	598189.286	0.205	477.8230
56	23	33	55	22	34	A	0	598189.286	0.196	477.8230
52	25	27	51	24	27	E	0	598218.155	0.074	450.3045
52	25	28	51	24	27	A	0	598254.176	0.057	450.3121
52	25	27	51	24	28	A	0	598254.176	0.057	450.3121
52	25	28	51	24	28	E	0	598261.000	0.142	450.2991
54	24	30	53	23	30	E	0	598367.515	0.150	463.3823
54	24	30	53	23	31	A	0	598406.963	0.131	463.3882
54	24	31	53	23	30	A	0	598406.963	0.132	463.3882
54	24	31	53	23	31	E	0	598406.963	-0.179	463.3755
37	33	5	36	32	4	A	0	598681.233	0.112	399.0533
37	33	4	36	32	5	A	0	598681.233	0.112	399.0533
37	33	5	36	32	5	E	0	598685.136	0.219	399.0396
37	33	4	36	32	4	E	0	598724.000	0.182	399.0467
39	32	8	38	31	8	E	0	599535.146	-0.190	402.4452
39	32	7	38	31	8	A	0	599537.292	0.213	402.4588
39	32	8	38	31	7	A	0	599537.292	0.213	402.4588
39	32	7	38	31	7	E	0	599577.136	0.078	402.4508
41	31	11	40	30	11	E	0	600372.569	0.184	407.0762
41	31	11	40	30	10	A	0	600379.782	0.122	407.0895
41	31	10	40	30	11	A	0	600379.782	0.122	407.0895
41	31	10	40	30	10	E	0	600416.459	0.124	407.0803
64	20	45	63	19	44	E	0	600469.166	0.032	556.6138
64	20	45	63	19	44	A	0	600571.135	0.026	556.6158
61	21	40	60	20	41	A	0	601020.036	0.239	523.6707
43	30	14	42	29	14	E	0	601186.941	0.137	412.9390
43	30	13	42	29	14	A	0	601199.666	0.141	412.9519
43	30	14	42	29	13	A	0	601199.666	0.141	412.9519
43	30	13	42	29	13	E	0	601232.530	0.189	412.9416
45	29	17	44	28	17	E	0	601965.963	0.606	420.0418
45	29	17	44	28	16	A	0	601983.485	0.119	420.0539
45	29	16	44	28	17	A	0	601983.485	0.119	420.0539
45	29	16	44	28	16	E	0	602011.876	0.072	420.0427
47	28	20	46	27	19	E	0	602689.132	-0.029	428.3946
64	20	44	63	19	45	A	0	602701.204	0.109	556.5586
47	28	19	46	27	20	A	0	602712.411	0.188	428.4059
47	28	20	46	27	19	A	0	602712.411	0.188	428.4059
64	20	44	63	19	45	E	0	602717.207	-0.117	556.5556
47	28	19	46	27	20	E	0	602735.968	0.164	428.3939
59	22	37	58	21	37	E	0	602977.080	-0.042	505.8429
59	22	38	58	21	38	E	0	603007.335	0.123	505.8338
49	27	22	48	26	22	E	0	603331.338	0.192	438.0104
49	27	23	48	26	22	A	0	603359.053	0.100	438.0206
49	27	22	48	26	23	A	0	603359.053	0.100	438.0206
49	27	23	48	26	23	E	0	603377.293	0.060	438.0081
51	26	25	50	25	25	E	0	603852.189	0.091	448.9057
51	26	25	50	25	26	A	0	603884.162	-0.113	448.9147
51	26	26	50	25	25	A	0	603884.162	-0.113	448.9147
51	26	26	50	25	26	E	0	603896.887	0.047	448.9018
57	23	34	56	22	34	E	0	603941.480	0.132	489.5233
36	34	2	35	33	3	A	0	603954.164	0.141	404.0462
36	34	3	35	33	2	A	0	603954.164	0.141	404.0462
36	34	3	35	33	3	E	0	603963.499	0.230	404.0326
57	23	35	56	22	35	E	0	603976.776	0.030	489.5152
57	23	34	56	22	35	A	0	603983.902	0.119	489.5273
36	34	2	35	33	2	E	0	603999.038	0.232	404.0410
53	25	28	52	24	28	E	0	604194.355	0.141	461.1021
53	25	29	52	24	28	A	0	604230.428	0.107	461.1096
53	25	28	52	24	29	A	0	604230.428	0.107	461.1096
53	25	29	52	24	29	E	0	604236.843	0.065	461.0967
55	24	31	54	23	31	E	0	604269.679	-0.280	474.6282
55	24	31	54	23	32	A	0	604309.561	0.063	474.6341
55	24	32	54	23	31	A	0	604309.561	0.063	474.6341
65	20	46	64	19	45	A	0	604352.376	0.015	570.3149
38	33	6	37	32	5	A	0	604815.087	0.068	406.6474
38	33	5	37	32	6	A	0	604815.087	0.068	406.6474
38	33	6	37	32	6	E	0	604818.986	0.201	406.6338
38	33	5	37	32	5	E	0	604857.859	0.176	406.6408
40	32	9	39	31	9	E	0	605665.449	-0.202	410.4566
40	32	8	39	31	9	A	0	605667.673	0.243	410.4702
40	32	9	39	31	8	A	0	605667.673	0.243	410.4702
40	32	8	39	31	8	E	0	605707.506	0.144	410.4622
62	21	41	61	20	41	E	0	606227.255	0.428	536.5708
62	21	42	61	20	41	A	0	606234.258	0.240	536.5717
62	21	42	61	20	42	E	0	606257.849	0.075	536.5608
62	21	41	61	20	42	A	0	606321.896	0.072	536.5693
42	31	12	41	30	12	E	0	606496.918	0.122	415.5066
42	31	12	41	30	11	A	0	606504.234	0.122	415.5199
42	31	11	41	30	12	A	0	606504.234	0.122	415.5199
42	31	11	41	30	11	E	0	606540.868	0.146	415.5107
44	30	15	43	29	15	E	0	607302.218	0.111	421.7907
44	30	14	43	29	15	A	0	607314.983	0.105	421.8036
44	30	15	43	29	14	A	0	607314.983	0.105	421.8036
44	30	14	43	29	14	E	0	607347.577	-0.030	421.7933
66	20	47	65	19	46	A	0	607496.239	0.491	584.2700
46	29	18	45	28	18	E	0	608067.277	0.075	429.3174
46	29	18	45	28	17	A	0	608085.368	0.102	429.3295
46	29	17	45	28	18	A	0	608085.368	0.102	429.3295
46	29	17	45	28	17	E	0	608113.693	0.101	429.3183
60	22	38	59	21	38	E	0	608560.892	0.070	518.2431
60	22	39	59	21	39	E	0	608590.243	-0.064	518.2341
60	22	39	59	21	38	A	0	608604.798	0.301	518.2451
60	22	38	59	21	39	A	0	608607.233	0.384	518.2451
48	28	21	47	27	20	E	0	608771.693	0.082	438.0974
48	28	20	47	27	21	A	0	608794.661	-0.073	438.1086
48	28	21	47	27	20	A	0	608794.661	-0.073	438.1086

J'	K'_a	K'_c	J''	K''_a	K''_c	Sym.	$v_{t_{CH_3}}$	$\nu_{Obs.}$	$\nu_{Obs.} - \nu_{Calc.}$	E_{low}
48	28	20	47	27	21	E	0	608818.291	0.121	438.0967
35	35	1	34	34	0	A	0	609225.493	0.134	409.6323
35	35	0	34	34	1	A	0	609225.493	0.134	409.6323
35	35	1	34	34	1	E	0	609240.104	0.182	409.6190
35	35	0	34	34	0	E	0	609271.796	0.185	409.6286
50	27	23	49	26	23	E	0	609386.034	0.004	448.1443
50	27	24	49	26	23	A	0	609413.981	0.078	448.1544
50	27	23	49	26	24	A	0	609413.981	0.078	448.1544
50	27	24	49	26	24	E	0	609432.233	0.235	448.1419
58	23	36	57	22	36	E	0	609740.466	0.048	501.4391
58	23	35	57	22	36	A	0	609748.143	0.164	501.4511
52	26	26	51	25	26	E	0	609868.019	0.017	459.4757
52	26	26	51	25	27	A	0	609900.301	0.052	459.4845
52	26	27	51	25	26	A	0	609900.301	0.052	459.4845
52	26	27	51	25	27	E	0	609912.669	0.089	459.4717
37	34	3	36	33	4	A	0	610089.149	0.125	411.4313
37	34	4	36	33	3	A	0	610089.149	0.125	411.4313
37	34	4	36	33	4	E	0	610098.413	0.170	411.4177
37	34	3	36	33	3	E	0	610133.968	0.185	411.4261
56	24	32	55	23	32	E	0	610151.160	0.048	486.0909
54	25	29	53	24	29	E	0	610154.978	0.162	472.1144
54	25	30	53	24	30	E	0	610197.110	-0.045	472.1089
39	33	7	38	32	6	A	0	610947.842	0.127	414.4487
39	33	6	38	32	7	A	0	610947.842	0.127	414.4487
39	33	7	38	32	7	E	0	610951.674	0.224	414.4350
39	33	6	38	32	6	E	0	610990.286	-0.058	414.4421
63	21	43	62	20	42	A	0	611394.891	0.154	549.7021
63	21	42	62	20	42	E	0	611413.975	0.064	549.7007
63	21	42	62	20	43	A	0	611555.110	0.251	549.6976
41	32	10	40	31	10	E	0	611793.970	-0.176	418.6756
41	32	9	40	31	10	A	0	611796.251	0.288	418.6892
41	32	10	40	31	9	A	0	611796.251	0.288	418.6892
41	32	9	40	31	9	E	0	611835.999	0.154	418.6812
43	31	13	42	30	13	E	0	612618.819	0.234	424.1453
43	31	13	42	30	12	A	0	612626.058	0.110	424.1586
43	31	12	42	30	13	A	0	612626.058	0.110	424.1586
43	31	12	42	30	12	E	0	612662.490	0.002	424.1494
66	20	46	65	19	47	E	0	613275.950	0.147	584.1149
66	20	46	65	19	47	A	0	613293.052	0.031	584.1171
45	30	16	44	29	16	E	0	613413.926	0.169	430.8514
45	30	15	44	29	16	A	0	613426.702	0.123	430.8642
45	30	16	44	29	15	A	0	613426.702	0.123	430.8642
45	30	15	44	29	15	E	0	613459.216	0.001	430.8539
61	22	39	60	21	39	E	0	614094.678	0.023	530.8674
61	22	40	60	21	40	E	0	614123.534	0.029	530.8584
61	22	40	60	21	39	A	0	614137.828	0.522	530.8693
61	22	39	60	21	40	A	0	614142.031	0.074	530.8691
47	29	19	46	28	19	E	0	614164.197	0.145	438.8028
47	29	19	46	28	18	A	0	614182.276	0.102	438.8149
47	29	18	46	28	19	A	0	614182.276	0.102	438.8149
47	29	18	46	28	18	E	0	614210.617	0.238	438.8037
49	28	22	48	27	21	E	0	614847.387	0.074	448.0109
49	28	21	48	27	22	A	0	614870.638	0.139	448.0221
49	28	22	48	27	21	A	0	614870.638	0.139	448.0221
49	28	21	48	27	22	E	0	614893.983	0.203	448.0101
36	35	2	35	34	1	A	0	615360.989	0.155	416.8089
36	35	1	35	34	2	A	0	615360.989	0.155	416.8089
36	35	2	35	34	2	E	0	615375.582	0.209	416.7956
36	35	1	35	34	1	E	0	615407.226	0.157	416.8052
51	27	24	50	26	24	E	0	615432.018	0.174	458.4901
59	23	36	58	22	36	E	0	615436.919	0.201	513.5912
51	27	25	50	26	24	A	0	615460.037	0.250	458.5001
51	27	24	50	26	25	A	0	615460.037	0.250	458.5001
51	27	25	50	26	25	E	0	615477.612	-0.072	458.4877
59	23	36	58	22	37	A	0	615479.697	0.329	513.5950
53	26	27	52	25	27	E	0	615871.852	0.155	470.2589
53	26	27	52	25	28	A	0	615904.111	0.092	470.2677
53	26	28	52	25	27	A	0	615904.111	0.092	470.2677
53	26	28	52	25	28	E	0	615916.144	0.045	470.2549
57	24	33	56	23	33	E	0	616009.289	-0.033	497.7708
55	25	30	54	24	30	E	0	616098.924	0.086	483.3417
55	25	31	54	24	30	A	0	616135.202	0.103	483.3489
55	25	30	54	24	31	A	0	616135.202	0.103	483.3489
55	25	31	54	24	31	E	0	616140.891	-0.046	483.3362
38	34	4	37	33	5	A	0	616223.319	0.159	419.0231
38	34	5	37	33	4	A	0	616223.319	0.159	419.0231
38	34	5	37	33	5	E	0	616232.494	0.144	419.0096
38	34	4	37	33	4	E	0	616268.043	0.151	419.0179
64	21	44	63	20	43	A	0	616432.848	0.334	563.0649
64	21	43	63	20	43	E	0	616488.296	-0.072	563.0629
64	21	44	63	20	44	E	0	616589.797	0.070	563.0506
64	21	43	63	20	44	A	0	616719.956	0.171	563.0570
40	33	8	39	32	7	A	0	617079.200	0.158	422.4572
40	33	7	39	32	8	A	0	617079.200	0.158	422.4572
40	33	8	39	32	8	E	0	617082.967	0.226	422.4435
40	33	7	39	32	7	E	0	617121.768	0.137	422.4505
42	32	11	41	31	11	E	0	617920.457	-0.154	427.1024
42	32	10	41	31	11	A	0	617922.706	0.235	427.1160
42	32	11	41	31	10	A	0	617922.706	0.235	427.1160
42	32	10	41	31	10	E	0	617962.418	0.123	427.1080
44	31	14	43	30	14	E	0	618737.386	-0.113	432.9925
44	31	14	43	30	13	A	0	618745.087	0.177	433.0057
44	31	13	43	30	14	A	0	618745.087	0.177	433.0057
44	31	13	43	30	13	E	0	618781.377	0.003	432.9965
46	30	17	45	29	17	E	0	619521.547	0.111	440.1212
46	30	16	45	29	17	A	0	619534.432	0.117	440.1339
46	30	17	45	29	16	A	0	619534.432	0.117	440.1339
46	30	16	45	29	16	E	0	619567.016	0.165	440.1237
62	22	40	61	21	40	E	0	619574.074	0.046	543.7168
62	22	41	61	21	41	E	0	619602.161	-0.090	543.7078
62	22	40	61	21	41	A	0	619623.603	-0.097	543.7183
48	29	20	47	28	20	E	0	620255.402	-0.114	448.4982
48	29	20	47	28	19	A	0	620273.769	0.068	448.5102
48	29	19	47	28	20	A	0	620273.769	0.068	448.5102
50	28	23	49	27	22	E	0	620915.982	0.206	458.1354
50	28	22	49	27	23	A	0	620939.016	-0.016	458.1465
50	28	23	49	27	22	A	0	620939.016	-0.016	458.1465
50	28	22	49	27	23	E	0	620962.264	0.118	458.1346
60	23	38	59	22	38	E	0	621166.585	-0.068	525.9480
60	23	38	59	22	37	A	0	621175.282	0.042	525.9597
52	27	25	51	26	25	E	0	621468.144	0.181	469.0481
52	27	26	51	26	25	A	0	621495.962	-0.020	469.0581
52	27	25	51	26	26	A	0	621495.962	-0.020	469.0581
37	35	3	36	34	2	A	0	621495.962	0.251	424.1920
37	35	2	36	34	3	A	0	621495.962	0.251	424.1920
37	35	3	36	34	3	E	0	621510.142	-0.084	424.1787

J'	K_a'	K_c'	J''	K_a''	K_c''	Sym.	$v_{t_{CH_3}}$	$\nu_{Obs.}$	$\nu_{Obs.} - \nu_{Calc.}$	E_{low}
52	27	26	51	26	26	E	0	621513.840	0.173	469.0457
37	35	2	36	34	2	E	0	621541.912	-0.016	424.1883
54	26	28	53	25	28	E	0	621862.513	0.148	481.2559
54	26	28	53	25	29	A	0	621894.896	0.128	481.2645
54	26	29	53	25	28	A	0	621894.896	0.128	481.2645
54	26	29	53	25	29	E	0	621906.648	0.069	481.2519
56	25	31	55	24	31	E	0	622025.242	0.076	494.7845
56	25	32	55	24	32	E	0	622067.209	0.201	494.7790
39	34	5	38	33	6	A	0	622356.453	0.168	426.8219
39	34	6	38	33	5	A	0	622356.453	0.168	426.8219
39	34	6	38	33	6	E	0	622365.443	-0.001	426.8083
39	34	5	38	33	5	E	0	622400.940	-0.046	426.8167
41	33	9	40	32	8	A	0	623208.924	0.105	430.6731
41	33	8	40	32	9	A	0	623208.924	0.105	430.6731
41	33	9	40	32	9	E	0	623212.649	0.169	430.6594
41	33	8	40	32	8	E	0	623251.464	0.101	430.6664
43	32	12	42	31	12	E	0	624044.675	-0.153	435.7372
43	32	11	42	31	12	A	0	624046.913	0.180	435.7507
43	32	12	42	31	11	A	0	624046.913	0.180	435.7507
43	32	11	42	31	11	E	0	624086.640	0.145	435.7428
45	31	15	44	30	14	A	0	624860.834	0.100	442.0614
45	31	14	44	30	15	A	0	624860.834	0.100	442.0614
63	22	41	62	21	41	E	0	624994.026	0.338	556.7924
63	22	42	62	21	42	E	0	625021.514	0.084	556.7834
63	22	42	62	21	41	A	0	625030.615	0.154	556.7940
63	22	41	62	21	42	A	0	625047.953	0.166	556.7935
47	30	18	46	29	18	E	0	625624.871	0.053	449.6004
47	30	17	46	29	18	A	0	625637.820	0.062	449.6131
47	30	18	46	29	17	A	0	625637.820	0.062	449.6131
66	21	46	65	20	45	A	0	625998.079	0.208	590.4978
49	29	21	48	28	21	E	0	626341.348	0.160	458.4038
49	29	21	48	28	20	A	0	626359.501	0.061	458.4158
49	29	20	48	28	21	A	0	626359.501	0.061	458.4158
49	29	20	48	28	20	E	0	626387.540	0.167	458.4046
36	36	0	35	35	1	A	0	626766.968	0.172	429.9539
36	36	1	35	35	0	A	0	626766.968	0.172	429.9539
36	36	1	35	35	1	E	0	626786.599	0.167	429.9411
61	23	38	60	22	38	E	0	626790.552	0.155	538.5425
36	36	0	35	35	0	E	0	626814.044	0.182	429.9517
61	23	39	60	22	39	E	0	626823.998	0.096	538.5345
66	21	45	65	20	46	A	0	626878.211	0.053	590.4740
51	28	24	50	27	23	E	0	626976.645	0.152	468.4712
51	28	23	50	27	24	A	0	626999.962	0.139	468.4823
51	28	24	50	27	23	A	0	626999.962	0.139	468.4823
51	28	23	50	27	24	E	0	627022.904	0.146	468.4704
53	27	26	52	26	26	E	0	627493.756	0.021	479.8187
53	27	27	52	26	26	A	0	627521.957	0.123	479.8286
53	27	26	52	26	27	A	0	627521.957	0.123	479.8286
53	27	27	52	26	27	E	0	627539.455	0.162	479.8162
38	35	4	37	34	3	A	0	627629.986	0.118	431.7817
38	35	3	37	34	4	A	0	627629.986	0.118	431.7817
38	35	4	37	34	4	E	0	627644.547	0.192	431.7684
59	24	35	58	23	35	E	0	627650.522	0.161	521.7847
38	35	3	37	34	3	E	0	627676.191	0.128	431.7780
59	24	35	58	23	36	A	0	627690.565	0.307	521.7901
59	24	36	58	23	35	A	0	627690.565	0.313	521.7901
55	26	29	54	25	29	E	0	627839.365	0.219	492.4669
55	26	29	54	25	30	A	0	627871.798	0.163	492.4755
55	26	30	54	25	29	A	0	627871.798	0.163	492.4755
55	26	30	54	25	30	E	0	627883.169	0.009	492.4629
57	25	32	56	24	32	E	0	627932.732	0.116	506.4433
57	25	33	56	24	32	A	0	627969.112	0.057	506.4504
57	25	32	56	24	33	A	0	627969.112	0.057	506.4504
57	25	33	56	24	33	E	0	627974.286	0.101	506.4378
40	34	6	39	33	7	A	0	628488.409	0.163	434.8277
40	34	7	39	33	6	A	0	628488.409	0.163	434.8277
40	34	7	39	33	7	E	0	628497.649	0.278	434.8142
40	34	6	39	33	6	E	0	628533.037	0.123	434.8225
42	33	10	41	32	9	A	0	629336.508	-0.352	439.0965
42	33	9	41	32	10	A	0	629336.508	-0.352	439.0965
42	33	10	41	32	10	E	0	629340.376	-0.103	439.0829
42	33	9	41	32	9	E	0	629379.181	-0.173	439.0899
44	32	13	43	31	13	E	0	630166.034	-0.533	444.5801
44	32	12	43	31	13	A	0	630168.281	-0.241	444.5936
44	32	13	43	31	12	A	0	630168.281	-0.241	444.5936
44	32	12	43	31	12	E	0	630207.883	-0.333	444.5857
64	22	43	63	21	42	A	0	630377.308	0.077	570.0969
67	21	46	66	20	46	E	0	630692.730	-0.470	604.5716
46	31	16	45	30	16	E	0	630965.598	-0.018	451.3127
46	31	16	45	30	15	A	0	630972.921	-0.217	451.3259
46	31	15	45	30	16	A	0	630972.921	-0.217	451.3259
48	30	19	47	29	19	E	0	631723.380	-0.181	459.2891
48	30	18	47	29	19	A	0	631736.276	-0.290	459.3018
48	30	19	47	29	18	A	0	631736.276	-0.290	459.3018
48	30	18	47	29	18	E	0	631768.683	-0.190	459.2915
62	23	39	61	22	39	E	0	632406.859	-0.104	551.3514
50	29	22	49	28	22	E	0	632420.321	-0.323	468.5200
62	23	40	61	22	39	A	0	632449.662	0.158	551.3547
50	29	21	49	28	21	E	0	632466.406	-0.345	468.5208
37	36	1	36	35	2	A	0	632901.100	-0.296	437.3351
37	36	2	36	35	1	A	0	632901.100	-0.296	437.3351
37	36	2	36	35	2	E	0	632920.827	-0.182	437.3223
37	36	1	36	35	1	E	0	632948.214	-0.234	437.3330
52	28	25	51	27	24	E	0	633028.695	-0.235	479.0187
52	28	24	51	27	25	A	0	633052.025	-0.314	479.0297
52	28	25	51	27	24	A	0	633052.025	-0.314	479.0297
52	28	24	51	27	25	E	0	633074.821	-0.261	479.0178
60	24	36	59	23	36	E	0	633429.268	-0.321	534.1199
60	24	37	59	23	37	E	0	633466.968	-0.348	534.1130
60	24	36	59	23	37	A	0	633469.349	-0.249	534.1251
60	24	37	59	23	36	A	0	633469.349	-0.235	534.1251
54	27	27	53	26	27	E	0	633508.192	-0.285	490.8022
54	27	28	53	26	27	A	0	633536.383	-0.278	490.8120
54	27	27	53	26	28	A	0	633536.383	-0.279	490.8120
54	27	28	53	26	28	E	0	633553.548	-0.330	490.7997
39	35	5	38	34	4	A	0	633762.871	-0.302	439.5781
39	35	4	38	34	5	A	0	633762.871	-0.302	439.5781
39	35	5	38	34	5	E	0	633777.404	-0.227	439.5649
56	26	30	55	25	30	E	0	633800.841	-0.292	503.8925
39	35	4	38	34	4	E	0	633809.032	-0.311	439.5744
58	25	33	57	24	33	E	0	633819.573	-0.356	518.3187
56	26	30	55	25	31	A	0	633833.413	-0.301	503.9010
56	26	31	55	25	30	A	0	633833.413	-0.301	503.9010
56	26	31	55	25	31	E	0	633844.661	-0.273	503.8885
58	25	34	57	24	33	A	0	633856.218	-0.249	518.3256
58	25	33	57	24	34	A	0	633856.218	-0.249	518.3256

J'	K'_a	K'_c	J''	K''_a	K''_c	Sym.	$v_{t_{CH_3}}$	$\nu_{Obs.}$	$\nu_{Obs.} - \nu_{Calc.}$	E_{low}
58	25	34	57	24	34	E	0	633860.880	-0.329	518.3131
41	34	7	40	33	8	A	0	634618.614	-0.271	443.0407
41	34	8	40	33	7	A	0	634618.614	-0.271	443.0407
41	34	8	40	33	8	E	0	634627.784	-0.188	443.0272
41	34	7	40	33	7	E	0	634663.155	-0.359	443.0355
43	33	11	42	32	10	A	0	635462.747	-0.223	447.7277
43	33	10	42	32	11	A	0	635462.747	-0.223	447.7277
43	33	11	42	32	11	E	0	635466.256	-0.288	447.7140
43	33	10	42	32	10	E	0	635505.098	-0.312	447.7210
65	22	44	64	21	43	A	0	635645.109	-0.244	583.6286
65	22	43	64	21	44	A	0	635705.684	-0.251	583.6269
45	32	13	44	31	14	A	0	636287.361	-0.242	453.6448
45	32	14	44	31	13	A	0	636287.361	-0.242	453.6448
45	32	13	44	31	13	E	0	636326.945	-0.276	453.6368
47	31	17	46	30	17	E	0	637073.948	-0.303	460.7862
47	31	17	46	30	16	A	0	637081.553	-0.282	460.7994
47	31	16	46	30	17	A	0	637081.553	-0.282	460.7994
47	31	16	46	30	16	E	0	637117.645	-0.380	460.7902
49	30	20	48	29	20	E	0	637816.961	-0.348	469.1877
49	30	19	48	29	20	A	0	637830.046	-0.337	469.2003
49	30	20	48	29	19	A	0	637830.046	-0.337	469.2003
49	30	19	48	29	19	E	0	637862.345	-0.218	469.1901
69	21	49	68	20	48	E	0	637924.657	-0.223	633.4684
63	23	40	62	22	40	E	0	637978.397	-0.532	564.3836
63	23	41	62	22	41	E	0	638010.922	-0.364	564.3755
51	29	23	50	28	23	E	0	638493.088	-0.355	478.8469
51	29	23	50	28	22	A	0	638511.534	-0.312	478.8588
51	29	22	50	28	23	A	0	638511.534	-0.312	478.8588
51	29	22	50	28	22	E	0	638539.319	-0.147	478.8477
38	36	2	37	35	3	A	0	639035.053	-0.344	444.9228
38	36	3	37	35	2	A	0	639035.053	-0.344	444.9228
38	36	3	37	35	3	E	0	639054.590	-0.394	444.9100
53	28	26	52	27	25	E	0	639072.178	-0.351	489.7780
38	36	2	37	35	2	E	0	639082.484	0.052	444.9207
53	28	25	52	27	26	A	0	639095.694	-0.330	489.7889
53	28	26	52	27	25	A	0	639095.694	-0.330	489.7889
53	28	25	52	27	26	E	0	639118.424	-0.139	489.7771
61	24	37	60	23	37	E	0	639178.262	-0.382	546.6748
61	24	38	60	23	38	E	0	639215.728	-0.220	546.6679
61	24	37	60	23	38	A	0	639218.402	-0.375	546.6799
61	24	38	60	23	37	A	0	639218.402	-0.345	546.6799
55	27	28	54	26	28	E	0	639511.259	-0.210	501.9990
55	27	29	54	26	28	A	0	639539.460	-0.286	502.0087
55	27	28	54	26	29	A	0	639539.460	-0.286	502.0087
55	27	29	54	26	29	E	0	639556.305	-0.400	501.9964
59	25	34	58	24	34	E	0	639685.487	-0.273	530.4110
59	25	35	58	24	34	A	0	639722.204	-0.200	530.4178
59	25	34	58	24	35	A	0	639722.204	-0.201	530.4178
57	26	31	56	25	31	E	0	639747.296	-0.070	515.5330
57	26	31	56	25	32	A	0	639779.719	-0.329	515.5414
57	26	32	56	25	31	A	0	639779.719	-0.329	515.5414
40	35	6	39	34	5	A	0	639895.253	-0.237	447.5815
40	35	5	39	34	6	A	0	639895.253	-0.237	447.5815
40	35	6	39	34	6	E	0	639909.724	-0.191	447.5682
40	35	5	39	34	5	E	0	639941.399	-0.233	447.5778
42	34	8	41	33	9	A	0	640747.770	-0.263	451.4611
42	34	9	41	33	8	A	0	640747.770	-0.263	451.4611
42	34	9	41	33	9	E	0	640756.922	-0.158	451.4476
42	34	8	41	33	8	E	0	640792.554	-0.065	451.4559
44	33	12	43	32	11	A	0	641586.716	-0.231	456.5667
44	33	11	43	32	12	A	0	641586.716	-0.231	456.5667
44	33	12	43	32	12	E	0	641590.146	-0.326	456.5531
44	33	11	43	32	11	E	0	641629.084	-0.242	456.5601
46	32	15	45	31	15	E	0	642401.157	-0.503	462.8910
46	32	14	45	31	15	A	0	642403.557	-0.169	462.9045
46	32	15	45	31	14	A	0	642403.557	-0.169	462.9045
46	32	14	45	31	14	E	0	642443.174	-0.090	462.8965
48	31	18	47	30	18	E	0	643178.334	-0.539	470.4690
48	31	18	47	30	17	A	0	643186.203	-0.320	470.4821
48	31	17	47	30	18	A	0	643186.203	-0.320	470.4821
48	31	17	47	30	17	E	0	643222.225	-0.382	470.4729
64	23	41	63	22	41	E	0	643502.446	-0.067	577.6399
64	23	42	63	22	41	A	0	643544.065	-0.089	577.6429
64	23	41	63	22	42	A	0	643547.264	-0.094	577.6428
50	30	21	49	29	21	E	0	643905.578	-0.114	479.2963
50	30	20	49	29	21	A	0	643918.477	-0.364	479.3089
50	30	21	49	29	20	A	0	643918.477	-0.364	479.3089
50	30	20	49	29	20	E	0	643950.552	-0.332	479.2987
37	37	1	36	36	0	A	0	644305.987	-0.258	450.8605
37	37	0	36	36	1	A	0	644305.987	-0.258	450.8605
37	37	1	36	36	1	E	0	644330.351	-0.338	450.8484
37	37	0	36	36	0	E	0	644353.216	-0.284	450.8600
52	29	24	51	28	24	E	0	644558.736	-0.389	489.3849
52	29	24	51	28	23	A	0	644577.301	-0.311	489.3967
52	29	23	51	28	24	A	0	644577.301	-0.311	489.3967
52	29	23	51	28	23	E	0	644604.770	-0.289	489.3856
62	24	38	61	23	38	E	0	644894.912	-0.414	559.4500
62	24	39	61	23	39	E	0	644931.913	-0.268	559.4431
62	24	39	61	23	38	A	0	644935.290	-0.250	559.4549
54	28	27	53	27	26	E	0	645106.412	-0.301	500.7496
54	28	26	53	27	27	A	0	645130.100	-0.199	500.7605
54	28	27	53	27	26	A	0	645130.100	-0.199	500.7605
54	28	26	53	27	27	E	0	645152.255	-0.363	500.7487
39	36	3	38	35	4	A	0	645168.380	-0.301	452.7171
39	36	4	38	35	3	A	0	645168.380	-0.301	452.7171
39	36	4	38	35	4	E	0	645188.086	-0.155	452.7044
39	36	3	38	35	3	E	0	645215.471	-0.226	452.7150
56	27	29	55	26	29	E	0	645501.875	-0.083	513.4094
56	27	30	55	26	30	E	0	645546.796	-0.221	513.4068
60	25	36	59	24	35	A	0	645565.194	-0.237	542.7276
60	25	35	59	24	36	A	0	645565.194	-0.238	542.7276
60	25	36	59	24	36	E	0	645568.816	-0.500	542.7153
58	26	32	57	25	32	E	0	645676.463	-0.370	527.3889
58	26	32	57	25	33	A	0	645709.248	-0.372	527.3972
58	26	33	57	25	32	A	0	645709.248	-0.372	527.3972
58	26	33	57	25	33	E	0	645719.814	-0.353	527.3848
41	35	7	40	34	6	A	0	646026.379	-0.298	455.7918
41	35	6	40	34	7	A	0	646026.379	-0.298	455.7918
41	35	7	40	34	7	E	0	646040.937	-0.129	455.7786
41	35	6	40	34	6	E	0	646072.432	-0.354	455.7881
43	34	9	42	33	10	A	0	646875.223	-0.294	460.0889
43	34	10	42	33	9	A	0	646875.223	-0.294	460.0889
43	34	10	42	33	10	E	0	646884.086	-0.433	460.0754
43	34	9	42	33	9	E	0	646919.748	-0.309	460.0837
45	33	13	44	32	12	A	0	647708.261	-0.318	465.6138
45	33	12	44	32	13	A	0	647708.261	-0.318	465.6138

J'	K'_a	K'_c	J''	K''_a	K''_c	Sym.	$v_{t_{CH_3}}$	$\nu_{Obs.}$	$\nu_{Obs.} - \nu_{Calc.}$	E_{low}
45	33	12	44	32	12	E	0	647750.581	-0.311	465.6071
47	32	15	46	31	16	A	0	648516.441	-0.196	472.3729
47	32	16	46	31	15	A	0	648516.441	-0.196	472.3729
47	32	15	46	31	15	E	0	648556.075	-0.012	472.3649
49	31	19	48	30	18	A	0	649286.650	-0.240	480.3743
49	31	18	48	30	19	A	0	649286.650	-0.240	480.3743
49	31	18	48	30	18	E	0	649322.427	-0.433	480.3651
41	41	1	40	40	0	A	0	714443.402	0.132	540.3372
41	41	0	40	40	1	A	0	714443.402	0.132	540.3372
60	31	30	59	30	29	A	0	716003.309	0.185	603.0778
60	31	29	59	30	30	A	0	716003.309	0.185	603.0778
45	39	7	44	38	6	A	0	716159.092	0.357	545.4977
45	39	6	44	38	7	A	0	716159.092	0.357	545.4977
45	39	6	44	38	6	E	0	716191.648	0.085	545.4874
62	30	32	61	29	33	A	0	716405.510	0.114	617.0924
62	30	33	61	29	32	A	0	716405.510	0.114	617.0924
66	28	38	65	27	39	A	0	716553.725	-0.171	649.0997
66	28	39	65	27	38	A	0	716553.725	-0.171	649.0997
64	29	36	63	28	35	A	0	716615.062	-0.042	632.4237
64	29	35	63	28	36	A	0	716615.062	-0.042	632.4237
47	38	9	46	37	10	A	0	717007.628	0.267	549.9081
47	38	10	46	37	9	A	0	717007.628	0.267	549.9081
47	38	9	46	37	10	E	0	717035.868	-0.091	549.8968
47	38	10	46	37	9	E	0	717054.007	0.030	549.9090
49	37	13	48	36	12	A	0	717843.856	0.035	555.5443
49	37	12	48	36	13	A	0	717843.856	0.035	555.5443
49	37	13	48	36	13	E	0	717867.616	-0.151	555.5323
49	37	12	48	36	12	E	0	717890.691	-0.021	555.5436
51	36	15	50	35	16	A	0	718661.551	0.063	562.4118
51	36	16	50	35	15	A	0	718661.551	0.063	562.4118
51	36	16	50	35	16	E	0	718680.701	0.277	562.3991
51	36	15	50	35	15	E	0	718707.999	0.039	562.4095
53	35	19	52	34	18	A	0	719451.628	0.177	570.5170
53	35	18	52	34	19	A	0	719451.628	0.177	570.5170
55	34	21	54	33	22	A	0	720201.822	0.093	579.8679
55	34	22	54	33	21	A	0	720201.822	0.093	579.8679
55	34	22	54	33	22	E	0	720209.824	-0.011	579.8547
42	41	2	41	40	1	A	0	720573.291	0.170	548.7412
42	41	1	41	40	2	A	0	720573.291	0.170	548.7412
59	32	27	58	31	28	A	0	721513.334	0.338	602.3472
59	32	28	58	31	27	A	0	721513.334	0.338	602.3472
46	39	8	45	38	7	A	0	722285.297	-0.008	554.7330
46	39	7	45	38	8	A	0	722285.297	-0.008	554.7330
46	39	7	45	38	7	E	0	722318.085	-0.005	554.7226
46	39	8	45	38	8	E	0	722330.995	0.053	554.7355
48	38	10	47	37	11	A	0	723130.319	-0.158	559.5608
48	38	11	47	37	10	A	0	723130.319	-0.158	559.5608
52	36	16	51	35	17	A	0	724772.051	-0.030	572.9043
52	36	17	51	35	16	A	0	724772.051	-0.030	572.9043
47	39	9	46	38	8	A	0	728410.917	0.321	564.1753
47	39	8	46	38	9	A	0	728410.917	0.321	564.1753
49	38	11	48	37	12	A	0	729251.752	-0.108	569.4211
49	38	12	48	37	11	A	0	729251.752	-0.108	569.4211
49	38	11	48	37	12	E	0	729280.585	0.244	569.4098
49	38	12	48	37	11	E	0	729298.454	0.060	569.4220
51	37	15	50	36	14	A	0	730077.425	-0.014	575.8958
51	37	14	50	36	15	A	0	730077.425	-0.014	575.8958
51	37	15	50	36	15	E	0	730101.229	-0.013	575.8837
51	37	14	50	36	14	E	0	730124.421	0.209	575.8951
53	36	17	52	35	18	A	0	730879.636	-0.044	583.6054
53	36	18	52	35	17	A	0	730879.636	-0.044	583.6054
53	36	17	52	35	17	E	0	730926.077	0.087	583.6031
55	35	21	54	34	20	A	0	731648.544	0.195	592.5571
55	35	20	54	34	21	A	0	731648.544	0.195	592.5571
42	42	0	41	41	1	A	0	731972.199	0.129	564.1684
42	42	1	41	41	0	A	0	731972.199	0.129	564.1684
42	42	1	41	41	1	E	0	732010.863	-0.215	564.1754
42	42	0	41	41	0	E	0	732014.442	-0.081	564.1617
57	34	23	56	33	24	A	0	732370.020	0.276	602.7595
57	34	24	56	33	23	A	0	732370.020	0.276	602.7595
57	34	24	56	33	24	E	0	732377.987	0.369	602.7463
57	34	23	56	33	23	E	0	732412.974	-0.004	602.7542
7	3	4	6	2	5	E	0	75823.428	-0.020	72.2759
7	3	4	6	2	5	A	0	75830.518	0.030	72.2643
56	37	20	55	36	19	A	0	760614.789	-0.055	630.4209
56	37	19	55	36	20	A	0	760614.789	-0.055	630.4209
56	37	20	55	36	20	E	0	760637.969	-0.218	630.4089
56	37	19	55	36	19	E	0	760661.264	0.037	630.4201
58	36	22	57	35	23	A	0	761365.163	0.052	640.2465
58	36	23	57	35	22	A	0	761365.163	0.052	640.2465
58	36	23	57	35	23	E	0	761383.600	0.267	640.2340
58	36	22	57	35	22	E	0	761410.957	0.056	640.2442
45	43	3	44	42	2	A	0	761753.200	-0.278	606.4167
45	43	2	44	42	3	A	0	761753.200	-0.278	606.4167
45	43	3	44	42	3	E	0	761789.142	-0.013	606.4249
45	43	2	44	42	2	E	0	761797.927	-0.018	606.4114
60	35	26	59	34	25	A	0	762064.116	0.191	651.3265
60	35	25	59	34	26	A	0	762064.116	0.191	651.3265
60	35	26	59	34	26	E	0	762076.676	-0.051	651.3137
47	42	5	46	41	6	A	0	762608.595	-0.431	609.2757
47	42	6	46	41	5	A	0	762608.595	-0.431	609.2757
47	42	6	46	41	6	E	0	762647.772	-0.234	609.2826
47	42	5	46	41	5	E	0	762651.206	-0.129	609.2690
62	34	28	61	33	29	A	0	762692.322	-0.067	663.6716
62	34	29	61	33	28	A	0	762692.322	-0.067	663.6716
62	34	29	61	33	29	E	0	762699.047	-0.495	663.6587
62	34	28	61	33	28	E	0	762734.425	-0.339	663.6663
49	41	9	48	40	8	A	0	763458.860	-0.449	613.3542
49	41	8	48	40	9	A	0	763458.860	-0.449	613.3542
70	30	40	69	29	41	A	0	763834.299	-0.028	726.0209
70	30	41	69	29	40	A	0	763834.299	-0.028	726.0209
68	31	38	67	30	38	E	0	763842.087	-0.644	708.4351
68	31	38	67	30	37	A	0	763853.023	-0.092	708.4469
68	31	37	67	30	38	A	0	763853.023	-0.092	708.4469
70	30	40	69	29	40	E	0	763860.678	-0.144	726.0112
51	40	11	50	39	12	A	0	764300.437	-0.182	618.6561
51	40	12	50	39	11	A	0	764300.437	-0.182	618.6561
51	40	11	50	39	11	E	0	764336.879	0.045	618.6469
51	40	12	50	39	12	E	0	764344.015	-0.473	618.6601
53	39	15	52	38	14	A	0	765127.856	-0.158	625.1859
53	39	14	52	38	15	A	0	765127.856	-0.158	625.1859
53	39	14	52	38	14	E	0	765160.568	0.202	625.1756
53	39	15	52	38	15	E	0	765173.369	-0.011	625.1883
55	38	17	54	37	18	A	0	765934.376	-0.569	632.9492
55	38	18	54	37	17	A	0	765934.376	-0.569	632.9492
55	38	17	54	37	18	E	0	765962.824	-0.127	632.9379

J'	K_a'	K_c'	J''	K_a''	K_c''	Sym.	$v_{t_{CH_3}}$	$\nu_{Obs.}$	$\nu_{Obs.} - \nu_{Calc.}$	E_{low}
55	38	18	54	37	17	E	0	765980.770	-0.355	632.9500
57	37	21	56	36	20	A	0	766712.700	-0.063	641.9523
57	37	20	56	36	21	A	0	766712.700	-0.063	641.9523
57	37	21	56	36	21	E	0	766735.292	-0.703	641.9404
57	37	20	56	36	20	E	0	766758.899	-0.151	641.9515
44	44	0	43	43	1	A	0	767022.312	-0.374	613.5850
44	44	1	43	43	0	A	0	767022.312	-0.374	613.5850
44	44	1	43	43	1	E	0	767054.008	-0.553	613.5944
44	44	0	43	43	0	E	0	767068.392	-0.272	613.5812
6	4	3	5	3	2	A	0	76712.155	0.002	72.0596
59	36	23	58	35	24	A	0	767449.887	-0.162	652.2031
59	36	24	58	35	23	A	0	767449.887	-0.162	652.2031
59	36	23	58	35	23	E	0	767495.808	0.096	652.2007
46	43	4	45	42	3	A	0	767879.265	-0.634	615.6424
46	43	3	45	42	4	A	0	767879.265	-0.634	615.6424
46	43	4	45	42	4	E	0	767915.489	-0.084	615.6507
46	43	3	45	42	3	E	0	767923.985	-0.353	615.6371
61	35	27	60	34	26	A	0	768131.817	0.131	663.7110
61	35	26	60	34	27	A	0	768131.817	0.131	663.7110
61	35	27	60	34	27	E	0	768144.125	-0.212	663.6982
61	35	26	60	34	26	E	0	768175.888	-0.093	663.7071
48	42	6	47	41	7	A	0	768733.507	-0.301	618.9171
48	42	7	47	41	6	A	0	768733.507	-0.301	618.9171
63	34	29	62	33	30	A	0	768737.501	-0.061	676.4873
63	34	30	62	33	29	A	0	768737.501	-0.061	676.4873
63	34	30	62	33	30	E	0	768744.358	-0.188	676.4744
48	42	7	47	41	7	E	0	768772.638	-0.139	618.9240
48	42	6	47	41	6	E	0	768775.994	-0.084	618.9104
63	34	29	62	33	29	E	0	768779.819	0.085	676.4819
65	33	32	64	32	32	E	0	769279.708	-0.274	690.5391
50	41	10	49	40	9	A	0	769581.332	-0.097	623.4123
50	41	9	49	40	10	A	0	769581.332	-0.097	623.4123
67	32	36	66	31	36	E	0	769599.008	-0.697	705.8914
67	32	35	66	31	36	A	0	769604.273	-0.161	705.9037
67	32	36	66	31	35	A	0	769604.273	-0.161	705.9037
50	41	10	49	40	10	E	0	769623.289	0.157	623.4178
67	32	35	66	31	35	E	0	769639.935	-0.142	705.8959
69	31	39	68	30	39	E	0	769767.228	-0.362	722.5706
69	31	39	68	30	38	A	0	769778.006	-0.199	722.5822
69	31	38	68	30	39	A	0	769778.006	-0.199	722.5822
6	4	2	5	3	3	A	0	77012.860	0.001	72.0506
52	40	12	51	39	13	A	0	770418.636	-0.130	629.1323
52	40	13	51	39	12	A	0	770418.636	-0.130	629.1323
52	40	12	51	39	12	E	0	770454.796	-0.118	629.1230
52	40	13	51	39	13	E	0	770462.344	-0.253	629.1362
54	39	16	53	38	15	A	0	771240.486	-0.028	636.0817
54	39	15	53	38	16	A	0	771240.486	-0.028	636.0817
54	39	15	53	38	15	E	0	771272.677	-0.105	636.0714
54	39	16	53	38	16	E	0	771285.825	0.002	636.0841
56	38	18	55	37	19	A	0	772039.279	-0.389	644.2663
56	38	19	55	37	18	A	0	772039.279	-0.389	644.2663
56	38	18	55	37	19	E	0	772067.460	-0.114	644.2551
56	38	19	55	37	18	E	0	772085.819	0.047	644.2671
58	37	22	57	36	21	A	0	772807.401	0.398	653.6929
58	37	21	57	36	22	A	0	772807.401	0.398	653.6929
58	37	22	57	36	22	E	0	772829.605	-0.511	653.6810
58	37	21	57	36	21	E	0	772853.020	-0.167	653.6921
45	44	1	44	43	2	A	0	773149.148	-0.236	622.6023
45	44	2	44	43	1	A	0	773149.148	-0.236	622.6023
45	44	2	44	43	2	E	0	773181.124	-0.139	622.6118
45	44	1	44	43	1	E	0	773194.995	-0.347	622.5985
60	36	24	59	35	25	A	0	773530.022	-0.331	664.3696
60	36	25	59	35	24	A	0	773530.022	-0.331	664.3696
60	36	25	59	35	25	E	0	773548.096	-0.211	664.3572
47	43	5	46	42	4	A	0	774005.269	-0.246	625.0747
47	43	4	46	42	5	A	0	774005.269	-0.246	625.0747
47	43	5	46	42	5	E	0	774040.839	-0.346	625.0829
47	43	4	46	42	4	E	0	774049.497	-0.426	625.0694
62	35	28	61	34	27	A	0	774193.349	-0.283	676.3062
62	35	27	61	34	28	A	0	774193.349	-0.283	676.3062
62	35	28	61	34	28	E	0	774205.861	-0.265	676.2935
64	34	30	63	33	31	A	0	774775.274	-0.171	689.5145
64	34	31	63	33	30	A	0	774775.274	-0.171	689.5145
64	34	31	63	33	31	E	0	774782.010	-0.239	689.5017
64	34	30	63	33	30	E	0	774817.105	-0.297	689.5092
49	42	7	48	41	8	A	0	774857.282	-0.211	628.7653
49	42	8	48	41	7	A	0	774857.282	-0.211	628.7653
49	42	8	48	41	8	E	0	774896.232	-0.216	628.7722
49	42	7	48	41	7	E	0	774899.387	-0.333	628.7586
66	33	33	65	32	33	E	0	775285.802	-0.287	704.0024
68	32	37	67	31	37	E	0	775564.342	-0.152	719.7956
68	32	36	67	31	37	A	0	775569.325	-0.119	719.8078
68	32	37	67	31	36	A	0	775569.325	-0.119	719.8078
70	31	40	69	30	40	E	0	775677.465	-0.218	736.9215
70	31	40	69	30	39	A	0	775688.338	-0.203	736.9331
70	31	39	69	30	40	A	0	775688.338	-0.203	736.9331
51	41	11	50	40	10	A	0	775701.947	-0.146	633.6777
51	41	10	50	40	11	A	0	775701.947	-0.146	633.6777
70	31	39	69	30	39	E	0	775718.973	-0.395	736.9242
51	41	11	50	40	11	E	0	775744.000	0.229	633.6831
53	40	13	52	39	14	A	0	776535.020	0.005	639.8162
53	40	14	52	39	13	A	0	776535.020	0.005	639.8162
53	40	13	52	39	13	E	0	776570.855	-0.236	639.8069
53	40	14	52	39	14	E	0	776578.863	0.058	639.8201
55	39	17	54	38	16	A	0	777350.218	-0.359	647.1857
55	39	16	54	38	17	A	0	777350.218	-0.359	647.1857
55	39	16	54	38	16	E	0	777382.770	0.013	647.1754
55	39	17	54	38	17	E	0	777395.942	0.115	647.1881
57	38	19	56	37	20	A	0	778141.304	0.007	655.7923
57	38	20	56	37	19	A	0	778141.304	0.007	655.7923
57	38	19	56	37	20	E	0	778168.971	-0.125	655.7811
57	38	20	56	37	19	E	0	778187.445	0.127	655.7930
59	37	23	58	36	22	A	0	778897.348	0.007	665.6429
59	37	22	58	36	23	A	0	778897.348	0.007	665.6429
59	37	22	58	36	22	E	0	778943.211	-0.203	665.6421
46	44	2	45	43	3	A	0	779275.319	-0.120	631.8260
46	44	3	45	43	2	A	0	779275.319	-0.120	631.8260
46	44	3	45	43	3	E	0	779307.290	-0.030	631.8355
46	44	2	45	43	2	E	0	779321.047	-0.327	631.8222
61	36	25	60	35	26	A	0	779605.482	-0.277	676.7462
61	36	26	60	35	25	A	0	779605.482	-0.277	676.7462
61	36	26	60	35	26	E	0	779623.468	-0.099	676.7338
61	36	25	60	35	25	E	0	779650.979	-0.168	676.7438
48	43	6	47	42	5	A	0	780130.116	-0.122	634.7136
48	43	5	47	42	6	A	0	780130.116	-0.122	634.7136
48	43	6	47	42	6	E	0	780165.978	0.076	634.7218

J'	K_a'	K_c'	J''	K_a''	K_c''	Sym.	$v_{t_{CH_3}}$	$\nu_{Obs.}$	$\nu_{Obs.} - \nu_{Calc.}$	E_{low}
48	43	5	47	42	5	E	0	780174.546	-0.066	634.7083
63	35	29	62	34	28	A	0	780249.925	0.477	689.1123
63	35	28	62	34	29	A	0	780249.925	0.477	689.1123
63	35	29	62	34	29	E	0	780261.798	0.024	689.0996
63	35	28	62	34	28	E	0	780293.228	-0.163	689.1084
65	34	31	64	33	32	A	0	780805.757	0.106	702.7537
65	34	32	64	33	31	A	0	780805.757	0.106	702.7537
65	34	32	64	33	32	E	0	780812.220	-0.048	702.7409
65	34	31	64	33	31	E	0	780847.214	-0.167	702.7483
50	42	8	49	41	9	A	0	780980.260	0.281	638.8205
50	42	9	49	41	8	A	0	780980.260	0.281	638.8205
50	42	9	49	41	9	E	0	781018.877	-0.041	638.8274
50	42	8	49	41	8	E	0	781022.336	0.179	638.8138
67	33	34	66	32	34	E	0	781282.389	-0.157	717.6787
69	32	38	68	31	38	E	0	781516.959	-0.142	733.9141
69	32	37	68	31	38	A	0	781522.452	0.168	733.9263
69	32	38	68	31	37	A	0	781522.452	0.168	733.9263
69	32	37	68	31	37	E	0	781557.157	-0.098	733.9184
52	41	12	51	40	11	A	0	781821.193	0.014	644.1504
52	41	11	51	40	12	A	0	781821.193	0.014	644.1504
52	41	11	51	40	11	E	0	781860.419	-0.136	644.1424
52	41	12	51	40	12	E	0	781863.378	0.550	644.1559
54	40	14	53	39	15	A	0	782649.077	-0.148	650.7079
54	40	15	53	39	14	A	0	782649.077	-0.148	650.7079
54	40	14	53	39	14	E	0	782685.297	0.075	650.6986
54	40	15	53	39	15	E	0	782692.783	-0.188	650.7118
56	39	18	55	38	17	A	0	783458.103	0.066	658.4980
56	39	17	55	38	18	A	0	783458.103	0.066	658.4980
56	39	17	55	38	17	E	0	783490.031	-0.091	658.4877
56	39	18	55	38	18	E	0	783503.380	0.157	658.5003
58	38	20	57	37	21	A	0	784239.836	0.201	667.5271
58	38	21	57	37	20	A	0	784239.836	0.201	667.5271
58	38	20	57	37	21	E	0	784267.334	0.015	667.5159
58	38	21	57	37	20	E	0	784285.303	-0.264	667.5278
45	45	1	44	44	0	A	0	784544.233	-0.124	639.1701
45	45	0	44	44	1	A	0	784544.233	-0.124	639.1701
45	45	1	44	44	1	E	0	784572.046	0.036	639.1806
45	45	0	44	44	0	E	0	784590.975	-0.215	639.1678
60	37	24	59	36	23	A	0	784983.442	-0.101	677.8025
60	37	23	59	36	24	A	0	784983.442	-0.101	677.8025
60	37	23	59	36	23	E	0	785029.698	0.198	677.8016
47	44	3	46	43	4	A	0	785400.931	0.158	641.2561
47	44	4	46	43	3	A	0	785400.931	0.158	641.2561
47	44	4	46	43	4	E	0	785432.768	0.115	641.2656
47	44	3	46	43	3	E	0	785446.606	-0.075	641.2523
62	36	26	61	35	27	A	0	785676.224	0.231	689.3331
62	36	27	61	35	26	A	0	785676.224	0.231	689.3331
62	36	27	61	35	27	E	0	785693.752	0.106	689.3207
62	36	26	61	35	26	E	0	785720.947	-0.282	689.3307
49	43	7	48	42	6	A	0	786253.921	-0.055	644.5593
49	43	6	48	42	7	A	0	786253.921	-0.055	644.5593
49	43	7	48	42	7	E	0	786289.835	0.202	644.5675
64	35	30	63	34	30	E	0	786310.805	-0.147	702.1170
66	34	32	65	33	33	A	0	786827.873	0.092	716.2049
66	34	33	65	33	32	A	0	786827.873	0.092	716.2049
66	34	33	65	33	33	E	0	786833.988	-0.211	716.1922
66	34	32	65	33	32	E	0	786869.189	-0.084	716.1996
51	42	9	50	41	10	A	0	787100.989	-0.165	649.0828
51	42	10	50	41	9	A	0	787100.989	-0.165	649.0828
51	42	10	50	41	10	E	0	787140.270	0.195	649.0897
51	42	9	50	41	9	E	0	787143.586	0.305	649.0761
68	33	35	67	32	35	E	0	787268.672	-0.187	731.5683
70	32	38	69	31	38	E	0	787496.660	-0.278	748.2515
53	41	13	52	40	12	A	0	787938.842	0.282	654.8307
53	41	12	52	40	13	A	0	787938.842	0.282	654.8307
53	41	12	52	40	12	E	0	787977.590	-0.279	654.8227
53	41	13	52	40	13	E	0	787980.513	0.335	654.8361
55	40	15	54	39	16	A	0	788761.125	-0.122	661.8075
55	40	16	54	39	15	A	0	788761.125	-0.122	661.8075
55	40	15	54	39	15	E	0	788796.957	-0.204	661.7983
55	40	16	54	39	16	E	0	788805.264	0.319	661.8114
57	39	19	56	38	18	A	0	789562.904	0.182	670.0188
57	39	18	56	38	19	A	0	789562.904	0.182	670.0188
57	39	18	56	38	18	E	0	789594.690	-0.014	670.0085
57	39	19	56	38	19	E	0	789607.959	0.121	670.0211
59	38	21	58	37	22	A	0	790334.537	0.060	679.4710
59	38	22	58	37	21	A	0	790334.537	0.060	679.4710
59	38	21	58	37	22	E	0	790361.906	-0.134	679.4598
46	45	2	45	44	1	A	0	790669.635	-0.320	648.3918
46	45	1	45	44	2	A	0	790669.635	-0.320	648.3918
46	45	2	45	44	2	E	0	790697.852	0.239	648.4023
46	45	1	45	44	1	E	0	790716.579	-0.190	648.3896
61	37	25	60	36	24	A	0	791065.479	0.108	690.1718
61	37	24	60	36	25	A	0	791065.479	0.108	690.1718
61	37	25	60	36	25	E	0	791087.777	-0.305	690.1600
61	37	24	60	36	24	E	0	791111.424	0.220	690.1709
48	44	4	47	43	5	A	0	791525.506	0.204	650.8927
48	44	5	47	43	4	A	0	791525.506	0.204	650.8927
48	44	5	47	43	5	E	0	791557.406	0.225	650.9022
48	44	4	47	43	4	E	0	791571.068	-0.113	650.8889
63	36	27	62	35	28	A	0	791741.083	0.313	702.1305
63	36	28	62	35	27	A	0	791741.083	0.313	702.1305
63	36	28	62	35	28	E	0	791758.190	-0.068	702.1182
63	36	27	62	35	27	E	0	791785.876	0.031	702.1281
65	35	31	64	34	30	A	0	792341.368	0.013	715.3583
65	35	30	64	34	31	A	0	792341.368	0.013	715.3583
65	35	31	64	34	31	E	0	792353.329	0.010	715.3457
50	43	8	49	42	7	A	0	792376.659	0.026	654.6117
50	43	7	49	42	8	A	0	792376.659	0.026	654.6117
65	35	30	64	34	30	E	0	792384.991	0.087	715.3543
50	43	8	49	42	8	E	0	792412.623	0.343	654.6199
50	43	7	49	42	7	E	0	792420.986	0.060	654.6064
67	34	33	66	33	34	A	0	792841.425	0.003	729.8686
67	34	34	66	33	33	A	0	792841.425	0.003	729.8686
69	33	37	68	32	36	A	0	793206.468	0.094	745.6781
69	33	36	68	32	37	A	0	793206.468	0.094	745.6781
69	33	37	68	32	37	E	0	793206.468	-0.207	745.6657
52	42	10	51	41	11	A	0	793221.065	0.155	659.5523
52	42	11	51	41	10	A	0	793221.065	0.155	659.5523
69	33	36	68	32	36	E	0	793244.463	-0.048	745.6714
52	42	11	51	41	11	E	0	793260.046	0.237	659.5592
52	42	10	51	41	10	E	0	793263.033	0.053	659.5456
54	41	14	53	40	13	A	0	794054.073	-0.032	665.7186
54	41	13	53	40	14	A	0	794054.073	-0.032	665.7186
54	41	13	53	40	13	E	0	794092.758	-0.584	665.7105
54	41	14	53	40	14	E	0	794095.912	0.224	665.7240

J'	K'_a	K'_c	J''	K''_a	K''_c	Sym.	$v_{t_{CH_3}}$	$\nu_{Obs.}$	$\nu_{Obs.} - \nu_{Calc.}$	E_{low}
56	40	16	55	39	17	A	0	794870.893	-0.036	673.1153
56	40	17	55	39	16	A	0	794870.893	-0.036	673.1153
56	40	16	55	39	16	E	0	794906.753	-0.001	673.1061
56	40	17	55	39	17	E	0	794914.901	0.327	673.1192
58	39	20	57	38	19	A	0	795664.595	0.143	681.7483
58	39	19	57	38	20	A	0	795664.595	0.143	681.7483
58	39	19	57	38	19	E	0	795696.272	-0.054	681.7380
58	39	20	57	38	20	E	0	795709.726	0.232	681.7506
60	38	22	59	37	23	A	0	796425.604	-0.013	691.6241
60	38	23	59	37	22	A	0	796425.604	-0.013	691.6241
60	38	22	59	37	23	E	0	796452.964	-0.087	691.6130
60	38	23	59	37	22	E	0	796471.678	0.324	691.6248
47	45	3	46	44	2	A	0	796794.704	-0.197	657.8199
47	45	2	46	44	3	A	0	796794.704	-0.197	657.8199
47	45	3	46	44	3	E	0	796822.742	0.178	657.8304
47	45	2	46	44	2	E	0	796841.467	-0.225	657.8176
62	37	26	61	36	25	A	0	797142.857	0.277	702.7511
62	37	25	61	36	26	A	0	797142.857	0.277	702.7511
62	37	26	61	36	26	E	0	797164.822	-0.318	702.7392
62	37	25	61	36	25	E	0	797188.411	0.132	702.7502
49	44	5	48	43	6	A	0	797648.923	-0.020	660.7360
49	44	6	48	43	5	A	0	797648.923	-0.020	660.7360
49	44	6	48	43	6	E	0	797680.919	0.099	660.7454
49	44	5	48	43	5	E	0	797695.334	0.546	660.7321
64	36	28	63	35	29	A	0	797799.923	0.127	715.1386
64	36	29	63	35	28	A	0	797799.923	0.127	715.1386
64	36	29	63	35	29	E	0	797817.019	-0.094	715.1264
64	36	28	63	35	28	E	0	797845.084	0.381	715.1362
66	35	32	65	34	31	A	0	798376.775	0.022	728.7985
66	35	31	65	34	32	A	0	798376.775	0.022	728.7985
66	35	32	65	34	32	E	0	798388.260	-0.261	728.7860
66	35	31	65	34	31	E	0	798419.929	-0.160	728.7946
51	43	9	50	42	8	A	0	798497.972	-0.137	664.8712
51	43	8	50	42	9	A	0	798497.972	-0.137	664.8712
51	43	9	50	42	9	E	0	798533.796	0.050	664.8794
51	43	8	50	42	8	E	0	798542.368	0.013	664.8659
68	34	34	67	33	35	A	0	798846.030	-0.111	743.7449
68	34	35	67	33	34	A	0	798846.030	-0.111	743.7449
68	34	35	67	33	35	E	0	798852.209	0.080	743.7324
70	33	38	69	32	37	A	0	799171.309	0.152	759.9950
70	33	37	69	32	38	A	0	799171.309	0.152	759.9950
53	42	11	52	41	12	A	0	799339.133	0.006	670.2292
53	42	12	52	41	11	A	0	799339.133	0.006	670.2292
53	42	12	52	41	12	E	0	799378.119	0.114	670.2360
53	42	11	52	41	11	E	0	799381.227	0.091	670.2225
55	41	15	54	40	14	A	0	800167.722	0.041	676.8142
55	41	14	54	40	15	A	0	800167.722	0.041	676.8142
55	41	14	54	40	14	E	0	800206.756	-0.083	676.8062
55	41	15	54	40	15	E	0	800209.570	0.345	676.8196
57	40	17	56	39	18	A	0	800978.221	0.106	684.6314
57	40	18	56	39	17	A	0	800978.221	0.106	684.6314
57	40	17	56	39	17	E	0	801013.938	0.095	684.6221
57	40	18	56	39	18	E	0	801022.186	0.483	684.6352
59	39	21	58	38	20	A	0	801763.189	0.145	693.6865
59	39	20	58	38	21	A	0	801763.189	0.145	693.6865
59	39	20	58	38	20	E	0	801794.774	-0.029	693.6763
46	46	0	45	45	1	A	0	802063.485	-0.018	665.3397
46	46	1	45	45	0	A	0	802063.485	-0.018	665.3397
46	46	1	45	45	1	E	0	802086.597	0.030	665.3511
46	46	0	45	45	0	E	0	802110.660	0.096	665.3390
61	38	23	60	37	24	A	0	802513.021	0.183	703.9867
61	38	24	60	37	23	A	0	802513.021	0.183	703.9867
61	38	24	60	37	23	E	0	802558.682	0.215	703.9874
48	45	4	47	44	3	A	0	802919.033	-0.089	667.4543
48	45	3	47	44	4	A	0	802919.033	-0.089	667.4543
48	45	4	47	44	4	E	0	802946.899	0.112	667.4648
48	45	3	47	44	3	E	0	802966.035	0.147	667.4520
63	37	27	62	36	26	A	0	803214.862	-0.050	715.5404
63	37	26	62	36	27	A	0	803214.862	-0.050	715.5404
63	37	27	62	36	27	E	0	803237.166	-0.149	715.5287
63	37	26	62	36	26	E	0	803260.574	0.102	715.5395
50	44	6	49	43	7	A	0	803771.463	-0.144	670.7859
50	44	7	49	43	6	A	0	803771.463	-0.144	670.7859
50	44	7	49	43	7	E	0	803803.481	0.001	670.7953
50	44	6	49	43	6	E	0	803817.303	-0.112	670.7821
65	36	29	64	35	30	A	0	803852.890	0.121	728.3577
65	36	30	64	35	29	A	0	803852.890	0.121	728.3577
65	36	30	64	35	30	E	0	803870.280	0.377	728.3455
67	35	33	66	34	33	E	0	804415.975	-0.219	742.4382
67	35	32	66	34	32	E	0	804447.691	-0.052	742.4467
52	43	10	51	42	9	A	0	804618.554	0.252	675.3377
52	43	9	51	42	10	A	0	804618.554	0.252	675.3377
52	43	10	51	42	10	E	0	804653.826	-0.098	675.3458
52	43	9	51	42	9	E	0	804662.669	0.173	675.3324
69	34	35	68	33	36	A	0	804841.556	0.063	757.8341
69	34	36	68	33	35	A	0	804841.556	0.063	757.8341
69	34	36	68	33	36	E	0	804847.176	-0.075	757.8217
69	34	35	68	33	35	E	0	804881.877	-0.312	757.8287
54	42	12	53	41	13	A	0	805455.746	0.057	681.1135
54	42	13	53	41	12	A	0	805455.746	0.057	681.1135
54	42	13	53	41	13	E	0	805494.393	-0.148	681.1203
54	42	12	53	41	12	E	0	805497.678	0.047	681.1068
56	41	16	55	40	15	A	0	806279.239	0.092	688.1178
56	41	15	55	40	16	A	0	806279.239	0.092	688.1178
56	41	15	55	40	15	E	0	806317.827	-0.394	688.1097
56	41	16	55	40	16	E	0	806321.174	0.525	688.1231
58	40	18	57	39	19	A	0	807082.804	0.164	696.3558
58	40	19	57	39	18	A	0	807082.804	0.164	696.3558
58	40	18	57	39	18	E	0	807118.251	-0.015	696.3466
58	40	19	57	39	19	E	0	807126.120	-0.046	696.3596
60	39	22	59	38	21	A	0	807858.167	-0.142	705.8337
60	39	21	59	38	22	A	0	807858.167	-0.142	705.8337
60	39	21	59	38	21	E	0	807889.700	-0.245	705.8235
60	39	22	59	38	22	E	0	807902.845	-0.341	705.8360
47	46	1	46	45	2	A	0	808187.909	-0.065	674.7657
47	46	2	46	45	1	A	0	808187.909	-0.065	674.7657
47	46	2	46	45	2	E	0	808210.974	-0.072	674.7772
47	46	1	46	45	1	E	0	808235.069	0.051	674.7650
62	38	24	61	37	25	A	0	808596.104	0.187	716.5589
62	38	25	61	37	24	A	0	808596.104	0.187	716.5589
62	38	24	61	37	25	E	0	808622.990	-0.080	716.5478
62	38	25	61	37	24	E	0	808641.605	0.172	716.5596
49	45	5	48	44	4	A	0	809042.825	0.288	677.2952
49	45	4	48	44	5	A	0	809042.825	0.288	677.2952
49	45	5	48	44	5	E	0	809070.381	0.176	677.3057
49	45	4	48	44	4	E	0	809089.134	-0.140	677.2929

J'	K_a'	K_c'	J''	K_a''	K_c''	Sym.	$v_{t_{CH_3}}$	$\nu_{Obs.}$	$\nu_{Obs.} - \nu_{Calc.}$	E_{low}
64	37	28	63	36	27	A	0	809282.134	0.026	728.5401
64	37	27	63	36	28	A	0	809282.134	0.026	728.5401
64	37	28	63	36	28	E	0	809304.434	0.092	728.5284
64	37	27	63	36	27	E	0	809327.390	-0.130	728.5392
51	44	7	50	43	8	A	0	809893.198	-0.004	681.0426
51	44	8	50	43	7	A	0	809893.198	-0.004	681.0426
66	36	30	65	35	31	A	0	809899.559	0.183	741.7879
66	36	31	65	35	30	A	0	809899.559	0.183	741.7879
66	36	31	65	35	31	E	0	809915.888	-0.429	741.7757
51	44	8	50	43	8	E	0	809925.300	0.229	681.0520
51	44	7	50	43	7	E	0	809939.063	0.094	681.0388
66	36	30	65	35	30	E	0	809943.910	-0.004	741.7854
68	35	34	67	34	33	A	0	810424.566	-0.048	756.3149
68	35	33	67	34	34	A	0	810424.566	-0.048	756.3149
68	35	34	67	34	34	E	0	810435.843	-0.115	756.3025
68	35	33	67	34	33	E	0	810467.445	-0.042	756.3109
53	43	11	52	42	10	A	0	810736.878	-0.227	686.0113
53	43	10	52	42	11	A	0	810736.878	-0.227	686.0113
53	43	11	52	42	11	E	0	810772.797	0.084	686.0195
70	34	36	69	33	37	A	0	810826.869	-0.144	772.1366
70	34	37	69	33	36	A	0	810826.869	-0.144	772.1366
70	34	37	69	33	37	E	0	810832.284	-0.245	772.1242
70	34	36	69	33	36	E	0	810867.430	0.014	772.1312
55	42	13	54	41	14	A	0	811570.394	-0.077	692.2054
55	42	14	54	41	13	A	0	811570.394	-0.077	692.2054
55	42	14	54	41	14	E	0	811609.304	0.011	692.2122
55	42	13	54	41	13	E	0	811612.421	0.080	692.1986
57	41	17	56	40	16	A	0	812388.439	0.080	699.6294
57	41	16	56	40	17	A	0	812388.439	0.080	699.6294
57	41	16	56	40	16	E	0	812426.907	-0.437	699.6213
57	41	17	56	40	17	E	0	812430.125	0.308	699.6347
59	40	19	58	39	20	A	0	813184.490	0.151	708.2888
59	40	20	58	39	19	A	0	813184.490	0.151	708.2888
59	40	19	58	39	19	E	0	813219.540	-0.315	708.2796
59	40	20	58	39	20	E	0	813227.892	0.092	708.2926
61	39	23	60	38	22	A	0	813950.118	0.067	718.1900
61	39	22	60	38	23	A	0	813950.118	0.067	718.1900
61	39	22	60	38	22	E	0	813981.430	-0.126	718.1798
61	39	23	60	38	23	E	0	813994.998	0.161	718.1923
48	46	2	47	45	3	A	0	814311.695	-0.091	684.3981
48	46	3	47	45	2	A	0	814311.695	-0.091	684.3981
48	46	3	47	45	3	E	0	814334.899	0.034	684.4095
48	46	2	47	45	2	E	0	814358.606	-0.203	684.3974
63	38	25	62	37	26	A	0	814674.764	0.138	729.3409
63	38	26	62	37	25	A	0	814674.764	0.138	729.3409
63	38	25	62	37	26	E	0	814701.431	-0.194	729.3298
63	38	26	62	37	25	E	0	814720.080	0.060	729.3415
50	45	6	49	44	5	A	0	815164.974	-0.093	687.3427
50	45	5	49	44	6	A	0	815164.974	-0.093	687.3427
50	45	6	49	44	6	E	0	815192.716	-0.020	687.3531
50	45	5	49	44	5	E	0	815211.665	-0.107	687.3404
65	37	29	64	36	28	A	0	815343.864	-0.031	741.7504
65	37	28	64	36	29	A	0	815343.864	-0.031	741.7504
65	37	29	64	36	29	E	0	815365.805	-0.147	741.7387
65	37	28	64	36	28	E	0	815389.149	-0.000	741.7494
67	36	31	66	35	32	A	0	815939.156	-0.136	755.4295
67	36	32	66	35	31	A	0	815939.156	-0.136	755.4295
67	36	32	66	35	32	E	0	815955.895	-0.138	755.4174
67	36	31	66	35	31	E	0	815983.859	0.228	755.4270
52	44	8	51	43	9	A	0	816013.704	0.069	691.5062
52	44	9	51	43	8	A	0	816013.704	0.069	691.5062
52	44	9	51	43	9	E	0	816045.598	0.101	691.5156
52	44	8	51	43	8	E	0	816059.393	0.036	691.5024
69	35	35	68	34	34	A	0	816436.341	0.036	770.3915
69	35	34	68	34	35	A	0	816436.341	0.036	770.3915
69	35	35	68	34	35	E	0	816447.676	0.253	770.3792
69	35	34	68	34	34	E	0	816479.145	0.214	770.3875
54	43	12	53	42	11	A	0	816854.392	-0.018	696.8923
54	43	11	53	42	12	A	0	816854.392	-0.018	696.8923
54	43	12	53	42	12	E	0	816890.140	0.140	696.9004
54	43	11	53	42	11	E	0	816898.508	0.020	696.8869
56	42	14	55	41	15	A	0	817683.234	-0.111	703.5049
56	42	15	55	41	14	A	0	817683.234	-0.111	703.5049
56	42	15	55	41	15	E	0	817722.075	-0.061	703.5117
56	42	14	55	41	14	E	0	817725.352	0.214	703.4982
58	41	18	57	40	17	A	0	818495.330	0.159	711.3491
58	41	17	57	40	18	A	0	818495.330	0.159	711.3491
58	41	17	57	40	17	E	0	818533.799	-0.260	711.3411
58	41	18	57	40	18	E	0	818537.148	0.569	711.3544
60	40	20	59	39	21	A	0	819283.115	0.076	720.4304
60	40	21	59	39	20	A	0	819283.115	0.076	720.4304
60	40	20	59	39	20	E	0	819318.439	0.001	720.4212
60	40	21	59	39	21	E	0	819326.731	0.302	720.4342
47	47	1	46	46	0	A	0	819579.914	-0.124	692.0936
47	47	0	46	46	1	A	0	819579.914	-0.124	692.0936
47	47	1	46	46	1	E	0	819597.944	-0.268	692.1058
47	47	0	46	46	0	E	0	819626.800	0.097	692.0945
62	39	24	61	38	23	A	0	820038.258	0.189	730.7557
62	39	23	61	38	24	A	0	820038.258	0.189	730.7557
62	39	23	61	38	23	E	0	820069.320	-0.114	730.7455
62	39	24	61	38	24	E	0	820082.703	-0.054	730.7579
49	46	3	48	45	4	A	0	820434.962	0.095	694.2368
49	46	4	48	45	3	A	0	820434.962	0.095	694.2368
49	46	4	48	45	4	E	0	820458.203	0.250	694.2482
49	46	3	48	45	3	E	0	820481.654	-0.213	694.2361
64	38	26	63	37	27	A	0	820748.757	0.029	742.3328
64	38	27	63	37	26	A	0	820748.757	0.029	742.3328
64	38	26	63	37	27	E	0	820775.311	-0.255	742.3218
64	38	27	63	37	26	E	0	820793.835	-0.159	742.3334
51	45	7	50	44	6	A	0	821286.510	-0.116	697.5968
51	45	6	50	44	7	A	0	821286.510	-0.116	697.5968
51	45	7	50	44	7	E	0	821314.391	0.095	697.6073
51	45	6	50	44	6	E	0	821333.214	-0.082	697.5945
66	37	29	65	36	29	E	0	821445.494	0.412	755.1704
5	5	1	4	4	1	E	0	82169.701	-0.070	72.3616
68	36	32	67	35	33	A	0	821972.153	-0.031	769.2827
68	36	33	67	35	32	A	0	821972.153	-0.031	769.2827
68	36	32	67	35	32	E	0	822016.161	-0.151	769.2802
53	44	9	52	43	10	A	0	822132.852	0.044	702.1769
53	44	10	52	43	9	A	0	822132.852	0.044	702.1769
53	44	10	52	43	10	E	0	822164.738	0.077	702.1862
53	44	9	52	43	9	E	0	822178.305	-0.174	702.1730
55	43	13	54	42	12	A	0	822970.140	0.040	707.9806
55	43	12	54	42	13	A	0	822970.140	0.040	707.9806
55	43	13	54	42	13	E	0	823005.711	0.041	707.9887
55	43	12	54	42	12	E	0	823014.142	0.028	707.9753

J'	K_a'	K_c'	J''	K_a''	K_c''	Sym.	$v_{t_{CH_3}}$	$\nu_{Obs.}$	$\nu_{Obs.} - \nu_{Calc.}$	E_{low}
57	42	15	56	41	16	A	0	823794.054	-0.127	715.0124
57	42	16	56	41	15	A	0	823794.054	-0.127	715.0124
7	4	4	6	3	3	A	0	82453.694	0.016	73.3142
59	41	19	58	40	18	A	0	824599.568	0.140	723.2772
59	41	18	58	40	19	A	0	824599.568	0.140	723.2772
61	40	21	60	39	22	A	0	825378.758	0.196	732.7810
61	40	22	60	39	21	A	0	825378.758	0.196	732.7810
61	40	21	60	39	21	E	0	825413.805	-0.032	732.7718
61	40	22	60	39	22	E	0	825422.121	0.246	732.7847
48	47	2	47	46	1	A	0	825703.342	-0.015	701.7240
48	47	1	47	46	2	A	0	825703.342	-0.015	701.7240
48	47	2	47	46	2	E	0	825721.864	0.323	701.7362
48	47	1	47	46	1	E	0	825750.318	0.312	701.7249
63	39	25	62	38	24	A	0	826122.103	-0.052	743.5308
63	39	24	62	38	25	A	0	826122.103	-0.052	743.5308
50	46	4	49	45	5	A	0	826557.125	-0.016	704.2819
50	46	5	49	45	4	A	0	826557.125	-0.016	704.2819
65	38	27	64	37	28	A	0	826817.839	-0.140	755.5349
65	38	28	64	37	27	A	0	826817.839	-0.140	755.5349
52	45	8	51	44	7	A	0	827407.195	0.065	708.0577
52	45	7	51	44	8	A	0	827407.195	0.065	708.0577
54	44	10	53	43	11	A	0	828250.426	-0.193	713.0546
54	44	11	53	43	10	A	0	828250.426	-0.193	713.0546
56	43	14	55	42	13	A	0	829084.072	0.010	719.2764
56	43	13	55	42	14	A	0	829084.072	0.010	719.2764
58	42	16	57	41	17	A	0	829902.970	0.129	726.7277
58	42	17	57	41	16	A	0	829902.970	0.129	726.7277
60	41	20	59	40	19	A	0	830701.076	0.102	735.4137
60	41	19	59	40	20	A	0	830701.076	0.102	735.4137
62	40	22	61	39	23	A	0	831471.021	0.296	745.3405
62	40	23	61	39	22	A	0	831471.021	0.296	745.3405
49	47	3	48	46	2	A	0	831826.078	0.068	711.5606
49	47	2	48	46	3	A	0	831826.078	0.068	711.5606
49	47	3	48	46	3	E	0	831844.630	0.427	711.5728
49	47	2	48	46	2	E	0	831872.150	-0.489	711.5615
64	39	26	63	38	25	A	0	832202.250	0.154	756.5155
64	39	25	63	38	26	A	0	832202.250	0.154	756.5155
51	46	5	50	45	6	A	0	832678.478	-0.054	714.5336
51	46	6	50	45	5	A	0	832678.478	-0.054	714.5336
51	46	6	50	45	6	E	0	832701.678	0.050	714.5450
51	46	5	50	45	5	E	0	832725.275	-0.200	714.5329
66	38	28	65	37	29	A	0	832882.270	0.142	768.9473
66	38	29	65	37	28	A	0	832882.270	0.142	768.9473
7	4	3	6	3	4	A	0	83346.792	0.013	73.2879
53	45	9	52	44	8	A	0	833526.320	-0.165	718.7255
53	45	8	52	44	9	A	0	833526.320	-0.165	718.7255
55	44	11	54	43	12	A	0	834366.918	-0.046	724.1396
55	44	12	54	43	11	A	0	834366.918	-0.046	724.1396
55	44	11	54	43	11	E	0	834412.469	-0.053	724.1357
57	43	15	56	42	14	A	0	835196.208	0.036	730.7799
57	43	14	56	42	15	A	0	835196.208	0.036	730.7799
59	42	17	58	41	18	A	0	836009.475	0.288	738.6512
59	42	18	58	41	17	A	0	836009.475	0.288	738.6512
61	41	21	60	40	20	A	0	836799.752	0.105	747.7588
61	41	20	60	40	21	A	0	836799.752	0.105	747.7588
48	48	0	47	47	1	A	0	837094.145	0.269	719.4319
48	48	1	47	47	0	A	0	837094.145	0.269	719.4319
48	48	1	47	47	1	E	0	837106.905	-0.016	719.4447
48	48	0	47	47	0	E	0	837139.451	-0.073	719.4343
63	40	23	62	39	24	A	0	837559.357	0.016	758.1092
63	40	24	62	39	23	A	0	837559.357	0.016	758.1092
63	40	24	62	39	24	E	0	837602.064	-0.423	758.1129
50	47	4	49	46	3	A	0	837947.986	0.061	721.6035
50	47	3	49	46	4	A	0	837947.986	0.061	721.6035
50	47	4	49	46	4	E	0	837966.434	0.306	721.6157
50	47	3	49	46	3	E	0	837994.701	0.168	721.6044
65	39	27	64	38	26	A	0	838277.925	0.253	769.7100
65	39	26	64	38	27	A	0	838277.925	0.253	769.7100
52	46	6	51	45	7	A	0	838798.946	-0.012	724.9920
52	46	7	51	45	6	A	0	838798.946	-0.012	724.9920
52	46	7	51	45	7	E	0	838822.377	0.318	725.0034
52	46	6	51	45	6	E	0	838845.822	-0.045	724.9912
67	38	29	66	37	30	A	0	838941.232	0.316	782.5703
67	38	30	66	37	29	A	0	838941.232	0.316	782.5703
69	37	33	68	36	32	A	0	839531.322	0.101	796.7008
69	37	32	68	36	33	A	0	839531.322	0.101	796.7008
54	45	10	53	44	9	A	0	839644.397	-0.204	729.6002
54	45	9	53	44	10	A	0	839644.397	-0.204	729.6002
56	44	12	55	43	13	A	0	840482.048	0.312	735.4319
56	44	13	55	43	12	A	0	840482.048	0.312	735.4319
56	44	13	55	43	13	E	0	840513.695	0.141	735.4412
58	43	16	57	42	15	A	0	841306.291	-0.018	742.4912
58	43	15	57	42	16	A	0	841306.291	-0.018	742.4912
58	43	16	57	42	16	E	0	841342.053	0.249	742.4992
58	43	15	57	42	15	E	0	841350.265	0.173	742.4858
60	42	18	59	41	19	A	0	842113.381	0.305	750.7828
60	42	19	59	41	18	A	0	842113.381	0.305	750.7828
62	41	22	61	40	21	A	0	842895.447	0.167	760.3126
62	41	21	61	40	22	A	0	842895.447	0.167	760.3126
49	48	1	48	47	2	A	0	843216.187	0.168	729.2665
49	48	2	48	47	1	A	0	843216.187	0.168	729.2665
49	48	2	48	47	2	E	0	843229.546	0.470	729.2793
49	48	1	48	47	1	E	0	843261.720	0.069	729.2689
51	47	5	50	46	4	A	0	844069.030	-0.001	731.8529
51	47	4	50	46	5	A	0	844069.030	-0.001	731.8529
51	47	5	50	46	5	E	0	844087.124	-0.120	731.8651
51	47	4	50	46	4	E	0	844115.645	0.030	731.8538
66	39	28	65	38	27	A	0	844348.831	0.176	783.1146
66	39	27	65	38	28	A	0	844348.831	0.176	783.1146
53	46	7	52	45	8	A	0	844918.689	0.352	735.6570
53	46	8	52	45	7	A	0	844918.689	0.352	735.6570
53	46	8	52	45	8	E	0	844941.652	0.210	735.6684
53	46	7	52	45	7	E	0	844965.490	0.281	735.6563
68	38	30	67	37	31	A	0	844994.068	-0.007	796.4040
68	38	31	67	37	30	A	0	844994.068	-0.007	796.4040
55	45	11	54	44	10	A	0	845761.463	0.082	740.6821
55	45	10	54	44	11	A	0	845761.463	0.082	740.6821
55	45	11	54	44	11	E	0	845789.426	0.387	740.6925
57	44	13	56	43	14	A	0	846594.955	0.132	746.9317
57	44	14	56	43	13	A	0	846594.955	0.132	746.9317
59	43	17	58	42	16	A	0	847414.463	0.123	754.4103
59	43	16	58	42	17	A	0	847414.463	0.123	754.4103
61	42	19	60	41	20	A	0	848214.296	-0.062	763.1229
61	42	20	60	41	19	A	0	848214.296	-0.062	763.1229
63	41	23	62	40	22	A	0	848987.945	0.244	773.0754
63	41	22	62	40	23	A	0	848987.945	0.244	773.0754

J'	K'_a	K'_c	J''	K''_a	K''_c	Sym.	$v_{t_{CH_3}}$	$\nu_{Obs.}$	$\nu_{Obs.} - \nu_{Calc.}$	E_{low}
50	48	2	49	47	3	A	0	849337.282	-0.203	739.3073
50	48	3	49	47	2	A	0	849337.282	-0.203	739.3073
50	48	3	49	47	3	E	0	849350.339	-0.217	739.3201
50	48	2	49	47	2	E	0	849382.750	-0.351	739.3097
65	40	25	64	39	26	A	0	849725.112	-0.036	784.2748
65	40	26	64	39	25	A	0	849725.112	-0.036	784.2748
52	47	6	51	46	5	A	0	850189.224	-0.031	742.3088
52	47	5	51	46	6	A	0	850189.224	-0.031	742.3088
52	47	6	51	46	6	E	0	850207.803	0.326	742.3210
52	47	5	51	46	5	E	0	850235.958	0.147	742.3096
67	39	29	66	38	28	A	0	850414.723	-0.090	796.7293
67	39	28	66	38	29	A	0	850414.723	-0.090	796.7293
54	46	8	53	45	9	A	0	851036.919	0.336	746.5289
54	46	9	53	45	8	A	0	851036.919	0.336	746.5289
69	38	31	68	37	32	A	0	851041.658	0.326	810.4487
69	38	32	68	37	31	A	0	851041.658	0.326	810.4487
54	46	9	53	45	9	E	0	851059.845	0.154	746.5403
56	45	12	55	44	11	A	0	851876.703	-0.021	751.9711
56	45	11	55	44	12	A	0	851876.703	-0.021	751.9711
58	44	14	57	43	15	A	0	852706.203	0.091	758.6391
58	44	15	57	43	14	A	0	852706.203	0.091	758.6391
8	3	5	7	2	6	E	0	85314.828	0.026	73.6709
8	3	5	7	2	6	A	0	85321.863	0.036	73.6593
60	43	18	59	42	17	A	0	853520.426	0.288	766.5374
60	43	17	59	42	18	A	0	853520.426	0.288	766.5374
62	42	20	61	41	21	A	0	854312.919	0.037	775.6714
62	42	21	61	41	20	A	0	854312.919	0.037	775.6714
49	49	1	48	48	0	A	0	854604.956	0.030	747.3543
49	49	0	48	48	1	A	0	854604.956	0.030	747.3543
49	49	1	48	48	1	E	0	854613.176	0.501	747.3676
49	49	0	48	48	0	E	0	854649.192	0.243	747.3583
64	41	24	63	40	23	A	0	855076.850	0.114	786.0472
64	41	23	63	40	24	A	0	855076.850	0.114	786.0472
51	48	3	50	47	4	A	0	855458.128	-0.081	749.5545
51	48	4	50	47	3	A	0	855458.128	-0.081	749.5545
51	48	4	50	47	4	E	0	855471.262	-0.031	749.5673
51	48	3	50	47	3	E	0	855503.810	0.004	749.5569
66	40	26	65	39	27	A	0	855802.108	0.174	797.6720
66	40	27	65	39	26	A	0	855802.108	0.174	797.6720
9	8	2	9	7	3	E	0	85581.919	-0.018	85.8376
9	8	1	9	7	2	E	0	85613.129	-0.036	85.8475
9	8	2	9	7	3	A	0	85629.079	0.019	85.8343
53	47	7	52	46	6	A	0	856308.725	0.205	752.9713
53	47	6	52	46	7	A	0	856308.725	0.205	752.9713
53	47	7	52	46	7	E	0	856327.262	0.513	752.9835
53	47	6	52	46	6	E	0	856354.905	-0.138	752.9721
68	39	30	67	38	29	A	0	856475.804	-0.101	810.5544
68	39	29	67	38	30	A	0	856475.804	-0.101	810.5544
8	8	0	8	7	1	A	0	85705.202	0.083	83.9836
8	8	1	8	7	2	A	0	85705.202	0.082	83.9836
70	38	32	69	37	33	A	0	857082.346	-0.053	824.7045
70	38	33	69	37	32	A	0	857082.346	-0.053	824.7045
70	38	32	69	37	33	E	0	857107.750	-0.313	824.6937
55	46	9	54	45	10	A	0	857153.856	0.250	757.6078
55	46	10	54	45	9	A	0	857153.856	0.250	757.6078
55	46	10	54	45	10	E	0	857177.185	0.468	757.6191
55	46	9	54	45	9	E	0	857200.681	0.291	757.6070
57	45	13	56	44	12	A	0	857990.721	0.191	763.4674
57	45	12	56	44	13	A	0	857990.721	0.191	763.4674
59	44	15	58	43	16	A	0	858815.639	0.157	770.5541
59	44	16	58	43	15	A	0	858815.639	0.157	770.5541
59	44	16	58	43	16	E	0	858847.331	0.083	770.5633
59	44	15	58	43	15	E	0	858860.729	-0.024	770.5503
61	43	19	60	42	18	A	0	859623.608	0.044	778.8727
61	43	18	60	42	19	A	0	859623.608	0.044	778.8727
61	43	18	60	42	18	E	0	859667.287	0.222	778.8674
63	42	21	62	41	22	A	0	860408.719	0.230	788.4286
63	42	22	62	41	21	A	0	860408.719	0.230	788.4286
50	49	2	49	48	1	A	0	860725.879	0.013	757.3931
50	49	1	49	48	2	A	0	860725.879	0.013	757.3931
50	49	2	49	48	2	E	0	860733.911	0.281	757.4064
50	49	1	49	48	1	E	0	860769.934	0.057	757.3971
65	41	25	64	40	24	A	0	861162.406	0.207	799.2282
65	41	24	64	40	25	A	0	861162.406	0.207	799.2282
52	48	4	51	47	5	A	0	861578.076	-0.045	760.0080
52	48	5	51	47	4	A	0	861578.076	-0.045	760.0080
52	48	5	51	47	5	E	0	861591.521	0.304	760.0208
52	48	4	51	47	4	E	0	861623.761	0.066	760.0104
67	40	27	66	39	28	A	0	861874.454	0.093	811.2790
67	40	28	66	39	27	A	0	861874.454	0.093	811.2790
54	47	8	53	46	7	A	0	862426.912	0.168	763.8405
54	47	7	53	46	8	A	0	862426.912	0.168	763.8405
54	47	8	53	46	8	E	0	862445.314	0.331	763.8526
54	47	7	53	46	7	E	0	862473.189	-0.044	763.8413
69	39	31	68	38	30	A	0	862531.675	-0.007	824.5900
69	39	30	68	38	31	A	0	862531.675	-0.007	824.5900
56	46	10	55	45	11	A	0	863269.352	0.036	768.8936
56	46	11	55	45	10	A	0	863269.352	0.036	768.8936
56	46	11	55	45	11	E	0	863292.723	0.296	768.9050
56	46	10	55	45	10	E	0	863316.363	0.312	768.8929
58	45	14	57	44	13	A	0	864102.703	0.011	775.1711
58	45	13	57	44	14	A	0	864102.703	0.011	775.1711
60	44	16	59	43	17	A	0	864922.738	-0.077	782.6770
60	44	17	59	43	16	A	0	864922.738	-0.077	782.6770
62	43	20	61	42	19	A	0	865724.469	-0.011	791.4163
62	43	19	61	42	20	A	0	865724.469	-0.011	791.4163
62	43	20	61	42	20	E	0	865760.028	0.187	791.4242
62	43	19	61	42	19	E	0	865767.961	0.087	791.4109
64	42	22	63	41	23	A	0	866501.226	0.206	801.3946
64	42	23	63	41	22	A	0	866501.226	0.206	801.3946
51	49	3	50	48	2	A	0	866845.953	-0.169	767.6382
51	49	2	50	48	3	A	0	866845.953	-0.169	767.6382
51	49	3	50	48	3	E	0	866853.805	-0.096	767.6514
51	49	2	50	48	2	E	0	866890.173	0.054	767.6421
66	41	26	65	40	25	A	0	867243.831	-0.076	812.6186
66	41	25	65	40	26	A	0	867243.831	-0.076	812.6186
66	41	25	65	40	25	E	0	867281.663	-0.105	812.6106
66	41	26	65	40	26	E	0	867285.036	0.268	812.6236
53	48	5	52	47	6	A	0	867696.885	-0.266	770.6681
53	48	6	52	47	5	A	0	867696.885	-0.266	770.6681
53	48	6	52	47	6	E	0	867710.646	0.387	770.6808
53	48	5	52	47	5	E	0	867742.800	0.101	770.6705
68	40	28	67	39	29	A	0	867942.325	0.113	825.0961
68	40	29	67	39	28	A	0	867942.325	0.113	825.0961
55	47	9	54	46	8	A	0	868544.076	0.229	774.9165
55	47	8	54	46	9	A	0	868544.076	0.229	774.9165

J'	K'_a	K'_c	J''	K''_a	K''_c	Sym.	$v_{t_{CH_3}}$	$\nu_{Obs.}$	$\nu_{Obs.} - \nu_{Calc.}$	E_{low}
70	39	32	69	38	31	A	0	868581.798	-0.091	838.8364
70	39	31	69	38	32	A	0	868581.798	-0.091	838.8364
55	47	8	54	46	8	E	0	868590.260	-0.037	774.9173
57	46	11	56	45	12	A	0	869383.492	-0.125	780.3866
57	46	12	56	45	11	A	0	869383.492	-0.125	780.3866
57	46	12	56	45	12	E	0	869406.908	0.180	780.3979
59	45	15	58	44	14	A	0	870213.227	0.125	787.0823
59	45	14	58	44	15	A	0	870213.227	0.125	787.0823
59	45	14	58	44	14	E	0	870259.139	-0.191	787.0799
61	44	17	60	43	18	A	0	871027.992	0.007	795.0078
61	44	18	60	43	17	A	0	871027.992	0.007	795.0078
63	43	21	62	42	20	A	0	871822.799	0.058	804.1682
63	43	20	62	42	21	A	0	871822.799	0.058	804.1682
50	50	0	49	49	0	E	0	872155.100	0.192	775.8663
65	42	23	64	41	24	A	0	872590.485	0.177	814.5695
65	42	24	64	41	23	A	0	872590.485	0.177	814.5695
52	49	4	51	48	3	A	0	872965.581	-0.047	778.0895
52	49	3	51	48	4	A	0	872965.581	-0.047	778.0895
67	41	27	66	40	26	A	0	873321.444	-0.221	826.2185
67	41	26	66	40	27	A	0	873321.444	-0.221	826.2185
54	48	6	53	47	7	A	0	873815.072	-0.152	781.5347
54	48	7	53	47	6	A	0	873815.072	-0.152	781.5347
56	47	10	55	46	9	A	0	874659.520	-0.224	786.1993
56	47	9	55	46	10	A	0	874659.520	-0.224	786.1993
58	46	12	57	45	13	A	0	875496.665	0.253	792.0869
58	46	13	57	45	12	A	0	875496.665	0.253	792.0869
60	45	16	59	44	15	A	0	876321.503	-0.144	799.2011
60	45	15	59	44	16	A	0	876321.503	-0.144	799.2011
62	44	18	61	43	19	A	0	877131.089	0.226	807.5467
62	44	19	61	43	18	A	0	877131.089	0.226	807.5467
8	4	5	7	3	4	E	0	87717.071	-0.020	74.8051
8	4	5	7	3	4	A	0	87760.823	0.015	74.7937
64	43	22	63	42	21	A	0	877918.391	0.192	817.1287
64	43	21	63	42	22	A	0	877918.391	0.192	817.1287
51	50	1	50	49	2	A	0	878232.540	-0.267	786.1039
51	50	2	50	49	1	A	0	878232.540	-0.267	786.1039
66	42	24	65	41	25	A	0	878676.343	0.160	827.9535
66	42	25	65	41	24	A	0	878676.343	0.160	827.9535
53	49	5	52	48	4	A	0	879084.478	0.159	788.7472
53	49	4	52	48	5	A	0	879084.478	0.159	788.7472
68	41	28	67	40	27	A	0	879395.396	0.119	840.0280
68	41	27	67	40	28	A	0	879395.396	0.119	840.0280
55	48	7	54	47	8	A	0	879932.222	-0.042	792.6080
55	48	8	54	47	7	A	0	879932.222	-0.042	792.6080
55	48	8	54	47	8	E	0	879945.681	0.282	792.6207
55	48	7	54	47	7	E	0	879977.906	0.155	792.6103
70	40	30	69	39	31	A	0	880063.433	0.150	853.3610
70	40	31	69	39	30	A	0	880063.433	0.150	853.3610
57	47	11	56	46	10	A	0	880774.447	0.100	797.6892
57	47	10	56	46	11	A	0	880774.447	0.100	797.6892
59	46	13	58	45	14	A	0	881607.570	-0.030	803.9944
59	46	14	58	45	13	A	0	881607.570	-0.030	803.9944
61	45	17	60	44	16	A	0	882428.367	0.153	811.5277
61	45	16	60	44	17	A	0	882428.367	0.153	811.5277
6	5	2	5	4	2	E	0	88313.056	-0.005	73.3897
63	44	19	62	43	20	A	0	883231.401	0.083	820.2937
63	44	20	62	43	19	A	0	883231.401	0.083	820.2937
63	44	20	62	43	20	E	0	883263.229	0.245	820.3028
63	44	19	62	43	19	E	0	883276.121	-0.086	820.2898
6	5	1	5	4	1	E	0	88354.393	0.021	73.3959
65	43	23	64	42	22	A	0	884010.833	0.129	830.2979
65	43	22	64	42	23	A	0	884010.833	0.129	830.2979
54	49	6	53	48	5	A	0	885201.915	-0.213	799.6113
54	49	5	53	48	6	A	0	885201.915	-0.213	799.6113
54	49	5	53	48	5	E	0	885246.091	0.027	799.6152
69	41	29	68	40	28	A	0	885464.564	0.026	854.0475
69	41	28	68	40	29	A	0	885464.564	0.026	854.0475
56	48	8	55	47	9	A	0	886048.131	-0.062	803.8880
56	48	9	55	47	8	A	0	886048.131	-0.062	803.8880
56	48	8	55	47	8	E	0	886093.611	-0.033	803.8903
58	47	12	57	46	11	A	0	886887.346	-0.219	809.3861
58	47	11	57	46	12	A	0	886887.346	-0.219	809.3861
58	47	12	57	46	12	E	0	886905.772	-0.060	809.3982
58	47	11	57	46	11	E	0	886934.103	0.229	809.3869
60	46	14	59	45	15	A	0	887717.054	-0.025	816.1095
60	46	15	59	45	14	A	0	887717.054	-0.025	816.1095
60	46	15	59	45	15	E	0	887740.677	0.491	816.1207
62	45	18	61	44	17	A	0	888532.818	0.136	824.0622
62	45	17	61	44	18	A	0	888532.818	0.136	824.0622
64	44	20	63	43	21	A	0	889329.450	0.238	833.2491
64	44	21	63	43	20	A	0	889329.450	0.238	833.2491
64	44	21	63	43	21	E	0	889361.217	0.368	833.2582
64	44	20	63	43	20	E	0	889374.105	0.115	833.2452
51	51	1	50	50	0	A	0	889618.252	-0.029	804.9514
51	51	0	50	50	1	A	0	889618.252	-0.029	804.9514
51	51	0	50	50	0	E	0	889657.084	-0.249	804.9583
66	43	24	65	42	23	A	0	890100.528	0.431	843.6759
66	43	23	65	42	24	A	0	890100.528	0.431	843.6759
55	49	7	54	48	6	A	0	891319.067	0.086	810.6820
55	49	6	54	48	7	A	0	891319.067	0.086	810.6820
55	49	7	54	48	7	E	0	891327.023	0.198	810.6952
55	49	6	54	48	6	E	0	891363.127	0.236	810.6859
57	48	10	56	47	10	E	0	892176.336	0.246	815.3876
59	47	13	58	46	12	A	0	892999.356	0.049	821.2903
59	47	12	58	46	13	A	0	892999.356	0.049	821.2903
59	47	13	58	46	13	E	0	893017.991	0.411	821.3023
59	47	12	58	46	12	E	0	893045.770	0.211	821.2910
61	46	15	60	45	16	A	0	893824.827	0.085	828.4321
61	46	16	60	45	15	A	0	893824.827	0.085	828.4321
61	46	15	60	45	15	E	0	893871.484	0.328	828.4312
63	45	19	62	44	18	A	0	894634.856	-0.077	836.8046
63	45	18	62	44	19	A	0	894634.856	-0.077	836.8046
65	44	21	64	43	22	A	0	895424.280	-0.127	846.4129
65	44	22	64	43	21	A	0	895424.280	-0.127	846.4129
52	51	2	51	50	1	A	0	895736.645	-0.097	815.3986
52	51	1	51	50	2	A	0	895736.645	-0.097	815.3986
52	51	1	51	50	1	E	0	895775.484	-0.302	815.4054
67	43	25	66	42	24	A	0	896186.289	0.069	857.2629
67	43	24	66	42	25	A	0	896186.289	0.069	857.2629
54	50	4	53	49	5	A	0	896587.414	-0.119	818.0703
54	50	5	53	49	4	A	0	896587.414	-0.119	818.0703
54	50	4	53	49	4	E	0	896629.713	0.419	818.0757
69	42	27	68	41	28	A	0	896911.587	0.038	869.3615
69	42	28	68	41	27	A	0	896911.587	0.038	869.3615
56	49	8	55	48	7	A	0	897434.664	-0.143	821.9593
56	49	7	55	48	8	A	0	897434.664	-0.143	821.9593

J'	K_a'	K_c'	J''	K_a''	K_c''	Sym.	$v_{t_{CH_3}}$	$\nu_{Obs.}$	$\nu_{Obs.} - \nu_{Calc.}$	E_{low}
56	49	8	55	48	8	E	0	897442.798	0.129	821.9725
56	49	7	55	48	7	E	0	897478.842	0.154	821.9632
58	48	10	57	47	11	A	0	898276.111	-0.278	827.0687
58	48	11	57	47	10	A	0	898276.111	-0.278	827.0687
60	47	14	59	46	13	A	0	899109.748	0.273	833.4017
60	47	13	59	46	14	A	0	899109.748	0.273	833.4017
8	4	4	7	3	5	A	0	89932.724	0.025	74.7316
62	46	16	61	45	17	A	0	899930.261	-0.218	840.9623
62	46	17	61	45	16	A	0	899930.261	-0.218	840.9623
64	45	20	63	44	19	A	0	900735.173	0.336	849.7551
64	45	19	63	44	20	A	0	900735.173	0.336	849.7551
66	44	22	65	43	23	A	0	901516.634	-0.126	859.7853
66	44	23	65	43	22	A	0	901516.634	-0.126	859.7853
53	51	3	52	50	3	E	0	901851.308	-0.165	826.0654
53	51	3	52	50	2	A	0	901854.618	0.119	826.0518
53	51	2	52	50	3	A	0	901854.618	0.119	826.0518
68	43	26	67	42	25	A	0	902269.098	0.191	871.0591
68	43	25	67	42	26	A	0	902269.098	0.191	871.0591
55	50	5	54	49	6	A	0	902703.908	-0.184	829.1385
55	50	6	54	49	5	A	0	902703.908	-0.184	829.1385
57	49	9	56	48	8	A	0	903549.658	0.127	833.4434
57	49	8	56	48	9	A	0	903549.658	0.127	833.4434
57	49	8	56	48	8	E	0	903593.563	0.184	833.4472
59	48	11	58	47	12	A	0	904388.551	0.064	838.9695
59	48	12	58	47	11	A	0	904388.551	0.064	838.9695
61	47	15	60	46	14	A	0	905218.214	0.241	845.7205
61	47	14	60	46	15	A	0	905218.214	0.241	845.7205
63	46	17	62	45	18	A	0	906034.153	-0.025	853.7004
63	46	18	62	45	17	A	0	906034.153	-0.025	853.7004
65	45	21	64	44	20	A	0	906832.279	0.010	862.9139
65	45	20	64	44	21	A	0	906832.279	0.010	862.9139
52	52	0	51	51	1	A	0	907120.458	0.068	834.6259
52	52	1	51	51	0	A	0	907120.458	0.068	834.6259
67	44	23	66	43	24	A	0	907606.090	-0.033	873.3665
67	44	24	66	43	23	A	0	907606.090	-0.033	873.3665
67	44	23	66	43	23	E	0	907650.667	0.142	873.3626
54	51	4	53	50	4	E	0	907968.289	-0.200	836.9249
54	51	4	53	50	3	A	0	907971.465	-0.028	836.9114
54	51	3	53	50	4	A	0	907971.465	-0.028	836.9114
54	51	3	53	50	3	E	0	908010.555	0.039	836.9182
69	43	27	68	42	26	A	0	908348.042	0.053	885.0644
69	43	26	68	42	27	A	0	908348.042	0.053	885.0644
56	50	6	55	49	7	A	0	908819.511	-0.187	840.4132
56	50	7	55	49	6	A	0	908819.511	-0.187	840.4132
56	50	6	55	49	6	E	0	908861.511	0.096	840.4186
58	49	10	57	48	9	A	0	909662.876	-0.197	845.1342
58	49	9	57	48	10	A	0	909662.876	-0.197	845.1342
58	49	9	57	48	9	E	0	909707.154	0.269	845.1381
60	48	12	59	47	13	A	0	910498.902	-0.231	851.0775
60	48	13	59	47	12	A	0	910498.902	-0.231	851.0775
62	47	16	61	46	15	A	0	911324.901	0.204	858.2469
62	47	15	61	46	16	A	0	911324.901	0.204	858.2469
64	46	18	63	45	19	A	0	912135.866	0.142	866.6464
64	46	19	63	45	18	A	0	912135.866	0.142	866.6464
66	45	22	65	44	21	A	0	912926.714	-0.383	876.2811
66	45	21	65	44	22	A	0	912926.714	-0.383	876.2811
53	52	1	52	51	2	A	0	913237.597	0.024	845.2771
53	52	2	52	51	1	A	0	913237.597	0.024	845.2771
55	51	5	54	50	4	A	0	914087.770	0.107	847.9772
55	51	4	54	50	5	A	0	914087.770	0.107	847.9772
70	43	28	69	42	27	A	0	914423.201	-0.089	899.2793
70	43	27	69	42	28	A	0	914423.201	-0.089	899.2793
59	49	11	58	48	11	E	0	915783.277	0.004	857.0451
61	48	13	60	47	14	A	0	916608.634	0.397	863.3928
61	48	14	60	47	13	A	0	916608.634	0.397	863.3928
63	47	17	62	46	16	A	0	917429.487	-0.059	870.9808
63	47	16	62	46	17	A	0	917429.487	-0.059	870.9808
65	46	19	64	45	20	A	0	918235.091	0.093	879.8004
65	46	20	64	45	19	A	0	918235.091	0.093	879.8004
67	45	23	66	44	22	A	0	919019.383	0.200	889.8567
67	45	22	66	44	23	A	0	919019.383	0.200	889.8567
54	52	2	53	51	3	A	0	919353.868	-0.174	856.1345
54	52	3	53	51	2	A	0	919353.868	-0.174	856.1345
56	51	6	55	50	5	A	0	920203.207	0.260	859.2495
56	51	5	55	50	6	A	0	920203.207	0.260	859.2495
58	50	8	57	49	9	A	0	921047.384	-0.384	863.5825
58	50	9	57	49	8	A	0	921047.384	-0.384	863.5825
60	49	12	59	48	11	A	0	921886.172	-0.123	869.1367
60	49	11	59	48	12	A	0	921886.172	-0.123	869.1367
64	47	18	63	46	17	A	0	923532.534	0.124	883.9225
64	47	17	63	46	18	A	0	923532.534	0.124	883.9225
64	47	17	63	46	17	E	0	923578.472	0.160	883.9232
9	4	6	8	3	5	E	0	92405.202	-0.016	76.5167
66	46	20	65	45	21	A	0	924332.153	0.275	893.1626
66	46	21	65	45	20	A	0	924332.153	0.275	893.1626
9	4	6	8	3	5	A	0	92434.196	-0.009	76.5054
53	53	1	52	52	1	E	0	924605.688	0.066	864.8974
53	53	0	52	52	0	E	0	924651.267	-0.068	864.8935
68	45	24	67	44	23	A	0	925108.484	0.094	903.6410
68	45	23	67	44	24	A	0	925108.484	0.094	903.6410
55	52	3	54	51	4	A	0	925469.525	-0.217	867.1980
55	52	4	54	51	3	A	0	925469.525	-0.217	867.1980
70	44	26	69	43	27	A	0	925854.876	0.138	915.3637
70	44	27	69	43	26	A	0	925854.876	0.138	915.3637
57	51	7	56	50	6	A	0	926317.242	-0.034	870.7282
57	51	6	56	50	7	A	0	926317.242	-0.034	870.7282
57	51	6	56	50	6	E	0	926356.379	0.130	870.7349
59	50	9	58	49	10	A	0	927159.935	-0.150	875.4773
59	50	10	58	49	9	A	0	927159.935	-0.150	875.4773
61	49	13	60	48	12	A	0	927995.763	-0.043	881.4485
61	49	12	60	48	13	A	0	927995.763	-0.043	881.4485
63	48	15	62	47	16	A	0	928821.027	-0.413	888.6454
63	48	16	62	47	15	A	0	928821.027	-0.413	888.6454
65	47	19	64	46	18	A	0	929633.098	-0.085	897.0720
65	47	18	64	46	19	A	0	929633.098	-0.085	897.0720
67	46	21	66	45	22	A	0	930426.039	-0.201	906.7330
67	46	22	66	45	21	A	0	930426.039	-0.201	906.7330
69	45	25	68	44	24	A	0	931194.488	-0.086	917.6340
69	45	24	68	44	25	A	0	931194.488	-0.086	917.6340
56	52	4	55	51	5	A	0	931584.650	0.037	878.4679
56	52	5	55	51	4	A	0	931584.650	0.037	878.4679
58	51	8	57	50	8	E	0	932427.290	-0.391	882.4270
58	51	8	57	50	7	A	0	932430.513	-0.074	882.4135
58	51	7	57	50	8	A	0	932430.513	-0.074	882.4135
60	50	10	59	49	11	A	0	933270.953	-0.202	887.5789
60	50	11	59	49	10	A	0	933270.953	-0.202	887.5789

J'	K'_a	K'_c	J''	K''_a	K''_c	Sym.	$v_{t_{\mathrm{CH_3}}}$	$\nu_{\mathrm{Obs.}}$	$\nu_{\mathrm{Obs.}} - \nu_{\mathrm{Calc.}}$	E_{low}
60	50	11	59	49	11	E	0	933274.241	0.524	887.5923
60	50	10	59	49	10	E	0	933312.689	-0.062	887.5842
62	49	14	61	48	13	A	0	934103.747	-0.056	893.9675
62	49	13	61	48	14	A	0	934103.747	-0.056	893.9675
66	47	20	65	46	19	A	0	935731.575	-0.174	910.4294
66	47	19	65	46	20	A	0	935731.575	-0.174	910.4294
68	46	22	67	45	23	A	0	936517.703	-0.251	920.5119
68	46	23	67	45	22	A	0	936517.703	-0.251	920.5119
55	53	2	54	52	2	E	0	936882.329	-0.036	886.8101
55	53	2	54	52	2	E	0	936882.329	-0.036	886.8101
55	53	2	54	52	2	E	0	936882.329	-0.036	886.8101
55	53	2	54	52	2	E	0	936882.329	-0.036	886.8101
70	45	26	69	44	25	A	0	937277.784	0.195	931.8359
70	45	25	69	44	26	A	0	937277.784	0.195	931.8359
57	52	6	56	51	6	E	0	937690.613	0.337	889.9576
57	52	5	56	51	6	A	0	937698.314	-0.279	889.9441
57	52	6	56	51	5	A	0	937698.314	-0.279	889.9441
57	52	5	56	51	5	E	0	937734.324	0.005	889.9522
59	51	9	58	50	8	A	0	938542.686	-0.122	894.3054
59	51	8	58	50	9	A	0	938542.686	-0.122	894.3054
61	50	11	60	49	12	A	0	939380.720	-0.180	899.8875
61	50	12	60	49	11	A	0	939380.720	-0.180	899.8875
61	50	12	60	49	12	E	0	939384.009	0.521	899.9009
63	49	15	62	48	14	A	0	940209.978	-0.218	906.6938
63	49	14	62	48	15	A	0	940209.978	-0.218	906.6938
63	49	15	62	48	15	E	0	940218.416	0.223	906.7069
65	48	17	64	47	18	A	0	941027.282	-0.031	914.7282
65	48	18	64	47	17	A	0	941027.282	-0.031	914.7282
67	47	21	66	46	20	A	0	941827.633	-0.361	923.9950
67	47	20	66	46	21	A	0	941827.633	-0.361	923.9950
69	46	23	68	45	24	A	0	942606.623	-0.265	934.4993
69	46	24	68	45	23	A	0	942606.623	-0.265	934.4993
58	52	7	57	51	7	E	0	943803.535	0.203	901.6402
58	52	6	57	51	7	A	0	943811.569	-0.050	901.6268
58	52	7	57	51	6	A	0	943811.569	-0.050	901.6268
66	48	18	65	47	19	A	0	947127.050	-0.195	928.0812
66	48	19	65	47	18	A	0	947127.050	-0.195	928.0812
