# Supplementary material for: Millimeter-Wave Spectroscopy of Methylfuran Isomers: Local vs. Global Treatments of the Internal Rotation
Source: Molecules. 2022 Jun 2;27(11):3591. doi: 10.3390/molecules27113591 (PMC9182110; doi:10.3390/molecules27113591)
Supplement: Supplementary file 1 [file molecules-27-03591-s001.zip › S4 linelist 3MF.pdf]

Supporting Informations - Line list of 3-methylfuran Millimeter-wave spectroscopy of methylfuran isomers: local vs global treatments of the internal rotation

Jonas Bruckhuisen, Sathapana Chawananon, Isabelle Kleiner, Anthony Roucou, Guillaume Dhont, Colwyn Bracquart, Pierre Asselin and Arnaud Cuisset

Table S4: Line list of the rotational transitions frequencies ($\nu_{Obs.}$) of 3-methylfuran measured in the microwave region by T. Ogata *et al.* (T. Ogata, K. Kozima. B. Chem. Soc. Jpn., 44 (1971), 2344-2346) and in the 70-220 GHz range in this study. The frequencies were fitted globally for the ground and excited torsional states using the BELGI-C_s code.

J'	K'_a	K'_c	J''	K''_a	K''_c	Sym.	$v_{t_{CH_3}}$	$\nu_{Obs.}$	$\nu_{Obs.} - \nu_{Calc.}$	E_{low}
upper level			lower level					MHz	MHz	cm^{-1}
3	-1	2	3	0	3	E	0	9049.310	-0.120	62.770
3	1	2	3	0	3	A	0	9052.390	-0.107	62.752
2	1	2	1	1	1	A	0	10804.930	0.044	61.978
2	1	2	1	1	1	E	0	10805.940	0.133	61.995
1	1	1	0	0	0	E	0	11367.740	0.069	61.616
1	1	1	0	0	0	A	0	11372.400	-0.168	61.599
4	-1	3	4	0	4	E	0	11525.900	-0.040	63.519
4	1	3	4	0	4	A	0	11528.800	0.032	63.502
2	0	2	1	0	1	E	0	11593.800	-0.075	61.811
2	0	2	1	0	1	A	0	11593.800	-0.128	61.794
3	0	3	2	1	2	A	0	12415.000	-0.006	62.338
3	0	3	2	1	2	E	0	12418.800	0.081	62.356
2	-1	1	1	1	0	E	0	12579.350	0.174	62.025
2	1	1	1	1	0	A	0	12580.180	0.093	62.008
4	2	2	4	1	3	A	0	15084.600	-0.117	63.886
3	2	1	3	1	2	A	0	15797.840	-0.046	63.054
2	1	2	1	0	1	E	0	16327.270	0.044	61.811
2	1	2	1	0	1	A	0	16331.290	0.085	61.794
3	0	3	2	0	2	A	0	17152.280	-0.002	62.180
3	2	2	2	2	1	A	0	17538.950	0.263	62.980
3	2	1	2	2	0	A	0	17925.170	0.040	62.983
3	1	2	2	1	1	A	0	18804.800	0.101	62.427
4	0	4	3	0	3	E	0	22464.070	-0.185	62.770
4	0	4	3	0	3	A	0	22464.630	-0.088	62.752
4	2	3	4	1	4	A	0	22507.750	-0.205	63.592
4	2	3	3	2	2	E	0	23314.120	-0.142	63.582
4	3	2	3	3	1	A	0	23563.030	0.092	64.563
4	3	2	3	3	1	E	0	23581.140	-0.007	64.576
4	-3	1	3	3	0	E	0	23586.150	0.156	64.583
4	3	1	3	3	0	A	0	23603.850	-0.102	64.563
26	8	18	26	7	19	E	0	70006.009	-0.296	141.8204
38	10	28	38	9	29	A	0	70028.165	0.143	227.7970
12	3	10	11	3	9	E	1	70160.889	0.049	192.6572
22	7	16	22	6	17	E	1	70331.904	0.228	235.3608
12	11	1	11	11	0	A	0	70584.700	0.183	98.5698
12	11	2	11	11	1	A	0	70584.700	0.183	98.5698
12	11	1	11	11	0	E	0	70584.700	-0.035	98.5844
12	11	2	11	11	1	E	0	70584.700	-0.035	98.5652
12	10	2	11	10	1	E	1	70588.815	0.263	211.1144
12	10	3	11	10	2	E	1	70593.932	-0.151	210.5314
12	10	2	11	10	1	A	1	70599.659	-0.070	211.0389
12	10	3	11	10	2	A	1	70599.659	-0.070	211.0389
12	10	2	11	10	1	A	0	70636.082	0.179	94.4015
12	10	3	11	10	2	A	0	70636.082	0.179	94.4015
12	10	2	11	10	1	E	0	70636.082	-0.086	94.4175
12	10	3	11	10	2	E	0	70636.082	-0.086	94.3991
12	9	3	11	9	2	E	1	70654.886	-0.048	207.3080
12	9	4	11	9	3	E	1	70664.934	0.072	206.7589
12	9	4	11	9	3	A	1	70670.043	-0.052	207.3078
12	9	3	11	9	2	A	1	70670.043	-0.052	207.3078
8	3	6	7	2	5	E	0	70699.679	-0.194	68.1308
12	9	3	11	9	2	E	0	70705.118	-0.557	90.6493
12	9	4	11	9	3	E	0	70705.118	-0.557	90.6320
12	9	3	11	9	2	A	0	70705.506	0.151	90.6320
12	9	4	11	9	3	A	0	70705.506	0.151	90.6320
8	3	6	7	2	5	A	0	70722.487	-0.398	68.1139
12	8	4	11	8	3	E	1	70747.970	0.037	203.8989
12	8	5	11	8	4	E	1	70764.585	-0.110	203.3912
12	8	4	11	8	3	A	1	70768.915	0.126	203.9740
12	8	5	11	8	4	A	1	70768.915	0.126	203.9740
12	7	5	11	7	4	E	1	70884.569	-0.104	200.8888
12	7	6	11	7	5	E	1	70912.733	-0.123	200.4298
12	7	6	11	7	5	A	1	70914.336	-0.012	201.0386
12	7	5	11	7	4	A	1	70914.336	-0.012	201.0386
12	7	5	11	7	4	A	0	70946.960	0.023	84.2935
12	7	6	11	7	5	A	0	70946.960	0.023	84.2935
12	7	5	11	7	4	E	0	70946.960	-0.017	84.3127
12	7	6	11	7	5	E	0	70948.002	0.122	84.2981
13	1	12	12	2	11	A	0	70988.484	0.013	77.1668
13	1	12	12	2	11	E	0	70988.484	0.074	77.1835
13	1	12	12	2	11	E	1	70989.738	0.135	193.4794
12	6	7	11	6	6	A	1	71138.299	-0.060	198.5040
12	6	6	11	6	5	A	1	71150.887	-0.009	198.5042
12	6	7	11	6	6	A	0	71168.970	0.050	81.7289
12	6	6	11	6	5	E	0	71174.955	0.012	81.7487
12	6	7	11	6	6	E	0	71176.213	0.060	81.7359
12	6	6	11	6	5	A	0	71180.918	0.030	81.7291
21	8	14	21	7	14	E	1	71302.155	0.523	233.7008
12	4	9	11	4	8	A	1	71430.541	-0.300	194.6385
12	5	8	11	5	7	A	1	71443.652	-0.040	196.3741
13	2	12	12	2	11	A	1	71463.714	-0.047	194.0147
12	4	9	11	4	8	A	0	71470.218	0.075	77.8156
12	5	8	11	5	7	A	0	71473.819	0.298	79.5729
12	4	9	11	4	8	E	0	71473.819	-0.127	77.8310
12	5	7	11	5	6	E	1	71477.770	-0.090	196.0827
12	5	8	11	5	7	E	0	71515.744	0.007	79.5840
13	2	12	12	2	11	E	0	71523.806	-0.006	77.1835
13	2	12	12	2	11	A	0	71525.439	0.051	77.1668
12	5	8	11	5	7	E	1	71571.038	-0.037	195.7421
18	2	16	18	1	17	E	0	71634.611	0.008	93.9945
12	5	7	11	5	6	E	0	71643.901	0.050	79.5960
12	5	7	11	5	6	A	1	71662.039	-0.020	196.3795
12	5	7	11	5	6	A	0	71684.277	0.067	79.5781
14	0	14	13	1	13	A	1	71764.815	-0.006	194.5207
14	1	14	13	1	13	A	1	71772.743	-0.003	194.5207
23	6	18	23	5	19	A	1	71776.642	0.101	238.0513
14	0	14	13	0	13	A	1	71780.330	0.012	194.5202
14	0	14	13	1	13	E	1	71783.581	-0.074	193.9726
14	1	14	13	1	13	E	1	71792.063	-0.111	193.9726
14	0	14	13	1	13	A	0	71798.846	-0.334	77.6624
14	0	14	13	1	13	E	0	71798.846	0.223	77.6795
14	0	14	13	0	13	E	1	71800.149	-0.036	193.9720
14	1	14	13	1	13	A	0	71807.343	-0.309	77.6624
14	1	14	13	1	13	E	0	71807.343	0.278	77.6795
14	0	14	13	0	13	A	0	71815.560	-0.112	77.6618
14	1	14	13	0	13	E	0	71823.673	0.169	77.6789
20	5	15	19	6	13	E	1	71828.190	0.150	223.0317
13	1	12	12	1	11	A	1	71831.699	0.000	193.9854
13	1	12	12	1	11	E	1	71903.042	-0.102	193.4489

J'	K_a'	K_c'	J''	K_a''	K_c''	Sym.	$v_{t_{CH_3}}$	$\nu_{Obs.}$	$\nu_{Obs.} - \nu_{Calc.}$	E_{low}
13	1	12	12	1	11	E	0	71906.367	0.013	77.1529
13	1	12	12	1	11	A	0	71908.829	0.059	77.1361
12	4	9	11	4	8	E	1	72117.865	0.142	194.0392
23	6	18	23	5	19	A	0	72289.721	-0.176	121.2795
38	9	29	38	8	30	E	1	72299.171	-0.419	341.6625
38	9	29	38	8	30	A	0	72422.684	0.012	225.3812
13	2	12	12	1	11	E	1	72437.836	-0.292	193.4489
12	4	8	11	4	7	E	1	72524.468	-0.135	194.3149
20	4	17	20	3	18	A	1	72570.450	0.120	220.4504
20	7	14	20	6	15	A	1	72623.116	0.226	227.2237
26	7	20	25	8	17	A	1	72656.757	-0.317	255.5615
25	8	17	25	7	18	A	1	72694.652	0.315	253.1367
37	10	27	37	9	28	E	0	72778.334	0.153	219.7988
12	2	10	11	2	9	A	1	72784.511	-0.023	192.8305
22	7	16	22	6	17	A	1	72814.008	0.359	235.8433
37	10	27	37	9	28	A	0	72845.529	0.141	219.7864
39	5	35	38	5	33	E	0	72845.529	-0.007	213.8059
12	2	10	11	2	9	E	0	72882.830	-0.017	75.9979
12	2	10	11	2	9	A	0	72885.948	-0.029	75.9811
35	8	27	35	7	28	E	1	72939.628	-0.001	316.1757
33	7	26	32	9	23	E	1	73003.045	-0.248	299.6214
41	10	31	41	9	32	A	0	73020.523	-0.009	252.9268
41	10	31	41	9	32	E	0	73026.005	-0.006	252.9381
35	8	27	35	7	28	A	0	73253.697	0.102	199.8822
35	8	27	35	7	28	E	0	73281.948	0.034	199.8943
12	4	8	11	4	7	A	1	73284.764	-0.056	194.7122
12	4	8	11	4	7	E	0	73285.402	0.017	77.9034
35	4	31	34	5	29	E	0	73285.402	0.017	186.0950
12	4	8	11	4	7	A	0	73286.604	0.075	77.8875
18	7	12	18	6	13	A	1	73314.806	0.122	219.4061
22	7	16	22	6	17	E	0	73575.107	-0.226	119.0990
35	8	27	35	7	28	A	1	73595.403	-0.126	316.5564
22	7	16	22	6	17	A	0	73626.420	-0.307	119.0854
23	4	19	23	3	20	A	1	73653.254	0.057	235.4394
19	7	13	19	6	14	E	0	73666.607	-0.268	106.4615
17	7	10	17	6	11	A	0	73674.633	-0.164	99.0725
31	9	22	31	8	23	E	0	73789.502	-0.148	174.1482
23	4	19	23	3	20	A	0	73812.409	0.140	118.6526
23	4	19	23	3	20	E	0	73812.409	0.031	118.6674
22	5	18	22	4	19	E	0	73817.159	-0.042	114.3182
31	9	22	31	8	23	A	0	73857.136	-0.121	174.1353
25	8	17	25	7	18	E	0	73926.544	-0.334	136.4119
18	7	12	18	6	13	A	0	74215.957	-0.293	102.6393
23	7	17	23	6	18	A	0	74228.592	-0.176	123.6908
32	7	25	32	6	26	E	1	74228.592	0.114	292.7438
22	5	18	22	3	19	A	1	74240.665	0.096	231.0662
16	7	10	16	6	11	A	1	74276.546	0.079	212.4074
45	11	34	44	13	31	A	1	74291.834	-0.062	408.6366
24	7	18	24	6	19	A	1	74571.990	0.111	245.2369
32	7	25	32	6	26	A	0	74582.081	0.228	176.4412
32	7	25	32	6	26	E	0	74609.029	0.278	176.4538
32	7	25	32	6	26	A	1	74893.145	-0.121	293.1430
32	10	22	31	11	20	E	1	74962.667	0.075	300.4535
12	3	9	11	3	8	E	1	74980.086	-0.204	193.1079
14	7	7	14	6	8	A	1	75019.739	0.165	206.2365
24	6	19	24	5	20	A	1	75071.857	0.008	242.7328
43	11	32	43	10	33	E	0	75131.426	0.406	273.2928
14	7	8	14	6	9	A	1	75136.901	0.314	206.2324
43	11	32	43	10	33	A	0	75171.899	0.315	273.2814
26	5	21	26	4	22	E	1	75175.846	0.155	252.1185
16	7	10	16	6	11	A	0	75183.977	-0.309	95.6379
12	3	9	11	3	8	A	1	75185.822	-0.040	193.6174
29	6	23	29	5	24	E	1	75217.004	0.262	271.3846
12	3	9	11	3	8	A	0	75227.920	0.184	76.7748
12	3	9	11	3	8	E	0	75227.920	-0.121	76.7913
24	7	18	24	6	19	E	0	75255.795	-0.172	128.4996
24	7	18	24	6	19	A	0	75292.273	-0.220	128.4861
13	3	11	12	3	10	A	1	75343.057	-0.060	195.5273
26	5	21	26	4	22	A	0	75366.320	0.134	135.8067
26	5	21	26	4	22	E	0	75375.869	0.120	135.8207
15	7	8	15	6	9	A	0	75388.594	-0.318	92.4552
13	3	11	12	3	10	E	0	75408.178	-0.048	78.7068
13	3	11	12	3	10	A	0	75409.534	0.049	78.6906
15	7	8	15	6	9	E	0	75456.869	-0.264	92.4718
15	7	9	15	6	10	E	0	75484.378	-0.291	92.4566
13	3	11	12	3	10	E	1	75502.439	0.000	194.9976
29	6	23	29	5	24	A	0	75510.328	0.269	155.0760
29	6	23	29	5	24	E	0	75529.827	0.346	155.0893
14	7	8	14	6	8	E	0	75529.827	0.346	89.4825
24	6	19	24	5	20	E	0	75531.496	-0.052	125.9802
24	6	19	24	5	20	A	0	75550.145	-0.141	125.9660
17	7	10	17	6	11	E	1	75624.366	-0.245	215.5832
15	7	9	15	6	10	A	0	75638.632	-0.328	92.4463
29	6	23	29	5	24	A	1	75688.881	-0.030	271.8063
17	1	16	17	1	17	E	1	75801.216	0.325	204.5436
43	9	35	42	10	32	A	0	75814.405	0.644	264.2236
44	11	33	44	10	34	E	0	75814.405	0.195	282.5282
44	11	33	44	10	34	A	0	75832.371	0.135	282.5172
14	7	8	14	6	9	E	0	75923.550	-0.436	89.4693
14	7	7	14	6	8	A	0	75929.106	-0.341	89.4644
14	7	7	14	6	8	E	0	75961.087	-0.271	89.4825
21	3	18	21	2	19	A	1	76004.315	0.049	224.3176
6	4	3	5	3	3	E	1	76116.451	0.209	182.5538
24	8	16	24	7	17	A	1	76123.775	0.320	247.9709
14	1	13	13	2	12	A	1	76187.225	0.041	196.3984
25	7	19	25	6	20	A	1	76206.354	0.310	250.2133
14	1	13	13	2	12	E	1	76230.794	0.033	195.8652
14	1	13	13	2	12	A	0	76235.214	0.018	79.5527
21	3	18	21	2	19	E	0	76335.628	0.030	107.5239
21	3	18	21	2	19	A	0	76345.421	-0.064	107.5084
13	7	7	13	6	8	A	0	76382.287	-0.176	86.6797
13	12	2	12	12	1	E	1	76414.603	-0.107	221.6389
13	12	2	12	12	1	A	1	76420.894	-0.081	222.0443
13	12	1	12	12	0	A	1	76420.894	-0.081	222.0443
13	12	1	12	12	0	A	0	76461.412	0.089	105.4896
13	12	2	12	12	1	A	0	76461.412	0.089	105.4896
13	12	1	12	12	0	E	0	76461.412	-0.132	105.5024
13	12	2	12	12	1	E	0	76461.412	-0.132	105.4827
13	11	2	12	11	1	A	1	76471.418	-0.090	217.5203
13	11	3	12	11	2	A	1	76471.418	-0.090	217.5203
14	2	13	13	2	12	A	1	76476.195	-0.031	196.3984
11	7	5	11	6	6	E	1	76508.488	-0.125	197.8777
13	11	2	12	11	1	A	0	76511.194	0.146	100.9243
13	11	3	12	11	2	A	0	76511.194	0.146	100.9243
13	11	2	12	11	1	E	0	76511.194	-0.123	100.9388
13	11	3	12	11	2	E	0	76511.194	-0.123	100.9196

J'	K'_a	K'_c	J''	K''_a	K''_c	Sym.	$v_{t_{CH_3}}$	$\nu_{Obs.}$	$\nu_{Obs.} - \nu_{Calc.}$	E_{low}
13	10	3	12	10	2	E	1	76523.417	0.026	213.4689
13	10	4	12	10	3	E	1	76531.196	-0.097	212.8861
14	2	13	13	2	12	E	1	76536.681	-0.156	195.8652
13	10	4	12	10	3	A	1	76537.676	0.000	213.3938
13	10	3	12	10	2	A	1	76537.676	0.000	213.3938
14	2	13	13	2	12	E	0	76539.568	-0.018	79.5693
14	2	13	13	2	12	A	0	76541.275	0.002	79.5527
42	11	31	42	10	32	E	0	76568.093	0.455	264.2351
13	10	3	12	10	2	A	0	76576.452	0.164	96.7576
13	10	4	12	10	3	A	0	76576.452	0.164	96.7576
13	10	3	12	10	2	E	0	76576.452	-0.105	96.7737
13	10	4	12	10	3	E	0	76576.452	-0.105	96.7553
34	11	23	33	12	22	E	0	76576.452	-0.105	201.1379
12	7	6	12	6	7	E	0	76584.312	-0.271	84.1101
13	9	4	12	9	3	E	1	76607.768	-0.083	209.6648
13	9	5	12	9	4	E	1	76621.224	-0.086	209.1160
13	9	5	12	9	4	A	1	76627.053	-0.054	209.6651
13	9	4	12	9	3	A	1	76627.053	-0.054	209.6651
12	7	5	12	6	6	E	0	76637.094	-0.194	84.1229
12	7	5	12	6	6	A	0	76644.100	-0.291	84.1034
12	7	6	12	6	7	A	0	76661.914	-0.030	84.1028
13	9	4	12	9	3	A	0	76664.761	0.152	92.9904
13	9	5	12	9	4	A	0	76664.761	0.152	92.9904
13	9	4	12	9	3	E	0	76664.761	-0.028	93.0078
19	2	17	19	1	18	A	0	76675.835	-0.104	97.3608
14	1	13	13	1	12	A	1	76696.709	0.097	196.3815
15	0	15	14	1	14	A	1	76726.395	-0.264	196.9148
15	1	15	14	1	14	A	1	76730.630	-0.047	196.9148
15	0	15	14	0	14	A	1	76734.503	-0.080	196.9145
15	1	15	14	0	14	A	1	76738.636	0.035	196.9145
13	8	6	12	8	5	E	1	76748.508	-0.042	205.7517
15	1	15	14	1	14	E	1	76749.945	-0.054	196.3673
13	8	5	12	8	4	A	1	76752.758	-0.080	206.3346
13	8	6	12	8	5	A	1	76752.758	-0.080	206.3346
15	0	15	14	0	14	E	1	76754.116	-0.049	196.3670
15	1	15	14	0	14	E	1	76758.339	-0.180	196.3670
15	0	15	14	1	14	A	0	76762.582	-0.050	80.0576
14	1	13	13	1	12	E	1	76765.483	-0.262	195.8473
15	1	15	14	1	14	A	0	76766.785	-0.164	80.0576
14	1	13	13	1	12	E	0	76769.883	0.029	79.5514
15	0	15	14	0	14	A	0	76770.963	-0.142	80.0573
14	1	13	13	1	12	A	0	76772.157	0.044	79.5347
15	1	15	14	0	14	E	0	76774.850	0.045	80.0744
13	8	5	12	8	4	A	0	76789.011	0.078	89.6239
13	8	6	12	8	5	A	0	76789.011	0.078	89.6239
13	8	5	12	8	4	E	0	76789.011	-0.051	89.6423
13	8	6	12	8	5	E	0	76789.903	0.135	89.6262
25	7	19	25	6	20	E	0	76841.348	-0.009	133.4804
36	10	26	36	9	27	E	0	76871.554	0.099	212.0002
25	7	19	25	6	20	A	0	76872.870	-0.011	133.4669
13	7	6	12	7	5	E	1	76901.244	-0.023	203.2533
13	7	7	12	7	6	E	1	76938.136	0.050	202.7952
13	7	7	12	7	6	A	1	76938.152	-0.164	203.4040
13	7	6	12	7	5	A	1	76939.620	-0.018	203.4040
36	10	26	36	9	27	A	0	76947.297	0.131	211.9879
13	7	7	12	7	6	A	0	76972.643	0.130	86.6600
13	7	7	12	7	6	E	0	76974.231	-0.104	86.6647
14	2	13	13	1	12	A	1	76985.705	0.051	196.3815
19	3	17	19	2	18	A	0	76987.992	-0.156	97.3613
14	2	13	13	1	12	E	1	77071.618	-0.204	195.8473
14	2	13	13	1	12	E	0	77074.932	-0.056	79.5514
14	2	13	13	1	12	A	0	77078.160	-0.030	79.5347
37	7	30	36	9	27	E	1	77111.589	-0.323	328.2923
13	6	7	12	6	6	E	1	77178.181	0.054	200.6528
23	6	18	23	4	19	E	0	77191.532	-0.218	121.1295
13	6	8	12	6	7	A	1	77220.087	-0.063	200.8769
23	6	18	23	4	19	A	0	77229.512	-0.055	121.1147
13	6	8	12	6	7	E	1	77242.815	-0.021	200.2510
13	6	8	12	6	7	A	0	77251.921	-0.073	84.1028
13	6	7	12	6	6	E	0	77267.892	0.081	84.1229
13	6	8	12	6	7	E	0	77267.892	0.081	84.1101
13	6	7	12	6	6	A	0	77282.049	0.039	84.1034
13	4	10	12	4	9	A	1	77328.463	-0.041	197.0212
24	8	16	24	7	17	E	0	77330.224	-0.266	131.2474
24	8	16	24	7	17	A	0	77345.702	-0.379	131.2342
13	4	10	12	4	9	A	0	77375.748	0.029	80.1996
13	4	10	12	4	9	E	0	77377.414	0.035	80.2151
13	5	9	12	5	8	A	1	77553.244	-0.035	198.7572
13	5	9	12	5	8	A	0	77586.013	0.051	81.9570
13	5	9	12	5	8	E	0	77616.466	0.060	81.9695
13	5	8	12	5	7	E	1	77681.597	-0.066	198.4669
27	9	19	27	8	19	E	1	77685.201	0.268	266.1371
21	4	18	21	3	19	A	0	77697.159	-0.178	107.5119
13	2	11	12	2	10	A	1	77751.607	-0.002	195.2583
13	5	9	12	5	8	E	1	77791.218	-0.036	198.1295
13	2	11	12	2	10	E	1	77853.695	-0.195	194.7261
13	2	11	12	2	10	E	0	77865.761	-0.035	78.4290
13	2	11	12	2	10	A	0	77869.756	-0.031	78.4123
17	4	14	16	4	12	E	1	77954.711	0.031	208.7166
13	5	8	12	5	7	E	0	77982.058	0.040	81.9858
13	5	8	12	5	7	A	1	77992.210	-0.040	198.7699
13	5	8	12	5	7	A	0	78010.098	0.031	81.9692
18	5	13	18	3	15	E	0	78010.098	0.078	98.3511
13	4	10	12	4	9	E	1	78055.988	0.219	196.4448
24	6	19	24	4	20	A	1	78078.076	0.169	242.6325
6	4	2	5	3	2	E	1	78160.824	-0.258	182.7682
42	10	32	42	9	33	A	0	78169.841	-0.156	261.6161
42	10	32	42	9	33	E	0	78193.655	-0.121	261.6268
23	5	19	23	4	20	E	0	78212.673	-0.030	118.6849
23	5	19	23	4	20	A	0	78226.768	-0.046	118.6701
30	9	21	30	8	22	E	0	78365.379	-0.187	167.6587
30	9	21	30	8	22	A	0	78427.696	-0.263	167.6460
42	10	32	42	9	33	A	1	78427.696	-0.152	378.2207
33	7	26	33	7	27	E	1	78568.567	0.309	299.4358
45	11	34	45	10	35	A	1	78679.799	0.111	408.4902
40	9	31	40	9	32	E	1	78712.268	0.255	358.0260
45	11	34	45	10	35	A	0	78739.604	-0.154	291.9134
45	11	34	45	10	35	E	0	78744.452	-0.117	291.9239
25	6	20	25	5	21	A	1	78807.927	0.005	247.5845
39	9	30	39	8	31	E	1	78837.602	-0.261	349.6429
26	7	20	26	6	21	E	0	78971.838	-0.286	138.6423
26	7	20	26	6	21	A	0	78999.162	-0.135	138.6290
39	9	30	39	8	31	A	0	79234.530	0.038	233.3593
25	6	20	25	5	21	E	0	79242.487	-0.052	130.8371
25	6	20	25	5	21	A	0	79259.071	-0.038	130.8231
39	9	30	39	8	31	E	0	79267.891	0.033	233.3704

J'	K_a'	K_c'	J''	K_a''	K_c''	Sym.	$v_{t_{\mathrm{CH_3}}}$	$\nu_{\mathrm{Obs.}}$	$\nu_{\mathrm{Obs.}} - \nu_{\mathrm{Calc.}}$	E_{low}
13	4	9	12	4	8	E	1	79335.297	-0.252	196.7340
20	5	15	19	6	14	E	0	79625.228	-0.207	106.4615
24	4	20	24	3	21	A	1	79691.357	0.100	239.9743
30	9	21	29	10	20	E	0	79762.063	0.079	167.6121
24	4	20	24	3	21	E	1	79860.433	-0.144	239.5057
24	4	20	24	3	21	E	0	79924.761	-0.000	123.2073
24	4	20	24	3	21	A	0	79928.418	0.034	123.1927
25	8	18	25	7	19	E	1	79993.375	0.061	252.2776
23	8	15	23	7	16	A	0	80087.811	-0.345	126.3118
23	8	15	23	7	16	E	0	80109.328	-0.235	126.3249
13	4	9	12	4	8	A	0	80138.164	-0.023	80.3321
13	4	9	12	4	8	E	0	80139.474	0.125	80.3479
28	7	22	27	8	19	A	1	80152.895	0.032	266.3517
14	3	12	13	3	11	A	1	80648.602	-0.026	198.0405
18	2	17	18	0	18	E	1	80657.142	0.381	207.5997
14	3	12	13	3	11	E	0	80722.024	-0.009	81.2221
14	3	12	13	3	11	A	0	80723.715	0.004	81.2060
14	3	12	13	3	11	E	1	80773.202	-0.091	197.5160
36	8	28	36	7	29	A	0	80989.597	0.078	207.1531
30	6	24	30	6	25	E	1	81000.242	0.543	277.3296
36	8	28	36	7	29	E	0	81023.292	0.142	207.1646
22	8	14	22	7	15	A	1	81102.118	0.282	238.3609
27	7	21	27	6	22	A	1	81113.550	0.185	260.7042
29	8	22	28	9	19	A	0	81118.610	0.178	158.2891
12	3	10	11	2	9	E	0	81209.807	-0.263	75.9979
12	3	10	11	2	9	A	0	81227.969	-0.202	75.9811
27	5	22	27	5	23	E	1	81246.922	0.088	257.3542
22	3	19	22	2	20	A	1	81283.284	0.042	228.3548
13	3	10	12	3	9	E	1	81297.825	-0.172	195.6090
15	1	14	14	2	13	A	1	81303.999	0.013	198.9494
20	3	18	20	2	19	A	1	81318.377	-0.303	217.7379
15	1	14	14	2	13	E	1	81353.739	-0.166	198.4182
15	1	14	14	2	13	E	0	81360.268	-0.046	82.1224
15	1	14	14	2	13	A	0	81361.540	0.035	82.1058
13	3	10	12	3	9	A	1	81396.573	-0.065	196.1254
36	8	28	36	7	29	A	1	81402.704	-0.102	323.8140
27	5	22	27	5	23	A	0	81437.303	0.253	141.0414
13	3	10	12	3	9	A	0	81459.716	-0.259	79.2841
13	3	10	12	3	9	E	0	81459.716	0.312	79.3007
15	2	14	14	2	13	A	1	81464.882	-0.048	198.9494
37	8	30	36	9	27	E	1	81492.462	-0.613	328.2923
15	2	14	14	2	13	E	0	81530.957	-0.013	82.1224
15	2	14	14	2	13	A	0	81532.720	-0.013	82.1058
15	1	14	14	1	13	A	1	81593.000	-0.027	198.9398
20	2	18	20	1	19	A	0	81634.996	-0.110	100.9085
27	7	21	27	6	22	E	0	81649.533	-0.099	143.9816
15	1	14	14	1	13	E	1	81659.925	-0.057	198.4080
15	1	14	14	1	13	E	0	81665.458	0.011	82.1122
15	1	14	14	1	13	A	0	81667.603	0.021	82.0956
27	7	21	27	6	22	A	0	81672.897	-0.047	143.9684
22	3	19	22	2	20	A	0	81677.843	-0.016	111.5494
16	0	16	15	1	15	A	1	81685.855	-0.015	199.4742
16	1	16	15	1	15	A	1	81687.868	-0.025	199.4742
16	0	16	15	0	15	A	1	81690.020	0.132	199.4741
16	1	16	15	0	15	A	1	81692.103	0.192	199.4741
35	10	25	35	9	26	E	0	81692.133	-0.150	204.4296
48	12	36	48	11	37	E	0	81694.414	0.244	324.2620
22	3	19	22	2	20	E	1	81699.303	-0.361	227.8616
16	0	16	15	1	15	E	1	81704.773	-0.104	198.9274
16	1	16	15	1	15	E	1	81706.994	-0.092	198.9274
16	0	16	15	0	15	E	1	81709.143	-0.088	198.9273
16	1	16	15	0	15	E	1	81711.238	-0.203	198.9273
48	12	36	48	11	37	A	0	81716.122	0.273	324.2518
16	0	16	15	1	15	A	0	81722.981	-0.330	82.6183
16	0	16	15	1	15	E	0	81722.981	0.245	82.6353
16	1	16	15	1	15	A	0	81725.197	-0.297	82.6183
16	1	16	15	1	15	E	0	81725.197	0.287	82.6353
16	0	16	15	0	15	E	0	81727.233	0.197	82.6352
16	1	16	15	0	15	A	0	81729.511	-0.300	82.6182
16	1	16	15	0	15	E	0	81729.511	0.301	82.6352
15	2	14	14	1	13	A	1	81753.919	-0.053	198.9398
35	10	25	35	9	26	A	0	81769.943	0.089	204.4176
20	3	18	20	2	19	E	0	81795.712	-0.134	100.9250
15	2	14	14	1	13	E	1	81831.725	-0.087	198.4080
15	2	14	14	1	13	E	0	81836.038	-0.066	82.1122
15	2	14	14	1	13	A	0	81838.799	-0.010	82.0956
20	3	18	20	2	19	E	1	81895.774	0.274	217.2188
7	4	4	6	3	4	E	1	81897.936	0.166	183.7423
21	5	16	20	6	14	E	1	82009.961	0.522	227.0921
27	5	22	27	4	23	E	1	82031.972	-0.158	257.3280
33	7	26	33	6	27	E	1	82093.323	0.045	299.3182
27	5	22	27	4	23	A	1	82117.138	-0.003	257.7679
27	5	22	27	4	23	A	0	82202.268	0.128	141.0159
27	5	22	27	4	23	E	0	82207.544	0.120	141.0296
22	8	14	22	7	15	A	0	82220.579	-0.319	121.6260
39	13	26	38	14	24	E	0	82254.082	0.326	247.0144
22	8	14	22	7	15	E	0	82285.828	-0.340	121.6391
25	8	18	25	7	19	A	1	82323.840	0.291	252.7552
14	12	2	13	12	1	E	1	82333.331	-0.015	224.8143
14	12	3	13	12	2	E	1	82336.136	-0.049	224.1878
14	13	1	13	13	0	A	0	82338.063	0.160	113.0023
14	13	2	13	13	1	A	0	82338.063	0.160	113.0023
14	13	1	13	13	0	E	0	82338.063	-0.061	113.0133
14	13	2	13	13	1	E	0	82338.063	-0.061	112.9933
14	12	3	13	12	2	A	1	82343.332	-0.028	224.5934
14	12	2	13	12	1	A	1	82343.332	-0.028	224.5934
14	12	2	13	12	1	A	0	82386.454	0.130	108.0400
14	12	3	13	12	2	A	0	82386.454	0.130	108.0400
14	12	2	13	12	1	E	0	82386.454	-0.140	108.0529
14	12	3	13	12	2	E	0	82386.454	-0.140	108.0332
14	11	3	13	11	2	E	1	82392.780	-0.066	220.2200
14	11	4	13	11	3	E	1	82398.929	-0.007	219.6113
14	11	3	13	11	2	A	1	82406.254	-0.039	220.0711
14	11	4	13	11	3	A	1	82406.254	-0.039	220.0711
24	5	20	24	4	21	A	1	82414.172	0.086	239.9837
26	8	19	26	7	20	A	1	82442.807	0.261	257.9851
14	11	3	13	11	2	A	0	82448.485	0.178	103.4764
14	11	4	13	11	3	A	0	82448.485	0.178	103.4764
14	11	3	13	11	2	E	0	82448.489	-0.143	103.4909
14	11	4	13	11	3	E	0	82448.489	-0.143	103.4718
14	10	4	13	10	3	E	1	82470.873	-0.043	216.0215
14	10	5	13	10	4	E	1	82481.618	-0.040	215.4389
22	4	19	22	3	20	E	0	82487.051	-0.151	111.5668
14	10	5	13	10	4	A	1	82488.807	-0.033	215.9469
14	10	4	13	10	3	A	1	82488.807	-0.033	215.9469
33	7	26	33	6	27	A	0	82499.458	0.229	183.0142

J'	K'_a	K'_c	J''	K''_a	K''_c	Sym.	$v_{t_{CH_3}}$	$\nu_{Obs.}$	$\nu_{Obs.} - \nu_{Calc.}$	E_{low}
22	4	19	22	3	20	A	0	82502.624	-0.046	111.5514
7	4	3	6	3	3	E	0	82507.122	0.020	67.5516
24	8	17	24	7	18	A	1	82518.760	0.195	247.7243
14	2	12	13	2	11	A	1	82526.242	-0.073	197.8518
14	10	4	13	10	3	A	0	82529.914	0.165	99.3120
14	10	5	13	10	4	A	0	82529.914	0.165	99.3120
14	10	4	13	10	3	E	0	82529.914	-0.228	99.3280
14	10	5	13	10	4	E	0	82529.914	-0.228	99.3096
22	4	19	22	3	20	E	1	82536.270	-0.167	227.8636
22	4	19	22	2	20	A	0	82560.845	0.011	111.5494
14	9	5	13	9	4	E	1	82576.552	-0.019	212.2202
14	9	6	13	9	5	E	1	82594.191	-0.046	211.6718
14	9	6	13	9	5	A	1	82600.582	-0.044	212.2211
14	9	5	13	9	4	A	1	82600.582	-0.044	212.2211
14	9	5	13	9	4	A	0	82640.304	0.108	95.5477
14	9	6	13	9	5	A	0	82640.304	0.108	95.5477
14	9	5	13	9	4	E	0	82640.304	-0.089	95.5651
14	2	12	13	2	11	E	1	82647.891	-0.228	197.3230
14	2	12	13	2	11	E	0	82650.140	-0.022	81.0264
14	2	12	13	2	11	A	0	82654.622	-0.055	81.0098
8	3	5	7	2	6	A	0	82669.669	-0.127	67.8067
29	9	20	29	8	21	E	0	82674.450	-0.158	161.4141
24	5	20	24	3	21	A	1	82697.253	-0.062	239.9743
29	9	20	29	8	21	A	0	82723.777	-0.218	161.4017
14	8	6	13	8	5	E	1	82725.170	-0.034	208.8181
14	8	7	13	8	6	E	1	82753.732	-0.037	208.3117
14	8	6	13	8	5	A	1	82758.185	-0.021	208.8948
14	8	7	13	8	6	A	1	82758.185	-0.021	208.8948
21	8	13	21	7	14	A	1	82763.239	0.240	233.9048
14	8	6	13	8	5	A	0	82796.112	0.060	92.1853
14	8	7	13	8	6	A	0	82796.112	0.060	92.1853
14	8	6	13	8	5	E	0	82796.112	-0.071	92.2037
14	8	7	13	8	6	E	0	82797.265	0.169	92.1876
7	4	4	6	3	4	E	0	82821.001	-0.078	67.5320
24	5	20	24	4	21	E	0	82821.001	-0.078	123.2175
24	5	20	24	4	21	A	0	82834.993	-0.072	123.2029
26	6	21	26	5	22	A	1	82916.795	0.106	252.6046
31	9	23	30	10	20	A	0	82925.523	-0.079	173.6476
23	8	16	23	7	17	A	1	82937.438	0.313	242.8958
14	7	7	13	7	6	E	1	82945.276	-0.057	205.8184
27	8	20	27	7	21	A	1	82963.278	0.221	263.4099
14	7	8	13	7	7	A	1	82990.715	-0.023	205.9704
14	7	8	13	7	7	E	1	82992.479	-0.042	205.3616
14	7	7	13	7	6	A	1	82994.393	-0.056	205.9705
14	7	8	13	7	7	A	0	83026.300	0.039	89.2275
14	7	7	13	7	6	E	0	83028.062	0.042	89.2467
14	7	7	13	7	6	A	0	83029.689	-0.084	89.2276
30	6	24	30	5	25	A	0	83035.337	0.200	160.9621
30	6	24	30	5	25	E	0	83051.673	0.408	160.9751
14	4	11	13	4	10	A	1	83135.341	-0.116	199.6006
30	6	24	30	5	25	A	1	83137.975	-0.083	277.6836
24	5	20	24	3	21	A	0	83142.162	-0.171	123.1927
16	3	13	15	4	12	E	0	83183.652	0.136	88.5366
14	4	11	13	4	10	A	0	83192.366	0.267	82.7806
14	4	11	13	4	10	E	0	83192.366	-0.194	82.7961
26	6	21	26	5	22	E	1	83199.492	-0.693	252.1615
25	8	18	25	7	19	E	0	83200.862	-0.129	136.0435
25	8	18	25	7	19	A	0	83270.176	-0.186	136.0311
26	8	19	26	7	20	E	0	83292.024	-0.094	141.2766
14	6	8	13	6	7	E	1	83297.269	-0.021	203.2272
14	6	9	13	6	8	A	1	83334.403	0.029	203.4527
26	6	21	26	5	22	E	0	83334.403	-0.417	135.8626
23	8	15	23	7	16	E	1	83340.421	0.400	242.7855
26	8	19	26	7	20	A	0	83347.801	-0.193	141.2641
26	6	21	26	5	22	A	0	83349.707	-0.060	135.8487
14	6	9	13	6	8	A	0	83367.474	0.034	86.6797
14	6	9	13	6	8	E	1	83380.524	-0.041	202.8276
14	6	9	13	6	8	E	0	83396.930	0.034	86.6875
15	2	13	14	3	12	A	0	83400.135	0.118	83.8986
15	2	13	14	3	12	E	0	83404.698	-0.081	83.9147
14	6	8	13	6	7	A	1	83406.587	-0.019	203.4543
14	6	8	13	6	7	E	0	83409.079	0.049	86.7002
14	6	8	13	6	7	A	0	83436.497	0.025	86.6813
15	2	13	14	3	12	E	1	83438.035	0.176	200.2104
15	2	13	14	3	12	A	1	83440.603	0.049	200.7306
24	8	17	24	7	18	A	0	83496.126	-0.282	130.9976
14	5	10	13	5	9	A	1	83660.226	-0.073	201.3441
14	5	10	13	5	9	A	0	83697.065	0.051	84.5449
14	5	10	13	5	9	E	0	83713.099	0.043	84.5585
13	3	11	12	2	10	E	0	83735.246	-0.201	78.4290
14	4	11	13	4	10	E	1	83738.270	0.226	199.0484
13	3	11	12	2	10	A	0	83751.419	-0.261	78.4123
27	8	20	27	7	21	E	0	83770.278	-0.121	146.7051
23	8	16	23	7	17	E	0	83809.702	-0.268	126.1789
46	11	35	46	10	36	A	0	83812.523	-0.338	301.4557
27	8	20	27	7	21	A	0	83817.182	-0.171	146.6927
46	11	35	46	10	36	E	0	83838.186	-0.294	301.4655
21	8	13	21	7	14	A	0	83844.491	-0.280	117.1700
23	8	16	23	7	17	A	0	83936.022	-0.180	126.1668
21	8	13	21	7	14	E	0	83942.012	-0.161	117.1834
28	8	21	28	7	22	A	1	83960.768	0.272	269.0253
14	5	9	13	5	8	E	1	83977.608	-0.064	201.0581
20	8	12	20	7	13	A	1	84021.310	0.244	229.6725
14	5	10	13	5	9	E	1	84066.265	0.007	200.7243
26	6	21	26	4	22	A	1	84082.383	-0.019	252.5657
46	11	35	46	10	36	A	1	84089.592	-0.396	418.0155
21	8	14	21	7	15	A	1	84118.164	0.282	233.8553
37	12	25	36	13	24	A	1	84213.200	0.202	343.3487
5	5	0	4	4	0	E	1	84246.205	-0.592	183.2137
49	12	37	49	11	38	A	1	84310.279	0.102	451.0338
40	11	29	40	10	30	E	0	84333.224	0.594	246.7279
22	8	15	22	7	16	E	0	84338.223	-0.296	121.5532
28	7	22	28	6	23	A	1	84355.024	0.107	266.2115
18	4	14	17	5	13	A	1	84381.068	-0.065	213.7168
49	12	37	49	11	38	A	0	84398.932	-0.087	334.5025
49	12	37	49	11	38	E	0	84402.498	-0.082	334.5121
14	5	9	13	5	8	E	0	84472.522	0.067	84.5870
14	5	9	13	5	8	A	1	84474.765	-0.051	201.3714
14	5	9	13	5	8	A	0	84485.510	0.057	84.5713
22	8	15	22	7	16	A	0	84506.064	-0.228	121.5413
26	6	21	26	4	22	A	0	84609.263	-0.187	135.8067
28	8	21	28	7	22	E	0	84715.224	-0.110	152.3248
20	8	13	20	7	14	A	1	84751.396	0.240	229.6461
28	8	21	28	7	22	A	0	84755.454	-0.121	152.3124
28	7	22	28	6	23	E	0	84847.770	-0.026	149.4946
28	7	22	28	6	23	A	0	84867.678	-0.058	149.4816

J'	K_a'	K_c'	J''	K_a''	K_c''	Sym.	$v_{t_{CH_3}}$	$\nu_{Obs.}$	$\nu_{Obs.} - \nu_{Calc.}$	E_{low}
21	8	14	21	7	15	E	0	84937.803	-0.284	117.1341
19	8	11	19	7	12	A	1	84980.843	0.236	225.6583
43	10	33	43	9	34	A	0	85062.781	-0.202	270.4440
20	8	12	20	7	13	A	0	85076.022	-0.262	112.9372
23	6	17	22	7	16	A	0	85096.924	-0.361	121.5413
43	10	33	43	9	34	E	0	85100.837	-0.161	270.4541
20	8	13	20	7	14	E	1	85100.837	0.110	229.0514
21	8	14	21	7	15	A	0	85135.757	-0.238	117.1228
20	8	12	20	7	13	E	0	85179.658	-0.197	112.9513
19	8	12	19	7	13	A	1	85355.005	0.273	225.6450
29	8	22	29	7	23	A	1	85490.415	0.289	274.8267
43	10	33	43	9	34	A	1	85542.933	-0.521	387.0321
20	8	13	20	7	14	E	0	85568.111	-0.255	112.9226
25	4	21	25	3	22	E	1	85689.768	-0.357	244.2082
25	4	21	25	3	22	E	0	85729.452	0.090	127.9099
25	4	21	25	3	22	A	0	85736.394	0.019	127.8954
20	8	13	20	7	14	A	0	85770.622	-0.216	112.9121
15	3	13	14	3	12	A	1	85860.814	-0.025	200.7306
19	8	12	19	7	13	E	1	85892.114	-0.797	225.0435
15	3	13	14	3	12	E	0	85941.867	-0.043	83.9147
15	3	13	14	3	12	A	0	85943.933	-0.024	83.8986
15	3	13	14	3	12	E	1	85969.485	-0.052	200.2104
19	8	11	19	7	12	A	0	86017.507	-0.132	108.9223
19	8	11	19	7	12	E	0	86099.125	-0.180	108.9376
21	3	19	21	2	20	A	1	86122.213	-0.134	221.4481
29	8	22	29	7	23	E	0	86185.989	-0.099	158.1312
19	8	12	19	7	13	E	0	86193.550	-0.208	108.9188
29	8	22	29	7	23	A	0	86220.573	-0.112	158.1189
14	3	12	13	2	11	A	1	86304.838	-0.190	197.8518
17	8	9	17	7	10	A	1	86304.838	-0.190	218.2682
16	1	15	15	2	14	A	1	86350.366	-0.004	201.6668
14	4	10	13	4	9	E	1	86366.967	-0.323	199.3804
19	8	12	19	7	13	A	0	86372.804	-0.258	108.9096
16	1	15	15	2	14	E	1	86404.466	-0.186	201.1376
16	1	15	15	2	14	E	0	86412.811	-0.050	84.8419
16	1	15	15	2	14	A	0	86414.376	0.043	84.8254
23	3	20	23	2	21	A	1	86423.826	-0.161	232.5566
28	9	19	28	8	20	E	0	86428.718	-0.118	155.4174
16	2	15	15	2	14	A	1	86438.561	-0.035	201.6668
28	9	19	28	8	20	A	0	86455.023	-0.231	155.4053
16	2	15	15	2	14	E	1	86499.497	-0.117	201.1376
16	2	15	15	2	14	E	0	86506.811	-0.013	84.8419
16	2	15	15	2	14	A	0	86508.624	-0.011	84.8254
16	1	15	15	1	14	A	1	86511.257	-0.057	201.6614
21	2	19	21	1	20	E	0	86529.644	-0.194	104.6376
16	1	15	15	1	14	E	1	86576.391	-0.093	201.1318
16	1	15	15	1	14	E	0	86583.528	0.011	84.8363
16	1	15	15	1	14	A	0	86585.564	0.003	84.8197
14	3	12	13	2	11	E	0	86591.451	-0.233	81.0264
16	2	15	15	1	14	A	1	86599.503	-0.038	201.6614
35	10	25	35	9	26	E	1	86616.451	0.160	320.7448
40	9	31	40	8	32	E	1	86624.575	-0.265	357.7620
21	3	19	21	2	20	E	0	86630.063	-0.027	104.6377
17	0	17	16	1	16	A	1	86643.631	-0.105	202.1990
17	1	17	16	1	16	A	1	86644.702	-0.046	202.1990
17	0	17	16	0	16	A	1	86645.741	-0.018	202.1990
17	0	17	16	1	16	E	1	86662.551	-0.132	201.6529
17	1	17	16	1	16	E	1	86663.720	-0.077	201.6529
17	0	17	16	0	16	E	1	86664.848	-0.045	201.6528
17	1	17	16	0	16	E	1	86665.998	-0.009	201.6528
34	10	24	34	9	25	E	0	86672.007	0.154	197.0998
16	2	15	15	1	14	E	0	86677.517	0.036	84.8363
16	2	15	15	1	14	A	0	86679.878	0.015	84.8197
17	0	17	16	1	16	E	0	86681.872	-0.114	85.3614
17	1	17	16	1	16	E	0	86683.080	0.001	85.3614
17	1	17	16	1	16	A	0	86683.900	0.240	85.3444
17	0	17	16	0	16	E	0	86683.900	-0.261	85.3613
17	0	17	16	0	16	A	0	86684.846	0.101	85.3443
17	1	17	16	0	16	A	0	86686.066	0.223	85.3443
34	10	24	34	9	25	A	0	86744.161	0.049	197.0882
18	8	10	18	7	11	A	0	86746.988	-0.210	105.1210
18	8	10	18	7	11	E	0	86791.695	-0.156	105.1378
23	3	20	23	3	21	A	0	86833.909	-0.127	115.7561
23	3	20	23	2	21	E	0	86851.514	-0.110	115.7704
23	3	20	23	2	21	A	0	86865.828	-0.171	115.7551
23	4	20	23	2	21	A	1	86910.334	-0.045	232.5566
18	8	11	18	7	12	A	0	86919.639	-0.194	105.1149
14	4	10	13	4	9	A	1	87031.846	-0.028	199.8298
14	4	10	13	4	9	A	0	87038.794	0.075	83.0052
14	4	10	13	4	9	E	0	87040.764	0.071	83.0211
25	5	21	25	4	22	A	1	87158.509	0.022	244.6772
40	9	31	40	8	32	A	0	87196.742	-0.014	241.4759
15	2	13	14	2	12	A	1	87219.613	-0.072	200.6046
40	9	31	40	8	32	E	0	87237.042	0.004	241.4864
14	3	11	13	3	10	E	1	87285.476	-0.206	198.3208
14	3	11	13	3	10	A	1	87303.889	-0.079	198.8405
17	8	9	17	7	10	A	0	87320.614	-0.239	101.5300
27	6	22	27	5	23	A	1	87323.944	0.044	257.7914
17	8	9	17	7	10	E	0	87333.429	-0.096	101.5480
23	4	20	23	3	21	E	0	87342.625	-0.125	115.7714
15	2	13	14	2	12	E	0	87346.271	-0.032	83.7833
15	2	13	14	2	12	E	1	87349.297	-0.218	200.0799
15	2	13	14	2	12	A	0	87350.924	-0.021	83.7668
23	4	20	23	3	21	A	0	87359.076	-0.083	115.7561
23	4	20	23	2	21	E	0	87374.459	-0.100	115.7704
14	3	11	13	3	10	E	0	87390.598	-0.001	82.0178
14	3	11	13	3	10	A	0	87392.343	0.018	82.0013
17	8	10	17	7	11	A	0	87399.887	-0.255	101.5273
8	4	5	7	3	4	E	0	87541.694	0.022	68.9573
16	8	9	16	7	10	E	1	87541.694	0.327	214.2769
19	8	11	19	7	12	E	1	87585.796	0.915	225.4830
16	8	9	16	7	10	E	0	87722.737	-0.201	98.1513
27	6	22	27	5	23	E	0	87734.613	-0.046	141.0551
27	6	22	27	5	23	A	0	87748.514	-0.026	141.0414
25	5	21	25	3	22	E	0	87756.245	-0.072	127.9099
16	8	8	16	7	9	E	0	87770.069	-0.252	98.1656
25	5	21	25	3	22	A	0	87771.271	-0.093	127.8954
16	8	8	16	7	9	A	0	87777.011	-0.118	98.1470
16	8	9	16	7	10	A	0	87811.213	-0.164	98.1458
15	8	8	15	7	9	E	1	87906.536	-0.653	211.1013
27	7	21	27	5	22	A	0	87984.202	-0.231	143.7578
15	8	8	15	7	9	E	0	88077.883	-0.228	94.9745
28	5	23	28	5	24	A	0	88207.293	0.073	146.4000
15	14	1	14	14	0	A	0	88214.268	0.061	121.1080
15	14	2	14	14	1	A	0	88214.268	0.061	121.1080
30	8	23	30	7	24	E	0	88215.968	-0.054	164.1198

J'	K'_a	K'_c	J''	K''_a	K''_c	Sym.	$v_{t_{CH_3}}$	$\nu_{Obs.}$	$\nu_{Obs.} - \nu_{Calc.}$	E_{low}
15	13	2	14	13	1	A	0	88261.729	0.136	115.7488
15	13	3	14	13	2	A	0	88261.729	0.136	115.7488
15	13	2	14	13	1	E	0	88261.729	-0.132	115.7598
15	13	3	14	13	2	E	0	88261.729	-0.132	115.7398
15	12	3	14	12	2	A	1	88275.467	-0.064	227.3401
15	12	4	14	12	3	A	1	88275.467	-0.064	227.3401
15	12	3	14	12	2	A	0	88321.178	0.176	110.7882
15	12	4	14	12	3	A	0	88321.178	0.176	110.7882
15	12	3	14	12	2	E	0	88321.178	-0.149	110.8010
15	12	4	14	12	3	E	0	88321.178	-0.149	110.7813
15	11	4	14	11	3	E	1	88336.014	-0.053	222.9683
15	11	5	14	11	4	E	1	88344.310	-0.155	222.3598
15	11	4	14	11	3	A	1	88352.781	-0.015	222.8199
15	11	5	14	11	4	A	1	88352.781	-0.015	222.8199
15	11	4	14	11	3	A	0	88397.300	0.146	106.2266
15	11	5	14	11	4	A	0	88397.300	0.146	106.2266
15	11	4	14	11	3	E	0	88397.300	-0.117	106.2411
6	3	4	5	1	5	E	1	88414.191	0.243	180.7931
14	8	6	14	7	7	E	0	88414.191	0.022	92.0163
15	10	5	14	10	4	E	1	88432.189	0.023	218.7724
27	6	22	27	4	23	E	1	88432.189	0.057	257.3280
15	10	6	14	10	5	E	1	88446.188	-0.075	218.1902
15	10	6	14	10	5	A	1	88454.287	-0.026	218.6984
15	10	5	14	10	4	A	1	88454.287	-0.026	218.6984
15	10	5	14	10	4	A	0	88497.458	0.093	102.0648
15	10	6	14	10	5	A	0	88497.458	0.093	102.0648
15	10	5	14	10	4	E	0	88497.458	-0.158	102.0809
29	7	23	29	6	24	E	0	88517.294	-0.005	155.1786
29	7	23	29	6	24	A	0	88534.347	-0.071	155.1657
15	9	6	14	9	5	E	1	88562.398	-0.048	214.9746
15	9	7	14	9	6	E	1	88584.951	-0.114	214.4269
15	9	7	14	9	6	A	1	88592.062	-0.010	214.9763
15	9	6	14	9	5	A	1	88592.062	-0.010	214.9763
8	4	4	7	3	5	A	1	88626.404	0.149	185.7401
15	9	6	14	9	5	A	0	88633.593	0.070	98.3043
15	9	7	14	9	6	A	0	88633.593	0.070	98.3043
15	9	6	14	9	5	E	0	88633.593	-0.144	98.3216
15	9	7	14	9	6	E	0	88634.584	0.130	98.3043
28	5	23	28	4	24	E	0	88665.334	0.063	146.3983
21	4	17	20	5	15	E	1	88728.933	0.007	225.4277
15	8	7	14	8	6	E	1	88746.174	-0.039	211.5775
15	8	8	14	8	7	E	1	88782.259	-0.092	211.0721
15	8	7	14	8	6	A	1	88786.796	-0.083	211.6553
15	8	8	14	8	7	A	1	88786.796	-0.083	211.6553
15	8	7	14	8	6	A	0	88826.332	-0.076	94.9470
15	8	7	14	8	6	E	0	88826.332	-0.028	94.9654
15	8	8	14	8	7	E	0	88827.577	0.062	94.9495
12	8	4	12	7	5	E	0	88830.205	0.105	86.6792
15	4	12	14	4	11	A	1	88835.352	-0.037	202.3737
6	5	2	5	4	1	A	0	88847.337	-0.108	67.7208
6	5	1	5	4	1	E	0	88849.216	-0.147	67.7408
12	8	4	12	7	5	A	0	88853.747	0.070	86.6600
15	4	12	14	4	11	A	0	88902.541	-0.151	85.5556
15	4	12	14	4	11	E	0	88902.541	0.226	85.5711
15	7	8	14	7	7	E	1	89019.679	-0.107	208.5852
15	7	9	14	7	8	A	1	89073.597	0.008	208.7387
15	7	9	14	7	8	E	1	89079.330	-0.028	208.1299
15	7	8	14	7	7	A	1	89083.066	-0.047	208.7389
15	7	9	14	7	8	A	0	89110.181	0.018	91.9970
15	7	8	14	7	7	E	0	89114.729	0.004	92.0163
15	7	9	14	7	8	E	0	89116.426	-0.006	92.0019
15	7	8	14	7	7	A	0	89119.185	0.004	91.9972
37	8	29	37	7	30	A	0	89242.589	0.125	214.5671
15	4	12	14	4	11	E	1	89260.758	0.180	201.8416
50	12	38	50	11	39	E	0	89391.743	-0.547	344.9075
15	6	9	14	6	8	E	1	89463.303	-0.074	206.0057
15	6	10	14	6	9	A	1	89477.934	-0.094	206.2324
16	2	14	15	3	13	A	1	89498.129	-0.067	203.5946
16	2	14	15	3	13	E	0	89506.172	0.177	86.7815
15	6	10	14	6	9	A	0	89512.567	0.081	89.4605
27	9	18	27	8	19	A	0	89514.206	-0.193	149.6545
27	9	18	27	8	19	E	0	89523.476	-0.136	149.6664
15	6	10	14	6	9	E	0	89555.777	0.028	89.4693
15	6	10	14	6	9	E	1	89568.484	-0.039	205.6088
15	6	9	14	6	8	E	0	89619.009	0.060	89.4825
15	6	9	14	6	8	A	1	89631.883	-0.049	206.2365
15	3	13	14	2	12	A	1	89639.872	-0.098	200.6046
15	6	9	14	6	8	A	0	89659.771	0.055	89.4644
15	5	11	14	5	10	A	1	89743.362	-0.037	204.1347
15	5	11	14	5	10	A	0	89785.807	0.042	87.3368
15	5	11	14	5	10	E	0	89793.769	0.044	87.3509
15	3	13	14	2	12	E	0	89883.248	-0.185	83.7833
15	3	13	14	2	12	A	0	89894.772	-0.113	83.7668
31	6	25	31	5	26	A	0	90261.917	0.147	167.0036
31	6	25	31	5	26	A	1	90265.418	-0.103	283.7167
31	6	25	31	5	26	E	0	90273.260	0.189	167.0163
15	5	11	14	5	10	E	1	90357.252	0.106	203.5285
34	7	27	34	6	28	A	0	90415.844	0.167	189.7367
15	5	10	14	5	9	E	1	90417.753	-0.109	203.8593
34	7	27	34	6	28	E	0	90439.202	0.215	189.7485
47	11	36	47	10	37	A	0	90755.734	-0.571	311.1346
47	11	36	47	10	37	E	0	90797.934	-0.502	311.1437
31	8	24	31	7	25	E	0	90809.968	-0.040	170.2865
31	8	24	31	7	25	A	0	90835.004	0.012	170.2745
22	2	20	22	1	21	A	1	90876.200	-0.192	225.3235
16	3	14	15	3	13	A	1	90995.014	-0.079	203.5946
16	3	14	15	3	13	E	0	91082.866	-0.018	86.7815
16	3	14	15	3	13	A	0	91085.220	-0.024	86.7654
16	3	14	15	3	13	E	1	91097.240	-0.137	203.0780
15	5	10	14	5	9	E	0	91139.273	0.025	87.4047
15	5	10	14	5	9	A	1	91142.140	-0.094	204.1892
15	5	10	14	5	9	A	0	91143.531	0.075	87.3894
29	7	23	29	5	24	E	0	91194.781	-0.096	155.0893
22	5	17	21	6	15	E	1	91225.176	0.214	231.3898
26	4	22	26	3	23	E	0	91283.840	0.010	132.7758
26	4	22	26	3	23	A	0	91293.770	0.004	132.7615
33	10	23	33	9	24	E	0	91345.032	0.153	190.0190
17	2	16	16	2	15	A	1	91403.196	-0.045	204.5501
33	10	23	33	9	24	A	0	91404.316	0.223	190.0077
22	2	20	22	1	21	E	0	91411.169	0.242	108.5157
17	1	16	16	2	15	E	1	91412.594	0.021	204.0229
17	1	16	16	2	15	E	0	91422.265	0.019	87.7275
17	1	16	16	2	15	A	0	91423.931	0.044	87.7111
22	2	20	22	1	21	A	0	91430.806	-0.135	108.4996
17	1	16	16	1	15	A	1	91443.683	-0.057	204.5471
17	2	16	16	2	15	E	1	91464.196	-0.411	204.0229

J'	K'_a	K'_c	J''	K''_a	K''_c	Sym.	$v_{t_{\mathrm{CH_3}}}$	$\nu_{\mathrm{Obs.}}$	$\nu_{\mathrm{Obs.}} - \nu_{\mathrm{Calc.}}$	E_{low}
35	8	28	34	9	25	E	1	91464.196	-0.411	313.4461
17	2	16	16	2	15	E	0	91473.263	-0.039	87.7275
17	2	16	16	2	15	A	0	91475.113	-0.026	87.7111
17	2	16	16	1	15	A	1	91491.442	-0.025	204.5471
17	1	16	16	1	15	E	1	91507.429	-0.105	204.0197
17	1	16	16	1	15	E	0	91516.190	-0.020	87.7244
17	1	16	16	1	15	A	0	91518.198	0.008	87.7079
17	2	16	16	1	15	E	0	91567.234	-0.032	87.7244
17	2	16	16	1	15	A	0	91569.324	-0.118	87.7079
18	0	18	17	0	17	A	1	91601.657	-0.021	205.0892
18	1	18	17	1	17	A	1	91601.657	-0.021	205.0892
18	1	18	17	1	17	E	1	91620.502	-0.099	204.5436
18	0	18	17	0	17	E	1	91620.502	-0.099	204.5436
18	1	18	17	1	17	A	0	91641.678	0.043	88.2358
18	0	18	17	0	17	E	0	91641.678	0.073	88.2528
17	3	14	16	4	13	E	0	91856.307	0.287	91.6886
44	12	32	44	11	33	A	0	91891.805	1.299	285.0467
16	2	14	15	2	13	A	1	91918.456	-0.026	203.5139
26	9	17	26	8	18	A	0	91932.492	-0.172	144.1436
24	3	21	24	3	22	A	0	91935.207	-0.104	120.1261
24	3	21	24	2	22	E	0	91936.496	-0.014	120.1407
24	3	21	24	2	22	A	0	91952.709	-0.001	120.1255
28	6	23	28	5	24	A	1	91956.869	0.071	263.1442
26	9	17	26	8	18	E	0	91986.411	-0.095	144.1555
26	5	22	26	4	23	A	1	91996.737	0.033	249.5359
16	2	14	15	2	13	E	0	92043.104	-0.021	86.6968
16	2	14	15	2	13	A	0	92047.674	0.066	86.6805
16	2	14	15	2	13	E	1	92047.675	-0.382	202.9935
26	5	22	26	3	23	A	1	92089.181	0.233	249.5328
30	7	24	30	6	25	A	1	92160.946	0.093	277.7356
24	4	21	24	3	22	E	0	92224.952	-0.152	120.1412
24	4	21	24	3	22	A	0	92242.386	-0.193	120.1261
24	4	21	24	2	22	A	0	92259.879	-0.100	120.1255
24	4	21	24	3	22	E	1	92296.335	-0.708	236.4374
28	6	23	28	5	24	E	1	92308.428	-0.643	262.7127
28	6	23	28	5	24	E	0	92368.975	0.012	146.4135
28	6	23	28	4	24	A	1	92376.104	0.381	263.1302
28	6	23	28	5	24	A	0	92382.273	-0.036	146.4000
21	5	16	20	6	15	A	1	92417.373	-0.157	227.2237
26	5	22	26	4	23	E	0	92437.499	-0.140	132.7792
26	5	22	26	4	23	A	0	92452.321	-0.091	132.7649
26	5	22	26	3	23	A	0	92553.254	-0.196	132.7615
30	7	24	30	6	25	E	0	92592.247	0.008	161.0313
30	7	24	30	6	25	A	0	92607.172	0.023	161.0186
25	9	16	25	8	17	A	1	92608.958	0.476	255.5615
27	9	19	27	8	20	A	1	92775.788	0.388	266.1772
30	9	22	30	8	23	E	0	92813.749	0.004	167.0624
28	6	23	28	4	24	E	0	92824.021	0.030	146.3983
28	6	23	28	4	24	A	0	92839.435	-0.092	146.3848
29	9	21	29	8	22	E	0	92856.773	-0.009	161.0061
15	3	12	14	3	11	A	1	92872.815	-0.050	201.7526
30	9	22	30	8	23	A	0	92873.730	0.056	167.0512
15	3	12	14	3	11	E	1	92921.196	-0.225	201.2323
29	9	21	29	8	22	A	0	92929.816	-0.090	160.9949
15	3	12	14	3	11	E	0	92985.813	-0.005	84.9329
15	3	12	14	3	11	A	0	92988.914	0.001	84.9164
26	9	18	26	8	19	E	1	93107.489	-0.800	260.1992
31	9	23	31	8	24	E	0	93162.818	-0.070	173.3156
28	9	20	28	8	21	E	0	93198.861	-0.017	155.1506
31	9	23	31	8	24	A	0	93213.708	0.027	173.3044
44	10	34	44	9	35	A	0	93224.090	-0.288	279.4076
44	10	34	44	9	35	E	0	93270.776	-0.271	279.4171
28	9	20	28	8	21	A	0	93292.915	-0.022	155.1396
15	4	11	14	4	10	E	1	93376.125	-0.273	202.2613
16	3	14	15	2	13	A	1	93415.444	0.065	203.5139
32	8	25	32	7	26	A	1	93424.452	0.235	293.3048
16	3	14	15	2	13	E	0	93619.897	-0.117	86.6968
16	3	14	15	2	13	A	0	93628.977	-0.207	86.6805
16	3	14	15	2	13	E	1	93628.981	-0.074	202.9935
27	9	19	27	8	20	E	0	93745.189	-0.108	149.4994
25	9	16	25	8	17	A	0	93812.366	-0.138	138.8657
44	10	34	44	9	35	A	1	93827.885	-0.525	395.9793
15	4	11	14	4	10	A	1	93833.330	0.006	202.7329
15	4	11	14	4	10	A	0	93856.307	0.035	85.9085
15	4	11	14	4	10	E	0	93858.129	0.058	85.9245
27	9	19	27	8	20	A	0	93872.032	-0.050	149.4885
25	9	16	25	8	17	E	0	93903.785	-0.009	138.8779
32	8	25	32	7	26	E	0	93945.064	0.051	176.6276
32	8	25	32	7	26	A	0	93965.997	0.065	176.6157
32	9	24	32	8	25	E	0	93985.689	0.053	179.7612
32	9	24	32	8	25	A	0	94029.258	0.004	179.7500
16	15	2	15	15	1	E	1	94031.707	-0.072	246.0171
16	15	1	15	15	0	E	1	94033.223	-0.042	246.6452
7	5	2	6	4	3	A	1	94044.459	0.247	185.7289
7	5	3	6	4	3	E	1	94053.324	0.004	185.0928
16	14	3	15	14	2	E	1	94078.245	-0.083	240.2346
16	14	2	15	14	1	E	1	94078.245	-0.083	240.8708
16	14	2	15	14	1	A	1	94086.697	-0.061	240.5168
16	14	3	15	14	2	A	1	94086.697	-0.061	240.5168
16	15	1	15	15	0	A	0	94090.273	0.081	129.8065
16	15	2	15	15	1	A	0	94090.273	0.081	129.8065
16	15	1	15	15	0	E	0	94090.273	-0.131	129.8133
16	15	2	15	15	1	E	0	94090.273	-0.131	129.7936
24	9	15	24	8	16	A	1	94102.999	0.348	250.5102
16	13	3	15	13	2	E	1	94133.278	-0.094	235.4904
16	13	4	15	13	3	E	1	94136.159	-0.078	234.8548
16	14	2	15	14	1	A	0	94136.894	0.143	124.0505
16	14	3	15	14	2	A	0	94136.894	0.143	124.0505
16	14	2	15	14	1	E	0	94136.894	-0.121	124.0594
16	14	3	15	14	2	E	0	94136.894	-0.121	124.0394
16	13	4	15	13	3	A	1	94145.072	-0.034	235.2013
16	13	3	15	13	2	A	1	94145.072	-0.034	235.2013
38	8	30	38	8	31	E	1	94158.608	0.282	338.5217
21	5	16	20	6	15	E	1	94160.865	-0.125	226.6868
16	13	3	15	13	2	A	0	94194.249	0.157	118.6929
16	13	4	15	13	3	A	0	94194.249	0.157	118.6929
16	13	3	15	13	2	E	0	94194.249	-0.165	118.7039
16	13	4	15	13	3	E	0	94194.249	-0.165	118.6839
16	12	4	15	12	3	E	1	94202.510	-0.113	230.5047
16	12	5	15	12	4	E	1	94208.950	0.023	229.8785
16	12	4	15	12	3	A	1	94218.159	-0.058	230.2846
16	12	5	15	12	4	A	1	94218.159	-0.058	230.2846
25	9	17	25	8	18	A	1	94237.483	0.418	255.5013
29	5	24	29	5	25	A	1	94260.741	0.080	268.6621
16	12	4	15	12	3	E	0	94266.286	-0.083	113.7471
16	11	5	15	11	4	E	1	94291.332	-0.071	225.9149

J'	K'_a	K'_c	J''	K''_a	K''_c	Sym.	$v_{t_{CH_3}}$	$\nu_{Obs.}$	$\nu_{Obs.} - \nu_{Calc.}$	E_{low}
16	11	6	15	11	5	E	1	94302.435	-0.072	225.3067
16	11	5	15	11	4	A	1	94311.850	-0.056	225.7670
16	11	6	15	11	5	A	1	94311.850	-0.056	225.7670
16	11	5	15	11	4	A	0	94358.632	0.162	109.1752
16	11	6	15	11	5	A	0	94358.632	0.162	109.1752
16	11	5	15	11	4	E	0	94358.632	-0.305	109.1898
16	11	6	15	11	5	E	0	94358.632	-0.305	109.1706
25	9	17	25	8	18	E	1	94385.616	-0.677	254.9459
16	10	6	15	10	5	E	1	94408.182	-0.034	221.7222
26	9	18	26	8	19	E	0	94414.859	-0.074	144.0549
16	4	13	15	4	12	A	1	94418.679	-0.028	205.3369
16	10	7	15	10	6	E	1	94426.181	-0.048	221.1405
16	10	7	15	10	6	A	1	94435.145	-0.071	221.6489
16	10	6	15	10	5	A	1	94435.145	-0.071	221.6489
16	10	6	15	10	5	A	0	94480.349	0.103	105.0168
16	10	7	15	10	6	A	0	94480.349	0.103	105.0168
16	10	6	15	10	5	E	0	94480.349	-0.175	105.0329
16	4	13	15	4	12	E	0	94496.370	-0.027	88.5366
16	4	13	15	4	12	A	0	94497.532	0.062	88.5211
29	5	24	29	4	25	A	1	94506.344	0.025	268.6539
16	9	7	15	9	6	E	1	94566.561	-0.328	217.9288
26	9	18	26	8	19	A	0	94583.363	-0.077	144.0443
16	9	8	15	9	7	E	1	94595.255	-0.030	217.3818
16	9	8	15	9	7	A	1	94602.944	0.003	217.9314
16	9	7	15	9	6	A	1	94602.944	0.003	217.9314
16	9	7	15	9	6	A	0	94646.145	0.084	101.2608
16	9	8	15	9	7	A	0	94646.145	0.084	101.2608
16	9	7	15	9	6	E	0	94646.145	-0.148	101.2781
16	9	8	15	9	7	E	0	94647.320	0.125	101.2609
7	5	3	6	4	3	E	0	94648.922	-0.166	68.9131
16	4	13	15	4	12	E	1	94708.308	0.097	204.8191
26	9	17	26	8	18	E	1	94718.597	0.264	260.6624
29	5	24	29	4	25	E	0	94761.963	0.094	151.9284
29	5	24	29	4	25	A	0	94765.188	0.066	151.9150
16	8	8	15	8	7	E	1	94791.414	-0.029	214.5377
16	8	9	15	8	8	E	1	94836.421	-0.037	214.0335
16	8	9	15	8	8	A	1	94840.427	-0.046	214.6169
16	8	8	15	8	7	A	1	94841.476	-0.071	214.6169
16	8	8	15	8	7	A	0	94881.871	-0.218	97.9100
16	8	8	15	8	7	E	0	94881.871	0.157	97.9284
16	8	9	15	8	8	E	0	94883.247	0.095	97.9124
21	1	20	21	0	21	E	0	94890.470	-0.277	101.4724
21	2	20	21	1	21	E	0	94894.317	-0.389	101.4724
21	1	20	21	0	21	A	0	94914.343	-0.294	101.4554
21	2	20	21	1	21	A	0	94918.398	-0.217	101.4554
45	10	35	45	10	36	E	1	94941.488	-0.376	404.9973
24	9	16	24	8	17	A	1	95013.927	0.471	250.4769
41	9	32	41	8	33	E	1	95109.609	-0.190	366.0210
16	7	9	15	7	8	E	1	95127.894	-0.049	211.5545
25	9	17	25	8	18	E	0	95152.317	-0.083	138.8188
16	7	10	15	7	9	A	1	95188.501	-0.057	211.7099
16	7	10	15	7	9	E	1	95202.275	-0.005	211.1013
17	2	15	16	3	14	E	0	95208.969	-0.053	89.8197
16	7	9	15	7	8	A	1	95211.157	-0.036	211.7104
16	7	10	15	7	9	A	0	95226.002	0.060	94.9694
16	7	9	15	7	8	E	0	95237.196	-0.000	94.9888
16	7	10	15	7	9	E	0	95238.477	0.152	94.9745
16	7	9	15	7	8	A	0	95247.466	0.079	94.9699
24	9	15	24	8	16	A	0	95272.470	-0.095	133.8142
33	9	25	33	8	26	E	0	95344.029	0.063	186.3946
25	9	17	25	8	18	A	0	95355.777	-0.054	138.8087
24	9	15	24	8	16	E	0	95377.854	-0.013	133.8269
33	9	25	33	8	26	A	0	95381.365	0.011	186.3835
32	10	22	32	9	23	E	0	95424.669	0.186	183.1898
32	10	22	32	9	23	A	0	95461.452	0.137	183.1788
9	4	5	8	3	6	A	0	95628.020	-0.305	70.4729
16	6	11	15	6	10	A	1	95644.202	-0.038	209.2171
34	8	27	33	9	24	A	1	95677.523	0.017	306.6680
16	6	11	15	6	10	A	0	95680.675	0.058	92.4463
16	6	10	15	6	9	E	1	95685.455	-0.036	208.9899
23	2	21	23	1	22	A	1	95703.311	-0.074	229.3643
34	8	27	33	9	24	A	0	95706.592	0.243	190.0077
16	6	11	15	6	10	E	0	95722.856	0.042	92.4566
23	3	21	23	2	22	A	1	95731.558	-0.092	229.3644
23	9	15	23	8	16	A	1	95760.599	0.436	245.6623
41	9	32	41	8	33	A	0	95765.149	-0.025	249.7324
16	5	12	15	5	11	A	1	95778.239	-0.058	207.1283
41	9	32	41	8	33	E	0	95807.008	0.028	249.7423
16	6	11	15	6	10	E	1	95813.161	-0.000	208.5965
16	5	12	15	5	11	A	0	95828.295	0.046	90.3317
16	5	12	15	5	11	E	0	95832.298	0.038	90.3461
24	9	16	24	8	17	E	0	95918.382	-0.202	133.7920
16	6	10	15	6	9	E	0	95934.969	0.052	92.4718
10	4	7	9	3	6	A	1	95941.701	-0.208	189.2546
16	6	10	15	6	9	A	1	95950.468	-0.088	209.2263
16	6	10	15	6	9	A	0	95974.093	0.068	92.4552
33	9	25	32	10	22	A	0	95995.160	0.474	186.3630
33	9	25	32	10	22	E	0	95997.957	-0.123	186.3728
34	9	26	34	8	27	E	1	96034.922	0.109	309.4999
17	3	15	16	3	14	A	1	96068.196	-0.025	206.6299
33	8	26	32	9	23	A	0	96074.621	-0.025	183.1788
24	9	16	24	8	17	A	0	96133.901	-0.062	133.7827
17	3	15	16	3	14	E	0	96161.622	-0.003	89.8197
17	3	15	16	3	14	A	0	96164.230	-0.004	89.8037
17	3	15	16	3	14	E	1	96168.495	-0.109	206.1167
22	9	13	22	8	14	A	1	96200.884	0.377	241.0662
27	4	23	27	3	24	A	1	96238.002	0.047	254.5578
23	2	21	23	1	22	E	0	96270.748	-0.180	112.5591
18	5	14	17	5	12	E	1	96277.255	0.059	213.5850
23	2	21	23	1	22	A	0	96292.223	-0.069	112.5431
23	3	21	23	2	22	A	0	96323.055	-0.115	112.5431
18	1	17	17	2	16	A	1	96337.195	-0.035	207.5990
18	2	17	17	2	16	A	1	96362.703	-0.052	207.5990
18	1	17	17	1	16	A	1	96384.915	-0.043	207.5974
43	14	30	42	15	27	A	0	96390.819	0.078	284.9016
16	6	10	15	6	10	E	0	96392.830	0.121	92.4566
18	1	17	17	2	16	E	1	96395.653	-0.273	207.0738
18	1	17	17	2	16	E	0	96406.971	-0.012	90.7787
18	1	17	17	2	16	A	0	96408.740	0.019	90.7623
18	2	17	17	1	16	A	1	96410.435	-0.047	207.5974
23	9	14	23	8	15	A	0	96417.898	0.104	128.9833
18	2	17	17	2	16	E	1	96423.763	-0.083	207.0738
41	13	29	40	14	27	E	1	96430.646	0.721	378.9112
23	3	21	23	2	22	E	1	96431.990	0.413	228.8513
18	2	17	17	2	16	E	0	96434.404	-0.006	90.7787
18	2	17	17	2	16	A	0	96436.271	0.010	90.7623

J'	K'_a	K'_c	J''	K''_a	K''_c	Sym.	$v_{t_{CH_3}}$	$\nu_{Obs.}$	$\nu_{Obs.} - \nu_{Calc.}$	E_{low}
25	3	22	25	2	23	A	1	96445.243	-0.055	241.4547
18	1	17	17	1	16	E	1	96447.595	-0.117	207.0721
18	1	17	17	1	16	E	0	96457.970	-0.068	90.7770
18	1	17	17	1	16	A	0	96459.947	-0.027	90.7606
23	9	15	23	8	16	E	1	96473.340	-0.745	245.0867
18	2	17	17	1	16	E	1	96475.557	-0.076	207.0721
18	2	17	17	1	16	E	0	96485.478	0.013	90.7770
18	2	17	17	1	16	A	0	96487.567	0.054	90.7606
10	4	7	9	3	6	E	0	96495.052	-0.258	72.4272
23	9	14	23	8	15	E	0	96508.474	0.124	128.9971
19	0	19	18	0	18	A	1	96558.063	-0.070	208.1447
19	1	19	18	0	18	A	1	96558.063	-0.070	208.1447
19	0	19	18	1	18	A	1	96558.063	-0.070	208.1447
19	1	19	18	1	18	A	1	96558.063	-0.070	208.1447
16	5	12	15	5	11	E	1	96564.656	0.103	206.5425
19	1	19	18	1	18	E	1	96576.821	-0.069	207.5998
19	0	19	18	0	18	E	1	96576.821	-0.069	207.5997
31	7	25	31	6	26	A	1	96582.320	0.041	283.7487
19	0	19	18	0	18	A	0	96599.370	-0.143	91.2926
19	0	19	18	1	18	A	0	96599.370	-0.143	91.2927
19	1	19	18	1	18	A	0	96599.370	-0.143	91.2927
19	0	19	18	0	18	E	0	96599.370	0.159	91.3096
19	1	19	18	0	18	E	0	96599.370	0.159	91.3096
19	1	19	18	1	18	E	0	96599.370	0.159	91.3097
27	4	23	27	3	24	E	0	96650.025	-0.051	137.8057
27	4	23	27	3	24	A	0	96662.436	-0.051	137.7915
17	2	15	16	2	14	A	1	96665.087	-0.032	206.5800
23	9	15	23	8	16	E	0	96679.837	-0.042	128.9745
29	6	24	29	5	25	A	1	96750.097	0.054	268.6621
17	2	15	16	2	14	E	0	96785.856	-0.055	89.7671
17	2	15	16	2	14	A	0	96790.087	-0.023	89.7509
17	2	15	16	2	14	E	1	96790.098	-0.257	206.0639
32	6	26	32	5	27	E	1	96849.112	-0.030	289.5132
31	7	25	31	6	26	E	1	96865.361	-0.640	283.3495
23	9	15	23	8	16	A	0	96878.792	-0.070	128.9666
27	5	23	27	4	24	A	1	96890.701	0.032	254.5595
21	9	12	21	8	13	A	1	96950.201	0.327	236.6655
25	3	22	25	2	23	E	0	96951.222	-0.093	124.6759
25	3	22	25	2	23	A	0	96969.059	-0.091	124.6608
32	6	26	32	5	27	A	1	96978.073	0.026	289.9082
31	7	25	31	6	26	E	0	96998.112	0.047	167.0510
31	7	25	31	6	26	A	0	97011.428	0.025	167.0385
21	9	13	21	8	14	A	1	97073.513	0.484	236.6611
32	6	26	32	5	27	A	0	97077.871	0.194	173.2030
32	6	26	32	5	27	E	0	97084.010	0.216	173.2154
16	5	11	15	5	10	E	1	97088.761	-0.140	206.8753
33	8	26	33	7	27	A	1	97099.235	0.254	299.8102
25	4	22	25	3	23	E	0	97118.363	-0.064	124.6762
25	4	22	25	2	23	E	0	97127.539	-0.230	124.6759
29	6	24	29	5	25	E	1	97129.188	-0.570	268.2366
25	4	22	25	3	23	A	0	97136.866	-0.179	124.6612
29	6	24	29	5	25	E	0	97171.415	0.019	151.9373
29	6	24	29	5	25	A	0	97184.622	-0.057	151.9240
33	8	26	33	7	27	E	1	97260.792	-0.635	299.4358
34	9	26	34	8	27	E	0	97274.611	0.035	193.2111
22	9	14	22	8	15	E	1	97293.261	-0.605	240.4775
34	9	26	34	8	27	A	0	97306.315	0.045	193.2001
22	9	13	22	8	14	A	0	97329.667	-0.025	124.3686
27	5	23	27	4	24	E	0	97354.986	-0.066	137.8076
27	5	23	27	4	24	A	0	97370.562	-0.060	137.7934
27	5	23	27	4	24	E	1	97379.966	-0.542	254.1059
22	9	13	22	8	14	E	0	97385.235	0.009	124.3839
22	9	14	22	8	15	E	0	97403.438	-0.094	124.3664
27	5	23	27	3	24	E	0	97411.727	0.016	137.8057
27	5	23	27	3	24	A	0	97427.504	-0.073	137.7915
29	6	24	29	4	25	E	0	97439.550	0.103	151.9284
29	6	24	29	4	25	A	0	97453.989	-0.102	151.9150
38	8	30	38	7	31	A	0	97539.681	0.124	222.1277
20	9	11	20	8	12	A	1	97563.199	0.326	232.4751
17	3	15	16	2	14	A	1	97565.046	-0.072	206.5800
22	9	14	22	8	15	A	0	97566.099	-0.074	124.3601
38	8	30	38	7	31	E	0	97570.593	0.116	222.1383
33	8	26	33	7	27	E	0	97573.933	0.077	183.1399
33	8	26	33	7	27	A	0	97591.394	0.094	183.1282
20	9	12	20	8	13	A	1	97621.073	0.519	232.4731
35	7	28	35	6	29	E	1	97657.576	-0.016	312.9182
17	3	15	16	2	14	E	0	97738.446	-0.068	89.7671
17	3	15	16	2	14	A	0	97745.758	-0.052	89.7509
17	3	15	16	2	14	E	1	97749.191	-0.411	206.0639
21	9	13	21	8	14	E	1	97988.469	-0.556	236.0791
16	5	11	15	5	10	A	0	97993.542	0.231	90.4297
16	5	11	15	5	10	E	0	97993.542	-0.104	90.4448
16	5	11	15	5	10	A	1	98000.889	-0.070	207.2294
35	7	28	35	6	29	A	0	98032.163	0.216	196.6122
31	7	25	31	5	26	E	0	98039.717	0.018	167.0163
35	7	28	35	6	29	E	0	98050.142	0.195	196.6237
21	9	13	21	8	14	E	0	98057.479	-0.054	119.9674
21	9	12	21	8	13	A	0	98067.233	-0.013	119.9667
19	9	10	19	8	11	A	1	98069.477	0.314	228.4930
21	9	12	21	8	13	E	0	98086.911	-0.024	119.9834
16	3	13	15	3	12	A	1	98094.287	-0.062	204.8505
35	7	28	35	6	29	A	1	98135.707	-0.003	313.2829
21	9	13	21	8	14	A	0	98183.241	-0.023	119.9626
16	3	13	15	3	12	E	1	98200.238	-0.216	204.3319
16	3	13	15	3	12	E	0	98233.593	-0.053	88.0346
16	3	13	15	3	12	A	0	98238.122	-0.057	88.0182
18	9	10	18	8	11	A	1	98500.733	0.450	224.7170
20	9	12	20	8	13	E	0	98625.877	-0.027	115.7769
20	9	11	20	8	12	E	0	98666.603	0.011	115.7926
20	9	11	20	8	12	A	0	98671.659	-0.030	115.7751
20	9	12	20	8	13	A	0	98725.870	-0.111	115.7731
31	10	21	31	9	22	E	0	98810.943	0.182	176.6096
31	10	21	31	9	22	A	0	98813.487	0.151	176.5989
35	9	27	35	8	28	E	1	98993.805	-0.740	316.4971
48	11	37	48	10	38	A	0	99088.997	-0.675	320.9465
19	9	11	19	8	12	E	0	99110.195	-0.050	111.7939
16	9	7	16	8	8	A	1	99128.675	0.399	217.7805
16	9	8	16	8	9	A	1	99130.217	0.366	217.7804
48	11	37	48	10	38	E	0	99141.724	-0.571	320.9550
19	9	10	19	8	11	E	0	99152.596	0.001	111.8096
19	9	10	19	8	11	A	0	99171.546	-0.033	111.7916
19	9	11	19	8	12	A	0	99195.687	-0.036	111.7907
18	9	10	18	8	11	E	1	99492.948	-0.477	224.1334
18	9	10	18	8	11	E	0	99519.062	-0.062	108.0171
18	9	9	18	8	10	E	0	99561.107	0.036	108.0328
18	9	9	18	8	10	A	0	99586.895	-0.064	108.0146

J'	K_a'	K_c'	J''	K_a''	K_c''	Sym.	$v_{t_{CH_3}}$	$\nu_{Obs.}$	$\nu_{Obs.} - \nu_{Calc.}$	E_{low}
18	9	10	18	8	11	A	0	99597.092	-0.020	108.0142
13	9	4	13	8	5	A	1	99719.876	0.356	208.8948
13	9	5	13	8	6	A	1	99719.876	0.356	208.8948
18	3	15	17	4	14	A	0	99752.600	0.295	95.0079
18	3	15	17	4	14	E	0	99765.321	0.206	95.0233
35	9	27	35	8	28	E	0	99785.824	0.060	200.2064
35	9	27	35	8	28	A	0	99812.389	0.178	200.1956
8	5	4	7	4	3	A	1	99830.605	0.547	187.1113
17	9	9	17	8	10	E	1	99843.723	-0.436	220.5632
12	9	3	12	8	4	A	1	99845.576	0.326	206.3346
12	9	4	12	8	5	A	1	99845.576	0.326	206.3346
17	9	9	17	8	10	E	0	99861.976	-0.045	104.4453
17	4	14	16	4	13	A	1	99883.157	-0.045	208.4864
8	5	3	7	4	4	A	1	99902.281	0.079	187.1091
17	9	8	17	8	9	E	0	99903.357	0.087	104.4611
17	16	2	16	16	1	E	1	99903.357	-0.195	255.3371
17	16	1	16	16	0	A	1	99911.227	-0.045	255.4809
17	16	2	16	16	1	A	1	99911.227	-0.045	255.4809
17	9	8	17	8	9	A	0	99932.602	0.267	104.4428
17	9	9	17	8	10	A	0	99936.250	-0.098	104.4426
17	15	3	16	15	2	E	1	99949.233	-0.262	249.1537
17	15	2	16	15	1	E	1	99950.376	0.060	249.7818
17	15	3	16	15	2	A	1	99958.062	-0.056	249.3683
17	15	2	16	15	1	A	1	99958.062	-0.056	249.3683
6	6	0	5	5	1	A	1	99963.894	0.045	186.3120
6	6	1	5	5	0	A	1	99963.894	0.045	186.3120
17	16	1	16	16	0	A	0	99965.907	0.087	139.0978
17	16	2	16	16	1	A	0	99965.907	0.087	139.0978
17	16	1	16	16	0	E	0	99965.907	-0.117	139.1023
17	16	2	16	16	1	E	0	99965.907	-0.117	139.0832
17	4	14	16	4	13	E	0	99971.935	-0.034	91.6886
17	4	14	16	4	13	A	0	99973.696	0.025	91.6732
17	14	3	16	14	2	E	1	100004.097	0.099	244.0089
17	14	4	16	14	3	E	1	100005.576	0.190	243.3727
17	15	2	16	15	1	A	0	100011.801	0.082	132.9450
17	15	3	16	15	2	A	0	100011.801	0.082	132.9450
17	15	2	16	15	1	E	0	100011.801	-0.175	132.9518
17	15	3	16	15	2	E	0	100011.801	-0.175	132.9321
37	11	26	37	10	27	A	0	100023.326	0.472	222.2162
17	14	3	16	14	2	A	0	100067.551	0.174	127.1905
17	14	4	16	14	3	A	0	100067.551	0.174	127.1905
17	14	3	16	14	2	E	0	100067.551	-0.143	127.1995
17	14	4	16	14	3	E	0	100067.551	-0.143	127.1795
17	13	4	16	13	3	E	1	100070.125	0.000	238.6303
17	13	5	16	13	4	E	1	100074.488	-0.023	237.9948
17	13	5	16	13	4	A	1	100084.528	-0.065	238.3416
17	13	4	16	13	3	A	1	100084.528	-0.065	238.3416
17	4	14	16	4	13	E	1	100092.299	0.005	207.9782
17	13	4	16	13	3	A	0	100136.224	0.215	121.8349
17	13	5	16	13	4	A	0	100136.224	0.215	121.8349
17	13	4	16	13	3	E	0	100136.224	-0.169	121.8459
17	13	5	16	13	4	E	0	100136.224	-0.169	121.8259
16	9	8	16	8	9	E	0	100147.602	-0.017	101.0774
16	4	12	15	4	11	E	1	100149.401	-0.191	205.3760
17	12	5	16	12	4	E	1	100153.124	-0.028	233.6470
17	12	6	16	12	5	E	1	100161.560	-0.088	233.0210
17	12	5	16	12	4	A	1	100172.129	-0.026	233.4274
17	12	6	16	12	5	A	1	100172.129	-0.026	233.4274
16	9	7	16	8	8	E	0	100188.287	0.061	101.0933
16	9	7	16	8	8	A	0	100218.593	-0.087	101.0749
17	12	5	16	12	4	A	0	100222.569	0.294	116.8786
17	12	6	16	12	5	A	0	100222.569	0.294	116.8786
17	12	5	16	12	4	E	0	100222.569	-0.167	116.8915
17	12	6	16	12	5	E	0	100222.569	-0.167	116.8718
30	5	25	30	4	26	A	1	100231.996	-0.029	274.3402
17	11	6	16	11	5	E	1	100259.698	-0.028	229.0601
42	9	33	42	9	34	E	1	100263.430	0.344	374.5400
17	11	7	16	11	6	E	1	100273.920	-0.051	228.4523
17	11	6	16	11	5	A	1	100284.477	-0.057	228.9129
17	11	7	16	11	6	A	1	100284.477	-0.057	228.9129
30	5	25	30	5	26	E	1	100314.728	-0.184	273.9253
17	11	6	16	11	5	A	0	100333.303	0.147	112.3226
17	11	7	16	11	6	A	0	100333.303	0.147	112.3226
17	11	6	16	11	5	E	0	100333.303	-0.183	112.3372
17	10	7	16	10	6	E	1	100400.323	0.147	224.8713
30	5	25	30	5	26	A	0	100406.287	0.028	157.6129
17	10	8	16	10	7	E	1	100422.622	-0.095	224.2902
16	4	12	15	4	11	A	1	100425.350	-0.039	205.8628
17	10	8	16	10	7	A	1	100432.650	-0.068	224.7989
17	10	7	16	10	6	A	1	100432.650	-0.068	224.7989
15	9	7	15	8	8	A	0	100454.986	-0.208	97.9100
16	4	12	15	4	11	A	0	100472.496	0.009	89.0392
16	4	12	15	4	11	E	0	100473.512	0.085	89.0552
17	10	7	16	10	6	A	0	100479.698	0.154	108.1683
17	10	8	16	10	7	A	0	100479.698	0.154	108.1683
17	10	7	16	10	6	E	0	100479.698	-0.154	108.1844
17	10	8	16	10	7	E	0	100480.702	0.139	108.1661
8	5	4	7	4	4	E	0	100487.198	-0.403	70.2946
8	5	4	7	4	3	A	0	100509.314	-0.366	70.2847
24	2	22	24	1	23	A	1	100516.162	-0.028	233.5704
24	3	22	24	2	23	A	1	100531.358	-0.175	233.5704
8	5	3	7	4	3	E	0	100540.935	-0.297	70.3038
7	3	5	6	1	6	A	0	100556.314	0.330	65.5422
30	5	25	30	4	26	A	0	100563.078	0.024	157.6077
18	2	16	17	3	15	A	1	100567.600	0.095	209.8344
14	9	6	14	8	7	E	0	100576.627	-0.009	94.9495
17	9	8	16	9	7	E	1	100591.369	-0.029	221.0832
17	9	9	16	9	8	E	1	100626.400	-0.083	220.5371
18	2	16	17	3	15	E	1	100628.075	-0.011	209.3245
18	2	16	17	3	15	E	0	100631.851	-0.067	93.0273
18	2	16	17	3	15	A	0	100632.715	-0.016	93.0114
17	9	9	16	9	8	A	1	100634.742	-0.070	221.0871
17	9	8	16	9	7	A	1	100634.742	-0.070	221.0871
14	9	5	14	8	6	A	0	100647.434	-0.215	94.9470
14	9	6	14	8	7	A	0	100647.434	-0.215	94.9470
17	9	8	16	9	7	A	0	100679.481	0.107	104.4178
17	9	9	16	9	8	A	0	100679.481	0.107	104.4178
17	9	8	16	9	7	E	0	100679.481	-0.143	104.4352
17	9	9	16	9	8	E	0	100680.834	0.094	104.4180
6	6	1	5	5	1	E	0	100696.364	-0.358	69.5151
13	9	5	13	8	6	E	0	100732.779	-0.001	92.1876
6	6	0	5	5	0	E	0	100752.445	-0.335	69.5261
13	9	4	13	8	5	E	0	100772.072	0.012	92.2037
13	9	4	13	8	5	A	0	100803.668	0.162	92.1853
13	9	5	13	8	6	A	0	100803.668	0.162	92.1853
17	8	10	16	8	9	E	1	100918.455	-0.022	217.1969

J'	K_a'	K_c'	J''	K_a''	K_c''	Sym.	$v_{t_{CH_3}}$	$\nu_{Obs.}$	$\nu_{Obs.} - \nu_{Calc.}$	E_{low}
17	8	10	16	8	9	A	1	100921.556	-0.009	217.7804
17	8	9	16	8	8	A	1	100924.353	-0.016	217.7805
12	9	3	12	8	4	A	0	100927.817	-0.013	89.6239
12	9	4	12	8	5	A	0	100927.817	-0.013	89.6239
11	9	3	11	8	4	E	0	100955.173	0.102	87.2645
17	8	10	16	8	9	A	0	100963.139	0.005	101.0748
17	8	9	16	8	8	E	0	100964.582	0.002	101.0933
17	8	9	16	8	8	A	0	100966.151	0.383	101.0749
17	8	10	16	8	9	E	0	100966.151	-0.187	101.0774
33	8	26	33	6	27	A	0	101009.190	-0.116	183.0142
11	9	2	11	8	3	A	0	101025.345	-0.040	87.2621
11	9	3	11	8	4	A	0	101025.345	-0.040	87.2621
18	3	16	17	3	15	A	1	101096.268	-0.042	209.8344
10	9	1	10	8	2	A	0	101100.466	0.021	85.0994
10	9	2	10	8	3	A	0	101100.466	0.021	85.0994
18	3	16	17	3	15	E	0	101194.197	-0.027	93.0273
18	3	16	17	3	15	A	0	101196.885	-0.132	93.0114
18	3	16	17	3	15	E	1	101196.896	0.254	209.3245
24	2	22	24	2	23	E	1	101254.410	-0.088	233.0594
30	5	25	29	6	23	E	1	101266.768	-0.213	273.8935
17	7	10	16	7	9	E	1	101273.575	-0.157	214.7277
19	1	18	18	2	17	A	1	101305.939	-0.066	210.8133
19	2	18	18	2	17	A	1	101319.466	-0.055	210.8133
19	1	18	18	1	17	A	1	101331.488	-0.042	210.8124
17	7	11	16	7	10	A	1	101336.287	-0.040	214.8850
19	2	18	18	1	17	A	1	101345.007	-0.038	210.8124
17	7	11	16	7	10	E	1	101365.582	-0.021	214.2769
19	1	18	18	2	17	E	1	101365.582	-0.021	210.2902
17	7	11	16	7	10	A	0	101374.352	-0.017	98.1458
19	1	18	18	2	17	E	0	101377.945	-0.084	93.9954
19	2	18	18	2	17	E	1	101380.276	-0.204	210.2902
17	7	10	16	7	9	A	1	101386.521	-0.087	214.8863
19	2	18	18	2	17	E	0	101392.568	-0.049	93.9954
19	2	18	18	2	17	A	0	101394.479	0.007	93.9791
17	7	11	16	7	10	E	0	101397.724	0.051	98.1513
17	7	10	16	7	9	E	0	101401.390	0.013	98.1656
19	1	18	18	1	17	E	0	101405.472	0.016	93.9945
19	1	18	18	1	17	A	0	101407.349	-0.010	93.9782
19	2	18	18	1	17	E	0	101420.027	-0.017	93.9945
19	2	18	18	1	17	A	0	101422.073	0.046	93.9782
17	7	10	16	7	9	A	0	101422.073	0.046	98.1470
28	4	24	28	3	25	A	1	101423.171	0.053	259.7471
18	2	16	17	2	15	A	1	101467.395	-0.108	209.8044
20	2	18	19	2	17	E	1	101476.290	0.006	313.2100
30	10	20	30	9	21	A	0	101495.722	0.185	170.2620
20	0	20	19	0	19	A	1	101514.584	-0.015	211.3655
20	1	20	19	0	19	A	1	101514.584	-0.015	211.3655
20	0	20	19	1	19	A	1	101514.584	-0.015	211.3655
20	1	20	19	1	19	A	1	101514.584	-0.015	211.3655
20	1	20	19	1	19	E	1	101533.105	-0.090	210.8212
20	0	20	19	0	19	E	1	101533.105	-0.090	210.8212
30	10	20	30	9	21	E	0	101537.565	0.225	170.2727
20	0	20	19	0	19	A	0	101557.048	-0.287	94.5149
20	0	20	19	1	19	A	0	101557.048	-0.287	94.5149
20	1	20	19	1	19	A	0	101557.048	-0.287	94.5149
20	0	20	19	0	19	E	0	101557.048	0.146	94.5318
20	1	20	19	0	19	E	0	101557.048	0.146	94.5318
20	1	20	19	1	19	E	0	101557.048	0.146	94.5318
18	2	16	17	2	15	E	0	101584.516	-0.005	92.9955
18	2	16	17	2	15	E	1	101587.162	-0.170	209.2925
18	2	16	17	2	15	A	0	101588.433	0.002	92.9795
34	8	27	34	7	28	A	0	101646.254	0.123	189.8096
32	7	26	32	6	27	E	0	101659.480	0.089	173.2366
32	7	26	32	6	27	A	0	101671.857	0.081	173.2243
17	5	13	16	5	12	A	1	101740.307	-0.128	210.3231
17	5	13	16	5	12	A	0	101800.004	-0.018	93.5282
17	5	13	16	5	12	E	0	101801.932	-0.011	93.5427
28	5	24	28	4	25	A	1	101813.084	-0.209	259.7480
17	6	12	16	6	11	A	1	101821.268	-0.079	212.4074
17	6	12	16	6	11	A	0	101860.707	0.069	95.6379
28	4	24	28	3	25	E	0	101880.293	-0.127	143.0000
17	6	12	16	6	11	E	0	101888.197	-0.048	95.6495
28	4	24	28	3	25	A	0	101894.876	-0.062	142.9859
26	3	23	26	2	24	E	0	101917.257	-0.212	129.3762
26	3	23	26	2	24	A	0	101936.717	-0.085	129.3612
17	6	11	16	6	10	E	1	101978.963	-0.125	212.1816
13	9	4	13	8	5	E	1	101992.374	-0.457	208.8181
18	3	16	17	2	15	A	1	101996.337	0.030	209.8044
26	4	23	26	3	24	E	0	102012.876	-0.126	129.3764
6	6	0	5	5	0	E	1	102023.654	0.055	186.0238
26	4	23	26	3	24	A	0	102032.658	-0.150	129.3614
12	4	9	11	3	8	A	1	102042.728	-0.016	193.6174
30	6	25	30	5	26	E	1	102057.574	-0.563	273.9253
30	6	25	30	5	26	E	0	102086.236	0.055	157.6261
45	10	35	45	9	36	A	0	102087.543	-0.210	288.5081
30	6	25	30	5	26	A	0	102099.882	0.091	157.6129
26	4	23	26	3	24	E	1	102104.803	-0.594	245.6718
10	4	6	9	3	7	A	1	102113.358	0.231	189.0853
17	6	12	16	6	11	E	1	102117.841	0.026	211.7925
45	10	35	45	9	36	E	0	102136.756	-0.286	288.5170
18	3	16	17	2	15	E	0	102146.745	-0.081	92.9955
18	3	16	17	2	15	A	0	102152.650	-0.068	92.9795
18	3	16	17	2	15	E	1	102155.658	-0.230	209.2925
34	10	25	34	9	26	E	0	102172.851	0.269	196.4558
34	10	25	34	9	26	A	0	102236.227	0.152	196.4459
32	7	26	32	5	27	E	0	102292.768	0.076	173.2154
28	5	24	28	4	25	E	0	102303.766	-0.062	143.0010
32	7	26	32	5	27	A	0	102308.274	-0.045	173.2030
28	5	24	28	4	25	A	0	102320.264	-0.097	142.9870
28	5	24	28	4	25	E	1	102339.623	-0.551	259.2990
36	9	28	36	8	29	E	1	102344.704	-0.721	323.6683
33	10	24	33	9	25	E	0	102347.036	0.291	189.5749
17	6	11	16	6	10	E	0	102385.750	0.025	95.6719
17	6	11	16	6	10	A	1	102393.291	-0.091	212.4269
35	10	26	35	9	27	E	0	102395.268	0.275	203.5349
17	6	11	16	6	10	A	0	102409.495	-0.025	95.6565
33	10	24	33	9	25	A	0	102423.342	0.224	189.5651
35	10	26	35	9	27	A	0	102449.427	0.226	203.5249
17	5	13	16	5	12	E	1	102555.104	0.221	209.7635
10	4	6	9	3	7	A	0	102613.564	-0.135	72.2437
12	4	9	11	3	8	E	0	102641.369	-0.369	76.7913
32	10	23	32	9	24	E	0	102823.533	0.183	182.8962
36	9	28	36	8	29	E	0	102857.620	0.090	207.3766
36	9	28	36	8	29	A	0	102879.377	0.141	207.3659
33	6	27	33	6	28	E	1	102894.348	-0.158	295.8860
32	10	23	32	9	24	A	0	102919.676	0.167	182.8865

J'	K'_a	K'_c	J''	K''_a	K''_c	Sym.	$v_{t_{CH_3}}$	$\nu_{Obs.}$	$\nu_{Obs.} - \nu_{Calc.}$	E_{low}
17	3	14	16	3	13	A	1	103007.561	-0.039	208.1226
36	10	27	36	9	28	E	0	103097.606	0.276	210.8075
36	10	27	36	9	28	A	0	103144.180	0.208	210.7976
17	3	14	16	3	13	E	1	103158.921	-0.309	207.6075
17	3	14	16	3	13	E	0	103168.800	-0.101	91.3113
17	3	14	16	3	13	A	0	103174.593	-0.081	91.2950
42	12	30	42	11	31	E	0	103177.943	-0.128	266.7891
39	8	31	39	8	32	E	1	103230.117	0.019	346.1995
33	6	27	33	5	28	A	1	103287.033	0.127	296.2598
33	6	27	33	5	28	E	1	103291.974	-0.214	295.8727
22	5	17	21	6	16	E	0	103423.027	0.468	114.6876
33	6	27	33	5	28	A	0	103482.237	0.075	179.5624
33	6	27	33	5	28	E	0	103483.654	0.200	179.5745
31	10	22	31	9	23	E	0	103507.114	0.201	176.4232
29	10	19	29	9	20	A	0	103613.370	0.196	164.1611
31	10	22	31	9	23	A	0	103634.042	0.239	176.4137
29	10	19	29	9	20	E	0	103696.213	0.205	164.1718
42	9	33	42	8	34	E	1	103767.213	-0.180	374.4231
24	6	18	24	5	20	E	1	103828.938	0.408	242.2781
34	8	27	34	6	28	A	0	103830.616	-0.101	189.7367
36	7	29	36	7	30	E	1	103995.276	-0.079	319.9797
17	5	12	16	5	11	E	1	104061.860	-0.187	210.1139
44	14	30	43	15	29	A	0	104107.825	-0.297	293.5132
30	9	21	30	8	23	E	1	104152.288	-0.465	283.3151
30	10	21	30	9	22	E	0	104314.148	0.238	170.1583
37	10	28	37	9	29	E	0	104344.583	0.261	218.2689
36	7	29	36	7	30	A	0	104361.159	0.238	203.6720
36	7	29	36	7	30	A	1	104427.052	0.105	320.3307
42	9	33	42	8	34	A	0	104431.328	-0.006	258.1326
29	10	20	29	9	21	E	1	104448.767	-0.418	280.2217
42	9	33	42	8	34	E	0	104470.115	0.009	258.1421
30	10	21	30	9	22	A	0	104481.880	0.212	170.1491
20	4	16	19	5	15	E	0	104669.143	-0.073	104.3145
31	9	22	31	8	24	E	1	104982.751	0.258	289.5849
17	5	12	16	5	11	A	0	104994.091	0.091	93.6984
17	5	12	16	5	11	E	0	104996.703	0.091	93.7135
17	5	12	16	5	11	A	1	105004.803	-0.091	210.4984
39	8	31	39	7	32	E	1	105049.299	0.040	346.1389
22	5	17	21	6	16	E	1	105084.507	-0.342	230.9274
29	10	20	29	9	21	E	0	105185.972	0.175	164.1035
36	7	29	36	6	30	A	1	105195.141	0.144	320.3051
36	7	29	36	6	30	A	0	105206.886	0.041	203.6438
36	7	29	36	6	30	E	0	105219.082	0.139	203.6549
18	4	15	17	4	14	A	1	105233.878	0.020	211.8182
28	10	18	28	9	19	A	0	105287.398	0.136	158.2891
25	2	23	25	1	24	A	1	105318.417	-0.193	237.9417
25	3	23	25	1	24	A	1	105327.054	-0.068	237.9417
18	4	15	17	4	14	E	0	105333.448	0.041	95.0233
18	4	15	17	4	14	A	0	105335.741	0.058	95.0079
18	4	15	17	4	14	E	1	105401.078	-0.015	211.3169
47	11	36	46	13	33	A	1	105458.621	0.273	427.2040
39	8	31	39	7	32	A	0	105543.219	0.119	229.8388
9	5	5	8	4	4	A	1	105549.228	-0.109	188.6963
14	4	11	13	3	10	E	1	105552.671	0.397	198.3208
39	8	31	39	7	32	E	0	105567.968	-0.349	229.8490
35	8	28	35	7	29	A	1	105627.572	0.234	313.3246
31	5	26	31	4	27	A	1	105734.729	0.019	280.1898
28	10	19	28	9	20	E	1	105741.491	0.180	274.3667
39	8	31	39	7	32	A	1	105754.669	0.047	346.4640
9	5	4	8	4	5	A	1	105764.473	-0.091	188.6898
18	17	2	17	17	1	E	1	105774.889	-0.090	265.2532
18	17	1	17	17	0	E	1	105778.192	-0.156	265.8396
18	17	2	17	17	1	A	1	105782.715	-0.095	265.3247
18	17	1	17	17	0	A	1	105782.715	-0.095	265.3247
19	2	17	18	3	16	A	1	105788.215	-0.101	213.2066
18	16	3	17	16	2	E	1	105820.111	-0.164	258.6695
18	16	2	17	16	1	E	1	105822.124	-0.108	259.2809
18	16	2	17	16	1	A	1	105829.066	-0.091	258.8136
18	16	3	17	16	2	A	1	105829.066	-0.091	258.8136
7	6	2	6	5	1	A	1	105836.388	0.296	187.4897
18	17	1	17	17	0	A	0	105841.178	0.123	148.9818
18	17	2	17	17	1	A	0	105841.178	0.123	148.9818
18	17	1	17	17	0	E	0	105841.178	-0.069	148.9841
18	17	2	17	17	1	E	0	105841.178	-0.069	148.9658
19	2	17	18	3	16	E	0	105868.855	-0.118	96.4027
19	2	17	18	3	16	A	0	105870.674	0.013	96.3869
18	15	4	17	15	3	E	1	105874.566	-0.084	252.4877
18	15	3	17	15	2	E	1	105874.566	-0.084	253.1158
18	16	2	17	16	1	A	0	105886.545	0.117	142.4323
18	16	3	17	16	2	A	0	105886.545	0.117	142.4323
18	16	2	17	16	1	E	0	105886.545	-0.131	142.4369
18	16	3	17	16	2	E	0	105886.545	-0.131	142.4177
18	14	4	17	14	3	E	1	105938.237	-0.006	247.3447
18	15	3	17	15	2	A	0	105940.877	0.171	136.2810
18	15	4	17	15	3	A	0	105940.877	0.171	136.2810
18	15	3	17	15	2	E	0	105940.877	-0.137	136.2878
18	15	4	17	15	3	E	0	105940.877	-0.137	136.2681
18	14	5	17	14	4	E	1	105940.877	0.029	246.7085
25	2	23	25	1	24	E	0	105950.575	0.293	121.1418
18	14	4	17	14	3	A	1	105951.559	-0.012	246.9914
18	14	5	17	14	4	A	1	105951.559	-0.012	246.9914
25	3	23	25	2	24	E	0	105959.162	-0.178	121.1418
25	2	23	25	1	24	A	0	105973.988	-0.253	121.1259
18	14	4	17	14	3	A	0	106006.772	0.167	130.5284
18	14	5	17	14	4	A	0	106006.772	0.167	130.5284
18	14	4	17	14	3	E	0	106006.772	-0.209	130.5374
18	14	5	17	14	4	E	0	106006.772	-0.209	130.5174
18	13	5	17	13	4	E	1	106016.655	-0.006	241.9683
18	13	6	17	13	5	E	1	106022.752	-0.067	241.3330
18	13	6	17	13	5	A	1	106034.214	-0.020	241.6801
18	13	5	17	13	4	A	1	106034.214	-0.020	241.6801
35	8	28	35	7	29	E	0	106042.860	0.081	196.6692
35	8	28	35	7	29	A	0	106055.277	-0.047	196.6579
28	10	19	28	9	20	E	0	106083.836	0.133	158.2594
18	13	5	17	13	4	A	0	106088.143	0.180	125.1750
18	13	6	17	13	5	A	0	106088.143	0.180	125.1750
18	13	5	17	13	4	E	0	106088.143	-0.273	125.1860
18	13	6	17	13	5	E	0	106088.143	-0.273	125.1661
19	3	17	18	3	16	A	1	106093.016	-0.140	213.2066
18	12	6	17	12	5	E	1	106115.266	0.031	236.9878
31	5	26	31	4	27	E	0	106117.689	0.029	163.4766
18	12	7	17	12	6	E	1	106126.099	-0.166	236.3620
31	5	26	31	4	27	A	0	106127.468	0.110	163.4636
18	12	7	17	12	6	A	1	106138.050	-0.054	236.7688
18	12	6	17	12	5	A	1	106138.050	-0.054	236.7688
38	10	29	38	9	30	E	0	106176.239	0.291	225.9144

J'	K'_a	K'_c	J''	K''_a	K''_c	Sym.	$v_{t_{CH_3}}$	$\nu_{Obs.}$	$\nu_{Obs.}-\nu_{Calc.}$	E_{low}
18	12	6	17	12	5	A	0	106190.559	0.208	120.2217
18	12	7	17	12	6	A	0	106190.559	0.208	120.2217
18	12	6	17	12	5	E	0	106190.559	-0.164	120.2345
19	3	17	18	3	16	E	1	106193.820	-0.148	212.7001
19	3	17	18	3	16	E	0	106194.606	0.007	96.4027
19	3	17	18	3	16	A	0	106197.578	0.057	96.3869
38	10	29	38	9	30	A	0	106209.981	0.435	225.9047
35	9	27	34	10	24	A	0	106225.573	0.086	199.9816
18	11	7	17	11	6	E	1	106241.879	-0.060	232.4044
35	9	27	34	10	24	E	0	106245.285	-0.305	199.9909
18	11	8	17	11	7	E	1	106259.704	-0.086	231.7970
20	1	19	19	2	18	A	1	106267.815	-0.012	214.1929
18	11	8	17	11	7	A	1	106271.598	-0.025	232.2581
18	11	7	17	11	6	A	1	106271.598	-0.025	232.2581
20	2	19	19	2	18	A	1	106274.864	0.067	214.1929
27	3	24	27	2	25	A	1	106274.864	0.067	251.0128
20	1	19	19	1	18	A	1	106281.301	-0.042	214.1925
20	2	19	19	1	18	A	1	106288.297	-0.141	214.1925
28	10	19	28	9	20	A	0	106307.614	0.163	158.2515
9	5	4	8	4	4	E	0	106310.355	0.267	71.8882
19	2	17	18	2	16	A	1	106317.049	-0.072	213.1890
18	11	7	17	11	6	A	0	106322.243	0.102	115.6694
18	11	8	17	11	7	A	0	106322.243	0.102	115.6694
18	11	7	17	11	6	E	0	106322.243	-0.267	115.6840
18	11	8	17	11	7	E	0	106323.081	0.014	115.6648
20	2	19	19	2	18	E	1	106335.519	-0.161	213.6719
20	1	19	19	2	18	E	0	106341.580	-0.097	97.3775
20	1	19	19	2	18	A	0	106343.525	0.030	97.3613
20	2	19	19	2	18	E	0	106349.336	-0.033	97.3775
20	2	19	19	2	18	A	0	106351.214	-0.008	97.3613
20	1	19	19	1	18	E	0	106356.252	-0.013	97.3770
20	1	19	19	1	18	A	0	106358.138	-0.008	97.3608
20	2	19	19	1	18	E	0	106363.966	0.009	97.3770
20	2	19	19	1	18	A	0	106365.883	0.009	97.3608
18	10	8	17	10	7	E	1	106409.169	-0.040	228.2203
19	2	17	18	2	16	E	0	106431.333	0.055	96.3840
19	2	17	18	2	16	A	0	106434.903	-0.044	96.3681
18	10	9	17	10	8	E	1	106436.881	-0.066	227.6399
33	7	27	33	5	28	A	1	106438.850	0.002	296.2598
9	5	4	8	4	5	A	0	106443.827	-0.129	71.8641
37	9	29	37	8	30	E	0	106445.008	0.127	214.7183
18	10	9	17	10	8	A	1	106448.000	-0.041	228.1490
18	10	8	17	10	7	A	1	106448.000	-0.041	228.1490
37	9	29	37	8	30	A	0	106462.635	-0.036	214.7079
30	8	22	29	9	21	A	0	106462.635	-0.036	164.0947
21	0	21	20	0	20	A	1	106471.001	-0.056	214.7517
21	1	21	20	0	20	A	1	106471.001	-0.056	214.7517
21	0	21	20	1	20	A	1	106471.001	-0.056	214.7517
21	1	21	20	1	20	A	1	106471.001	-0.056	214.7517
21	1	21	20	1	20	E	1	106489.385	-0.110	214.2080
21	0	21	20	0	20	E	1	106489.385	-0.110	214.2080
18	10	8	17	10	7	A	0	106496.610	0.143	111.5200
18	10	9	17	10	8	A	0	106496.610	0.143	111.5200
18	10	8	17	10	7	E	0	106496.610	-0.197	111.5361
18	10	9	17	10	8	E	0	106497.851	0.167	111.5177
33	7	27	33	6	28	E	0	106506.545	0.083	179.5872
21	0	21	20	0	20	A	0	106514.889	-0.229	97.9025
21	0	21	20	1	20	A	0	106514.889	-0.229	97.9025
21	1	21	20	1	20	A	0	106514.889	-0.229	97.9025
21	0	21	20	0	20	E	0	106514.889	0.267	97.9194
21	1	21	20	0	20	E	0	106514.889	0.267	97.9194
21	1	21	20	1	20	E	0	106514.889	0.267	97.9194
33	7	27	33	6	28	A	0	106518.487	0.038	179.5751
17	4	13	16	4	12	E	1	106588.607	-0.149	208.7166
31	6	26	31	5	27	A	1	106612.209	0.062	280.1925
19	3	17	18	2	16	A	1	106621.943	-0.018	213.1890
27	10	17	27	9	18	A	0	106625.294	0.004	152.6403
7	6	1	6	5	1	E	0	106627.990	-0.321	70.7044
18	9	9	17	9	8	E	1	106637.547	-0.025	224.4386
18	9	10	17	9	9	E	1	106680.361	0.003	223.8937
18	9	10	17	9	9	A	1	106689.379	-0.008	224.4439
18	9	9	17	9	8	A	1	106689.379	-0.008	224.4439
27	10	17	27	9	18	E	0	106720.818	0.004	152.6526
17	4	13	16	4	12	A	1	106726.675	-0.090	209.2126
18	9	9	17	9	8	A	0	106735.206	0.069	107.7761
18	9	10	17	9	9	A	0	106735.206	0.069	107.7761
18	9	9	17	9	8	E	0	106735.206	-0.199	107.7935
18	9	10	17	9	9	E	0	106736.786	0.017	107.7763
29	5	25	29	4	26	A	1	106746.936	0.437	265.1015
19	3	17	18	2	16	E	0	106756.909	0.004	96.3840
19	3	17	18	2	16	A	0	106761.948	0.140	96.3681
19	3	17	18	2	16	E	1	106762.425	-0.100	212.6811
29	5	25	29	3	26	A	1	106762.519	0.191	265.1009
17	4	13	16	4	12	A	0	106803.752	-0.176	92.3906
17	4	13	16	4	12	E	0	106803.752	0.210	92.4067
27	3	24	27	2	25	E	0	106849.232	-0.090	134.2416
33	7	27	33	5	28	E	0	106886.185	0.052	179.5745
33	7	27	33	5	28	A	0	106900.276	0.106	179.5624
27	4	24	27	3	25	E	0	106903.150	-0.171	134.2417
19	3	16	18	4	15	A	0	106911.275	-0.290	98.5216
19	3	16	18	4	15	E	0	106920.069	0.173	98.5369
27	4	24	27	3	25	A	0	106923.958	-0.377	134.2268
18	8	10	17	8	9	E	1	106963.553	-0.091	221.0641
27	10	18	27	9	19	E	0	106975.960	0.192	152.6264
29	4	25	29	3	26	E	0	107014.335	-0.009	148.3588
29	4	25	29	3	26	A	0	107030.957	0.267	148.3449
18	8	11	17	8	10	E	1	107030.957	-0.135	220.5632
18	8	11	17	8	10	A	1	107032.107	0.112	221.1468
18	8	10	17	8	9	A	1	107038.834	0.003	221.1470
31	6	26	31	5	27	E	0	107069.559	0.008	163.4795
18	8	11	17	8	10	A	0	107074.259	0.029	104.4426
18	8	10	17	8	9	E	0	107077.612	0.008	104.4611
18	8	11	17	8	10	E	0	107079.642	-0.024	104.4453
18	8	10	17	8	9	A	0	107080.713	0.057	104.4428
31	6	26	31	5	27	A	0	107083.780	-0.010	163.4666
31	6	26	31	4	27	E	0	107159.382	0.087	163.4766
31	6	26	31	4	27	A	0	107174.040	0.001	163.4636
27	10	18	27	9	19	A	0	107189.811	0.221	152.6198
26	10	17	26	9	17	E	0	107243.013	-0.184	147.2239
29	5	25	29	4	26	E	0	107264.825	-0.081	148.3594
29	5	25	29	4	26	A	0	107282.480	-0.033	148.3454
29	5	25	29	3	26	E	0	107282.480	0.084	148.3588
29	5	25	29	3	26	A	0	107300.194	0.090	148.3449
35	8	28	35	6	29	A	0	107425.688	0.004	196.6122
15	4	12	14	3	11	A	1	107455.648	0.022	201.7526
18	7	11	17	7	10	E	1	107462.050	0.021	218.1058

J'	K'_a	K'_c	J''	K''_a	K''_c	Sym.	$v_{t_{CH_3}}$	$\nu_{Obs.}$	$\nu_{Obs.} - \nu_{Calc.}$	E_{low}
6	4	3	5	2	4	E	0	107474.045	0.253	65.3281
18	7	12	17	7	11	A	1	107515.737	-0.081	218.2652
18	7	12	17	7	11	A	0	107554.656	0.117	101.5273
18	7	12	17	7	11	E	1	107574.619	0.018	217.6581
18	7	12	17	7	11	E	0	107592.754	0.016	101.5335
18	5	14	17	5	13	A	1	107607.684	-0.102	213.7168
18	7	11	17	7	10	E	0	107619.293	0.016	101.5480
18	7	11	17	7	10	A	1	107620.868	-0.078	218.2682
18	7	11	17	7	10	A	0	107654.398	0.087	101.5300
18	5	14	17	5	13	A	0	107679.158	0.228	96.9239
18	5	14	17	5	13	E	0	107679.158	-0.380	96.9385
18	3	15	17	3	14	A	1	107706.264	-0.077	211.5585
26	10	16	26	9	17	A	0	107711.695	0.199	147.2102
26	10	16	26	9	17	E	0	107775.784	0.231	147.2239
26	10	17	26	9	18	E	0	107831.963	0.196	147.2042
26	10	17	26	9	18	E	1	107841.435	-0.337	263.3049
18	3	15	17	3	14	E	0	107880.986	-0.078	94.7526
18	3	15	17	3	14	E	1	107886.484	-0.411	211.0485
18	3	15	17	3	14	A	0	107887.567	-0.042	94.7366
18	6	13	17	6	12	A	1	107992.564	-0.017	215.8038
26	10	17	26	9	18	A	0	108012.601	0.150	147.1993
18	6	13	17	6	12	A	0	108036.353	0.048	99.0356
18	6	13	17	6	12	E	0	108051.380	0.037	99.0482
15	4	12	14	3	11	A	0	108065.417	-0.512	84.9164
25	10	16	25	9	16	E	0	108098.784	0.020	142.0101
49	11	38	49	10	39	A	0	108226.419	-0.619	330.8924
49	11	38	49	10	39	E	0	108282.875	-0.649	330.9002
18	5	14	17	5	13	E	1	108287.747	0.243	213.1844
37	11	27	37	10	28	E	1	108331.520	0.180	337.9667
18	6	12	17	6	11	E	1	108373.687	-0.081	215.5832
18	6	13	17	6	12	E	1	108472.636	0.075	215.1988
39	10	30	39	9	31	E	0	108604.192	0.285	233.7393
25	10	15	25	9	16	A	0	108607.716	0.179	141.9950
25	10	16	25	9	17	E	0	108620.664	0.166	141.9927
39	10	30	39	9	31	A	0	108631.785	0.164	233.7297
25	10	15	25	9	16	E	0	108634.232	0.279	142.0101
25	10	16	25	9	17	E	1	108682.637	-0.226	258.0943
25	10	16	25	9	17	A	0	108762.441	0.267	141.9894
18	6	12	17	6	11	E	0	108991.659	0.133	99.0871
18	6	12	17	6	11	A	1	108996.489	-0.034	215.8423
18	6	12	17	6	11	A	0	109002.037	0.134	99.0725
16	4	13	15	3	12	E	1	109314.242	0.283	204.3319
24	10	15	24	9	16	E	0	109322.101	0.176	136.9915
24	10	14	24	9	15	E	0	109354.698	0.316	137.0083
24	10	14	24	9	15	A	0	109357.293	0.295	136.9921
34	6	28	34	5	29	E	1	109378.050	0.065	302.3934
36	11	26	36	10	27	E	1	109500.365	0.114	330.4333
34	6	28	34	5	29	E	0	109530.274	0.132	186.0950
34	6	28	34	5	29	A	0	109533.251	0.242	186.0831
16	4	13	15	3	12	E	0	109546.231	-0.296	88.0346
43	9	34	43	9	35	E	1	109933.998	0.329	383.0481
23	10	13	23	9	14	E	0	109970.234	0.203	132.2163
23	10	13	23	9	14	A	0	109990.621	0.266	132.1994
9	6	4	8	4	5	A	1	170059.538	0.324	188.6898
40	4	37	40	3	38	E	1	170059.544	-0.484	328.8096
40	3	37	40	3	38	E	1	170059.544	-0.484	328.8096
40	4	37	40	2	38	E	1	170059.544	-0.484	328.8096
40	3	37	40	2	38	E	1	170059.544	-0.484	328.8096
29	6	24	28	6	23	A	1	170217.987	-0.083	266.2115
30	4	26	29	5	25	A	1	170225.452	0.002	268.6621
9	6	4	8	4	5	E	1	170237.167	-0.105	188.0575
28	8	21	27	8	20	E	1	170316.033	0.132	265.6683
30	5	26	29	5	25	A	1	170367.663	-0.070	268.6621
29	27	2	28	27	1	E	1	170372.686	-0.409	402.5729
31	3	28	30	4	27	A	1	170374.729	-0.130	270.6198
29	27	2	28	27	1	A	1	170377.373	-0.177	402.0238
29	27	3	28	27	2	A	1	170377.373	-0.177	402.0238
31	4	28	30	4	27	A	1	170379.459	-0.088	270.6198
30	4	26	29	5	25	E	1	170380.925	0.585	268.2366
31	3	28	30	3	27	A	1	170383.444	-0.061	270.6195
31	4	28	30	3	27	A	1	170388.298	0.106	270.6195
30	4	26	29	5	25	E	0	170388.302	-0.052	151.9373
30	4	26	29	5	25	A	0	170392.858	0.014	151.9240
29	6	24	28	6	23	E	0	170401.872	-0.047	149.4946
29	6	24	28	6	23	A	0	170407.098	-0.044	149.4816
29	6	24	28	6	23	E	1	170421.052	-0.173	265.7918
14	8	7	13	7	6	A	1	170427.205	0.385	205.9705
29	26	3	28	26	2	E	1	170430.685	0.083	392.0902
29	28	2	28	28	1	E	0	170433.628	-0.357	296.7877
30	4	26	29	4	25	A	1	170471.057	-0.051	268.6539
29	25	5	28	25	4	E	1	170473.560	0.025	381.8580
29	27	2	28	27	1	A	0	170478.079	-0.276	285.8963
29	27	3	28	27	2	A	0	170478.079	-0.276	285.8963
29	27	3	28	27	2	E	0	170478.079	-0.403	285.8789
32	2	30	31	2	29	A	1	170502.914	-0.085	272.1105
32	3	30	31	2	29	A	1	170502.914	-0.085	272.1105
32	2	30	31	3	29	A	1	170502.914	-0.085	272.1105
32	3	30	31	3	29	A	1	170502.914	-0.085	272.1105
31	4	28	30	4	27	E	1	170514.905	-0.106	270.1799
31	3	28	30	3	27	E	1	170519.524	-0.059	270.1796
31	3	28	30	4	27	E	0	170522.862	-0.107	153.8826
31	3	28	30	4	27	A	0	170526.958	-0.137	153.8688
29	26	4	28	26	3	E	0	170526.958	-0.102	275.3656
31	4	28	30	4	27	E	0	170528.197	0.001	153.8826
31	3	28	30	3	27	E	0	170532.439	-0.127	153.8823
31	3	28	30	3	27	E	0	170532.439	-0.127	153.8823
31	4	28	30	4	27	A	0	170532.445	0.090	153.8688
31	3	28	30	3	27	A	0	170536.647	-0.104	153.8685
31	4	28	30	3	27	E	0	170538.055	0.262	153.8823
30	5	26	29	5	25	E	1	170542.802	-0.300	268.2366
30	5	26	29	5	25	E	0	170544.206	-0.107	151.9373
30	5	26	29	5	25	A	0	170549.593	-0.046	151.9240
29	25	4	28	25	3	A	0	170581.063	-0.073	265.2694
29	25	5	28	25	4	A	0	170581.063	-0.073	265.2694
29	25	5	28	25	4	E	0	170581.063	-0.019	265.2502
32	3	30	31	3	29	E	1	170599.377	-0.088	271.6472
32	2	30	31	2	29	E	1	170599.377	-0.088	271.6472
32	2	30	31	2	29	E	0	170623.039	-0.026	155.3550
32	3	30	31	2	29	E	0	170623.039	-0.026	155.3550
32	2	30	31	3	29	E	0	170623.039	-0.026	155.3550
32	3	30	31	3	29	E	0	170623.039	-0.026	155.3550
32	2	30	31	2	29	A	0	170625.981	-0.041	155.3405
32	3	30	31	2	29	A	0	170625.981	-0.041	155.3405
32	2	30	31	3	29	A	0	170625.981	-0.041	155.3405
32	3	30	31	3	29	A	0	170625.981	-0.041	155.3405
29	24	5	28	24	4	A	0	170641.185	0.025	255.5528

J'	K_a'	K_c'	J''	K_a''	K_c''	Sym.	$v_{t_{CH_3}}$	$\nu_{Obs.}$	$\nu_{Obs.} - \nu_{Calc.}$	E_{low}
29	24	6	28	24	5	A	0	170641.185	0.025	255.5528
29	24	6	28	24	5	E	0	170641.185	0.007	255.5331
30	4	26	29	4	25	E	0	170656.378	-0.027	151.9284
30	4	26	29	4	25	E	1	170658.848	-0.137	268.2273
30	4	26	29	4	25	A	0	170662.299	0.040	151.9150
29	22	8	28	22	7	E	1	170673.506	-0.063	353.7651
29	22	7	28	22	6	E	1	170682.613	-0.031	354.1175
33	1	32	32	1	31	A	1	170688.467	-0.110	273.1687
33	2	32	32	1	31	A	1	170688.467	-0.110	273.1687
33	1	32	32	2	31	A	1	170688.467	-0.110	273.1687
33	2	32	32	2	31	A	1	170688.467	-0.110	273.1687
29	22	7	28	22	6	A	1	170690.009	0.214	353.4822
29	22	8	28	22	7	A	1	170690.009	0.214	353.4822
29	23	6	28	23	5	A	0	170708.418	0.047	246.2346
29	23	7	28	23	6	A	0	170708.418	0.047	246.2346
29	23	7	28	23	6	E	0	170708.418	-0.059	246.2146
33	2	32	32	2	31	E	1	170746.078	-0.076	272.6733
33	1	32	32	2	31	E	1	170746.078	-0.076	272.6733
33	2	32	32	1	31	E	1	170746.078	-0.076	272.6733
33	1	32	32	1	31	E	1	170746.078	-0.076	272.6733
29	21	9	28	21	8	E	1	170758.868	-0.046	345.1990
29	21	8	28	21	7	E	1	170766.568	-0.046	345.6113
29	21	9	28	21	8	A	1	170777.677	-0.011	344.9841
29	21	8	28	21	7	A	1	170777.677	-0.011	344.9841
33	1	32	32	1	31	E	0	170779.688	-0.019	156.3889
33	2	32	32	1	31	E	0	170779.688	-0.019	156.3889
33	1	32	32	2	31	E	0	170779.688	-0.019	156.3889
33	2	32	32	2	31	E	0	170779.688	-0.019	156.3889
33	1	32	32	1	31	A	0	170781.501	0.034	156.3734
33	2	32	32	1	31	A	0	170781.501	0.034	156.3734
33	1	32	32	2	31	A	0	170781.501	0.034	156.3734
33	2	32	32	2	31	A	0	170781.501	0.034	156.3734
29	22	7	28	22	6	A	0	170784.298	0.133	237.3150
29	22	8	28	22	7	A	0	170784.298	0.133	237.3150
29	22	8	28	22	7	E	0	170784.298	-0.078	237.2951
30	5	26	29	4	25	E	0	170812.118	-0.247	151.9284
30	5	26	29	4	25	A	0	170818.974	-0.078	151.9150
30	5	26	29	4	25	E	1	170821.683	-0.064	268.2273
28	15	13	28	14	14	A	0	170856.896	-1.891	180.3968
28	15	14	28	14	15	A	0	170856.896	-1.891	180.3968
29	20	10	28	20	9	E	1	170856.898	-0.053	337.0338
29	20	9	28	20	8	E	1	170862.628	0.001	337.5002
29	21	8	28	21	7	A	0	170870.486	0.200	228.7946
29	21	9	28	21	8	A	0	170870.486	0.200	228.7946
29	21	9	28	21	8	E	0	170870.486	-0.132	228.7749
29	20	9	28	20	8	A	1	170878.516	0.224	336.8899
29	20	10	28	20	9	A	1	170878.516	0.224	336.8899
34	0	34	33	0	33	A	1	170900.428	-0.037	273.8163
34	1	34	33	0	33	A	1	170900.428	-0.037	273.8163
34	0	34	33	1	33	A	1	170900.428	-0.037	273.8163
34	1	34	33	1	33	A	1	170900.428	-0.037	273.8163
34	1	34	33	1	33	E	1	170916.939	-0.082	273.2802
34	0	34	33	1	33	E	1	170916.939	-0.082	273.2802
34	1	34	33	0	33	E	1	170916.939	-0.082	273.2802
34	0	34	33	0	33	E	1	170916.939	-0.082	273.2802
34	0	34	33	0	33	A	0	170961.250	-0.232	156.9896
34	1	34	33	0	33	A	0	170961.250	-0.232	156.9896
34	0	34	33	1	33	A	0	170961.250	-0.232	156.9896
34	1	34	33	1	33	A	0	170961.250	-0.232	156.9896
34	0	34	33	0	33	E	0	170961.250	0.272	157.0063
34	1	34	33	0	33	E	0	170961.250	0.272	157.0063
34	0	34	33	1	33	E	0	170961.250	0.272	157.0063
34	1	34	33	1	33	E	0	170961.250	0.272	157.0063
29	20	9	28	20	8	A	0	170969.185	0.247	220.6738
29	20	10	28	20	9	A	0	170969.185	0.247	220.6738
29	20	10	28	20	9	E	0	170969.185	-0.228	220.6547
42	4	38	42	3	39	E	0	170986.907	0.055	233.0355
42	5	38	42	3	39	E	0	170986.907	0.055	233.0355
42	5	38	42	4	39	E	0	170986.907	0.055	233.0355
42	4	38	42	3	39	A	0	171018.880	-0.011	233.0234
42	5	38	42	3	39	A	0	171018.880	-0.011	233.0234
42	5	38	42	4	39	A	0	171018.880	-0.011	233.0234
29	19	10	28	19	9	A	0	171083.297	0.351	212.9533
29	19	11	28	19	10	A	0	171083.297	0.351	212.9533
29	19	11	28	19	10	E	0	171083.297	-0.291	212.9350
29	18	12	28	18	11	E	1	171102.926	-0.047	321.9111
29	18	11	28	18	10	E	1	171102.926	-0.047	322.4652
29	18	11	28	18	10	A	1	171129.939	0.085	321.9143
29	18	12	28	18	11	A	1	171129.939	0.085	321.9143
28	8	20	27	8	19	E	1	171145.402	-0.108	266.1371
16	7	9	15	6	10	E	0	171152.486	0.365	92.4566
29	18	11	28	18	10	A	0	171216.453	0.469	205.6340
29	18	12	28	18	11	A	0	171216.453	0.469	205.6340
29	18	11	28	18	10	E	0	171216.453	-0.370	205.6341
29	18	12	28	18	11	E	0	171216.453	-0.370	205.6168
29	17	12	28	17	11	E	1	171254.655	0.011	315.5430
29	17	13	28	17	12	E	1	171260.457	-0.048	314.9566
50	8	42	50	7	43	E	1	171281.797	-0.189	441.4764
29	17	12	28	17	11	A	1	171289.482	0.090	315.0339
29	17	13	28	17	12	A	1	171289.482	0.090	315.0339
50	9	42	50	8	43	E	1	171349.292	-0.363	441.4766
29	17	12	28	17	11	A	0	171372.923	0.019	198.7170
29	17	13	28	17	12	A	0	171372.923	0.019	198.7170
29	17	12	28	17	11	E	0	171374.027	0.131	198.7194
29	17	13	28	17	12	E	0	171374.027	0.131	198.7011
14	8	7	13	7	7	E	0	171396.998	0.464	89.2323
43	11	32	42	12	31	E	0	171404.741	-0.032	270.0814
29	16	13	28	16	12	E	1	171436.204	0.138	309.0195
14	8	6	13	7	6	E	0	171442.204	0.016	89.2467
29	16	14	28	16	13	E	1	171448.427	-0.097	308.4091
32	14	19	31	14	17	E	1	171448.427	-0.097	314.9903
14	8	7	13	7	6	A	0	171464.650	-0.163	89.2276
14	8	6	13	7	7	A	0	171466.624	-0.145	89.2275
29	16	13	28	16	12	A	1	171479.694	0.033	308.5596
29	16	14	28	16	13	A	1	171479.694	0.033	308.5596
29	16	13	28	16	12	A	0	171560.278	0.026	192.2036
29	16	14	28	16	13	A	0	171560.278	0.026	192.2036
29	16	13	28	16	12	E	0	171561.548	0.131	192.2084
29	16	14	28	16	13	E	0	171561.548	0.131	192.1893
16	4	12	15	2	13	E	1	171573.537	0.232	202.9935
29	5	24	28	5	23	A	1	171615.588	-0.090	266.0819
29	15	14	28	15	13	E	1	171655.456	-0.015	302.8965
12	9	4	11	8	4	E	0	171660.924	0.022	87.2645
29	15	15	28	15	14	E	1	171676.511	0.039	302.2711
37	2	36	37	1	37	E	1	171682.496	-0.467	297.0767
37	1	36	37	1	37	E	1	171682.496	-0.467	297.0767

J'	K'_a	K'_c	J''	K''_a	K''_c	Sym.	$v_{t_{CH_3}}$	$\nu_{Obs.}$	$\nu_{Obs.} - \nu_{Calc.}$	E_{low}
37	2	36	37	0	37	E	1	171682.496	-0.467	297.0767
37	1	36	37	0	37	E	1	171682.496	-0.467	297.0767
12	9	3	11	8	3	E	0	171699.370	0.001	87.2805
29	15	15	28	15	14	A	1	171709.810	0.039	302.4929
29	15	14	28	15	13	A	1	171709.810	0.039	302.4929
12	9	4	11	8	3	A	0	171730.816	0.076	87.2621
12	9	3	11	8	4	A	0	171730.816	0.076	87.2621
44	5	39	44	4	40	E	0	171754.099	0.110	254.5662
44	6	39	44	4	40	A	0	171782.646	0.007	254.5554
44	6	39	44	5	40	A	0	171782.646	0.007	254.5554
29	15	14	28	15	13	A	0	171787.046	-0.017	186.0960
29	15	15	28	15	14	A	0	171787.046	-0.017	186.0960
29	15	14	28	15	13	E	0	171788.479	0.052	186.1030
29	15	15	28	15	14	E	0	171788.479	0.052	186.0834
10	10	1	9	9	1	E	0	171821.805	0.337	86.5094
10	10	0	9	9	0	E	0	171853.129	0.435	86.5267
29	5	24	28	5	23	E	0	171882.931	-0.130	149.3559
10	10	1	9	9	0	A	0	171891.401	0.178	86.5094
10	10	0	9	9	1	A	0	171891.401	0.178	86.5094
29	5	24	28	5	23	A	0	171893.205	-0.056	149.3423
29	5	24	28	5	23	E	1	171922.864	-0.189	265.6498
29	14	15	28	14	14	E	1	171925.167	0.015	297.1770
29	14	16	28	14	15	E	1	171957.685	0.029	296.5457
30	25	6	29	25	4	E	1	171986.878	-0.083	387.6915
29	14	16	28	14	15	A	1	171992.679	-0.010	296.8359
29	14	15	28	14	14	A	1	171992.679	-0.010	296.8359
29	14	15	28	14	14	A	0	172066.195	0.010	180.3968
29	14	16	28	14	15	A	0	172066.195	0.010	180.3968
29	14	16	28	14	15	E	0	172068.236	-0.041	180.3861
46	6	40	46	5	41	A	0	172140.192	0.236	277.0992
46	7	40	46	5	41	A	0	172144.032	0.379	277.0992
46	7	40	46	6	41	A	0	172144.032	0.379	277.0992
46	6	40	46	6	41	E	1	172146.889	-0.209	393.4008
46	6	40	46	5	41	E	1	172146.889	-0.209	393.4008
46	7	40	46	6	41	E	1	172151.008	-0.216	393.4008
46	7	40	46	5	41	E	1	172151.008	-0.216	393.4008
29	13	16	28	13	15	E	1	172263.165	-0.008	291.8647
29	13	17	28	13	16	E	1	172311.396	0.002	291.2372
29	13	17	28	13	16	A	1	172347.562	-0.011	291.5919
29	13	16	28	13	15	A	1	172347.562	-0.011	291.5919
28	8	20	27	8	19	A	0	172405.207	0.108	149.6545
28	8	20	27	8	19	E	0	172410.698	0.070	149.6664
29	13	16	28	13	15	A	0	172416.627	0.052	175.1098
29	13	17	28	13	16	A	0	172416.627	0.052	175.1098
29	13	17	28	13	16	E	0	172419.322	0.124	175.1013
28	8	20	27	8	19	A	1	172456.705	-0.111	266.3517
29	12	17	28	12	16	E	1	172697.288	-0.108	286.9655
29	12	18	28	12	17	E	1	172767.912	0.043	286.3516
29	12	18	28	12	17	A	1	172804.183	-0.020	286.7662
29	12	17	28	12	16	A	1	172804.183	-0.020	286.7662
29	12	17	28	12	16	A	0	172867.679	0.030	170.2409
29	12	18	28	12	17	A	0	172867.679	0.030	170.2409
29	12	17	28	12	16	E	0	172868.690	-0.072	170.2541
29	12	18	28	12	17	E	0	172871.048	0.067	170.2347
19	5	15	18	4	15	E	0	173207.037	-0.260	98.5369
29	11	18	28	11	17	E	1	173273.968	0.066	282.4882
29	11	19	28	11	18	E	1	173377.798	0.095	281.8986
29	11	19	28	11	18	A	1	173407.706	0.026	282.3671
18	6	12	17	5	13	A	0	173415.926	-0.467	96.9239
29	11	18	28	11	17	A	1	173417.175	0.030	282.3674
29	11	19	28	11	18	A	0	173464.229	0.105	165.7989
29	11	18	28	11	17	E	0	173469.693	0.052	165.8139
29	11	18	28	11	17	A	0	173472.881	-0.002	165.7992
29	11	19	28	11	18	E	0	173472.881	0.133	165.7953
41	3	38	41	2	39	A	1	173677.973	-0.010	336.4188
41	4	38	41	2	39	A	1	173677.973	-0.010	336.4188
41	3	38	41	3	39	A	1	173677.973	-0.010	336.4188
41	4	38	41	3	39	A	1	173677.973	-0.010	336.4188
30	5	25	29	6	24	A	1	173706.990	-0.442	271.8894
30	5	25	29	6	24	E	1	173728.338	0.081	271.4765
30	5	25	29	6	24	A	0	173771.229	0.009	155.1657
30	5	25	29	6	24	E	0	173773.327	0.079	155.1786
29	7	23	28	7	22	A	1	173923.028	-0.092	269.0253
45	16	30	45	15	31	A	0	173995.181	-0.465	311.3721
29	7	23	28	7	22	E	0	174071.508	0.087	152.3248
29	7	23	28	7	22	A	0	174073.927	0.103	152.3124
29	10	19	28	10	18	E	1	174078.423	0.087	278.4475
29	6	24	28	5	23	A	1	174105.100	0.041	266.0819
29	10	20	28	10	19	A	1	174211.124	-0.056	278.4078
29	10	20	28	10	19	E	1	174236.522	0.075	277.8938
29	7	23	28	7	22	E	1	174244.265	0.057	268.6080
29	10	20	28	10	19	A	0	174260.029	0.056	161.7975
29	10	20	28	10	19	E	0	174302.223	0.050	161.7980
29	10	19	28	10	18	A	1	174321.383	-0.069	278.4116
29	10	19	28	10	18	E	0	174327.541	0.107	161.8158
28	6	22	27	6	21	A	1	174357.004	-0.056	262.5394
29	10	19	28	10	18	A	0	174362.951	0.107	161.8011
28	6	22	27	6	21	E	1	174556.431	-0.268	262.0853
29	6	24	28	5	23	E	0	174560.502	-0.137	149.3559
29	6	24	28	5	23	A	0	174582.098	-0.134	149.3423
28	6	22	27	6	21	E	0	174610.985	0.002	145.7987
28	6	22	27	6	21	A	0	174619.296	-0.007	145.7841
41	3	38	41	2	39	E	0	174661.603	-0.170	219.7052
41	4	38	41	2	39	E	0	174661.603	-0.170	219.7052
41	4	38	41	3	39	E	0	174661.603	-0.170	219.7052
29	6	24	28	5	23	E	1	174678.017	-0.502	265.6498
41	3	38	41	2	39	A	0	174698.588	-0.142	219.6916
41	4	38	41	2	39	A	0	174698.588	-0.142	219.6916
41	3	38	41	3	39	A	0	174698.588	-0.142	219.6916
41	4	38	41	3	39	A	0	174698.588	-0.142	219.6916
29	10	19	28	10	19	E	0	174862.028	0.087	161.7980
41	4	38	41	3	39	E	1	174876.380	0.494	335.9871
41	3	38	41	3	39	E	1	174876.380	0.494	335.9871
41	4	38	41	2	39	E	1	174876.380	0.494	335.9871
41	3	38	41	2	39	E	1	174876.380	0.494	335.9871
43	4	39	43	3	40	A	1	174922.972	0.280	357.3752
43	5	39	43	3	40	A	1	174922.972	0.280	357.3752
43	5	39	43	4	40	A	1	174922.972	0.280	357.3752
29	9	21	28	9	20	A	1	175125.334	-0.062	274.9021
29	9	21	28	9	20	A	0	175175.899	-0.006	158.2515
29	9	21	28	9	20	E	0	175200.189	0.110	158.2594
31	4	27	30	5	26	A	1	175222.963	-0.429	274.3450
30	6	25	29	6	24	A	1	175266.861	-0.076	271.8894
31	5	27	30	5	26	A	1	175304.808	-0.089	274.3450
32	3	29	31	4	28	A	1	175322.080	-0.153	276.3030
32	4	29	31	4	28	A	1	175324.709	-0.049	276.3030

J'	K'_a	K'_c	J''	K''_a	K''_c	Sym.	$v_{t_{CH_3}}$	$\nu_{Obs.}$	$\nu_{Obs.} - \nu_{Calc.}$	E_{low}
32	3	29	31	3	28	A	1	175326.863	-0.058	276.3029
32	4	29	31	3	28	A	1	175329.465	0.020	276.3029
29	9	20	28	9	19	E	1	175338.165	0.041	274.8707
31	4	27	30	4	26	A	1	175365.614	-0.062	274.3402
31	4	27	30	5	26	E	1	175385.294	-0.083	273.9253
31	4	27	30	5	26	E	0	175392.990	-0.094	157.6261
31	4	27	30	5	26	A	0	175397.832	-0.078	157.6129
31	5	27	30	4	26	A	1	175447.257	0.077	274.3402
29	8	22	28	8	21	A	1	175452.702	-0.047	271.8259
33	2	31	32	2	30	A	1	175455.565	-0.050	277.7979
33	3	31	32	2	30	A	1	175455.565	-0.050	277.7979
33	2	31	32	3	30	A	1	175455.565	-0.050	277.7979
33	3	31	32	3	30	A	1	175455.565	-0.050	277.7979
30	6	25	29	6	24	E	0	175459.119	0.020	155.1786
32	4	29	31	4	28	E	1	175459.127	-0.098	275.8677
32	3	29	31	3	28	E	1	175461.687	-0.058	275.8675
30	6	25	29	6	24	A	0	175464.696	-0.055	155.1657
32	4	29	31	3	28	E	1	175464.702	-0.038	275.8675
32	3	29	31	4	28	E	0	175471.401	-0.088	159.5708
30	6	25	29	6	24	E	1	175471.403	-0.079	271.4765
32	4	29	31	4	28	E	0	175474.164	-0.153	159.5708
32	3	29	31	3	28	E	0	175476.663	-0.053	159.5706
32	4	29	31	4	28	A	0	175478.547	0.099	159.5572
32	3	29	31	3	28	A	0	175480.866	0.004	159.5570
31	5	27	30	5	26	E	0	175482.760	-0.068	157.6261
31	5	27	30	5	26	A	0	175488.164	0.007	157.6129
29	9	21	28	9	20	E	1	175528.767	0.195	274.3667
29	8	22	28	8	21	A	0	175538.946	0.010	155.1396
29	8	22	28	8	21	E	0	175542.249	0.073	155.1506
31	4	27	30	4	26	E	0	175548.986	-0.058	157.6209
33	3	31	32	3	30	E	1	175551.549	-0.060	277.3378
33	2	31	32	2	30	E	1	175551.549	-0.060	277.3378
31	4	27	30	4	26	A	0	175554.660	-0.043	157.6077
33	2	31	32	2	30	E	0	175576.840	-0.026	161.0464
33	3	31	32	2	30	E	0	175576.840	-0.026	161.0464
33	2	31	32	3	30	E	0	175576.840	-0.026	161.0464
33	3	31	32	3	30	E	0	175576.840	-0.026	161.0464
33	2	31	32	2	30	A	0	175579.780	-0.028	161.0320
33	3	31	32	2	30	A	0	175579.780	-0.028	161.0320
33	2	31	32	3	30	A	0	175579.780	-0.028	161.0320
33	3	31	32	3	30	A	0	175579.780	-0.028	161.0320
31	5	27	30	4	26	E	0	175638.292	-0.496	157.6209
34	1	33	33	1	32	A	1	175643.487	-0.085	278.8622
34	2	33	33	1	32	A	1	175643.487	-0.085	278.8622
34	1	33	33	2	32	A	1	175643.487	-0.085	278.8622
34	2	33	33	2	32	A	1	175643.487	-0.085	278.8622
31	5	27	30	4	26	A	0	175645.223	0.272	157.6077
34	2	33	33	2	32	E	1	175700.815	-0.128	278.3688
34	1	33	33	1	32	E	1	175700.815	-0.128	278.3688
49	13	36	48	14	35	A	0	175713.690	-0.188	334.5908
34	1	33	33	1	32	E	0	175735.945	-0.071	162.0855
34	2	33	33	1	32	E	0	175735.945	-0.071	162.0855
34	1	33	33	2	32	E	0	175735.945	-0.071	162.0855
34	2	33	33	2	32	E	0	175735.945	-0.071	162.0855
34	1	33	33	1	32	A	0	175737.764	-0.006	162.0701
34	2	33	33	1	32	A	0	175737.764	-0.006	162.0701
34	1	33	33	2	32	A	0	175737.764	-0.006	162.0701
34	2	33	33	2	32	A	0	175737.764	-0.006	162.0701
28	7	21	27	7	20	E	1	175796.671	-0.074	263.7934
43	16	27	43	15	28	E	0	175818.749	-0.091	293.5214
43	16	28	43	15	29	E	0	175824.077	-0.477	293.5027
43	4	39	43	3	40	E	0	175830.908	0.053	240.7050
43	5	39	43	3	40	E	0	175830.908	0.053	240.7050
43	5	39	43	4	40	E	0	175830.908	0.053	240.7050
35	0	35	34	0	34	A	1	175856.033	-0.008	279.5169
35	1	35	34	0	34	A	1	175856.033	-0.008	279.5169
35	0	35	34	1	34	A	1	175856.033	-0.008	279.5169
35	1	35	34	1	34	A	1	175856.033	-0.008	279.5169
43	4	39	43	3	40	A	0	175863.908	0.020	240.6930
43	5	39	43	3	40	A	0	175863.908	0.020	240.6930
43	5	39	43	4	40	A	0	175863.908	0.020	240.6930
35	1	35	34	1	34	E	1	175872.332	-0.129	278.9814
35	0	35	34	0	34	E	1	175872.332	-0.129	278.9814
35	0	35	34	0	34	A	0	175918.121	-0.249	162.6922
35	1	35	34	0	34	A	0	175918.121	-0.249	162.6922
35	0	35	34	1	34	A	0	175918.121	-0.249	162.6922
35	1	35	34	1	34	A	0	175918.121	-0.249	162.6922
35	0	35	34	0	34	E	0	175918.121	0.252	162.7089
35	1	35	34	0	34	E	0	175918.121	0.252	162.7089
35	0	35	34	1	34	E	0	175918.121	0.252	162.7089
35	1	35	34	1	34	E	0	175918.121	0.252	162.7089
43	5	39	43	4	40	E	1	175988.902	-0.428	356.9918
43	4	39	43	4	40	E	1	175988.902	-0.428	356.9918
43	5	39	43	3	40	E	1	175988.902	-0.428	356.9918
43	4	39	43	3	40	E	1	175988.902	-0.428	356.9918
29	9	20	28	9	19	E	0	176022.157	0.076	158.3004
29	9	20	28	9	19	A	0	176037.025	0.094	158.2891
29	9	20	28	9	19	A	1	176037.036	-0.206	274.9421
30	5	25	29	5	24	A	1	176196.672	-0.142	271.8063
30	29	1	29	29	0	A	0	176301.690	-0.413	313.7970
30	29	2	29	29	1	A	0	176301.690	-0.413	313.7970
30	29	2	29	29	1	E	0	176301.690	-0.442	313.7778
28	7	21	27	7	20	A	1	176304.207	-0.136	264.1954
28	7	21	27	7	20	A	0	176342.671	0.060	147.4559
30	28	2	29	28	1	A	0	176346.747	0.032	302.4912
30	28	3	29	28	2	A	0	176346.747	0.032	302.4912
28	7	21	27	7	20	E	0	176346.747	-0.196	147.4697
30	27	3	29	27	2	A	0	176395.332	-0.176	291.5828
30	27	4	29	27	3	A	0	176395.332	-0.176	291.5828
30	27	4	29	27	3	E	0	176395.332	-0.342	291.5655
17	7	11	16	6	11	E	0	176397.876	0.022	95.6495
30	25	5	29	25	4	E	1	176406.962	-0.451	387.6915
29	8	22	28	8	21	E	1	176434.074	0.102	271.3494
30	26	4	29	26	3	A	0	176448.976	-0.202	281.0721
30	26	5	29	26	4	A	0	176448.976	-0.202	281.0721
30	26	5	29	26	4	E	0	176448.976	-0.105	281.0538
30	5	25	29	5	24	E	0	176450.796	-0.030	155.0893
30	5	25	29	5	24	A	0	176460.149	-0.041	155.0760
13	9	5	12	8	4	A	1	176472.522	0.165	206.3346
13	9	4	12	8	5	A	1	176472.522	0.165	206.3346
30	24	6	29	24	5	A	1	176474.433	0.035	377.3778
30	24	7	29	24	6	A	1	176474.433	0.035	377.3778
30	5	25	29	5	24	E	1	176483.658	-0.065	271.3846
30	25	5	29	25	4	A	0	176508.494	-0.067	270.9593
30	25	6	29	25	5	A	0	176508.494	-0.067	270.9593
30	25	6	29	25	5	E	0	176508.494	-0.037	270.9402

J'	K_a'	K_c'	J''	K_a''	K_c''	Sym.	$v_{t_{CH_3}}$	$\nu_{Obs.}$	$\nu_{Obs.} - \nu_{Calc.}$	E_{low}
30	23	7	29	23	6	E	1	176544.723	-0.150	368.7094
30	24	6	29	24	5	A	0	176574.666	-0.010	261.2448
30	24	7	29	24	6	A	0	176574.666	-0.010	261.2448
30	24	7	29	24	6	E	0	176574.666	-0.062	261.2251
30	22	9	29	22	8	E	1	176617.252	0.097	359.4581
30	22	8	29	22	7	E	1	176626.661	0.098	359.8109
30	22	8	29	22	7	A	1	176635.487	0.047	359.1758
30	22	9	29	22	8	A	1	176635.487	0.047	359.1758
30	23	7	29	23	6	A	0	176648.839	0.065	251.9288
30	23	8	29	23	7	A	0	176648.839	0.065	251.9288
30	23	8	29	23	7	E	0	176648.839	-0.084	251.9088
42	16	26	42	15	27	E	0	176656.161	0.377	284.9095
45	5	40	45	4	41	A	0	176676.554	0.413	262.7167
45	6	40	45	4	41	A	0	176676.554	0.413	262.7167
45	5	40	45	5	41	A	0	176676.554	0.413	262.7167
45	6	40	45	5	41	A	0	176676.554	0.413	262.7167
30	21	10	29	21	9	E	1	176711.482	0.021	350.8949
30	21	9	29	21	8	E	1	176719.325	0.057	351.3075
42	16	27	42	15	28	A	0	176727.469	0.108	284.9015
30	22	8	29	22	7	A	0	176732.510	0.104	243.0118
30	22	9	29	22	8	A	0	176732.510	0.104	243.0118
30	22	9	29	22	8	E	0	176732.510	-0.160	242.9918
30	21	10	29	21	9	A	1	176732.515	0.046	350.6807
30	21	9	29	21	8	A	1	176732.515	0.046	350.6807
45	6	40	45	5	41	E	1	176746.581	-0.250	379.0171
45	5	40	45	5	41	E	1	176746.581	-0.250	379.0171
45	6	40	45	4	41	E	1	176746.581	-0.250	379.0171
45	5	40	45	4	41	E	1	176746.581	-0.250	379.0171
30	20	11	29	20	10	E	1	176819.810	-0.056	342.7330
30	20	10	29	20	9	E	1	176825.292	-0.070	343.1996
30	21	9	29	21	8	A	0	176827.646	0.137	234.4942
30	21	10	29	21	9	A	0	176827.646	0.137	234.4942
30	21	10	29	21	9	E	0	176827.646	-0.260	234.4745
30	20	10	29	20	9	A	0	176936.869	0.333	226.3767
30	20	11	29	20	10	A	0	176936.869	0.333	226.3767
30	20	11	29	20	10	E	0	176936.869	-0.221	226.3576
30	19	12	29	19	11	E	1	176945.520	-0.041	334.9737
30	19	11	29	19	10	E	1	176947.934	0.044	335.4877
30	19	11	29	19	10	A	1	176972.148	0.097	334.9036
30	19	12	29	19	11	A	1	176972.148	0.097	334.9036
30	19	11	29	19	10	A	0	177062.973	0.338	218.6600
30	19	12	29	19	11	A	0	177062.973	0.338	218.6600
30	19	12	29	19	11	E	0	177062.973	-0.400	218.6417
47	6	41	47	5	42	A	0	177101.261	0.362	285.7533
30	18	12	29	18	11	A	1	177121.897	0.002	327.6225
30	18	13	29	18	12	A	1	177121.897	0.002	327.6225
30	18	12	29	18	11	A	0	177209.943	0.040	211.3452
30	18	13	29	18	12	A	0	177209.943	0.040	211.3452
30	18	12	29	18	11	E	0	177210.884	0.040	211.3453
30	18	13	29	18	12	E	0	177210.884	0.040	211.3280
15	8	7	14	7	7	E	0	177240.368	-0.162	92.0163
30	17	13	29	17	12	E	1	177259.399	0.049	321.2555
15	8	7	14	7	8	A	0	177266.609	-0.308	91.9970
30	17	14	29	17	13	E	1	177266.617	-0.183	320.6692
15	3	12	14	2	13	E	1	177288.401	0.539	198.4182
30	17	14	29	17	13	A	1	177298.674	0.061	320.7475
30	17	13	29	17	12	A	1	177298.674	0.061	320.7475
30	17	13	29	17	12	A	0	177383.714	-0.055	204.4334
30	17	14	29	17	13	A	0	177383.714	-0.055	204.4334
30	17	13	29	17	12	E	0	177385.004	0.127	204.4358
30	17	14	29	17	13	E	0	177385.004	0.127	204.4175
30	16	14	29	16	13	E	1	177460.600	0.019	314.7380
30	16	15	29	16	14	E	1	177475.325	0.008	314.1280
30	16	14	29	16	13	A	1	177509.671	0.066	314.2796
30	16	15	29	16	14	A	1	177509.671	0.066	314.2796
13	9	5	12	8	5	E	0	177522.592	0.045	89.6262
30	16	14	29	16	13	A	0	177591.529	-0.042	197.9263
30	16	15	29	16	14	A	0	177591.529	-0.042	197.9263
30	16	14	29	16	13	E	0	177593.037	0.168	197.9311
30	16	15	29	16	14	E	0	177593.037	0.168	197.9120
11	10	2	10	9	2	E	0	177698.779	0.258	88.4718
30	15	15	29	15	14	E	1	177704.254	-0.011	308.6224
30	15	16	29	15	15	E	1	177728.596	-0.012	307.9976
30	6	25	29	5	24	A	1	177756.632	0.314	271.8063
30	15	15	29	15	14	A	1	177765.232	0.101	308.2205
30	15	16	29	15	15	A	1	177765.232	0.101	308.2205
11	10	2	10	9	1	A	0	177768.579	0.294	88.4718
11	10	1	10	9	2	A	0	177768.579	0.294	88.4718
30	15	15	29	15	14	A	0	177843.515	0.032	191.8262
30	15	16	29	15	15	A	0	177843.515	0.032	191.8262
30	15	15	29	15	14	E	0	177845.243	0.244	191.8333
30	15	16	29	15	15	E	0	177845.243	0.244	191.8137
40	2	38	40	1	39	E	0	177951.156	-0.387	206.5906
40	3	38	40	1	39	E	0	177951.156	-0.387	206.5906
40	3	38	40	2	39	E	0	177951.156	-0.387	206.5906
40	2	38	40	1	39	A	0	177993.004	-0.325	206.5756
40	3	38	40	1	39	A	0	177993.004	-0.325	206.5756
40	3	38	40	2	39	A	0	177993.004	-0.325	206.5756
30	14	16	29	14	15	E	1	178004.329	0.020	302.9118
30	14	17	29	14	16	E	1	178041.498	-0.011	302.2816
30	14	17	29	14	16	A	1	178079.909	0.031	302.5730
30	14	16	29	14	15	A	1	178079.909	0.031	302.5730
30	6	25	29	5	24	E	0	178136.543	-0.133	155.0893
30	14	16	29	14	15	A	0	178154.039	-0.011	186.1363
30	14	17	29	14	16	A	0	178154.039	-0.011	186.1363
30	14	16	29	14	15	E	0	178155.570	0.320	186.1455
30	14	17	29	14	16	E	0	178156.567	0.176	186.1257
30	13	17	29	13	16	E	1	178381.286	-0.024	297.6108
30	13	18	29	13	17	E	1	178436.166	0.018	296.9848
24	9	15	24	7	17	E	1	178447.873	0.772	247.6694
42	3	39	42	2	40	A	1	178457.042	-0.048	343.7584
42	4	39	42	2	40	A	1	178457.042	-0.048	343.7584
42	3	39	42	3	40	A	1	178457.042	-0.048	343.7584
42	4	39	42	3	40	A	1	178457.042	-0.048	343.7584
30	13	18	29	13	17	A	1	178475.728	0.004	297.3408
30	13	17	29	13	16	A	1	178475.728	0.004	297.3408
29	8	21	28	8	20	E	1	178501.453	0.062	271.8459
30	13	17	29	13	16	A	0	178544.923	0.016	180.8610
30	13	18	29	13	17	A	0	178544.923	0.016	180.8610
30	13	17	29	13	16	E	0	178546.293	0.159	180.8723
30	13	18	29	13	17	E	0	178547.961	0.118	180.8526
40	16	24	40	15	26	E	0	178696.764	-0.461	268.2973
30	12	18	29	12	17	E	1	178867.436	0.011	292.7260
30	12	19	29	12	18	E	1	178947.433	0.010	292.1146
30	12	19	29	12	18	A	1	178986.367	-0.069	292.5303
30	12	18	29	12	17	A	1	178987.844	0.043	292.5304

J'	K'_a	K'_c	J''	K''_a	K''_c	Sym.	$v_{t_{CH_3}}$	$\nu_{Obs.}$	$\nu_{Obs.} - \nu_{Calc.}$	E_{low}
30	12	19	29	12	18	A	0	179049.446	0.015	176.0072
30	12	18	29	12	17	A	0	179051.142	0.454	176.0072
30	12	18	29	12	17	E	0	179051.142	-0.132	176.0203
30	12	19	29	12	18	E	0	179054.007	0.213	176.0011
29	6	23	28	6	22	A	1	179146.328	0.021	268.3554
14	4	11	13	2	12	E	1	179169.828	-0.042	195.8652
17	5	12	16	3	13	E	1	179202.166	0.385	207.6075
31	5	26	30	6	25	A	1	179308.776	-0.081	277.7356
30	7	24	29	7	23	A	1	179367.799	-0.024	274.8267
31	5	26	30	6	25	E	1	179392.503	0.150	277.3296
31	5	26	30	6	25	E	0	179424.603	0.039	161.0313
38	16	23	38	15	24	A	0	179427.970	-0.351	252.5510
29	6	23	28	6	22	E	0	179444.438	0.022	151.6231
29	6	23	28	6	22	E	1	179444.438	-0.304	267.9079
29	6	23	28	6	22	A	0	179455.337	-0.050	151.6088
42	3	39	42	2	40	E	0	179467.956	-0.219	227.0491
42	4	39	42	2	40	E	0	179467.956	-0.219	227.0491
42	4	39	42	3	40	E	0	179467.956	-0.219	227.0491
42	3	39	42	2	40	A	0	179506.007	-0.173	227.0357
42	4	39	42	2	40	A	0	179506.007	-0.173	227.0357
42	3	39	42	3	40	A	0	179506.007	-0.173	227.0357
42	4	39	42	3	40	A	0	179506.007	-0.173	227.0357
30	11	19	29	11	18	E	1	179516.965	0.061	288.2680
30	7	24	29	7	23	E	0	179534.092	0.053	158.1312
30	7	24	29	7	23	A	0	179537.538	0.055	158.1189
30	7	24	29	7	23	E	1	179632.848	0.005	274.4202
30	11	20	29	11	19	E	1	179635.193	0.068	287.6819
30	11	20	29	11	19	A	1	179663.419	-0.007	288.1514
30	11	19	29	11	18	A	1	179682.481	-0.027	288.1519
30	11	20	29	11	19	A	0	179718.698	0.071	171.5851
30	11	19	29	11	18	E	0	179729.026	0.021	171.6002
30	11	20	29	11	19	E	0	179731.997	0.047	171.5817
30	11	19	29	11	18	A	0	179736.359	0.057	171.5856
29	8	21	28	8	20	A	0	179768.259	0.069	155.4053
29	8	21	28	8	20	E	0	179776.411	0.102	155.4174
29	8	21	28	8	20	A	1	179829.794	-0.111	272.1042
31	9	23	31	7	25	E	1	180011.044	0.718	286.5806
18	7	12	17	6	11	A	1	180152.562	0.178	215.8423
32	4	28	31	5	27	A	1	180193.242	-0.185	280.1925
32	5	28	31	5	27	A	1	180239.554	-0.098	280.1925
31	6	26	30	6	25	A	1	180267.808	0.012	277.7356
33	3	30	32	4	29	A	1	180269.323	-0.193	282.1512
33	4	30	32	4	29	A	1	180270.805	-0.062	282.1512
33	3	30	32	3	29	A	1	180272.026	-0.015	282.1512
33	4	30	32	3	29	A	1	180273.401	0.010	282.1512
32	4	28	31	4	27	A	1	180274.920	-0.010	280.1898
32	5	28	31	4	27	A	1	180320.884	-0.270	280.1898
22	9	14	22	7	16	E	1	180356.142	0.621	237.7068
32	4	28	31	5	27	E	1	180359.196	0.043	279.7786
32	4	28	31	5	27	E	0	180367.342	-0.100	163.4795
32	4	28	31	5	27	A	0	180372.453	0.001	163.4666
33	3	30	32	4	29	E	1	180402.625	-0.120	281.7204
36	16	21	36	15	22	E	0	180404.213	-0.104	237.6105
33	4	30	32	4	29	E	1	180404.219	-0.143	281.7204
33	3	30	32	3	29	E	1	180405.731	-0.009	281.7203
34	2	32	33	2	31	A	1	180408.336	-0.101	283.6505
34	3	32	33	2	31	A	1	180408.336	-0.101	283.6505
34	2	32	33	3	31	A	1	180408.336	-0.101	283.6505
34	3	32	33	3	31	A	1	180408.336	-0.101	283.6505
33	7	26	32	8	25	E	0	180412.793	-0.114	179.7612
32	5	28	31	5	27	E	1	180412.800	-0.124	279.7786
32	5	28	31	5	27	E	0	180418.492	-0.080	163.4795
33	4	30	32	4	29	E	0	180421.243	-0.105	165.4240
33	3	30	32	3	29	E	0	180422.602	-0.054	165.4239
33	3	30	32	4	29	A	0	180423.955	0.054	165.4105
32	5	28	31	5	27	A	0	180423.955	0.054	163.4666
33	4	30	32	3	29	E	0	180423.955	-0.221	165.4239
33	4	30	32	4	29	A	0	180425.408	-0.042	165.4105
33	3	30	32	3	29	A	0	180426.802	0.036	165.4104
33	4	30	32	3	29	A	0	180428.455	0.158	165.4104
30	10	20	29	10	19	E	1	180435.358	0.055	284.2542
32	4	28	31	4	27	E	1	180453.043	-0.131	279.7755
32	4	28	31	4	27	E	0	180457.144	-0.043	163.4766
32	4	28	31	4	27	A	0	180462.624	-0.076	163.4636
31	6	26	30	6	25	E	0	180466.100	-0.098	161.0313
31	6	26	30	6	25	A	0	180472.101	-0.055	161.0186
31	6	26	30	6	25	E	1	180474.085	-0.194	277.3296
34	3	32	33	3	31	E	1	180503.908	-0.072	283.1936
34	2	32	33	3	31	E	1	180503.908	-0.072	283.1936
34	3	32	33	2	31	E	1	180503.908	-0.072	283.1936
34	2	32	33	2	31	E	1	180503.908	-0.072	283.1936
32	5	28	31	4	27	E	1	180506.754	-0.192	279.7755
32	5	28	31	4	27	E	0	180508.325	0.009	163.4766
32	5	28	31	4	27	A	0	180514.076	-0.055	163.4636
34	2	32	33	2	31	E	0	180530.882	-0.006	166.9030
34	3	32	33	2	31	E	0	180530.882	-0.006	166.9030
34	2	32	33	3	31	E	0	180530.882	-0.006	166.9030
34	3	32	33	3	31	E	0	180530.882	-0.006	166.9030
34	2	32	33	2	31	A	0	180533.812	-0.005	166.8887
34	3	32	33	2	31	A	0	180533.812	-0.005	166.8887
34	2	32	33	3	31	A	0	180533.812	-0.005	166.8887
34	3	32	33	3	31	A	0	180533.812	-0.005	166.8887
30	10	21	29	10	20	A	1	180548.244	-0.101	284.2188
30	10	21	29	10	20	A	0	180596.383	0.092	167.6102
35	1	34	34	1	33	A	1	180598.447	-0.069	284.7211
35	2	34	34	1	33	A	1	180598.447	-0.069	284.7211
35	1	34	34	2	33	A	1	180598.447	-0.069	284.7211
35	2	34	34	2	33	A	1	180598.447	-0.069	284.7211
30	10	21	29	10	20	E	1	180616.046	0.149	283.7058
30	10	21	29	10	20	E	0	180649.085	0.038	167.6121
35	2	34	34	2	33	E	1	180655.630	-0.057	284.2295
35	1	34	34	2	33	E	1	180655.630	-0.057	284.2295
35	2	34	34	1	33	E	1	180655.630	-0.057	284.2295
35	1	34	34	1	33	E	1	180655.630	-0.057	284.2295
44	4	40	44	3	41	E	0	180669.786	0.017	248.5397
44	5	40	44	3	41	E	0	180669.786	0.017	248.5397
44	5	40	44	4	41	E	0	180669.786	0.017	248.5397
35	1	34	34	1	33	E	0	180692.257	-0.022	167.9474
35	2	34	34	1	33	E	0	180692.257	-0.022	167.9474
35	1	34	34	2	33	E	0	180692.257	-0.022	167.9474
35	2	34	34	2	33	E	0	180692.257	-0.022	167.9474
35	1	34	34	1	33	A	0	180694.068	0.041	167.9320
35	2	34	34	1	33	A	0	180694.068	0.041	167.9320
35	1	34	34	2	33	A	0	180694.068	0.041	167.9320
35	2	34	34	2	33	A	0	180694.068	0.041	167.9320
44	4	40	44	3	41	A	0	180703.935	0.155	248.5278

J'	K_a'	K_c'	J''	K_a''	K_c''	Sym.	$v_{t_{CH_3}}$	$\nu_{Obs.}$	$\nu_{Obs.} - \nu_{Calc.}$	E_{low}
44	5	40	44	3	41	A	0	180703.935	0.155	248.5278
44	5	40	44	4	41	A	0	180703.935	0.155	248.5278
30	10	20	29	10	19	E	0	180739.643	0.086	167.6308
30	10	20	29	10	19	A	1	180749.884	-0.020	284.2263
30	10	20	29	10	19	A	0	180784.675	0.070	167.6172
36	0	36	35	0	35	A	1	180811.480	-0.042	285.3828
36	1	36	35	0	35	A	1	180811.480	-0.042	285.3828
36	0	36	35	1	35	A	1	180811.480	-0.042	285.3828
36	1	36	35	1	35	A	1	180811.480	-0.042	285.3828
36	1	36	35	1	35	E	1	180827.727	-0.081	284.8479
36	0	36	35	1	35	E	1	180827.727	-0.081	284.8479
36	1	36	35	0	35	E	1	180827.727	-0.081	284.8479
36	0	36	35	0	35	E	1	180827.727	-0.081	284.8479
44	5	40	44	4	41	E	1	180833.714	-0.333	364.8252
44	4	40	44	4	41	E	1	180833.714	-0.333	364.8252
44	5	40	44	3	41	E	1	180833.714	-0.333	364.8252
44	4	40	44	3	41	E	1	180833.714	-0.333	364.8252
31	5	26	30	5	25	A	1	180868.312	-0.050	277.6836
36	0	36	35	0	35	A	0	180874.916	-0.246	168.5602
36	1	36	35	0	35	A	0	180874.916	-0.246	168.5602
36	0	36	35	1	35	A	0	180874.916	-0.246	168.5602
36	1	36	35	1	35	A	0	180874.916	-0.246	168.5602
36	0	36	35	0	35	E	0	180874.916	0.250	168.5769
36	1	36	35	0	35	E	0	180874.916	0.250	168.5769
36	0	36	35	1	35	E	0	180874.916	0.250	168.5769
36	1	36	35	1	35	E	0	180874.916	0.250	168.5769
31	5	26	30	5	25	E	0	181110.333	-0.081	160.9751
31	5	26	30	5	25	A	0	181118.949	-0.059	160.9621
31	5	26	30	5	25	E	1	181135.432	-0.148	277.2714
30	9	22	29	9	21	A	1	181450.226	-0.013	280.7436
30	8	23	29	8	22	A	1	181458.938	-0.065	277.6784
30	9	22	29	9	21	A	0	181506.187	0.046	164.0947
30	9	22	29	9	21	E	0	181521.001	0.066	164.1035
46	5	41	46	4	42	E	0	181532.054	0.303	271.0534
46	6	41	46	4	42	E	0	181532.054	0.303	271.0534
46	6	41	46	5	42	E	0	181532.054	0.303	271.0534
30	8	23	29	8	22	A	0	181562.294	-0.079	160.9949
30	8	23	29	8	22	E	0	181564.102	0.130	161.0061
30	9	21	29	9	20	E	1	181969.572	0.029	280.7194
30	9	22	29	9	21	E	1	182064.238	0.154	280.2217
31	29	2	30	29	1	A	0	182213.919	-0.416	319.6778
31	29	3	30	29	2	A	0	182213.919	-0.416	319.6778
31	28	3	30	28	2	A	0	182262.886	-0.264	308.3735
31	28	4	30	28	3	A	0	182262.886	-0.264	308.3735
31	28	4	30	28	3	E	0	182262.886	-0.395	308.3551
30	8	23	29	8	22	E	1	182287.648	0.134	277.2346
31	27	4	30	27	3	A	0	182316.436	-0.160	297.4667
31	27	5	30	27	4	A	0	182316.436	-0.160	297.4667
31	27	5	30	27	4	E	0	182316.436	-0.367	297.4494
31	25	7	30	25	6	A	1	182335.805	-0.190	392.9685
31	25	6	30	25	5	A	1	182335.805	-0.190	392.9685
31	25	6	30	25	5	E	1	182335.805	0.179	393.5758
31	26	5	30	26	4	A	0	182375.325	-0.116	286.9578
31	26	6	30	26	5	A	0	182375.325	-0.116	286.9578
31	26	6	30	26	5	E	0	182375.325	-0.040	286.9395
31	24	8	30	24	7	E	1	182395.174	-0.127	383.6700
31	24	7	30	24	6	E	1	182406.812	-0.073	383.8894
31	24	8	30	24	7	A	1	182410.129	-0.015	383.2643
31	24	7	30	24	6	A	1	182410.129	-0.015	383.2643
31	25	6	30	25	5	A	0	182440.600	-0.011	276.8470
31	25	7	30	25	6	A	0	182440.600	-0.011	276.8470
31	25	7	30	25	6	E	0	182440.600	-0.009	276.8279
31	23	9	30	23	8	A	1	182493.532	0.221	373.9640
31	23	8	30	23	7	A	1	182493.532	0.221	373.9640
31	24	7	30	24	6	A	0	182513.200	-0.035	267.1347
31	24	8	30	24	7	A	0	182513.200	-0.035	267.1347
31	24	8	30	24	7	E	0	182513.200	-0.123	267.1150
31	22	10	30	22	9	E	1	182566.703	-0.017	365.3494
31	22	9	30	22	8	E	1	182576.523	0.063	365.7025
29	7	22	28	7	21	E	1	182593.276	-0.360	269.6574
31	23	8	30	23	7	A	0	182594.850	0.154	257.8212
31	23	9	30	23	8	A	0	182594.850	0.154	257.8212
31	23	9	30	23	8	E	0	182594.850	-0.042	257.8012
31	21	11	30	21	10	E	1	182670.557	-0.064	356.7894
31	21	10	30	21	9	E	1	182678.539	0.026	357.2022
31	22	10	30	22	9	E	0	182686.873	-0.160	248.8870
31	21	11	30	21	10	A	1	182694.107	0.085	356.5758
31	21	10	30	21	9	A	1	182694.107	0.085	356.5758
31	20	12	30	20	11	E	1	182789.962	-0.173	348.6310
31	21	10	30	21	9	A	0	182791.735	0.308	240.3925
31	21	11	30	21	10	A	0	182791.735	0.308	240.3925
31	21	11	30	21	10	E	0	182791.735	-0.161	240.3729
31	20	11	30	20	10	E	1	182795.415	0.009	349.0978
31	20	11	30	20	10	A	1	182816.473	0.008	348.4887
31	20	12	30	20	11	A	1	182816.473	0.008	348.4887
29	7	22	28	7	21	A	1	182876.781	-0.081	270.0763
30	9	21	29	9	20	E	0	182898.236	0.011	164.1718
30	9	21	29	9	20	A	0	182902.300	0.057	164.1611
31	20	11	30	20	10	A	0	182911.998	0.430	232.2787
31	20	12	30	20	11	A	0	182911.998	0.430	232.2787
31	20	12	30	20	11	E	0	182911.998	-0.211	232.2596
30	9	21	29	9	20	A	1	182923.783	-0.030	280.8140
31	19	13	30	19	12	E	1	182928.819	0.012	340.8759
31	19	12	30	19	11	E	1	182930.632	0.114	341.3900
31	19	13	30	19	12	A	1	182958.151	0.090	340.8067
31	19	12	30	19	11	A	1	182958.151	0.090	340.8067
29	7	22	28	7	21	A	0	182972.630	-0.037	153.3380
29	7	22	28	7	21	E	0	182974.272	0.094	153.3520
16	8	8	15	7	8	E	0	183007.360	-0.158	94.9888
16	8	9	15	7	8	A	0	183022.791	-0.201	94.9699
31	19	12	30	19	11	A	0	183050.933	0.302	224.5662
31	19	13	30	19	12	A	0	183050.933	0.302	224.5662
31	18	13	30	18	12	E	1	183088.363	0.107	334.0797
31	18	14	30	18	13	E	1	183091.258	-0.006	333.5257
31	18	13	30	18	12	A	1	183123.428	0.025	333.5307
31	18	14	30	18	13	A	1	183123.428	0.025	333.5307
31	18	13	30	18	12	A	0	183213.185	0.008	217.2562
31	18	14	30	18	13	A	0	183213.185	0.008	217.2562
31	18	13	30	18	12	E	0	183214.257	0.030	217.2564
31	18	14	30	18	13	E	0	183214.257	0.030	217.2391
27	16	11	27	15	12	E	0	183237.939	-0.284	180.5744
31	17	14	30	17	13	E	1	183274.665	0.206	327.1682
31	17	15	30	17	14	E	1	183283.686	0.031	326.5822
31	17	15	30	17	14	A	1	183318.582	-0.010	326.6615
31	17	14	30	17	13	A	1	183318.582	-0.010	326.6615
31	17	14	30	17	13	A	0	183405.216	-0.046	210.3502

J'	K'_a	K'_c	J''	K''_a	K''_c	Sym.	$v_{t_{CH_3}}$	$\nu_{Obs.}$	$\nu_{Obs.} - \nu_{Calc.}$	E_{low}
31	17	15	30	17	14	A	0	183405.216	-0.046	210.3502
31	17	14	30	17	13	E	0	183406.635	0.140	210.3528
31	17	15	30	17	14	E	0	183406.635	0.140	210.3344
14	9	5	13	8	5	E	0	183412.388	-0.066	92.2037
14	9	6	13	8	5	A	0	183443.817	0.115	92.1853
14	9	5	13	8	6	A	0	183443.817	0.115	92.1853
30	6	24	30	5	26	E	0	183451.836	0.241	157.6261
31	16	15	30	16	14	E	1	183497.088	0.058	320.6574
31	16	16	30	16	15	E	1	183514.368	0.038	320.0480
31	16	15	30	16	14	A	1	183551.955	0.045	320.2007
31	16	16	30	16	15	A	1	183551.955	0.045	320.2007
12	10	3	11	9	3	E	0	183573.609	0.286	90.6320
31	16	15	30	16	14	A	0	183635.131	0.028	203.8501
31	16	16	30	16	15	A	0	183635.131	0.028	203.8501
31	16	15	30	16	14	E	0	183636.711	0.168	203.8549
31	16	16	30	16	15	E	0	183636.711	0.168	203.8358
12	10	3	11	9	2	A	0	183643.377	0.272	90.6320
12	10	2	11	9	3	A	0	183643.377	0.272	90.6320
30	6	24	29	6	23	A	1	183645.866	-0.094	274.3310
31	15	16	30	15	15	E	1	183767.019	0.078	314.5499
31	15	17	30	15	16	E	1	183794.959	0.026	313.9260
31	15	17	30	15	16	A	1	183834.918	0.018	314.1501
31	15	16	30	15	15	A	1	183834.918	0.018	314.1501
31	15	16	30	15	15	A	0	183914.151	0.014	197.7584
31	15	17	30	15	16	A	0	183914.151	0.014	197.7584
31	15	16	30	15	15	E	0	183916.016	0.200	197.7655
31	15	17	30	15	16	E	0	183916.016	0.200	197.7460
30	6	24	29	6	23	E	0	183972.458	-0.065	157.6087
30	6	24	29	6	23	A	0	183985.222	-0.047	157.5948
30	6	24	29	6	23	E	1	184009.277	-0.628	273.8935
31	14	17	30	14	16	E	1	184100.003	0.086	308.8493
31	14	18	30	14	17	E	1	184142.216	-0.152	308.2204
13	11	3	12	10	2	E	1	184142.216	-0.152	213.4689
31	14	18	30	14	17	A	1	184184.229	0.047	308.5131
31	14	17	30	14	16	A	1	184184.229	0.047	308.5131
31	14	17	30	14	16	A	0	184258.850	0.031	192.0789
31	14	18	30	14	17	A	0	184258.850	0.031	192.0789
31	14	17	30	14	16	E	0	184260.384	0.256	192.0881
31	14	18	30	14	17	E	0	184261.607	0.177	192.0684
43	3	40	43	2	41	E	0	184271.855	-0.141	234.5584
43	4	40	43	2	41	E	0	184271.855	-0.141	234.5584
43	4	40	43	3	41	E	0	184271.855	-0.141	234.5584
43	3	40	43	2	41	A	0	184310.805	-0.234	234.5450
43	4	40	43	2	41	A	0	184310.805	-0.234	234.5450
43	3	40	43	3	41	A	0	184310.805	-0.234	234.5450
43	4	40	43	3	41	A	0	184310.805	-0.234	234.5450
17	4	13	16	3	14	A	0	184357.317	-0.306	89.8037
43	4	40	43	3	41	E	1	184498.521	-0.363	350.8375
43	3	40	43	3	41	E	1	184498.521	-0.363	350.8375
43	4	40	43	2	41	E	1	184498.521	-0.363	350.8375
43	3	40	43	2	41	E	1	184498.521	-0.363	350.8375
31	13	18	30	13	17	E	1	184519.535	0.087	303.5609
34	18	17	33	18	15	E	1	184548.914	-0.666	353.0026
31	13	19	30	13	18	E	1	184581.517	-0.027	302.9368
31	13	19	30	13	18	A	1	184624.795	0.041	303.2941
31	13	18	30	13	17	A	1	184624.795	0.041	303.2941
32	5	27	31	6	26	A	1	184655.159	-0.001	283.7487
31	7	25	30	7	24	A	1	184689.210	-0.012	280.8098
31	13	18	30	13	17	A	0	184693.909	0.054	186.8166
31	13	19	30	13	18	A	0	184693.909	0.054	186.8166
31	13	18	30	13	17	E	0	184695.401	0.210	186.8280
31	13	19	30	13	18	E	0	184697.301	0.171	186.8083
32	6	26	31	7	25	E	1	184766.413	0.575	286.5806
32	5	27	31	6	26	E	1	184782.706	0.009	283.3495
32	5	27	31	6	26	E	0	184805.409	0.385	167.0510
32	5	27	31	6	26	A	0	184807.987	0.050	167.0385
31	7	25	30	7	24	E	0	184872.039	0.016	164.1198
32	6	26	31	7	25	A	0	184874.346	0.135	170.2745
31	7	25	30	7	24	A	0	184876.526	0.115	164.1077
19	7	13	18	6	12	A	1	184879.308	0.202	219.4781
32	6	26	31	7	25	E	0	184890.914	0.161	170.2865
31	7	25	30	7	24	E	1	184928.356	-0.019	280.4121
48	17	31	48	16	32	E	0	185062.775	0.231	345.4582
31	12	19	30	12	18	E	1	185062.775	0.059	298.6924
33	4	29	32	5	28	A	1	185147.760	-0.263	286.2047
33	4	29	32	4	28	A	1	185194.253	0.006	286.2031
31	12	19	30	12	18	A	1	185198.108	0.088	298.5008
34	3	31	33	3	30	A	1	185218.046	-0.088	288.1644
34	4	31	33	4	30	A	1	185218.046	-0.088	288.1644
33	5	29	32	4	28	A	1	185220.429	0.202	286.2031
32	6	27	31	6	26	A	1	185235.362	-0.101	283.7487
31	12	20	30	12	19	A	0	185257.405	0.110	181.9796
31	12	19	30	12	18	A	0	185260.059	0.079	181.9797
31	12	19	30	12	18	E	0	185260.059	0.092	181.9928
31	12	20	30	12	19	E	0	185262.891	0.083	181.9737
33	4	29	32	5	28	E	1	185315.196	-0.156	285.7966
33	4	29	32	5	28	E	0	185324.739	-0.091	169.4977
33	4	29	32	5	28	A	0	185329.837	-0.093	169.4849
33	5	29	32	5	28	E	1	185345.798	-0.029	285.7966
34	4	31	33	4	30	E	1	185350.738	-0.008	287.7380
34	3	31	33	3	30	E	1	185350.738	-0.008	287.7380
33	5	29	32	5	28	E	0	185353.713	0.015	169.4977
33	5	29	32	5	28	A	0	185359.006	0.030	169.4849
35	2	33	34	2	32	A	1	185361.408	-0.011	289.6683
35	3	33	34	2	32	A	1	185361.408	-0.011	289.6683
35	2	33	34	3	32	A	1	185361.408	-0.011	289.6683
35	3	33	34	3	32	A	1	185361.408	-0.011	289.6683
34	3	31	33	3	30	E	0	185369.350	-0.244	171.4422
34	4	31	33	4	30	E	0	185369.350	-0.244	171.4422
33	4	29	32	4	28	E	1	185369.350	0.226	285.7948
34	3	31	33	3	30	A	0	185373.677	0.008	171.4288
34	4	31	33	4	30	A	0	185373.677	0.008	171.4289
33	4	29	32	4	28	E	0	185376.067	0.107	169.4960
20	5	16	19	3	16	E	0	185381.357	0.451	102.1034
33	5	29	32	4	28	E	1	185399.491	-0.107	285.7948
33	5	29	32	4	28	E	0	185404.544	-0.283	169.4960
33	5	29	32	4	28	A	0	185410.481	0.075	169.4832
32	6	27	31	6	26	E	0	185438.332	0.008	167.0510
32	6	27	31	6	26	E	1	185443.065	-0.243	283.3495
32	6	27	31	6	26	A	0	185444.545	0.064	167.0385
35	3	33	34	3	32	E	1	185456.485	-0.043	289.2146
35	2	33	34	3	32	E	1	185456.485	-0.043	289.2146
35	3	33	34	2	32	E	1	185456.485	-0.043	289.2146
35	2	33	34	2	32	E	1	185456.485	-0.043	289.2146
35	2	33	34	2	32	E	0	185485.033	-0.051	172.9248
35	3	33	34	2	32	E	0	185485.033	-0.051	172.9248

J'	K'_a	K'_c	J''	K''_a	K''_c	Sym.	$v_{t_{CH_3}}$	$\nu_{Obs.}$	$\nu_{Obs.} - \nu_{Calc.}$	E_{low}
35	2	33	34	3	32	E	0	185485.033	-0.051	172.9248
35	3	33	34	3	32	E	0	185485.033	-0.051	172.9248
35	2	33	34	2	32	A	0	185487.944	-0.057	172.9106
35	3	33	34	2	32	A	0	185487.944	-0.057	172.9106
35	2	33	34	3	32	A	0	185487.944	-0.057	172.9106
35	3	33	34	3	32	A	0	185487.944	-0.057	172.9106
45	4	41	45	3	42	E	0	185504.068	0.033	256.5395
45	5	41	45	3	42	E	0	185504.068	0.033	256.5395
45	5	41	45	4	42	E	0	185504.068	0.033	256.5395
45	4	41	45	3	42	A	0	185538.747	-0.260	256.5277
45	5	41	45	3	42	A	0	185538.747	-0.260	256.5277
45	5	41	45	4	42	A	0	185538.747	-0.260	256.5277
36	1	35	35	1	34	A	1	185553.355	-0.046	290.7452
36	2	35	35	1	34	A	1	185553.355	-0.046	290.7452
36	1	35	35	2	34	A	1	185553.355	-0.046	290.7452
36	2	35	35	2	34	A	1	185553.355	-0.046	290.7452
36	2	35	35	2	34	E	1	185610.319	-0.057	290.2555
36	1	35	35	2	34	E	1	185610.319	-0.057	290.2555
36	2	35	35	1	34	E	1	185610.319	-0.057	290.2555
36	1	35	35	1	34	E	1	185610.319	-0.057	290.2555
32	5	27	31	5	26	A	1	185614.053	-0.047	283.7167
36	1	35	35	1	34	E	0	185648.437	-0.051	173.9746
36	2	35	35	1	34	E	0	185648.437	-0.051	173.9746
36	1	35	35	2	34	E	0	185648.437	-0.051	173.9746
36	2	35	35	2	34	E	0	185648.437	-0.051	173.9746
36	1	35	35	1	34	A	0	185650.257	0.028	173.9593
36	2	35	35	1	34	A	0	185650.257	0.028	173.9593
36	1	35	35	2	34	A	0	185650.257	0.028	173.9593
36	2	35	35	2	34	A	0	185650.257	0.028	173.9593
45	5	41	45	4	42	E	1	185673.541	-0.331	372.8237
45	4	41	45	4	42	E	1	185673.541	-0.331	372.8237
45	5	41	45	3	42	E	1	185673.541	-0.331	372.8237
45	4	41	45	3	42	E	1	185673.541	-0.331	372.8237
40	1	39	40	0	40	E	0	185683.594	-0.203	200.3969
40	2	39	40	1	40	E	0	185683.594	-0.203	200.3969
37	0	37	36	0	36	A	1	185766.898	-0.008	291.4140
37	1	37	36	0	36	A	1	185766.898	-0.008	291.4140
37	0	37	36	1	36	A	1	185766.898	-0.008	291.4140
37	1	37	36	1	36	A	1	185766.898	-0.008	291.4140
37	1	37	36	1	36	E	1	185782.940	-0.117	290.8796
37	0	37	36	1	36	E	1	185782.940	-0.117	290.8796
37	1	37	36	0	36	E	1	185782.940	-0.117	290.8796
37	0	37	36	0	36	E	1	185782.940	-0.117	290.8796
31	11	20	30	11	19	E	1	185793.960	0.002	294.2561
37	0	37	36	0	36	A	0	185831.566	-0.292	174.5936
37	1	37	36	0	36	A	0	185831.566	-0.292	174.5936
37	0	37	36	1	36	A	0	185831.566	-0.292	174.5936
37	1	37	36	1	36	A	0	185831.566	-0.292	174.5936
37	0	37	36	0	36	E	0	185831.566	0.199	174.6102
37	1	37	36	0	36	E	0	185831.566	0.199	174.6102
37	0	37	36	1	36	E	0	185831.566	0.199	174.6102
37	1	37	36	1	36	E	0	185831.566	0.199	174.6102
32	5	27	31	5	26	E	0	185846.685	0.026	167.0163
32	5	27	31	5	26	A	0	185854.658	0.041	167.0036
32	5	27	31	5	26	E	1	185864.894	0.272	283.3135
31	11	21	30	11	20	E	1	185928.534	0.103	293.6739
31	11	21	30	11	20	A	1	185951.040	0.007	294.1443
31	11	20	30	11	19	A	1	185988.258	-0.032	294.1455
31	11	21	30	11	20	A	0	186004.788	0.070	177.5798
31	11	20	30	11	19	E	0	186024.942	0.087	177.5953
31	11	21	30	11	20	E	0	186025.909	0.038	177.5769
31	11	20	30	11	19	A	0	186039.325	0.062	177.5810
30	8	22	29	8	21	E	1	186103.521	0.029	277.8001
32	6	27	31	5	26	A	1	186194.430	0.028	283.7167
12	6	7	11	4	8	E	1	186226.182	0.080	194.0392
47	5	42	47	4	43	E	0	186408.776	0.286	279.5446
47	6	42	47	4	43	E	0	186408.776	0.286	279.5446
47	6	42	47	5	43	E	0	186408.776	0.286	279.5446
47	5	42	47	4	43	A	0	186439.521	0.338	279.5343
47	6	42	47	4	43	A	0	186439.521	0.338	279.5343
47	5	42	47	5	43	A	0	186439.521	0.338	279.5343
47	6	42	47	5	43	A	0	186439.521	0.338	279.5343
32	6	27	31	5	26	E	0	186480.057	0.099	167.0163
32	6	27	31	5	26	A	0	186491.029	-0.133	167.0036
47	6	42	47	5	43	E	1	186518.503	-0.126	395.8321
47	5	42	47	5	43	E	1	186518.503	-0.126	395.8321
47	6	42	47	4	43	E	1	186518.503	-0.126	395.8321
47	5	42	47	4	43	E	1	186518.503	-0.126	395.8321
32	6	27	31	5	26	E	1	186525.146	-0.088	283.3135
31	10	21	30	10	20	E	1	186846.658	0.042	290.2728
31	10	22	30	10	21	A	1	186910.074	-0.114	290.2413
19	6	13	18	5	14	E	1	186927.904	-0.813	216.7965
14	3	12	13	1	13	A	0	186957.981	0.198	77.6624
31	10	22	30	10	21	A	0	186957.981	0.198	173.6343
49	7	43	49	5	44	A	0	186983.905	0.247	303.5560
49	7	43	49	6	44	A	0	186983.905	0.247	303.5560
14	3	12	13	1	13	E	1	187004.504	0.204	193.9726
31	10	22	30	10	21	E	0	187008.229	0.074	173.6379
31	10	22	30	10	21	E	1	187049.431	0.105	289.7304
30	8	22	29	8	21	A	0	187198.378	0.100	161.4017
30	8	22	29	8	21	E	0	187207.349	0.083	161.4141
31	10	21	30	10	20	E	0	187250.163	0.031	173.6596
30	8	22	29	8	21	A	1	187251.896	-0.107	278.1027
31	10	21	30	10	20	A	1	187266.646	0.064	290.2555
31	10	21	30	10	20	A	0	187291.634	0.088	173.6476
31	8	24	30	8	23	A	1	187342.813	-0.017	283.7312
31	8	24	30	8	23	A	0	187465.971	0.144	167.0512
31	8	24	30	8	23	E	0	187465.971	-0.038	167.0624
42	2	40	42	1	41	E	0	187517.157	-0.412	220.7942
42	3	40	42	1	41	E	0	187517.157	-0.412	220.7942
42	3	40	42	2	41	E	0	187517.157	-0.412	220.7942
42	2	40	42	1	41	A	0	187561.238	-0.373	220.7793
42	3	40	42	1	41	A	0	187561.238	-0.373	220.7793
42	3	40	42	2	41	A	0	187561.238	-0.373	220.7793
17	8	10	16	7	9	A	1	187687.082	0.290	214.8863
31	9	23	30	9	22	A	1	187741.459	-0.079	286.7961
31	9	23	30	9	22	A	0	187805.878	0.044	170.1491
31	9	23	30	9	22	E	0	187815.204	0.052	170.1583
31	8	24	30	8	23	E	1	187963.378	0.127	283.3151
32	30	2	31	30	1	E	1	187966.260	-0.457	454.2365
32	30	2	31	30	1	A	1	187967.889	-0.196	453.6112
32	30	3	31	30	2	A	1	187967.889	-0.196	453.6112
32	30	3	31	30	2	E	1	187975.388	-0.087	454.0149
31	6	25	30	6	24	A	1	187995.829	0.003	280.4568
44	3	41	44	2	42	A	1	188008.169	-0.081	358.9332
44	4	41	44	2	42	A	1	188008.169	-0.081	358.9332

J'	K'_a	K'_c	J''	K''_a	K''_c	Sym.	$v_{t_{CH_3}}$	$\nu_{Obs.}$	$\nu_{Obs.} - \nu_{Calc.}$	E_{low}
44	3	41	44	3	42	A	1	188008.169	-0.081	358.9332
44	4	41	44	3	42	A	1	188008.169	-0.081	358.9332
32	29	4	31	29	3	E	1	188024.069	-0.132	442.3511
32	28	5	31	28	4	A	1	188071.804	-0.158	430.5777
32	28	4	31	28	3	A	1	188071.804	-0.158	430.5777
32	30	2	31	30	1	A	0	188080.821	-0.368	337.4561
32	30	3	31	30	2	A	0	188080.821	-0.368	337.4561
32	30	3	31	30	2	E	0	188080.821	-0.392	337.4364
32	29	3	31	29	2	A	0	188129.721	-0.346	325.7558
32	29	4	31	29	3	A	0	188129.721	-0.346	325.7558
32	29	4	31	29	3	E	0	188129.721	-0.440	325.7367
32	27	6	31	27	5	A	1	188131.480	0.003	419.6653
32	27	5	31	27	4	A	1	188131.480	0.003	419.6653
32	27	6	31	27	5	E	1	188135.033	0.104	420.2126
15	9	7	14	8	6	A	1	188154.297	0.285	211.6553
15	9	6	14	8	7	A	1	188154.297	0.285	211.6553
32	28	4	31	28	3	A	0	188183.081	-0.272	314.4531
32	28	5	31	28	4	A	0	188183.081	-0.272	314.4531
32	28	5	31	28	4	E	0	188183.081	-0.443	314.4347
32	26	7	31	26	6	E	1	188185.847	-0.126	409.6635
32	26	6	31	26	5	A	1	188196.992	-0.135	409.1561
32	26	7	31	26	6	A	1	188196.992	-0.135	409.1561
32	26	6	31	26	5	E	1	188198.671	0.252	409.7374
32	27	5	31	27	4	A	0	188241.644	-0.107	303.5482
32	27	6	31	27	5	A	0	188241.644	-0.107	303.5482
32	27	6	31	27	5	E	0	188241.644	-0.361	303.5308
32	25	8	31	25	7	E	1	188255.844	-0.153	399.5100
32	25	7	31	25	6	E	1	188268.380	-0.198	399.6578
32	25	8	31	25	7	A	1	188270.072	0.132	399.0506
32	25	7	31	25	6	A	1	188270.072	0.132	399.0506
32	26	6	31	26	5	A	0	188306.002	-0.109	293.0412
32	26	7	31	26	6	A	0	188306.002	-0.109	293.0412
32	26	7	31	26	6	E	0	188306.002	-0.055	293.0229
31	6	25	30	6	24	E	0	188332.168	-0.053	163.7454
32	24	9	31	24	8	E	1	188334.392	0.065	389.7541
31	6	25	30	6	24	A	0	188345.569	-0.073	163.7319
32	24	8	31	24	7	E	1	188346.797	0.189	389.9739
32	24	8	31	24	7	A	1	188351.186	0.024	389.3489
32	24	9	31	24	8	A	1	188351.186	0.024	389.3489
19	7	12	18	6	13	A	0	188359.656	-0.301	102.6393
32	25	7	31	25	6	A	0	188377.461	0.011	282.9326
32	25	8	31	25	7	A	0	188377.461	0.011	282.9326
32	25	8	31	25	7	E	0	188377.461	-0.018	282.9135
31	6	25	30	6	24	E	1	188388.372	-0.227	280.0314
19	7	12	18	6	12	E	1	188412.582	0.310	219.1982
32	23	10	31	23	9	E	1	188422.217	-0.297	380.3968
32	23	9	31	23	8	E	1	188434.239	0.252	380.6854
32	23	10	31	23	9	A	1	188442.335	0.006	380.0513
32	23	9	31	23	8	A	1	188442.335	0.006	380.0513
32	24	8	31	24	7	A	0	188457.142	0.128	273.2227
32	24	9	31	24	8	A	0	188457.142	0.128	273.2227
32	24	9	31	24	8	E	0	188457.142	-0.001	273.2030
32	22	11	31	22	10	E	1	188522.367	-0.113	371.4392
32	22	10	31	22	9	E	1	188532.561	0.014	371.7926
32	23	9	31	23	8	A	0	188546.508	0.176	263.9119
32	23	10	31	23	9	A	0	188546.508	0.176	263.9119
32	23	10	31	23	9	E	0	188546.508	-0.070	263.8919
31	9	23	30	9	22	E	1	188582.937	0.243	286.2947
32	21	12	31	21	11	E	1	188636.614	-0.019	362.8826
32	21	11	31	21	10	E	1	188644.623	0.035	363.2957
32	22	11	31	22	10	E	0	188647.473	-0.208	254.9808
32	21	12	31	21	11	A	1	188662.628	0.032	362.6698
32	21	11	31	21	10	A	1	188662.628	0.032	362.6698
17	8	10	16	7	10	E	0	188689.113	-0.163	98.1513
17	8	9	16	7	9	E	0	188734.672	-0.230	98.1656
32	21	11	31	21	10	A	0	188762.654	0.369	246.4898
32	21	12	31	21	11	A	0	188762.654	0.369	246.4898
32	21	12	31	21	11	E	0	188762.654	-0.177	246.4702
32	20	13	31	20	12	E	1	188768.010	-0.018	354.7283
32	20	12	31	20	11	E	1	188773.130	0.103	355.1952
31	9	22	30	9	21	E	1	188793.115	0.124	286.7892
32	20	12	31	20	11	A	1	188797.215	0.102	354.5868
32	20	13	31	20	12	A	1	188797.215	0.102	354.5868
29	9	20	29	7	23	E	1	188845.433	0.338	274.4202
32	20	12	31	20	11	A	0	188894.675	0.364	238.3799
32	20	13	31	20	12	A	0	188894.675	0.364	238.3799
32	20	13	31	20	12	E	0	188894.675	-0.371	238.3609
30	7	23	29	7	22	E	1	188919.348	-0.175	275.7481
32	19	13	31	19	12	E	1	188921.799	0.187	347.4919
32	19	13	31	19	12	A	1	188952.868	0.069	346.9095
32	19	14	31	19	13	A	1	188952.868	0.069	346.9095
28	10	19	28	8	20	A	1	188975.809	0.246	272.1042
30	7	23	29	7	22	A	1	189010.969	-0.078	276.1764
32	19	13	31	19	12	A	0	189047.331	0.079	230.6721
32	19	14	31	19	13	A	0	189047.331	0.079	230.6721
32	19	14	31	19	13	E	0	189048.212	0.005	230.6539
44	3	41	44	2	42	E	0	189073.257	-0.180	242.2329
44	4	41	44	2	42	E	0	189073.257	-0.180	242.2329
44	4	41	44	3	42	E	0	189073.257	-0.180	242.2329
32	18	14	31	18	13	E	1	189095.264	0.029	340.1869
32	18	15	31	18	14	E	1	189099.467	-0.005	339.6329
32	18	14	31	18	13	A	1	189134.854	0.101	339.6390
32	18	15	31	18	14	A	1	189134.854	0.101	339.6390
30	7	23	29	7	22	E	0	189170.169	-0.007	159.4554
30	7	23	29	7	22	A	0	189172.030	-0.033	159.4413
15	9	7	14	8	7	E	0	189211.241	0.151	94.9495
32	18	14	31	18	13	A	0	189226.072	-0.102	223.3676
32	18	15	31	18	14	A	0	189226.072	-0.102	223.3676
32	18	14	31	18	13	E	0	189227.427	0.085	223.3678
32	18	15	31	18	14	E	0	189227.427	0.085	223.3504
15	9	6	14	8	6	E	0	189250.128	0.121	94.9654
15	9	7	14	8	6	A	0	189281.170	-0.003	94.9470
15	9	6	14	8	7	A	0	189281.170	-0.003	94.9470
32	17	15	31	17	14	E	1	189300.401	0.003	333.2816
32	17	16	31	17	15	E	1	189311.529	0.023	332.6959
32	17	16	31	17	15	A	1	189349.874	0.100	332.7764
32	17	15	31	17	14	A	1	189349.874	0.100	332.7764
32	17	15	31	17	14	A	0	189437.766	-0.057	216.4680
32	17	16	31	17	15	A	0	189437.766	-0.057	216.4680
32	17	15	31	17	14	E	0	189439.330	0.292	216.4705
32	17	16	31	17	15	E	0	189439.333	-0.009	216.4522
13	10	4	12	9	3	A	0	189514.332	0.294	92.9904
13	10	3	12	9	4	A	0	189514.332	0.294	92.9904
32	16	16	31	16	15	E	1	189545.986	0.054	326.7782
32	16	17	31	16	16	E	1	189566.022	-0.009	326.1694
32	16	16	31	16	15	A	1	189607.164	0.044	326.3233

J'	K'_a	K'_c	J''	K''_a	K''_c	Sym.	$v_{t_{CH_3}}$	$\nu_{Obs.}$	$\nu_{Obs.} - \nu_{Calc.}$	E_{low}
32	16	17	31	16	16	A	1	189607.164	0.044	326.3233
32	16	16	31	16	15	A	0	189691.355	-0.026	209.9755
32	16	17	31	16	16	A	0	189691.355	-0.026	209.9755
32	16	16	31	16	15	E	0	189693.093	0.119	209.9804
32	16	17	31	16	16	E	0	189693.093	0.119	209.9613
36	8	28	35	9	27	A	0	189758.902	0.107	203.5249
33	5	28	32	6	27	A	1	189835.692	0.321	289.9275
32	15	17	31	15	16	E	1	189844.255	0.102	320.6797
11	11	1	10	10	1	E	1	189871.449	0.262	208.3742
32	15	18	31	15	17	E	1	189876.111	-0.012	320.0568
32	7	26	31	7	25	A	1	189902.283	0.007	286.9704
32	15	18	31	15	17	A	1	189919.836	0.076	320.2822
32	15	17	31	15	16	A	1	189919.836	0.076	320.2822
31	9	22	30	9	21	A	0	189973.830	0.084	170.2620
31	9	22	30	9	21	E	0	189976.795	0.085	170.2727
33	5	28	32	6	27	E	1	189992.281	0.016	289.5353
32	15	17	31	15	16	A	0	189999.674	-0.023	203.8931
32	15	18	31	15	17	A	0	189999.674	-0.023	203.8931
32	15	18	31	15	17	E	0	190002.162	0.127	203.8807
33	5	28	32	6	27	E	0	190007.702	0.147	173.2366
20	7	14	19	6	13	E	0	190007.702	0.147	106.5847
33	5	28	32	6	27	A	0	190012.108	-0.022	173.2243
31	9	22	30	9	21	A	1	190019.898	-0.027	286.9157
41	17	25	41	16	25	E	0	190083.071	0.002	282.4263
34	4	30	33	5	29	A	1	190094.583	0.003	292.3814
32	7	26	31	7	25	E	0	190099.628	-0.022	170.2865
32	7	26	31	7	25	A	0	190104.921	0.066	170.2745
34	5	30	33	5	29	A	1	190108.988	-0.072	292.3814
34	4	30	33	4	29	A	1	190120.507	-0.052	292.3806
32	7	26	31	7	25	E	1	190132.265	-0.111	286.5806
34	5	30	33	4	29	A	1	190135.006	-0.034	292.3806
35	3	32	34	3	31	A	1	190165.655	-0.217	294.3426
35	4	32	34	3	31	A	1	190165.655	-0.217	294.3426
35	4	32	34	4	31	A	1	190165.655	-0.217	294.3426
33	6	28	32	6	27	A	1	190181.634	-0.044	289.9275
32	18	15	32	17	15	E	1	190206.227	-0.998	339.5960
32	14	18	31	14	17	E	1	190212.853	0.020	314.9903
32	14	19	31	14	18	E	1	190260.812	0.044	314.3628
34	4	30	33	5	29	E	0	190273.136	-0.117	175.6804
34	5	30	33	5	29	E	1	190279.182	-0.142	291.9790
34	5	30	33	5	29	E	0	190289.348	-0.070	175.6804
34	4	30	33	4	29	E	1	190292.516	-0.154	291.9780
34	5	30	33	5	29	A	0	190294.590	-0.064	175.6678
35	4	32	34	4	31	E	1	190297.332	-0.041	293.9206
35	3	32	34	3	31	E	1	190297.332	-0.041	293.9206
34	4	30	33	4	29	E	0	190302.008	-0.113	175.6794
32	14	19	31	14	18	A	1	190306.902	0.397	314.6568
32	14	18	31	14	17	A	1	190306.902	0.397	314.6568
34	5	30	33	4	29	E	1	190309.480	-0.319	291.9780
36	2	34	35	2	33	A	1	190314.472	-0.053	295.8513
36	3	34	35	2	33	A	1	190314.472	-0.053	295.8513
36	2	34	35	3	33	A	1	190314.472	-0.053	295.8513
36	3	34	35	3	33	A	1	190314.472	-0.053	295.8513
35	3	32	34	3	31	E	0	190318.134	-0.027	177.6254
35	4	32	34	4	31	E	0	190318.134	-0.027	177.6255
34	5	30	33	4	29	E	0	190318.134	-0.027	175.6794
35	3	32	34	3	31	A	0	190322.178	0.032	177.6122
35	4	32	34	4	31	A	0	190322.178	0.032	177.6122
46	4	42	46	3	43	E	0	190334.094	0.057	264.7046
46	5	42	46	3	43	E	0	190334.094	0.057	264.7046
46	5	42	46	4	43	E	0	190334.094	0.057	264.7046
46	4	42	46	3	43	A	0	190369.993	0.037	264.6929
46	5	42	46	3	43	A	0	190369.993	0.037	264.6929
46	5	42	46	4	43	A	0	190369.993	0.037	264.6929
32	14	18	31	14	17	A	0	190381.390	0.008	198.2251
32	14	19	31	14	18	A	0	190381.390	0.008	198.2251
32	14	18	31	14	17	E	0	190382.919	0.110	198.2344
32	14	19	31	14	18	E	0	190384.402	0.115	198.2147
33	6	28	32	6	27	E	0	190387.440	-0.140	173.2366
33	6	28	32	6	27	E	1	190389.871	-0.076	289.5353
33	6	28	32	6	27	A	0	190393.822	-0.029	173.2243
36	3	34	35	3	33	E	1	190409.226	0.014	295.4007
36	2	34	35	3	33	E	1	190409.226	0.014	295.4007
36	3	34	35	2	33	E	1	190409.226	0.014	295.4007
36	2	34	35	2	33	E	1	190409.226	0.014	295.4007
33	5	28	32	5	27	A	1	190415.654	-0.020	289.9082
36	2	34	35	2	33	E	0	190439.315	-0.099	179.1120
36	3	34	35	2	33	E	0	190439.315	-0.099	179.1120
36	2	34	35	3	33	E	0	190439.315	-0.099	179.1120
36	3	34	35	3	33	E	0	190439.315	-0.099	179.1120
36	2	34	35	2	33	A	0	190442.231	-0.087	179.0979
36	3	34	35	2	33	A	0	190442.231	-0.087	179.0979
36	2	34	35	3	33	A	0	190442.231	-0.087	179.0979
36	3	34	35	3	33	A	0	190442.231	-0.087	179.0979
35	9	27	34	9	25	E	1	190459.770	0.370	313.4461
37	1	36	36	1	35	A	1	190508.177	-0.040	296.9346
37	2	36	36	1	35	A	1	190508.177	-0.040	296.9346
37	1	36	36	2	35	A	1	190508.177	-0.040	296.9346
37	2	36	36	2	35	A	1	190508.177	-0.040	296.9346
37	2	36	36	2	35	E	1	190564.777	-0.224	296.4468
37	1	36	36	2	35	E	1	190564.777	-0.224	296.4468
37	2	36	36	1	35	E	1	190564.777	-0.224	296.4468
37	1	36	36	1	35	E	1	190564.777	-0.224	296.4468
13	10	3	12	9	3	E	1	190568.338	0.158	209.6648
37	1	36	36	1	35	E	0	190604.510	-0.123	180.1672
37	2	36	36	2	35	E	0	190604.510	-0.123	180.1672
37	1	36	36	1	35	A	0	190606.334	-0.034	180.1520
37	2	36	36	1	35	A	0	190606.334	-0.034	180.1520
37	1	36	36	2	35	A	0	190606.334	-0.034	180.1520
37	2	36	36	2	35	A	0	190606.334	-0.034	180.1520
41	17	24	41	16	25	E	0	190626.195	-0.023	282.4263
33	5	28	32	5	27	E	0	190641.129	-0.080	173.2154
33	5	28	32	5	27	A	0	190648.580	-0.094	173.2030
33	5	28	32	5	27	E	1	190652.778	-0.099	289.5132
32	13	19	31	13	18	E	1	190678.846	0.058	309.7158
38	0	38	37	0	37	A	1	190722.158	-0.029	297.6106
38	1	38	37	0	37	A	1	190722.158	-0.029	297.6106
38	0	38	37	1	37	A	1	190722.158	-0.029	297.6106
38	1	38	37	1	37	A	1	190722.158	-0.029	297.6106
33	9	25	33	7	27	E	1	190732.095	0.707	299.4358
38	1	38	37	1	37	E	1	190738.115	-0.091	297.0767
38	0	38	37	1	37	E	1	190738.115	-0.091	297.0767
38	1	38	37	0	37	E	1	190738.115	-0.091	297.0767
38	0	38	37	0	37	E	1	190738.115	-0.091	297.0767
32	13	20	31	13	19	E	1	190748.935	0.081	309.0938
38	0	38	37	0	37	A	0	190788.207	-0.246	180.7922

J'	K_a'	K_c'	J''	K_a''	K_c''	Sym.	$v_{t_{CH_3}}$	$\nu_{Obs.}$	$\nu_{Obs.} - \nu_{Calc.}$	E_{low}
38	1	38	37	0	37	A	0	190788.207	-0.246	180.7922
38	0	38	37	1	37	A	0	190788.207	-0.246	180.7922
38	1	38	37	1	37	A	0	190788.207	-0.246	180.7922
38	0	38	37	0	37	E	0	190788.207	0.241	180.8089
38	1	38	37	0	37	E	0	190788.207	0.241	180.8089
38	0	38	37	1	37	E	0	190788.207	0.241	180.8089
38	1	38	37	1	37	E	0	190788.207	0.241	180.8089
32	13	20	31	13	19	A	1	190796.076	0.127	309.4526
32	13	19	31	13	18	A	1	190796.076	0.127	309.4526
41	2	40	41	1	41	E	1	190804.687	-0.327	323.5177
41	1	40	41	1	41	E	1	190804.687	-0.327	323.5177
41	1	40	41	0	41	E	1	190804.687	-0.327	323.5177
30	20	10	29	20	10	E	1	190813.952	0.024	342.7330
32	13	19	31	13	18	A	0	190864.667	-0.011	192.9774
32	13	20	31	13	19	A	0	190864.667	-0.011	192.9774
20	7	13	19	6	13	E	0	190866.295	0.202	106.5847
32	13	19	31	13	18	E	0	190866.295	0.202	192.9887
32	13	20	31	13	19	E	0	190868.442	0.119	192.9692
34	7	27	33	8	26	A	0	190941.920	-0.283	186.3835
34	7	27	33	8	26	E	0	190986.742	-0.291	186.3946
33	6	28	32	5	27	E	0	191020.558	-0.322	173.2154
33	6	28	32	5	27	A	0	191030.281	-0.113	173.2030
33	6	28	32	5	27	E	1	191050.453	-0.106	289.5132
48	5	43	48	4	44	E	0	191278.551	0.322	288.2009
48	6	43	48	4	44	E	0	191278.551	0.322	288.2009
48	6	43	48	5	44	E	0	191278.551	0.322	288.2009
32	12	20	31	12	19	E	1	191285.198	0.063	304.8655
48	5	43	48	4	44	A	0	191310.031	0.212	288.1908
48	6	43	48	4	44	A	0	191310.031	0.212	288.1908
48	5	43	48	5	44	A	0	191310.031	0.212	288.1908
48	6	43	48	5	44	A	0	191310.031	0.212	288.1908
48	12	36	47	13	34	E	1	191343.409	0.461	436.8571
32	12	21	31	12	20	E	1	191387.414	0.028	304.2597
48	6	43	48	5	44	E	1	191393.144	-0.054	404.4870
48	5	43	48	5	44	E	1	191393.144	-0.054	404.4870
48	6	43	48	4	44	E	1	191393.144	-0.054	404.4870
48	5	43	48	4	44	E	1	191393.144	-0.054	404.4870
31	10	22	31	8	24	E	1	191414.092	0.053	289.5849
32	12	21	31	12	20	A	1	191431.569	0.028	304.6781
32	12	20	31	12	19	A	1	191437.533	-0.037	304.6783
32	12	21	31	12	20	A	0	191492.648	0.067	188.1591
32	12	20	31	12	19	E	0	191496.807	-0.027	188.1724
32	12	20	31	12	19	A	0	191498.239	0.100	188.1593
32	12	21	31	12	20	E	0	191500.093	0.103	188.1534
33	6	27	32	7	26	E	1	191729.627	0.241	292.9228
33	6	27	32	7	26	A	0	191822.582	0.065	176.6157
33	6	27	32	7	26	E	0	191831.995	0.024	176.6276
33	6	27	32	7	26	A	1	191872.717	-0.468	293.3048
15	4	12	14	2	13	E	1	191893.808	0.198	198.4182
50	7	44	50	6	45	E	1	191932.277	-0.006	429.0007
50	6	44	50	6	45	E	1	191932.277	-0.006	429.0007
50	7	44	50	5	45	E	1	191932.277	-0.006	429.0007
50	6	44	50	5	45	E	1	191932.277	-0.006	429.0007
32	11	21	31	11	20	E	1	192108.576	0.049	300.4535
32	11	22	31	11	21	E	1	192261.519	0.191	299.8758
32	11	22	31	11	21	A	1	192271.047	0.019	300.3469
32	11	22	31	11	21	A	0	192322.947	-0.087	183.7843
32	6	26	31	6	25	A	1	192326.579	-0.045	286.7277
32	11	21	31	11	20	A	1	192341.598	0.041	300.3494
32	11	22	31	11	21	E	0	192355.978	0.082	183.7821
32	11	21	31	11	20	E	0	192363.256	0.064	183.8004
32	11	21	31	11	20	A	0	192388.617	0.112	183.7866
21	7	15	20	6	14	A	1	192445.745	0.063	227.4360
43	3	41	43	2	42	E	1	192594.114	-0.382	344.4133
43	2	41	43	2	42	E	1	192594.114	-0.382	344.4133
43	3	41	43	1	42	E	1	192594.114	-0.382	344.4133
43	2	41	43	1	42	E	1	192594.114	-0.382	344.4133
32	6	26	31	6	25	E	0	192657.380	-0.002	170.0274
32	6	26	31	6	25	A	0	192670.525	0.001	170.0144
32	6	26	31	6	25	E	1	192717.607	-0.214	286.3154
32	8	25	31	8	24	A	1	193091.584	0.006	289.9803
32	8	25	31	8	24	E	0	193234.671	0.014	173.3156
32	8	25	31	8	24	A	0	193235.861	0.066	173.3044
32	10	23	31	10	22	A	1	193288.206	-0.020	296.4759
32	10	22	31	10	21	E	1	193324.890	0.045	296.5054
18	8	11	17	7	10	A	1	193332.352	0.174	218.2682
32	10	23	31	10	22	A	0	193337.173	0.098	179.8705
32	10	23	31	10	22	E	0	193373.896	0.053	179.8758
21	7	15	20	6	14	E	0	193501.848	-0.313	110.6796
32	10	23	31	10	22	E	1	193541.081	0.198	295.9697
32	8	25	31	8	24	E	1	193541.981	0.042	289.5849
44	10	35	43	10	33	E	1	193583.034	0.064	389.5365
31	8	23	30	8	22	E	1	193698.494	0.197	284.0078
34	17	17	34	16	18	A	0	193799.968	-0.313	229.5656
34	17	18	34	16	19	A	0	193799.968	-0.313	229.5656
45	3	42	45	2	43	E	0	193872.482	-0.189	250.0726
45	4	42	45	2	43	E	0	193872.482	-0.189	250.0726
45	4	42	45	3	43	E	0	193872.482	-0.189	250.0726
32	10	22	31	10	21	E	0	193882.079	0.097	179.9056
32	10	22	31	10	21	A	1	193897.459	-0.166	296.5021
32	10	22	31	10	21	A	0	193908.843	0.114	179.8949
33	29	4	32	29	3	E	1	193928.764	-0.209	448.7724
33	29	5	32	29	4	A	1	193933.589	-0.189	448.1647
33	29	4	32	29	3	A	1	193933.589	-0.189	448.1647
33	29	5	32	29	4	E	1	193939.886	-0.194	448.6229
16	9	8	15	8	7	A	1	193970.483	0.409	214.6169
16	9	7	15	8	8	A	1	193970.483	0.409	214.6169
32	9	24	31	9	23	A	1	193975.272	0.023	293.0585
33	28	5	32	28	4	A	1	193992.692	-0.162	436.8511
33	28	6	32	28	5	A	1	193992.692	-0.162	436.8511
33	30	3	32	30	2	A	0	193995.811	-0.415	343.7298
33	30	4	32	30	3	A	0	193995.811	-0.415	343.7298
33	28	6	32	28	5	E	1	193997.620	-0.052	437.3573
33	27	7	32	27	5	E	1	194037.600	-0.672	426.4889
33	29	4	32	29	3	A	0	194049.154	-0.256	332.0312
33	29	5	32	29	4	A	0	194049.154	-0.256	332.0312
33	29	5	32	29	4	E	0	194049.154	-0.388	332.0120
32	9	24	31	9	23	A	0	194051.497	0.129	176.4137
33	27	6	32	27	5	A	1	194057.474	-0.267	425.9407
33	27	7	32	27	6	A	1	194057.474	-0.267	425.9407
32	9	24	31	9	23	E	0	194057.474	0.069	176.4232
33	27	7	32	27	6	E	1	194060.726	0.092	426.4881
33	28	5	32	28	4	A	0	194107.196	-0.251	320.7302
33	28	6	32	28	5	A	0	194107.196	-0.251	320.7302
33	26	8	32	26	7	A	1	194129.550	0.176	415.4337
33	26	7	32	26	6	A	1	194129.550	0.176	415.4337

J'	K'_a	K'_c	J''	K''_a	K''_c	Sym.	$v_{t_{\mathrm{CH_3}}}$	$\nu_{\mathrm{Obs.}}$	$\nu_{\mathrm{Obs.}} - \nu_{\mathrm{Calc.}}$	E_{low}
33	26	7	32	26	6	E	1	194129.550	-0.310	416.0150
33	27	6	32	27	5	A	0	194171.043	-0.068	309.8272
33	27	7	32	27	6	A	0	194171.043	-0.068	309.8272
33	27	7	32	27	6	E	0	194171.043	-0.371	309.8099
33	25	9	32	25	8	E	1	194192.852	-0.141	405.7896
33	25	8	32	25	7	E	1	194206.383	-0.047	405.9378
33	25	9	32	25	8	A	1	194208.971	0.092	405.3306
33	25	8	32	25	7	A	1	194208.971	0.092	405.3306
33	26	7	32	26	6	A	0	194241.219	-0.114	299.3224
33	26	8	32	26	7	A	0	194241.219	-0.114	299.3224
33	26	8	32	26	7	E	0	194241.219	-0.085	299.3041
33	24	9	32	24	8	E	1	194291.805	0.138	396.2564
33	24	10	32	24	9	A	1	194297.644	0.012	395.6316
33	24	9	32	24	8	A	1	194297.644	0.012	395.6316
33	25	8	32	25	7	A	0	194319.203	-0.034	289.2162
33	25	9	32	25	8	A	0	194319.203	-0.034	289.2162
33	25	9	32	25	8	E	0	194319.203	-0.096	289.1971
18	8	11	17	7	11	E	0	194371.117	-0.151	101.5335
33	23	11	32	23	10	E	1	194375.212	0.039	386.6819
32	17	15	32	16	16	A	0	194385.464	-0.342	216.3029
32	17	16	32	16	17	A	0	194385.464	-0.342	216.3029
33	23	10	32	23	9	E	1	194387.399	0.201	386.9709
18	8	11	17	7	10	A	0	194390.816	-0.110	101.5300
33	23	10	32	23	9	A	1	194397.374	0.053	386.3371
33	23	11	32	23	10	A	1	194397.374	0.053	386.3371
33	24	9	32	24	8	A	0	194406.287	0.096	279.5089
33	24	10	32	24	9	A	0	194406.287	0.096	279.5089
33	24	10	32	24	9	E	0	194406.287	-0.075	279.4893
18	8	10	17	7	10	E	0	194411.171	0.043	101.5480
18	8	10	17	7	11	A	0	194484.571	-0.384	101.5273
33	22	12	32	22	11	E	1	194484.571	-0.076	377.7277
33	22	11	32	22	10	E	1	194495.098	0.056	378.0814
33	23	10	32	23	9	A	0	194504.014	0.138	270.2011
33	23	11	32	23	10	A	0	194504.014	0.138	270.2011
33	23	11	32	23	10	E	0	194504.014	-0.163	270.1812
33	22	11	32	22	10	A	1	194510.178	0.133	377.4474
33	22	12	32	22	11	A	1	194510.178	0.133	377.4474
20	7	13	19	6	14	A	0	194536.484	-0.312	106.4482
31	8	23	30	8	22	A	0	194544.547	0.099	167.6460
31	8	23	30	8	22	E	0	194552.701	-0.024	167.6587
16	9	8	15	8	7	E	0	194552.701	-0.024	97.9284
31	8	23	30	8	22	A	1	194567.575	-0.120	284.3488
33	21	13	32	21	12	E	1	194609.760	0.020	369.1748
33	22	11	32	22	10	A	0	194614.705	0.323	261.2933
33	22	12	32	22	11	A	0	194614.705	0.323	261.2933
33	22	12	32	22	11	E	0	194614.705	-0.130	261.2734
33	21	12	32	21	11	E	1	194618.011	0.275	369.5882
33	21	13	32	21	12	A	1	194638.571	0.132	368.9629
33	21	12	32	21	11	A	1	194638.571	0.132	368.9629
31	7	24	30	7	23	A	1	194670.914	-0.096	282.4811
33	21	12	32	21	11	A	0	194740.711	0.382	252.7862
33	21	13	32	21	12	A	0	194740.711	0.382	252.7862
33	21	13	32	21	12	E	0	194740.711	-0.247	252.7666
31	7	24	30	7	23	E	1	194747.297	-0.161	282.0497
33	20	13	32	20	12	A	1	194785.951	0.102	360.8844
33	20	14	32	20	13	A	1	194785.951	0.102	360.8844
20	6	14	19	5	15	E	1	194801.115	0.609	220.5942
33	20	13	32	20	12	A	0	194885.272	0.226	244.6808
33	20	14	32	20	13	A	0	194885.272	0.226	244.6808
31	7	24	30	7	23	E	0	194894.941	0.017	165.7654
31	7	24	30	7	23	A	0	194900.529	0.011	165.7514
34	5	29	33	6	28	A	1	194911.003	-0.016	296.2713
22	7	16	21	6	15	A	1	194941.332	0.012	231.7696
33	19	15	32	19	14	A	1	194956.697	0.103	353.2123
33	19	14	32	19	13	A	1	194956.697	0.103	353.2123
32	9	24	31	9	23	E	1	194977.421	0.122	292.5851
33	7	27	32	7	26	A	1	195025.112	-0.016	293.3048
16	9	8	15	8	8	E	0	195030.869	0.098	97.9124
35	5	31	34	5	30	A	1	195045.263	-0.109	298.7228
35	4	31	34	4	30	A	1	195052.092	0.249	298.7223
33	19	14	32	19	13	A	0	195052.113	-0.709	236.9780
33	19	15	32	19	14	A	0	195052.113	-0.709	236.9780
33	19	15	32	19	14	E	0	195054.028	0.131	236.9598
35	5	31	34	4	30	A	1	195059.885	0.033	298.7223
16	9	7	15	8	7	E	0	195069.880	-0.059	97.9284
34	5	29	33	6	28	E	1	195086.498	0.005	295.8860
34	5	29	33	6	28	E	0	195098.055	-0.156	179.5872
25	17	9	25	16	9	E	0	195098.055	-0.156	176.2437
16	9	8	15	8	7	A	0	195101.031	0.028	97.9100
16	9	7	15	8	8	A	0	195101.031	0.028	97.9100
34	5	29	33	6	28	A	0	195103.204	-0.062	179.5751
33	18	15	32	18	14	E	1	195112.020	-0.095	346.4944
36	3	33	35	3	32	A	1	195113.841	-0.135	300.6859
36	4	33	35	3	32	A	1	195113.841	-0.135	300.6859
36	3	33	35	4	32	A	1	195113.841	-0.135	300.6859
36	4	33	35	4	32	A	1	195113.841	-0.135	300.6859
34	6	29	33	6	28	A	1	195115.242	0.095	296.2713
33	18	16	32	18	15	E	1	195117.782	0.080	345.9406
33	18	15	32	18	14	A	1	195156.452	0.121	345.9479
33	18	16	32	18	15	A	1	195156.452	0.121	345.9479
47	4	43	47	3	44	A	0	195197.212	0.242	273.0233
47	5	43	47	3	44	A	0	195197.212	0.242	273.0233
47	5	43	47	4	44	A	0	195197.212	0.242	273.0233
35	4	31	34	5	30	E	1	195204.503	0.021	298.3261
35	5	31	34	5	30	E	1	195213.961	-0.075	298.3261
35	4	31	34	4	30	E	1	195221.599	-0.011	298.3255
35	5	31	34	5	30	E	0	195226.328	-0.016	182.0278
35	5	31	34	5	30	A	0	195231.473	-0.062	182.0153
35	4	31	34	4	30	E	0	195233.388	-0.138	182.0272
33	7	27	32	7	26	E	0	195234.764	0.113	176.6276
35	4	31	34	4	30	A	0	195238.759	-0.001	182.0148
33	7	27	32	7	26	A	0	195240.576	0.052	176.6157
35	5	31	34	4	30	E	0	195242.418	-0.091	182.0272
36	4	33	35	4	32	E	1	195244.769	-0.003	300.2683
36	3	33	35	3	32	E	1	195244.769	-0.003	300.2683
35	5	31	34	4	30	A	0	195247.668	-0.136	182.0148
33	18	15	32	18	14	A	0	195249.315	0.041	229.6795
33	18	16	32	18	15	A	0	195249.315	0.041	229.6795
33	18	15	32	18	14	E	0	195250.668	0.100	229.6797
33	18	16	32	18	15	E	0	195250.668	0.100	229.6624
33	7	27	32	7	26	E	1	195254.277	-0.128	292.9228
34	5	29	33	5	28	A	1	195257.269	-0.057	296.2598
37	2	35	36	2	34	A	1	195267.465	-0.252	302.1995
37	3	35	36	2	34	A	1	195267.465	-0.252	302.1995
37	2	35	36	3	34	A	1	195267.465	-0.252	302.1995
37	3	35	36	3	34	A	1	195267.465	-0.252	302.1995

J'	K'_a	K'_c	J''	K''_a	K''_c	Sym.	$v_{t_{\mathrm{CH_3}}}$	$\nu_{\mathrm{Obs.}}$	$\nu_{\mathrm{Obs.}} - \nu_{\mathrm{Calc.}}$	E_{low}
36	3	33	35	3	32	E	0	195267.469	0.109	183.9738
36	4	33	35	3	32	E	0	195267.469	0.109	183.9738
36	3	33	35	4	32	E	0	195267.469	0.109	183.9738
36	4	33	35	4	32	E	0	195267.469	0.109	183.9738
36	3	33	35	3	32	A	0	195271.355	-0.026	183.9607
36	4	33	35	3	32	A	0	195271.355	-0.026	183.9607
36	3	33	35	4	32	A	0	195271.355	-0.026	183.9607
36	4	33	35	4	32	A	0	195271.355	-0.026	183.9607
42	1	41	42	0	42	A	0	195276.692	0.453	214.2656
42	2	41	42	0	42	A	0	195276.692	0.453	214.2656
42	1	41	42	1	42	A	0	195276.692	0.453	214.2656
42	2	41	42	1	42	A	0	195276.692	0.453	214.2656
14	10	5	13	9	5	E	0	195309.555	0.232	95.5477
34	6	29	33	6	28	E	0	195322.967	-0.068	179.5872
34	6	29	33	6	28	E	1	195322.967	0.049	295.8860
34	6	29	33	6	28	A	0	195329.354	0.003	179.5751
33	17	16	32	17	15	E	1	195337.718	0.107	339.5960
47	5	43	47	4	44	E	1	195341.169	0.791	389.3162
47	4	43	47	4	44	E	1	195341.169	0.791	389.3162
47	5	43	47	3	44	E	1	195341.169	0.791	389.3162
47	4	43	47	3	44	E	1	195341.169	0.791	389.3162
37	3	35	36	3	34	E	1	195361.976	-0.021	301.7521
37	2	35	36	3	34	E	1	195361.976	-0.021	301.7521
37	3	35	36	2	34	E	1	195361.976	-0.021	301.7521
37	2	35	36	2	34	E	1	195361.976	-0.021	301.7521
14	10	5	13	9	4	A	0	195379.363	0.184	95.5477
14	10	4	13	9	5	A	0	195379.363	0.184	95.5477
37	2	35	36	2	34	E	0	195393.818	-0.026	185.4643
37	3	35	36	2	34	E	0	195393.818	-0.026	185.4643
37	2	35	36	3	34	E	0	195393.818	-0.026	185.4643
37	3	35	36	3	34	E	0	195393.818	-0.026	185.4643
37	2	35	36	2	34	A	0	195396.705	-0.029	185.4503
37	3	35	36	2	34	A	0	195396.705	-0.029	185.4503
37	2	35	36	3	34	A	0	195396.705	-0.029	185.4503
37	3	35	36	3	34	A	0	195396.705	-0.029	185.4503
38	1	37	37	1	36	A	1	195462.862	-0.095	303.2893
38	2	37	37	1	36	A	1	195462.862	-0.095	303.2893
38	1	37	37	2	36	A	1	195462.862	-0.095	303.2893
38	2	37	37	2	36	A	1	195462.862	-0.095	303.2893
34	5	29	33	5	28	E	0	195477.886	-0.008	179.5745
33	17	16	32	17	15	A	0	195481.950	0.044	222.7869
33	17	17	32	17	16	A	0	195481.950	0.044	222.7869
33	17	16	32	17	15	E	0	195483.646	0.230	222.7895
33	17	17	32	17	16	E	0	195483.646	0.230	222.7712
34	5	29	33	5	28	A	0	195485.001	0.016	179.5624
38	2	37	37	2	36	E	1	195519.511	-0.043	302.8034
38	1	37	37	2	36	E	1	195519.511	-0.043	302.8034
38	2	37	37	1	36	E	1	195519.511	-0.043	302.8034
38	1	37	37	1	36	E	1	195519.511	-0.043	302.8034
26	17	10	26	16	11	E	0	195537.178	-0.347	181.3460
38	1	37	37	1	36	E	0	195560.663	-0.043	186.5251
38	2	37	37	1	36	E	0	195560.663	-0.043	186.5251
38	1	37	37	2	36	E	0	195560.663	-0.043	186.5251
38	2	37	37	2	36	E	0	195560.663	-0.043	186.5251
38	1	37	37	1	36	A	0	195562.471	0.036	186.5099
38	2	37	37	1	36	A	0	195562.471	0.036	186.5099
38	1	37	37	2	36	A	0	195562.471	0.036	186.5099
38	2	37	37	2	36	A	0	195562.471	0.036	186.5099
33	16	17	32	16	16	E	1	195607.948	0.116	333.1008
33	16	18	32	16	17	E	1	195630.998	0.022	332.4926
33	16	17	32	16	16	A	1	195675.798	-0.002	332.6479
33	16	18	32	16	17	A	1	195675.798	-0.002	332.6479
39	0	39	38	0	38	A	1	195677.382	0.018	303.9724
39	1	39	38	0	38	A	1	195677.382	0.018	303.9724
39	0	39	38	1	38	A	1	195677.382	0.018	303.9724
39	1	39	38	1	38	A	1	195677.382	0.018	303.9724
39	1	39	38	1	38	E	1	195693.217	-0.033	303.4390
39	0	39	38	1	38	E	1	195693.217	-0.033	303.4390
39	1	39	38	0	38	E	1	195693.217	-0.033	303.4390
39	0	39	38	0	38	E	1	195693.217	-0.033	303.4390
34	6	29	33	5	28	E	0	195702.707	0.000	179.5745
34	6	29	33	5	28	A	0	195710.667	-0.406	179.5624
34	6	29	33	5	28	E	1	195720.682	0.081	295.8727
39	0	39	38	0	38	A	0	195744.672	-0.274	187.1563
39	1	39	38	0	38	A	0	195744.672	-0.274	187.1563
39	0	39	38	1	38	A	0	195744.672	-0.274	187.1563
39	1	39	38	1	38	A	0	195744.672	-0.274	187.1563
39	0	39	38	0	38	E	0	195744.672	0.209	187.1729
39	1	39	38	0	38	E	0	195744.672	0.209	187.1729
39	0	39	38	1	38	E	0	195744.672	0.209	187.1729
39	1	39	38	1	38	E	0	195744.672	0.209	187.1729
33	16	17	32	16	16	A	0	195760.990	0.024	216.3029
33	16	18	32	16	17	A	0	195760.990	0.024	216.3029
33	16	17	32	16	16	E	0	195762.891	0.167	216.3079
33	16	18	32	16	17	E	0	195762.891	0.167	216.2888
32	9	23	31	9	22	E	1	195905.418	0.084	293.0867
33	15	18	32	15	17	E	1	195936.521	-0.072	327.0123
33	15	19	32	15	18	E	1	195972.908	0.016	326.3904
33	15	19	32	15	18	A	1	196020.554	0.116	326.6172
33	15	18	32	15	17	A	1	196020.554	0.116	326.6172
31	7	25	30	6	24	E	0	196098.399	-0.449	163.7454
33	15	18	32	15	17	A	0	196100.886	0.005	210.2309
33	15	19	32	15	18	A	0	196100.886	0.005	210.2309
33	15	18	32	15	17	E	0	196102.627	0.252	210.2381
33	15	19	32	15	18	E	0	196103.584	0.110	210.2185
31	7	25	30	6	24	A	0	196141.897	-0.057	163.7319
49	5	44	49	4	45	E	0	196141.897	0.333	297.0223
49	6	44	49	4	45	E	0	196141.897	0.333	297.0223
49	6	44	49	5	45	E	0	196141.897	0.333	297.0223
49	5	44	49	4	45	A	0	196174.480	0.455	297.0123
49	6	44	49	4	45	A	0	196174.480	0.455	297.0123
49	5	44	49	5	45	A	0	196174.480	0.455	297.0123
49	6	44	49	5	45	A	0	196174.480	0.455	297.0123
33	14	19	32	14	18	E	1	196343.990	0.005	321.3351
33	14	20	32	14	19	E	1	196398.039	0.005	320.7092
33	14	20	32	14	19	A	1	196447.932	0.104	321.0047
33	14	19	32	14	18	A	1	196447.932	0.104	321.0047
33	14	19	32	14	18	A	0	196522.753	0.048	204.5755
33	14	20	32	14	19	A	0	196522.753	0.048	204.5755
33	14	19	32	14	18	E	0	196524.410	0.153	204.5849
33	14	20	32	14	19	E	0	196526.067	0.141	204.5652
39	9	30	38	10	29	E	0	196618.368	-0.139	229.4560
33	6	27	32	6	26	A	1	196724.563	0.030	293.1430
33	13	20	32	13	19	E	1	196860.715	0.051	316.0762
33	13	21	32	13	20	E	1	196939.602	0.124	315.4565
33	13	21	32	13	20	A	1	196990.269	-0.021	315.8168

J'	K'_a	K'_c	J''	K''_a	K''_c	Sym.	$v_{t_{\mathrm{CH_3}}}$	$\nu_{\mathrm{Obs.}}$	$\nu_{\mathrm{Obs.}} - \nu_{\mathrm{Calc.}}$	E_{low}
33	13	20	32	13	19	A	1	196991.138	-0.028	315.8168
33	6	27	32	6	26	E	0	197040.845	-0.024	176.4538
33	6	27	32	6	26	A	0	197053.174	0.014	176.4412
33	13	20	32	13	19	A	0	197058.757	-0.016	199.3439
33	13	21	32	13	20	A	0	197058.757	-0.016	199.3439
33	13	20	32	13	19	E	0	197060.527	0.171	199.3553
33	13	21	32	13	20	E	0	197063.016	0.194	199.3358
33	6	27	32	6	26	E	1	197095.707	-0.216	292.7438
32	9	23	31	9	22	A	0	197260.819	0.068	176.5989
32	9	23	31	9	22	E	0	197268.328	0.066	176.6096
32	9	23	31	9	22	A	1	197329.722	-0.176	293.2541
44	3	42	44	2	43	E	1	197381.378	-0.390	351.9266
44	2	42	44	2	43	E	1	197381.378	-0.390	351.9266
44	3	42	44	1	43	E	1	197381.378	-0.390	351.9266
44	2	42	44	1	43	E	1	197381.378	-0.390	351.9266
33	12	21	32	12	20	E	1	197536.879	0.062	311.2460
33	12	22	32	12	21	E	1	197652.205	0.110	310.6436
33	12	22	32	12	21	A	1	197697.396	0.022	311.0636
33	12	21	32	12	20	A	1	197709.412	-0.059	311.0640
23	7	17	22	6	16	A	0	197746.667	-0.533	119.5707
50	18	32	50	17	34	E	0	197746.667	-0.313	371.4552
33	12	22	32	12	21	A	0	197757.010	0.093	194.5466
33	12	21	32	12	20	E	0	197764.307	0.090	194.5601
33	12	21	32	12	20	A	0	197767.890	-0.188	194.5470
33	12	22	32	12	21	E	0	197767.890	0.345	194.5411
32	7	26	31	6	25	E	0	197866.154	-0.124	170.0274
32	7	26	31	6	25	A	0	197901.028	-0.139	170.0144
34	6	28	33	7	27	E	1	198045.094	0.140	299.4358
32	7	26	31	6	25	E	1	198085.514	1.156	286.3154
34	6	28	33	7	27	A	0	198118.102	0.276	183.1282
34	6	28	33	7	27	E	0	198121.944	0.042	183.1399
25	7	19	24	6	18	A	0	198255.661	-0.487	129.4180
24	7	18	23	6	17	E	0	198323.611	-0.289	124.3945
19	6	14	18	4	14	E	1	198392.779	0.055	216.0298
24	7	18	23	6	17	A	0	198394.704	-0.392	124.3798
33	11	22	32	11	21	E	1	198465.069	0.123	306.8615
15	2	13	14	1	14	E	0	198526.455	-0.081	80.0747
15	2	13	14	1	14	A	0	198550.190	0.228	80.0576
22	6	17	21	5	17	E	1	198593.321	0.506	228.7365
33	11	23	32	11	22	A	1	198622.748	0.002	306.7604
33	11	23	32	11	22	E	1	198638.381	0.139	306.2889
46	3	43	46	2	44	E	0	198669.663	-0.193	258.0776
46	4	43	46	2	44	E	0	198669.663	-0.193	258.0776
46	4	43	46	3	44	E	0	198669.663	-0.193	258.0776
33	11	23	32	11	22	A	0	198673.263	0.162	190.1995
33	8	26	32	8	25	A	1	198699.868	-0.023	296.4211
33	11	23	32	11	22	E	0	198719.728	0.092	190.1984
33	11	22	32	11	21	A	1	198752.348	0.052	306.7652
33	11	22	32	11	21	E	0	198755.527	0.074	190.2170
33	11	22	32	11	21	A	0	198793.631	0.105	190.2040
33	8	26	32	8	25	E	0	198863.514	0.022	179.7612
33	8	26	32	8	25	A	0	198865.943	0.053	179.7500
19	8	12	18	7	11	A	1	198884.820	0.024	221.8580
33	8	26	32	8	25	E	1	199042.417	0.067	296.0407
19	8	11	18	7	12	A	1	199105.128	0.213	221.8516
43	10	34	42	10	32	E	0	199315.634	0.626	264.2351
48	18	30	48	17	32	E	0	199549.452	0.535	351.6135
33	10	24	32	10	23	A	1	199669.753	-0.027	302.9233
33	10	24	32	10	23	A	0	199721.676	0.078	186.3196
34	31	4	33	31	3	A	1	199740.635	-0.334	478.4654
34	31	3	33	31	2	A	1	199740.635	-0.334	478.4654
33	10	24	32	10	23	E	0	199745.325	0.108	186.3261
17	9	9	16	8	8	A	1	199763.204	0.217	217.7805
17	9	8	16	8	9	A	1	199765.241	0.476	217.7804
34	29	5	33	29	4	E	1	199847.244	-0.191	455.2412
34	29	5	33	29	4	A	1	199853.325	-0.246	454.6337
34	29	6	33	29	5	A	1	199853.325	-0.246	454.6337
32	7	25	31	7	24	A	1	199857.267	0.085	288.9747
34	29	6	33	29	5	E	1	199859.542	-0.129	455.0920
34	31	3	33	31	2	A	0	199861.222	-0.377	362.2952
34	31	4	33	31	3	A	0	199861.222	-0.377	362.2952
34	31	4	33	31	3	E	0	199861.222	-0.394	362.2752
33	7	27	32	6	26	A	1	199876.684	0.208	293.1430
33	10	23	32	10	22	E	1	199892.178	0.023	302.9540
34	30	4	33	30	3	A	0	199914.361	-0.371	350.2008
34	30	5	33	30	4	A	0	199914.361	-0.371	350.2008
34	28	6	33	28	5	A	1	199917.729	-0.061	443.3220
34	28	7	33	28	6	A	1	199917.729	-0.061	443.3220
19	8	12	18	7	11	A	0	199952.683	-0.116	105.1210
34	29	5	33	29	4	A	0	199972.191	-0.290	338.5040
34	29	6	33	29	5	A	0	199972.191	-0.290	338.5040
34	27	8	33	27	6	E	1	199976.449	-0.175	432.9616
36	4	32	35	5	31	A	1	199978.525	-0.218	305.2288
48	4	44	48	3	45	E	0	199982.930	0.350	281.5302
48	5	44	48	3	45	E	0	199982.930	0.350	281.5302
48	5	44	48	4	45	E	0	199982.930	0.350	281.5302
36	5	32	35	5	31	A	1	199982.933	-0.209	305.2288
36	4	32	35	4	31	A	1	199986.603	-0.150	305.2285
36	5	32	35	4	31	A	1	199990.864	-0.288	305.2285
34	27	7	33	27	7	E	1	199990.864	0.215	432.9613
19	8	12	18	7	12	E	0	200004.532	0.017	105.1224
19	8	11	18	7	11	E	0	200015.823	0.037	105.1378
48	4	44	48	3	45	A	0	200020.543	0.192	281.5188
48	5	44	48	3	45	A	0	200020.543	0.192	281.5188
48	5	44	48	4	45	A	0	200020.543	0.192	281.5188
34	28	6	33	28	5	A	0	200035.417	-0.140	327.2050
34	28	7	33	28	6	A	0	200035.417	-0.140	327.2050
34	28	7	33	28	6	E	0	200035.417	-0.402	327.1866
35	6	30	34	6	29	A	1	200042.041	0.107	302.7796
46	18	29	46	17	29	E	0	200046.590	0.061	332.6328
37	3	34	36	3	33	A	1	200062.737	-0.125	307.1942
37	4	34	36	3	33	A	1	200062.737	-0.125	307.1942
37	3	34	36	4	33	A	1	200062.737	-0.125	307.1942
37	4	34	36	4	33	A	1	200062.737	-0.125	307.1942
34	26	8	33	26	7	A	1	200066.271	-0.093	421.9092
34	26	9	33	26	8	A	1	200066.271	-0.093	421.9092
34	26	8	33	26	7	E	1	200066.271	0.308	422.4905
34	7	28	33	7	27	A	1	200076.892	-0.013	299.8102
32	7	25	31	7	24	E	1	200079.844	-0.259	288.5458
33	10	24	32	10	23	E	1	200088.445	0.164	302.4256
34	27	7	33	27	6	A	0	200104.720	-0.090	316.3041
34	27	8	33	27	7	A	0	200104.720	-0.090	316.3041
34	27	8	33	27	7	E	0	200104.720	-0.279	316.2868
35	5	30	34	6	29	E	1	200109.329	-0.144	302.4013
35	5	30	34	6	29	E	0	200119.419	0.000	186.1025
35	5	30	34	6	29	A	0	200124.776	-0.181	186.0906

J'	K_a'	K_c'	J''	K_a''	K_c''	Sym.	$v_{t_{CH_3}}$	$\nu_{Obs.}$	$\nu_{Obs.} - \nu_{Calc.}$	E_{low}
35	5	30	34	5	29	A	1	200126.776	-0.002	302.7728
33	9	25	32	9	24	A	1	200126.776	-0.002	299.5288
34	25	10	33	25	9	E	1	200134.660	-0.349	412.2671
32	7	25	31	7	24	E	0	200143.410	-0.091	172.2664
36	4	32	35	5	31	E	1	200145.220	0.296	304.8377
36	5	32	35	5	31	E	1	200150.063	-0.153	304.8377
32	7	25	31	7	24	A	0	200152.816	0.036	172.2526
36	4	32	35	4	31	E	1	200154.558	0.080	304.8374
36	4	32	35	5	31	E	0	200159.723	-0.041	188.5398
36	5	32	35	4	31	E	1	200159.723	-0.047	304.8374
19	8	11	18	7	12	A	0	200161.330	-0.016	105.1149
36	5	32	35	5	31	E	0	200164.756	0.034	188.5398
36	4	32	35	4	31	E	0	200168.677	-0.070	188.5395
36	5	32	35	5	31	A	0	200169.955	0.087	188.5276
36	4	32	35	4	31	A	0	200173.867	-0.052	188.5273
36	5	32	35	4	31	A	0	200178.826	-0.085	188.5273
34	26	8	33	26	7	A	0	200181.290	0.031	305.8016
34	26	9	33	26	8	A	0	200181.290	0.031	305.8016
34	26	9	33	26	8	E	0	200181.290	0.033	305.7833
37	4	34	36	4	33	E	1	200192.786	-0.026	306.7809
37	3	34	36	3	33	E	1	200192.786	-0.026	306.7809
37	3	34	36	3	33	E	0	200217.242	-0.009	190.4872
37	4	34	36	3	33	E	0	200217.242	-0.009	190.4872
37	3	34	36	4	33	E	0	200217.242	-0.009	190.4872
37	4	34	36	4	33	E	0	200217.242	-0.009	190.4872
37	3	34	36	3	33	A	0	200221.104	-0.142	190.4742
37	4	34	36	3	33	A	0	200221.104	-0.142	190.4742
37	3	34	36	4	33	A	0	200221.104	-0.142	190.4743
37	4	34	36	4	33	A	0	200221.104	-0.142	190.4743
34	24	11	33	24	10	E	1	200228.611	0.134	402.5167
34	24	10	33	24	9	E	1	200242.590	0.353	402.7373
35	6	30	34	5	29	A	1	200245.880	-0.183	302.7728
35	6	30	34	6	29	E	1	200248.411	-0.057	302.4013
35	6	30	34	6	29	E	0	200251.188	0.120	186.1025
35	6	30	34	6	29	A	0	200257.431	0.044	186.0906
34	25	9	33	25	8	A	0	200266.238	0.103	295.6980
34	25	10	33	25	9	A	0	200266.238	0.103	295.6980
34	25	10	33	25	9	E	0	200266.238	0.004	295.6788
34	7	28	33	7	27	E	0	200296.005	-0.017	183.1399
34	7	28	33	7	27	A	0	200302.397	-0.013	183.1282
34	7	28	33	7	27	E	1	200308.575	0.058	299.4358
38	3	36	37	3	35	E	1	200314.830	-0.025	308.2687
38	2	36	37	3	35	E	1	200314.830	-0.025	308.2687
38	3	36	37	2	35	E	1	200314.830	-0.025	308.2687
38	2	36	37	2	35	E	1	200314.830	-0.025	308.2687
35	7	28	34	8	27	A	0	200324.504	0.234	193.2001
34	23	12	33	23	11	E	1	200334.020	0.173	393.1655
35	5	30	34	5	29	E	0	200344.197	-0.036	186.0950
34	23	11	33	23	10	E	1	200346.060	-0.376	393.4549
35	5	30	34	5	29	E	1	200346.060	0.163	302.3934
38	2	36	37	2	35	E	0	200348.323	-0.020	191.9820
38	3	36	37	2	35	E	0	200348.323	-0.020	191.9820
38	2	36	37	3	35	E	0	200348.323	-0.020	191.9820
38	3	36	37	3	35	E	0	200348.323	-0.020	191.9820
38	2	36	37	2	35	A	0	200351.160	-0.026	191.9681
38	3	36	37	2	35	A	0	200351.160	-0.026	191.9681
38	2	36	37	3	35	A	0	200351.160	-0.026	191.9681
38	3	36	37	3	35	A	0	200351.160	-0.026	191.9681
35	5	30	34	5	29	A	0	200351.160	-0.026	186.0831
47	18	29	47	17	31	E	0	200351.160	0.159	342.0092
35	7	28	34	8	27	E	0	200356.948	-0.165	193.2111
34	23	12	33	23	11	A	1	200358.708	0.220	392.8215
34	23	11	33	23	10	A	1	200358.708	0.220	392.8215
34	24	10	33	24	9	A	0	200361.076	0.133	285.9936
34	24	11	33	24	10	A	0	200361.076	0.133	285.9936
34	24	11	33	24	10	E	0	200361.076	-0.086	285.9739
39	1	38	38	1	37	A	1	200417.586	-0.029	309.8092
39	2	38	38	1	37	A	1	200417.586	-0.029	309.8092
39	1	38	38	2	37	A	1	200417.586	-0.029	309.8092
39	2	38	38	2	37	A	1	200417.586	-0.029	309.8092
34	22	13	33	22	12	E	1	200453.478	0.033	384.2150
34	22	12	33	22	11	E	1	200464.314	0.152	384.5690
34	23	11	33	23	10	A	0	200467.714	0.185	276.6890
34	23	12	33	23	11	A	0	200467.714	0.185	276.6890
34	23	12	33	23	11	E	0	200467.714	-0.176	276.6691
39	2	38	38	2	37	E	1	200473.973	-0.055	309.3252
39	1	38	38	2	37	E	1	200473.973	-0.055	309.3252
39	2	38	38	1	37	E	1	200473.973	-0.055	309.3252
39	1	38	38	1	37	E	1	200473.973	-0.055	309.3252
35	6	30	34	5	29	E	0	200475.898	0.016	186.0950
34	22	12	33	22	11	A	1	200481.668	0.123	383.9355
34	22	13	33	22	12	A	1	200481.668	0.123	383.9355
35	6	30	34	5	29	A	0	200483.493	0.020	186.0831
35	6	30	34	5	29	E	1	200484.992	0.100	302.3934
39	1	38	38	1	37	E	0	200516.616	-0.085	193.0483
39	2	38	38	1	37	E	0	200516.616	-0.085	193.0483
39	1	38	38	2	37	E	0	200516.616	-0.085	193.0483
39	2	38	38	2	37	E	0	200516.616	-0.085	193.0483
39	1	38	38	1	37	A	0	200518.416	-0.008	193.0332
39	2	38	38	1	37	A	0	200518.416	-0.008	193.0332
39	1	38	38	2	37	A	0	200518.416	-0.008	193.0332
39	2	38	38	2	37	A	0	200518.416	-0.008	193.0332
34	22	12	33	22	11	A	0	200588.488	0.297	267.7849
34	22	13	33	22	12	A	0	200588.488	0.297	267.7849
34	22	13	33	22	12	E	0	200588.488	-0.229	267.7650
34	21	13	33	21	12	E	1	200598.609	0.405	376.0799
34	21	14	33	21	13	A	1	200621.997	0.191	375.4554
34	21	13	33	21	12	A	1	200621.997	0.191	375.4554
40	0	40	39	0	39	A	1	200632.404	-0.030	310.4995
40	1	40	39	0	39	A	1	200632.404	-0.030	310.4995
40	0	40	39	1	39	A	1	200632.404	-0.030	310.4995
40	1	40	39	1	39	A	1	200632.404	-0.030	310.4995
40	1	40	39	1	39	E	1	200648.105	-0.084	309.9666
40	1	40	39	0	39	E	1	200648.105	-0.084	309.9666
40	0	40	39	0	39	E	1	200648.105	-0.084	309.9666
33	10	23	32	10	22	E	0	200656.160	-0.014	186.3728
33	10	23	32	10	22	A	0	200668.383	0.064	186.3630
33	10	23	32	10	22	A	1	200676.125	-0.080	302.9698
40	0	40	39	0	39	A	0	200701.115	-0.216	193.6856
40	1	40	39	0	39	A	0	200701.115	-0.216	193.6856
40	0	40	39	1	39	A	0	200701.115	-0.216	193.6856
40	1	40	39	1	39	A	0	200701.115	-0.216	193.6856
40	0	40	39	0	39	E	0	200701.115	0.263	193.7022
40	1	40	39	0	39	E	0	200701.115	0.263	193.7022
40	0	40	39	1	39	E	0	200701.115	0.263	193.7022
40	1	40	39	1	39	E	0	200701.115	0.263	193.7022

J'	K'_a	K'_c	J''	K''_a	K''_c	Sym.	$v_{t_{CH_3}}$	$\nu_{Obs.}$	$\nu_{Obs.} - \nu_{Calc.}$	E_{low}
34	21	13	33	21	12	A	0	200726.078	0.267	259.2821
34	21	14	33	21	13	A	0	200726.078	0.267	259.2821
34	21	14	33	21	13	E	0	200726.078	-0.451	259.2625
34	20	15	33	20	14	E	1	200747.851	0.043	367.5212
34	20	14	33	20	13	E	1	200752.273	0.159	367.9885
34	20	14	33	20	13	A	1	200783.069	0.107	367.3817
34	20	15	33	20	14	A	1	200783.069	0.107	367.3817
33	16	17	33	15	19	E	1	200808.185	-0.932	332.9273
17	9	9	16	8	9	E	0	200828.340	-0.019	101.0774
34	20	14	33	20	13	A	0	200884.199	0.140	251.1814
34	20	15	33	20	14	A	0	200884.199	0.140	251.1814
34	20	15	33	20	14	E	0	200885.092	0.090	251.1624
33	24	9	32	24	9	E	1	200892.354	-0.156	396.0362
17	9	9	16	8	8	A	0	200898.006	0.048	101.0749
17	9	8	16	8	9	A	0	200899.652	0.025	101.0748
34	19	15	33	19	14	E	1	200930.851	0.069	360.2955
34	19	16	33	19	15	E	1	200930.851	0.069	359.7814
34	19	15	33	19	14	A	1	200969.925	0.142	359.7154
34	19	16	33	19	15	A	1	200969.925	0.142	359.7154
50	5	45	50	4	46	A	0	201032.285	-0.049	305.9989
50	6	45	50	4	46	A	0	201032.285	-0.049	305.9989
50	5	45	50	5	46	A	0	201032.285	-0.049	305.9989
50	6	45	50	5	46	A	0	201032.285	-0.049	305.9989
32	8	24	31	8	23	E	1	201032.285	0.192	290.4689
34	19	15	33	19	14	A	0	201067.609	-0.064	243.4843
34	19	16	33	19	15	A	0	201067.609	-0.064	243.4843
34	19	16	33	19	15	E	0	201069.022	0.143	243.4661
50	6	45	50	5	46	E	1	201122.797	0.046	422.2920
50	5	45	50	5	46	E	1	201122.797	0.046	422.2920
50	6	45	50	4	46	E	1	201122.797	0.046	422.2920
50	5	45	50	4	46	E	1	201122.797	0.046	422.2920
33	9	25	32	9	24	E	1	201130.704	0.160	299.0889
34	18	16	33	18	15	E	1	201139.437	0.159	353.0026
34	18	17	33	18	16	E	1	201146.393	0.048	352.4490
34	18	17	33	18	16	A	1	201188.708	0.176	352.4576
34	18	16	33	18	15	A	1	201188.708	0.176	352.4576
15	10	5	14	9	5	E	0	201198.220	0.209	98.3216
34	6	28	33	6	27	A	1	201225.264	0.071	299.7050
15	10	6	14	9	5	A	0	201236.689	0.341	98.3043
15	10	5	14	9	6	A	0	201236.689	0.341	98.3043
15	10	6	14	9	6	E	1	201267.997	-0.230	214.4269
34	18	16	33	18	15	A	0	201282.869	-0.002	236.1923
34	18	17	33	18	16	A	0	201282.869	-0.002	236.1923
34	18	16	33	18	15	E	0	201284.469	0.170	236.1926
34	18	17	33	18	16	E	0	201284.469	0.170	236.1753
34	17	17	33	17	16	E	1	201386.648	0.089	346.1117
34	17	18	33	17	17	E	1	201402.168	0.141	345.5269
34	17	18	33	17	17	A	1	201447.822	0.214	345.6100
34	17	17	33	17	16	A	1	201447.822	0.214	345.6100
36	11	26	35	11	24	E	1	201501.051	0.203	327.3645
34	6	28	33	6	27	E	0	201524.657	0.075	183.0264
34	6	28	33	6	27	A	0	201535.807	-0.026	183.0142
34	17	17	33	17	16	A	0	201538.020	0.036	229.3075
34	17	18	33	17	17	A	0	201538.020	0.036	229.3075
34	17	17	33	17	16	E	0	201539.767	0.120	229.3102
34	17	18	33	17	17	E	0	201539.767	0.120	229.2918
34	6	28	33	6	27	E	1	201569.865	-0.107	299.3182
13	11	3	12	10	3	E	1	201615.357	0.277	212.8861
32	8	24	31	8	23	A	1	201620.081	-0.073	290.8388
21	6	15	20	5	16	A	0	201632.573	-0.230	108.2724
32	8	24	31	8	23	A	0	201648.212	0.106	174.1353
32	8	24	31	8	23	E	0	201654.145	0.116	174.1482
34	16	18	33	16	17	E	1	201683.351	0.051	339.6256
34	16	19	33	16	18	E	1	201709.890	0.136	339.0182
34	16	19	33	16	18	A	1	201758.662	0.113	339.1749
34	16	18	33	16	17	A	1	201758.662	0.113	339.1749
31	16	15	30	16	15	E	1	201768.066	0.303	320.0480
34	16	18	33	16	17	A	0	201844.462	0.014	222.8328
34	16	19	33	16	18	A	0	201844.462	0.014	222.8328
34	16	18	33	16	17	E	0	201846.445	0.065	222.8378
34	16	19	33	16	18	E	0	201846.445	0.065	222.8187
45	2	43	45	1	44	E	0	201858.441	-0.133	243.3393
45	3	43	45	1	44	E	0	201858.441	-0.133	243.3393
45	3	43	45	2	44	E	0	201858.441	-0.133	243.3393
34	15	19	33	15	18	E	1	202045.118	0.119	333.5480
34	15	20	33	15	19	E	1	202086.059	0.056	332.9273
34	15	20	33	15	19	A	1	202137.810	0.101	333.1558
34	15	19	33	15	18	A	1	202137.810	0.101	333.1558
45	3	43	45	2	44	E	1	202167.725	-0.233	359.6053
45	2	43	45	2	44	E	1	202167.725	-0.233	359.6053
45	3	43	45	1	44	E	1	202167.725	-0.233	359.6053
45	2	43	45	1	44	E	1	202167.725	-0.233	359.6053
34	15	19	33	15	18	A	0	202218.475	0.025	216.7721
34	15	20	33	15	19	A	0	202218.475	0.025	216.7721
34	15	19	33	15	18	E	0	202220.201	0.127	216.7793
34	15	20	33	15	19	E	0	202221.475	0.155	216.7598
24	10	15	24	8	16	A	1	202258.778	-0.229	250.5102
18	10	9	17	9	8	E	1	202412.123	-0.027	224.4386
34	14	20	33	14	19	E	1	202494.546	0.169	327.8844
33	31	2	32	31	2	E	1	202517.573	0.506	472.3443
34	14	21	33	14	20	E	1	202555.127	0.031	327.2603
34	14	21	33	14	20	A	1	202609.286	0.075	327.5575
34	14	20	33	14	19	A	1	202609.286	0.075	327.5575
34	14	20	33	14	19	A	0	202683.903	0.070	211.1308
34	14	21	33	14	20	A	0	202683.903	0.070	211.1308
34	14	20	33	14	19	E	0	202685.659	0.140	211.1402
34	14	21	33	14	20	E	0	202687.519	0.123	211.1206
34	13	21	33	13	20	E	1	203066.614	0.060	322.6427
34	13	22	33	13	21	E	1	203155.104	0.115	322.0257
34	13	22	33	13	21	A	1	203209.766	0.012	322.3877
34	13	21	33	13	20	A	1	203211.682	0.076	322.3878
22	7	16	21	6	16	E	1	203241.065	0.402	230.9274
34	13	22	33	13	21	A	0	203276.888	0.041	205.9171
34	13	21	33	13	20	E	0	203279.187	-0.230	205.9286
34	13	22	33	13	21	E	0	203282.280	0.096	205.9092
33	9	24	32	9	23	E	1	203368.238	0.149	299.6214
25	6	19	24	5	19	E	1	203437.607	0.389	244.2007
47	3	44	47	2	45	E	0	203464.918	-0.213	266.2479
47	4	44	47	2	45	E	0	203464.918	-0.213	266.2479
47	4	44	47	3	45	E	0	203464.918	-0.213	266.2479
47	3	44	47	2	45	A	0	203508.058	-0.172	266.2350
47	4	44	47	2	45	A	0	203508.058	-0.172	266.2350
47	3	44	47	3	45	A	0	203508.058	-0.172	266.2350
47	4	44	47	3	45	A	0	203508.058	-0.172	266.2350
34	7	28	33	6	27	E	0	203698.579	-0.122	183.0264
34	7	28	33	6	27	A	0	203720.388	-0.030	183.0142

J'	K'_a	K'_c	J''	K''_a	K''_c	Sym.	$v_{t_{CH_3}}$	$\nu_{Obs.}$	$\nu_{Obs.} - \nu_{Calc.}$	E_{low}
35	26	10	34	26	8	E	1	203737.195	0.051	429.1640
34	12	22	33	12	21	E	1	203820.331	0.090	317.8352
35	6	29	34	7	28	A	1	203825.248	-0.115	306.4840
35	6	29	34	7	28	E	1	203884.105	0.056	306.1173
35	6	29	34	7	28	A	0	203938.708	0.253	189.8096
35	6	29	34	7	28	E	0	203938.708	0.049	189.8210
34	12	23	33	12	22	E	1	203950.165	0.101	317.2366
34	12	23	33	12	22	A	1	203994.067	-0.019	317.6581
34	12	22	33	12	21	A	1	204017.681	0.018	317.6588
34	12	23	33	12	22	A	0	204051.930	0.122	201.1431
34	12	22	33	12	21	E	0	204065.079	0.053	201.1568
31	7	25	31	5	27	E	0	204067.916	0.301	163.4795
34	12	22	33	12	21	A	0	204073.614	0.029	201.1438
34	8	27	33	8	26	A	1	204169.957	-0.033	303.0490
34	8	27	33	8	26	E	0	204353.699	0.041	186.3946
34	8	27	33	8	26	A	0	204357.288	0.046	186.3835
34	8	27	33	8	26	E	1	204454.459	0.079	302.6801
34	25	9	33	25	9	E	1	204607.249	-0.326	412.2671
33	7	26	32	7	25	A	1	204619.408	0.067	295.6412
33	9	24	32	9	23	A	0	204725.660	0.120	183.1788
33	9	24	32	9	23	E	0	204735.843	0.066	183.1898
49	4	45	49	3	46	E	0	204801.878	0.186	290.1908
49	5	45	49	3	46	E	0	204801.878	0.186	290.1908
49	5	45	49	4	46	E	0	204801.878	0.186	290.1908
33	9	24	32	9	23	A	1	204807.291	-0.065	299.8363
49	4	45	49	3	46	A	0	204840.538	0.169	290.1796
49	5	45	49	3	46	A	0	204840.538	0.169	290.1796
49	5	45	49	4	46	A	0	204840.538	0.169	290.1796
34	11	23	33	11	22	E	1	204869.075	0.098	313.4816
36	5	31	35	6	30	A	1	204897.394	0.061	309.4523
37	4	33	36	5	32	A	1	204919.882	-0.102	311.8995
37	5	33	36	5	32	A	1	204922.345	-0.041	311.8995
37	4	33	36	4	32	A	1	204924.332	-0.052	311.8994
37	5	33	36	4	32	A	1	204926.736	-0.049	311.8994
33	7	26	32	7	25	E	0	204958.057	-0.009	178.9425
33	7	26	32	7	25	E	1	204960.533	-0.189	295.2198
36	6	31	35	6	30	A	1	204966.001	-0.011	309.4523
33	7	26	32	7	25	A	0	204970.535	-0.001	178.9290
49	5	45	49	4	46	E	1	204991.575	0.088	406.4692
49	4	45	49	4	46	E	1	204991.575	0.088	406.4692
49	5	45	49	3	46	E	1	204991.575	0.088	406.4692
49	4	45	49	3	46	E	1	204991.575	0.088	406.4692
34	11	24	33	11	23	A	1	205003.720	0.062	313.3858
38	3	35	37	3	34	A	1	205012.179	-0.067	313.8675
38	4	35	37	3	34	A	1	205012.179	-0.067	313.8675
38	3	35	37	4	34	A	1	205012.179	-0.067	313.8675
38	4	35	37	4	34	A	1	205012.179	-0.067	313.8675
36	5	31	35	5	30	A	1	205016.312	-0.019	309.4484
34	11	24	33	11	23	A	0	205052.789	0.091	196.8265
34	11	24	33	11	23	E	1	205064.426	0.128	312.9148
35	7	29	34	7	28	A	1	205075.777	0.007	306.4840
37	4	33	36	5	32	E	1	205084.965	-0.028	311.5140
36	6	31	35	5	30	A	1	205084.980	-0.030	309.4484
37	5	33	36	5	32	E	1	205087.893	-0.013	311.5140
37	4	33	36	4	32	E	1	205090.253	-0.033	311.5138
37	5	33	36	4	32	E	1	205093.102	-0.096	311.5138
36	5	31	35	6	30	E	0	205099.557	-0.030	192.7821
37	4	33	36	5	32	E	0	205101.791	-0.080	195.2166
37	5	33	36	5	32	E	0	205104.548	-0.041	195.2166
37	4	33	36	5	32	A	0	205107.003	0.051	195.2045
37	4	33	36	4	32	E	0	205107.003	-0.043	195.2164
34	11	24	33	11	23	E	0	205107.003	-0.043	196.8270
37	5	33	36	5	32	A	0	205109.577	-0.112	195.2045
37	5	33	36	4	32	E	0	205109.577	0.030	195.2164
37	4	33	36	4	32	A	0	205111.884	-0.059	195.2043
37	5	33	36	4	32	A	0	205114.712	0.030	195.2043
38	3	35	37	3	34	E	0	205167.663	-0.002	197.1657
38	4	35	37	3	34	E	0	205167.663	-0.002	197.1657
38	3	35	37	4	34	E	0	205167.663	-0.002	197.1657
38	4	35	37	4	34	E	0	205167.663	-0.002	197.1657
38	3	35	37	3	34	A	0	205171.616	-0.020	197.1529
38	4	35	37	3	34	A	0	205171.616	-0.020	197.1529
38	3	35	37	4	34	A	0	205171.616	-0.020	197.1529
38	4	35	37	4	34	A	0	205171.616	-0.020	197.1529
39	2	37	38	2	36	A	1	205174.178	-0.080	315.3915
39	3	37	38	2	36	A	1	205174.178	-0.080	315.3915
39	2	37	38	3	36	A	1	205174.178	-0.080	315.3915
39	3	37	38	3	36	A	1	205174.178	-0.080	315.3915
36	6	31	35	6	30	E	0	205175.950	0.038	192.7821
36	6	31	35	6	30	A	0	205182.219	0.019	192.7705
34	11	23	33	11	22	E	0	205222.796	0.072	196.8468
37	18	19	37	17	20	A	0	205228.917	0.203	257.4142
37	18	20	37	17	21	A	0	205228.917	0.203	257.4142
36	5	31	35	5	30	E	1	205228.917	0.029	309.0762
36	5	31	35	5	30	E	0	205231.447	0.210	192.7777
34	11	23	33	11	22	A	1	205234.677	0.012	313.3949
36	5	31	35	5	30	A	0	205237.766	-0.068	192.7661
34	11	23	33	11	22	A	0	205267.792	0.006	196.8350
39	3	37	38	3	36	E	1	205267.792	0.034	314.9504
39	2	37	38	3	36	E	1	205267.792	0.034	314.9504
39	3	37	38	2	36	E	1	205267.792	0.034	314.9504
39	2	37	38	2	36	E	1	205267.792	0.034	314.9504
39	2	37	38	2	36	E	0	205302.752	0.032	198.6649
39	3	37	38	2	36	E	0	205302.752	0.032	198.6649
39	2	37	38	3	36	E	0	205302.752	0.032	198.6649
39	3	37	38	3	36	E	0	205302.752	0.032	198.6649
35	7	29	34	7	28	E	0	205302.752	0.032	189.8210
39	2	37	38	2	36	A	0	205305.708	-0.045	198.6511
39	3	37	38	2	36	A	0	205305.708	-0.045	198.6511
39	2	37	38	3	36	A	0	205305.708	-0.045	198.6511
39	3	37	38	3	36	A	0	205305.708	-0.045	198.6511
35	7	29	34	7	28	A	0	205308.783	-0.031	189.8096
35	7	29	34	7	28	E	1	205310.207	0.074	306.1173
36	6	31	35	5	30	A	0	205314.516	-0.116	192.7661
40	1	39	39	1	38	A	1	205372.078	-0.105	316.4944
40	2	39	39	1	38	A	1	205372.078	-0.105	316.4944
40	1	39	39	2	38	A	1	205372.078	-0.105	316.4944
40	2	39	39	2	38	A	1	205372.078	-0.105	316.4944
40	2	39	39	2	38	E	1	205428.391	-0.026	316.0123
40	1	39	39	2	38	E	1	205428.391	-0.026	316.0123
40	2	39	39	1	38	E	1	205428.391	-0.026	316.0123
40	1	39	39	1	38	E	1	205428.391	-0.026	316.0123
40	1	39	39	1	38	E	0	205472.529	-0.082	199.7368
40	2	39	39	1	38	E	0	205472.529	-0.082	199.7368
40	1	39	39	2	38	E	0	205472.529	-0.082	199.7368
40	2	39	39	2	38	E	0	205472.529	-0.082	199.7368

J'	K'_a	K'_c	J''	K''_a	K''_c	Sym.	$v_{t_{CH_3}}$	$\nu_{Obs.}$	$\nu_{Obs.} - \nu_{Calc.}$	E_{low}
40	1	39	39	1	38	A	0	205474.323	-0.006	199.7217
40	2	39	39	1	38	A	0	205474.323	-0.006	199.7217
40	1	39	39	2	38	A	0	205474.323	-0.006	199.7217
40	2	39	39	2	38	A	0	205474.323	-0.006	199.7217
36	18	18	36	17	19	E	0	205490.009	0.122	250.0858
20	8	12	19	7	12	E	0	205507.040	-0.069	108.9376
18	9	10	17	8	9	A	1	205527.839	-0.013	221.1470
18	9	9	17	8	10	A	1	205532.710	-0.030	221.1468
41	0	41	40	0	40	A	1	205587.421	0.028	317.1918
41	1	41	40	0	40	A	1	205587.421	0.028	317.1918
41	0	41	40	1	40	A	1	205587.421	0.028	317.1918
41	1	41	40	1	40	A	1	205587.421	0.028	317.1918
41	1	41	40	1	40	E	1	205602.982	-0.035	316.6595
41	0	41	40	1	40	E	1	205602.982	-0.035	316.6595
41	1	41	40	0	40	E	1	205602.982	-0.035	316.6595
41	0	41	40	0	40	A	0	205657.367	-0.240	200.3803
41	1	41	40	0	40	A	0	205657.367	-0.240	200.3803
41	0	41	40	1	40	A	0	205657.367	-0.240	200.3803
41	1	41	40	1	40	A	0	205657.367	-0.240	200.3803
41	0	41	40	0	40	E	0	205657.367	0.235	200.3969
41	1	41	40	0	40	E	0	205657.367	0.235	200.3969
41	0	41	40	1	40	E	0	205657.367	0.235	200.3969
41	1	41	40	1	40	E	0	205657.367	0.235	200.3969
35	30	5	34	30	4	E	1	205708.129	-0.621	473.6377
35	30	5	34	30	4	A	1	205713.310	-0.284	473.0127
35	30	6	34	30	5	A	1	205713.310	-0.284	473.0127
35	29	6	34	29	5	E	1	205769.498	-0.158	461.9074
35	29	6	34	29	5	A	1	205777.029	-0.200	461.3000
35	29	7	34	29	6	A	1	205777.029	-0.200	461.3000
35	29	7	34	29	6	E	1	205782.938	-0.153	461.7586
35	6	29	34	6	28	A	1	205828.998	-0.022	306.4172
35	30	5	34	30	4	A	0	205836.492	-0.322	356.8692
35	30	6	34	30	5	A	0	205836.492	-0.322	356.8692
35	28	8	34	28	7	A	1	205846.867	-0.029	449.9906
35	28	7	34	28	6	A	1	205846.867	-0.029	449.9906
35	29	6	34	29	5	A	0	205899.157	-0.239	345.1743
35	29	7	34	29	6	A	0	205899.157	-0.239	345.1743
35	27	9	34	27	8	A	1	205923.561	0.031	439.0846
35	27	8	34	27	7	A	1	205923.561	0.031	439.0846
35	27	8	34	27	7	E	1	205925.275	0.169	439.6323
35	28	7	34	28	6	A	0	205967.694	-0.117	333.8774
35	28	8	34	28	7	A	0	205967.694	-0.117	333.8774
35	28	8	34	28	7	E	0	205967.694	-0.429	333.8591
35	26	10	34	26	9	E	1	205991.348	-0.079	429.0888
35	26	9	34	26	8	A	1	206008.411	0.164	428.5827
35	26	10	34	26	9	A	1	206008.411	0.164	428.5827
34	10	25	33	10	24	A	1	206037.682	-0.033	309.5836
35	27	8	34	27	7	A	0	206042.899	-0.086	322.9788
35	27	9	34	27	8	A	0	206042.899	-0.086	322.9788
35	27	9	34	27	8	E	0	206042.899	-0.022	322.9616
34	10	25	33	10	24	A	0	206095.239	0.124	192.9815
35	25	10	34	25	9	A	1	206102.430	0.025	418.4851
35	6	29	34	6	28	E	0	206112.682	-0.096	189.7485
35	6	29	34	6	28	A	0	206123.005	-0.034	189.7367
35	26	9	34	26	8	A	0	206126.014	-0.021	312.4789
35	26	10	34	26	9	A	0	206126.014	-0.021	312.4789
35	26	10	34	26	9	E	0	206126.014	-0.050	312.4606
35	6	29	34	6	28	E	1	206147.529	-0.085	306.0418
34	9	26	33	9	25	A	1	206171.954	-0.005	306.2043
35	24	12	34	24	11	E	1	206184.091	0.134	409.1956
35	24	11	34	24	10	A	1	206207.696	0.042	408.7923
35	24	12	34	24	11	A	1	206207.696	0.042	408.7923
35	25	10	34	25	9	A	0	206218.406	0.096	302.3781
35	25	11	34	25	10	A	0	206218.406	0.096	302.3781
34	9	26	33	9	25	A	0	206282.187	0.029	189.5651
34	9	26	33	9	25	E	0	206284.271	0.004	189.5749
35	23	13	34	23	12	E	1	206298.881	0.144	399.8480
35	23	12	34	23	11	E	1	206311.986	0.081	400.1378
35	24	11	34	24	10	A	0	206321.579	0.125	292.6769
35	24	12	34	24	11	A	0	206321.579	0.125	292.6769
35	24	12	34	24	11	E	0	206321.579	-0.145	292.6573
35	23	13	34	23	12	A	1	206326.196	0.163	399.5047
35	23	12	34	23	11	A	1	206326.196	0.163	399.5047
30	11	20	30	9	21	E	1	206395.608	-0.211	286.7892
35	22	14	34	22	13	E	1	206429.256	0.159	390.9014
35	23	12	34	23	11	A	0	206437.694	0.201	283.3759
35	23	13	34	23	12	A	0	206437.694	0.201	283.3759
35	23	13	34	23	12	E	0	206437.694	-0.224	283.3560
35	22	13	34	22	12	E	1	206440.339	0.208	391.2558
35	22	13	34	22	12	A	1	206460.254	0.183	390.6229
35	22	14	34	22	13	A	1	206460.254	0.183	390.6229
35	21	15	34	21	14	E	1	206578.238	-0.007	382.3573
35	21	14	34	21	13	E	1	206586.355	0.111	382.7712
34	10	24	33	10	23	E	1	206589.708	0.122	309.6217
18	9	10	17	8	10	E	0	206598.793	0.004	104.4453
20	5	15	19	4	16	A	0	206600.789	-0.046	102.2106
35	21	15	34	21	14	A	1	206613.125	0.169	382.1474
35	21	14	34	21	13	A	1	206613.125	0.169	382.1474
18	9	9	17	8	9	E	0	206638.421	-0.253	104.4611
18	9	10	17	8	9	A	0	206667.353	0.168	104.4428
18	9	9	17	8	10	A	0	206671.361	-0.412	104.4426
34	10	25	33	10	24	E	1	206671.361	0.287	309.0998
35	21	14	34	21	13	A	0	206719.173	0.187	265.9776
35	21	15	34	21	14	A	0	206719.173	0.187	265.9776
35	20	16	34	20	15	E	1	206750.330	0.057	374.2174
35	20	15	34	20	14	E	1	206754.264	0.114	374.6848
35	20	15	34	20	14	A	1	206788.858	0.107	374.0791
35	20	16	34	20	15	A	1	206788.858	0.107	374.0791
35	20	15	34	20	14	A	0	206891.669	0.023	257.8822
35	20	16	34	20	15	A	0	206891.669	0.023	257.8822
35	20	16	34	20	15	E	0	206892.923	0.218	257.8632
35	19	16	34	19	15	E	1	206948.898	-0.001	366.9979
35	19	17	34	19	16	E	1	206950.566	0.096	366.4837
35	19	17	34	19	16	A	1	206992.857	0.145	366.4190
35	19	16	34	19	15	A	1	206992.857	0.145	366.4190
34	9	26	33	9	25	E	1	207019.374	0.141	305.7979
16	10	6	15	9	6	E	0	207045.190	0.391	101.2781
16	10	7	15	9	6	A	0	207083.370	0.299	101.2608
16	10	6	15	9	7	A	0	207083.370	0.299	101.2608
35	19	16	34	19	15	A	0	207092.135	-0.013	250.1912
35	19	17	34	19	16	A	0	207092.135	-0.013	250.1912
35	19	17	34	19	16	E	0	207093.682	0.189	250.1731
16	10	7	15	9	7	E	1	207109.734	0.344	217.3818
35	18	17	34	18	16	E	1	207177.340	0.218	359.7119
35	18	18	34	18	17	E	1	207185.924	0.118	359.1586
35	18	17	34	18	16	A	1	207232.013	0.244	359.1685

J'	K_a'	K_c'	J''	K_a''	K_c''	Sym.	$v_{t_{\mathrm{CH_3}}}$	$\nu_{\mathrm{Obs.}}$	$\nu_{\mathrm{Obs.}} - \nu_{\mathrm{Calc.}}$	E_{low}
35	18	18	34	18	17	A	1	207232.013	0.244	359.1685
35	18	17	34	18	16	A	0	207327.355	-0.015	242.9064
35	18	18	34	18	17	A	0	207327.355	-0.015	242.9064
35	18	17	34	18	16	E	0	207329.031	0.091	242.9067
35	18	18	34	18	17	E	0	207329.031	0.091	242.8894
48	12	36	47	13	35	A	1	207402.717	0.462	436.5896
35	17	18	34	17	17	E	1	207447.758	0.035	352.8293
35	17	19	34	17	18	E	1	207465.748	0.087	352.2449
27	18	10	27	17	11	E	0	207469.025	-0.317	193.1848
35	7	29	34	6	28	E	0	207476.078	-0.097	189.7485
14	11	4	13	10	4	E	1	207482.273	-0.451	215.4389
35	7	29	34	6	28	A	0	207493.258	-0.141	189.7367
35	17	19	34	17	18	A	1	207515.429	0.188	352.3296
35	17	18	34	17	17	A	1	207515.429	0.188	352.3296
35	7	29	34	6	28	E	1	207573.558	-0.139	306.0418
32	11	22	32	9	23	A	1	207578.893	-0.016	299.8363
34	10	24	33	10	23	E	0	207605.050	-0.063	193.0659
34	10	24	33	10	23	A	0	207606.804	-0.182	193.0566
35	17	18	34	17	17	A	0	207606.811	0.258	236.0301
35	17	19	34	17	18	A	0	207606.811	0.258	236.0301
35	17	18	34	17	17	E	0	207608.590	0.211	236.0328
35	17	19	34	17	18	E	0	207608.590	0.211	236.0145
34	10	24	33	10	23	A	1	207639.083	-0.174	309.6636
18	6	12	17	4	13	E	1	207641.758	0.418	212.2720
25	18	7	25	17	8	E	0	207678.155	0.036	182.7699
22	3	20	21	1	20	E	0	207731.938	-0.177	104.6376
35	16	19	34	16	18	E	1	207773.022	0.081	346.3530
32	16	16	31	16	16	E	1	207799.597	0.232	326.1694
35	16	20	34	16	19	E	1	207803.196	0.208	345.7465
35	16	19	34	16	18	A	1	207856.104	0.107	345.9049
35	16	20	34	16	19	A	1	207856.104	0.107	345.9049
35	16	19	34	16	18	A	0	207942.494	0.046	229.5656
35	16	20	34	16	19	A	0	207942.494	0.046	229.5656
35	16	20	34	16	19	E	0	207944.887	-0.126	229.5516
33	8	25	32	8	24	E	1	207947.921	-0.016	297.1746
35	15	20	34	15	19	E	1	208170.298	0.138	340.2875
35	15	21	34	15	20	E	1	208216.365	0.087	339.6682
48	3	45	48	2	46	E	0	208258.494	-0.126	274.5834
48	4	45	48	2	46	E	0	208258.494	-0.126	274.5834
48	4	45	48	3	46	E	0	208258.494	-0.126	274.5834
35	15	21	34	15	20	A	1	208272.528	0.126	339.8984
35	15	20	34	15	19	A	1	208272.528	0.126	339.8984
33	8	25	32	8	24	A	1	208286.968	-0.080	297.5642
35	15	20	34	15	19	A	0	208353.317	0.092	223.5174
35	15	21	34	15	20	A	0	208353.317	0.092	223.5174
35	15	20	34	15	19	E	0	208355.072	0.087	223.5247
35	15	21	34	15	20	E	0	208356.539	0.149	223.5052
33	8	25	32	8	24	A	0	208381.333	0.067	180.8615
33	8	25	32	8	24	E	0	208383.955	0.081	180.8747
22	7	15	21	6	16	E	0	208401.815	-0.159	114.6876
48	4	45	48	3	46	E	1	208514.543	0.047	390.8548
48	3	45	48	3	46	E	1	208514.543	0.047	390.8548
48	4	45	48	2	46	E	1	208514.543	0.047	390.8548
48	3	45	48	2	46	E	1	208514.543	0.047	390.8548
36	7	29	35	8	28	A	0	208581.560	0.073	200.1956
35	14	21	34	14	20	E	1	208665.189	0.084	334.6389
35	14	22	34	14	21	E	1	208733.316	0.215	334.0168
35	14	22	34	14	21	A	1	208791.917	0.095	334.3158
35	14	21	34	14	20	A	1	208791.917	0.095	334.3158
38	18	21	37	18	19	E	1	208814.605	-0.576	381.0497
36	7	29	35	8	28	A	1	208836.966	-0.191	316.8480
35	14	21	34	14	20	A	0	208865.958	0.046	217.8916
35	14	22	34	14	21	A	0	208865.958	0.046	217.8916
35	14	21	34	14	20	E	0	208867.843	0.104	217.9011
35	14	22	34	14	21	E	0	208869.911	0.065	217.8815
34	7	27	33	7	26	A	1	209064.444	0.085	302.4665
36	6	30	35	7	29	A	1	209269.366	-0.132	313.3246
23	10	13	23	8	15	E	1	209286.604	0.268	245.5654
35	13	22	34	13	21	E	1	209298.254	0.132	329.4163
36	6	30	35	7	29	E	1	209390.002	0.143	312.9658
35	13	23	34	13	22	E	1	209397.391	0.237	328.8022
36	6	30	35	7	29	E	0	209427.447	-0.053	196.6692
36	6	30	35	7	29	A	0	209429.926	-0.040	196.6579
34	7	27	33	7	26	E	0	209437.548	-0.072	185.7791
34	7	27	33	7	26	A	0	209452.257	-0.023	185.7661
35	13	23	34	13	22	A	1	209455.769	0.073	329.1660
35	13	22	34	13	21	A	1	209459.537	0.036	329.1662
34	7	27	33	7	26	E	1	209485.788	-0.103	302.0565
35	8	28	34	8	27	A	1	209511.023	-0.019	309.8594
21	8	14	20	7	13	A	1	209514.263	0.129	229.6725
35	13	23	34	13	22	A	0	209521.480	0.020	212.6977
35	13	22	34	13	21	A	0	209525.054	0.095	212.6978
35	13	22	34	13	21	E	0	209525.054	-0.031	212.7092
35	13	23	34	13	22	E	0	209528.201	0.038	212.6899
50	4	46	50	3	47	E	0	209617.769	0.076	299.0166
50	5	46	50	3	47	E	0	209617.769	0.076	299.0166
50	5	46	50	4	47	E	0	209617.769	0.076	299.0166
50	4	46	50	3	47	A	0	209657.416	0.152	299.0055
50	5	46	50	3	47	A	0	209657.416	0.152	299.0055
50	5	46	50	4	47	A	0	209657.416	0.152	299.0055
35	8	28	34	8	27	E	0	209713.302	-0.040	193.2111
35	8	28	34	8	27	A	0	209718.003	-0.004	193.2001
35	8	28	34	8	27	E	1	209769.278	0.084	309.4999
37	5	32	36	6	31	A	1	209850.384	-0.162	316.2893
38	5	34	37	5	33	A	1	209862.832	-0.185	318.7350
38	4	34	37	4	33	A	1	209864.105	-0.011	318.7349
37	6	32	36	6	31	A	1	209889.774	-0.049	316.2893
37	5	32	36	5	31	A	1	209919.165	-0.060	316.2870
37	6	32	36	5	31	A	1	209958.329	-0.173	316.2870
39	3	36	38	3	35	A	1	209961.892	-0.151	320.7060
39	4	36	38	3	35	A	1	209961.892	-0.151	320.7060
39	3	36	38	4	35	A	1	209961.892	-0.151	320.7060
39	4	36	38	4	35	A	1	209961.892	-0.151	320.7060
38	5	34	37	5	33	E	1	210027.004	-0.030	318.3550
38	4	34	37	4	33	E	1	210028.502	0.149	318.3549
38	5	34	37	4	33	E	1	210030.103	0.157	318.3549
36	7	30	35	7	29	A	1	210037.533	-0.015	313.3246
38	4	34	37	5	33	E	0	210044.078	-0.316	202.0581
38	5	34	37	5	33	E	0	210045.727	-0.148	202.0581
37	5	32	36	6	31	E	1	210045.734	-0.135	315.9246
38	4	34	37	4	33	E	0	210047.095	-0.017	202.0581
38	5	34	37	5	33	A	0	210050.802	-0.129	202.0462
38	4	34	37	4	33	A	0	210052.160	-0.015	202.0461
38	5	34	37	4	33	A	0	210053.819	0.151	202.0461
37	5	32	36	6	31	E	0	210056.113	-0.238	199.6261
39	4	36	38	4	35	E	1	210090.517	0.114	320.3014

J'	K_a'	K_c'	J''	K_a''	K_c''	Sym.	$v_{t_{\mathrm{CH_3}}}$	$\nu_{\mathrm{Obs.}}$	$\nu_{\mathrm{Obs.}} - \nu_{\mathrm{Calc.}}$	E_{low}
39	3	36	38	4	35	E	1	210090.517	0.114	320.3014
39	4	36	38	3	35	E	1	210090.517	0.114	320.3014
39	3	36	38	3	35	E	1	210090.517	0.114	320.3014
37	6	32	36	6	31	E	1	210092.641	0.120	315.9246
37	6	32	36	6	31	E	0	210100.245	0.046	199.6261
37	6	32	36	6	31	A	0	210106.453	0.014	199.6146
39	3	36	38	3	35	E	0	210118.400	-0.115	204.0094
39	4	36	38	3	35	E	0	210118.400	-0.115	204.0094
39	3	36	38	4	35	E	0	210118.400	-0.115	204.0094
39	4	36	38	4	35	E	0	210118.400	-0.115	204.0094
39	3	36	38	3	35	A	0	210122.374	-0.088	203.9967
39	4	36	38	3	35	A	0	210122.374	-0.088	203.9967
39	3	36	38	4	35	A	0	210122.374	-0.088	203.9967
39	4	36	38	4	35	A	0	210122.374	-0.088	203.9967
37	5	32	36	5	31	E	1	210127.300	0.534	315.9219
40	2	38	39	2	37	A	1	210127.308	-0.252	322.2354
40	3	38	39	2	37	A	1	210127.308	-0.252	322.2354
40	2	38	39	3	37	A	1	210127.308	-0.252	322.2354
40	3	38	39	3	37	A	1	210127.308	-0.252	322.2354
37	5	32	36	5	31	E	0	210132.584	-0.092	199.6235
37	5	32	36	5	31	A	0	210138.890	-0.216	199.6121
37	6	32	36	5	31	E	1	210173.448	0.031	315.9219
37	6	32	36	5	31	E	0	210176.551	0.028	199.6235
40	3	38	39	3	37	E	1	210220.587	-0.099	321.7974
40	2	38	39	3	37	E	1	210220.587	-0.099	321.7974
40	3	38	39	2	37	E	1	210220.587	-0.099	321.7974
40	2	38	39	2	37	E	1	210220.587	-0.099	321.7974
40	2	38	39	2	37	E	0	210257.459	0.004	205.5130
40	3	38	39	2	37	E	0	210257.459	0.004	205.5130
40	2	38	39	3	37	E	0	210257.459	0.004	205.5130
40	3	38	39	3	37	E	0	210257.459	0.004	205.5130
40	2	38	39	2	37	A	0	210260.342	0.033	205.4993
40	3	38	39	2	37	A	0	210260.342	0.033	205.4993
40	2	38	39	3	37	A	0	210260.342	0.033	205.4993
40	3	38	39	3	37	A	0	210260.342	0.033	205.4993
36	7	30	35	7	29	E	0	210268.921	0.031	196.6692
36	7	30	35	7	29	E	1	210273.783	-0.022	312.9658
36	7	30	35	7	29	A	0	210275.942	0.052	196.6579
35	12	24	34	12	23	E	1	210284.707	0.254	324.0397
35	12	24	34	12	23	A	1	210322.861	0.073	324.4626
41	1	40	40	1	39	A	1	210326.597	-0.061	323.3449
41	2	40	40	1	39	A	1	210326.597	-0.061	323.3449
41	1	40	40	2	39	A	1	210326.597	-0.061	323.3449
41	2	40	40	2	39	A	1	210326.597	-0.061	323.3449
35	12	23	34	12	22	A	1	210367.396	-0.079	324.4641
35	12	24	34	12	23	A	0	210378.467	0.039	207.9495
41	2	40	40	2	39	E	1	210382.587	-0.128	322.8647
41	1	40	40	2	39	E	1	210382.587	-0.128	322.8647
41	2	40	40	1	39	E	1	210382.587	-0.128	322.8647
41	1	40	40	1	39	E	1	210382.587	-0.128	322.8647
35	12	23	34	12	22	E	0	210403.305	0.063	207.9637
35	12	24	34	12	23	E	0	210403.305	0.063	207.9449
35	12	23	34	12	22	A	0	210419.842	0.097	207.9510
41	1	40	40	1	39	E	0	210428.392	-0.040	206.5906
41	2	40	40	1	39	E	0	210428.392	-0.040	206.5906
41	1	40	40	2	39	E	0	210428.392	-0.040	206.5906
41	2	40	40	2	39	E	0	210428.392	-0.040	206.5906
41	1	40	40	1	39	A	0	210430.201	0.057	206.5756
41	2	40	40	1	39	A	0	210430.201	0.057	206.5756
41	1	40	40	2	39	A	0	210430.201	0.057	206.5756
41	2	40	40	2	39	A	0	210430.201	0.057	206.5756
36	6	30	35	6	29	A	1	210519.987	0.082	313.2829
42	0	42	41	0	41	A	1	210542.126	-0.113	324.0495
42	1	42	41	0	41	A	1	210542.126	-0.113	324.0495
42	0	42	41	1	41	A	1	210542.126	-0.113	324.0495
42	1	42	41	1	41	A	1	210542.126	-0.113	324.0495
42	1	42	41	1	41	E	1	210557.597	-0.136	323.5177
42	1	42	41	0	41	E	1	210557.597	-0.136	323.5177
42	0	42	41	0	41	E	1	210557.597	-0.136	323.5177
42	0	42	41	0	41	A	0	210613.540	-0.231	207.2403
42	1	42	41	0	41	A	0	210613.540	-0.231	207.2403
42	0	42	41	1	41	A	0	210613.540	-0.231	207.2403
42	1	42	41	1	41	A	0	210613.540	-0.231	207.2403
42	0	42	41	0	41	E	0	210613.540	0.240	207.2569
42	1	42	41	0	41	E	0	210613.540	0.240	207.2569
42	0	42	41	1	41	E	0	210613.540	0.240	207.2569
42	1	42	41	1	41	E	0	210613.540	0.240	207.2569
20	5	15	19	3	16	E	1	210688.409	0.674	218.3999
36	6	30	35	6	29	E	0	210790.897	0.001	196.6237
36	6	30	35	6	29	A	0	210800.200	-0.125	196.6122
21	8	13	20	7	13	E	0	210816.279	0.077	112.9513
36	6	30	35	6	29	E	1	210816.279	0.337	312.9182
40	9	31	39	10	30	E	0	210884.002	-0.568	237.3619
34	9	25	33	9	24	E	1	211087.324	0.232	306.4050
19	9	11	18	8	10	A	1	211257.593	0.459	224.7174
19	9	10	18	8	11	A	1	211269.992	0.326	224.7170
35	11	24	34	11	23	E	1	211329.047	0.081	320.3153
35	11	25	34	11	24	A	1	211408.587	0.007	320.2239
35	11	25	34	11	24	A	0	211457.218	0.129	203.6663
21	8	13	20	7	14	A	0	211490.927	-0.257	112.9121
35	11	25	34	11	24	E	0	211506.281	0.030	203.6686
35	11	25	34	11	24	E	1	211545.017	0.192	319.7550
36	31	6	35	31	5	A	1	211572.657	-0.237	491.9879
36	31	5	35	31	4	A	1	211572.657	-0.237	491.9879
36	31	6	35	31	5	E	1	211581.273	0.011	492.3332
36	7	30	35	6	29	E	0	211632.347	0.060	196.6237
36	30	6	35	30	5	A	1	211635.894	-0.125	479.8745
36	30	7	35	30	6	A	1	211635.894	-0.125	479.8745
36	7	30	35	6	29	A	0	211646.301	0.051	196.6122
36	7	30	35	6	29	E	1	211699.942	0.054	312.9182
36	29	8	35	29	7	A	1	211704.648	-0.223	468.1640
36	29	7	35	29	6	A	1	211704.648	-0.223	468.1640
47	3	45	47	2	46	E	1	211737.140	-0.150	375.4582
47	2	45	47	2	46	E	1	211737.140	-0.150	375.4582
47	3	45	47	1	46	E	1	211737.140	-0.150	375.4582
47	2	45	47	1	46	E	1	211737.140	-0.150	375.4582
36	30	6	35	30	5	A	0	211762.241	-0.340	363.7352
36	30	7	35	30	6	A	0	211762.241	-0.340	363.7352
36	28	8	35	28	7	A	1	211780.295	-0.007	456.8569
36	28	9	35	28	8	A	1	211780.295	-0.007	456.8569
35	11	24	34	11	23	E	0	211791.701	0.034	203.6923
35	11	24	34	11	23	A	1	211808.493	0.043	320.2408
35	11	24	34	11	23	A	0	211830.104	-0.094	203.6820
36	29	8	35	29	7	A	0	211830.104	-0.094	352.0424
36	27	10	35	27	9	E	1	211847.731	-0.070	446.5005
36	27	10	35	27	9	A	1	211863.361	0.032	445.9535

J'	K'_a	K'_c	J''	K''_a	K''_c	Sym.	$v_{t_{CH_3}}$	$\nu_{Obs.}$	$\nu_{Obs.} - \nu_{Calc.}$	E_{low}
36	27	9	35	27	8	A	1	211863.361	0.032	445.9535
36	27	9	35	27	9	E	1	211883.728	-0.190	446.5005
36	28	8	35	28	7	A	0	211904.209	-0.125	340.7478
36	28	9	35	28	8	A	0	211904.209	-0.125	340.7478
36	26	11	35	26	10	E	1	211936.196	0.001	435.9599
36	26	10	35	26	9	A	1	211955.342	0.166	435.4544
36	26	11	35	26	10	A	1	211955.342	0.166	435.4544
36	27	9	35	27	8	A	0	211985.764	-0.010	329.8517
36	27	10	35	27	9	A	0	211985.764	-0.010	329.8517
36	27	10	35	27	9	E	0	211985.764	0.030	329.8344
36	25	12	35	25	11	E	1	212034.921	0.175	425.8171
36	25	11	35	25	10	E	1	212051.051	0.055	425.9668
36	25	11	35	25	10	A	1	212057.403	0.080	425.3599
36	25	12	35	25	11	A	1	212057.403	0.080	425.3599
36	26	10	35	26	9	A	0	212075.830	0.016	319.3546
36	26	11	35	26	10	A	0	212075.830	0.016	319.3546
36	26	11	35	26	10	E	0	212075.830	-0.045	319.3362
35	9	27	34	9	26	A	1	212092.311	-0.025	313.0815
36	24	13	35	24	12	E	1	212145.287	0.007	416.0731
36	24	12	35	24	11	E	1	212160.866	0.233	416.2947
36	24	12	35	24	11	A	1	212171.740	0.164	415.6706
36	24	13	35	24	12	A	1	212171.740	0.164	415.6706
36	25	11	35	25	10	A	0	212176.008	0.083	309.2568
36	25	12	35	25	11	A	0	212176.008	0.083	309.2568
36	25	12	35	25	11	E	0	212176.008	-0.099	309.2377
35	9	27	34	9	26	A	0	212224.336	0.388	196.4459
35	9	27	34	9	26	E	0	212224.336	-0.194	196.4558
19	9	11	18	8	11	E	1	212252.719	0.527	224.1334
34	9	25	33	9	24	A	0	212267.023	0.056	190.0077
36	23	14	35	23	13	E	1	212270.097	0.054	406.7293
34	9	25	33	9	24	E	0	212278.198	0.058	190.0190
36	23	13	35	23	12	E	1	212283.817	0.014	407.0196
36	24	12	35	24	11	A	0	212288.080	0.174	299.5591
36	24	13	35	24	12	A	0	212288.080	0.174	299.5591
36	24	13	35	24	12	E	0	212288.080	-0.152	299.5394
36	23	14	35	23	13	A	1	212300.353	0.190	406.3870
36	23	13	35	23	12	A	1	212300.353	0.190	406.3870
21	8	13	20	7	13	E	1	212322.032	0.691	229.4856
19	9	11	18	8	11	E	0	212336.321	0.092	108.0171
34	9	25	33	9	24	A	1	212341.534	-0.019	306.6680
35	10	26	34	10	25	A	1	212370.829	-0.006	316.4563
19	9	10	18	8	10	E	0	212376.559	0.028	108.0328
19	9	11	18	8	10	A	0	212401.303	-0.023	108.0146
36	23	13	35	23	12	A	0	212414.277	0.304	290.2619
36	23	14	35	23	13	A	0	212414.277	0.304	290.2619
36	23	14	35	23	13	E	0	212414.277	-0.190	290.2420
36	22	14	35	22	13	E	1	212423.343	0.169	398.1419
35	10	26	34	10	25	A	0	212437.189	0.115	199.8561
35	10	26	34	10	25	E	0	212447.022	0.080	199.8639
36	21	16	35	21	15	E	1	212574.267	0.111	389.2480
36	21	15	35	21	14	E	1	212582.284	0.173	389.6622
36	21	16	35	21	15	A	1	212612.331	0.176	389.0393
36	21	15	35	21	14	A	1	212612.331	0.176	389.0393
36	21	15	35	21	14	A	0	212720.058	-0.061	272.8730
36	21	16	35	21	15	A	0	212720.058	-0.061	272.8730
36	21	16	35	21	15	E	0	212721.457	0.420	272.8534
35	9	27	34	9	26	E	1	212729.034	0.109	312.7033
36	20	17	35	20	16	E	1	212761.635	0.119	381.1138
36	20	16	35	20	15	E	1	212765.082	0.176	381.5814
36	20	17	35	20	16	A	1	212803.754	0.236	380.9768
36	20	16	35	20	15	A	1	212803.754	0.236	380.9768
17	10	8	16	9	8	E	0	212846.719	0.286	104.4180
17	10	7	16	9	7	E	0	212878.604	0.246	104.4352
36	20	16	35	20	15	A	0	212908.071	-0.035	264.7834
36	20	17	35	20	16	A	0	212908.071	-0.035	264.7834
36	20	17	35	20	16	E	0	212909.505	0.215	264.7644
17	10	8	16	9	7	A	0	212916.812	0.259	104.4178
17	10	7	16	9	8	A	0	212916.812	0.259	104.4178
17	10	8	16	9	8	E	1	212936.597	-0.225	220.5371
36	19	17	35	19	16	E	1	212977.021	-0.093	373.9009
36	19	18	35	19	17	A	1	213026.012	0.274	373.3236
36	19	17	35	19	16	A	1	213026.012	0.274	373.3236
49	3	46	49	2	47	E	0	213050.050	-0.384	283.0842
49	4	46	49	2	47	E	0	213050.050	-0.384	283.0842
49	4	46	49	3	47	E	0	213050.050	-0.384	283.0842
36	19	17	35	19	16	A	0	213126.585	-0.016	257.0991
36	19	18	35	19	17	A	0	213126.585	-0.016	257.0991
36	19	18	35	19	17	E	0	213128.208	0.114	257.0810
36	18	18	35	18	17	E	1	213226.222	0.162	366.6226
35	10	26	34	10	25	E	1	213231.164	0.148	315.9936
36	18	19	35	18	18	E	1	213236.605	0.100	366.0695
36	18	18	35	18	17	A	1	213286.703	0.235	366.0810
36	18	19	35	18	18	A	1	213286.703	0.235	366.0810
49	4	46	49	3	47	E	1	213311.675	0.056	399.3539
49	3	46	49	3	47	E	1	213311.675	0.056	399.3539
49	4	46	49	2	47	E	1	213311.675	0.056	399.3539
49	3	46	49	2	47	E	1	213311.675	0.056	399.3539
35	7	28	34	7	27	A	1	213337.614	0.127	309.4402
15	11	5	14	10	5	E	1	213345.753	0.222	218.1902
36	18	18	35	18	17	A	0	213383.313	0.120	249.8221
36	18	19	35	18	18	A	0	213383.313	0.120	249.8221
36	18	18	35	18	17	E	0	213385.080	0.163	249.8224
36	18	19	35	18	18	E	0	213385.080	0.163	249.8051
35	10	25	34	10	24	E	1	213489.361	0.208	316.5128
36	17	19	35	17	18	E	1	213521.803	0.195	359.7490
36	17	20	35	17	19	E	1	213542.369	0.143	359.1652
36	17	20	35	17	19	A	1	213596.217	0.174	359.2515
36	17	19	35	17	18	A	1	213596.217	0.174	359.2515
36	17	19	35	17	18	A	0	213688.122	-0.010	242.9551
36	17	20	35	17	19	A	0	213688.122	-0.010	242.9551
36	17	19	35	17	18	E	0	213690.140	0.008	242.9579
36	17	20	35	17	19	E	0	213690.140	0.008	242.9396
35	7	28	34	7	27	E	0	213723.768	0.029	192.7652
35	7	28	34	7	27	A	0	213739.330	0.019	192.7526
35	7	28	34	7	27	E	1	213795.832	-0.177	309.0442
46	19	27	46	18	28	A	0	213800.618	0.335	339.3214
46	19	28	46	18	29	A	0	213800.618	0.335	339.3214
36	16	20	35	16	19	E	1	213877.570	0.175	353.2836
36	16	21	35	16	20	E	1	213911.399	0.065	352.6780
16	2	14	15	1	15	E	1	213948.619	0.577	198.9274
36	16	21	35	16	20	A	1	213969.060	0.246	352.8382
36	16	20	35	16	19	A	1	213969.060	0.246	352.8382
36	16	20	35	16	19	A	0	214055.727	0.099	236.5018
36	16	21	35	16	20	A	0	214055.727	0.099	236.5018
36	16	21	35	16	20	E	0	214058.570	0.118	236.4879
36	15	21	35	15	20	E	1	214313.028	0.102	347.2313

J'	K'_a	K'_c	J''	K''_a	K''_c	Sym.	$v_{t_{CH_3}}$	$\nu_{Obs.}$	$\nu_{Obs.} - \nu_{Calc.}$	E_{low}
36	15	22	35	15	21	E	1	214364.739	0.143	346.6135
34	8	26	33	8	25	E	1	214368.350	-0.055	304.1110
36	15	22	35	15	21	A	1	214425.595	0.180	346.8456
36	15	21	35	15	20	A	1	214425.595	0.180	346.8456
34	8	26	33	8	25	A	1	214490.172	-0.004	304.5119
36	15	21	35	15	20	A	0	214506.174	0.086	230.4673
36	15	22	35	15	21	A	0	214506.174	0.086	230.4673
36	15	21	35	15	20	E	0	214508.063	0.069	230.4746
37	6	31	36	7	30	A	1	214509.747	-0.029	320.3307
36	15	22	35	15	21	E	0	214509.754	0.183	230.4552
34	8	26	33	8	25	E	0	214658.294	-0.050	187.8256
34	8	26	33	8	25	A	0	214659.875	0.149	187.8124
37	6	31	36	7	30	E	1	214671.864	0.148	319.9797
37	6	31	36	7	30	E	0	214697.706	0.013	203.6830
37	6	31	36	7	30	A	0	214702.007	0.077	203.6720
36	8	29	35	8	28	A	1	214737.758	0.023	316.8480
35	10	25	34	10	24	A	0	214757.305	0.086	199.9816
35	10	25	34	10	24	E	0	214762.251	0.077	199.9909
39	4	35	38	4	34	A	1	214805.329	0.121	325.7352
39	5	35	38	5	34	A	1	214805.329	0.121	325.7353
38	6	33	37	6	32	A	1	214814.700	-0.054	323.2904
35	10	25	34	10	24	A	1	214817.411	-0.109	316.5897
38	5	33	37	5	32	A	1	214831.703	-0.052	323.2891
36	14	22	35	14	21	E	1	214857.547	0.171	341.5992
40	3	37	39	3	36	A	1	214912.081	-0.099	327.7096
40	4	37	39	3	36	A	1	214912.081	-0.099	327.7096
40	3	37	39	4	36	A	1	214912.081	-0.099	327.7096
40	4	37	39	4	36	A	1	214912.081	-0.099	327.7096
36	14	23	35	14	22	E	1	214933.532	0.212	340.9794
36	8	29	35	8	28	E	0	214956.414	-0.042	200.2064
36	8	29	35	8	28	A	0	214962.098	0.035	200.1956
39	5	35	38	5	34	E	1	214967.873	0.033	325.3608
39	4	35	38	4	34	E	1	214967.873	0.033	325.3607
37	7	31	36	7	30	A	1	214974.993	-0.056	320.3307
39	4	35	38	4	34	E	0	214988.787	-0.007	209.0645
39	5	35	38	5	34	E	0	214988.787	-0.007	209.0645
39	4	35	38	4	34	A	0	214993.712	-0.096	209.0527
39	5	35	38	5	34	A	0	214993.712	-0.096	209.0528
36	14	23	35	14	22	A	1	214997.038	0.095	341.2804
36	14	22	35	14	21	A	1	214997.038	0.095	341.2804
38	5	33	37	6	32	E	0	215000.464	-0.004	206.6343
35	23	12	34	23	12	E	1	215000.464	0.237	399.8480
38	5	33	37	6	32	A	0	215006.356	-0.124	206.6230
38	6	33	37	6	32	E	1	215015.306	0.104	322.9325
38	6	33	37	6	32	E	0	215025.481	0.033	206.6343
38	6	33	37	6	32	A	0	215031.631	0.002	206.6230
38	5	33	37	5	32	E	1	215035.269	0.093	322.9310
40	4	37	39	4	36	E	1	215039.762	-0.026	327.3093
40	3	37	39	4	36	E	1	215039.762	-0.026	327.3093
40	4	37	39	3	36	E	1	215039.762	-0.026	327.3093
40	3	37	39	3	36	E	1	215039.762	-0.026	327.3093
38	5	33	37	5	32	E	0	215044.413	0.097	206.6328
38	5	33	37	5	32	A	0	215050.595	-0.016	206.6216
38	6	33	37	5	32	E	1	215061.870	0.016	322.9310
36	14	23	35	14	22	A	0	215069.725	-0.223	224.8586
40	3	37	39	3	36	E	0	215069.725	-0.001	211.0182
40	4	37	39	3	36	E	0	215069.725	-0.001	211.0182
40	3	37	39	4	36	E	0	215069.725	-0.001	211.0182
40	4	37	39	4	36	E	0	215069.725	-0.001	211.0182
36	14	22	35	14	21	E	0	215072.086	-0.096	224.8682
40	3	37	39	3	36	A	0	215073.612	-0.038	211.0056
40	4	37	39	3	36	A	0	215073.612	-0.038	211.0056
40	3	37	39	4	36	A	0	215073.612	-0.038	211.0056
40	4	37	39	4	36	A	0	215073.612	-0.038	211.0056
41	2	39	40	2	38	A	1	215080.702	-0.154	329.2445
41	3	39	40	2	38	A	1	215080.702	-0.154	329.2445
41	2	39	40	3	38	A	1	215080.702	-0.154	329.2445
41	3	39	40	3	38	A	1	215080.702	-0.154	329.2445
41	3	39	40	3	38	E	1	215173.734	0.117	328.8096
41	2	39	40	3	38	E	1	215173.734	0.117	328.8096
41	3	39	40	2	38	E	1	215173.734	0.117	328.8096
41	2	39	40	2	38	E	1	215173.734	0.117	328.8096
37	7	31	36	7	30	E	0	215209.700	-0.035	203.6830
41	2	39	40	2	38	E	0	215211.960	-0.067	212.5265
41	3	39	40	2	38	E	0	215211.960	-0.067	212.5265
41	2	39	40	3	38	E	0	215211.960	-0.067	212.5265
41	3	39	40	3	38	E	0	215211.960	-0.067	212.5265
37	7	31	36	7	30	E	1	215211.960	0.036	319.9797
41	2	39	40	2	38	A	0	215214.819	-0.051	212.5128
41	3	39	40	2	38	A	0	215214.819	-0.051	212.5128
41	2	39	40	3	38	A	0	215214.819	-0.051	212.5128
41	3	39	40	3	38	A	0	215214.819	-0.051	212.5128
37	7	31	36	7	30	A	0	215216.894	0.022	203.6720
37	6	31	36	6	30	A	1	215277.853	0.027	320.3051
42	1	41	41	1	40	A	1	215280.992	-0.040	330.3606
42	2	41	41	1	40	A	1	215280.992	-0.040	330.3606
42	1	41	41	2	40	A	1	215280.992	-0.040	330.3606
42	2	41	41	2	40	A	1	215280.992	-0.040	330.3606
42	2	41	41	2	40	E	1	215336.876	-0.040	329.8823
42	1	41	41	2	40	E	1	215336.876	-0.040	329.8823
42	2	41	41	1	40	E	1	215336.876	-0.040	329.8823
42	1	41	41	1	40	E	1	215336.876	-0.040	329.8823
42	1	41	41	1	40	E	0	215384.068	-0.088	213.6098
42	2	41	41	1	40	E	0	215384.068	-0.088	213.6098
42	1	41	41	2	40	E	0	215384.068	-0.088	213.6098
42	2	41	41	2	40	E	0	215384.068	-0.088	213.6098
42	1	41	41	1	40	A	0	215385.847	-0.015	213.5948
42	2	41	41	1	40	A	0	215385.847	-0.015	213.5948
42	1	41	41	2	40	A	0	215385.847	-0.015	213.5948
42	2	41	41	2	40	A	0	215385.847	-0.015	213.5948
43	0	43	42	0	42	A	1	215496.914	-0.054	331.0724
43	1	43	42	0	42	A	1	215496.914	-0.054	331.0724
43	0	43	42	1	42	A	1	215496.914	-0.054	331.0724
43	1	43	42	1	42	A	1	215496.914	-0.054	331.0724
43	1	43	42	1	42	E	1	215512.277	-0.056	330.5412
43	0	43	42	1	42	E	1	215512.277	-0.056	330.5412
43	1	43	42	0	42	E	1	215512.277	-0.056	330.5412
43	0	43	42	0	42	E	1	215512.277	-0.056	330.5412
37	6	31	36	6	30	E	0	215539.072	-0.012	203.6549
37	6	31	36	6	30	A	0	215548.145	0.289	203.6438
22	8	15	21	7	14	A	0	215555.597	-0.141	117.1700
37	6	31	36	6	30	E	1	215555.597	-0.065	319.9502
36	13	23	35	13	22	E	1	215557.401	0.143	336.3978
43	1	43	42	0	42	A	0	215569.539	-0.280	214.2656
43	0	43	42	1	42	A	0	215569.539	-0.280	214.2656
43	1	43	42	1	42	A	0	215569.539	-0.280	214.2656

J'	K'_a	K'_c	J''	K''_a	K''_c	Sym.	$v_{t_{CH_3}}$	$\nu_{Obs.}$	$\nu_{Obs.} - \nu_{Calc.}$	E_{low}
43	0	43	42	0	42	E	0	215569.539	0.187	214.2822
43	1	43	42	0	42	E	0	215569.539	0.187	214.2822
43	0	43	42	1	42	E	0	215569.539	0.187	214.2822
43	1	43	42	1	42	E	0	215569.539	0.187	214.2822
36	13	24	35	13	23	E	1	215668.159	0.166	335.7870
36	13	24	35	13	23	A	1	215729.662	-0.061	336.1527
36	13	23	35	13	22	A	1	215737.452	0.122	336.1530
36	13	24	35	13	23	A	0	215793.912	0.105	219.6865
36	13	23	35	13	22	E	0	215799.396	0.014	219.6982
36	13	23	35	13	22	A	0	215801.001	0.197	219.6868
36	13	24	35	13	23	E	0	215802.937	0.199	219.6790
37	7	30	36	8	29	A	0	215884.836	0.191	207.3659
37	7	30	36	8	29	E	0	215898.215	0.151	207.3766
37	7	30	36	8	29	A	1	215986.119	-0.170	324.0108
21	6	15	20	4	16	E	0	216051.189	0.047	107.8058
37	7	31	36	6	30	E	0	216051.189	0.047	203.6549
37	7	31	36	6	30	A	0	216062.852	0.055	203.6438
37	7	31	36	6	30	E	1	216095.903	0.033	319.9502
36	12	24	35	12	23	E	1	216494.804	0.147	331.6433
48	3	46	48	2	47	E	1	216520.725	0.205	383.6325
48	2	46	48	2	47	E	1	216520.725	0.205	383.6325
48	3	46	48	1	47	E	1	216520.725	0.205	383.6325
48	2	46	48	1	47	E	1	216520.725	0.205	383.6325
23	7	16	22	6	17	E	0	216629.002	0.032	119.0990
23	7	16	22	6	17	A	0	216643.222	-0.259	119.0854
36	12	25	35	12	24	E	1	216659.198	0.182	331.0540
36	12	25	35	12	24	A	1	216683.997	0.126	331.4782
36	12	25	35	12	24	A	0	216737.368	0.080	214.9670
36	12	24	35	12	23	A	1	216766.302	0.021	331.4812
36	12	25	35	12	24	E	0	216774.357	0.088	214.9632
36	12	24	35	12	23	E	0	216785.856	0.038	214.9820
36	12	24	35	12	23	A	0	216813.702	0.121	214.9698
20	9	12	19	8	11	A	1	216941.428	0.294	228.4930
20	9	11	19	8	12	A	1	216971.286	-0.065	228.4921
22	8	14	21	7	15	A	0	217224.118	-0.160	117.1228
23	2	21	22	1	21	E	0	217489.223	0.061	108.5157
36	7	29	35	7	28	A	1	217579.122	-0.070	316.5564
13	7	6	12	5	7	A	0	217601.099	-0.373	81.9692
37	31	6	36	31	5	A	0	217624.720	-0.346	382.8875
37	31	7	36	31	6	A	0	217624.720	-0.346	382.8875
37	29	8	36	29	7	A	1	217636.727	0.113	475.2258
37	29	9	36	29	8	A	1	217636.727	0.113	475.2258
37	29	9	36	29	8	E	1	217641.903	0.021	475.6847
22	8	14	21	7	14	E	1	217672.283	0.139	233.7008
37	30	7	36	30	6	A	0	217691.921	-0.220	370.7988
37	30	8	36	30	7	A	0	217691.921	-0.220	370.7988
37	28	9	36	28	8	E	1	217704.051	0.023	464.5017
37	28	10	36	28	9	A	1	217718.391	0.258	463.9211
37	28	9	36	28	8	A	1	217718.391	0.258	463.9211
37	28	10	36	28	9	E	1	217721.114	0.066	464.4279
37	29	8	36	29	7	A	0	217765.026	-0.203	359.1083
37	29	9	36	29	8	A	0	217765.026	-0.203	359.1083
37	27	11	36	27	10	E	1	217790.439	0.132	453.5670
37	27	10	36	27	9	A	1	217808.026	0.108	453.0205
37	27	11	36	27	10	A	1	217808.026	0.108	453.0205
37	27	10	36	27	9	E	1	217808.026	0.121	453.5682
36	11	26	35	11	25	A	1	217828.927	0.057	327.2758
37	28	9	36	28	8	A	0	217845.155	-0.099	347.8161
37	28	10	36	28	9	A	0	217845.155	-0.099	347.8161
36	11	25	35	11	24	E	1	217858.570	0.242	327.3645
36	9	28	35	9	27	A	1	217874.304	0.037	320.1561
36	11	26	35	11	25	A	0	217878.262	0.052	210.7198
37	26	11	36	26	10	E	1	217903.894	0.165	443.1056
37	26	12	36	26	11	A	1	217907.443	0.139	442.5244
37	26	11	36	26	10	A	1	217907.443	0.139	442.5244
36	11	26	35	11	25	E	0	217913.621	0.027	210.7237
37	27	10	36	27	9	A	0	217933.291	-0.027	336.9228
37	27	11	36	27	10	A	0	217933.291	-0.027	336.9228
37	27	11	36	27	10	E	0	217933.291	-0.012	336.9055
20	9	12	19	8	12	E	1	217938.297	-0.263	227.9086
36	7	29	35	7	28	E	0	217959.908	0.016	199.8943
36	7	29	35	7	28	A	0	217975.219	-0.005	199.8822
36	9	28	35	9	27	E	0	218028.557	0.335	203.5349
36	9	28	35	9	27	A	0	218028.865	-0.224	203.5249
37	26	11	36	26	10	A	0	218030.908	0.160	326.4287
37	26	12	36	26	11	A	0	218030.908	0.160	326.4287
37	26	12	36	26	11	E	0	218030.908	0.064	326.4103
36	7	29	35	7	28	E	1	218037.573	-0.078	316.1757
36	11	26	35	11	25	E	1	218083.636	0.205	326.8114
37	25	12	36	25	11	A	0	218139.228	0.078	316.3343
37	25	13	36	25	12	A	0	218139.228	0.078	316.3343
37	25	13	36	25	12	E	0	218139.228	-0.151	316.3152
37	24	13	36	24	12	A	1	218141.762	0.074	422.7479
37	24	14	36	24	13	A	1	218141.762	0.074	422.7479
37	24	13	36	24	12	A	0	218260.601	0.115	306.6402
37	24	14	36	24	13	A	0	218260.601	0.115	306.6402
37	24	14	36	24	13	E	0	218260.601	-0.270	306.6206
37	23	14	36	23	13	A	1	218281.326	0.237	413.4686
37	23	15	36	23	14	A	1	218281.326	0.237	413.4686
36	9	28	35	9	27	E	1	218338.432	0.039	319.7992
37	23	14	36	23	13	A	0	218397.436	0.259	297.3473
37	23	15	36	23	14	A	0	218397.436	0.259	297.3473
37	23	15	36	23	14	E	0	218397.436	-0.309	297.3274
37	22	16	36	22	15	E	1	218401.964	0.090	404.8724
37	22	15	36	22	14	E	1	218413.707	0.184	405.2275
37	22	15	36	22	14	A	1	218439.373	0.231	404.5961
37	22	16	36	22	15	A	1	218439.373	0.231	404.5961
36	11	25	35	11	24	E	0	218482.896	0.081	210.7569
36	11	25	35	11	24	A	1	218500.316	-0.019	327.3060
36	11	25	35	11	24	A	0	218506.138	0.044	210.7479
37	22	15	36	22	14	A	0	218552.357	0.119	288.4564
37	22	16	36	22	15	A	0	218552.357	0.119	288.4564
37	22	16	36	22	15	E	0	218553.280	0.262	288.4365
37	21	17	36	21	16	E	1	218578.320	0.131	396.3387
37	21	16	36	21	15	E	1	218586.309	0.239	396.7531
37	21	17	36	21	16	A	1	218619.937	0.264	396.1313
37	21	16	36	21	15	A	1	218619.937	0.264	396.1313
36	10	27	35	10	26	A	1	218645.079	0.006	323.5402
18	10	8	17	9	8	E	0	218695.880	0.338	107.7935
36	10	27	35	10	26	A	0	218723.927	0.068	206.9423
17	4	14	16	2	15	E	0	218723.927	0.068	87.7275
37	21	16	36	21	15	A	0	218729.371	-0.103	279.9685
37	21	17	36	21	16	A	0	218729.371	-0.103	279.9685
36	10	27	35	10	26	E	0	218730.727	0.197	206.9504
37	21	17	36	21	16	E	0	218730.727	0.197	279.9490
18	10	9	17	9	8	A	0	218733.981	0.334	107.7761

J'	K'_a	K'_c	J''	K''_a	K''_c	Sym.	$v_{t_{CH_3}}$	$\nu_{Obs.}$	$\nu_{Obs.} - \nu_{Calc.}$	E_{low}
18	10	8	17	9	9	A	0	218733.981	0.334	107.7761
37	20	17	36	20	16	E	1	218784.804	0.120	388.6785
35	9	26	34	9	25	E	1	218807.995	0.197	313.4461
37	20	18	36	20	17	A	1	218827.856	0.277	388.0752
37	20	17	36	20	16	A	1	218827.856	0.277	388.0752
37	20	17	36	20	16	A	0	218933.706	-0.046	271.8852
37	20	18	36	20	17	A	0	218933.706	-0.046	271.8852
37	20	18	36	20	17	E	0	218935.287	0.219	271.8663
37	19	18	36	19	17	E	1	219015.606	0.132	381.0051
37	19	19	36	19	18	E	1	219019.441	0.195	380.4911
37	19	19	36	19	18	A	1	219069.413	0.180	380.4293
37	19	18	36	19	17	A	1	219069.413	0.180	380.4293
37	19	18	36	19	17	A	0	219171.446	0.050	264.2082
37	19	19	36	19	18	A	0	219171.446	0.050	264.2082
37	19	19	36	19	18	E	0	219173.157	0.108	264.1902
20	9	11	19	8	11	E	1	219251.427	0.034	228.4045
37	18	19	36	18	18	E	1	219286.717	0.198	373.7351
37	18	20	36	18	19	E	1	219298.991	0.111	373.1823
37	18	19	36	18	18	A	1	219353.319	0.242	373.1955
37	18	20	36	18	19	A	1	219353.319	0.242	373.1955
37	18	19	36	18	18	A	0	219450.858	0.075	256.9398
37	18	20	36	18	19	A	0	219450.858	0.075	256.9398
37	18	19	36	18	18	E	0	219452.805	0.136	256.9402
37	18	20	36	18	19	E	0	219452.805	0.136	256.9229
47	2	46	47	1	47	E	1	219472.894	0.075	368.1374
47	1	46	47	1	47	E	1	219472.894	0.075	368.1374
47	2	46	47	0	47	E	1	219472.894	0.075	368.1374
47	1	46	47	0	47	E	1	219472.894	0.075	368.1374
37	17	20	36	17	19	E	1	219608.948	0.203	366.8713
38	6	32	37	7	31	A	1	219619.442	-0.112	327.5015
37	17	21	36	17	20	E	1	219632.365	0.097	366.2882
36	10	27	35	10	26	E	1	219660.446	0.114	323.1062
37	17	21	36	17	20	A	1	219690.764	0.195	366.3763
37	17	20	36	17	19	A	1	219690.764	0.195	366.3763
39	5	34	38	6	33	A	1	219728.809	-0.156	330.4559
35	9	26	34	9	25	A	0	219731.417	-0.060	197.0882
35	9	26	34	9	25	E	0	219741.613	-0.131	197.0998
39	6	34	38	6	33	A	1	219741.618	0.118	330.4559
40	4	36	39	4	35	A	1	219748.021	-0.056	332.9004
40	5	36	39	4	35	A	1	219748.021	-0.056	332.9004
40	4	36	39	5	35	A	1	219748.021	-0.056	332.9004
40	5	36	39	5	35	A	1	219748.021	-0.056	332.9004
39	5	34	38	5	33	A	1	219751.231	-0.009	330.4551
35	9	26	34	9	25	A	1	219773.413	-0.010	313.7509
37	17	20	36	17	19	A	0	219783.381	0.114	250.0830
37	17	21	36	17	20	A	0	219783.381	0.114	250.0830
37	17	20	36	17	19	E	0	219785.586	0.133	250.0858
37	17	21	36	17	20	E	0	219785.586	0.133	250.0675
38	6	32	37	7	31	E	1	219808.287	0.103	327.1584
38	6	32	37	7	31	E	0	219827.034	0.116	210.8616
38	6	32	37	7	31	A	0	219832.370	0.086	210.8508
41	3	38	40	3	37	A	1	219862.543	-0.054	334.8783
41	4	38	40	3	37	A	1	219862.543	-0.054	334.8783
41	3	38	40	4	37	A	1	219862.543	-0.054	334.8783
41	4	38	40	4	37	A	1	219862.543	-0.054	334.8783
37	8	30	36	8	29	A	1	219868.307	0.029	324.0108
38	7	32	37	7	31	A	1	219897.918	-0.048	327.5015
40	5	36	39	5	35	E	1	219909.389	0.098	332.5313
40	4	36	39	4	35	E	1	219909.389	0.098	332.5313
39	5	34	38	6	33	E	1	219924.676	0.179	330.1047
40	4	36	39	4	35	E	0	219932.390	0.019	216.2357
40	5	36	39	5	35	E	0	219932.390	0.019	216.2358
40	4	36	39	4	35	A	0	219937.389	0.047	216.2242
40	5	36	39	5	35	A	0	219937.389	0.047	216.2242
39	6	34	38	6	33	E	1	219939.796	0.159	330.1047
39	5	34	38	6	33	A	0	219943.948	-0.392	213.7957
39	5	34	38	5	33	E	1	219950.992	-0.184	330.1038
39	6	34	38	6	33	E	0	219952.464	0.017	213.8067
39	6	34	38	6	33	A	0	219958.476	-0.087	213.7957
39	5	34	38	5	33	E	0	219963.247	-0.058	213.8059
39	5	34	38	5	33	A	0	219969.414	-0.075	213.7949
39	6	34	38	5	33	E	0	219977.476	0.048	213.8059
39	6	34	38	5	33	A	0	219984.098	0.387	213.7949
16	11	5	15	10	5	E	1	219984.098	-0.461	221.7222
41	4	38	40	4	37	E	1	219989.524	0.048	334.4822
41	3	38	40	4	37	E	1	219989.524	0.048	334.4822
41	4	38	40	3	37	E	1	219989.524	0.048	334.4822
41	3	38	40	3	37	E	1	219989.524	0.048	334.4822
37	16	21	36	16	20	E	1	219997.580	0.238	360.4178
